# Tenascin-C expression contributes to pediatric brainstem glioma tumor phenotype and represents a novel biomarker of disease

**DOI:** 10.1186/s40478-019-0727-1

**Published:** 2019-05-15

**Authors:** J. Qi, D. R. Esfahani, T. Huang, P. Ozark, E. Bartom, R. Hashizume, E. R. Bonner, S. An, C. M. Horbinski, C. D. James, A. M. Saratsis

**Affiliations:** 1 Department of Neurological Surgery, Northwestern University Feinberg School of Medicine, Chicago, IL USA; 2 Department of Neurological Surgery, University of Illinois at Chicago, Chicago, IL USA; 3 Department of Biochemistry and Molecular Genetics, Northwestern University Feinberg School of Medicine, Chicago, IL USA; 4 Center for Genetic Medicine, Children’s National Health System, Washington, DC 20010 USA; 5 Institute for Biomedical Sciences, The George Washington University School of Medicine and Health Sciences, Washington, DC USA; 6 Department of Pathology, Northwestern University Feinberg School of Medicine, Chicago, IL USA; 7 Division of Pediatric Neurosurgery, Department of Surgery, Ann & Robert H. Lurie Children’s Hospital of Chicago, Chicago, IL USA

**Keywords:** Tenascin-C, Diffuse midline glioma, Diffuse intrinsic pontine glioma (DIPG), Histone H3 mutation (H3K27 M)

## Abstract

Diffuse intrinsic pontine glioma (DIPG), an infiltrative, high grade glioma (HGG) affecting young children, has the highest mortality rate of all pediatric cancers. Despite treatment, average survival is less than twelve months, and five-year survival under 5%. We previously detected increased expression of Tenascin-C (TNC) protein in DIPG cerebrospinal fluid and tumor tissue relative to normal specimens. TNC is an extracellular matrix (ECM) glycoprotein that mediates cell-matrix interactions, guides migrating neurons during normal brain development and is thought to maintain the periventricular stem cell niche in the developing brain. Tumor TNC expression is reported in adult glioma and other cancers. However, the pattern and effects of TNC expression in DIPG has not been previously explored. Here, we characterize TNC expression in patient derived pediatric supratentorial HGG (*n* = 3) and DIPG (*n* = 6) cell lines, as well as pediatric glioma tumor (*n* = 50) and normal brain tissue specimens (*n* = 3). We found tumor specific TNC gene and protein overexpression that directly correlated with higher tumor grade (WHO III and IV, *p* = 0.05), H3K27 M mutation (*p* = 0.012), shorter progression free survival (*p* = 0.034), and poorer overall survival (0.041) in association with these factors. TNC knockdown via lentiviral shRNA transfection of HGG (*n* = 1) and DIPG (*n* = 3) cell lines resulted in decreased cell proliferation, migration, and invasion in vitro (*p* < 0.01), while TNC cDNA transfection resulted in increased cell migration, invasion and proliferation (*p* < 0.01) as well as altered cell morphology in H3K27 M mutant DIPG lines. Whole transcriptome sequencing analysis (RNA-Seq) on DIPG (*n* = 3) and HGG (*n* = 2) cell lines after TNC cDNA, shRNA, and empty vector control transfection revealed the effects of TNC expression level on global gene expression profiles. Together, our findings reveal TNC expression in DIPG in association with H3K27 M mutation and VEGF signaling, and suggest that TNC may contribute to DIPG tumor phenotype, and serve as a clinically detectable biomarker for DIPG.

**Electronic supplementary material:**

The online version of this article (10.1186/s40478-019-0727-1) contains supplementary material, which is available to authorized users.

## Introduction

Brain tumors are the most common solid cancer in children. Approximately 15% of pediatric brain tumors arise in the brainstem, of which up to 80% are a subtype known as diffuse intrinsic pontine glioma (DIPG), an infiltrative, high grade glioma affecting young children [76]. DIPG typically affects children between six to nine years of age, and has the highest mortality rate of all pediatric cancers. Due to its characteristic appearance on MR imaging, DIPG is most often diagnosed radiographically at the time of symptom onset, which may include symptoms due to obstructive hydrocephalus and brainstem compression, including cranial nerve deficits and hyperreflexia [76]. The diffuse nature and location of DIPG precludes surgical resection, while chemotherapeutic agents that are more effective in other pediatric and adult gliomas are not effective in DIPG [4, 14, 17, 25, 39]. Standard treatment is focal radiation, which provides temporary symptom improvement but has no effect on overall survival [25, 31, 52, 59]. Despite treatment, average survival is less than twelve months, and five-year survival under 5% [52], with no improvement in overall survival despite more than 40 years of clinical trials [24]. Given the rapid clinical progression of DIPG and its poor response to treatment, improving our understanding of tumor biology is necessary to achieve more effective therapies.

Historically, our understanding of DIPG biology was impeded by the limited tumor tissue available for molecular analysis. However, recent analyses of rare pediatric DIPG and thalamic glioma tissue specimens revealed a high rate of somatic missense mutations in genes encoding Histone H3.3 and H3.1 isoforms (*H3F3A* and *HIST1H3B*, respectively) [44, 68, 78]. This mutation results in Lys27Met (K27 M) substitution on the H3 N-terminal tail in up to 80% of tumors, and is associated with as a more aggressive clinical course and poorer response to therapy. This mutation at a critical H3 transcriptional regulatory residue alters chromatin structure, resulting in epigenetic dysregulation of gene transcription [44, 61, 68]. As a result, H3 mutant gliomas exhibit distinct patterns of DNA methylation, gene and protein expression [35, 44, 49, 61, 65, 68, 78]. Due to the significant impact of this mutation on tumor biology and clinical outcome, the most recent WHO grading system for central nervous system tumors designates H3K27 M mutant thalamic and brainstem tumors, including DIPG, as a distinct group termed *diffuse midline glioma, H3K27 M mutant, WHO grade IV* [48]. In turn, novel therapeutic strategies targeting the effects of H3K27 M mutation on chromatin structure and function in DMG are currently being investigated [29, 32, 54, 61].

We previously reported detection of Histone H3 mutations and tumor-specific proteins in cerebrospinal fluid (CSF) from children with DIPG [35, 66]. We also reported over-expression of specific genes and proteins on multi-dimensional molecular analysis of DIPG specimens relative to non-tumor controls [35, 65]. In these studies, we detected increased expression of Tenascin-C (TNC) protein in DIPG CSF and tumor tissue relative to normal specimens. We also detected *TNC* promoter hypomethylation on genome-wide CpG methylation analysis in association with H3K27 M mutation [65], suggesting a potential epigenetic mechanism for observed TNC expression patterns in H3K27 M mutant DIPG. TNC is an extracellular matrix (ECM) glycoprotein that mediates cell-cell and cell-matrix interactions [7] and guides migrating neurons during normal brain development [45]. In the developing brain, TNC is known to maintain a stem cell niche by modulating the mitogenic effects of PDGF and NOTCH signaling in oligodendroglial precursor cells (OPCs), the purported cells of origin for DIPG [7, 8, 58, 67, 69]. 7Importantly, elevated TNC expression in the brain is transient, decreasing, with little to no expression in healthy adult tissues. In contrast, TNC overexpression is found in a variety of disease states and multiple cancers, including adult and pediatric supratentorial high grade glioma (HGG) [7, 8, 56, 65] and pediatric ependymoma [2], as well as breast [11, 19], non-small cell lung [37], colorectal [21], and pancreatic cancers [57].

TNC overexpression is thought to contribute to tumorigenesis by facilitating extracellular matrix remodeling, cell migration and angiogenesis [10], and co-localizes with CD133 expression in glioma stem cells [56]. Elevated TNC mRNA and protein in adult glioma is also associated with mesenchymal and classic glioma subtypes, as well as higher tumor histological grade, disease recurrence, local tumor invasion and poorer overall survival [6, 28, 56, 64, 67]. In turn, RNA interference (RNAi) inhibition of TNC has been shown to improve overall survival in preclinical models of adult high grade glioma [63]. A query of recently published pediatric glioma genomic data demonstrates that *TNC* mRNA overexpression is associated with poorer overall and disease free survival, younger age, increased tumor grade, Histone H3 mutation and midline tumor location [50, 51]. Importantly, Puget et al. recently reported detection of two distinct DIPG molecular subtypes, one enriched for a mesenchymal, pro-angiogenic gene expression signature that included *TNC* overexpression [62]. However, the role of TNC as a biomarker of disease and potential therapeutic target in DIPG has not, to our best knowledge, been previously explored. Therefore, we sought to better characterize the pattern and effects of TNC protein and gene expression in pediatric glioma, including DIPG, and to investigate the effects of TNC expression on clinical outcomes and tumor cell biological properties. Our findings reveal increased TNC expression in DIPG tumor specimens relative to controls, in association with H3K27 M mutation and VEGF signaling, and suggest that TNC expression may serve as a clinically detectable biomarker for treatment stratification and measuring response to therapy.

## Materials and methods

### Human cell lines and tissues studied

Human patient-derived primary pediatric glioma cell lines (*n* = 9), including six DIPG lines (SF8628, SF7761, SUDIPG IV, SUDIPG XIII, HSJD-DIPG 007, and HSJD-DIPG 014) and three supratentorial high grade glioma lines (SF9427, SF9402 and KNS42) were utilized (Table 1). All lines were generously shared by Dr. Rintaro Hashizume and are well characterized [32]. Cell lines were cultured under the following conditions: SF8628 in DMEM with 10% serum; SF7761, DIPG IV, DIPG XIII, DIPG 007, DIPG 014 in serum-free media supplemented with EGF and FGF (Shenandoah, PA, USA); SF9427 and SF9402 in serum-free media supplemented with EGF and FGF with 5% serum; KNS42 in EMEM with 5% serum. All cells were maintained in a humidified incubator containing 5% CO_2_ at 37 °C and passaged every 4–5 days at a density of 5000 cell/cm^2^.

**Table 1:** Patient-derived pediatric supratentorial high-grade glioma (HGG) and brainstem glioma (DIPG) cell lines analyzed

Cell Line	Diagnosis	WHO Grade	Histone H3 Status
KNS42	HGG	IV	H3.3 G34 V
SF9427	HGG	IV	Wild-type
SF9402	HGG	IV	Wild-type
SF7761	DIPG	IV	H3.3 K27 M
SF8628	DIPG	IV	H3.3 K27 M
SU-DIPG IV	DIPG	IV	H3.1 K27 M
SU-DIPG XIII	DIPG	IV	H3.3 K27 M
HSJD-DIPG-007	DIPG	IV	H3.3 K27 M
HSJD-DIPG-014	DIPG	IV	H3.3 K27 M

Summary of pediatric glioma cell lines utilized for the described experiments, including tumor location, WHO Grade and Histone H3 mutation status. WHO: World Health Organization; HGG: high-grade glioma; DIPG: diffuse intrinsic pontine glioma

Pediatric astrocytoma (glioma) tumor tissue specimens (*n* = 50) and normal brainstem tissue (*n* = 3) were submitted for tissue immunohistochemistry. Specimens were collected at our institution between 1998 and 2015, during the course of treatment or post mortem, and were formalin fixed and paraffin embedded at the time of collection. Informed consent for specimen collection and analysis was obtained under protocols approved by the Institutional Review Boards of Ann & Robert H. Lurie Children’s Hospital of Chicago (Lurie Children’s Protocols 2012–14,877, 2005–12,252; Northwestern University Protocols STU00200351, STU00202063). Tissues were evaluated by a board-certified neuropathologist in order to determine histological diagnosis and tumor grade, according to the most recent WHO classification^46^. All brainstem gliomas met both classic tumor histologic and molecular criteria (H3K27 M mutant) for Grade IV designation.

Additional paired fresh frozen brainstem glioma tissue (*n* = 8) and normal frontal lobe tissue (n = 8) were collected post-mortem for western blot analysis. Informed consent for specimen collection and analysis was obtained under protocols approved by the Institutional Review Board of Children’s National Medical Center (IRB 1339, Study Number Pro00001339). Initial diagnosis of DIPG was made based on MRI radiographic appearance; tissue histology and H3 mutation status was confirmed post mortem by a neuropathologist and molecular analysis, respectively.

### Quantitative real-time polymerase chain reaction (qPCR)

Transcription of *TNC* and associated genes of interest in pediatric glioma cell lines (*n* = 9) was quantified via real-time PCR (qPCR). Briefly, total RNA was extracted from cultured cell lines using the RNeasy Mini Kit (QIAGEN, Hilden, Germany). A total of 2 μg of RNA was reverse transcribed using SuperScript® VILO mastermix (Invitrogen, CA, USA) using the manufacturer’s protocol. Expression was performed using Applied Biosystems QuantStudio 3 (Thermo Fisher Scientific). The final PCR reaction mix (20 μL) included 2 μL of primer (5 μM), 1 μL of first-strand cDNA, and 10 μL of TagMan Fast Advanced Master Mix (Applied Biosystems, CA, USA) and analyzed in triplicate, with gene expression level normalized to *GAPDH* expression. The following Taqman probes were used for the PCR reaction (Lifetech USA): TNC (Hs01115665_m1), NOTCH1 (Hs01062014_m1), NOTCH2 (Hs01050702_m1), PDGFRA, (Hs00998026_m1), MYCN (Hs00232074_m1), CD133 (Hs01009257_m1). Relative gene expressions between cell lines were expressed as 2^-∆CT^ method [10].

### Protein expression determination by Western blot

TNC protein expression was determined in pediatric glioma cell lines (*n* = 9), tumor tissue (*n* = 8) and normal frontal lobe brain tissue from the same patient (*n* = 8) via western blot. For cell lines, cells were washed twice with phosphate-buffered saline (PBS). Whole cell lysates were extracted using RIPA Buffer (Pierce, IL, USA), Halt Protease and Phosphatase Inhibitor Cocktail (Pierce, IL, USA) on ice for 15 min. Lysates were then centrifuged for 15 min at 21,000 g, 4 °C, and supernatant transferred to sterile microcentrifuge tubes. Protein concentration was determined using Pierce BCA Protein Assay kit (Pierce, IL, USA). 20μg protein was denatured at 95 °C in 4X loading dye for 10 min and loaded into 12% Mini-PROTEAN TGX Precast Gels (Bio-Rad). After separation by electrophoresis, proteins were transferred to a polyvinyl difluoride (PVDF) membrane at 100 V for one hour. PVDF membranes were blocked using 5% skim milk for one hour, then incubated at 4 °C overnight with rabbit monoclonal anti-human TNC Ab (11,000, Abcam, ab108930) and rabbit monoclonal anti-human GAPDH Ab (15,000, CST, #2118S). Membranes were then incubated for one hour at room temperature with HRP-conjugated anti-rabbit IgG secondary antibody (15,000, Cell signaling technology, #7074). Bands were detected with Pierce ECL Plus western blotting substrate (Pierce, IL, USA) and intensity quantified using the ChemiDoc XRS+ System (Bio-Rad).

Similar Western Blotting procedure was used for protein derived from tumor (*n* = 8) and matched normal frontal lobe tissue (n = 8). 15μg total protein from each sample was loaded in gel for electrophoresis, proteins were then transferred to nitrocellulose membranes. Membranes were left to dry for 24 h, then blocked for 2 h with 5% milk, and finally incubated overnight at 4 °C in the same anti-TNC and anti-GAPDH antibody described above. Membranes were then incubated for 30 min at room temperature with HRP-conjugated anti-rabbit IgG secondary antibody, and bands were detected the same way as described above.

### Exogenous TNC in vitro

Cells in suspension were seeded into 24-well plates at a density of 200,000/well in medium containing FBS and growth factors. After 24 h the medium was changed with serum and growth factor-free medium containing soluble TNC (MilliporeSigma, USA) at each of three increasing concentrations (0.01, 0.1 and 1 μg/ml respectively) for 72 h. Cell proliferation was subsequently measured using 3-(4,5-dimethylthiazol-2-yl)-2,5-diphenyltetrazolium bromide (MTT) assay (Invitrogen, USA). To test the effect of TNC coating on adherent cell lines, 200,000/well cells were seeded into 24-well plates coated with TNC in serum and growth factor free medium. Cells were grown up to 72 h and cells were quantified by MTT assay to determine cell proliferation.

### Transfection of pediatric glioma cell lines with TNC cDNA and shRNA

In order to evaluate the effects of altered TNC expression in vitro, pediatric glioma cell lines were genetically modified via lentiviral transfection with short-hairpin RNA (shRNA) against the TNC transcript (*n* = 4, SF7761, SF8628, DIPGIV, and SF9427) and TNC cDNA (*n* = 4, DIPGIV, SF8628, SF9427, KNS42). In brief, to stably knock-down *TNC* gene expression VSVG-pseudotyped lentivirus expressing TNC shRNA and non-silencing controls were generated at our institution (DNA/RNA Delivery Core, Northwestern University, Chicago IL) as previously described [20, 81]. Briefly, 293 T packaging cells (Gene Hunter Corporation, Nashville TN, USA), 2nd generation packaging vectors psPAX2, pMD2.G (Addgene, Cambridge MA, USA) and 2rd generation lentiviral expression vector pGIPZ (Open Biosystems, Dharmacon Lafayette CO, USA) were employed to generate TNC shRNA (TTGTGGTGAAGATGGTCTG) and non-silencing control constructs (Cat# RHS4348, sense: CTTACTCTCGCCCAAGCGAGA, Open Biosystems, Dharmacon, Lafayette CO, USA). The pGIPZ vector also encoded green fluorescent protein (GFP) expression inframe with shRNA to facilitate confirmation of transfection. Lentiviral transfection efficacy (> 90%) was verified by GFP expression measurement using fluorescent microscopy. Bulk cell populations, but not individual stably infected cell clones, were used to establish stable cell lines expressing specific or non-silencing shRNA, then maintained in 8 μg/ml puromycin. To stably express human TNC protein, VSVG-pseudotyped lentivirus expressing a custom designed TNC cDNA (Genecopoeia, MD, USA) was generated in a similar fashion then sub-cloned into 3rd generation commercial lentiviral vector CD510B-1 (System Biosciences, Palo Alto CA, USA) downstream of FLAG tag. The CD510B-1 empty and GFP expressing virus was used as a control. Stable cell lines were maintained in the presence of 2 μg/ml puromycin.

### Proliferation assay

Cell proliferation was measured by counting of cells using the TC20™ Automated Cell Counter (Bio-Rad, CA, USA). 2-5 × 10^5^ cells were seeded into 10 cm cell culture dishes. At two, four, six and eight days after seeding, the cells were trypsinized and counted. Cells numbers at two, four, six and eight days were normalized to the cell numbers initially plated. Experiments were performed in triplicate.

### Wound healing assay

Cell migration was characterized using wound healing (scratch) assay. Cells were seeded in 24-well plates at a concentration of 200,000/well and culture overnight allowed to grow into monolayer. Artificial scratch gaps (wounds) were made in each cell monolayer using a 200 μl pipette tip, then cells immediately washed and incubated in culture media. Digital images of the wounded area were acquired at zero and 24 h. Free Java-based ImageJ image processing software (Wayne Rasband, National Institutes of Health, Bethesda MD, USA) to measure cell confluence after wounding by determining the percentage of wound region remaining (% WR) [42].

### Invasion assay

The effect of TNC knockdown and overexpression on cell invasion was evaluated using Matrigel-coated Transwell inserts (BD Biosciences, San Diego, CA, USA). Briefly, 50,000 cells in 500 ul of serum-free medium were added to the upper chamber, and medium containing 10%FBS was added to the lower chamber. The cells were left to invade the Matrigel coating for 24 h at 37 °C. Non-invading cells on upper surface of the membrane were removed by wiping, and invading cells were fixed and stained with crystal violet. The number of invading cells was counted in duplicate under light microscopy at 20x magnification in five predetermined fields for each membrane.

### Mouse glioma xenografts

All animal protocols used in this study were approved by Northwestern University Center for Comparative Medicine, animal care and use committee. Four-six weeks old female athymic nude mice (Athymic Nude-Foxn1nu, Envigo, IN, USA) housed under aseptic conditions, which included filtered air and sterilized food, water, bedding, and cages. SF8628 cells were injected intracranially into nude mice under general anesthesia as previously described [33, 71]. In brief, a sterile 25 gauge sharp needle is used to puncture the skull 2 mm to the right of the bregma and 1 mm anterior to the coronal suture, creating an opening for the injection of tumor cells. A syringe containing 5 × 10^5^ SF8628 cells suspended in 2ul culture medium is then placed in this location perpendicular to the skull, then passed to a depth of 3 mm to reach the right frontal lobe. The cell suspension is then injected intracranially over two minutes. Cell viability was determined by trypan blue dye exclusion. Mouse body weight and behavior was measured daily, and bioluminescence imaging using the IVIS Spectrum (Perkin Elmer, CT, USA) performed weekly to monitor tumor growth. Mice were sacrificed when deemed too ill or when bioluminescence signals reached 10^9^ p/sec/cm^2^/sr. Mouse brains were collected and fixed with 4% paraformaldehyde for 48 h at 4 °C for preparation for hematoxylin and eosin (H&E) and TNC immunohistochemical staining, detailed below.

### Tissue immunohistochemistry

Human and mouse normal brain and glioma tumor tissue was deparaffinized then hydrated, respectively, in a standard xylene and ethanol sequence, followed by heat-induced epitope recovery in 1X Dako Target Retrieval Solution (Dako, CA, USA) at 110 °C for 5 min. Sections were permeabilized and blocked in background sniper (Biocare Medical, USA) at room temperature for 15 min. For human normal brain and tumor tissues, primary antibodies used were as follows: Rabbit Anti-TNC for one hour (1100, Sigma HPA004823); Rabbit anti-.K27 M overnight (11,000, Millipore ABE419); Rabbit Anti-Notch1 overnight (1100, CST#3608); Rabbit Anti-Notch3 overnight (1100, Santa Cruz sc-5593); Rabbit Anti-PDGFRA overnight (1200, CST #3174). Mouse normal brain and tumor tissues were stained with H&E. Tissue sections were then washed and incubated with HRP-labeled anti-rabbit secondary antibody (Dako kit K4011) for one hour at room temperature. After washing, sections were stained by DAB chromogen (Dako kit k4011), then counter stained for one minute at room temperature with hematoxylin.

Results of tissue IHC staining was evaluated independently by three pathologists, including one board certified neuropathologist, blinded to specimen identifiers including histological diagnosis and anatomic tumor location. In cases where two individual pathological evaluations were not concordant, the third pathologist evaluation determined the final designation. H3K27 M mutation status (wild type or mutant) and H3K27me3 positivity was defined as nuclear staining in > 80% of tumor cells visualized in the absence of staining in tumor vascular endothelial cells (internal control), as previously described by Venneti et al. [75]. TNC and PDGFRA staining intensity score was defined as: 0 = negative, 1 = weak, 2 = moderate, or 3 = strong positive. NOTCH1 and NOTCH3 staining intensity score was defined as 0 – absent or 1 = present. TNC expression was also quantified using the H-score as follows. The entirety of each slide was assessed by light microscopy. TNC staining intensity score was defined as: 0 = negative, 1 = weak, 2 = moderate, or 3 = strong positive. The fraction of positive cells in each high-powered field at each intensity level was determined as a percentage. The H-score was then generated as the cross-product of the intensity score and the fraction score, with a final value ranging from 0 to 300. Study measures were estimated using t-tests and chi-square tests for continuous or dichotomous variables, respectively, to test the null hypothesis that staining characteristics were the same across subgroups. H-score values were utilized for survival and multivariate analysis (Kaplan-Meier survival analysis, log-rank test, Cox Regression analysis and Chi-square testing, respectively). When appropriate, the Fischer’s exact test was implemented for categorical variables requiring small sample adjustment. Statistical tests were considered significant for *p*-values < 0.05. Data were analyzed using Intercooled Stata, Version 14.0 (Stata Corp, College Station TX, USA) and SPSS (IBM, Version 25).

### Clinical-pathological data collection

A retrospective chart review of pediatric glioma patients from whom tissue specimens were collected and analyzed was performed to determine the following: patient gender, age at diagnosis, tumor anatomic location, tumor histologic diagnosis, tumor WHO-grade, date of surgery, extent of tumor resection, date of recurrent and/or progressive disease, date and nature of adjuvant therapy, overall survival, progression free survival, and mortality. Tumor recurrence was defined as confirmed radiographic evidence on magnetic resonance imaging (MRI) of new disease burden after confirmation of tumor gross total resection (GTR) on initial post-operative MRI. Disease progression was defined as radiographic MRI evidence of increased disease burden after tumor biopsy or subtotal resection (STR), or evidence of recurrent and increasing disease burden after GTR.

### Cell line RNA-Seq

Proliferating DIPG cells were harvested at confluence with a cell scraper. Cells were washed once with PBS, and homogenized by running cell pellet (with no more than 10 million cells) through the QIA shredder homogenizer (QIAGEN, Hilden, Germany). RNA was purified from the homogenized lysate using the RNeasy Mini Kit (QIAGEN, Hilden, Germany) per manufacturer’s protocol. Extracted total RNA underwent DNase I (New England Biolabs M0303S) treatment for 30 min at room temperature. DNase-treated RNA was purified again with the RNeasy Mini Kit, and the resulting total RNA was used for library preparation.

RNA-Sequencing libraries were prepared using the Illumina TruSeq Stranded Total RNA Preparation Kit (RS-122-2201) with Ribo-Depletion. Input RNA quality was validated using the Agilent RNA 6000 Nano Kit (5067–1511). 1μg of total RNA was used as starting material. Libraries were validated using the Agilent DNA 1000 Kit (5067–1504). Resulting RNA-seq libraries were single-read sequenced on the NextSeq 500 system (Illumina). The raw BCL output files were processed using bcl2fastq (Illumina, version 2.17.1.14), followed by removing low quality bases from the 3′ end of the reads and requiring a minimum read length of 20 bases using Trimmomatic version 0.33 [5]. Reads were then mapped to the human genome (UCSC hg19) using TopHat version 2.1.0 [74]. Only uniquely mapped reads with up to 2 mismatches over the entire length of the gene were considered for ensuing analyses. Exonic reads were then assigned to specific genes from Ensembl version 72 and quantified using the htseq-count script from the Python package HTSeq version 0.6.0 [1].

### RNA-seq data analysis

Gene count tables from HTSeq were used as input for EBSeq version 1.16.0 [47]. Genes with a log_2_ fold change > 1 were treated as up-regulated, while genes with a log_2_ fold change <− 1 were treated as down-regulated. Under a false discovery rate of 0.05, genes with an empirical Bayesian posterior probability for being differentially expressed greater than 0.95 were considered to be differentially expressed unless otherwise specified. Custom R scripts were used to generate the RNA-seq heatmaps. RNA-seq heatmaps display the normalized log2 RPKMs of differentially expressed genes, where the genes and samples were subject to hierarchical clustering based on the Euclidean distance metric and centroid linking. GO functional analyses accounted for gene length bias and were conducted on genes identified as up- or down-regulated genes using the R package goseq version 1.28.0 [80]. Additional functional pathways and upstream regulator analysis was performed on differentially expressed gene sets using Ingenuity Pathways Analysis software (Qiagen, Germantown MD).

## Results

### TNC is highly expressed in DIPG tumor tissue and primary cell lines

**Fig. 1:**
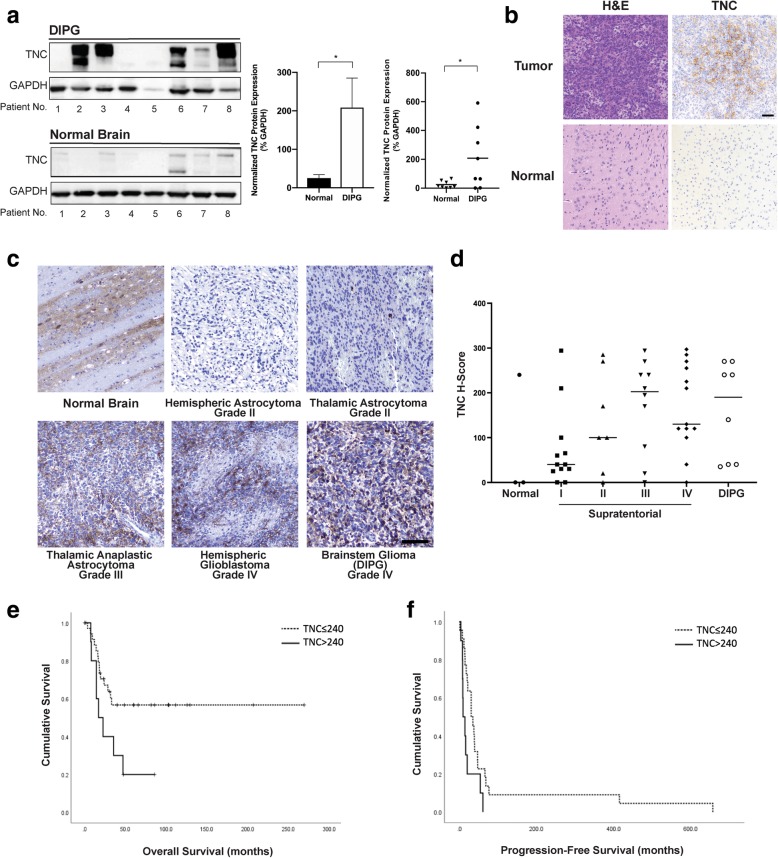
Tenascin C (TNC) is Overexpressed in Pediatric Glioma Tissue: TNC expression patterns were evaluated in pediatric glioma tumor and normal brain tissue specimens. **a** Western blot analysis of protein extract from DIPG tumor tissue and paired normal brain tissue from the same patient demonstrates increased TNC expression in tumor relative to normal specimens. Densitometry analysis of western blot signal intensity confirms statistical significance of TNC overexpression in tumor tissues (*p = 0.0388, paired two-tailed t-test). Data depicted in histogram, with error bars representing standard error of the mean, and in dot plot, with horizontal bars representing mean protein expression levels. *Y-axis:* Normalized TNC protein expression relative to GAPDH. **b** Immunohistochemical (IHC) staining of mouse glioma xenograft tumor and normal brain tissue demonstrates TNC staining of the plasma membrane and extracellular matrix in tumor specimens. Scale bar = 50 μm. **c** IHC staining was strongest in high-grade and midline glioma tissue, compared to low grade glioma and normal brainstem tissue. Scale bar = 100 μm. **d** H-score semi-quantification of pediatric glioma TNC staining demonstrating correlation with tumor histological grade. *Y-axis:* TNC H-score from 0 to 300. Horizontal bars represent the mean H-score**. e & f** Kaplan–Meier estimates of overall survival (e) and progression-free survival (f) stratified by 75% (240) TNC H-score. Higher TNC expression is associated with worse overall and progression-free survival (*p* = 0.041 and 0.034, respectively; log-rank test)

Increased TNC protein expression was detected in paired post-mortem tumor tissue specimens from children with DIPG, relative to normal brain tumor specimens (*n* = 8, *p* = 0.008, Paired t-test, Fig. 1a). Robust TNC expression was also observed in mouse glioma xenograft tissue generated with DIPG cell line SF8628 (Fig. 1b). To further evaluate TNC expression patterns in pediatric glioma and the relationship between tumor TNC expression and clinical pathological correlates, we performed tissue immunohistochemistry (IHC) on a cohort of 50 glioma tissue specimens (Tables 2 and 3). We quantified TNC expression in all patient samples by H-score, and found TNC expression directly correlated with tumor histologic grade, with highest TNC in high-grade tumors (WHO III and IV, *p* = 0.05, Fisher’s Exact Test, Fig. 1c, d). TNC also directly correlated with likelihood of clinical tumor recurrence (*p* = 0.07, Fisher’s Exact Test), and was inversely related to progression free survival (*p* = 0.0179, Fisher’s Exact Test). High TNC expression was also more common in H3K27 M mutant tumors (*p* = 0.012, Fisher’s Exact Test). High tissue TNC expression was associated with NOTCH3 immunopositivity (*n* = 39, *p* = 0.029, Fisher’s Exact Test), but we found no relationship between TNC expression patterns and NOTCH1 (*n* = 448 *p* = 0.57) or PDGFRa (*n* = 42 *p* = 0.68).

**Table 2:** Patient-derived pediatric glioma tumor and normal brain tissue specimens analyzed

		Tumor Tissue (*n* = 50)	Normal Tissue (*n* = 3)
Gender	Male	27 (54)	2 (66.67)
	Female	23 (46)	1 (33.33)
Age	< 5 years	7 (14)	2 (66.67)
	5–10 years	19 (38)	1 (33.33)
	> 10 years	24 (48)	0
Anatomic Location	Brainstem	18 (36)	3 (100)
	Thalamus	10 (20)	0
	Cerebellar Hemisphere	9 (18)	0
	Cerebral Hemisphere	13 (26)	0
WHO Grade	I	12 (24)	N/A
	II	7 (14)	N/A
	III	10 (20)	N/A
	IV	21 (42)	N/A

Formalin fixed paraffin embedded pediatric glioma tumor tissue (n = 50) and normal brainstem tissue specimens (n = 3) were submitted for immunohistochemical (IHC) analysis. Gender, age, tumor anatomical location and WHO-grade of analyzed specimens are summarized. Number in parentheses indicate percentage of total cohort

**Table 3:** Patient-derived pediatric glioma tumor and normal brain tissue specimens analyzed

		TNC score				*p*-value
		0	1	2	3	
WHO-Grade (n = 50)	I	2	7	1	2	*0.05
	II	1	1	3	2	
	III	1	1	2	6	
	IV	1	4	5	11	
Recurrence (*n* = 13)	Yes	2	0	0	4	0.07
	No	0	2	2	3	
Progression free survival (*n* = 33)		15.2	15.2	19.9	5.7	*0.0179
K27 M Status (n = 50)	MT	1	11	4	13	*0.012
	WT	4	2	7	8	
Notch3 (n = 39)	Present	2	8	4	13	*0.029
	Abscent	1	2	7	2	
Notch1 (*n* = 48)	Present	4	8	8	15	0.57
	Abscent	1	5	3	4	
PDGFRa (n = 42)	1	0	2	4	3	0.68
	2	1	5	5	6	
	3	2	5	2	7	

Descriptive statistical analysis of immunohistochemical (IHC) staining results. TNC expression was found to directly correlate with tumor histologic grade, with highest TNC expression in high-grade tumors (WHO III and IV, *p* = 0.05). TNC expression also directly correlated with clinical tumor recurrence (*p* = 0.07), and inversely correlated to progression free survival (*p* = 0.018). TNC over-expression was more common in H3K27 M mutant tumors (*p* = 0.012), and was associated with NOTCH3 immunopositivity (p = 0.012). Between groups comparison was performed using Fischer’s exact test. MT = H3K27 M positive, WT = H3K27 M negative. * = *p* < 0.05

We analyzed overall (OS) and progression-free survival (PFS) using H-score results by Kaplan-Meier survival analysis and the log-rank test. We found high TNC expression (cutoff of 75%, H-score > 240) was associated with poorer OS (*X2* = 4.190, *P* = 0.041 Kaplan-Meier analysis, log-rank test, (Fig. 1e). All other cut-off points analyzed (H-score mean, medium, and 25%) also indicated longer OS with low TNC expression, but were not statistically significant. We also found shorter PFS in patients with high TNC expression (cutoff of 75%, H-score > 240, *X2* = 4.517, *P* = 0.034 Kaplan-Meier analysis, log-rank test (Fig. 1f). These results suggest TNC expression is associated with a more aggressive clinical course in pediatric glioma. To clarify the statistical relationship between these variables and their contribution to clinical outcomes, we also performed multivariate analysis using a Cox regression model to. On direct analysis, TNC (*p* = 0.041), H3K27 M mutation status (*p* = 0.022) and tumor grade p (=0.004) were significant predictors of overall survival. However, on multivariate analysis, only tumor grade and H3K27 M mutation status were independent predictors of overall survival (χ2 = 14.095; *P* = 0.001; forward: Wald; *P* = 0.05, entry; *P* = 0.10, removal).

**Fig. 2:**
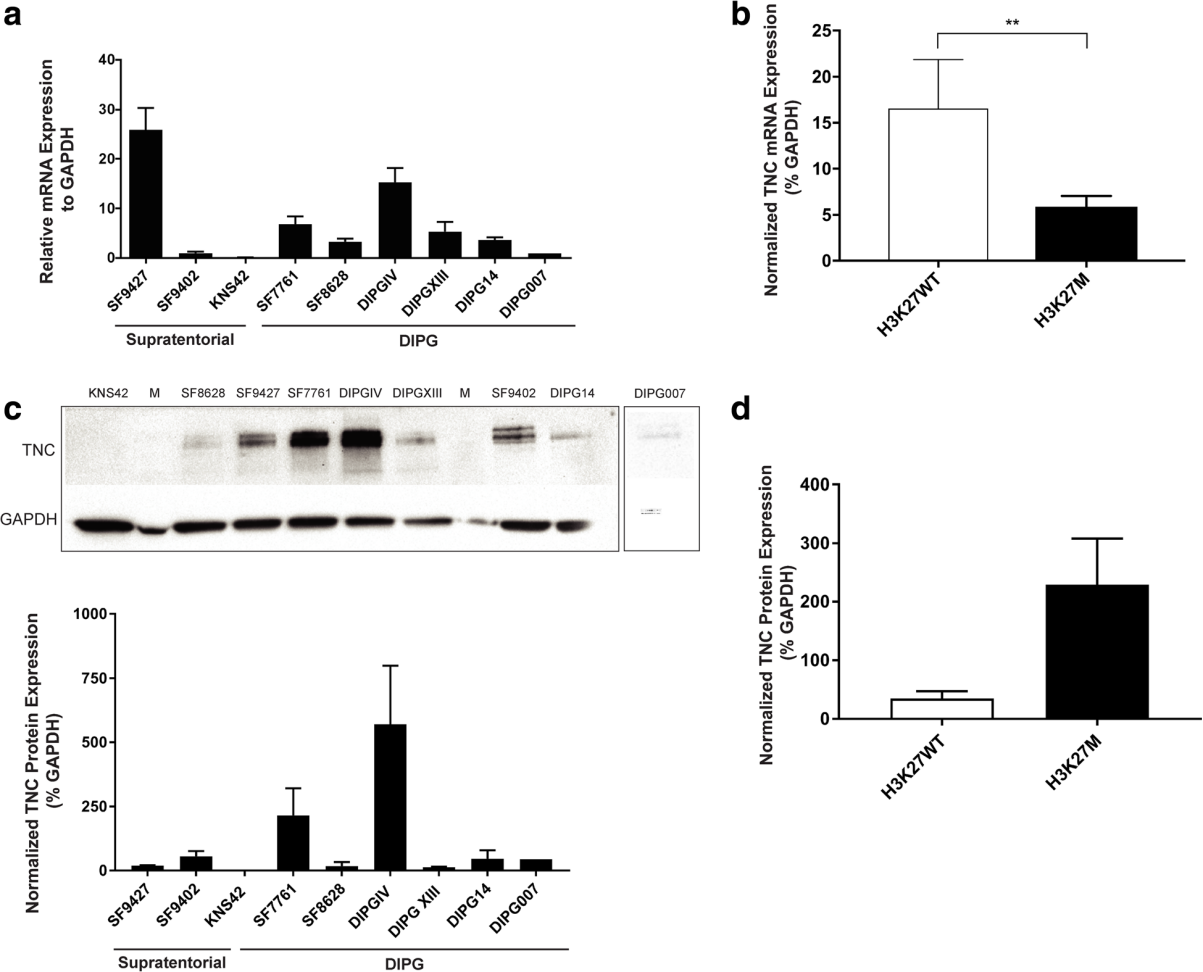
TNC Expression Patterns in Pediatric Glioma Cell Lines. *TNC* mRNA and protein expression levels were measured in pediatric supratentorial high grade glioma (HGG *n* = 3) and brainstem glioma (DIPG *n* = 6) cell lines. **a** *TNC* expression determined via quantitative PCR, greatest in DIPGIV (H3.1K27 M) and SF9427 (HGG). **b** Due to the high level of *TNC* expressed by SF9427 (H3K27 wild-type), overall *TNC* mRNA expression was greater in Histone H3K27 wild-type compared to H3K27 M cell lines (**p = 0.0061). *a & b Y-axis*: Relative *TNC* mRNA expression level in percent normalized to *GAPDH* (percent). **c** TNC protein expression determined via western blot, greatest in DIPGIV and SF7761 (H3.3K27 M mutant lines). **d** Greater TNC protein expression was seen in Histone H3K27 M compared to wild type cell lines, though this did not reach statistical significance (*p* = 0.09). *c & d Y-axis*: TNC protein expression relative to GAPDH (percent). Error bars represent standard error of the mean

Next, we determined endogenous TNC gene and protein expression levels in pediatric glioma cell lines, including three supratentorial high-grade glioma (HGG) cell lines (KNS42, SF9402, SF9427) and six DIPG cell lines (SF7761, SF8628, DIPGIV, DIPGXIII, DIPG14 and DIPG007). Differential endogenous *TNC* gene expression was detected by qPCR across cell lines (*p* < 0.0001, ANOVA), with highest expression in SF9427 (H3 WT) and DIPG IV (H3.1 K27 M, Fig. 2a). Due to the high level of *TNC* expressed by SF9427 (H3K27 wild-type), overall *TNC* mRNA expression was greater in Histone H3K27 wild-type compared to H3K27 M cell lines (**p = 0.0061, Fig. 2b). Differential TNC protein expression across cell lines was determined by Western blot (Fig. 2c), with DIPG IV exhibiting significantly greater TNC expression than all other cell lines studied (*p* = 0.0448, ANOVA). Greater TNC protein expression was seen in Histone H3K27 M compared to wild type cell lines, though this did not reach statistical significance (*p* = 0.09, Fig. 2d).

### TNC expression level affects glioma cell proliferation, adhesion and migration in vitro

**Fig. 3:**
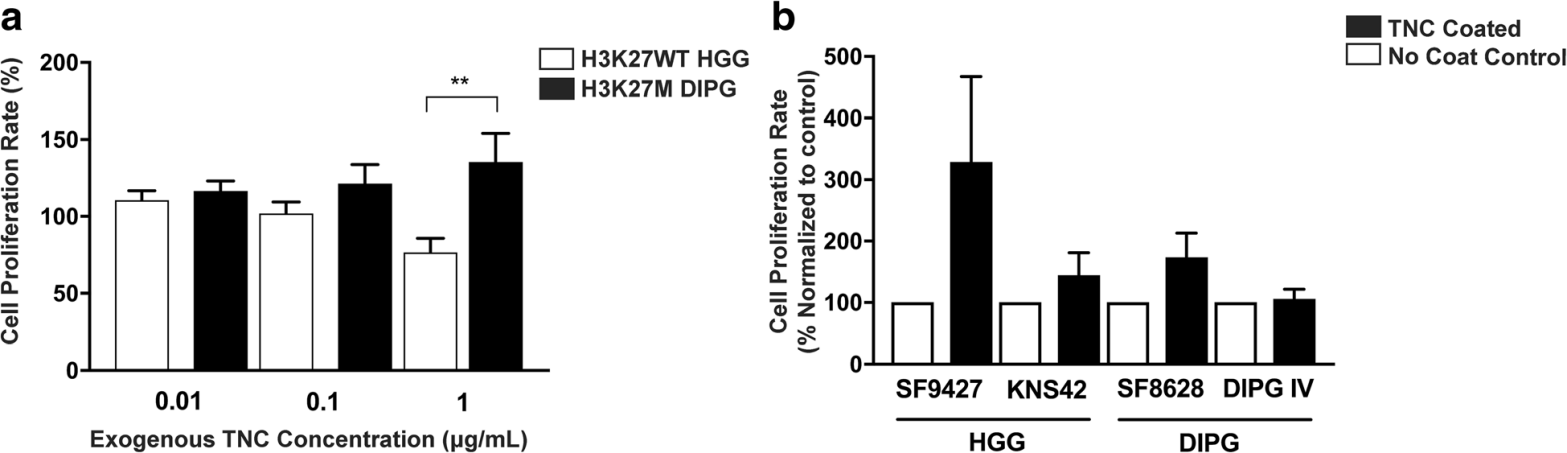
Exogenous TNC increases pediatric glioma cell proliferation in vitro. Exogenous TNC was added to pediatric supratentorial high grade glioma (HGG *n* = 2) and brainstem glioma (DIPG n = 2) cell lines in vitro in technical triplicate**. a** Exposure of glioma cells in suspension to exogenous TNC at increasing concentration promotes cell proliferation in H3K27 M DIPG relative to H3K27WT HGG lines, with greatest effect at 1μg/mL dose (**p = 0.0079). *X-axis:* Concentration of exogenous TNC added to cell culture media (ug/mL). *Y-axis:* Rate of cell proliferation relative to control with no exogenous TNC (percent). **b** Exposure of adherent glioma cells in monolayer to exogenous TNC promotes cell proliferation relative to uncoated controls, with greatest effect in cell lines with low endogenous TNC expression. *Y–axis:* Rate of cell proliferation relative to no coat control (% normalized to control)

To determine the effects of TNC on pediatric glioma cell biological function, exogenous TNC was added to the culture media of pediatric glioma cell lines in suspension at increasing concentrations (Fig. 3a). Increased cell proliferation was observed across DIPG cell lines (*n* = 2). By contrast, H3K27WT HGG lines in suspension (*n* = 2) exhibited decreased proliferation with exposure to increasing concentrations of exogenous TNC (*p* = 0.0120, ANOVA). The differential effect of 1 μg/mL TNC on cell proliferation between DIPG and HGG lines was statistically significant (*p* = 0.0079, t-test). Treatment of adherent DIPG (n = 2) and HGG cell lines (n = 2) by coating the culture dish with exogenous TNC also resulted in mild increase tumor cell proliferation, but was not statistically significant (Fig. 3b).

Next, the effect of TNC knockdown on glioma cell biological function was evaluated via lentiviral transfection of two distinct TNC shRNA constructs (shRNA232 and shRNA234) in three DIPG (DIPGIV, SF7761, SF8628) and one HGG cell line (SF9427). Efficacy of vector transfection and decreased *TNC* mRNA and protein expression relative to empty vector controls using both shRNA constructs was confirmed in all four cell lines (Additional file 1: Figure S1). The relative change in TNC expression after shRNA knockdown was most significant in cell lines with high starting endogenous TNC expression (DIPGIV and SF7761), with the greatest relative decrease in DIPGIV (Additional file 1: Figure S1a, b). Because *TNC* knockdown was greatest using shRNA234 compared to shRNA232, all subsequent experiments were conducted using the shRNA234 vector.

**Fig. 4:**
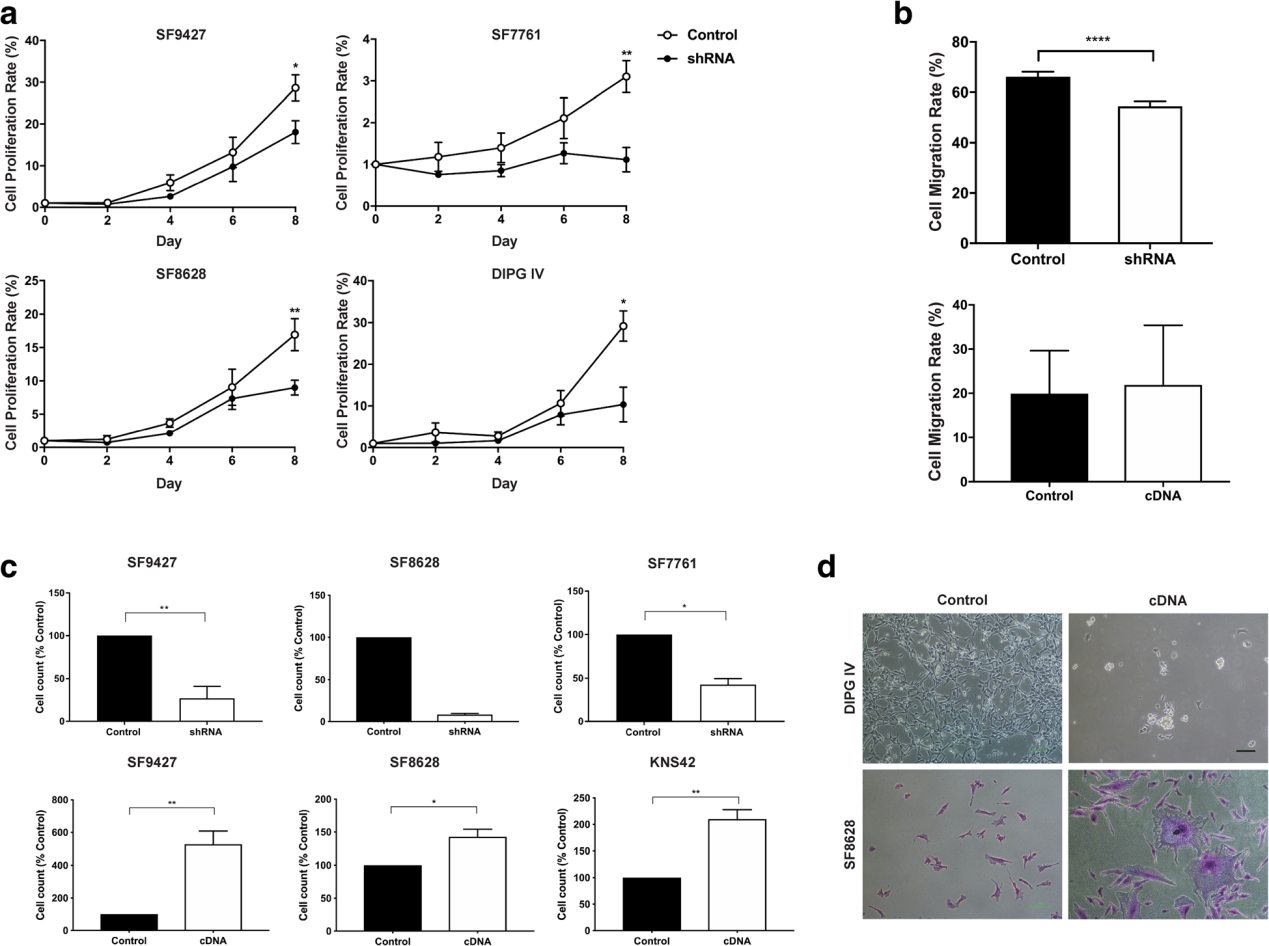
Altered TNC expression affects glioma cell proliferation, invasion and morphology in vitro. TNC expression was altered in pediatric supratentorial high-grade glioma (HGG *n* = 1) and brainstem glioma (DIPG n = 3) cell lines via lentiviral transfection of two distinct shRNA TNC constructs and one TNC cDNA. **a** TNC shRNA knockdown results in decreased cell proliferation, in all lines compared to empty vector controls (*p < 0.05, **p < 0.01, data for TNC shRNA 234 shown). *X-axis*: Time in days, day zero defined as day of plating. *Y-axis:* Cell proliferation calculated as number of cells collected on days 2, 4, 6, 8, relative to cell count on day 0 (percent). **b** TNC knockdown (shRNA) inhibits scratch gap closure on wound healing assay in all cell lines, with a mean decrease in cell motility of 17.7% compared to empty-vector controls (****p < 0.0001). TNC overexpression (via cDNA) did not significantly increase cell motility compared to empty vector controls. *Y-axis:* Cell migration rate relative to hour 0 (percent). **c** Top row: TNC knockdown results in decreased cell invasion compared to empty-vector controls (*p = 0.016, **p = 0.0067). Bottom row: Overexpressing TNC results in increased cell invasion compared to empty-vector controls (*p = 0.0227, SF9427 **p = 0.006, KNS42 **p = 0.0035) *Y-axis:* Cell count normalized to empty-vector controls. **d** Light microscopy demonstrates alteration of cell morphology after TNC cDNA transfection. SF8628 TNC cDNA cells appear larger and spread widely relative to control. DIPGIV cells changed from attached to suspended with TNC cDNA transfection. Cells stained by crystal violet. Scale bar = 100 μm. Error bars represent standard error of the mean

Compared to control and empty vector cell lines, cell proliferation after TNC knockdown was significantly decreased in all lines studied, with the greatest effect observed in those cell lines with higher baseline endogenous TNC expression (Fig. 4a). The effect of TNC knockdown on cell migration was evaluated in two adherent DIPG lines (SF8628, DIPG IV) via scratch (wound healing) assay. As expected, TNC knockdown inhibited closure of the scratch gap with a mean decrease in cell motility of 17.7% after TNC shRNA234 transfection versus empty-vector controls (*p* < 0.0001, t-test, Fig. 4b). TNC knockdown also resulted in decreased Matrigel cell invasion compared to empty-vector controls (SF9427 *p* = 0.016, SF7761 *p* = 0.0067, t-test, Fig. 4c).

Finally, the effect of increased TNC expression via cDNA expression was also evaluated via lentiviral transfection of two DIPG (DIPGIV, SF8628) and two HGG cell lines (SF9427, KNS42, (Additional file 1: Figure S1). Increased *TNC* mRNA and protein expression relative to empty vector controls was confirmed in all four cell lines (Additional file 1: Figure S1a, b). TNC cDNA transfection resulted in increased cell invasion but had no effect on cell migration (Fig. 4b, c). Interestingly, TNC cDNA transfection also resulted in altered H3K27 M mutant DIPG cell morphology, with SF8628 appearing larger relative to control and shRNA lines, and DIPGIV changing from an attached to a suspended cell line (Fig. 4d). Of note, DIPGIV has greater endogenous TNC expression (Fig. 2a,c). No change in cell morphology was observed with TNC cDNA transfection in HGG lines.

### Altered TNC expression affects gene transcription in vitro

Our previous analysis of protein and gene expression patterns in DIPG tumor tissue specimens revealed two molecular subgroups of DIPG with relative activation of either Sonic Hedgehog (SHh) or MYCN signaling [65], with TNC hypomethylation and increased expression in the SHh group. In this study, *TNC* expression was also associated with increased *GLI1*, and decreased *NOTCH1* and *MYCN* expression (ANOVA, *p* < 0.05, Fold change in expression <− 2 or > 2). Therefore, we performed qPCR for *TNC, NOTCH1, NOTCH2, MYCN* and *PDGFRA* transcripts in our DIPG (*n* = 4) and supratentorial pediatric HGG (*n* = 3) cell lines to determine relative gene expression patterns in vitro. We detected increased *NOTCH2* transcription in association with increased *TNC* transcription across cell lines studied, with inverse *NOTCH1, PDGFRA* and *MYCN* expression in five of seven (71%) cell lines (Additional file 2: Figure S2a). Interestingly, *NOTCH1* and *PDGFRA* expression directly correlated across all cell lines. However, these correlations did not meet statistical significance (Additional file 2: Figure S2b).

**Fig. 5:**
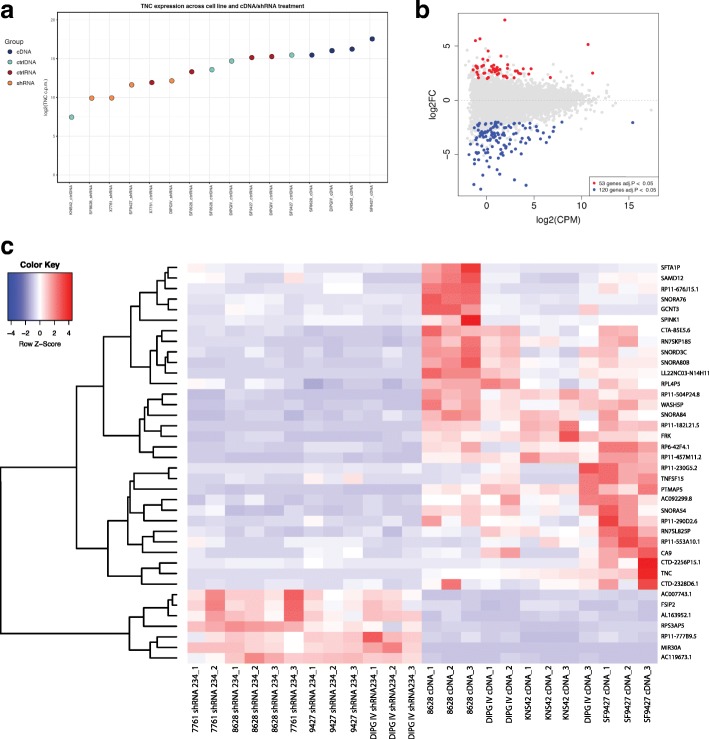
TNC expression level affects global gene expression in pediatric glioma cell lines. TNC expression was modified in pediatric supratentorial high-grade glioma (HGG n = 1) and brainstem glioma (DIPG n = 3) cell lines via lentiviral transfection of TNC shRNA and cDNA, with resulting gene expressions determined via RNA-Seq. **a** Dot plot representing relative TNC expression across cDNA and shRNA lines studied. As expected, cDNA lines expressed greater TNC compared to shRNA and respective controls. **b**MA plot demonstrating distribution of 173 statistically significantly differentially expressed genes between TNC cDNA and shRNA cell lines (Log_2_Fold Change > 2 or < − 2, adjusted p-value < 0.05). **c** Heat map depicting expression of 38 differentially expressed genes, including TNC and miRNA30, with consistent expression pattern across all lines by experimental condition. Cell lines are ordered on x axis by TNC expression level, as in **b**

To further evaluate the effect of *TNC* expression level on transcription, whole transcriptome sequencing analysis (RNA-Seq) was performed in triplicate on DIPG (n = 3) and HGG (*n* = 2) cell lines after TNC cDNA, shRNA, and empty vector control transfection (Additional file 5: Online Resource 1). Resulting transcriptomes of cell lines transfected with TNC cDNA were compared to TNC knockdown (shRNA) and controls (Additional file 6: Online Resource 2) to determine effects on gene expressions. As expected, cDNA lines expressed greater *TNC* compared to shRNA and respective controls (Fig. 5a). Overall, gene expression profiles clustered by technical replicate for a given cell line, followed by experimental condition, on unsupervised (Additional file 3: Figure S3a) and supervised hierarchical clustering by *TNC* expression level (Fig. 5a), and when adjusted for culture conditions (Additional file 3: Figure S3b). Given this, we performed explicit modeling for differentially expressed genes both as means and as sums across technical replicates, and observed minimal change in data sets when adjusted for culture conditions (Additional file 3: Figure S3b). Together, these data demonstrate that observed transcriptomes and resulting cell functions are indeed driven by induced differences in TNC expression in cDNA vs shRNA lines. Interestingly, TNC expression positively correlated with NOTCH2 and PDGFRA expression across control cell lines (Additional file 2: Figure S2c). Comparison of gene expression profiles revealed 173 statistically significantly differentially expressed genes between cDNA and shRNA lines (Log_2_ Fold Change in Expression, FC, > 2 or < − 2, adjusted *p*-value< 0.05, Additional file 6: Online Resource 2, Fig. 5b). Of these, 38 genes demonstrated a consistent pattern of expression across experimental conditions (Fig. 5c, Table 4), with *TNC* as a top upregulated gene (Log_2_FC − 5.130), and mir-30a the top downregulated gene (Log_2_FC − 7.866) in cDNA relative to shRNA lines.

**Table 4:** Differentially expressed genes in TNC cDNA vs. shRNA cell lines

Gene Name	logFC	PValue	adj.p
MIR30A	−7.866092897	2.51674E-50	3.9996E-46
AC119673.1	−5.51977944	3.22561E-14	7.32306E-11
RPS3AP5	−3.022216042	4.38211E-10	4.0965E-07
AC007743.1	−2.374800701	1.81455E-08	1.35278E-05
AL163952.1	−2.225772302	9.52366E-06	0.001872888
CTD-2269F5.1	−2.180041946	0.000403098	0.025023561
RP11-777B9.5	−2.123763807	2.03315E-07	0.000100971
FSIP2	−2.121252851	1.13045E-06	0.000403974
RPL4P5	2.070466961	0.0002471	0.018610989
SAMD12	2.077983472	0.000150356	0.014061765
TNFSF15	2.09066531	0.000821066	0.038724462
SNORA84	2.212964724	4.60974E-07	0.000203494
RN7SL825P	2.21448533	2.17857E-05	0.003532848
AC092299.8	2.347033959	1.55329E-05	0.002773582
GCNT3	2.370843556	0.000972904	0.043553199
RP6-42F4.1	2.453535316	2.25622E-08	1.494E-05
RP11-553A10.1	2.484443136	0.001201734	0.048910983
RP11-290D2.6	2.494377813	1.86588E-05	0.003188456
SNORA76	2.503062118	3.45134E-05	0.005126049
SNORD3C	2.578909604	5.76928E-06	0.00130979
SNORA54	2.680209334	1.90238E-11	2.51939E-08
CA9	2.683101502	0.000191849	0.016161347
CTA-85E5.6	2.749870719	4.52651E-09	3.99641E-06
FRK	2.751839555	1.18463E-08	9.90845E-06
RP11-504P24.8	2.777831969	2.00742E-10	2.2787E-07
RP11-676 J15.1	2.80969822	0.000726166	0.035617995
SPINK1	2.838338406	0.000284132	0.020068546
WASH5P	2.896364617	2.37249E-27	1.88518E-23
RN7SKP185	2.897676479	9.16004E-13	1.81964E-09
RP11-230G5.2	2.921550791	5.15934E-05	0.00717505
CTD-2256P15.1	3.208660084	0.000516546	0.029320523
SNORA80B	3.276449172	1.68241E-12	2.97077E-09
SFTA1P	3.678556083	2.99678E-06	0.000780734
RP11-457 M11.2	3.793167305	2.15375E-11	2.63288E-08
TNC	5.130171422	3.0407E-21	1.20807E-17
RP11-182 L21.5	5.497636404	5.54817E-12	8.01559E-09
PTMAP5	5.663770365	4.27628E-15	1.13264E-11
LL22NC03-N14H11.1	7.391276313	7.34761E-27	3.89227E-23

TNC expression was modified in pediatric supratentorial high-grade glioma (HGG n = 1) and brainstem glioma (DIPG n = 3) cell lines via lentiviral transfection of TNC shRNA and cDNA, with resulting gene expressions determined via RNA-Seq. Results were filtered for statistical significance (Log_2_Fold Change > 2 or < −2, adjusted p-value < 0.05), with 173 differentially expressed genes identified between TNC cDNA and shRNA cell lines. Of these, 38 genes, listed here, demonstrated the same expression pattern across all lines by experimental condition, including TNC and miRNA30, with differential expression values (cDNA vs shRNA, Log_2_Fold Change), pvalue and adjusted *p*-value indicated

**Fig. 6:**
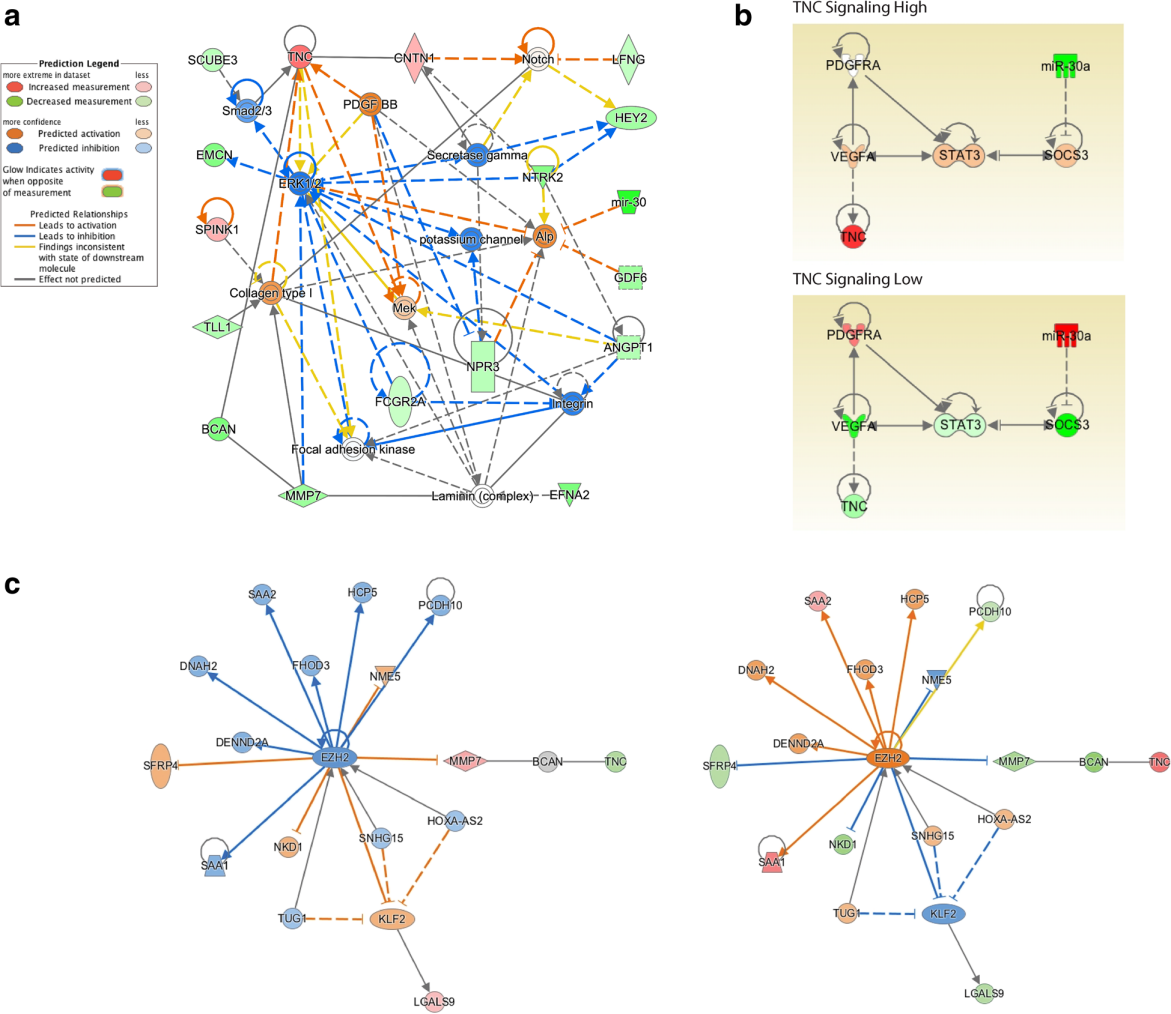
Functional pathways analysis reveals networks of molecular interaction and a potential mechanism of subgroup TNC overexpression in pediatric glioma Pathways analysis was performed to evaluate the biological functions and interactions of differentially expressed genes between TNC cDNA and shRNA cell lines (Log_2_Fold Change > 2 or < − 2, adjusted p-value < 0.05). **a** Organismal Development and Small Molecule Biochemistry is indicated as the top network of molecular interaction (22 focus molecules, score 45), with *TNC* over expression, *miRNA30a* downregulation, PDGF and NOTCH signaling activation, and interaction with extracellular matrix proteins. These results suggest that the observed biological effects of elevated TNC expression in vitro may be due to these pathway activations. **b** VEGFRA and STAT3 activation, potentially affected by miRNA30 expression level, are identified as potential upstream regulators of TNC overexpression in a subgroup of DIPGs lacking PDGFRA amplification or mutation. **c** EZH2 is implicated as a top activated upstream regulator in TNC cDNA (left) relative to TNC shRNA (right) cell lines (activation z-score 1.639, p-value of overlap 0.004), with a direct downstream effect on TNC expression

Functional pathway analysis of the 173 significantly differentially expressed genes between cDNA and shRNA lines mapped to Cancer as the top disease process (p5.59E-4 – 3.10E-10, 118 molecules), with Cellular Movement (p-value 5.32E-3 – 1.79E -9, 50 molecules) and Cellular Growth and Proliferation Interaction (p-value 5.30E-3 – 6.03E-7, 36 molecules) as the top molecular and cellular processes, respectively. Nervous System Development and Function was identified as the top represented physiological system process (*p*-value 5.59E-0 – 6.03E-7, 52 molecules). Top canonical pathways implicated by this gene set included Calcium Signaling (p-value 1.61E-4), Axonal Guidance Signaling (*p*-value 2.50E-4) and NOTCH signaling (*p*-value 1.25 E-3). Consistent with our analyses of DIPG tissues, this data set specifically implicated differential NOTCH1 signaling activity in cDNA vs shRNA cell lines (z-activation score − 0.586, p-value of overlap 1.02 E-04, 8 target molecules in data set). Organismal Development and Small Molecule Biochemistry was the top network of molecular interaction (22 focus molecules, score 45), with interaction between high *TNC* expression, low *miRNA30a* expression, PDGF and NOTCH signaling (Fig. 6 a, b). Importantly, enhancer of zest 2 (EZH2), the functional enzymatic component of the polycomb repressor-2 complex (PRC2), was identified as a top upstream regulator affecting TNC expression (activation z-score 1.639, p-value of overlap 4.19E-03, Fig. 6c). EZH2 is known to methylate H3K9 and H3K27, leading to transcriptional repression of the affected target genes. Functional pathways analysis specifically implicated EZH2 activation in TNC cDNA relative to shRNA lines.

## Discussion

Diffuse intrinsic pontine glioma (DIPG) is a devastating pediatric brain tumor and the most common cause of cancer death in children. New, more effective therapeutic approaches are needed to improve clinical outcomes of this challenging disease. Tenascin-C (TNC) is a large extracellular matrix (ECM) glycoprotein that mediates cell-cell and cell-matrix interactions [7], and functions in early cell fate determination in the central nervous system by guiding migrating neurons during brain development in the perivascular stem cell niche [45]. TNC binding on the cell surface is known to induce PDGFRB translocation and Notch signaling [56]. TNC overexpression is reported in adult high grade glioma (HGG) [22, 56, 70] and has been explored as a biomarker of disease and potential therapeutic target [63]. While pediatric glioma is biologically distinct from the adult disease, our previous work characterizing gene and protein expression profiles of pediatric brainstem (DIPG), cerebellar and supratentorial gliomas revealed increased expression of TNC in tumor specimens, suggesting this protein may play a role in DIPG tumor biology [65]. Further, this work revealed greater TNC gene and protein expression, as well as TNC promoter hypomethylation, in histone H3K27 M mutant tumors compared to wild-type. Since H3K27 M mutation is associated with global changes in DNA methylation, altered chromatin structure, and poorer clinical outcomes in pediatric diffuse midline glioma [12, 13], our findings suggest TNC overexpression in H3K27 M mutant tumors may be clinically and biologically relevant. We also demonstrated increased secreted TNC in the cerebrospinal fluid (CSF) from children with DIPG and high grade glioma (HGG) compared to normal controls [66], suggesting TNC may serve as a clinically detectable biomarker of disease and response to therapy. Therefore, in the present study we aimed to comprehensively characterize TNC expression patterns, clinicopathological correlates and biological effects in pediatric glioma, including DIPG.

First, we found statistically significantly increased TNC expression in DIPG tumor tissue compared to normal brain tissue from the same patient (Fig. 1a), and validated these findings via immunohistochemical staining of a large cohort of pediatric glioma tissue specimens (*n* = 50, Tables 2 and 3) and mouse xenograft tumors generated with DIPG cell line SF8628 (Fig. 1b). These data confirm increased tumor TNC expression, and statistically significant greater TNC expression with increasing tumor grade (WHO III and IV) (Fig. 1d). We also found that high TNC expression (H-score > 75%) was associated with poorer overall survival and tumor recurrence (Fig. 1 e, f). While some inter and intra-tumoral variability in TNC expression was observed, overall TNC tissue expression levels correlated directly with tumor recurrence, and inversely with progression free survival, supporting an association between tumor TNC expression and clinical outcome (Table 3). These findings are consistent with previous reports of elevated TNC expression in higher grade, clinically aggressive cancers, including supratentorial adult and pediatric HGG [2, 7, 8, 11, 19, 37, 56, 65]. Importantly, while direct analysis suggested TNC, H3K27 M mutation and high tumor grade were significant predictors of overall survival, only tumor grade and H3K27 M mutation were independent predictors of overall survival on multivariate analysis. Taken together, these results indicate that TNC is associated with, but is not an independent predictor of, these clinical outcomes.

We next evaluated the pattern and biological effects of TNC expression level in DIPG (*n* = 3) and pediatric HGG (*n* = 2) cell lines. While endogenous TNC gene and protein expression did vary across lines studied, greater TNC expression was associated with H3K27 M mutation, independent of cell culture conditions. Interestingly, DIPGIV, the only H3.1K27 M line in our cohort, exhibited the highest TNC expression of cell lines studied. DIPGIV also harbors a gain-of-function c.983G > T mutation corresponding to ACVR1/ALK2 p/G328 V, which is known to occur in H3.1K27 M pontine gliomas and results in hyper-activation of bone morphogenetic protein (BMP)/ACVR1 signaling [9, 73]. Further, we found high concentration of exogenous TNC had an anti-proliferative effect on H3 wild-type HGG cell lines with greater endogenous TNC expression, and a pro-proliferative effect on H3K27 M DIPG cell lines (Fig. 3). Decreased tumor cell proliferation with higher levels of exogenous TNC has also been reported in pancreatic cancer [60], suggesting a potential negative feedback loop regulating TNC effects in these cancers lacking the H3K27 M mutation. High TNC expression in DIPG may therefore be due to the downstream effects of H3K27 M and ACVR1 mutations, while its effect on tumor formation may be influenced by the tumor microenvironment in the developing brainstem relative to the cerebral hemispheres.

In multiple tumor types, TNC over-expression is associated with tumor cell proliferation and decreased adhesion [53]. Despite variability in starting endogenous TNC expression levels across our cell lines, TNC knockdown resulted in decreased cell proliferation and motility in all lines studied, with the largest effect observed in those with high endogenous TNC expression prior to knockdown (Fig. 4, Additional file 2: Figure S2a, b). Conversely, increased TNC expression via cDNA transfection resulted in increased cell migration, invasion and proliferation (Fig. 4b, c). In addition, TNC cDNA transfection resulted in altered H3K27 M mutant DIPG cell morphology (Fig. 4d), suggesting a unique effect of TNC overexpression on the extracellular matrix in the setting of H3K27 M mutation.

The results of whole transcriptome (RNA-Seq) analysis of control, TNC shRNA, and TNC cDNA pediatric glioma cell lines provide insight on the effects of TNC levels on gene expression. Functional pathways analyses of these data implicated differential PDGF, VEGF and NOTCH1 signaling activation in the setting of elevated TNC expression (Fig. 6a, b), suggesting a potential mechanism for the observed pro-proliferative, anti-adhesive effects of TNC expression in DIPG. TNC expression is known to increase responsiveness to PDGF signaling during normal brain development and disease, and promotes PDGF-induced proliferation of myofibroblasts, smooth muscle cell and fibroblasts [36, 38, 40, 72], while TNC binding of integrin stimulates PDGF-dependent cell proliferation in multiple human cancers [79]. TNC also contributes to the stem cell niche of the subventricular zone in the developing brain by regulating the maturation, survival and PDGF responsiveness of immature oligodendroglial precursor cells (OPCs), while TNC knockout results in OPC apoptosis and loss of PDGF signaling response in vitro and in vivo [18, 26, 27]. Strong co-expression of TNC and VEGF is also reported in tumor perivascular zones in adult HGG [3, 7, 8]. Our tissue IHC staining of pediatric glioma tissue also revealed high TNC in regions of tumor microvasculature proliferation (Fig. 1), but we saw no relationship between TNC and NOTCH1 or PDGFRA expression via IHC staining or on qPCR analysis.

Given the known inter- and intratumoral heterogeneity of DIPG, understanding the mechanism and effects of tumor subclonal diversity and communication, including the effects of differential PDGFRA and TNC expression by tumor cell subpopulations, is an important stem in identifying more effective treatment for this disease. Indeed, using single cell RNA-Seq of H3K27 M mutant pediatric glioma, Filbin et al identified tumor cell subpopulations with distinct gene expressions and developmental hierarchy [23]. They found that oligodendrocyte precursor cells (OPC-like) exhibited greater self-renewal and tumor-propagating potential, and gave rise to oligodendrocyte (OC-like) and astrocytic (AC-like) populations sustained by PDGFRA signaling. In contrast, AC-like cells were more differentiated, with *TNC* identified as one of the top 20 genes defining the AC-like state, an expression level significantly greater than all other populations (3.717log_2_ TPM, *p* = 0.0002, Additional file 4: Figure S4). High TNC expression by more differentiated AC-like cells (in the absence of PDGFRA amplification) may therefore potentiate OPC response to normal PDGFRA signaling, thereby maintaining OPC stemness and proliferation, and contributing to a specific phenotype in this subgroup of tumors.

Further, Puget et al recently analyzed 23 DIPG tissues, identifying molecular subgroups driven either by PDGFRA mutation / amplification with oligodendroglial features (group 1), or exhibiting mesenchymal, pro-angiogenic characteristics with *TNC* overexpression (group 2, Expression Log_2_ Ratio 1.495, *p*-value 6.57E-4) [62]. The group 2 phenotype was also largely driven by a STAT3 and ZNF238. As expected, we found significant overlap in gene expression patterns between group 2 tumor tissue and TNC cDNA lines (Upstream Regulator match score 63.25, p-value 9.74E-6), which implicated STAT3 signaling activation and extracellular matrix interaction on functional pathways analysis (Fig. 6a, b). Similarly, Hoeman et al recently characterized a novel genetic mouse model of DIPG, demonstrating brainstem glioma formation with ACVR1 R206H or G328 V, H3.1K27 M, and p53 deletion, but not with ACVR1 or H3.1K27 M mutations alone [34]. Importantly, in their model the introduction of the ACVR1 mutation resulted in STAT3 signaling activation and upregulation of mesenchymal markers, including TNC, independent of H3.1K27 M status. Similarly, we observed greatest TNC expression and STAT3 signaling activation in DIPGIV (H3.1K27 M, ACVR1H328V, Fig. 2c), the only cell line studied harboring an ACVR1 mutation [55]. Clinically, ACVR1 mutations are detected in up to 32% of DIPGs, and enrich with H3.1K27 M mutation: as such, ACVR1 mutations are associated with better overall survival, relative to H3.3K27 M mutant tumors [46, 73]. However, in the model reported by Hoeman et al, ACVR1 mutation resulted in increased TNC expression, increased tumor formation and decreased animal survival. These data are in accordance with our observation of an association between TNC expression and malignant tumor behavior in both H3.3K27 M specimens and our H3.1K27 M / ACVR1 mutant cell line. Further investigation of the relationship between H3K27 M and ACVR1 mutation, STAT3 signaling, and TNC expression is therefore currently underway.

We also observed increased *miRNA30a* expression with TNC knockdown. miRNA30a overexpression in adult glioma exerts an oncogenic effect by blocking expression of tumor suppressor genes *SEPT7* and *SOCS3* facilitating subsequent JAK/STAT signaling activation [15, 16, 30, 41, 43, 77]: further mechanistic investigation of the role of *miRNA30a* and STAT3 on TNC expression in this subgroup of pediatric glioma is therefore warranted. Taken together, these findings suggest that in DIPGs lacking PDGFRA amplification or mutation, TNC overexpression may contribute to the observed mesenchymal, pro-angiogenic phenotype through VEGF signaling and interaction with extracellular matrix and cell adhesion molecules (Fig. 6b). Given the known inter- and intratumoral heterogeneity of DIPG, understanding the mechanism and effects of tumor subclonal diversity and communication, including the effects of differential TNC expression by tumor cell subpopulations, will be an important stem in identifying more effective treatment.

Lastly, TNC expression in vitro was associated with activation of EZH2 (enhancer of zeste 2) on functional pathways analysis. As EZH2 inhibition has been reported as feasible a therapeutic strategy for DIPG treatment, TNC may serve as a biomarker of treatment response (Fig. 6c) [54]. Our recent report on the effects of BET / bromodomain inhibition on DIPG also revealed decreased *TNC* expression after treatment in vitro (log fold change − 3.522, adjusted *p*-value 8.04E-119), again suggesting TNC expression may serve as a useful biomarker of disease and treatment response [29, 32, 54, 61]. While serial tumor sampling is not clinically feasible in diffuse midline glioma, TNC is detectable in cerebrospinal fluid from children with DIPG [35]: measuring changes in TNC levels over time via a liquid biopsy approach may therefore represent a more clinically feasible approach for longitudinal monitoring of response to these promising epigenetic therapeutic approaches, and is therefore worthy of further investigation.

In conclusion, we characterized TNC expression in pediatric glioma tissue and cell lines, including DIPG, with higher endogenous expression levels in tumors harboring the H3K27 M mutation. TNC overexpression is associated with more malignant cell behavior, including increased cell proliferation and migration, and patterns of gene expression consistent with a pro-angiogenic, mesenchymal tumor phenotype. Our findings suggest TNC overexpression in pediatric high grade and diffuse midline glioma is clinically detectable and may significantly contribute to tumor biology. Given this, we are currently investigating the effects of TNC expression in vivo on tumor growth and animal survival in an animal xenograft model of pediatric glioma, and evaluating changes in TNC expression by DIPG cells in vitro in response to novel epigenetic therapies. Our results suggest TNC may serve as a feasible disease biomarker and novel therapeutic target for this challenging disease.

## Additional files

Additional file 1: **Figure S1.** TNC expression in pediatric glioma cell lines modified via shRNA and cDNA transfection in vitro. Pediatric supratentorial high-grade glioma (HGG *n* = 1) and brainstem glioma (DIPG *n* = 3) cell lines were modified via lentiviral transfection of TNC cDNA and shRNA constructs. Two distinct shRNA constructs, 232 and 234, were generated. **a)** *TNC* transcript level after transfection, measured via qPCR, demonstrating TNC shRNA knockdown (left) and cDNA amplification (right), relative to empty vector controls (**p = 0.005, ****p < 0.001). Y-axis: Normalized TNC mRNA expression level (percent GAPDH). **b)** TNC protein level after transfection, measured via western blot, demonstrating decreased protein expression after shRNA knockdown (upper panels) and increased expression cDNA amplification (lower panels), relative to empty vector controls. Densitometry analysis of western blot signal intensity confirms statistical significance of TNC expression patterns between transfected lines and empty vector controls (*p = 0.0148, **p = 0.002, ***p = 0.0001, ****p < 0.0001). Y-axis: TNC protein band intensity (expression normalized to controls). Error bars represent standard error of the mean. c) Fluorescent microscopy images showed transfection efficiency of > 95% in both cDNA and shRNA. Scale bar = 200 μm. (TIF 138230 kb)

Additional file 2: **Figure S2.** Targeted gene expression analysis in pediatric glioma cell lines. Levels of *TNC* and related transcripts were measured via qPCR in pediatric supratentorial highgrade glioma (HGG n = 3) and brainstem glioma (DIPG *n* = 5) cell lines. **a)** Expression levels of *TNC*, *Notch 1, Notch 2, MYCN* and *PDGFRA* across cell lines studied. Values are normalized to *GAPDH* expression levels in each cell line. Y-axis: Relative gene expression to GAPDH. Error bars represent standard error of the mean. **b)** Correlation between *TNC* expression each gene of interest across cell lines, as measured by qPCR. Positive correlation was found between *TNC* and *Notch 2,* and between *TNC* and *PDGFRA*, but did not reach statistical significance. **c)** Correlation between *TNC* transcription and genes of interest (*Notch 1, Notch 2, MYCN,* and *PDGFRA*) across cell lines, as measured by RNA-Seq. TNC expression positively correlated with *Notch2* (R2 = 0.817) and *PDGFRA (*R2 = 0.820). Each data point represents one glioma cell line. (TIF 101751 kb)

Additional file 3: **Figure S3.** Unsupervised hierarchical clustering of cell line gene expression profiles RNA-Seq analysis was performed in triplicate on DIPG (*n* = 3) and HGG (*n* = 2) cell lines after TNC cDNA, shRNA, and empty vector control transfection. Differentially expressed genes (Log2Fold Change > 2 or < − 2, adjusted *p*-value < 0.05, *X-axis*) were identified between cDNA and shRNA lines. **a)** Unsupervised analysis of resulting gene expression profiles by cell line (*Y-axis)* demonstrates clustering by technical replicate, followed by experimental condition. **b)** Scatterplot depicting differential TNC expression levels across shRNA and cDNA cell lines holds when adjusted for cell culture conditions (with or without serum FBS). **c)** Explicit modeling for differentially expressed genes both as means and as sums across technical replicates results in minimal change in data sets when adjusted for culture conditions. Together, these data demonstrate that observed transcriptomes and resulting cell functions are driven by induced differences in TNC expression. (TIF 136432 kb)

Additional file 4: **Figure S4.** Relative TNC expression differs across single-cell subpopulations. Single cell RNA-Seq of H3K27 M mutant pediatric glioma was performed by Filbin et al. [23], revealing three tumor cell subpopulations with distinct gene expressions and developmental hierarchy: oligodendrocyte precursor (OPC-like), oligodendrocyte (OC-like), and astrocytic (AC-like) cells. Subgroup analysis of these data revealed TNC as a top expressed gene in AC-like cells (3.717log2 TPM), with statistically significantly greater TNC expression in AC-like cells compared to other cell populations, regardless of PDGFRA amplification status (***p = 0.0002). *Y-axis:* mean gene expression (in log2 TPM/10). Error bars represent standard error of the mean. Relative gene expression of the three DIPG subpopulations was normalized to all DIPG cells. Published data analyzed and presented with permission of the Dr. Mariella Filbin. (TIF 17718 kb)

Additional file 5: **Online Resource 1.** Whole transcriptome (RNA-Seq) analysis of pediatric glioma cell lines. Whole transcriptome sequencing analysis (RNA-Seq) was performed in triplicate on DIPG (n = 3) and HGG (n = 2) cell lines after TNC cDNA, shRNA, and empty vector control transfection. (PDF 28227 kb)

Additional file 6: **Online Resource 2.** Differential gene expression analysis of RNA-Seq data Resulting transcriptomes of cell lines transfected with TNC cDNA were compared to TNC knockdown (shRNA) and controls. Gene count tables from HTSeq were used as input for EBSeq version 1.16.0 [45]. Genes with a log2 fold change > 1 were treated as up-regulated, while genes with a log2 fold change <− 1 were treated as down-regulated. Under a false discovery rate of 0.05, genes with an empirical Bayesian posterior probability for being differentially expressed greater than 0.95 were considered to be differentially expressed. (PDF 56 kb)
