# Supplementary figures and images for: Tenascin-C expression contributes to pediatric brainstem glioma tumor phenotype and represents a novel biomarker of disease
Source: Acta Neuropathol Commun. 2019 May 15;7:75. doi: 10.1186/s40478-019-0727-1 (PMC6518697; doi:10.1186/s40478-019-0727-1)

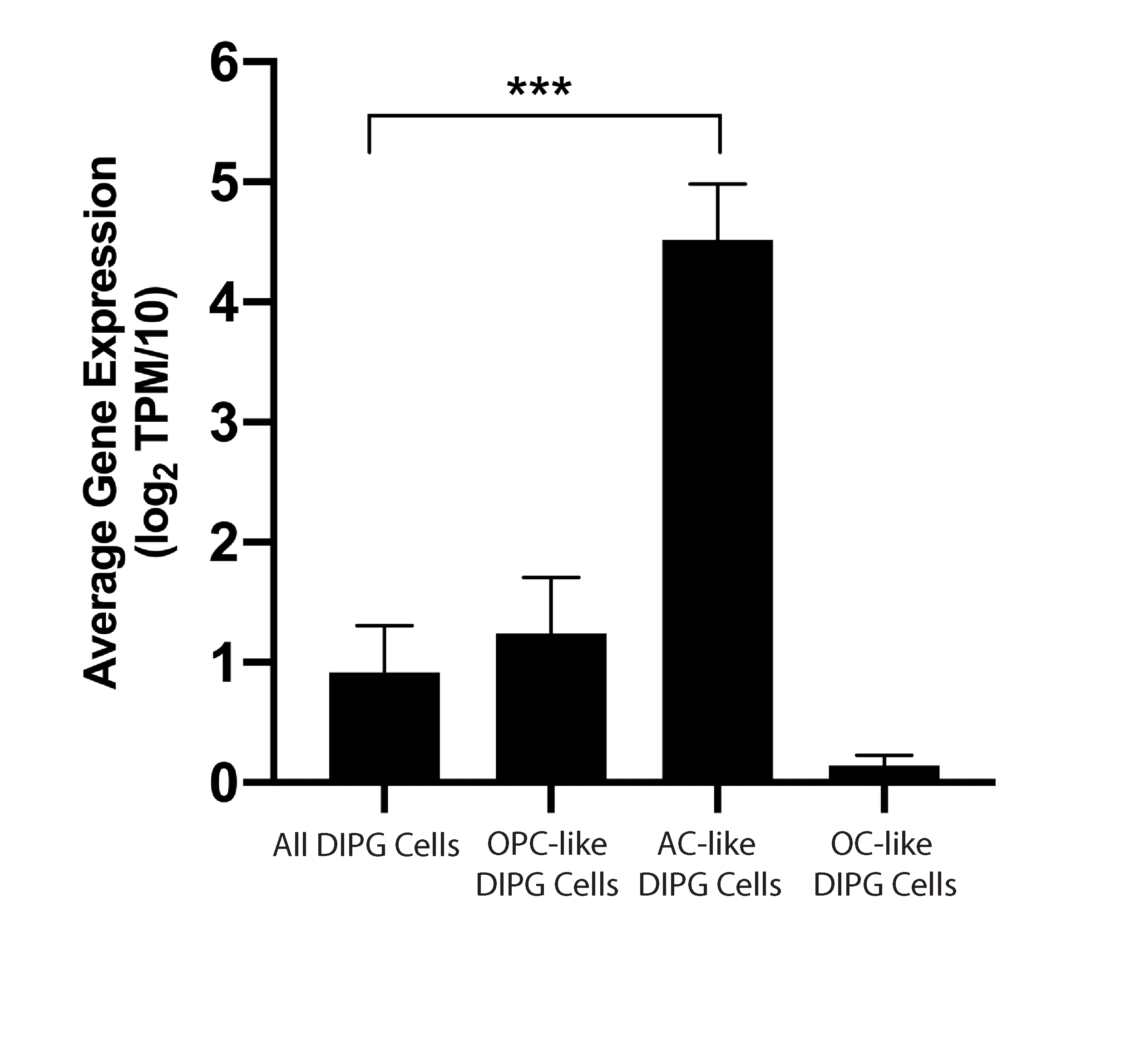

Supplement: Supplementary file 4 — Figure S4. Relative TNC expression differs across single-cell subpopulations. Single cell RNA-Seq of H3K27 M mutant pediatric glioma was performed by Filbin et al. [23], revealing three tumor cell subpopulations with distinct gene expressions and developmental hierarchy: oligodendrocyte precursor (OPC-like), oligodendrocyte (OC-like), and astrocytic (AC-like) cells. Subgroup analysis of these data revealed TNC as a top expressed gene in AC-like cells (3.717log2 TPM), with statistically significantly greater TNC expression in AC-like cells compared to other cell populations, regardless of PDGFRA amplification status (***p = 0.0002). Y-axis: mean gene expression (in log2 TPM/10). Error bars represent standard error of the mean. Relative gene expression of the three DIPG subpopulations was normalized to all DIPG cells. Published data analyzed and presented with permission of the Dr. Mariella Filbin. (TIF 17718 kb) [file 40478_2019_727_MOESM4_ESM.tif]
