# Supplementary material for: Tenascin-C expression contributes to pediatric brainstem glioma tumor phenotype and represents a novel biomarker of disease
Source: Acta Neuropathol Commun. 2019 May 15;7:75. doi: 10.1186/s40478-019-0727-1 (PMC6518697; doi:10.1186/s40478-019-0727-1)
Supplement: Supplementary file 6 — Online Resource 2. Differential gene expression analysis of RNA-Seq data Resulting transcriptomes of cell lines transfected with TNC cDNA were compared to TNC knockdown (shRNA) and controls. Gene count tables from HTSeq were used as input for EBSeq version 1.16.0 [45]. Genes with a log2 fold change > 1 were treated as up-regulated, while genes with a log2 fold change <− 1 were treated as down-regulated. Under a false discovery rate of 0.05, genes with an empirical Bayesian posterior probability for being differentially expressed greater than 0.95 were considered to be differentially expressed. (PDF 56 kb) [file 40478_2019_727_MOESM6_ESM.pdf]

Article Name: Tenascin-C Expression Contributes to Pediatric Brainstem Glioma Tumor Phenotype and Represents a Novel Biomarker of Disease
Journal Name: Acta Neuropathologica Communications
Authors: Qi J, Esfahani DR, Huang T, Ozark P, Bartom E, Hashizume R, Bonner ER, Horbinski CM, James CD, **Saratsis AM***
***Corresponding Author** Amanda M. Saratsis, MD
Attending Physician, Division of Pediatric Neurosurgery
Ann & Robert H. Lurie Hospital of Chicago
Assistant Professor, Department of Neurological Surgery
Department of Biochemistry & Molecular Genetics
Northwestern University Feinberg School of Medicine
asaratsis@luriechildrens.org

Gene ID	logFC	PValue	adj.p	gene
ENSG00000002587	-4.515103544	6.98E-08	3.96E-05	HS3ST1
ENSG00000005108	-3.184392786	0.000109236	0.011871293	THSD7A
ENSG00000006016	-3.351783681	0.000125692	0.01280448	CRLF1
ENSG00000009694	2.806837549	0.001156123	0.047744528	TENM1
ENSG00000011201	-2.848035405	1.68E-05	0.00292924	KAL1
ENSG00000018236	2.730963493	0.001204863	0.048910983	CNTN1
ENSG00000038295	-2.913168924	2.04E-05	0.003385226	TLL1
ENSG00000041982	5.130171422	3.04E-21	1.21E-17	TNC
ENSG00000058404	2.931026015	0.000279262	0.01984896	CAMK2B
ENSG00000060718	-2.48839719	0.000710428	0.03517171	COL11A1
ENSG00000068976	-2.230708372	0.000522112	0.029528128	PYGM
ENSG00000072163	-2.642341255	9.55E-06	0.001872888	LIMS2
ENSG00000073282	-4.50340168	2.52E-06	0.000689278	TP63
ENSG00000082458	-2.285818989	0.000662089	0.033616342	DLG3
ENSG00000085741	-3.696644152	5.62E-05	0.007702039	WNT11
ENSG00000086967	-6.840985947	8.31E-05	0.009919787	MYBPC2
ENSG00000089472	-4.909439033	7.17E-05	0.009263983	HEPH
ENSG00000099617	-4.817258445	3.85E-06	0.000957187	EFNA2
ENSG00000101846	2.411811341	9.30E-06	0.001872888	STS
ENSG00000102349	-3.000842111	0.000339775	0.022405405	KLF8
ENSG00000102468	-3.594972974	0.000470591	0.027658473	HTR2A
ENSG00000102935	-4.536743455	0.000424038	0.026221032	ZNF423
ENSG00000103460	-7.787467146	6.96E-07	0.000291158	TOX3
ENSG00000106003	-2.20785227	0.000167245	0.014914132	LFNG
ENSG00000106483	-2.898835151	0.000515139	0.029320523	SFRP4
ENSG00000106819	-4.946910554	0.000123782	0.012691249	ASPN
ENSG00000107159	2.683101502	0.000191849	0.016161347	CA9
ENSG00000108231	-5.484089541	0.000990155	0.043709862	LGI1
ENSG00000108950	-3.720860866	0.000133303	0.013165329	FAM20A
ENSG00000109846	-3.457848504	0.000182782	0.015873048	CRYAB
ENSG00000110195	-6.158084588	4.75E-08	2.91E-05	FOLR1
ENSG00000110446	-3.173200605	7.40E-06	0.001599304	SLC15A3
ENSG00000111816	2.751839555	1.18E-08	9.91E-06	FRK
ENSG00000111859	-2.524824716	0.000123301	0.012691249	NEDD9
ENSG00000112936	-3.31950692	0.000122161	0.012691249	C7
ENSG00000113389	-2.578184154	2.87E-05	0.004424978	NPR3
ENSG00000115844	-2.189321217	0.000308847	0.021270835	DLX2
ENSG00000116574	-3.229227821	5.11E-05	0.00717505	RHOU
ENSG00000118733	-5.019406317	0.000866122	0.040364847	OLFM3
ENSG00000121101	2.002517032	0.000500603	0.028824551	TEX14
ENSG00000122585	-7.352034453	0.000137445	0.013268531	NPY
ENSG00000127083	-5.622541011	0.000377974	0.023931305	OMD
ENSG00000130303	-2.818069777	1.88E-06	0.000568367	BST2
ENSG00000130528	-4.221722673	0.000216676	0.017131409	HRC
ENSG00000131620	-6.429215003	0.000185625	0.016032359	ANO1
ENSG00000132000	-3.0993064	4.95E-05	0.007094127	PODNL1
ENSG00000132530	-3.955452471	2.44E-06	0.000679604	XAF1
ENSG00000132692	-4.856971706	1.96E-08	1.36E-05	BCAN
ENSG00000134339	3.115803879	0.000342711	0.022424787	SAA2
ENSG00000134962	3.118705376	0.00034944	0.022666548	KLB
ENSG00000135439	-3.125205703	0.000259888	0.019073348	AGAP2
ENSG00000135547	-3.40240372	0.000693701	0.034667594	HEY2
ENSG00000136160	3.228829039	3.80E-05	0.005538796	EDNRB

ENSG00000137558	-4.952858293	6.68E-05	0.008777811	PI15
ENSG00000137573	-3.558944499	4.28E-05	0.006186156	SULF1
ENSG00000137673	-3.651425491	0.000251107	0.018823564	MMP7
ENSG00000137857	-3.010527635	5.19E-05	0.00717505	DUOX1
ENSG00000138435	-4.839497848	2.24E-06	0.000651525	CHRNA1
ENSG00000138650	-2.430779435	0.000104885	0.011679507	PCDH10
ENSG00000140254	-3.049672743	0.001142267	0.047645437	DUOXA1
ENSG00000140297	2.370843556	0.000972904	0.043553199	GCNT3
ENSG00000140807	-4.060014287	0.000542864	0.029710994	NKD1
ENSG00000142303	-2.335135026	2.31E-06	0.000656171	ADAMTS10
ENSG00000142408	-2.368286116	1.58E-06	0.000512063	CACNG8
ENSG00000142765	-2.019890298	0.000327682	0.02197264	SYTL1
ENSG00000143226	-2.032314346	0.000989398	0.043709862	FCGR2A
ENSG00000144285	-3.116426982	0.00062264	0.032656759	SCN1A
ENSG00000144481	2.937162114	0.000374521	0.023807521	TRPM8
ENSG00000146197	-2.538622911	0.000441126	0.026781686	SCUBE3
ENSG00000148053	-4.413574863	0.000604191	0.031794064	NTRK2
ENSG00000149131	-4.74062248	0.000237116	0.018060735	SERPING1
ENSG00000149527	-2.722756547	0.000439085	0.026781686	PLCH2
ENSG00000154188	-2.781543331	0.000471649	0.027658473	ANGPT1
ENSG00000154493	2.064804868	9.52E-05	0.011086842	C10orf90
ENSG00000155511	-3.331752301	0.001038321	0.044881637	GRIA1
ENSG00000156466	-3.512731925	0.000401232	0.025005419	GDF6
ENSG00000156687	-5.302803447	2.10E-05	0.00343902	UNC5D
ENSG00000156966	-2.534327637	0.000133779	0.013165329	B3GNT7
ENSG00000157601	-2.87410569	1.00E-06	0.000369721	MX1
ENSG00000159217	-3.216748778	3.74E-06	0.000942776	IGF2BP1
ENSG00000160588	-2.336797156	0.000656686	0.033616342	MPZL3
ENSG00000163485	-2.490972098	0.001135686	0.047620896	ADORA1
ENSG00000163536	-3.002747292	0.00027354	0.019670155	SERPINI1
ENSG00000164035	-4.599786702	7.72E-06	0.001628697	EMCN
ENSG00000164161	-2.516240118	0.001059422	0.045380954	HHIP
ENSG00000164266	2.838338406	0.000284132	0.020068546	SPINK1
ENSG00000164434	-5.890621771	1.14E-07	6.27E-05	FABP7
ENSG00000164794	3.020531718	9.56E-05	0.011086842	KCNV1
ENSG00000165949	-3.913210484	0.00016049	0.014742849	IFI27
ENSG00000168309	3.159381956	0.000596029	0.031468768	FAM107A
ENSG00000168427	-3.028943165	0.000734645	0.035812818	KLHL30
ENSG00000168675	-4.145450806	1.79E-06	0.000559262	LDLRAD4
ENSG00000168961	-3.032083746	1.14E-06	0.000403974	LGALS9
ENSG00000169783	-6.935876729	1.35E-06	0.000447046	LINGO1
ENSG00000172201	-2.134747266	0.00093722	0.042555163	ID4
ENSG00000173227	-3.046717983	6.55E-05	0.008680936	SYT12
ENSG00000173432	4.546419394	0.000379784	0.023950478	SAA1
ENSG00000175084	-4.027718182	0.000132887	0.013165329	DES
ENSG00000175093	-3.46389659	0.000557979	0.03026417	SPSB4
ENSG00000177570	2.077983472	0.000150356	0.014061765	SAMD12
ENSG00000178429	-3.022216042	4.38E-10	4.10E-07	RPS3AP5
ENSG00000181634	2.09066531	0.000821066	0.038724462	TNFSF15
ENSG00000183486	-2.126872662	0.000588992	0.031200857	MX2
ENSG00000184524	-2.171336605	0.00070846	0.03517171	CEND1
ENSG00000185008	-3.856038699	0.000300802	0.020874847	ROBO2
ENSG00000185275	-3.899940604	0.000191792	0.016161347	CD24P4
ENSG00000186369	3.072682611	0.000525708	0.029585735	LINC00643
ENSG00000186462	2.600242872	7.76E-05	0.009633761	NAP1L2
ENSG00000186832	-4.8210773	0.000625437	0.03268943	KRT16
ENSG00000187151	-4.840929644	0.001036723	0.044881637	ANGPTL5
ENSG00000187634	-4.274259678	6.75E-08	3.96E-05	SAMD11
ENSG00000188738	-2.121252851	1.13E-06	0.000403974	FSIP2
ENSG00000188859	-2.942185085	3.39E-05	0.005122651	FAM78B
ENSG00000196562	-3.484838954	0.000672965	0.033951617	SULF2
ENSG00000198216	-3.191087546	0.000234423	0.018060735	CACNA1E
ENSG00000198695	-2.12696367	9.64E-07	0.000369721	MT-ND6
ENSG00000199683	2.897676479	9.16E-13	1.82E-09	RN7SKP185
ENSG00000205517	-3.696863118	0.000201685	0.016436789	RGL3
ENSG00000206532	2.484443136	0.001201734	0.048910983	RP11-553A10.1

ENSG00000206579	2.99725314	2.56E-06	0.000689461	XKR4
ENSG00000206633	3.276449172	1.68E-12	2.97E-09	SNORA80B
ENSG00000207008	2.680209334	1.90E-11	2.52E-08	SNORA54
ENSG00000207827	-7.866092897	2.52E-50	4.00E-46	MIR30A
ENSG00000211459	-2.07648852	1.19E-05	0.002279091	MT-RNR1
ENSG00000213622	-2.225772302	9.52E-06	0.001872888	AL163952.1
ENSG00000214182	5.663770365	4.28E-15	1.13E-11	PTMAP5
ENSG00000223764	-4.282453363	2.94E-06	0.000777387	RP11-54O7.3
ENSG00000224259	-2.762942782	0.00013255	0.013165329	LINC01133
ENSG00000225373	2.896364617	2.37E-27	1.89E-23	WASH5P
ENSG00000225383	3.678556083	3.00E-06	0.000780734	SFTA1P
ENSG00000225971	2.749870719	4.53E-09	4.00E-06	CTA-85E5.6
ENSG00000226958	2.497643191	8.32E-06	0.00171644	CTD-2328D6.1
ENSG00000230207	2.070466961	0.0002471	0.018610989	RPL4P5
ENSG00000231231	-2.966707919	0.00044153	0.026781686	AP001422.3
ENSG00000231672	2.458663067	1.48E-05	0.002679096	DIRC3
ENSG00000233251	-2.374800701	1.81E-08	1.35E-05	AC007743.1
ENSG00000233631	3.793167305	2.15E-11	2.63E-08	RP11-457M11.2
ENSG00000234961	2.093573547	0.000130321	0.013165329	RP11-124N14.3
ENSG00000235750	-5.104587909	2.55E-05	0.004014153	KIAA0040
ENSG00000238266	2.844854708	0.00017214	0.015198011	LINC00707
ENSG00000239183	2.212964724	4.61E-07	0.000203494	SNORA84
ENSG00000239731	2.21448533	2.18E-05	0.003532848	RN7SL825P
ENSG00000240583	-3.615863734	0.000450565	0.027020326	AQP1
ENSG00000241781	-3.393135482	7.92E-07	0.000322911	AL161626.1
ENSG00000242094	-2.360697584	0.000327148	0.02197264	FOXP1-IT1
ENSG00000242173	-2.544688091	0.000884364	0.041094474	ARHGDIG
ENSG00000247317	-2.500575557	0.000778099	0.037245618	RP11-273G15.2
ENSG00000248968	3.208660084	0.000516546	0.029320523	CTD-2256P15.1
ENSG00000249072	-2.123763807	2.03E-07	0.000100971	RP11-777B9.5
ENSG00000249669	-3.64421322	3.23E-07	0.000150918	MIR143HG
ENSG00000250241	-2.12878452	0.001129656	0.047619358	RP11-9G1.3
ENSG00000250320	-2.180041946	0.000403098	0.025023561	CTD-2269F5.1
ENSG00000250748	2.921550791	5.16E-05	0.00717505	RP11-230G5.2
ENSG00000251705	-2.017588165	0.000254461	0.018937028	RNA5-8SP6
ENSG00000253864	-3.422371465	5.07E-06	0.001185137	AC131025.8
ENSG00000255495	4.069164656	1.48E-07	7.83E-05	FAM85A
ENSG00000259869	-5.369451323	3.62E-20	1.15E-16	AL022344.7
ENSG00000263682	-8.225961846	5.24E-06	0.001207217	RP11-93H10.1
ENSG00000263958	2.80969822	0.000726166	0.035617995	RP11-676J15.1
ENSG00000264940	2.578909604	5.77E-06	0.00130979	SNORD3C
ENSG00000266402	2.503062118	3.45E-05	0.005126049	SNORA76
ENSG00000268313	-5.51977944	3.23E-14	7.32E-11	AC119673.1
ENSG00000269720	-4.059275059	0.000119952	0.012624368	CTD-2521M24.5
ENSG00000269936	-3.749520666	1.28E-06	0.000441879	MIR145
ENSG00000270562	-3.420637539	1.87E-08	1.35E-05	RP11-154H23.3
ENSG00000272098	2.347033959	1.55E-05	0.002773582	AC092299.8
ENSG00000272316	2.615020542	1.53E-07	7.84E-05	XXbac-BPGBPG55C20.2
ENSG00000272489	5.497636404	5.55E-12	8.02E-09	RP11-182L21.5
ENSG00000272645	2.777831969	2.01E-10	2.28E-07	RP11-504P24.8
ENSG00000272667	2.94518142	3.62E-07	0.000164142	RP11-395A13.2
ENSG00000272872	7.391276313	7.35E-27	3.89E-23	LL22NC03-N14H11.1
ENSG00000273131	2.453535316	2.26E-08	1.49E-05	RP6-42F4.1
ENSG00000273149	2.494377813	1.87E-05	0.003188456	RP11-290D2.6
