# Supplementary material for: Tenascin-C expression contributes to pediatric brainstem glioma tumor phenotype and represents a novel biomarker of disease
Source: Acta Neuropathol Commun. 2019 May 15;7:75. doi: 10.1186/s40478-019-0727-1 (PMC6518697; doi:10.1186/s40478-019-0727-1)
Supplement: Supplementary file 5 — Online Resource 1. Whole transcriptome (RNA-Seq) analysis of pediatric glioma cell lines. Whole transcriptome sequencing analysis (RNA-Seq) was performed in triplicate on DIPG (n = 3) and HGG (n = 2) cell lines after TNC cDNA, shRNA, and empty vector control transfection. (PDF 28227 kb) [file 40478_2019_727_MOESM5_ESM.pdf]

Article Name: Tenascin-C Expression Contributes to Pediatric Brainstem Glioma Tumor Phenotype and Represents a Novel Biomarker of Disease
Journal Name: Acta Neuropathologica Communications

Authors: Qi J, Esfahani DR, Huang T, Ozark P, Bartom E, Hashizume R, Bonner ER, Horbinski CM, James CD, **Saratsis AM***
***Corresponding Author:** Amanda M. Saratsis, MD
Attending Physician, Division of Pediatric Neurosurgery
Ann & Robert H. Lurie Hospital of Chicago
Assistant Professor, Department of Neurological Surgery
Department of Biochemistry & Molecular Genetics
Northwestern University Feinberg School of Medicine
asaratsis@luriechildrens.org

	X7761.234.RNA.170302	X7761.234.RNA.170321	X7761.234.RNA.170418	X7761.control.RNA.170216	X7761.control.RNA.170302	X7761.control.RNA.170315	X8628.234.RNA.170118	X8628.234.RNA.170123	X8628.234.RNA.170126	X8628.control.RNA.161209	X8628.control.RNA.170123	[illegible]
ENSG00000000003	7.3513403	11.1963387	10.3173689	8.3095155	4.3181973	8.08065909	10.0630846	10.0529203	8.82649535	9.41814529	10.1233727	[illegible]
ENSG00000000005	0.11994055	0.06886121	0.11729971	0.132889	0.04778957	0.16708787	0	0	0	0	0.02158625	[illegible]
ENSG00000000419	3.5049783	3.04377062	3.25225781	2.8942068	3.89449318	3.1642184	10.3372245	11.5949924	11.8168802	11.0595439	12.9791541	[illegible]
ENSG00000000457	1.2028079	1.57044324	1.25140907	1.3791901	1.19036303	1.20940714	1.44812666	1.38833903	1.26620274	1.14824926	1.4458822	[illegible]
ENSG00000000460	1.94651669	1.36963676	1.57084092	1.1850509	1.93514468	1.24446698	0.8811398	1.16811534	1.5874639	0.65099632	0.81495775	[illegible]
ENSG00000000938	0	0.00150031	0	0.0057906	0	0	0.00899399	0.00219399	0	0	0.00846558	[illegible]
[illegible]	[illegible]	[illegible]	[illegible]	[illegible]	[illegible]	[illegible]	[illegible]	[illegible]	[illegible]	[illegible]	[illegible]	[illegible]

ENSG00000006837	0.10863402	0.10142971	0.07887673	0.1021252	0.07345257	0.12346831	0.09517227	0.09189772	0.0838166	0.08497517
ENSG00000007001	0	0	0	0	0	0	0	0.00705301	0	0.00978258
ENSG00000007038	0	0	0	0	0	0	0	0	0	0
ENSG00000007047	2.5579505	2.43846199	2.39703933	3.3092709	2.17927803	2.91255434	1.99112657	2.34947154	1.93686729	2.87227079
ENSG00000007062	0.01299656	0.00232141	0.00932096	0.0089598	0.02174928	0.01299873	0	0	0	0.00078475
ENSG00000007080	7.99159795	6.28236306	7.24110558	7.1878459	8.31121241	6.00989206	6.5805343	8.15523375	10.5058935	8.31151253
ENSG00000007129	0	0	0	0	0	0	0	0	0	0
ENSG00000007168	11.1241535	12.3177089	11.3171851	10.953494	11.443661	11.2360494	7.68410486	8.80695563	8.54106989	6.65923846
ENSG00000007171	0.15435699	0.12406891	0.18869796	0.2095007	1.19253508	0.39367586	0.01859403	0.0075597	0.00381564	0.02097069
ENSG00000007174	0.01931125	0.00746249	0.00871664	0.0172814	0.02941647	0.01727159	0.0101401	0.0109128	0.00477366	0.0040363
ENSG00000007202	6.32349783	6.25222561	5.82944055	4.9735969	6.53327439	4.99701355	10.2533305	10.4574714	10.8561253	9.6939936
ENSG00000007216	0	0	0	0	0	0	0	0	0	0
ENSG00000007237	0.25478662	0.26832705	0.31522358	0.0406717	0.0522268	0.10016623	0.0006163	0.0022511	0.0034147	0.00347539
ENSG00000007255	1.83020675	2.09480969	1.72106666	3.4056487	1.66041085	2.50438108	1.21944362	1.01455118	0.72088777	2.21324902
ENSG00000007264	0.00220918	0.00295949	0	0.0028556	0.00246466	0.00331432	0	0.00216391	0.0010922	0
ENSG00000007306	0	0	0	0	0	0	0	0	0	0
ENSG00000007312	0	0	0	0	0	0	0.00511924	0	0	0
ENSG00000007314	0	0	0	0.0024866	0	0	0.01544878	0.01318998	0.01141275	0.03484685
ENSG00000007341	0.29220249	0.29640361	0.28196698	0.2964698	0.29346646	0.2984072	0.25859479	0.28494542	0.25657289	0.3127734
ENSG00000007350	0.07599795	0.12615496	0.1680383	0.1922013	0.03133416	0.12145173	0	0.00323655	0	0

[illegible]

ENSG00000013619	0.83711745	0.91272401	0.74566411	0.8185142	0.75610989	0.74857654	0.18680516	0.2306586	0.25910846	0.23799299	[illegible]
ENSG00000013725	0	0.00086442	0.00252423	0.0033363	0.00287954	0.00290418	0.00414557	0.00379226	0.00638028	0.00116886	[illegible]
ENSG00000013810	8.23505936	6.02560126	6.97863149	6.0057652	8.70991358	5.8646798	2.2823311	3.15912061	5.18736587	3.13142952	[illegible]
ENSG00000014123	11.0690776	9.94098839	9.68088709	10.464115	10.0259918	9.63541876	10.0612697	10.7545947	12.5240299	10.4681835	[illegible]
ENSG00000014138	3.07235113	2.28375948	2.42111907	1.4136366	2.8646209	1.59744485	1.62825823	2.31190868	3.06201545	1.17536346	[illegible]
ENSG00000014164	4.11927411	4.19233586	3.32831236	3.8655382	3.48197082	3.90511541	4.05223541	4.29065059	3.58163374	3.4867698	[illegible]
ENSG00000014216	1.6279494	1.49678368	1.45098233	1.560503	1.43244751	1.49329768	2.04836866	2.11616546	1.96036321	1.545341	[illegible]
ENSG00000014257	0.00339618	0.00227481	0.0033214	0	0.00378892	0.00254756	0.00545477	0.00665317	0	0	[illegible]
ENSG00000014641	12.2901003	8.3004987	10.3622218	11.376306	15.0056611	10.8634588	6.39613646	6.86076041	6.59168219	7.56000097	[illegible]
ENSG00000014824	9.06922486	9.48507439	9.28826048	10.04404	10.0550784	8.82552119	10.2290976	11.0029208	10.6308416	11.4003176	[illegible]
ENSG00000014914	1.40826406	1.19386367	1.20346647	1.2072312	0.87574252	1.25682435	0.67277065	0.72111255	0.57997449	0.74666173	[illegible]
ENSG00000014919	7.22390719	7.82809531	6.96760252	8.8992099	6.76393753	8.48584774	11.4523579	13.023047	10.5523686	11.846014	[illegible]
ENSG00000015133	0.83943511	1.44009186	0.90193076	1.4522933	0.69999311	1.36430916	0.41043921	0.47264435	0.50676328	0.69176539	[illegible]
ENSG00000015153	0.67194965	0.71359859	0.73452935	0.6318171	0.5580098	0.57314924	0.45607088	0.48089606	0.47794815	0.37399454	[illegible]
[illegible]											

[illegible]								
ENSG00000036828	0	0	0	0	0	0	0	[illegible]
ENSG00000037042	1.74611383	2.27526511	1.8152941	2.7691612	1.54287958	2.81772835	1.99144496	[illegible]
ENSG00000037241	2.45069009	2.10186103	2.59129945	1.8235324	2.79967212	2.08252893	2.82504075	[illegible]
ENSG00000037280	3.09567079	1.36103549	1.862715	0.9984012	4.34609718	1.44341777	0.01671132	[illegible]
ENSG00000037474	1.59191828	1.321804	1.25324603	1.2231174	1.79535635	1.15078188	4.38432357	[illegible]
ENSG00000037637	3.89514502	4.02514049	3.97793859	4.0769564	3.51236449	4.52067193	2.38297933	[illegible]
ENSG00000037749	4.39361837	4.36832337	3.97414839	3.9730244	4.48752877	4.02409893	5.0386361	[illegible]
ENSG00000037757	1.30809792	1.87519908	1.35501786	1.7646144	1.3138874	1.77600801	3.00750769	[illegible]
ENSG00000037897	0.59960075	0.552632	0.51603092	0.6758471	0.74921295	0.50015029	1.00927487	[illegible]
ENSG00000037965	0.02868265	0	0	0	0	0	0	[illegible]
ENSG00000038002	2.04356363	2.2410112	2.4129059	2.2791376	2.75949689	2.233962	4.04779821	[illegible]
ENSG00000038210	1.8381424	1.55496319	1.5899713	1.4104623	1.89751944	1.23600866	2.1877837	[illegible]
ENSG00000038219	2.95506767	4.39708213	3.33772731	3.6339087	2.92895175	3.57915301	8.33519131	[illegible]
ENSG00000038274	3.76588317	3.59422457	3.62345677	3.6588939	3.21452055	3.58224194	3.45503406	[illegible]
ENSG00000038295	0.00665011	0.0167038	0.00812961	0.0365332	0.05657096	0.02993046	0.02937293	[illegible]
ENSG00000038358	2.92040573	2.95343272	2.43892057	2.6315185	2.68765134	2.3763015	1.85669925	[illegible]
ENSG00000038382	3.74701066	3.27451145	3.05558122	1.5247778	3.44145511	2.08934395	2.78333776	[illegible]
ENSG00000038427	4.49079019	5.67145842	5.41026749	2.3464206	1.84085093	2.53843643	0.66314902	[illegible]
ENSG00000038532	1.01950271	1.22358878	0.99251811	0.9625079	1.14192953	0.96660076	1.07581478	[illegible]
ENSG00000038945	0.00939423	0.00125848	0.00183748	0.0097145	0.00628836	0.00563748	0.02866824	[illegible]
ENSG00000039068	0.01080189	0.09095773	0.03471045	0.0498668	0.01377262	0.03125351	0.0161102	[illegible]
ENSG00000039123	4.4830841	4.60112702	4.17222852	3.6098195	4.32454837	3.83637242	5.29522676	[illegible]
ENSG00000039139	0.00673206	0.00338193	0.00329192	0	0.00187764	0.00126247	0.1256976	[illegible]
ENSG00000039319	1.15364631	1.31783511	1.23796902	0.9752322	1.29476659	1.11968672	0.8226533	[illegible]
ENSG00000039523	0.51415968	0.50600985	0.42904346	0.6033674	0.6998928	0.61801577	0.34526495	[illegible]
ENSG00000039537	0	0	0	0	0	0	0.01182407	[illegible]
ENSG00000039560	1.22849676	1.42935766	1.03648214	0.809415	1.29244829	0.89407118	2.39551101	[illegible]
ENSG00000039600	0	0	0.00824378	0	0	0.00632309	0.03046241	[illegible]
ENSG00000039650	1.78784397	1.83375622	1.79802298	1.7980485	2.13353358	1.88736555	2.19349421	[illegible]
ENSG00000039987	0.00478612	0	0	0.0123732	0	0	0.00384361	[illegible]
ENSG00000040199	0.87885203	0.83431848	0.78661346	0.5894708	0.86276072	0.62880487	0.33244735	[illegible]
ENSG00000040275	2.70327317	2.20398932	2.38027811	1.9346615	2.81936592	2.04851944	1.61987668	[illegible]
ENSG00000040341	2.04686414	2.26261	2.22664373	1.9148708	1.83947235	2.02320553	0.75646379	[illegible]
ENSG00000040487	1.58199799	1.42680584	1.3469841	1.2960111	1.55980825	1.06438307	2.00877994	[illegible]
ENSG00000040531	0.84062707	0.99682207	0.88425502	0.9507826	0.88331381	0.78271989	1.63080475	[illegible]
ENSG00000040608	2.10452224	2.07467524	1.89635186	2.2322151	2.12067901	1.7186964	0.04770412	[illegible]
[illegible]								

ENSG00000052723	5.22199725	5.82962755	5.21994421	6.91049	5.40560737	5.75121638	6.60460019	6.7860536	6.0996298	5.75457635	[illegible]
ENSG00000052749	0.93056439	0.76253655	0.67851765	1.0249248	0.94679923	0.88081019	8.71680727	8.96123864	9.2366497	5.39986821	[illegible]
ENSG00000052795	2.65316024	2.30630396	2.33198427	1.6744489	2.59065029	1.90262782	1.22138793	1.54298355	1.45085513	1.34708588	[illegible]
ENSG00000052802	43.4576565	25.1436208	35.289601	14.577105	54.5676708	33.9222851	25.297074	19.5431217	21.3949298	28.7224684	[illegible]
ENSG00000052841	3.30923166	3.73659066	3.40456046	4.2376235	2.92445612	3.85603786	4.67204124	5.3931523	4.13165483	4.45510281	[illegible]
ENSG00000052850	0	0	0	0	0	0	0	0	0	0	[illegible]
ENSG00000053108	0	0	0.00607031	0	0	0	0.00249233	0.00303989	0	0.0056218	[illegible]
ENSG00000053254	4.58496864	6.46711306	5.46381809	7.7638399	3.71329152	7.44953442	0.7313372	0.83841776	0.91548373	0.89639512	[illegible]
ENSG00000053328	0	0.01138607	0.01662453	0	0.02844689	0	0.01365131	0	0	0	[illegible]
ENSG00000053371	8.90316782	7.96498828	8.73376555	9.3879555	8.96074058	7.6741337	4.50422461	4.96707892	5.22948255	4.37097471	[illegible]
ENSG00000053372	11.6640033	8.07424844	9.02074523	8.0116735	12.6535079	7.06546271	15.3478812	17.687214	22.1012123	11.1743442	[illegible]
ENSG00000053438	0	0	0	0	0	0	0	0	0	0	[illegible]
ENSG00000053501	1.29366335	1.3734407	1.16309011	1.5276394	1.23114327	1.28082809	3.6855802	3.57505284	3.76372971	3.2686047	[illegible]
ENSG00000053524	0.03210825	0.03720063	0.03819076	0.0370168	0.01742659	0.04231183	0.02648223	0.0331503	0.02016432	0.02908117	[illegible]
ENSG00000053702	0.02469712	0.01102835	0.00805111	0	0.02296096	0.00617531	0.01322242	0.00806367	0.01221004	0.04473748	[illegible]
ENSG00000053747	0.03332962	0.01899975	0.02496697	0.0137497	0.0498423	0.01861805	0.30126186	0.33549617	0.18932027	0.33013444	[illegible]
ENSG00000053770	5.17333312	5.1220751	4.90899489	4.0212093	5.68228515	4.47404557	2.3545945	2.24462641	2.14793045	2.1551509	[illegible]
ENSG00000053900	0.99342767	1.08667354	0.91614257	0.8642185	0.9155148	1.00020443	1.7548495	1.80369497	1.84691829	1.87651645	[illegible]
ENSG00000053918	0.00852075	0.00285367	0.00416657	0	0	0.00319581	0	0	0	0	[illegible]
ENSG00000054116	4.00530118	3.60596521	4.03488772	4.5142997	4.28437103	3.98812408	6.1859612	6.46152439	6.62634748	6.56458762	[illegible]
ENSG00000054118	21.9464941	22.2638577	19.8633269	16.303682	21.6018512	17.3475181	20.3863529	23.3426679	23.9836898	17.6497936	[illegible]
ENSG00000054148	8.9265745	7.42429625	9.68003275	10.758823	7.78285995	8.29527291	2.50863696	2.5940499	2.21419359	2.5514522	[illegible]
ENSG00000054179	0	0.01466077	0.01070292	0.0141462	0.0183142	0.02462784	0	0.00535981	0	0	[illegible]
ENSG00000054219	0.0014157	0.00568953	0.0055381	0.00183	0.00394853	0.00743364	0.01477983	0.00832011	0.0090988	0.00641114	[illegible]
ENSG00000054267	3.54145642	4.28418646	3.82061899	3.2871792	3.3078136	3.5482629	2.10465025	2.29381718	2.42386166	1.83320297	[illegible]
ENSG00000054277	6.32987172	4.79328842	5.40760101	4.4753935	4.81636421	4.5573228	2.73913696	2.42517541	2.24601203	3.99038844	[illegible]
ENSG00000054282	2.14813371	2.46327662	2.42418722	2.1119019	1.76128048	2.02907702	2.55273115	3.0302273	3.85232738	4.70729872	[illegible]
ENSG00000054356	0.08391895	0.00733177	0.02140988	0.0110047	0.05291766	0.01368471	1.67310925	1.20558937	1.12621055	2.11057929	[illegible]
ENSG00000054392	0.18876039	0.18891697	0.16850506	0.1049745	0.08264399	0.0979632	0.40629302	0.37838426	0.33422123	0.26936859	[illegible]
ENSG00000054523	9.83655896	12.5819639	10.3114759	9.489312	10.0871708	9.62758217	1.80425358	2.07184255	2.17132974	1.95864807	[illegible]
ENSG00000054598	0	0.01252567	0.02743264	0.0120861	0.00521568	0.02805492	20.4690355	20.4050975	17.9356917	12.3895372	[illegible]
ENSG00000054611	0.46106309	0.45366501	0.44392055	0.4157985	0.41369839	0.40215685	1.02469561	1.05026737	1.04961005	0.57783536	[illegible]
ENSG00000054654	0.99058492	1.45896289	1.2194218	0.9022991	0.90851895	0.98750115	0.08448232	0.14019573	0.13758626	0.14823556	[illegible]
ENSG00000054690	0.13371716	0.37656893	0.27300053	0.2245724	0.13720256	0.24380599	0.19125483	0.17877886	0.17275097	0.2864223	[illegible]
ENSG00000054793	5.30708075	6.52759844	5.4503998	3.9719351	4.11375746	3.91782331	2.22293781	2.39301197	1.91455661	3.98172811	[illegible]
ENSG00000054796	0.00457095	0	0.0044703	0	0	0	0	0	0	0	[illegible]
ENSG00000054803	0	0	0	0	0	0	0	0	0	0	[illegible]
ENSG00000054938	0.00264547	0	0	0	0	0	0.00212451	0	0.0013079	0	[illegible]
ENSG00000054965	16.3046611	14.2826596	13.4151025	13.884404	14.0717662	14.1367299	6.07804689	6.68679671	6.66845823	5.83141781	[illegible]
ENSG00000054967	0.75646193	0.33960468	0.44626378	0.3460365	0.81000248	0.37727974	0.63518299	0.67937932	0.70987583	0.46839653	[illegible]
ENSG00000054983	0.21983971	0.25509875	0.15595019	0.3281946	0.26426175	0.28336172	0.21757643	0.27599242	0.20971971	0.08833923	[illegible]
ENSG00000055044	8.12915086	7.10398336	7.65381052	6.2984589	8.51704976	6.68584463	14.1922743	17.0886524	18.2631975	9.69746729	[illegible]
ENSG00000055070	10.7627302	7.66972679	9.10428421	8.3084667	10.1412633	7.17017724	8.63232491	9.56124574	9.28574396	6.18517579	[illegible]
ENSG00000055118	1.54427735	1.10184111	1.32641589	1.7114892	0.48539752	1.28532129	0.00107841	0.00131533	0	0.01702749	[illegible]
ENSG00000055130	14.5186449	12.7050436	12.8854898	11.595369	15.4243955	12.1487739	7.05975724	8.54041542	9.52140475	10.6389343	[illegible]
ENSG00000055147	1.21116639	1.22585185	1.26302929	1.1785053	1.48747147	1.22098871	1.47779414	1.56158675	1.50524613	1.41819626	[illegible]
ENSG00000055163	1.12585321	1.03516598	0.9702605	0.8375467	0.74107392	0.91855308	0.16438988	0.18412988	0.14273139	0.5898146	[illegible]
ENSG00000055208	3.03537474	3.24043431	3.21902936	3.0412999	3.01857141	2.90234499	3.25601253	3.78719347	4.17666602	3.23291036	[illegible]
ENSG00000055211	20.1326863	16.8898697	19.506645	17.971915	16.2758001	16.9056745	12.1060302	13.4355566	11.4131803	9.71535701	[illegible]
ENSG00000055332	9.6282259	11.0831429	9.92024125	9.9555018	10.0351661	9.33644535	16.7792861	18.6252832	10.5694603	8.41736045	[illegible]
ENSG00000055483	0.51469791	0.63402198	0.49628674	0.7037702	0.53384414	0.65393605	0.97974718	1.10991928	1.03096954	1.25138468	[illegible]
ENSG00000055609	2.10488138	2.58726978	2.34218169	2.1756232	2.07337975	2.53424821	0.67482489	0.75381785	0.63318322	0.97727746	[illegible]
ENSG00000055732	0	0	0	0	0	0	0.00170204	0	0	0.00191959	[illegible]
ENSG00000055813	0	0.01084279	0	0	0	0	0.01949992	0.007928	0.00400153	0.03665395	[illegible]
ENSG00000055917	5.60217341	6.78715804	5.67548125	6.9313122	5.59326681	5.77337566	1.54776234	1.97153422	2.40850982	2.49609537	[illegible]
ENSG00000055950	3.00847614	2.6488332	2.67542724	3.4973378	2.75882485	2.91009634	4.21297996	4.82230234	4.66379153	4.65173218	[illegible]
ENSG00000055955	0	0.00160601	0.00351735	0.0015496	0.00401245	0.00359713	0.0077021	0.00939422	0.00533429	0.01194403	[illegible]
ENSG00000055957	0.00177048	0.00237179	0	0.0022885	0	0	0.00142182	0	0.00087531	0	[illegible]
ENSG00000056050	14.0178753	11.4272557	14.0012119	11.393752	14.4335032	11.4536294	10.0700094	12.0595511	12.4338035	13.2757537	[illegible]
ENSG00000056097	15.9820029	16.5323952	15.9970055	15.633227	14.9701955	15.0069795	11.5130722	12.0410811	13.9833285	13.249608	[illegible]
ENSG00000056277	3.40159428	3.97828354	3.74671164	3.2488282	3.30493945	3.59130514	3.70371495	3.94855102	4.15966048	3.02343808	[illegible]
ENSG00000056291	0.01247281	0.00278483	0.01219818	0.0214968	0.09508718	0.01247489	0.27712591	0.23212711	0.22815863	0.42551681	[illegible]
ENSG00000056487	0.49521409	0.59444847	0.45480053	0.6768315	0.39307406	0.92402096	0.00570171	0	0.0105303	0.02572196	[illegible]
ENSG00000056558	0.02247228	0.00150523	0.01098874	0.0058096	0.03259234	0.0050571	0.43493077	0.54809318	0.27997452	0.37450718	[illegible]
ENSG00000056586	3.36356928	3.09616492	2.91852839	2.865939	3.37974884	3.15919171	2.61090473	2.88911672	2.76737675	2.44687635	[illegible]
ENSG00000056661	1.21379057	1.31598379	1.16946059	1.1368356	1.06295543	1.12461502	1.2391076	1.33501333	1.20526067	1.64670732	[illegible]
ENSG00000056736	2.22538679	2.0156134	2.06381651	1.15488	1.49514536	1.60764367	0.03961667	0.12885423	0.06774705	0.00992898	[illegible]
ENSG00000056972	1.01812952	0.75247207	0.87657989	0.680381	0.76423659	0.59225295	1.24879432	1.22411821	1.08700771	0.91397039	[illegible]
ENSG00000056998	0.63730645	0.80380212	0.79235398	0.7339645	0.47652544	0.71073195	0.29129314	0.28721991	0.30334716	0.42984684	[illegible]
ENSG00000057019	4.9240121	4.09369341	4.29334068	2.6826543	4.16746874	3.0498409	67.2476231	73.0870853	63.9601456	45.5039853	[illegible]
ENSG00000057149	0	0	0	0	0	0	0.02340225	0.04757276	0.00480232	0.00879783	[illegible]
ENSG00000057252	9.11809155	9.69062321	8.34246682	7.1967193	9.3594162	7.25929937	10.7962764	11.8077212	11.638295	7.75398125	[illegible]
ENSG00000057294	0.0753912	0.15149467	0.10533035	0.1252953	0.02102741	0.08280948	0.01297388	0.00263737	0.01464287	0.01463218	[illegible]
ENSG00000057468	0.03947304	0.05948926	0.03860393	0.0127559	0.0220189	0.02220728	0.11887428	0.10632644	0.12684836	0.09831698	[illegible]
ENSG00000057593	0	0	0.00361676	0.0047803	0	0	0	0	0	0	[illegible]
ENSG00000057608	20.3495208	17.0115555	18.1227079	23.223959	20.1369647	20.7121166	16.6152633	18.6389696	20.6281962	17.5906443	[illegible]
ENSG00000057657	0.34300814	0.32863302	0.34819463	0.1936271	0.11988853	0.21821556	0.05578961	0.09994318	0.1202088	0.06292056	[illegible]
ENSG00000057663	2.18307473	1.85594292	1.99335831	1.910195	1.84889598	1.67851811	2.68201656	2.86208462	3.27212678	2.00958089	[illegible]
ENSG00000057704	0.27410126	0.24288402	0.25968904	0.4465756	0.52081398	0.43263807	0.54343068	0.84460707	1.23514726	2.2110632	[illegible]
ENSG00000057757	14.1849718	11.6962065	12.6374119	13.643338	14.2684428	12.6215148	6.88035239	8.24486992	8.77270975	4.64787362	[illegible]
ENSG00000057935	2.40523449	2.66624048	2.56281026	2.6708412	1.86860353	2.57994538	1.05990684	1.17156846	1.15729777	1.53249505	[illegible]
ENSG00000058056	9.15303189	7.68223979	7.98259139	7.4587771	10.1803946	7.3529919	2.23814268	2.77575424	3.46724546	2.66770924	[illegible]
ENSG00000058063	16.6934993	12.9867005	13.4988317	9.4424341	15.4415461	11.2636596	7.00028252	7.64479742	8.05794961	7.40052658	[illegible]
ENSG00000058085	0.02450232	0.01025753	0.02396283	0.011877	0.02562734	0.01378485	0.13528073	0.10800098	0.09388145	0.23301923	[illegible]
ENSG00000058091	5.74187612	6.80204732	5.81119429	5.9339666	6.18971268	6.27274857	1.45801452	1.76999349	1.91447874	1.9786508	[illegible]
ENSG00000058262	29.086956	25.146857	25.633567	31.856906	29.2283496	22.7895763	36.3197295	39.776737	38.2530108	33.9404311	[illegible]
ENSG00000058272	2.74444854	2.76470102	2.90000431	2.2431163	2.58793788	2.41902353	2.56129758	2.74031547	3.03802786	2.39737718	[illegible]
ENSG00000058335	0	0.00100775	0	0	0	0.00112857	0.00966591	0.00294737	0.00223146	0.01226407	[illegible]
ENSG00000058404	0.00081683	0.00164138	0.00319539	0.0063351	0.01230245	0.00612726	0.01246357	0.00960112	0.00484602	0.06658409	[illegible]
ENSG00000058453	0.30568878	0.63596773	0.38437474	0.5408116	0.24910022	0.46177962	0.61994952	0.69129825	0.59198346	0.81912472	[illegible]
ENSG00000058600	0.95576882	1.06515571	0.95186517	1.2233602	0.9978605	1.00354906	2.25199182	2.27865611	2.51858686	1.52707768	[illegible]
ENSG00000058668	1.74006086	2.19775847	2.21683161	1.909284	1.17866564	1.64084232	3.40661421	3.8735823	3.12566308	2.64034095	[illegible]
ENSG00000058673	5.41676299	6.28364725	5.3728207	5.5007881	4.83500927	5.80727114	5.89705821	6.12069445	6.18911118	5.22728034	[illegible]
ENSG00000058729	2.97291888	2.87019579	2.70947526	2.7476583	2.92565204	2.70813135	2.4988718	2.65127662	2.58556207	2.00674688	[illegible]
ENSG00000058799	1.61525391	2.0447531	1.96577009	2.0450555	1.51795482	1.81520266	1.42059448	1.31649387	1.31406087	1.25826518	[illegible]
ENSG00000058804	5.11028534	4.0109954	4.08578154	4.0988846	5.18594646	4.03484885	3.9952046	5.0055652	6.70381223	3.79576669	[illegible]
ENSG00000058866	0.36728686	0.17195481	0.26101035	0.1051374	0.47852696	0.11535236	0.19800005	0.21535831	0.21111433	0.18071843	[illegible]
ENSG00000059122	0.82542244	1.02081611	0.75058056	1.2563217	0.69645091	0.99969286	0.83496034	0.84598835	0.76481544	0.65848549	[illegible]
ENSG00000059145	0.19567643	0.28344084	0.21399283	0.2678864	0.11291636	0.29500964	0.66263178	0.72606716	0.74676227	0.47493768	[illegible]
ENSG00000059377	0.00099514	0.00466594	0.0058394	0.0012863	0.00222045	0.0044789	0.02637279	0.02046979	0.03050343	0.04777021	[illegible]
ENSG00000059378	0.29799603	0.34860149	0.3817385	0.4774244	0.29148524	0.35419157	2.51784357	2.49750511	1.20351363	1.71444641	[illegible]
ENSG00000059573	8.07502153	7.6506549	6.79646641	12.13688	9.11003755	8.73180696	13.7427605	16.7801367	17.9366226	16.5690695	[illegible]
ENSG00000059588	0.82684659	1.0431936	0.84002122	1.0368914	0.8660159	1.19974433	4.6402092	5.15678426	5.61195826	6.87193812	[illegible]
ENSG00000059691	0.82701528	0.74481142	0.776402	0.9165423	0.8620304	0.70387537	1.16120717	1.11773813	1.27837157	1.13317291	[illegible]
ENSG00000059728	1.90358952	1.89985425	1.96303805	1.9470539	1.42994949	2.33755484	2.15297724	2.07438877	1.86344829	2.16532476	[illegible]
ENSG00000059758	4.57505888	5.10007035	4.71090423	3.9162214	4.37440753	4.40082131	2.45946892	2.46839939	2.81688697	2.43986837	[illegible]
ENSG00000059769	0.64569541	0.54553602	0.58124727	0.7303071	0.68966791	0.59443223	1.34644074	1.14993418	1.33494612	1.07327941	[illegible]
ENSG00000059804	9.2209818	5.43035026	8.46737636	4.6526109	11.9878908	8.69756332	7.04606177	6.54558047	5.63390903	3.27476075	[illegible]
ENSG00000059915	2.03647357	2.01881217	2.11611173	2.6652846	1.65428298	2.00116841	0.01839871	0.01745399	0.01006815	0.03919521	[illegible]
ENSG00000060069	1.22646662	1.10275765	0.96745801	1.1161855	1.22405803	1.08727641	1.1163974	1.14324224	1.21153621	1.84781668	[illegible]
ENSG00000060138	3.99180242	1.98254788	2.96606378	3.6222035	6.45617022	3.61786212	7.20153582	7.81350412	7.3805766	5.83643803	[illegible]
ENSG00000060140	0.01177342	0.00394301	0.0057571	0.0076093	0.01641866	0.00883154	0.23164598	0.28253791	0.22118576	0.3145719	[illegible]
ENSG00000060237	2.72249894	3.21552887	2.66151256	2.8804927	3.0542825	2.9293859	3.43064965	3.74947276	3.93675435	3.75136206	[illegible]
ENSG00000060303	0.7899446	0.05291174	0.23176549	0.4084383	0.26438875	0.59255709	0.88813847	0.85113273	0.5858127	0.64392367	[illegible]
ENSG00000060339	2.77646194	2.73458053	2.7144016	2.289535	2.75972207	2.68792023	3.56600612	3.64561388	4.65108439	3.18873134	[illegible]
ENSG00000060491	4.34102533	3.87017263	3.94159588	5.0033003	4.05591953	4.76793857	8.28571379	9.12969066	7.45397838	5.04563493	[illegible]
ENSG00000060558	0	0	0	0	0	0	0.00737194	0	0.00907668	0.00831421	[illegible]
ENSG00000060566	0	0	0	0.0042472	0	0	0	0	0.00162444	0	[illegible]
ENSG00000060642	0.93905942	0.96960892	0.93496056	1.1218197	0.87367592	0.9358251	1.47015484	1.47104177	1.3056346	1.70412533	[illegible]
ENSG00000060656	0.01006288	0.00898704	0.00984132	0.0108395	0.01309768	0.00943552	0.65727489	0.78606982	0.7450067	0.64103081	[illegible]
ENSG00000060688	7.19356638	5.83554708	6.53398212	5.6210044	6.95973323	5.03991527	4.83280481	5.63987727	6.81852286	4.81229987	[illegible]
ENSG00000060709	0	0	0	0.0025721	0	0.00149264	0	0.00194908	0.00098377	0	[illegible]
ENSG00000060718	1.64533474	1.19289389	1.14498017	1.6859956	1.74134162	1.89837053	0.29335733	0.33600164	0.20661158	0.21170979	[illegible]
ENSG00000060749	7.6921248	9.40105114	8.2330045	8.1108328	7.57280295	8.30308814	2.08635302	2.72857059	2.99463703	2.48331004	[illegible]
ENSG00000060762	15.1198312	11.4657987	14.7869233	15.495936	24.236611	14.006213	4.16418494	4.36456069	5.46816165	2.80781344	[illegible]
ENSG00000060971	0.98649429	1.0912968	1.17847358	1.1694464	1.29730996	1.17996438	0.58698813	0.70593441	0.77010842	0.55322324	[illegible]
ENSG00000060982	5.22744074	3.57109833	5.04666821	6.0490312	7.12321211	5.00673341	18.4036387	21.9709442	24.0258214	19.6921681	[illegible]
ENSG00000061273	0.52731462	0.50897557	0.46016685	0.5977246	0.4449921	0.57659283	1.15206398	1.22306651	1.24367174	1.5858796	[illegible]
ENSG00000061337	9.03411283	1.97820932	5.31049296	2.0505972	14.5273746	2.94565653	3.16168678	2.92001698	1.64715184	0.55737181	[illegible]
ENSG00000061455	0.0120081	0.01005402	0.01467963	0.0194023	0.00502378	0.00450379	0.02410851	0.01176203	0.00742088	0.02447102	[illegible]
ENSG00000061492	0	0	0	0	0	0	0	0	0	0	[illegible]
ENSG00000061656	0.02708381	0.0108847	0.01059499	0.0140036	0.01208634	0.01218975	0.30015455	0.38732066	0.22227408	0.3630503	[illegible]
ENSG00000061676	17.6315832	19.0367981	17.0720727	16.982119	19.2125115	17.8236513	8.57865267	8.41767512	9.18131883	15.303431	[illegible]
ENSG00000061794	7.2540895	6.45310656	6.32043805	6.555981	7.38400009	6.86255183	11.3141365	11.938311	13.6904079	8.318625	[illegible]
ENSG00000061918	2.59475127	2.27374385	2.55230969	2.5482778	3.59128822	2.38721183	0.19752178	0.21333084	0.21720731	0.39962329	[illegible]
ENSG00000061936	1.52259557	1.9007605	1.51981165	1.6073354	1.34799688	1.73442575	2.24559387	2.31775758	2.49137453	2.37547227	[illegible]
ENSG00000061938	1.13813105	1.84956246	1.14113235	1.5877304	1.0224287	1.60089137	1.30736642	1.3048478	1.02235231	1.29379822	[illegible]
ENSG00000061987	0.70206908	0.85953767	0.63996913	0.7849284	0.64374416	0.72007341	0.91341431	0.97122896	0.98015119	0.98094267	[illegible]
ENSG00000062038	0.00383642	0.00642423	0.00562792	0.0123975	0.00214004	0.02014454	0	0.0037578	0.00284504	0.00868682	[illegible]
ENSG00000062096	0	0.00710365	0.0155578	0.0068543	0	0.00397768	0.00425846	0.00519403	0.0026216	0.00960553	[illegible]
ENSG00000062194	5.16816054	5.28305987	4.89679795	5.3635851	4.65206265	5.43583918	2.59475874	2.78363294	2.50117356	2.40668895	[illegible]
ENSG00000062282	0.01541355	0.01935795	0.02449554	0.0273952	0.00752326	0.02023366	0.9314623	1.00965856	0.86966991	1.14998834	[illegible]
ENSG00000062370	0.731134	1.03173411	0.84990035	0.6188398	0.85051771	0.82754207	1.09490884	1.08569599	0.99821311	1.33619229	[illegible]
ENSG00000062485	6.13686214	5.32461888	5.18123716	6.8033339	6.95856874	6.43330779	4.11788177	4.58621377	4.72388571	3.58703936	[illegible]
ENSG00000062524	0.34164203	0.70015105	0.49564787	0.5235006	0.32940481	0.69075227	0.55417917	0.38339674	0.21141074	1.34019297	[illegible]
ENSG00000062582	0.24745961	0.16575237	0.1393397	0.2035539	0.1087574	0.23062576	0.46370003	0.49946754	0.34107438	0.59767942	[illegible]
ENSG00000062598	1.18542078	1.30904949	1.10361715	1.2757619	1.04648739	1.1749369	0.9662756	0.93535399	1.01284856	0.88585215	[illegible]
ENSG00000062650	6.88764322	7.42664425	6.79346947	6.5567833	6.90236732	6.3433615	7.564456	8.44032272	8.88121308	7.58340777	[illegible]
ENSG00000062716	2.3516446	1.95484487	2.3908374	1.9632665	2.44360781	1.96245289	13.3213733	14.0485106	12.1012974	15.2913214	[illegible]
ENSG00000062725	7.49308253	8.82928484	7.7385665	7.5033401	6.29086499	7.62180484	4.08085925	4.30384701	3.45246767	4.91817704	[illegible]
ENSG00000062822	2.53242553	2.07528773	1.94454063	2.0235543	2.4333151	1.98359716	1.30463162	1.99782483	2.34184928	1.67273553	[illegible]
ENSG00000063015	0.04100644	0.04784533	0.00646832	0.0324873	0.05829248	0.02480641	0	0	0.00065398	0.00119808	[illegible]
ENSG00000063046	26.4240247	30.5124593	27.4375292	45.230089	18.6812439	37.7632615	25.4804854	29.012812	33.2253857	25.5016964	[illegible]
ENSG00000063127	0.14080428	0.2490829	0.18007454	0.2380078	0.1490315	0.28436368	0.08988122	0.04950936	0.03212881	0.09155974	[illegible]
ENSG00000063169	0.16574908	0.2424344	0.24811167	0.1967602	0.20567269	0.24866502	0.23090322	0.21536562	0.21406019	0.26654439	[illegible]
ENSG00000063176	0.53083721	0.53057311	0.43358791	0.4202595	0.48340827	0.38400464	0.79563714	0.80946889	0.79258453	0.99263593	[illegible]
ENSG00000063177	21.1718767	21.7174481	21.2434067	34.102787	18.2097598	26.0160163	24.2644584	27.6764276	28.5229394	23.9583392	[illegible]
ENSG00000063180	0.35476365	0.65527256	0.50465814	1.1533817	0.26985643	0.97576246	4.95556384	5.24399761	2.41828485	4.88792046	[illegible]
ENSG00000063241	2.75149243	1.60756572	2.00342603	3.3914143	2.63596574	2.11190011	2.72856667	3.12632616	2.58722973	2.53389188	[illegible]
ENSG00000063244	15.2762371	13.7760309	12.6753207	13.067957	14.5445519	12.2304723	18.3706173	19.6556931	21.8000442	18.1268751	[illegible]
ENSG00000063245	11.9141561	9.25791735	9.34852044	9.7305548	11.306068	8.50567721	12.8391308	14.1266675	12.3869943	13.6295964	[illegible]
ENSG00000063322	3.20182982	3.54067324	3.28440746	3.7944356	2.49066502	2.93939014	3.5989504	3.8274768	3.21207238	3.10142576	[illegible]
ENSG00000063438	3.07641665	2.48064204	2.56807852	2.7049626	2.70081699	2.46218679	1.42653473	1.23921204	1.02093915	1.53725586	[illegible]
ENSG00000063515	0	0	0	0	0	0	0	0	0	0	[illegible]
ENSG00000063587	1.12388303	1.10893744	1.02890106	1.2573128	1.13929019	0.98765693	0.54133493	0.57510898	0.6503208	0.53977485	[illegible]
ENSG00000063601	1.57112158	1.62721762	1.51992878	1.586565	1.44509194	1.45069117	1.16716247	1.31343861	1.35000123	0.78404346	[illegible]
ENSG00000063660	46.0631101	27.2185744	37.6232743	38.551263	64.4853002	33.3831637	2.87732851	2.87409974	1.83468407	2.81782622	[illegible]
ENSG00000063761	0.30792745	0.34552067	0.29085187	0.3844242	0.30830241	0.29810761	0.35930849	0.39184481	0.33179788	0.32657155	[illegible]
ENSG00000063854	1.80741828	1.8364754	1.66276364	2.0954096	2.03494524	1.63920301	1.01276427	1.03772309	1.04250068	0.87400206	[illegible]
ENSG00000063978	3.26788675	2.6447457	2.60444414	2.8582298	3.47595373	2.54123254	2.78699695	2.88379781	3.09500621	2.93733861	[illegible]
ENSG00000064012	0.67539961	0.51790764	0.67462533	0.5349988	0.52492619	0.44716633	0.92273538	0.85518641	0.7589366	0.56603189	[illegible]
ENSG00000064042	1.13828217	2.81662544	1.83774634	2.681337	0.89096853	2.17779894	0.00074836	0.00410749	0.00092142	0.00126603	[illegible]
ENSG00000064102	2.43726486	1.77175694	2.06244855	1.9860683	2.83507383	2.09718019	6.05821305	6.855062	8.38409793	5.92664984	[illegible]
ENSG00000064115	6.75337597	5.44423438	5.84017404	5.5250954	6.52888428	5.74371629	5.20652758	5.46994912	5.17899793	7.0975207	[illegible]
ENSG00000064195	0	0	0	0	0	0	0	0	0	0	[illegible]
ENSG00000064199	1.9295466	1.27130284	1.46820471	1.4393892	2.01185453	1.44401911	0.98839414	1.10397562	1.13225984	1.34339164	[illegible]
ENSG00000064201	0	0	0	0	0	0	0.01634302	0.0090607	0.0036586	0.00167563	[illegible]
ENSG00000064205	0.01894196	0.0021146	0.00617496	0.0204039	0.01232726	0.00473628	1.13327975	0.825644	0.1716869	0.0142968	[illegible]
ENSG00000064218	0	0	0	0	0.00670837	0	0	0	0	0	[illegible]
ENSG00000064225	0.60844226	0.48090224	0.50957542	0.2345148	0.37827901	0.26969544	0.29228639	0.33157829	0.32377945	0.55308388	[illegible]
ENSG00000064270	0.00164027	0.00219736	0.00320832	0.0021202	0.00182996	0.00492164	0	0	0	0.00148563	[illegible]

ENSG00000064300	0.2541607	0.09779794	0.27500764	0.139801	0.10557806	0.34074021	0.01737107	0.0264843	0.00534702	0.03918283	0.00681271	0.00456678	0.08696508	0.22261714	0.07995161	0.0625999	0.04881063	0.03261485	5.54655382	4.26862538	2.72217715	2.49403239	0.82717049	0.53795193
ENSG00000064309	0.15068068	0.04266888	0.07907283	0.055423	0.12163752	0.07535288	0.91592128	1.09554749	1.02718624	0.90983272	1.24856246	0.98385758	0.10265224	0.09555412	0.09908392	0.07090642	0.08661697	0.06649665	0.54601461	1.25308543	0.42589954	0.68113193	0.67632666	0.62300127
ENSG00000064313	4.20926179	4.44830279	3.91190641	3.7149428	4.36553365	3.68371728	8.56687292	8.95916381	9.46078404	9.09321826	8.91990086	9.08842911	7.41493409	6.63252196	7.31899742	8.56190323	7.46301819	5.1165041	5.21303194	4.64570714	6.76903494	6.43733704	7.8381683	7.98576317
ENSG00000064393	6.89294595	10.7359977	8.50717183	6.4342109	6.68029932	6.3652306	2.47239572	2.78795585	3.20511513	4.29254845	2.93876016	3.03741526	4.92097179	5.69555517	6.11834837	6.08975051	5.77814788	5.86143997	2.51502735	2.21186437	2.97285505	2.70400326	3.44553136	3.77049482
ENSG00000064419	12.3633457	10.5762379	11.2314738	10.718949	10.4969044	10.2223408	3.29986592	3.8268632	4.14873088	4.78547196	4.11009928	4.60369374	4.19772632	3.47442923	3.88205937	4.47708574	3.78633986	2.88189983	2.80557055	2.30592861	3.34820447	3.87422858	4.80563665	4.47259329
ENSG00000064489	0	0	0	0.005059	0.00218317	0.0029358	0.00314303	0.00383355	0	0	0.00986126	0	0.01325055	0.00423992	0.00340378	0.0037755	0	0	0	0.00347511	0	0.00334265	0	0
ENSG00000064490	2.34815671	2.39221244	2.41792427	2.6717618	2.13864027	2.34854767	2.24332204	2.54106017	2.50023992	2.22653787	1.91903108	1.8998961	2.16602146	1.81268374	1.79964901	1.54631257	1.31851056	1.14202477	1.59899765	1.44617185	1.9431228	1.71128862	1.30565848	2.01099137
ENSG00000064545	0.44806631	0.35468954	0.34335215	0.5967289	0.48365193	0.52816608	1.36299537	1.64154212	1.43077066	1.56005002	1.03610905	1.10568992	0.88162597	0.72706106	0.79454587	0.54076676	0.43769766	0.4305097	0.81330243	1.06781925	1.13969961	0.88133241	0.67395668	0.99725127
ENSG00000064547	0.04484978	0.11092095	0.05398434	0.1739207	0.036565	0.17080258	0.1967122	0.20275714	0.16033033	0.30622332	0.19124008	0.09614576	0.49641697	0.55688776	0.63009202	0.20634334	0.26988139	0.42863632	0.20122854	0.25731906	0.09583751	0.06187768	0.0803808	0.06094242
ENSG00000064601	3.23641398	2.78037342	3.02162744	2.32236	3.47797362	2.20869884	6.7340431	6.30840995	4.51070445	3.96808996	5.96123624	4.43044127	5.56496479	4.80147929	4.94150817	3.71372643	3.02582412	3.65979136	2.16423903	2.71457333	3.06249658	2.25486162	1.94986988	2.6297686
ENSG00000064607	2.42548387	2.72977541	2.37793664	2.5662366	2.53476182	2.98462803	2.30014046	2.6206915	2.56852465	2.78941357	2.04843645	2.41436152	2.58013191	2.07127967	2.79174604	2.3318642	2.38636566	1.98931098	1.76634259	2.02469225	1.85333132	2.33625215	3.06367218	1.87151508
ENSG00000064651	4.51398562	2.84834707	2.89276806	2.4026985	6.546629	2.97161412	1.88451626	2.11287103	2.10599853	1.56376709	1.81785711	2.21005021	1.48623548	1.17478879	1.2753731	2.35729014	1.67777373	1.07055923	1.05272934	0.89894666	2.8709046	1.82097575	1.89782731	3.02458453
ENSG00000064652	2.05888554	2.24670362	2.13356618	1.73164	2.16007426	1.74130939	0.9444313	0.94490832	0.82072019	0.8891663	1.03495532	0.84057676	1.36469992	1.19647808	1.28959183	1.49948435	1.22559173	0.80179747	0.70526104	0.60836898	0.85509859	0.84137751	0.95649473	1.10920664
ENSG00000064655	1.9615006	1.25421435	1.79642261	1.1085483	1.7678822	1.2910663	3.04799003	3.26418462	2.92685208	2.1152269	2.90480711	2.68897007	0	0.00642956	0	0.00572531	0	0	1.46632455	1.33062018	1.78856475	1.61191409	1.13628254	1.9698554
ENSG00000064666	6.13298061	3.9626652	4.47168212	3.1824715	7.45195147	4.36036523	11.7769445	13.7701751	13.3955608	8.07083537	9.78809489	9.02819843	18.6106474	20.5232369	17.2580242	11.1540964	9.58425267	11.3203592	9.66061358	9.93937856	10.2420584	8.12508854	4.39282715	6.39311558
ENSG00000064687	0.03853439	0.06094252	0.03663911	0.0539611	0.03940806	0.10197256	0.49513604	0.41414375	0.23390484	0.25206621	0.43287287	0.22056426	0.30260303	0.21916556	0.19456292	0.1022264	0.09500593	0.19754784	0.68441411	1.9341324	0.27121718	0.2322082	0.2840893	0.09852136
ENSG00000064692	2.1137201	3.98575589	2.54557354	2.6295935	0.92658014	2.31942163	0.00372355	0.0022708	0.00229231	0.00157481	0.00219049	0.00048945	0.02992418	0.02888245	0.01814604	0.01230031	0.00784706	0.01328312	0.06633486	0.04116968	0.03171245	0.15048149	0.0546012	0.04580151
ENSG00000064703	3.07905584	2.54350925	2.7426754	1.7886283	2.84443736	2.05810784	2.39000574	2.96940117	2.81735351	1.94448592	2.21233024	2.61587079	2.6821374	1.88811056	2.3149803	3.52563164	2.97545569	1.60221873	1.47050435	1.36698362	2.78976359	1.96667814	2.11326351	3.17198174
ENSG00000064726	12.3538668	11.2864307	12.3546611	12.534267	17.5142712	12.380749	11.063921	11.9201033	11.2920146	14.2080713	14.580896	14.1380233	9.80015739	8.7430205	8.74578399	15.1739321	13.7482688	9.39843443	10.3281086	9.02563376	13.0420123	13.8017245	17.4521694	18.1228289
ENSG00000064763	0.00272995	0	0	0	0.00304564	0.0020478	2.5803968	3.13393225	3.14471165	4.00309698	2.37995864	2.5590352	0.5337609	0.56783181	0.44872842	0.47666627	0.6119176	0.26673121	0.7871402	0.66174765	0.81810431	0.8743455	1.00108312	1.02284121
ENSG00000064787	0.01432672	0.01679346	0.01576268	0.0138892	0.01398556	0.01746359	0.01438178	0.01754142	0.01859289	0.01946405	0.0225614	0.03024725	0.03789461	0.02134095	0.01557487	0.01554823	0.01616444	0.02160188	0.02956274	0.01431116	0.01658265	0.02141325	0.01088468	0.01498471
ENSG00000064835	0	0	0	0	0.00913306	0	0	0	0	0	0	0	0	0	0	0	0	0	0	0	0	0	0	0
ENSG00000064886	0.0020449	0	0.00199988	0	0.00228138	0	0.30052467	0.27441103	0.13243879	0.05185926	0.17518271	0.13124594	0.00346166	0.01329195	0.00533534	0	0	0	0.02700543	0.02178863	0.01549245	0.06636734	0.05800142	0.04372703
ENSG00000064932	0.74723099	0.7073283	0.55865297	0.7184256	0.64676206	0.69138095	4.22165403	4.40814673	3.76370349	3.05531642	3.38234877	2.89050993	5.5811005	4.85201026	5.09015547	4.73607379	4.10391838	4.23109368	3.56460798	3.59570077	2.7603788	2.87319123	2.49133904	2.14872314
ENSG00000064933	1.1525145	1.08955217	1.02680779	0.970076	1.10122554	1.00318733	0.91887535	1.10535958	1.06876212	1.19043304	1.07703786	1.31203952	0.70404953	0.64781156	0.76360946	0.78905094	0.79946682	0.53624076	0.74148821	0.79028179	1.3661835	1.20693872	1.75170624	2.06310463
ENSG00000064961	4.55512465	4.41212222	4.19933248	4.5112704	4.11284415	3.66983079	2.67185188	3.15386668	3.08216866	3.3409916	3.40943184	3.27732214	3.99724926	5.15729527	4.90536351	2.95963845	3.55056723	4.73773473	3.56069901	2.13333035	1.56762005	4.22417225	3.28204898	2.61571121
ENSG00000064989	16.9078652	23.9224089	19.3373321	15.962064	14.5482652	16.1045015	0.40950883	0.38935585	0.28187903	0.46185165	0.44766805	0.42215521	0.1903147	0.26533702	0.24967631	0.16849019	0.25067111	0.15014125	0.430829	0.77007501	0.99708048	0.42179987	0.70975182	0.57114202
ENSG00000064995	9.43699576	8.59387218	8.53646277	9.1266622	8.63551203	9.28144797	11.7073984	13.1156342	13.516595	8.5059098	13.8896186	15.1382585	8.34536371	6.4855897	7.91917219	6.6458855	7.87176002	5.55384253	8.32354788	9.37349218	11.6947771	10.3042271	10.6822555	15.3592905
ENSG00000064999	2.94420274	3.22991051	2.57684702	2.8836959	2.50106503	2.57706131	5.52378806	5.99588307	5.42112238	4.47624866	6.16208599	5.34770819	2.43039551	2.57058079	2.59537905	2.22231046	2.80312076	2.13348829	5.3308201	4.78898125	5.05484445	5.07267614	7.03773106	5.2478346
ENSG00000065000	5.28416445	5.39169216	4.69819235	5.0693672	5.86044403	5.21523055	6.91201726	7.65180859	6.93910909	6.47547617	4.54908635	5.34209088	8.06939676	6.72743705	8.44037169	8.64771647	7.31776639	5.54394689	2.41787143	3.01894468	4.15312181	3.1685503	3.30577943	3.82287593
ENSG00000065029	0.89160629	0.92722224	0.83622552	0.9778832	0.9237896	0.85598918	1.17933788	1.26407914	1.07882671	0.99899882	1.57577531	1.2898313	1.00890968	0.93319848	0.94544031	0.79725167	0.84159972	0.7246643	0.98997328	1.15717717	1.07832572	1.16600716	1.07867997	1.3047284
ENSG00000065054	0.16375236	0.12065232	0.14733512	0.2413027	0.14797818	0.18425208	1.92523904	1.95363232	1.82964528	1.28440121	1.61738321	1.09524685	0.70132424	0.76281724	0.7405573	0.43915132	0.52224716	0.61232793	2.99873213	2.86728078	2.23033833	1.73143426	1.14125727	1.48588959
ENSG00000065057	2.3623128	1.43876437	1.8625301	2.2413798	2.27685183	1.76472224	1.89320236	2.10875393	2.43936056	1.58815691	1.69974874	2.06901153	1.17907779	1.01839778	1.09290678	1.07600175	1.08445125	0.66978485	1.39368874	1.17636017	2.20588364	1.61824046	1.25818178	2.53590419
ENSG00000065060	2.66429469	2.85748163	2.74418227	3.9076291	2.76591308	3.46233147	3.05530069	3.08866472	2.91456874	2.41932082	2.95640172	2.54909363	1.60331553	1.73031573	1.77262273	1.54299166	1.45060381	1.19345599	2.30092034	2.01063438	2.81405574	2.46091484	3.89097974	2.22210483
ENSG00000065135	43.2678727	33.1496187	37.4092345	32.616457	42.0020207	32.4940123	42.1828179	45.4830118	48.6323213	32.5874383	46.1763116	46.4556597	49.709065	42.1689804	48.6985722	57.1299881	53.853993	31.7951422	33.2677546	29.5280334	49.0231789	43.5138668	52.3232761	60.9008713
ENSG00000065150	5.25414031	4.9858297	4.86899411	4.7288341	5.22902353	4.68778642	4.91468534	6.24472692	8.49924953	6.04197589	4.91546108	7.58824162	8.09978406	6.99614248	8.49346387	13.8716369	10.8435094	6.08195258	1.42715441	0.98013258	3.75561691	3.31900435	3.66098031	5.10949164
ENSG00000065154	4.08183444	4.01764327	4.16778286	5.3554706	3.94759755	4.38594414	8.13762345	9.11699232	10.3244438	10.4434057	10.2435919	10.2447026	6.99972803	7.79053056	8.94774038	11.395144	11.2607683	8.935575	4.86534832	4.83247776	5.75083398	6.26749535	9.76808539	7.78360889
ENSG00000065183	4.70173806	4.31725051	3.88767437	4.363624	4.64188399	4.05436713	16.7808497	19.3695785	19.9031999	13.760125	17.4725882	18.9305387	4.72632155	3.45474972	3.90855184	5.40424102	4.31335467	2.59471445	7.83861557	6.99253542	16.4999215	11.1804478	10.9187841	16.877153
ENSG00000065243	4.53602459	4.78311424	4.33424819	3.8632506	4.13642532	4.15943853	3.09467853	3.40544815	3.42824953	3.44934077	3.5705217	3.91279698	4.90647195	4.83503722	5.62595503	5.86343481	6.23079217	4.41159258	2.45394052	2.22353121	3.42660855	3.82962506	5.54492892	4.70964103
ENSG00000065268	2.36730917	2.2957792	2.15318175	2.6512418	2.48146421	2.15759224	4.46982391	5.21877099	6.33135375	4.37827175	4.17382672	4.37736304	2.33001862	1.82066475	1.89643845	2.12969906	1.96188247	1.44957314	1.52802388	1.09110951	1.72657007	1.77492218	1.01597779	2.10291405
ENSG00000065308	3.20524482	3.56258096	2.97953527	2.2736081	2.17052907	2.28534208	21.7614129	23.1605596	18.1230843	21.8384705	18.5561702	15.3859661	14.9587465	12.9038634	11.9010227	13.2907579	13.040758	9.03263186	8.42063995	12.7469919	10.9575468	13.3489488	10.7379245	13.6115179
ENSG00000065320	0.38971453	0.34261102	0.58361115	0.0539734	0.07861016	0.08352416	0.02794374	0.02044974	0.01634267	0.03781857	0.01534285	0.00881554	0.6449907	1.18741793	0.66727511	0.16951252	0.21461748	0.7240232	1.56046119	1.24974914	1.12770101	0.26300841	0.33309486	0.21674672
ENSG00000065325	0	0	0	0	0	0	0.01080367	0	0	0	0.02542236	0.00284024	0.00569333	0.01093053	0.01169992	0	0	0	0.00370128	0.00298628	0.00849339	0.00287245	0	0

ENSG00000068971	4.33079651	3.34157092	3.43398341	3.9665813	3.49691906	3.76465114	3.39628899	3.22841074	2.78284952	2.866026	3.4816129	2.73796417	4.53646936	3.70023912	[illegible]
ENSG00000068976	0.02943691	0.01971731	0.01439439	0.0332943	0.0123154	0.02484153	0.07683014	0.0648758	0.07094761	0.16996821	0.06489924	0.08079321	0.03425908	0.02391762	[illegible]
ENSG00000068985	0	0	0	0	0	0	0	0	0	0	0	0	0	0	[illegible]
ENSG00000069011	0.01520749	0.02037244	0.0297453	0.0196575	0.01413844	0.01521002	0.00407092	0	0.00751845	0.00459126	0	0.00856181	0.80234037	0.81825188	[illegible]
ENSG00000069018	0.00303382	0	0	0	0.00338465	0.00113787	0	0	0	0	0	0	0	0	[illegible]
ENSG00000069020	1.10484769	1.22380899	1.11363055	0.6050165	1.28597249	0.68285151	0.63524773	0.76638792	0.59325159	0.73931977	0.51790491	0.50736484	0.51942897	0.52336354	[illegible]
ENSG00000069122	0	0	0	0	0	0	0.00096769	0	0	0	0	0	0.00203982	0	[illegible]
ENSG00000069188	0.00193149	0.01552488	0.00566688	0.003745	0.00592583	0.00434657	0.0085312	0.00567572	0.00334218	0.00437348	0.00486666	0.00815569	0.01226122	0.01046227	[illegible]
ENSG00000069206	0	0	0	0	0.00179569	0.00241474	0	0	0	0	0	0.00543707	0	0	[illegible]
ENSG00000069248	17.0216706	18.1960198	15.397825	17.194223	14.29868	16.6843977	7.87974332	8.96662823	9.70032475	8.65845765	8.50771955	9.31624309	5.51050607	5.02255366	[illegible]
ENSG00000069275	264.361517	206.861365	230.972441	208.55134	276.97592	216.278192	79.1268205	102.698778	126.584193	66.5745312	95.0419908	130.799365	72.9781305	70.7038573	[illegible]
ENSG00000069329	9.74307683	7.79782684	8.43419663	7.4736446	8.7515538	7.51872833	4.60647211	4.86787312	4.86495844	5.07267782	5.08177233	5.3544116	4.65658284	3.94652996	[illegible]
ENSG00000069345	10.2026708	9.19960278	10.027081	11.10808	10.2004478	10.2148205	5.77002885	7.0131226	6.50919883	8.62751382	9.89949955	9.36445717	4.47171308	4.03448654	[illegible]
ENSG00000069399	0.43362029	0.36916468	0.40821966	0.4819294	0.46341999	0.48942399	2.53524121	1.95695441	1.55273881	2.1251991	1.53163882	1.30733967	1.80423175	1.93610487	[illegible]
ENSG00000069424	0.42683763	0.34013706	0.36171576	0.3669636	0.45110582	0.34417639	0.1340625	0.15764082	0.11267837	0.15572506	0.1951982	0.14182192	0.11337581	0.17760007	[illegible]
ENSG00000069431	0.13195909	0.32040756	0.29657515	0.1984546	0.05308384	0.19416445	0.70410924	0.72457361	0.55390792	0.88489543	0.3612632	0.49933403	0.97837449	0.93334075	[illegible]
ENSG00000069482	0	0	0	0	0	0.01651389	0.01767957	0	0.04353581	0	0.0277348	0	0.1490687	0.0476991	[illegible]
ENSG00000069493	0.19309622	0.32003089	0.26389827	0.2559994	0.13118913	0.24883848	0.16898732	0.18428956	0.13830201	0.27130624	0.22143528	0.15470707	0.12781734	0.13409533	[illegible]
ENSG00000069509	8.29577849	6.79568975	7.97396075	7.3205119	8.12006138	6.79925908	8.74190414	7.91671299	8.3926657	7.91320117	9.17841834	9.66148543	6.03402464	6.15072003	[illegible]
ENSG00000069535	3.59980037	4.21366693	5.27213888	3.6388646	3.75395658	2.65374289	0.18513211	0.22146267	0.11835512	0.06424475	0.22340438	0.09359694	0.00375235	0	[illegible]
ENSG00000069667	2.04150643	2.37717765	2.32515136	2.3036578	2.49922196	2.81169139	1.02595896	1.05114163	0.77118847	1.80275485	1.3857549	0.93862581	1.073752	1.45320789	[illegible]
ENSG00000069696	0.03713104	0.03730644	0.05447023	0.2159829	0.10356241	0.12533811	0.178914	0.12729551	0.14685811	0.16815211	0.18711403	0.07839283	0.01571404	0.10056377	[illegible]
ENSG00000069702	0.06938814	0.11760159	0.09048047	0.1291025	0.05923219	0.12144946	0.54457417	0.61993446	0.37371548	0.3523208	0.59999663	0.43682318	1.02778922	1.13895295	[illegible]
ENSG00000069712	0.63560502	0.85382878	0.72807394	0.5537814	0.26640523	0.56634461	0.36943331	0.35428596	0.30208481	0.77605735	0.6237882	0.39738622	0.64498444	0.78748731	[illegible]
ENSG00000069764	0	0.05451513	0.01989906	0	0	0.01526283	0.01634021	0	0.01005941	0	0.02563368	0	0	0	[illegible]
ENSG00000069812	0.04009672	0.03760038	0.04705664	0.0207319	0.0335502	0.05413958	0.02576053	0.03927505	0.03964694	0.05084311	0.05556621	0.03724781	0.03733208	0.03909451	[illegible]
ENSG00000069849	9.66691813	9.36840944	9.52966009	8.8028076	10.2674604	9.98274761	7.07359985	7.25092599	7.27146584	5.91135538	7.3352889	7.4336372	9.56323586	6.97756625	[illegible]
ENSG00000069869	0.83196009	0.96429335	1.02779735	0.9735457	0.96705518	0.91678624	0.81173074	0.89655112	1.01704665	1.73557835	1.1741256	1.43973291	2.59096936	2.45270443	[illegible]
ENSG00000069943	0.53135585	0.59598232	0.71330205	0.4730121	0.47955542	0.42302466	0.95206534	1.16368629	0.90828986	1.33027816	1.20936577	0.94614214	0.66188869	0.61636778	[illegible]
ENSG00000069956	9.51233074	8.86584384	8.51812405	6.6161666	7.8571454	6.3874131	9.05932525	9.34792757	11.0464139	9.21530466	8.61737691	10.3621792	12.4099965	11.7103194	[illegible]
ENSG00000069966	1.31254883	1.20366505	1.30568608	1.4903094	1.13229365	1.33671269	0.82637061	0.8405479	0.82158491	1.11227272	1.14496695	0.90546916	1.66245821	1.36904533	[illegible]
ENSG00000069974	0.34065491	0.22025328	0.3400951	0.2323997	0.44602988	0.19342483	1.21017342	1.33237962	1.34265657	0.98989557	1.64215088	1.52032376	3.38591875	3.98773442	[illegible]
ENSG00000069998	3.31858962	1.97190788	2.70961361	3.3211472	4.07041251	3.25902289	1.86128713	2.19352869	2.26267615	1.42309663	1.87380226	2.1910388	1.82210885	1.57799283	[illegible]
ENSG00000070010	2.33548839	2.23832004	2.16224886	1.9421535	2.70545049	2.07163112	2.70082358	2.85763049	3.28881476	1.55827454	2.51566682	2.99792361	3.6059773	2.73253629	[illegible]
ENSG00000070018	3.92991436	5.16137534	4.25353419	4.2996533	3.45371939	4.6178955	5.210363	5.7292836	5.78999235	5.32491905	6.20684759	5.42837221	2.46598754	2.99492112	[illegible]
ENSG00000070019	0	0	0	0	0	0	0.00481274	0	0	0.00542789	0	0	0	0	[illegible]
ENSG00000070031	0.0880594	0	0	0.1138271	0	0.06605554	0.07071829	0.08625488	0	0	0	0	0	0	[illegible]
ENSG00000070047	2.23437978	2.16851118	1.87367844	1.7052651	2.06581619	1.82851433	2.46821891	2.6379528	2.78867411	2.20780161	2.22798041	2.51122548	2.83479	2.20428311	[illegible]
ENSG00000070061	3.97760341	4.0885214	3.56282635	3.6134773	4.03391568	4.07307479	5.69602759	5.66814954	6.03915462	5.99760885	5.2827018	5.59219308	7.11303577	6.30121071	[illegible]
ENSG00000070081	3.25977277	2.9937773	3.19726666	3.7175547	3.23060328	3.39300276	1.64756591	1.89350748	1.56709706	1.81381141	2.14688299	1.76189375	4.05310255	5.40495063	[illegible]
ENSG00000070087	37.9161437	38.0475383	39.6071358	43.018174	44.8041764	39.6323258	8.2359116	9.91175133	9.66450385	10.0904687	10.8611685	10.7060409	10.7940011	13.683196	[illegible]
ENSG00000070159	0.21536001	0.14851514	0.17119215	0.1919848	0.2284291	0.17507546	0.51799864	0.61933163	0.63253888	0.81481708	0.66692788	0.70419159	0.66358144	0.78727067	[illegible]
ENSG00000070182	0.00538981	0.00406146	0.00658893	0.0026126	0.00225491	0.00454841	0.00757474	0.00791905	0.01998508	0.01952671	0.00594143	0.01365508	0.01767772	0.01240787	[illegible]
[illegible]															

ENSG00000073737	0.00206946	0	0	0.002675	0.00115439	0.00155235	0.0614914	0.05270332	0.03785552	0.00187436	0.01825005	0.01398125	0.0175161	0.00672578	0	0.00399272	0.00622643	[illegible]
ENSG00000073754	0	0	0	0	0	0.00897747	0.01922235	0	0.01183372	0	0.01507751	0	0	0	0.0104085	0	0	[illegible]
ENSG00000073756	0.15018254	0.03796024	0.02216994	0.0293024	0.06164577	0.03826041	0.35727221	0.38025253	0.45950295	1.26014439	0.42838486	0.45467014	20.8518202	54.4702758	46.5081704	91.0487443	73.2436443	[illegible]
ENSG00000073792	14.7229287	16.9941799	14.1151956	13.046681	14.0850631	12.778328	4.5230762	5.15481329	4.58713355	4.62232719	4.97071779	4.59634675	5.57717842	6.94126666	8.15340001	5.62337762	6.55136455	[illegible]
ENSG00000073803	3.49926255	3.56906187	3.62578208	3.5383549	2.59714065	3.17431242	0.92726137	0.99826113	0.86548803	0.98531215	0.96975983	0.81091802	0.79363894	0.80731883	0.83255168	0.59765426	0.59524607	[illegible]
ENSG00000073849	0.6719172	0.38960486	0.3454527	0.2448596	1.29038968	0.40204724	0.68098746	0.83278128	0.92991184	1.156587	0.53485088	0.70105805	0.38008808	0.36576786	0.43706169	0.35150218	0.38890102	[illegible]
ENSG00000073861	0	0	0	0	0	0	0	0	0	0	0	0	0.73795804	0.15919031	0.21299463	0.20790523	0.08842291	[illegible]
ENSG00000073905	0.03800677	0.05091507	0.03716994	0	0.06360295	0	0.03052228	0.03722793	0.01879022	0	0.04788178	0	0.03216931	0	0.03305436	0	0	[illegible]
ENSG00000073910	1.26601613	2.14642802	1.55153584	2.24454	1.40713813	2.47127391	0.12803899	0.08658863	0.09911502	0.33599126	0.11136845	0.11997939	0.08283947	0.10944837	0.08511857	0.02741064	0.04512021	[illegible]
ENSG00000073921	6.14529187	5.84047315	6.15132542	5.9668114	5.8244562	5.91436112	6.73558336	7.65517698	7.89254864	8.48035196	9.01181544	7.38553479	7.7577199	7.76946172	7.47376063	8.08144542	8.03875142	[illegible]
ENSG00000073969	2.74845076	2.50712503	2.3053735	2.7264857	2.90370408	2.43566066	2.87040171	2.99515049	3.18656829	2.19756106	2.55424163	2.77448183	2.94955238	2.83070663	2.70606252	3.13231224	2.62311014	[illegible]
ENSG00000074047	1.77972212	2.1388188	1.69745743	1.3195652	1.73119923	1.4457336	0.26748014	0.30959186	0.44662565	0.65303303	0.2872034	0.37068572	0.19230313	0.16827469	0.15794063	0.18637176	0.15926908	[illegible]
ENSG00000074054	4.58725344	4.0614825	4.02035377	2.8919565	4.77364899	3.45241349	1.13677994	1.1847851	1.24497344	1.73444124	1.38123248	1.43308488	1.39995334	1.46367006	1.47174173	1.41255068	1.52713729	[illegible]
ENSG00000074071	12.3487372	10.4636065	11.6341769	10.291409	13.906258	9.92474451	13.8688312	15.9722175	17.7358012	12.2462208	17.1070875	17.3775648	13.7036383	12.5857606	13.0006341	13.0769238	12.134402	[illegible]
ENSG00000074181	7.40085332	6.15854641	5.61557548	5.3824072	5.72735491	5.53304209	6.00833551	6.32133303	3.70108941	6.27820343	4.14860665	2.30323573	0.5737868	0.83167458	0.93121289	0.19272351	0.25664281	[illegible]
ENSG00000074201	8.59358614	9.41947522	9.29731898	11.241528	7.08984872	9.52718034	12.3628875	14.4342207	16.1590575	11.9237492	13.4632465	15.4700655	6.11319172	6.47707544	6.2286166	6.90006991	6.61680777	[illegible]
ENSG00000074211	0	0.00248038	0.00362154	0	0.00309848	0.00277777	0.00297385	0.0018136	0.00183077	0.01173887	0.00233261	0.00156362	0.00626864	0.00401169	0.00644111	0.03572273	0.05292235	[illegible]
ENSG00000074219	6.12013426	7.01512799	6.48162392	5.9548166	5.77018132	5.33041153	2.00528971	2.2801736	2.86730278	3.58792619	2.14486018	2.47709651	2.11349816	1.95771179	2.00325556	1.60802169	1.73089791	[illegible]
ENSG00000074266	1.44646147	1.05647951	1.21252572	1.1492777	1.25841643	1.28127009	0.99567154	1.34625804	1.28496315	0.89457199	1.19415756	1.31279056	1.29008742	0.87694969	0.9513184	1.35147682	0.98675898	[illegible]
ENSG00000074276	0.00213853	0.00572968	0	0.0082929	0.00119292	0.00481249	0.01202178	0.0020947	0.00211454	0.00193691	0.00538833	0.00180598	0.00181007	0.0046335	0	0.00206299	0.00321712	[illegible]
ENSG00000074317	0.49655515	0.11086684	0.21367369	0.119813	0.38409146	0.14899135	0.00531695	0.01945519	0.01963941	0.0479724	0.00834095	0.01118241	0.06724628	0.05020752	0.04606425	0.08302931	0.06971993	[illegible]
ENSG00000074319	4.60217455	3.89750749	4.17428439	4.0309544	4.15123684	3.7723786	6.62228965	6.89973783	6.41527183	5.6567824	6.82581879	5.98795794	4.21727445	4.17101561	4.71461127	4.41440183	4.0884842	[illegible]
ENSG00000074356	1.68848709	2.02751498	1.52476493	1.5354741	1.72637467	1.55776138	2.11233295	2.20864499	2.76230313	2.15178037	2.53606385	2.79452846	2.60155986	2.15781764	2.25620671	2.8873107	2.84324992	[illegible]
ENSG00000074370	0.00379665	0.00445035	0.00464132	0.0147228	0.0042357	0.01067985	0.13796689	0.12272193	0.17034045	0.37138064	0.18773672	0.13225847	0.3904427	0.34446853	0.22122939	0.10438232	0.05425959	[illegible]
ENSG00000074410	9.74449047	5.45341787	6.64200574	5.4284592	8.47639178	5.70468943	12.2707949	12.8002709	8.43822252	13.6343336	11.1939238	8.06255742	0.64980463	0.94120241	0.70954314	1.15385059	1.65455121	[illegible]
ENSG00000074416	0.01957747	0.01049064	0.00510571	0.0067483	0.00436829	0.00979037	0.43393203	0.36179267	0.32134059	1.15847502	0.16442757	0.14549181	12.9548695	13.6020666	15.765367	8.96704098	9.76419557	[illegible]
[illegible]	[illegible]	[illegible]	[illegible]	[illegible]	[illegible]	[illegible]	[illegible]	[illegible]	[illegible]	[illegible]	[illegible]	[illegible]	[illegible]	[illegible]	[illegible]	[illegible]	[illegible]	[illegible]

ENSG00000078295	2.42548109	4.86588709	3.83550584	4.2625253	1.65463411	3.68754393	0.01809471	0.01428063	0.01376059	0.02761022	0.00834884	0.01231229	0.0179493	0.01292272	0.02766469	0.01662157	0.02990823	[illegible]
ENSG00000078304	4.43900597	3.74333473	4.01331949	3.8858029	3.82913202	3.81750207	1.5967707	1.76827576	1.95314498	1.61577843	1.86975237	1.88836138	3.40527174	3.96870808	4.36151773	3.73570338	4.1187756	[illegible]
ENSG00000078319	8.86858611	7.05111101	8.57929822	6.89685	9.49197016	7.40974748	1.64060095	1.85978647	2.36456897	3.85297801	2.02867333	2.45591116	1.93255733	1.95278196	2.21565245	2.08666322	2.09704944	[illegible]
ENSG00000078328	0	0	0	0	0	0	0	0	0.00048535	0	0	0	0.00415467	0.00106353	0.00170759	0.00094704	0	[illegible]
ENSG00000078369	39.5136225	29.0006826	32.6447059	28.643285	47.1330886	30.2624596	16.5067106	18.9911442	19.8614143	18.7703344	21.3873832	21.0775773	30.3404587	31.3138195	32.6934189	34.107093	32.790895	[illegible]
ENSG00000078399	0.00589856	0.00395095	0.00576869	0.0076246	0	0	0	0	0	0	0	0	4.24371094	3.96188868	4.32968506	4.17092051	3.67366123	[illegible]
ENSG00000078401	1.39508957	0.09556907	0.54264817	0.1229534	1.4856749	0.60648933	0.20370206	0.41926746	0.3370224	0.07179342	0.17975096	0.18743329	0.4696434	0.37783882	0.2895386	0.41291914	0.16694373	[illegible]
ENSG00000078403	1.58083207	1.93333701	1.61539077	1.7652577	1.30898564	1.70253301	1.13050605	1.30469115	1.27187563	1.43506191	1.26006401	1.38780281	1.10705613	1.0457792	0.87822748	1.14577346	1.22072099	[illegible]
ENSG00000078487	0.06053845	0.05302642	0.04554271	0.078253	0.04416029	0.06462391	0.14024107	0.18017437	0.1346836	0.17503758	0.11733491	0.14354236	0.14780869	0.1538694	0.19642531	0.10781546	0.11559114	[illegible]
ENSG00000078549	72.0582576	63.7812233	48.5990085	77.526309	113.131341	76.9159842	0.01229494	0.05998439	0.0315377	0.00924431	0.03536072	0.01508399	0.01511812	0.08292869	0.04216387	0.02215355	0.02303154	[illegible]
ENSG00000078579	0	0	0	0	0.0084724	0	0.03659221	0.07438569	0.02252701	0.08253875	0	0.01282657	0.01285559	0	0.02641856	0	0	[illegible]
ENSG00000078589	0	0	0	0	0	0	0	0	0	0	0.00642837	0	0	0	0	0	0	[illegible]
ENSG00000078596	0	0	0	0	0	0	0.11399972	0.04231808	0.02441073	0	0.86308229	0.70364396	0	0	0	0	0	[illegible]
ENSG00000078618	4.25486305	4.35097305	4.20455184	4.1662388	4.58028445	4.08597481	6.014795	6.73317988	6.94203105	6.8981931	6.78509304	6.8868379	6.23999933	6.83788389	6.75993884	6.15936804	6.17716224	[illegible]
ENSG00000078668	8.88267852	5.99782507	7.67406275	6.0494727	9.52178296	6.64673528	5.78794598	6.46971735	7.33462454	6.04735541	5.33783988	7.23002579	6.73670758	5.05652946	5.76339973	7.04595266	6.4068091	[illegible]
ENSG00000078674	8.14997763	7.23680033	7.50887976	5.4161728	7.53685286	6.06391622	4.05817577	4.41729308	4.23996996	4.16976983	3.83758493	4.21102587	4.23108512	3.77936532	3.96197976	5.26040124	4.72170605	[illegible]
ENSG00000078687	0.69817352	0.99390834	0.76838054	0.8152131	0.74962445	0.8359785	0.28611529	0.31040316	0.34084474	0.46364902	0.43073169	0.4682018	0.1920426	0.14287529	0.15057668	0.26831938	0.19558079	[illegible]
ENSG00000078699	0.55313742	0.83997882	0.63274563	0.9094457	0.54024417	0.91472605	0.52065133	0.5880937	0.51863177	0.53655873	0.58867883	0.53963798	0.51663296	0.59058224	0.49727077	0.48479797	0.45260891	[illegible]
ENSG00000078725	0.02975969	0.02790691	0.04074621	0	0.13280469	0.01339409	0.02867911	0.03497982	0.00588517	0	0.07498383	0.01005281	0	0.0193439	0.03105828	0.01148339	0.00895387	[illegible]
ENSG00000078747	7.80038661	7.25605229	7.15343919	7.7921047	7.60979794	7.55489954	6.14932097	6.78002384	7.05763549	5.93333173	6.50129963	6.84388296	7.10172205	7.04988723	7.86872534	8.67576086	7.93293798	[illegible]

[illegible]

ENSG00000083622	0	0	0	0	0	0	0	0	0	0
ENSG00000083635	3.72875569	3.61036268	3.40294393	3.2569793	3.61936993	2.88011255	3.63931852	4.31229949	4.32119069	3.31034298
ENSG00000083642	2.48258013	2.9897616	2.72087198	2.1898808	3.05184131	1.98625345	0.97472182	1.25806584	1.67653894	1.41412902
ENSG00000083720	6.22067529	3.97637302	5.42076191	2.9296139	8.02670091	3.46440072	3.35977213	4.01406802	4.4786109	0.89924047
ENSG00000083750	2.11387561	3.46069832	2.62041091	2.6259727	1.77946691	2.34349769	2.14735413	2.65138985	2.23674666	1.61123523
ENSG00000083782	0.01075041	0	0	0	0	0	0.01726677	0.03159035	0.03720438	0
ENSG00000083799	0.33650907	0.39751437	0.34824071	0.4243681	0.37119768	0.42765236	0.62971954	0.61284576	0.5303149	1.01956788
ENSG00000083807	0.08270701	0.15828133	0.17101605	0.2077079	0.10677126	0.20207505	0.51238221	0.58097302	0.51170454	0.80902383
ENSG00000083812	1.19859181	1.14960055	0.9658278	1.3383796	1.2367531	1.16713314	1.9160797	1.89057449	1.57463181	1.58761427
ENSG00000083814	1.22468929	1.44727233	1.41730688	1.349365	0.87021286	1.14396275	0.00936683	0.02856174	0.01297448	0
ENSG00000083817	4.94583668	4.42549592	5.52617254	5.2218217	4.80173743	5.22042567	3.04207368	2.76123399	2.84959793	3.9780285
ENSG00000083828	2.3362319	2.91558144	2.53179747	2.3873159	1.9834174	2.67310217	1.88567664	1.80903958	1.73056833	1.72995516
ENSG00000083838	1.26406468	1.52229725	1.17760709	1.6474271	1.04968705	1.3880959	1.58026718	1.49089952	1.35296868	1.57924284
ENSG00000083842	0	0	0	0	0	0	0	0	0	0
ENSG00000083844	2.4959207	2.89522569	2.68809713	3.4325668	2.70047469	3.47513951	1.40629197	1.67616846	1.55395884	2.10803071
ENSG00000083845	36.296642	32.7357459	35.7350255	58.749345	30.6107646	43.1347459	64.980632	70.7091461	68.4909973	59.2757828
ENSG00000083857	13.3862673	16.5843334	13.8034026	12.159276	11.5054366	13.7753856	19.1708883	21.8175876	28.2222715	39.0729243
ENSG00000083896	6.02433347	7.35024284	6.56080516	5.9695775	5.2997289	6.90847485	4.91113126	5.59844062	5.85462239	5.22902647
ENSG00000083937	10.3538793	10.5313543	10.5871517	9.22249	8.16478527	7.98884323	9.24420481	9.87849557	10.2106247	6.67071465
ENSG00000084070	4.92364553	3.44659069	4.04351279	3.5806316	6.39859884	3.84132956	4.10258041	4.71808265	4.73024707	4.00532236
ENSG00000084072	0.77909252	0.63582828	0.4764155	0.9186531	0.89245402	0.80341651	1.77384698	2.02302278	1.99434065	1.82681134
ENSG00000084073	8.03021388	7.31147316	7.10134159	6.0256717	9.96861705	5.57954561	20.2920567	20.9773112	20.2305271	14.4291837
ENSG00000084090	56.4191678	47.4502503	50.6666177	47.272846	55.7395163	46.2432954	17.6046452	19.1540218	20.4415573	14.9402455
ENSG00000084092	7.46521937	7.8007556	7.50390825	12.32132	5.848914	9.25992352	7.95813683	8.21818331	8.71126644	8.95823841
ENSG00000084093	9.65653709	11.0257832	10.1813364	9.647692	9.62317679	9.79021114	7.86608254	8.29193293	8.94132387	7.38358042
ENSG00000084110	0.00178291	0.00119422	0	0	0.00198909	0.00133741	0.03293171	0.03842033	0.02996955	0.0565189
ENSG00000084112	1.16872287	0.93170197	0.97762564	0.6996832	1.31574121	0.78495094	1.77978514	1.87601167	1.7786346	3.08796672
ENSG00000084207	44.2561815	34.0727628	39.6268472	38.991076	44.8833903	32.7198971	29.2940789	32.7436166	37.5824469	25.5603326
ENSG00000084234	11.5188254	9.63459794	9.83215309	8.5727003	10.1489712	7.89543804	18.2011833	20.2012498	15.5619045	23.9117899
ENSG00000084444	1.87354436	1.45223452	1.63764407	1.752871	1.47291419	1.65820357	0.55836294	0.67063615	0.5326675	0.72587438
ENSG00000084453	0.02082563	0.05691329	0.03258734	0.0053839	0.00185872	0.00499898	0.00200694	0.00407977	0.00123552	0.00226347
ENSG00000084463	17.8240228	16.4258273	15.8518951	13.182845	17.3393415	13.741247	15.7720735	19.1145041	22.3566519	14.1930049
ENSG00000084623	26.7531887	23.0661853	24.7010119	30.040158	26.9393311	26.5782205	43.0729171	44.3932735	52.4945272	43.8327943
ENSG00000084628	0.18404374	0.29586099	0.31678499	0.1570127	0.16426163	0.19880033	0.01773611	0.00721089	0.01637813	0.28671125
ENSG00000084636	0.02215255	0.16470321	0.04982902	0.0472473	0.00617858	0.0523441	0.21081331	0.22458047	0.25025384	0.14446126
ENSG00000084652	11.9301652	10.7830547	10.6323652	10.962009	11.964971	9.96850881	15.3314033	17.1541604	16.0019377	12.8972702
ENSG00000084674	0	0	0.00365472	0	0.00104229	0	0	0	0.00092377	0
ENSG00000084676	3.82435979	4.85535212	3.91589821	3.8658491	3.48542268	3.89036809	0.73729461	0.75700646	0.7217574	0.88415555
ENSG00000084693	5.71005826	5.48753506	5.27151482	6.6526951	5.7119346	5.7368086	1.34237054	1.41089526	1.52933949	3.06341318
ENSG00000084710	0.95946774	1.56586252	0.99605771	1.0015302	0.95573488	1.03674041	0.04586455	0.06153494	0.0894117	0.49830234
[illegible]

ENSG00000088538	0.03313166	0.18493424	0.12960866	[illegible]
ENSG00000088543	2.05445821	1.87900553	1.88684529	[illegible]
ENSG00000088682	4.67168236	4.59237498	4.82137977	[illegible]
ENSG00000088726	0.00640432	0.00214486	0.01878993	[illegible]
ENSG00000088727	0.21351497	0.2306705	0.20746662	[illegible]
ENSG00000088756	0.42337119	0.31291667	0.29411786	[illegible]
ENSG00000088766	2.54315001	2.61794662	2.82269612	[illegible]
ENSG00000088782	0	0	0	[illegible]
ENSG00000088808	0.28851527	0.32016393	0.30532537	[illegible]
ENSG00000088812	7.2294844	9.41658725	7.14060724	[illegible]
ENSG00000088826	1.0969159	0.9892637	1.0210415	[illegible]
ENSG00000088827	0.0236836	0.06345461	0.05523279	[illegible]
ENSG00000088832	6.27481659	6.4399251	7.16619641	[illegible]
ENSG00000088833	1.68894818	1.5516163	1.53908644	[illegible]
ENSG00000088836	0.03260469	0.02495902	0.01822103	[illegible]
ENSG00000088854	2.07479614	2.53568138	2.06029052	[illegible]
ENSG00000088876	0.91618185	0.65229591	0.83021849	[illegible]
ENSG00000088881	1.09309498	1.82056995	1.23999963	[illegible]
ENSG00000088882	18.7905718	20.0801719	19.2337246	[illegible]
ENSG00000088888	4.78171822	5.52108333	4.59543886	[illegible]
ENSG00000088899	1.44923124	2.00734906	2.09536203	[illegible]
ENSG00000088926	0.00688449	0	0.00336645	[illegible]
ENSG00000088930	12.7310754	11.4710932	11.7767079	[illegible]
ENSG00000088970	0.76391584	1.47928849	1.09350585	[illegible]
ENSG00000088986	23.2613964	18.6158448	21.5104464	[illegible]
ENSG00000088992	0.03297401	0.04732825	0.02764113	[illegible]
ENSG00000089006	4.01275545	3.27617002	3.77302753	[illegible]
ENSG00000089009	32.2646961	33.3517284	34.0698622	[illegible]
ENSG00000089012	0	0	0	[illegible]
ENSG00000089022	2.90389037	2.99833202	2.81750241	[illegible]
ENSG00000089041	0.15611709	0.2077263	0.27647406	[illegible]
ENSG00000089048	9.83127323	10.3375328	9.89903846	[illegible]
ENSG00000089050	6.67337092	6.62411257	5.86929997	[illegible]
ENSG00000089053	1.83896517	1.98595885	1.80016124	[illegible]
ENSG00000089057	6.22651281	4.1912421	4.1621059	[illegible]
ENSG00000089060	0.40417809	0.33903865	0.33001479	[illegible]
ENSG00000089063	5.7668395	5.29660918	5.47608148	[illegible]
ENSG00000089091	0.16530522	0.14528532	0.11774484	[illegible]
ENSG00000089094	1.39947454	1.53467534	1.28112557	[illegible]
ENSG00000089101	0.00839629	0.00642739	0.0058653	[illegible]
ENSG00000089116	0	0	0	[illegible]
ENSG00000089123	0.47847729	0.51977176	0.54293294	[illegible]
ENSG00000089127	0.41958523	0.20984681	0.45411714	[illegible]
ENSG00000089154	7.90084058	9.24698133	7.47061916	[illegible]
ENSG00000089157	23.5654532	21.0227698	22.6127967	[illegible]
ENSG00000089159	0.69316682	0.61381257	0.60075816	[illegible]
ENSG00000089163	0.10515413	0.19369323	0.25709712	[illegible]
ENSG00000089169	3.30710509	2.31777683	1.99408824	[illegible]
ENSG00000089177	1.07722209	0.98458038	0.96545886	[illegible]
ENSG00000089195	1.27334889	1.01206459	1.06953859	[illegible]
ENSG00000089199	0.12984481	0.200414	0.14354927	[illegible]
ENSG00000089220	80.4738409	74.8982093	79.0165168	[illegible]
ENSG00000089225	0.65090311	0.66838263	0.56085599	[illegible]
ENSG00000089234	3.30652598	3.6150656	3.30326545	[illegible]
ENSG00000089248	11.9983661	9.2633684	10.2442988	[illegible]
ENSG00000089250	0.01039459	0.00696246	0	[illegible]
ENSG00000089280	2.96080283	4.63223121	3.70373034	[illegible]
ENSG00000089289	8.08484118	9.16632933	8.31780592	[illegible]
ENSG00000089327	0.02312033	0.00387159	0.00282641	[illegible]
ENSG00000089335	2.42276697	3.13881274	2.71595605	[illegible]
ENSG00000089351	1.97494049	1.83266139	1.83540877	[illegible]
ENSG00000089356	0	0	0	[illegible]
ENSG00000089472	0.0309738	0.02141599	0.02442889	[illegible]
ENSG00000089486	1.81463685	1.92286972	1.89832034	[illegible]
ENSG00000089505	0.16861598	0.12235346	0.14723518	[illegible]
ENSG00000089558	0	0.00564909	0.00412405	[illegible]
ENSG00000089597	12.9606789	10.4423442	9.7621724	[illegible]
ENSG00000089639	0.37387883	0.49931407	0.38370341	[illegible]
ENSG00000089682	0.70460306	0.96306541	0.90013857	[illegible]
ENSG00000089685	15.6272278	8.50589448	11.6773997	[illegible]
ENSG00000089692	0.08977643	0.06962847	0.11552597	[illegible]
ENSG00000089693	15.1825785	12.3811293	12.9690332	[illegible]
ENSG00000089723	0.72395096	0.72737074	0.61950966	[illegible]
ENSG00000089737	7.86183942	6.7041118	6.91379752	[illegible]
ENSG00000089775	1.20936692	1.26207173	1.10214733	[illegible]
ENSG00000089818	3.15511627	2.7396563	2.82176021	[illegible]
ENSG00000089820	0.02373732	0.03709914	0.03869112	[illegible]
ENSG00000089847	0.00833348	0.02651402	0.01222499	[illegible]
ENSG00000089876	5.04243481	4.12568375	4.3869441	[illegible]
ENSG00000089902	4.63810331	3.97012496	4.12338822	[illegible]
ENSG00000089916	2.33462635	3.06976963	2.48391354	[illegible]
ENSG00000090006	0.5964243	0.4769209	0.60704194	[illegible]
ENSG00000090013	6.93971834	4.34165158	5.90014252	[illegible]
ENSG00000090020	0.38111372	0.29408991	0.25502057	[illegible]
ENSG00000090054	5.69106535	5.43644414	4.94792371	[illegible]
ENSG00000090060	5.71705431	6.53577583	6.11497293	[illegible]
ENSG00000090061	0.91143067	1.1849004	0.92718592	[illegible]
ENSG00000090097	4.41709782	3.47656795	3.97726706	[illegible]
ENSG00000090104	0	0	0	[illegible]
ENSG00000090238	2.01926668	2.652475	2.30119818	[illegible]
ENSG00000090263	5.4649719	4.88434205	5.40314296	[illegible]
ENSG00000090266	8.4693092	5.99171811	8.16796088	[illegible]
ENSG00000090273	16.075021	13.0307332	13.624418	[illegible]
ENSG00000090316	1.01356422	0.96669653	0.9452114	[illegible]
ENSG00000090339	0.91128817	0.16897948	1.40984502	[illegible]
ENSG00000090372	3.3437225	3.00305759	2.63544634	[illegible]
ENSG00000090376	0.02131773	0.02141843	0.03127253	[illegible]
ENSG00000090382	0.35070446	0.43066358	0.38871368	[illegible]
ENSG00000090402	0.08360503	0.02799999	0.04088211	[illegible]
ENSG00000090432	13.6539784	10.1382025	11.8653275	[illegible]
ENSG00000090447	1.06225716	1.14066016	1.05750284	[illegible]
ENSG00000090470	8.80096472	9.2868434	8.03902639	[illegible]
ENSG00000090487	4.9714728	4.38391732	4.64834629	[illegible]
ENSG00000090512	0	0	0	[illegible]
ENSG00000090520	11.6428007	8.62711252	9.61602587	[illegible]
ENSG00000090530	0.07329426	0.08496979	0.1213054	[illegible]
ENSG00000090534	0	0	0	[illegible]
ENSG00000090539	0.00088436	0.00177708	0.00432446	[illegible]
ENSG00000090554	0.04055756	0.04527683	0.02379874	[illegible]
ENSG00000090565	1.65888366	1.83964664	1.53180105	[illegible]
ENSG00000090581	3.66449763	4.02036494	3.60562105	[illegible]
ENSG00000090612	1.20712863	1.48301264	1.22804351	[illegible]
ENSG00000090615	2.02243356	2.49030751	2.03038861	[illegible]
ENSG00000090621	3.62874566	2.90923314	3.00624676	[illegible]
ENSG00000090659	0	0.00677483	0.00824314	[illegible]
ENSG00000090661	0.73744396	0.91624192	0.84639053	[illegible]
ENSG00000090674	0.80404821	0.87814088	0.67897234	[illegible]
ENSG00000090686	2.22845409	2.36071598	2.30625735	[illegible]
ENSG00000090776	16.0565179	15.0803253	14.0195043	[illegible]
ENSG00000090857	0.9995263	1.2728738	0.93667278	[illegible]
ENSG00000090861	14.3709675	13.6374189	13.0515587	[illegible]
ENSG00000090863	5.57025527	6.02581012	5.39509702	[illegible]
ENSG00000090889	11.4007142	8.71754686	9.15064177	[illegible]
ENSG00000090905	2.26585018	2.92285265	2.37299162	[illegible]
ENSG00000090920	0.14295449	0.35441114	0.20698688	[illegible]
ENSG00000090924	1.66980669	1.58805195	1.42765232	[illegible]
ENSG00000090932	6.09226473	12.8581603	6.57480306	[illegible]
ENSG00000090971	4.09906318	4.11078537	4.04599764	[illegible]
ENSG00000090975	0.13756476	0.12064774	0.1442147	[illegible]
ENSG00000090989	2.91825057	3.3842604	2.79683788	[illegible]
ENSG00000091009	7.06119996	7.74124419	7.07235285	[illegible]
ENSG00000091010	0.02726712	0.0365279	0	[illegible]
ENSG00000091039	6.23423557	6.4089801	5.81376041	[illegible]
ENSG00000091073	0.28537266	0.29420036	0.25846099	[illegible]
ENSG00000091106	0.00276484	0	0.00270396	[illegible]
ENSG00000091127	2.47739581	1.77905632	1.74049533	[illegible]
ENSG00000091128	0.03283021	0.02057148	0.01864298	[illegible]
ENSG00000091129	7.21300676	8.254427	6.07788147	[illegible]
ENSG00000091136	4.36141622	2.76233318	4.25199229	[illegible]
ENSG00000091137	0.05878658	0.04593888	0.02874611	[illegible]
ENSG00000091138	0	0	0.00320979	[illegible]
ENSG00000091140	8.7668119	7.61770154	8.39541419	[illegible]
ENSG00000091157	1.30887187	1.4511273	1.29655226	[illegible]
ENSG00000091164	2.74986042	2.38375325	2.56793668	[illegible]
ENSG00000091181	0.0653046	0.21227762	0.09016479	[illegible]
ENSG00000091262	0.02131981	0.02596427	0.03032784	[illegible]
ENSG00000091317	19.0684574	17.2226529	17.3136118	[illegible]
ENSG00000091409	3.00207913	1.54453634	3.10044766	[illegible]
ENSG00000091428	0.0664683	0.10883038	0.06590765	[illegible]
ENSG00000091436	3.73044529	3.30515931	3.39123527	[illegible]
ENSG00000091482	0	0	0	[illegible]
ENSG00000091483	61.4151403	46.7571271	52.8779931	[illegible]
ENSG00000091490	1.00403684	0.36941228	0.51939333	[illegible]
ENSG00000091513	0.07822752	0.09261048	0.09073862	[illegible]
ENSG00000091527	24.8102557	28.7773273	26.308747	[illegible]
ENSG00000091536	0.00261725	0.00467488	0.00085321	[illegible]
ENSG00000091542	19.7127582	13.6423876	17.1980763	[illegible]
ENSG00000091583	0	0.00710599	0	[illegible]
ENSG00000091592	0.83600248	0.72795803	0.78633441	[illegible]
ENSG00000091622	0.06962987	0.05358545	0.05505695	[illegible]
ENSG00000091640	7.91277944	5.35641871	6.34561607	[illegible]
ENSG00000091651	2.28956065	1.37982624	1.6502063	[illegible]
ENSG00000091656	0.53758176	1.02931459	0.73101638	[illegible]
ENSG00000091664	0	0	0.01394078	[illegible]
ENSG00000091704	0	0	0	[illegible]
ENSG00000091732	2.86957562	2.4883453	2.66898494	[illegible]
ENSG00000091831	0.02857694	0.0399715	0.03698964	[illegible]
ENSG00000091844	2.2162442	1.68408812	2.03009721	[illegible]
ENSG00000091879	0.05537756	0.0285329	0.05415826	[illegible]
ENSG00000091947	2.52805186	2.59869937	2.40762719	[illegible]
ENSG00000091972	2.38421009	3.10775104	2.61044146	[illegible]
ENSG00000091986	19.8386196	17.1562921	16.6469719	[illegible]
ENSG00000092009	0	0	0	[illegible]
ENSG00000092010	10.3869404	9.29537791	9.71124969	[illegible]
ENSG00000092020	2.69122099	2.13987975	2.4140651	[illegible]
ENSG00000092036	0.74879771	0.57717293	0.64426572	[illegible]
ENSG00000092051	0.44518398	0.83281827	0.53585469	[illegible]
ENSG00000092054	0.02647424	0.00709315	0.03624786	[illegible]
ENSG00000092067	0	0	0	[illegible]
ENSG00000092068	0.00277627	0.00185959	0.00135757	[illegible]
ENSG00000092094	0.75209626	1.14618319	0.94472595	[illegible]

ENSG00000092096	2.95814919	2.90472775	2.74147781	3.6052531	2.51222138	[illegible]
ENSG00000092098	0.61500208	0.62018731	0.59369304	0.7084255	0.56712709	[illegible]
ENSG00000092108	1.96574496	1.75818546	1.90899048	1.858801	1.74795266	[illegible]
ENSG00000092140	1.02805311	1.03843298	0.93148972	1.0588392	1.16993995	[illegible]
ENSG00000092148	3.97654815	4.06339473	3.51737951	3.7250256	4.01682854	[illegible]
ENSG00000092199	11.1162951	9.54785924	10.35362	10.262758	10.3089439	[illegible]
ENSG00000092200	0	0.00139075	0	0.0013419	0.00057911	[illegible]
ENSG00000092201	20.4109116	19.1903272	18.4205336	14.612232	19.9797116	[illegible]
ENSG00000092203	3.21706568	3.23918795	3.10722958	3.2706133	2.79728629	[illegible]
ENSG00000092208	1.16102754	0.80345392	0.99117859	1.0613135	1.06807898	[illegible]
ENSG00000092295	0.01927385	0.01549192	0.00753979	0.0348792	0.01075137	[illegible]
ENSG00000092330	2.98699228	2.52429154	2.74649734	3.1008572	2.76455552	[illegible]
ENSG00000092345	0.00468524	0.02510596	0.02749246	0.0242249	0.00261352	[illegible]
ENSG00000092377	0	0	0	0	0	[illegible]
ENSG00000092421	5.50123059	3.74463218	4.71544715	2.672074	7.69332666	[illegible]
ENSG00000092439	2.29432046	2.17264385	2.10807859	2.0362789	2.08691499	[illegible]
ENSG00000092445	10.827319	11.5919304	11.6548634	10.776126	8.70657721	[illegible]
ENSG00000092470	10.7407333	6.40139854	8.25517824	4.4004695	9.82725779	[illegible]
ENSG00000092529	0.01581495	0.02194286	0.01767627	0.0160621	0.00882192	[illegible]
ENSG00000092531	1.46565605	2.16150555	1.89142194	2.0797114	2.03573914	[illegible]
ENSG00000092607	0.02332109	0.02677857	0.01629114	0.0215323	0.00743371	[illegible]
ENSG00000092621	12.6458674	10.926929	11.6552211	23.058616	15.1165243	[illegible]
ENSG00000092758	0.044179	0.0249194	0.05457633	0.0631177	0.02723809	[illegible]
ENSG00000092820	10.7720778	9.93100673	9.33302498	9.2656979	9.86069817	[illegible]
ENSG00000092841	41.3916608	27.763491	41.1987308	23.501661	39.7081806	[illegible]
ENSG00000092847	4.80009231	6.8328961	4.86340261	5.2268223	4.04530853	[illegible]
ENSG00000092850	0.20949331	0.4587448	0.19700067	0.5624195	0.08539757	[illegible]
ENSG00000092853	2.60211529	1.81175387	1.93503485	0.904423	2.7893994	[illegible]
ENSG00000092871	0.97201125	0.71705982	0.78671135	0.6918943	0.95523892	[illegible]
ENSG00000092929	0.00891186	0.00895396	0	0.0134396	0.00082854	[illegible]
ENSG00000092931	1.00945255	0.9163463	0.88363633	0.8924348	0.79445391	[illegible]
ENSG00000092964	60.4567247	62.0919362	57.0170379	46.000116	49.4885515	[illegible]
ENSG00000092969	0.67510976	0.96951512	0.95075311	1.1065306	0.73209212	[illegible]
ENSG00000092978	4.68397013	5.38627412	5.14151422	4.083687	4.8343942	[illegible]
ENSG00000093000	4.53297771	3.46654692	3.63572468	2.8482246	4.28588774	[illegible]
ENSG00000093009	2.25480689	1.37970249	1.66487184	0.7819662	2.52392518	[illegible]
ENSG00000093010	3.16501385	2.88625051	3.11231066	3.3868436	2.69791562	[illegible]
ENSG00000093072	0.00379374	0.00762333	0.00556532	0.0024519	0.00317435	[illegible]
ENSG00000093100	0.03723399	0.06484378	0.01456567	0.0577551	0.02907788	[illegible]
ENSG00000093134	0.00509051	0.00909255	0.00663791	0.0065801	0.00662573	[illegible]
ENSG00000093144	1.82661195	1.77253842	1.9319343	1.5854492	2.26345518	[illegible]
ENSG00000093167	1.23538544	1.08823259	1.14524774	0.9327633	1.31527994	[illegible]
ENSG00000093183	4.21007217	3.25767381	3.71669564	3.3008913	4.13418815	[illegible]
ENSG00000093217	0.75639504	0.92853598	0.76174049	0.4688737	0.73563828	[illegible]
ENSG00000094631	1.24510953	1.60037957	1.24879635	1.5460009	1.08790762	[illegible]
ENSG00000094661	0	0	0	0	0	[illegible]
ENSG00000094755	0.04657477	0.05910919	0.06472793	0.0918893	0.0642673	[illegible]
ENSG00000094796	0	0	0	0	0	[illegible]
ENSG00000094804	9.44897823	4.6130647	5.90626474	2.6286108	9.80486134	[illegible]
ENSG00000094841	0.67522548	0.94978113	0.77742192	0.7577558	0.90397235	[illegible]
ENSG00000094880	7.04191646	6.83685902	5.97345167	7.1286942	5.91287076	[illegible]
ENSG00000094914	1.89754332	1.77289422	1.6869085	2.257786	1.70274771	[illegible]
ENSG00000094916	36.9735199	36.812799	33.9895905	24.349476	41.0106524	[illegible]
ENSG00000094963	0	0	0	0	0	[illegible]
ENSG00000094975	9.34195356	8.82037127	8.93839896	12.144017	8.51960891	[illegible]
ENSG00000095002	12.90107	10.9360973	10.8225949	7.2819142	13.0384434	[illegible]
ENSG00000095015	39.7071356	35.3431882	34.6533063	20.014541	35.3365771	[illegible]
ENSG00000095059	3.73376734	3.1844304	3.38163489	4.6404123	3.65488508	[illegible]
ENSG00000095066	0.30790832	0.40544134	0.29672204	0.3785925	0.30497485	[illegible]
ENSG00000095110	0	0.00236788	0	0	0	[illegible]
ENSG00000095139	11.8775793	11.1821995	11.0814406	13.558873	12.6348756	[illegible]
ENSG00000095203	0.14132968	0.2905991	0.26036392	0.3058913	0.20900893	[illegible]
ENSG00000095209	0.9716448	0.90785105	0.846963	1.3788305	0.83068029	[illegible]
ENSG00000095261	3.43488769	2.85533641	3.01451357	3.1842912	3.44569523	[illegible]
ENSG00000095303	0.01326873	0.00533256	0.00519063	0.0034303	0.00296063	[illegible]
ENSG00000095319	6.0586773	4.6750442	4.27094008	3.759143	6.26048068	[illegible]
ENSG00000095321	9.86087017	8.00831258	7.95057575	10.103004	9.70478581	[illegible]
ENSG00000095370	0.03439826	0.02112046	0.04485451	0.0092633	0.00559652	[illegible]
ENSG00000095380	2.17989023	1.62547508	1.87410215	3.2125739	3.13449802	[illegible]
ENSG00000095383	0.05920503	0.04130882	0.02171305	0.070152	0.03990621	[illegible]
ENSG00000095397	0.72911738	1.00070675	0.81800516	1.202093	0.830317	[illegible]
ENSG00000095464	0	0	0	0	0.00475243	[illegible]
ENSG00000095485	2.14490869	1.9731051	1.79836962	1.9285836	2.07569334	[illegible]
ENSG00000095539	0.45584396	0.49590049	0.46579167	0.5465631	0.46910177	[illegible]
ENSG00000095564	8.01746556	10.8124286	8.37409029	7.8192161	7.90366916	[illegible]
ENSG00000095574	2.60009888	3.88185154	3.01261346	3.5364017	2.20762251	[illegible]
ENSG00000095585	0	0	0	0	0	[illegible]
ENSG00000095587	0.04443293	0.03401357	0.031039	0.0574347	0.05665287	[illegible]
ENSG00000095596	0.00496224	0.00997136	0	0	0	[illegible]
ENSG00000095627	0	0	0.00154419	0	0	[illegible]
ENSG00000095637	0.813848	1.12318918	0.88349652	0.6479371	0.47338076	[illegible]
ENSG00000095713	0.00493489	0	0	0.0031895	0.00137639	[illegible]
ENSG00000095739	0.37705625	0.31569781	0.27656568	0.4763122	0.23901137	[illegible]
ENSG00000095752	0.03123037	0.01673488	0.02443419	0.0322951	0.01393677	[illegible]
ENSG00000095777	0	0	0.00258362	0	0	[illegible]
ENSG00000095787	4.90926568	5.38103347	4.84917616	4.8902063	4.8035514	[illegible]
ENSG00000095794	0.19583528	0.16416572	0.17643367	0.1733625	0.22642698	[illegible]
ENSG00000095906	1.96129953	1.79532353	1.75624207	2.4661921	1.61824778	[illegible]
ENSG00000095917	0	0	0	0	0	[illegible]
ENSG00000095932	0.01197686	0.00802229	0.03513947	0.030963	0.0267238	[illegible]
ENSG00000095951	1.66986822	1.75326455	1.47893351	1.2842007	1.54089186	[illegible]
ENSG00000095970	0	0	0	0	0	[illegible]
ENSG00000095981	0	0	0	0	0	[illegible]
ENSG00000096006	0	0	0	0	0	[illegible]
ENSG00000096060	1.3549898	0.66132262	0.93923315	0.7190297	2.13580661	[illegible]
ENSG00000096063	2.88921035	2.59588136	2.63017867	2.4402062	3.0049383	[illegible]
ENSG00000096070	0.76869554	0.90635589	0.73368661	0.667541	0.72239388	[illegible]
ENSG00000096080	6.05675864	5.76765937	6.03045059	5.9897085	7.40168777	[illegible]
ENSG00000096088	0	0	0	0.0122459	0	[illegible]
ENSG00000096092	16.6671941	14.7546952	17.5711885	15.79075	15.2780451	[illegible]
ENSG00000096093	1.81327209	1.49224072	1.31326433	1.2208606	1.11001579	[illegible]
ENSG00000096264	0	0	0	0	0	[illegible]
ENSG00000096384	111.689949	96.6341911	93.5130064	118.91269	111.346824	[illegible]
ENSG00000096395	0.10793617	0.1012163	0.10555963	0	0.04816727	[illegible]
ENSG00000096401	20.1718128	20.1091309	17.5880526	14.451198	19.000096	[illegible]
ENSG00000096433	0.50662817	0.65584347	0.66730405	0.2425467	0.41963003	[illegible]
ENSG00000096654	7.50952937	6.39004521	6.88188995	5.9088763	6.45425817	[illegible]
ENSG00000096696	0	0	0	0	0	[illegible]
ENSG00000096717	2.11094645	2.31726085	2.11106512	2.2252246	1.91478769	[illegible]
ENSG00000096746	4.5517333	5.59357414	5.16205975	4.9736428	5.67200425	[illegible]
ENSG00000096872	1.42658977	1.55324112	1.4389495	1.2980552	1.21707857	[illegible]
ENSG00000096968	1.49505672	1.58895141	1.41000402	1.3061058	1.4949364	[illegible]
ENSG00000096996	0.00656678	0.00879706	0.01284438	0.0127325	0.0164839	[illegible]
ENSG00000097007	11.227234	12.4405008	10.9644631	10.363727	9.84178111	[illegible]
ENSG00000097021	1.5103627	0.76109358	0.97234765	0.6955949	2.01979811	[illegible]
ENSG00000097033	12.0093426	11.9541293	11.8274693	12.154311	12.5920612	[illegible]
ENSG00000097046	3.43854298	3.14166015	3.22613273	1.7586148	3.58667699	[illegible]
ENSG00000097096	0.04922305	0.09130259	0.08516946	0.014683	0.02323341	[illegible]
ENSG00000099139	0.29922789	0.16255878	0.22790819	0.224586	0.34460013	[illegible]
ENSG00000099194	138.133467	84.1335768	108.045423	88.853374	228.442136	[illegible]
ENSG00000099203	3.43282512	2.65685846	2.97762392	4.1158955	2.94678526	[illegible]
ENSG00000099204	2.42844886	2.84929378	2.54310779	2.5852575	2.3830799	[illegible]
ENSG00000099219	1.27076224	1.25249524	1.16684888	1.9119161	1.00553923	[illegible]
ENSG00000099246	8.52990948	9.99963199	9.30345532	9.2838932	8.69205329	[illegible]
ENSG00000099250	3.9584177	3.51377146	4.141192	3.110293	3.4016647	[illegible]
ENSG00000099251	0.2884332	0.5080369	0.36043874	0.3037903	0.27113649	[illegible]
ENSG00000099256	26.3603961	18.3548241	22.4277682	17.043015	30.2731282	[illegible]
ENSG00000099260	0.12633843	0.23046395	0.26288663	0.201528	0.05697919	[illegible]
ENSG00000099282	1.50479266	2.26532318	2.12135633	1.1254375	0.57270468	[illegible]
ENSG00000099284	14.9542421	13.6270453	14.1229287	11.064321	12.3072597	[illegible]
ENSG00000099290	0.0748019	0.08791746	0.08971829	0.0675007	0.06770649	[illegible]
ENSG00000099308	0.75238899	1.08303387	0.79596229	1.1736068	0.86764613	[illegible]
ENSG00000099326	0.61436934	1.04529718	0.61938669	0.9730716	0.49079213	[illegible]
ENSG00000099330	0.25467989	0.23200053	0.30386827	0.2995752	0.18752694	[illegible]
ENSG00000099331	3.00434987	2.74864411	2.67601745	1.7347654	2.83269555	[illegible]
ENSG00000099337	0	0.00686859	0.01002867	0	0.00572015	[illegible]
ENSG00000099338	0.017651	0.03546875	0.02205746	0.0494346	0.01148712	[illegible]
ENSG00000099341	19.7289815	13.175486	16.3338417	15.733993	22.1915853	[illegible]
ENSG00000099364	1.42119181	1.35227463	1.25591606	1.3116271	1.21770977	[illegible]
ENSG00000099365	0.57387486	0.84449424	0.58958473	0.6631245	0.48988002	[illegible]
ENSG00000099377	0.17982609	0.15330047	0.29977276	0.1373546	0.17782373	[illegible]
ENSG00000099381	2.18597418	2.73262213	1.96069638	2.1764245	2.13564243	[illegible]
ENSG00000099385	9.73758716	6.66899309	8.75092175	6.5015104	7.68452415	[illegible]
ENSG00000099399	0	0	0	0	0	[illegible]
ENSG00000099617	9.18916052	7.26315502	8.28083063	5.9876324	5.99840459	[illegible]
ENSG00000099622	2.74393006	3.81901969	2.81200083	3.2680362	2.58689076	[illegible]
ENSG00000099624	5.21942497	3.97865488	4.44493315	5.6854351	5.49120383	[illegible]
ENSG00000099625	1.3621866	0.88141921	0.98146779	0.7962787	1.39226395	[illegible]
ENSG00000099715	0.01064541	0.02139139	0.01636017	0.0196578	0.01866303	[illegible]
ENSG00000099721	0	0	0	0	0	[illegible]
ENSG00000099725	0.01953712	0.03271569	0.01592246	0.012627	0.01634733	[illegible]
ENSG00000099769	0	0	0	0.0083622	0.00360868	[illegible]
ENSG00000099783	8.84479808	9.29829684	8.10660355	5.9761559	10.1461322	[illegible]
ENSG00000099785	2.26905785	2.32323424	2.48319447	3.3410161	1.94456631	[illegible]
ENSG00000099795	56.4020446	43.766596	53.762597	64.14176	63.7895227	[illegible]
ENSG00000099797	2.03721671	1.56455517	1.54666481	2.132895	2.2050678	[illegible]
ENSG00000099800	12.4265203	9.58360872	10.6156427	11.343102	15.0786874	[illegible]
ENSG00000099804	11.4885149	10.8833495	10.3536244	15.057171	12.1622327	[illegible]
ENSG00000099810	1.70108131	1.39756964	1.51364399	1.0068264	1.44867555	[illegible]
ENSG00000099812	0	0.00751671	0	0	0.0062599	[illegible]
ENSG00000099814	0.69332641	0.56777527	0.58315513	0.4700166	0.62871695	[illegible]
ENSG00000099817	9.34987727	6.81665023	7.56022313	8.8742574	8.88305324	[illegible]
ENSG00000099821	2.7574397	2.5676069	2.12691609	3.7283607	3.01839485	[illegible]
ENSG00000099822	3.38563581	4.04774823	3.79203161	4.8530806	3.43426091	[illegible]
ENSG00000099834	0	0.00134295	0.00196082	0	0	[illegible]
ENSG00000099840	0.05854147	0.15150092	0.10149308	0.1410245	0.0757018	[illegible]
ENSG00000099849	0.51714532	0.25932089	0.35651852	0.4493	0.3136965	[illegible]
ENSG00000099860	0.31905369	0.17452756	0.35363261	0.4742763	0.40044361	[illegible]
ENSG00000099864	4.13931479	3.50211342	3.9393808	4.2235886	3.09117096	[illegible]
ENSG00000099866	0.01852639	0.03309138	0.01207898	0.047895	0.01377922	[illegible]
ENSG00000099869	0	0	0	0	0	[illegible]
ENSG00000099875	2.21077846	2.05581156	1.77683352	2.302872	1.94494426	[illegible]
ENSG00000099889	1.30333213	2.44066419	1.56346238	2.0646646	1.10368797	[illegible]
ENSG00000099899	0.93221872	1.06329471	0.95707156	1.4649038	0.95139349	[illegible]
ENSG00000099901	7.61540719	5.28276313	6.83049107	4.0880876	8.32651349	[illegible]
ENSG00000099904	1.20119507	1.53743291	1.16336399	1.5286123	1.04013914	[illegible]
ENSG00000099910	2.38923869	2.58915932	2.47212167	2.6803059	2.27660121	[illegible]
ENSG00000099917	0.61670988	0.6060516	0.54430423	0.5450872	0.55699171	[illegible]
ENSG00000099937	0	0	0	0	0	[illegible]
ENSG00000099940	6.02272915	6.26923968	5.68317074	5.6529505	5.63869098	[illegible]
ENSG00000099942	29.1827554	29.616776	28.0820945	27.439927	26.0250179	[illegible]

ENSG00000099949	1.00467538	1.6158997	1.11396361	1.4372902	1.04659316	1.35191408	3.06595213	3.24447021	2.97838873	4.28517306	[illegible]
ENSG00000099953	1.12097894	0.94461706	1.09837753	1.0915588	0.61462707	0.97490346	0.39801439	0.50837553	0.36175635	0.7744763	[illegible]
ENSG00000099954	1.71598433	2.47643671	1.84420637	2.010698	1.04154199	2.32472357	0.02129929	0.00909254	0.00983425	0	[illegible]
ENSG00000099956	8.37888793	8.21922185	7.60506562	6.9310469	8.05250614	6.54145771	5.15376879	5.33861942	7.53534986	5.07552235	[illegible]
ENSG00000099957	0.00139035	0.00279384	0.00407922	0.0017972	0.00077557	0.00417175	0.27132319	0.30505674	0.2075881	0.25815095	[illegible]
ENSG00000099958	0.33761145	0.271365	0.2694252	0.2059819	0.44445138	0.23299069	0	0	0.0013353	0	[illegible]
ENSG00000099960	0	0	0	0	0	0	0	0	0.00316404	0	[illegible]
ENSG00000099968	2.76498846	2.45412767	2.56335702	3.0348856	2.87382817	2.74288119	3.44191049	3.53743267	2.81537704	2.01825294	[illegible]
ENSG00000099974	0	0	0	0	0	0	0.05025804	0	0	0	[illegible]
ENSG00000099977	4.68535054	3.39946711	4.29454763	5.0130769	5.59346047	4.74598996	3.54296811	3.63797172	3.61831342	4.16928731	[illegible]
ENSG00000099984	0.07206202	0.09653657	0.03523768	0.0232871	0.11054376	0.04054164	0.10127459	0.14117065	0.1692275	0.19580463	[illegible]
ENSG00000099985	0	0	0	0.0251119	0	0	0.0156015	0	0	0.0263935	[illegible]
ENSG00000099991	2.87619165	2.07855677	2.07085148	1.9560291	3.23459421	2.0700046	1.92919115	1.99070756	1.8438259	1.38389351	[illegible]
ENSG00000099992	0.42840824	0.21831058	0.24645621	0.2714547	0.421721	0.24196493	0.3723769	0.33241222	0.31313374	0.20542185	[illegible]
ENSG00000099994	0.02704485	0.02846654	0.04912028	0.0149823	0.03017239	0.00869444	0.19547138	0.20814083	0.09741541	0.04199167	[illegible]

[illegible]

ENSG00000100473	0.34756327	0.25934056	0.21310459	0.6424738	0.30036051	0.46334475	2.09267171	2.52773024	2.68699393	4.05320152	2.18707104	2.59450702	0.40240304	0.24191495	0.33516456	0.20499365	0.15171106	0.10644063	2.09680966	1.48868216	1.84641156	2.96692508	3.19790843	3.05219572	4.04852158	4.798846	2.5643972	6.39833371	6.25773199	5.3265743	0.00273613	0	[illegible]	[illegible]	[illegible]
ENSG00000100478	0.5779465	0.5477304	0.59528626	0.4053216	0.54017618	0.40355376	0.23206714	0.20476073	0.19302093	0.18098325	0.22075672	0.18432634	0.32525244	0.24644839	0.26736068	0.41814868	0.32372816	0.20024346	0.16577638	0.12556348	0.26395787	0.19954515	0.16816325	0.3143921	0.48421999	0.43768262	0.23805814	0.30134097	0.32451285	0.2942758	0.33629702	0.31970112	[illegible]	[illegible]	[illegible]
ENSG00000100479	1.65428113	1.2278544	1.35237873	0.8049779	1.62824907	0.96063298	0.41035105	0.60373289	0.87154441	0.40495134	0.39026588	0.78752014	1.13259422	0.77498277	0.78879772	1.59276583	1.25873267	0.52005363	0.57639497	0.23819607	1.91409818	1.0937592	1.07081661	2.74644205	2.18893562	1.95277308	0.50285339	1.65432693	0.83018122	0.50094722	0.18923649	0.12112581	[illegible]	[illegible]	[illegible]
[illegible]	[illegible]	[illegible]	[illegible]	[illegible]	[illegible]	[illegible]	[illegible]	[illegible]	[illegible]	[illegible]	[illegible]	[illegible]	[illegible]	[illegible]	[illegible]	[illegible]	[illegible]	[illegible]	[illegible]	[illegible]	[illegible]	[illegible]	[illegible]	[illegible]	[illegible]	[illegible]	[illegible]	[illegible]	[illegible]	[illegible]	[illegible]	[illegible]	[illegible]	[illegible]	[illegible]

ENSG00000101335	0	0.02913359	0.08507451	0	0	0	46.6311224	50.300681	39.0503853	24.069942	65.88291	32.4705853	7.28927847	8.32449078	5.03104247	5.51055559	4.34033317	4.94924069
ENSG00000101336	0	0	0	0	0	0.00138793	0	0	0	0	0	0	0	0.00400893	0	0	0	0
ENSG00000101337	6.21269842	6.50324993	5.74561763	7.5846661	5.62461404	6.5065583	9.03608563	9.60287014	7.98206791	8.77386652	8.7635524	7.30874351	5.73765602	6.49356459	6.0878025	5.21039726	5.24144884	5.47764206
ENSG00000101342	0	0	0	0	0	0	0.00549531	0.02010783	0.00676607	0.01859313	0.00862075	0.00577877	0	0	0	0	0	0
ENSG00000101343	2.53769572	2.76636066	2.47200017	2.2845055	1.99173565	2.31142367	3.44805705	3.71940634	3.51916283	2.91171466	3.2711434	3.41027479	2.2244259	2.03119572	2.25817732	2.4854205	2.48393194	1.60581124
ENSG00000101346	8.9446035	7.82551091	7.65737331	7.4524273	7.83313815	6.43486615	15.1809867	17.4464354	15.9933877	12.3016939	13.6652323	13.5173108	8.91214992	7.39041085	8.24696374	7.27495355	6.10879762	4.94512576
ENSG00000101347	1.37199042	1.49294095	1.29940992	1.66145	1.00432184	1.47694081	32.006642	31.7673317	10.9527638	4.83104653	21.6816115	6.5046729	8.6544902	11.3013311	5.72743365	3.72445229	4.36692954	4.36675536
ENSG00000101349	0.04175738	0.05283176	0.06352573	0.0449802	0.02588129	0.10441084	0.00558905	0.00227232	0.00344075	0	0	0	0.00196355	0	0	0.00223791	0	0
ENSG00000101350	6.86096891	8.05944323	6.95054648	8.1900732	11.1773296	7.47838869	6.91944999	7.64427444	6.81239272	10.2962239	16.207399	13.1985955	6.10570912	5.58456382	5.34280194	10.6973949	9.90186958	7.8406773
ENSG00000101353	0.1759754	0.19024811	0.14945594	0.20552	0.1713544	0.18758395	0.06694172	0.07560057	0.03510555	0.09786793	0.0914017	0.07951997	0.10060477	0.09866511	0.0778651	0.08190146	0.09753243	0.08006641
ENSG00000101361	5.37287005	4.36670158	4.46812235	4.1530165	5.85858715	4.45958867	9.18081251	11.0805742	12.834934	7.11063502	9.65970496	13.3768354	8.58877569	4.02157055	4.63210555	8.69560049	5.48040076	3.42208738
ENSG00000101363	2.53984362	2.97581491	2.85767765	3.329357	2.46492871	2.83391015	3.59981931	4.09043749	4.30236593	3.91932327	5.03033059	4.66296357	4.50166858	4.80005191	4.16504481	3.24507574	2.9943941	3.40941173
ENSG00000101365	4.96353711	5.05380083	4.28072729	4.802195	5.56957145	4.3921919	7.18321885	6.98776186	7.89531787	6.73516858	7.32710615	7.80136742	4.62591137	4.0247089	4.69271407	5.05357265	4.37012806	3.27802767
ENSG00000101367	60.4783756	46.7314565	52.7898698	37.356451	61.4954323	40.7946755	51.834859	57.7466406	58.0625925	35.5124434	49.9226376	53.8133815	46.8422396	36.7660229	43.2366601	51.2300879	43.4574053	25.9525154
ENSG00000101384	27.3018473	15.3209382	16.6188105	17.532123	31.3077519	19.9588066	4.90832074	4.94353327	6.97045578	9.0434327	4.95411524	5.19222623	7.93053143	3.81583014	4.90418778	3.00224693	3.16435155	2.89631891
ENSG00000101391	0.66189431	0.76477409	0.66644613	0.7389063	0.75186361	0.70074682	1.18270021	1.39833154	1.35133114	0.97281313	1.17689268	1.23354347	1.29363107	0.98921144	1.04924785	1.10143678	0.94594286	0.72763762
ENSG00000101400	8.51844958	5.77031686	7.2272832	10.637532	8.16015218	8.43384334	6.05628743	6.21410813	3.12286695	2.01920385	5.68660479	1.92920002	4.77567238	4.05511601	3.78202803	2.58872757	2.31867877	2.3405818
ENSG00000101405	0	0	0	0	0	0	0	0	0.03106063	0	0	0	0	0	0	0	0	0
ENSG00000101407	3.1729278	2.97170088	2.6234126	2.4582402	2.87962045	2.43452634	3.88846252	4.52215328	4.61445756	4.07725	4.00678262	4.33205597	3.53067137	2.55090938	2.9139981	3.19120145	2.90986664	1.5273067
ENSG00000101412	35.4102215	17.577437	21.5339122	18.125651	33.7445816	17.9559714	5.9252487	11.3260965	13.4322663	5.18980694	7.17013799	13.2012148	11.269308	6.94687945	9.08206395	9.72611816	7.94204715	4.11601195
ENSG00000101413	4.11147357	3.79956697	3.51694934	4.3712618	3.3699074	3.78954369	4.78205171	5.36103085	5.58846719	4.06114821	4.35040127	4.50456475	5.09740844	4.95536201	5.08716104	4.13316645	3.88198731	3.33502799
ENSG00000101417	2.44771807	2.45636325	2.0701043	2.9500281	1.87558808	2.50911094	1.1262556	1.16891684	1.45560207	0.92307328	1.05350258	1.37561196	1.45982577	1.5098699	1.74241124	1.26886042	0.94349635	1.11377058
ENSG00000101421	33.2479333	37.4445401	35.9436453	29.638325	38.1304475	24.7785344	22.0775099	27.3114608	27.6765538	23.161431	31.599877	29.3676497	30.4947833	33.388443	30.5844766	32.9801812	31.1943243	31.082623
ENSG00000101425	0	0	0	0	0	0	0.00621438	0.01136949	0.00191286	0.03153912	0.0146232	0	0	0.00419157	0.00336496	0	0	0.00933419
ENSG00000101435	0	0	0	0	0	0	0	0	0	0	0	0	0	0	0	0	0	0
ENSG00000101438	0	0.01677389	0	0	0	0	0	0	0	0	0	0	0	0	0	0	0	0
ENSG00000101439	43.2726664	30.2175057	31.3708009	22.474853	37.655359	23.3810554	33.3412525	30.9628242	26.0732284	21.9087685	31.1259763	22.3402402	10.5959603	12.2572629	9.89904891	8.99192601	9.64600878	9.6476091
ENSG00000101440	0.24253149	0.05415048	0.10541842	0.0696667	0.12025709	0.08085729	0.02164121	0.02639572	0.01332283	0	0.06789924	0.02275752	0	0	0	0	0	0
ENSG00000101441	0	0	0	0	0	0	0	0	0	0	0	0	0.14529732	0	0	0	0.06456089	0
ENSG00000101442	2.58010886	2.67436198	2.34004369	2.4035021	2.70962996	2.81120109	2.48873968	2.4989999	3.33504533	2.98961817	2.30620556	2.88491225	3.61124931	2.9200553	2.84563117	3.33716801	2.58038564	1.4952653
ENSG00000101443	0	0	0	0	0.00897575	0	0.01292206	0.0078805	0.00397756	0.00728687	0	0.00679431	0	0.01743172	0.01399406	0	0	0.00970467
ENSG00000101444	15.4224271	11.5058052	12.35318	16.824426	15.1425568	13.4381188	17.4916091	20.4905699	24.0446056	16.3352094	18.7836612	20.8050917	10.4592285	8.99023456	9.69736593	9.64025498	8.01213996	7.57526208
ENSG00000101445	0.65337294	0.17371952	0.21462159	0.1289406	0.33803464	0.1253338	0.0020027	0	0.00369872	0.01581077	0.00314173	0.002106	0	0.00270162	0	0.0024057	0	0
ENSG00000101446	0	0	0	0	0	0	0	0	0	0	0	0	0	0	0	0	0	0
ENSG00000101447	18.9079371	12.6115103	14.4169775	16.139449	17.5195371	14.2032203	6.59592859	10.0465454	17.0689759	7.78752641	7.38857776	18.4889603	19.2495625	14.2739116	18.3943884	19.9424525	16.5276945	10.8035842
ENSG00000101448	0.00689406	0.02539764	0.01685567	0.0133671	0.00769131	0.03619985	0.02214579	0.01688197	0.01022509	0.01248822	0.01302794	0.02328812	0.01458801	0.01120291	0.01199148	0.01330108	0.02074234	0.00831591
ENSG00000101452	1.02109001	1.13903197	0.93750741	1.092748	0.90175334	1.05445585	0.88899928	0.95044436	0.98936625	0.80104291	0.90268806	0.95958751	1.24433717	1.0681543	1.23272644	1.1112101	0.96042343	0.62643899
ENSG00000101457	3.76754316	3.7808973	3.6067461	3.8239706	3.88132915	2.70671397	5.19362427	5.13918623	5.96176245	3.46041054	5.0138397	5.85923405	7.43324839	6.16540568	5.82638294	4.83101829	4.79962859	3.6164556
ENSG00000101460	0	0	0	0	0	0.00591977	1.75552431	1.83973807	1.34604956	1.74403877	1.71004999	0.84639687	0.94182657	1.02592776	0.94028473	0.70800422	0.74793571	0.73298754
ENSG00000101463	0	0	0	0	0	0	0.22897421	0.24705464	1.18191051	13.8655747	0.55261987	1.34284064	0.12994693	0.11880143	0.21935766	0.24331388	0.3464409	0.0793676
ENSG00000101464	4.30919037	3.96796448	3.93886564	4.2413095	4.72896208	4.10568017	2.90268385	3.36567404	3.76421775	3.31196471	3.05149032	3.55982459	2.85668695	2.35958405	2.36782033	3.10641121	2.83877981	1.82323931
ENSG00000101470	0.0216307	0.08693151	0.02115443	0	0.02413213	0.03245145	0.13896855	0.06356232	0.05347015	0.05877424	0.08175251	0.14613686	0.12815907	0.02343338	0.09406072	0.08346653	0.13016169	0.10436751
ENSG00000101473	0.64290295	0.75217999	0.7425012	1.0087139	0.63467179	0.81698226	1.30263639	1.25117121	1.12709749	1.21246939	1.33747245	1.11443741	1.20645878	1.25779148	1.32793489	0.8854079	0.81763149	0.67856003
ENSG00000101474	13.0721329	12.1488415	12.6632476	14.50348	11.6256311	11.7297355	22.4909573	23.6927279	22.9011456	21.4774446	24.2100377	22.6893251	10.339331	9.37666556	10.0596557	9.16163446	7.72456334	7.26291407
ENSG00000101489	0	0.00110764	0.00242587	0.0021375	0.00046122	0	0.00163511	0.00973539	0.00208331	0.00069826	0	0	0	0.00071909	0	0	0	0
ENSG00000101493	2.40920242	2.54124487	2.35462473	2.9056188	2.1879952	2.76955569	1.16614539	1.29192416	1.30802936	1.62307363	1.28471631	1.1178227	1.35370321	1.91930516	1.79147379	1.22854008	1.32435961	1.43414656
ENSG00000101542	7.20498051	8.22699168	8.2212596	3.0472211	4.07147518	3.00230515	0.00260051	0.00317184	0.0048028	0.00293291	0.00407955	0	0.00274084	0.0280645	0.01408123	0	0.00487142	0.0078121
ENSG00000101544	10.5698142	9.02266594	9.52775055	10.199379	10.6860509	9.28393175	4.88244727	5.21705924	6.35379291	2.48609485	4.32514789	4.69836422	6.29368552	5.37916343	6.13904304	7.06638008	5.42970472	4.74409836
ENSG00000101546	3.01421895	3.13759105	2.86320084	2.4219808	3.10433325	2.49020012	1.34116727	1.57597072	1.76458261	5.02199425	1.23158279	1.66403829	2.21942809	1.57755167	1.98823256	2.29869193	1.94553685	1.13621265
ENSG00000101557	1.38175122	1.36724378	1.35516688	1.4385006	1.89298545	1.38247204	1.23732238	1.5110809	1.70587403	1.8718921	1.86439461	1.87630263	2.61316409	2.34954597	2.45803149	2.98208203	2.60422461	2.27517805
ENSG00000101558	10.9617081	10.1944032	10.5014912	10.217591	10.7201852	10.3225282	4.1993509	4.49759586	4.7258996	4.54427272	4.55376163	4.81925489	9.07176855	8.55035207	8.91993003	11.459248	10.7130414	7.07641486

[illegible]

[illegible]																	
ENSG00000102753	16.1084979	14.8230706	15.5731013	13.944557	16.6170786	15.1902678	8.34106663	9.4058749	11.0984289	7.7104491	10.4680276	11.7040589	14.9341906	13.7647269	14.0039634	18.4900308	16.4780762
ENSG00000102755	0.00425054	0.01565894	0.01247086	0.0082415	0.00829865	0.00956531	0.01194726	0.00416344	0.00420286	0.00384981	0.00267746	0.00717916	0.01618966	0.02762868	0.00924171	0.0041004	0.00959154
ENSG00000102760	0.75015188	0.93314672	0.5502263	1.3505963	0.85184605	0.80386798	0.21515289	0.44611627	0.34437801	0.26691876	0.1350083	0.29412625	1.51931118	4.17944038	1.42130983	0.2584479	0.44333959
ENSG00000102763	2.64474845	3.49637059	2.77710191	4.39386	3.19906505	3.77357063	1.60077318	1.97972724	1.73696477	2.25420561	2.49016663	1.94195908	2.55664992	2.85611188	2.64151614	2.31499395	2.47514646
ENSG00000102780	0.4171646	0.4071599	0.39554583	0.2670492	0.5044121	0.35643709	0.46327704	0.5160237	0.54290904	0.56999175	0.46448899	0.46435861	0.60126569	0.70759448	0.67862114	0.7289714	0.71961017
ENSG00000102781	6.43649555	4.68719179	5.9452277	4.0740509	6.55798473	4.3340574	1.80523154	1.95879454	2.14090355	1.5811569	1.87184345	1.94824037	7.3080552	6.30729782	7.49884005	8.39758114	7.85202091
ENSG00000102786	1.49466001	1.41525513	1.4347281	1.3739571	1.65064826	1.42171792	1.12612703	1.23651871	1.11930623	1.15353987	1.28035707	1.27171879	2.37013485	2.53032955	2.42858392	2.21402313	2.07574871
ENSG00000102794	0	0	0	0	0	0	0.00716779	0.00874253	0.00441266	0	0.0562223	0.00753752	0.13598236	0.08702345	0.07762419	0.12915238	0.21483347
ENSG00000102796	0.80627958	0.60325722	0.65011531	0.6319787	0.60765436	0.55012007	0.20664984	0.15123012	0.09541381	0.18645082	0.23773317	0.10141101	0.22506101	0.38562961	0.35060929	0.26064548	0.36775224
ENSG00000102802	0.0114128	0	0.03348453	0	0	0	10.017706	7.65756426	3.16538002	0.99233562	12.925909	3.83596327	0.03863962	0.08654752	0.02977701	0.01100966	0.05150697
ENSG00000102804	19.3040213	19.0821733	18.7091266	23.109386	14.1145311	20.1306556	7.78340408	7.92198157	6.75982082	7.75368777	7.12573703	6.21248755	13.3196579	12.3887379	16.4765991	11.6821033	13.0641616
ENSG00000102805	5.64983486	5.47734716	5.5056089	4.8832738	5.54922548	4.89711348	3.97822611	4.32927523	3.27101892	3.59916739	4.37624765	2.99061168	5.64010452	5.36658388	5.4308674	5.08517507	4.90037335
ENSG00000102837	0	0.00745184	0	0.0287613	0.00620588	0.0083453	0	0	0	0	0.01401579	0	0	0	0	0	0
ENSG00000102854	0	0	0	0.0032791	0.00141507	0	0.09167494	0.12423962	0.03637065	0.01608331	0.06072185	0.01499614	0.0042943	0.00824457	0	0.00244717	0
ENSG00000102858	1.43432187	1.514972	1.26488726	1.3755695	1.39431458	1.35277027	1.40073345	1.43865645	1.22725541	0.86541552	1.04922041	0.94412888	1.02914289	0.95469543	0.95826181	1.03177425	0.88867195
ENSG00000102870	11.1179083	11.9960902	10.0486867	10.35521	11.9481621	9.12329324	8.33668429	9.05169715	7.78750244	5.48865747	7.26044926	6.99814433	5.97287563	6.05956692	6.04959418	6.14767575	5.17633062
ENSG00000102871	0.91076622	0.55666666	0.76823997	0.7357948	0.67950991	0.61059963	0.8091261	0.61332066	0.59942855	0.60320931	0.73864107	0.38457069	1.44540299	1.49850321	1.21783876	0.92252479	1.07040863
ENSG00000102878	0.16241577	0.2036894	0.13630925	0.1846293	0.11694336	0.30414825	0.19056025	0.17601175	0.10478468	0.24204288	0.14511703	0.09922212	0.28566529	0.22087513	0.2594637	0.09556291	0.10223833
ENSG00000102879	0.29560993	0.16371298	0.24710885	0.1238123	0.33716475	0.15237182	0.04641839	0.07926292	0.08899454	0.06132606	0.03744965	0.0725218	0.09085736	0.02504716	0.03447029	0.07487652	0.04223452
ENSG00000102882	2.44345228	2.22230968	2.16151942	2.2690494	2.11566067	2.1828563	1.60513018	1.72435209	1.28020436	1.20230378	1.72309567	1.07426383	1.71305204	2.13985044	1.9805695	1.19799785	1.40873512
ENSG00000102886	0.26114008	0.30469197	0.14005275	0.3593317	0.10337835	0.53079239	0.50061005	0.36305505	0.31651647	0.51881914	0.28653976	0.19207678	0.47058332	0.24639964	0.64470681	0.22753683	0.22810615
ENSG00000102890	0.15464973	0.17017838	0.11163296	0.2427392	0.13967024	0.19610641	0.35484372	0.44362188	0.39138954	0.44021926	0.26441374	0.35759956	0.33347587	0.18349413	0.23377092	0.20246808	0.16063883
ENSG00000102891	0	0	0	0	0	0	0	0	0	0	0	0	0	0	0	0	0
ENSG00000102893	2.42493057	2.29874192	1.97799184	1.9425802	2.25162624	1.84117494	0.81173603	0.80980419	0.78069998	1.33955327	0.98629175	0.90917221	0.45278973	0.49585952	0.5481787	0.48938022	0.61540766
ENSG00000102897	1.79039653	1.60885872	1.81714624	1.9610972	1.78550465	1.73148197	1.34795848	1.26175188	1.47954956	2.01050456	1.25237228	1.43726814	1.04845188	1.15587033	1.08408679	1.39327101	1.36236252
ENSG00000102898	5.92360035	5.90790499	6.26307305	7.3774622	10.0814012	6.89170842	4.23894036	5.16013793	5.72650473	6.23238937	7.2205574	8.48623966	5.58024265	3.79236038	4.2473457	8.8363999	7.21779902
ENSG00000102900	3.42243641	2.90419085	2.72647039	3.2054352	3.28895815	3.09064187	1.69707664	1.92659399	2.38497579	1.61964112	1.49996153	2.16573259	4.17960857	3.21041274	3.72261078	5.63134808	4.52297097
ENSG00000102901	0.56165656	0.78762989	0.62437871	0.7723935	0.51534549	0.80130769	0.48791387	0.53216307	0.54380359	0.46108485	0.50256584	0.56030199	0.92535384	0.49816343	0.69677853	0.67223958	0.5084678
ENSG00000102904	0.03311275	0.05267618	0.02563707	0.021401	0.02154949	0.02897848	0.08310004	0.10541115	0.05184041	0.06623005	0.12514853	0.07223953	0.0922554	0.05978723	0.08039453	0.05456946	0.04566246
ENSG00000102908	0.20501949	0.32696501	0.28974129	0.233948	0.29261326	0.29518709	0.30878662	0.33138685	0.28087451	0.31016486	0.43426465	0.34056973	0.40840062	0.46861853	0.4369446	0.39048387	0.49313906
[illegible]																	

ENSG00000103942	0.65849639	0.46067449	0.47047605	0.4539645	0.58975797	0.46376841	1.11787068	1.50142238	1.91263525	2.63913687	1.99562108	1.77179542	0.86545295	0.58258956	0.62041638	0.87874338	0.7567218	0.43686866	3.70596628	3.23531053	2.7069138	2.66206268	4.07829357	2.58830051	[illegible]
ENSG00000103966	7.71569103	3.03036389	5.26919972	1.6117184	7.02227781	2.17951647	3.04072315	3.31659588	3.65621976	3.16518669	3.60281679	3.50670119	9.18839903	11.4630207	10.0530292	12.1551187	11.2974456	7.99615341	9.77532295	7.3841928	12.4507657	10.9032221	16.2866316	13.3949222	[illegible]
ENSG00000103978	4.10030848	3.43697178	3.47065542	3.4712682	3.30484198	2.85775224	3.75450147	3.63813487	3.37474999	3.05668087	3.63707366	3.58631152	3.68419657	3.29072936	3.32988807	3.8961301	3.47827432	2.86062733	3.88916576	3.69161575	6.19610562	4.02918539	5.3891436	5.94397638	[illegible]
ENSG00000103994	3.47319114	4.15296068	3.39131264	3.6606734	2.91846365	3.31850942	4.33995679	4.68251285	4.74689135	6.60356753	5.35286053	4.89369887	3.40916678	4.03411351	3.67282043	4.05793486	3.9485027	3.5305255	3.24439776	3.22544658	3.60848619	3.89956733	5.53693252	4.29458116	[illegible]
ENSG00000103995	1.62145559	1.80491125	1.64668641	1.3670799	1.80375174	1.75024847	0.66671089	0.90167449	1.43000357	1.17797493	0.88891826	1.69553402	1.83911674	1.46804055	1.91680759	2.01044535	2.21431189	1.407249	1.68844664	1.04695002	3.03011377	3.30182383	4.21440011	3.96639088	[illegible]
ENSG00000104043	0	0.00216134	0	0	0.00179996	0.00060512	0.003887	0.00711145	0.00239293	0.00146128	0.00304886	0.002725	0.26492314	0.22372362	0.1445248	0.18676714	0.29246716	0.72785322	0.00177556	0.02435354	0.00543253	0	0.01144045	0.00524994	[illegible]
ENSG00000104044	0	0.00213383	0.00311556	0.0041179	0.00177705	0.00716902	0.03070028	0.03744504	0.03464963	0.05770724	0.01204025	0.03497419	0	0	0.00831178	0.00307317	0	0	0.08764797	0.1782056	0.10190449	0.06802097	0.17426347	0.15697485	[illegible]
ENSG00000104047	0.70114626	0.73910381	0.67221911	0.6269906	0.60013606	0.73460173	0.99506984	1.07407631	0.99218788	1.51577733	1.03102235	1.03474957	3.02176719	3.93985043	3.75867163	4.65925701	5.0387289	4.78612878	1.03726428	1.54926712	1.53844004	0.99936551	1.8839304	1.80171258	[illegible]
ENSG00000104055	0	0	0	0.0122065	0.00263382	0	0.02275086	0.02312429	0.01400595	0	0.01189679	0.0159496	0	0	0	0	0	0	0	0.00419243	0.01192385	0	0	0.0087795	[illegible]
ENSG00000104059	0	0	0.00440102	0.0058169	0.00502051	0	0	0	0.00444962	0	0.01133866	0	0.12569476	0.15112929	0.17220387	0.41674994	0.10154684	0.11399258	0.22781226	0.27171095	0.075763	0.01153032	0.01823434	0.02091903	[illegible]
ENSG00000104064	3.38009475	3.34604696	3.52577581	2.9390602	3.3427241	2.852627	1.36990315	1.56991428	1.81459336	1.36021153	1.46007072	1.88286041	2.06159009	1.40592655	1.79086744	2.72502719	2.02509605	1.38528918	0.99998509	1.01038294	2.10522982	2.23296501	3.03836588	3.88757273	[illegible]
ENSG00000104067	5.35611916	7.48677448	5.85157691	4.9065093	5.49190983	4.85512087	4.10959369	4.15387825	4.42837936	5.14387846	4.49810288	4.73785953	3.09104091	2.88410446	2.82411098	3.80603844	3.49569169	2.79600862	6.45176037	4.31558228	5.10936359	6.2724933	5.71036912	5.51729884	[illegible]
ENSG00000104081	0.34986231	0.36098339	0.50632627	0.2649474	0.0246415	0.20014395	0.5605122	0.69230892	0.9155122	1.57958994	0.42518176	0.29844288	1.79620431	1.78215764	1.57515604	0.4500054	0.73365808	1.0571788	0.63977114	1.43715358	0.59199726	0.07243876	0.11455643	0.0607831	[illegible]
ENSG00000104093	1.21910994	1.777472	1.39622787	1.4747502	1.11416256	1.62364168	1.17669104	1.15882977	1.09209368	1.97640473	1.57063613	1.27105782	1.51600051	2.04502333	1.82444003	1.46451144	1.77169769	1.45019569	1.21001758	1.23497672	1.06818975	1.2343971	1.96738105	1.26529926	[illegible]
ENSG00000104112	0.4301347	0.68807695	0.65326646	0.0523293	0.03387364	0.02657157	0.02031943	0.01487013	0.03002187	0.00916665	0.03825129	0.00854702	0.01284954	0.01644642	0.01320306	0.01952663	0.00761269	0	0.02784533	0.01797303	0.05963734	0.0302539	0.05126171	0.03293311	[illegible]
ENSG00000104129	1.28122421	1.21806802	1.13910388	1.2141729	1.35184082	0.99771749	0.78753031	0.93110627	0.880481	1.22168839	0.99876314	0.98045688	1.19574717	1.13156635	1.18616508	1.1236066	1.05132346	0.84298239	1.81936658	1.08758723	1.89453328	1.98636565	1.31309879	1.95617603	[illegible]
ENSG00000104131	7.91204088	6.88397006	7.87365982	6.7153058	9.24342376	6.42987484	8.4800805	9.97752709	11.2671678	8.8210432	10.0716535	12.5497441	7.14132627	5.62338094	5.44673112	7.749793	6.54217611	5.30329887	6.75379763	4.34311683	10.5439379	8.36625705	7.99287977	12.0769626	[illegible]
ENSG00000104133	1.96094352	2.43943867	1.93297695	1.920707	1.72240923	1.77426355	1.91483335	1.81897477	1.67089155	2.82545113	2.32527754	1.75511356	1.30974018	1.61433516	1.6322659	1.70550953	1.86632986	1.29183208	1.72317403	1.66019862	1.62511833	1.80339825	2.08309894	1.6482964	[illegible]
ENSG00000104140	0	0.03798473	0	0	0.04217815	0.02835933	0.34915339	0.37031422	0.56073098	0.5307492	0.26195965	0.51083619	0.0959985	0.12287064	0.06575975	0.01823536	0.05687417	0.57004249	0.97774826	1.07420738	0.60467313	0.45205184	0.80424664	0.66782883	[illegible]
ENSG00000104142	6.78608306	6.19291101	5.85110264	6.179647	6.94972505	5.33142499	7.63852298	7.91673934	7.30153406	7.39564077	7.09065059	6.58695958	7.46929511	6.69148363	6.08972818	6.66394441	5.04186383	4.65079423	4.92596388	4.67777514	7.12076706	4.39538161	3.13130108	5.73228	[illegible]
ENSG00000104147	5.78674584	4.25237002	4.01098393	4.7446238	4.52333664	4.29863846	2.12056238	2.69650503	4.63857457	2.23895365	2.09978694	4.42826112	3.7566872	2.49543068	3.06197422	3.63123049	3.77514278	2.12343311	2.8441454	1.01433522	4.41406394	7.29354324	7.32269222	10.5510991	[illegible]
ENSG00000104154	1.67145027	2.41266017	1.81445966	2.7060822	1.48246916	2.25369906	1.77183551	1.98510361	1.76839029	3.22832866	2.02150112	1.44682694	2.43333928	2.29059577	2.08962841	2.27349708	2.38874461	1.67342539	1.51755412	1.94419802	1.79745914	1.43825637	2.94611879	1.96965688	[illegible]
ENSG00000104164	4.23948086	4.64432252	4.48770083	3.8088918	4.21813393	4.17414019	3.32448391	3.48278386	3.6521553	3.88631276	4.15410534	4.11436183	3.65665261	3.59602091	3.7049293	4.95608879	4.89201239	3.32426417	2.87624474	2.84051687	3.94058301	3.86650073	6.24192519	5.71238208	[illegible]
ENSG00000104177	4.49633497	5.35386457	4.99252175	4.0092539	4.82646048	4.10964562	0.76848044	0.86149157	0.89340258	1.21000445	1.11475767	1.22739624	1.06976501	1.12776009	1.31741093	1.33125121	1.53793887	1.65495262	0.90770119	0.32233116	0.42242553	1.22194096	3.03515965	1.77436448	[illegible]
ENSG00000104205	0.03495791	0.03902561	0.04700879	0.0131796	0.01706273	0.0196671	0.07837275	0.10129793	0.07633277	0.03561995	0.06239108	0.06765443	0.1319164	0.08994429	0.09247511	0.10116926	0.12928226	0.06676559	0.05770767	0.0646666	0.08092503	0.06220167	0.17853657	0.13812859	[illegible]
ENSG00000104213	0.07047714	0.09441342	0.14845467	0.1681845	0.0453619	0.13013331	1.62829299	1.97540365	0.94076888	2.04264791	2.44510358	1.10794848	0.27531949	0.47572331	0.37247728	0.09936659	0.09786758	0.15040684	2.00466126	3.68730831	0.75299752	1.48167417	1.57064675	0.69555772	[illegible]
ENSG00000104218	1.27440907	1.43993272	1.31220011	1.0699105	1.0471007	1.1013319	1.50087806	1.68101425	1.58407336	1.0769138	1.3365615	1.47705274	1.0050003	1.04080045	0.99551467	1.04878617	1.10682205	0.9924752	1.2362321	1.00033852	1.66862622	1.18242199	1.44402398	1.33446662	[illegible]
ENSG00000104219	9.74112705	11.4872145	10.9004361	8.5301207	7.55265145	7.62875621	10.8240746	10.9774533	8.95684951	11.9816723	10.8827961	9.2167914	3.36688817	3.11468325	3.27683785	4.30169943	3.49567641	2.50205696	3.03404053	3.34162908	4.00053923	5.91647118	9.27370497	8.26717063	[illegible]
ENSG00000104221	2.4483364	2.27171258	2.34209178	3.5019893	1.78366375	2.64946075	2.30464113	2.53576463	2.58292577	2.30233673	2.56196317	2.5139074	2.25914268	1.95667294	2.05367852	2.1101804	1.60007295	1.791339	2.34842917	2.58971656	3.32235989	2.782382	3.19844988	2.92927443	[illegible]
ENSG00000104228	3.54298558	2.80251126	2.82835369	2.7876572	3.40941645	2.41539646	4.58916536	5.02305026	4.28201447	4.13609598	4.30702694	3.68865461	3.36473296	2.37401203	2.53247532	3.26444079	2.50424789	2.2117754	3.35225548	3.25620665	6.17824335	3.00052369	3.20646744	5.48839608	[illegible]
ENSG00000104231	0.64863641	0.69287539	0.62053439	0.9206381	0.5786018	0.89785377	1.53660879	1.5918218	1.44550639	1.12505241	1.57736301	1.39509059	1.13151557	1.23851602	1.28063129	1.33460626	1.2415212	1.17788119	1.41833969	1.38275734	1.66303147	1.72472023	2.54094077	2.31101958	[illegible]
ENSG00000104237	0.0045394	0	0	0	0	0	0.0656186	0.04446378	0.04712904	0.01233431	0.02287535	0.02300112	0.24590037	0.22621466	0.41847746	0.49921213	0.76483742	0.22997601	0.01498706	0.00806128	0.07260336	0.00775401	0.13795176	0.16037298	[illegible]
ENSG00000104267	5.55563147	2.86322932	3.93828298	5.0040413	3.86492927	3.59406565	0.02841776	0.04159326	0.03149033	0.14102032	0.12482486	0.11355771	0	0	0.00615505	0.00682725	0	0	0.36606517	0.15081716	1.2093847	0.43520518	0.63805972	1.25674246	[illegible]
ENSG00000104290	8.70926007	12.8192452	9.97636731	9.4522497	8.47895261	9.66943607	1.58798848	1.62389035	1.37297612	2.38629677	1.81718552	1.46790885	0.64443586	0.73977381	0.76855684	0.80317384	0.97508817	0.55941151	1.89282543	2.03785791	2.65589731	2.35627044	5.01233072	3.58174353	[illegible]
ENSG00000104299	0.66340492	0.56870654	0.54467001	0.5875657	0.57184383	0.52323512	1.19597797	1.2675314	1.14456155	1.26477506	0.8443708	0.97869951	0.43788734	0.36674024	0.50929271	0.46351833	0.44355637	0.24204413	0.66849661	0.7490428	1.19733257	1.00612283	0.86837959	1.38838173	[illegible]
ENSG00000104312	2.09473422	2.1904721	2.16098359	2.0221978	1.5727741	1.99894784	3.63773001	4.11227513	4.29541359	3.94659665	3.21801244	3.13867135	4.89633452	6.55418211	6.5184477	8.67554624	7.99776921	6.27425047	2.86945473	1.78148504	3.29601339	2.66850358	5.77494488	3.81694102	[illegible]
ENSG00000104313	3.13948484	3.88369122	3.26193712	2.9031282	2.61551257	3.12664916	0.57037089	0.55305508	0.54223709	0.25617141	0.46981939	0.46267134	0.13122426	0.10213595	0.07561666	0.05962186	0.10243247	0.07076108	0.89690826	1.01385042	1.10402939	0.95820605	0.66216128	0.80153157	[illegible]
ENSG00000104320	4.15706599	4.10983758	3.99231439	3.4655805	4.10381644	3.69330873	4.71561108	5.20509093	5.28169768	3.40994622	4.07384922	4.7661592	4.02705636	3.72910934	3.79057408	5.42377783	4.86461316	3.02328088	3.14280666	1.9848681	4.87842916	3.75143017	5.83629549	5.95476655	[illegible]
ENSG00000104321	0.73400409	0.27761134	0.43008686	0.0736127	0.92301183	0.20884627	0.02286699	0.05268262	0.08602884	0.01719321	0.05181591	0.07213956	1.49961329	1.91253399	1.26296388	0.56768219	1.25651326	1.21359119	0.07311838	0.01404612	0.22105139	0.36749141	0.46471431	1.1648093	[illegible]
ENSG00000104324	0.00902415	0.00906677	0	0.0058324	0.00755079	0.00676924	1.22113045	1.25958935	0.87667695	1.41808044	1.56321293	0.83067646	0.72944118	0.97762121	0.78875106	0.78348463	0.62447726	0.69121742	0.9782259	1.25399622	0.79003102	0.65126936	0.83186928	0.77606309	[illegible]
ENSG00000104325	8.96477317	6.1517589	8.27613022	6.2707488	10.2892588	6.60758567	1.97156266	2.00133853	1.52248789	1.7686051	2.19210054	1.58217469	1.61065153	2.27231763	1.83010301	2.39885698	2.65504626	1.9269229	1.47415558	1.157239	1.53710321	1.54987614	1.70990195	2.09734146	[illegible]
ENSG00000104327	0.60456964	0.18326219	0.6883627	0.0998237	0.82956563	0.16716706	0.0035439	0	0.00218171	0	0.01667847	0.00372671	0	0.00478069	0.00575685	0.0106426	0.00331931	0.01330763	0.01699772	0.00391833	0.02414592	0.00565347	0.00894053	0.01435962	[illegible]
ENSG00000104331	19.715871	19.7319977	20.570014	19.172991	19.6197553	17.6250411	20.2744109	23.1749867	22.4503261	18.2948031	23.3508358	22.2943977	21.5003934	22.1062663	25.7795518	26.940765	31.2955446	20.7030058	15.6760071	15.724084	28.6463753	28.3022693	42.297089	34.0877038	[illegible]
[illegible]	[illegible]																								

ENSG00000105146	0.0919099	0.06669294	0.0749052	0.0940534	0.0918575	0.19534106	0.03075439	[illegible]	[illegible]
ENSG00000105171	1.33045291	1.16560942	1.22572954	1.1807812	1.39288503	1.19507581	1.79817584	[illegible]	[illegible]
ENSG00000105173	1.03614472	0.50753614	0.64108691	0.2505564	0.91808957	0.37275696	0.52643171	[illegible]	[illegible]
ENSG00000105176	4.6954111	4.64234745	4.68250193	4.8052207	4.54752987	4.68911174	5.00494573	[illegible]	[illegible]
ENSG00000105185	5.53533579	3.75731176	4.88177998	4.4000473	6.57520682	4.87511951	8.36964537	[illegible]	[illegible]
ENSG00000105186	2.53645683	1.90421393	2.17588464	2.3233597	2.40746701	2.49953421	2.1858595	[illegible]	[illegible]
ENSG00000105193	19.7280583	23.3315723	23.0809302	35.684546	22.51644	22.5756896	25.04988	[illegible]	[illegible]
ENSG00000105197	1.74412446	1.56008795	1.55508322	1.9025356	2.01091161	1.66194029	1.50811737	[illegible]	[illegible]
ENSG00000105198	0	0	0	0	0	0	0	[illegible]	[illegible]
ENSG00000105202	10.7456586	8.70499126	9.76599622	12.023986	11.5099927	10.8695778	5.89251305	[illegible]	[illegible]
ENSG00000105204	0.92153717	1.46659734	1.00949054	1.1943046	0.67377188	1.19843843	0.25699326	[illegible]	[illegible]
ENSG00000105205	0	0	0	0	0	0	0	[illegible]	[illegible]
ENSG00000105219	0.01591854	0.04264996	0.0103787	0.0205766	0.0147995	0.04776357	0.14062158	[illegible]	[illegible]
ENSG00000105220	16.0878939	9.40073335	12.0958035	15.453834	21.4532517	18.0510322	7.03895705	[illegible]	[illegible]
ENSG00000105221	1.93035142	1.78300502	1.738933	1.6023347	2.00146474	1.66554277	2.99871593	[illegible]	[illegible]
ENSG00000105223	3.49906207	3.04488997	3.31189526	4.5606825	2.93591938	3.44750453	20.8355946	[illegible]	[illegible]
ENSG00000105227	0.66649388	0.42849132	0.59362092	0.8730594	0.52116217	0.75439433	0.227001	[illegible]	[illegible]
ENSG00000105229	5.20975414	5.16667811	4.57895296	4.3249919	4.65666833	4.01324541	4.03955266	[illegible]	[illegible]
ENSG00000105245	3.1881676	2.25043549	3.06360118	3.5524388	3.66472074	3.39600007	1.92947317	[illegible]	[illegible]
ENSG00000105246	0	0	0	0	0.02312339	0	0.24967421	[illegible]	[illegible]
ENSG00000105248	[illegible]	[illegible]	[illegible]	[illegible]	[illegible]	[illegible]	[illegible]	[illegible]	[illegible]
[illegible]	[illegible]	[illegible]	[illegible]	[illegible]	[illegible]	[illegible]	[illegible]	[illegible]	[illegible]

ENSG00000105948	1.26964561	1.25892122	1.22599507	0.8620727	1.19067095	0.94169171	0.64871917	[illegible]
ENSG00000105953	8.67301384	7.05803838	6.90209979	7.6637632	10.451686	6.66861354	5.28623469	[illegible]
ENSG00000105954	0	0	0.02648748	0	0	0.02031624	0	[illegible]
ENSG00000105963	0.01066739	0.02143556	0.01043252	0.0252796	0.0019835	0.01200281	0.01570564	[illegible]
ENSG00000105967	0.00126884	0	0.0012409	0.0016401	0.00212335	0.00095179	0.01528457	[illegible]
ENSG00000105968	58.1124561	53.2921904	53.7237619	45.20889	54.9315327	45.4693992	7.72605558	[illegible]
ENSG00000105971	3.36482442	2.71263389	3.62664361	2.7817279	3.9964105	2.71181297	1.8295211	[illegible]
ENSG00000105974	4.66945909	1.83811767	3.2116213	1.2009167	4.16845661	1.55696931	11.4007209	[illegible]
ENSG00000105976	0.01457288	0.01342157	0.01603351	0.0070639	0.00406453	0.00136643	4.91676947	[illegible]
ENSG00000105982	0.12444573	0.11875336	0.10670088	0.0793285	0.08082974	0.09334984	0.04243995	[illegible]
ENSG00000105983	3.54491814	3.75178793	3.70786078	3.4548748	3.83898371	3.3524611	1.24004465	[illegible]
ENSG00000105988	0	0	0	0	0	0	0	[illegible]
ENSG00000105989	0.03917489	0.02623996	0.01094638	0	0.04058337	0.01259402	0.00898868	[illegible]
ENSG00000105991	0.13509046	0.1381098	0.07648824	0.0643338	0.06742409	0.13866856	0	[illegible]
ENSG00000105993	4.1706113	3.95348453	4.13552192	3.5543825	4.37569084	3.66220932	3.11020189	[illegible]
ENSG00000105996	3.26041414	5.59030733	3.57004112	3.5086942	2.12330017	3.59251361	0	[illegible]
ENSG00000105997	1.03906618	1.05899571	0.94308101	0.850317	0.66509576	0.8971849	0	[illegible]
ENSG00000106003	9.07612281	13.1712206	7.89745392	7.4898664	4.61389067	7.84193507	0.18126611	[illegible]
ENSG00000106004	0	0.01087009	0	0	0	0	0	[illegible]
ENSG00000106006	0	0.03592012	0	0	0	0	0	[illegible]
ENSG00000106009	3.39714396	[illegible]	[illegible]	[illegible]	[illegible]	[illegible]	[illegible]	[illegible]
ENSG00000106012	1.74465102	[illegible]	[illegible]	[illegible]	[illegible]	[illegible]	[illegible]	[illegible]
ENSG00000106013	0.15213921	[illegible]	[illegible]	[illegible]	[illegible]	[illegible]	[illegible]	[illegible]
ENSG00000106018	0.06383707	[illegible]	[illegible]	[illegible]	[illegible]	[illegible]	[illegible]	[illegible]
ENSG00000106025	2.4475154	[illegible]	[illegible]	[illegible]	[illegible]	[illegible]	[illegible]	[illegible]
ENSG00000106028	6.99218149	[illegible]	[illegible]	[illegible]	[illegible]	[illegible]	[illegible]	[illegible]
ENSG00000106031	0.02457003	[illegible]	[illegible]	[illegible]	[illegible]	[illegible]	[illegible]	[illegible]
ENSG00000106034	0.40990259	[illegible]	[illegible]	[illegible]	[illegible]	[illegible]	[illegible]	[illegible]
ENSG00000106038	0.00409943	[illegible]	[illegible]	[illegible]	[illegible]	[illegible]	[illegible]	[illegible]
ENSG00000106049	14.0302766	[illegible]	[illegible]	[illegible]	[illegible]	[illegible]	[illegible]	[illegible]
ENSG00000106052	11.2724977	[illegible]	[illegible]	[illegible]	[illegible]	[illegible]	[illegible]	[illegible]
ENSG00000106066	1.23574301	[illegible]	[illegible]	[illegible]	[illegible]	[illegible]	[illegible]	[illegible]
ENSG00000106069	0.28800001	[illegible]	[illegible]	[illegible]	[illegible]	[illegible]	[illegible]	[illegible]
ENSG00000106070	13.7875971	[illegible]	[illegible]	[illegible]	[illegible]	[illegible]	[illegible]	[illegible]
ENSG00000106077	1.88044274	[illegible]	[illegible]	[illegible]	[illegible]	[illegible]	[illegible]	[illegible]
ENSG00000106078	0.12880956	[illegible]	[illegible]	[illegible]	[illegible]	[illegible]	[illegible]	[illegible]
ENSG00000106080	3.97982918	[illegible]	[illegible]	[illegible]	[illegible]	[illegible]	[illegible]	[illegible]
ENSG00000106086	3.12501073	[illegible]	[illegible]	[illegible]	[illegible]	[illegible]	[illegible]	[illegible]
ENSG00000106089	0.44654288	[illegible]	[illegible]	[illegible]	[illegible]	[illegible]	[illegible]	[illegible]
ENSG00000106100	2.33810938	[illegible]	[illegible]	[illegible]	[illegible]	[illegible]	[illegible]	[illegible]
ENSG00000106105	21.5055141	[illegible]	[illegible]	[illegible]	[illegible]	[illegible]	[illegible]	[illegible]
ENSG00000106113	0	[illegible]	[illegible]	[illegible]	[illegible]	[illegible]	[illegible]	[illegible]
ENSG00000106123	0.01745653	[illegible]	[illegible]	[illegible]	[illegible]	[illegible]	[illegible]	[illegible]
ENSG00000106125	0.03664969	[illegible]	[illegible]	[illegible]	[illegible]	[illegible]	[illegible]	[illegible]
ENSG00000106128	0.03659005	[illegible]	[illegible]	[illegible]	[illegible]	[illegible]	[illegible]	[illegible]
ENSG00000106133	0.29702954	[illegible]	[illegible]	[illegible]	[illegible]	[illegible]	[illegible]	[illegible]
ENSG00000106144	4.62203414	[illegible]	[illegible]	[illegible]	[illegible]	[illegible]	[illegible]	[illegible]
ENSG00000106153	222.920683	[illegible]	[illegible]	[illegible]	[illegible]	[illegible]	[illegible]	[illegible]
ENSG00000106178	0	[illegible]	[illegible]	[illegible]	[illegible]	[illegible]	[illegible]	[illegible]
ENSG00000106211	69.2532492	[illegible]	[illegible]	[illegible]	[illegible]	[illegible]	[illegible]	[illegible]
ENSG00000106236	0	[illegible]	[illegible]	[illegible]	[illegible]	[illegible]	[illegible]	[illegible]
ENSG00000106244	36.8624023	[illegible]	[illegible]	[illegible]	[illegible]	[illegible]	[illegible]	[illegible]
ENSG00000106245	24.4485884	[illegible]	[illegible]	[illegible]	[illegible]	[illegible]	[illegible]	[illegible]
ENSG00000106246	0.91565264	[illegible]	[illegible]	[illegible]	[illegible]	[illegible]	[illegible]	[illegible]
ENSG00000106258	0.12937843	[illegible]	[illegible]	[illegible]	[illegible]	[illegible]	[illegible]	[illegible]
ENSG00000106261	9.89090532	[illegible]	[illegible]	[illegible]	[illegible]	[illegible]	[illegible]	[illegible]
ENSG00000106263	11.59639	[illegible]	[illegible]	[illegible]	[illegible]	[illegible]	[illegible]	[illegible]
ENSG00000106266	3.50109174	[illegible]	[illegible]	[illegible]	[illegible]	[illegible]	[illegible]	[illegible]
ENSG00000106268	9.84249393	[illegible]	[illegible]	[illegible]	[illegible]	[illegible]	[illegible]	[illegible]
ENSG00000106278	291.697293	[illegible]	[illegible]	[illegible]	[illegible]	[illegible]	[illegible]	[illegible]
ENSG00000106290	2.95380551	[illegible]	[illegible]	[illegible]	[illegible]	[illegible]	[illegible]	[illegible]
ENSG00000106299	43.3411878	[illegible]	[illegible]	[illegible]	[illegible]	[illegible]	[illegible]	[illegible]
ENSG00000106302	0.00493187	[illegible]	[illegible]	[illegible]	[illegible]	[illegible]	[illegible]	[illegible]
ENSG00000106304	0	[illegible]	[illegible]	[illegible]	[illegible]	[illegible]	[illegible]	[illegible]
ENSG00000106305	9.74444135	[illegible]	[illegible]	[illegible]	[illegible]	[illegible]	[illegible]	[illegible]
ENSG00000106327	0.10115408	[illegible]	[illegible]	[illegible]	[illegible]	[illegible]	[illegible]	[illegible]
ENSG00000106328	0.02776033	[illegible]	[illegible]	[illegible]	[illegible]	[illegible]	[illegible]	[illegible]
ENSG00000106330	4.04779083	[illegible]	[illegible]	[illegible]	[illegible]	[illegible]	[illegible]	[illegible]
ENSG00000106331	0	[illegible]	[illegible]	[illegible]	[illegible]	[illegible]	[illegible]	[illegible]
ENSG00000106333	1.11107448	[illegible]	[illegible]	[illegible]	[illegible]	[illegible]	[illegible]	[illegible]
ENSG00000106336	0.0668141	[illegible]	[illegible]	[illegible]	[illegible]	[illegible]	[illegible]	[illegible]
ENSG00000106341	0	[illegible]	[illegible]	[illegible]	[illegible]	[illegible]	[illegible]	[illegible]
ENSG00000106344	3.48925129	[illegible]	[illegible]	[illegible]	[illegible]	[illegible]	[illegible]	[illegible]
ENSG00000106346	2.64037851	[illegible]	[illegible]	[illegible]	[illegible]	[illegible]	[illegible]	[illegible]
ENSG00000106348	2.03653237	[illegible]	[illegible]	[illegible]	[illegible]	[illegible]	[illegible]	[illegible]
ENSG00000106351	2.13505841	[illegible]	[illegible]	[illegible]	[illegible]	[illegible]	[illegible]	[illegible]
ENSG00000106355	9.59964274	[illegible]	[illegible]	[illegible]	[illegible]	[illegible]	[illegible]	[illegible]
ENSG00000106366	2.58347479	[illegible]	[illegible]	[illegible]	[illegible]	[illegible]	[illegible]	[illegible]
ENSG00000106367	29.56033	[illegible]	[illegible]	[illegible]	[illegible]	[illegible]	[illegible]	[illegible]
ENSG00000106384	0.03761641	[illegible]	[illegible]	[illegible]	[illegible]	[illegible]	[illegible]	[illegible]
ENSG00000106392	2.49858316	[illegible]	[illegible]	[illegible]	[illegible]	[illegible]	[illegible]	[illegible]
ENSG00000106397	8.63897258	[illegible]	[illegible]	[illegible]	[illegible]	[illegible]	[illegible]	[illegible]
ENSG00000106399	7.01514049	[illegible]	[illegible]	[illegible]	[illegible]	[illegible]	[illegible]	[illegible]
ENSG00000106400	9.6368859	[illegible]	[illegible]	[illegible]	[illegible]	[illegible]	[illegible]	[illegible]
ENSG00000106404	0.88930367	[illegible]	[illegible]	[illegible]	[illegible]	[illegible]	[illegible]	[illegible]
ENSG00000106410	0	[illegible]	[illegible]	[illegible]	[illegible]	[illegible]	[illegible]	[illegible]
ENSG00000106415	9.4728947	[illegible]	[illegible]	[illegible]	[illegible]	[illegible]	[illegible]	[illegible]
ENSG00000106436	0.03194226	[illegible]	[illegible]	[illegible]	[illegible]	[illegible]	[illegible]	[illegible]
ENSG00000106443	5.836724	[illegible]	[illegible]	[illegible]	[illegible]	[illegible]	[illegible]	[illegible]
ENSG00000106459	1.77166491	[illegible]	[illegible]	[illegible]	[illegible]	[illegible]	[illegible]	[illegible]
ENSG00000106460	13.6159169	[illegible]	[illegible]	[illegible]	[illegible]	[illegible]	[illegible]	[illegible]
ENSG00000106462	5.45043102	[illegible]	[illegible]	[illegible]	[illegible]	[illegible]	[illegible]	[illegible]
ENSG00000106477	0.34155978	[illegible]	[illegible]	[illegible]	[illegible]	[illegible]	[illegible]	[illegible]
ENSG00000106479	4.1858475	[illegible]	[illegible]	[illegible]	[illegible]	[illegible]	[illegible]	[illegible]
ENSG00000106483	0.65448479	[illegible]	[illegible]	[illegible]	[illegible]	[illegible]	[illegible]	[illegible]
ENSG00000106484	0.10574372	[illegible]	[illegible]	[illegible]	[illegible]	[illegible]	[illegible]	[illegible]
ENSG00000106511	0.02578379	[illegible]	[illegible]	[illegible]	[illegible]	[illegible]	[illegible]	[illegible]
ENSG00000106524	13.5037112	[illegible]	[illegible]	[illegible]	[illegible]	[illegible]	[illegible]	[illegible]
ENSG00000106526	0.00687568	[illegible]	[illegible]	[illegible]	[illegible]	[illegible]	[illegible]	[illegible]
ENSG00000106536	1.10835304	[illegible]	[illegible]	[illegible]	[illegible]	[illegible]	[illegible]	[illegible]
ENSG00000106537	29.2138791	[illegible]	[illegible]	[illegible]	[illegible]	[illegible]	[illegible]	[illegible]
ENSG00000106538	0	[illegible]	[illegible]	[illegible]	[illegible]	[illegible]	[illegible]	[illegible]
ENSG00000106540	0	[illegible]	[illegible]	[illegible]	[illegible]	[illegible]	[illegible]	[illegible]
ENSG00000106541	0	[illegible]	[illegible]	[illegible]	[illegible]	[illegible]	[illegible]	[illegible]
ENSG00000106546	6.47592674	[illegible]	[illegible]	[illegible]	[illegible]	[illegible]	[illegible]	[illegible]
ENSG00000106554	14.9058691	[illegible]	[illegible]	[illegible]	[illegible]	[illegible]	[illegible]	[illegible]
ENSG00000106560	1.79673139	[illegible]	[illegible]	[illegible]	[illegible]	[illegible]	[illegible]	[illegible]
ENSG00000106565	0.00439157	[illegible]	[illegible]	[illegible]	[illegible]	[illegible]	[illegible]	[illegible]
ENSG00000106571	6.11311359	[illegible]	[illegible]	[illegible]	[illegible]	[illegible]	[illegible]	[illegible]
ENSG00000106588	2.16978958	[illegible]	[illegible]	[illegible]	[illegible]	[illegible]	[illegible]	[illegible]
ENSG00000106591	7.25766949	[illegible]	[illegible]	[illegible]	[illegible]	[illegible]	[illegible]	[illegible]
ENSG00000106603	3.65192135	[illegible]	[illegible]	[illegible]	[illegible]	[illegible]	[illegible]	[illegible]
ENSG00000106605	14.3290985	[illegible]	[illegible]	[illegible]	[illegible]	[illegible]	[illegible]	[illegible]
ENSG00000106608	2.61223677	[illegible]	[illegible]	[illegible]	[illegible]	[illegible]	[illegible]	[illegible]
ENSG00000106609	22.4309807	[illegible]	[illegible]	[illegible]	[illegible]	[illegible]	[illegible]	[illegible]
ENSG00000106610	3.79428779	[illegible]	[illegible]	[illegible]	[illegible]	[illegible]	[illegible]	[illegible]
ENSG00000106615	27.2465039	[illegible]	[illegible]	[illegible]	[illegible]	[illegible]	[illegible]	[illegible]
ENSG00000106617	0.14910556	[illegible]	[illegible]	[illegible]	[illegible]	[illegible]	[illegible]	[illegible]
ENSG00000106624	9.99728135	[illegible]	[illegible]	[illegible]	[illegible]	[illegible]	[illegible]	[illegible]
ENSG00000106628	21.0076726	[illegible]	[illegible]	[illegible]	[illegible]	[illegible]	[illegible]	[illegible]
ENSG00000106631	0.00606849	[illegible]	[illegible]	[illegible]	[illegible]	[illegible]	[illegible]	[illegible]
ENSG00000106633	0.18121936	[illegible]	[illegible]	[illegible]	[illegible]	[illegible]	[illegible]	[illegible]
ENSG00000106635	4.1992201	[illegible]	[illegible]	[illegible]	[illegible]	[illegible]	[illegible]	[illegible]
ENSG00000106636	15.0048947	[illegible]	[illegible]	[illegible]	[illegible]	[illegible]	[illegible]	[illegible]
ENSG00000106638	2.75559793	[illegible]	[illegible]	[illegible]	[illegible]	[illegible]	[illegible]	[illegible]
ENSG00000106648	0	[illegible]	[illegible]	[illegible]	[illegible]	[illegible]	[illegible]	[illegible]
ENSG00000106665	21.8098082	[illegible]	[illegible]	[illegible]	[illegible]	[illegible]	[illegible]	[illegible]
ENSG00000106682	73.2404071	[illegible]	[illegible]	[illegible]	[illegible]	[illegible]	[illegible]	[illegible]
ENSG00000106683	11.4250153	[illegible]	[illegible]	[illegible]	[illegible]	[illegible]	[illegible]	[illegible]
ENSG00000106686	0.05052725	[illegible]	[illegible]	[illegible]	[illegible]	[illegible]	[illegible]	[illegible]
ENSG00000106688	0.2417884	[illegible]	[illegible]	[illegible]	[illegible]	[illegible]	[illegible]	[illegible]
ENSG00000106689	0	[illegible]	[illegible]	[illegible]	[illegible]	[illegible]	[illegible]	[illegible]
ENSG00000106692	1.79740141	[illegible]	[illegible]	[illegible]	[illegible]	[illegible]	[illegible]	[illegible]
ENSG00000106701	0.43954518	[illegible]	[illegible]	[illegible]	[illegible]	[illegible]	[illegible]	[illegible]
ENSG00000106714	0.00710767	[illegible]	[illegible]	[illegible]	[illegible]	[illegible]	[illegible]	[illegible]
ENSG00000106723	8.20834437	[illegible]	[illegible]	[illegible]	[illegible]	[illegible]	[illegible]	[illegible]
ENSG00000106733	0.80851782	[illegible]	[illegible]	[illegible]	[illegible]	[illegible]	[illegible]	[illegible]
ENSG00000106771	6.44833475	[illegible]	[illegible]	[illegible]	[illegible]	[illegible]	[illegible]	[illegible]
ENSG00000106772	0.28827796	[illegible]	[illegible]	[illegible]	[illegible]	[illegible]	[illegible]	[illegible]
ENSG00000106780	10.1436037	[illegible]	[illegible]	[illegible]	[illegible]	[illegible]	[illegible]	[illegible]
ENSG00000106785	1.01769272	[illegible]	[illegible]	[illegible]	[illegible]	[illegible]	[illegible]	[illegible]
ENSG00000106789	0.02905151	[illegible]	[illegible]	[illegible]	[illegible]	[illegible]	[illegible]	[illegible]
ENSG00000106799	5.61440978	[illegible]	[illegible]	[illegible]	[illegible]	[illegible]	[illegible]	[illegible]
ENSG00000106803	27.4280578	[illegible]	[illegible]	[illegible]	[illegible]	[illegible]	[illegible]	[illegible]
ENSG00000106804	0.80531514	[illegible]	[illegible]	[illegible]	[illegible]	[illegible]	[illegible]	[illegible]
ENSG00000106809	0	[illegible]	[illegible]	[illegible]	[illegible]	[illegible]	[illegible]	[illegible]
ENSG00000106819	0	[illegible]	[illegible]	[illegible]	[illegible]	[illegible]	[illegible]	[illegible]
ENSG00000106823	0.2200424	[illegible]	[illegible]	[illegible]	[illegible]	[illegible]	[illegible]	[illegible]
ENSG00000106829	3.44071922	[illegible]	[illegible]	[illegible]	[illegible]	[illegible]	[illegible]	[illegible]
ENSG00000106852	0	[illegible]	[illegible]	[illegible]	[illegible]	[illegible]	[illegible]	[illegible]
ENSG00000106853	0.97272276	[illegible]	[illegible]	[illegible]	[illegible]	[illegible]	[illegible]	[illegible]
ENSG00000106868	1.75791087	[illegible]	[illegible]	[illegible]	[illegible]	[illegible]	[illegible]	[illegible]
ENSG00000106927	0.02184573	[illegible]	[illegible]	[illegible]	[illegible]	[illegible]	[illegible]	[illegible]
ENSG00000106948	0.1444817	[illegible]	[illegible]	[illegible]	[illegible]	[illegible]	[illegible]	[illegible]
ENSG00000106952	0	[illegible]	[illegible]	[illegible]	[illegible]	[illegible]	[illegible]	[illegible]
ENSG00000106976	0.12262713	[illegible]	[illegible]	[illegible]	[illegible]	[illegible]	[illegible]	[illegible]
ENSG00000106991	0.95442056	[illegible]	[illegible]	[illegible]	[illegible]	[illegible]	[illegible]	[illegible]
ENSG00000106992	0.84909519	[illegible]	[illegible]	[illegible]	[illegible]	[illegible]	[illegible]	[illegible]
ENSG00000106993	3.50272579	[illegible]	[illegible]	[illegible]	[illegible]	[illegible]	[illegible]	[illegible]
ENSG00000107014	0.0690884	[illegible]	[illegible]	[illegible]	[illegible]	[illegible]	[illegible]	[illegible]
ENSG00000107018	0.01151473	[illegible]	[illegible]	[illegible]	[illegible]	[illegible]	[illegible]	[illegible]
ENSG00000107020	6.56434292	[illegible]	[illegible]	[illegible]	[illegible]	[illegible]	[illegible]	[illegible]
ENSG00000107021	1.46353936	[illegible]	[illegible]	[illegible]	[illegible]	[illegible]	[illegible]	[illegible]
ENSG00000107036	0.70231704	[illegible]	[illegible]	[illegible]	[illegible]	[illegible]	[illegible]	[illegible]
ENSG00000107077	0.47179616	[illegible]	[illegible]	[illegible]	[illegible]	[illegible]	[illegible]	[illegible]
ENSG00000107099	0.00475909	[illegible]	[illegible]	[illegible]	[illegible]	[illegible]	[illegible]	[illegible]
ENSG00000107104	1.4359694	[illegible]	[illegible]	[illegible]	[illegible]	[illegible]	[illegible]	[illegible]
ENSG00000107105	1.48967983	[illegible]	[illegible]	[illegible]	[illegible]	[illegible]	[illegible]	[illegible]
ENSG00000107130	8.8477556	[illegible]	[illegible]	[illegible]	[illegible]	[illegible]	[illegible]	[illegible]
ENSG00000107140	4.37047245	[illegible]	[illegible]	[illegible]	[illegible]	[illegible]	[illegible]	[illegible]
ENSG00000107147	0	[illegible]	[illegible]	[illegible]	[illegible]	[illegible]	[illegible]	[illegible]
ENSG00000107159	1.73094669	[illegible]	[illegible]	[illegible]	[illegible]	[illegible]	[illegible]	[illegible]
ENSG00000107164	12.022305	[illegible]	[illegible]	[illegible]	[illegible]	[illegible]	[illegible]	[illegible]
ENSG00000107165	0.01341648	[illegible]	[illegible]	[illegible]	[illegible]	[illegible]	[illegible]	[illegible]
ENSG00000107175	9.20988057	[illegible]	[illegible]	[illegible]	[illegible]	[illegible]	[illegible]	[illegible]
ENSG00000107185	5.02328166	[illegible]	[illegible]	[illegible]	[illegible]	[illegible]	[illegible]	[illegible]
ENSG00000107186	2.10881207	[illegible]	[illegible]	[illegible]	[illegible]	[illegible]	[illegible]	[illegible]
ENSG00000107187	0	[illegible]	[illegible]	[illegible]	[illegible]	[illegible]	[illegible]	[illegible]
ENSG00000107201	0.7516427	[illegible]	[illegible]	[illegible]	[illegible]	[illegible]	[illegible]	[illegible]
ENSG00000107223	62.4730105	[illegible]	[illegible]	[illegible]	[illegible]	[illegible]	[illegible]	[illegible]
ENSG00000107242	0.36265669	[illegible]	[illegible]	[illegible]	[illegible]	[illegible]	[illegible]	[illegible]

ENSG00000107249	1.10732783	1.39477996	1.3985224	1.2648097	0.67661125	0.94469397	1.7459091	2.00555415	1.7140913	1.71886132	1.32192119	1.39094352	0.79086887	1.16817531	1.27092909	0.75357196	0.92894954	[illegible]
ENSG00000107262	2.38822876	2.46623366	2.45649657	3.2304973	3.03720197	3.12892401	2.44854595	2.27753384	2.34398422	1.73822441	2.023833	1.99768977	1.41734242	1.18571454	1.02426086	1.63971236	1.229203	[illegible]
ENSG00000107263	2.18157333	2.37693221	2.16573862	1.8170468	2.19031078	1.99831885	2.07959817	2.22660571	2.08350018	2.17297187	1.73848592	1.71177769	2.71644688	2.31375911	2.33552991	2.54364385	2.05225066	[illegible]
ENSG00000107281	1.9581757	2.2435556	2.1110464	2.7828076	2.54553432	3.17397538	4.47398375	5.17087981	3.99414155	6.10375729	3.6787902	3.14295504	4.95771942	3.7961332	4.67085237	3.79458353	3.28173252	[illegible]
ENSG00000107282	2.63871946	4.44835858	3.03279971	4.7027513	2.84747565	4.43518809	0.0225036	0.02744758	0.07388659	0.62180949	0.07060498	0.05521691	0.53365342	0.41488057	0.37368043	1.38764162	0.80094423	[illegible]
ENSG00000107290	2.93123393	3.01481821	2.71218032	2.0497664	3.02043557	2.04914695	3.94730631	3.98511701	3.73524776	3.10396024	4.41470679	3.53851709	4.79319842	5.98269364	6.01861525	6.24606976	7.26396731	[illegible]
ENSG00000107295	5.7371903	9.55748287	7.57276478	7.526976	6.38759122	6.99702437	0	0.01529149	0	0	0	0.01318382	0.02642729	0.02536867	0.03394296	0.04517987	0.02348522	[illegible]
ENSG00000107317	0.30657451	1.17928689	1.0836509	4.0251066	0.69871347	3.26227681	0.26730532	0.28742251	0.12341949	0.29353842	0.56830898	0.15164256	0.96010453	0.88250142	0.19044771	0.0506992	0.11859412	[illegible]
ENSG00000107331	1.06683208	1.16296499	1.01832933	1.2238749	1.00143892	1.18175383	0.53758934	0.63738866	0.44694379	0.71277382	0.65001188	0.38338708	0.5158086	0.46144387	0.51716672	0.44838405	0.43877701	[illegible]
ENSG00000107338	0	0.02542755	0.02227569	0.0098141	0.02541122	0.00569525	2.49378165	2.72187491	2.7326334	1.69852248	2.41039901	1.94276824	2.45484021	1.8917834	1.86206983	1.92626788	1.90742834	[illegible]
ENSG00000107341	21.3072194	21.4661442	20.1496633	22.40988	21.7417737	22.4973352	12.3158108	13.2009975	13.1461258	14.0547599	12.3056749	12.5302946	15.4439199	16.9566264	16.3847076	15.641013	16.3838431	[illegible]
ENSG00000107362	6.63312016	7.84118048	7.04907122	6.6427915	5.88901361	6.60138184	4.48302776	4.74423822	4.85815696	3.4871818	4.1058805	4.28444173	5.64213266	4.97144664	5.7973602	7.38081374	6.79237802	[illegible]
ENSG00000107371	2.82581886	2.34420749	2.31163142	1.7921258	2.67461109	2.09082819	5.47426079	5.73858433	6.53786224	4.94877059	5.09442061	6.19570094	3.6753061	2.3311929	2.67112768	3.87305887	3.43995279	[illegible]
ENSG00000107372	6.52630639	6.36975255	6.46156677	6.921608	5.89750481	7.65175332	5.94223931	6.29693802	6.37419913	6.11678719	5.09971308	5.43535314	10.9890623	10.8798964	12.0457883	12.1912745	13.8004678	[illegible]
ENSG00000107404	7.48427232	6.31077502	6.27715233	7.5986084	6.58733664	6.60194297	9.39224896	10.216868	10.5384295	6.89880359	8.93354006	7.5925553	10.3160406	7.92326644	9.27745021	9.43157053	7.33976869	[illegible]
ENSG00000107438	0.49052249	0.44167047	0.47185788	0.2702541	0.18839677	0.10857633	8.41452323	10.0032421	9.78786618	5.21479633	9.23917556	9.43937301	10.3047088	14.2171882	11.8330635	8.34686111	10.3914202	[illegible]
ENSG00000107443	1.75502179	1.78634522	1.47972365	1.6759106	1.71358513	1.60500408	1.46669043	1.6467027	1.98845748	1.20428433	1.38308814	1.70798108	1.10386004	0.74506048	0.99245096	1.6758282	1.23770877	[illegible]
ENSG00000107447	0	0.00541324	0	0	0	0	0	0	0	0	0	0	0	0	0	0	0	[illegible]
ENSG00000107485	0	0	0.00445892	0	0	0	0.1135054	0.17416922	0.19159697	0.06194201	0.11487833	0.23101987	0	0	0	0	0	[illegible]
ENSG00000107518	1.75986122	2.01849306	1.90424278	2.1312393	1.34019452	1.88077796	1.08195844	1.10515819	1.05285916	1.18288344	0.86765265	0.77727398	0.00268632	0.00171914	0.00276023	0.00306167	0.00477452	[illegible]
[illegible]	[illegible]	[illegible]	[illegible]	[illegible]	[illegible]	[illegible]	[illegible]	[illegible]	[illegible]	[illegible]	[illegible]	[illegible]	[illegible]	[illegible]	[illegible]	[illegible]	[illegible]	[illegible]

ENSG00000108691	0.19548973	0.08729475	0.20393114	0	0.48708255	0.08798502	0.85822774	0.57445124	0.28350208	0.99153309	2.2329597	1.18865467	5.04115027	15.4741822	[illegible]
ENSG00000108700	0	0	0	0	0	0	0.02052569	0	0	0.02314925	0.03219964	0	0.12979971	0.19382251	[illegible]
ENSG00000108702	0	0	0	0	0	0	0	0	0	0	0	0	0	0	[illegible]
ENSG00000108733	2.10965086	2.36763442	1.92321948	2.7350142	1.80513346	2.37141829	2.14399709	2.17004559	2.10752312	1.79239305	2.19522083	1.89196305	1.29049918	1.38880918	[illegible]
ENSG00000108753	0	0	0	0	0	0	0	0	0	0.0024753	0	0	0	0	[illegible]
ENSG00000108759	0	0	0	0	0	0	0.01479022	0	0.0091052	0.03336138	0	0	0.03117665	0.01995185	[illegible]
ENSG00000108771	0.26502594	0.31823452	0.2844775	0.4846211	0.15324579	0.4097272	4.21258872	4.02688857	1.74009635	1.44902197	2.96018078	1.16547152	1.8219209	2.52449354	[illegible]
ENSG00000108773	2.33484217	2.85360056	2.35479101	3.3795941	2.14583509	3.8494822	7.00540623	7.19848032	7.42496048	9.10871371	6.0770289	6.18917458	6.40903551	3.82487874	[illegible]
ENSG00000108774	5.58335939	4.94035988	5.41294324	4.4714914	5.46620256	4.26105529	5.72503852	6.0237655	6.13281036	5.22920554	6.26437395	6.01693215	5.013469	4.70744494	[illegible]
ENSG00000108784	3.58481055	3.0492147	3.06285957	4.1928175	3.31304841	3.49031859	4.52714832	4.360429	2.65983159	3.73817633	3.07892359	1.99305779	2.81174146	3.22317794	[illegible]
ENSG00000108785	0.06749684	0.38428884	0.16502674	0.7852279	0.18825586	0.60757268	0.10841002	0.06611369	0.05005478	0.18340025	0.08503402	0.08550154	0.17138998	0.03656099	[illegible]
ENSG00000108786	0.18819786	0.25669975	0.15393618	0.5042276	0.11643322	0.62628988	0.40394789	0.46923242	0.34848983	0.53925787	0.48712424	0.34676281	0.09557553	0.10379454	[illegible]
ENSG00000108788	2.17653374	1.60703679	1.84264983	2.0474919	2.46063897	1.79971594	2.55345528	2.77682263	2.75237642	2.95349623	3.02503269	3.07600579	2.52078722	2.21946975	[illegible]
ENSG00000108797	1.01633065	0.79805951	1.07537866	1.2654819	1.43013918	1.55274661	6.59637946	6.38078673	3.0687276	5.2396473	6.18134692	2.77610897	4.85520985	5.85661333	[illegible]
ENSG00000108798	0	0	0	0	0	0	0.02351707	0.00573674	0.01447765	0.15383336	0.02213542	0.05935233	0.03965775	0.06344856	[illegible]
ENSG00000108799	0.73639806	1.10465543	0.91153005	0.9249968	0.45761414	0.95108239	0.71757739	0.70169061	0.58608992	1.08766737	0.70647275	0.6999488	0.7145721	0.53407124	[illegible]
ENSG00000108813	0	0	0.01079826	0	0	0	0.01330056	0.0108151	0.01091751	0.02500103	0.02086523	0.02331106	0	0	[illegible]
ENSG00000108819	8.11566392	8.07599512	7.89782256	7.5785221	7.54785698	7.76940305	13.0963462	14.8292946	14.5288958	15.6536855	14.7220671	14.0853716	12.2043435	11.220664	[illegible]
ENSG00000108821	0.09689593	0.08365202	0.1789958	0.0333998	0.04564274	0.04522563	98.3093408	129.071729	109.157352	118.231406	61.1768882	58.9001827	61.340765	45.3871982	[illegible]
ENSG00000108823	0	0	0	0.0036359	0	0	0.01355362	0.02479696	0.01529719	0.03057204	0.01771851	0.00237546	0	0.00304728	[illegible]
[illegible]	[illegible]	[illegible]	[illegible]	[illegible]	[illegible]	[illegible]	[illegible]	[illegible]	[illegible]	[illegible]	[illegible]	[illegible]	[illegible]	[illegible]	[illegible]

ENSG00000110172	0.88366607	1.04023469	1.00697878	0.7808998	1.15218612	1.08107906	2.18507338
ENSG00000110195	0	0.02913359	0.03544771	0.0187408	0	0.01087554	0.15718356
ENSG00000110200	1.31539673	1.19164358	1.32832975	1.3224586	1.53920143	1.11902948	0.90863321
ENSG00000110203	0	0	0	0	0.00710189	0	0.01022433
ENSG00000110218	8.50271808	7.04474923	7.31033457	5.8048705	8.40122919	6.18973608	6.06857458
ENSG00000110237	2.20445343	2.45392261	1.76902849	1.8823834	2.1171794	1.73317811	3.53607784
ENSG00000110243	0	0.01488143	0.00724267	0	0	0.01111045	0.00594736
ENSG00000110244	0	0	0	0	0	0	0
ENSG00000110245	0	0	0	0	0	0	0.04357389
ENSG00000110274	1.00288474	1.04223657	0.92970819	0.9616154	1.04410124	0.92949837	3.29271038
ENSG00000110315	3.6418497	3.99414965	3.67997491	3.6715367	3.7438671	3.53721554	1.52407086
ENSG00000110318	0.21415109	0.31665453	0.19955687	0.2063057	0.14537818	0.24550651	0.54676455
ENSG00000110321	20.7387085	18.086611	17.694625	17.548443	21.6689382	17.8399752	13.9991829
ENSG00000110324	0	0.0008567	0.00125085	0.0049598	0	0.00095942	0.05751982
ENSG00000110328	0.08003412	0.0428865	0.08609912	0.4034684	0.58484604	0.27616368	0.32779427
ENSG00000110330	5.84941441	5.59296079	5.64864208	5.1207748	4.82510978	5.62862932	3.82716899
ENSG00000110344	6.19014565	7.41469724	5.55395158	6.5781588	5.8006297	6.2277738	11.6963984
ENSG00000110347	0	0	0	0	0	0	0
ENSG00000110367	21.309245	22.9925377	22.6445679	19.126569	17.5235501	19.1882789	10.2351577
ENSG00000110375	0.01719837	0.01151974	0.03363939	0.0222309	0	0.01290092	0

[illegible]

ENSG00000111700	0	0	0	0	0	0	0	0	0	0	[illegible]
ENSG00000111701	0	0	0	0	0	0	0	0	0	0	[illegible]
ENSG00000111704	0.01475721	0.01976922	0.02886457	0.0095377	0.01234783	0.0221395	0.02962785	0.0289096	0.01823959	0	[illegible]
ENSG00000111707	5.42270054	5.2744034	5.27555227	5.182829	4.69326112	4.68924579	5.04759704	5.65623338	5.08129336	4.4694055	[illegible]
ENSG00000111711	3.71919059	3.14113849	3.45321478	4.3670115	3.97368491	3.17206295	5.62172988	6.23375002	6.69068772	5.34677315	[illegible]
ENSG00000111713	0.01029047	0.01378543	0.01006389	0.0133016	0.01722072	0.04631481	0	0.01007959	0.01526255	0.04660154	[illegible]
ENSG00000111716	67.8255098	55.7821276	63.0566728	71.416016	72.6859209	71.7367873	80.1795247	92.008398	98.9204244	80.2910606	[illegible]
ENSG00000111725	1.67802552	1.66719432	1.59287624	2.0271439	1.29220783	1.73768418	1.82975822	1.8499172	1.7057224	1.50968007	[illegible]
ENSG00000111726	5.09620698	4.26993002	4.60651259	4.6151077	5.00933565	4.6778302	6.96958879	7.27794936	7.58241583	6.70239709	[illegible]
ENSG00000111727	2.69457059	2.51742552	2.44834507	2.8943051	2.83739112	2.73802485	1.40170226	1.40703117	1.32272444	1.15103185	[illegible]
ENSG00000111728	3.56033225	4.78483246	4.3162929	3.0652024	2.53087561	3.1342859	0.75202702	0.76590625	0.45034489	1.11464192	[illegible]
ENSG00000111729	0.04375474	0.02930762	0.01711654	0.0678697	0.02440732	0.08533594	0.13352565	0.16286083	0.09518065	0.15851863	[illegible]
ENSG00000111731	1.39538192	1.60717775	1.40328934	1.3999706	1.13809617	1.39676271	1.12944873	1.34789202	1.655403	1.60079467	[illegible]
ENSG00000111732	0.00513049	0.00687297	0	0	0	0.00769702	0.0123605	0.00502536	0.00507294	0.0046468	[illegible]
ENSG00000111737	4.83257859	3.67250301	4.01992652	3.7117266	5.01723768	3.40168169	4.53232345	5.05202456	4.71238013	3.54311869	[illegible]
ENSG00000111752	1.14367647	1.33236526	1.0905606	1.1652414	1.13699628	1.22214349	0.6817324	0.79681422	0.73488146	0.92823255	[illegible]
ENSG00000111775	49.314369	33.9239852	45.2266522	48.94206	60.9441664	44.0765733	23.5939294	25.3248125	25.3644944	25.1585121	[illegible]
ENSG00000111780	0	0.03910868	0.05710164	0	0.03256963	0	0.0468893	0	0	0	[illegible]
ENSG00000111783	4.8947192	6.48609305	5.172142	7.029754	4.06270568	6.63245552	0.00540691	0.0049461	0.00166431	0.00304901	[illegible]
ENSG00000111785	1.18048749	1.07478555	1.07391864	1.1208688	1.01336833	1.03444943	0.74301322	0.88243711	0.73854332	0.7817107	[illegible]
[illegible]	[illegible]	[illegible]	[illegible]	[illegible]	[illegible]	[illegible]	[illegible]	[illegible]	[illegible]	[illegible]	[illegible]

ENSG00000112851	4.13431616	4.07169994	4.40704105	2.6619836	3.44928476	2.89028924	2.77061172	2.74844682	2.82754085	2.15105891	2.96769089	2.84961711	2.93510937	3.000835	2.88702964	3.09411325	2.94141033	2.29182711	2.04960439	1.91292039	2.47949108	2.77330065	3.36489733	2.99245057	2.46853659	2.42537356	2.24527467	2.47633437	2.62621751	2.14185868	3.34584667	3.38178862	3.61796212	4.91369647	4.52819528	3.79317328	3.09820935	2.90968776	2.45871008	3.27261567	3.38090365	2.76888818	3.53834669	2.95279455	2.97890455	3.05561138	2.70463234	2.2645488
ENSG00000112852	0.63611328	0.64700849	0.55298433	0.7004358	0.67681974	0.60971006	3.14076036	2.80385113	2.74304228	4.81185454	3.79918084	3.15354643	0.31905933	0.43389445	0.24587802	0.44318713	0.26581814	0.21314089	4.42070836	3.80730642	5.84064299	5.04053022	8.15021296	8.40011477	4.84670919	5.01204149	4.18602186	5.05403235	5.30387186	4.34941843	0.66222132	1.39800229	1.29479077	0.44495636	0.34413186	0.36675306	4.52387301	4.53444766	3.9037292	3.04643276	3.25689579	2.19377669	0.49903082	0.25142857	0.26078628	0.25887522	0.20979516	0.20925068
ENSG00000112855	1.4943151	1.53670036	1.45453609	1.4157321	1.41018871	1.50863038	2.76293138	2.67425421	2.37016885	2.33905427	2.9035897	2.49717233	1.84810064	1.51410105	1.57786815	1.74001283	1.49689848	1.08574547	2.56545006	3.11104506	3.3963357	2.80477433	2.96691979	3.7723173	4.19390417	3.26396704	2.70761571	3.14296728	3.05508574	3.08147245	1.00431936	1.08189892	1.12190724	1.05417742	1.01346297	0.96488025	3.24879964	2.91767681	2.93596495	2.52456057	2.59777292	2.10919355	1.92427035	1.74406754	1.38002073	1.9944957	1.49660058	1.58771647
ENSG00000112874	1.51121882	2.16908211	1.67969607	1.9472254	1.41836418	1.78137586	2.33092439	2.48118241	2.21056658	2.57671707	2.76211842	2.56460079	2.28615649	2.55709211	2.84974011	2.8138582	3.1611388	1.88076686	2.5659598	2.31734898	2.67349545	3.83112393	6.49764162	3.93846194	2.65169976	2.72426467	3.17021527	4.50371807	4.20646461	3.48869521	1.33391642	1.29132311	1.47964911	1.63093264	1.58839544	1.53206165	1.59823598	1.97241041	1.93777903	4.26924992	3.78207984	3.32465156	2.68053546	1.67113245	1.73474504	2.04271741	1.31580724	1.97800758
ENSG00000112877	1.29767999	1.19879427	1.24080049	1.3219681	1.3616357	1.08921945	0.5966121	0.70878507	0.9015255	0.54616087	0.56520705	0.89626672	1.02895558	0.57487376	0.88524918	1.25649545	0.92166205	0.61099749	0.87598375	0.60396279	1.64059488	1.4173319	1.29011018	1.95098412	1.43680856	1.38555625	0.86335498	1.60049031	1.42724102	1.17968677	1.42192859	0.75547612	1.4957576	1.21478882	1.2777537	1.3860626	0.94639577	0.98194468	0.7434467	1.18558248	1.3968458	1.08843937	1.13228075	0.52718034	0.59183379	1.88838257	0.58851922	0.44650167
ENSG00000112893	3.03610212	2.24729679	2.91615194	3.0062615	2.8584273	3.17689798	4.83464673	5.34723355	4.84420966	4.05718015	4.8367651	4.80126425	6.17170167	6.72776291	7.4453092	5.53252841	4.69673006	3.80964125	4.40441742	7.33819542	6.39322311	5.45665307	10.4940239	7.51108815	9.32557208	7.91477133	9.91593186	7.11352658	8.46908243	7.01743959	4.28982103	5.75735005	6.73263746	5.16650372	4.67049204	6.34732217	6.09644742	5.92046929	4.93588907	8.77982387	7.98779857	7.00045018	6.02761046	4.24353537	4.35531024	5.71756984	4.06747539	3.41381476
ENSG00000112902	4.64816189	6.23774381	4.85783146	5.0570795	2.46943677	3.20895355	2.42371596	3.20711108	3.90293479	3.79021659	1.17058485	1.9312187	2.26869855	3.78926617	3.52198938	2.75418437	3.4612288	3.51408953	7.33990608	7.78091497	8.37053553	2.89189757	6.19002049	4.55528542	2.21416017	1.38753096	4.44018637	5.41351418	7.19576267	9.84211819	2.15475914	2.51580429	1.91018223	4.31382623	3.70540495	3.53605673	0.36634581	0.43177636	0.35184797	2.88777282	4.00665345	4.16585902	3.50349103	3.56082152	4.02982634	4.26044271	4.81305257	5.33625509
ENSG00000112936	0.00426941	0.01143886	0	0	0.00476313	0.01281035	0.33257923	0.30109798	0.0147753	0.01546759	0.06992299	0.00721102	0.00722733	0	0.00742617	0.0041186	0	0.00514994	0.06577975	0.39046341	0.0359395	0.02552485	0.02594933	0.01984662	0.00815129	0.01054076	0.03673484	0.0096577	0.03065859	0.03434355	0.00648676	0	0	0.01343887	0	0.01139341	0.00790167	0.01290651	0.02150208	0.01371584	0.0079757	0.12232233	0.01466471	0.01093509	0.00393822	0.00586404	0	0.02394921
ENSG00000112941	5.00398862	3.92342803	3.97907997	3.7026138	5.35874756	4.41137618	2.13134823	2.87826388	2.96524647	2.72851299	2.61690401	3.16281608	4.26181471	3.22981164	4.06724456	4.68812921	3.8283984	3.1543408	3.4954708	3.1628109	4.51085272	4.59333549	5.49143651	5.82259142	4.04770796	5.15407311	3.51417436	4.86921378	4.6400279	3.86537973	4.03827968	4.5773905	4.73115413	4.19539509	4.50089514	4.55524404	2.89232613	2.21488263	2.17079844	3.47431577	3.87315042	3.25901637	4.24788914	3.69990001	3.85011365	4.89781278	3.53283882	3.92409538
ENSG00000112964	0.09990752	0.11532959	0.1268122	0.0604495	0.05454478	0.08610485	0.04609142	0.04164264	0.05044434	0.03465517	0.05088189	0.04846892	0.09175954	0.0506626	0.06655345	0.09022665	0.11512124	0.03846151	0.15205841	0.20384488	0.21651617	0.36491705	0.25839797	0.36758891	0.30844155	0.24141419	0.21033371	0.13944553	0.16180463	0.12653475	0.02583753	0.03380953	0.03487261	0.01003661	0.01655973	0.01701797	0.04524283	0.04926613	0.0406815	0.11609252	0.06750729	0.07612875	0.1241239	0.05081504	0.05098078	0.10218759	0.05678666	0.0596203
ENSG00000112972	65.4129479	54.7493793	56.6785257	18.511363	88.8739336	46.9951518	20.8806131	14.4267586	18.3949293	15.7432527	15.8447857	15.4233515	8.25342456	32.4659313	18.6467226	6.81369861	8.65335837	13.0522866	30.5887147	30.8064815	24.0654527	52.2514942	64.8396551	44.7396043	25.5274255	39.2440637	37.6485952	45.1133483	26.5856131	32.8035363	7.42439188	7.59405852	6.58093097	10.1637629	9.65733919	7.97649051	10.4456664	10.8815322	10.2673684	4.27725762	2.47255862	2.70144513	13.8505583	21.8154578	21.1920574	4.67403799	21.6115634	40.4566511
ENSG00000112977	6.86781193	5.80799332	6.60773777	8.3091195	7.67754615	5.84473513	9.52451118	10.7720447	10.0183177	9.71359946	11.7022364	10.866516	10.4906938	14.0302073	13.4124313	11.0062386	14.3504892	11.513362	14.0505533	16.6882208	12.499531	14.4644851	17.7309266	17.0700463	17.096935	22.6537839	15.1896283	15.7956642	15.1789933	16.193083	26.4587625	23.2739841	21.4289263	18.1667593	16.2804378	14.7036338	13.1587835	10.694839	9.48520392	10.2360815	9.58595965	9.40791966	11.7885367	13.5277202	11.468832	13.1098329	16.5330901	11.4315693
ENSG00000112981	0.11936941	0.23415545	0.12507979	0.1212348	0.14744219	0.19827148	0.50670213	0.59296809	0.2992914	0.37840453	0.5800535	0.36722686	0.12268591	0.19397634	0.07415369	0.13982852	0.11544102	0.08227915	0.15013494	0.10599095	0.1220165	0.16020847	0.08637191	0.24573999	0.12209145	0.04911865	0.02200883	0.0128582	0.04285957	0.02057615	1.04932676	1.11620566	0.67141389	1.04670687	1.04616084	0.71294799	0.37872837	0.48114209	0.3005911	0.21913412	0.28670743	0.5343815	0.078098	0.01455891	0.02359495	0.32790861	0.05314811	0.03985727
ENSG00000112983	1.85541289	2.03558687	1.78575794	1.6595963	1.48432758	1.58106543	1.777687	1.6521034	1.84876707	1.22990783	1.83509555	2.27135883	1.73617491	1.5073156	1.75002043	1.53494536	1.50517234	1.45364706	1.64421781	1.18241513	1.52836908	2.35626779	1.97950836	1.81380568	1.28566447	1.54987419	1.15263857	1.73332711	1.66332096	1.43488783	0.9807987	1.06247443	1.31234628	1.28030861	1.43859711	1.5024799	1.53134974	1.37637224	1.42712291	1.74524323	1.8021974	1.68902088	1.64315661	1.19196032	1.10721619	1.30973946	1.28788765	1.36257119
ENSG00000112984	13.2576946	8.24863256	10.6080741	8.2984281	11.4857561	8.87393437	3.791539	5.13545407	10.1003615	3.57962504	3.86702335	11.4304831	9.58563253	9.26956972	12.1844686	8.299395	10.4324479	9.75653238	14.3327347	4.09119122	6.51036161	28.5405248	28.0115974	14.9651149	6.38705849	16.8104118	11.1166177	26.1525621	23.7180301	20.8155296	1.93557732	0.15136379	3.80138749	1.89449208	3.43836243	9.28289806	4.41849766	5.25617409	5.50662694	21.7415417	15.8532343	14.2381984	12.1396453	2.22665705	2.47361426	7.61446055	4.46622742	2.22575509
ENSG00000112992	9.41271872	8.03407013	8.21991476	10.660744	10.7951177	9.78773838	2.96565201	3.02236896	3.42262346	3.64648515	3.6763996	3.66889144	2.8575689	3.3569751	3.31873341	3.30434921	3.76287057	2.96570723	4.64387494	3.80071223	4.62011289	6.72433232	6.94304046	7.07872729	4.72073668	5.74190724	5.15872129	6.70510687	7.78229577	6.60129988	5.8826137	5.7691619	6.71255759	6.22793891	5.86237914	7.72474967	4.75539207	5.26923726	4.97447126	5.58647205	4.89959153	4.7876359	4.68101812	3.01128586	2.97493172	2.52481451	2.63951516	2.33544086
ENSG00000112996	7.79922626	8.52935536	8.11960019	7.9989461	7.41838191	7.49923118	6.80214612	8.2273833	8.13071446	6.34035239	7.61250715	7.82768836	6.28379086	5.45588346	5.88854613	7.80475483	6.4075593	4.74381209	6.11557759	5.29548509	9.53774044	6.68753976	9.81424832	11.55161	8.15314049	7.74436081	6.30425531	6.88256861	7.98096621	6.60397945	13.3989629	14.7425653	13.1279484	10.5520835	9.5332159	9.32881328	7.58487586	7.62335444	6.0108341	8.70639471	8.9958284	8.78897298	6.62525499	5.10985493	5.93208426	6.53526989	5.31000678	4.6464998

ENSG00000114520	11.9540539	9.99880954	10.9391995	10.614757	13.1629195	9.62591965	4.33524675	[illegible]
ENSG00000114529	0.12255689	0.02736352	0.07191506	0.0633676	0.1291336	0.07558933	0.42649688	[illegible]
ENSG00000114541	1.55593621	0.66924149	0.89162766	0.6432609	2.50409817	0.88066505	0.04130688	[illegible]
ENSG00000114544	1.73828809	1.66678006	1.65471437	2.0939289	1.40172796	1.74887256	1.05498956	[illegible]
ENSG00000114547	0	0	0	0	0	0	0	[illegible]
ENSG00000114554	5.4364737	5.39332684	4.96973625	5.6459071	5.36204302	4.87966383	5.49477835	[illegible]
ENSG00000114573	15.1677509	14.2516353	13.996899	15.303274	14.4995352	14.994393	18.743858	[illegible]
ENSG00000114626	0.23083714	0.45521187	0.30147351	0.4207029	0.09277541	0.30651959	0.51468718	[illegible]
ENSG00000114631	64.8428159	65.3861325	57.8349257	60.528083	47.684797	52.1792873	8.96310601	[illegible]
ENSG00000114638	0.02016564	0.03714499	0.02465205	0.0456163	0.01687324	0.03403519	0.04858356	[illegible]
ENSG00000114646	36.0360535	62.7638759	40.9215996	60.650782	24.46667	43.3217335	0.11137013	[illegible]
ENSG00000114648	1.51006967	1.47065326	1.33167324	1.336821	1.53094633	1.35186697	3.38647598	[illegible]
ENSG00000114650	6.25184016	6.70908385	5.60407973	6.561894	5.14561078	5.73592488	4.65198995	[illegible]
ENSG00000114654	0.09656193	0.02939942	0.02146269	0.4482086	0.26197638	0.32265835	0	[illegible]
ENSG00000114656	0.2830895	0.30907707	0.24640224	0.7684451	0.53690539	0.60732808	0.05228867	[illegible]
ENSG00000114670	0.77614966	1.01090988	0.84916824	0.5501771	0.43351145	0.50182674	0.27505902	[illegible]
ENSG00000114686	17.1690139	14.5581775	14.8962979	14.947215	17.7777917	13.2261886	15.195117	[illegible]
ENSG00000114698	1.11573471	1.8416048	1.57676705	2.0362197	0.85808142	2.07701428	2.18453365	[illegible]
ENSG00000114735	0.64338334	0.86189671	0.65803646	0.8887842	0.48491639	0.82892377	1.98785664	[illegible]
ENSG00000114737	0.06967086	0.03966665	0.07835738	0.1035664	0.06606861	0.07839277	0.12029446	[illegible]
ENSG00000114738	2.25097683	2.61158002	3.01898757	4.8449887	2.98716848	3.79851332	7.84713616	[illegible]
ENSG00000114739	6.15299693	9.23874868	7.7609299	8.3710241	4.30845563	7.42713454	0.90179034	[illegible]
ENSG00000114742	2.8567378	3.87981762	3.33507136	3.0035753	1.94959739	2.89785998	3.34815048	[illegible]
ENSG00000114744	13.6384836	14.2043513	13.8967761	13.874596	12.0250697	11.9718735	5.68768375	[illegible]
ENSG00000114745	0.91596942	1.00395955	0.87387642	1.1453588	1.01117531	1.04745409	0.63013998	[illegible]
ENSG00000114757	0.03634092	0.03432808	0.02733905	0.0325211	0.01663322	0.02795921	0.24395232	[illegible]
[illegible]								

ENSG00000115750	2.20956918	2.04484659	1.94540179	1.7584926	2.28971408	1.55606605	2.09064116	[illegible]	[illegible]
ENSG00000115756	0.9357995	0.95257654	0.97095968	1.4394165	0.98788778	1.09194786	8.37712521	[illegible]	[illegible]
[illegible]	[illegible]	[illegible]	[illegible]	[illegible]	[illegible]	[illegible]	[illegible]	[illegible]	[illegible]

ENSG00000117010	0.8586093	0.73942729	0.97405718	0.7356738	0.8539004	0.75079482	0.52404091	0.50941458	0.61368986	0.43993445	[illegible]	[illegible]
ENSG00000117013	0	0.00732107	0.00267233	0.0035321	0.00304848	0.00819884	0.01755516	0.02141198	0.00270184	0.05197247	[illegible]	[illegible]
ENSG00000117016	4.36665802	4.66578345	4.73366868	5.7745599	3.00784647	4.61540478	0.68577837	0.63554968	0.61944457	1.07740308	[illegible]	[illegible]
ENSG00000117020	14.6757065	15.5355183	14.8510159	15.287291	11.3463383	16.8670244	7.20460298	8.97156985	8.64654991	12.0416091	[illegible]	[illegible]
ENSG00000117036	7.37208496	7.54095117	7.59698123	6.9536491	6.20806956	6.65018541	6.75326108	7.4149253	7.80215846	5.01267075	[illegible]	[illegible]
ENSG00000117054	4.04542043	3.84273904	4.03102371	3.1318128	4.51242419	2.85392817	1.45988524	1.60945975	1.60945427	1.20627018	[illegible]	[illegible]
ENSG00000117069	9.55714975	7.92410201	7.16868512	3.8473515	10.0131873	5.53929667	0.00336185	0.00410044	0	0	[illegible]	[illegible]
ENSG00000117090	0	0	0	0	0	0	0	0	0	0	[illegible]	[illegible]
ENSG00000117091	0	0	0	0	0	0	0	0	0	0	[illegible]	[illegible]
ENSG00000117114	0.00244822	0.00515383	0.00239431	0.0027125	0.01229101	0.00341059	0.18312837	0.26412796	0.18518781	0.0034845	[illegible]	[illegible]
ENSG00000117115	0	0	0.00220636	0	0	0	0.0054353	0.00220981	0.00557683	0.02247678	[illegible]	[illegible]
ENSG00000117118	12.0215666	8.12914358	10.2128739	10.485674	15.9416903	9.77560878	9.90779058	10.5212212	12.2074435	9.6818714	[illegible]	[illegible]
ENSG00000117122	0.00850726	0.00284915	0.00831994	0	0	0	3.67218023	6.23302799	9.28876123	8.69148229	[illegible]	[illegible]
ENSG00000117133	18.246709	14.2896324	15.4088335	13.748624	15.9240003	13.7217005	22.3483867	25.8661582	27.614583	18.5472814	[illegible]	[illegible]
ENSG00000117139	13.5688132	15.9583977	13.9634899	15.835112	10.8181156	18.5210234	8.27878885	8.34097644	8.69108129	7.34891408	[illegible]	[illegible]
ENSG00000117143	5.84818681	4.38493458	4.88049466	5.8553751	6.12656979	5.08904057	12.2066313	14.5110226	14.7421725	10.1329579	[illegible]	[illegible]
[illegible]	[illegible]	[illegible]	[illegible]	[illegible]	[illegible]	[illegible]	[illegible]	[illegible]	[illegible]	[illegible]	[illegible]	[illegible]

ENSG00000118690	0.21688923	0.24828963	0.19668732	0.2191864	0.17377907	0.16269401	0.25968398	0.41330157	0.39576941	0.19286983	[illegible]	[illegible]
ENSG00000118702	0	0.03075212	0	0	0	0.01721961	0	0	0	0	[illegible]	[illegible]
ENSG00000118705	27.3152375	21.1837755	22.3245334	21.622087	29.554862	19.7715155	40.2361595	43.7701313	41.2135754	28.7788179	[illegible]	[illegible]
ENSG00000118707	2.55051136	2.7370488	2.39955699	2.5607236	1.62718354	2.78118676	5.23252839	6.77671533	6.46798886	4.48019171	[illegible]	[illegible]
ENSG00000118729	0.01512068	0	0	0	0	0.0056712	0	0	0	0	[illegible]	[illegible]
ENSG00000118733	0.26034709	0.0576705	0.05012102	0.0768452	0.89652036	0.15531162	0	0	0.00101349	0	[illegible]	[illegible]
ENSG00000118762	2.88442518	3.23722191	2.98166675	2.3542735	2.83792192	2.41027484	6.05715469	5.8661692	4.70670171	6.26229052	[illegible]	[illegible]
ENSG00000118777	0.695171	0.55336518	0.64045231	0.2127096	0.44398136	0.29222197	0.28587961	0.2796071	0.28391498	0.23421099	[illegible]	[illegible]
ENSG00000118785	4.59944193	0.32452977	4.39945533	0.6349789	8.24316594	0.96412546	8.74111679	5.72788605	7.8414629	16.8552627	[illegible]	[illegible]
ENSG00000118804	0.11829839	0.11005297	0.1606857	0.1189335	0.07698759	0.11831809	0.08444663	0.13518659	0.1137222	0.2797689	[illegible]	[illegible]
ENSG00000118816	41.8832513	39.4108626	37.2471205	56.761758	37.8439507	50.1334062	21.8548969	23.2456907	24.3347023	32.8666928	[illegible]	[illegible]
ENSG00000118849	0.00831521	0.01113931	0.02439637	0.0429935	0.0185536	0.02494977	0.34056438	0.30950281	0.16032769	0.03765635	[illegible]	[illegible]
ENSG00000118855	2.29127914	2.92668023	2.44631425	2.4106312	2.52202838	2.25139551	3.06308608	2.90981245	2.84789437	1.29460881	[illegible]	[illegible]
ENSG00000118873	10.6776491	9.57211739	10.6479833	10.801029	13.3789849	9.72626204	9.01710544	9.28495696	9.31275003	7.47003288	[illegible]	[illegible]
ENSG00000118894	0.79685866	0.62673611	0.62996767	0.5455226	1.00981561	0.62273626	0.70237002	0.7424548	0.77556595	0.59096601	[illegible]	[illegible]
ENSG00000118898	0.0308801	0.01095034	0.01776481	0.01174	0.05269	0.01635101	1.4164622	1.18320328	0.28108973	0.18920074	[illegible]	[illegible]
ENSG00000118900	2.02373859	1.66947746	1.58289238	1.6713227	1.85077807	1.70510755	2.09154688	2.32602704	2.0986355	1.22963173	[illegible]	[illegible]
ENSG00000118903	0	0	0	0	0	0	0	0	0	0	[illegible]	[illegible]
ENSG00000118922	9.38718585	13.6521077	10.2647173	9.3147012	9.76425435	10.8054977	1.65094649	1.99949046	1.61569184	2.75256944	[illegible]	[illegible]
ENSG00000118939	0.69986629	0.63105206	0.68445666	0.6031059	0.69571338	0.61248496	1.16011852	1.38862676	1.60583134	0.9345737	[illegible]	[illegible]
[illegible]	[illegible]	[illegible]	[illegible]	[illegible]	[illegible]	[illegible]	[illegible]	[illegible]	[illegible]	[illegible]	[illegible]	[illegible]

ENSG00000120210	0	0.03595003	0.02624488	0.0693767	0.02993911	0.0603905	0.06465335	0.02628583	0.01326737	0.0486115	[illegible]
ENSG00000120211	0	0	0	0	0	0	0	0	0	0	[illegible]
ENSG00000120215	0.01250426	0.03350222	0.00611447	0.0161632	0.01395029	0.0468988	0.01004186	0.01224803	0.015455	0.02265079	[illegible]
ENSG00000120217	0.1586763	0.04943437	0.09744022	0.085859	0.17290915	0.10795476	0.32301755	0.48434674	0.38129472	0.53810215	[illegible]
ENSG00000120235	0	0	0	0	0	0	0	0	0	0	[illegible]
ENSG00000120242	0	0	0	0	0	0	0	0	0	0	[illegible]
ENSG00000120251	1.1140623	1.99380706	1.7353484	0.5057409	0.66960602	0.9183588	0	0.00179923	0.00045407	0	[illegible]
ENSG00000120253	4.18032038	4.00469735	3.67336198	3.871602	3.67208699	3.6114019	4.57366897	5.47008247	6.61508184	3.85512711	[illegible]
ENSG00000120254	3.87207047	3.48798036	3.29598699	3.3577048	3.40701184	3.42931512	3.30253594	4.15024694	5.02113799	2.63371019	[illegible]
ENSG00000120256	4.63835946	4.86954831	4.21824882	5.6738409	4.67881403	5.16757608	5.34875036	5.61320232	6.87195973	5.56307824	[illegible]
ENSG00000120262	0.03572837	0.0533855	0.04031735	0.039078	0.03986009	0.02680074	0.0331068	0.05384035	0.04212137	0.02240307	[illegible]
ENSG00000120265	3.97412413	3.64130402	3.90519291	4.7618242	4.88766477	4.33834982	5.0367181	5.69155899	5.97612776	3.73458745	[illegible]
ENSG00000120278	3.67561557	3.57662243	3.54642224	2.5337831	4.03350324	2.35144307	0.00475584	0.00580069	0.00487968	0.00178791	[illegible]
ENSG00000120279	0.00524658	0	0	0.0203455	0.10243289	0.06690503	0.00842679	0	0.00518773	0.07127919	[illegible]
ENSG00000120280	0.05212357	0.04655092	0.01699197	0.0449172	0.02907564	0.01303306	0.11162433	0.01701848	0.12884721	0.0786825	[illegible]
ENSG00000120289	0	0	0	0	0	0	0	0	0	0	[illegible]
ENSG00000120306	9.70683927	6.2160714	9.51165571	6.9352816	10.9298309	6.66982264	8.66315642	7.74376036	5.00518372	4.88523422	[illegible]
ENSG00000120314	1.50010575	1.52228539	1.36003656	1.9862207	1.83519383	1.77080585	3.68475326	3.56725794	3.17875749	3.46181504	[illegible]
ENSG00000120318	4.77716807	2.87152217	3.26310187	1.9306388	5.8477945	2.6978518	0.94813514	1.08182873	1.17557416	1.93168278	[illegible]
ENSG00000120322	2.81757592	2.42078537	2.77879214	2.3050923	2.55318977	2.74670212	0.17185248	0.10480398	0.1410618	0.49531372	[illegible]
[illegible]											

ENSG00000121797	0	0	0	0	0	0	0.02854731	0.03481907	0.01366896	0	0.04478354	0.00333554	0.06351861	0.02139445	0.01030518	0.00381021	0.01188363	0	0.00434674	0.00701411	0.0299236	0.01686687	0.01200316	0.03672111	0.04901613	0.05200799	0.08496056	0.02382548	0.01985407	0.0127088
ENSG00000121807	0	0	0	0	0	0	0	0	0	0	0	0	0	0	0	0	0	0	0	0	0	0.00315023	0	0	0	0	0.00278118	0	0	0
ENSG00000121848	7.62009207	7.01833466	7.64834853	6.6346199	7.4746377	6.65236353	4.89755451	4.88175647	5.06912364	3.93910736	5.01616189	4.91678176	2.73258439	2.82958327	2.69946813	3.20229656	2.77789478	2.75127688	3.6866495	3.68910823	4.9981695	3.42138655	4.32873702	4.82841753	4.76349932	4.03909736	4.20461597	4.36483166	4.36406713	4.1117005
ENSG00000121851	11.1961409	8.92164365	9.98390846	9.3762972	6.99092336	9.02783458	2.81426522	3.30164852	2.38956523	2.60911956	3.53564056	2.57696249	3.60711232	4.61682461	1.49063139	3.10841197	3.37307976	3.54647581	5.43357215	4.08728051	2.60619993	3.82368508	5.33237638	3.13380534	3.13978366	3.82494112	4.10760544	6.24204543	5.85401902	6.19929303
ENSG00000121853	0	0	0	0	0	0	0	0	0	0	0	0	0	0	0.01345014	0	0	0	0	0	0	0	0	0	0	0	0	0	0	0
ENSG00000121858	0.85741119	0.78676822	0.94516697	0.3843829	0.23222952	0.36805422	0.74030725	0.61167617	0.12986431	0.09426654	1.51725513	0.20508712	0.66699247	1.06846572	0.45258494	0.00956213	0.04473495	0.19728397	0.92723272	0.55888507	1.27664201	0.66456963	0.62254719	0.43773956	0.18451711	0.13459855	0.5117231	0.11211129	0.11388796	0.17143098
ENSG00000121864	8.18143835	7.84118523	7.8050826	7.7370905	7.35862972	7.95983592	4.27651806	5.06047534	5.29713259	4.10383031	4.40230819	5.00306486	4.30680437	3.94258676	4.01814763	4.32256453	3.78541951	2.83022202	3.16482783	3.48131604	4.9541846	4.88131756	7.39857668	7.62452904	7.44835948	6.78810779	5.62247059	7.04518188	6.83959593	6.26741679
ENSG00000121871	10.8311674	16.294618	12.7952253	13.226869	8.08661745	12.3354831	0	0.01987365	0	0	0	0.00285573	0.05151949	0.04762403	0.02058658	0.02283485	0.00508711	0.02855296	0.0744295	0.14112109	0.01707946	0.10974878	0.10961648	0.09431673	0.07101825	0.10296828	0.07273929	0.06884417	0.08499065	0.06528409
ENSG00000121879	15.371153	14.8719957	14.8177482	11.244186	13.2805947	12.5940968	6.6182247	6.86006737	7.04709036	6.68147004	7.29076965	7.47325061	6.65678655	6.97401489	7.5757718	6.61096391	7.0571737	5.53153169	7.02495165	8.00724777	8.17673121	9.13700152	15.4336541	12.3983967	10.8564208	12.0973956	11.0572173	13.0523302	14.4629429	12.0062509
ENSG00000121892	6.75767469	7.64875703	6.52449329	6.6878347	7.44287955	6.80734665	7.33695116	7.82540778	9.05225704	10.4924215	9.08873436	11.0569292	8.49975969	7.5604032	8.46468807	13.1298581	12.3106182	8.52438002	3.85596936	3.43672457	5.8746926	8.27889987	11.6003725	12.1091175	11.0330952	11.7145028	8.74563794	9.7896572	8.97339985	7.82058854
ENSG00000121895	0.05705821	0.06005764	0.03985851	0.0842907	0.07729727	0.07948717	0.67423823	0.57485803	0.60851035	1.24767675	0.47237539	0.61952938	7.10621134	6.5433901	8.43596651	11.8892307	11.5143223	4.83748955	0.06279354	0.05066335	0.12350863	0.05569394	0.09908523	0.09093907	0.1711874	0.15428773	0.09818811	0.12906975	0.13462718	0.10491025
ENSG00000121897	0.99256868	1.14485657	1.03817711	1.897271	0.82516955	1.40860897	0.84942668	1.07358098	0.80523125	1.04477445	0.9028419	1.0809523	0.92121267	0.70993957	0.63326014	0.95011408	0.69182253	0.59170546	0.48923281	0.44576692	0.58390748	0.69716921	0.85406521	0.77316055	1.21458489	1.018701	0.77818623	0.85822724	0.87237827	0.92482563
ENSG00000121898	0.00991532	0.00332072	0	0.0192251	0.00829646	0.01487545	0.00398137	0	0	0	0	0	0.00419621	0	0.00431166	0	0.00745812	0	0.01091198	0.02641214	0	0.01270269	0.02008835	0	0	0	0	0	0	0
ENSG00000121900	5.85802549	3.58301005	5.10707275	3.4435617	3.40963523	3.40302641	4.08020167	4.27753108	4.08745963	6.61778278	4.24643422	3.59246445	2.829037	3.22287375	2.9929085	2.22226659	2.1260789	1.48313532	5.30368694	4.8877606	4.85985647	6.28268444	5.71224946	5.51198259	7.58214393	9.21160511	8.1898332	9.92113703	10.4633358	10.7944244
ENSG00000121903	1.21927322	1.29927721	1.34669239	1.3776687	1.18429373	1.32074392	1.18116375	1.27780447	1.23299626	1.00779164	1.36419991	1.22408953	1.33871976	1.05763291	1.22724352	1.24611884	1.14158344	0.92049798	0.94303506	1.08640722	0.97254332	1.02314954	1.33876468	1.01466836	1.70114084	1.57179689	1.5296138	1.56869991	1.80622476	1.64952111
ENSG00000121904	0.53535053	0.77153402	0.61531419	0.532236	0.52042182	0.67469865	0.00179385	0.00218796	0.00147245	0.00269752	0.00187607	0.00440156	0.21238312	0.17665174	0.23765292	0.19680754	0.18929917	0.15807268	0.0172078	0.01322255	0.02569791	0.11510275	0.22627582	0.20628838	0.26654442	0.3155731	0.37542025	0.32057562	0.22830845	0.21082874
ENSG00000121905	0.02697802	0.03011717	0.00879467	0.0232482	0.06019565	0.04721939	0.01444358	0.00880839	0.0044459	0.01628974	0	0	0.00761149	0	0.02346269	0.00867502	0	0	0.02968977	0.06387847	0.01513992	0.0153609	0.00910954	0.01672122	0.01716911	0	0.05416236	0.02034207	0.01291527	0.05787041
ENSG00000121931	5.29459737	4.90185335	4.97177792	4.9415172	5.19855346	4.67842097	3.56371722	3.61708521	3.50714475	3.51936303	2.63983698	3.1450553	5.38147768	4.75880704	5.43978153	5.92565292	5.8589975	4.71956771	3.347938	3.73307113	5.03484079	5.31526822	8.39509037	6.80801081	7.7699317	5.78633185	6.39711525	6.96275875	6.61492094	5.82548732
[illegible]																														

ENSG00000123374	7.11718898	4.81625413	5.56599926	[illegible]
ENSG00000123384	4.3907389	5.4659377	4.71068188	[illegible]
ENSG00000123388	0	0	0	[illegible]
ENSG00000123395	3.17751038	2.30708823	2.78374447	[illegible]
ENSG00000123405	0	0.01081563	0	[illegible]
ENSG00000123407	0	0	0	[illegible]
ENSG00000123411	0.07552445	0.08059699	0.05257941	[illegible]
ENSG00000123415	0.754123	0.77897184	0.75431872	[illegible]
ENSG00000123416	80.411573	39.5801206	57.9741474	[illegible]
ENSG00000123427	1.07972328	1.09386384	0.9899531	[illegible]
ENSG00000123444	1.4389265	1.49465585	1.44188413	[illegible]
ENSG00000123447	0	0	0	[illegible]
ENSG00000123453	0.07790928	0.11582496	0.09663614	[illegible]
ENSG00000123454	0	0	0	[illegible]
ENSG00000123472	4.98589631	5.02889896	4.93291515	[illegible]
ENSG00000123473	2.26462117	1.60712218	1.84660119	[illegible]
ENSG00000123485	9.22489898	5.78975625	7.02814737	[illegible]
ENSG00000123496	12.4718313	6.19378622	8.89797704	[illegible]
ENSG00000123500	0.0041204	0.00275991	0	[illegible]
ENSG00000123505	6.98837298	4.63173641	5.5106923	[illegible]
ENSG00000123545	7.16116353	4.92939863	6.28111683	[illegible]
ENSG00000123552	0.00329001	0.0022037	0.00241318	[illegible]
ENSG00000123560	0.73827042	3.65710937	2.01513261	[illegible]
ENSG00000123561	0	0	0	[illegible]
ENSG00000123562	11.3626433	9.14396165	10.277257	[illegible]
ENSG00000123569	0	0	0	[illegible]
ENSG00000123570	1.70263833	1.87771743	1.8249368	[illegible]
ENSG00000123572	0.0034572	0.00347353	0.00169054	[illegible]
ENSG00000123575	5.46914813	7.1217654	6.02179054	[illegible]
ENSG00000123576	0	0	0	[illegible]
ENSG00000123584	0	0	0	[illegible]
ENSG00000123594	0	0	0	[illegible]
ENSG00000123595	12.2370842	12.8159252	13.6367608	[illegible]
ENSG00000123600	1.11907797	1.17312117	1.10306711	[illegible]
ENSG00000123607	6.11445287	5.64762485	6.08619475	[illegible]
ENSG00000123609	2.42757139	1.80770466	2.17517252	[illegible]
ENSG00000123610	0.01676014	0	0	[illegible]
ENSG00000123612	0.06183848	0.07328223	0.06745503	[illegible]
ENSG00000123636	2.94115534	5.12104985	3.64909112	[illegible]
ENSG00000123643	0.51390855	0.54203613	0.42754546	[illegible]
ENSG00000123684	24.1204002	19.440644	21.2721902	[illegible]
ENSG00000123685	0.17987171	0.06571687	0.05597178	[illegible]
ENSG00000123689	0.22330074	0.07478519	0	[illegible]
ENSG00000123700	10.0145821	9.68820942	9.95442113	[illegible]
ENSG00000123728	5.5939587	5.03064515	5.09679428	[illegible]
ENSG00000123737	2.89059866	2.08050669	2.29477105	[illegible]
ENSG00000123739	3.48159428	4.05266121	3.60472696	[illegible]
ENSG00000123810	1.86696663	1.85227968	1.68857714	[illegible]
ENSG00000123815	1.60426522	1.64697111	1.66647597	[illegible]
ENSG00000123836	1.24685865	1.94994632	1.51664661	[illegible]
ENSG00000123838	0.00920061	0	0	[illegible]
ENSG00000123843	0.01415448	0.0521449	0.03460706	[illegible]
ENSG00000123870	0.26399403	0.39503991	0.32410009	[illegible]
ENSG00000123892	2.35087514	1.87307565	2.38254924	[illegible]
ENSG00000123901	0.01561771	0.03835695	0.05091279	[illegible]
ENSG00000123908	2.99072364	2.3167109	2.47941202	[illegible]
ENSG00000123933	3.52265798	6.69398406	4.86793359	[illegible]
ENSG00000123965	0.34368206	0.21315157	0.32989053	[illegible]
ENSG00000123975	134.755013	79.9776943	109.009178	[illegible]
ENSG00000123977	0.00442412	0.00889004	0.00865343	[illegible]
ENSG00000123983	33.4053818	18.9066844	23.0613397	[illegible]
ENSG00000123989	34.3367935	13.1576439	22.2271269	[illegible]
ENSG00000123992	2.7955251	2.57268945	2.55510086	[illegible]
ENSG00000123999	0.18303686	0.38823646	0.08950338	[illegible]
ENSG00000124003	0.06150714	0.02059923	0	[illegible]
ENSG00000124006	4.74157711	3.48088187	3.62272708	[illegible]
ENSG00000124019	0.0045184	0.01815897	0.01767565	[illegible]
ENSG00000124067	1.42787044	1.09600567	1.25171193	[illegible]
ENSG00000124074	2.73425643	2.63002575	2.45723852	[illegible]
ENSG00000124089	0	0	0	[illegible]
ENSG00000124091	0	0	0	[illegible]
ENSG00000124092	0.00162426	0.00217592	0.001059	[illegible]
ENSG00000124097	0	0.13577353	0.14867975	[illegible]
ENSG00000124098	11.5974228	13.4777482	15.9667425	[illegible]
ENSG00000124102	0	0.0374142	0	[illegible]
ENSG00000124103	0	0	0	[illegible]
ENSG00000124104	1.57213716	1.48569735	1.51755409	[illegible]
ENSG00000124107	0	0	0	[illegible]
ENSG00000124116	0.01423418	0.01430142	0.01392077	[illegible]
ENSG00000124120	1.13914532	1.27850506	1.01581553	[illegible]
ENSG00000124126	36.5634424	23.805847	26.7698958	[illegible]
ENSG00000124134	0	0	0	[illegible]
ENSG00000124140	0.03977247	0.03604267	0.04347285	[illegible]
ENSG00000124143	0	0.00348531	0	[illegible]
ENSG00000124145	2.62037125	1.24578043	2.09379523	[illegible]
ENSG00000124151	3.85073396	3.04886989	3.21341565	[illegible]
ENSG00000124155	4.99694364	4.3299023	4.47415274	[illegible]
ENSG00000124157	0	0	0	[illegible]
ENSG00000124159	0	0	0	[illegible]
ENSG00000124160	14.0367172	13.8651016	12.6335973	[illegible]
ENSG00000124164	2.11855827	2.00773954	1.94601181	[illegible]
ENSG00000124171	0.72778856	1.21871078	0.72403593	[illegible]
ENSG00000124172	22.5504878	16.5507928	22.2307675	[illegible]
ENSG00000124177	3.77113205	4.7100409	3.8938789	[illegible]
ENSG00000124181	4.50280407	5.19543383	3.99494525	[illegible]
ENSG00000124191	0	0.00176286	0	[illegible]
ENSG00000124193	19.7176536	18.403933	17.2910971	[illegible]
ENSG00000124194	0.07297693	0.18504989	0.16822958	[illegible]
ENSG00000124196	0.02490706	0.01668315	0	[illegible]
ENSG00000124198	4.4923942	3.97967024	3.93611533	[illegible]
ENSG00000124201	1.52796018	1.57363132	1.51652878	[illegible]
ENSG00000124203	0	0	0	[illegible]
ENSG00000124205	0	0	0	[illegible]
ENSG00000124207	21.9144863	16.0206489	17.4616295	[illegible]
ENSG00000124208	0	0	0	[illegible]
ENSG00000124209	8.87297825	9.30539853	8.46643969	[illegible]
ENSG00000124212	0.03724905	0.07173126	0.07741141	[illegible]
ENSG00000124214	4.21918015	3.8396887	3.71964537	[illegible]
ENSG00000124215	0.00441806	0.0103575	0.00432078	[illegible]
ENSG00000124216	0.0191161	0.01280426	0	[illegible]
ENSG00000124217	3.54028875	4.11910805	3.2443397	[illegible]
ENSG00000124222	0.19134002	0.29257832	0.25273342	[illegible]
ENSG00000124224	0.01518301	0.01652596	0.02227307	[illegible]
ENSG00000124225	0.04834537	0.05262155	0.06057865	[illegible]
ENSG00000124226	32.5731714	24.1360813	27.9288814	[illegible]
ENSG00000124227	0	0	0	[illegible]
ENSG00000124228	2.79441367	3.2583429	2.6201395	[illegible]
ENSG00000124232	0	0	0	[illegible]
ENSG00000124233	0	0	0	[illegible]
ENSG00000124237	0	0	0	[illegible]
ENSG00000124243	0.07497468	0.09102235	0.07332389	[illegible]
ENSG00000124249	0	0	0	[illegible]
ENSG00000124251	0	0	0	[illegible]
ENSG00000124253	0	0	0	[illegible]
ENSG00000124256	0.00375135	0.01005086	0.00366875	[illegible]
ENSG00000124257	0.82032709	1.58735225	0.90923385	[illegible]
ENSG00000124260	0.02253828	0.04528949	0.03526725	[illegible]
ENSG00000124275	0.65090226	0.70678751	0.70498178	[illegible]
ENSG00000124279	1.03391119	0.96322591	1.04737658	[illegible]
ENSG00000124299	2.71995776	3.06619849	2.75103341	[illegible]
ENSG00000124302	0	0	0.00368226	[illegible]
ENSG00000124313	0.01568951	0.01401211	0.02216364	[illegible]
ENSG00000124333	3.54034179	3.00720081	3.12372186	[illegible]
ENSG00000124334	0	0	0	[illegible]
ENSG00000124343	0	0	0	[illegible]
ENSG00000124356	2.88211829	3.02276984	2.59677279	[illegible]
ENSG00000124357	1.24931488	1.24356625	1.21305949	[illegible]
ENSG00000124370	1.76362376	1.79791743	1.59211603	[illegible]
ENSG00000124374	0.90339771	1.47151775	1.28008078	[illegible]
ENSG00000124380	9.08310801	9.12601458	10.1186891	[illegible]
ENSG00000124383	7.20408088	6.95650239	6.76542128	[illegible]
ENSG00000124391	0	0	0.01521241	[illegible]
ENSG00000124399	0	0	0	[illegible]
ENSG00000124406	0.10328929	0.269743	0.16911512	[illegible]
ENSG00000124422	20.12081	20.4561082	18.4678599	[illegible]
ENSG00000124429	0	0	0	[illegible]
ENSG00000124440	0.01695128	0.01792774	0.01745058	[illegible]
ENSG00000124444	1.0044223	0.85915566	1.00221859	[illegible]
ENSG00000124449	0	0	0	[illegible]
ENSG00000124459	3.21057792	2.95589409	3.04694364	[illegible]
ENSG00000124466	0.10922691	0.09406532	0.17549321	[illegible]
ENSG00000124467	0	0	0	[illegible]
ENSG00000124469	0.01131265	0.00757739	0.01106357	[illegible]
ENSG00000124479	7.80185687	8.81179248	9.80070093	[illegible]
ENSG00000124486	8.43804931	8.25781218	7.41376813	[illegible]
ENSG00000124490	0	0	0	[illegible]
ENSG00000124491	0	0	0	[illegible]
ENSG00000124493	0	0	0	[illegible]
ENSG00000124496	0.5937085	0.79394054	0.65167154	[illegible]
ENSG00000124507	0	0	0.00212457	[illegible]
ENSG00000124508	0.47478134	0.53356029	0.57267073	[illegible]
ENSG00000124523	0.94042216	0.94587075	0.90208565	[illegible]
ENSG00000124529	745.256308	500.45437	581.04712	[illegible]
ENSG00000124532	2.31800719	1.896888	2.12130892	[illegible]
ENSG00000124535	3.47252141	2.9803915	3.27388047	[illegible]
ENSG00000124541	12.2597524	11.4195017	11.1702012	[illegible]
ENSG00000124549	0.30894209	0.3707573	0.37767477	[illegible]
ENSG00000124557	0	0	0.01088777	[illegible]
ENSG00000124562	20.5254246	17.6968396	18.2915152	[illegible]
ENSG00000124564	0	0	0	[illegible]
ENSG00000124568	0	0	0	[illegible]
ENSG00000124570	2.74262838	2.30613549	2.8119785	[illegible]
ENSG00000124571	5.33739618	4.76267762	4.21567258	[illegible]
ENSG00000124574	0.43613327	0.5296969	0.38171991	[illegible]
ENSG00000124575	78.9359006	55.5119757	73.1412513	[illegible]
ENSG00000124578	0	0	0	[illegible]
ENSG00000124587	3.58630243	4.33228382	3.56860294	[illegible]
ENSG00000124588	1.15277163	0.82558033	1.0688749	[illegible]
ENSG00000124593	0.38425645	0.56155868	0.31106551	[illegible]
ENSG00000124596	1.77945416	1.61024973	1.63003877	[illegible]
ENSG00000124602	0.06138998	0.09457596	0.05403447	[illegible]

ENSG00000124608	4.36846767	5.14178986	4.21733394	[illegible]
ENSG00000124610	0.08253454	0	0	[illegible]
ENSG00000124613	0.45634057	0.75863631	0.57534142	[illegible]
ENSG00000124614	17.8909196	16.6701836	21.8337325	[illegible]
ENSG00000124615	0.66082818	0.55248962	0.54910222	[illegible]
ENSG00000124635	230.88142	130.608661	171.006521	[illegible]
ENSG00000124641	2.69348034	2.53906632	2.44725625	[illegible]
ENSG00000124657	0.23949913	0.16042031	0.16730418	[illegible]
ENSG00000124659	3.90741583	3.1004335	3.66180828	[illegible]
ENSG00000124664	0	0	0	[illegible]
ENSG00000124678	0.00820513	0.00274796	0.00401223	[illegible]
ENSG00000124688	6.45741081	4.56841294	5.83878679	[illegible]
ENSG00000124693	1886.60059	1218.21214	1443.58082	[illegible]
ENSG00000124701	0	0	0	[illegible]
ENSG00000124702	25.7180778	21.0878054	22.2539548	[illegible]
ENSG00000124713	0.12026022	0.16110441	0.02940308	[illegible]
ENSG00000124721	0.0005771	0.0007731	0.00056439	[illegible]
ENSG00000124731	0	0	0	[illegible]
ENSG00000124733	68.7212633	47.3191308	62.8086606	[illegible]
ENSG00000124743	0	0.0028227	0.00412135	[illegible]
ENSG00000124749	0.00134369	0.00090002	0	[illegible]
ENSG00000124762	59.8287244	26.0700636	48.4770283	[illegible]
ENSG00000124766	28.6723325	56.684764	39.3027372	[illegible]
ENSG00000124767	53.4878268	38.1437009	44.5238459	[illegible]
ENSG00000124772	2.26482918	0.73807037	1.02790165	[illegible]
ENSG00000124780	0	0	0	[illegible]
ENSG00000124782	0.64927634	0.73202283	0.70478343	[illegible]
ENSG00000124783	6.23548214	5.68259786	5.94075815	[illegible]
ENSG00000124784	2.98887034	2.44907157	2.57267811	[illegible]
ENSG00000124785	2.05721743	0.8195691	2.4458656	[illegible]
ENSG00000124786	3.03986671	2.62543694	2.70012454	[illegible]
ENSG00000124787	0.7641057	0.74372407	0.7239291	[illegible]
ENSG00000124788	2.11881455	2.35827121	2.23641326	[illegible]
ENSG00000124789	10.9613026	10.4648319	9.33744149	[illegible]
ENSG00000124795	31.978281	26.324007	28.6046343	[illegible]
ENSG00000124802	1.6170189	1.43653911	1.5730922	[illegible]
ENSG00000124812	0	0	0	[illegible]
ENSG00000124813	0.23370279	0.29955649	0.25764622	[illegible]
ENSG00000124818	0	0	0	[illegible]
ENSG00000124827	0	0	0	[illegible]
ENSG00000124831	1.28401333	0.773374	0.98911183	[illegible]
ENSG00000124835	0.05778528	0.06773462	0.09889769	[illegible]
ENSG00000124839	0.00447697	0	0.0065676	[illegible]
ENSG00000124875	0.06867132	0.0766619	0.04477288	[illegible]
ENSG00000124882	0.05448396	0.07962375	0.04844031	[illegible]
ENSG00000124900	0.02179158	0.01459634	0	[illegible]
ENSG00000124915	0	0	0	[illegible]
ENSG00000124920	0.18558391	0.17771304	0.17074231	[illegible]
ENSG00000124935	0	0.08994996	0	[illegible]
ENSG00000124939	0	0	0	[illegible]
ENSG00000124942	1.96129991	1.12355554	1.72857365	[illegible]
ENSG00000125037	8.49412908	7.22003085	7.79987457	[illegible]
ENSG00000125046	0.02433351	0.01901547	0.01322097	[illegible]
ENSG00000125084	0	0	0	[illegible]
ENSG00000125089	0.01193488	0.00639534	0.0116721	[illegible]
ENSG00000125107	5.94532585	6.32268714	5.31988395	[illegible]
ENSG00000125122	0.08423535	0.08897342	0.11089704	[illegible]
ENSG00000125124	2.28538594	3.16109284	2.51504036	[illegible]
ENSG00000125144	0	0	0	[illegible]
ENSG00000125148	7.39354433	4.5108279	5.96023356	[illegible]
ENSG00000125149	1.01380292	1.00063184	0.93404008	[illegible]
ENSG00000125166	7.84245536	5.69587087	6.3765329	[illegible]
ENSG00000125170	0.8399411	0.40309782	0.58313732	[illegible]
ENSG00000125207	0.00378239	0.00506701	0.00369911	[illegible]
ENSG00000125245	0.19105109	0.03490065	0.09625324	[illegible]
ENSG00000125246	0.11915308	0.13771244	0.12566915	[illegible]
ENSG00000125247	2.25836488	2.29133454	2.04676017	[illegible]
ENSG00000125249	20.1700672	22.9934059	22.1134019	[illegible]
ENSG00000125255	0.00853316	0.01714693	0	[illegible]
ENSG00000125257	0.7215289	0.66476842	0.66171678	[illegible]
ENSG00000125266	45.4583104	25.9336565	33.7952634	[illegible]
ENSG00000125285	68.4367118	94.1801953	74.2937977	[illegible]
ENSG00000125304	36.5986911	33.3309599	31.9091595	[illegible]
ENSG00000125319	1.18271218	0.68731663	0.70703568	[illegible]
ENSG00000125337	0	0	0	[illegible]
ENSG00000125347	0.25458554	0.17316923	0.33004337	[illegible]
ENSG00000125351	1.99480902	2.44698153	2.45303888	[illegible]
ENSG00000125352	8.3125352	7.86836413	8.59196729	[illegible]
ENSG00000125354	0.81389549	0.5110876	0.78832157	[illegible]
ENSG00000125355	1.61934114	1.41114159	1.62661101	[illegible]
ENSG00000125356	101.065059	72.908788	104.230326	[illegible]
ENSG00000125363	0	0	0	[illegible]
ENSG00000125375	0.70145236	0.77352351	0.83101759	[illegible]
ENSG00000125378	0	0.00419632	0	[illegible]
ENSG00000125384	0	0	0	[illegible]
ENSG00000125385	0	0	0	[illegible]
ENSG00000125386	1.33696525	1.68593299	1.37154513	[illegible]
ENSG00000125388	0.32025323	0.40011568	0.37761932	[illegible]
ENSG00000125398	72.6827727	104.807363	75.1676444	[illegible]
ENSG00000125409	0.00327205	0	0.00320001	[illegible]
ENSG00000125414	0	0	0	[illegible]
ENSG00000125430	1.89238839	1.29924053	1.68492802	[illegible]
ENSG00000125434	0.06488242	0.05472649	0.0446527	[illegible]
ENSG00000125445	6.73886863	5.05250843	6.18065658	[illegible]
ENSG00000125447	0.59883531	0.81690585	0.62410196	[illegible]
ENSG00000125449	0.91788376	0.83438919	0.82286768	[illegible]
ENSG00000125450	1.55268734	1.1748459	1.25601506	[illegible]
ENSG00000125454	0.15363753	0.1372118	0.1022567	[illegible]
ENSG00000125457	0.71503065	0.69696327	0.81670475	[illegible]
ENSG00000125458	1.61926311	1.60546509	1.69693828	[illegible]
ENSG00000125459	0.41510001	0.56139401	0.43311057	[illegible]
ENSG00000125462	48.9709338	64.7848319	57.7620449	[illegible]
ENSG00000125482	5.20750268	4.73592857	5.16225253	[illegible]
ENSG00000125484	6.36275455	6.37114024	5.69531563	[illegible]
ENSG00000125485	0.6685601	0.56263578	0.55324904	[illegible]
ENSG00000125492	0	0	0	[illegible]
ENSG00000125498	0	0	0	[illegible]
ENSG00000125503	1.37117753	1.76585975	1.43603117	[illegible]
ENSG00000125505	1.87836055	1.60019972	1.54276433	[illegible]
ENSG00000125508	0.08503889	0	0	[illegible]
ENSG00000125510	0.32436152	0.2238462	0.28517734	[illegible]
ENSG00000125514	0	0	0	[illegible]
ENSG00000125520	7.37738368	5.51637244	5.86185918	[illegible]
ENSG00000125522	0	0	0	[illegible]
ENSG00000125531	0.52859059	0.39233493	0.41915035	[illegible]
ENSG00000125533	0	0	0	[illegible]
ENSG00000125534	26.5833531	18.0820126	20.2206993	[illegible]
ENSG00000125538	0.50187873	0.14057865	0.00446208	[illegible]
ENSG00000125551	0.00854447	0.00572322	0.00835634	[illegible]
ENSG00000125571	0	0	0.00934206	[illegible]
ENSG00000125611	2.33629456	2.01199774	2.00740755	[illegible]
ENSG00000125618	0.21237622	0.21117054	0.19737962	[illegible]
ENSG00000125629	3.17968695	2.31741514	3.24041389	[illegible]
ENSG00000125630	1.12199824	1.18525877	1.08683465	[illegible]
ENSG00000125631	0	0	0.01342996	[illegible]
ENSG00000125633	4.31134935	5.05614441	4.32129036	[illegible]
ENSG00000125637	0.00384798	0.00451052	0.00094081	[illegible]
ENSG00000125648	5.55859684	6.46905239	5.87341427	[illegible]
ENSG00000125650	0.71343787	0.77346292	0.62579856	[illegible]
ENSG00000125651	9.10804924	10.1444936	9.00346019	[illegible]
ENSG00000125652	11.6191834	10.0471212	10.9327122	[illegible]
ENSG00000125656	7.11822474	4.88428788	5.69576941	[illegible]
ENSG00000125657	0	0	0.03804479	[illegible]
ENSG00000125675	0.30076278	0.24879776	0.33384958	[illegible]
ENSG00000125676	2.13924107	2.69499481	2.25299992	[illegible]
ENSG00000125686	4.81704859	5.42070004	4.45170694	[illegible]
ENSG00000125691	22.0484424	25.4839269	24.6636865	[illegible]
ENSG00000125695	0	0	0	[illegible]
ENSG00000125703	1.55606548	1.27643278	1.37170834	[illegible]
ENSG00000125726	0.02724407	0.01824851	0.01332211	[illegible]
ENSG00000125730	0.02661923	0.02880227	0.01401784	[illegible]
ENSG00000125731	0.0208708	0.04019134	0.03316832	[illegible]
ENSG00000125733	1.42353956	1.06489493	1.18846011	[illegible]
ENSG00000125734	1.37653989	1.64249048	1.48145098	[illegible]
ENSG00000125735	0.01920151	0	0	[illegible]
ENSG00000125740	0.01428442	0.01776902	0.01197421	[illegible]
ENSG00000125741	1.92103492	1.90414386	1.68215822	[illegible]
ENSG00000125743	23.4559744	20.5619541	23.2137259	[illegible]
ENSG00000125744	0.05508348	0.06852075	0.06733832	[illegible]
ENSG00000125746	0.01765809	0.01743025	0.02908514	[illegible]
ENSG00000125753	2.31201151	1.80923148	2.10743835	[illegible]
ENSG00000125755	1.53689839	1.74208266	1.46741335	[illegible]
ENSG00000125772	2.28156041	2.74309215	2.23484454	[illegible]
ENSG00000125775	0.1932298	0.10951632	0.15021112	[illegible]
ENSG00000125779	1.84696944	1.79062539	1.79427696	[illegible]
ENSG00000125780	0.01019928	0.01366329	0	[illegible]
ENSG00000125787	0.04133784	0.03691833	0.02695178	[illegible]
ENSG00000125788	0	0	0	[illegible]
ENSG00000125798	0.00668251	0	0	[illegible]
ENSG00000125804	0.36963446	0.61763643	0.43534985	[illegible]
ENSG00000125810	0.00480467	0.01930947	0.01879553	[illegible]
ENSG00000125812	1.85787883	1.79644363	1.65436373	[illegible]
ENSG00000125813	0	0	0	[illegible]
ENSG00000125814	0.67439618	0.69454374	0.6804026	[illegible]
ENSG00000125815	0	0	0	[illegible]
ENSG00000125816	0	0	0	[illegible]
ENSG00000125817	34.2043788	26.9849882	30.9651752	[illegible]
ENSG00000125818	5.26441383	5.51620324	4.95054738	[illegible]
ENSG00000125820	1.81792987	1.62013122	1.47656331	[illegible]
ENSG00000125821	15.3926707	12.8176905	13.8334066	[illegible]
ENSG00000125823	0.04314557	0.05779917	0.0140652	[illegible]
ENSG00000125826	1.48215971	1.6958993	1.53442187	[illegible]
ENSG00000125827	14.849867	11.9576124	12.8220565	[illegible]
ENSG00000125831	0	0	0	[illegible]
ENSG00000125834	2.3270497	1.78971318	1.97223883	[illegible]
ENSG00000125835	24.1810686	15.1872007	18.6775659	[illegible]
ENSG00000125841	7.86091214	5.06014938	6.54845492	[illegible]
ENSG00000125843	0.92161074	0.90277051	0.91173862	[illegible]
ENSG00000125844	3.32618331	2.22130695	2.59172037	[illegible]
ENSG00000125845	0.01790044	0.07793498	0.04376577	[illegible]
ENSG00000125846	0.68201676	0.83817573	0.85550018	[illegible]

ENSG00000125848	7.69259594	8.43324108	8.1493142	6.73138	6.14192798	6.60175932	0.43119727	0.43490367	0.40669065	0.44890364
ENSG00000125850	0	0	0	0	0	0	0	0	0	0
ENSG00000125851	0.05549492	0.13295915	0.20248016	0.002759	0	0	0.0017141	0.00418136	0	0.00773277
ENSG00000125861	0	0	0	0	0	0	0	0	0	0
ENSG00000125863	4.29771752	3.61097493	3.95422334	4.1339892	4.91037868	3.82711365	2.67432169	3.24647603	3.55989567	2.89379635
ENSG00000125864	0.01987445	0.01774963	0.04211319	0.0342534	0.04619331	0.01739304	0.68630897	0.4607234	0.36355307	0.74703087
ENSG00000125868	13.3651818	10.8095014	12.3413921	11.637068	16.1361117	11.862416	37.9381649	36.1850038	32.1072861	33.911189
ENSG00000125869	1.13593468	1.46370187	1.37453273	3.5588294	2.49700486	2.15911663	0	0	0	0
ENSG00000125870	13.4340149	11.2806215	12.9055433	9.8742345	13.7457057	9.9815797	20.5602836	21.8729464	24.0334873	17.6855766
ENSG00000125871	3.96087921	2.88465273	3.29154834	3.2360024	3.25682133	3.01055052	2.78019664	3.58391143	3.58106254	2.6717644
ENSG00000125872	0	0	0	0	0	0	0.00961474	0.01172708	0.02367623	0.01084369
ENSG00000125875	4.50862983	4.24798189	3.80355495	4.156666	4.90595024	4.01509501	3.10160687	3.41041957	2.95148345	2.74497122
ENSG00000125877	2.5023697	1.74945744	2.00752832	1.5414915	2.31628024	1.46061046	4.97059554	5.38857049	4.79588932	3.31822883
ENSG00000125878	0	0	0	0	0.03468508	0.01554748	0	0	0	0
ENSG00000125879	0	0	0.0203684	0	0.00580886	0	0	0	0	0.00943173
ENSG00000125885	4.03911225	2.83436868	3.0346671	2.109908	4.2172783	2.3151531	2.86972875	4.00986961	5.60659678	2.8986771
ENSG00000125888	0	0	0	0	0	0	0	0	0	0.01174224
ENSG00000125895	0.06846986	0.07206916	0.14349062	0.0885053	0.09275672	0.17609466	0.01571041	0	0.00483584	0.07973319
ENSG00000125898	0.81001949	0.61822022	0.72590613	0.6882964	0.73627515	0.64876903	0.25139087	0.32874796	0.36536599	0.21921903
ENSG00000125899	0	0	0	0	0	0	0	0	0	0
[illegible]	[illegible]	[illegible]	[illegible]	[illegible]	[illegible]	[illegible]	[illegible]	[illegible]	[illegible]	[illegible]

ENSG0000127419	0.39170155	0.54967573	0.48503676	0.4678166	0.45693804	0.45135107	0.97359864	0.8898921	0.55224488	0.89839849	0.6886125	0.57982873	0.41726148	0.34367158	0.37433905	0.29022361	0.27782664	0.22995577	0.54908696	0.56335854	0.595522	0.7136088	0.51447167	0.56079446	0.6397953	0.51234114	0.48567918	0.49861755	0.5411714	0.42650815	0.87346916	0.82727678	0.89914855	0.81338188	0.9601088	0.94063879	0.92341292	0.94849915	0.9961178	0.51196271	0.56758532	0.54672858	0.20633355	0.32043061	0.24728902	0.52982092	0.24800812	0.31886554
ENSG0000127423	1.91088669	1.00960958	1.36938874	0.8197991	2.04916484	1.12448044	1.1178658	1.93627111	2.71608698	0.88028682	1.12067582	2.51799454	2.32848893	1.42768174	1.88395468	2.55849547	1.74710384	0.9041139	0.98803022	0.31447864	3.06460059	1.51245758	1.15141951	3.19324438	2.70479516	2.56903233	0.90712608	1.63338192	1.10013168	0.8414503	0.98863225	0.35270404	1.14855015	1.11483572	1.31538752	1.47267869	1.5548866	2.02514956	1.78372743	3.0165242	3.69283271	2.31560765	1.44285709	0.64694698	0.70658219	2.88480809	0.82146626	0.39272603
ENSG0000127445	5.84084894	3.84011067	5.02192788	4.9903692	5.77840697	4.35105217	2.22199538	2.66793856	2.59330463	2.21075256	2.02967814	2.01353521	2.44907304	2.44290526	2.5750291	2.28188126	2.18453052	1.86211338	1.62181309	1.13628179	2.31324445	2.05938489	1.60199938	2.85041509	2.24522529	2.74042913	1.66437765	3.4229586	3.2250546	2.97986914	4.61728334	3.22696927	4.10597686	3.82462367	3.89326095	5.43052223	2.62522584	2.93197846	2.8900768	3.20671172	2.94927829	2.63407242	3.41467404	2.48643037	2.19188654	3.31554176	2.38946564	1.87144933
ENSG0000127452	1.36003591	1.14020553	1.10406221	1.2380723	1.19500871	1.18022762	1.60262274	1.53242625	1.49090403	1.46529671	1.17026584	1.03782313	1.02136164	0.99910589	1.03206716	0.73102993	0.87589672	0.76933529	1.08098085	1.38519784	1.29265211	0.98696122	0.7789016	0.9793241	1.11374878	0.97295777	0.75145372	1.30868472	1.44900397	1.46227836	1.95833811	1.73738174	1.96504185	1.73478678	2.02552551	1.72970483	1.69041124	1.53394521	1.47512032	1.09587834	0.95898624	1.07045793	1.17296435	1.34138139	1.22583756	1.24229048	1.21504421	1.37540153
ENSG0000127463	1.83098611	1.71869307	1.47135203	1.4674828	1.5844964	1.45482047	3.9155577	4.50118022	3.64371096	3.03458969	2.82416599	2.84894951	3.33780486	2.89475058	3.63633649	3.13750935	2.60693805	1.63384564	2.23215205	2.813095	4.68705555	3.70701809	4.44235785	4.56411772	5.99040477	5.25828938	4.40362567	5.4093351	5.6130925	4.89841197	4.3420504	5.07884225	4.44038082	4.38158176	4.15243652	4.53007556	4.30370529	4.05978171	3.57584511	5.97671927	5.22015867	4.98521413	3.28789512	3.24364835	3.0916267	3.61937864	2.73489672	2.3654802
ENSG0000127472	0.66213034	0.18520005	0.2490583	0.2398347	0.81378851	0.27290134	0	0	0	0	0.00458333	0	0	0	0	0	0	0	0	0.00323034	0	0	0	0	0	0	0	0	0	0	0.00552755	0	0	0	0.00708541	0.01618106	0	0	0	0.00584382	0	0	0	0	0	0	0	0
ENSG0000127481	1.20409111	1.254538	1.1406824	0.9641921	1.2746704	1.20013444	2.7964226	2.71616627	2.74314974	3.41477227	3.13820661	2.73276143	2.88453794	3.26837166	3.42409602	3.03806522	3.13409938	3.1998891	1.75932396	2.397193	2.44301441	1.91095633	2.6555071	2.05908071	3.47153256	2.79833649	2.73338842	2.47952433	2.66101453	2.26714422	2.65235961	3.26125138	3.10726412	2.11245245	2.24106159	2.14074995	4.38035237	3.87615454	3.45977366	3.01982221	3.38301341	3.05444162	4.06557183	4.17396528	4.25547512	2.93217715	4.54142038	5.06426325
ENSG0000127483	20.6252104	23.3609627	20.2482312	18.847695	17.5432994	18.5523676	8.00728235	9.12772375	9.49061182	9.28637906	8.97054351	9.76300709	12.274769	12.6020266	14.0058997	12.4426005	12.9612152	10.6252807	8.03105748	5.81503259	7.44744849	11.8934149	14.4615504	10.580281	7.59888779	10.3262978	11.1024058	17.1142266	18.3418728	16.8689264	12.8214269	12.4156235	12.2766475	11.0742006	12.1128424	13.3446387	7.62735167	9.09167778	8.91846251	11.8458729	11.0259013	10.976997	12.1325135	9.13370171	8.73811332	10.0881925	9.45010678	14.461309
ENSG0000127507	0.00391423	0.00861452	0.00492177	0.0028912	0.00343112	0.00671122	0.03682288	0.03176756	0.02736869	0.01063561	0.0528346	0.05761121	0.12021603	0.10298199	0.09677624	0.05070579	0.10599151	0.07554413	0.12123022	0.06802119	0.07719629	0.10697781	0.1484078	0.10709378	0.05604878	0.06028407	0.08852714	0.06240145	0.08633187	0.07466787	0.00254877	0.00342069	0.00550406	0.00440031	0.0032671	0.00646631	0.12367087	0.1284704	0.11827972	0.05748478	0.06789889	0.04706132	0.04481577	0.06206191	0.0660223	0.0552981	0.06323774	0.07528058
ENSG0000127511	1.61206221	1.72033514	1.502029	1.7305958	1.77602423	1.72379861	1.86857578	2.24669876	2.32840422	2.16081613	1.80444986	1.68078257	2.1982379	1.64047277	1.91227973	1.57037887	1.49704527	1.57852833	1.45283283	1.54375447	1.58944525	1.37805589	1.14500209	1.42121113	1.31376325	1.22134762	1.22124858	1.66227696	1.81956203	1.77451365	4.95322932	3.85842674	4.49658874	3.61674954	3.90139761	3.52805577	1.71268043	1.96352215	1.86132578	2.0627788	2.06318821	2.10643402	1.75516652	2.47637262	2.19138668	2.04035766	2.15006524	2.73867045
ENSG0000127515	0	0	0	0	0	0	0	0	0	0	0	0	0	0	0	0	0	0	0	0	0	0	0	0	0	0	0	0	0	0	0	0	0	0	0	0	0	0	0	0	0	0	0	0	0	0	0	0
ENSG0000127526	2.90882834	2.82567378	2.76066197	3.5502691	3.28119797	2.88878322	8.03944279	8.39064808	7.90278282	6.67854855	7.20361222	6.0372531	3.60870671	4.34355467	4.07181868	3.60188927	3.38781975	3.66674204	3.46152909	3.73710184	4.286744	3.46108491	4.12884679	3.84028811	3.96368068	3.34145076	3.18402529	4.78168597	5.29647275	5.24117032	7.05109565	6.66811947	6.69942745	5.72875583	5.15497707	5.05678579	8.69763589	8.93331764	7.9549894	6.00068546	6.45287351	6.36395957	5.12101582	6.5039842	5.91094474	4.2986916	5.83155967	6.27972866
ENSG0000127527	1.32260726	1.3129975	1.25644119	1.1370294	1.75317768	1.23916415	1.0444078	1.2553097	1.3129732	0.92631166	1.05250804	1.24400728	0.91552351	1.0042365	0.85743847	0.98051576	0.97822049	0.77802025	0.70635372	0.53533543	1.01150117	0.75307362	0.62992762	0.97628207	0.8125931	0.88150912	0.58846375	0.83146113	0.84020486	0.778684	1.19899568	1.1109688	1.06331129	0.78991339	1.07174194	1.0379876	1.15186603	1.19916392	1.21987134	1.05633253	1.15577791	1.21527261	0.84449791	0.81387504	0.78907622	0.98273027	0.96225643	0.89911322
ENSG0000127528	0.41192154	0.33443827	0.39064423	0.306566	0.50133514	0.30899259	1.61392166	1.47942824	1.93159373	2.45332756	2.9878821	2.13991174	3.48654489	0.81136502	1.37870717	4.03391923	3.81196807	0.88835669	0.57696123	0.35467149	1.02974881	0.57569454	1.01157502	1.07927635	0.45280701	0.35954397	0.6873711	0.64002143	0.80672933	0.85348774	0.39827274	0.29016806	0.2252565	0.31433013	0.21879429	0.28869476	2.62209258	3.45902403	2.71579755	3.24818376	1.74889311	2.30226949	1.41488353	1.89694384	2.29132359	4.32046905	2.27865816	1.69215883
ENSG0000127529	0	0	0	0	0	0	0	0	0	0	0	0	0	0	0	0	0	0	0	0	0	0	0	0	0	0	0	0	0	0	0	0	0	0	0	0	0	0	0	0	0	0	0	0	0	0	0	0
ENSG0000127530	0.06046855	0	0	0.0781627	0.01686532	0.02267948	0	0	0	0	0	0	0	0	0	0.02916631	0	0.03646989	0	0	0	0.02582245	0	0.02810922	0	0.04976374	0.05202838	0	0	0	0	0	0	0.04758445	0	0	0	0	0	0	0	0	0	0.07743811	0.02788896	0	0	0
[illegible]																																																

ENSG00000129028	3.87503343	2.73526311	3.11336203	2.525751	4.20680683	2.11625227	1.25182928	[illegible]
ENSG00000129038	0.01331074	0.01188766	0.03471377	0	0.00742501	0	16.7148471	[illegible]
ENSG00000129048	0.02757035	0.01846706	0	0.0178189	0.00768966	0.0103406	0	[illegible]
ENSG00000129055	17.3967622	16.1070849	16.2905427	18.098038	12.3148888	15.2137535	9.89996226	[illegible]
ENSG00000129071	5.6899634	5.24527144	5.21121302	5.1487839	5.99514514	5.50514657	2.39257017	[illegible]
ENSG00000129083	9.68497307	9.40739299	9.52482496	13.150551	9.86483339	10.5654136	11.5803861	[illegible]
ENSG00000129084	2.53898042	2.15374762	2.32662774	2.0560057	2.75993135	2.25940595	3.11813335	[illegible]
ENSG00000129103	6.31545217	5.30731324	5.84006025	7.204086	5.63617492	6.39943666	4.30981274	[illegible]
ENSG00000129116	1.79661295	1.88928705	1.70196736	1.7920101	2.48882394	1.89949666	2.12607546	[illegible]
ENSG00000129128	17.7102284	16.7487196	15.4844947	11.23779	19.1274246	13.0662376	17.899239	[illegible]
ENSG00000129151	0.88608929	2.81213842	1.55777965	1.486259	0.67080785	1.3214424	0.00564759	[illegible]
ENSG00000129152	0	0.02397334	0	0	0.02994742	0.01342384	0	[illegible]
ENSG00000129158	0.63603215	0.47558958	0.58756663	0.6763914	0.65258191	0.75067813	0.36504599	[illegible]
ENSG00000129159	0.17305222	0.22520263	0.14022905	0.1629743	0.12273381	0.17060845	0.00198534	[illegible]
ENSG00000129167	0.17006824	0.08240616	0.19817289	0.0654822	0.15340306	0.12485811	0.13367161	[illegible]
ENSG00000129170	0	0	0.01126926	0	0	0	0.02776142	[illegible]
ENSG00000129173	2.50136352	1.48806675	1.77034043	0.7976902	2.21689663	0.92582318	0.24779384	[illegible]
ENSG00000129187	2.83612217	2.63623596	2.70404449	3.8628695	2.70404665	3.29798602	5.02060774	[illegible]
ENSG00000129194	0.01821404	0	0.0089065	0	0.00508009	0.03415701	0.08044979	[illegible]
ENSG00000129195	12.2738961	6.95036324	9.20311019	8.9858841	11.1317944	7.83631691	0.78107077	[illegible]
ENSG00000129197	1.54432494	1.82189638	1.56407484	1.9234415	1.46642172	1.91866053	1.6288502	[illegible]
ENSG00000129204	0.058786	0.06115815	0.04709423	0.0501196	0.06977039	0.06051588	0.0512274	[illegible]
ENSG00000129214	0.01292513	0.01484136	0.01805792	0.0238675	0.01235985	0.02077599	0.02669104	[illegible]
ENSG00000129219	1.13154767	1.26731119	1.06077282	1.1938448	0.69147827	1.27243839	1.05607703	[illegible]
ENSG00000129221	0.00865553	0.00695714	0.00677196	0.0134259	0.00482824	0.00649273	0.00834125	[illegible]
ENSG00000129226	0.00629487	0	0.01231254	0.0081369	0.00351141	0	0.04044202	[illegible]
ENSG00000129235	2.94328487	1.92501087	2.5469271	2.2408936	4.10671639	2.54883128	3.87346182	[illegible]
ENSG00000129244	16.4679696	17.9748956	16.8033762	16.470059	18.431039	17.7518502	0.03619985	[illegible]
ENSG00000129245	10.9544201	8.49018481	8.67888403	9.6849365	10.3540871	8.66995127	10.8603191	[illegible]
ENSG00000129250	8.8363578	7.03535272	7.85646668	5.8791663	9.6808336	5.77783913	11.1435216	[illegible]
ENSG00000129255	2.30751995	1.70981697	1.94169321	1.6304657	2.50201809	1.43157056	1.62419072	[illegible]
ENSG00000129270	0	0	0	0	0	0	0.08295799	[illegible]
ENSG00000129277	0	0	0	0	0	0	0	[illegible]
ENSG00000129282	0.83113942	0.47423475	0.6422435	0.875392	0.84712142	0.59267028	1.40085697	[illegible]
ENSG00000129292	1.68887115	1.68222272	1.61658075	1.3223656	2.1841898	1.40149382	1.61284634	[illegible]
ENSG00000129295	0.21510762	0.30368156	0.30422939	0.166831	0.18275645	0.17376969	0.28968429	[illegible]
ENSG00000129315	6.4254582	6.03228543	6.00845183	5.1770675	5.43085536	5.97023491	9.99629349	[illegible]
ENSG00000129317	0.93022363	1.085863	0.87765566	0.8955804	0.84294099	0.95836707	1.61186876	[illegible]
ENSG00000129347	1.39565641	1.52124739	1.31327256	1.5247523	1.43206087	1.43350734	2.13366449	[illegible]
ENSG00000129351	6.34877464	6.25187447	5.67543431	5.3261092	6.96195748	5.9745821	5.96720603	[illegible]
ENSG00000129353	3.95904973	3.27369831	3.25885603	3.7359145	2.89208622	3.677295	1.61938744	[illegible]
ENSG00000129354	0.00377973	0	0.01108952	0	0.00632524	0	0.54940818	[illegible]
ENSG00000129355	6.32258053	3.58078312	5.03969807	4.9833376	5.77775862	4.7427249	1.23841592	[illegible]
ENSG00000129422	0.85520673	1.55519734	1.33052576	1.6442475	0.80392755	1.42235656	0.01864602	[illegible]
ENSG00000129437	0.01469664	0.01968809	0	0.0189971	0.0081981	0.01102432	0.0118025	[illegible]
ENSG00000129450	0	0	0	0	0	0	0.15782252	[illegible]
ENSG00000129451	0.00951759	0.04037522	0.02171875	0.0328069	0.01061823	0.02141815	0.01528668	[illegible]
ENSG00000129455	0	0	0	0	0	0	0	[illegible]
ENSG00000129460	2.27152416	2.29393254	2.40663565	2.3116217	1.9548422	2.32608424	1.93021572	[illegible]
ENSG00000129465	0	0	0	0	0	0	0	[illegible]
ENSG00000129467	0.00356031	0.00715426	0.0043524	0.0034516	0.00297903	0.00734437	0.06790596	[illegible]
ENSG00000129472	7.32805124	6.87932299	6.60699398	7.9284795	6.27041731	7.05084611	3.23723498	[illegible]
ENSG00000129473	3.82395182	3.15165319	3.53523828	3.7586668	3.8134391	4.06736022	2.49112345	[illegible]
ENSG00000129474	1.9248605	2.18938088	2.02708992	1.9448815	1.32696595	1.93037015	3.02703179	[illegible]
ENSG00000129480	5.05628509	6.96584149	5.76167131	3.9805367	4.20346001	3.85157312	2.55685255	[illegible]
ENSG00000129484	4.31978519	3.11740651	3.48101425	2.7460189	3.70362693	2.96960697	0.97182623	[illegible]
ENSG00000129493	0.72937909	1.58928577	0.9968962	0.8902811	0.56015724	0.8886267	0.3180396	[illegible]
ENSG00000129514	0	0	0	0	0	0	0	[illegible]
ENSG00000129515	5.92056928	5.51346576	6.17435059	6.2735009	4.9069018	5.83100697	3.83758006	[illegible]
ENSG00000129518	6.52334356	7.07256638	7.11860613	6.5861545	5.04711926	6.30326255	6.43742613	[illegible]
ENSG00000129521	0.97742041	0.40216775	0.8903522	0.8735716	1.30231104	1.82458911	0.00448538	[illegible]
ENSG00000129534	6.68464962	6.03939739	6.17945143	4.9426304	6.3051801	5.84189194	2.15254491	[illegible]
ENSG00000129535	0.1503594	0.17148448	0.14704879	0.2258744	0.15641306	0.12193332	0.02937158	[illegible]
ENSG00000129538	0.4395609	0.24693698	0.28427725	0.1557923	0.58135283	0.26058956	0	[illegible]
ENSG00000129559	7.84536565	6.471582	7.49920753	8.05337	7.74476226	6.72623231	6.088734	[illegible]
ENSG00000129562	30.7191642	23.3047459	26.7733215	36.955268	30.9148963	23.5633299	21.3088893	[illegible]
ENSG00000129566	0.20071965	0.24743639	0.20674172	0.2980956	0.20249115	0.31340972	0.42527489	[illegible]
ENSG00000129595	0.0646734	0.14995131	0.15569089	0.073952	0.04301393	0.05411079	0.0059928	[illegible]
ENSG00000129596	13.193406	29.1958443	17.3534579	27.281587	9.26082663	23.7197746	1.04524174	[illegible]
ENSG00000129625	4.44963979	4.62910476	4.01599363	5.2400634	4.98663107	4.67150795	5.10307439	[illegible]
ENSG00000129636	4.4109333	4.0887572	4.01568231	3.7170025	4.30719298	3.83162542	2.127177	[illegible]
ENSG00000129646	1.09472938	1.32986014	1.14031136	0.8038242	0.69919147	0.82361327	0.28091171	[illegible]
ENSG00000129654	1.57426597	3.95629967	1.51573402	3.4388614	0.8236996	2.48079715	0.00980041	[illegible]
ENSG00000129657	7.24485801	5.95692696	6.00983182	4.5776063	7.24564823	4.92248636	1.24313405	[illegible]
ENSG00000129667	0.01920342	0.01543531	0.02253672	0.0198581	0.02071001	0.01344463	0.43180993	[illegible]
ENSG00000129673	0	0.00514981	0.00751911	0.0099381	0.03859874	0.00576725	0.01234871	[illegible]
ENSG00000129675	6.96577105	11.3853659	9.03071127	8.1349977	6.96397878	8.66132661	1.18359956	[illegible]
ENSG00000129680	1.71894296	1.94493849	1.9466222	1.1576848	1.54991596	1.1545285	2.50036587	[illegible]
ENSG00000129682	1.86485778	2.18798581	2.1009431	1.9300127	1.57908349	1.83088209	0.03425929	[illegible]
ENSG00000129691	1.64889094	1.47086454	1.55619095	1.4565129	1.51182294	1.22752156	1.2591001	[illegible]
ENSG00000129696	2.09614908	1.56951854	1.71776842	1.8163248	1.78944538	1.55789498	2.40259214	[illegible]
ENSG00000129744	0	0	0	0	0	0	0	[illegible]
ENSG00000129749	0.03241885	0.08685864	0.01585253	0.0419051	0.07836367	0.01621212	0.16922595	[illegible]
ENSG00000129757	0.0862588	0.12479943	0.12822653	0.1828593	0.0288702	0.11646877	0.03325069	[illegible]
ENSG00000129810	2.69166996	1.9070599	2.25955798	1.5465487	2.37475217	1.73108197	0.28770833	[illegible]
ENSG00000129816	0	0	0	0	0	0	0	[illegible]
ENSG00000129824	0	0	0	0	0	0	0	[illegible]
ENSG00000129845	0	0	0	0	0	0	0	[illegible]
ENSG00000129862	0	0	0	0	0	0	0	[illegible]
ENSG00000129864	0	0	0	0	0	0	0	[illegible]
ENSG00000129873	0	0	0	0	0	0	0	[illegible]
ENSG00000129910	0.01702275	0.02660492	0.00554931	0.0146693	0.0189913	0.01276919	0.01367054	[illegible]
ENSG00000129911	2.4480085	1.24885262	1.60767159	1.5361752	2.58819599	1.51068695	3.57182776	[illegible]
ENSG00000129925	4.61530369	3.14738472	3.61458003	2.8408662	4.44971447	2.61221384	6.32157088	[illegible]
ENSG00000129932	1.22542751	0.67066246	0.86716831	0.9980539	1.24765337	0.97153719	1.34415022	[illegible]
ENSG00000129933	1.0686404	1.3872802	1.0845302	1.4040532	0.93509769	1.30397903	2.2799521	[illegible]
ENSG00000129946	2.80607901	4.29914848	3.50192229	4.1464137	2.24070231	3.66960221	0.13594682	[illegible]
ENSG00000129951	0.11689543	0.24235223	0.21775549	0.1942727	0.0962576	0.22130319	0.19222183	[illegible]
ENSG00000129965	0	0	0	0	0	0	0	[illegible]
ENSG00000129968	1.24328991	1.23172626	1.14463039	1.2948306	1.3127185	1.06384597	2.60066542	[illegible]
ENSG00000129988	0.01643536	0	0	0	0.00916799	0	0.09239176	[illegible]
ENSG00000129990	0.00869136	0.02183104	0.02124999	0.0112346	0.02545319	0.01629902	0.03489907	[illegible]
ENSG00000129991	0.00575942	0.00385775	0	0.0446684	0	0.01728115	0.03237674	[illegible]
ENSG00000129993	0.00332849	0.00074316	0.00108507	0.0028683	0	0.00083226	0	[illegible]
ENSG00000130005	6.8256695	4.94643514	5.9463302	6.9258847	6.57541606	6.15111525	8.63386697	[illegible]
ENSG00000130021	6.82470589	4.84332674	6.02685507	4.5021633	6.3556144	4.17895188	2.5099422	[illegible]
ENSG00000130023	0.42832005	0.50896953	0.43641607	0.5513371	0.42186942	0.49202272	1.08229177	[illegible]
ENSG00000130024	7.91614482	7.5371301	6.76220452	7.8142156	8.53154904	7.2508522	3.47755396	[illegible]
ENSG00000130032	0.00745369	0.01997039	0.0255135	0.0192695	0.0166313	0.01118239	0.02394347	[illegible]
ENSG00000130035	0.26222421	0.28209159	0.25645057	0.1437993	0.10194872	0.13113394	0	[illegible]
ENSG00000130037	0	0	0	0	0	0	0.00903417	[illegible]
ENSG00000130038	0.0368112	0.045204	0.04800092	0.0297392	0.04534607	0.03911841	1.07162788	[illegible]
ENSG00000130045	0.01491082	0.08988754	0.02187377	0.009637	0.00831757	0.02236996	0.02394901	[illegible]
ENSG00000130052	0.52640945	0.4471967	0.50540157	0.7634269	0.43956835	0.62361013	0.24230574	[illegible]
ENSG00000130054	0.01606267	0.00537951	0	0	0	0	0	[illegible]
ENSG00000130055	1.03702031	2.30790652	1.56143197	1.124265	0.92284097	1.44857229	0.00488449	[illegible]
ENSG00000130066	4.54289002	3.16738201	3.84105861	3.6247587	4.39554767	3.86623002	8.71939067	[illegible]
ENSG00000130119	2.7581047	2.95347114	2.35215943	2.652612	3.08039642	2.05994713	9.97935178	[illegible]
ENSG00000130147	19.8348045	19.6509259	17.7864475	16.414513	14.6520325	14.5198557	4.93161938	[illegible]
ENSG00000130150	2.42714212	2.24537556	2.03640253	2.3165667	2.36799669	2.219609	2.205443	[illegible]
ENSG00000130158	0.50703421	0.33728807	0.37899473	0.2789578	0.26471236	0.33595159	0.43979859	[illegible]
ENSG00000130159	3.19913727	3.45038105	3.20476911	5.1828573	3.35494813	4.38731209	1.92434118	[illegible]
ENSG00000130164	5.90315806	4.18871459	4.79157189	2.5918362	6.15686575	4.6015868	11.1816845	[illegible]
ENSG00000130165	3.77544044	2.93041596	3.61809559	3.3597341	3.50297974	3.00567504	8.12687482	[illegible]
ENSG00000130167	0	0	0	0	0	0	0.00587313	[illegible]
ENSG00000130173	0.01782618	0	0	0.0230424	0	0.01337186	0	[illegible]
ENSG00000130175	7.86151893	7.91439304	6.88551924	10.379891	6.23798266	8.26612951	11.9019794	[illegible]
ENSG00000130176	0.67165281	0.46273752	0.6349689	0.2687254	0.22836555	0.31189072	0.16695326	[illegible]
ENSG00000130177	2.06281716	2.3500941	2.01739813	2.2434808	2.0583344	2.26636414	2.13643828	[illegible]
ENSG00000130182	0	0.00167388	0.00244399	0	0	0	0.00401379	[illegible]
ENSG00000130193	3.69226533	2.1846758	2.78100371	2.9634848	4.28175737	2.39435449	3.49411422	[illegible]
ENSG00000130201	0.04705071	0.06303063	0.0368118	0.0608185	0.02099671	0.04235269	0.02267115	[illegible]
ENSG00000130202	7.29738128	5.14607569	6.26420824	6.2642201	7.05898779	5.50318489	10.2112568	[illegible]
ENSG00000130203	3.24229894	3.97433804	4.76686489	5.4770455	3.62922439	5.09512249	5.43753419	[illegible]
ENSG00000130204	5.3605543	3.49314066	3.89709885	3.532023	6.58556549	3.60118418	4.45985276	[illegible]
ENSG00000130208	0.0557126	0.05597578	0.11578259	0.0360075	0.13208012	0.08358282	0.36352377	[illegible]
ENSG00000130222	0.30616486	0.85973364	0.56429858	1.4308023	0.10509863	0.86564862	0.00945667	[illegible]
ENSG00000130224	3.52594117	3.88430411	3.81825347	3.3420074	3.15934165	3.42847268	2.22681503	[illegible]
ENSG00000130226	8.25454879	8.99466616	8.25892689	5.3082443	4.6988567	5.10482774	0.00094931	[illegible]
ENSG00000130227	4.67002695	4.79620563	4.45380306	5.3305888	4.72169441	4.3199479	3.60328085	[illegible]
ENSG00000130234	0.03124719	0.01627878	0.0101864	0.0179514	0.00968353	0.02083493	0	[illegible]
ENSG00000130244	0.67362872	0.61909824	0.74050801	0.7593696	0.59277568	0.77754397	0.47387668	[illegible]
ENSG00000130254	3.63190575	4.76818114	3.70730242	3.7883022	3.53548896	4.1256114	5.20649332	[illegible]
ENSG00000130255	52.3272944	52.8736756	53.8296193	81.717036	49.8420346	65.034633	94.3142815	[illegible]
ENSG00000130270	0.04758386	0.01673302	0.02675829	0.0276785	0.01459881	0.04372495	0.12897023	[illegible]
ENSG00000130283	0	0	0	0	0	0	0	[illegible]
ENSG00000130287	0.18652369	2.38073491	0.61058964	0.723311	0.18786243	1.11155537	0.00208045	[illegible]
ENSG00000130294	5.9193559	4.74400031	4.31670711	6.566674	7.97612144	5.56542301	0	[illegible]
ENSG00000130299	0.24962404	0.22697123	0.23749993	0.2306859	0.22871563	0.26858823	0.54243954	[illegible]
ENSG00000130300	0	0	0	0	0	0	0.04857596	[illegible]
ENSG00000130303	0.21332426	0.32149793	0.48679701	0.8272395	0.23799362	0.50672946	36.2902996	[illegible]
ENSG00000130304	1.28227757	0.99735067	1.0629369	1.2671266	0.96301784	1.18598118	0.97399671	[illegible]
ENSG00000130305	0.70616237	0.61428409	0.49927514	0.9009428	0.50816406	0.51365814	0.96726356	[illegible]
ENSG00000130307	0.01302387	0.02471685	0.02971993	0.0224465	0.02179498	0.0325651	0.02440467	[illegible]
ENSG00000130309	7.92158592	5.44308543	6.38408115	6.7321214	8.37314019	6.31273764	16.6479083	[illegible]
ENSG00000130311	4.0959161	4.08097042	3.76538843	3.6994968	4.29540369	3.8175173	7.57778731	[illegible]
ENSG00000130312	3.92670026	2.16601912	3.05713409	3.7520508	4.11451045	3.18808739	8.10618066	[illegible]
ENSG00000130313	4.64153647	4.8915042	4.83345297	6.1464205	4.59589571	4.61393328	6.44793892	[illegible]
ENSG00000130332	12.90156	11.1392305	12.6647505	14.553139	13.5804121	12.3660535	8.41420373	[illegible]
ENSG00000130338	5.13325813	6.2247824	6.25836525	6.0907011	4.60909121	6.08971066	3.57432692	[illegible]
ENSG00000130340	16.6215785	9.68836928	12.1335141	9.6857659	19.8199612	9.20290004	45.7050698	[illegible]
ENSG00000130347	1.40465198	1.14314267	1.30503819	1.2982089	1.70812762	1.11161593	1.48901365	[illegible]
ENSG00000130348	3.01686197	3.11679241	3.0448508	3.3131396	3.65115439	3.35289516	3.55855855	[illegible]
ENSG00000130349	2.49209814	2.5342627	2.49866992	2.481415	2.46098569	2.22022171	3.08890488	[illegible]
ENSG00000130363	1.05254781	1.32574897	1.0341056	1.2667105	0.98395489	0.98738162	1.61087973	[illegible]
ENSG00000130368	0	0	0	0	0	0	0	[illegible]
ENSG00000130377	0	0	0.00152463	0	0	0	0.00250391	[illegible]
ENSG00000130382	9.38779025	10.398888	8.26052639	9.0037733	9.63330474	7.823415	16.5094152	[illegible]
ENSG00000130383	0	0.01577301	0.0076766	0.0405852	0	0.02944025	0.00630368	[illegible]
ENSG00000130385	0.05467301	0	0	0	0.01524888	0	0	[illegible]
ENSG00000130396	1.58608412	1.96838662	1.6274257	1.4832786	1.36930847	1.67580481	1.37963655	[illegible]
ENSG00000130402	7.89906129	6.42620418	6.41477209	3.85036	8.16936251	3.99644663	17.5704127	[illegible]
ENSG00000130413	0.86828107	1.07439621	0.99307066	1.087508	0.78635681	1.01868687	0.4943305	[illegible]
ENSG00000130414	5.94487482	5.84350106	5.68622024	7.1061453	6.5674694	6.34947462	2.85703446	[illegible]
ENSG00000130427	0	0	0.07109798	0	0.05407048	0.01817769	0	[illegible]
ENSG00000130429	1.92267559	0.74408452	1.26748993	0.8547269	2.29255973	0.90808131	3.42142398	[illegible]
ENSG00000130433	0.04678888	0.0470099	0.03813224	0.03024	0.02609983	0.11114209	0.08767493	[illegible]
ENSG00000130449	7.86262694	8.97504716	8.17081841	6.0283438	7.6966396	6.42094478	4.14992025	[illegible]
ENSG00000130475	0.0607053	0.08040901	0.07204291	0.1331323	0.04870142	0.12688838	0.02684038	[illegible]
ENSG00000130477	0.02786031	0.03465666	0.02594942	0.0128617	0.02923202	0.00895659	0.00692526	[illegible]
ENSG00000130479	1.93646356	1.63894725	1.53894139	1.6701981	1.6581363	1.44368569	1.21877145	[illegible]

ENSG00000130487	0.01077918	0	0	0	0	0	0.57998455	0.38009844	0.10658265	0.01952589	[illegible]
ENSG00000130489	1.38868086	0.84334548	1.19513235	1.2924232	1.43565825	0.91668196	3.31590486	3.42776439	2.94759592	1.35838053	[illegible]
ENSG00000130508	[illegible]										
ENSG00000130511	[illegible]										
ENSG00000130513	[illegible]										
ENSG00000130517	[illegible]										
ENSG00000130518	[illegible]										
ENSG00000130520	[illegible]										
ENSG00000130522	[illegible]										
ENSG00000130528	[illegible]										
ENSG00000130529	[illegible]										
ENSG00000130538	[illegible]										
ENSG00000130540	[illegible]										
ENSG00000130544	[illegible]										
ENSG00000130545	[illegible]										
ENSG00000130558	[illegible]										
ENSG00000130559	[illegible]										
ENSG00000130560	[illegible]										
ENSG00000130561	[illegible]										
ENSG00000130584	[illegible]										
ENSG00000130589	[illegible]										
ENSG00000130590	[illegible]										
ENSG00000130592	[illegible]										
ENSG00000130595	[illegible]										
ENSG00000130598	[illegible]										
ENSG00000130600	[illegible]										
ENSG00000130612	[illegible]										
ENSG00000130635	[illegible]										
ENSG00000130638	[illegible]										
ENSG00000130640	[illegible]										
ENSG00000130643	[illegible]										
ENSG00000130649	[illegible]										
ENSG00000130653	[illegible]										
ENSG00000130656	[illegible]										
ENSG00000130669	[illegible]										
ENSG00000130675	[illegible]										
ENSG00000130684	[illegible]										
ENSG00000130695	[illegible]										
ENSG00000130699	[illegible]										
ENSG00000130700	[illegible]										
ENSG00000130701	[illegible]										
ENSG00000130702	[illegible]										
ENSG00000130703	[illegible]										
ENSG00000130706	[illegible]										
ENSG00000130707	[illegible]										
ENSG00000130711	[illegible]										
ENSG00000130713	[illegible]										
ENSG00000130714	[illegible]										
ENSG00000130717	[illegible]										
ENSG00000130720	[illegible]										
ENSG00000130723	[illegible]										
ENSG00000130724	[illegible]										
ENSG00000130725	[illegible]										
ENSG00000130726	[illegible]										
ENSG00000130731	[illegible]										
ENSG00000130733	[illegible]										
ENSG00000130734	[illegible]										
ENSG00000130741	[illegible]										
ENSG00000130748	[illegible]										
ENSG00000130749	[illegible]										
ENSG00000130751	[illegible]										
ENSG00000130755	[illegible]										
ENSG00000130758	[illegible]										
ENSG00000130762	[illegible]										
ENSG00000130764	[illegible]										
ENSG00000130766	[illegible]										
ENSG00000130768	[illegible]										
ENSG00000130770	[illegible]										
ENSG00000130772	[illegible]										
ENSG00000130775	[illegible]										
ENSG00000130779	[illegible]										
ENSG00000130783	[illegible]										
ENSG00000130787	[illegible]										
ENSG00000130803	[illegible]										
ENSG00000130810	[illegible]										
ENSG00000130811	[illegible]										
ENSG00000130812	[illegible]										
ENSG00000130813	[illegible]										
ENSG00000130816	[illegible]										
ENSG00000130818	[illegible]										
ENSG00000130821	[illegible]										
ENSG00000130822	[illegible]										
ENSG00000130826	[illegible]										
ENSG00000130827	[illegible]										
ENSG00000130829	[illegible]										
ENSG00000130830	[illegible]										
ENSG00000130844	[illegible]										
ENSG00000130856	[illegible]										
ENSG00000130876	[illegible]										
ENSG00000130881	[illegible]										
ENSG00000130921	[illegible]										
ENSG00000130935	[illegible]										
ENSG00000130939	[illegible]										
ENSG00000130940	[illegible]										
ENSG00000130943	[illegible]										
ENSG00000130948	[illegible]										
ENSG00000130950	[illegible]										
ENSG00000130956	[illegible]										
ENSG00000130957	[illegible]										
ENSG00000130958	[illegible]										
ENSG00000130962	[illegible]										
ENSG00000130985	[illegible]										
ENSG00000130988	[illegible]										
ENSG00000130997	[illegible]										
ENSG00000131002	[illegible]										
ENSG00000131007	[illegible]										
ENSG00000131013	[illegible]										
ENSG00000131015	[illegible]										
ENSG00000131016	[illegible]										
ENSG00000131018	[illegible]										
ENSG00000131019	[illegible]										
ENSG00000131023	[illegible]										
ENSG00000131037	[illegible]										
ENSG00000131042	[illegible]										
ENSG00000131043	[illegible]										
ENSG00000131044	[illegible]										
ENSG00000131050	[illegible]										
ENSG00000131051	[illegible]										
ENSG00000131055	[illegible]										
ENSG00000131059	[illegible]										
ENSG00000131061	[illegible]										
ENSG00000131067	[illegible]										
ENSG00000131068	[illegible]										
ENSG00000131069	[illegible]										
ENSG00000131080	[illegible]										
ENSG00000131089	[illegible]										
ENSG00000131094	[illegible]										
ENSG00000131095	[illegible]										
ENSG00000131096	[illegible]										
ENSG00000131097	[illegible]										
ENSG00000131100	[illegible]										
ENSG00000131115	[illegible]										
ENSG00000131116	[illegible]										
ENSG00000131126	[illegible]										
ENSG00000131127	[illegible]										
ENSG00000131142	[illegible]										
ENSG00000131143	[illegible]										
ENSG00000131148	[illegible]										
ENSG00000131149	[illegible]										
ENSG00000131152	[illegible]										
ENSG00000131153	[illegible]										
ENSG00000131165	[illegible]										
ENSG00000131171	[illegible]										
ENSG00000131174	[illegible]										
ENSG00000131183	[illegible]										
ENSG00000131187	[illegible]										
ENSG00000131188	[illegible]										
ENSG00000131196	[illegible]										
ENSG00000131203	[illegible]										
ENSG00000131233	[illegible]										
ENSG00000131236	[illegible]										
ENSG00000131238	[illegible]										
ENSG00000131242	[illegible]										
ENSG00000131263	[illegible]										
ENSG00000131264	[illegible]										
ENSG00000131269	[illegible]										
ENSG00000131323	[illegible]										
ENSG00000131351	[illegible]										
ENSG00000131355	[illegible]										
ENSG00000131368	[illegible]										
ENSG00000131370	[illegible]										
ENSG00000131373	[illegible]										
ENSG00000131374	[illegible]										
ENSG00000131375	[illegible]										
ENSG00000131378	[illegible]										
ENSG00000131379	[illegible]										
ENSG00000131381	[illegible]										
ENSG00000131386	[illegible]										
ENSG00000131389	[illegible]										
ENSG00000131398	[illegible]										
ENSG00000131400	[illegible]										
ENSG00000131401	[illegible]										
ENSG00000131408	[illegible]										
ENSG00000131409	[illegible]										
ENSG00000131435	[illegible]										
ENSG00000131437	[illegible]										
ENSG00000131446	[illegible]										
ENSG00000131459	[illegible]										
ENSG00000131462	[illegible]										
ENSG00000131467	[illegible]										
ENSG00000131469	[illegible]										

ENSG00000131470	2.16779989	1.34687225	1.66273544	1.1700747	2.48556662	1.46075241	1.00055124	1.35451184	1.71742872	1.10726308	1.21613456	2.10150306	2.6830049	1.13261759	1.74588935	2.91933894	2.17075542	1.31751949	1.22777204	0.91051816	3.48940143	2.24230574	2.84562741	5.14261456	5.35689039	5.27787535	2.13537444	4.69719266	2.91910587	2.41552748	1.4768222	0.80916134	1.92338266	1.24591471	1.34009203	2.07096264	1.39094068	1.61560822	1.62504242	1.89397487	2.55834901	1.68073477	1.81657377	0.72719115	0.69324987	3.41332172	1.20463166	0.63393261
ENSG00000131471	0.0157065	0.00789035	0.00768034	0.0203025	0.00219036	0.01767276	0.04414723	0.05769241	0.02329548	0.07824154	0.02473425	0.02984429	0.02991181	0.0255232	0.05122459	0.0151517	0.02953535	0.02368232	0.06914109	0.11505596	0.10246742	0.06371928	0.13921789	0.040157	0.12369791	0.11956514	0.15879179	0.09770537	0.08177155	0.132662	0.01193192	0.04323718	0.05797571	0.0123599	0.05353162	0.03492886	0.07267266	0.03956761	0.04350661	0.05676583	0.05868281	0.04921935	0.07642814	0.14415238	0.11952704	0.04314587	0.03146927	0.12114513
ENSG00000131473	8.99607171	8.11999025	7.17907587	6.5354823	14.4846404	8.27679339	12.1867913	11.8309694	15.6914076	20.2173499	16.908489	18.6205523	7.60590958	9.01146147	8.43074727	11.8858591	10.5750826	8.31072226	6.51436715	6.65736724	10.3297382	18.3223069	17.0102229	18.9816705	18.950647	25.1676278	19.1791108	28.5329147	22.9617611	20.8957263	7.93774537	7.31379033	8.02062386	7.52172284	7.67076536	11.0819438	23.7357547	28.6275815	27.7132087	16.4514683	15.4086994	11.4864221	13.265517	16.8570342	15.5473424	13.7771708	15.3468952	12.817315
ENSG00000131475	6.85022344	5.94233887	5.82078624	6.7499047	6.62054303	5.17011225	8.93388172	9.7210304	8.6575769	10.2601496	8.49743013	8.04922285	8.57833246	7.77088149	7.64235135	8.72749617	7.30657201	6.29887019	6.12526506	5.45304039	9.80180291	9.08765669	9.42770514	13.3900262	18.0635786	15.3645542	11.2084001	14.5483823	13.0538753	10.9530343	9.50152584	8.89631428	10.0405035	12.4271894	13.0405589	12.1496215	11.0024633	11.1038835	10.2227159	11.9617664	10.0828359	9.11194937	10.2298263	7.88578114	7.23818222	10.9801542	7.42499416	6.22065075
ENSG00000131477	0.13189604	0.11358779	0.15663309	0.1217793	0.06306379	0.10600554	0.34803071	0.5075448	0.47508879	1.22874414	0.61718642	0.49328133	0.17543133	0.14288816	0.11470953	0.09997197	0.05669129	0.10227772	0.96423625	1.17950172	1.27683348	1.15868133	1.25020885	1.54157814	1.16017211	0.9536571	0.91599458	1.83277214	1.7792798	1.88703898	0.28628227	0.13831855	0.29365771	0.28172332	0.29357334	0.23465335	0.10461804	0.13290829	0.08540618	0.43886075	0.66878845	0.40488654	0.24809413	0.31369113	0.23463904	0.25879955	0.18456577	0.09688777
ENSG00000131480	0.02465461	0.04954215	0.02411177	0.0637379	0.0206293	0.09247018	0.57418542	0.55543591	0.28644213	0.6699072	0.20965789	0.2394392	0.20867908	0.18696515	0.19297827	0.19621578	0.13908557	0.15613212	0.31202743	0.50897546	0.59667887	0.36849703	0.49950023	0.33809544	0.65899962	0.5275394	0.75837661	0.46010653	0.36294163	0.55034995	0.19666097	0.1960681	0.25279138	0.09700712	0.13204555	0.14803585	0.61600398	0.40992312	0.4780485	0.26731722	0.32240193	0.58496839	0.28228193	0.64199537	0.57423852	0.28783717	0.37871456	0.56472472
ENSG00000131482	0.01114445	0.00746473	0	0.0072027	0.0031083	0.01253959	0	0.01637411	0.00275486	0	0	0.00470574	0.00471639	0	0.00484614	0	0.01676529	0	0	0.00494771	0	0.00475912	0	0.01554171	0.00531934	0	0	0.01260477	0.00400141	0.00448236	0.00846621	0.00681748	0.0091414	0	0.01085229	0.00495671	0.00515644	0	0	0	0	0.00997808	0.00637989	0	0	0	0	0.00520956
ENSG00000131484	0	0.29438169	0.14327321	0.1420251	0.1021502	0.10989241	0.08823713	0.10762257	0.1448555	0.09951548	0.09228124	0.03092953	0.18599708	0.19838489	0.38222855	0.070662	0.16529055	0.35342634	0.52397946	0.1951197	0.30830374	0.18768224	0.22260398	0.2043029	0.34962551	0.30140992	0.3466391	0.19331129	0.34190248	0.35353562	0.22258446	0.40328501	0.30041957	0.34585245	0.64196234	0.13031641	0.16945943	0.07381157	0.07378145	0.05883004	0.06841882	0	0.20966651	0.09380571	0.30405309	0.10060833	0.09784083	0.20544612
ENSG00000131495	11.2827718	8.35664151	9.63627893	13.011334	9.9184548	11.3437323	8.09314788	7.07068704	6.54770243	6.3026468	7.8587894	6.55506593	4.36993151	5.03001701	5.59985378	4.60442738	3.99896503	3.47725918	5.79887933	5.76966855	7.80704621	8.43561016	8.32969731	10.248097	13.5564472	14.6815797	8.92519438	13.8346741	12.5138006	12.145659	6.49803032	4.81340171	5.89209993	6.43359927	5.75235068	8.34445409	18.5312083	15.131372	13.7447706	14.6885325	13.8603283	12.0588435	9.23885333	7.46411056	6.54967415	6.47463253	6.09138084	4.83792471
ENSG00000131503	0.06706369	0.07751737	0.07371291	0.0560018	0.07647439	0.09437966	0.03526929	0.04999371	0.0396108	0.0387023	0.05607632	0.05412925	0.08137758	0.06815211	0.07019653	0.06412226	0.07856775	0.05727087	0.05747625	0.07957226	0.06544628	0.08718369	0.1064118	0.08938675	0.04928982	0.05616816	0.07608622	0.07294267	0.05838675	0.06588186	0.05590638	0.05518462	0.05160229	0.04390079	0.04045479	0.03484319	0.03954241	0.02870586	0.02630297	0.07245153	0.06541275	0.05419978	0.0686302	0.06333633	0.07116806	0.04809399	0.05654803	0.06991206
ENSG00000131504	0.83230952	0.60221289	0.64531066	0.4530682	0.97169826	0.49830032	0.6934274	0.75530868	0.73688017	0.68178322	0.67007044	0.78510724	0.869763	0.55571058	0.68176078	0.93279213	0.67877647	0.47648192	0.79045344	0.70642336	1.36801068	1.35994295	1.73528378	1.7193466	1.89764632	2.45964643	1.64816629	1.91630402	1.817814	1.77315026	0.50464774	0.56565253	0.66902344	0.58141579	0.61545247	0.82005083	1.49664881	1.1588149	0.96052167	1.33514638	1.26590758	1.0899058	1.02938909	0.70488957	0.60198278	1.56117444	0.77781744	0.64082071
ENSG00000131507	16.0849068	15.4820578	17.2821059	18.126028	15.1488421	14.9822905	6.66676279	6.59491828	6.12756369	5.44042606	6.96787446	6.05505775	8.42882995	8.26780929	9.31679482	8.53879568	9.03099043	6.94792201	8.98469656	8.19746026	11.4154997	10.0436579	14.8977241	12.5979711	9.11267172	8.2273072	9.97568408	10.8180139	11.5328481	10.3329481	14.6857544	13.5035075	13.1736429	20.8880806	22.9645063	22.9908766	6.02825547	6.56077734	5.29760998	9.60326564	8.77149754	8.72988718	12.4874374	8.6434361	8.36969073	8.63275108	7.70941097	7.57010035
ENSG00000131508	8.92831694	7.89902565	7.99044741	8.038209	8.28188368	7.83450478	3.61929327	3.83904801	4.08774994	3.30099481	4.36873221	4.21938865	5.67244025	4.72157994	5.5318578	6.52901687	5.47329585	4.13340853	4.03874746	3.33427477	5.63351437	5.41633215	7.54068709	8.4593345	8.11912316	7.95332308	6.23372928	7.49420515	6.80150516	6.02609915	6.78026521	6.51087169	5.39877958	6.35243337	6.05860909	5.58221618	6.31568062	5.65524653	4.91172638	6.92978091	6.56069758	5.47441251	7.88650152	5.88884787	5.59051161	7.92089688	5.46719805	4.70066623
ENSG00000131538	0	0	0	0	0	0	0	0	0	0	0	0	0	0	0	0	0	0	0	0	0	0	0	0	0	0	0	0	0	0	0	0	0	0	0	0	0	0	0	0	0	0	0	0	0	0	0	0
ENSG00000131548	0	0	0	0	0	0	0	0	0	0	0	0	0	0	0	0	0	0	0	0	0	0	0	0	0	0	0	0	0	0	0	0	0	0	0	0	0	0	0	0	0	0	0	0	0	0	0	0
ENSG00000131558	6.09235344	7.08690079	6.19322812	6.176996	4.6508389	6.21897768	2.46468731	2.61835093	2.29181826	3.4876055	2.72325492	2.28741288	1.91194305	2.32688964	2.472102	2.00799012	2.16561046	1.80408682	1.9280532	1.88683833	1.77628489	2.23908161	2.70902681	2.47141343	2.60424765	2.66888768	2.35318287	3.63947436	3.61070387	3.25121385	5.96524783	6.43041394	5.39720582	4.59433836	4.98769672	3.66144467	3.51036422	3.91131876	3.60312307	3.41888901	3.40536965	3.10440267	2.78937973	1.93146847	2.11849384	2.3387283	2.14294523	1.91287733
ENSG00000131584	2.81417892	4.12345518	2.80626094	2.8286919	2.35895193	2.87164511	1.40570213	1.28961937	1.17342334	0.97349901	1.30362232	1.04769838	1.7610286	1.07909682	1.40817309	1.31807098	1.159705	1.01987489	1.6040205	2.26325135	1.51301291	1.3522067	0.85228632	1.00442284	1.34389038	1.22563977	1.55719394	1.17817099	1.42363073	1.71365285	0.25398424	0.23832809	0.36943018	0.42182069	0.48431296	0.73735549	1.22347251	1.40744734	1.51541519	1.04521732	0.98330239	0.94254919	0.95064876	1.6418513	1.32916156	1.38899778	1.68910236	2.24095284
ENSG00000131591	0.32750686	0.31494246	0.26035743	0.261184	0.30181292	0.25788276	0.19610582	0.25982566	0.25329054	0.23678391	0.18458441	0.19651687	0.33313241	0.29048038	0.31647955	0.21524449	0.19880442	0.18482005	0.22342252	0.22107691	0.35388608	0.27644503	0.17655224	0.33386794	0.4068022	0.43975614	0.26696237	0.30254736	0.27781804	0.2349481	0.08875337	0.06443954	0.06912435	0.09645833	0.11376719	0.13203558	0.2357206	0.23448815	0.2064197	0.2199659	0.25760734	0.17833887	0.22038158	0.3049559	0.25881814	0.38406696	0.27799479	0.29634312
ENSG00000131620	0	0	0	0	0	0.00090267	0	0	0	0	0	0	0.00203708	0	0	0	0	0.00145155	0.21983784	0.1442471	0.96233403	0.05036066	0.0597312	0.10404703	0.01033877	0.00099033	0.0031062	0.0027221	0.00691309	0.01452	0.00182835	0	0.00197416	0	0	0.00107044	0	0	0	0	0	0	0	0	0.00222004	0	0	0
ENSG00000131626	1.03895302	1.08646153	0.98518597	1.0549528	1.17957735	1.04382213	1.41916107	1.57039495	1.89632442	1.34230218	1.76598812	1.75190627	1.83463127	1.61940676	1.72027991	1.84309297	1.75175093	1.46330283	1.20257475	1.1673519	1.71898136	1.22274581	1.18217397	1.56675695	1.57449556	1.46555811	1.09299707	1.45285387	1.4099962	1.24915356	5.6176444	3.56899345	4.33320991	4.85846667	4.20149146	3.69827572	2.45768911	2.23235376	1.98809091	1.63869546	1.54855275	1.38653549	1.84247507	1.79224662	1.75292478	2.10826313	1.8579383	1.83252445
[illegible]	[illegible]	[illegible]	[illegible]	[illegible]	[illegible]	[illegible]	[illegible]	[illegible]	[illegible]	[illegible]	[illegible]	[illegible]	[illegible]	[illegible]	[illegible]	[illegible]	[illegible]	[illegible]	[illegible]	[illegible]	[illegible]	[illegible]	[illegible]	[illegible]	[illegible]	[illegible]	[illegible]	[illegible]	[illegible]	[illegible]	[illegible]	[illegible]	[illegible]	[illegible]	[illegible]	[illegible]	[illegible]	[illegible]	[illegible]	[illegible]	[illegible]	[illegible]	[illegible]	[illegible]	[illegible]	[illegible]	[illegible]	[illegible]

ENSG00000132646	92.5636218	53.3510089	68.1594611	37.098524	81.6911353	46.4833088	28.0182552	[illegible]
ENSG00000132661	14.8377342	8.09309352	11.9007348	11.574478	14.4083629	9.25718756	15.0503374	[illegible]
ENSG00000132664	2.11840795	1.83216866	2.12056453	1.4365759	1.68271067	1.53802968	2.25860569	[illegible]
ENSG00000132669	0.7716907	0.91583312	0.82054592	1.9280544	0.96349158	1.28399114	3.59981417	[illegible]
ENSG00000132670	8.27479946	9.74855383	9.01601257	7.6449337	6.67121259	6.84251604	3.30313487	[illegible]
ENSG00000132671	0	0	0	0	0	0	0	[illegible]
ENSG00000132676	4.03254445	3.95589405	3.90668531	4.8620171	4.3142714	4.18303976	5.19133062	[illegible]
ENSG00000132677	0.00578657	0.01744171	0.01839227	0.0168296	0.01210452	0.0086813	0.01974995	[illegible]
ENSG00000132680	4.97073313	5.5501474	4.67345508	5.4942711	4.88616519	5.92573908	9.23969323	[illegible]
ENSG00000132681	0.00852751	0.01999152	0.00416988	0.0165342	0.01189207	0.0159175	0.01712058	[illegible]
ENSG00000132688	491.970335	551.088031	438.485629	390.33485	424.857785	400.997351	0.50294214	[illegible]
ENSG00000132692	211.702955	470.999219	238.900773	481.40743	183.958372	372.398826	0.01228035	[illegible]
ENSG00000132693	0	0	0	0	0	0	0	[illegible]
ENSG00000132694	3.99785517	5.45469768	4.16190649	3.8718255	3.68150039	4.06025567	3.62020592	[illegible]
ENSG00000132698	0.01689632	0.02829357	0	0	0.00942513	0.01267435	0.02713803	[illegible]
ENSG00000132702	0.12071064	0.08936483	0.04970646	0.0903347	0.09568653	0.16203335	0	[illegible]
ENSG00000132703	0	0	0	0	0	0	0	[illegible]
ENSG00000132704	0.00794162	0.01241199	0.00517784	0.0102655	0.00886001	0.01191441	0.01062952	[illegible]
ENSG00000132716	1.41656004	2.21979033	1.72024808	2.0509667	1.18132704	2.22908129	2.23755002	[illegible]
ENSG00000132718	207.877401	221.709518	218.402348	167.59513	181.387121	169.89011	7.19451329	[illegible]
[illegible]	[illegible]	[illegible]	[illegible]	[illegible]	[illegible]	[illegible]	[illegible]	[illegible]

ENSG00000133943	0.73113619	0.94644721	0.83973299	1.1114829	0.64560878	0.91761472	0.34675409	0.36139518	0.25884357	0.25829964	0.40739544	0.27725158	0.27266539	0.34565416	0.34498577	0.19073677	0.20756219	[illegible]
ENSG00000133958	0.10027335	0.13203313	0.0913602	0.0764396	0.12812372	0.10350447	0.00688265	0.00755527	0.00338969	0.01474852	0.00539857	0.0043426	0.00507783	0.01114154	0.00670826	0.00992117	0.01160366	[illegible]
ENSG00000133961	0.62720414	0.71457108	0.7332851	0.609525	0.63071626	0.61504753	0.71615823	0.78581135	0.87471967	0.87999674	0.99489053	0.91151979	3.07953918	2.88776809	2.92921298	2.54175699	2.75342921	[illegible]
ENSG00000133962	0	0.00438379	0.00640067	0.0042299	0.00182541	0.0098188	0	0	0	0.00592775	0	0	0.0138489	0.01063532	0.01422992	0.0031568	0	[illegible]
ENSG00000133980	0	0	0	0	0	0	0	0	0	0.00399652	0.0073216	0.01018403	0.00682668	0	0.0087574	0	0	[illegible]
ENSG00000133983	8.16148371	7.18693103	8.28953985	7.229936	7.67834894	7.91917561	5.97548356	5.13063055	5.55560779	3.99023103	4.38648334	5.10069431	5.01772293	4.83871693	4.87326355	5.91468796	5.72660189	[illegible]
ENSG00000133985	1.19849904	0.64966734	1.11773428	0.5749608	1.73684709	0.72292647	0.00992252	0	0.0091628	0.0055954	0	0.00521717	0.00522898	0	0	0.01787883	0.0092937	[illegible]
ENSG00000133997	1.58545928	1.64574806	1.52436879	1.5303147	1.35232345	1.49944562	1.61006642	1.64912011	1.66914718	1.50064623	1.44277267	1.75340632	1.86060028	1.71436774	1.88074556	2.2066632	1.95104384	[illegible]
ENSG00000134001	9.78035631	7.52847294	7.74866585	7.3650017	10.1806265	7.64994987	9.73737966	10.9508197	10.6019681	6.53032336	9.43019684	10.1556322	20.0372081	14.5440339	15.6879687	19.6760016	16.9583551	[illegible]
ENSG00000134007	0.04612485	0.05406652	0.04510928	0.134149	0.04502649	0.25084564	0.00926043	0.04517966	0.03990653	0.06266449	0.0726364	0	0.01952027	0.04996886	0.03008597	0.06674336	0.03469426	[illegible]
ENSG00000134013	4.39784399	2.26683693	3.68862451	2.6864665	6.25330961	2.81460139	4.74689277	5.555889	5.27001145	10.2173635	8.60082356	5.1769326	27.9229975	25.1996208	25.69284	14.6172273	16.6113592	[illegible]
ENSG00000134014	1.42576535	1.47144816	1.44441565	1.5513403	1.3564591	1.4655493	1.6651944	1.73030767	1.50463859	1.52518102	1.77716472	1.68430591	1.18217387	1.14143255	1.12569439	1.50778245	1.36284887	[illegible]
ENSG00000134020	0	0	0.01728076	0	0.0098566	0	0.01419018	0.05192317	0.0087358	0.0320079	0	0	0.07477953	0.15313922	0.01536738	0.06818264	0.05316363	[illegible]
ENSG00000134028	0	0	0	0	0	0	0	0	0	0	0	0	0.01744218	0.00744155	0.00298701	0.00331322	0	[illegible]
ENSG00000134030	0.59176148	0.83783857	0.71561784	1.2687609	0.68391386	1.18150497	0.93196057	1.18877255	0.99681227	1.85022598	0.99773922	0.89325163	1.59259557	1.57582452	1.57169456	1.00840285	1.01016447	[illegible]
ENSG00000134042	0.04953341	0.01843237	0.02153013	0.0675848	0.04912141	0.03302777	0.03535914	0.02965012	0.01496543	0.02990902	0.0138674	0.03485911	0	0	0	0	0	[illegible]
ENSG00000134046	5.88200331	4.71738646	4.87564074	3.6784144	6.26744529	3.69419252	5.26632166	5.69782989	6.64474254	5.94730126	5.53861059	6.4048786	10.6317377	10.4076219	9.8054474	13.7969914	13.3707419	[illegible]
ENSG00000134049	29.3353742	23.9785581	27.4763387	20.760026	31.8173384	19.1974094	13.264764	15.2410804	16.0524547	13.0877522	12.447063	16.6873299	23.1351405	21.5954215	23.6013011	31.1962491	27.8274442	[illegible]
ENSG00000134056	3.8401071	3.20888905	3.34237086	3.7447822	3.90963627	3.72378856	2.42349182	1.9065721	2.38340201	1.7971178	2.35714626	2.17896835	2.8480078	2.76252824	2.76889035	3.51523623	3.40486161	[illegible]
ENSG00000134057	24.1571633	14.2509715	18.5058988	16.921515	22.7565963	17.3745057	7.4382917	9.40298624	17.5681533	7.24490475	6.5020494	19.2937193	17.9898711	16.7220625	23.9365714	22.2563499	21.8476396	[illegible]

[illegible]

ENSG00000134962	0.026496	0.0319454	0.04664271	0.0273993	0.02069205	0.02385037	0.03404524	0.04152488	0.02619879	0.03839685	[illegible]
ENSG00000134970	18.883338	21.3693354	20.78829	20.684287	19.6032877	16.6465377	27.3852499	28.2303459	24.5777525	21.7086142	[illegible]
ENSG00000134982	3.01619655	4.04218268	3.68345972	2.4104656	2.90447868	2.7046111	1.16297617	1.23477396	1.17985854	0.98049426	[illegible]
ENSG00000134986	3.74179918	6.28898173	4.07325958	5.2212625	2.36908478	4.94511456	3.05231534	4.30695809	7.41106441	3.17765339	[illegible]
ENSG00000134987	4.41536701	4.4787074	3.88156324	4.2789565	4.07277142	3.98181144	6.96440231	7.18504734	7.11076944	5.63187895	[illegible]
ENSG00000134996	7.74588894	2.32410513	5.63968549	2.5268053	9.45946467	3.09764948	5.57296551	4.93045723	4.57848823	2.54510138	[illegible]
ENSG00000135002	2.31514533	1.90238062	2.38183611	1.6871642	2.9713228	1.61084367	2.40675766	2.95694225	3.2138586	2.33133389	[illegible]
ENSG00000135018	8.57845935	8.36959291	8.34674068	9.4973289	9.46118357	8.14697337	8.41656867	9.47756058	10.7629297	9.42345592	[illegible]
ENSG00000135040	4.92607977	5.48106303	4.90353684	5.3617064	4.9881756	5.26268229	6.03228631	5.67337456	6.31655738	5.71206431	[illegible]
ENSG00000135045	5.69248949	3.62737615	4.51061259	4.6190207	5.90267484	5.5999319	3.54819199	4.99247854	6.31338429	4.42823198	[illegible]
ENSG00000135046	3.42692299	0.6066087	1.51985228	0.1779371	0.44253987	0.13315052	24.9665038	22.7801305	17.5853638	15.5356144	[illegible]
ENSG00000135047	6.75707771	5.27984561	6.21732722	6.6167328	5.0078801	5.08820954	34.9665073	37.5026272	25.8120908	27.2902554	[illegible]
ENSG00000135048	2.79953219	1.68847539	1.76529171	1.2451517	2.40558277	1.18673763	0.88729579	0.94790857	0.99216099	0.46551633	[illegible]
ENSG00000135049	2.05113851	1.59286885	1.58198295	1.9255718	2.1368917	2.13997941	1.65064227	1.77380574	1.76108619	1.66979776	[illegible]
ENSG00000135052	17.073261	23.8981286	20.4306939	22.374191	16.2474855	19.1790959	16.3017316	17.5560822	14.9832688	12.7497446	[illegible]
ENSG00000135063	0	0.00193354	0	0	0	0.00216537	0	0	0	0	[illegible]
ENSG00000135069	44.1670121	63.8522255	51.9963526	70.252515	54.2949736	57.0316856	26.199327	29.1483023	35.3481239	27.9907899	[illegible]
ENSG00000135070	5.68577749	4.89654499	5.47569378	4.3840716	5.32297258	4.97200772	5.72630368	6.58958758	6.27493534	3.99287181	[illegible]
ENSG00000135074	8.63577026	3.42607589	4.00857598	3.0653188	13.9613888	4.2625097	0.99512903	1.31845689	2.80541512	1.81914654	[illegible]
ENSG00000135077	0.26468183	0.12663435	0.28658845	0.0977521	0.32692857	0.14890832	0.10627966	0.0694441	0.06309149	0.09417899	[illegible]
[illegible]	[illegible]	[illegible]	[illegible]	[illegible]	[illegible]	[illegible]	[illegible]	[illegible]	[illegible]	[illegible]	[illegible]

ENSG00000135905	6.79573835	12.0662002	9.44242619	7.2651469	4.00662266
ENSG00000135912	1.38565568	1.6262821	1.4139358	1.2814423	1.42142908
ENSG00000135913	3.34380582	2.73605789	2.74470436	1.8994683	3.00418931
ENSG00000135914	0.02869968	0.038447	0.02806777	0.0185488	0.03201858
ENSG00000135916	22.7426167	20.2729421	23.0092673	25.490623	15.7178463
ENSG00000135917	0.66648631	0.61984129	0.5690817	0.7289712	0.8264961
ENSG00000135919	16.9999873	18.9813019	17.1723952	17.520748	10.9981487
ENSG00000135924	4.68181632	4.02930746	4.81507324	5.7631429	4.01426237
ENSG00000135925	0.08419072	0.01025314	0.05988147	0.0296799	0.03842458
ENSG00000135926	1.04123237	0.6482496	0.7971229	0.9586555	0.91014496
ENSG00000135929	6.11053284	5.59997591	5.85419806	7.549795	4.42745592
ENSG00000135930	4.168907	3.82515287	3.82486563	4.0255465	3.79733025
ENSG00000135931	0.86155908	1.06529825	1.01560559	0.7523532	1.08454825
ENSG00000135932	12.5440577	12.3872562	11.6221861	11.016215	12.7004396
ENSG00000135940	63.2300729	46.8574036	61.6610612	64.064625	78.7380151
ENSG00000135945	2.70541152	2.37657449	2.39245297	1.9606949	2.77832596
ENSG00000135951	0.15012022	0.15944818	0.13632811	0.1580104	0.12620853
ENSG00000135953	0.99263876	0.88824504	0.99859176	0.9990816	0.89546103
ENSG00000135956	7.16396448	6.61724975	6.28057217	5.7086093	6.91700722
ENSG00000135960	0	0	0	0	0
ENSG00000135966	4.84171992	4.79652088	4.35176247	3.9080366	4.22128795
ENSG00000135968	0.82640358	1.35993618	1.12450911	1.0514448	0.7970705
ENSG00000135972	11.8162001	10.5460158	10.3692501	10.926961	12.7179931
ENSG00000135973	0	0.02502955	0.03654505	0	0.01042228
ENSG00000135974	6.42268088	7.53907566	6.29074771	6.7794976	5.66203082
ENSG00000135976	0.52529177	1.05968565	0.8204505	0.617092	0.46452119
ENSG00000135999	8.13778115	11.3587433	9.32836838	6.775057	6.58006738
ENSG00000136002	0.60517269	0.75927794	0.4238778	1.4417547	1.46697177
ENSG00000136003	2.74172126	2.68769618	2.77178413	2.7267914	2.33187067
ENSG00000136010	0.68789249	0.6196445	0.62944328	1.7252163	1.15733805
ENSG00000136011	0	0.00201474	0	0	0
ENSG00000136014	0.32480833	0.33637209	0.21627691	0.259058	0.142635
ENSG00000136021	14.1593097	12.2332507	13.3691182	10.201959	14.8171301
ENSG00000136026	21.887842	16.7490547	19.6466219	24.568419	21.0767866
ENSG00000136040	0.37068653	0.67428239	0.55622672	0.7375238	0.15812346
ENSG00000136044	1.27407459	2.20740451	1.66953875	1.930017	1.44286361
ENSG00000136045	8.83174601	9.19184184	9.11213993	10.160825	9.08900267
ENSG00000136048	1.34671009	1.3393116	1.69208504	1.3749212	0.93202652
ENSG00000136051	3.3961123	3.69690936	3.38493352	2.551127	3.65009841
ENSG00000136052	2.01163011	2.60486538	2.06258487	3.4120363	3.55185087
ENSG00000136059	0	0	0.0017484	0.0023109	0.00299175
ENSG00000136068	0.42300008	0.51840445	0.40648864	0.4676646	0.35674671
ENSG00000136098	0.67018999	0.74215767	0.54839057	0.5485108	0.59458461
ENSG00000136099	0.00851175	0.04275979	0.06659471	0.0825182	0.03561026
ENSG00000136100	5.53426473	6.05585965	5.4559897	3.265812	5.09799118
ENSG00000136104	7.91654724	6.6507548	7.75536345	7.8048776	8.00496929
ENSG00000136108	15.4752387	11.689402	13.1243678	9.8049199	13.4916922
ENSG00000136110	0	0	0.00879222	0	0
ENSG00000136111	0.30492822	0.34407554	0.28100965	0.3259361	0.32187293
ENSG00000136114	1.21863693	0.7894116	0.91345583	0.419716	0.96376934
ENSG00000136122	4.49392878	3.31378492	3.80053844	3.6837103	3.8991721
ENSG00000136141	2.65636657	2.33714933	2.46212266	1.3267047	2.48253514
ENSG00000136143	3.42649484	2.85807466	3.07537909	3.1555567	3.85447953
ENSG00000136144	2.810061	3.45868478	3.10765698	3.1418905	2.99943724
ENSG00000136146	6.79776195	6.64015436	6.91051366	6.1469769	6.19931698
ENSG00000136147	0	0.00522712	0.00572399	0.0025218	0.00326485
ENSG00000136149	0.05266297	0.14109798	0.10300688	0	0.08812958
ENSG00000136152	4.41990441	5.49227881	4.60082263	7.0331986	4.47193733
ENSG00000136153	0.97961594	0.8396866	0.81279285	0.9204181	0.91463931
ENSG00000136155	0.00282172	0	0	0	0
ENSG00000136156	47.8787239	47.2033923	52.0428348	43.668075	37.9070882
ENSG00000136158	61.9104146	48.824643	53.5151061	27.231505	53.9649284

ENSG00000136842	2.11730798	0.5350829	0.66026984	0.3929393	0.74137876	0.41893617	0.00283866	0	[illegible]
ENSG00000136848	1.16488403	1.12532229	1.14174223	1.219176	1.2073788	1.15834898	1.27407639	1.35691448	[illegible]
ENSG00000136854	0.83459061	0.73689266	0.67399541	0.6905992	0.78121106	0.62130439	1.00789685	1.00018437	[illegible]
ENSG00000136856	0.88821182	0.98307996	0.88491346	1.1174192	0.74849427	0.83907237	0.08963937	0.10002795	[illegible]
ENSG00000136859	2.72278486	2.17725568	2.32484195	2.9943064	2.62342908	2.70455387	6.67981913	6.5919928	[illegible]
ENSG00000136861	3.24237619	3.00686756	3.06864228	2.1218976	2.65588724	2.25864123	1.03255375	1.23834804	[illegible]
ENSG00000136866	1.68296788	2.32728478	1.95267739	2.159838	1.26686895	2.00903804	0.49169261	0.56426481	[illegible]
ENSG00000136867	0.91315101	0.62166996	0.73932438	0.6966025	0.74736179	0.68497728	1.50272252	1.60559076	[illegible]
ENSG00000136868	9.13679662	6.62243783	7.81669088	11.963849	10.7623753	8.90621645	8.5380607	8.63767828	[illegible]
ENSG00000136869	0.98039268	0.37140243	0.49512136	0.1246497	1.12664062	0.2069616	2.50826569	2.0911504	[illegible]
ENSG00000136870	4.82576902	4.35485894	4.17887929	4.2289882	4.21242296	4.29801411	1.92097413	2.03650847	[illegible]
ENSG00000136872	0	0	0	0	0.00673602	0	0.0096976	0	[illegible]
ENSG00000136874	1.62797576	1.62244374	1.47856964	1.5910198	1.57787389	1.67063697	1.55916546	1.60598908	[illegible]
ENSG00000136875	6.23880586	4.25118364	4.57487993	4.7586851	5.38586379	4.84189356	14.1871049	15.2846121	[illegible]
ENSG00000136877	1.23066054	1.11361467	1.10202589	1.1810009	1.16216582	0.94287892	1.55417421	1.59595613	[illegible]
ENSG00000136878	1.17630638	1.56992861	1.09537959	1.8000696	0.89058478	1.63681532	2.02346891	2.03572815	[illegible]
ENSG00000136881	0.01717698	0.00383514	0	0	0	0.01288488	0.02299067	0.01121666	[illegible]
ENSG00000136883	0	0	0	0	0.00296234	0	0.01279431	0.00520173	[illegible]
ENSG00000136888	43.2294381	41.8807017	47.8292199	38.838526	48.5765715	38.1986004	28.2707982	28.2494199	[illegible]
ENSG00000136891	3.37273724	2.70403989	3.02759865	2.6297348	3.28832126	2.78186831	7.09422352	7.9325366	[illegible]
ENSG00000136895	0.29697726	0.43707352	0.29345336	0.3214478	0.30495241	0.38310278	0.02393202	0.02717672	[illegible]
ENSG00000136897	6.90742931	6.43974019	6.69024751	5.3916099	6.10969763	6.00247554	6.022316	6.16463127	[illegible]
ENSG00000136908	3.98422504	2.94941018	3.40803433	3.0351313	3.99983351	2.8680861	8.50161276	9.07040607	[illegible]
ENSG00000136918	0	0.03448561	0	0.0332753	0.02871954	0	0	0.02521509	[illegible]
ENSG00000136925	1.77121462	1.73465406	1.56544304	1.6958184	1.51944728	1.58787744	1.78395669	2.02444316	[illegible]
ENSG00000136928	0.58844334	1.34945818	0.85347651	0.5865889	1.15442527	0.67333116	0.00400478	0.00488462	[illegible]
ENSG00000136929	0	0	0	0	0	0	0	0	[illegible]
ENSG00000136930	15.6134033	14.8398474	15.2190035	14.954695	17.1302254	12.9836742	25.1606035	26.239078	[illegible]
ENSG00000136931	0	0.01838058	0	0	0.00382683	0.00514609	0.01652803	0.00671973	[illegible]
ENSG00000136932	1.23075105	1.22470331	1.17767431	0.8641162	0.90633045	0.77377538	1.36525148	1.35296944	[illegible]
ENSG00000136933	1.78101155	1.44718496	1.52050339	1.8209751	1.65513166	1.60153467	2.26559574	2.56315011	[illegible]
ENSG00000136935	2.18585144	2.43229556	1.95935798	1.9106217	2.08786724	1.87856613	2.27349733	2.22371497	[illegible]
ENSG00000136936	2.79750401	3.61713672	3.53610481	3.2937741	2.75153515	3.46044173	2.17776756	2.13718576	[illegible]
ENSG00000136937	8.41841893	7.80123919	7.1761753	5.8693579	10.2759243	6.73381279	11.5484814	12.9025783	[illegible]
ENSG00000136938	10.452547	12.7919426	12.8552592	14.164639	14.594259	9.75585997	8.37387703	12.4543932	[illegible]
ENSG00000136939	0	0	0	0	0	0	0	0	[illegible]
ENSG00000136940	9.0212834	10.9752166	9.31877234	9.0272141	7.41398212	8.23212894	5.4861955	6.35195252	[illegible]
ENSG00000136942	81.4865119	77.2012948	85.639535	111.61924	79.7507937	92.6785509	124.210941	139.658664	[illegible]
ENSG00000136943	0.56846155	0.31277089	0.49637963	0.3149155	0.40203744	0.3807296	0.09782511	0.06960158	[illegible]
ENSG00000136944	0.01465559	0.02804728	0.04095116	0.0216504	0.01401463	0.02041654	0.01513226	0.02050753	[illegible]
ENSG00000136950	3.5905061	2.26863269	2.75386195	2.3534642	3.88586525	2.56184262	6.77496067	7.08924336	[illegible]
ENSG00000136960	0.18755438	0.5587091	0.94769489	0.1020783	0.04221582	0.16660564	3.36252995	3.06829618	[illegible]
ENSG00000136982	8.34319747	4.32904683	6.70729481	3.1345639	9.51659288	3.7101891	5.70504594	8.35011299	[illegible]
ENSG00000136986	3.29502929	3.04162419	2.97779613	3.7676384	3.88541865	3.58459782	7.08856119	7.29803883	[illegible]
ENSG00000136997	2.04381783	1.01877699	1.32867108	1.1673393	4.70395577	1.88668664	4.18604974	4.77593611	[illegible]
ENSG00000136999	2.13393254	0.35205464	2.28227778	1.4403243	2.02887753	1.21433618	3.2332511	2.56384619	[illegible]
ENSG00000137033	0.77612808	0.64103394	0.85605939	0.1961214	0.61523162	0.37645561	0.00468638	0	[illegible]
ENSG00000137038	8.06600668	6.47639717	8.12313386	8.9858589	9.11353647	7.09215194	8.15724143	8.83268344	[illegible]
ENSG00000137040	10.2307459	9.60928848	9.73980339	8.5846748	9.39869764	9.3349421	5.75123816	6.26743195	[illegible]
ENSG00000137054	5.53180725	4.05643537	4.25110215	5.5622896	4.48394719	4.92374583	10.4987538	12.3666878	[illegible]
ENSG00000137055	5.11859966	4.63319594	4.51099341	4.6142344	5.35767281	4.77832608	7.70500708	8.29254766	[illegible]
ENSG00000137070	0.01826876	0.06363085	0.03394638	0.1582172	0.01528605	0.12059387	0.40932587	0.48851691	[illegible]
ENSG00000137073	1.24674998	1.22109911	1.154236	1.1045341	1.00536191	1.07196871	1.29057128	1.4480082	[illegible]
ENSG00000137074	0.3730397	0.36610863	0.35177071	0.3997541	0.40128588	0.39498352	0.57056954	0.61020298	[illegible]
ENSG00000137075	2.29353484	2.80815222	2.31252936	2.2623562	1.91968365	2.36908675	0.73657645	0.76455026	[illegible]
ENSG00000137076	8.98344301	9.00734915	7.75187299	9.2596068	10.0531744	8.02965186	19.0648972	19.8577317	[illegible]
ENSG00000137077	0	0	0	0	0	0	0	0	[illegible]
ENSG00000137078	0	0	0	0	0	0	0.00847508	0.01033703	[illegible]
ENSG00000137080	0	0	0	0	0	0	0	0	[illegible]
ENSG00000137090	0.01801215	0.00402161	0	0	0	0.02251894	0	0.01764304	[illegible]
ENSG00000137094	1.72381195	1.25100363	1.42381348	1.2181891	1.61244871	1.19604414	0.35509743	0.40725388	[illegible]
ENSG00000137098	0.04023409	0.13025558	0.06120834	0.0866788	0.03117143	0.11066216	0.07000713	0.10728172	[illegible]
ENSG00000137100	4.59357821	3.88827727	3.87153106	4.6132107	4.11571792	3.7329037	1.8201252	1.95021739	[illegible]
ENSG00000137101	0.01230025	0.00411945	0.00300736	0.0437237	0.00514601	0.04382695	0.11359728	0.08433738	[illegible]
ENSG00000137103	0.8268879	0.92076147	0.6957945	1.0688492	0.55491165	0.91308661	0.28483485	0.27546294	[illegible]
ENSG00000137106	4.6192402	4.52895754	4.52992045	5.3324202	4.58620158	4.90095578	2.28562231	2.47230422	[illegible]
ENSG00000137124	9.72358424	6.2736137	7.44310411	9.4028721	12.0501252	8.68960045	8.72049188	10.8525859	[illegible]
ENSG00000137133	1.71934138	1.10218509	1.26885064	1.4589086	1.6710442	1.26598354	1.20287048	1.19850672	[illegible]
ENSG00000137135	0.69406252	0.51032691	0.53222576	0.5872299	0.57282504	0.56796389	0.14821351	0.18232067	[illegible]
ENSG00000137142	0.05897482	0.05925341	0.02883816	0	0	0.04423848	0	0	[illegible]
ENSG00000137145	2.37097888	2.83097248	2.49087429	2.5202551	2.34720388	2.6639988	1.20810177	1.26072746	[illegible]
ENSG00000137154	101.5784	115.656456	117.334341	146.7709	95.6321255	122.834406	195.070786	211.5656	[illegible]
ENSG00000137161	13.3440325	10.7738947	11.5338938	11.003494	9.93222046	8.87665973	15.8655223	18.1753653	[illegible]
ENSG00000137166	0.56730956	0.59767733	0.54506044	0.5693316	0.5072859	0.44052221	0.75435966	0.88937401	[illegible]
ENSG00000137168	12.9608254	10.0105037	11.9400534	8.4104399	12.9216361	8.24940857	14.073718	16.4598129	[illegible]
ENSG00000137171	0.54319149	0.81047064	0.62993633	1.1257668	0.54021968	0.94847497	0.60743427	0.58315596	[illegible]
ENSG00000137177	1.45187873	1.71377523	1.35624481	1.3395378	1.1265816	1.31111033	1.55320456	1.79730726	[illegible]
ENSG00000137185	0.70031118	0.96708908	0.8508347	0.8454168	0.70213473	0.9210471	1.27345715	1.4323578	[illegible]
ENSG00000137193	4.46533204	2.60014658	3.34753727	4.1932206	4.06174636	4.33575012	2.18033539	3.2232219	[illegible]
ENSG00000137198	1.3813979	0.7517918	0.99634947	1.4396546	0.93913604	1.12041387	3.48756898	4.04235711	[illegible]
ENSG00000137200	4.88942734	5.74722763	4.69638333	5.6840362	4.15312696	5.51045261	6.98943278	7.16052535	[illegible]
ENSG00000137203	0.02082662	0.01688684	0.02251206	0	0.00305725	0.00246672	0	0	[illegible]
ENSG00000137204	0	0	0.00257412	0.0034023	0	0	0	0	[illegible]
ENSG00000137207	6.34230591	5.87549724	5.91745785	8.398854	5.93234521	6.01024619	6.404171	6.41578658	[illegible]
ENSG00000137210	3.13994001	2.58301857	3.09582631	2.9433375	3.09436151	2.56820068	3.47794949	3.79095715	[illegible]
ENSG00000137216	1.99857204	1.74497058	1.78687865	2.0376352	1.96332203	1.97908171	2.72435916	2.6781793	[illegible]
ENSG00000137218	1.06366959	0.8970386	0.86777311	0.8263751	0.84850343	0.82268973	0.66389089	0.62080506	[illegible]
ENSG00000137221	0.77344314	1.01100171	0.73267442	0.9698154	0.67578384	0.86323342	1.17492437	1.2309547	[illegible]
ENSG00000137225	0	0	0	0.0045491	0	0	0.00565252	0	[illegible]
ENSG00000137251	0	0	0	0	0	0	0.03371925	0.03290181	[illegible]
ENSG00000137252	0	0	0.01715847	0	0	0	0.08453857	0.01718524	[illegible]
ENSG00000137259	360.675788	208.638651	280.046566	171.97049	332.054533	177.546503	31.0377664	46.7913748	[illegible]
ENSG00000137261	0.18807638	0.22718241	0.13640147	0.1406764	0.10078721	0.09907357	0.06448873	0.05174784	[illegible]
ENSG00000137265	0	0.00507117	0.00370215	0	0.00211163	0.0028396	0.00608008	0	[illegible]
ENSG00000137266	1.33624278	1.12062152	1.37672395	1.2245779	1.75094441	1.21863095	2.80193696	2.58330843	[illegible]
ENSG00000137267	59.3551012	32.3103839	46.5483498	28.861954	59.2945483	34.1194964	1.11601195	1.36119645	[illegible]
ENSG00000137269	1.61832498	2.19238716	1.76548263	2.8520198	1.96821905	3.05025259	1.20811239	1.44227435	[illegible]
ENSG00000137270	0.0466253	0.07026832	0.02279935	0.0301343	0.04551503	0.1049244	0.07488721	0.05708732	[illegible]
ENSG00000137273	0.01473696	0	0	0.0190492	0	0	0	0	[illegible]
ENSG00000137274	0.43200658	0.45063143	0.48762231	0.4414379	0.48386993	0.36760829	0.26328558	0.29604046	[illegible]
ENSG00000137275	1.81320976	1.67060095	1.82316806	1.6647139	1.95034297	1.25109348	3.66186201	3.36975534	[illegible]
ENSG00000137285	109.57948	81.6105979	97.629993	87.452608	97.4929996	91.9411765	0.00656426	0.08006413	[illegible]
ENSG00000137288	5.4478971	4.05671605	4.79943618	4.9212491	5.39624628	4.47006453	4.95762959	5.25995821	[illegible]
ENSG00000137309	8.08891715	6.79459076	6.49058975	8.6687786	7.26072303	8.3086662	25.9668092	30.7163466	[illegible]
ENSG00000137310	12.7044856	6.6725047	8.55062538	4.865787	14.9319271	5.61419378	2.50302493	3.86048179	[illegible]
ENSG00000137312	14.9783273	9.29456762	12.5980402	11.121639	17.8543474	10.0464588	10.5051074	11.05739	[illegible]
ENSG00000137331	0.09939781	0.04161139	0.01215116	0.0160604	0.04851544	0.01864019	11.823912	12.656923	[illegible]
ENSG00000137337	3.52153128	3.27257867	2.70294403	2.5735251	3.65604311	2.52816122	2.22493301	2.85033649	[illegible]
ENSG00000137338	2.28211868	1.96633451	2.05433588	1.6023347	1.87769622	1.57570219	1.46616965	1.47838147	[illegible]
ENSG00000137343	1.95900132	2.29629774	1.82245255	1.7487707	1.39696644	1.71180606	0.62482681	0.62090687	[illegible]
ENSG00000137364	4.39726723	2.79605536	3.12129317	3.3396665	5.23358404	2.68288094	7.2009934	8.89219774	[illegible]
ENSG00000137392	0	0	0	0	0	0	0	0	[illegible]
ENSG00000137393	0.09929063	0.18621803	0.07768357	0.1604309	0.14400472	0.15640879	1.04855218	0.95310847	[illegible]
ENSG00000137404	4.48744619	3.00214772	3.15623305	2.6648339	3.81274078	2.77914436	0.56559235	0.83522226	[illegible]
ENSG00000137409	15.5775796	12.5857111	14.0635675	15.958857	15.2060995	14.3336442	13.2188291	14.2997817	[illegible]
ENSG00000137411	0.59993623	1.00428043	0.57296777	0.9274676	0.46022372	1.13072132	0.95874521	1.04732231	[illegible]
ENSG00000137413	1.13448987	1.25973289	1.15581751	1.1947125	0.88006325	1.18203597	2.14004995	2.18909082	[illegible]
ENSG00000137414	11.4116846	12.8226294	11.342386	14.884549	9.91754593	12.3337015	12.1915785	11.9743243	[illegible]
ENSG00000137434	0.11399586	0.06247328	0.12162099	0.0937701	0.09249356	0.12437983	0.29128657	0.27407419	[illegible]
ENSG00000137440	0	0	0	0	0	0	0.03059444	0.01243865	[illegible]
ENSG00000137441	0	0.01337546	0	0	0.06683433	0.02995828	0.03207298	0.01955966	[illegible]
ENSG00000137449	0.46224952	0.45730363	0.48999222	0.3930997	0.55853932	0.42316561	0.68932312	0.64118598	[illegible]
ENSG00000137460	0.21711836	0.13881889	0.16649218	0.0861088	0.21607703	0.09808968	0.05151613	0.0483339	[illegible]
ENSG00000137462	0	0.01028	0	0	0	0.00575627	0.03697556	0.01503299	[illegible]
ENSG00000137463	0.45733014	0.6448989	0.77682115	1.1200786	0.48336345	0.9749976	1.140471	1.48533612	[illegible]
ENSG00000137473	0.00283737	0	0	0	0	0.00212839	0.01367175	0.00833769	[illegible]
ENSG00000137474	0.00473978	0.00370391	0.00386285	0.0061267	0.00660988	0.00533314	0.0044408	0.01083286	[illegible]
ENSG00000137478	2.39506524	3.06966455	2.58082265	3.1476875	2.1706655	3.0378839	0.99318239	1.11412983	[illegible]
ENSG00000137486	0.17822487	0.27307681	0.13944058	0.2476547	0.18019445	0.18883841	1.63881452	1.7871878	[illegible]
ENSG00000137491	0.00300997	0.00336021	0.00588739	0.0077815	0.00111935	0.00376309	0.01450338	0.00393105	[illegible]
ENSG00000137492	4.14856642	4.2446597	4.05722345	3.742082	4.98981204	3.82119487	3.13538131	3.71235042	[illegible]
ENSG00000137494	0.2125115	0.24821895	0.17176234	0.158915	0.21063504	0.17390199	1.14245235	1.05970687	[illegible]
ENSG00000137496	0.19444025	0.27293598	0.1967733	0.2054405	0.13958705	0.21941585	0.43043107	0.46206227	[illegible]
ENSG00000137497	8.30990986	9.17912633	7.38217626	7.5169457	6.6052794	8.09424147	4.55523267	4.56262504	[illegible]
ENSG00000137500	3.6163711	3.09446933	3.7246764	2.9047956	3.5522148	3.04506434	2.16369494	2.11490171	[illegible]
ENSG00000137501	0.19792671	0.13494185	0.15110215	0.1135631	0.15716203	0.15718099	0.04095394	0.05044597	[illegible]
ENSG00000137502	0.95965915	1.17305303	1.31449817	1.0895536	0.64651202	1.0797963	0.62431787	0.59551535	[illegible]
ENSG00000137504	2.26892595	3.59484321	2.35563888	2.6669669	1.98388168	3.1801661	3.8031182	3.69431097	[illegible]
ENSG00000137507	0	0	0	0	0.00286715	0.00192778	0.37768674	0.36248922	[illegible]
ENSG00000137509	6.60103896	5.55452908	6.95956525	8.334868	5.78225077	8.0631732	3.17728782	3.5480785	[illegible]
ENSG00000137513	1.36769015	1.40125438	1.40880828	1.6946726	1.51231029	1.52373099	2.01148908	2.32657519	[illegible]
ENSG00000137522	0.69517865	0.76865976	0.67303935	0.6208917	0.73952049	0.65773452	1.1600451	1.12405276	[illegible]
ENSG00000137547	20.9288852	14.5579913	19.1229251	18.804177	26.4043144	17.548985	11.4736544	12.3922498	[illegible]
ENSG00000137558	6.20457114	57.2711234	16.0909274	34.167402	9.72535109	28.3585821	0.06991082	0.08914593	[illegible]
ENSG00000137561	0.45497916	0.51066593	0.66142917	0.5404288	0.54189102	0.37818753	0.11850237	0.13249224	[illegible]
ENSG00000137563	7.51163855	4.28785576	5.62988291	5.3843912	7.79055688	5.678285	2.4537169	3.00832469	[illegible]
ENSG00000137571	0.05945171	0.06204776	0.07166426	0.1912271	0.08098047	0.1773477	0.02331692	0.02573105	[illegible]
ENSG00000137573	0.0062187	0.02737252	0.0104259	0.0103351	0.01040677	0.0179928	0.6799084	0.82580152	[illegible]
ENSG00000137574	4.87038652	4.90203871	4.72107324	3.9096881	4.40880859	4.16009726	7.29737942	8.56344825	[illegible]
ENSG00000137575	12.8151999	11.4489906	12.6830499	9.8034459	11.9421925	9.9791101	8.01263805	7.32291353	[illegible]
ENSG00000137601	0.44282016	0.61239612	0.54508831	0.5063001	0.43584085	0.45874806	0.66442203	0.72224197	[illegible]
ENSG00000137628	2.37328731	2.88856752	2.42542662	2.589421	1.76052902	2.44485733	16.4561248	16.3631929	[illegible]
ENSG00000137634	0	0	0	0	0	0	0	0	[illegible]
ENSG00000137642	0.21609254	0.11489265	0.08223137	0.1271634	0.18245314	0.12299142	0.00607722	0.00905956	[illegible]
ENSG00000137648	0.00601526	0.00402911	0.00705937	0.0077754	0.00335544	0.00270732	0.0048307	0.01060558	[illegible]
ENSG00000137656	7.60036813	5.98743093	6.37298752	5.0050236	6.28662456	5.04979651	3.87509272	4.84795774	[illegible]
ENSG00000137672	0.01638642	0.00470395	0.02289374	0.0151295	0.0300337	0.0351196	0.01127959	0.00458589	[illegible]
ENSG00000137673	0.02749196	0	0	0	0.03578307	0	0.02943747	0.0089762	[illegible]
ENSG00000137674	0	0	0	0.0264512	0.01141488	0.02302508	0	0	[illegible]
ENSG00000137675	0	0	0	0	0	0	0	0	[illegible]
ENSG00000137691	1.67759288	1.67925159	1.93951121	1.8863398	1.5689385	1.72153429	1.06175953	1.15452694	[illegible]
ENSG00000137692	1.73281706	1.68484113	1.78470304	1.7170908	2.02858081	1.53414373	4.8480913	5.33025604	[illegible]
ENSG00000137693	4.33133647	4.87002396	4.28449711	3.4939492	3.33462748	3.50175746	5.01676357	5.63033792	[illegible]
ENSG00000137699	0	0	0	0	0	0	0.00236319	0.00288238	[illegible]
ENSG00000137700	0.40015227	0.36496091	0.36949498	0.448194	0.4328824	0.42256121	0.75580168	0.87333068	[illegible]
ENSG00000137707	0	0	0	0	0	0	0	0.00997135	[illegible]
ENSG00000137709	0	0.00165223	0	0.006377	0	0.00185032	0.02971402	0.05798736	[illegible]
ENSG00000137710	8.50128	8.37011591	7.88078774	6.3709583	7.7178601	6.97323766	3.65065364	4.48419863	[illegible]
ENSG00000137713	0.891521	0.7422383	0.80895569	0.5396779	0.95784591	0.68641278	2.14409274	2.28459921	[illegible]
ENSG00000137714	3.98096599	3.19847022	3.68684965	3.846403	4.26749502	3.73277699	4.98927874	5.02193895	[illegible]
ENSG00000137720	0.72954642	0.55381666	0.7821891	0.6426534	0.57275363	0.62021771	2.40861929	2.98013221	[illegible]
ENSG00000137726	7.3718323	8.59898515	8.49448386	8.230858	3.88599115	7.55871225	0.0027478	0.01005445	[illegible]
ENSG00000137727	0.00166296	0.00111387	0.00081317	0.0021496	0.02087172	0.00561341	0.0120193	0.0146599	[illegible]
ENSG00000137731	0.03174171	0.01328819	0.02716247	0.0718023	0.00663983	0.06845446	0	0	[illegible]
ENSG00000137745	0.01539882	0.04125751	0	0	0.03006427	0.0288776	0.4451907	0.59578922	[illegible]
ENSG00000137747	0	0	0	0	0	0	0.00381247	0.00232503	[illegible]
ENSG00000137752	0.14836369	0.1713735	0.21764553	0.1428546	0.11654019	0.11696942	0.3501467	0.32771912	[illegible]
ENSG00000137757	0	0	0	0.0038221	0	0	0	0	[illegible]
ENSG00000137760	0.87712275	0.72227478	0.94624435	0.7831289	0.98990471	0.71730289	1.42331401	1.51236746	[illegible]
ENSG00000137764	0.99662193	1.11851523	1.02659826	1.4442127	0.7053534	1.16573496	0.85656174	0.98329016	[illegible]
ENSG00000137766	0	0	0	0	0	0	0.00370643	0.00226036	[illegible]

ENSG00000137767	0.11839496	0.06461711	0.1629611	0.1700437	0.10273391	0.11841467	0.7430327
ENSG00000137770	3.85167575	3.68475113	3.60874208	3.2516539	4.12230119	3.26437799	1.4554975
ENSG00000137776	2.42944372	2.91304874	2.62030193	1.8379915	2.45997431	2.04332762	2.65819012
ENSG00000137801	0.1032429	0.09771723	0.15364954	0.0435174	0.08012673	0.05892559	65.3651185
ENSG00000137802	0.47664199	0.66028361	0.53315237	0.5703076	0.49650897	0.64330799	0.54831813
ENSG00000137804	19.8075012	14.6982063	16.0664251	12.139613	18.2591331	13.1508673	1.74058128
ENSG00000137806	2.53826491	2.10784028	2.27269438	2.7803602	2.49614752	2.45394931	2.0828508
ENSG00000137807	4.61684831	3.21513595	3.63935975	2.5607673	4.1049593	2.92969307	1.39480487
ENSG00000137808	0	0	0	0	0	0	0
ENSG00000137809	0.00492584	0.0049491	0.00722607	0	0	0.00554249	3.72637702
ENSG00000137812	5.72400033	4.86929256	4.99191645	3.6800326	5.0489684	4.08795028	0.90969959
ENSG00000137814	3.72765672	2.63909001	3.23753033	2.7095604	3.22775624	2.4157588	1.40449805
ENSG00000137815	14.9153812	14.5652565	13.6403516	13.018632	13.5100084	12.9848014	9.19052097
ENSG00000137817	0.98659559	1.35478026	1.04946181	1.2544038	0.83771056	1.3168681	1.13656042
ENSG00000137818	65.9918533	57.2067428	67.3548851	79.728376	59.3662078	66.4234224	103.405648
ENSG00000137819	0.00852413	0.0042822	0.00416822	0.0027546	0.00356621	0.00479562	1.16544895
ENSG00000137821	0.69230639	0.83924744	0.68900136	0.8148655	0.61478038	0.72534221	0.19536108
ENSG00000137822	0.68948391	0.72464698	0.62376965	0.6386861	0.75840893	0.57170478	0.68208425
ENSG00000137824	2.24775985	1.96776481	2.089889	2.1649294	2.12512707	1.81157781	0.86645729
ENSG00000137825	2.47613862	1.71740891	1.88261356	1.6622982	2.67337324	1.88137978	0.18602327

[illegible]

ENSG00000138668	3.29347907	3.87139926	3.48884861	2.81579	4.67520681	2.89605313	2.23359536	3.39744902	4.74297059	3.92103153	5.55200318	6.86711889	4.21711872	3.92791118	3.4718693	3.76972998	3.93050991
ENSG00000138669	0	0.00718737	0	0	0	0.00321965	0.02757533	0.03573564	0.02440301	0.00777499	0.02703672	0.01993594	0.29789918	0.23481755	0.24823562	0.49479401	0.41970168
ENSG00000138670	0.01402425	0.02952296	0.04310576	0.0051794	0.00670545	0.01202279	0.01448039	0.00981205	0.00594297	0.00362917	0.03028812	0.01184347	0.02713204	0.00868173	0.01742407	0.01739426	0.00904182
ENSG00000138674	1.73860492	1.7849738	1.5621921	2.9872202	1.74617433	1.91557401	2.57563045	2.8464487	2.70345853	2.84052729	2.43217437	2.35822913	2.95734554	3.34050536	3.4591397	3.05891783	3.03516925
ENSG00000138675	0.02322195	0.05910683	0.05904768	0.0480273	0.02720276	0.05922582	8.87131064	10.4631976	9.38205273	17.2593875	7.3402116	7.71885663	0.43634737	0.53333361	0.48874434	0.93414976	0.82444802
ENSG00000138678	0.05683541	0.01359616	0.08337602	0.0419808	0.05887887	0.01522631	1.64641149	1.8729228	1.40293988	0.93026517	0.81320009	1.10051329	0.66317641	0.21110412	0.13416596	0.70492893	0.17100219
ENSG00000138684	0	0	0	0	0	0	0	0	0	0	0	0	0	0	0	0	0
ENSG00000138685	1.49729903	1.88831602	1.73353191	1.6695561	1.05963934	1.74713971	6.32800895	6.17756575	4.82584311	7.27930878	4.6186522	3.51751977	7.26089794	8.39223031	8.34983742	16.6588577	10.6344421
ENSG00000138686	1.97892159	1.9907518	1.98608928	2.1603975	2.06306521	2.07654571	2.85702851	2.85310561	2.95523883	3.35982494	3.3801439	3.13897259	3.90514749	3.85410048	3.88044532	4.72250011	4.64392273
ENSG00000138688	1.25323795	1.72953307	1.38779011	1.5130694	1.12001596	1.55319739	1.35549566	1.37326784	1.19912037	1.78166356	1.84374496	1.27662739	1.6357966	2.23909465	2.13939836	1.50519918	1.88782528
ENSG00000138696	4.61794385	7.9091752	6.51906262	6.8155759	3.49419492	6.11867231	1.91488523	2.11580753	2.63601998	3.70963497	1.79621656	2.03777501	0.41089414	0.13921218	0.25367403	0.52340238	0.31912308
ENSG00000138698	2.20081275	1.69386436	1.84731781	1.9237641	2.63005763	2.09865021	1.69184052	1.83044712	1.92288688	2.07612759	1.67340342	1.85404325	1.93447934	1.87446541	1.90936233	2.58477975	2.42294685
ENSG00000138709	1.03066329	1.05026118	1.1388754	1.2494503	1.13811867	1.16686111	0.51136181	0.615278	0.65230097	0.63387609	0.65886231	0.70325927	0.78820242	0.86072326	0.93544514	0.95459735	0.89318681
ENSG00000138722	0.00758838	0.00254141	0	0.0024522	0.00952416	0.00142306	0	0	0	0.00171824	0.00239	0.00160209	0.00160572	0	0	0	0
ENSG00000138735	0.73200285	0.49609814	0.51436665	0.2887025	0.31347922	0.39452587	0.85053544	0.82144789	0.58558746	1.09367509	0.54460248	0.36993201	0.3744279	0.46675042	0.44362915	0.24603912	0.36200802
ENSG00000138738	0.7713707	0.87337755	0.80804599	0.7446861	0.61932522	0.77593767	0.3602766	0.40307271	0.43880222	0.41948108	0.38017727	0.56147667	0.45620755	0.54195273	0.44630332	0.52617932	0.4879597
ENSG00000138741	0.54115239	0.44599145	0.50728739	0.451292	0.46740592	0.3965786	0	0.00732806	0.00493163	0.00225868	0.00628346	0.00842401	0.16463995	0.06483889	0.10410434	0.09382235	0.07127974
ENSG00000138744	0.23806392	0.25001594	0.27018877	0.5090766	0.24100218	0.31526674	0.47441742	0.43470384	0.37924508	0.34605661	0.52207989	0.38719739	0.27861683	0.18785599	0.21471164	0.25517193	0.28297052
ENSG00000138750	2.41423023	2.21214527	2.25742434	2.0065796	2.12221867	1.9512691	2.03268135	2.40139698	2.80786693	2.07369225	2.21485684	3.02597614	2.74391947	2.44658395	2.58491169	3.2746657	2.79483502
ENSG00000138755	0	0.00787883	0	0	0	0	0.06612418	0.04608655	0.01163073	0.01065372	0.01481889	0.01986715	0	0	0	0	0

[illegible]

ENSG00000139865	0.00160819	0.00969472	0.01258225	0.0041575	0.00807374	0.00482537	0.00258299	[illegible]	[illegible]
ENSG00000139874	0.02251204	0.01005261	0.00733879	0	0.0041859	0.01125789	0	[illegible]	[illegible]
ENSG00000139880	1.20985485	1.16612052	1.1616641	0.8574271	1.09407228	1.11252442	0.71498726	[illegible]	[illegible]
ENSG00000139890	0.05942792	0.02653717	0.07749258	0.1280292	0.01105005	0.16345398	0.07158759	[illegible]	[illegible]
ENSG00000139899	0.21037689	0.22546204	0.20574482	0.1767589	0.15842616	0.17358983	0.29565967	[illegible]	[illegible]
ENSG00000139908	0.0046866	0.00941747	0	0	0.00261428	0.01757767	0.00376369	[illegible]	[illegible]
ENSG00000139910	2.86042128	4.52151116	3.22276714	2.2642629	2.44170074	2.3863188	0.11965354	[illegible]	[illegible]
ENSG00000139914	0.01616336	0.02165295	0	0.020893	0.00901627	0.04849815	0.01298039	[illegible]	[illegible]
ENSG00000139915	0.24244841	0.30793081	0.33687786	0.0548009	0.14041611	0.07851071	0.00638374	[illegible]	[illegible]
ENSG00000139921	20.5863833	19.0052112	19.7355623	13.541605	20.5472456	14.5406464	7.94674758	[illegible]	[illegible]
ENSG00000139926	2.29623957	2.01631294	1.66411784	2.0097186	2.56885288	2.21210958	1.38431149	[illegible]	[illegible]
ENSG00000139946	3.09344968	3.60743938	3.02969123	3.7339864	4.27301293	4.09004329	2.35558987	[illegible]	[illegible]
ENSG00000139970	3.01717558	2.06921357	2.24388631	3.1025785	6.0966413	3.44555763	0.00723289	[illegible]	[illegible]
ENSG00000139971	5.18203659	9.03327265	7.55325375	8.5789414	4.61123982	7.15506386	0.120938	[illegible]	[illegible]
ENSG00000139973	0	0.02115433	0.02316519	0.0255149	0.02642592	0.01184534	0.01902221	[illegible]	[illegible]
ENSG00000139974	0.23801524	0.33728355	0.35387126	0.341452	0.28626126	0.30651284	0.32925391	[illegible]	[illegible]
ENSG00000139977	2.65733452	2.7419165	2.70165665	2.5236742	2.27813045	2.68884808	1.78860057	[illegible]	[illegible]
ENSG00000139985	0.05091586	0.03836728	0.04357045	0.1069489	0.03905268	0.07638651	0.5315602	[illegible]	[illegible]
ENSG00000139988	0	0.00349264	0	0	0	0	0.01256248	[illegible]	[illegible]
ENSG00000139990	3.12798136	3.85530866	3.30722171	3.5010716	3.01288948	3.3941365	1.77460863	[illegible]	[illegible]
[illegible]	[illegible]	[illegible]	[illegible]	[illegible]	[illegible]	[illegible]	[illegible]	[illegible]	[illegible]

ENSG00000141098	2.29934644	2.248342	2.02289674	1.7877028	2.07307897	1.67556913	1.76261328	[illegible]
ENSG00000141101	4.75668277	4.49498647	4.73996019	7.4862969	4.82269547	5.75236557	5.84352502	[illegible]
ENSG00000141127	1.11718768	1.13920574	1.1952489	1.3067934	0.95918447	1.16893035	0.59310572	[illegible]
ENSG00000141140	0.32757314	0.36605699	0.25999118	0.4212988	0.3374486	0.39512885	1.63391585	[illegible]
ENSG00000141141	1.7647297	1.81647914	1.72085323	1.4864602	1.46694064	1.54712334	2.56643846	[illegible]
ENSG00000141150	4.64556541	7.79129104	6.32877071	9.5448104	4.76433569	8.05425668	5.28400052	[illegible]
ENSG00000141161	0	0.00086452	0	0	0.00143995	0	0	[illegible]
ENSG00000141179	1.29601527	0.64672822	0.89568562	0.7399026	1.7073549	0.88629798	0.70947678	[illegible]
ENSG00000141194	0	0	0	0	0	0	0	[illegible]
ENSG00000141198	0.52225401	0.56937813	0.52162217	0.656402	0.44442468	0.53845574	1.01282824	[illegible]
ENSG00000141200	0	0	0	0	0	0	0	[illegible]
ENSG00000141219	1.8427909	1.95933221	1.70436655	1.820025	1.62868365	1.72756296	1.58654521	[illegible]
ENSG00000141232	7.62688498	10.4761786	8.98512091	12.121129	9.15978717	11.3209551	3.39271941	[illegible]
ENSG00000141252	0.6345567	0.88124851	0.66155277	0.7340033	0.71096755	0.61798113	1.18739725	[illegible]
ENSG00000141255	0	0	0	0	0	0	0.00174684	[illegible]
ENSG00000141258	2.4691665	3.00047697	2.43205728	2.4390016	2.23500072	2.65429589	3.33668066	[illegible]
ENSG00000141279	1.65908202	2.1283389	1.71395055	1.7721864	1.74667924	1.8993003	1.39503932	[illegible]
ENSG00000141293	0	0	0	0	0	0.00298473	0	[illegible]
ENSG00000141294	0.02869542	0.03844129	0.0140318	0.0432742	0.06135985	0.0287002	0.11906361	[illegible]
ENSG00000141295	0.83954329	1.14220627	0.95541325	1.9251867	0.76025182	1.36421236	0.75477177	[illegible]
ENSG00000141298	1.71014359	1.41019335	1.47010441	1.4786605	1.78846428	1.53938549	0.982135	[illegible]
ENSG00000141314	2.84075511	4.31518571	2.72097023	5.6243985	2.6268254	4.55902286	0.00180772	[illegible]
ENSG00000141316	0.04578088	0	0.09850033	0.0118354	0	0	0	[illegible]
ENSG00000141337	0.04470144	0.05988347	0.05191418	0.039725	0.0171431	0.05448895	0.19968598	[illegible]
ENSG00000141338	0.80195874	0.73928659	0.71627512	0.2155229	0.23793981	0.29618075	0.84693609	[illegible]
ENSG00000141349	2.71666071	2.54987028	2.42023791	2.65254	2.75603214	2.38111809	3.35445285	[illegible]
ENSG00000141367	19.8984292	16.973043	17.0113374	15.258217	24.5681258	14.7990576	28.6745857	[illegible]
ENSG00000141371	0	0	0	0.0206753	0	0	0.01284511	[illegible]
ENSG00000141376	0.27625684	0.41521442	0.31630153	0.3536104	0.18642577	0.31084069	0.34685122	[illegible]
ENSG00000141378	0.90872144	0.81507828	0.92868997	0.8819875	1.05415019	0.84911986	3.80541651	[illegible]
ENSG00000141380	2.96387237	2.8272035	2.76541963	2.3636254	2.94669234	2.33464744	1.84768944	[illegible]
ENSG00000141384	0.04440814	0.04957547	0.0641115	0.1339397	0.03656791	0.09517615	0.94252381	[illegible]
ENSG00000141385	11.3749556	8.71158407	9.69754809	11.771438	13.0762886	10.9858096	7.35374496	[illegible]
ENSG00000141391	0.30106597	0.33399732	0.34657702	0.4337545	0.24491397	0.47990378	0.15111157	[illegible]
ENSG00000141401	0.43541529	0.32181855	0.36709339	0.5628251	0.41206513	0.45951469	0.8850295	[illegible]
ENSG00000141404	0.3214208	0.30616306	0.44089775	0.2697883	0.45755245	0.347568	0.0419034	[illegible]
ENSG00000141424	15.3257305	14.7332176	14.2165249	12.956081	14.8532229	13.2318931	7.18331997	[illegible]
ENSG00000141425	5.20528586	5.56744665	5.35127985	5.1816475	6.31040945	5.24919225	3.31893303	[illegible]
ENSG00000141428	3.58645689	3.26319326	3.81836626	3.5640266	3.22254967	3.87733584	2.44178233	[illegible]
ENSG00000141429	14.7709281	13.0375402	14.0890851	10.276806	15.9643942	10.7816441	10.2848282	[illegible]
ENSG00000141431	0.77177261	1.23817566	0.82199576	1.1438231	0.6805112	1.25666646	0.0126488	[illegible]
ENSG00000141433	0	0	0	0	0	0	0	[illegible]
ENSG00000141434	0.00568827	0	0.00556303	0.0073528	0.00317304	0.01706765	0	[illegible]
ENSG00000141437	0	0	0	0	0.00812401	0	0	[illegible]
ENSG00000141441	1.78941142	1.9710351	1.92046797	1.3179726	1.78893125	1.59069134	0.23794998	[illegible]
ENSG00000141446	5.5865753	5.71466106	5.77650419	5.3431718	4.99046823	5.24134788	3.28813164	[illegible]
ENSG00000141447	3.44840414	3.40402061	3.42023739	4.6101877	3.66879936	4.21983832	1.30685899	[illegible]
ENSG00000141448	0	0	0	0	0	0.00414902	2.27424765	[illegible]
ENSG00000141449	0.65374072	1.02622767	0.8212298	0.8918671	0.56676621	0.98620968	0.04202608	[illegible]
ENSG00000141452	1.73734367	1.17163826	1.30966762	1.2622598	1.93825458	1.38145374	0.87177267	[illegible]
ENSG00000141456	1.88142342	1.81199288	1.42313994	1.9180878	1.63354073	1.79004187	1.78664603	[illegible]
ENSG00000141458	2.59901763	1.97156894	1.8479664	1.981309	2.80848461	2.08237384	5.80648705	[illegible]
ENSG00000141469	0.01303687	0.00349292	0.00127498	0.0050555	0.00799947	0.00195586	0.68052262	[illegible]
ENSG00000141480	1.08910537	1.23389722	1.19294056	1.2790831	0.93732608	1.26629579	0.55976519	[illegible]
ENSG00000141485	0.01341664	0.02696003	0.02478454	0.0134887	0.01330504	0.0257195	0.02514065	[illegible]
ENSG00000141497	0.09032999	0.12273761	0.09086514	0.1034178	0.13100878	0.07937456	0.05388815	[illegible]
ENSG00000141499	0.86984911	0.7834681	0.81660074	1.0500236	0.89167748	0.92316991	0.58096168	[illegible]
ENSG00000141503	1.23773171	1.35174201	1.0693156	1.3305397	1.26059396	1.36561139	1.93581955	[illegible]
ENSG00000141504	4.04817987	3.98197454	3.85384176	6.2097683	3.45830239	5.13468287	2.30297499	[illegible]
ENSG00000141505	0.13241757	0.08402721	0.13290995	0.220713	0.12051713	0.16990609	0.16790719	[illegible]
ENSG00000141506	0	0	0	0	0	0	0.0170276	[illegible]
ENSG00000141510	2.39353608	2.05245516	2.04103033	2.3152525	1.83697251	2.19510949	5.94072374	[illegible]
ENSG00000141519	0.11729858	0.2226106	0.1325606	0.1768925	0.11414187	0.17108891	0.19467904	[illegible]
ENSG00000141522	13.3818984	7.64131658	9.19505181	6.7144801	15.4106371	6.85945106	9.70285169	[illegible]
ENSG00000141524	0.01395348	0.00810009	0.00636825	0.0132268	0.00570793	0.00976906	0.07993389	[illegible]
ENSG00000141526	0.07550692	0.00871995	0.01782451	0.0269246	0.17356085	0.05663962	1.38421254	[illegible]
ENSG00000141527	0.0024044	0.0016105	0.00940583	0.0108779	0.00737673	0.00811619	0.00772364	[illegible]
ENSG00000141540	0.95105809	1.95684579	1.57575796	2.327925	1.06386282	1.92773118	0.86330477	[illegible]
ENSG00000141542	0.40854921	0.53201761	0.46651811	0.4749928	0.28009742	0.45883835	0.13197141	[illegible]
ENSG00000141543	8.03435791	6.38032372	7.02414361	6.792007	7.79501214	6.94024172	5.60824519	[illegible]
ENSG00000141551	1.47983837	1.75542602	1.4562136	1.9036548	1.62826133	1.77350596	2.03600864	[illegible]
ENSG00000141552	4.12464547	3.0135344	3.79511713	4.2861245	4.09969704	3.59363191	1.36050889	[illegible]
ENSG00000141556	2.27038767	2.25658438	2.06664174	1.9867882	2.27474457	1.82027506	0.86106396	[illegible]
ENSG00000141560	4.54443983	3.38907724	3.91595418	3.2331332	3.79848583	2.9847983	1.41095818	[illegible]
ENSG00000141562	1.45817915	1.2454475	1.37894704	1.7711249	1.43806444	1.93460925	0.8076048	[illegible]
ENSG00000141564	0.80158144	1.04491326	0.82111878	0.8940008	0.85357725	0.76702531	1.56805995	[illegible]
ENSG00000141568	5.81285327	4.73613408	4.62543122	4.5432818	6.04061404	4.23965345	4.56729117	[illegible]
ENSG00000141569	1.47141064	1.52405013	1.29606485	1.8452889	1.28281502	1.49114775	4.53488625	[illegible]
ENSG00000141570	4.54564613	4.47057803	4.55398857	4.5716422	4.26423493	4.69887379	1.31328747	[illegible]
ENSG00000141574	0.00891556	0.01492945	0.00435963	0	0.00497329	0.00334389	0.83770358	[illegible]
ENSG00000141576	13.718412	11.7757127	12.0985546	6.6426568	12.0495068	6.48138384	1.17409357	[illegible]
ENSG00000141577	1.23784457	1.50611145	1.28291518	1.201751	1.03427076	1.06722433	0.66460662	[illegible]
ENSG00000141579	0	0	0	0	0.00344019	0	0	[illegible]
ENSG00000141580	12.6090428	8.9553588	9.87738946	11.770826	12.773976	11.6897767	5.55181098	[illegible]
ENSG00000141582	11.5170399	10.3576209	10.1869118	9.6504926	11.0569046	9.240256	3.9810583	[illegible]
ENSG00000141622	1.18248525	1.16477553	1.2435709	1.4117727	1.06872678	1.60650706	0.00392002	[illegible]
ENSG00000141627	2.42150013	2.3287575	2.40397482	3.2562207	2.38737104	2.95264785	1.70789211	[illegible]
ENSG00000141639	1.24773927	0.79609257	1.18924287	0.8638308	1.45691386	0.8978873	0	[illegible]
ENSG00000141642	1.73054586	1.85553384	1.85643916	1.0317635	1.52657679	1.19246411	1.03962503	[illegible]
ENSG00000141644	0.95199726	1.00530797	0.83830353	0.8887822	0.79974008	0.84011353	0.89375891	[illegible]
ENSG00000141646	3.15701953	3.97530558	3.25951444	4.0820794	2.73723513	3.76589299	1.3630009	[illegible]
ENSG00000141655	0	0	0	0	0	0.00188407	0.21380818	[illegible]
ENSG00000141664	0.50368981	0.51336614	0.57908654	0.5417366	0.43110496	0.52684728	0.82032312	[illegible]
ENSG00000141665	0.0909049	0.10324749	0.10243213	0.0970695	0.06062995	0.08746112	0.05395906	[illegible]
ENSG00000141668	0.03066313	0.0074686	0.02180945	0.0108097	0.0342091	0.03554728	0.00223862	[illegible]
ENSG00000141682	4.31978077	2.49547275	4.09117478	3.383505	4.74766844	3.44515705	18.0548329	[illegible]
ENSG00000141696	3.22768822	2.57034973	2.82159511	6.4593385	3.1170802	3.6069999	6.11800407	[illegible]
ENSG00000141698	3.38553176	3.01024271	3.17774658	3.1024184	2.99102691	2.73836935	2.33514082	[illegible]
ENSG00000141699	4.54336636	4.47931627	4.28359112	5.3704861	3.06849272	4.57416659	3.4677377	[illegible]
ENSG00000141720	20.3836541	21.2706027	19.4960431	19.496948	18.2497723	19.8732825	13.5206628	[illegible]
ENSG00000141736	1.22286435	1.4325279	1.30811261	0.9473525	0.75038655	0.92171318	0.87954787	[illegible]
ENSG00000141738	0	0	0.00172213	0.0045523	0	0.00264179	0.0084848	[illegible]
ENSG00000141741	3.01880601	2.37684913	2.96239713	3.6894351	3.21586495	2.96658645	2.42432711	[illegible]
ENSG00000141744	0.03514694	0.00784732	0.02291538	0.0302877	0.02614095	0	0	[illegible]
ENSG00000141748	0	0.03453465	0.01120516	0.007405	0	0	0.02300293	[illegible]
ENSG00000141750	1.10246037	1.07305351	1.34211749	1.2469264	1.3452597	1.29646805	0.06916861	[illegible]
ENSG00000141753	9.4871717	7.24698709	7.78929119	10.885657	9.01310382	10.1145139	36.4133403	[illegible]
ENSG00000141756	7.60189514	8.25847154	7.14452632	13.761199	7.39652932	10.0163691	21.1126667	[illegible]
ENSG00000141759	5.60728684	4.18402577	4.95379061	5.043114	6.4650274	5.06876799	2.90160702	[illegible]
ENSG00000141837	0.00220601	0.00073881	0.00215743	0	0.00061528	0	1.14001652	[illegible]
ENSG00000141854	0.12618869	0.15367852	0.14023895	0.2224277	0.13438232	0.26676142	0.01842527	[illegible]
ENSG00000141858	14.3067572	13.1208657	13.2287526	10.275489	13.4911776	10.8030311	12.115937	[illegible]
ENSG00000141867	1.58859493	1.88366977	1.65719179	1.5290263	1.95044343	1.72085697	2.31599616	[illegible]
ENSG00000141873	1.73866284	1.24500277	1.54382421	1.2185525	1.80082006	1.06071644	2.29974929	[illegible]
ENSG00000141905	2.18889047	2.12680836	1.87900428	1.8259361	2.63901245	1.95585886	3.1433778	[illegible]
ENSG00000141933	1.54025041	1.39793201	1.60424915	1.5330342	1.10834796	0.95318861	1.70697213	[illegible]
ENSG00000141934	1.4823061	0.79350021	1.24590004	1.1311642	0.98293623	0.98688009	9.48019507	[illegible]
ENSG00000141946	0.0245373	0.03287094	0	0.0158587	0.02737485	0.02760906	0.01970529	[illegible]
ENSG00000141956	0.17928169	0.18464793	0.16166574	0.1843973	0.17581901	0.16918912	0.27479408	[illegible]
ENSG00000141959	4.02173699	2.48514212	2.8456916	3.5725094	4.22723029	3.48567743	1.99214166	[illegible]
ENSG00000141965	12.8700964	11.7992633	11.3895495	10.881203	12.8290445	10.2642481	14.3554441	[illegible]
ENSG00000141968	0.01885331	0.01262825	0.01106292	0.009748	0.0094651	0.03677008	0.01362656	[illegible]
ENSG00000141971	1.21811679	1.06968244	1.11859913	1.7016387	1.05267676	1.35621995	3.78586169	[illegible]
ENSG00000141977	0	0.0101495	0.01481904	0	0	0	0.01216873	[illegible]
ENSG00000141979	0.01255541	0	0	0.0162293	0	0	0.01008293	[illegible]
ENSG00000141985	12.1373199	10.5275727	10.7672926	9.6789003	11.2898368	8.69026937	15.316991	[illegible]
ENSG00000141994	1.52154619	1.24785185	1.18998767	1.2647792	1.62336384	0.97289744	2.33157661	[illegible]
ENSG00000142002	1.56803903	1.16546461	1.11061454	1.193766	1.41798991	1.07582503	2.14312732	[illegible]
ENSG00000142025	0	0	0	0	0	0	0	[illegible]
ENSG00000142039	3.7975074	4.27309722	3.38163711	2.752007	3.54599257	2.7636563	3.2861391	[illegible]
ENSG00000142046	0.10215011	0.13869276	0.08640084	0.1891397	0.06930183	0.21123742	0.15076552	[illegible]
ENSG00000142065	3.01707874	4.77229772	3.61354796	4.1041457	2.20892536	3.53622781	1.3219757	[illegible]
ENSG00000142082	1.17849234	1.07845558	0.99060239	1.2227698	1.10842122	1.09015477	0.6040662	[illegible]
ENSG00000142089	0.18855152	0.04209827	0.15981333	0	0.0701187	0.04714573	122.045309	[illegible]
ENSG00000142102	0.56291321	0.72805402	0.75221066	0.502568	0.61669446	0.74179968	2.73273172	[illegible]
ENSG00000142149	2.0625955	1.75855324	1.76997775	1.1065375	1.89315947	1.17809992	0.01894596	[illegible]
ENSG00000142156	11.3984905	14.4903795	12.5279125	17.354918	9.7708051	16.2199877	171.53706	[illegible]
ENSG00000142163	0	0	0	0	0	0	0	[illegible]
ENSG00000142166	11.0492505	10.1932088	10.4880594	8.1352753	9.62722697	7.42358713	3.86261323	[illegible]
ENSG00000142168	34.4284537	28.8116444	32.4741142	27.950598	35.6286545	26.2553892	27.5615813	[illegible]
ENSG00000142173	0.18495069	0.09821347	0.11080842	0.0516909	0.05762617	0.06249374	67.7146142	[illegible]
ENSG00000142178	0.19731235	0.28084625	0.30754265	0.3905442	0.15477895	0.37464754	1.60437291	[illegible]
ENSG00000142182	0	0	0	0	0	0	0	[illegible]
ENSG00000142185	0.00108273	0	0	0	0	0	0	[illegible]
ENSG00000142186	2.37642877	2.44359105	2.18519856	3.0565121	2.97843254	2.63594696	2.14925068	[illegible]
ENSG00000142188	3.32613592	3.81983706	3.89574352	3.3514227	3.46463845	3.10907002	2.94973576	[illegible]
ENSG00000142192	11.9697564	7.48038221	9.92383012	9.1486042	14.1331079	8.85597828	18.3338341	[illegible]
ENSG00000142197	11.3008375	7.64285464	9.73457638	8.7906148	12.2839978	8.32157874	1.28867608	[illegible]
ENSG00000142207	3.18113834	3.3117113	2.57708222	3.6309142	3.29897615	3.11170389	11.8113605	[illegible]
ENSG00000142208	4.92606903	3.9703873	4.36343917	4.1079675	4.97910223	3.98173622	1.70216451	[illegible]
ENSG00000142224	0	0	0	0	0	0	0	[illegible]
ENSG00000142227	1.18119465	0.60146047	1.30253245	1.4800923	1.23710996	1.03974491	21.3916203	[illegible]
ENSG00000142230	11.8784262	7.41809552	9.26108192	6.8778986	13.6470292	7.05655027	5.12363482	[illegible]
ENSG00000142233	0	0	0	0.033577	0	0.00974263	0.03129102	[illegible]
ENSG00000142235	0.5337002	0.94186573	0.7251031	0.966644	0.4367592	0.96129976	0.20018499	[illegible]
ENSG00000142252	1.49480099	1.59892121	1.4022196	1.3308554	1.4804769	1.43021352	3.28895252	[illegible]
ENSG00000142273	0	0	0	0	0	0	0	[illegible]
ENSG00000142279	1.29984026	1.17557415	1.20961944	0.6698576	1.1115742	0.7774568	0.44375929	[illegible]
ENSG00000142303	0.07871651	0.1349417	0.09264096	0.0983011	0.02753662	0.16713363	0.43179226	[illegible]
ENSG00000142319	0	0	0	0	0	0	0	[illegible]
ENSG00000142327	2.98284761	2.51537253	2.58916408	2.8640868	3.36063185	2.6122105	0.83525553	[illegible]
ENSG00000142330	1.08433794	1.13920202	0.91739335	1.1345869	1.06022994	1.13087019	0.55333304	[illegible]
ENSG00000142347	0.01712526	0.00917662	0.02344748	0.0376319	0.00955283	0.01798452	0.02750573	[illegible]
ENSG00000142396	0.76249987	1.0829463	0.76987774	0.734652	0.75025014	0.99035566	2.17438978	[illegible]
ENSG00000142405	0.00628791	0.00772153	0.00717438	0.0149011	0.00935342	0.0117918	0.01009933	[illegible]
ENSG00000142408	1.03907472	1.33768045	1.28646142	1.4383825	2.27530897	1.52570411	0.50599916	[illegible]
ENSG00000142409	4.62627844	3.44668497	3.69097194	3.7453716	4.5908167	3.11108142	5.35126035	[illegible]
ENSG00000142444	4.14417091	3.82592593	3.45865423	4.2728925	3.40993696	3.97469065	5.11411604	[illegible]
ENSG00000142449	0	0.00071838	0	0	0	0	0	[illegible]
ENSG00000142453	2.93964753	2.69758993	2.60951236	2.9607303	3.28565134	2.77347854	5.38231658	[illegible]
ENSG00000142459	2.87676693	2.75412029	2.50432156	2.6858348	3.00864939	2.5574706	1.89897187	[illegible]
ENSG00000142484	0	0	0	0	0	0	0	[illegible]
ENSG00000142494	0.00563531	0.01132386	0.00413342	0.0054632	0.00471524	0.01902233	0.09956264	[illegible]
ENSG00000142507	32.3863118	24.8229622	28.7403065	26.746295	34.6400639	22.1977733	18.0870737	[illegible]
ENSG00000142511	0	0	0	0	0	0	0	[illegible]
ENSG00000142512	0.00834967	0.01997408	0.01633166	0.0107929	0.00332688	0.01968465	0.00957916	[illegible]
ENSG00000142513	0	0	0	0	0	0	0	[illegible]
ENSG00000142515	0	0	0	0	0	0	0	[illegible]
ENSG00000142528	1.08056657	0.95921493	0.93968886	0.9404311	1.09079561	0.99017255	0.93310552	[illegible]
ENSG00000142530	0.0641771	0.12036315	0.07531686	0.2737561	0.05369903	0.1781211	0.3607731	[illegible]
ENSG00000142534	142.000718	161.932639	167.353963	227.16848	111.656003	180.444078	145.336275	[illegible]

ENSG00000142538	0	0	0	0	0	0	0	0	0	0	[illegible]
ENSG00000142539	0	0	0	0	0	0	0	0	0	0	[illegible]
ENSG00000142541	46.1883438	53.765585	48.7015773	92.967336	34.477356	68.2873365	86.5194279	85.7212521	86.9433318	85.5983725	[illegible]
ENSG00000142544	1.60608191	1.00337091	1.10252409	1.1378342	1.00789501	1.19317773	2.57960789	2.93456715	3.03106846	2.18199721	[illegible]
ENSG00000142546	2.00359815	1.78178543	1.79665533	2.2572837	1.63689772	1.68460833	3.50980229	3.6897241	3.60961039	2.59953389	[illegible]
ENSG00000142549	16.4240577	17.1974936	19.3402343	13.716379	13.3837928	14.741438	0	0.08479854	0	0.05600769	[illegible]
ENSG00000142552	0.5549603	0.59700678	0.55096456	1.8477222	0.95684862	0.84519388	16.3886722	21.2740589	21.7498669	22.1085499	[illegible]
ENSG00000142556	5.21580522	5.00973216	4.94857654	4.4681898	4.72562936	4.456934	1.9263985	2.07248753	2.3029445	2.53287566	[illegible]
ENSG00000142583	0.00419031	0.01824377	0.01024511	0.0108329	0.00818105	0.00628651	0.04711181	0.03693994	0.02278816	0.03036204	[illegible]
ENSG00000142599	1.27275675	1.50612523	1.42034943	1.1934114	1.25869567	1.41139195	0.88002116	0.94637504	0.83711001	0.92506785	[illegible]
ENSG00000142606	0.01455067	0.0682239	0.03557574	0.1285244	0.01352779	0.10914821	0.00973773	0.00712625	0.00119895	0.02635775	[illegible]
ENSG00000142609	0.01514081	0.00676104	0.00822636	0.0086983	0.01313801	0.02397694	0.02026534	0.01647838	0.01081237	0.02437934	[illegible]
ENSG00000142611	0	0.00036665	0	0	0	0	0.09671082	0.10294508	0.14884353	0.1457602	[illegible]
ENSG00000142615	0.03165986	0.01060314	0	0.0613861	0	0.02374885	0	0.01550554	0.00782618	0.02867506	[illegible]
ENSG00000142619	0	0	0	0	0	0	0.12986086	0.1583909	0.17488043	0.64991397	[illegible]
ENSG00000142621	0.00123431	0.00289367	0.00060357	0.0015955	0.0055082	0.00277766	0.00941683	0.01027666	0.00884838	0.01285636	[illegible]
ENSG00000142623	0	0	0	0	0	0	0.00454844	0	0.00840037	0.02308416	[illegible]
ENSG00000142627	24.5226281	12.6143265	13.6421015	13.530123	26.9617308	15.9105093	17.2552618	15.7794443	13.6746745	22.5664562	[illegible]
ENSG00000142632	0.061157	0.05188764	0.03189891	0.0421614	0.04776055	0.04281703	0.74652745	0.97443466	1.10258769	1.2444575	[illegible]
ENSG00000142634	4.71109819	2.60253051	2.85941896	3.0636562	6.14994688	2.87813445	11.00686	13.5392089	13.7058279	10.4870156	[illegible]
[illegible]	[illegible]	[illegible]	[illegible]	[illegible]	[illegible]	[illegible]	[illegible]	[illegible]	[illegible]	[illegible]	[illegible]

ENSG00000143549	9.01896398	5.72816511	7.46470402	4.5438636	9.61801338	5.02311564	11.5256025	12.7846768	13.4921625	8.01492374	11.8118252	13.9025701	10.2777736	7.40789577
ENSG00000143552	0.00239075	0	0.01169057	0	0.00266723	0.00896682	0.00383991	0.00936705	0.01181968	0.00216536	0.00602385	0.00605697	0.01214134	0.00776999
ENSG00000143553	25.1731231	22.7980542	22.9018459	23.231323	21.5315415	19.3219769	7.87384839	7.60155196	7.24163161	7.03661709	8.00321188	6.56096827	4.27140674	4.29096086
ENSG00000143554	1.29217278	0.75800681	0.80865508	0.4442914	1.35116364	0.62389743	0.35024388	0.3255548	0.24287107	0.18942933	0.23080755	0.18073217	0.17153512	0.16510286
ENSG00000143556	0	0	0	0	0	0	0	0	0	0	0.04408659	0	0	0
ENSG00000143569	9.41946782	9.64432567	8.55946201	6.9294602	9.35520663	7.79813862	9.89513074	11.2893156	10.7175763	7.12490152	8.32217386	9.9545267	5.21975561	4.35809974
ENSG00000143570	13.5557146	9.37301549	12.1838452	12.077709	12.9896739	11.5016169	11.3861411	12.3170418	11.4571579	9.10419795	11.93478	11.5276633	5.45665917	5.40427911
ENSG00000143575	10.3637433	8.03840268	9.61261897	10.810271	11.185204	8.92782338	6.89711903	7.48847376	7.79254836	6.00475275	6.49417716	7.40763657	3.81644499	3.77177224
ENSG00000143578	1.03701733	1.27627647	1.04922728	1.5911222	0.58787637	1.20636579	0.95806142	1.02608957	0.94410435	1.03661141	1.03826201	0.87576034	0.37107783	0.43156596
ENSG00000143590	2.27693878	1.6612305	2.32031936	2.4797117	1.85018198	2.74354044	0.54232651	0.73757281	0.75046594	1.00136717	0.67007849	0.63591078	0.17198321	0.20070239
ENSG00000143595	0	0.00437447	0	0.0084419	0	0.0097979	0.01048952	0.01279404	0.00968639	0.00591514	0.00822771	0	0	0
ENSG00000143603	0.36008787	1.80517559	0.74283638	0.9066688	0.51680808	0.98337237	0.02711039	0.01469621	0.03245235	0.03227425	0.07797053	0.05384997	1.14451974	0.9041318
ENSG00000143612	36.2367906	25.6697439	31.5666406	36.872116	34.5596216	31.3784944	24.4854403	25.6236213	23.3374239	21.3785645	25.4549473	23.7766355	11.1902918	11.3771635
ENSG00000143614	11.421344	16.1751152	12.64092	15.548946	11.1649976	17.0863735	5.86674285	6.47596764	5.88739414	4.57503259	4.62615551	5.1393157	3.3561389	2.69875616
ENSG00000143621	147.403785	117.057238	131.571394	122.91468	137.150041	107.965526	62.7638405	72.8321153	97.1407975	53.8856539	61.9393394	91.7138651	45.9360079	41.6570335
ENSG00000143622	14.8381319	14.1965817	15.1026491	12.607844	12.0820922	13.0763349	3.41288982	3.65008832	2.84824366	2.50887467	4.33091459	2.99458856	4.26147856	4.37424253
ENSG00000143624	3.61836865	4.54857435	3.47166884	3.365487	3.29697328	3.97419113	4.99958438	4.99591288	4.68059947	4.67520451	4.53943771	4.48321079	2.10352868	1.75600876
ENSG00000143627	0.01880382	0	0.00612993	0	0.00699278	0	0	0	0	0	0	0.00529327	0.00530525	0.0067903
ENSG00000143630	0.88086487	1.13916751	0.84655279	0.4091148	0.90189972	0.73226932	0.31235862	0.21290219	0.22057387	0.22104084	0.14892524	0.24796324	0.23238632	0.2106842
ENSG00000143631	0	0	0	0	0	0	0	0	0.00247661	0	0	0	0	0

[illegible]

ENSG00000144681	0	0	0	0	0.00405057	0.00272348	1.68237344	1.77815066	2.12167541	1.56857107	1.31732326	1.68636869	0.00921919	0.01573313	0.00631522	0.00350245	0	0	0.56338655	0.4900156	0.80074923	1.426416
ENSG00000144711	1.04127581	1.37427229	1.15753115	1.566722	1.36677387	1.85752781	2.27246769	2.72990227	2.53529114	2.03874409	1.98715709	1.90293576	1.05801698	0.89422123	0.93447398	0.97932504	0.87778675	0.75819678	3.4665392	4.5723222	3.56205623	2.69027779
ENSG00000144712	4.824178	4.38450711	4.05135372	3.9685901	3.8972396	3.77961758	0.91309749	1.04463547	0.94123066	0.97658821	0.82539974	0.61283899	0.24867432	0.35011227	0.27595717	0.1728868	0.2077307	0.22681144	1.5099544	1.74522743	1.97359358	1.94468679
ENSG00000144713	67.9881993	74.0746267	74.7647037	104.37462	55.5617422	87.3733464	75.7256147	84.9353423	84.3321813	50.657778	83.2299547	72.4518838	32.6920316	34.413506	32.5518281	36.4653575	32.4445264	29.3332941	40.3647599	34.1798128	54.2517818	48.2110416
ENSG00000144724	10.0341349	8.50469271	8.12956585	7.7390817	12.4017591	7.71646038	8.54533757	9.47654076	6.90552207	4.348548	11.9991822	7.38805351	3.92962767	5.10214765	4.11616622	3.94094421	4.378038	4.69377272	12.1103215	10.7887543	8.9067092	8.03548469
ENSG00000144730	3.45698529	3.60439243	3.08079235	2.4149599	1.95956878	2.42643028	0.48624881	0.64650651	0.76050059	0.87447635	0.73531366	0.7612377	0.18352594	0.19944254	0.22889939	0.15260171	0.1887383	0.11185703	1.00251946	1.1152056	1.74029723	0.64990751
ENSG00000144736	1.1413766	0.90825306	1.02168781	0.9693498	1.06815609	0.8349475	2.62105242	3.00054903	2.90537725	1.66812995	2.80456102	2.74430315	1.26946719	0.94525678	1.09109606	1.74713577	1.43678334	0.88750948	0.82788701	0.88363274	1.98711198	1.52467499
ENSG00000144741	0.83659711	0.75349476	0.781861	1.084223	0.65187819	0.87496848	1.03321069	1.11471355	1.07667253	0.55197198	1.01818948	0.84669995	0.62675534	0.71464276	0.68199279	0.54364966	0.53890733	0.40312879	0.43991164	0.38790316	0.69872429	0.65295525
ENSG00000144744	5.82238618	5.98983935	6.14373036	4.7593369	5.87539866	4.74018358	5.3208799	5.79630571	5.47243498	4.04341206	5.69733456	6.03254032	4.71787463	4.52951455	5.04755704	5.9123377	5.61929943	4.25369212	3.68668971	2.83640114	4.16644196	4.38191087
ENSG00000144746	29.1708398	29.4470755	32.9941132	30.505696	24.7114795	29.0182738	21.6031247	20.8489351	14.6293198	13.7719365	26.4228792	17.3737569	19.6468902	24.4437748	21.1497787	16.3370331	18.7607606	20.8345806	22.2418479	21.2824721	18.7581045	18.5484956
ENSG00000144747	5.18860996	5.58087549	5.1934923	6.0864857	4.27758568	5.50813371	6.24880305	6.92358908	6.15055752	4.40305085	7.77581876	6.50664736	6.69986792	7.82492334	7.56565877	6.8468166	7.20659856	7.60081947	3.79943309	3.87421787	3.28574428	4.608491
ENSG00000144749	48.0263977	63.0490124	51.0313408	44.559972	43.659968	40.5555758	1.49407334	2.12364477	3.01686588	4.05855213	2.56118771	2.74365483	7.87210126	9.02826102	10.2573637	6.72982346	7.95313493	7.55312487	5.15533919	6.31577797	2.35395142	4.27337819
ENSG00000144771	0	0	0.01149949	0	0	0	0.00944286	0	0	0	0	0	0.04976208	0.03821497	0.04090492	0.01134304	0	0.04255042	0	0	0.00989811	0.01004259
ENSG00000144785	0.01599491	0.01071364	0.01564273	0.0206753	0	0	0	0	0	0	0.02015074	0.01350769	0	0.01732791	0	0	0.02406215	0	0	0	0	0
ENSG00000144791	0.13487892	0.10163704	0.07625998	0.1743468	0.09639912	0.12963179	3.90113565	4.23593648	3.7175759	2.92045678	3.56308285	3.53639884	3.69067125	3.94066369	3.79404264	3.36468133	3.70305027	2.74296558	5.21151465	7.4795247	2.63093381	4.02290178
ENSG00000144792	3.35261411	4.35612756	3.73640049	2.169597	2.60529266	2.52775953	1.60302945	1.84588664	2.13501733	1.90901469	1.87119018	2.33825306	0.09810182	0.15811604	0.10080082	0.19049001	0.20019208	0.12945158	0.93066963	0.84998868	1.78510655	0.9899063
ENSG00000144802	0.5724845	0.41647223	0.56173346	0.3136437	0.44200599	0.44792047	1.58171541	1.55971068	0.92313221	1.57098893	1.71948574	0.96852631	3.49458351	5.659792	4.27326215	5.10934253	6.57040162	3.56253421	2.04655101	1.34486691	2.26275576	2.0059767
ENSG00000144810	0.01168845	0.05010631	0.05258305	0.0362609	0.01434415	0.04208544	4.68771908	5.74736418	3.95723342	4.21977762	3.59299116	2.9119124	1.92126386	2.03360705	1.19138572	0.82311728	0.98468588	2.64781366	12.2536193	7.37285551	20.758835	22.2617695
ENSG00000144815	1.23483216	1.15130408	1.1311798	1.3298699	1.44824359	1.37219521	2.39693326	2.47862056	2.457914	2.81908145	3.57882067	3.05822475	1.7258505	2.18744364	1.93199337	1.23311592	1.36102165	1.38771214	1.30159098	1.08431556	1.23587417	1.85172097
ENSG00000144820	0.32075161	0.18119417	0.20030763	0.0899152	0.53892191	0.17682927	0.00310346	0.00757057	0	0.00350014	0	0	0	0.00418654	0	0	0	0	0	0	0.00325308	0
ENSG00000144821	0.06261676	0.04393892	0.02624488	0.0423969	0.03825553	0.03131359	0.29932108	0.21028667	0.24618333	0.47531245	0.19533673	0.32483308	0.62589823	0.62020745	0.99838894	0.48323946	0.84778503	0.48915331	0.08859961	0.05030378	0.06275032	0.14261234
ENSG00000144824	2.65655655	1.92750689	1.70970228	1.3220844	3.09035345	1.69061171	1.33487627	1.3875009	0.91639246	0.38354777	1.20301963	0.74472607	1.68434984	1.28523772	1.49340894	1.1382237	1.18463038	0.91321834	0.99611101	1.8059985	0.28572411	0.63003744
ENSG00000144827	12.0604584	9.76418941	10.9292677	12.102914	14.9434187	11.6208752	3.66194251	3.82545959	4.33661519	3.7538607	4.02179489	4.06339129	4.61938354	4.08554622	4.63702319	4.69424535	4.19662599	3.07149251	2.36831852	2.3950395	2.15407322	2.84116819
ENSG00000144834	2.70459355	3.83218767	3.51400866	3.7537212	2.37181882	3.62514891	0.01044226	0.03396373	0.03428533	0.00392566	0.02730214	0.036603	0.1797605	0.06104151	0.03769512	0.1547038	0.13040675	0.0418256	0.00476995	0.00384851	0.02189139	0.05922911
ENSG00000144837	0.00825768	0	0.02018967	0.010674	0.03224418	0.00619429	0	0	0.00204126	0	0	0	0	0	0	0	0	0	0.00908772	0.0036661	0.03823187	0.01410542
ENSG00000144840	3.27927396	2.21795648	2.52860198	2.6626428	3.07057798	2.35378453	1.5835299	1.74586337	1.78218201	1.51768172	1.50932044	1.91533174	2.34199161	2.34717958	2.94015472	2.3783711	2.53685445	1.37110344	0.86037983	0.75814371	1.13877634	1.65675397
ENSG00000144843	0.22470242	0.13344118	0.14499294	0.1497186	0.21967469	0.11121146	0.20091658	0.19967636	0.17866172	0.07133623	0.15467538	0.17411063	0.02156798	0.01254788	0.0261907	0.02458162	0.03484887	0.02235429	0.2090483	0.16043767	0.34320479	0.18004257
ENSG00000144847	4.3141667	5.78583032	4.9912839	3.6638896	1.93755468	3.49803593	0.00385813	0.00156858	0.00316687	0.00290085	0.00201748	0.00270476	0.00542176	0.01040914	0.00557092	0.00617933	0.00240908	0.00193168	0.00881183	0.00568768	0.00674022	0.00820632
ENSG00000144848	8.7757308	6.93326391	8.21382008	6.0668898	9.36999562	6.27586727	4.49798786	5.07735627	5.51150444	3.96565305	4.98344905	5.51454819	6.34525271	5.92479497	5.62522226	6.40061147	5.18920367	3.89451085	2.83008242	1.92951522	4.49949842	3.72113341
ENSG00000144852	0.00850838	0.00284952	0.00832104	0.0082486	0.00593269	0.00478676	0.00170822	0.00208351	0.00105162	0	0.00535952	0	0	0	0	0	0	0	0	0	0.0037774	0.00358114

ENSG00000146001	0.09012791	0.18915652	0.2232968	0.0932007	0.11730895	0.17577868	0.07720475	0.0941664	0.05941128	0.08163089	0.15139353	0.11163233	0.06102813	0.11065764	[illegible]
ENSG00000146005	0.1285476	0.10523727	0.11174866	0.3507881	0.09162508	0.33749363	0.02294074	0.03497594	0.03530712	0.03880949	0.06297944	0.00603102	0.03626799	0.04642022	[illegible]
ENSG00000146006	8.02488808	6.36348225	11.5209173	7.2490916	8.09448545	8.01787744	0.5254321	0.5395062	0.29541221	0.33560061	0.35325889	0.4538681	0.03955609	0.05062873	[illegible]
ENSG00000146007	22.0924272	21.1995561	22.3803664	19.417635	18.8493627	18.3873951	13.1295227	14.648586	12.6149596	11.3734596	15.384464	14.4660324	20.7381386	19.028475	[illegible]
ENSG00000146013	0.05820644	0.06683588	0.05692486	0.0322451	0.02783039	0.0436621	0.0200332	0.00814481	0.01644387	0.00753127	0.01047568	0.02106656	0.01407615	0	[illegible]
ENSG00000146021	0.14094638	0.16259177	0.14124656	0.2035576	0.13346479	0.19579146	0.08943446	0.08692547	0.0752745	0.0709213	0.13043531	0.09551719	0.08763279	0.10085262	[illegible]
ENSG00000146038	0.26212045	0.18546384	0.19858028	0.0906705	0.58898424	0.16892968	0.01482411	0.01446474	0.01825213	0.03678159	0.01395318	0.00935326	0.07187058	0.19197659	[illegible]
ENSG00000146039	0	0	0	0	0.00236216	0	0	0	0	0	0.00533488	0	0.00358423	0	[illegible]
ENSG00000146047	0	0	0	0	0	0	0	0.07224093	0	0	0	0.06228373	0	0	[illegible]
ENSG00000146049	0	0	0	0.0301452	0	0	0	0	0	0	0	0	0	0	[illegible]
ENSG00000146054	0.02754237	0.04957985	0.0471379	0.0467273	0.02592628	0.04390296	0.02626582	0.05395595	0.03489282	0.06236438	0.03036118	0.04215787	0.36862318	0.30024218	[illegible]
ENSG00000146063	1.26677557	1.46202938	1.17682247	1.4486372	1.20555298	1.46847086	1.69328234	1.64073974	1.46625972	1.54534025	1.55367626	1.54199553	1.47758439	1.42423227	[illegible]
ENSG00000146066	118.497367	112.464382	118.908187	162.33953	107.601472	129.314409	48.4723034	45.7471103	43.7710831	43.3061159	47.5939109	43.7606616	26.176955	32.6159022	[illegible]
ENSG00000146067	0.834103	1.30537539	0.99232098	1.0629542	0.68916239	1.36692984	0.81721348	0.66994863	0.64378754	0.72080344	0.62183911	0.55937595	1.18191547	0.9036165	[illegible]
ENSG00000146070	0	0.0098065	0.0096274	0	0	0.00738434	0.00395279	0.00964242	0.00243343	0.00445803	0	0	0.00833218	0.00533228	[illegible]
ENSG00000146072	41.9121094	37.3030869	48.8978121	34.070227	38.3323239	27.2429236	12.8370508	15.2270218	13.593853	4.83948788	9.01301493	9.81210456	8.98442665	5.57486387	[illegible]
ENSG00000146083	2.17569499	2.47911056	1.81256239	2.7297296	1.94369838	2.89724096	2.7491787	2.85508939	2.32987696	2.84638799	1.94284668	2.00163342	1.37115189	1.27634058	[illegible]
ENSG00000146085	11.0212127	11.1078244	11.3754879	11.268059	10.7707562	9.91171466	8.19498361	8.38703913	8.2029404	7.38150534	10.6789258	9.77204999	11.0020575	12.4705729	[illegible]
ENSG00000146090	0.05318439	0.01335891	0.01625418	0.0300768	0.05933477	0.03490807	0.00266944	0.00651182	0	0	0	0.00561427	0	0	[illegible]
ENSG00000146094	0.00858887	0.0071912	0.00629982	0.0111021	0.01676869	0.01449613	0.18105954	0.16194771	0.15498863	0.15558271	0.05951248	0.15775917	0.10359331	0.04419714	[illegible]
[illegible]	[illegible]														

ENSG00000147394	0.02042641	0.02104913	0.01229333	0.0111707	0.01051781	0.01709033	0.0820197	0.06079304	0.07651664	0.09890741
ENSG00000147400	22.7826311	20.6661153	22.5162287	14.33598	23.9414317	15.0751241	11.4069299	12.0282648	12.5583476	10.7712162
ENSG00000147402	0.01044047	0.01398639	0.0051053	0	0.0145598	0	0.23476547	0.47553348	0.57810813	0.62410742
ENSG00000147403	14.5395666	12.1953927	14.1697109	23.850243	11.4580971	18.2571524	18.8122912	22.9436353	20.5064572	17.4085013
ENSG00000147408	0.73734031	0.63595794	0.71012985	0.3670227	0.53275466	0.40661491	0.87063425	0.97836428	0.8195095	1.02562599
ENSG00000147416	10.0368983	7.44473448	8.08383687	6.5825991	9.99671098	6.86856316	6.42536397	5.88855846	5.20223957	4.55355534
ENSG00000147419	2.23811928	2.19156236	2.23908932	3.0364866	2.33070771	2.55144912	3.85883197	4.07248229	3.49326965	4.86768297
ENSG00000147421	0.54834268	0.92198475	0.70989746	0.8613045	0.78224338	0.89176713	0.69573302	0.66037625	0.56441276	1.12356071
ENSG00000147432	0	0.00642118	0	0	0	0	0	0	0	0
ENSG00000147434	0.00540767	0.00724429	0.00528861	0.0139801	0.00603303	0.01216929	0.01737107	0	0.00534702	0.00979571
ENSG00000147437	0.34686715	0.30978282	0.28269154	0.2802289	0.33054522	0.55291157	0.5339072	0.31144588	0.42157499	0.99485725
ENSG00000147439	0.84207709	1.0737082	0.8260168	0.8524264	0.86871481	0.77626052	1.47064205	1.55523519	1.4194859	1.48172881
ENSG00000147443	0	0	0	0	0.00242199	0	0.36611932	0.35724371	0.43575689	0.42078123
ENSG00000147454	0.80509175	0.79400089	0.67638426	0.8008243	0.72882079	0.87309483	2.9556522	2.92455638	2.73465853	5.45017335
ENSG00000147457	2.75933244	2.18827774	2.51239973	2.8020758	2.30653787	2.53970459	3.82859682	4.03012172	3.98630939	4.14554063
ENSG00000147459	0.00787903	0.00904714	0.01981427	0.0174593	0.02385907	0.0151978	1.46435308	1.65486832	1.38562278	2.13984571
ENSG00000147465	0.13332357	0.20506441	0.20765506	0.2680787	0.12670569	0.17038626	0.01982756	0.01934689	0.00976504	0.00894475
ENSG00000147471	6.79254426	5.43356372	5.69788291	5.2488761	6.52064905	4.78856517	3.68263155	3.50415898	3.36276927	3.30331511
ENSG00000147475	4.16072209	3.49639206	3.35267493	3.4556698	3.27443503	3.05207425	2.92751993	3.21501642	2.81351493	2.88666361
ENSG00000147481	0.51829666	0.47325565	0.78901883	0.1580092	0.25093166	0.16138337	0.00196335	0.0023947	0.00483474	0.00221431

ENSG00000148835	3.46746847	2.58025142	2.55943068	2.7219809	3.29807006	2.53268599	2.55698751	3.17329096	3.79645641	1.97144946	[illegible]
ENSG00000148840	2.32373814	1.93863343	1.71459621	1.8153592	2.54092122	1.82279786	7.09168627	7.49581074	7.64558418	6.4248688	[illegible]
ENSG00000148841	2.81190895	1.95527426	2.69280415	3.4772371	3.63999765	3.44723348	2.59278996	3.25311393	3.68627525	6.75985974	[illegible]
ENSG00000148842	0.71962027	0.83916732	0.79563397	0.9245897	0.55860184	0.98855866	0.41080273	0.46708504	0.46007729	0.56147003	[illegible]
ENSG00000148843	6.66797264	6.44030819	5.49913226	5.749205	6.78553297	4.91501277	39.4152925	43.0451636	43.2149816	32.4565078	[illegible]
ENSG00000148848	0.51222506	0.69371697	0.66353494	0.6156468	0.44238028	0.58140483	1.75367152	2.32819639	3.36875379	3.81321069	[illegible]
ENSG00000148908	0.26902955	0.16578423	0.33677576	0.4033924	0.24011265	0.33903367	3.31854116	4.46924127	5.08614518	5.71153485	[illegible]
ENSG00000148925	4.10727993	3.06180682	3.92385295	3.1567043	3.48520572	3.12446482	1.78053144	1.85367619	2.05468209	2.20766768	[illegible]
ENSG00000148926	1.61619277	0.46736663	0.75856464	0.541157	1.88998954	1.11983803	2.81477435	2.81329361	2.48855076	0.54085006	[illegible]
ENSG00000148935	0.26489471	0.09936118	0.22020293	0.0992981	0.40339548	0.1013391	0.12125626	0.10897592	0.06155198	0.03598817	[illegible]
ENSG00000148942	0.03129101	0.01921261	0.01785119	0.016853	0.01454566	0.02347216	0.00628226	0.00255415	0.00128917	0.00708524	[illegible]
ENSG00000148943	8.28539089	9.5700827	8.65767242	7.4848199	7.85980813	6.99869459	13.0365828	13.889361	13.147678	8.59023913	[illegible]
ENSG00000148948	1.92624998	2.51222444	2.66703247	1.3322359	0.88022037	1.28497288	0.00761094	0.01392455	0.0035141	0.00429188	[illegible]
ENSG00000148950	1.27291421	1.17266245	1.34345602	1.4668582	1.27019288	1.4000654	0.26080747	0.44242341	0.47614075	0.30090542	[illegible]
ENSG00000148965	0	0	0	0	0	0	0	0	0	0	[illegible]
ENSG00000148985	0.32166956	0.38003918	0.35476323	0.5189929	0.34935609	0.463979	0.40398252	0.40998088	0.36902777	0.45351288	[illegible]
ENSG00000149016	0.61774658	0.50727401	0.49667695	0.7294078	0.5384253	0.71735883	1.54553211	1.73962712	1.43013442	1.65700176	[illegible]
ENSG00000149021	0	0	0	0	0	0	0	0	0	0	[illegible]
ENSG00000149043	0	0	0	0.0019629	0	0	0.01341462	0.00297487	0.00600607	0.0206308	[illegible]
[illegible]	[illegible]	[illegible]	[illegible]	[illegible]	[illegible]	[illegible]	[illegible]	[illegible]	[illegible]	[illegible]	[illegible]

ENSG00000150672	0.25574466	0.25381715	0.21878923	0.3369494	0.33864526	0.36667766	0.01662052	0.01834133	0.02022033	0.02677847	[illegible]
ENSG00000150676	0	0	0	0	0	0	0	0	0	0	[illegible]
ENSG00000150681	0	0	0	0	0	0	0	0	0	0	[illegible]
ENSG00000150687	0.69675382	0.62463739	0.77504195	0.5889653	0.66458597	0.45881697	7.22568239	7.51939117	6.62818117	3.81048091	[illegible]
ENSG00000150712	2.0022174	1.52003112	1.66883852	1.2355537	2.08845382	1.46051524	2.47483001	2.75042006	2.73280822	2.53123747	[illegible]
ENSG00000150722	0.31372388	0.36894322	0.31618475	0.300274	0.19236845	0.23712851	0.01730911	0.01407458	0.01183988	0.00650718	[illegible]
ENSG00000150732	0	0	0	0	0	0	0	0	0	0	[illegible]
ENSG00000150750	0	0	0	0	0	0	0	0	0	0	[illegible]
ENSG00000150753	12.8298074	8.41971761	10.011935	8.1406509	15.077054	8.65570605	23.9190411	27.4419667	32.3717333	23.3013341	[illegible]
ENSG00000150756	1.1845195	1.21274067	1.24212829	0.6869708	1.2612038	0.87840213	1.37444092	1.60140457	1.78356882	1.59907111	[illegible]
ENSG00000150760	18.4797058	18.0287228	18.204385	11.540893	17.4709155	12.5847567	9.10620663	10.1948268	11.4447623	12.8160426	[illegible]
ENSG00000150764	0.37653688	0.51523942	0.47684441	0.4593155	0.26297269	0.43313999	0.92499704	1.2785131	1.49025085	2.13034455	[illegible]
ENSG00000150768	5.11147429	4.1577321	4.66389301	4.2493564	6.66566361	4.03165735	5.60802081	6.03785389	7.01926172	4.23093719	[illegible]
ENSG00000150773	0.19681082	0.29001941	0.21942429	0.1170244	0.19541777	0.15058534	0.22127534	0.14265555	0.1362222	0.11408385	[illegible]
ENSG00000150776	2.57760396	2.86415859	2.64663444	2.9970745	2.01081714	2.61283569	3.8952388	4.45763231	4.54861962	4.30854736	[illegible]
ENSG00000150779	7.73696878	6.3966688	7.54870735	7.3498	8.59083173	6.70343678	11.6178893	10.9416278	11.1176599	10.623277	[illegible]
ENSG00000150782	0.010124	0.00678121	0	0.0065432	0	0.00379713	0.13821554	0.10412361	0.06757042	0.07794103	[illegible]
ENSG00000150783	0	0.03348706	0.04889365	0	0.01859197	0.02500137	0.05353235	0.06529325	0.03295575	0.10565587	[illegible]
ENSG00000150787	1.66477877	1.12141303	1.18073563	1.1887382	1.35745866	1.15681295	3.37833483	3.33061981	3.96370783	3.49833157	[illegible]
ENSG00000150867	3.74943866	3.10346453	3.27584088	2.6386472	3.06977522	2.70993789	3.99411707	4.15014675	3.5357102	2.2840872	[illegible]

ENSG00000152147	4.0482608	3.5456367	3.79530043	4.5399483	3.91214733	3.96655993	5.72185915	6.43746545	6.77171876	7.83894139	[illegible]	[illegible]
ENSG00000152154	3.85701437	1.99089824	2.39076176	3.2301339	7.80522955	3.45791949	0.10594972	0.0608125	0.00383677	0.16869467	[illegible]	[illegible]
ENSG00000152192	0	0	0	0	0.00794627	0	0	0	0	0	[illegible]	[illegible]
ENSG00000152193	16.3477569	14.7304849	15.496567	11.049707	15.7827123	13.0506964	4.70131317	5.46580738	5.97359635	4.06972895	[illegible]	[illegible]
ENSG00000152207	0	0.01694505	0.01237053	0	0	0	0.04063248	0.03716949	0.01250715	0.02291303	[illegible]	[illegible]
ENSG00000152208	0.15042129	0.5108433	0.30454209	0.4400418	0.21492275	0.54437817	0.00635787	0.00258489	0.00260937	0.01434105	[illegible]	[illegible]
ENSG00000152213	0.00810403	0.03799747	0.04755359	0.0419016	0.01356181	0.05471133	0.03254072	0.05556576	0.01201969	0.08808008	[illegible]	[illegible]
ENSG00000152214	0.56367298	0.64650187	1.48765237	0.5589361	0.848527	0.59658887	0	0	0	0	[illegible]	[illegible]
ENSG00000152217	5.72812023	7.82967937	6.40481678	4.9205554	4.11542354	5.192297	0.45335615	0.57832214	0.55307196	1.13518752	[illegible]	[illegible]
ENSG00000152219	3.65950398	3.79497899	3.7774959	3.5743095	3.2089902	3.88010686	4.06082281	4.6451883	4.28048341	4.12013276	[illegible]	[illegible]
ENSG00000152223	0.52425052	0.85808042	0.67853883	0.7932786	0.61741754	0.8372679	0.59091651	0.61407581	0.61258398	1.16522481	[illegible]	[illegible]
ENSG00000152229	0.18903073	0.21183794	0.20264449	0.3007314	0.22305751	0.20996812	0.71815831	0.77979628	0.94713214	0.91201941	[illegible]	[illegible]
[illegible]												

ENSG0000153767	6.55678439	5.45121787	5.93742349	5.062709	5.97776017	5.1939873	3.30036885	3.46432669	3.88501567	3.08492848	[illegible]
ENSG0000153774	7.04981051	6.46463173	6.79708884	6.3942336	6.87730089	5.93279787	2.19217311	2.55753539	2.56297587	2.38637789	[illegible]
ENSG0000153779	0	0	0	0	0	0	0	0	0	0	[illegible]
ENSG0000153786	3.36559966	3.98292596	3.67082451	4.4530723	4.38685399	4.14431724	3.14921199	3.2427847	3.07569154	4.19348704	[illegible]
ENSG0000153789	0.01698985	0	0.03323153	0.0439227	0.0094773	0.01274451	0	0.03328338	0	0	[illegible]
ENSG0000153790	0.95938928	1.40056929	1.0866237	1.2560217	0.79131644	1.05488882	0.51364092	0.47789659	0.46215189	0.48645821	[illegible]
ENSG0000153802	0	0	0	0	0	0	0	0.0063667	0	0	[illegible]
ENSG0000153814	3.10066062	2.953518	3.00223403	2.8830783	2.78267262	2.69339781	1.62610711	2.09074792	1.71079478	1.75016906	[illegible]
ENSG0000153815	1.37295363	1.39221174	1.23761176	1.2324326	1.17006811	1.45640538	1.33507391	1.34312101	1.44839922	1.31259942	[illegible]
ENSG0000153820	0.0158155	0.02270031	0.06628834	0.0262844	0.03024763	0.06101282	0	0	0	0.00204635	[illegible]
ENSG0000153822	2.20502285	2.44805132	2.35974682	1.3416865	1.75427469	1.41097184	0.0062206	0	0	0	[illegible]
ENSG0000153823	5.99913885	5.86561279	4.96468122	7.2521776	4.47198488	6.51564246	1.08569181	1.30435183	1.5890661	2.97238478	[illegible]
ENSG0000153827	7.94714581	8.84906581	7.71116263	7.3477644	7.92015316	7.03684983	8.8802744	8.8867206	9.38696334	8.37204319	[illegible]
ENSG0000153832	0.98928704	1.17721784	0.92852124	1.5504489	0.834843	1.10633754	0.38705023	0.42948996	0.18990501	0.39713694	[illegible]
ENSG0000153879	10.2070297	7.04038817	9.58997461	11.381532	11.0777357	9.58056201	5.15770491	5.52085466	6.17211201	6.41516577	[illegible]
ENSG0000153885	1.35081796	1.28203988	1.11426194	1.1296588	1.339716	1.04727404	2.44168424	2.66318301	3.013782	3.52381795	[illegible]
ENSG0000153896	0.60776701	0.71968046	0.65453134	0.8838111	0.5912764	0.61600929	0.38930381	0.48546327	0.32551433	0.72085143	[illegible]
ENSG0000153898	0	0	0.00638641	0.0126615	0.00182134	0.00244923	0.29892106	0.33261127	0.30347602	0.12124805	[illegible]
ENSG0000153902	0	0	0	0	0.00119936	0	0.01899345	0.01895421	0.00531491	0.07984253	[illegible]
ENSG0000153904	12.1329699	13.0571479	11.5872376	7.0074715	9.46346996	8.12384815	2.97817778	3.5050193	4.44666645	4.55331217	[illegible]
[illegible]	[illegible]	[illegible]	[illegible]	[illegible]	[illegible]	[illegible]	[illegible]	[illegible]	[illegible]	[illegible]	[illegible]

ENSG00000155254	1.68799315	1.41533925	1.12232482	1.8522844	1.96109907	1.84009741	18.0078226	18.0684803	16.4384253	15.0025642	15.19963[illegible]
ENSG00000155256	0.78742906	0.54212873	0.62584834	0.7846537	0.74929978	0.71502173	0.89079204	0.89546406	0.81234349	0.74520874	1.02119[illegible]
ENSG00000155265	1.6958248	0.29156543	1.26825415	0.7267765	4.19867784	0.99317504	0.03641375	0.0177655	0.031384	0.07392258	0.03427[illegible]
ENSG00000155269	0	0.0016108	0	0	0	0	0	0	0	0.00217812	[illegible]
ENSG00000155275	0.51419196	0.67624652	0.49368565	0.7101773	0.49638136	0.59177145	0.84661024	0.85552376	0.76495778	1.00373347	0.79272[illegible]
ENSG00000155282	0.04207538	0.11273102	0.02057448	0	0.04694108	0.03156179	0.01689484	0.02060658	0.01040085	0.01905431	[illegible]
ENSG00000155287	2.91134868	3.08236475	2.68025278	2.6044836	2.41934967	2.75386004	3.46933607	3.67123627	2.92934283	2.54408129	2.96864[illegible]
ENSG00000155304	26.9845611	23.0539997	26.7267018	24.107472	26.7347397	24.2008947	17.502183	19.0591884	20.2704488	14.9638918	20.0852[illegible]
ENSG00000155307	0.00966409	0.00323658	0	0.0187379	0.00539084	0.01087391	0.03104395	0.03786421	0.01194461	0.00437649	0.04870[illegible]
ENSG00000155313	2.51305402	2.72758672	2.79748605	2.8159072	2.08726485	2.88052484	4.08322184	4.52703238	3.93073179	3.6779166	4.45213[illegible]
ENSG00000155324	0.47878425	0.59075815	0.53993501	0.4382532	0.27027074	0.41155952	0.48752339	0.36126627	0.26445473	0.2105983	0.29870[illegible]
ENSG00000155329	1.76332923	1.7322886	1.72131079	1.6124	1.50457825	1.55786682	1.14335777	1.18344844	1.28828938	0.7541801	1.08194[illegible]
ENSG00000155330	2.90091215	2.59227202	2.84362244	2.4969451	2.69948727	2.67840508	0.83510638	0.89331489	0.93504931	0.82907049	0.89034[illegible]
ENSG00000155363	0.93655857	1.0978131	0.93842699	1.2903279	0.9480747	1.51719522	3.45424218	3.55786702	2.33115229	3.07021735	3.00505[illegible]
ENSG00000155366	8.22855228	5.00067856	7.94340978	5.7882846	6.46270692	5.22670948	5.59864896	5.68871936	4.71541088	5.36323592	5.66492[illegible]
ENSG00000155367	0.18067238	0.08397113	0.15866431	0.190645	0.2324177	0.1742488	0.03849418	0.04695123	0.04374997	0.15362035	0.09290[illegible]
ENSG00000155368	79.0380713	71.2667856	89.2680994	35.424638	85.3796595	45.4252176	13.8179586	12.712163	12.8143944	10.3631274	18.82285[illegible]
ENSG00000155380	10.4636923	9.82537071	9.13715153	8.9739706	10.9982377	9.23074833	8.94287931	10.2703827	10.7745088	6.9978676	8.61545[illegible]
ENSG00000155393	2.06944821	1.67107989	1.58162723	1.6099558	2.18049985	1.5120986	1.88351133	2.03454857	2.24896352	1.87434618	2.17261[illegible]
[illegible]	[illegible]	[illegible]	[illegible]	[illegible]	[illegible]	[illegible]	[illegible]	[illegible]	[illegible]	[illegible]	[illegible]

ENSG00000156873	0.7584559	0.78501784	0.74901059	1.08826	0.86271662	0.98624957	0.82801429	0.7556285	0.73802963	0.55930217	0.67984407	0.76893173	0.80206338	0.64688543	0.69188206	0.67978637	0.70021821	0.59053516	0.5938774	0.54666448	0.77895475	0.7174688	0.62178833	0.73790414	0.86034429	0.71270091	0.61589271	0.61244372	0.56462182	0.53552441	1.72714274	1.52465556	1.52415396	1.45053726	1.3218465	1.15635195	0.74991159	0.84089131	0.89471686	0.70298174	0.73788396	0.49810013	0.84291245	0.87393678	0.78686032	0.96278346	0.83226632	1.35230356
ENSG00000156875	17.6746939	15.5521375	17.2661003	15.898875	17.496276	14.8232796	24.7100242	24.6987824	21.9757053	19.2478391	26.0845268	20.9001278	20.762412	24.4754612	24.5306524	21.8137354	21.8213424	20.7327476	15.1871044	15.2262335	20.1556894	22.3506169	33.4637919	30.2415313	28.794312	26.2787821	31.6377633	31.1266245	30.3356975	28.8030537	19.0953318	17.4673959	17.032419	22.9075716	16.5824878	17.772684	19.9598549	24.1876239	20.605131	20.3475045	19.6178939	16.8300522	26.9138377	26.0573998	26.4945675	18.5312843	25.7185063	25.5528359
ENSG00000156876	1.6714336	1.37791104	1.33285382	1.0137839	1.55811908	1.06089408	1.60041821	2.30150082	2.83498994	1.51676658	1.60762809	2.99406041	2.02685564	1.29188592	1.76952721	2.36277619	2.07925088	1.10500806	1.28407145	0.79342581	3.01422477	2.24836842	2.25971049	3.49340908	2.74009884	2.58706921	1.47408907	2.08434344	1.91579331	1.48414207	1.60672819	1.43147118	2.03543471	1.92741944	1.82793836	2.22448958	2.01073151	2.63975558	2.21130968	2.35437502	3.29654926	2.61312394	1.42055491	0.75740606	0.78867888	2.02199957	0.85295634	0.73324342
ENSG00000156885	0	0	0.02731378	0	0	0	0	0	0	0	0	0	0	0	0	0	0	0	0	0	0	0	0	0	0	0	0.02403044	0	0	0	0	0	0	0	0	0	0	0	0	0	0	0	0	0	0	0	0	0
ENSG00000156886	0.00560517	0	0	0	0	0.00420458	0	0	0.00277115	0	0	0.00473356	0	0	0.0146244	0	0	0	0	0.00497697	0	0	0	0	0.01070158	0	0	0	0	0.01352658	0	0.00685779	0	0	0	0	0.00518693	0.00564819	0	0	0	0.01003708	0	0.00478545	0	0	0	0.00524036
ENSG00000156920	0	0	0	0	0	0.00051757	0.00055411	0	0	0	0	0	0	0	0.00060007	0.00066561	0.00103798	0	0	0.00490121	0.00058082	0.0005893	0	0	0	0	0	0	0	0	0.00314499	0.00084418	0.00226387	0	0	0	0	0	0.00069499	0	0	0	0	0.00117815	0	0	0	0
ENSG00000156925	0.34990067	0.3534136	0.33133319	0.3589583	0.27573336	0.16664711	0	0.00544017	0	0	0.00699703	0	0	0.00601684	0.00966055	0.02678897	0.0250656	0.02679779	0.00611225	0	0.00467529	0	0	0.00516361	0	0	0	0	0	0	0	0	0	0	0	0	0.00513956	0	0	0	0	0	0.09538507	0.06638442	0.10246299	0.03814205	0.12364285	0.08827256
ENSG00000156928	5.0040424	3.65386154	4.48302072	4.5118453	5.5310309	4.44414412	4.68839056	4.84703829	5.14643813	5.41075263	5.21458871	5.46237995	2.65632026	2.21527898	2.34793003	2.93212779	2.88104679	1.78101947	2.49516234	2.53427465	3.66111425	4.13138354	4.0802907	4.58914618	6.24540934	6.43146478	3.4763576	5.35696548	4.05477662	4.19426829	5.10617379	4.63331555	4.42895723	4.91985104	5.05333587	4.35786873	7.78636347	8.06168927	6.96942685	7.01585155	6.68214611	5.26576274	2.67370014	2.40510874	2.7809205	3.31774128	2.33252644	2.16590694
ENSG00000156931	1.90093347	2.34667273	2.03350037	2.1006908	1.50782804	2.08898209	1.24293219	1.20240414	1.08475447	1.37658687	1.22865622	0.9705492	1.47568599	1.64465715	1.74554409	1.26560636	1.32318109	0.93503525	1.50605551	1.84238166	1.4954637	1.5432979	1.86570889	1.68805688	2.2855614	2.01977025	2.48446832	2.49146897	2.57018592	2.41301784	2.81153631	3.7818659	3.01149412	3.44501874	2.9837865	2.18779326	1.33964708	1.39565753	1.21894942	1.71468779	1.43671023	1.66069949	1.55789133	1.23446962	1.31691228	1.48052784	1.25038502	1.25096274
ENSG00000156958	0.68829282	0.74158472	0.74620103	0.7662175	0.65038266	0.6320609	0.67667693	0.67178905	0.55462991	0.75651325	0.8131241	0.6381479	0.85002468	0.99509067	1.01400856	1.01605496	1.13677559	1.23650872	0.60786935	0.71554765	0.71548585	0.6025002	1.01732173	0.83917697	1.23577924	1.07745226	1.21710946	1.01396813	0.91465021	0.95857823	1.07181575	0.87207803	1.10706369	0.79992725	0.74419102	0.61771049	1.2580025	1.28101613	1.23484964	1.02297561	0.91973861	0.96057085	1.38961826	1.61965401	1.68327851	0.86294692	1.7863871	1.54805425
ENSG00000156959	1.7784917	2.5277997	2.0221513	1.2335605	0.76624106	1.42086099	0	0	0	0	0	0	0.03671544	0.03132862	0.01257519	0.02789707	0.02175201	0.01744141	0	0	0	0	0	0	0	0	0	0	0	0	1.23025599	1.39755614	0.85395172	1.16059951	0.78849269	0.70741524	0	0.01457025	0	0	0	0	0.14899585	0.06172351	0.05335062	0	0.01287572	0.01351821
ENSG00000156966	0.05950564	0.06476895	0.03637215	0.1249917	0.04149189	0.12833039	2.49092228	3.27860122	5.39472253	1.81223967	1.67737604	2.0917588	0.20146465	0.33844014	0.24582127	0.07893009	0.06713856	0.24225161	0.28650548	0.30380973	0.18784257	0.27952366	0.1657671	0.11756179	0.1065095	0.06733552	0.19839948	0.16264891	0.22433766	0.20941797	0.41814852	0.51872647	0.41488823	0.39802697	0.39113329	0.29112921	0.24779481	0.39725036	0.4045805	1.6010243	1.68133759	1.57169579	0.16181048	0.2794182	0.36364429	0.051082	0.37754413	0.45897033
ENSG00000156968	0.03054538	0.01753695	0.02133774	0.045124	0.02190711	0.01636632	0.90060972	0.91468012	0.91902639	0.32803549	0.69816892	0.6891097	0.01846708	0.00472729	0.01138509	0.0378854	0.01312895	0.03158156	0.01440673	0.00387457	0.01469306	0.03354191	0.03094233	0.02839845	0.03749031	0.07182256	0.07133651	0.03948333	0.06893741	0.05616235	0.08618895	0.0587267	0.12169718	0.32278313	0.22945851	0.1785543	0.85202346	0.76949512	0.84390159	0.77802847	1.21460539	0.87515281	0.07494173	0.09313682	0.05232673	0.0539411	0.07771453	0.10199058
ENSG00000156970	9.95077507	7.24753181	8.07378239	5.466579	9.2846378	6.17538134	2.78250879	4.32831075	7.97022256	4.4947071	2.97250303	8.91672376	7.92533771	5.47060516	8.32181745	8.51070341	8.32586336	5.11676525	5.08600462	1.80126417	7.24090588	12.193944	13.5509678	13.8555959	7.66544263	13.9659257	7.52471918	14.3236128	11.0149813	9.28564327	3.74073676	0.75306397	5.14559651	3.69731227	4.44037713	9.05236051	2.84080182	3.4164649	3.76381107	10.797351	10.1473486	7.60048933	6.31318593	1.34001764	1.47145965	8.30260855	3.01227582	1.32593696
ENSG00000156973	6.61159801	7.18795664	7.37464841	6.5260675	7.01862033	6.3209621	1.67732712	1.88357533	2.28240483	3.5159911	2.58593685	3.27832334	3.8587641	3.78416416	4.4033868	4.46741772	4.88644434	3.99628446	2.1876086	1.33655178	3.1162446	4.55805707	6.135745	6.79640032	5.92652351	6.7273346	4.45557116	7.22271445	5.32186569	4.54210972	6.59844805	5.92145556	6.90018435	7.52657361	7.54638481	7.14337782	3.73893765	3.72307212	3.44588237	5.06713978	4.23814445	3.67558941	6.41546725	3.6217041	3.65392171	4.71858665	3.80956125	2.59237038
ENSG00000156976	11.8204059	15.1926419	13.7094478	25.423928	9.76791915	22.8290583	13.1294567	14.2697552	12.4227974	13.5131241	14.6308755	11.4298775	12.198063	12.5890689	14.5438914	9.57141412	10.4994558	9.05648684	18.1344062	20.7255988	18.2093358	15.7589358	26.6444493	16.6363189	15.5776687	15.1663697	23.6556914	27.871927	25.7928315	24.1012307	11.7809781	15.4447505	12.8196843	15.0544819	12.3216642	11.5473175	8.49321577	8.98522184	8.64202528	20.1686963	13.5817129	20.5372917	12.7688818	12.400155	13.4060744	8.32666058	11.1809207	13.2666671
ENSG00000156983	1.79224071	1.57956742	1.45358505	1.3577131	1.77836046	1.32528051	0.91517584	1.0900169	1.238353	0.78854587	0.89770185	1.1217033	1.17387181	0.83132718	0.98555424	1.18910258	0.88594273	0.82108303	0.70843364	0.67457931	1.0751855	0.84483234	0.76185194	0.91846343	1.32272496	1.15395032	0.87852529	0.98615101	0.94374455	0.85825091	1.56101397	1.59388636	1.55375993	1.30002438	1.34059529	1.29831926	1.12651244	1.02245549	0.97966735	1.19167826	1.28351581	1.09108174	0.93260301	1.05128884	0.96182732	1.59849614	1.11126712	1.06422888
ENSG00000156990	1.41458083	1.2820611	1.30657716	1.4334623	1.24207557	1.22486415	1.71944873	1.9073289	1.80278581	1.41566674	1.59510784	1.74029959	1.01993525	0.84948079	0.89155595	0.81034444	0.84070839	0.67199839	1.37995198	1.08701743	1.22018449	1.4751522	1.2082895	1.36711167	1.49375628	1.87558465	1.36138726	2.03054289	1.81967965	1.65347263	1.11442994	0.95081977	0.97983499	0.87404812	0.98635593	0.84975762	1.31549365	1.28641309	1.30875624	2.07306158	1.74214961	1.90448876	0.81780732	0.6202546	0.56865681	1.02663559	0.64538015	0.78534483
ENSG00000157005	0	0	0	0	0	0	0	0	0	0	0	0	0	0	0	0	0	0	0	0	0	0	0	0	0	0	0	0	0	0	0	0	0.08555638	0	0	0	0	0	0	0	0	0	0	0	0	0	0	0
ENSG00000157014	1.23038936	0.91430216	0.82677416	0.8839544	1.09483578	0.75936011	1.83348868	1.91720587	1.99925039	1.26070116	1.74340699	1.58474142	1.25106384	0.86625806	1.08411439	1.20251136	1.01660618	0.8606113	0.82369787	0.59286224	1.19211121	1.18421726	1.16183248	1.57444018	1.89675709	2.11820344	1.67948105	1.73368138	1.48481506	1.52467341	1.61578731	1.83475046	1.57239065	1.69493026	1.55206888	1.70039458	1.36031853	1.31308262	1.10102069	1.96771764	2.67571247	1.84647535	1.24227101	1.36995575	1.10017782	1.46437178	1.42648789	1.14779353
[illegible]																																																

ENSG00000158406	60.608127	38.0920169	48.0621053	47.36685	71.1976179	42.1185109	19.3352986	27.3115144	57.3449638	18.4204467	25.5936954	62.6146927	91.8469463	32.4025889	[illegible]
ENSG00000158411	1.87294928	2.17388216	2.12534378	1.9913234	2.08555429	1.8500241	1.97487303	2.04813494	1.67522812	2.22729843	1.96331739	1.98014882	3.04955185	2.96611489	[illegible]
ENSG00000158417	5.61132013	5.64822225	5.7879618	3.909731	6.82391749	4.25575836	9.54163351	10.8305119	13.1803783	12.566744	14.7337935	17.3938834	7.96347498	7.65568265	[illegible]
ENSG00000158423	0.14079246	0.2436213	0.18358999	0.0909953	0.10471604	0.09241043	0.16017808	0.05171525	0.08410795	0.14877207	0.13303011	0.15357803	0.13406415	0.15888124	[illegible]
ENSG00000158427	1.32951188	1.60545916	1.5199974	1.3312706	1.1855635	1.52404284	0.55139308	0.6817034	0.79925115	0.97238065	0.71165662	0.95672805	0.62077075	0.44967499	[illegible]
ENSG00000158428	0.00547659	0.04035138	0.05891609	0.0283165	0.03665951	0.02054064	0.0351849	0.05364365	0.01895306	0.03968218	0.03449766	0.0601247	0.04171897	0.02373202	[illegible]
ENSG00000158435	16.4308477	13.9744983	13.8508983	13.629529	16.6311389	12.8446896	12.319228	13.3552583	13.2035228	14.5760012	11.4514995	12.340577	14.0464519	12.4638787	[illegible]
ENSG00000158445	0.00543962	0.04554428	0.0212794	0.0175784	0.00758584	0.02652256	0.00873684	0.00532815	0.00672325	0.02463393	0.01713238	0	0.18877004	0.14437733	[illegible]
ENSG00000158457	4.30100869	5.62863987	4.94494026	6.3200128	2.89145033	5.37914383	0.05107626	0.07008475	0.07467884	0.2232184	0.12018869	0.10070791	0.03364525	0	[illegible]
ENSG00000158458	0.14840623	0.15939053	0.13262668	0.2166383	0.12703086	0.16602513	0.51063027	0.54135963	0.32321086	0.2456548	0.48191965	0.34357419	0.37900327	0.3395664	[illegible]
ENSG00000158467	1.69778283	2.34253435	1.88686755	2.5260778	1.3587194	2.56901402	0.35454827	0.38474571	0.29637383	0.43612848	0.32035857	0.28054132	0.29949371	0.33526614	[illegible]
ENSG00000158470	28.516699	15.6126615	20.4460158	18.068852	33.7181322	17.3872966	33.8472813	33.3543355	24.2386022	17.420758	38.2476617	23.402169	22.9293992	23.6690573	[illegible]
ENSG00000158473	0	0	0.02798057	0.0184912	0	0.02146146	0.01148819	0.04203633	0.02828958	0.01295659	0	0.01208078	0.95654071	1.34827763	[illegible]
ENSG00000158477	0	0	0	0	0	0	0	0	0	0	0	0	0	0	[illegible]
ENSG00000158480	1.34768777	1.58576958	1.3629103	1.4877308	1.31330922	1.29871827	2.44372005	2.44743072	2.27444806	2.13239252	1.71850219	1.89409967	1.45986928	1.52039245	[illegible]
ENSG00000158481	0	0	0	0	0.1570169	0	0	0	0	0	0	0	0	0	[illegible]
ENSG00000158482	0.12853336	0.19371078	0.14141623	0.0830722	0.04481165	0.12052009	0.15483286	0.12589945	0.11914835	0.14551947	0.10120575	0.08140974	0.14958888	0.13924523	[illegible]
ENSG00000158483	0.34260102	0.28986896	0.26099227	0.1724791	0.39831377	0.23805718	0.5502685	0.6782257	0.64007428	0.77412061	0.5951763	0.63956339	0.37239654	0.281295	[illegible]
ENSG00000158485	0	0	0	0	0	0	0	0	0	0	0	0	0	0	[illegible]
ENSG00000158486	0	0.0012393	0	0.0011958	0.00154813	0.00208183	0.00222879	0.0018123	0.00365891	0.00335156	0.00116547	0.00703126	0.00156604	0.00400881	[illegible]

[illegible]

ENSG00000159763	0	0	0	0	0	0	0	0	0	0	0	[illegible]
ENSG00000159784	4.93714696	4.6267951	5.12499097	4.8556534	3.99816801	4.60914134	0.04370754	0.05766179	0.02855476	0.3088431	[illegible]	[illegible]
ENSG00000159788	2.80002984	2.90965715	2.58564778	2.413702	2.39172697	2.5928477	0.39198619	0.40791843	0.43479222	0.55746517	[illegible]	[illegible]
ENSG00000159792	5.10693461	4.79733124	5.20157895	5.0958888	5.44043077	4.27465534	3.66111641	4.08113099	3.00207784	2.97834916	[illegible]	[illegible]
ENSG00000159840	18.1304032	11.4281032	13.4167218	8.7815416	18.1147147	10.403575	7.2838082	7.34008929	5.76972565	7.87651936	[illegible]	[illegible]
ENSG00000159842	0.64849362	0.67867226	0.80257039	0.8839767	0.62746062	0.76476024	2.94545576	3.34541123	3.04948358	3.264272	[illegible]	[illegible]
ENSG00000159860	0	0.01719816	0.01883297	0.0082973	0.0071613	0.00481504	0	0	0.00634698	0.02325529	[illegible]	[illegible]
ENSG00000159871	0.3154374	0.39180028	0.34143787	0.7798516	0.22720751	0.49620744	0.15248378	0.14098788	0.12718214	0.18861669	[illegible]	[illegible]
ENSG00000159873	3.9754287	3.33140837	3.40657137	3.9290715	3.68887558	3.74643409	3.72698067	4.55597193	4.37635287	2.54587249	[illegible]	[illegible]
ENSG00000159882	0.63749866	0.80764164	0.71655841	0.8314988	0.71604794	0.82008671	0.51890879	0.48033462	0.56331976	0.79163479	[illegible]	[illegible]
ENSG00000159884	1.46885868	1.17279539	1.37406013	1.4790375	1.81387447	1.61281986	0.74793696	0.83927595	0.78670699	0.86281802	[illegible]	[illegible]
ENSG00000159885	1.36663208	1.505311	1.48504625	1.6978331	1.39338165	1.56049806	1.170675	1.13783295	0.96467965	1.24466627	[illegible]	[illegible]
ENSG00000159899	0.10758461	0.11791943	0.13072268	0.0969246	0.05273868	0.13694866	1.69917027	1.69885298	1.35389931	2.02560869	[illegible]	[illegible]
ENSG00000159904	0.02570155	0.00860765	0	0.0332223	0	0.02891905	0.02064027	0.05034974	0.07623974	0.05819618	[illegible]	[illegible]
ENSG00000159905	1.33159374	2.00321774	1.44556713	1.3034636	1.30048581	1.88135094	0.96900788	1.15656967	0.83942206	1.54172035	[illegible]	[illegible]
ENSG00000159915	0.61185409	0.86325803	0.82436712	0.6436522	0.52829694	0.73727673	0.36590982	0.29647013	0.24778878	0.47163415	[illegible]	[illegible]
ENSG00000159917	0.69354356	0.67927017	0.69937497	0.541876	0.55365877	0.63816631	0.8688691	0.65517743	0.57146993	0.67003534	[illegible]	[illegible]
ENSG00000159921	2.49143221	1.96934865	1.93969887	2.1304281	2.65488699	2.01032858	2.46911018	2.43011683	2.60504498	2.64238926	[illegible]	[illegible]
ENSG00000159958	0.27844268	0.4662633	0.20423395	0.3149297	0.1359061	0.4438419	0.39131803	0.47728943	0.51622372	0.34676379	[illegible]	[illegible]
ENSG00000159961	0	0	0	0	0	0	0	0	0	0	[illegible]	[illegible]
[illegible]	[illegible]	[illegible]	[illegible]	[illegible]	[illegible]	[illegible]	[illegible]	[illegible]	[illegible]	[illegible]	[illegible]	[illegible]

ENSG0000160994	0	0	0	0	0	0	0	0	0	0	[illegible]
ENSG0000160999	1.63343277	1.35459896	1.309489	1.1275177	1.54959792	1.21694332	0.12939215	0.14693514	0.19227502	0.31199014	[illegible]
ENSG0000161010	0.36884301	0.56265215	0.4142483	0.5090702	0.31061384	0.50337603	0.54846354	0.50929483	0.39411735	0.74572928	[illegible]
ENSG0000161011	7.79825069	5.77512518	6.39041203	8.3857446	9.1333999	7.10354121	20.8634774	18.3233739	13.6065493	14.5853128	[illegible]
ENSG0000161013	3.30361997	2.63965946	3.13746436	3.0691232	3.26320127	2.59788831	6.04471128	5.75542356	3.84607034	5.10929534	[illegible]
ENSG0000161016	42.3550519	39.7829214	42.5096842	60.368598	44.457534	47.270843	102.332373	119.752573	126.150819	96.4994281	[illegible]
ENSG0000161021	3.69962436	4.01074434	3.30677615	3.4000588	3.14397286	3.43109953	2.92337006	3.0675553	3.05548572	2.19299905	[illegible]
ENSG0000161031	0	0	0	0	0	0.00388875	0	0	0	0	[illegible]
ENSG0000161036	4.94185884	3.39527798	3.94001333	3.2794725	4.69141068	2.83912303	3.19786222	3.52689714	3.8283407	3.48208046	[illegible]
ENSG0000161040	0.05461826	0.05321335	0.0497737	0.0561595	0.04154622	0.03910822	0.19339398	0.15806529	0.19086068	0.19000593	[illegible]
ENSG0000161048	0.68607324	0.8389891	0.67923627	0.6854195	0.60370565	0.58621913	0.35599526	0.51939126	0.3959192	0.31069605	[illegible]
ENSG0000161055	0	0	0	0	0	0	0	0	0	0	[illegible]
ENSG0000161057	18.7640538	14.9799136	16.7799824	14.069397	20.7155436	13.1282324	11.8151265	12.8648953	14.4031196	11.2624639	[illegible]
ENSG0000161082	0.05985763	0.1587706	0.14049524	0.1454612	0.05075258	0.1813988	0.01922806	0.02814289	0.01775586	0.02602292	[illegible]
ENSG0000161091	0.96817454	1.00782131	0.91968481	1.1188716	1.29831006	0.96352612	4.91568996	5.22316782	3.55770113	3.93345697	[illegible]
ENSG0000161103	0	0.00759516	0.01478602	0	0.00421683	0.00283527	0.142664	0.09255684	0.04484796	0.04108062	[illegible]
ENSG0000161132	0	0.03481934	0.02541944	0.100792	0.02899748	0.01949704	0	0	0	0	[illegible]
ENSG0000161133	0.0173418	0.00580791	0	0.0224163	0	0.01300852	0.02785353	0.03397287	0.02572091	0	[illegible]
ENSG0000161149	0.19575547	0.30438598	0.143584	0.1897776	0.13259583	0.15208537	0.36494317	0.40403209	0.42513991	0.48757546	[illegible]
ENSG0000161179	0.12142352	0.12309617	0.12195948	0.2163421	0.10983669	0.13785505	1.32563843	1.35971982	1.4261357	1.25134893	[illegible]
[illegible]	[illegible]	[illegible]	[illegible]	[illegible]	[illegible]	[illegible]	[illegible]	[illegible]	[illegible]	[illegible]	[illegible]

ENSG0000162461	0.40287175	0.51811177	0.37036125	0.2916249	0.27866574	0.38077719	0.49177496	[illegible]
ENSG0000162482	0.09423901	0.23986656	0.1658953	0.2192669	0.15770557	0.21207306	0.19677053	[illegible]
ENSG0000162490	11.1922225	5.37789583	8.51291745	9.8735751	8.25255688	11.3641254	0.05989779	[illegible]
ENSG0000162493	0.00254559	0.00170508	0.00248954	0.0032905	0.00283997	0.00190951	0.18603137	[illegible]
ENSG0000162494	0	0	0	0	0	0	0	[illegible]
ENSG0000162496	0.34386725	2.3870329	1.29932544	2.5861154	0.28336525	2.43287505	2.51046485	[illegible]
ENSG0000162510	0.00563851	0.00377677	0	0.0145769	0	0.00845918	0.00452815	[illegible]
ENSG0000162511	0.04675494	0.02087813	0.00762092	0.0201454	0.00869363	0.05845341	0.13141701	[illegible]
ENSG0000162512	31.6802871	25.0261856	27.2636903	17.989424	23.2574326	14.7834621	0.33964765	[illegible]
ENSG0000162517	5.76751485	4.66148749	5.0699525	6.5017414	4.90025097	4.91404057	4.60831952	[illegible]
ENSG0000162520	0.69887988	0.61508899	0.70733472	0.3834129	0.532644	0.52424718	6.38261952	[illegible]
ENSG0000162521	12.0467553	12.4902628	11.8791822	10.421463	10.5422261	10.707091	3.97477236	[illegible]
ENSG0000162522	0.17409594	0.17062209	0.16667822	0.1800315	0.09813668	0.15671243	1.24211983	[illegible]
ENSG0000162526	0.09833635	0.23053537	0.18032098	0.2383335	0.05485411	0.13830852	0.14807147	[illegible]
ENSG0000162542	0.43425434	0.20980818	0.24367628	0.3435428	0.26871023	0.21716332	0.58123121	[illegible]
ENSG0000162543	0	0.00387507	0.00565789	0	0	0	0	[illegible]
ENSG0000162545	29.5882935	29.9200771	25.8028319	22.246392	31.782081	18.9463166	6.33094896	[illegible]
ENSG0000162551	0.00216467	0.01884907	0.01905306	0	0.002415	0.00487131	0.00347678	[illegible]
ENSG0000162552	0	0.00349943	0.01021887	0.0135065	0	0.00783801	0	[illegible]
ENSG0000162571	0	0.01258628	0	0.0048578	0.00209637	0.0084572	0.00301806	[illegible]
[illegible]	[illegible]	[illegible]	[illegible]	[illegible]	[illegible]	[illegible]	[illegible]	[illegible]

ENSG00000163113	3.5922398	4.10297172	3.88588767	3.5548185	3.01847255	3.934089	5.01639125	5.63785168	4.93751915	3.62357914	5.62103965	5.51221168	3.23174655	3.17213542	3.18965874	2.89072746	2.52348418
ENSG00000163114	0	0	0	0	0	0	0	0	0	0	0	0	0	0	0	0.04483242	0
ENSG00000163116	0.02512452	0.03702337	0.0294856	0.0324764	0.01681799	0.02638514	0.04035375	0.02953161	0.02981124	0.01365351	0.0506439	0.0297047	0.00850626	0	0.00874028	0	0
ENSG00000163121	0.02333375	0.01172199	0.005705	0.0150808	0.00325402	0.01312742	0.00468469	0.01714169	0.01442	0.01056695	0.02204728	0.00985267	0.01974992	0	0.01014664	0.05064643	0
ENSG00000163125	5.43190944	6.38494797	5.35596444	4.9939521	5.03403049	5.31812279	2.67617664	3.02440372	2.9343288	2.26681797	2.22613781	2.77002427	1.68974667	1.61080311	1.53008249	2.0015783	1.8501217
ENSG00000163126	0.03441816	0.12103261	0.05329554	0.0852715	0.05439769	0.09896869	0.08983117	0.05899751	0.05388413	0.13248656	0.09756173	0.05813222	0.02913187	0.01864327	0.02743891	0.03873613	0.0258887
ENSG00000163131	0.16532207	0.05131638	0.09069967	0.1146673	0.0899709	0.07561719	1.50899887	1.29942392	0.71566919	0.46381624	1.0109027	0.58229406	6.9248408	7.19894182	7.07678334	9.95403358	9.12320592
ENSG00000163132	0.02633149	0.07054899	0.07725516	0	0.05140892	0.0197519	0.05286539	0.0773757	0.0650903	0.08347159	0.08293249	0.10006614	2.87505298	2.69570063	3.77856656	2.75604909	3.20858135
ENSG00000163138	0.45031368	0.58813876	0.49256437	0.4667272	0.40053802	0.44551543	0.40611923	0.52247069	0.52437508	0.40693025	0.52983881	0.55094341	0.35336684	0.34671265	0.36308874	0.49872712	0.39504197
ENSG00000163141	0.08937337	0.10262349	0.08324338	0.1595352	0.05222839	0.16281431	0.08544465	0.09587925	0.07364237	0.14647641	0.05361646	0.06828758	0.06483987	0.04610556	0.09253299	0.04926652	0.04481667
ENSG00000163145	0	0	0	0	0.00143266	0	0	0	0	0.00232618	0	0	0	0.00278235	0	0	0
ENSG00000163154	0	0	0.02675731	0	0	0.0205232	0	0	0	0.02478031	0	0	0	0	0	0	0
ENSG00000163155	12.4494543	12.12697	11.9852445	18.210106	11.1371293	15.3931425	4.57150459	5.0409315	4.98341208	4.35939898	5.35241788	5.1668845	2.47568779	1.99547173	2.12519953	1.91976612	1.55954787
ENSG00000163156	6.0333393	5.72665166	5.82662583	6.6454526	5.36956628	5.37920673	5.71721138	5.64612556	5.51520305	3.8354346	5.62041002	5.82075089	2.93294363	2.05597306	2.27049097	2.85546122	2.09508461
ENSG00000163157	0	0	0	0	0	0	0.00454964	0	0	0	0	0	0	0	0	0	0
ENSG00000163159	13.5778508	13.9343092	13.6755963	13.337604	11.695377	11.8395824	5.7530466	6.13364389	6.57669057	4.92369936	5.83117051	6.56975435	3.1298397	2.86419725	2.93876587	2.83346776	2.33735963
ENSG00000163161	3.03943628	3.38729173	2.91045411	3.1842843	3.13769436	3.1392124	4.0533488	4.24419481	4.6737254	5.11671386	3.82300004	4.35724194	3.03259383	2.23687783	2.56272717	2.93364327	2.60686436
ENSG00000163162	4.46801324	3.99525688	4.06307128	4.6340398	4.03160248	4.55044885	7.12665948	7.10525182	6.24267693	7.77765701	7.34204708	5.51443948	5.43349293	4.95516551	4.89566706	4.57425161	4.90165743
ENSG00000163166	6.30096512	6.29380483	6.26196936	5.4939539	6.56990103	5.29303029	5.06457354	5.57787964	6.68134089	6.25864655	6.14976762	6.83883148	5.50817332	5.07925052	5.10596936	5.60775794	5.05875137
ENSG00000163170	2.309589	1.26699945	1.93678995	1.3103399	2.80111479	1.46594475	1.49388939	1.54058939	1.7721784	1.65170736	1.4087551	1.738633	0.79167371	0.7189201	0.59077694	0.93253683	0.98259625

ENSG00000163684	2.20820202	2.08047643	2.03202419	[illegible]
ENSG00000163686	1.14257908	1.32216156	1.32168173	[illegible]
ENSG00000163687	0	0	0	[illegible]
ENSG00000163689	0.00161391	0.00648613	0.00789186	[illegible]
ENSG00000163694	0.1459587	0.26822829	0.22692793	[illegible]
ENSG00000163697	3.51691196	3.53885467	3.20148881	[illegible]
ENSG00000163701	0.02193139	0.02518284	0.03064072	[illegible]
ENSG00000163702	0.88463351	1.02019359	0.90246509	[illegible]
ENSG00000163703	2.09225168	2.20416402	2.09543088	[illegible]
ENSG00000163704	0.52068036	0.6071936	0.51287947	[illegible]
ENSG00000163705	0.0086546	0	0	[illegible]
ENSG00000163710	0.02877655	0.08673746	0.0562859	[illegible]
ENSG00000163714	6.47601902	7.56434601	6.18800135	[illegible]
ENSG00000163719	1.91638972	1.97472744	1.87742888	[illegible]
ENSG00000163728	2.39689785	4.43915718	2.8410824	[illegible]
ENSG00000163734	0.0702343	0.07317963	0.07631987	[illegible]
ENSG00000163735	0.26667633	0.28069491	0.22354686	[illegible]
ENSG00000163736	0	0	0	[illegible]
ENSG00000163737	0	0.07148341	0.05218561	[illegible]
ENSG00000163738	0.83037509	0.97887075	0.86959757	[illegible]
ENSG00000163739	0.38575392	0.12919204	0.03772604	[illegible]
ENSG00000163743	1.46665482	1.58459289	1.54911104	[illegible]
ENSG00000163746	0.00710219	0.00475716	0.01389163	[illegible]
ENSG00000163749	0.01635695	0.04163336	0.0415917	[illegible]
ENSG00000163751	0	0.01844339	0	[illegible]
ENSG00000163754	3.82957935	3.59302366	3.73708834	[illegible]
ENSG00000163755	9.8213672	8.43532084	8.78671875	[illegible]
ENSG00000163762	0	0	0	[illegible]
ENSG00000163781	28.4215416	23.7032349	23.9663407	[illegible]
ENSG00000163785	9.19080493	9.42275066	9.40868576	[illegible]
ENSG00000163788	5.49204008	5.36494656	4.60887333	[illegible]
ENSG00000163792	0	0	0	[illegible]
ENSG00000163793	0	0.01019263	0	[illegible]
ENSG00000163794	1.45945991	2.49823287	1.46697352	[illegible]
ENSG00000163795	4.49462357	4.00185539	4.41948585	[illegible]
ENSG00000163798	5.63844245	5.79321584	5.3665911	[illegible]
ENSG00000163803	0.01059457	0.00283856	0.00932517	[illegible]
ENSG00000163806	0.04970775	0.11781321	0.06357122	[illegible]
ENSG00000163807	22.7274938	24.6111533	21.5548908	[illegible]
ENSG00000163808	10.7885426	8.61222452	8.9736508	[illegible]
ENSG00000163810	0	0	0	[illegible]
ENSG00000163811	9.66792318	8.76698532	9.19621332	[illegible]
ENSG00000163812	3.40128776	2.91580262	3.16504319	[illegible]
ENSG00000163814	0.09687085	0.02703568	0	[illegible]
ENSG00000163815	0.02690295	0	0	[illegible]
ENSG00000163817	0.0055251	0.01850399	0.01080689	[illegible]
ENSG00000163818	1.77845398	2.31181611	2.05040276	[illegible]
ENSG00000163820	1.37888469	1.70118753	1.45429108	[illegible]
ENSG00000163823	0.50746203	0.12647669	0.25391518	[illegible]
ENSG00000163825	0	0	0	[illegible]
ENSG00000163827	0.0039913	0.04812184	0.02342051	[illegible]
ENSG00000163832	2.38953672	1.87255048	1.99651657	[illegible]
ENSG00000163833	0.0108774	0	0	[illegible]
ENSG00000163840	12.134706	9.76811289	11.5790109	[illegible]
ENSG00000163848	6.19541324	6.89455338	6.96183443	[illegible]
ENSG00000163864	0	0	0	[illegible]
ENSG00000163866	2.89807284	1.93927945	2.25855348	[illegible]
ENSG00000163867	0.50928009	0.54813067	0.5165137	[illegible]
ENSG00000163870	1.59913594	1.68651641	1.59995163	[illegible]
ENSG00000163872	16.8172208	14.8354527	14.8814775	[illegible]
ENSG00000163873	18.3648798	14.6937402	11.2848878	[illegible]
ENSG00000163874	0.56173838	0.32922861	0.46353098	[illegible]
ENSG00000163875	5.65603397	5.16734828	5.64535079	[illegible]
ENSG00000163877	2.95571401	2.64350482	2.78542053	[illegible]
ENSG00000163879	3.24049014	3.8177892	3.35072438	[illegible]
ENSG00000163882	3.74298322	2.86086228	3.2526631	[illegible]
ENSG00000163884	3.48010968	6.27372875	4.97898512	[illegible]
ENSG00000163885	0	0	0	[illegible]
ENSG00000163888	10.8852278	6.19743342	7.45189011	[illegible]
ENSG00000163898	0.02170645	0.01744719	0.02547422	[illegible]
ENSG00000163900	2.53095818	2.53552704	2.28918482	[illegible]
ENSG00000163902	78.4143744	60.3423351	67.9375391	[illegible]
ENSG00000163904	8.29453815	8.71575006	8.03063087	[illegible]
ENSG00000163909	0	0	0	[illegible]
ENSG00000163913	2.30308434	2.04485255	1.89631582	[illegible]
ENSG00000163914	0.01243431	0.0083287	0	[illegible]
ENSG00000163915	0.01300635	0	0.00635999	[illegible]
ENSG00000163918	8.71967117	5.66131425	6.8317601	[illegible]
ENSG00000163923	0	0.10003703	0.10224316	[illegible]
ENSG00000163930	10.4865068	10.7739879	10.2284895	[illegible]
ENSG00000163931	12.5198968	10.1987284	11.1499321	[illegible]
ENSG00000163932	2.29094034	1.46827039	1.85767951	[illegible]
ENSG00000163933	3.10910887	2.48615996	2.52724874	[illegible]
ENSG00000163935	1.70211818	1.45827478	1.40171806	[illegible]
ENSG00000163938	8.67491524	7.30128221	8.09022632	[illegible]
ENSG00000163939	2.49879003	3.14476242	2.5321994	[illegible]
ENSG00000163945	0.4128161	0.64817707	0.41353386	[illegible]
ENSG00000163946	8.70400962	9.86360068	8.93250327	[illegible]
ENSG00000163947	0.99446782	0.98584326	0.89978566	[illegible]
ENSG00000163950	7.30018049	4.953352	5.77532299	[illegible]
ENSG00000163956	7.2412198	6.49138616	6.5795487	[illegible]
ENSG00000163958	0	0.00562847	0	[illegible]
ENSG00000163959	0.01179604	0.00395059	0.00576816	[illegible]
ENSG00000163960	11.8267198	14.4328459	12.3417349	[illegible]
ENSG00000163961	21.0225512	14.7275336	17.7204044	[illegible]
ENSG00000163964	5.46660429	4.89608193	5.37147877	[illegible]
ENSG00000163975	0.13884323	0.11523838	0.13873806	[illegible]
ENSG00000163982	0	0	0	[illegible]
ENSG00000163993	0.02024481	0.13560296	0.05939719	[illegible]
ENSG00000163995	0.01434614	0.0207358	0.01107653	[illegible]
ENSG00000164002	0.8305808	0.6600902	0.61379061	[illegible]
ENSG00000164007	0.00809589	0.02169102	0.01979409	[illegible]
ENSG00000164008	1.1257535	1.10553584	1.09658039	[illegible]
ENSG00000164010	0.7845427	0.78976749	0.76948621	[illegible]
ENSG00000164011	0.68510033	0.61612067	0.65666889	[illegible]
ENSG00000164022	5.84400724	4.90502643	5.57498485	[illegible]
ENSG00000164023	0.59320013	0.27346933	0.49793313	[illegible]
ENSG00000164024	3.2856839	2.58201686	2.7675437	[illegible]
ENSG00000164031	1.94349026	2.22109523	2.08193323	[illegible]
ENSG00000164032	50.8828658	31.476546	40.010207	[illegible]
ENSG00000164035	0.08706818	0.02721584	0.04541393	[illegible]
ENSG00000164037	0.15895227	0.11227613	0.08761866	[illegible]
ENSG00000164038	0.60538169	0.60562528	0.53284717	[illegible]
ENSG00000164039	1.46650945	1.9625446	1.72284922	[illegible]
ENSG00000164040	2.55225375	2.58281882	2.73436311	[illegible]
ENSG00000164045	8.90628406	4.9705682	5.71383003	[illegible]
ENSG00000164047	0	0	0	[illegible]
ENSG00000164048	1.63227837	1.2041828	1.32320109	[illegible]
ENSG00000164049	0.01685832	0.01355037	0.00989228	[illegible]
ENSG00000164050	2.24497737	3.10569737	2.17683549	[illegible]
ENSG00000164051	1.7070297	1.32624132	1.4985844	[illegible]
ENSG00000164053	1.49675079	1.25207688	1.21587717	[illegible]
ENSG00000164054	11.3285029	7.91378166	9.78064006	[illegible]
ENSG00000164056	17.2919242	13.2736049	13.9369211	[illegible]
ENSG00000164061	0.48295739	0.86007931	0.45545495	[illegible]
ENSG00000164062	7.69530821	6.45876778	6.83826652	[illegible]
ENSG00000164066	0.84713772	1.23386095	0.99855581	[illegible]
ENSG00000164068	1.04450382	1.2607262	0.99423552	[illegible]
ENSG00000164070	3.97528036	3.31116223	3.71103676	[illegible]
ENSG00000164073	0.19493794	0.22850192	0.20199377	[illegible]
ENSG00000164074	0.37753065	0.47457553	0.35151595	[illegible]
ENSG00000164076	0.06492394	0.03177902	0.06837863	[illegible]
ENSG00000164077	1.49168446	1.34555045	1.2351856	[illegible]
ENSG00000164078	0	0.00177628	0.005187	[illegible]
ENSG00000164080	2.84886589	2.96353909	2.59815131	[illegible]
ENSG00000164081	2.59652311	1.59665608	2.14868328	[illegible]
ENSG00000164082	0.21983213	0.07193749	0.16903941	[illegible]
ENSG00000164086	0.88690353	0.78236851	0.72008551	[illegible]
ENSG00000164087	6.79690287	4.14434466	5.09269533	[illegible]
ENSG00000164088	0.91619468	0.83619449	0.92706196	[illegible]
ENSG00000164089	0.02413987	0.50528955	0.09443342	[illegible]
ENSG00000164091	26.7466993	27.3803747	24.8405622	[illegible]
ENSG00000164093	0.00375769	0.00125848	0.00551244	[illegible]
ENSG00000164096	41.8103905	29.7141313	38.0894274	[illegible]
ENSG00000164099	2.2377125	2.76760915	2.7530515	[illegible]
ENSG00000164100	2.32526804	3.33203166	2.7338551	[illegible]
ENSG00000164104	70.8651771	49.4886436	61.2016204	[illegible]
ENSG00000164105	21.4015663	16.7242931	19.4145584	[illegible]
ENSG00000164106	0.29241763	0.5911591	0.81114098	[illegible]
ENSG00000164107	0.006633	0	0	[illegible]
ENSG00000164109	10.2943195	6.200037	7.75358966	[illegible]
ENSG00000164111	25.71228	19.4266602	24.1368905	[illegible]
ENSG00000164112	0.01161641	0.0136165	0.00852048	[illegible]
ENSG00000164113	0	0	0	[illegible]
ENSG00000164114	2.14636094	2.71168532	2.19191319	[illegible]
ENSG00000164116	0.10208422	0.20237014	0.13513227	[illegible]
ENSG00000164117	2.29138007	2.69346118	2.62831007	[illegible]
ENSG00000164118	1.01378324	0.94684169	0.969119	[illegible]
ENSG00000164120	0.04742574	0.07147465	0.04251639	[illegible]
ENSG00000164122	0.00600629	0.02011553	0.01762212	[illegible]
ENSG00000164123	0.01421064	0.00951851	0.02779551	[illegible]
ENSG00000164124	0.09579736	0.03072766	0.06597753	[illegible]
ENSG00000164125	0.93993101	1.17425083	1.04925911	[illegible]
ENSG00000164128	1.49141889	1.45684002	1.21548407	[illegible]
ENSG00000164129	1.54046553	1.28643519	1.33045764	[illegible]
ENSG00000164134	6.52077952	5.53164395	5.35509318	[illegible]
ENSG00000164136	0.17191546	0.12105691	0.14441955	[illegible]
ENSG00000164142	0.01649424	0.01288945	0.0241966	[illegible]
ENSG00000164144	2.23033846	1.98034235	2.06029577	[illegible]
ENSG00000164151	12.6518658	10.8052227	11.2651432	[illegible]
ENSG00000164161	0.1411286	0.19041078	0.17154068	[illegible]
ENSG00000164162	0.89393233	0.85154422	0.93183507	[illegible]
ENSG00000164163	10.0704307	7.37994084	8.19461159	[illegible]
ENSG00000164164	1.51300815	1.85387511	1.68665652	[illegible]
ENSG00000164167	6.23047728	5.2426735	5.94730969	[illegible]
ENSG00000164168	8.25892742	9.05615901	8.39148666	[illegible]
ENSG00000164169	1.15340729	1.40745514	1.18385377	[illegible]
ENSG00000164171	0.43150911	0.38676778	0.31711596	[illegible]
ENSG00000164172	2.85955833	2.5999556	2.6387752	[illegible]
ENSG00000164175	0.00675676	0.00226289	0.00330399	[illegible]
ENSG00000164176	3.5363269	3.55898319	7.22975429	[illegible]

ENSG00000164180	1.08857257	1.51830478	1.12973595	0.9062459	0.93887458	1.16717073	1.10175141	1.13505682	0.88240115	0.88833742	0.997665	0.81908594	0.97569797	0.8749596	1.04045282
ENSG00000164181	0.07312187	0.10137346	0.13969762	0.0769337	0.07968071	0.08418919	0.07510996	0.0782861	0.05212444	0.03850473	0.08569355	0.05457097	1.05214834	0.88611152	1.00123323
ENSG00000164182	4.89166185	3.31174396	4.77109733	4.1983653	5.9121251	4.27105289	5.86087532	6.42720283	7.69725862	5.16890392	7.18972885	8.40637247	7.16770488	7.10850878	6.66729689
ENSG00000164185	0.19432229	1.12185653	0.40723652	0.5980577	0.09807358	0.77741857	0.02972483	0.00906382	0.01829929	0.02514316	0.03497308	0.04688715	0.03916103	0.10024617	0.02414306
ENSG00000164187	5.43448081	5.73081395	5.11108382	5.2093289	6.09299241	5.29168738	3.93449967	4.00377225	3.40417699	3.66638688	4.00019066	3.81895132	3.20739429	2.87170592	3.42058985
ENSG00000164188	0.2195721	0.87378589	0.52210703	0.3339089	0.12248199	0.32941298	0.33537812	0.21507262	0.13622532	1.5285804	0.6237551	0.25814552	0.03279671	0.02332069	0.00374434
ENSG00000164190	4.13397008	5.49537397	4.88087679	4.0819057	4.35531597	4.47078404	2.47485826	2.87944664	3.53198608	3.21522153	4.61396435	4.54480851	3.94434745	4.24582704	3.80678776
ENSG00000164197	4.38878313	6.04220833	5.29566809	3.4304427	2.84353971	3.48379021	0.51957731	0.60950803	0.55008419	0.75021512	0.65933712	0.80042678	1.08127701	1.32144988	1.08952161
ENSG00000164199	0.35389078	1.14812011	0.50698376	0.6358591	0.25560059	0.63626672	0.01868894	0.01336249	0.01071187	0.00944864	0.01516461	0.01016532	0.02037664	0.01217091	0.01256235
ENSG00000164209	3.03060518	3.7296211	3.20006911	3.1933296	3.08356777	3.0558518	3.94634311	3.97388275	3.2602853	2.52918553	4.17721831	3.69383981	4.53973423	4.29883147	4.292687
ENSG00000164211	5.82991404	3.72053219	4.67638631	2.5553141	6.53693863	5.17799654	5.82258332	5.0259376	4.67451751	3.83035684	5.05991758	4.2465967	3.81097492	7.29914276	7.21892175
ENSG00000164219	4.83991952	5.54934044	4.7986011	3.9982985	4.90228685	3.93536412	3.76017936	3.43376887	3.46628263	2.64226011	3.85101385	4.01560118	4.01955311	3.52836906	3.96662223
ENSG00000164220	0.21415109	0.12102902	0.2879744	0.1038057	0.36957331	0.16064006	46.4236766	48.2519768	29.0923151	20.0447091	31.9619597	22.1428753	0.07363672	0.02899979	0.09312324
ENSG00000164221	2.36020683	2.0115233	2.30536894	2.045279	1.98796066	1.98184684	0.96657127	1.08403508	1.03914924	0.8720936	0.90608594	1.11807212	1.02121362	0.69965053	0.74804786
ENSG00000164236	0.03060754	0.04783664	0.1197345	0.0659397	0.05122061	0.13775686	0.20483454	0.08994099	0.16645314	0.1940536	0.19280031	0.09477616	0.06044856	0.07736948	0.04436545
ENSG00000164237	3.82277234	5.23651225	4.78646457	8.7552896	3.87140039	7.77875215	3.2342806	3.13266896	2.52081488	3.20391288	4.74139958	3.1919528	0.3144488	0.34997402	0.36524345
ENSG00000164241	0.16974016	0.12943699	0.16089505	0.3071726	0.2068496	0.24094057	0.31037661	0.36321818	0.32276182	0.1915806	0.41781524	0.43665226	0.2166098	0.25744065	0.19077361
ENSG00000164244	4.08925976	4.66848122	3.98052493	5.2556409	3.98980956	4.35180303	3.01955708	3.20645077	3.35338774	2.73403006	2.66552401	2.75552555	5.60443102	5.93282241	7.06849056
ENSG00000164251	0.01830715	0.11649299	0.04476016	0.0946565	0.0255303	0.04806427	2.66106423	3.21879409	3.67918993	0.58034207	3.15973479	2.61280342	34.9807639	6.74317075	10.8108115
ENSG00000164252	8.56647039	9.74961327	8.98780502	8.0321929	8.47503631	7.9876831	7.59766755	8.64247408	7.35716736	6.91567422	8.86834788	8.34586936	7.38271867	6.72679482	6.84400127
[illegible]	[illegible]	[illegible]	[illegible]	[illegible]	[illegible]	[illegible]	[illegible]	[illegible]	[illegible]	[illegible]	[illegible]	[illegible]	[illegible]	[illegible]	[illegible]

ENSG00000164867	0.00188743	0.00379269	0.00184587	0.0024397	0.00105285	0.00707904	0.00757873	[illegible]	[illegible]
ENSG00000164871	0	0	0	0	0	0	0	[illegible]	[illegible]
ENSG00000164877	0.44503187	0.38233205	0.3004055	0.3470292	0.39733212	0.44722263	0.67788297	[illegible]	[illegible]
ENSG00000164879	0.19539755	0.20359169	0.22294451	0.2525741	0.05449846	0.1628584	0.14820114	[illegible]	[illegible]
ENSG00000164880	6.90458023	8.33556147	6.16044132	7.0495553	6.04589186	6.36293504	6.45560759	[illegible]	[illegible]
ENSG00000164885	5.89065879	4.85062374	5.2456434	5.3876867	5.06456019	4.38832556	4.20441035	[illegible]	[illegible]
ENSG00000164889	3.62643906	3.84069111	3.2226743	3.5736443	3.21676008	3.38571473	3.68315553	[illegible]	[illegible]
ENSG00000164893	0.01582216	0.01059793	0	0.010226	0	0	0	[illegible]	[illegible]
ENSG00000164896	2.8946193	2.91225328	2.70238429	3.5055522	2.73095719	3.01994673	1.47938422	[illegible]	[illegible]
ENSG00000164897	5.39782634	3.67697888	4.22403597	4.8491251	5.13530366	3.99662898	2.43397392	[illegible]	[illegible]
ENSG00000164898	2.63935744	2.3393975	2.36984917	3.999898	3.17938438	3.24285392	0.7491695	[illegible]	[illegible]
ENSG00000164900	0	0	0	0	0	0	0	[illegible]	[illegible]
ENSG00000164902	6.02901205	6.45563675	6.22429717	5.6143984	6.06307385	5.48115701	7.82180948	[illegible]	[illegible]
ENSG00000164904	3.11977994	3.41249126	3.21678144	3.7229842	2.55743787	3.13741565	2.4119618	[illegible]	[illegible]
ENSG00000164916	8.98435164	9.8550623	8.10058216	11.393216	8.86765755	11.0244914	3.94942434	[illegible]	[illegible]
ENSG00000164919	11.2187749	9.15038878	11.0078814	11.332714	14.4270438	13.7175821	5.85803902	[illegible]	[illegible]
ENSG00000164920	0.00272119	0	0	0	0	0	0.00218532	[illegible]	[illegible]
ENSG00000164924	13.5020711	11.5725567	12.32007	14.016146	14.7817863	12.8376003	8.79178278	[illegible]	[illegible]
ENSG00000164929	38.2351157	11.0674447	23.1634162	6.6167758	41.6472836	11.4019479	0.44427259	[illegible]	[illegible]
ENSG00000164930	2.08319687	1.48818674	1.66638684	1.5820997	2.67320685	1.42149332	1.95647629	[illegible]	[illegible]

[illegible]

ENSG00000165623	0	0	0	0	0	0	0	0	0.01002776	0	0.02555302	0	0	0	0	0	0
ENSG00000165626	1.6597492	1.70194374	1.5179515	1.1650911	1.53838221	1.34719398	0.59825323	0.62097575	0.4871849	0.76610543	0.59318839	0.5538462	0.28751297	0.25322384	0.2632483	0.34228637	0.3010405
ENSG00000165629	19.4804817	15.831372	18.1346412	20.136351	23.080967	19.8003056	12.4201847	12.9197032	13.9492753	13.2420079	14.6837761	14.9861363	10.3570132	10.5910504	10.819672	13.0448877	12.4045999
ENSG00000165630	2.19563408	2.21508398	2.33285903	1.9621525	2.15721059	1.90455524	2.80815236	2.54005756	3.0108178	2.58604462	3.36941648	3.54818213	1.23128993	1.22356818	1.1944419	1.29873872	1.20975201
ENSG00000165632	2.36166197	2.88017116	2.6331452	2.4284974	2.54989708	2.19333694	1.67877547	1.87264448	2.20761929	1.37807419	1.81683425	2.46928405	1.9597394	1.26849061	1.08737433	1.67836764	1.36339828
ENSG00000165633	0.09786712	0.15217649	0.2495356	0.1265047	0.06629073	0.1258501	1.87223613	2.40338785	2.2205103	2.96629002	2.31178465	2.266936	1.49695951	1.22684093	1.35575554	1.32511374	1.28824218
ENSG00000165637	7.96524461	5.31279235	6.67515468	8.9642299	8.16702918	8.3353935	4.42060638	4.74601025	4.79647402	3.49581951	3.54202285	4.18999648	3.98571938	3.22984531	3.6094504	4.7258921	3.82946233
ENSG00000165643	0	0	0	0	0	0	0	0	0	0	0	0	0	0	0	0	0
ENSG00000165644	0.26701347	0.13413739	0.26706925	0.2588596	0.30127673	0.24126256	0.48734499	0.50525125	0.43503018	0.57162219	0.38990605	0.22549267	0.15922929	0.18407847	0.14777681	0.18147802	0.12780893
ENSG00000165646	0	0	0	0	0	0	0	0	0.00199751	0	0	0	0	0.00437705	0	0	0
ENSG00000165650	6.83069432	7.88628938	6.87126301	8.5630435	10.3134453	8.51989797	6.55421168	7.55141025	8.13063072	9.13760411	10.3639089	10.8285644	7.47716768	6.31875811	7.22373224	12.2160518	11.6847608
ENSG00000165655	2.03235353	1.20248397	1.41340818	1.9483986	1.70998252	1.89717911	2.52073714	2.37225855	1.92861567	2.05038951	1.82073196	1.45887286	0.70241674	0.42199793	0.55971811	0.88225393	0.49258071
ENSG00000165659	0.9271594	1.6075773	1.14057127	0.7242673	0.59252784	0.71054504	0.00293141	0.00178772	0	0	0	0	0.01235838	0.03954442	0.03492055	0.01408518	0.02471075
ENSG00000165660	10.696568	10.3538658	10.1871468	8.6885957	9.9085693	9.27621194	13.6335023	15.1726719	14.9275727	12.2540625	14.8192813	15.3283136	8.0963437	6.73341673	7.39162567	10.5057774	9.83640849
ENSG00000165661	4.13278581	3.81530924	3.5966076	3.6697961	3.67520508	3.41011596	6.20015771	7.4449777	7.48289993	6.0433012	6.52708411	7.02579242	9.98206247	9.29831957	10.8140633	11.5270595	9.80336917
ENSG00000165669	2.53889085	2.54838558	2.65565592	2.5746642	2.40387531	2.40438921	2.76382404	3.1542494	2.95176199	2.31304443	3.09830291	2.97568805	2.35520728	2.00696146	2.26213086	3.12568014	2.74952886
ENSG00000165671	0.51636351	0.53534068	0.37501933	0.4940939	0.48459852	0.42712262	1.60632854	1.7341103	1.89550361	0.82106652	1.84328576	2.00220867	2.29208884	2.21381137	2.35408887	2.22303224	2.25427381
ENSG00000165672	20.9372061	15.4859132	17.0751877	18.914016	21.9381177	17.2062043	19.885745	20.9022364	21.9720633	14.5257199	17.1218939	19.1661569	12.5855483	10.6813033	12.0882176	17.300108	16.200434
ENSG00000165675	0.69877629	0.62328458	0.74005371	0.533326	0.8794811	0.54392461	0.86995396	0.82547571	0.80811964	0.74421117	0.99977626	1.07496014	0.78018061	0.54398333	0.71002852	0.98404068	0.83991409
ENSG00000165678	66.8979138	58.1901851	65.7952602	73.65017	76.7969804	68.314036	83.0004039	82.8248724	80.8610925	60.536087	87.6186081	81.3214213	45.9189044	46.384775	49.2239006	53.649994	52.8826209

[illegible]

ENSG00000166311	2.1683617	1.93653738	1.97687669	2.051855	2.04776101	1.77959783	4.61445637	4.36472194	2.70569471	2.94276928	[illegible]
ENSG00000166313	2.85667328	2.44015941	2.62682356	2.8568814	2.58122795	2.74839379	0.83933086	0.88408945	0.75733465	0.78870702	[illegible]
ENSG00000166317	0.188144	0.22503899	0.2365733	0.2562266	0.09370601	0.20413663	0.02967912	0.03619954	0.02989825	0.01521485	[illegible]
ENSG00000166321	0.02352321	0.01772575	0.0143783	0.0076016	0.0180424	0.03529069	0.15584991	0.14112718	0.1279264	0.17577042	[illegible]
ENSG00000166323	0.04747919	0.06649575	0.05065505	0.1283241	0.05537753	0.06799289	0.03812934	0.05918977	0.0234733	0.03909362	[illegible]
ENSG00000166326	18.3133112	16.1387047	17.0599792	14.825057	21.6880466	15.0983361	25.6050192	29.0315097	27.3245501	24.2457466	[illegible]
ENSG00000166329	0	0	0	0	0	0	0	0	0	0	[illegible]
ENSG00000166333	6.24400092	4.657452	5.78965847	4.4848482	5.74744914	4.28972934	3.83342666	3.78517166	3.59162762	3.7621499	[illegible]
ENSG00000166337	5.48138239	4.45600637	5.57666392	5.0259944	5.47734041	4.30991214	6.23558248	6.52784715	5.64799994	6.4289944	[illegible]
ENSG00000166340	4.06778909	5.05096169	4.3348638	4.9859286	3.60101466	4.60796149	1.60199208	1.62438949	0.96905288	1.16190752	[illegible]
ENSG00000166341	29.1543944	27.0677258	23.6949905	27.315765	27.314259	25.9755712	6.34627462	7.20376925	6.71236962	16.2432532	[illegible]
ENSG00000166342	0.06754736	0.16481849	0.18638386	0.1060227	0.12784067	0.10857633	0.17629815	0.17485982	0.22780046	1.46174528	[illegible]
ENSG00000166343	0.26132221	0.30266946	0.23427106	0.274454	0.17310317	0.34712635	0.24046606	0.36795284	0.27184876	0.39940671	[illegible]
ENSG00000166347	2.34443454	1.70816176	2.0214578	1.797318	2.36338684	1.84046926	0.98644416	1.16957215	1.21147291	1.8862184	[illegible]
ENSG00000166348	1.41660899	1.64085469	1.57821779	0.8487265	0.74214076	0.78869234	0.311446	0.38514572	0.34938075	0.22831547	[illegible]
ENSG00000166349	0.21880136	0.12045749	0.11432011	0.1084813	0.22571271	0.11916167	0.03369855	0.04697374	0.02519109	0.04343526	[illegible]
ENSG00000166351	0	0	0	0	0	0	0	0.0518663	0.02617871	0	[illegible]
ENSG00000166352	1.51930682	1.62170294	1.85514618	1.8260652	1.21177873	1.80947579	1.29146894	1.50993005	1.41441267	1.33181106	[illegible]
ENSG00000166359	0	0.00513511	0.02249294	0.0099098	0	0	0	0.00750935	0	0	[illegible]
ENSG00000166363	0	0	0	0	0	0	0	0	0	0	[illegible]
ENSG00000166368	0.0290358	0	0	0.0375322	0.04859036	0.04356095	0.02331792	0.0568816	0.12919545	0.05259677	[illegible]
ENSG00000166377	0.6695216	1.07629483	0.85709036	0.8132869	0.60330319	0.76557089	0.25298342	0.2682061	0.25843907	0.32341381	[illegible]
ENSG00000166387	3.16012554	2.45688646	2.76807308	2.1940235	2.87131556	2.19701812	0.00444296	0.00433525	0.00382926	0.01803906	[illegible]
ENSG00000166391	0	0	0	0	0	0	0	0	0	0	[illegible]
ENSG00000166394	0.02552109	0.02507184	0.0482544	0.0395868	0.05219944	0.02935415	0.00546543	0.00333308	0.00168232	0.001541	[illegible]
ENSG00000166396	0.00729427	0.00488582	0.00356683	0.0047143	0.00203445	0	0.18745107	0.18576473	0.24882953	0.93483211	[illegible]
[illegible]	[illegible]	[illegible]	[illegible]	[illegible]	[illegible]	[illegible]	[illegible]	[illegible]	[illegible]	[illegible]	[illegible]

ENSG0000166971	1.37234921	1.56137739	1.44115996	1.5789852	0.98905428	1.3542623	0.51904196	0.58664851	0.58794293	1.0985728	[illegible]
ENSG0000166974	1.75728819	2.23846902	1.76237422	1.9259809	1.53886843	1.88038117	1.02607182	1.20389225	1.31393139	1.14911415	[illegible]
ENSG0000166979	0.08279132	0.03820229	0.07017263	0.0618323	0.08928667	0.05106315	0.09012767	0.13155371	0.13188979	0.20329531	[illegible]
ENSG0000166984	0	0	0	0	0	0	0	0	0	0	[illegible]
ENSG0000166986	2.91199272	2.54177502	2.45886787	4.0926581	3.73700377	2.90476555	7.66391221	8.41944879	8.89089854	7.80833942	[illegible]
ENSG0000166987	0.5360043	0.69810256	0.49896319	0.5876379	0.54483462	0.59714863	0.83380061	0.88086769	0.6467863	0.71741862	[illegible]
ENSG0000166997	6.3492718	7.44247506	7.23799773	6.2651482	4.75513938	5.58103753	2.26619571	2.77047045	2.43177572	3.1593239	[illegible]
ENSG0000167004	26.764332	18.9350437	23.3565602	16.25117	27.7072881	18.1339961	26.7267643	31.1125112	25.2206749	27.8903152	[illegible]
ENSG0000167005	34.3414382	35.0206544	35.6057145	31.541581	31.3012832	31.0450733	16.5244391	19.1632212	21.8244705	21.3416096	[illegible]
ENSG0000167011	0.32606755	0.30030717	0.45341918	0.5992924	0.11652163	0.42803492	0.02864057	0.04491362	0.03022594	0.26763989	[illegible]
ENSG0000167014	0.03390225	0.04541653	0.03315579	0.0438226	0.0126076	0.02543092	0.0181507	0.03320752	0.02234797	0.03070604	[illegible]
ENSG0000167034	0.05662856	0.04334938	0.03164669	0.010457	0.07671521	0.01820507	0.40929273	0.34865678	0.45594579	0.30773862	[illegible]
ENSG0000167037	0.01426515	0.01592504	0.02015154	0.0327811	0.0141465	0.02972402	0.00891021	0.00310507	0.00548533	0.00430676	[illegible]
ENSG0000167046	0.03334686	0	0	0	0	0	0.30797027	0.14698582	0.0659456	0.09060902	[illegible]
ENSG0000167065	0.53468069	0.55156213	0.52290811	0.4520096	0.56253398	0.45015425	0.93305962	1.06512644	0.97705981	0.38619193	[illegible]
ENSG0000167074	2.13460285	2.05908387	2.08084725	3.4155436	1.68975324	2.91224887	1.00968523	0.96761505	0.69159926	1.36085893	[illegible]
ENSG0000167077	0.00526286	0.00088129	0.00128675	0	0.00293573	0.00493475	0.01056617	0.01675379	0.0058543	0.0131084	[illegible]
ENSG0000167080	0	0	0	0	0	0	0	0.00582889	0.00588409	0.01077962	[illegible]
ENSG0000167081	3.94286033	4.97778285	4.18640755	4.0438099	3.55821012	3.97120212	2.1189791	2.37679561	2.14137157	1.6045475	[illegible]
ENSG0000167083	0	0	0	0	0	0	0	0.00752187	0.00379655	0	[illegible]
[illegible]	[illegible]	[illegible]	[illegible]	[illegible]	[illegible]	[illegible]	[illegible]	[illegible]	[illegible]	[illegible]	[illegible]

ENSG00000167716	0.47245635	0.6583718	0.45100471	0.6571717	0.3689647	0.55317414	1.82945366	1.6368142	1.4954092	1.07071633	1.43368227	1.2177839	1.03363326	0.83450625	[illegible]
ENSG00000167720	3.25031792	2.44666197	2.75996452	2.4875047	3.58877997	2.62390769	0.50133344	0.53567223	0.42341308	0.92522152	0.96195843	0.58819093	1.89520321	1.83884728	[illegible]
ENSG00000167721	7.54063154	6.64003791	6.50542531	7.328073	8.36678626	6.38475948	23.1757534	25.8513803	27.0949476	18.7551373	22.2699113	26.1599541	10.0177536	6.94751999	[illegible]
ENSG00000167723	0.02185154	0.02841205	0.01634208	0.0564913	0.01935941	0.03760377	0.1011615	0.13219969	0.151245	0.15134629	0.11011624	0.14762889	0.01523148	0.02088762	[illegible]
ENSG00000167733	0.39499928	0.39235999	0.38869416	0.4505141	0.34108205	0.39995749	0.36730039	0.37013506	0.29564703	0.49510412	0.42059656	0.25715347	0.11385896	0.14838031	[illegible]
ENSG00000167740	1.42323446	1.44704863	1.34753447	1.3925991	1.42967147	1.2590066	2.38610763	2.44934122	1.96513159	2.60892554	3.20029519	2.39426378	1.69897408	1.97184254	[illegible]
ENSG00000167741	0.03856001	0.03658981	0.06285166	0.0290753	0.02867946	0.05302884	0.03354712	0.05665475	0.02224102	0.03201428	0.04453053	0.02713658	0.01359899	0.03133018	[illegible]
ENSG00000167747	3.56373679	2.42439169	2.67393099	3.2689757	3.08763325	2.69499492	2.29242337	3.06474602	4.03755149	2.77732553	1.86835667	2.82312261	3.66194095	1.96177568	[illegible]
ENSG00000167748	0	0.00333251	0	0.0192933	0.00832592	0.00373207	0	0.00487331	0	0	0	0	0.00421111	0	[illegible]
ENSG00000167749	0	0	0	0	0	0	0	0	0.00263287	0	0	0	0.00450753	0.01153858	[illegible]
ENSG00000167751	0.00125726	0.00168426	0.00122957	0	0.0028053	0.0018862	0.00100967	0.00123149	0.00310788	0.00227745	0.00475175	0.0021235	0	0.00136203	[illegible]
ENSG00000167754	0	0	0	0	0	0	0	0	0	0	0	0	0	0	[illegible]
ENSG00000167755	0	0	0.00658177	0.0043496	0	0	0	0	0	0	0.00423927	0	0	0	[illegible]
ENSG00000167757	0.00295604	0.00198	0	0	0	0.0022174	0.00474785	0.00579094	0.00292288	0.00267736	0.00372409	0.00249638	0	0	[illegible]
ENSG00000167759	0	0	0	0	0	0	0	0	0	0	0	0	0	0	[illegible]
ENSG00000167765	0.28471502	0.43226836	0.51976619	0.5397735	0.25411216	0.62647732	0.15243164	0.13014429	0.11260852	0.30944742	0.16738882	0.12823553	0.25705133	0.18506577	[illegible]
ENSG00000167766	1.05497171	1.64226993	1.13165334	1.4460437	0.91009339	1.6927376	0.59459821	0.55878351	0.44105828	0.20521059	0.54921586	0.44845108	1.7653301	1.72584399	[illegible]
ENSG00000167767	0.00578475	0.00387472	0.00282869	0	0.00161343	0.00216964	1.73977265	1.72252772	1.00812567	0.43224886	0.77614626	0.64240613	2.86187025	2.92035099	[illegible]
ENSG00000167768	0	0	0	0	0	0	0	0	0	0	0	0	0	0	[illegible]
ENSG00000167769	0.02230432	0.04481936	0	0	0.06220912	0	0.01791204	0.06554177	0.05513531	0	0	0.05650794	0.03775719	0.02416314	[illegible]
[illegible]															

ENSG00000168395	2.99443281	3.344975	2.74359218	3.3635281	2.62484887	3.35600143	1.64745551	1.65289072	1.5540339	1.86017146	[illegible]
ENSG00000168397	1.54268331	1.64779097	1.37896695	1.9728279	1.65223986	1.9209605	1.26284201	1.24512217	1.13742402	1.31992914	[illegible]
ENSG00000168398	0.01159204	0.00517636	0	0.0049947	0.00215543	0	1.25830392	1.35875482	0.57978672	1.01492025	[illegible]
ENSG00000168404	0.01425884	0.0095508	0.02556564	0.024575	0.01193083	0.02495713	0.95615169	0.88688234	0.80598705	0.73612981	[illegible]
ENSG00000168405	0.01782478	0.01364493	0.02739363	0.006583	0.00284087	0.00955058	0.27197793	0.31427117	0.25556004	0.36439998	[illegible]
ENSG00000168411	6.24384802	4.34424808	4.58088384	3.3049207	6.2824271	3.34910925	2.4690141	3.10666405	3.59766329	2.74569738	[illegible]
ENSG00000168412	0	0.01674786	0	0	0	0	0.02007982	0	0	0.04529279	[illegible]
ENSG00000168418	0	0	0	0	0	0	0	0	0	0	[illegible]
ENSG00000168421	0	0.00153673	0	0.0029656	0.00255957	0.00344196	0.00368492	0.00449449	0.00113426	0	[illegible]
ENSG00000168427	0.01304672	0.02330373	0.03827836	0.0562147	0.01212956	0.03914667	0.1082674	0.06389682	0.03870111	0.03151122	[illegible]
ENSG00000168434	4.67652768	4.81422325	4.18973248	5.183139	4.30205803	4.46212183	2.98853235	3.00593708	3.04842628	2.26985612	[illegible]
ENSG00000168438	2.95104194	2.62793118	2.68610763	3.03051	3.53561949	2.91063442	4.61210634	5.34054334	5.13166475	3.10903733	[illegible]
ENSG00000168439	11.2796522	8.66864764	9.01843088	8.864881	14.3259059	9.17633413	14.2175633	16.1898213	21.1827085	13.0512883	[illegible]
ENSG00000168447	0.02042119	0.02222746	0.044936	0.0230972	0.04129373	0.02680727	0	0	0	0	[illegible]
ENSG00000168453	0.00346911	0	0.00169636	0.0022421	0.00290271	0.01040905	0.07939965	0.08155243	0.026584	0.14453425	[illegible]
ENSG00000168454	0.07082544	0.05023059	0.04889365	0.0161559	0.05577591	0.06562861	0.02676618	0.04896994	0.02883628	0.01132027	[illegible]
ENSG00000168461	21.5659448	29.1264336	26.8714821	24.715111	21.3332534	22.8236987	4.92413017	5.4408803	4.63506562	8.10345383	[illegible]
ENSG00000168476	2.59551462	1.58942073	1.83189569	2.4049637	2.0592969	1.96271217	1.0219594	1.36578098	1.95386933	1.89054333	[illegible]
ENSG00000168477	0.01082289	0.00836463	0.008142	0.0247513	0.00371522	0.02123305	0.17717476	0.07910058	0.02222617	0.06560161	[illegible]
ENSG00000168481	0	0.00352371	0.00257244	0	0	0	0	0	0.00130042	0.01191186	[illegible]

[illegible]

ENSG00000169169	0.02380673	0.03373219	[illegible]
ENSG00000169174	0.0063382	0.00636814	[illegible]
ENSG00000169180	4.73114075	3.76601979	[illegible]
ENSG00000169181	0	0.00312236	[illegible]
ENSG00000169184	0.77141727	1.53528884	[illegible]
ENSG00000169188	3.2367647	2.26040878	[illegible]
ENSG00000169189	2.3911886	2.29105544	[illegible]
ENSG00000169193	1.25183962	1.68158602	[illegible]
ENSG00000169194	0	0	[illegible]
ENSG00000169202	0	0	[illegible]
ENSG00000169203	0	0.00248024	[illegible]
ENSG00000169208	0	0	[illegible]
ENSG00000169213	0.12797545	0.3378376	[illegible]
ENSG00000169214	0	0	[illegible]
ENSG00000169217	15.4228203	12.9592158	[illegible]
ENSG00000169218	0	0	[illegible]
ENSG00000169220	0.20737493	0.06505584	[illegible]
ENSG00000169221	3.84079956	3.67541549	[illegible]
ENSG00000169223	15.8328769	12.0393972	[illegible]
ENSG00000169224	0	0.00308974	[illegible]
ENSG00000169228	0.22067103	0.37247857	[illegible]
ENSG00000169230	10.2226046	7.51455217	[illegible]
ENSG00000169231	0.90828291	0.89101659	[illegible]
ENSG00000169239	0.62368598	0.76188879	[illegible]
ENSG00000169241	0.64133188	0.51059046	[illegible]
ENSG00000169242	2.48458365	1.48041483	[illegible]
ENSG00000169245	0	0.01840408	[illegible]
ENSG00000169246	0.02886307	0.05385605	[illegible]
ENSG00000169247	0.00124242	0.00291269	[illegible]
ENSG00000169248	0	0.00909734	[illegible]
ENSG00000169249	2.47921075	2.64868038	[illegible]
ENSG00000169251	8.41385169	10.9283335	[illegible]
ENSG00000169252	0.07895576	0.02115433	[illegible]
ENSG00000169253	0	0.06788676	[illegible]
ENSG00000169255	4.34984144	4.08205608	[illegible]
ENSG00000169258	3.74266035	3.63473885	[illegible]
ENSG00000169271	0.25344487	0.25464209	[illegible]
ENSG00000169282	0.13320827	0.05130438	[illegible]
ENSG00000169288	2.90220406	2.99497327	[illegible]
ENSG00000169291	0.00934805	0.01095758	[illegible]
ENSG00000169297	0	0	[illegible]
ENSG00000169299	3.19428433	1.97658283	[illegible]
ENSG00000169302	0.02119758	0.02839695	[illegible]
ENSG00000169306	0	0.00374922	[illegible]
ENSG00000169313	0	0	[illegible]
ENSG00000169314	0	0	[illegible]
ENSG00000169325	0	0	[illegible]
ENSG00000169327	0	0	[illegible]
ENSG00000169330	2.45614553	1.62944707	[illegible]
ENSG00000169340	0.01170713	0	[illegible]
ENSG00000169344	0	0	[illegible]
ENSG00000169347	0.01400786	0.01563781	[illegible]
ENSG00000169359	4.64584841	4.77797515	[illegible]
ENSG00000169371	0.49124189	0.34334776	[illegible]
ENSG00000169372	0.83035452	0.88110347	[illegible]
ENSG00000169375	3.2183763	3.36308028	[illegible]
ENSG00000169379	2.15082905	1.93008367	[illegible]
ENSG00000169385	0	0	[illegible]
ENSG00000169393	0	0	[illegible]
ENSG00000169397	0	0.02998332	[illegible]
ENSG00000169398	1.56269316	1.71664581	[illegible]
ENSG00000169402	0	0.00093893	[illegible]
ENSG00000169403	0.04099272	0.01267273	[illegible]
ENSG00000169410	12.1052646	8.29193552	[illegible]
ENSG00000169413	0.75941894	0.36624301	[illegible]
ENSG00000169418	0.99477607	0.59228158	[illegible]
ENSG00000169427	0.00651106	0.01744484	[illegible]
ENSG00000169429	0.41842871	0.13654188	[illegible]
ENSG00000169432	0.12919042	0.08817684	[illegible]
ENSG00000169435	0.02126253	0.00854519	[illegible]
ENSG00000169436	0.04998462	0.03463499	[illegible]
ENSG00000169439	0.05721056	0.07213279	[illegible]
ENSG00000169442	0	0	[illegible]
ENSG00000169446	6.10039198	6.26719396	[illegible]
ENSG00000169469	0	0	[illegible]
ENSG00000169474	0	0	[illegible]
ENSG00000169484	0	0	[illegible]
ENSG00000169488	0	0	[illegible]
ENSG00000169490	1.97770567	1.55314295	[illegible]
ENSG00000169495	0.19999361	0.2060906	[illegible]
ENSG00000169499	4.95957899	4.01442488	[illegible]
ENSG00000169504	27.0052123	24.2350266	[illegible]
ENSG00000169507	0.01286514	0.00172345	[illegible]
ENSG00000169508	0	0	[illegible]
ENSG00000169509	0	0	[illegible]
ENSG00000169515	0.02006209	0.04031371	[illegible]
ENSG00000169519	0.528502	0.62569574	[illegible]
ENSG00000169548	0.12003627	0.11055302	[illegible]
ENSG00000169550	0	0	[illegible]
ENSG00000169551	0	0	[illegible]
ENSG00000169554	3.25784912	4.58977788	[illegible]
ENSG00000169562	0.04020103	0.00769351	[illegible]
ENSG00000169564	61.3250083	55.7977926	[illegible]
ENSG00000169567	23.3841044	16.7719025	[illegible]
ENSG00000169570	0.55001917	0.53060533	[illegible]
ENSG00000169575	0	0	[illegible]
ENSG00000169583	0	0	[illegible]
ENSG00000169592	1.76876867	1.55643535	[illegible]
ENSG00000169594	0.02485904	0.00832549	[illegible]
ENSG00000169598	0.24006764	0.29774711	[illegible]
ENSG00000169599	4.30785511	3.34235833	[illegible]
ENSG00000169604	3.58079115	5.45840691	[illegible]
ENSG00000169605	0	0	[illegible]
ENSG00000169607	8.97574765	6.60609395	[illegible]
ENSG00000169609	0.75045111	0.82960002	[illegible]
ENSG00000169612	8.26298175	7.75130018	[illegible]
ENSG00000169618	0	0	[illegible]
ENSG00000169621	0.59545708	0.68876449	[illegible]
ENSG00000169627	0.02753502	0.01844339	[illegible]
ENSG00000169629	0.02884416	0.02173531	[illegible]
ENSG00000169635	0.51122314	0.58434362	[illegible]
ENSG00000169641	1.35111393	0.9319402	[illegible]
ENSG00000169660	0.29244078	0.54396813	[illegible]
ENSG00000169662	0	0	[illegible]
ENSG00000169668	0.04488822	0.08017824	[illegible]
ENSG00000169676	0	0	[illegible]
ENSG00000169679	13.4612187	9.4584165	[illegible]
ENSG00000169682	0.16086853	0.12591279	[illegible]
ENSG00000169683	3.75686817	3.24178066	[illegible]
ENSG00000169684	1.62357183	0.86225264	[illegible]
ENSG00000169688	0	0	[illegible]
ENSG00000169689	3.07163433	2.03990867	[illegible]
ENSG00000169692	0.92434983	0.47741237	[illegible]
ENSG00000169696	0.43885543	0.56610593	[illegible]
ENSG00000169704	0	0	[illegible]
ENSG00000169710	17.6003116	16.5668904	[illegible]
ENSG00000169714	43.2945803	41.2221569	[illegible]
ENSG00000169715	0	0	[illegible]
ENSG00000169717	0	0	[illegible]
ENSG00000169718	1.08947886	1.25721497	[illegible]
ENSG00000169727	2.93534704	2.96628286	[illegible]
ENSG00000169733	1.23017199	1.21031362	[illegible]
ENSG00000169738	4.33706275	3.32623225	[illegible]
ENSG00000169740	8.70856977	9.90634384	[illegible]
ENSG00000169744	0.73713852	0.66191438	[illegible]
ENSG00000169750	8.67184648	6.16531479	[illegible]
ENSG00000169752	0.00382981	0.00513053	[illegible]
ENSG00000169756	3.37282489	2.90585286	[illegible]
ENSG00000169758	0.03382893	0.03707861	[illegible]
ENSG00000169760	7.13431704	7.75118023	[illegible]
ENSG00000169762	3.20047592	2.38393598	[illegible]
ENSG00000169763	0	0	[illegible]
ENSG00000169764	2.65571057	2.36239905	[illegible]
ENSG00000169777	0	0	[illegible]
ENSG00000169783	4.85666768	4.0538789	[illegible]
ENSG00000169789	0	0	[illegible]
ENSG00000169800	0	0	[illegible]
ENSG00000169807	0	0	[illegible]
ENSG00000169811	0	0	[illegible]
ENSG00000169813	14.9651805	11.09915	[illegible]
ENSG00000169814	0.68725905	0.61213272	[illegible]
ENSG00000169826	3.68921434	4.1369526	[illegible]
ENSG00000169836	0.01241994	0.01663814	[illegible]
ENSG00000169840	0	0	[illegible]
ENSG00000169849	0	0	[illegible]
ENSG00000169851	6.39780457	8.9234056	[illegible]
ENSG00000169855	12.9632706	13.6958985	[illegible]
ENSG00000169856	0	0	[illegible]
ENSG00000169857	3.35783796	2.36896395	[illegible]
ENSG00000169860	32.4580251	33.9458293	[illegible]
ENSG00000169861	0	0	[illegible]
ENSG00000169862	2.59152496	4.01389534	[illegible]
ENSG00000169871	3.73477671	3.40675454	[illegible]
ENSG00000169876	0	0	[illegible]
ENSG00000169877	0.05714493	0	[illegible]
ENSG00000169884	0.04724509	0.05178356	[illegible]
ENSG00000169885	0.01491427	0.01498472	[illegible]
ENSG00000169891	0.00876126	0.01858336	[illegible]
ENSG00000169894	0.00268604	0	[illegible]
ENSG00000169895	8.12804072	8.09269095	[illegible]
ENSG00000169896	0.00286665	0.00384025	[illegible]
ENSG00000169900	0	0	[illegible]
ENSG00000169902	13.9223706	11.5870235	[illegible]
ENSG00000169903	0	0	[illegible]
ENSG00000169905	9.06628811	8.93498325	[illegible]
ENSG00000169906	0	0	[illegible]
ENSG00000169908	5.29797215	3.60128727	[illegible]
ENSG00000169914	2.23723478	2.30986353	[illegible]
ENSG00000169918	0.07860912	0.14343577	[illegible]
ENSG00000169919	3.99898688	3.6867694	[illegible]
ENSG00000169925	3.23023293	4.44887643	[illegible]

ENSG00000169926	6.91991472	6.63936533	6.21749554	9.234789	5.59446595	7.66057809	1.69761325	[illegible]
ENSG00000169933	0.00380741	0.00255026	0.00372358	0	0	0	0.02751873	[illegible]
ENSG00000169946	1.46498817	1.773037	1.55152285	1.1373498	1.1658963	1.14336993	0.69114627	[illegible]
ENSG00000169951	1.50570063	1.49822017	1.45834352	1.3767995	1.15319089	1.40547386	0.74650975	[illegible]
ENSG00000169953	0	0	0	0	0	0	0	[illegible]
ENSG00000169955	0.86841072	1.05402859	0.80712675	0.9354352	0.73349816	0.79463571	1.0090025	[illegible]
ENSG00000169957	14.4663759	13.0198969	13.7164741	15.694034	14.612952	13.6892647	8.98186207	[illegible]
ENSG00000169962	0.12204679	0.20751637	0.07345204	0.266978	0.10997586	0.27465099	0.28649867	[illegible]
ENSG00000169964	2.06106708	2.89280127	2.50806804	4.2097951	1.57097382	3.26914486	3.10822143	[illegible]
ENSG00000169967	5.89027887	6.60171252	5.6670487	7.1977487	6.44652338	7.19725266	5.01321806	[illegible]
ENSG00000169972	2.39899584	1.38169247	1.72758717	2.2097261	2.03433494	1.50888336	1.49183575	[illegible]
ENSG00000169976	73.6867185	64.1781375	68.0013155	86.826104	68.6449337	64.9841364	54.2943718	[illegible]
ENSG00000169981	2.19486932	1.88786989	1.93870292	1.3825355	2.05806536	1.41906246	2.55163745	[illegible]
ENSG00000169989	1.27771622	1.10035874	1.22408181	1.0785942	1.12001577	1.03668726	0.19893809	[illegible]
ENSG00000169991	1.61878822	1.13179756	1.38321245	1.2134185	1.31958222	1.26436726	1.41057577	[illegible]
ENSG00000169992	3.53804556	4.8813887	3.88351142	3.778157	3.36619052	4.57792543	3.94438346	[illegible]
ENSG00000169994	0.00090755	0.00182367	0	0.0011731	0.00050625	0.00068077	0.07288287	[illegible]
ENSG00000170004	0.32307876	0.22875237	0.21121588	0.3325049	0.36631716	0.33125477	4.71251817	[illegible]
ENSG00000170006	0.92627653	0.71105999	0.84990035	1.008978	1.07983819	0.90170858	2.12504253	[illegible]
ENSG00000170011	0.00224848	0.00075303	0.00219897	0.0029064	0.00125425	0.00168664	0.0072228	[illegible]
[illegible]	[illegible]	[illegible]	[illegible]	[illegible]	[illegible]	[illegible]	[illegible]	[illegible]

ENSG00000170689	0	0	0	0	0	0	0	0	0	0	0
ENSG00000170703	0.0035775	0.00119813	0.0052481	0	0.0009978	0	0	0.0017521	0.00088434	0	
ENSG00000170727	0.57905441	0.41084411	0.3985108	0.3714755	0.60773523	0.38610055	1.6465295	1.8276071	2.36021552	1.18878216	
ENSG00000170734	3.7487214	2.81235602	2.97048125	3.6524217	4.45908007	3.51190905	2.39405464	2.67695626	2.99510801	2.23856186	
ENSG00000170743	0.06324517	0.11014281	0.08350107	0.1021897	0.03704348	0.09725564	0	0	0	0	
ENSG00000170745	0	0.01343372	0.01471069	0	0	0.01504439	0.03221269	0.00491121	0.0421406	0.04995384	
ENSG00000170748	0.02910135	0.01949254	0	0	0	0.02182964	0	0	0	0	
ENSG00000170759	65.6483022	57.0416313	59.008054	52.947215	66.216289	56.1394784	39.4601375	43.0701995	48.0899356	42.4924261	
ENSG00000170775	17.6778149	10.3402259	13.8141745	19.472	13.9316506	14.1808857	1.13093076	1.36894292	1.17620176	2.40602792	
ENSG00000170776	1.40005865	1.91732499	1.5902693	1.3067541	1.19553984	1.25149335	1.42522215	1.50714227	1.49801948	2.00671896	
ENSG00000170777	0	0	0	0	0	0	0	0	0	0	
ENSG00000170779	8.50120081	4.37731298	5.80735973	4.619821	6.91785669	4.31698843	3.39437293	4.55333749	6.06432613	2.99914591	
ENSG00000170782	0	0	0	0	0	0	0	0	0	0	
ENSG00000170786	0	0	0	0	0	0	0	0	0	0	
ENSG00000170788	0.01568357	0.06303063	0.04601475	0.0405457	0.01749726	0.07058782	0.04408279	0.08449201	0.0348922	0.0426149	
ENSG00000170790	0	0	0	0	0	0	0	0	0	0	
ENSG00000170791	2.23054019	2.67111177	2.48601143	2.8719886	2.53216028	2.98353968	2.7246621	2.86311822	2.50486478	2.19832286	
ENSG00000170801	0	0	0.00690625	0	0.00787837	0.01059436	3.11910407	4.57906834	6.5356366	7.841459	
ENSG00000170802	13.6493456	13.9811182	14.144275	15.33035	13.4781548	16.7708985	4.40090824	5.01921784	5.3055042	5.31335205	
ENSG00000170803	0	0	0	0	0	0	0	0	0	0	

ENSG00000171459	0	0	0	0	0	[illegible]
ENSG00000171462	0.10904214	0.08608068	0.06474647	0.0402713	0.08906668	[illegible]
ENSG00000171466	2.7650621	2.61703297	2.61950741	2.6433193	2.77434574	[illegible]
ENSG00000171467	3.14820645	3.87551715	3.23663836	2.8871083	3.35560757	[illegible]
ENSG00000171469	1.33053781	1.29409347	1.42474549	1.5364445	1.19391449	[illegible]
ENSG00000171471	0.10147903	0.05437781	0.03969787	0.0787041	0.04528573	[illegible]
ENSG00000171475	2.10243794	2.46149522	2.0614221	1.7920081	1.91828149	[illegible]
ENSG00000171476	0.0483952	0.04322117	0.26533284	0.0492869	0.13743355	[illegible]
ENSG00000171478	0	0	0	0	0	[illegible]
ENSG00000171481	0	0	0	0	0	[illegible]
ENSG00000171483	0	0	0	0	0	[illegible]
ENSG00000171484	0	0	0	0	0	[illegible]
ENSG00000171487	0	0.00473836	0	0	0.0039461	[illegible]
ENSG00000171488	0.89113304	0.70354487	0.63542526	0.5610087	0.82084807	[illegible]
ENSG00000171489	0	0.01656791	0	0.0319729	0	[illegible]
ENSG00000171490	7.77544975	7.38673771	7.76308003	10.131197	7.11716192	[illegible]
ENSG00000171492	6.27676605	6.83859065	5.59565914	11.314034	8.31009044	[illegible]
ENSG00000171495	0	0	0	0	0	[illegible]
ENSG00000171496	0.03037676	0	0	0	0	[illegible]
ENSG00000171497	8.4444733	6.1343292	6.79363496	6.1334534	8.63032173	[illegible]
ENSG00000171501	0	0	0	0	0	[illegible]
ENSG00000171502	0.03389037	0.03877969	0.03452518	0.0492832	0.02441866	[illegible]
ENSG00000171503	1.81709693	1.6712697	1.59142223	1.9281276	2.27874347	[illegible]
ENSG00000171505	0	0	0	0	0	[illegible]
ENSG00000171509	0.09905638	0.01658738	0.03846521	0.0225956	0.37135118	[illegible]
ENSG00000171517	0	0	0.00530999	0	0.00605742	[illegible]
ENSG00000171522	0.01497201	0.00668566	0.00976157	0.0193531	0.02227121	[illegible]
ENSG00000171530	19.05478	18.4825822	21.8157406	18.866366	18.2019319	[illegible]
ENSG00000171532	0	0.0078189	0.0038054	0	0.00217052	[illegible]
ENSG00000171533	3.46261747	6.2504352	4.41294406	5.7388001	3.07865049	[illegible]
ENSG00000171540	0	0.00466969	0	0	0	[illegible]
ENSG00000171551	0	0	0	0	0	[illegible]
ENSG00000171552	3.47558891	2.99968173	2.99217747	2.8047752	3.28124886	[illegible]
ENSG00000171557	0.00223771	0.00299771	0.00218844	0.0028925	0	[illegible]
ENSG00000171560	0	0	0	0	0	[illegible]
ENSG00000171561	0	0	0	0	0	[illegible]
ENSG00000171564	0	0	0	0	0	[illegible]
ENSG00000171566	3.93235309	4.30258198	4.30216233	4.0333024	3.90985342	[illegible]
ENSG00000171570	0	0.01179672	0	0	0.00491214	[illegible]
ENSG00000171574	1.25156733	0.79967965	0.97626478	0.9920759	1.1878359	[illegible]
ENSG00000171587	6.01964334	6.65497943	4.96589932	7.4580016	6.24584723	[illegible]
ENSG00000171595	0.00228094	0	0	0	0	[illegible]
ENSG00000171596	0.03909004	0.03927469	0.02867202	0.1010569	0.03270789	[illegible]
ENSG00000171603	13.3749464	14.8119352	12.226432	14.116516	10.6833088	[illegible]
ENSG00000171604	7.22239169	4.99631145	6.11299038	4.1471335	5.51338674	[illegible]
ENSG00000171606	0.82008645	0.75634748	0.77748927	0.8890329	0.84493618	[illegible]
ENSG00000171608	0.37336113	0.39533355	0.44443867	0.4704257	0.21983928	[illegible]
ENSG00000171611	0	0	0	0	0	[illegible]
ENSG00000171612	2.3415483	1.80366709	2.24092092	2.1187072	2.82225044	[illegible]
ENSG00000171617	6.93849132	5.68075659	5.18548712	6.4420437	3.07382234	[illegible]
ENSG00000171621	1.40765457	0.50016008	1.07073625	0.7891273	1.62672753	[illegible]
ENSG00000171631	0	0	0.00192442	0	0	[illegible]
ENSG00000171634	2.67020245	3.70599813	2.97476291	2.5828411	2.10980804	[illegible]
ENSG00000171643	0	0	0	0	0	[illegible]
ENSG00000171649	3.31202852	3.17687287	2.94357283	2.7265061	3.13352792	[illegible]
ENSG00000171657	0.0585691	0.07285667	0.09819348	0.0432614	0.07934408	[illegible]
ENSG00000171658	0.01089579	0	0.00532794	0.0281682	0.01519476	[illegible]
ENSG00000171659	0	0	0	0	0	[illegible]
ENSG00000171671	0.05745052	0.17957924	0.09364262	0.0247538	0.04272949	[illegible]
ENSG00000171680	0.07485556	0.1259408	0.07320739	0.1219018	0.03419391	[illegible]
ENSG00000171681	2.90370647	3.8048579	3.18331824	3.275146	2.65772703	[illegible]
ENSG00000171695	0.33634474	0.15902755	0.21284295	0.1790208	0.29798511	[illegible]
ENSG00000171700	2.1015986	1.17657175	1.51081776	1.267053	2.50210823	[illegible]
ENSG00000171703	2.07533272	2.27325382	2.08939006	2.6443961	1.8579805	[illegible]
ENSG00000171711	0	0	0	0	0	[illegible]
ENSG00000171714	2.09751164	1.55298843	2.06828184	2.095079	1.44078893	[illegible]
ENSG00000171720	3.91810595	3.56287459	3.59625378	3.9938608	3.97768761	[illegible]
ENSG00000171722	0	0	0	0	0	[illegible]
ENSG00000171723	0.83090173	1.09033016	0.85782569	0.8877069	0.97553871	[illegible]
ENSG00000171724	0.0145179	0.01458648	0.03549562	0.0187661	0.02834438	[illegible]
ENSG00000171729	0.38922513	0.50022246	0.33113907	0.2863279	0.27360407	[illegible]
ENSG00000171735	0.78626823	0.65386235	0.85649892	0.6129333	0.85222802	[illegible]
ENSG00000171747	0.02302453	0.02467551	0	0.0357143	0.02054972	[illegible]
ENSG00000171757	0.0057277	0.00639417	0.01493755	0.0049358	0.00852008	[illegible]
ENSG00000171759	0.02344291	0.01998491	0.0208425	0.0330575	0.01307695	[illegible]
ENSG00000171763	1.44007393	1.72621832	1.49826217	1.4698082	1.09765876	[illegible]
ENSG00000171766	4.03985478	8.26052501	5.30258688	7.0647068	1.94076424	[illegible]
ENSG00000171772	1.02865412	1.28653102	1.02270244	1.2082762	0.9642784	[illegible]
ENSG00000171773	0	0	0	0	0	[illegible]
ENSG00000171777	0.00439397	0.00353178	0.00687555	0.0056797	0.00196084	[illegible]
ENSG00000171786	0	0	0.03689439	0.0325093	0.02805842	[illegible]
ENSG00000171790	0.04334867	0.04210166	0.02543654	0.0420249	0.02297177	[illegible]
ENSG00000171791	4.29922871	6.84371136	5.48103958	3.0842454	3.06302254	[illegible]
ENSG00000171792	10.1165913	7.6184839	8.49188284	6.0831296	9.11601928	[illegible]
ENSG00000171793	4.52175186	2.57771409	3.05184535	2.2590861	5.25252317	[illegible]
ENSG00000171794	0	0	0	0	0	[illegible]
ENSG00000171798	0	0	0	0	0	[illegible]
ENSG00000171804	0	0.00234678	0	0	0	[illegible]
ENSG00000171806	4.32898796	3.76382664	3.98463292	3.8072857	3.87788735	[illegible]
ENSG00000171811	0	0	0	0	0	[illegible]
ENSG00000171812	0	0.01160853	0.01412444	0.0149348	0.0080563	[illegible]
ENSG00000171813	1.44302522	0.94906685	1.06642176	1.1985048	1.00527716	[illegible]
ENSG00000171815	0.2035027	0.25558	0.17414424	0.1150848	0.0425693	[illegible]
ENSG00000171817	0.06246728	0.09499168	0.06439414	0.1112989	0.06404052	[illegible]
ENSG00000171819	0	0	0.01107912	0	0	[illegible]
ENSG00000171823	7.87362784	7.16316568	7.00737335	8.4496406	7.89679082	[illegible]
ENSG00000171824	4.78991265	5.09471388	4.60867905	3.7830121	4.68368484	[illegible]
ENSG00000171827	1.00082796	0.92407986	0.84025509	1.0548525	0.80564552	[illegible]
ENSG00000171840	0	0.00542003	0.00791366	0	0.00451379	[illegible]
ENSG00000171843	1.99891528	2.07741463	2.18989161	2.7292132	1.50762956	[illegible]
ENSG00000171847	0.04733577	0.03963281	0.04050684	0.061187	0.03960735	[illegible]
ENSG00000171848	25.4660934	13.353962	17.543625	7.3010432	23.5161586	[illegible]
ENSG00000171853	1.95958215	2.3686525	1.88653633	2.1415611	1.79800592	[illegible]
ENSG00000171855	0	0	0.03752394	0	0	[illegible]
ENSG00000171858	34.2052712	31.8660046	35.5299891	59.857146	30.6617565	[illegible]
ENSG00000171860	0.06069631	0	0.02374396	0.0156914	0.13543077	[illegible]
ENSG00000171861	3.1498379	2.20444669	2.51965783	2.8253648	3.25447689	[illegible]
ENSG00000171862	9.29821451	10.2048778	10.0574305	10.545776	9.51227076	[illegible]
ENSG00000171863	24.2797186	24.8627742	25.2226766	37.076101	20.6633734	[illegible]
ENSG00000171864	0.02429377	0.0596653	0.0316785	0.0104675	0.02710317	[illegible]
ENSG00000171865	3.94462659	3.14602974	3.53479754	3.7154706	4.5474865	[illegible]
ENSG00000171867	11.1161345	8.43433378	9.36363675	10.826801	8.96168745	[illegible]
ENSG00000171872	0	0.02256978	0	0.0217777	0	[illegible]
ENSG00000171873	0	0	0	0	0	[illegible]
ENSG00000171877	1.46976938	1.82899047	1.55278994	2.2066906	1.13623963	[illegible]
ENSG00000171885	0.04992505	0.08569151	0.06713548	0.0605006	0.01392463	[illegible]
ENSG00000171889	0	0	0	0	0	[illegible]
ENSG00000171903	0	0	0.00546181	0.0036095	0	[illegible]
ENSG00000171914	1.24430872	1.24566556	0.98005259	0.8036939	1.32353316	[illegible]
ENSG00000171916	0	0	0	0.0021465	0	[illegible]
ENSG00000171928	0.67356742	0.68138447	0.73769502	0.7901579	0.58547008	[illegible]
ENSG00000171931	0.01102814	0.00492455	0.00179755	0.0047517	0.00410115	[illegible]
ENSG00000171936	0	0	0	0	0	[illegible]
ENSG00000171940	7.52233832	8.94364171	8.67607136	8.2908556	7.43628065	[illegible]
ENSG00000171942	0	0	0	0	0	[illegible]
ENSG00000171943	4.5243641	3.17804278	3.24812876	3.3950625	3.98890619	[illegible]
ENSG00000171944	0	0	0	0	0	[illegible]
ENSG00000171951	6.71706359	1.36780204	5.0819692	1.9319603	8.56023624	[illegible]
ENSG00000171953	1.54958352	1.40174809	1.36704304	1.23898	1.20004754	[illegible]
ENSG00000171954	0	0	0	0	0	[illegible]
ENSG00000171956	5.34514346	7.01609693	6.53709254	4.5621403	4.36166962	[illegible]
ENSG00000171960	5.04129206	3.95957144	4.08606482	4.4454745	4.55335551	[illegible]
ENSG00000171962	0.03012125	0.09629308	0.05623808	0.0495539	0.02443967	[illegible]
ENSG00000171970	1.38046411	0.94998981	1.04183415	1.4096033	1.35022864	[illegible]
ENSG00000171984	1.78741842	1.58879177	1.83601598	3.0515338	1.16010132	[illegible]
ENSG00000171987	0	0	0	0	0	[illegible]
ENSG00000171988	3.59648742	4.15368366	3.65511721	2.8175327	2.82355252	[illegible]
ENSG00000171989	0.01936883	0	0	0	0	[illegible]
ENSG00000171992	0.34713766	0.06098841	0.18087816	0.062526	0.7174232	[illegible]
ENSG00000171999	0	0	0	0	0	[illegible]
ENSG00000172000	0.00838879	0.00561895	0.00820409	0.021687	0.00467945	[illegible]
ENSG00000172005	0	0	0	0	0	[illegible]
ENSG00000172006	0.55167744	0.67884152	0.57893455	0.9134186	0.44604637	[illegible]
ENSG00000172007	2.98016866	3.5300588	3.40542324	3.9089855	2.50585156	[illegible]
ENSG00000172009	2.99239303	2.34860112	2.44328372	2.1982035	2.97426763	[illegible]
ENSG00000172014	0.12810375	0.16381131	0.15375662	0.0526874	0.07470714	[illegible]
ENSG00000172016	0	0	0	0	0	[illegible]
ENSG00000172020	41.0319042	3.02303988	27.4221418	2.4936111	48.6849213	[illegible]
ENSG00000172023	0	0	0	0	0	[illegible]
ENSG00000172031	0.44928707	0.2743861	0.3360077	0.1537295	0.56021364	[illegible]
ENSG00000172037	14.2504376	15.1499729	12.0828043	16.900675	11.3458233	[illegible]
ENSG00000172046	3.18310074	3.42956623	2.8304646	3.706134	3.19448026	[illegible]
ENSG00000172053	8.60405368	9.47796755	8.27088798	14.199096	7.31369665	[illegible]
ENSG00000172057	4.64813787	4.74030341	4.84272133	6.6436603	4.11304334	[illegible]
ENSG00000172058	0.02780138	0.06384618	0.03884178	0.0359365	0.01329274	[illegible]
ENSG00000172059	0.45394	0.2617391	0.31121929	0.3417787	0.40462573	[illegible]
ENSG00000172061	0	0	0.00210893	0	0	[illegible]
ENSG00000172062	0.27206733	0.15284227	0.19168986	0.1852931	0.2170402	[illegible]
ENSG00000172071	5.68396385	4.84901441	5.42158859	6.6893217	5.16455486	[illegible]
ENSG00000172073	0.04054055	0	0	0.0524034	0	[illegible]
ENSG00000172081	2.68326706	2.70233373	2.55326302	2.0179094	2.41869993	[illegible]
ENSG00000172086	29.5638457	26.4646844	29.7759821	21.935441	28.4188496	[illegible]
ENSG00000172113	1.50347848	1.47469705	1.39263098	1.6172841	1.39103923	[illegible]
ENSG00000172115	72.7636094	44.6097572	63.4361323	49.20267	104.824805	[illegible]
ENSG00000172116	0	0	0	0	0	[illegible]
ENSG00000172123	0.4022655	0.6478117	0.56918666	0.4056544	0.1893807	[illegible]
ENSG00000172137	0.00726059	0	0.00710072	0.0093852	0	[illegible]
ENSG00000172139	0.02692093	0.03967055	0.03685946	0.0382783	0.01501707	[illegible]
ENSG00000172146	0	0	0	0	0	[illegible]
ENSG00000172148	0	0	0	0	0	[illegible]
ENSG00000172150	0	0	0	0	0	[illegible]
ENSG00000172154	0	0	0	0	0	[illegible]
ENSG00000172155	0.07495288	0.10040926	0.07330257	0	0	[illegible]
ENSG00000172156	0	0	0	0	0	[illegible]
ENSG00000172159	0.36222959	0.27174237	0.46761532	0.0561869	0.2069088	[illegible]
ENSG00000172164	4.25710986	4.76426055	4.47031208	4.1813335	2.23777088	[illegible]
ENSG00000172167	0.94298089	0.86472272	0.82615396	0.6892662	0.81563607	[illegible]
ENSG00000172171	0.76903934	1.00012538	0.78140952	0.8326976	0.68526418	[illegible]
ENSG00000172172	3.70812393	2.48676352	3.13534728	2.9269136	3.74425671	[illegible]
ENSG00000172175	2.3247955	1.82386444	1.91146466	2.173727	3.34688385	[illegible]
ENSG00000172179	0	0	0	0	0	[illegible]

ENSG00000172183	0.06074797	0.03937738	0.06515982	0.0835899	0.04809699	0.0984869	0.79472614	0.68524566	0.35167996	0.55375791	0.78012911	0.33925263	1.01840199	1.27375478	1.49464293	0.58980239	1.04358066	[illegible]
ENSG00000172186	0	0	0.05306415	0.0701359	0.03026673	0.08140178	0	0.05314695	0	0.04914344	0.06835647	0.09164307	0	0	0.04718871	0.10468445	0	[illegible]
ENSG00000172188	0	0	0	0	0	0	0	0	0	0	0	0	0	0	0	0	0	[illegible]
ENSG00000172197	8.94668079	6.14153635	8.89817277	6.1818202	7.53134306	6.54436642	0.4354457	0.49924511	0.62460407	0.47145959	0.39620074	0.77386279	0.59662591	0.24671284	0.23578477	0.53876212	0.39153617	[illegible]
ENSG00000172199	0	0	0	0	0	0	0	0	0	0	0	0	0	0	0	0	0	[illegible]
ENSG00000172201	43.5871481	62.7930545	48.3290372	31.814273	34.8523928	37.3784205	10.7771771	9.4546243	7.75632011	4.9938881	8.85175503	9.30102838	0.27931303	0.16385374	0.21524816	0.51730375	0.37232685	[illegible]
ENSG00000172208	0	0	0	0	0	0	0	0	0	0	0	0	0	0	0	0	0	[illegible]
ENSG00000172209	1.49433825	2.32314953	1.6178036	0.8266959	2.42525349	1.54071628	0	0.02409409	0.01824168	0.03898845	0	0.04154626	0	0.00666204	0	0.00593232	0	[illegible]
ENSG00000172215	0	0	0.0038472	0	0	0	0	0	0	0	0	0	0	0	0	0	0	[illegible]
ENSG00000172216	1.49130532	0.56408467	0.68633874	2.8575114	1.98672962	1.30291595	22.3463624	19.573989	19.2996106	19.783915	24.5125495	15.3943888	45.9312562	39.9905934	40.4506144	49.6241908	43.3648513	[illegible]
ENSG00000172232	0.01225465	0.05745853	0.02396966	0.0475217	0.01367181	0.07354017	0.04920702	0.09602825	0.10299612	0.3107809	0.15438686	0.04139618	0.05186231	0.05310379	0.01065783	0.01182178	0.05530635	[illegible]
ENSG00000172236	0	0	0	0	0	0	0	0	0	0	0	0	0	0	0	0	0	[illegible]
ENSG00000172238	0	0	0	0	0	0	0	0	0	0	0	0	0	0	0	0	0	[illegible]
ENSG00000172239	6.94617231	6.32962093	6.75413402	6.2043731	6.85463551	6.05954515	4.66730819	4.87036831	5.24136828	4.30660249	4.51712337	5.12750735	5.54939128	5.12137243	5.57603075	6.69840084	6.03669108	[illegible]
ENSG00000172243	0.02240015	0.02455196	0.0099577	0.0184258	0.00908748	0.0290232	0.03597799	0.01396253	0.01812181	0.02951029	0.04361305	0.02235635	0.01034166	0.01323652	0.00531309	0.01178667	0.00919035	[illegible]
ENSG00000172244	2.20538993	1.33737261	1.40822267	1.2731389	1.91996906	1.41339776	1.10908259	1.41041989	1.55080316	1.34780856	1.18014539	1.96642185	1.59028898	1.26997964	1.71318294	2.46829974	1.96485566	[illegible]
ENSG00000172247	0.38193858	0.31486586	0.34479606	0.6266195	0.22124783	0.40771385	0.02359425	0.01438892	0.00726258	0.01330502	0.01850672	0.0248113	0	0	0	0	0	[illegible]
ENSG00000172250	0	0	0.00726605	0	0	0.00557315	0	0	0	0.01345837	0	0	0.00628851	0	0	0	0	[illegible]
ENSG00000172260	0.95935773	1.74806196	1.52405917	0.5807213	0.39766443	0.75123196	0.40588825	0.49811662	0.80592131	1.25038563	0.42252578	0.58359082	0.20201223	0.19772236	0.14516335	0.21519062	0.13141536	[illegible]
ENSG00000172262	1.3978104	1.44635305	1.33503672	1.1841393	1.3228322	1.32187855	1.12617038	1.26308089	1.31687283	0.92576424	1.32891703	1.45865107	1.2786106	1.2319752	1.16366883	1.36911344	1.25931252	[illegible]

ENSG0000172954	1.77851784	1.67076477	1.62539117	1.3341754	2.24807824	1.41736229	1.29459027	1.60210869	1.62962442	1.83198944	[illegible]
ENSG0000172955	0.002579	0.00172745	0	0.0033337	0.00143862	0.00193457	0	0.0101046	0.00255007	0.00233586	[illegible]
ENSG0000172965	0.83660671	0.22492908	0.51255293	0.2107624	1.15207509	0.31305172	1.27980335	1.25486204	1.54504395	2.03585984	[illegible]
ENSG0000172967	0.01907085	0.01277396	0.01865095	0	0	0	0.18378385	0.13076036	0.10371311	0	[illegible]
ENSG0000172969	0.00776059	0.0155945	0.01517944	0.0401259	0.00432902	0.01164283	0	0	0	0	[illegible]
ENSG0000172971	0.37530992	0.25138854	0.18352318	0.4244904	0.13084742	0.28152931	0.30140195	0.18380953	0.06958125	0.33992671	[illegible]
ENSG0000172974	0.01505359	0.01008313	0.02944428	0.0194585	0.02519164	0.04516829	0.01208916	0	0.01488473	0.01363438	[illegible]
ENSG0000172977	2.75538759	2.77806626	2.5307085	2.5313894	2.45338796	2.34688813	2.33442751	2.25204726	2.71823911	1.80148271	[illegible]
ENSG0000172985	0.1310152	0.10903026	0.11259954	0.6979374	0.59352311	0.60753525	0.9756301	0.87109143	0.70072378	1.89861504	[illegible]
ENSG0000172986	0.65060034	0.32085079	0.39854616	0.2772451	0.49851472	0.33250497	8.87068969	11.3517777	11.3709	7.08411261	[illegible]
ENSG0000172987	0	0	0	0	0	0	0	0	0	0	[illegible]
ENSG0000172992	6.69964325	5.04678768	6.13207206	6.5378609	7.08702788	5.82050625	2.09861292	2.16648114	2.00243024	3.99929342	[illegible]
ENSG0000172995	0.04884972	0.04929861	0.04968511	0.0479894	0.02107288	0.02833755	0.00523066	0.00701781	0.00676225	0.0123884	[illegible]
ENSG0000173011	1.98607838	2.25379191	1.91858936	2.2217994	2.23946962	1.93629322	1.63886779	1.61817507	1.55842848	1.46877453	[illegible]
ENSG0000173013	0.37476442	0.46188259	0.29321029	0.2906561	0.4933617	0.2586305	0.38523375	0.45518507	0.30385969	0.21723688	[illegible]
ENSG0000173020	3.23363991	2.4505777	2.48856019	2.9018276	3.02583946	2.68343668	1.91704988	2.03806553	1.97533692	1.55792749	[illegible]
ENSG0000173039	1.52466092	1.45270656	1.33060409	1.567144	1.3840075	1.49000708	2.89434429	3.16196672	2.6028087	1.98209156	[illegible]
ENSG0000173040	0.00149572	0.00200371	0.00146279	0	0.00250303	0.00112198	0.02882817	0.04102191	0.0473261	0.00135471	[illegible]
ENSG0000173041	11.1256333	11.8307067	11.6642202	9.3030466	11.9196008	9.58516336	0.80427068	1.03447381	1.26707647	0.91531748	[illegible]
ENSG0000173064	0.73651673	1.10344674	0.88874909	1.04833	0.7577344	1.20760767	0.7989621	0.78921923	0.66373604	0.8766789	[illegible]
[illegible]	[illegible]	[illegible]	[illegible]	[illegible]	[illegible]	[illegible]	[illegible]	[illegible]	[illegible]	[illegible]	[illegible]

ENSG00000173852	14.0725559	11.8454135	12.3884996	10.466992	15.7611418	10.9739898	6.78078924	7.24547785	7.71671329	7.15066076	[illegible]
ENSG00000173862	0.03046289	0.12242717	0	0	0	0.02285097	0	0	0	0.05518186	[illegible]
ENSG00000173867	0.12698873	0.20414175	0.07451562	0.1969773	0.08500442	0.19051481	0.12237774	0.17414106	0.07533856	0.13801989	[illegible]
ENSG00000173868	0	0	0	0	0	0	0.01231785	0.01878006	0.02843683	0.01736538	[illegible]
ENSG00000173875	2.35509909	2.50193033	2.63708298	2.3776186	2.35210095	2.54768847	3.17915445	3.46146021	3.14214149	3.62332178	[illegible]
ENSG00000173876	0	0.00319775	0	0.006171	0	0.00358115	0.00383393	0.00935248	0.01888207	0.09080363	[illegible]
ENSG00000173889	5.22114171	6.54393524	5.74481364	5.6588101	4.92053415	6.00985225	1.72845266	1.88350632	1.58235961	1.46048859	[illegible]
ENSG00000173890	0.11631349	0.08521255	0.07820484	0.1832376	0.19870155	0.15541342	0.22768305	0.23142028	0.28931893	0.22715611	[illegible]
ENSG00000173894	14.5475755	12.9830836	11.7145039	9.6778768	10.0326088	9.71755841	2.3274795	2.77841938	4.52719642	4.12363022	[illegible]
ENSG00000173898	0.01869068	0.04772988	0.0365583	0.0573797	0.02997492	0.05608137	0.30489091	0.42908599	0.49609991	0.68454913	[illegible]
ENSG00000173905	49.1348911	44.3328642	47.2555174	41.968642	56.4959862	39.1941772	16.8358375	20.2294734	24.3031914	20.4608156	[illegible]
ENSG00000173908	0	0	0	0	0	0	0	0	0.00945644	0	[illegible]
ENSG00000173914	2.15698203	3.01873065	2.27014618	1.6525797	1.80680782	1.74926793	1.37946497	1.58458581	1.48659466	1.29055859	[illegible]
ENSG00000173915	20.1788082	13.4943088	20.4340167	17.930632	25.2205887	18.6972285	38.3630769	40.2205877	37.1885091	36.9061653	[illegible]
ENSG00000173917	0	0	0	0	0	0	1.85385478	1.64709323	2.31901398	3.84094819	[illegible]
ENSG00000173918	0.00801496	0.00751596	0.00470309	0.0062162	0.00089418	0.00360733	0.02703376	0.04710428	0.01505759	0.17277199	[illegible]
ENSG00000173926	4.58768173	4.28155599	5.13993679	6.0260515	5.63831461	5.51866125	0.42467613	0.55886907	0.54810754	0.46635347	[illegible]
ENSG00000173928	0.32535961	0.3204868	0.29947845	0.2226522	0.2455487	0.30148629	0.44572677	0.99357019	0.92728167	0.34668891	[illegible]
ENSG00000173930	0	0.00404724	0.00590928	0	0	0	1.53336972	1.12451528	1.52649567	0.60746605	[illegible]
ENSG00000173933	0.52982758	0.5444682	0.51309839	0.4729354	0.42358829	0.61104297	0.36035085	0.44289977	0.34897184	0.27979791	[illegible]
[illegible]	[illegible]	[illegible]	[illegible]	[illegible]	[illegible]	[illegible]	[illegible]	[illegible]	[illegible]	[illegible]	[illegible]

ENSG0000174842	0.8609314	0.85021196	0.94992102	0.694345	0.69779314	0.79483823	1.16118457	1.23610335	1.33511797	0.81975305	[illegible]
ENSG0000174844	0.00317963	0.00496945	0	0.00548	0	0.00318015	0.01617202	0.00830525	0.00419194	0.00959953	[illegible]
ENSG0000174851	2.53914714	2.28217862	2.30610472	5.261149	2.48392544	2.35841618	4.11118367	4.19763394	3.89609238	3.56571621	[illegible]
ENSG0000174871	5.71764811	3.73044009	4.73231321	2.9250527	4.22192969	2.97054081	0.75866693	0.86078492	0.89065893	0.51736289	[illegible]
ENSG0000174876	0	0	0	0	0	0.00784691	0	0	0	0	[illegible]
ENSG0000174885	0	0	0	0	0	0	0	0	0	0	[illegible]
ENSG0000174886	3.57419856	2.95905179	3.41627061	4.552308	3.86258555	3.67488786	3.96873572	4.49989539	4.33752597	3.56527227	[illegible]
ENSG0000174891	4.72295523	4.59452463	4.93765209	3.8304153	4.4909165	3.74599429	1.14489888	1.31264396	1.67446555	1.24881179	[illegible]
ENSG0000174898	0.01990104	0.01666254	0.01459715	0.0385866	0.02497776	0.0447848	0.04794608	0.05360638	0.02213753	0.03154345	[illegible]
ENSG0000174899	0	0	0	0	0	0	0.34620909	0.39014094	0.2641012	0.17400981	[illegible]
ENSG0000174903	36.4869035	28.0327042	30.815688	29.946235	33.5101717	26.4423563	43.3914004	47.690894	49.0619505	28.9185732	[illegible]
ENSG0000174912	0	0	0	0	0.01027339	0	0.10353157	0.16235633	0.10015723	0.10008414	[illegible]
ENSG0000174914	0	0	0	0	0	0	0	0	0	0	[illegible]
ENSG0000174915	1.64634198	1.78326935	1.4408639	2.029034	1.76778292	1.82092004	1.78666999	1.85473797	1.91018614	1.81577453	[illegible]
ENSG0000174917	5.35395348	4.12890609	4.73245962	6.2256125	7.23615112	4.99318457	13.1238915	14.820356	14.0584037	10.8163845	[illegible]
ENSG0000174928	1.55922017	1.53569516	1.64516228	2.1801185	1.3793757	1.89114378	0.62079437	0.6023031	0.47989061	0.80357368	[illegible]
ENSG0000174930	0	0.01519746	0.01109472	0	0	0.01701959	0	0	0	0	[illegible]
ENSG0000174937	0	0	0	0	0	0	0	0	0	0	[illegible]
ENSG0000174938	0.44771931	0.30934546	0.34516104	0.5344124	0.48149491	0.42207596	1.65361576	1.96159826	1.2233995	2.72896954	[illegible]
ENSG0000174939	0.12587851	0.04864351	0.14204645	0.0938727	0.2592654	0.09805632	0.38103102	0.36041247	0.36861232	0.64460077	[illegible]
[illegible]											

ENSG00000175749	0.1473921	0.2961767	0.28829366	0.381043	0.08221845	0.29483329	0.07891126	0	0.04857959	0.04449879	[illegible]	[illegible]
ENSG00000175756	8.06534214	5.80905497	6.95978802	8.3076101	8.57460198	5.79852296	9.17966379	9.58541447	10.417371	8.5482394	[illegible]	[illegible]
ENSG00000175764	0.20757668	0.21875336	0.18947252	0.2897825	0.18989674	0.24082904	0.26005139	0.26838638	0.2148265	0.17296636	[illegible]	[illegible]
ENSG00000175766	0	0.00233662	0	0	0.00583779	0.00523354	0	0	0	0	[illegible]	[illegible]
ENSG00000175768	0.97423891	0.86294908	0.94237517	0.9565308	1.08987209	0.85859115	1.18426846	1.30365406	1.4870779	1.39832339	[illegible]	[illegible]
ENSG00000175772	0.00687641	0.01381779	0.00672501	0	0	0	0.00552227	0.0067355	0.00339964	0	[illegible]	[illegible]
ENSG00000175773	0.02253828	0.0150965	0.0661261	0.1165334	0	0.05071957	0.0723997	0.0883057	0.05571366	0.16330745	[illegible]	[illegible]
ENSG00000175779	0	0	0	0	0	0	0	0	0	0	[illegible]	[illegible]
ENSG00000175782	2.12482552	1.88903004	1.81356322	1.5917709	1.9530065	1.55041489	2.85820975	2.79554218	2.90079984	2.05571875	[illegible]	[illegible]
ENSG00000175785	0	0.00226741	0	0	0.00188829	0	0	0	0	0	[illegible]	[illegible]
ENSG00000175787	0.23656689	0.28566761	0.22158247	0.2713351	0.17471032	0.37490431	0.35320395	0.3851107	0.357459	0.46172382	[illegible]	[illegible]
ENSG00000175792	6.93153919	5.2753876	6.15072208	5.0251847	7.51920268	4.81117405	9.61441576	11.1561873	11.1516224	9.05889073	[illegible]	[illegible]
ENSG00000175793	0.02450931	0.03283345	0.02396966	0	0.02734363	0.01838504	0.15746246	0.21606356	0.27869538	0.02219864	[illegible]	[illegible]
ENSG00000175800	0	0	0	0	0	0	0	0	0	0	[illegible]	[illegible]
ENSG00000175806	2.59536236	2.56806425	2.78798091	1.9683196	2.9631652	2.41380151	1.98428674	1.59159737	1.50881585	2.10202129	[illegible]	[illegible]
ENSG00000175809	0	0	0	0	0	0	0	0.02090681	0	0	[illegible]	[illegible]
ENSG00000175820	0.00875927	0	0.01284962	0.0113224	0.00732916	0.00657055	0	0.00428989	0.01948729	0.00396674	[illegible]	[illegible]
ENSG00000175826	3.78223972	3.45054686	3.40957	4.3639247	3.2397497	3.55148483	2.97045133	3.31739689	3.33550926	2.91887698	[illegible]	[illegible]
ENSG00000175827	0	0	0	0	0	0	0	0	0	0	[illegible]	[illegible]
ENSG00000175832	6.77372955	3.2649336	3.81032759	2.321022	7.86534385	2.99404644	1.23114212	1.29836237	1.36509254	1.3482306	[illegible]	[illegible]
[illegible]	[illegible]	[illegible]	[illegible]	[illegible]	[illegible]	[illegible]	[illegible]	[illegible]	[illegible]	[illegible]	[illegible]	[illegible]

ENSG00000176624	8.81252078	8.23314231	7.92676466	9.2365187	9.91479767	9.62177759	2.1799352	2.75997205	2.98268309	5.31189922	[illegible]
ENSG00000176635	0.02392261	0.00801187	0.01754691	0.0386535	0.01000841	0.01345871	0	0	0	0	[illegible]
ENSG00000176641	0.8670258	0.53058338	0.69299725	0.9643509	1.29829078	1.35909475	1.34168181	1.91576957	1.89688341	5.48134099	[illegible]
ENSG00000176654	0	0.01598518	0.01166979	0.0154242	0	0.01790176	0.01916541	0.011688	0	0	[illegible]
ENSG00000176658	0.01555148	0.01799236	0.019357	0.0255845	0.01025221	0.01272606	1.67920333	2.07304568	2.05563031	1.42774027	[illegible]
ENSG00000176659	0	0.0079019	0	0	0	0	0	0	0	0	[illegible]
ENSG00000176678	0	0	0	0	0	0	0.43811297	0.42582217	0.34135481	0.47095043	[illegible]
ENSG00000176679	0	0	0	0	0	0	0	0	0	0	[illegible]
ENSG00000176681	0.02526202	0.04768617	0.02695178	0.0326541	0.02690232	0.03962203	0.01844299	0.02699383	0.01930168	0.02912049	[illegible]
ENSG00000176692	0	0	0.01872852	0	0	0	4.30612593	5.00831823	4.90425825	4.44025478	[illegible]
ENSG00000176695	0	0	0	0	0	0	0	0	0	0	[illegible]
ENSG00000176697	0.00083044	0	0	0.0005367	0.00046324	0.00031147	0.10170292	0.06954752	0.07841729	0.11507851	[illegible]
ENSG00000176700	0.3421176	0.47346238	0.38988815	0.4531913	0.25392824	0.48781084	0.73114233	0.78376245	0.56193617	0.96032129	[illegible]
ENSG00000176714	2.04106948	2.45789193	1.98892305	1.8763508	1.60301547	1.95665766	0.18344071	0.25261203	0.09835868	0.13347602	[illegible]
ENSG00000176715	0.50798868	0.55589281	0.49800093	0.6724572	0.54420101	0.50684899	0.72841715	0.6918146	0.68928774	0.72950299	[illegible]
ENSG00000176716	0.06584217	0.16538297	0.12878491	0.0425543	0.07345632	0.07408477	0	0	0	0	[illegible]
ENSG00000176720	1.35000042	0.74946986	1.28459302	0.6917249	1.10584336	0.64774316	2.25620687	2.64467233	2.3029292	0.57280858	[illegible]
ENSG00000176723	0.13497797	0.16072957	0.18334171	0.213247	0.09620847	0.15750052	0.30712599	0.38929088	0.27063511	0.27846428	[illegible]
ENSG00000176728	0	0	0	0	0.00829646	0	0	0.0072841	0.00367654	0	[illegible]
ENSG00000176731	3.01183124	2.91281171	3.42618214	3.5881976	2.70871871	3.38592487	3.99121341	4.5407087	3.78476313	4.74708771	[illegible]
ENSG00000176732	0.62775265	0.86146851	0.8684875	0.7124871	0.54661275	0.78099306	0.15984684	0.209962	0.22708939	0.51309959	[illegible]
ENSG00000176734	0.02598649	0.03046078	0.03812148	0.0083976	0.01087186	0.00974655	0	0	0.00321187	0	[illegible]
ENSG00000176742	0	0	0	0	0	0	0	0	0	0	[illegible]
ENSG00000176746	0.08268276	0.06645867	0.16172451	0.1068771	0.07379553	0.08683135	0	0	0	0.01497753	[illegible]
ENSG00000176748	0	0	0	0	0	0	0	0	0	0	[illegible]
ENSG00000176749	2.17717281	2.16206392	2.02860038	1.6591474	1.32927431	1.73877818	0.6958936	0.81164525	1.06566526	0.64258915	[illegible]

[illegible]

ENSG00000177427	0.8043544	0.92866818	0.85909822	1.1081247	0.49444017	1.11146412	0.60133475	0.60904543	0.41990377	0.49127307	[illegible]
ENSG00000177432	7.22079478	7.42232506	7.29267439	4.7925065	7.29671942	4.70818651	2.50911282	2.23066051	2.03896136	2.93043992	[illegible]
ENSG00000177447	0.05817643	0.03896749	0	0	0	0.08727917	0	0	0.02876192	0	[illegible]
ENSG00000177452	0	0	0	0	0.03078499	0	0	0	0	0	[illegible]
ENSG00000177453	0.8705787	2.51972423	1.7924428	1.6406049	0.73099693	1.9385058	0.11407018	0.11669057	0.08381602	0.11205041	[illegible]
ENSG00000177455	0	0	0	0	0	0	0	0.00674918	0	0	[illegible]
ENSG00000177459	0	0	0	0	0	0	0.14275673	0.14733227	0.141967	0.23531308	[illegible]
ENSG00000177462	0.06864694	0	0	0	0	0	0.11025726	0	0	0.12435019	[illegible]
ENSG00000177463	2.59826277	2.9777353	2.4203801	2.1395427	2.36087412	2.38933642	1.44674668	1.59538509	1.55907431	1.61250921	[illegible]
ENSG00000177464	0.00892791	0	0.00873133	0	0	0	0.00716978	0.01748991	0.01765552	0.00808621	[illegible]
ENSG00000177465	0.01364511	0.01827942	0.01334467	0.0529137	0.02283459	0.01023553	0.06574825	0.01336549	0.05396818	0.03707605	[illegible]
ENSG00000177468	0	0	0	0	0	0	0	0	0	0	[illegible]
ENSG00000177469	8.12479072	3.90253121	6.21673994	5.1041978	9.00800375	5.7093526	69.5484414	81.9333502	61.4928265	63.2323621	[illegible]
ENSG00000177476	0	0	0	0	0	0	0	0	0	0	[illegible]
ENSG00000177479	4.0927827	4.12201884	3.97857003	4.4192832	4.10700029	4.15877226	4.31480485	5.1517311	4.61333772	3.48486763	[illegible]

[illegible]

ENSG00000178338	1.28233573	1.87335332	1.74060613	1.5361089	1.33813082	[illegible]
ENSG00000178342	0	0	0	0	0	[illegible]
ENSG00000178343	0.06540789	0.01095281	0.07995968	0.1056842	0.05472887	[illegible]
ENSG00000178358	0	0	0	0	0	[illegible]
ENSG00000178363	0	0	0.02420899	0	0	[illegible]
ENSG00000178372	0	0	0	0	0	[illegible]
ENSG00000178381	2.83592499	2.78002446	2.66635343	3.4376373	2.50869779	[illegible]
ENSG00000178385	0.76195309	1.04420172	0.89935087	1.1886885	0.6579065	[illegible]
ENSG00000178386	0.3307499	0.43077518	0.32945759	0.3879465	0.22208253	[illegible]
ENSG00000178394	0.01498361	0	0.0146537	0	0.00835818	[illegible]
ENSG00000178395	0	0.0102898	0.01502388	0	0	[illegible]
ENSG00000178397	9.50221638	8.86915474	9.36286911	11.51313	7.89545557	[illegible]
ENSG00000178401	0.76607191	0.67439563	0.70104142	0.6295081	0.65931103	[illegible]
ENSG00000178403	0.04227775	0.1038338	0.12404065	0.0182163	0	[illegible]
ENSG00000178404	0.04082815	0.15314511	0.07985839	0.1055503	0.07287939	[illegible]
ENSG00000178409	1.21200283	1.31437314	1.08960817	1.0638983	1.24718044	[illegible]
ENSG00000178412	0.02900967	0	0.02837093	0	0	[illegible]
ENSG00000178425	0.62188863	0.60545183	0.49504318	0.7290857	0.55867472	[illegible]
ENSG00000178429	10.2459952	11.2804482	11.594485	31.105981	6.26288966	[illegible]
ENSG00000178430	0.19545021	0.20946504	0.1911468	0.0505284	0.04361052	[illegible]
ENSG00000178440	0.31881937	0.40768687	0.2834542	0.1498586	0.46886206	[illegible]
ENSG00000178445	16.0637278	22.8246241	18.060791	17.062246	13.9987592	[illegible]
ENSG00000178449	2.66991927	2.3631824	2.45959412	3.4665895	2.55315053	[illegible]
ENSG00000178457	0	0.03556506	0	0	0	[illegible]
ENSG00000178458	0.15683572	0	0.23007377	0.2027285	0.0874863	[illegible]
ENSG00000178460	0	0.00200613	0.00146455	0.0019357	0.00250605	[illegible]
ENSG00000178462	0	0	0	0	0	[illegible]
ENSG00000178464	0.19987436	0.36816728	0.29321029	0.5813121	0.36235605	[illegible]
ENSG00000178467	2.48100394	2.49941552	2.30261577	3.5470571	2.06943342	[illegible]
ENSG00000178473	0	0	0	0	0	[illegible]
ENSG00000178498	3.52488728	4.40980035	3.58060841	4.5648556	2.98434416	[illegible]
ENSG00000178502	11.1985835	11.6138497	10.6742451	10.454511	10.6150474	[illegible]
ENSG00000178503	0	0	0	0	0	[illegible]
ENSG00000178522	0	0.00613818	0	0	0.00511187	[illegible]
ENSG00000178531	16.3682483	10.098156	11.9439398	6.9434504	16.4378301	[illegible]
ENSG00000178537	0.0279853	0.03436583	0.02736912	0.0904357	0.03642519	[illegible]
ENSG00000178538	0.01643536	0.01926516	0.00803674	0	0.004584	[illegible]
ENSG00000178550	0	0	0	0	0	[illegible]
ENSG00000178556	0	0	0	0	0	[illegible]
ENSG00000178562	0	0	0	0	0.00184868	[illegible]
ENSG00000178567	13.1405835	15.3022549	13.0885347	8.921293	10.8682649	[illegible]
ENSG00000178568	1.90380023	2.91729602	2.48070313	2.060886	1.74420345	[illegible]
ENSG00000178573	10.1875795	11.3533406	10.7199738	10.424323	12.104345	[illegible]
ENSG00000178585	7.90259621	6.29142855	6.48072702	7.361803	6.65521608	[illegible]
ENSG00000178586	0	0	0	0	0	[illegible]
ENSG00000178591	0	0	0	0	0	[illegible]
ENSG00000178596	0	0	0	0	0	[illegible]
ENSG00000178597	0	0	0	0	0	[illegible]
ENSG00000178602	0	0	0	0	0	[illegible]
ENSG00000178605	7.04706089	5.94822409	5.87211122	6.0257814	6.17022627	[illegible]
ENSG00000178607	0.4913597	0.56231646	0.46605229	0.8521725	0.50135221	[illegible]
ENSG00000178623	0.05459198	0.04570822	0.03670561	0.0573353	0.03616242	[illegible]
ENSG00000178631	0.79932038	1.43410035	1.00506983	1.4022203	0.58919583	[illegible]
ENSG00000178636	0.09932123	0.09563247	0.05463809	0.0401201	0.05886622	[illegible]
ENSG00000178642	0.02072652	0.03470738	0.02027017	0.0401872	0.02312339	[illegible]
ENSG00000178645	0	0.00290395	0	0.0112082	0.00483681	[illegible]
ENSG00000178654	0.03641779	0.02439321	0	0	0	[illegible]
ENSG00000178660	0	0	0	0	0	[illegible]
ENSG00000178662	1.43475485	1.76551021	1.47568511	1.7020966	1.34496694	[illegible]
ENSG00000178665	2.04366976	2.72244149	2.11053831	1.6609082	1.59089902	[illegible]
ENSG00000178685	0.2860001	0.31849066	0.31653627	0.3696885	0.18120246	[illegible]
ENSG00000178690	0.01030033	0.07589258	0.03022062	0.0399431	0.01149149	[illegible]
ENSG00000178691	6.41572399	6.17272978	6.01381063	5.4375563	7.8136455	[illegible]
ENSG00000178694	1.93946837	2.02194478	1.94559285	1.7375174	1.99665174	[illegible]
ENSG00000178695	3.93053426	3.74939138	3.93805371	2.9754684	3.33351267	[illegible]
ENSG00000178700	1.17581953	1.33614752	1.16995404	1.0245984	0.85658212	[illegible]
ENSG00000178715	0.40195821	0.26923798	0.17868541	0.5668123	0.10191857	[illegible]
ENSG00000178718	0.03806962	0.01019985	0.00744628	0.0098419	0.07220255	[illegible]
ENSG00000178719	7.58731571	7.76773359	6.87333426	8.8986076	7.83352612	[illegible]
ENSG00000178722	0.09528793	0.08635196	0.15897097	0.0796987	0.08442048	[illegible]
ENSG00000178723	0.02872526	0.01924063	0	0	0.03204712	[illegible]
ENSG00000178726	0.01568739	0.00525383	0.00767099	0	0	[illegible]
ENSG00000178732	0.00917312	0.02150504	0.00448557	0.0237146	0.0127924	[illegible]
ENSG00000178734	0.36307333	0.35543485	0.38239297	0.4332135	0.1402131	[illegible]
ENSG00000178741	21.5454484	12.9021765	16.9040233	20.27439	26.7322153	[illegible]
ENSG00000178750	0.05566449	0.01864248	0.19053605	0.0359764	0.03105084	[illegible]
ENSG00000178752	0.88702576	0.58271807	0.60057364	0.7496898	0.63943624	[illegible]
ENSG00000178761	2.52791916	2.4187971	2.41935126	2.3839718	2.32849299	[illegible]
ENSG00000178762	0	0	0	0	0.02613145	[illegible]
ENSG00000178764	6.34623811	9.72343524	7.57170896	8.5216796	3.55642563	[illegible]
ENSG00000178772	0	0.00428673	0.01251791	0.0165452	0	[illegible]
ENSG00000178773	0	0.00238015	0	0	0.00198219	[illegible]
ENSG00000178776	0	0	0	0	0	[illegible]
ENSG00000178789	0	0	0	0	0	[illegible]
ENSG00000178795	0	0	0	0	0	[illegible]
ENSG00000178796	0	0	0.01692355	0	0.00482643	[illegible]
ENSG00000178802	0.76908271	0.59706268	0.66129315	1.0534121	0.76618426	[illegible]
ENSG00000178803	0.00837136	0	0	0.0162314	0.00466972	[illegible]
ENSG00000178804	0	0	0	0	0	[illegible]
ENSG00000178809	0.03553444	0.06148729	0.02606404	0.0956925	0.02642916	[illegible]
ENSG00000178814	0	0	0.00320489	0	0.00182801	[illegible]
ENSG00000178821	0	0	0	0.0283342	0.02853073	[illegible]
ENSG00000178826	0.00521517	0.01746601	0.01020068	0.016853	0.0087274	[illegible]
ENSG00000178828	0	0	0	0	0	[illegible]
ENSG00000178836	0	0	0	0	0	[illegible]
ENSG00000178852	0.04325034	0.0772527	0.07653933	0.0692171	0.04135879	[illegible]
ENSG00000178860	0	0.03040562	0.05549315	0.0146693	0	[illegible]
ENSG00000178863	0	0	0	0	0	[illegible]
ENSG00000178878	1.17789849	0.97771696	1.09237925	1.6800787	0.73210861	[illegible]
ENSG00000178882	0.00802933	0.00537817	0.01177881	0.0311365	0.01567626	[illegible]
ENSG00000178894	0	0	0	0	0	[illegible]
ENSG00000178896	4.30905825	3.53610948	4.06631555	3.4690108	4.68085915	[illegible]
ENSG00000178904	1.7908617	2.22644272	1.93579169	1.9444148	1.59772544	[illegible]
ENSG00000178913	54.3965441	49.5840258	54.2522856	67.877266	52.2117787	[illegible]
ENSG00000178917	0.53330804	0.6796629	0.56310631	0.4758434	0.4475522	[illegible]
ENSG00000178919	0	0	0	0	0	[illegible]
ENSG00000178921	2.18129454	2.23529679	1.79999085	1.7123826	2.18351159	[illegible]
ENSG00000178922	0.34925469	0.55971432	0.34915514	0.4481084	0.29728348	[illegible]
ENSG00000178927	0.78765131	0.76422074	0.78922105	0.5826134	0.8256314	[illegible]
ENSG00000178928	0.01170076	0.01959338	0.02288626	0.0151246	0.0097904	[illegible]
ENSG00000178934	0	0	0	0	0	[illegible]
ENSG00000178935	0.36425135	0.35290156	0.419844	0.2522343	0.37734758	[illegible]
ENSG00000178947	0.94902102	1.25070003	1.19933102	1.354172	0.86282738	[illegible]
ENSG00000178950	1.38719544	1.39943213	1.18892703	1.3797611	1.29200382	[illegible]
ENSG00000178951	1.49446362	1.51590553	1.28516357	1.3600096	1.74633966	[illegible]
ENSG00000178952	34.6168399	30.9419184	31.6569853	40.072671	39.8171371	[illegible]
ENSG00000178965	0.09214007	0.29423164	0.22423053	0.1634188	0.0741083	[illegible]
ENSG00000178966	4.92948324	3.65809782	4.04705736	2.4984684	5.66235677	[illegible]
ENSG00000178971	0.47405318	0.65998658	0.50767813	0.610237	0.33546341	[illegible]
ENSG00000178972	0.05051683	0.01691849	0.01235114	0.0326495	0.01408968	[illegible]
ENSG00000178974	4.14073359	3.86224261	4.46660763	4.8570867	3.9421554	[illegible]
ENSG00000178977	0.82044967	0.45623035	0.711549	1.420706	0.85488236	[illegible]
ENSG00000178980	9.3934257	7.5829524	9.65265587	8.7832553	7.19454807	[illegible]
ENSG00000178982	10.9577832	10.1506446	10.8032235	13.417166	13.5822818	[illegible]
ENSG00000178988	17.6657095	19.8122042	19.5644963	16.4291	14.8418747	[illegible]
ENSG00000178996	2.27150472	2.14256983	1.91845669	2.0718927	2.01305449	[illegible]
ENSG00000178997	0.00758704	0.04573727	0.07048988	0.0245178	0.0359738	[illegible]
ENSG00000178999	5.21637407	3.39734477	4.06735887	4.2532379	4.85504103	[illegible]
ENSG00000179002	0	0	0	0	0	[illegible]
ENSG00000179008	0	0	0	0	0	[illegible]
ENSG00000179010	42.7938743	40.3979862	42.3363297	37.533744	39.5188002	[illegible]
ENSG00000179021	12.1148645	12.070988	12.9392252	8.9836456	10.382171	[illegible]
ENSG00000179023	0	0	0.00623543	0	0.00355656	[illegible]
ENSG00000179028	0	0	0	0	0	[illegible]
ENSG00000179029	2.35998783	1.58571261	1.66771137	1.721319	2.23053922	[illegible]
ENSG00000179031	0	0	0	0	0	[illegible]
ENSG00000179038	0	0	0	0	0	[illegible]
ENSG00000179041	5.99579079	5.21963152	5.01288081	4.2051883	7.07953632	[illegible]
ENSG00000179044	0.04111685	0.03737669	0.04595605	0.0189815	0.02457413	[illegible]
ENSG00000179046	0.00366414	0.00736289	0.00716692	0	0.00408787	[illegible]
ENSG00000179051	16.7828196	13.1592882	13.0100382	15.384271	14.9559735	[illegible]
ENSG00000179055	0	0	0	0	0	[illegible]
ENSG00000179057	0.00247769	0	0.00242313	0	0.00276421	[illegible]
ENSG00000179058	0.09947451	0.05330368	0.09728428	0.1542989	0.0887824	[illegible]
ENSG00000179059	0.01256275	0.03365892	0.01228615	0.0243582	0.0245272	[illegible]
ENSG00000179061	0	0.17695074	0.25836153	0	0.09824281	[illegible]
ENSG00000179066	0	0	0	0	0	[illegible]
ENSG00000179071	0.33185482	0.21339035	0.12981922	0.0686338	0.19992494	[illegible]
ENSG00000179073	0.21630698	0.12418787	0.2014708	0.1331438	0.51137127	[illegible]
ENSG00000179082	0.25619825	0.24711213	0.25055729	0.1457131	0.27439265	[illegible]
ENSG00000179083	0.04866703	0.05215672	0.03331684	0.0314539	0.03529176	[illegible]
ENSG00000179085	14.9507646	13.9893848	16.0056066	19.650161	16.0430794	[illegible]
ENSG00000179088	0	0	0	0	0	[illegible]
ENSG00000179091	24.299165	18.2667612	22.523477	30.200729	31.1448283	[illegible]
ENSG00000179094	1.45071218	0.90929248	1.22187527	1.3948863	1.3303336	[illegible]
ENSG00000179097	0	0	0	0	0	[illegible]
ENSG00000179101	0.4834461	1.02542955	0.63040213	0.1041518	0.22473044	[illegible]
ENSG00000179104	1.27474441	2.73210809	1.80247563	2.9105397	1.4382106	[illegible]
ENSG00000179111	0.04288721	0.05027144	0.09437158	0.1385918	0.02990425	[illegible]
ENSG00000179115	3.4203395	2.75718898	2.77374994	3.1905813	3.43179289	[illegible]
ENSG00000179119	3.1947958	2.6551392	2.81163522	3.4257855	2.79532378	[illegible]
ENSG00000179131	0	0	0	0	0	[illegible]
ENSG00000179133	0.00874145	0.01171033	0	0	0.00975234	[illegible]
ENSG00000179134	4.74544626	4.5536748	4.21353005	3.9494221	4.42974503	[illegible]
ENSG00000179136	0.06267668	0.13527479	0.12259333	0.1170244	0.0815789	[illegible]
ENSG00000179141	0.01191047	0	0.01164823	0.0307914	0.01328783	[illegible]
ENSG00000179142	0	0	0	0	0	[illegible]
ENSG00000179144	0	0	0	0	0	[illegible]
ENSG00000179148	0	0	0.00303984	0.0040178	0	[illegible]
ENSG00000179151	1.16251549	1.40844308	1.31511978	1.5073976	1.16888287	[illegible]
ENSG00000179152	1.54516099	2.01392245	1.63455661	1.7469271	1.30148023	[illegible]
ENSG00000179157	0.1739797	0.08740077	0	0.2248891	0.07278719	[illegible]
ENSG00000179163	6.97497544	8.02562815	7.83768162	11.519276	5.29604119	[illegible]
ENSG00000179165	0.01528925	0.01024098	0.02990522	0.0790526	0.02558601	[illegible]
ENSG00000179168	0.24007255	0.16884462	0.18195965	0.1086126	0.22096401	[illegible]
ENSG00000179170	0	0	0	0	0	[illegible]
ENSG00000179172	0	0.01948375	0	0	0	[illegible]
ENSG00000179178	0	0	0	0	0	[illegible]
ENSG00000179195	5.25280653	6.73884376	6.03703002	7.618508	7.82317789	[illegible]
ENSG00000179213	0	0	0	0	0	[illegible]
ENSG00000179218	109.16053	80.075873	96.720515	87.649789	116.993449	[illegible]

ENSG00000179219	0.04116186	0.05514174	0.04025556	0	0.03444145	[illegible]
ENSG00000179222	19.6866104	26.2815386	20.0739727	25.259869	18.470873	[illegible]
ENSG00000179240	0.45119803	0.50932439	0.49726149	0.4648046	0.32578887	[illegible]
ENSG00000179241	12.0485966	10.933494	12.0270576	7.6745722	7.17613804	[illegible]
ENSG00000179242	0	0.00224618	0	0	0.00187061	[illegible]
ENSG00000179253	0.01611487	0.010794	0	0.0208304	0	[illegible]
ENSG00000179256	0	0	0	0	0	[illegible]
ENSG00000179262	9.32576211	6.22921365	7.28024064	7.9411628	10.5603462	[illegible]
ENSG00000179270	0	0	0	0	0	[illegible]
ENSG00000179271	48.5831878	37.306837	44.7984288	34.539694	55.7258894	[illegible]
ENSG00000179277	0	0.02253444	0	0.0434872	0.01876663	[illegible]
ENSG00000179284	0.09850712	0.16195492	0.14012829	0.127332	0.07493096	[illegible]
ENSG00000179292	0.12579914	0.11796716	0.17224102	0.1138271	0.07719078	[illegible]
ENSG00000179294	4.44444816	3.52552557	3.60425142	3.3566107	3.50431844	[illegible]
ENSG00000179295	24.4469496	24.223178	23.7796112	19.258162	29.6019429	[illegible]
ENSG00000179296	0	0.00454867	0	0	0.00378812	[illegible]
ENSG00000179299	0	0	0.00810286	0	0	[illegible]
ENSG00000179300	0	0	0	0	0	[illegible]
ENSG00000179304	0.03350782	0.0400787	0.02808861	0.0185626	0.02803707	[illegible]
ENSG00000179314	7.56642489	6.0425893	6.42556322	3.9364192	5.65054214	[illegible]
ENSG00000179331	4.244463	2.73683815	2.68695991	1.2976284	4.17531961	[illegible]
ENSG00000179335	1.02292859	0.89527014	0.90906814	1.0409481	0.94118926	[illegible]
ENSG00000179342	0	0.08988754	0	0.028911	0.02495272	[illegible]
ENSG00000179344	0	0	0.00231646	0	0	[illegible]
ENSG00000179348	0.19137357	0.18922555	0.20795546	0.270932	0.16605906	[illegible]
ENSG00000179361	0.0776353	0.0767639	0.09038801	0.0812379	0.05361772	[illegible]
ENSG00000179362	0.17632527	0.06186476	0.20118343	0.141094	0.10772541	[illegible]
ENSG00000179363	0	0.05930767	0	0.0572262	0.02469565	[illegible]
ENSG00000179364	2.30597269	2.58063788	2.28873088	2.2931683	2.6671755	[illegible]
ENSG00000179381	0	0	0	0	0	[illegible]
ENSG00000179387	5.34790403	5.32338537	5.34768564	4.3905009	5.00136034	[illegible]
ENSG00000179388	0.53339442	0.17272285	0.48364916	0.0365285	0.53202296	[illegible]
ENSG00000179397	0.02241549	0.05755463	0.04567072	0.0241455	0.02083973	[illegible]
ENSG00000179399	0.00982914	0	0	0	0	[illegible]
ENSG00000179403	1.2364553	0.78749535	1.08520738	1.0313577	1.20111686	[illegible]
ENSG00000179406	0.22977476	0.23599039	0.18975983	0.2442088	0.08829713	[illegible]
ENSG00000179407	0	0	0	0	0	[illegible]
ENSG00000179409	2.14732991	1.81446497	1.5822958	1.7108133	2.21763471	[illegible]
ENSG00000179412	0.01582993	0	0	0.0409241	0.03532109	[illegible]
ENSG00000179420	0.00798359	0.02673766	0.0078078	0	0	[illegible]
ENSG00000179428	1.46498817	0.37984734	0.94745189	0.1221721	0.98855031	[illegible]
ENSG00000179431	18.2841793	13.7855437	18.4520986	8.9188777	9.33495664	[illegible]
ENSG00000179443	0	0	0	0	0	[illegible]
ENSG00000179447	0	0	0	0	0	[illegible]
ENSG00000179449	0	0	0	0	0	[illegible]
ENSG00000179452	0.12443915	0	0	0.3217043	0	[illegible]
ENSG00000179454	3.69398331	4.80146687	4.21820803	4.8262111	3.18238965	[illegible]
ENSG00000179455	1.42389453	2.0967298	1.72962717	1.6725633	1.05903825	[illegible]
ENSG00000179456	48.516178	58.5807586	58.1292644	63.048188	36.0969253	[illegible]
ENSG00000179460	0.05679249	0.01902026	0.02777102	0	0.03168006	[illegible]
ENSG00000179467	0	0	0.07147416	0	0.04076743	[illegible]
ENSG00000179468	0	0.10398839	0	0	0.03464053	[illegible]
ENSG00000179476	0.30102497	0.40057417	0.35327641	0.4513675	0.26643011	[illegible]
ENSG00000179477	0	0.00922367	0.00673363	0	0.00768145	[illegible]
ENSG00000179520	0	0.00318219	0	0.006141	0	[illegible]
ENSG00000179523	1.49058991	1.94349077	1.64694644	1.6399535	0.9901627	[illegible]
ENSG00000179526	2.64081182	2.39068291	2.40904189	3.2079793	2.24266225	[illegible]
ENSG00000179528	0.0071447	0.0143569	0.00698739	0.0092354	0.00398547	[illegible]
ENSG00000179532	0.11176446	0.1625994	0.11629906	0.2155472	0.0887787	[illegible]
ENSG00000179542	0.01935591	0.01296489	0.00420661	0.0222398	0.00479873	[illegible]
ENSG00000179546	0.00798754	0.02140073	0.00781167	0	0	[illegible]
ENSG00000179562	9.08156291	9.0548983	8.65589752	11.559987	7.73722839	[illegible]
ENSG00000179564	0	0.05779917	0.08439118	0.083656	0	[illegible]
ENSG00000179571	0.02468766	0.00826809	0	0.0159558	0.0137713	[illegible]
ENSG00000179577	0	0	0	0	0	[illegible]
ENSG00000179580	0	0.01773869	0	0	0.00738637	[illegible]
ENSG00000179583	0.02034947	0.02044559	0.04602202	0.018084	0.02625003	[illegible]
ENSG00000179588	0.56300271	0.35390148	0.49978132	0.2687065	0.32130232	[illegible]
ENSG00000179593	0	0	0	0	0	[illegible]
ENSG00000179598	1.54853828	1.35768222	1.52675516	2.7177099	1.42563371	[illegible]
ENSG00000179600	0	0	0	0	0	[illegible]
ENSG00000179603	1.49079788	1.16331861	1.39502326	0.9315713	1.32978867	[illegible]
ENSG00000179604	6.90704588	10.1680887	6.9853545	9.5818397	4.45036789	[illegible]
ENSG00000179611	0	0	0.01135655	0	0.00647755	[illegible]
ENSG00000179615	0	0	0	0	0	[illegible]
ENSG00000179626	0	0	0	0	0	[illegible]
ENSG00000179627	1.32357788	0.90726731	1.3428255	0.9433819	1.02467764	[illegible]
ENSG00000179630	0.31284107	0.15239702	0.22251123	0.1516439	0.33513738	[illegible]
ENSG00000179632	12.4088785	11.6331383	11.4135769	13.570823	9.79434104	[illegible]
ENSG00000179636	0	0	0	0	0	[illegible]
ENSG00000179639	0	0	0	0	0	[illegible]
ENSG00000179673	0.08854324	0.03953844	0.05772913	0	0.01646377	[illegible]
ENSG00000179674	0	0	0	0	0.02796024	[illegible]
ENSG00000179676	0.0451081	0	0	0	0	[illegible]
ENSG00000179695	0	0	0	0	0	[illegible]
ENSG00000179698	0.02276027	0.05777124	0.02694527	0.0480016	0.02138301	[illegible]
ENSG00000179709	0.00458852	0.00307346	0	0	0.00255957	[illegible]
ENSG00000179715	0.00452284	0	0	0	0	[illegible]
ENSG00000179743	1.95766947	2.47573344	2.00327132	2.2178657	1.37874021	[illegible]
ENSG00000179750	0.34811118	0.09326815	0.19379261	0.1938351	0.29575691	[illegible]
ENSG00000179751	0	0	0	0	0	[illegible]
ENSG00000179755	0	0	0	0	0	[illegible]
ENSG00000179761	4.96425494	3.28997069	3.57988541	4.6222643	5.59039843	[illegible]
ENSG00000179766	0	0	0	0	0.00252506	[illegible]
ENSG00000179772	0	0.01637936	0	0.031609	0	[illegible]
ENSG00000179774	0.34702277	0.24696894	0.1909024	0.1682128	0.06049272	[illegible]
ENSG00000179776	0	0	0	0	0.01248069	[illegible]
ENSG00000179796	0	0.00297232	0.00867964	0.0573602	0.48516774	[illegible]
ENSG00000179799	0	0	0.01672154	0.0221012	0	[illegible]
ENSG00000179813	0	0	0	0	0	[illegible]
ENSG00000179817	0.02231976	0	0	0	0	[illegible]
ENSG00000179818	0.30872048	0.38084958	0.35500846	0.4911474	0.19756938	[illegible]
ENSG00000179820	0.57825551	0.34064388	0.55447093	0.3335583	0.73863829	[illegible]
ENSG00000179826	0	0.03283345	0.02396966	0	0.00683591	[illegible]
ENSG00000179832	0.25105238	0.27955748	0.24707377	0.2876611	0.24960231	[illegible]
ENSG00000179833	8.14225005	5.75575985	7.3221185	5.3645288	6.17773765	[illegible]
ENSG00000179837	25.8912243	27.6751497	25.750972	23.468862	21.56595	[illegible]
ENSG00000179840	0	0	0	0	0	[illegible]
ENSG00000179841	1.45757296	0.83615456	0.97459837	0.5317607	1.38280437	[illegible]
ENSG00000179846	0.00625031	0.01465296	0.00611269	0.0282774	0.00522984	[illegible]
ENSG00000179855	0.05947818	0.02987957	0.0290843	0.1345444	0.10782914	[illegible]
ENSG00000179859	1.10873363	1.18652854	0.87244265	1.4990605	1.04500986	[illegible]
ENSG00000179862	0.29981153	0.33469753	0.29321029	0.4844268	0.26479866	[illegible]
ENSG00000179869	0.00768391	0.00800614	0.0083497	0.0077252	0.009525	[illegible]
ENSG00000179873	0.48057688	0.55963401	0.58781116	0.6145041	0.26952117	[illegible]
ENSG00000179886	2.63904378	2.46131733	2.55480135	3.2990025	2.4224674	[illegible]
ENSG00000179889	1.47442049	1.25948234	1.33158476	2.1940927	1.53881838	[illegible]
ENSG00000179899	0.0663491	0.05866302	0.03893294	0.0445973	0.03997182	[illegible]
ENSG00000179902	0.18723377	0.59855796	0.3412528	0.3630323	0.06646372	[illegible]
ENSG00000179909	0.09375924	0.15700357	0.05730928	0.1174074	0.06864493	[illegible]
ENSG00000179912	0.4230795	0.63970076	0.45737164	0.8270214	0.41300481	[illegible]
ENSG00000179913	0	0	0	0	0	[illegible]
ENSG00000179914	0	0	0	0	0	[illegible]
ENSG00000179915	0.0294518	0.09405688	0.04989149	0.0686618	0.01936257	[illegible]
ENSG00000179918	2.84538257	2.87863893	3.05627202	3.6710365	3.31844945	[illegible]
ENSG00000179919	0	0	0	0	0	[illegible]
ENSG00000179921	0	0.00642595	0	0.0124009	0.00267576	[illegible]
ENSG00000179922	0.92728022	1.20000283	0.81881841	1.1869809	0.79346166	[illegible]
ENSG00000179930	0.04416243	0.06507753	0.12093217	0.1141702	0.06405033	[illegible]
ENSG00000179933	8.93512311	7.98149274	8.55843955	8.8668131	8.90915825	[illegible]
ENSG00000179934	0	0	0	0	0	[illegible]
ENSG00000179935	0.27409456	0.41783208	0.36049389	0.3298648	0.15289578	[illegible]
ENSG00000179938	0.00590288	0.01581538	0.02886457	0	0	[illegible]
ENSG00000179941	8.81274943	10.0463722	9.81989414	8.5291452	6.26619724	[illegible]
ENSG00000179943	1.10599355	1.07842797	1.02844632	1.0124563	1.02554754	[illegible]
ENSG00000179950	6.54271008	5.53217229	5.63122941	5.6627029	6.73960251	[illegible]
ENSG00000179954	0.00548218	0.00367205	0.00268074	0.0212591	0.00611615	[illegible]
ENSG00000179958	5.95495081	3.43260077	4.54987104	3.7377135	5.75325891	[illegible]
ENSG00000179965	1.19268554	0.96303304	0.98001007	0.8025211	1.14225947	[illegible]
ENSG00000179967	0.43455829	0.14553702	0.3541585	0.3744782	0.12120293	[illegible]
ENSG00000179978	0.01615256	0.05770261	0.0421251	0.0347984	0.02703072	[illegible]
ENSG00000179979	0.07983047	0.25277539	0.19163316	0.1594758	0.15383484	[illegible]
ENSG00000179981	3.33204659	3.96872155	3.72159383	3.5041712	3.13085883	[illegible]
ENSG00000179988	0.5889389	0.52335475	0.48418335	0.5732289	0.52485093	[illegible]
ENSG00000179994	0	0	0	0	0	[illegible]
ENSG00000179997	0.04604249	0	0	0	0	[illegible]
ENSG00000180008	3.54377299	3.51154006	3.56461633	2.7255755	4.43997759	[illegible]
ENSG00000180011	9.35427803	9.32548504	9.51996624	8.5052687	8.84097843	[illegible]
ENSG00000180015	0.22767145	0.25734028	0.16699394	0.1287527	0.30162496	[illegible]
ENSG00000180016	0	0	0	0	0	[illegible]
ENSG00000180019	0	0	0	0	0	[illegible]
ENSG00000180035	5.8668051	4.92261722	5.34447335	5.4481254	5.51511532	[illegible]
ENSG00000180042	0.01783605	0	0	0	0	[illegible]
ENSG00000180043	0	0	0	0	0	[illegible]
ENSG00000180044	0.06253345	0.03350872	0.06115659	0.0484991	0.09069447	[illegible]
ENSG00000180053	0.01985813	0	0	0	0	[illegible]
ENSG00000180061	0	0.00344032	0	0.0398349	0.00286509	[illegible]
ENSG00000180066	0	0	0	0	0	[illegible]
ENSG00000180068	0	0	0	0	0	[illegible]
ENSG00000180071	0.00625759	0.01467003	0.00305991	0	0.00349062	[illegible]
ENSG00000180081	0.07180781	0.04008168	0.09363567	0.09282	0.06675988	[illegible]
ENSG00000180083	0.02566062	0	0.02509563	0	0	[illegible]
ENSG00000180089	0.18383468	0.22793145	0.16831125	0.2679633	0.17891161	[illegible]
ENSG00000180090	0	0	0	0	0	[illegible]
ENSG00000180096	0.03747354	0.02175365	0.01954584	0.0419804	0.03065852	[illegible]
ENSG00000180098	1.02691404	1.19467521	1.09610962	1.143555	0.96794752	[illegible]
ENSG00000180104	2.28100386	2.22306587	2.06701317	1.938342	1.88670901	[illegible]
ENSG00000180105	0	0	0	0	0	[illegible]
ENSG00000180113	0.00197886	0.00132547	0	0	0	[illegible]
ENSG00000180116	0.00710219	0.01665006	0.00694581	0.0091804	0.00990438	[illegible]
ENSG00000180138	0.01339557	0.00897256	0.01310063	0.0173153	0.00747233	[illegible]
ENSG00000180139	0.00974002	0.03262011	0.00952557	0	0.00543319	[illegible]
ENSG00000180150	0	0	0	0	0	[illegible]
ENSG00000180152	0.04054055	0.02715471	0.07929587	0.1048068	0	[illegible]
ENSG00000180155	0.38054284	0.26988737	0.34151525	0.2025454	0.27845472	[illegible]
ENSG00000180172	0	0	0.03974793	0	0.02267142	[illegible]
ENSG00000180176	0	0	0	0	0	[illegible]
ENSG00000180178	0.0170006	0.02277454	0.0232768	0.0175802	0.01137996	[illegible]
ENSG00000180182	3.8757282	3.29220109	2.96391599	3.5062541	4.58265865	[illegible]
ENSG00000180185	4.67046657	3.38773855	4.24629144	2.8668752	3.90792853	[illegible]
ENSG00000180189	0	0	0	0	0	[illegible]

ENSG00000180190	0.45071306	0.38994739	0.35630467	0.4757894	0.38760173	0.41979642	1.275896	1.42744241	1.10857254	[illegible]
ENSG00000180198	3.0045341	1.89231652	2.18836072	1.7965987	3.039662	1.78349971	3.38022852	3.97416151	5.43872697	[illegible]
ENSG00000180205	0	0	0	0	0	0	0	0	0	[illegible]
ENSG00000180209	0.02264119	0.00758272	0	0.0146332	0.00631487	0.00849186	0.01818257	0	0.01119361	[illegible]
ENSG00000180210	0	0	0	0	0	0	0.00427251	0.01042235	0	[illegible]
ENSG00000180211	0.38368738	0.20559991	0.60038298	0.5951529	0.42805798	0.40293882	0.30812967	0.90197962	0.15175339	[illegible]
ENSG00000180219	0	0	0	0	0	0	0	0	0	[illegible]
ENSG00000180221	0	0.21762087	0.1270972	0.0839934	0.03624684	0.4386834	0.36528276	0	0.12850085	[illegible]
ENSG00000180228	4.35825542	3.5596557	3.92639577	4.3376566	4.94122166	4.25453262	1.91959817	2.10492221	2.31989928	[illegible]
ENSG00000180229	0.00934737	0.02156573	0.01422021	0.0147676	0.00811092	0.02103509	0.0258562	0.03153673	0.02413325	[illegible]
ENSG00000180230	0	0.03459614	0	0	0	0	0	0	0	[illegible]
ENSG00000180233	5.68760114	5.82808909	6.16089597	4.0757839	4.5753169	4.25643116	5.61997999	5.68833589	5.64682334	[illegible]
ENSG00000180245	0.02215102	0.08902264	0	0.0572656	0.12356313	0.03323207	0.03557786	0.04339421	0.0876102	[illegible]
ENSG00000180251	1.30063518	1.2937113	1.23383791	1.6055678	1.34947091	1.53175291	0.1410085	0.1019186	0.08359297	[illegible]
ENSG00000180257	1.3700179	1.32959513	1.29689169	1.3379406	1.22214764	1.27070408	0.42112893	0.50289263	0.44250093	[illegible]
ENSG00000180259	0	0	0	0	0	0	0	0	0	[illegible]
ENSG00000180263	0.29362492	0.37564854	0.2799809	0.3188174	0.29564137	0.37663666	0.54352542	0.70463948	0.68590761	[illegible]
ENSG00000180264	0	0	0	0	0	0	0	0	0	[illegible]
ENSG00000180269	0	0	0	0	0	0	0	0	0	[illegible]
ENSG00000180279	0	0.01450806	0	0.0559956	0.04832913	0.03249507	0.01739442	0	0.02141681	[illegible]
ENSG00000180284	0	0	0	0	0	0.06499013	0.06957767	0	0	[illegible]
ENSG00000180287	1.90914549	1.87863447	2.00274241	1.0584431	1.550532	1.18972552	0	0.0057806	0	[illegible]
ENSG00000180304	6.67393822	6.16473795	6.65135767	7.6024251	8.08541892	7.36719683	3.74757587	4.15941425	4.07418225	[illegible]
ENSG00000180305	0	0	0	0	0	0	0	0	0	[illegible]
ENSG00000180316	0.01219284	0.01633391	0.00794959	0.0105071	0.00680143	0.02438974	0.01305568	0.03980995	0.00803738	[illegible]
ENSG00000180318	3.34564773	3.64530775	3.14110405	2.9984173	2.46348106	2.61003971	0.01791204	0	0	[illegible]
ENSG00000180329	4.14926177	3.36285365	3.5036129	3.3897159	3.90240571	3.4011844	3.65485466	4.07252086	4.21981953	[illegible]
ENSG00000180332	0.00809792	0	0	0	0	0	1.18358956	1.19772922	0.81672299	[illegible]
ENSG00000180336	0.0103089	0.01381013	0.01008192	0.0186556	0.01725157	0.02319888	0.17219939	0.27465603	0.23750298	[illegible]
ENSG00000180340	9.8818365	14.5661782	9.61657313	13.487731	5.96561283	10.7897385	11.9951479	12.3220514	16.1751661	[illegible]
ENSG00000180344	0.07784961	0.41715924	0.30454209	0	0.17370469	0.23358772	0	0	0	[illegible]
ENSG00000180346	4.36331571	5.80378047	4.6455462	4.1000691	3.7113428	4.66581105	4.37387336	4.56186046	4.76569741	[illegible]
ENSG00000180347	0.00173511	0	0.00169691	0	0	0.0026031	0.00139343	0.00509867	0	[illegible]
ENSG00000180353	0.00249785	0.0016731	0.00244285	0	0.00139335	0.0018737	0.02407151	0.01712664	0.01234915	[illegible]
ENSG00000180354	4.09253413	8.59338533	6.72730808	8.0232854	1.651069	6.95226454	1.1503139	1.10744564	0.98508803	[illegible]
ENSG00000180357	4.91746709	7.02696842	5.41556164	5.8569482	4.06510373	6.18436808	4.95576121	5.84649022	5.28878582	[illegible]
ENSG00000180370	41.7612186	45.4040513	39.1472806	35.647996	36.4488483	38.6371259	18.6310807	20.053667	20.4848098	[illegible]
ENSG00000180376	0.71503177	0.97261583	0.84328368	0.7350344	0.56406821	0.78074158	0.67411861	0.74430843	0.78775259	[illegible]
ENSG00000180383	0.04095266	0.02743074	0.04005096	0	0	0	0.03288805	0	0.02024664	[illegible]
ENSG00000180385	1.03267532	1.00124863	0.86486396	0.6121151	0.82429008	0.7789152	1.30124651	1.47535701	1.27777479	[illegible]
ENSG00000180386	0	0	0	0	0	0	0	0	0	[illegible]
ENSG00000180389	0.08371361	0.11214541	0.08187041	0.3246288	0.09339447	0.18838698	0	0.1639963	0.16554915	[illegible]
ENSG00000180398	7.31990194	6.88206227	7.04146703	8.080059	6.80974557	7.12171601	7.56719415	8.53828879	6.93076544	[illegible]
ENSG00000180409	0	0	0	0	0	0	0	0	0	[illegible]
ENSG00000180422	0	0.00829988	0	0	0	0.00464751	0.02985339	0.03034341	0.00306307	[illegible]
ENSG00000180423	0.86817398	0.88207589	0.84905856	0.9204695	0.84341932	0.87807492	1.34741451	1.18480374	1.23942648	[illegible]
ENSG00000180424	0	0	0	0	0	0	0	0	0	[illegible]
ENSG00000180425	4.94321162	5.16522474	6.04296523	6.773059	5.79060023	4.63503247	4.12855956	2.37253843	2.88377982	[illegible]
ENSG00000180432	0.01102625	0.02584946	0.0269587	0.0071264	0.0153767	0.02067767	0.01328236	0.02700076	0.02180514	[illegible]
ENSG00000180433	0	0	0	0	0	0	0	0	0	[illegible]
ENSG00000180437	0	0	0	0	0	0	0	0	0	[illegible]
ENSG00000180438	0	0.01588131	0.01545861	0	0.01028683	0.01580927	0.01692522	0.00258046	0.00651222	[illegible]
ENSG00000180440	0	0	0	0	0.005606	0	0	0	0	[illegible]
ENSG00000180447	80.9278958	60.2187295	61.7910087	55.695022	65.6855419	52.4761516	3.24223071	3.23959419	1.74226419	[illegible]
ENSG00000180448	0.08200508	0.04441011	0.06569533	0.068788	0.08321547	0.08376381	0.10158671	0.09997852	0.11127651	[illegible]
ENSG00000180458	0.59191441	0.72686837	0.76219419	0.484575	0.47876457	0.32560713	0.29313346	0.33820785	0.11217766	[illegible]
ENSG00000180475	0	0	0.03039547	0	0.01733697	0	0	0	0	[illegible]
ENSG00000180479	0.35797107	0.57545927	0.42976476	0.4850576	0.3098543	0.58334307	0.25773849	0.23456307	0.19284478	[illegible]
ENSG00000180481	0.05329821	0.07139997	0.05825701	0.0851045	0.03147975	0.09407132	0.04783803	0.03992225	0.02945019	[illegible]
ENSG00000180483	0	0	0	0	0	0	0	0	0	[illegible]
ENSG00000180488	1.75219807	1.93029581	1.65851798	1.8352411	1.27912299	1.92506215	2.45685005	2.45026892	2.27709662	[illegible]
ENSG00000180509	0.00897639	0.01954072	0.01097344	0.0087023	0.01627348	0.01178348	0.01621961	0.02417923	0.00998517	[illegible]
ENSG00000180525	0	0	0	0	0	0	0	0	0	[illegible]
ENSG00000180530	3.22585298	3.61804768	3.21113852	3.131331	3.18822985	3.44303674	4.19472632	4.70135153	4.92209255	[illegible]
ENSG00000180532	0	0	0	0	0.00800465	0.02152834	0.01152399	0	0	[illegible]
ENSG00000180535	0	0.01437283	0	0.0554736	0	0.03219218	0.10339371	0.27323617	0.12730311	[illegible]
ENSG00000180537	1.25844071	1.06378257	1.16776471	1.3366531	0.99910426	1.22791575	1.49634739	1.41038402	1.12953796	[illegible]
ENSG00000180539	0	0.00565871	0.00826215	0	0.00471256	0.01267435	0.01356901	0.04137521	0.0125301	[illegible]
ENSG00000180543	0.0072264	0.00484036	0	0	0.00403104	0	1.02719103	0.92725932	0.51803706	[illegible]
ENSG00000180549	0	0	0	0	0	0	0	0	0	[illegible]
ENSG00000180573	114.803105	96.7231242	100.631113	103.3131	104.574238	74.0548065	23.7072088	27.3611936	41.278696	[illegible]
ENSG00000180574	0.00860837	0.02883012	0.00841883	0	0.00480193	0.01937206	0.01382633	0.03372787	0.04681495	[illegible]
ENSG00000180581	0.02641782	0.10617045	0.15501692	0.1024443	0.05894569	0.03963333	0.02121549	0.02587646	0.03918223	[illegible]
ENSG00000180592	0.13924998	0.23762113	0.14266893	0.1885681	0.09802048	0.10196785	0.47393837	0.54558586	0.49174279	[illegible]
ENSG00000180596	368.729185	321.116972	325.297751	272.65744	326.315904	225.842296	33.6267174	48.9965598	104.426686	[illegible]
ENSG00000180610	0	0.00906297	0	0	0	0	0	0.01325327	0.00668938	[illegible]
ENSG00000180611	9.12920776	8.6916019	9.55523422	5.9868705	5.68498613	5.44110976	1.18348143	1.40599555	2.30401109	[illegible]
ENSG00000180613	0.01563791	0	0	0.0101069	0.00872316	0.01173039	0.00627921	0.02297619	0.00386562	[illegible]
ENSG00000180616	0.07846028	0.00583933	0.06820688	0.0112688	0.04862979	0.04577612	0.02100316	0.02561749	0.02155005	[illegible]
ENSG00000180626	1.91358009	2.33577113	1.80811542	1.2304849	1.60205665	1.52528982	2.27781936	2.16614656	1.85689796	[illegible]
ENSG00000180628	4.58576449	4.41796987	4.75553098	4.3484889	4.9280819	4.37435593	5.38391232	5.55874101	5.06094919	[illegible]
ENSG00000180636	0	0	0	0	0	0	0	0	0	[illegible]
ENSG00000180638	0.00158611	0.00531201	0.00620475	0.0102512	0.00353906	0.01308758	0.00254753	0.00310721	0	[illegible]
ENSG00000180644	0	0	0	0.0082907	0	0.01443363	0	0	0	[illegible]
ENSG00000180658	0	0	0	0	0	0	0	0	0	[illegible]
ENSG00000180660	2.81608321	2.47292771	2.79745012	2.1066815	1.92957404	2.37022837	0.39176033	0.51040815	0.6632359	[illegible]
ENSG00000180662	0	0	0.05891609	0	0.10081365	0.1355682	0	0	0	[illegible]
ENSG00000180663	0	0	0	0	0	0	0	0	0	[illegible]
ENSG00000180667	2.13343519	2.33458797	2.07800724	2.409089	2.16219565	3.12287314	1.31205523	1.46652733	1.49238001	[illegible]
ENSG00000180672	14.2350865	17.5016106	16.4786984	23.481085	14.1177424	22.5483369	2.8133579	3.68252551	6.7757967	[illegible]
ENSG00000180673	0	0	0	0	0	0.01227848	0	0.03206631	0.00809249	[illegible]
ENSG00000180694	4.77218884	3.0916055	3.54333212	3.518693	3.09606953	3.42433247	1.76241238	1.96319954	2.079449	[illegible]
ENSG00000180697	0.01158926	0	0	0	0.00646474	0	0	0	0	[illegible]
ENSG00000180708	0	0	0	0	0	0	0	0	0	[illegible]
ENSG00000180712	0.08303577	0.14301944	0.06960642	0.1533335	0.07278719	0.21355609	0.08573649	0.01161917	0.05278136	[illegible]
ENSG00000180714	0	0	0	0	0	0	0	0	0	[illegible]
ENSG00000180720	0.39518754	0.33823146	0.3435434	0.1418961	0.8817761	0.27997112	0.0176314	0.02150496	0.05427148	[illegible]
ENSG00000180723	0	0	0	0	0	0	0	0	0	[illegible]
ENSG00000180725	0	0	0	0	0	0	0	0	0	[illegible]
ENSG00000180730	1.13818312	0.66424586	0.77147114	0.0728334	1.25723322	0.21133155	9.27621152	9.98958202	8.85289985	[illegible]
ENSG00000180739	1.26494482	0.66193757	0.88916082	0.7068353	0.89304164	0.78578013	0	0.0129065	0.01954306	[illegible]
ENSG00000180745	0.0279772	0	0	0	0	0.0209864	0.06740337	0	0	[illegible]
ENSG00000180747	0.09202187	0.12327541	0.12059429	0.0832643	0.09753034	0.10906407	0.2143113	0.20551043	0.14467352	[illegible]
ENSG00000180758	0.09782115	0.10296339	0.12755644	0.1144029	0.10653502	0.14675615	2.03127774	2.17640552	1.86538885	[illegible]
ENSG00000180764	0.17205879	0.18334862	0.25622994	0.1668046	0.20722535	0.19066506	0.16329901	0.08043618	0.18172845	[illegible]
ENSG00000180767	0	0	0.01761884	0	0	0	0.0144678	0	0.00890671	[illegible]
ENSG00000180769	0.34124662	0.41680849	0.49471019	0.6279204	0.47924578	0.53604716	0.24825395	0.26740303	0.21237535	[illegible]
ENSG00000180770	0	0.02230164	0	0	0	0	0	0	0	[illegible]
ENSG00000180771	3.41571243	4.09728198	3.71895047	3.0877593	3.12163083	2.92505117	1.6673579	1.51614215	1.76595951	[illegible]
ENSG00000180772	0	0	0	0	0	0	0	0	0	[illegible]
ENSG00000180773	2.8401259	2.67346775	2.52885253	2.0590988	2.63092978	2.21078022	1.25418973	1.30027175	1.42619399	[illegible]
ENSG00000180776	2.07524366	1.82574421	1.96748524	1.9236851	2.14530503	1.91849183	1.47800312	1.47325411	1.68016412	[illegible]
ENSG00000180777	0.06130874	0.03928011	0.0391036	0.0206736	0.02527776	0.05398734	0.08990832	0.08094019	0.07643521	[illegible]
ENSG00000180785	0	0	0	0	0	0	0.00636412	0	0	[illegible]
ENSG00000180787	6.63662717	8.17324806	6.51472046	5.2736079	5.65322668	5.49377596	1.8146861	1.80707789	1.20286274	[illegible]
ENSG00000180801	1.00879847	0.43278052	0.77496243	0.3900151	0.89084633	0.39779492	2.42797442	2.59420428	2.02359372	[illegible]
ENSG00000180803	0	0	0	0	0	0	0	0	0	[illegible]
ENSG00000180806	0	0	0	0	0	0	0	0	0	[illegible]
ENSG00000180815	0.19810565	0.20361712	0.2405095	0.2560747	0.144803	0.22674917	0.26607043	0.28437785	0.23218943	[illegible]
ENSG00000180817	8.81493172	6.35712305	7.90054162	9.7373004	10.2971656	9.14042884	19.1931719	21.0766312	24.500358	[illegible]
ENSG00000180818	0	0	0	0	0	0	0	0	0	[illegible]
ENSG00000180822	0.60724359	0.68301854	0.69696077	0.6457187	0.61355484	0.74772153	0.97900454	1.09308515	0.96069118	[illegible]
ENSG00000180828	0	0	0	0	0	0	0	0	0	[illegible]
ENSG00000180834	3.01690147	2.0373321	2.36199265	2.3014608	3.69224637	2.20122078	0.35084228	0.58132702	0.65203501	[illegible]
ENSG00000180846	0	0	0	0	0	0	0	0	0	[illegible]
ENSG00000180855	1.60078407	1.32782038	1.45631448	1.8767166	1.74956182	1.9617524	0.5231887	0.70194487	0.70399022	[illegible]
ENSG00000180861	0	0	0	0	0	0	0	0	0	[illegible]
ENSG00000180867	1.69123634	1.98933701	1.37160874	1.1197202	1.08146729	1.0726699	1.78883473	1.41415321	1.08765225	[illegible]
ENSG00000180869	0	0	0	0	0.00316466	0	0	0	0.00841442	[illegible]
ENSG00000180871	0.01077918	0.0108301	0.01581276	0	0.00901928	0.01617146	0.00865649	0.01055829	0.01332283	[illegible]
ENSG00000180872	0	0	0	0	0	0	0	0	0	[illegible]
ENSG00000180875	0.00772895	0.0207079	0.04535267	0.0399623	0.0172455	0.02319072	0.72000373	1.12801528	1.62397957	[illegible]
ENSG00000180878	0	0	0	0	0	0	0	0	0	[illegible]
ENSG00000180879	6.41166929	5.67226148	6.77081656	9.4492689	6.20720281	7.42958023	4.97804083	5.24501406	5.03918813	[illegible]
ENSG00000180881	0.24154439	0.29294568	0.2446128	0.3066819	0.16981848	0.32056419	0.14691842	0.13439696	0.11659102	[illegible]
ENSG00000180884	1.05851353	1.12705698	1.0059089	1.1810857	0.85505493	1.13484552	0.53730559	0.58198995	0.54553638	[illegible]
ENSG00000180891	2.40299473	2.02530442	2.00666874	1.7150077	1.89313556	1.47079789	1.23926924	1.23079316	0.93089709	[illegible]
ENSG00000180900	4.95568244	4.94642689	4.01533878	4.6847778	4.52699377	4.5221744	5.93548513	6.57832483	6.54646959	[illegible]
ENSG00000180901	3.55816745	3.40099155	3.67896271	3.452041	3.09154524	3.14100375	3.519956	3.48525336	2.99296862	[illegible]
ENSG00000180902	0.45315556	0.50616724	0.45184349	0.6904727	0.46178213	0.63711873	0.78374481	0.74515519	0.55007771	[illegible]
ENSG00000180909	0	0	0	0	0	0	0	0	0	[illegible]
ENSG00000180910	0	0	0	0	0	0	0	0	0	[illegible]
ENSG00000180913	0	0	0	0	0	0	0	0	0	[illegible]
ENSG00000180914	0.32028272	0.23875162	0.37385605	0.2670986	0.2708724	0.30612759	0.19913109	0.16191973	0.20176219	[illegible]
ENSG00000180917	1.62902698	1.80193636	1.8140143	1.6465897	1.58381451	1.74003786	1.14800313	1.21552492	1.09587623	[illegible]
ENSG00000180919	0	0	0	0	0	0	0	0	0	[illegible]
ENSG00000180921	0.01043597	0.00233006	0.00680412	0	0.0058214	0.00521885	0.00558724	0.00340737	0.00515945	[illegible]
ENSG00000180926	0	0	0	0	0	0	0	0	0	[illegible]
ENSG00000180929	0.01478428	0.0198055	0	0	0.00824699	0.01109006	0.01187289	0.01448132	0.00730922	[illegible]
ENSG00000180934	0	0	0	0	0	0	0	0	0	[illegible]
ENSG00000180938	1.07268447	1.3974516	1.26080427	1.3611283	0.83990857	1.06302028	1.79402035	2.07248753	1.32824757	[illegible]
ENSG00000180953	0.31838989	0.18418133	0.22645788	0.4489703	0.31484462	0.4125283	1.04600823	1.23328601	0.91583543	[illegible]
ENSG00000180957	2.19589878	2.81134696	2.55403324	2.1583442	3.34105393	1.81840599	2.463843	3.04044119	4.00995453	[illegible]
ENSG00000180964	13.3887998	14.0915185	14.8826199	13.158359	11.0616781	11.13043	16.0636511	17.8915159	17.355778	[illegible]
ENSG00000180974	0	0	0	0	0	0	0	0	0	[illegible]
ENSG00000180979	2.83877199	2.70092966	2.50179607	2.3726293	2.21693844	2.23832496	0.95334846	1.04904446	0.95212301	[illegible]
ENSG00000180987	0	0.17133326	0	0	0.0356715	0	0	0.06263747	0.06323058	[illegible]
ENSG00000180988	0	0	0	0	0.01737047	0.02335877	0.02500763	0	0	[illegible]
ENSG00000180992	26.7007886	16.7555324	21.9915388	20.350992	25.2939279	17.8793566	26.7221915	29.5219063	33.7349681	[illegible]
ENSG00000180998	2.17922606	2.01469807	2.27745719	1.5918784	1.54496654	1.51304019	0.1139588	0.10672846	0.13530021	[illegible]
ENSG00000180999	0.0097136	0.00650633	0.00949973	0	0.00541846	0.02185925	0	0.00951455	0	[illegible]
ENSG00000181001	0	0.08966974	0	0	0	0	0	0	0	[illegible]
ENSG00000181004	0.89836015	1.02008556	0.82837551	0.9787555	0.74930032	1.01082343	1.01690154	0.7793851	0.71274138	[illegible]
ENSG00000181007	2.0270979	2.51358047	2.17088147	2.2809983	2.07617204	2.42538243	0.84534788	0.9298759	1.05187459	[illegible]
ENSG00000181009	0.06233992	0.10439067	0.03048366	0.0402908	0.0521618	0.02338136	0	0	0	[illegible]
ENSG00000181013	0.03036245	0.06101175	0.05938786	0.0588705	0.02540523	0.0797147	0	0	0	[illegible]
ENSG00000181016	0.05747328	0.08554781	0.05620784	0.0825455	0.07480625	0.17244856	0.13333767	0.16263155	0.09787146	[illegible]
ENSG00000181017	0	0.01177741	0	0	0	0.01318949	0	0	0	[illegible]
ENSG00000181019	1.47458482	0.79988256	1.38727184	1.7397714	4.00972466	1.74455601	4.13543454	3.23770987	2.7823525	[illegible]
ENSG00000181023	0	0	0.05794137	0	0	0	0	0	0	[illegible]
ENSG00000181026	5.45449784	3.4173055	4.44368002	4.9255648	4.07147021	4.97837513	1.96811038	2.44025816	3.07669716	[illegible]
ENSG00000181027	0.61852268	0.55130767	0.55409848	0.4091995	0.58228649	0.42256517	1.21507359	1.2546299	1.00887375	[illegible]
ENSG00000181029	0.08627464	0.06604357	0.09642862	0.1911772	0.1100019	0.08320725	0.14846784	0.16901339	0.09749357	[illegible]
ENSG00000181031	0.09860098	0.10395894	0.10000147	0.0778881	0.08453969	0.07807211	0.04399109	0.05186728	0.04874748	[illegible]
ENSG00000181035	1.75558721	1.68911072	1.51348119	2.2168321	1.34158977	2.10477175	1.14908308	1.06357484	0.99839011	[illegible]
ENSG00000181036	0	0	0	0.005098	0	0	0.00316727	0	0.00194984	[illegible]
ENSG00000181038	1.48474082	1.20534502	1.3485266	1.5483234	1.42957305	1.29344407	1.59503046	1.57164297	1.26839336	[illegible]
ENSG00000181039	0.49982427	0.63371005	0.54992155	0.5653211	0.48294328	0.72977566	2.81694179	2.76264044	2.14455144	[illegible]
ENSG00000181045	0.78004685	0.93245681	0.7238336	0.9610039	0.5316154	0.81344652	0.76668286	0.62884429	0.49912282	[illegible]
ENSG00000181061	26.7737956	22.5083491	26.9658279	23.661733	31.9417076	22.8308041	11.5147938	12.7944024	13.0974595	[illegible]
ENSG00000181072	0	0	0.0045863	0.0080824	0.01133571	0.00117258	0	0	0	[illegible]

ENSG00000181074	0.12431915	0.18735961	0.21276832	0.1205228	0.08668484	0.30307838	0	0	0	0	[illegible]
ENSG00000181085	0	0.0076517	0.00372402	0.0147663	0	0.00856911	0.30885777	0.40655154	0.41981398	0.96913149	[illegible]
ENSG00000181090	1.67374931	2.0547149	1.74939826	1.8636476	1.8491249	1.72364479	1.21635199	1.38780542	1.62653847	1.33448987	[illegible]
ENSG00000181092	0.03037015	0.04850882	0.03427092	0.0422767	0.03127591	0.01927657	0.02063727	0.01830634	0.0173247	0.04020244	[illegible]
ENSG00000181097	0.09851169	0.24194386	0.11239977	0.1910068	0.11906248	0.30790027	0.39556127	0.4824649	0.3815094	0.50560418	[illegible]
ENSG00000181101	0	0	0	0	0	0	0	0.03085952	0.01557586	0	[illegible]
ENSG00000181104	32.3431449	27.8572686	28.4664346	22.232723	29.3694162	25.40698	109.767022	110.947937	80.8180221	52.6236031	[illegible]
ENSG00000181109	0.03346806	0.04483487	0.03273116	0.2595682	0	0.07531567	0	0	0	0	[illegible]
ENSG00000181123	0	0	0	0	0	0	0	0	0	0	[illegible]
ENSG00000181126	0.03445189	0.06346015	0.04632832	0.061233	0.04083824	0.07106885	0.04495959	0.02109119	0.02129089	0.02730337	[illegible]
ENSG00000181135	0.08220811	0.12964504	0.10889356	0.149307	0.08010539	0.11474624	0.42620029	0.39344391	0.38224979	0.31008352	[illegible]
ENSG00000181143	0	0	0	0	0	0	0	0	0	0	[illegible]
ENSG00000181163	46.2736355	41.8767446	45.1424419	51.698301	48.8200558	48.010448	59.6756184	67.6521458	93.5633798	51.9050563	[illegible]
ENSG00000181171	0	0	0	0	0	0	0	0	0	0	[illegible]
ENSG00000181191	3.82891099	5.04113711	4.28286113	6.63426	5.27498653	5.99124169	0.91570329	0.98533112	1.04413372	1.71249988	[illegible]
ENSG00000181192	11.7448649	13.205148	11.7212133	14.664055	9.59332015	12.5029266	5.32847056	5.85667792	5.71983862	5.52602106	[illegible]
ENSG00000181195	0	0	0	0	0.00277231	0.00372804	0.15166537	0.16064556	0.22113639	0.01350403	[illegible]
ENSG00000181201	10.4693057	7.58691021	10.3086823	12.378124	7.69365401	7.58525634	0.22956002	0.24499447	0.44163263	0.03236276	[illegible]
ENSG00000181211	0	0	0	0	0	0	0	0	0	0	[illegible]
ENSG00000181214	0	0	0	0	0	0	0	0	0	0	[illegible]
ENSG00000181215	0	0	0	0	0	0	0	0	0	0	[illegible]
ENSG00000181218	90.2562672	63.1971014	76.5760655	83.405398	77.2057794	60.3433383	23.3780966	30.9328093	41.2808982	9.88734405	[illegible]
ENSG00000181220	3.16557353	3.26887496	2.95732414	3.659322	2.92260274	3.16793282	1.07493938	1.09577365	1.17015144	1.22118574	[illegible]
ENSG00000181222	1.861479	2.06949313	1.72520552	2.0217206	2.58162889	2.17529264	2.81890851	2.71239696	3.38076844	2.05522813	[illegible]
ENSG00000181227	0	0	0	0	0.01996864	0	0	0	0.01179867	0	[illegible]
ENSG00000181234	0	0	0	0	0	0	0	0	0	0	[illegible]
ENSG00000181240	0	0.0094539	0	0.0091221	0.0039366	0.0105874	0.02833687	0.00691248	0.02442276	0.10866006	[illegible]
ENSG00000181260	0	0	0	0	0	0	0	0	0	0	[illegible]
ENSG00000181264	1.47836364	1.43488945	1.47804459	1.6188367	1.41164397	1.65813843	1.53508939	1.49879749	1.15801877	2.28992444	[illegible]
ENSG00000181273	0	0	0	0	0	0	0	0	0	0	[illegible]
ENSG00000181274	7.88195542	6.57238733	8.22325831	9.299939	6.45233307	8.68768279	8.65503261	10.8000192	11.2203864	6.83423885	[illegible]
ENSG00000181282	0	0	0	0	0	0	0	0	0	0	[illegible]
ENSG00000181284	0.05733372	0.08229221	0.12816308	0.0741105	0.10051476	0.10444666	2.12456781	2.15008103	2.00441733	2.21066822	[illegible]
ENSG00000181291	0.04419357	0.03805914	0.03704616	0.0734469	0.16904307	0.10892372	0	0	0	0	[illegible]
ENSG00000181296	0	0	0	0	0	0	0	0	0	0	[illegible]
ENSG00000181315	1.17995442	0.99093397	0.95727407	1.4096812	0.86264575	1.28408461	0.72864058	0.90185454	0.86620016	0.50601869	[illegible]
ENSG00000181322	0.00524359	0.00702448	0.01025628	0.0203339	0.00584997	0.01770007	0.0021055	0.00513614	0	0.00474924	[illegible]
ENSG00000181323	0.03282051	0	0	0	0	0	0	0	0	0.02972628	[illegible]
ENSG00000181325	0	0	0	0	0	0	0	0	0	0	[illegible]
ENSG00000181333	0	0	0	0	0	0	0	0	0	0	[illegible]
ENSG00000181350	1.82559126	0.93808705	1.13337604	0.6188665	2.47849312	1.16132306	0.00359335	0	0.01769719	0.04052646	[illegible]
ENSG00000181355	0	0.00428248	0.0041685	0.0027548	0	0.00479594	0	0	0.00421454	0.0038605	[illegible]
ENSG00000181358	0	0	0	0	0	0	0	0	0	0	[illegible]
ENSG00000181359	0.18576219	0.1451642	0.39362285	0.2801392	0.43175877	0.39481037	0.19890792	0.42456294	0.22959806	0.39258106	[illegible]
ENSG00000181371	0	0	0	0	0	0	0	0	0	0	[illegible]
ENSG00000181374	0	0	0	0	0	0	0	0	0	0	[illegible]
ENSG00000181378	0.00753579	0.00841265	0.00573212	0.0021646	0.0060719	0.00565278	0.00067242	0	0	0.00151674	[illegible]
ENSG00000181381	0.51184675	0.62222091	0.50917463	0.5606108	0.45933689	0.60156892	1.67872072	1.61506156	0.70072396	0.79801388	[illegible]
ENSG00000181392	0.01303089	0.02327546	0.02973595	0.0112293	0.00484594	0.02606612	0.06278869	0.0212731	0.0279169	0.09048487	[illegible]
ENSG00000181395	0	0	0	0	0	0	0	0	0	0	[illegible]
ENSG00000181396	1.30623945	1.12623842	1.15645442	1.2238212	1.18446418	1.01225855	1.77148272	1.98366253	1.72082124	1.96232938	[illegible]
ENSG00000181404	0.31655078	0.32946314	0.29529263	0.3714085	0.19559494	0.3397399	0.6140245	0.50564285	0.41171533	0.59546885	[illegible]
ENSG00000181408	0	0	0	0	0	0	0	0	0	0	[illegible]
ENSG00000181409	0.04860739	0.04578469	0.06239252	0.0667577	0.0127098	0.06836553	0.01829783	0.04463562	0.00901165	0.07429188	[illegible]
ENSG00000181418	1.35830858	1.08256661	2.53068874	2.0224721	2.13401002	1.50255661	1.33246153	1.21258397	1.88285109	1.08230933	[illegible]
ENSG00000181433	0	0	0	0	0	0	0	0	0	0	[illegible]
ENSG00000181444	0.12088477	0.19308359	0.13641131	0.3245352	0.09336752	0.31388772	0.57500944	0.3916559	0.25284934	1.22963535	[illegible]
ENSG00000181449	274.357981	368.121039	320.748869	276.92692	236.780415	279.76281	0.00677918	0.36381577	0.00834683	0.06116544	[illegible]
ENSG00000181450	5.29933695	7.38633181	6.39421807	5.7513034	6.31122324	5.88795524	0.72063738	0.9399981	0.95752513	0.98207074	[illegible]
ENSG00000181458	1.55435963	1.59281168	1.34753074	1.3808187	1.81010663	2.06482437	1.26815979	1.59832973	1.5353932	1.36294805	[illegible]
ENSG00000181464	0	0	0	0	0	0	0	0	0	0	[illegible]
ENSG00000181467	19.2231252	19.2066313	18.8099787	16.77917	15.2947853	17.5091358	7.62335639	8.47314192	9.32235864	9.45426273	[illegible]
ENSG00000181472	7.11348949	5.65898782	5.99800087	5.3390415	7.83138302	6.07929059	10.2023829	11.0850085	12.566801	6.83017544	[illegible]
ENSG00000181481	0.00680742	0.01367916	0	0.0043997	0	0.00255321	1.58539209	1.52362067	1.52290268	2.28128552	[illegible]
ENSG00000181495	0	0	0	0	0	0	0	0	0	0	[illegible]
ENSG00000181499	0	0	0	0	0	0	0	0	0	0	[illegible]
ENSG00000181511	0	0	0	0	0	0	0	0	0	0	[illegible]
ENSG00000181513	0.07743969	0.16112912	0.13374413	0.1895508	0.08823321	0.22370613	0.18921597	0.24531116	0.13033367	0.49097218	[illegible]
ENSG00000181514	0	0	0	0	0	0	0	0	0	0	[illegible]
ENSG00000181518	0	0	0	0	0	0	0	0	0	0	[illegible]
ENSG00000181523	0.70120083	0.87388095	0.73979153	0.9173704	0.53416939	0.83626807	1.54032334	1.47634147	0.88522808	0.99690433	[illegible]
ENSG00000181524	1.22488322	1.12811352	0.97330496	2.0780873	0.93949067	2.06733458	1.16811154	1.2747693	1.40038457	0.55470226	[illegible]
ENSG00000181541	0	0	0	0	0	0	0.06547895	0.19395659	0.18427589	0.5169386	[illegible]
ENSG00000181544	1.14892891	1.06374924	1.00336544	0.9446665	1.11911985	1.2308237	0.72798281	1.1081773	1.36359673	0.62373012	[illegible]
ENSG00000181552	0	0	0	0	0	0	0.02888716	0.03523358	0	0	[illegible]
ENSG00000181555	3.30891764	4.22945314	3.43900294	3.3417078	3.1021916	3.57819533	2.53787887	2.88625736	3.12817319	2.12301106	[illegible]
ENSG00000181562	0	0	0	0	0	0	0	0	0	0	[illegible]
ENSG00000181577	0.04061522	0.01883404	0.00611092	0.0121154	0.07668196	0.03515364	0.01505403	0.00612045	0.01081221	0.01980791	[illegible]
ENSG00000181585	0.01766981	0.02367104	0	0	0.0098566	0.01325457	0.02838036	0.10384634	0.03494321	0.01600395	[illegible]
ENSG00000181588	6.54040644	4.36674518	4.46222104	3.7011143	5.25968608	3.21064691	2.95978649	3.91156805	4.98475025	3.05165168	[illegible]
ENSG00000181609	0	0	0	0	0	0	0	0	0	0	[illegible]
ENSG00000181610	9.69377777	8.96658734	9.59609563	9.1338374	11.9173982	7.75986481	17.8889475	19.7206599	21.7857784	18.9319391	[illegible]
ENSG00000181616	0	0.0610405	0	0.1177965	0.05083441	0.13671817	0	0	0	0	[illegible]
ENSG00000181617	0	0	0	0	0	0	0.04572949	0.16732838	0.16891278	0.10314914	[illegible]
ENSG00000181619	0.52071088	0.65510058	0.44282252	0.5150522	0.52283451	0.53325143	0.40362477	0.38585669	0.29101345	0.40190197	[illegible]
ENSG00000181625	0.01924164	0.00429612	0.00627266	0	0	0	0.00515082	0	0.00634193	0.01161839	[illegible]
ENSG00000181626	0.00572159	0.01149724	0	0.0110937	0.00319163	0.02360551	0.01148717	0.00280217	0.01131483	0.00777327	[illegible]
ENSG00000181631	0	0	0	0	0	0	0	0	0	0	[illegible]
ENSG00000181634	0.02380838	0.01366905	0.00997893	0.0131893	0.01328082	0.00765397	3.36510381	2.54193392	0.46241864	6.14875978	[illegible]
ENSG00000181638	0.52678095	0.64370596	0.53954904	0.5935097	0.40106503	0.42986074	2.22670054	2.18248189	1.53094111	1.74728148	[illegible]
ENSG00000181649	0.16874209	0.02260523	0.03300535	0.0436238	0.16943028	0.02531553	9.89241447	8.92534811	10.4280795	11.095715	[illegible]
ENSG00000181652	0.02087689	0.00489428	0.00204172	0.0067465	0.02794937	0.01252822	0.00167657	0	0.00464461	0.0198541	[illegible]
ENSG00000181656	0.04616119	0.04328729	0.00902896	0.0596687	0.05664932	0.08310397	0	0	0	0.00836185	[illegible]
ENSG00000181666	0.99514554	1.22799219	1.06943219	1.2863418	0.91409835	1.10912836	1.29750605	1.30172059	1.15625733	1.48609777	[illegible]
ENSG00000181689	0	0	0	0	0	0	0	0	0	0	[illegible]
ENSG00000181690	2.90241899	4.14397331	3.62050547	3.4524421	2.56169066	3.85840108	2.14876121	2.1532484	2.0627132	1.94132439	[illegible]
ENSG00000181693	0	0	0	0	0	0	0	0	0	0	[illegible]
ENSG00000181698	0	0	0	0	0	0	0	0	0	0	[illegible]
ENSG00000181704	5.61489443	6.02245025	5.60181218	5.7650331	4.86744608	5.10770118	4.96305848	5.65147038	4.21556465	3.28723785	[illegible]
ENSG00000181705	0	0	0	0	0	0	0	0	0	0	[illegible]
ENSG00000181718	0	0	0	0	0	0	0	0	0	0	[illegible]
ENSG00000181722	3.61950031	4.28307975	4.25272135	3.6025569	1.8838997	2.81171974	0.58099219	0.57165335	0.32609687	0.34607771	[illegible]
ENSG00000181733	0	0	0	0	0	0	0	0	0	0	[illegible]
ENSG00000181741	0	0	0	0	0	0	0	0	0	0	[illegible]
ENSG00000181744	17.4485143	20.8526553	17.6130857	17.249423	14.5793163	16.3256307	1.93523058	1.9435423	2.08255676	2.61223242	[illegible]
ENSG00000181751	3.89913728	6.44639401	4.95226559	5.3764639	3.3600993	4.79686535	1.89453867	1.8344033	2.02471757	1.99373427	[illegible]
ENSG00000181752	0	0	0	0	0	0	0	0	0	0	[illegible]
ENSG00000181754	0.71797835	1.38373207	1.051101	2.2376187	0.57004195	1.69832353	0.22285393	0.27181428	0.23954511	0.4228875	[illegible]
ENSG00000181761	0	0	0	0	0	0	0	0	0	0	[illegible]
ENSG00000181767	0	0	0	0	0	0	0	0	0	0	[illegible]
ENSG00000181773	1.69148989	0.62263235	0.84947805	0.6894206	2.22711583	0.68585332	0.8076931	1.17918376	1.08487527	1.13176308	[illegible]
ENSG00000181778	0	0	0	0	0	0	0	0	0	0	[illegible]
ENSG00000181780	0	0	0	0	0	0	0	0	0	0	[illegible]
ENSG00000181781	0	0	0.00914421	0	0	0.02104119	0.08259699	0.00915848	0.04622601	0.09315442	[illegible]
ENSG00000181785	0	0	0	0	0	0	0	0	0	0	[illegible]
ENSG00000181786	0	0	0	0	0	0	0	0	0	0	[illegible]
ENSG00000181788	27.588885	20.2721148	24.0484677	28.718405	28.2382133	31.195723	8.64287405	9.96533436	11.4673009	8.19176077	[illegible]
ENSG00000181789	28.99013	27.1418691	24.5841418	50.371611	31.240441	29.5773078	17.3560464	17.9215665	17.6064434	17.1425312	[illegible]
ENSG00000181790	2.29352463	2.05335902	1.73819789	0.9045679	2.53692709	1.315158	0.00483431	0.01179279	0.00892834	0.04770696	[illegible]
ENSG00000181791	0	0	0	0	0	0	0	0	0	0	[illegible]
ENSG00000181798	0.35196284	0.63989364	0.66384	0.4874497	0.40668847	0.60346532	0.34322089	0.41862549	0.18643649	0.22770052	[illegible]
ENSG00000181800	0	0	0	0.0168803	0	0.00979592	0.0104874	0	0	0.01182788	[illegible]
ENSG00000181803	0	0	0	0	0	0	0	0	0	0	[illegible]
ENSG00000181804	2.12637803	4.05263199	3.29306807	4.2085672	1.48193418	3.53615611	1.33289658	0.92453653	0.57413114	0.90291984	[illegible]
ENSG00000181817	5.16932468	3.08946431	4.66447302	5.3714064	6.66723531	4.37037766	3.85319093	4.39200535	5.21017715	3.75075313	[illegible]
ENSG00000181819	0	0	0	0	0	0	0	0	0.01572962	0	[illegible]
ENSG00000181826	12.3047106	7.29620221	10.0494915	7.4571086	12.361152	6.82435987	1.70450149	2.28046197	2.65337294	4.12420001	[illegible]
ENSG00000181827	1.02919885	1.59852481	1.33666399	1.2037314	1.28318636	1.42895945	0.75069583	0.90752884	1.05499918	1.24110999	[illegible]
ENSG00000181830	0.67177839	0.55251861	0.51947825	0.7269924	0.72680634	0.63985826	1.2872442	1.34356926	1.09213888	1.36121863	[illegible]
ENSG00000181837	0	0	0	0	0	0	0	0	0	0	[illegible]
ENSG00000181847	0.03006251	0.06407018	0.03474615	0.0353266	0.01676951	0.06560184	0.00219477	0.00267695	0	0	[illegible]
ENSG00000181852	3.46785411	3.07307068	3.27766151	3.4322173	3.04928959	3.07908413	5.7797778	6.11809552	5.36384879	4.54328553	[illegible]
ENSG00000181856	0.01903224	0.01821157	0.02393124	0.0316304	0.01668321	0.01835557	0.07642157	0.09321116	0.0766192	0.10589015	[illegible]
ENSG00000181867	0	0	0	0	0	0	0	0	0	0	[illegible]
ENSG00000181873	0.49691511	0.50685355	0.52178267	0.5070954	0.44642151	0.48653473	0.34865244	0.45428349	0.41376092	0.42637953	[illegible]
ENSG00000181885	0	0.0026547	0	0	0	0	0.00318284	0.00388211	0.00783773	0.00358967	[illegible]
ENSG00000181894	2.56337638	2.21464524	2.42749913	2.8379057	2.23481102	2.64145797	1.0187396	1.09981208	1.08660418	1.54924792	[illegible]
ENSG00000181896	0.90711673	0.66915038	0.74427915	0.6913502	0.76229931	0.76705366	0.82309074	0.89775953	0.86432533	0.77678186	[illegible]
ENSG00000181903	0.03046289	0.06121358	0.11916865	0	0.1019571	0.04570195	0	0	0	0	[illegible]
ENSG00000181904	5.0823732	5.61986774	5.51899734	4.5163874	4.82639108	4.18831068	3.70678614	3.70383545	3.14227242	2.42756911	[illegible]
ENSG00000181908	0.01379107	0.02771244	0.04046227	0.0713063	0.00769295	0.1137953	0.02215053	0.01350847	0.00681819	0.06245444	[illegible]
ENSG00000181915	22.9062292	18.0160856	19.2016121	15.778026	19.3160731	15.5396068	13.5706168	14.8137373	13.5630352	10.3576641	[illegible]
ENSG00000181924	8.74063122	7.20236922	7.78756675	7.1294494	8.06780779	6.34247295	12.2624287	14.0269114	13.7081988	11.516212	[illegible]
ENSG00000181927	0	0	0	0	0	0	0	0	0	0	[illegible]
ENSG00000181929	2.4314911	2.40294473	2.71089786	3.1468978	2.57172393	2.6184119	3.95272756	4.29796403	4.25113945	4.30036173	[illegible]
ENSG00000181935	0	0	0	0	0	0	0	0	0	0	[illegible]
ENSG00000181938	1.81591223	1.21994681	1.31344789	0.8592716	1.74101555	1.01148557	0.36457835	0.60878142	0.73745504	0.3230686	[illegible]
ENSG00000181939	0	0	0	0	0	0	0	0	0	0	[illegible]
ENSG00000181943	0	0	0	0	0	0	0	0	0	0	[illegible]
ENSG00000181950	0	0	0	0	0	0	0	0	0	0	[illegible]
ENSG00000181958	0	0	0	0	0	0	0	0	0	0	[illegible]
ENSG00000181961	6.20146704	3.2751438	3.29488626	0.8863979	2.14543767	1.69972343	0	0	0	0	[illegible]
ENSG00000181963	0	0	0	0	0	0	0	0	0.02986711	0	[illegible]
ENSG00000181965	0	0	0	0	0	0	0	0	0	0	[illegible]
ENSG00000181977	0	0	0	0	0	0	0	0	0	0	[illegible]
ENSG00000181982	0.74829598	1.06253284	0.83551115	0.9066415	0.66649984	0.70508559	1.10854168	1.24224055	1.13505426	1.67682769	[illegible]
ENSG00000181984	0	0	0	0	0	0	0	0	0	0	[illegible]
ENSG00000181991	2.1754507	2.01091231	2.08633238	1.9026213	2.52287666	1.76318668	2.22872509	2.23249257	2.50207611	2.83367006	[illegible]
ENSG00000181995	0	0	0	0	0	0	0	0	0	0	[illegible]
ENSG00000181997	0	0	0	0	0	0	0	0	0	0	[illegible]
ENSG00000182000	0.15994908	0.10713643	0.07821366	0	0.17844601	0.11998178	0	0	0	0	[illegible]
ENSG00000182004	27.0045321	21.9802766	25.273362	26.247898	25.5128388	26.7436057	20.6063688	23.8786429	26.710664	15.7062282	[illegible]
ENSG00000182010	3.21011335	2.87141726	2.70538745	1.9329314	2.55307562	1.94643948	0.53260314	0.85215585	1.52721064	0.73543294	[illegible]
ENSG00000182013	7.57797726	9.39856303	8.07920585	10.568743	5.40820484	9.81884889	1.48905004	1.9699634	1.95085809	0.43041273	[illegible]
ENSG00000182021	0.02986386	0.02222587	0.03894176	0.0042892	0.0111058	0.01991255	0.01332384	0.02275147	0.02460739	0.0120215	[illegible]
ENSG00000182022	3.90325568	2.31234686	2.71211067	1.8740108	6.76420473	2.38695939	0.37615277	0.32285391	0.45088434	0.18854758	[illegible]
ENSG00000182035	0	0	0.01310607	0	0	0.01005253	0.02152424	0	0	0.01213772	[illegible]
ENSG00000182040	0.01231553	0.02887198	0.04215528	0.0238789	0.00343493	0.06928639	0.00494515	0	0	0.00557723	[illegible]
ENSG00000182048	0	0.00411357	0	0.0079384	0.01370308	0.00921354	0	0	0	0	[illegible]
ENSG00000182050	0.02423809	0.039428	0.04571567	0.0201411	0.00869177	0.01688291	0.00556143	0.00678326	0.00171187	0.00313614	[illegible]
ENSG00000182053	0	0	0	0	0	0	0	0	0	0	[illegible]
ENSG00000182054	22.0376606	15.8064658	18.7689113	17.789515	23.4650654	16.6529973	1.40874088	1.82064853	3.10717928	3.84750129	[illegible]
ENSG00000182057	0.02842129	0.02855554	0.05559102	0	0.039635	0.04263903	0.23965641	0.13919439	0.19671735	0.15445083	[illegible]
ENSG00000182070	0	0	0	0	0	0	0	0	0	0	[illegible]
ENSG00000182077	0	0	0	0	0	0	0.03070331	0.02496583	0.01260111	0	[illegible]
ENSG00000182083	0	0	0	0	0	0.02267948	0	0	0	0	[illegible]
ENSG00000182087	4.84985439	5.77402349	5.01695333	6.5446954	4.68341439	6.3731019	7.24744825	8.46001662	7.00246078	6.3008525	[illegible]
ENSG00000182093	2.89963899	3.16315811	3.10456458	2.5301636	2.69527917	2.36861976	1.67102639	1.69204565	1.98673742	1.66110231	[illegible]
ENSG00000182095	7.25475068	10.3845611	7.35146222	7.1074017	5.7858641	6.90627381	1.71835078	1.65637444	1.46751642	1.05132676	[illegible]
ENSG00000182103	25.5717083	37.8080935	30.2620225	26.560383	14.691116	25.5846592	0	0.03405533	0	0	[illegible]
ENSG00000182107	0	0	0	0	0	0	0	0	0.00170418	0	[illegible]

ENSG00000182108	4.28529501	3.61809898	3.87397959	5.0504874	4.31382002	4.30851904	3.91858319	4.36502709	4.12150326	[illegible]
ENSG00000182109	0.03397969	0.02655349	0.05538588	0.0366023	0.0221137	0.0679707	0.28197845	0.28290874	0.18479195	[illegible]
ENSG00000182111	0.09922568	0.05815506	0.13949633	0.1042119	0.07264713	0.12095143	0.03486247	0.03644713	0.03372622	[illegible]
ENSG00000182117	45.8139057	34.9082401	41.8671325	40.732095	50.8387154	37.3055214	80.1399391	79.9307475	83.9002493	[illegible]
ENSG00000182118	1.00966378	0.50056022	1.22068494	1.2023439	1.8847644	0.90049969	3.7541048	3.31734489	3.03824951	[illegible]
ENSG00000182132	3.10483709	3.93420788	3.91194302	1.4453274	2.79747048	1.92212428	0.05344944	0.0521537	0.02961424	[illegible]
ENSG00000182134	1.48904343	1.72640386	1.54292521	1.7327703	1.02696118	1.52611792	0.37488196	0.32153677	0.33136917	[illegible]
ENSG00000182141	2.81301798	3.89863981	3.33770863	2.1068305	3.12447709	2.49490038	1.05312244	1.26017818	1.35391834	[illegible]
ENSG00000182149	4.0045587	4.07525751	3.65500603	4.1699247	3.53555969	4.27557263	1.94500852	1.93095902	1.84932376	[illegible]
ENSG00000182150	2.6613507	2.47918289	2.43734713	2.0199785	2.77574936	2.32242907	1.94868166	2.05094942	1.98882624	[illegible]
ENSG00000182154	17.6982644	12.0427459	14.5112377	16.241732	24.4176209	13.9272516	13.8643704	12.7577942	15.2017932	[illegible]
ENSG00000182156	0	0	0	0	0	0	0	0	0	[illegible]
ENSG00000182158	3.48443053	3.69399684	3.22541498	3.6141494	4.22565314	3.53845413	1.71847398	2.24451592	2.77568972	[illegible]
ENSG00000182162	0	0	0	0.0090468	0	0	0.05620606	0.0548435	0.07612385	[illegible]
ENSG00000182165	4.39087465	3.77277557	4.47018821	5.1756037	4.23110573	4.80556854	0.28903286	0.32609258	0.21352235	[illegible]
ENSG00000182168	0.01588259	0.01861721	0.04854027	0.0333614	0.02879385	0.02382785	1.34245384	1.62377446	1.45756724	[illegible]
ENSG00000182170	0	0	0	0	0	0	0	0	0	[illegible]
ENSG00000182173	1.19395116	1.13914661	1.04546552	0.9466302	1.1287344	1.16377549	1.03408929	1.32586925	1.23203615	[illegible]
ENSG00000182175	7.48403263	10.5352843	6.30029058	9.2194105	7.04680707	7.89870977	0.09094817	0.09458175	0.11080085	[illegible]
ENSG00000182177	0.02010171	0.01570852	0.01638259	0.0216532	0.01121316	0.02010506	0.01883371	0.02953468	0.00993811	[illegible]
[illegible]										

ENSG00000182831	10.6169191	10.4114095	10.5896545	12.128002	10.7031033	11.3794851	11.7696943	12.7221097	13.0245854	7.22302147	9.90368521	10.4382114	8.69298655	8.32276788	[illegible]
ENSG00000182836	0.31083662	0.11330792	0.67829648	0.1776634	0.19344334	0.0729636	0	0.00414241	0.00104541	0	0.00266394	0.00357145	0.0196874	0.00916303	[illegible]
ENSG00000182841	0.57674693	0.97651654	0.70506017	0.9474225	0.54513957	1.35197567	0.96815592	1.16516443	1.1385747	1.2841664	0.95870655	1.08574313	1.01022898	0.59010076	[illegible]
ENSG00000182851	0	0	0	0	0	0	0.03404151	0.08304065	0.01397116	0	0.01780085	0.01193248	0	0	[illegible]
ENSG00000182853	0.17967799	0.11282922	0.21965231	0.1741911	0.12528526	0.17689997	0.12625801	0.16499627	0.06107148	0.18308074	0.04244294	0.05690172	0.19960663	0.19465222	[illegible]
ENSG00000182854	0	0	0.06400022	0	0	0	0	0.22435026	0.06470703	0.08890723	0	0.02763248	0.027695	0.07089491	[illegible]
ENSG00000182858	3.38609631	3.49447036	3.09759348	3.6330936	3.06405378	3.58881324	2.70019258	3.0884517	2.57691893	1.71003525	2.24677652	2.03221238	2.62853192	2.39057165	[illegible]
ENSG00000182866	0.00437547	0.00146538	0	0.0028279	0	0.00492323	0.00175692	0	0.0010816	0.00990741	0.00275616	0.00184754	0	0.00237006	[illegible]
ENSG00000182870	0	0	0	0	0	0	0.00200069	0.00488046	0	0	0.00941573	0.00210389	0.01265188	0.03238687	[illegible]
ENSG00000182871	0	0.00085345	0.0024922	0	0.00071075	0.00477888	0.79608185	0.92979319	0.86741503	1.60756475	1.2103271	0.82100521	0.09274731	0.15873929	[illegible]
ENSG00000182872	10.6685488	10.0073883	8.81908282	8.7303408	10.1047603	8.40915805	5.56520918	6.31274597	7.33737368	5.43898817	5.71425705	6.97817345	6.87871634	4.59417651	[illegible]
ENSG00000182873	0.13250437	0.22449422	0.16007793	0.1712774	0.07826163	0.15786237	0.59464929	0.71765731	0.49324438	0.53652519	0.63826927	0.57266389	0.09236131	0.16887904	[illegible]
ENSG00000182885	0.01049316	0.01581409	0.00256553	0.0101727	0.00146333	0.01967795	0	0	0	0.00237597	0	0	0	0	[illegible]
ENSG00000182888	0	0	0	0	0	0	0.05726304	0	0	0	0.17966259	0.06021679	0	0	[illegible]
ENSG00000182890	0.04144416	0.01850664	0.02702109	0.0178571	0	0.06217659	0.85425749	0.86602416	0.62834878	0.86334901	1.07905367	0.53665991	0.01169292	0	[illegible]
ENSG00000182896	0.0108081	0.00723943	0.03171037	0.0977951	0.00602899	0.05675195	0	0	0	0.04894568	0.01361628	0	0.01829616	0.02341767	[illegible]
ENSG00000182898	0	0	0	0	0	0	0	0	0	0	0	0	0	0	[illegible]
ENSG00000182899	122.394455	114.988571	128.886719	184.65253	88.2641346	156.327714	64.9043762	74.8621714	70.4601397	51.736818	63.6065778	58.268982	42.9602475	44.0110912	[illegible]
ENSG00000182901	0.0290435	0.03433023	0.05012474	0.0022084	0.00667103	0.00897081	0.01783608	0.00502029	0.00929102	0.00464212	0.00860933	0.00577111	0.17063289	0.22950183	[illegible]
ENSG00000182902	0.63773819	0.74075228	0.56601358	0.7767007	0.85748684	0.66641832	0.02664372	0	0.003645	0	0.00928829	0.00622624	0.03432178	0.04792278	[illegible]
ENSG00000182903	2.96986692	3.56588213	3.04089028	3.2904855	3.06691904	3.31113915	1.18084426	1.2808745	1.61481693	1.54233585	1.93664965	2.43688258	1.62088524	1.72516692	[illegible]
ENSG00000182909	0.06735578	0.07895294	0.09880911	0.2394293	0.09393122	0.03789393	0.52739436	0.62676745	0.54112688	0.83882777	0.53035195	0.4123938	1.25423322	1.55059458	[illegible]
ENSG00000182912	0.00897764	0.03006684	0.0351199	0.034814	0.00500792	0.02693741	0.04325832	0.04396837	0.01775388	0.03252502	0.05655118	0.04548968	0.0227963	0.04862917	[illegible]
ENSG00000182916	16.8376166	10.3843661	14.5405716	6.9810935	8.68352209	4.81381817	10.422042	10.2067285	10.680327	15.2358243	10.3860738	12.0596223	21.0680582	20.9081449	[illegible]
ENSG00000182919	1.49202209	1.68213622	1.44067901	1.7330852	1.03555786	1.41834466	0.89175193	0.96307371	0.64161331	1.0041775	1.11524954	0.72290983	0.64452537	0.700177	[illegible]
ENSG00000182921	0	0	0	0	0	0	0	0	0	0.02014425	0	0	0	0	[illegible]
ENSG00000182923	2.2264676	1.95124024	[illegible]
ENSG00000182931	0	0	[illegible]
ENSG00000182934	6.20657208	5.47020575	[illegible]
ENSG00000182938	0	0	[illegible]
ENSG00000182944	6.49560548	6.77658968	[illegible]
ENSG00000182950	0.02694794	0.05415048	[illegible]
ENSG00000182952	38.107211	33.3823262	[illegible]
ENSG00000182957	1.56353174	1.76288951	[illegible]
ENSG00000182963	3.58663954	3.26478372	[illegible]
ENSG00000182965	0.07443358	0	[illegible]
ENSG00000182968	0.01569121	0.01576533	[illegible]
ENSG00000182973	2.39144248	2.38487838	[illegible]
ENSG00000182974	0	0	[illegible]
ENSG00000182979	2.96872204	3.32236517	[illegible]
ENSG00000182983	0.36684919	0.69484553	[illegible]
ENSG00000182985	11.6427666	8.15556211	[illegible]
ENSG00000182986	1.77557582	1.97594695	[illegible]
ENSG00000182993	0.30131309	0.24146834	[illegible]
ENSG00000183010	2.18558676	1.62969746	[illegible]
ENSG00000183011	5.99623066	4.07593515	[illegible]
ENSG00000183016	0.00722802	0.00968288	[illegible]
ENSG00000183018	0.00963232	0.02150627	[illegible]
ENSG00000183019	0	0	[illegible]
ENSG00000183020	2.34904912	2.48804326	[illegible]
ENSG00000183022	0.05012401	0	[illegible]
ENSG00000183023	1.61203412	1.35597625	[illegible]
ENSG00000183024	0	0	[illegible]
ENSG00000183032	0.24955984	0.25277723	[illegible]
ENSG00000183034	0	0	[illegible]
ENSG00000183035	0.04591131	0.01025071	[illegible]
ENSG00000183036	0.07336056	0.11465549	[illegible]
ENSG00000183038	0	0	[illegible]
ENSG00000183044	7.11103691	9.17241564	[illegible]
ENSG00000183048	0.17501653	0.10530727	[illegible]
ENSG00000183049	1.13380466	1.16771884	[illegible]
ENSG00000183054	0.00139874	0	[illegible]
ENSG00000183055	0.04578088	0.03066476	[illegible]
ENSG00000183060	0.15070687	0.12566728	[illegible]
ENSG00000183066	0.02505811	0.04028236	[illegible]
ENSG00000183067	0.32631356	0.52120538	[illegible]
ENSG00000183072	0	0	[illegible]
ENSG00000183077	0.53795344	0.57582105	[illegible]
ENSG00000183086	0	0	[illegible]
ENSG00000183087	0.94697804	0.79967609	[illegible]
ENSG00000183090	0	0	[illegible]
ENSG00000183091	0.05686822	0.08230205	[illegible]
ENSG00000183092	0.2889934	0.27367127	[illegible]
ENSG00000183098	4.33583943	6.08549664	[illegible]
ENSG00000183103	0	0	[illegible]
ENSG00000183111	1.8792696	1.59039375	[illegible]
ENSG00000183114	2.28517721	2.30015397	[illegible]
ENSG00000183117	0.02398467	0.0622531	[illegible]
ENSG00000183122	0	0	[illegible]
ENSG00000183128	0	0	[illegible]
ENSG00000183130	0	0	[illegible]
ENSG00000183134	0.0111329	0.01491398	[illegible]
ENSG00000183137	0.87959	0.69914841	[illegible]
ENSG00000183145	0.04209599	0.01057371	[illegible]
ENSG00000183146	0	0.02458769	[illegible]
ENSG00000183148	0.03027688	0.05069984	[illegible]
ENSG00000183150	2.40304167	2.33755312	[illegible]
ENSG00000183153	0	0	[illegible]
ENSG00000183154	0.57166844	0.48640247	[illegible]
ENSG00000183155	8.80680955	6.36978981	[illegible]
ENSG00000183160	0.01486955	0.04481936	[illegible]
ENSG00000183161	7.19787782	6.92198791	[illegible]
ENSG00000183166	0	0.00070402	[illegible]
ENSG00000183169	0	0	[illegible]
ENSG00000183171	0.03007909	0.01007372	[illegible]
ENSG00000183172	7.71898705	9.42585439	[illegible]
ENSG00000183185	0	0.00345851	[illegible]
ENSG00000183186	1.35244228	0.996476	[illegible]
ENSG00000183196	4.46660421	3.06018635	[illegible]
ENSG00000183199	0.38562965	0.30797409	[illegible]
ENSG00000183206	0.08931571	0.20163267	[illegible]
ENSG00000183207	6.47966956	4.68328303	[illegible]
ENSG00000183208	0.17301771	0.25959362	[illegible]
ENSG00000183230	0.30615796	0.37216308	[illegible]
ENSG00000183239	0	0.04744613	[illegible]
ENSG00000183242	0.01299979	0.00870747	[illegible]
ENSG00000183246	0.00572515	0.0019174	[illegible]
ENSG00000183248	0.11534339	0.19900002	[illegible]
ENSG00000183249	0	0	[illegible]
ENSG00000183250	0.29932238	0.3522137	[illegible]
ENSG00000183251	0	0.263536	[illegible]
ENSG00000183255	24.9325099	31.2076764	[illegible]
ENSG00000183258	3.24857069	2.58234616	[illegible]
ENSG00000183260	0	0	[illegible]
ENSG00000183269	0	0.02034683	[illegible]
ENSG00000183273	0	0	[illegible]
ENSG00000183281	0.10153383	0.1586876	[illegible]
ENSG00000183283	9.80033926	10.1579875	[illegible]
ENSG00000183287	0.02559439	0.00642882	[illegible]
ENSG00000183291	12.4132964	11.1794951	[illegible]
ENSG00000183292	0	0.03301477	[illegible]
ENSG00000183298	0.1465821	0.17182028	[illegible]
ENSG00000183303	0	0	[illegible]
ENSG00000183304	0	0	[illegible]
ENSG00000183305	0	0	[illegible]
ENSG00000183307	1.39909246	1.76045644	[illegible]
ENSG00000183308	0.51863949	0.64515834	[illegible]
ENSG00000183309	3.99229773	4.32828524	[illegible]
ENSG00000183310	0	0	[illegible]
ENSG00000183313	0	0	[illegible]
ENSG00000183317	0.0076631	0.01283217	[illegible]
ENSG00000183318	0	0	[illegible]
ENSG00000183323	0.06996579	0.14293579	[illegible]
ENSG00000183324	0	0	[illegible]
ENSG00000183336	0	0	[illegible]
ENSG00000183337	1.35228595	1.42811027	[illegible]
ENSG00000183340	2.04783293	1.60078522	[illegible]
ENSG00000183346	1.52999797	2.81824645	[illegible]
ENSG00000183347	0.06373292	0.07257185	[illegible]
ENSG00000183354	2.86642911	3.8088787	[illegible]
ENSG00000183378	0	0	[illegible]
ENSG00000183379	0	0.01174324	[illegible]
ENSG00000183385	0	0	[illegible]
ENSG00000183386	1.78834026	1.56176508	[illegible]
ENSG00000183389	0	0	[illegible]
ENSG00000183395	0	0	[illegible]
ENSG00000183396	0	0	[illegible]
ENSG00000183397	2.05620002	1.69757196	[illegible]
ENSG00000183401	0.20137993	0.28626119	[illegible]
ENSG00000183405	0.47088906	0.33293167	[illegible]
ENSG00000183421	0	0.00333251	[illegible]
ENSG00000183423	0	0.00308709	[illegible]
ENSG00000183426	0.34087763	0.49845651	[illegible]
ENSG00000183431	8.21453468	8.04505216	[illegible]
ENSG00000183432	0	0	[illegible]
ENSG00000183434	0	0	[illegible]
ENSG00000183439	0.02037278	0.01364601	[illegible]
ENSG00000183444	2.06139452	2.71767597	[illegible]
ENSG00000183454	0	0	[illegible]
ENSG00000183458	0.16011936	0.21231219	[illegible]
ENSG00000183461	0	0	[illegible]
ENSG00000183463	0	0	[illegible]
ENSG00000183470	0	0	[illegible]
ENSG00000183473	0	0	[illegible]
ENSG00000183474	0.63038933	0.74678	[illegible]
ENSG00000183475	1.85711031	2.11741121	[illegible]
ENSG00000183476	0	0	[illegible]
ENSG00000183479	0.00265178	0.00355241	[illegible]
ENSG00000183484	0.00450608	0.00452737	[illegible]
ENSG00000183486	0.01591878	0.01260131	[illegible]
ENSG00000183495	0.47059187	0.65798298	[illegible]
ENSG00000183496	2.39027328	2.55397929	[illegible]
ENSG00000183504	0	0	[illegible]
ENSG00000183506	0.14658766	0.28510519	[illegible]
ENSG00000183508	0.03362518	0.0375378	[illegible]
ENSG00000183513	10.7313625	13.2059279	[illegible]
ENSG00000183514	0	0	[illegible]
ENSG00000183520	5.15190703	4.28439669	[illegible]
ENSG00000183527	3.65340755	2.28999245	[illegible]
ENSG00000183530	1.89337988	2.22150182	[illegible]
ENSG00000183531	0	0	[illegible]

ENSG00000183535	0	0	0	0	0	0	0	0	0.00276346	0	0	0	0	0	0	0	0.00840882	[illegible]
ENSG00000183542	0	0.09305168	0.06793126	0	0.01937331	0.02605208	0.02789105	0.03401863	0	0	0	0	0.91127882	0.82774387	0.81553075	0.53605658	1.0971873	[illegible]
ENSG00000183549	0	0	0	0	0	0	0.10633289	0.04472204	0.01354365	0	0.07477669	0.01927893	0.00386451	0	0.00794166	0	0	[illegible]
ENSG00000183558	1.42287352	1.30769142	1.45626776	2.7802317	1.10750113	2.87931644	1.78044539	1.16683196	0.62165915	0.38961569	2.54294503	1.14572651	1.82050535	2.07917147	1.43891652	1.34069183	1.3440464	[illegible]
ENSG00000183559	0	0.01275886	0	0	0	0	0	0	0	0	0	0	0	0	0	0	0	[illegible]
ENSG00000183560	0	0	0	0	0	0.0174181	0	0	0	0	0	0	0	0	0	0	0	[illegible]
ENSG00000183562	0	0	0	0	0	0	0	0	0	0	0	0	0	0	0	0	0	[illegible]
ENSG00000183566	0	0	0	0	0	0	0	0	0	0	0	0	0	0	0	0	0	[illegible]
ENSG00000183569	0.12165226	0.1177	0.1612755	0.1607442	0.15532451	0.1642579	0.01519714	0.02647986	0.022721	0.07590399	0.03405783	0.01369803	0.0549161	0.07614572	0.09874718	0.03390259	0.05693619	[illegible]
ENSG00000183570	0	0	0	0	0	0	0.31602315	0.27054402	0.21951242	0.1344969	0.23572023	0.25457299	0.00377067	0.00321745	0.00774883	0.00429754	0.00893572	[illegible]
ENSG00000183571	0.01300635	0	0	0	0	0	0.01044507	0	0	0	0	0	0	0.01409029	0	0.01254693	0.01956627	[illegible]
ENSG00000183576	3.61165707	3.0807073	3.32789661	3.3568655	3.77303183	3.24918956	2.05989784	2.22847687	2.35889867	2.04503114	2.38026224	2.32591611	4.51678849	4.65259309	4.78208186	5.47498561	5.32236089	[illegible]
ENSG00000183578	0.01424834	0.0763501	0	0.0368353	0.06358421	0.08550426	0.7323188	0.69781801	1.21161191	3.71665226	0.89751868	0.92651869	0.07235961	0.0926147	0.06195865	0.08247024	0.04286935	[illegible]
ENSG00000183579	1.402403	1.25740897	1.11186731	1.1813802	1.13546012	1.05325404	0.72166485	0.84853815	0.86330423	0.5040674	0.68183931	0.7675137	0.48114153	0.36322549	0.38780612	0.50732461	0.37893087	[illegible]
ENSG00000183580	5.04212682	4.4038388	4.25683914	3.8171082	4.76567536	4.22151112	1.24744869	1.41980427	1.27719247	1.08714673	1.40229429	1.49067603	2.1514302	2.2047311	2.4237362	1.98727781	2.31179573	[illegible]
ENSG00000183586	0	0	0	0	0.09315251	0	0	0	0	0	0	0	0	0	0	0	0	[illegible]
ENSG00000183597	0.76054745	0.68084195	0.84331036	0.8768157	0.67879929	0.65608139	0.30868975	0.30402515	0.32773679	0.25133536	0.34830216	0.3037819	0.4828012	0.46206935	0.43887803	0.46202166	0.38807974	[illegible]
ENSG00000183598	50.8147722	33.5638102	37.1185684	49.769841	47.1551166	40.529175	8.56465547	15.9766707	21.5556269	10.1565511	8.29857566	23.1783375	2.65494622	1.44420342	1.36399483	2.11814086	1.65156499	[illegible]
ENSG00000183604	0.0496752	0.10298858	0.05783506	0.0794993	0.05410024	0.09581811	0.05129087	0.04634024	0.04677903	0.05998927	0.01788055	0.03795536	0.03003256	0.01793836	0.03703058	0.04335665	0.04981969	[illegible]
ENSG00000183605	2.18486548	2.03184212	1.84591176	2.8805751	2.14776826	2.51638468	2.20361242	2.55956693	2.46617922	2.97730192	2.30793926	2.37085844	0.84577415	0.68302201	0.6172966	1.23936705	0.84705986	[illegible]
ENSG00000183607	0	0	0	0	0	0	0	0	0	0	0	0	0	0	0	0	0	[illegible]
ENSG00000183615	0.17216741	0.23064092	0.40410393	0.2225465	0.11524638	0.28412353	0.35948462	0.06745574	0.08511808	0.24949749	0.13014021	0	0.43717264	0.522244	0.56898695	0.365389	0.20720183	[illegible]
ENSG00000183617	8.48302909	6.49379125	8.07050179	11.189087	9.5284096	9.00389707	12.6749707	14.1312833	15.4062971	8.33661128	12.1411378	13.4262425	10.452692	10.5653721	11.8193144	9.38027037	7.76979055	[illegible]
ENSG00000183621	0.12905934	0.07658812	0.09078818	0.0848755	0.09409487	0.08746924	0.16001226	0.15191936	0.17014887	0.3219656	0.19396921	0.17782663	0.29129896	0.28821523	0.23433076	0.31780403	0.28100962	[illegible]
ENSG00000183624	6.27158639	5.18685621	5.49920243	5.2368329	6.45500531	5.55078023	1.7951351	2.00632214	2.00444007	1.31693761	1.64558421	2.08898843	2.25968005	2.26148382	2.68127629	1.86245043	1.88331806	[illegible]
ENSG00000183625	0	0	0	0	0.00504257	0.00226032	0.00483973	0.00590301	0	0	0	0.00254469	0	0.00326437	0	0	0	[illegible]
ENSG00000183628	0.38487526	0.39146732	0.45446947	0.6412174	0.41029953	0.53036087	0.37318987	0.40770596	0.35095907	0.23497565	0.38789956	0.27928235	0.13995712	0.12971793	0.13636932	0.09350761	0.06004363	[illegible]
ENSG00000183629	0	0.00074974	0	0.0014469	0	0.00167926	0.00898899	0.00328915	0.00166015	0.00304138	0.00141015	0.0028358	0	0.00242521	0.00486735	0.00323935	0	[illegible]
ENSG00000183631	0.89235436	0.79109086	0.92404221	0.1696283	0.453853	0.51187668	0	0	0	0	0	0	0	0	0	0	0	[illegible]
ENSG00000183632	0	0	0	0.0045367	0	0.00789818	0	0	0	0	0	0	0	0	0	0	0	[illegible]
ENSG00000183638	0.02033636	0.0295135	0.00662953	0.0175247	0.00567203	0.01271234	0.03266324	0.00995981	0.03351374	0.02762865	0.04270033	0.03148574	0.05737632	0.06976539	0.04421615	0.0523147	0.03569214	[illegible]
ENSG00000183640	0	0	0	0	0	0	0	0	0	0	0	0	0	0	0	0	0	[illegible]
ENSG00000183643	0	0	0	0	0	0	0	0	0	0	0	0	0	0	0	0	0	[illegible]
ENSG00000183644	0.00616247	0.0536604	0.01205358	0.0159314	0.01031268	0.01386788	0.00989786	0.03621716	0.01828004	0.00558149	0.02329087	0.01040841	0	0.0133521	0.01607845	0.01188959	0.0278118	[illegible]
ENSG00000183647	1.19989008	0.7785896	0.93369924	1.0402126	1.19062484	1.13157511	0.64626159	0.69218501	0.79855907	0.65833041	0.52689664	0.81600384	0.86179426	0.51558058	0.67228075	0.94603865	0.72463158	[illegible]
ENSG00000183648	9.68557586	7.90248444	10.0037978	9.120714	11.9644358	9.54692147	4.73733662	4.44734582	3.88710704	3.6201224	5.22316331	4.19706301	5.92034235	5.99179573	6.54060932	5.58915892	6.09197061	[illegible]
ENSG00000183653	0	0	0	0	0	0	0	0	0	0	0	0	0	0	0	0	0	[illegible]
ENSG00000183654	0	0	0	0	0	0	0	0	0	0	0	0	0	0	0.01003225	0	0	[illegible]
ENSG00000183655	4.84850263	5.11483785	4.44690382	4.9860451	4.33795401	4.73365342	0.30844554	0.47734186	0.53698997	0.40771931	0.45785874	0.61034705	0.77951623	0.48320093	0.4741123	0.63345897	0.4597523	[illegible]
ENSG00000183657	0.0776353	0.08171641	0.16269842	0.1576971	0.11754655	0.17470851	0.20485428	0.07604439	0.07676444	0.1004515	0.22355813	0.11239363	0.31916912	0.44455693	0.37617813	0.17118393	0.30032145	[illegible]
ENSG00000183662	0.0140772	0.00471456	0	0	0.00785256	0	0	0	0	0	0	0.00594409	0	0	0	0	0.01058861	[illegible]
ENSG00000183663	0	0	0	0	0	0	0.05431877	0	0	0	0	0.02856033	0	0	0	0	0	[illegible]
ENSG00000183665	2.95521371	3.07114509	2.88138791	2.616093	3.17207731	2.55937246	12.0748477	12.2189543	11.0064424	8.84090437	10.8758017	10.3230223	4.13097166	4.21045619	4.1044344	2.99768681	2.61354112	[illegible]
ENSG00000183666	0.9711263	1.34778584	1.0751103	1.2804	0.68256081	1.23547832	0.72061567	0.71532191	0.74706123	0.53126096	0.60682951	0.76106711	0.67072831	0.77010735	0.63850641	0.61455589	0.66033879	[illegible]
ENSG00000183668	0	0	0	0	0	0	0	0	0	0.00207427	0	0	0.00775373	0.00992418	0.01195058	0.03093	0.00689053	[illegible]
ENSG00000183671	0.24945619	0.27569802	0.2520958	0.1236063	0.07189519	0.1153927	0.71785628	0.45610941	0.47481661	0.23723504	0.28284343	0.2422654	0.89383532	0.88280255	0.84610964	0.6296867	0.48785447	[illegible]
ENSG00000183674	0	0	0	0	0.00324872	0	0.00467707	0.00570461	0	0.00527488	0	0	0	0.00630931	0.00506507	0.00561823	0	[illegible]
ENSG00000183675	0.00368593	0.00246889	0.00504668	0.0066703	0.00328974	0.00165894	0.00118403	0.00072208	0.00328012	0	0	0	0	0	0	0	0	[illegible]
ENSG00000183678	0	0	0	0	0	0	0	0	0	0	0	0	0	0	0	0	0	[illegible]
ENSG00000183682	0.15238648	0.16586518	0.13040233	0.1969773	0.32939213	0.1285975	0	0	0.00941732	0.01725249	0	0.01608628	0.01612268	0.02063578	0	0	0.05731115	[illegible]
ENSG00000183684	27.7982687	19.8348761	22.3440686	16.488405	27.7345427	16.0821142	15.0850749	17.6032787	24.7536866	17.1330387	14.8678035	22.893115	22.5050475	12.7748008	15.6297972	20.5178762	15.1240938	[illegible]
ENSG00000183688	16.1282211	15.0776106	14.9287442	13.978943	16.1512977	14.1546032	1.25804725	1.90932721	3.67879375	2.41043101	1.56987692	2.81124786	3.45044026	2.35278678	3.09639257	5.62407889	3.99022324	[illegible]
ENSG00000183690	0.53063148	0.68897842	0.71055965	0.4643037	0.68307124	0.50214261	1.03584018	1.17545217	0.68208304	0.99817955	1.4912723	0.66183564	0.24184018	0.34491234	0.38339034	0.34650871	0.307024	[illegible]
ENSG00000183691	0.03406949	0	0.04997903	0.0220194	0	0.01277819	0.3967251	0.20022803	0.62321554	0.49372019	0.30045055	0.20140162	4.1957482	5.70241233	5.05193551	3.02367647	5.63781736	[illegible]
ENSG00000183695	0	0	0	0	0	0	0	0	0	0	0	0	0	0	0	0	0	[illegible]
ENSG00000183696	2.0183615	1.25573547	1.47664497	0.8780181	1.79708569	0.93898552	0.0966303	0.09885006	0.13048945	0.04921742	0.30806719	0.37695778	2.63810401	1.57054609	1.67265749	3.03293049	1.95610909	[illegible]
ENSG00000183704	0	0	0	0	0	0	0	0	0	0	0	0	0	0	0	0	0	[illegible]
ENSG00000183706	0	0	0	0	0	0	0	0	0	0	0	0	0	0	0	0	0	[illegible]
ENSG00000183709	0	0	0	0	0	0	0	0	0	0	0	0	0	0	0	0	0	[illegible]
ENSG00000183715	0	0.00733869	0.00357168	0.0023604	0.00203722	0.01095811	0	0	0.00090278	0.03638564	0	0.00308419	0.01545585	0.0138476	0.0222335	0.00704618	0.01098815	[illegible]
ENSG00000183718	1.60222557	2.5589213	1.80697163	2.0112063	1.33288397	2.67120651	1.55074227	1.39703166	1.1561384	1.09467023	1.1842759	1.11061916	2.93568962	2.26751655	2.61775301	2.30504331	2.438194	[illegible]
ENSG00000183722	54.4349713	54.789239	55.4438182	45.213923	60.0054887	48.6458772	3.71636213	4.21170845	3.67806034	4.17996146	2.41462022	3.34368117	8.08995146	7.22630174	7.15008652	6.05769108	5.72869298	[illegible]
ENSG00000183723	3.24775114	2.99055823	2.91729657	3.0708597	2.95259345	3.04299594	1.84348308	1.96296817	2.18091163	2.05931331	2.5124868	2.20935905	2.22875012	2.28683896	2.32175709	2.04806907	2.02082694	[illegible]
ENSG00000183726	7.93956726	8.22655287	8.63160322	6.6427978	7.28763069	6.40835413	7.58009817	8.57579954	7.40530326	6.76402439	7.85227489	7.58488476	12.0714902	12.3706738	12.6947294	12.2982484	11.4546	[illegible]
ENSG00000183729	0	0	0	0	0	0	0.02889791	0	0.02965036	0.01086387	0.04533355	0.04051804	0	0	0	0	0	[illegible]
ENSG00000183733	0	0	0	0	0	0	0	0	0	0	0	0	0	0	0	0	0	[illegible]
ENSG00000183734	0	0	0	0	0	0	0	0	0	0	0	0	0	0	0	0	0	[illegible]
ENSG00000183735	4.31765756	3.83926363	3.95609502	3.4373116	4.66233759	3.91896185	5.13507535	6.06995471	5.9462551	5.75739097	6.47529115	6.87988277	6.15977518	6.62686522	6.67655587	7.69123986	7.56963274	[illegible]
ENSG00000183741	4.32582053	3.74034506	3.68910497	3.7410053	4.38776138	3.79446294	12.3980089	14.4452905	11.5697144	11.8831459	11.977506	10.6902519	10.8659133	8.48204722	8.58947521	6.4847685	5.33817074	[illegible]
ENSG00000183742	0.01841582	0.07401127	0.04202414	0.0370294	0.01940406	0.05372177	0.04601111	0.04409398	0.0364185	0.02038621	0.05671274	0.05011248	0.08832825	0.11527005	0.0871992	0.02960886	0.04925171	[illegible]
ENSG00000183747	0.00111723	0.00074834	0.00109263	0.0014441	0	0.00083806	0.00089722	0	0.00055235	0.00202379	0.00140751	0	0.00378253	0.00121033	0.00097165	0.00107776	0.00168071	[illegible]
ENSG00000183748	0	0	0	0	0	0	0	0	0	0	0	0	0	0	0	0	0	[illegible]
ENSG00000183751	1.57822684	1.44880957	1.46056342	2.0203727	1.4806198	1.53609215	2.66929333	2.80644274	2.79647527	1.53181967	2.17725465	1.87254489	1.81606219	1.5425476	1.69398112	1.83704053	1.57954713	[illegible]
ENSG00000183753	0	0	0	0	0	0	0	0	0	0	0	0	0	0	0	0	0	[illegible]
ENSG00000183760	0.00416513	0.00557973	0.00407342	0.0161517	0.00697019	0.00624873	0.32445601	0.35086052	0.23886744	0.75071725	0.22563467	0.16531992	2.05178485	2.57649113	2.66608284	2.9893918	2.97001792	[illegible]
ENSG00000183762	1.59791808	1.2150121	1.31304676	1.9639518	1.05568032	1.6553259	0.36131669	0.48791442	0.38661302	0.42690441	0.56231644	0.35432239	1.97056074	2.16627689	2.28253035	1.19184396	1.29995851	[illegible]
ENSG00000183763	1.93895493	1.37422523	1.44245655	1.3624129	1.57893701	1.33014564	0.73198226	0.99019256	1.34532092	0.49832787	0.57206017	1.07763538	1.26803443	0.62477777	0.96566037	0.98798911	0.88254685	[illegible]
ENSG00000183765	0.51337299	0.35860245	0.38462115	0.3057275	0.43569208	0.36446382	0.50135819	0.54775052	0.56653389	0.47410697	0.54628016	0.59070247	0.53927533	0.58595123	0.62028651	0.57217793	0.63714697	[illegible]
ENSG00000183770	0	0	0	0	0	0	0	0	0	0	0	0	1.47760454	1.63985485	1.83536096	1.59879749	1.313107	[illegible]
ENSG00000183773	0.00244526	0.00327575	0.00478284	0.0063216	0	0.0036685	0.02454658	0.01676606	0.0157159	0.09744798	0.04158799	0.01755267	0.00206969	0.00529809	0.00212663	0.00235888	0.00183928	[illegible]
ENSG00000183775	0.92945631	0.84026305	0.86053787	1.0835933	2.27839603	1.27103004	0.13209617	0.12035269	0.08426078	0.16513503	0.0449405	0.05188205	0.03019322	0.04079193	0.0517065	0.0191178	0.03875718	[illegible]
ENSG00000183778	0.12211055	0.29325275	0.16456924	0.2021149	0.20268623	0.21559079	0.16264258	0.12544281	0.13472907	0.03371899	0.03752137	0.05659149	0.01134391	0.00322652	0.00259022	0.01005586	0.00672068	[illegible]
ENSG00000183779	10.1975783	12.5193575	8.64519208	9.8955059	5.72602681	9.96813103	0.66505192	0.78286549	0.81408187	0.54946101	0.57017509	0.62616829	0.84764731	0.7093727	0.69509943	0.88248335	0.55047467	[illegible]
ENSG00000183780	0	0	0.00557582	0	0	0	0.00915722	0	0.0056374	0	0	0.00481479	0	0.03088249	0.03966757	0	0	[illegible]
ENSG00000183783	1.478913	1.22409779	1.93191083	0.2867502	0.28873921	0.28526642	0.05090048	0.01034719	0.04178068	0.0191355	0.03992502	0.09813107	0.00894119	0	0	0.01019052	0	[illegible]
ENSG00000183784	0.08097925	0.10073362	0.05656875	0.1196287	0.03871881	0.09545572	0.06503239	0.10198262	0.05719349	0.0523891	0.07287104	0.13677382	0.09791663	0.03759771	0.09054948	0.02231966	0.05220951	[illegible]
ENSG00000183785	0.01788306	0.03294048	0.01748931	0	0.01246944	0.03018268	0.03949394	0.04379149	0.03315461	0.03239418	0.03379421	0.03020446	0.0227046	0.00484335	0.00777642	0.00431284	0.02690261	[illegible]
ENSG00000183791	0	0	0	0	0	0	0	0	0	0	0	0	0	0	0	0	0	[illegible]
ENSG00000183793	0.18585957	0.2999118	0.14871872	0.1528833	0.17907747	0.22180119	0.00678451	0.00827504	0.01670679	0.03825846	0.02128637	0.00713447	0.02145183	0.01830445	0.02204201	0.00814975	0.0127091	[illegible]
ENSG00000183795	0	0	0	0	0	0	0	0	0	0	0	0	0	0	0	0	0	[illegible]
ENSG00000183798	0.04245224	0.1251148	0.10794557	0.0548745	0.03315307	0.08916454	0	0.01663292	0.02518563	0.01537998	0.01069646	0.02151053	0.29464238	0.13797053	0.14768226	0.13104861	0.17881802	[illegible]
ENSG00000183801	0.00832487	0.00278807	0	0	0	0.00312235	0	0	0	0.00754002	0	0.00351517	0	0	0.00724011	0	0	[illegible]
ENSG00000183807	0.03120014	0	0.03051317	0	0	0.023404	4.48503174	5.83710918	6.35513209	7.54506493	7.97924519	8.8530928	0	0	0.02713465	0.06019609	0.09387266	[illegible]
ENSG00000183808	4.55133606	5.54160522	4.88816593	5.0765118	3.89976296	5.31966825	3.16524288	3.49783991	3.22344436	2.97363421	3.41597931	3.42711068	2.83171878	2.4969411	2.94814052	2.7458043	2.97373651	[illegible]
ENSG00000183813	0.05206743	0.09067654	0.08147362	0.0673032	0.09294183	0.13279405	0.10871651	0.10200093	0.07207673	0.11318077	0.03935743	0.1407069	0.08814078	0.05640669	0.10867888	0.07031954	0	[illegible]
ENSG00000183814	4.00920227	3.07722632	3.46898286	2.8145123	3.79355081	2.92776707	0.95621098	1.27843072	1.77422966	0.72963875	0.99129469	1.83351833	0.86157146	0.60211219	0.84906762	1.24903296	0.99582141	[illegible]
ENSG00000183822	0	0	0	0	0	0	0	0	0	0	0	0	0	0	0	0	0	[illegible]
ENSG00000183826	0.70997189	0.85615734	0.70041979	0.7168193	0.67624606	0.67713495	1.52874922	1.55282746	1.30320218	0.88854063	1.38160192	1.16239017	0.51252466	0.46875807	0.53635764	0.42580939	0.41525099	[illegible]
ENSG00000183828	4.1450959	2.55954071	2.9057722	2.7731942	5.55926991	2.7449638	0.71517664	0.63439913	0.48830969	1.03390105	0.611963	0.43756629	1.87071672	1.60501901	1.49972619	1.11681822	0.88907856	[illegible]
ENSG00000183831	2.49361595	2.46908516	2.66041267	1.8345997	3.11736167	1.55260831	0.32452557	0.34755178	0.29236891	0.40171385	0.18625571	0.33294178	0.04171188	0.10677597	0.09429084	0.03803215	0.02965457	[illegible]
ENSG00000183833	0.0012602	0.00168821	0.00123246	0.0048869	0.01054454	0.00472656	0.13460116	0.12467245	0.08535572	0.15865406	0.13971181	0.101103	0.11839814	0.13379248	0.10083162	0.07172573	0.08720706	[illegible]
ENSG00000183837	0.01008361	0.01688541	0.01232699	0.0097757	0.00703107	0.01134595	0.00404895	0.00246924	0.00498525	0.00228324	0.00635178	0	0	0	0.00219242	0	0	[illegible]
ENSG00000183840	0.96749688	0.72004972	0.97904855	0.3821286	0.28108873	0.2721538	1.84530942	2.49421716	2.06276412	2.6288516	1.70134517	1.98021237	5.45932657	5.03683409	5.39332428	4.47216688	4.47758111	[illegible]
ENSG00000183844	0.0448939	0.04373912	0.03193122	0.0422041	0.02276616	0.03979895	0.00327756	0.00399763	0.01210645	0	0.01028333	0.00689325	0.00345442	0.00442139	0.00354946	0	0	[illegible]
ENSG00000183850	5.05198951	4.43059311	4.72367438	4.3152683	5.44305787	5.114992	0.44207695	0.90446442	1.14567535	0.80416546	0.82773475	1.79953657	0.30811638	0.25970386	0.27026261	0.94216006	0.98840412	[illegible]
ENSG00000183853	5.84086576	4.33224728	4.83246511	3.4564907	5.27275251	4.06201707	12.0416045	13.2061161	12.8806392	12.6629485	11.8036493	10.4574709	8.0906979	8.23730341	8.32089914	7.21829716	6.52538146	[illegible]
ENSG00000183856	10.3434267	6.95041331	6.79869906	6.3996961	6.61539215	6.53559177	5.87586137	7.56173036	11.6329442	6.61072531	3.57199786	9.63804373	5.90996201	4.52862337	7.51967359	6.29014374	6.75698817	[illegible]
ENSG00000183862	0	0	0	0	0	0	0	0	0	0	0	0	0	0	0	0	0	[illegible]
ENSG00000183864	10.0589634	10.9638521	9.7252933	10.856133	8.60526442	9.90966817	8.40296983	10.0006864	8.92823332	6.56774798	6.81546566	7.89821422	10.6655054	9.93574585	10.5703595	7.66506688	6.57611578	[illegible]
ENSG00000183873	0.01505452	0.01432955	0.02634651	0.0204839	0.01303858	0.01812786	0.00159078	0.00116416	0.00332968	0.00179411	0.00049911	0	0.0057005	0.00300432	0.0013782	0.00382177	0.00357591	[illegible]
ENSG00000183876	0.006367	0.00852943	0.02490724	0.0246903	0.01065494	0.00955209	0.08692398	0.06860177	0.16053721	0.06343407	0.10427671	0.09678464	0	0.02069285	0	0	0	[illegible]
ENSG00000183878	0.00085397	0.00343202	0.00167034	0.0044154	0.00047636	0.00064059	0.00137161	0.00083647	0.00253318	0.00464077	0.00107585	0.00288471	0.00144562	0	0	0.00082381	0.00128468	[illegible]
ENSG00000183888	0	0.0568292	0.02074374	0.0137087	0.01774771	0.0556875	0.01703382	0.0207761	0.01572962	0.05763318	0.02672178	0.03582493	0.03590599	0.08042454	0.05534076	0.01023076	0.03190867	[illegible]
ENSG00000183889	0.00414237	0.00832388	0.00810233	0.0160635	0.01039817	0.04505582	0.00665327	0.00811498	0.00921579	0.01875921	0.01043731	0.00524735	0.01402459	0.01121899	0.02161565	0.01598421	0.01557909	[illegible]
ENSG00000183891	2.95210026	4.18860881	3.74598772	4.2946358	3.0160802	4.23046079	1.41905551	1.25406556	1.43856829	3.12420658	1.63961282	1.6888379	1.69265896	1.4557778	1.7760368	1.50046567	2.2284741	[illegible]
ENSG00000183900	0	0	0	0	0	0	0	0	0	0	0	0	0	0	0	0	0	[illegible]
ENSG00000183908	0	0.01596155	0	0	0.00332319	0	0	0	0	0	0	0	0.02016974	0	0.00518116	0.03448201	0	[illegible]
ENSG00000183909	0	0	0	0	0	0	0	0	0	0	0	0	0	0	0	0	0	[illegible]
ENSG00000183911	0	0	0	0	0.03891436	0	0	0	0	0	0	0.0589134	0	0	0	0	0	[illegible]
ENSG00000183914	0.05927205	0.0864384	0.06457084	0.0620687	0.02887799	0.06190833	0.00903795	0.00514433	0.00445118	0.00475681	0.02174002	0.00316805	0.00444531	0.00325123	0.00522012	0.00217133	0.00338608	[illegible]
ENSG00000183918	0	0.00490971	0.0143371	0	0	0	0	0	0.00362386	0	0	0	0.00620413	0	0	0	0	[illegible]
ENSG00000183921	0	0.00721283	0	0	0	0.00807762	0.00864781	0.01054771	0.01597137	0.02925948	0	0.00909388	0.00911446	0.0233316	0.00936522	0	0	[illegible]
ENSG00000183929	0.21474563	0.28768005	0.20126664	0.3469798	0.12977216	0.30874824	0.51018472	0.4995695	0.47775774	0.4295208	0.21417856	0.11334532	0.20448319	0.13570805	0.17898172	0.12947508	0.13460641	[illegible]
ENSG00000183935	0.16305096	0.16018071	0.116938	0.1826608	0.12733462	0.09784686	1.28323279	1.33091428	0.97270593	0.88607371	0.95860444	0.76192014	0.89245144	1.08338894	0.91700475	0.90180447	1.0138564	[illegible]
ENSG00000183938	0	0	0	0	0	0	0	0	0	0	0	0	0	0	0	0	0	[illegible]
ENSG00000183941	0.10535243	0.02171284	0.03962799	0.0419016	0.09493265	0.07902748	0.01301629	0.01587593	0.01201969	0.01468001	0.01020964	0.0273754	0	0	0	0	0	[illegible]
ENSG00000183943	1.18423998	2.09657452	1.69427735	3.207582	1.33519408	3.0824663	1.74076063	1.78299659	1.89090787	2.656847	2.94168915	3.11335364	1.34945691	1.10160026	1.05540473	2.19197171	2.50546887	[illegible]
ENSG00000183955	4.42556039	3.24121615	3.68797169	3.2953129	5.25368838	3.50061799	2.66873437	3.0629632	3.73132152	2.88263762	2.20679786	3.56953586	3.57088303	2.94553123	3.44326263	3.5764433	3.15740023	[illegible]
ENSG00000183960	0.29849889	0.5834592	0.3821584	0.1683686	0.19224225	0.24223147	0.03704716	0.02658019	0.01475753	0.02212014	0.02051212	0.00458331	0.00459368	0.00293978	0.00236003	0.00261777	0	[illegible]
ENSG00000183963	1.28771681	0.8177515	0.85473758	0.9431052	1.24205404	0.99429452	0.80614126	1.00412787	1.19710578	0.79421705	0.69945423	1.04574428	5.45870289	3.7715257	5.37546127	5.30915676	4.88452645	[illegible]
ENSG00000183971	0.01340671	0.04490015	0.06555763	0.0173297	0.06730695	0.06034026	0.35529761	0.32829957	0.28501104	0.38856847	0.27024123	0.31701487	0.07943303	0.01452402	0.02331955	0.05173258	0.06050569	[illegible]
ENSG00000183977	0	0	0	0	0	0	0	0	0	0	0.00798815	0	0	0	0	0	0	[illegible]
ENSG00000183978	18.0757002	16.314281	18.0450774	15.362991	14.9443122	13.6492004	10.0273584	10.007979	10.6153085	9.9617543	10.8655244	9.59291419	9.36026356	8.95275423	8.84679417	9.71149598	8.47643869	[illegible]
ENSG00000183979	0.27064862	0.24171298	0.19851712	0.1457687	0.11952074	0.10151013	0.25357628	0.32033216	0.27318795	0.32684346	0.31255507	0.39046103	0.06681491	0.07330107	0.07846072	0.04351474	0.06785896	[illegible]
ENSG00000183981	0	0	0	0	0	0	0	0	0	0	0	0	0	0	0	0	0	[illegible]
ENSG00000184005	1.69454472	1.58792782	1.33068567	1.4189024	1.06399182	1.27112014	0.03687022	0.10220567	0.14237934	0.04536318	0.03154914	0.00704947	0.01413083	0.01808637	0.0217794	0.03623693	0.01255769	[illegible]
ENSG00000184007	8.97857153	7.94492254	8.20479999	7.4968883	9.33481303	6.96379156	12.613535	13.7353425	12.5129831	10.7606113	13.6107907	12.8797738	11.583148	13.1737752	11.6508527	12.7797552	12.5291364	[illegible]
ENSG00000184009	66.0174586	44.4931937	52.2984496	39.282286	82.2031669	44.1650834	70.6643859	84.8228601	97.6312532	78.4663822	82.8975527	76.353789	107.345414	101.944651	94.6834348	117.094515	94.3629614	[illegible]
ENSG00000184012	0.00865693	0.00869782	0.00846632	0.005595	0.00845078	0	0.01042824	0	0.00855982	0.00588058	0.00545309	0.00182769	0.00915914	0.0023446	0.00941112	0.00417557	0	[illegible]
ENSG00000184014	7.97527725	8.93560808	8.06073438	7.2768293	6.65139585	6.95056483	3.38438261	3.5198947	3.2407295	3.8124078	3.78325321	3.43242075	5.80185149	6.04822432	6.28122897	5.56914981	5.75640161	[illegible]
ENSG00000184022	0	0	0	0	0	0	0	0	0	0	0	0	0	0	0	0	0	[illegible]
ENSG00000184029	0	0	0	0	0	0	0	0	0	0	0	0	0	0	0	0	0	[illegible]
ENSG00000184032	0	0	0	0	0	0	0	0	0	0	0	0	0.07104055	0	0	0	0	[illegible]
ENSG00000184033	0	0	0	0	0	0	0	0	0	0	0	0	0	0	0	0	0	[illegible]
ENSG00000184040	0.00399278	0	0	0	0.00445452	0.00299509	0.0032065	0.00391096	0.00592199	0	0	0	0	0	0	0	0	[illegible]
ENSG00000184047	0.42421565	0.35429129	0.33849657	0.5093444	0.32376629	0.45535327	0.46753986	0.5059292	0.42384475	0.48871719	0.41815731	0.42870059	0.30647829	0.26728098	0.31182283	0.32019329	0.37382598	[illegible]
ENSG00000184055	0	0	0	0	0	0	0	0	0	0	0	0	0	0	0	0	0	[illegible]
ENSG00000184056	0.72815001	0.67062382	0.63645513	0.6676772	0.58895749	0.64178903	1.17682737	1.15231017	0.94947649	1.41380736	1.08647412	1.00117009	0.98417548	0.83320535	1.05874754	1.23803184	1.11594075	[illegible]
ENSG00000184058	0.0052321	0.00350454	0.0255845	0.0067631	0	0.00392473	0.59244933	0.69185937	0.67254341	0.86246746	0.61301109	0.5302206	0.03099951	0.04534512	0.03640272	0.02523643	0.06296783	[illegible]
ENSG00000184060	0.00522108	0.01224007	0.00255306	0.0067489	0.00145622	0.01174939	0.08176193	0.07926842	0.04775327	0.11112803	0.07235399	0.0683426	0.00883835	0.01414051	0	0.01259164	0.0078544	[illegible]
ENSG00000184068	0.31270072	0.26723173	0.25308885	0.3345122	0.12029732	0.51765978	0.0952532	0.08449458	0.06397098	0.15625938	0.12225952	0.04553026	0.11864652	0.12849554	0.20631049	0.09361711	0.09732733	[illegible]
ENSG00000184076	67.789886	47.3640513	61.6533286	64.907374	78.0863361	61.3756415	23.4499007	24.434628	25.5583052	17.4563413	25.6886361	27.4720252	21.9145899	24.2780789	22.0316537	20.5617011	18.3274167	[illegible]
ENSG00000184083	2.49037177	2.93469215	2.51462991	2.4412249	3.03576821	2.30522639	3.07156394	3.21051493	2.92458452	2.78124334	3.43708456	3.42886421	1.53007196	1.55896853	1.650776	1.8379422	2.05391304	[illegible]
ENSG00000184084	0	0	0	0	0	0	0	0	0	0	0	0	0	0	0	0	0	[illegible]
ENSG00000184100	0	0	0.00805523	0	0.00459454	0.01235693	0.01322918	0	0	0	0	0.00695579	0	0	0.00716333	0	0	[illegible]
ENSG00000184106	0	0	0	0	0	0	0	0	0	0	0	0	0	0	0.01036999	0	0	[illegible]
ENSG00000184108	0	0	0	0	0	0	0.00694656	0.0084727	0.00855293	0.00783446	0	0.01460976	0.00732141	0.01874168	0.02256851	0.05841089	0.01301268	[illegible]
ENSG00000184110	0.18105066	0.16412563	0.18238953	0.2111541	0.17768854	0.15316985	0.37606501	0.47201924	0.42937824	0.35138933	0.3961592	0.47133706	0.20624444	0.1312511	0.16337865	0.14182541	0.12696757	[illegible]
ENSG00000184111	0	0	0	0	0	0	0	0	0	0	0	0	0	0	0	0	0	[illegible]
ENSG00000184113	0.00483567	0	0	0.0062507	0.00539488	0.02176414	0.0038834	0.00473658	0	0.00875955	0	0	0	0	0	0	0	[illegible]
ENSG00000184115	0	0	0	0	0	0	0	0	0	0	0	0	0	0	0	0	0	[illegible]
ENSG00000184117	18.7379313	17.3154109	17.4002674	24.397936	18.1936625	20.9039589	17.2919113	18.6099696	14.8388088	15.224068	19.0513807	12.4659343	9.59710002	15.081389	14.2180066	7.10795092	8.50995819	[illegible]
ENSG00000184139	0.05644438	0.07561468	0.11040318	0	0	0.12702099	0	0	0	0.05112295	0	0	0	0	0	0	0	[illegible]
ENSG00000184140	0	0	0	0	0	0	0	0	0	0	0	0	0	0	0	0	0	[illegible]
ENSG00000184144	0.04856314	0.07318882	0.08311429	0.0549268	0.01580224	0.04705338	0.00649997	0.001982	0.00400153	0.0018327	0.00254921	0	0	0	0	0	0	[illegible]
ENSG00000184148	0	0	0	0	0	0	0	0	0	0	0	0	0	0	0	0	0	[illegible]
ENSG00000184154	0.3587088	0.37916398	0.36042202	0.4014265	0.3080921	0.34574473	0.1223311	0.138618	0.11660879	0.12728594	0.13742997	0.12117172	0.09898671	0.092626	0.09316299	0.0824803	0.08722758	[illegible]
ENSG00000184155	0	0	0	0	0	0	0	0	0	0	0	0	0	0	0	0	0	[illegible]
ENSG00000184156	0.98681927	0.79510053	0.96664561	0.1787043	0.57174295	0.24674589	0.03062762	0.01867821	0.0054994	0.0086356	0.04604507	0.02952351	0.07263075	0.06713904	0.05804477	0.04905438	0.05976387	[illegible]
ENSG00000184160	0	0	0	0	0	0	0	0	0	0	0	0	0	0	0	0	0	[illegible]
ENSG00000184162	2.18251594	0.96408714	1.42144089	1.3376174	2.04920574	1.23139798	1.42031051	1.90742626	2.31616594	1.46126441	1.43404938	1.78354901	1.23418965	0.698111	0.99815278	1.3204266	0.87035657	[illegible]
ENSG00000184163	0.11882774	0.04898013	0.09833272	0.2008599	0.13256929	0.40453869	0.13213048	0.09848615	0.08134257	0.23180765	0.08060868	0.08491149	0.10057698	0.08912129	0.10334408	0.05290632	0.08250464	[illegible]
ENSG00000184164	2.05124516	1.67767323	1.91457548	1.4172206	2.28310333	1.33353134	1.15985569	1.28072512	0.95955719	0.5866924	1.16062514	0.75976973	1.48439538	1.49705725	1.84655598	1.7334651	1.75043444	[illegible]
ENSG00000184166	0	0	0	0	0	0	0	0	0	0	0	0	0	0	0	0	0	[illegible]
ENSG00000184178	2.27881555	2.09832645	2.24459754	3.5853794	3.17641267	2.18264195	3.33254254	3.95814717	3.58154379	4.17228345	3.64515566	3.91321769	2.67455815	2.2448359	2.50691018	4.08184565	3.93543626	[illegible]
ENSG00000184182	0.91626724	0.67648611	0.72060157	0.7040609	0.91858677	0.63371143	0.80979685	0.7994981	0.83847185	0.71913693	0.73621296	0.93203111	0.75000007	0.64512234	0.75958571	0.85785957	0.6760603	[illegible]
ENSG00000184185	0	0	0.00413758	0	0	0	0.01019279	0.00828808	0.00209164	0.00766375	0.01065995	0.00714571	0.02506655	0.05499986	0.01839728	0.01632517	0.01272912	[illegible]
ENSG00000184188	0	0	0	0	0	0	0	0	0	0	0	0	0	0	0	0	0	[illegible]
ENSG00000184194	2.66035372	3.32090139	2.6453486	4.6317092	1.75027023	3.27984563	3.24558389	3.60952154	2.55814134	2.09826206	2.46958008	1.6070654	1.09894012	1.49619192	1.14024473	0.63852554	0.61276751	[illegible]
ENSG00000184203	6.8621117	8.52400245	8.065197	7.7378352	10.0837982	6.66240646	3.03799915	3.49187934	3.3850046	3.67921446	5.12727094	4.83243698	4.5228534	4.09822791	4.19486687	6.29131684	5.7312234	[illegible]
ENSG00000184205	1.23807588	1.76333591	1.43120004	1.3776098	1.33096911	1.47957465	2.69963872	2.56563753	2.25570416	2.65811181	2.75211697	2.48067234	3.08541681	4.06972162	4.10410191	2.3298641	2.34509458	[illegible]

ENSG00000184206	0.03110979	0.0324144	0.02704428	0.0134043	0.02121008	0.02592914	0.01665566	0.01354324	0.01367148	0.0313076	0.01741902	0.02335306	0.00292574	0.01123415	0.02104361	0.01333817	0.00520005
ENSG00000184207	5.40416066	4.68373804	5.1723523	3.6934874	6.86566337	3.8740704	4.57511483	4.29384022	4.84340651	2.53172631	3.34265413	3.76195796	4.80697312	4.00877319	4.11344713	5.42727774	4.43201986
ENSG00000184208	1.1244227	1.20430414	1.02345079	1.0577069	0.98120647	0.90609041	3.45552263	3.62583346	2.76301242	4.1795354	3.75882684	3.27650078	2.19555799	2.27947328	1.84930843	1.70760408	1.52406075
ENSG00000184209	1.97132049	2.21577616	2.06876834	1.7802227	1.84529392	1.70831206	1.52167264	1.77662653	1.61543926	1.6835593	1.75206777	1.991653	1.47807207	1.5212556	1.56570835	1.19832339	1.27289807
ENSG00000184210	0	0	0	0	0	0	0.01666638	0	0	0	0	0	0	0	0	0	0
ENSG00000184216	3.60874071	2.89783563	2.99615097	2.6640128	4.01830745	2.55157498	11.9072921	13.5793157	12.0495829	12.7327366	13.0627168	12.0810955	4.17856758	3.39726429	3.16148883	3.55102793	2.30752537
ENSG00000184220	3.12272843	2.06767226	2.62991756	2.5144512	3.40396437	2.506556	3.25532802	3.99778922	5.74666208	3.35075559	3.57826143	5.47248911	4.50170185	2.99080778	3.52192489	4.47834189	3.42305001
ENSG00000184221	13.0659664	14.6852699	11.6451023	8.6206456	5.376255	8.21097999	0	0.0156905	0	0	0.00672693	0	0.00451948	0.02313833	0.00464382	0.00515097	0
ENSG00000184224	0.49835133	0.4916017	0.38104149	0.632465	0.31337165	0.53694968	0.90957593	0.71002051	0.60027274	1.13252361	0.66207962	0.57389635	0.4908329	0.72638877	0.81166704	0.62934279	0.62702293
ENSG00000184226	2.95713206	4.50478835	4.00067123	4.1035477	3.16524198	4.35674575	0.18368051	0.18853183	0.16992589	0.23206256	0.10077325	0.16465686	0.61145511	0.37776755	0.38261888	0.507598	0.5114198
ENSG00000184227	0.33482639	0.311087	0.40139546	0.4048795	0.41572118	0.27546756	0.16480394	0.28564703	0.1121368	0.21521667	0.13607153	0.15506228	0.35653597	0.44463746	0.36634506	0.29174125	0.24372602
ENSG00000184232	0.65412401	0.42191513	0.74239289	0.8768477	1.15771932	0.77538716	18.0680879	20.4001981	17.4831992	21.5331775	19.6761822	17.5610939	8.14764479	7.46257266	7.92231165	8.66286783	6.34159101
ENSG00000184247	0	0	0	0	0	0	0	0	0	0	0	0	0	0	0	0	0
ENSG00000184254	0.00578423	0.00387437	0.00282844	0	0.00161328	0	0.34374265	0.51841166	0.5962421	1.90696322	0.50645375	0.51290183	5.09656047	4.53679053	7.30180925	6.02072742	6.86990103
ENSG00000184258	0	0.01661893	0	0	0	0	0.01992524	0	0	0	0	0	0	0	0	0	0
ENSG00000184260	1483.60056	1034.34633	1327.09026	1129.6056	1421.85971	908.742303	103.057742	158.930048	232.302485	71.6086341	165.109509	312.788899	206.56317	139.982561	130.400871	144.215168	102.185117
ENSG00000184261	0.11869309	0.11313819	0.32591612	0	0.00509304	0.00684882	0.00366613	0	0.00225696	0.00826946	0	0.00385524	0.00772792	0.00494557	0.01985134	0.01321159	0.05494077
ENSG00000184270	736.176177	553.963258	616.119584	589.85231	593.569268	431.774133	32.8735846	46.4378572	89.238104	32.3839944	32.6280095	80.4996375	47.5565761	25.9529954	33.6611394	47.4697251	27.9222972
ENSG00000184271	0.85200045	0.91446859	0.81316303	1.3202452	0.62127883	1.10496163	0.58323582	0.60831017	0.54555819	1.17146014	0.61751058	0.6479958	0.68421573	0.88964386	0.85703926	0.5619652	0.7373739
ENSG00000184274	0	0	0.01356871	0	0	0	0	0	0	0.01256617	0	0	0	0	0	0	0
ENSG00000184276	0	0	0	0	0	0	0	0	0	0	0	0	0	0	0	0	0
ENSG00000184277	1.43672936	1.6076282	1.55668934	1.7257187	1.34626746	1.76787143	1.33182387	1.41545078	1.16351848	1.81605322	1.31447197	1.17531273	0.81258901	0.80170602	0.86248672	0.85216835	0.77478078
ENSG00000184281	2.24647137	2.05503212	2.0546923	2.4610028	2.00906601	2.15815413	3.26975118	3.15960128	3.15054931	2.8639289	2.66974113	2.92124234	2.39924751	1.81623601	2.022301	1.42134917	1.17667098
ENSG00000184292	0.01558498	0	0	0	0	0	0.05006362	0.01526561	0	0.05646268	0.03926861	0.03948451	0	0	0	0	0
ENSG00000184293	0	0	0	0	0	0	0	0	0	0	0	0	0	0	0	0	0
ENSG00000184302	0	0	0	0	0	0	0	0	0	0	0	0	0.00978815	0	0	0.01115582	0

[illegible]

ENSG00000184916	2.35929031	1.41137212	1.39215163	1.2257902	3.13901801	1.59935236	0.04351754	0.02245617	0.0123648	0.0471922	0.04463681	0.00528026	0.09173162	0.15805108	0.09969317	0.04423229	0.05330114	[illegible]
ENSG00000184922	0	0.00601253	0.00146313	0.0096692	0.00500722	0.01122236	0.31237767	0.26523887	0.22485114	1.54743339	0.60689811	0.4965265	3.1150605	2.93355114	3.15522339	2.55305564	2.94606903	[illegible]
ENSG00000184923	0.01331074	0.03269107	0.04339222	0.0229409	0.00495001	0.0166412	0.03206856	0.04345992	0.01754857	0.04420475	0.03353834	0.02622879	0.01126634	0.00961336	0.00771754	0	0	[illegible]
ENSG00000184924	4.40489555	4.22979216	4.75593124	4.6917257	3.89211692	3.67429454	3.43558186	3.33434628	3.76313884	3.33212545	3.16093009	2.52955303	1.16920975	1.27507737	1.23815415	1.22379854	1.27229992	[illegible]
ENSG00000184925	0.00686104	0.0137869	0.01341995	0	0	0.01286659	0.01377482	0.02352155	0.01526418	0.0466065	0.03025292	0.01448536	0.01161451	0.00743283	0.01193405	0.00661869	0	[illegible]
ENSG00000184933	0	0.01572323	0.02295711	0	0.05237709	0	0	0.02299293	0.01160532	0.02126089	0.02957301	0.01982374	0	0	0	0.02264478	0.0706267	[illegible]
ENSG00000184937	0.00181372	0	0.00354756	0.0023444	0	0.00136051	0	0	0.00089669	0	0	0	0.01535148	0.01178922	0.01104168	0.02624475	0.02455639	[illegible]
ENSG00000184939	1.45507796	1.62306767	1.53892285	1.4535296	1.28757321	1.58212744	0.70511851	0.70334514	0.64205072	0.77163519	0.9001492	0.762504	0.91466802	1.05389058	1.0160909	1.00132217	1.16400201	[illegible]
ENSG00000184945	0	0	0	0	0	0	0	0	0	0	0	0	0	0	0	0	0	[illegible]
ENSG00000184949	0.04218207	0.18541832	0.07219329	0.1005309	0.05735452	0.1147016	0.16196657	0.18334719	0.14467777	0.30206033	0.19097876	0.14917017	0.20529412	0.24276794	0.23157787	0.18947243	0.18243931	[illegible]
ENSG00000184954	0	0	0	0	0	0	0	0	0	0	0	0	0	0	0	0	0	[illegible]
ENSG00000184956	0	0	0	0	0	0	0	0	0	0.0030338	0	0	0	0	0	0	0	[illegible]
ENSG00000184961	0	0	0	0	0	0	0	0	0.02742531	0	0	0	0	0	0	0	0	[illegible]
ENSG00000184967	2.69161254	2.10336553	2.37516522	2.2994868	2.64909026	1.87399913	2.63984386	3.03798513	3.34589448	2.03154543	2.13396213	2.66497245	2.98523759	2.23722174	2.26689263	2.86514333	2.38528246	[illegible]
ENSG00000184979	3.37587223	3.43744894	3.24232191	3.972345	3.0822587	3.80416644	14.9048503	11.2546213	3.98165529	0.90494111	7.15190439	1.87930701	2.22951884	3.72281509	1.42191176	0.686374	1.25255407	[illegible]
ENSG00000184983	43.660921	31.932716	40.6027414	34.324506	52.3683479	36.6988251	11.3715306	11.3878537	13.6950547	15.7119513	10.8478878	13.9527302	17.5070611	16.5821825	14.877588	18.249952	16.3687238	[illegible]
ENSG00000184984	0	0.00342558	0	0	0	0	0.03285673	0.04508467	0.03286947	0.06948081	0.04510095	0.03455156	0	0	0.00444781	0	0.00769363	[illegible]
ENSG00000184985	0.03226494	0.05108189	0.05163469	0.0947868	0.0278152	0.09901118	0.17902256	0.18962258	0.16676576	1.59132986	0.34735639	0.27000009	0.33267771	0.50206472	0.34948333	0.3254002	0.3000542	[illegible]
ENSG00000184986	0.86776004	0.63178199	0.80253125	0.5120718	0.64716052	0.5801753	0.36358761	0.40651116	0.55025591	0.48694725	0.32083732	0.18320567	0.43909174	0.44960276	0.67265663	0.72791997	0.6810919	[illegible]
ENSG00000184988	0.00976067	0.02179284	0.02863729	0.0084112	0.01996394	0.00488115	0.60618121	0.58320003	0.51472984	0.65714103	0.42218713	0.42038689	0.3111843	0.29960004	0.34238253	0.29189271	0.31814455	[illegible]
ENSG00000184990	3.89021538	2.70576927	3.08101484	3.5783575	3.32288135	3.01715588	1.43654378	1.38130515	1.70691894	1.05389095	1.13335166	1.40211225	2.83114865	2.03947967	2.29883623	2.41586532	2.14235845	[illegible]
ENSG00000184991	0	0	0	0	0	0	0	0	0	0	0	0	0	0	0	0	0	[illegible]
ENSG00000184992	4.50382495	2.61406925	3.45166029	1.4914632	6.24701939	2.4910929	1.85712091	2.69250686	3.73185954	1.91446539	1.80176066	3.9263016	3.61511783	2.09557474	2.24729843	3.84431915	2.68389958	[illegible]
ENSG00000184995	0	0	0	0	0	0	2.09243494	2.14465264	1.52629679	2.28056299	1.71021209	1.12791945	0	0	0	0	0	[illegible]
ENSG00000184999	0	0	0	0	0	0	0	0.00287831	0	0	0	0	0.0024872	0	0.00255562	0	0	[illegible]
ENSG00000185000	2.06640437	1.81018916	1.76829306	2.7557857	2.16988528	2.2354429	2.57590511	2.53762688	2.41489373	3.46034514	2.57416088	2.39296927	1.84039221	1.48684258	1.59261896	1.20869218	0.82355294	[illegible]

[illegible]

ENSG00000185596	0.6508107	0.73662145	0.76897318	0.6729991	0.61197508	0.83291071	0.79357422	0.93669688	0.71574116	1.28958102	0.96046113	1.03640581	0.98478964	0.89498027	0.8270645	0.43819412	0.45955669	[illegible]
ENSG00000185607	0.14337073	0.11523838	0.2523852	0.0370647	0.17594554	0.15056433	0.25330233	0.42129831	0.28352501	0.1038833	0.2528703	0.31479882	0.07281025	0.18638296	0.04987561	0.05532257	0.04313633	[illegible]
ENSG00000185608	5.68413626	4.74764825	5.09813482	5.4185562	5.5419361	4.49209897	7.67104921	8.13512539	8.01801469	5.01487674	7.86597074	7.5858853	6.14060434	6.57262511	5.26791589	5.50229509	5.52344678	[illegible]
ENSG00000185610	0.06891605	0.16156372	0.10109799	0	0.01281428	0.01723188	0	0	0	0	0	0	0	0	0	0	0	[illegible]
ENSG00000185614	0.86772376	0.51894208	1.09108061	0.4005837	0.53589566	0.62765471	2.26475311	2.91408802	2.75782595	3.22787377	3.27953327	2.09369159	2.30827152	2.61868061	2.26396911	1.37519132	1.49185321	[illegible]
ENSG00000185615	0.08543091	0.09454231	0.07991729	0.0960256	0.13882161	0.13652647	0.00894879	0.00727654	0.00918181	0.0033642	0.00935894	0.0031368	0.01257558	0.00402394	0	0.00358318	0	[illegible]
ENSG00000185619	2.41956088	2.48302159	2.32004473	2.2771327	2.43485449	2.33889291	2.11884517	2.35145336	2.48459036	2.35051797	2.24488997	2.24639408	2.34181014	1.80747541	2.09135964	2.21549982	2.18271938	[illegible]
ENSG00000185621	0.88694081	0.81474791	0.74074247	0.5896155	1.20782916	0.68538146	0.30074038	0.36681214	0.24848101	0.3162285	0.49839044	0.50053615	0.72688329	0.75495968	0.81055004	0.85289499	1.00388759	[illegible]
ENSG00000185624	21.7124838	14.8072967	17.3723277	22.588684	21.6463655	18.1311604	18.2383184	19.9024999	17.2089485	20.0263116	16.5185409	15.1981795	24.0400619	26.1334469	26.6517711	20.6324922	20.862509	[illegible]
ENSG00000185627	4.77045989	3.8094158	4.30070038	4.3611547	5.25711846	3.82030589	6.89929694	7.49230709	9.01232928	6.11338768	7.78893033	9.14975905	5.45492807	5.34688719	5.5864232	5.83675907	5.20997793	[illegible]
ENSG00000185630	8.85403838	11.8880669	9.74638932	9.8071314	8.48771657	9.59156741	2.57664627	2.77226869	2.4035163	1.93256861	2.22554265	2.34557542	0.26743371	0.3619273	0.31590733	0.12617721	0.20506417	[illegible]
ENSG00000185631	0.07766202	0.10403851	0.15190413	0	0	0.05825622	0	0	0	0	0.09784035	0.06558552	0	0	0	0	0.11683188	[illegible]
ENSG00000185633	0.07801293	0.05922153	0.11189968	0.1411771	0.14505757	0.09753239	0.16289056	0.15282856	0.1388481	0.11776346	0.11138675	0.10541097	0.0792371	0.06197727	0.08594026	0.01505145	0.02347195	[illegible]
ENSG00000185634	0.2068552	0.20878134	0.1912157	0.1648261	0.09009766	0.14453934	0.05006362	0.05551132	0.05603695	0.09495996	0.05711798	0.06461102	0.06715562	0.15348928	0.13800644	0.16947947	0.20035219	[illegible]
ENSG00000185638	0	0	0	0	0	0	0	0	0	0	0	0	0.04950921	0.06336795	0.20348525	0	0.17598994	[illegible]
ENSG00000185640	0	0	0	0	0	0	0.01776451	0.0361122	0.06197204	0.06678382	0.07431479	0.13076591	0.72396028	0.85472074	1.0773406	0.18494013	0.33277439	[illegible]
ENSG00000185641	0	0.02886095	0	0.0556961	0.07210602	0.0323213	0	0.04220493	0.02130228	0.03902568	0.05428308	0	0	0.0466788	0.07494677	0	0	[illegible]
ENSG00000185650	10.6223499	15.7109455	12.5677132	10.461736	7.16837092	11.6204401	7.87419279	8.85954782	7.26086261	6.95692999	5.57386082	5.81953955	15.410204	14.5379093	14.1381944	10.5754453	11.4430224	[illegible]
ENSG00000185651	14.2773222	11.6305576	12.4590948	10.594686	14.2969703	10.2461718	10.304706	11.2281586	12.0110527	8.66639967	10.0617202	12.1255356	14.217387	13.1317622	13.4823439	15.142849	13.5200794	[illegible]
ENSG00000185652	0	0	0	0	0	0	0	0	0	0	0	0	0	0	0	0	0	[illegible]
[illegible]	[illegible]	[illegible]	[illegible]	[illegible]	[illegible]	[illegible]	[illegible]	[illegible]	[illegible]	[illegible]	[illegible]	[illegible]	[illegible]	[illegible]	[illegible]	[illegible]	[illegible]	[illegible]

ENSG00000186198	0.13714783	0.02296595	0	0	0.01912599	0.0257195	0.11013997	0.20150609	0	0.03105447	[illegible]	[illegible]
ENSG00000186204	0	0	0	0	0.00167756	0.00112794	0.00483025	0.00294572	0	0.00272382	[illegible]	[illegible]
ENSG00000186205	0.36085385	0.3967125	0.25700951	0.4360254	0.2888102	0.38543254	0.08504784	0.07683896	0.06980988	0.10302361	[illegible]	[illegible]
ENSG00000186207	0	0	0	0	0	0	0	0	0	0	[illegible]	[illegible]
ENSG00000186212	0	0	0	0.028525	0.01846467	0.00827673	0	0	0.01091003	0.03997426	[illegible]	[illegible]
ENSG00000186222	7.08804109	5.36208821	6.25916736	6.1440104	6.48898236	5.56712371	13.4771854	11.9456875	11.5125458	10.6304328	[illegible]	[illegible]
ENSG00000186223	0	0	0	0	0	0	0	0	0	0	[illegible]	[illegible]
ENSG00000186226	0.09182262	0.09840679	0.08980087	0	0.04097649	0.04132706	0	0	0	0	[illegible]	[illegible]
ENSG00000186230	1.55477887	1.35333709	1.41036133	1.1747716	1.41196285	1.12683894	1.91211302	1.96434388	2.04978472	2.23815121	[illegible]	[illegible]
ENSG00000186231	0.07904122	0.11683986	0.08529754	0.1162624	0.03800938	0.09200294	0.01969945	0.02135766	0.01077994	0.0098744	[illegible]	[illegible]
ENSG00000186232	0	0	0	0	0	0	0	0	0	0	[illegible]	[illegible]
ENSG00000186234	0	0	0	0	0	0	0	0	0	0	[illegible]	[illegible]
ENSG00000186235	0.01017674	0.03067444	0.00497634	0	0.0056768	0.01526765	0.00408634	0	0	0	[illegible]	[illegible]
ENSG00000186244	0	0	0	0	0	0	0	0	0	0	[illegible]	[illegible]
ENSG00000186260	0.88913183	0.75542178	0.87531838	0.7227206	0.830463	0.71116602	0.45315176	0.56425266	0.57979283	0.38030233	[illegible]	[illegible]
ENSG00000186265	0.0128252	0.00429526	0.00627141	0.0165781	0.00715417	0.03367177	0	0	0.00317034	0.01161608	[illegible]	[illegible]
ENSG00000186268	0	0	0	0	0	0	0	0	0	0	[illegible]	[illegible]
ENSG00000186272	1.04019006	1.30908432	1.0994284	0.9854591	0.98568748	1.17040865	0.74974	0.76890215	0.76500316	0.88653257	[illegible]	[illegible]
ENSG00000186275	0.29273626	0.38953271	0.29779356	0.347989	0.22817493	0.33428466	0.24978219	0.24193477	0.21967382	0.21897547	[illegible]	[illegible]
ENSG00000186280	0.72749544	0.70260127	0.67287017	0.6269152	0.58827075	0.61762801	0.64310949	0.62972856	0.52416655	0.64358565	[illegible]	[illegible]
ENSG00000186281	0	0	0	0	0	0	0	0	0.00059916	0	[illegible]	[illegible]

[illegible]

ENSG00000186943	0	0	0	0	0	0	0	0	0	0
ENSG00000186951	1.210805	1.18718993	1.19254373	1.9602686	1.2262814	1.58590331	0.39308454	0.36925602	0.26279472	0.32977383
ENSG00000186952	0.02942083	0.04874774	0.03634489	0.0580456	0.02850429	0.04762321	0.03730599	0.03791833	0.02602861	0.04347686
ENSG00000186960	0.05013961	0.02686744	0.00980713	0.0907358	0.02237515	0.01504439	0.03221269	0.05893457	0.00991544	0.01816503
ENSG00000186965	0	0	0	0	0	0	0	0	0	0
ENSG00000186967	0	0	0	0	0	0	0	0	0	0
ENSG00000186970	0	0	0	0	0	0	0	0	0	0
ENSG00000186971	0	0	0	0	0	0	0	0	0	0
ENSG00000186973	0	0.00769077	0.04491643	0	0.02561943	0.01722574	0.02766251	0	0.01702968	0
ENSG00000186976	0.060605	0.11061917	0.07557002	0.0528789	0.05578115	0.05569012	0.06448822	0.02226116	0.01872662	0.03979621
ENSG00000186977	0	0	0	0	0	0	0	0	0	0
ENSG00000186980	0	0	0	0	0	0	0	0	0	0
ENSG00000186994	0.00491682	0.01976017	0.00961712	0.0444889	0.01097082	0.02581759	0	0.01444819	0	0.01335982
ENSG00000186998	0.36137898	0.30136944	0.30427073	0.5475566	0.62344465	0.45957825	0.02882924	0.05391651	0.02129753	0.04985503
ENSG00000187003	0	0	0	0	0	0	0	0	0	0
ENSG00000187005	0	0	0	0	0	0	0	0	0	0
ENSG00000187008	0	0.0062345	0.00910284	0	0	0.00465466	0	0	0.0015339	0
ENSG00000187010	0.00920455	0.01233071	0.00900189	0.0089235	0.0051345	0.01208299	0.02402382	0.01126992	0.00796364	0.00416839
ENSG00000187012	0	0	0	0	0	0	0	0	0	0
ENSG00000187013	0.12639114	0.07054899	0.22661515	0.2995214	0.22326161	0.1422137	0.25375385	0.57773858	0.32284789	0.57237659

[illegible]

ENSG00000187764	0.0325083	0.05331017	0.03672586	0.0681026	0.05377617	0.07189455	0.130533	0.14823066	0.15988694	0.20255102	[illegible]
ENSG00000187766	0	0	0	0	0	0	0	0	0	0	[illegible]
ENSG00000187772	0.65571859	0.75293264	0.82450415	0.4692032	0.59764825	0.70706431	2.52011452	2.64367688	2.87711187	0.9650862	[illegible]
ENSG00000187773	0.21216465	0.51030857	0.46214399	0.436303	0.19904312	0.30383179	0	0	0.00476784	0	[illegible]
ENSG00000187775	0.00066992	0.00179489	0.00131033	0.0043297	0.00037369	0.00050252	0.01667781	0.01771712	0.02484011	0.04732725	[illegible]
ENSG00000187778	2.86777266	2.63306535	2.52744741	2.7119127	2.82614487	2.33485625	2.99125323	3.15397183	3.33805331	2.21159311	[illegible]
ENSG00000187783	0	0	0	0	0	0	0	0	0	0	[illegible]
ENSG00000187786	0	0	0	0	0	0	0	0	0	0	[illegible]
ENSG00000187790	0.7922656	0.63978954	0.65103348	0.5408317	0.79693499	0.64177165	0.35671332	0.47181734	0.52611519	0.32588001	[illegible]
ENSG00000187791	0	0	0	0	0	0	0	0.00475298	0	0	[illegible]
ENSG00000187792	2.59762082	3.03950315	2.6423572	2.7566534	2.06617835	2.16504442	3.62489268	3.77732014	3.09565581	3.65253153	[illegible]
ENSG00000187796	0.00904517	0.00121172	0.00707681	0.0116919	0.00201823	0.00949901	0.16852355	0.17719627	0.19944463	0.1507404	[illegible]
ENSG00000187800	0	0.00126187	0	0	0	0	0.00605165	0.03137	0.01303937	0.03583209	[illegible]
ENSG00000187801	0.6069559	0.71337787	0.54505929	0.5575814	0.69843974	0.6127839	0.56407109	0.53843152	0.94551544	0.57739328	[illegible]
ENSG00000187806	0	0	0	0	0	0	0	0	0	0	[illegible]
ENSG00000187808	0	0	0	0	0.01131431	0	0	0	0.01002776	0.0183708	[illegible]
ENSG00000187811	0.04583296	0.04911943	0.12550668	0.10664	0.05624653	0.11001743	0.04416876	0.02693625	0.02719131	0.04151195	[illegible]
ENSG00000187812	0	0	0	0	0	0	0.01590347	0	0	0.00896811	[illegible]
ENSG00000187815	1.61820517	1.29911417	1.35404482	1.0873919	1.69454164	1.10868431	0.91014703	1.05288178	1.21014867	0.95240477	[illegible]
ENSG00000187821	0	0.04581006	0.02508231	0	0	0	0	0	0	0	[illegible]
ENSG00000187823	0	0	0	0	0	0	0	0	0	0	[illegible]
ENSG00000187824	0.0151337	0.00337893	0.01973398	0.0130414	0.0084419	0.01135217	0.84669347	0.81529697	0.75318514	0.51629462	[illegible]
ENSG00000187833	0	0	0	0	0	0	0	0	0	0.01046575	[illegible]
ENSG00000187837	2374.43362	1582.38201	1794.32082	1756.7499	1923.69255	1566.54027	164.879684	248.629698	349.636068	162.067025	[illegible]
ENSG00000187838	0.01266084	0.02226112	0.00773879	0.0122742	0.00794529	0.02374302	0.04956704	0.02635295	0.02973219	0.06306963	[illegible]
ENSG00000187840	18.6988869	11.0772196	14.7583939	26.356771	25.3794701	17.000964	47.796572	55.0575725	67.6851069	59.7611225	[illegible]
ENSG00000187847	0	0	0	0	0	0	0	0	0	0	[illegible]
ENSG00000187848	0	0	0	0	0	0	0	0	0	0	[illegible]
ENSG00000187855	0	0	0	0	0	0	0	0	0	0	[illegible]
ENSG00000187857	0	0	0	0	0	0	0	0	0	0	[illegible]
ENSG00000187860	0.09811184	0.09286079	0.07926438	0.0882233	0.11421678	0.08319563	0.322016	0.32173054	0.25623636	0.16806531	[illegible]
ENSG00000187862	0.00943217	0.01895346	0.0092245	0.0182883	0.02630734	0.01061296	0.00378737	0	0.00466319	0	[illegible]
ENSG00000187866	4.87954371	4.45851807	4.82397682	3.8849805	4.27517914	3.90561108	3.40751192	3.27295404	3.30831537	2.62608767	[illegible]
ENSG00000187867	0.90775341	0.46956589	0.76470978	0.9061726	1.67451124	0.95060302	0.36810584	0.43137062	1.17306298	3.4514979	[illegible]
ENSG00000187870	0.03063663	0.0205209	0.02996208	0.0396014	0.03417953	0.09192521	0.19682808	0.33009711	0.15146488	0	[illegible]
ENSG00000187871	0.03373076	0.0338901	0	0.0872019	0.00940787	0	0.01354416	0.01651977	0.02501429	0.03055071	[illegible]
ENSG00000187889	0.00754088	0.0025255	0	0.0048737	0.00420647	0.0028283	0.00302795	0.00369318	0	0.00341497	[illegible]
ENSG00000187893	0	0	0	0	0	0	0	0	0	0	[illegible]
ENSG00000187900	0	0	0	0	0	0	0	0	0	0	[illegible]
ENSG00000187902	0.007716	0.03876225	0.00754611	0.0199477	0.00645623	0.03183381	0.00309826	0	0.00190736	0.04193135	[illegible]
ENSG00000187904	0.70894521	0.36527903	0.53333514	0.2114756	0.24336291	0.36816745	0.61313113	0.74783421	0.51226397	0.74089356	[illegible]
ENSG00000187905	0.00577628	0.0141865	0.00941517	0.0049777	0	0.0129988	0.01855515	0.01885972	0.01618256	0.04185369	[illegible]
ENSG00000187908	0	0.00046814	0	0	0.00038987	0	0.01515458	0.01026888	0.01589471	0.01899068	[illegible]
ENSG00000187912	0	0	0	0	0	0	0	0	0	0.00357341	[illegible]
ENSG00000187918	0	0	0	0	0	0	0	0	0	0	[illegible]
ENSG00000187922	0	0	0	0	0.00171845	0	0	0	0	0	[illegible]
ENSG00000187942	0	0	0	0	0	0	0.00422095	0.01029657	0	0	[illegible]
ENSG00000187944	0.03862162	0.01293469	0	0	0.01077198	0.02897104	0.01550802	0.03783018	0.03818839	0.12243166	[illegible]
ENSG00000187950	0.0217084	0.03392814	0.02123043	0.0093535	0.02421882	0.02171202	0.07554504	0.00708785	0.03577482	0.01966179	[illegible]
ENSG00000187951	1.50654495	1.47826868	1.49260227	1.3437911	1.31746805	1.28495513	0.42631576	0.52719794	0.69865111	0.73901788	[illegible]
ENSG00000187952	0.18334675	0.03508816	0.11527061	0	0.27029748	0.02947135	0	0	0	0	[illegible]
ENSG00000187953	2.68537964	2.74320614	2.54006328	3.1428131	2.70674203	3.67097554	2.03582669	2.01592518	1.85816736	2.92992001	[illegible]
ENSG00000187954	1.52220756	1.61579713	1.48208699	1.7860573	1.24768621	1.78217096	2.35377911	2.21469224	1.89510482	2.12493191	[illegible]
ENSG00000187955	0.01452706	0.02529919	0.032203	0.0701042	0.02431051	0.05521203	1.18452013	1.23036645	0.81683897	2.3402788	[illegible]
ENSG00000187957	108.380553	92.9462461	79.0538413	125.72351	103.492147	118.972059	2.84573003	3.54788913	5.94324265	23.5052546	[illegible]
ENSG00000187959	0.03353771	0.06739227	0.01639964	0.0433514	0	0.06289368	0	0.01642523	0.01658075	0.01518793	[illegible]
ENSG00000187961	1.52574532	1.92164998	1.4028775	1.8120678	1.30937419	2.06401194	3.33026901	4.01721251	3.45889038	4.6831128	[illegible]
ENSG00000187969	0	0	0	0	0	0	0	0	0	0	[illegible]
ENSG00000187979	0.06934855	0.06967614	0.18650951	0.0448206	0.01934205	0.11704517	0.01392302	0.05094559	0.00857133	0.01570264	[illegible]
ENSG00000187980	0.03959427	0.0212167	0.05421149	0.0614163	0.00883461	0.01782039	0.03179717	0.03102633	0.03523512	0.02868915	[illegible]
ENSG00000187984	0	0	0.00324281	0	0	0	0	0	0	0	[illegible]
ENSG00000187987	1.14870437	1.86606555	1.4010371	1.4848345	0.81017118	1.74315198	1.1557703	1.22862853	0.62992265	0.98061406	[illegible]
ENSG00000187988	0	0	0	0	0	0	0.01182407	0.01442178	0	0	[illegible]
ENSG00000187990	0.72426382	0.53363572	1.0624755	1.9660107	3.87849385	1.52120724	83.5232221	126.489974	249.144957	71.4364576	[illegible]
ENSG00000187994	0.01815322	0.02084454	0.03297082	0.040226	0.01591268	0.03890623	0.22700638	0.26417813	0.26155114	0.21139432	[illegible]
ENSG00000187997	0.02984235	0.03198221	0.01167411	0.0154299	0.0332934	0.03133969	0.00479313	0.05846164	0.02655684	0.07027513	[illegible]
ENSG00000187999	0.03488067	0.04672725	0.10233801	0.135262	0.07782872	0.13082429	0.05602358	0.03416589	0	0.03159221	[illegible]
ENSG00000188000	0.06014881	0.13429543	0.14706115	0.116624	0.06151253	0.19551619	0.29787466	0.26512309	0.22798409	0.34502825	[illegible]
ENSG00000188001	0.05834347	0.01617078	0.0708317	0.0208043	0.02581174	0.01660048	0.05493248	0.03152987	0.01889813	0.00728869	[illegible]
ENSG00000188002	1.14269723	0.9032632	0.92665365	1.0045908	0.91456034	0.99825187	0.16244493	0.15294523	0.11754956	0.32784662	[illegible]
ENSG00000188004	0.0994466	0.06055537	0.01768309	0.0701162	0.08573167	0.11528684	0.10890418	0.06198738	0.08045271	0.07369449	[illegible]
ENSG00000188010	5.49973922	6.17861348	6.09166555	4.834977	5.33562111	4.78533198	2.57747025	2.94191289	2.67592673	2.59110535	[illegible]
ENSG00000188011	0	0	0	0	0	0	0	0	0.00325319	0	[illegible]
ENSG00000188013	0.0948631	0.03971301	0.0115968	0.1226216	0.05953124	0.06226428	1.41889294	1.18471937	0.6859052	0.22553912	[illegible]
ENSG00000188015	0.02245975	0	0.02196523	0	0.01252853	0	4.18455128	4.42190011	2.38733984	1.79012275	[illegible]
ENSG00000188021	11.4001625	11.3119049	10.5312937	10.811306	10.8827917	10.8574212	6.33820396	7.1211001	6.7176846	5.77333395	[illegible]
ENSG00000188026	2.80039883	3.11127648	3.14326419	3.634293	3.2894986	2.87633749	2.19506322	2.68278712	2.40697292	3.39703423	[illegible]
ENSG00000188029	0	0	0	0	0	0	0	0	0	0.02020153	[illegible]
ENSG00000188032	0	0.0076175	0.02224425	0	0	0.00853081	0.02739897	0.03341844	0.01686744	0.01030036	[illegible]
ENSG00000188033	0.72936426	0.81309412	0.76817478	0.6856644	0.50927993	0.81493245	0.81101642	0.81933265	0.85987181	0.75299361	[illegible]
ENSG00000188037	0	0	0	0	0	0	0.011478	0	0	0	[illegible]
ENSG00000188038	0	0	0	0	0.01005505	0	0	0	0	0.01632618	[illegible]
ENSG00000188039	0.0090175	0.01046944	0.00470344	0.0116562	0.00838359	0.00856805	0.00820729	0.00471078	0.00653865	0.00925633	[illegible]
ENSG00000188042	3.04958338	1.5640934	2.70975775	3.654744	5.80325802	3.60128616	28.6258215	34.0955125	47.8303163	33.6657707	[illegible]
ENSG00000188050	0.13382591	0.19421722	0.06543967	0.1441547	0.07465094	0.16731024	0.07164815	0.08738903	0.0882165	0.02020153	[illegible]
ENSG00000188051	0.11286679	0.09239996	0.09811706	0.1783143	0.055964	0.11288558	0.06042699	0.04913508	0.03100021	0.35211183	[illegible]
ENSG00000188056	0	0	0	0	0	0	0	0	0	0	[illegible]
ENSG00000188060	1.29520303	1.02246672	1.34585314	1.330392	0.96117216	0.91084126	0.17645316	0.22654673	0.18295349	0.31422181	[illegible]
ENSG00000188064	1.3605958	1.52712473	1.42218093	0.6092894	1.10022577	0.7673433	0.57451914	0.49444687	0.94867509	0.08477894	[illegible]
ENSG00000188069	0	0	0	0	0	0	0	0	0	0	[illegible]
ENSG00000188070	4.07181411	5.6505871	4.18703109	5.455098	3.42040639	4.98585642	3.43469665	3.66776548	3.51693865	3.3440434	[illegible]
ENSG00000188073	0.04337785	0.07263792	0.06363413	0.1682128	0.07259126	0.17896341	0	0	0	0.01964415	[illegible]
ENSG00000188076	0	0	0	0	0	0	0	0	0	0	[illegible]
ENSG00000188078	0	0	0	0	0	0	0	0	0	0	[illegible]
ENSG00000188086	0.01689632	0.01697614	0	0.0109202	0	0.01901153	0	0	0.0041767	0	[illegible]
ENSG00000188089	0.00762654	0.00681117	0.00497241	0.0032861	0.00567233	0.01716256	0.00612468	0.00249008	0.00251366	0.00921004	[illegible]
ENSG00000188092	0.02211724	0.0246908	0.00961345	0.0381188	0.01644996	0.01843411	0.0177618	0.00962845	0.0206542	0.01558051	[illegible]
ENSG00000188095	0	0	0.01031417	0	0	0.01582221	0.01693907	0.03099079	0.03128424	0	[illegible]
ENSG00000188100	0	0	0	0	0	0	0	0.09150518	0	0.08461219	[illegible]
ENSG00000188101	0	0	0	0	0.0189546	0	0	0	0	0.01538809	[illegible]
ENSG00000188107	0.0092164	0.01719702	0.01545166	0.0119133	0.00954776	0.01283926	0.01004483	0.01160683	0.00781116	0.01132875	[illegible]
ENSG00000188112	0.00907369	0.0425439	0.02662171	0.0410508	0.01012299	0.034032	0.04007768	0.071102	0.0381306	0.06163684	[illegible]
ENSG00000188120	0	0	0	0	0	0	0	0	0	0	[illegible]
ENSG00000188124	0	0.04038913	0	0.0389717	0.15136194	0.02261584	0.07263674	0.05906321	0.10433932	0.05461404	[illegible]
ENSG00000188130	0.77059992	0.77121569	0.72713798	0.4280076	0.74133549	0.67288187	1.18330774	1.37398783	1.25008393	1.1641585	[illegible]
ENSG00000188133	0	0	0	0	0	0	0	0	0	0	[illegible]
ENSG00000188152	0	0	0	0	0	0	0	0.01176422	0	0	[illegible]
ENSG00000188153	1.36966904	2.32102683	1.73156393	1.6468	0.81654706	1.53429991	2.30232751	2.84681343	1.56525842	2.10286711	[illegible]
ENSG00000188155	0	0	0	0	0	0	0	0	0	0	[illegible]
ENSG00000188157	14.6009068	17.0367698	13.6796763	12.812538	8.77422697	11.324668	16.3700404	16.5042129	6.64271971	6.20428849	[illegible]
ENSG00000188158	1.37944695	1.19098446	1.05895083	0.6255217	0.6412374	0.60499933	1.1748031	1.44016824	1.08897873	0.45005139	[illegible]
ENSG00000188162	0.00242101	0.00243245	0	0.0015647	0.00135049	0	0.00194425	0.0047428	0.00359078	0.00328915	[illegible]
ENSG00000188163	0.0072918	0.01465248	0.00713125	0.018851	0.01220256	0.00546976	0.03513515	0.02856949	0.02523501	0.11227387	[illegible]
ENSG00000188167	0.69563717	0.87947837	0.81638479	0.7249738	0.75668039	0.62617769	0.81004062	0.90283135	0.7738133	0.33471638	[illegible]
ENSG00000188171	0.44604227	0.71703883	0.42375786	0.7138383	0.46207919	0.69785371	1.42940978	1.55204218	1.64024468	1.2389045	[illegible]
ENSG00000188175	0	0	0.00350769	0	0.00200072	0	0.00576072	0	0.00177321	0.00324852	[illegible]
ENSG00000188176	0	0	0	0	0	0	0	0.00651989	0	0	[illegible]
ENSG00000188177	2.91705822	3.46779883	2.98524594	3.2785146	2.55894703	3.19877314	0.5608929	0.60664714	0.48249017	0.84506612	[illegible]
ENSG00000188185	1.41125389	2.00104775	1.41602981	1.5991455	1.03770893	1.37482681	1.46451397	1.38233442	0.96501691	1.95880713	[illegible]
ENSG00000188186	9.15503104	8.25659487	9.14585199	11.536237	8.09658566	9.47689076	3.38708689	3.46853263	3.25703838	4.93690789	[illegible]
ENSG00000188191	1.14980932	1.48680138	1.38941575	1.8691644	1.12188565	1.55250102	0.95781414	0.98689813	0.94132429	0.46245592	[illegible]
ENSG00000188199	0.01369381	0.0055034	0.01071384	0.0177008	0.01069417	0.01232648	0	0.00268264	0	0.01984446	[illegible]
ENSG00000188206	3.82707861	7.51264972	5.23041071	5.9685218	4.14283051	8.8641127	4.31671354	4.59700566	3.48976714	6.94234935	[illegible]
ENSG00000188211	5.12142814	3.89056151	4.1341924	4.0218294	7.07620821	4.95141696	7.59527311	7.08502095	5.95350078	5.11924278	[illegible]
ENSG00000188215	2.137324	1.72799189	1.96662592	1.8077824	1.84897119	1.84923137	3.18856424	3.23896704	3.00204953	3.19536004	[illegible]
ENSG00000188219	0	0	0	0	0	0	0	0	0	0	[illegible]
ENSG00000188223	0	0	0	0.0803485	0	0	0.0249594	0.0304429	0.01536558	0	[illegible]
ENSG00000188227	0.27539431	0.49094184	0.33666336	0.4653688	0.23763219	0.49277966	0.17508677	0.20231319	0.11133309	0.1221172	[illegible]
ENSG00000188229	112.672432	58.9671965	79.5298987	69.389122	110.842535	65.1483604	46.2043468	55.2548493	63.8101909	32.3392775	[illegible]
ENSG00000188234	0	0.00950176	0	0.0091683	0.00395652	0.01596149	0.26201872	0.20147652	0.18935777	0.22484424	[illegible]
ENSG00000188242	4.36029916	3.7201527	4.05351165	4.3182261	4.10156169	3.35160533	3.69470304	3.70257068	3.26632769	2.23739184	[illegible]
ENSG00000188243	5.10581101	5.42543543	6.35456903	7.9694585	5.03725682	6.86519136	2.359998	2.62173344	2.08804314	2.57805499	[illegible]
ENSG00000188257	0.00516337	0.00345851	0	0	0.00576047	0.00774634	0	0	0	0	[illegible]
ENSG00000188263	0	0	0	0	0	0	0.00361089	0	0.00222295	0.00407243	[illegible]
ENSG00000188266	0.03155525	0.07133469	0.05400581	0.0611833	0.04400547	0.0917227	0.06652071	0.1506795	0.18330754	0.3215284	[illegible]
ENSG00000188269	0	0	0	0	0	0	0	0	0	0	[illegible]
ENSG00000188277	0.01296971	0.00868732	0	0	0.01446957	0	0	0	0.01923634	0	[illegible]
ENSG00000188279	0	0	0	0	0	0	0	0	0	0	[illegible]
ENSG00000188280	0.08279073	0.09935605	0.09108882	0.0579674	0.05195523	0.08021687	0.03324357	0.06757848	0.04775286	0.08748301	[illegible]
ENSG00000188282	0	0.00132678	0	0.0025604	0	0.00148585	0.00318148	0.00776087	0.00097929	0.00538219	[illegible]
ENSG00000188283	0.73446619	0.7929435	0.64252525	0.8933016	0.59467131	1.08328553	0.6147187	0.67691836	0.53930818	0.75784862	[illegible]
ENSG00000188290	6.05855427	5.57472534	5.81270902	5.6591788	6.32008102	4.74370898	0.19888062	0.18193058	0.14867168	0.1762367	[illegible]
ENSG00000188293	0	0	0	0	0	0	0	0	0	0	[illegible]
ENSG00000188295	3.71093337	3.95774959	3.90642913	3.6807521	3.47600915	3.83039936	2.04863918	1.89646372	1.99203267	1.54032884	[illegible]
ENSG00000188305	0	0.02153774	0	0	0.02391544	0.0482401	0.00860755	0.0209972	0.005299	0.0194155	[illegible]
ENSG00000188306	0.0176408	0	0	0.0228028	0.01968083	0.0132328	0.17000258	0.20735164	0.25292231	0.25564275	[illegible]
ENSG00000188312	0.79993392	0.55825849	0.51571999	0.4188021	0.63318393	0.48104593	0.39118626	0.48588337	0.6937478	0.28535497	[illegible]
ENSG00000188313	1.89031235	1.29031139	1.92593023	1.0386325	2.01314034	1.1771343	7.71991881	7.59970207	3.19496979	1.50683352	[illegible]
ENSG00000188314	0	0	0	0	0	0	0	0	0	0	[illegible]
ENSG00000188315	1.35948491	1.7397729	1.36558304	1.5572759	1.23103367	1.38182038	1.1746123	1.07901426	0.76865484	0.91764766	[illegible]
ENSG00000188316	0	0	0	0	0	0	0.02382592	0	0	0	[illegible]
ENSG00000188321	1.8056831	2.18161934	1.90320136	1.9482764	1.4747638	2.19682587	0.40602762	0.44450624	0.49924618	0.37646165	[illegible]
ENSG00000188322	3.18940133	4.47587545	2.92343938	4.0475642	2.26531162	4.24248088	0.09851261	0.16442337	0.12767713	0.24559909	[illegible]
ENSG00000188324	0	0	0	0	0	0	0	0	0	0	[illegible]
ENSG00000188334	0	0	0	0	0	0	0	0	0	0	[illegible]
ENSG00000188338	0.16558041	0.49800039	0.28894225	0.2308188	0.07787576	0.24841197	0.03910984	0.09540431	0.11075383	0.08821761	[illegible]
ENSG00000188340	0	0	0	0	0	0	0	0	0	0	[illegible]
ENSG00000188342	8.56617257	7.91213485	8.35860915	9.6573666	7.97299809	8.46098839	6.56021603	7.28010914	8.23054515	5.4239824	[illegible]
ENSG00000188343	1.75478099	1.58228301	1.896224	2.0872055	2.06744541	2.01636655	0.92136475	1.00971155	0.97725653	0.95645781	[illegible]
ENSG00000188352	1.75239319	1.57900438	1.5864904	1.773533	1.42819344	1.52035526	1.7281228	1.79649395	1.8583059	1.9871568	[illegible]
ENSG00000188365	0.42801779	0.77407137	0.50231246	0.7192419	0.19100595	0.96320037	0.03437303	0.04192468	0.08464332	0.07753308	[illegible]
ENSG00000188368	0.48841254	0.49623339	0.32738405	0.2411823	0.29387532	0.27992345	0.34375372	0.45690267	0.4151061	0.43739591	[illegible]
ENSG00000188372	0.24785046	0.15064249	0.20648318	0.4212347	0.19202259	0.29953584	0.0516036	0.00449577	0.04992172	0.07482793	[illegible]
ENSG00000188373	0	0	0	0	0	0	0	0	0	0	[illegible]
ENSG00000188375	0.1530377	0.10250708	0.14966812	0.0395638	0.11951476	0.36735163	0.07374043	0.08994099	0.03026421	0.02772194	[illegible]
ENSG00000188379	0	0	0	0	0	0	0	0	0	0	[illegible]
ENSG00000188383	0	0	0	0	0	0	0	0	0	0	[illegible]
ENSG00000188384	0	0	0	0	0	0	0	0	0	0	[illegible]
ENSG00000188385	0.00534224	0.01789159	0.00783692	0.0207164	0.00447002	0.03005511	0.17160877	0.0968062	0.10432574	0.15725414	[illegible]
ENSG00000188386	0	0	0	0	0	0	0	0	0	0	[illegible]
ENSG00000188388	0	0	0	0	0.01483981	0	0	0	0.00657619	0	[illegible]
ENSG00000188389	0	0	0	0	0	0	0	0	0	0	[illegible]
ENSG00000188393	0	0	0	0	0	0	0	0.01915612	0	0	[illegible]
ENSG00000188394	0.05509357	0.18451274	0.13470131	0	0.03073237	0.16530824	0.11061065	0.16189378	0.05447557	0.0498995	[illegible]
ENSG00000188396	0.01591592	0.01066074	0	0	0	0.02387786	0.03834503	0.12471817	0.20458605	0.05766164	[illegible]
ENSG00000188399	0	0	0	0	0	0	0	0	0	0	[illegible]
ENSG00000188403	0	0	0	0	0	0	0	0	0	0	[illegible]
ENSG00000188404	0.08278187	0.04066231	0.10794557	0.0428021	0.05849148	0.07451608	0.23046411	0.16757669	0.05184041	0.09497139	[illegible]
ENSG00000188408	0	0	0	0	0	0	0	0	0	0	[illegible]
ENSG00000188419	3.4972748	3.60476718	3.54123856	2.8779084	3.65683722	2.97815119	3.51753361	3.66316806	3.40888418	3.41138862	[illegible]
ENSG00000188424	0	0	0.00958058	0	0	0	0	0	0	0	[illegible]

ENSG00000188425	0.02063364	0	0	0	0	0.0154778	0	0	0	0	[illegible]	[illegible]
ENSG00000188428	1.51654858	1.38193972	1.53540943	1.7796908	1.66900034	1.56304501	1.61066713	2.06114191	1.94452167	1.35868277	[illegible]	[illegible]
ENSG00000188438	0	0	0	0	0	0	0	0	0	0	[illegible]	[illegible]
ENSG00000188439	0	0	0	0	0	0	0	0	0	0	[illegible]	[illegible]
ENSG00000188451	0	0	0	0	0	0	0	0	0	0	[illegible]	[illegible]
ENSG00000188452	0.00473851	0	0.00463417	0	0.00176216	0.00236965	0.00507383	0	0.00390446	0.00143059	[illegible]	[illegible]
ENSG00000188459	0	0	0	0	0	0.00716973	0	0	0	0	[illegible]	[illegible]
ENSG00000188460	0.2003625	0.07668913	0.13996495	0	0.15966639	0.12882591	0.09194633	0.16822	0.07075535	0.07777408	[illegible]	[illegible]
ENSG00000188467	0.0090292	0	0.0176608	0.0058356	0.02266507	0.01015954	0	0	0	0	[illegible]	[illegible]
ENSG00000188474	0	0	0	0	0	0	0	0	0	0	[illegible]	[illegible]
ENSG00000188477	0.05414488	0.05440065	0.05295272	0.0524914	0.05285554	0.06092313	0.02174119	0.01325884	0.02007657	0.03678018	[illegible]	[illegible]
ENSG00000188483	2.81087161	0.48664724	1.46767681	0.8159709	2.06625768	1.04531801	16.2700667	14.6763351	13.5257355	6.63436506	[illegible]	[illegible]
ENSG00000188486	66.8872303	48.7121046	53.2686636	49.22016	73.6832947	37.6930586	13.1364129	18.5061337	28.2781028	15.1770818	[illegible]	[illegible]
ENSG00000188487	0	0	0	0	0.00112809	0	0.00162408	0.00198088	0.00099982	0.00366333	[illegible]	[illegible]
ENSG00000188488	0	0	0	0	0	0	0	0	0	0.00187255	[illegible]	[illegible]
ENSG00000188493	0.41412784	0.41930954	0.35085133	0.7158196	0.35591553	0.617683	0.3364428	0.35611552	0.33091897	0.48848276	[illegible]	[illegible]
ENSG00000188501	0.20725214	0.25586573	0.27025183	0.2889092	0.30602556	0.19204498	1.16180933	1.17424532	1.01860944	2.28936203	[illegible]	[illegible]
ENSG00000188505	0.06517642	0.09822645	0.09561205	0.0631861	0.08180279	0.20778443	0.0915977	0.07980103	0.06444532	0.08854764	[illegible]	[illegible]
ENSG00000188508	0	0	0	0.0291946	0	0	0	0	0	0	[illegible]	[illegible]
ENSG00000188511	0	0.00187461	0.00273707	0.0036176	0.00156117	0.00419874	0.00224756	0.00548268	0.00138365	0.00760452	[illegible]	[illegible]
[illegible]	[illegible]	[illegible]	[illegible]	[illegible]	[illegible]	[illegible]	[illegible]	[illegible]	[illegible]	[illegible]	[illegible]	[illegible]

ENSG00000189089	0	0	0	0	0	0	0	0	0	0
ENSG00000189090	0	0	0	0	0	0	0	0	0	0
ENSG00000189091	16.0977831	15.853365	13.2328222	14.054933	16.5980662	14.6644298	12.6726568	13.6197067	15.5872582	16.3753327
ENSG00000189099	0	0	0	0	0	0.0312154	0	0	0	0
ENSG00000189108	0.01158302	0	0.02265596	0	0.00969188	0.00434435	0.00465101	0.01134566	0.00572654	0.010491
ENSG00000189114	1.7716933	1.90883285	1.52623674	1.9782743	1.50135703	1.71355036	1.48334706	1.39569479	1.72945613	1.48858081
ENSG00000189120	0	0.00847363	0.0164962	0.0218033	0.00235227	0.0094896	0.01015945	0.00826097	0.00833919	0.00381934
ENSG00000189127	0.00719414	0	0.00703574	0	0	0.0053965	0	0	0	0
ENSG00000189129	0	0	0.01421105	0	0	0	5.49632211	5.30899188	2.55750295	3.8298652
ENSG00000189132	0	0	0	0	0	0	0	0	0	0
ENSG00000189134	0	0	0	0	0	0	0	0	0.00972185	0.01781038
ENSG00000189136	0.4661326	0.83935275	0.80517177	0.5868807	0.32080228	0.6947743	0.48129346	0.58703219	0.51776864	0.60860704
ENSG00000189139	0	0	0	0	0	0.00822884	0	0	0	0
ENSG00000189143	0.0028608	0.00383241	0.00559562	0.0110937	0.00478744	0.00429191	0.76964043	0.73977381	0.54876912	0.23578908
ENSG00000189144	0.3842343	0.49608272	0.38394325	0.4714744	0.28344904	0.41667122	0.33316496	0.40226943	0.30834432	0.31522619
ENSG00000189145	0	0	0	0	0	0	0	0	0	0
ENSG00000189149	0	0.01524576	0.02225996	0	0.01269663	0.01707368	0.05483664	0.09661034	0.05251353	0.03435876
ENSG00000189152	0	0.0327256	0	0	0	0.01221644	0	0	0.0040258	0
ENSG00000189157	0.06296855	0.08163354	0.09932598	0.0656405	0.07704882	0.07618431	0.07993078	0.09152248	0.07933411	0.11222434
ENSG00000189159	13.6173789	8.85984588	11.7520709	10.838274	12.9548281	10.0667437	4.13557348	5.04414662	5.66050895	4.85504829

ENSG00000196301	0	0	0	0	0	0	0	0	0	0	0	0	0	0	0	0	0
ENSG00000196302	0	0	0	0	0	0	0	0	0	0	0	0	0	0	0	0	0
ENSG00000196305	5.8845813	4.9134598	4.75890557	6.5256368	7.51468795	4.94371496	14.3927502	16.4869628	17.2271216	16.5414869	13.8392397	14.8205781	15.263111	13.9705297	15.228797	21.9504827	17.103559
ENSG00000196312	0.80863146	1.26012848	0.90380234	1.1519088	0.80088267	0.91605139	0.78192046	0.92137704	0.97089531	0.91175811	1.01873196	0.8919362	1.42473958	1.35872829	1.37782335	1.44869772	1.31577939
ENSG00000196313	0.81641252	0.85009704	0.71980286	0.7483097	0.98948669	0.77757682	0.54338677	0.6651905	0.60666408	0.53553623	0.41453066	0.51918409	0.40518881	0.38460249	0.37222846	0.39736699	0.41497625
ENSG00000196323	2.10504893	2.37429019	2.31109606	2.0149761	1.49743204	2.00199537	2.70848124	3.18677616	3.10780646	3.29217778	2.45612441	2.83418619	1.65716209	1.53042693	1.66128965	1.43078288	1.42511897
ENSG00000196326	0	0	0	0	0	0	0	0	0	0	0	0	0.00750427	0.02256375	0.01925323	0.01545635	0.04286088
ENSG00000196329	0.00294362	0	0.00287881	0	0	0	0	0	0.0014553	0	0.00370844	0	0	0	0	0	0
ENSG00000196331	1001.19237	624.156421	757.832457	502.51549	898.459778	457.797175	67.3398599	112.68951	171.010143	39.0674602	73.904033	171.584078	197.09053	105.943481	135.288718	170.968762	102.784533
ENSG00000196335	0.01139665	0.01866006	0.01362255	0.0163683	0.01130186	0.01424819	0.01525394	0.00992277	0.013773	0.01376294	0.01435776	0.01069385	0.00643083	0.00411548	0.00881034	0.00732939	0.00952484
ENSG00000196337	0	0	0	0.0112881	0.00162377	0.00218356	0.01636382	0.01995891	0.06620022	0.06063925	0.04400695	0.03933236	0.01231917	0.00946055	0.01012648	0.0084243	0.00875817
ENSG00000196338	8.93812067	8.63683199	8.48855558	5.9030508	7.96127832	6.18872509	0.06210456	0.07176197	0.04225753	0.06266976	0.04614936	0.03265403	0.03445043	0.03527511	0.01592921	0.01374244	0.00306152
ENSG00000196341	0	0	0	0	0	0	0	0	0	0	0	0	0	0.03403094	0	0	0
ENSG00000196344	0.01482679	0.02482805	0.02537559	0.0335394	0.02687978	0.00278049	0	0	0.00183256	0.00335724	0.00466978	0.0031303	0.00313739	0	0	0	0
ENSG00000196345	0.49113395	0.75547222	0.55514393	0.6476087	0.43228644	0.56835386	0.67387882	0.87269412	0.60886122	0.38671251	0.79285972	0.73573403	0.09400266	0.11229506	0.04507481	0.12856491	0.14108574
ENSG00000196350	0.03450722	0.01733511	0.0421843	0.0167267	0.02887329	0.02588472	0.00346398	0	0.00426502	0	0	0.01092799	0.00730181	0.00934576	0.00375135	0.00416104	0
ENSG00000196352	0.03445496	0.03942573	0.06458463	0.1002084	0.03203285	0.09369001	1.52645656	1.4680773	1.25698444	1.28207258	1.24976338	1.08265771	1.78380456	2.23958431	2.57702065	2.85430432	2.70610211
ENSG00000196353	0.00578865	0	0.0014153	0.0056119	0.01372338	0.00759886	0.00116218	0	0.00214639	0.01048582	0	0.00122213	0	0.00156777	0.00251718	0	0
ENSG00000196355	0.45896781	0.72870854	0.71485474	0.3735401	0.3129158	0.58655651	0.34128287	0.31635889	0.25212192	0.67743306	0.40689407	0.2153322	0.30214716	0.23940118	0.14783805	0.1475852	0.05114476
ENSG00000196357	0.53775588	0.62497829	0.61995348	0.7319394	0.52445379	0.6518259	0.31745868	0.27906552	0.29403266	0.39996791	0.28714251	0.21954843	0.30143174	0.26235032	0.2973358	0.32293707	0.21965684

ENSG00000196781	12.0325256	[illegible]
ENSG00000196782	0.72217099	[illegible]
ENSG00000196787	279.386788	[illegible]
ENSG00000196792	3.97050787	[illegible]
ENSG00000196793	0.93223579	[illegible]
ENSG00000196796	0	[illegible]
ENSG00000196800	0	[illegible]
ENSG00000196805	0	[illegible]
ENSG00000196810	1.50314653	[illegible]
ENSG00000196811	0	[illegible]
ENSG00000196812	3.93163994	[illegible]
ENSG00000196814	7.087795	[illegible]
ENSG00000196821	6.62384156	[illegible]
ENSG00000196826	0	[illegible]
ENSG00000196832	0	[illegible]
ENSG00000196834	0.05050244	[illegible]
ENSG00000196839	10.5093275	[illegible]
ENSG00000196843	1.30850834	[illegible]
ENSG00000196844	0.04037128	[illegible]
ENSG00000196850	2.08800505	[illegible]
ENSG00000196859	0	[illegible]
ENSG00000196860	0	[illegible]
ENSG00000196861	0	[illegible]
ENSG00000196862	0.0029713	[illegible]
ENSG00000196865	5.76929841	[illegible]
ENSG00000196866	196.658819	[illegible]
ENSG00000196867	2.2451385	[illegible]
ENSG00000196872	1.12433654	[illegible]
ENSG00000196873	0.01971439	[illegible]
ENSG00000196876	0.20450071	[illegible]
ENSG00000196878	0.00695156	[illegible]
ENSG00000196890	215.126383	[illegible]
ENSG00000196893	0	[illegible]
ENSG00000196900	0	[illegible]
ENSG00000196911	5.02618128	[illegible]
ENSG00000196912	0.14010949	[illegible]
ENSG00000196914	6.01906827	[illegible]
ENSG00000196917	0.10029259	[illegible]
ENSG00000196922	2.23727038	[illegible]
ENSG00000196923	1.73328003	[illegible]
ENSG00000196924	12.1194988	[illegible]
ENSG00000196927	0.0169809	[illegible]
ENSG00000196932	0.17044355	[illegible]
ENSG00000196933	0	[illegible]
ENSG00000196934	0.00557222	[illegible]
ENSG00000196935	0.50203545	[illegible]
ENSG00000196936	0.03432347	[illegible]
ENSG00000196937	39.6379052	[illegible]
ENSG00000196943	7.73819612	[illegible]
ENSG00000196944	0	[illegible]
ENSG00000196946	0	[illegible]
ENSG00000196950	6.46259736	[illegible]
ENSG00000196951	0.02265711	[illegible]
ENSG00000196954	0.58452548	[illegible]
ENSG00000196960	0	[illegible]
ENSG00000196961	5.69793401	[illegible]
ENSG00000196963	1.3323054	[illegible]
ENSG00000196966	0.48832939	[illegible]
ENSG00000196967	0.47920888	[illegible]
ENSG00000196968	4.73253276	[illegible]
ENSG00000196970	0.0634194	[illegible]
ENSG00000196972	0.36675221	[illegible]
ENSG00000196975	0.44491736	[illegible]
ENSG00000196976	4.28239339	[illegible]
ENSG00000196979	0.00989249	[illegible]
ENSG00000196981	4.72465715	[illegible]
ENSG00000196987	0	[illegible]
ENSG00000196990	0	[illegible]
ENSG00000196993	0	[illegible]
ENSG00000196998	0.38956483	[illegible]
ENSG00000197006	13.1082301	[illegible]
ENSG00000197008	2.65747954	[illegible]
ENSG00000197013	1.68728973	[illegible]
ENSG00000197015	0.11190882	[illegible]
ENSG00000197016	0.87404912	[illegible]
ENSG00000197019	4.66523196	[illegible]
ENSG00000197020	3.65881861	[illegible]
ENSG00000197021	1.35699929	[illegible]
ENSG00000197023	0	[illegible]
ENSG00000197024	2.18130482	[illegible]
ENSG00000197037	1.1541001	[illegible]
ENSG00000197038	0	[illegible]
ENSG00000197043	6.74123219	[illegible]
ENSG00000197044	1.57002611	[illegible]
ENSG00000197045	8.33298468	[illegible]
ENSG00000197046	0.04673009	[illegible]
ENSG00000197047	0.09678601	[illegible]
ENSG00000197050	1.80478229	[illegible]
ENSG00000197054	0.11172112	[illegible]
ENSG00000197056	2.05347748	[illegible]
ENSG00000197057	0.03377874	[illegible]
ENSG00000197061	1115.69263	[illegible]
ENSG00000197062	3.22744274	[illegible]
ENSG00000197063	4.37005991	[illegible]
ENSG00000197067	0	[illegible]
ENSG00000197070	1.00943856	[illegible]
ENSG00000197071	0	[illegible]
ENSG00000197077	0.1422427	[illegible]
ENSG00000197079	0	[illegible]
ENSG00000197081	10.7725249	[illegible]
ENSG00000197083	0.69851775	[illegible]
ENSG00000197084	0	[illegible]
ENSG00000197085	0	[illegible]
ENSG00000197092	0	[illegible]
ENSG00000197093	1.19311372	[illegible]
ENSG00000197099	0.02679114	[illegible]
ENSG00000197102	20.5826374	[illegible]
ENSG00000197106	0.00462539	[illegible]
ENSG00000197110	0.0491307	[illegible]
ENSG00000197111	1.3213745	[illegible]
ENSG00000197114	0.13865762	[illegible]
ENSG00000197119	0.77551012	[illegible]
ENSG00000197121	1.99771477	[illegible]
ENSG00000197122	0.01752064	[illegible]
ENSG00000197123	0.25955323	[illegible]
ENSG00000197124	0.67302246	[illegible]
ENSG00000197125	0	[illegible]
ENSG00000197128	2.2359318	[illegible]
ENSG00000197134	0.07386044	[illegible]
ENSG00000197136	4.69575874	[illegible]
ENSG00000197140	0.29238424	[illegible]
ENSG00000197142	0	[illegible]
ENSG00000197146	0.07012563	[illegible]
ENSG00000197147	1.97358984	[illegible]
ENSG00000197149	0	[illegible]
ENSG00000197150	1.01741265	[illegible]
ENSG00000197153	0.4834461	[illegible]
ENSG00000197157	10.4243103	[illegible]
ENSG00000197161	0	[illegible]
ENSG00000197162	1.63052607	[illegible]
ENSG00000197165	0.01919579	[illegible]
ENSG00000197168	0.06474199	[illegible]
ENSG00000197170	4.1463065	[illegible]
ENSG00000197171	0	[illegible]
ENSG00000197172	0	[illegible]
ENSG00000197176	0	[illegible]
ENSG00000197177	0	[illegible]
ENSG00000197180	1.26391136	[illegible]
ENSG00000197181	2.11477422	[illegible]
ENSG00000197182	0.82216717	[illegible]
ENSG00000197183	1.59696909	[illegible]
ENSG00000197185	0	[illegible]
ENSG00000197191	0.04748979	[illegible]
ENSG00000197208	0.42933049	[illegible]
ENSG00000197210	0	[illegible]
ENSG00000197213	0	[illegible]
ENSG00000197214	0	[illegible]
ENSG00000197217	3.67542465	[illegible]
ENSG00000197223	2.08550606	[illegible]
ENSG00000197226	2.99523695	[illegible]
ENSG00000197233	0	[illegible]
ENSG00000197238	32.8329702	[illegible]
ENSG00000197241	0.020942	[illegible]
ENSG00000197245	0	[illegible]
ENSG00000197249	0	[illegible]
ENSG00000197251	0.01529651	[illegible]
ENSG00000197253	0	[illegible]
ENSG00000197254	0	[illegible]
ENSG00000197256	1.32540278	[illegible]
ENSG00000197258	0.12341804	[illegible]
ENSG00000197261	0.02573918	[illegible]
ENSG00000197262	0	[illegible]
ENSG00000197263	0	[illegible]
ENSG00000197265	4.80001974	[illegible]
ENSG00000197272	0	[illegible]
ENSG00000197273	0	[illegible]
ENSG00000197275	1.04517808	[illegible]
ENSG00000197279	0.29063363	[illegible]
ENSG00000197283	0.72292398	[illegible]
ENSG00000197284	0	[illegible]
ENSG00000197291	0.0175241	[illegible]
ENSG00000197296	3.54057398	[illegible]
ENSG00000197299	2.09892955	[illegible]
ENSG00000197301	0.05328698	[illegible]
ENSG00000197302	0.80434909	[illegible]
ENSG00000197308	0	[illegible]
ENSG00000197309	0	[illegible]
ENSG00000197312	1.24536881	[illegible]
ENSG00000197320	0	[illegible]
ENSG00000197321	1.44371117	[illegible]
ENSG00000197322	0.00806348	[illegible]

ENSG00000197323	3.50767386	4.78838008	3.6350762	3.1391409	3.53282406	3.22318102	3.35278857	3.78760435	3.83161395	2.74124557	3.6012421	3.93340454
ENSG00000197324	6.7646399	6.31106319	6.31240499	8.2291538	6.13064212	7.02424214	9.9822155	9.34755149	6.06176601	7.12938637	8.31577916	4.98879388
ENSG00000197329	14.7602199	12.5066246	13.4338773	11.569526	14.3975081	14.3599543	1.78311557	2.09034542	2.50827789	2.98007674	2.50738816	2.29771722
ENSG00000197332	0.08555986	0.1146187	0.083676	0.1616405	0.06608369	0.11355025	0.02114184	0.06446659	0.05206161	0.03576623	0.03316622	0.05002285
ENSG00000197334	0	0	0	0.1827224	0.07885279	0	0	0	0	0	0	0
ENSG00000197343	3.14974301	3.57585209	3.38908041	3.1171887	2.97878516	3.25998465	1.78147361	1.73149625	1.41209506	1.83818501	1.72066302	1.50778563
ENSG00000197345	2.59565323	2.26536883	2.60963365	2.8792305	3.02768003	2.40740318	5.34450703	5.6459599	5.83426485	3.58199569	5.33174708	5.57412164
ENSG00000197353	0	0	0	0	0	0	0	0	0	0	0	0
ENSG00000197355	2.5538665	2.38112397	2.24271945	3.4624657	2.65121205	3.95832012	2.10216387	2.03436254	1.82194151	1.25072879	1.31351701	1.21301894
ENSG00000197358	6.06636672	2.3509374	4.84794326	4.7609031	8.15531591	7.1963422	2.83953219	2.63148129	2.83206338	3.02194526	4.18156343	3.49098724
ENSG00000197360	0.07187721	0.09628899	0.08435354	0	0	0	0	0	0	0.00651008	0	0
ENSG00000197361	0.05704379	0.07641767	0.1115756	0.0632021	0.05909473	0.11003133	0.02617739	0.0399106	0.01208655	0.02214251	0.02053287	0.01376384
ENSG00000197362	1.66534495	1.93665351	1.7672883	1.6396802	1.65830112	1.33958553	0.83373874	0.86072814	0.84960875	0.94030601	0.70529812	0.89769098
ENSG00000197363	0.32239153	0.36568442	0.36822554	0.3741433	0.27303698	0.34598135	0.48757201	0.49788011	0.3024874	0.48808311	0.47434282	0.26629751
ENSG00000197364	0	0	0	0	0	0	0	0	0	0	0	0
ENSG00000197372	2.64784721	2.86841201	2.86497328	2.0485945	2.50586086	2.3341663	0.5033339	0.55171203	0.70982051	0.44013127	0.50089431	0.76246748
ENSG00000197375	0.31124337	0.4076028	0.28391574	0.4023184	0.28962315	0.41982973	0.32953276	0.37048661	0.2573801	0.37418141	0.32881086	0.25577273
ENSG00000197376	0	0.01969108	0.01916698	0.0253334	0.00546623	0.02205199	0	0.00959845	0	0	0	0.00827546
ENSG00000197380	2.48067861	2.26744199	2.11253764	2.6293848	2.13314291	2.67099056	0.31568241	0.46353956	0.39434317	1.00933478	0.38464175	0.28039848
ENSG00000197381	0.02687547	0.0423038	0.0394256	0.0460301	0.02810946	0.02771995	0.13651258	0.13886301	0.11758997	0.14909307	0.20145701	0.13334086
ENSG00000197385	0.14097207	0.05311425	0.07755084	0.0683336	0.07863722	0.06609166	0.21934655	0.37109878	0.32669708	0.1356617	0.31079958	0.26786431
ENSG00000197386	2.36218454	2.64643459	2.3790203	2.063825	2.33920361	2.07703449	2.09487798	2.20460194	2.33278707	2.18730371	2.94267526	2.61990912
ENSG00000197403	0	0	0	0	0	0	0	0	0	0	0	0
ENSG00000197405	0.07368903	0.13573472	0.15314142	0.1309711	0.0565198	0.11746145	0.55479193	0.32480547	0.29144977	0.30868095	0.44096665	0.31893044
ENSG00000197406	0	0	0	0	0	0.00575079	0.0615673	0.03003738	0.0303218	0.00694367	0.01931672	0.00647431
ENSG00000197408	0.00370712	0.01241545	0.02175301	0.0047919	0.00827165	0.00556161	0.00893129	0.00726232	0.00183277	0.00671525	0.00934064	0.00626133

[illegible]

ENSG00000197849	0	0	0	0	0	0	0	0	0	0
ENSG00000197851	0	0	0	0	0	0	0	0	0	0
ENSG00000197852	11.9910693	9.75577381	9.9831151	7.894998	12.8950023	8.77778669	1.10557585	0.89409262	0.45867734	0.25976853
ENSG00000197857	0.73996337	0.75898972	0.78874211	0.9434579	0.55785447	1.06929402	0.33031655	0.39301145	0.31300026	0.26296739
ENSG00000197858	4.03988367	2.74505361	3.03629965	3.4782056	3.82978597	3.17947654	8.50318686	9.44096496	6.46863934	7.08190982
ENSG00000197859	0.01068714	0.00894802	0.02090366	0.0034536	0.00596151	0.01603338	0.02145643	0.00523407	0.0171718	0.01693927
ENSG00000197860	3.45825716	3.05003391	3.40194983	3.3428191	3.43954956	3.71621677	2.7039397	2.63739637	2.62977921	3.17355625
ENSG00000197863	1.60692833	1.88933086	1.90591875	1.4289562	1.54482309	1.85083993	1.35455025	1.13863764	1.26774177	1.74445327
ENSG00000197866	0	0	0	0	0	0	0	0	0	0
ENSG00000197870	0	0	0	0	0	0	0	0	0	0
ENSG00000197872	20.2673239	17.7315993	19.7783686	16.582427	17.5176057	16.4806347	0.18120363	0.17467198	0.16193197	0.05603551
ENSG00000197879	0.69230905	0.51575629	0.54723075	0.564391	0.66485212	0.52669492	4.16666082	4.85570193	4.30027347	5.85356098
ENSG00000197880	0.01146966	0.05377791	0.04486848	0.0444776	0.01279604	0.02581103	0.02763298	0.02246924	0.0170115	0.02077666
ENSG00000197882	0.045394	0.01520281	0.04439452	0.1466926	0.02532174	0.01702558	0	0	0	0
ENSG00000197885	1.94693938	1.70647925	1.86976415	1.6125809	1.50064896	1.64135896	2.46327665	2.87989208	2.49851109	1.56877911
ENSG00000197887	0	0	0	0	0	0	0	0	0	0
ENSG00000197888	0	0	0	0	0	0	0	0	0	0
ENSG00000197889	0.1700137	0.13014614	0.08313517	0.1255786	0.1354818	0.15485968	0.14628613	0.08326489	0.08405331	0.31896947
ENSG00000197891	0	0	0	0	0	0	0	0	0	0
ENSG00000197892	1.21951225	0.99447067	0.99543718	1.0406452	0.97080183	0.92788888	2.57719496	2.79966085	2.12904833	3.29923768

[illegible]

ENSG00000198356	19.4103765	16.7507598	17.7801776	16.998545	20.7446864	15.1575177	12.599549	13.2825767	13.8526934	10.4896972	[illegible]
ENSG00000198358	0.24142127	0.31330885	0.23610567	0.1950407	0.13466993	0.26032564	0.14540951	0.26603332	0.14919574	0.35532367	[illegible]
ENSG00000198363	1.96253658	1.54449245	1.56333973	1.8503541	2.11596494	1.73542105	10.162257	10.3231823	9.25214679	8.87646433	[illegible]
ENSG00000198366	0	0.04602983	0	0	0.0766671	0	23.5098343	38.9062853	75.5256214	14.8756893	[illegible]
ENSG00000198367	0	0	0	0	0	0	0	0	0	0	[illegible]
ENSG00000198369	4.93137731	3.96790559	4.17468735	2.919748	5.80710914	3.37007623	2.85838683	3.21208884	2.95178573	3.89159724	[illegible]
ENSG00000198373	0.8382372	0.84658787	0.6975404	0.8112273	0.82400551	0.72582097	0.62403091	0.62756725	0.74370394	0.57712661	[illegible]
ENSG00000198374	529.062709	328.981385	388.747982	255.70801	477.165863	213.578672	42.099225	68.8684268	124.302708	26.6949251	[illegible]
ENSG00000198380	4.45522599	5.04646738	4.33495296	6.7848589	4.93638238	5.47356494	5.0908439	5.08840313	5.17366847	6.49202568	[illegible]
ENSG00000198382	1.94279203	1.59630684	1.80249566	2.01934	1.87821943	2.00461701	1.11710314	1.15415997	1.25546915	1.10183896	[illegible]
ENSG00000198384	0	0	0	0	0.00473241	0	0.00681308	0.01661979	0	0.00768392	[illegible]
ENSG00000198388	0	0.23986656	0	0	0	0	0.2875877	0	0.17704562	0.32434674	[illegible]
ENSG00000198390	0	0	0	0	0	0	0	0	0	0	[illegible]
ENSG00000198393	1.43176445	1.31159887	1.38258984	1.3569375	1.27350744	1.43501124	2.01459006	2.25389682	2.18155228	1.33492217	[illegible]
ENSG00000198398	0	0	0	0	0	0	0	0	0.01100422	0	[illegible]
ENSG00000198399	1.77895835	1.95357501	1.76416853	1.5408191	1.57510407	1.63702751	1.737113	1.75138288	1.72595201	2.22905709	[illegible]
ENSG00000198400	0.00348524	0.0035017	0.0034085	0.0022525	0.00486035	0	0.02519016	0.02560364	0.03963065	0.10416975	[illegible]
ENSG00000198406	0.02327057	0	0	0	0	0	0	0	0.01150477	0	[illegible]
ENSG00000198408	9.73006163	11.7585307	9.79905803	11.873595	7.85687078	12.6433412	5.67554402	6.13708617	5.91944262	6.48356145	[illegible]
ENSG00000198414	0	0	0	0	0	0	0	0	0	0	[illegible]
ENSG00000198416	0	0	0	0	0	0	0	0	0	0	[illegible]
ENSG00000198417	0.06560284	0.15065768	0.04582743	0.0484568	0.06273371	0.03515023	0.43652451	0.33965211	0.41236853	0.57720326	[illegible]
ENSG00000198420	16.6839234	14.8455399	15.4873343	12.46363	15.0558011	14.5098792	5.07789727	6.18461338	5.03937382	7.75365749	[illegible]
ENSG00000198429	2.55944411	3.54156977	3.38191834	1.9601376	2.29597639	2.03425425	0.19974351	0.31435685	0.24593342	0.03633459	[illegible]
ENSG00000198431	3.14001264	2.32719722	2.56881808	2.6181212	3.39777445	2.55844633	2.32412952	2.39642838	2.62921834	1.97124311	[illegible]
ENSG00000198434	0	0	0	0	0	0.08855798	0	0	0	0	[illegible]
ENSG00000198435	2.61920434	2.7674799	2.21278124	2.7816188	3.97158103	2.80412214	0.20737915	0.16862648	0.52890774	0.47890953	[illegible]
ENSG00000198440	0.95275999	1.0876843	0.97489805	0.7313339	0.90855379	0.85983146	0.43862642	0.42223531	0.47951256	0.30391331	[illegible]
ENSG00000198443	0	0	0	0	0	0	0	0	0	0.02352233	[illegible]
ENSG00000198444	0	0	0	0	0	0	0	0	0	0	[illegible]
ENSG00000198445	0	0	0.01549661	0	0	0.0118861	0	0	0	0	[illegible]
ENSG00000198452	0	0	0	0	0	0	0	0	0	0	[illegible]
ENSG00000198453	0.79094779	0.95121273	0.81951195	0.9991562	0.66489664	0.98331733	0.76178412	0.82891726	0.73422127	0.86040667	[illegible]
ENSG00000198454	0	0	0	0	0	0.02472017	0	0	0	0	[illegible]
ENSG00000198455	2.72678004	3.53035248	3.13076618	3.1330503	2.10460573	2.93306302	2.20817158	2.31814203	2.2072608	2.13316371	[illegible]
ENSG00000198464	2.47446488	2.84996307	2.64840439	2.4209747	2.46685712	2.51564732	1.42270087	1.42878703	1.72990767	1.64047115	[illegible]
ENSG00000198466	1.56566758	2.03273378	1.68793125	1.9706899	1.45904295	1.82640254	2.19128404	2.19370921	1.78783792	2.42670744	[illegible]
ENSG00000198467	0.95181598	1.78232386	1.04976428	1.0686837	0.59876443	1.9386925	20.0775144	21.4567712	19.8394898	16.9773388	[illegible]
ENSG00000198468	1.03325923	0.91036902	1.10378672	0.9554736	1.27245644	1.0433681	0.7659549	0.70845995	0.70337975	0.88545459	[illegible]
ENSG00000198471	0	0	0	0	0	0	0	0	0	0	[illegible]
ENSG00000198477	0.9506306	0.82306261	0.92073149	0.7112032	1.18333023	0.74060645	0.80515827	1.01198966	1.03366162	1.0049704	[illegible]
ENSG00000198478	2.94636253	2.89759219	2.7255502	3.6113708	1.87557345	3.16700034	4.1866929	4.71264467	4.98690545	3.13323552	[illegible]
ENSG00000198482	1.51210641	1.59250447	1.40750747	1.4464668	1.79483737	1.45528805	0.58807398	0.60548942	0.50778501	0.07465047	[illegible]
ENSG00000198483	0	0.03075943	0.00748518	0.0197866	0.0085388	0.02296493	0.17210187	0.0599749	0.08703026	0.05545705	[illegible]
ENSG00000198488	0.00496453	0.00997597	0.01942089	0.0320862	0.01384661	0.01489607	0.01196067	0.01945119	0.00490884	0.00899298	[illegible]
ENSG00000198491	0	0	0	0	0	0	0	0	0	0	[illegible]
ENSG00000198492	5.47641198	5.74261085	5.23633122	4.6149272	5.55575406	4.90459346	6.48009003	6.99411924	7.60404026	5.94609599	[illegible]
ENSG00000198496	0.38206296	0.49354424	0.31137559	0.6584813	0.25371768	0.46401084	0.85472719	0.72173645	0.69708901	0.73328178	[illegible]
ENSG00000198498	2.46844588	1.947632	1.93888004	1.8089317	2.95773554	1.89829821	3.32784508	3.99804019	4.0416625	3.20395582	[illegible]
ENSG00000198502	0.02557916	0	0.07504787	0	0	0	0.06162593	0.02505499	0	0	[illegible]
ENSG00000198513	1.94927532	1.95848326	1.99473038	1.5991258	1.78224139	1.63972968	0.02764528	0.04847087	0.06169413	0.15979165	[illegible]
ENSG00000198515	0	0.0013344	0.00194833	0	0	0.0014944	0	0	0	0.00360875	[illegible]
ENSG00000198517	0.7994384	0.65340884	0.80510534	0.5105327	0.57601069	0.67820797	1.17701623	0.9648372	0.997499	1.09041417	[illegible]
ENSG00000198518	561.149735	469.435375	490.026924	441.85772	505.514293	372.236543	46.6773813	69.4121012	136.30502	67.3324488	[illegible]
ENSG00000198520	0	0.00403791	0.00786087	0.0025975	0.00448368	0.01205877	0.03550244	0.03542909	0.00794768	0.03640028	[illegible]
ENSG00000198521	2.85007452	3.11008871	2.88143498	2.742827	2.87726534	2.67329206	0.8797221	1.1311215	1.27901031	0.90645995	[illegible]
ENSG00000198522	3.4353877	3.1452505	3.20811476	3.292544	2.98384445	3.08538545	2.34975611	2.65988218	2.84604083	3.07740433	[illegible]
ENSG00000198523	0.93419536	0.30208083	1.11840458	0.1040997	0.49415988	0.26580672	0.06467489	0.04733027	0.02388921	0.04376493	[illegible]
ENSG00000198526	0	0	0	0	0	0	0	0.00921462	0	0	[illegible]
ENSG00000198535	4.24740257	0.52011704	1.82990459	0.4353513	8.21423997	0.98249234	0.17280306	0.09163799	0.14800911	0.033894	[illegible]
ENSG00000198538	1.27817953	1.27150234	1.33667282	1.2289232	1.16391262	1.21629222	0.43574321	0.51288083	0.58421404	0.56164488	[illegible]
ENSG00000198542	0.00368635	0	0.00360518	0	0.00205632	0	2.53115329	2.51673258	2.27994578	2.1735646	[illegible]
ENSG00000198546	3.23914274	2.48370192	2.71979515	2.8911691	3.27399929	2.38933289	3.67034999	3.95935872	4.66831986	4.58370279	[illegible]
ENSG00000198547	0.00326609	0.01531373	0	0.021109	0.01457514	0.00734992	0.01573747	0.00959747	0.0129178	0.01479084	[illegible]
ENSG00000198551	3.78679449	4.52383574	4.81796171	4.6467706	3.66483483	4.83526588	2.06806239	2.61641236	2.65897914	2.31429042	[illegible]
ENSG00000198553	0.06138998	0.05482664	0.04002553	0.0925793	0.03995208	0.02302508	0.05751754	0.07015397	0.07081825	0.06486935	[illegible]
ENSG00000198554	3.7450871	2.65023662	2.80236656	2.015632	3.62709769	2.22443225	0.63416164	1.07700451	1.53361441	0.83291357	[illegible]
ENSG00000198555	0.08007389	0.02681738	0.05220722	0.0172508	0.02977795	0.05005451	0.01071755	0.02614434	0.01319595	0	[illegible]
ENSG00000198556	0.88836329	1.23306218	0.98059067	0.5495314	0.72598335	0.84839782	0.6473944	0.56570151	0.48480448	0.72469458	[illegible]
ENSG00000198558	0.35417296	0.11861533	0	0	0.19756522	0.06641849	7.82175327	15.4377282	39.0473134	13.3926688	[illegible]
ENSG00000198561	1.16303047	1.52010458	1.25052752	1.0273723	0.95840447	1.06548788	0.85559296	0.92944272	0.8779278	0.72157828	[illegible]
ENSG00000198563	0.82007779	1.26515605	0.87831351	1.0688695	0.73704403	1.56898906	2.80818265	2.75844428	2.25619066	2.53740577	[illegible]
ENSG00000198566	0	0	0	0	0	0	0	0	0	0	[illegible]
ENSG00000198569	0.01503954	0.01007372	0.0073542	0.0097202	0	0.03948537	0.04227257	0.03682838	0.02602397	0.02043248	[illegible]
ENSG00000198570	0.00878673	0.01177099	0.03007644	0.0056789	0.00245071	0.0131823	0.0070564	0.01291001	0.00868817	0.01591669	[illegible]
ENSG00000198573	0.08328098	0	0.0814473	0	0.0464559	0	0	0	0	0	[illegible]
ENSG00000198574	0	0	0.00612517	0	0	0	0.01005942	0	0	0	[illegible]
ENSG00000198576	48.3631112	27.3877492	31.8675154	15.095428	58.5176386	27.3239709	0.5348933	0.40594261	0.5049154	0.54293633	[illegible]
ENSG00000198580	1.00993296	1.82964463	1.239592	0.9462368	0.82424791	1.21008757	0.11975261	0.12614436	0.02680817	0.07980771	[illegible]
ENSG00000198585	4.83936407	5.67067906	5.22191625	5.9744658	5.30145028	6.22675339	1.92940427	1.9786835	2.04994044	2.5446087	[illegible]
ENSG00000198586	2.1438478	2.78738631	2.12042857	2.2730394	2.47733724	2.08456553	0.84430947	1.0059807	1.05851256	0.87344034	[illegible]
ENSG00000198589	0.96331694	1.14847081	0.91565785	1.0969782	0.84631732	1.02893536	1.73922336	1.9088751	1.92213654	3.1573552	[illegible]
ENSG00000198590	0.04424659	0.05927414	0.05090868	0.0605582	0.01742237	0.05661904	0.03553333	0.04079049	0.03474286	0.0306457	[illegible]
ENSG00000198597	3.71505855	3.1910136	3.02981242	3.5592384	4.56201354	3.90146094	0	0.00251829	0	0.00232859	[illegible]
ENSG00000198598	0.09103667	0.11271655	0.11331374	0.1034118	0.07848157	0.10346799	1.73025243	1.48078141	1.20838975	1.60660324	[illegible]
ENSG00000198601	0	0	0	0	0.01722072	0	0	0	0	0	[illegible]
ENSG00000198604	2.78196524	2.83256437	2.46040982	2.271071	2.91335149	2.46382975	2.47288783	2.82757636	2.98593867	1.81956168	[illegible]
ENSG00000198610	0.00943494	0	0	0	0	0	0.00757696	0	0	0	[illegible]
ENSG00000198612	10.3942849	8.45859264	9.15617386	8.6899151	9.66303448	7.78358781	6.10545006	6.03573446	5.38509653	3.87649278	[illegible]
ENSG00000198618	0.14517901	0.121554	0.07099123	0.1407456	0.1417219	0.08167679	0.11658961	0.14220399	0.08971906	0.13149192	[illegible]
ENSG00000198624	0.03959427	0.02504749	0.03441999	0.0454935	0.01963247	0.04785105	0.10422404	0.08833885	0.07068775	0.05578445	[illegible]
ENSG00000198625	4.24921995	5.02465824	4.37911026	5.2533733	4.13041194	5.1531487	5.03921981	5.10859826	4.49751959	4.65556386	[illegible]
ENSG00000198626	0.00116738	0.00195483	0.00342504	0.0113174	0.00455834	0.00744332	0.11718716	0.14178936	0.08686031	0.24318516	[illegible]
ENSG00000198632	0.05169164	0.05193582	0.0505535	0.1670437	0.0576694	0.03877519	0.02075613	0.02531619	0	0.02340915	[illegible]
ENSG00000198633	0	0.00652797	0	0.0188966	0.00815472	0.01096598	0.00391335	0.0095462	0.00722744	0.00441355	[illegible]
ENSG00000198642	10.1623039	10.2512266	10.2126692	8.3470043	9.05436403	8.57452378	5.73411837	5.67043384	5.10410135	4.33197631	[illegible]
ENSG00000198643	0	0.01147382	0	0	0	0	0.02063478	0	0.00423442	0.00775743	[illegible]
ENSG00000198646	2.44894721	2.9788548	2.54757584	2.1910178	2.71958151	2.28294442	1.83446185	1.95398199	2.22439975	2.10190499	[illegible]
ENSG00000198648	25.2622422	19.413929	21.42773	20.211822	22.4553902	21.2440236	3.81838746	4.5431998	5.23209433	4.36606887	[illegible]
ENSG00000198650	0	0.02671046	0.01299977	0.0229094	0.01482961	0.02658931	0.0142331	0.0260401	0.01533389	0.04815706	[illegible]
ENSG00000198657	0	0	0	0	0	0	0	0	0	0	[illegible]
ENSG00000198658	0	0	0	0	0	0	0	0	0	0	[illegible]
ENSG00000198663	8.31376213	7.80187537	7.79029011	7.8846498	7.66900255	7.61827028	17.3180151	18.1608637	12.8470262	11.6434545	[illegible]
ENSG00000198668	60.9529157	34.566112	41.903544	17.325399	68.2525558	24.5062441	8.30585941	8.31386282	7.72255596	5.63816568	[illegible]
ENSG00000198670	0	0	0	0	0	0	0	0	0	0	[illegible]
ENSG00000198671	0.05371623	0.03597998	0	0.0694345	0.05992812	0.04029388	0.04313815	0	0.05311368	0.14595603	[illegible]
ENSG00000198673	0.12122362	0.10334228	0.1447289	0.1200655	0.0711339	0.14643732	0.04804367	0.0323835	0.02490677	0.02851818	[illegible]
ENSG00000198674	0	0	0	0	0	0	0	0	0	0	[illegible]
ENSG00000198677	6.0419168	7.14091066	5.83450345	6.0042822	5.66236885	6.49284868	7.5396178	7.69880674	7.22968536	7.40617228	[illegible]
ENSG00000198678	0	0	0	0	0	0	0	0	0	0	[illegible]
ENSG00000198680	8.43395059	7.04394816	7.71032271	7.126841	7.13731451	6.07109775	5.11137173	5.36203247	5.5487722	3.85499473	[illegible]
ENSG00000198681	0	0	0	0	0	0	0	0	0	0	[illegible]
ENSG00000198682	0.32346804	0.11142721	0.16570501	0.1871586	0.32478725	0.12016528	1.91239495	2.13942118	2.53282521	3.15852088	[illegible]
ENSG00000198683	0	0	0	0	0	0	0	0	0	0	[illegible]
ENSG00000198685	0.01195022	0.00800445	0.0233742	0.0463412	0.00666609	0.04482078	0	0.04682134	0.01772426	0.01082358	[illegible]
ENSG00000198689	2.47957199	2.84516064	2.66380036	2.4888369	2.58818211	2.47998483	1.97053933	2.24936344	2.21726223	1.6014118	[illegible]
ENSG00000198690	1.50800923	2.28134902	1.77628561	2.3585187	1.29572632	2.36863531	1.62320074	1.6533806	1.27031282	2.39964936	[illegible]
ENSG00000198691	0.00773081	0.00388366	0.00189015	0.0074947	0.0010781	0	0.01396894	0.00567929	0.01911022	0.0437623	[illegible]
ENSG00000198692	0.02376824	0.05094511	0.02324492	0.0122893	0.00530337	0.03209247	0.03817536	0.0372499	0.03525245	0.03013841	[illegible]
ENSG00000198695	3.13088901	6.25023727	6.96444261	2.9360876	6.30101341	7.87457586	19.3751939	12.5675828	21.427578	20.9064639	[illegible]
ENSG00000198700	31.9450667	37.5318959	30.8867348	24.519283	27.9005832	25.0678604	13.8920987	16.0727776	15.7124401	12.5135864	[illegible]
ENSG00000198703	0	0	0	0	0	0	0	0	0	0	[illegible]
ENSG00000198704	0	0	0	0	0	0	0	0	0	0	[illegible]
ENSG00000198707	1.04596573	1.28588048	1.16250879	0.7759907	1.09450477	0.90754476	1.41600215	1.63510219	1.5390587	1.97977156	[illegible]
ENSG00000198711	0.03138241	0.08408175	0	0.1216963	0.01750578	0.14124438	0.05040485	0	0	0	[illegible]
ENSG00000198712	689.169845	1156.75649	1033.34338	793.86783	1190.4405	1331.96023	1607.65306	1002.09409	1347.15407	2930.13134	[illegible]
ENSG00000198715	1.37642609	1.37661328	1.39836666	1.003334	0.94660247	0.86040954	7.64928311	7.82768101	5.53561016	4.46293013	[illegible]
ENSG00000198718	1.01776274	1.15249078	1.03578723	1.1120436	0.76793173	1.01947184	0.77495383	0.75478848	0.62798789	0.43660528	[illegible]
ENSG00000198719	13.7387643	25.5260645	18.9974682	15.028829	3.60248414	13.7182008	0.04892796	0.02983865	0.02008079	0.13795466	[illegible]
ENSG00000198720	1.69335179	2.17303565	1.58982493	1.8507146	1.4852827	1.97279128	1.90759489	2.14595196	2.34987351	2.64220716	[illegible]
ENSG00000198721	4.15570706	2.89987933	3.39165111	3.6589765	4.20907916	3.52894425	2.02360457	2.10076347	1.96954782	1.75848745	[illegible]
ENSG00000198722	0.32652407	0.47022865	0.35126814	0.57214	0.40577207	0.44632386	1.30820271	1.36639734	1.24480995	1.2749691	[illegible]
ENSG00000198723	0.0109793	0	0.01073756	0	0.00306224	0.01647169	0.0088172	0.01075431	0.00542807	0.0099442	[illegible]
ENSG00000198727	188.943672	336.950503	281.167086	203.00921	293.78433	359.424255	374.723491	230.862515	276.298227	416.096213	[illegible]
ENSG00000198728	13.6118121	17.1196534	13.8286467	17.980741	11.1732739	17.3290968	14.2876887	14.8399266	14.3106091	11.813755	[illegible]
ENSG00000198729	39.8378754	29.1317494	34.7424224	19.475217	30.4597463	21.4576158	0.01202737	0.07334871	0.05183027	0.02712937	[illegible]
ENSG00000198730	6.57994559	7.76203639	6.47955997	6.1531462	6.59801413	5.88198646	11.3953811	12.6147516	13.2780804	10.5220196	[illegible]
ENSG00000198732	45.7692139	57.7256873	45.4050869	24.045927	18.5755951	25.9008593	3.13934894	3.70872509	3.81759129	10.375475	[illegible]
ENSG00000198734	0	0.00263702	0.00385025	0.005089	0.00329416	0.00738299	0	0	0	0	[illegible]
ENSG00000198736	2.81659182	2.4343198	2.74649827	2.1353512	2.67234554	1.92072325	2.24866751	2.2491701	2.81358244	1.30170935	[illegible]
ENSG00000198738	0	0	0	0	0	0	0	0	0	0	[illegible]
ENSG00000198739	7.67132635	9.2294939	9.09365332	9.4395872	5.36410416	9.24431131	0	0.01245091	0	0.0057565	[illegible]
ENSG00000198740	2.56729499	3.54022301	2.92125909	3.2948248	2.61299925	3.11183504	1.2844905	1.51879045	1.32163019	1.61476201	[illegible]
ENSG00000198742	4.66479852	3.90405394	3.83608443	4.0789802	4.07401861	4.3280316	3.19724063	3.19412549	4.0007868	5.02164194	[illegible]
ENSG00000198743	0	0	0	0	0	0	0	0	0	0	[illegible]
ENSG00000198744	222.249686	322.754272	283.277594	163.90097	316.808291	303.463761	715.162778	525.308316	665.358141	951.782749	[illegible]
ENSG00000198746	3.61700528	4.0536697	3.42867618	3.7481572	3.11100192	3.28161462	3.57005416	3.35485525	3.35665168	2.27856124	[illegible]
ENSG00000198750	0.97948558	1.06221661	0.97312437	1.1455186	0.55505057	1.15458592	0.14982861	0.21320309	0.27671384	0.09857137	[illegible]
ENSG00000198752	8.57394539	9.4247038	8.29753224	7.6113762	8.71145076	7.54922161	5.86163531	5.9532518	5.58434135	5.66247722	[illegible]
ENSG00000198753	0.89026333	1.13512928	0.87425932	0.8971562	0.38100629	0.84809861	0.73858271	0.75791272	0.47226984	0.75413115	[illegible]
ENSG00000198754	0	0.0827579	0.0517855	0.0228153	0.04922901	0.03972015	0	0.01728877	0.02617871	0.01598642	[illegible]
ENSG00000198755	46.283996	53.0077022	49.8205017	72.97657	42.5257579	59.1236979	71.512999	79.7689499	82.475676	57.8238235	[illegible]
ENSG00000198756	11.4368392	12.9522249	9.24983345	11.725658	9.5286654	10.2722296	0.78857138	0.85147971	0.46759938	0.75846315	[illegible]
ENSG00000198758	0	0	0	0	0	0	0	0	0	0	[illegible]
ENSG00000198759	0	0.01258286	0	0	0.00349299	0	0	0	0	0	[illegible]
ENSG00000198763	55.6442436	91.0134765	87.7541548	67.688646	85.6477461	88.8862935	147.820628	103.554532	133.528415	237.731839	[illegible]
ENSG00000198765	0.01003338	0.02184169	0.04415611	0.0421503	0.00839525	0.03198674	0.00805756	0.00982778	0.00620052	0.01590306	[illegible]
ENSG00000198768	0.01411521	0.00630306	0.0184059	0.0060819	0	0	15.4806148	20.3886898	27.8951522	16.7220866	[illegible]
ENSG00000198771	0.00365334	0	0.0035729	0.0047224	0.00611373	0.00822138	0.02053732	0.02504931	0.01806178	0.02978019	[illegible]
ENSG00000198774	0.58246518	0.82757905	0.39702215	0.6844584	0.72858937	0.6752425	1.3465908	1.62514401	1.40492387	0.55952476	[illegible]
ENSG00000198780	1.12381223	1.07062517	1.12603918	0.928335	1.06788622	0.96798409	0.45863511	0.46822673	0.48629472	0.60884397	[illegible]
ENSG00000198783	6.17135723	5.33197357	5.8727221	5.4316664	4.85820017	4.77492908	6.25911604	6.95502361	8.17959889	7.10939016	[illegible]
ENSG00000198785	0	0	0.0038123	0	0	0	0	0	0	0	[illegible]
ENSG00000198786	36.4985795	57.4488358	52.4117446	44.488664	67.7663969	60.1206072	96.5037653	60.5774891	81.4380523	88.6690945	[illegible]
ENSG00000198787	0	0	0	0	0	0	0	0	0	0	[illegible]
ENSG00000198788	0.00191479	0	0	0	0	0	0	0	0	0	[illegible]
ENSG00000198791	9.46329962	8.41156036	9.22954629	8.1180312	9.85526424	8.91957375	7.96252352	8.99981522	9.76917002	9.50230904	[illegible]
ENSG00000198792	2.35942458	2.17955013	2.07070788	1.8458112	1.9810727	2.04534609	3.7665219	3.97705892	3.34018775	2.59597143	[illegible]
ENSG00000198793	4.80623554	5.41967286	4.56110235	5.1351299	4.85487395	4.15536845	5.09410126	5.14355271	5.89823602	5.5652357	[illegible]
ENSG00000198794	0.82755151	0.89779579	0.81198406	0.86278	0.78098013	0.75000862	0.13945407	0.11560921	0.13548383	0.35142	[illegible]
ENSG00000198795	5.98528437	7.50785241	6.75781062	7.2632619	5.25432658	6.66980051	1.43035861	1.48952909	1.64339396	2.20725073	[illegible]
ENSG00000198796	0.00225778	0.00302459	0.00220806	0	0.00377831	0	0.68537537	0.8381618	1.05594843	1.11448038	[illegible]
ENSG00000198797	45.5981844	63.994532	51.1827661	60.84732	36.0324214	50.1287253	0.00402847	0.03930806	0.0148801	0.02726027	[illegible]
ENSG00000198798	0	0	0	0	0	0	0	0	0	0	[illegible]
ENSG00000198799	2.04227444	2.25721702	1.9460947	2.3372677	1.64096391	2.2482623	1.19853402	1.35473667	1.18710076	0.9736912	[illegible]
ENSG00000198804	576.561201	997.177564	838.328543	621.88459	822.472595	1171.62694	1774.72381	983.684237	1196.30825	1959.72948	[illegible]
ENSG00000198805	1.64954041	1.52932712	1.53124829	0.8234197	1.63644342	1.09400846	1.95205421	2.57467189	3.02007075	1.81894648	[illegible]
ENSG00000198807	0	0	0.00284016	0.0037539	0	0	0	0	0	0	[illegible]
ENSG00000198812	0	0	0	0	0	0	0	0	0.00614742	0	[illegible]
ENSG00000198814	0.94488137	0.78898661	0.87956463	0.6754009	1.2430438	0.70604769	0.51610807	0.57064745	0.74886605	0.68760848	[illegible]
ENSG00000198815	2.00762584	2.09582588	1.90116717	2.2254486	1.95299013	2.17300463	2.13763908	2.46242356	2.50462253	2.06759957	[illegible]

ENSG00000198816	8.78332998	10.1132929	8.67512845	10.621602	8.15508291	8.53794695	7.17609028	7.45859952	6.75405101	8.29714016	5.17606302	4.97157442	3.61270014	[illegible]
ENSG00000198818	2.71385034	1.89487994	2.19515028	2.3920568	2.7782609	2.27815411	4.42284594	4.90017776	4.87215917	3.78924312	5.03642609	5.17814795	2.47314805	[illegible]
ENSG00000198821	0.00319549	0.00642118	0.01875081	0.0247833	0.00356503	0.02157317	0.0128311	0.00939003	0.01105877	0.00289423	0.00805151	0.01619155	0.0027047	[illegible]
ENSG00000198822	4.5227659	4.4512402	5.151631	1.2143448	3.35138292	1.49665388	0	0	0	0	0	0	0	[illegible]
ENSG00000198824	11.8828495	10.053645	9.38137056	6.1868932	12.19423	7.37490089	4.72875917	4.90283054	4.96566459	4.76198462	3.83874749	5.58375256	6.22007965	[illegible]
ENSG00000198825	8.7285004	5.57866231	6.54168014	5.549151	11.6951466	6.23822771	1.81712941	1.77004176	2.17756913	3.69171343	2.03057776	2.57050075	1.92567861	[illegible]
ENSG00000198826	6.80441792	5.31981159	5.95914186	4.2526742	6.93750233	4.66585548	2.03111008	2.7190305	4.75930695	3.00308278	2.26235977	5.35561611	5.62154409	[illegible]
ENSG00000198829	0	0	0	0	0.01290627	0.00578519	0.02477425	0.00755427	0	0.01397043	0.00971614	0.00651304	4.64777593	[illegible]
ENSG00000198830	66.5045919	49.7728838	54.2052597	52.905713	49.5757919	48.4305167	10.4655303	13.3446508	18.199546	6.17318806	6.76567653	15.566587	20.228662	[illegible]
ENSG00000198832	0.03073681	0.00882343	0.04294292	0.0397309	0.02939254	0.03293778	3.6920148	3.84508752	2.95453917	2.27484605	3.47953075	2.26569377	2.28568625	[illegible]
ENSG00000198833	35.5203286	27.5108495	28.132981	26.336556	47.0414762	25.68101	18.2809242	19.1844128	21.2937591	14.9698494	19.7245233	20.0359941	20.3738345	[illegible]
ENSG00000198835	0.12932129	0.09624606	0.08431593	0.2228838	0.15229169	0.15089996	0.43849751	0.76002741	0.46175517	0.24727281	0.57927768	0.58246257	0.19459347	[illegible]
ENSG00000198836	6.6708609	6.11419364	5.96646899	5.4423149	6.9246061	5.94289359	2.13902608	2.42791803	2.64104207	1.9008118	2.42007995	2.49636996	3.05259382	[illegible]
ENSG00000198837	3.34997461	3.59423089	2.96700701	3.9077716	3.29546844	4.53814152	2.74591173	2.80287248	2.53698709	3.14837094	2.15270338	2.17653924	1.58009538	[illegible]
ENSG00000198838	0.00193305	0.00258957	0.02268586	0.003748	0.00431319	0.00145003	0	0.00189344	0.00047784	0.00175081	0.0024353	0.00326492	0.03926766	[illegible]
ENSG00000198839	3.30250515	4.18525611	3.89597865	6.5277807	2.57232671	5.45485621	1.53515884	1.74666852	1.68033667	2.7585803	1.77026398	1.6836181	1.77798696	[illegible]
ENSG00000198840	424.38929	630.85599	872.08665	714.37268	583.415804	604.058866	635.028542	493.247297	658.456185	1179.88156	494.755462	710.89301	478.811687	[illegible]
ENSG00000198841	2.1812426	2.69535005	2.31711402	2.4549189	3.18870726	2.55304295	4.48495864	5.24926881	5.74520255	6.1822683	7.81752387	8.43217797	3.03990568	[illegible]
ENSG00000198842	0	0.00342721	0.00250199	0	0	0.00383812	0.00616357	0	0.00252962	0	0.00644606	0.0021605	0.06712706	[illegible]
ENSG00000198843	18.2221022	19.167144	19.4588832	18.828163	19.9547048	16.9561779	6.16901453	7.18306123	7.34861519	5.68008103	8.08055779	8.90132515	14.5528951	[illegible]
ENSG00000198844	[illegible]	[illegible]	[illegible]	[illegible]	[illegible]	[illegible]	[illegible]	[illegible]	[illegible]	[illegible]	[illegible]	[illegible]	[illegible]	[illegible]
[illegible]	[illegible]	[illegible]	[illegible]	[illegible]	[illegible]	[illegible]	[illegible]	[illegible]	[illegible]	[illegible]	[illegible]	[illegible]	[illegible]	[illegible]

ENSG00000199222	0	0	0	0	0.35251833	0	0	0.30950281	0.15621672	0	0.39807593	0	0	0	0	0	0	0.38114606	0	0	0	0	0	0	0.30163769	0.78011978	0.54374761	0.23825481	0.68071083	0.25417593	0.48008414	0	0	0	0	0.28107461	0.29240058	0.63680571	0.31827293	0.50755328	0	0	0.72355502	0	0	0.86799339	0.84411698	0.29541272
ENSG00000199223	0.63195568	0.84658787	0.61804131	1.633753	0.35251833	0.23702284	0	0	0	0	1.1942278	0	0	0	0.27480484	0	0	0.38114606	0	0.28056427	0	0	0	0	0.30163769	1.56023956	0.2718738	0.47650961	0.45380722	0	2.40042069	1.93295647	1.03674204	0.49730417	0	0.56214923	0.87720174	1.27361141	1.27309173	0	0	0	1.44711003	0.26976807	0.58293403	0.86799339	0.28137233	0
ENSG00000199224	0	0	0	0	0	0	0	0	0	0	0	0	0	0	0	0	0	0	0	0	0	0	0	0	0	0	0	0	0	0	0	0	0	0	0	0	0	0	0	0	0	0	0	0	0	0	0	0
ENSG00000199226	0	0	0.30602045	0	0	0	0	0	0	0	0	0.26425234	0	0	0	0	0	0.37744561	0	0	0	0	0	0	0	0	0	0.23594165	0	0	0	0	0	0	0	0	0	0	0	0	0	0	0	0	0	0	0.27864056	0
ENSG00000199231	0	0	0	0	0	0	0	0	0	0	0	0	0	0	0	0	0	0	0	0	0	0	0	0	0	0	0	0	0	0	0	0	0	0	0	0	0	0	0	0	0	0	0	0	0	0	0	0
ENSG00000199235	0	0	0	0	0	0	0	0	0	0	0	0	0	0	0	0	0	0	0	0	0	0	0	0	0	0	0	0	0	0	0	0	0	0	0	0	0	0	0	0	0	0	0	0	0	0	0	0
ENSG00000199237	0	0	0	0.3787337	0.16344032	0.21978481	0.23529902	0.86098055	0.28971101	0	1.10737487	0	0	0.31741583	0	0	0	0.35342634	0.3224489	0.5203192	0.49328598	0.75072894	0	0	0.55940082	0.24112793	0.75630349	0.88370874	0	0.23569041	0.44516893	0.35847556	0	0	0	0	0.27113508	0	1.18050324	0.47064032	0.54735055	0.52466547	0	0	0.27026942	0	0	0.27392816
ENSG00000199240	0	0	0	0	0	0	0	0	0	0	0	0	0	0	0	0	0	0	0	0	0	0	0	0	0	0	0	0	0	0	0	0	0	0	0	0	0	0	0	0	0	0	0	0	0	0	0	0
ENSG00000199241	0	0	0	0	0	0	0	0	0	0	0	0	0	0	0	0	0	0	0	0	0	0	0	0	0	0	0	0	0	0	0	0	0	0	0	0	0	0	0	0	0	0	0	0	0	0	0	0
ENSG00000199245	0	0	0	0	0	0	0	0	0	0	0	0	0	0	0	0	0	0	0	0	0	0	0	0	0	0	0	0	0	0	0	0	0	0	0	0	0	0	0	0	0	0	0	0	0	0	0	0
ENSG00000199246	0	0	0	0	0	0	0	0	0	0	0	0	0	0	0	0	0	0	0	0	0	0	0	0	0	0	0	0	0	0	0	0	0	0	0	0	0	0	0	0	0	0	0	0	0	0	0	0
ENSG00000199248	0	0	0	0	0	0.225947	0.24189619	0	0.59566749	0	0	0.25437374	0.50989855	0.32631534	0.52392699	0	0.90626597	0	0.33148952	0	0	0.51451828	0	0	0	0	0	0.22712141	0.21630064	0.48459711	0	0	0	0	0	0	0	0	0.30340037	0	0	0	0	0	0.27784706	0.41371648	0.26822409	0.28160839
ENSG00000199251	0	0	0	0	0	0	0	0	0	0	0	0	0	0	0	0	0	0	0	0	0	0	0	0	0	0	0	0	0	0	0	0	0	0	0	0	0	0	0	0	0	0	0	0	0	0	0	0
ENSG00000199260	0	0	0	0	0	0	0	0	0	0	0	0	0	0	0	0	0	0	0	0	0	0	0	0	0	0	0	0	0	0	0	0	0	0	0	0	0	0	0	0	0	0	0	0	0	0	0	0
ENSG00000199262	0	0	0	0	0	0	0	0	0	0	0	0	0	0	0	0	0	0	0	0	0	0	0	0	0	0	0	0	0	0	0	0	0	0	0	0	0	0	0	0	0	0	0	0	0	0	0	0
ENSG00000199263	0	0	0	0	0	0	0	0	0	0	0	0	0	0	0	0	0	0	0	0	0	0	0	0	0	0	0	0	0	0	0	0	0	0	0	0	0	0	0	0	0	0	0	0	0	0	0	0
ENSG00000199266	0	0.64441763	0	1.2436031	0.5366697	0.72168147	2.70418281	3.06269201	1.30802358	3.26767234	3.33314326	2.03119333	1.22147339	1.56339141	1.88261824	0.23202449	2.17098042	0.87037831	1.85287798	2.34920236	1.82221312	0.61627003	1.70552303	1.11807556	1.37762888	0.79176336	1.44864101	1.26950696	0.34543534	2.12824923	1.46174872	1.17708394	1.57832371	0.75708993	1.40529068	0.85580927	2.00316218	2.42366351	2.18040711	3.09077224	2.24658806	3.4455643	0.82614864	1.23207505	1.33117772	2.97320125	1.07089468	2.47353038
ENSG00000199270	0	0	0	0	0	0	0	0	0	0	0	0	0	0	0	0	0	0	0	0	0	0	0	0	0	0	0	0	0	0	0	0	0	0	0	0	0	0	0	0	0	0	0	0	0	0	0	0
ENSG00000199272	0	0	0	0	0	0	0	0	0	0	0	0	0	0	0	0	0	0	0	0	0	0	0	0	0	0	0	0	0	0	0	0	0	0	0	0	0	0	0	0	0	0	0	0	0	0	0	0
ENSG00000199273	0	0	0	0	0	0	0	0	0	0	0	0	0	0	0	0	0	0	0	0	0	0	0	0	0	0	0	0	0	0	0	0	0	0	0	0	0	0	0	0	0	0	0	0	0	0	0	0
ENSG00000199276	0	0	0	0	0	0	0.2175033	0	0	0	0	0	0	0	0	0	0	0	0	0	0	0	0	0	0	0	0	0	0	0	0	0	0	0	0	0	0	0	0	0	0	0	0	0	0	0.37199717	0	0
ENSG00000199279	0	0.20175692	0	0	0.33604551	0.225947	0	0	0.29783375	0.27281501	0	0.25437374	0	0	0	0	0	0	0	0	0	0	0.30512695	0	0	0	0	0.22712141	0.43260127	0	0	0.36852628	0	0	0	0	0	0	0	0	0	0	0	0	0	0	0.26822409	0
ENSG00000199282	0	0	0	0	0	0	0	0	0	0	0	0	0	0	0	0	0	0	0	0	0	0	0	0	0	0	0	0	0	0	0	0	0	0	0	0	0	0	0	0	0	0	0	0	0	0	0	0
ENSG00000199283	0	0	0	0	0	0	0	0	0	0	0	0	0	0	0	0	0	0	0	0	0	0	0	0	0	0	0	0	0	0	0	0	0	0	0	0	0	0	0	0	0	0	0	0	0	0	0	0
ENSG00000199285	0.31291009	0.20959214	0.30602045	0	0.52364374	0.23472164	0	0	0	0	0	0	0	0	0	0	0	0	0	0	0	0	0	0	0	0.25751527	0	0	0	0	0	0	0	0.49247598	0	0	0.28956174	0	0	0	0	0	0	0	0	0	0	0
ENSG00000199286	0	0	0	0	0	0	0	0	0	0	0	0	0	0	0	0	0	0	0	0	0	0	0	0	0	0	0	0	0	0	0	0	0	0	0	0	0	0	0	0	0	0	0	0	0	0	0	0
ENSG00000199287	0	0	0	0	0	0	0	0	0	0	0	0	0	0	0	0	0	0	0	0	0	0	0	0	0	0	0	0	0	0	0	0	0	0	0	0	0	0	0	0	0	0	0	0	0	0	0	0
ENSG00000199289	0	0	0	0	0	0	0	0	0	0	0	0	0	0	0	0	0	0	0	0	0	0	0	0	0	0	0	0	0	0	0	0	0	0	0	0	0	0	0	0	0	0	0	0	0	0	0	0
ENSG00000199290	0	0	0	0	0	0	0	0	0	0	0	0	0	0	0	0	0	0	0	0	0	0	0	0	0	0	0	0	0	0	0	0	0	0	0	0	0	0	0	0	0	0	0	0	0	0	0.28137233	0
ENSG00000199291	0.31597784	0	0	0	0	0	0	0.30950281	0.15621672	0	0.39807593	0.26684305	0	0	0	0	0	0.38114606	0	0	0	0	0	0	0.30163769	0.26003993	0.54374761	0	0	0.25417593	0.96016828	0	1.03674204	0.49730417	0	0	0	0	0	0	0	0	0	0	0	0	0	0
ENSG00000199293	0	0	0	0	0	0	0	0	0	0	0	0	0	0	0	0	0	0	0	0	0	0	0	0	0	0	0	0	0	0	0	0	0	0	0	0	0	0	0	0	0	0	0	0	0	0	0	0
ENSG00000199295	0	0	0	0	0	0	0	0	0	0	0	0	0	0	0	0	0	0	0	0	0	0	0	0	0	0	0	0	0	0	0	0	0	0	0	0	0	0	0	0	0	0	0	0	0	0	0	0
ENSG00000199299	0	0	0.57309285	0	0	0	0	0	0	0	0	0	0	0	0	0	0	0	0	0	0	0	0	0	0	0	0	0	0	0	0	0	0	0	0	0	0	0	0	0	0	0	0	0	0	0	0	0
ENSG00000199301	0	0	0	0	0	0	0	0	0	0	0	0	0	0	0	0	0	0	0	0	0	0	0	0	0	0	0	0	0	0	0	0	0	0	0	0	0	0	0	0	0	0	0	0	0	0	0	0
ENSG00000199303	0	0	0	0	0	0	0	0	0	0	0	0	0	0	0	0	0	0	0	0	0	0	0	0	0	0	0	0	0	0	0	0	0	0	0	0	0	0	0	0	0	0.51993876	0	0	0	0	0	0
ENSG00000199306	0	0.20175692	0.29458044	0.3893524	0	0.225947	0	0	0.29783375	0	0	0	0	0	0	0	0	0	0	0.26745379	0	0	0	0	0	0	0	0	0	0	0.4576503	0	0	0	0	0	0	0	0	0	0	0	0	0.51432416	0	0	0	0.28160839
ENSG00000199308	0	0	0	0	0	0	0	0	0	0	0	0	0	0	0	0	0	0	0	0	0	0	0	0	0	0	0	0.22712141	0	0	0	0	0	0	0	0	0	0	0	0	0	0	0	0	0	0	0	0
ENSG00000199311	0	0	0	0	0.21924921	0	0	0	0	0	0	0	0	0	0	0	0	0	0	0	0	0	0	0	0	0	0	0	0	0	0	0	0	0	0	0	0	0	0	0	0	0	0	0	0	0	0	0
ENSG00000199313	0	0.15419993	0	0	0	0	0.36975561	0	0	0	0	0.19441422	0	0	0	0	0	0	0	0.20441111	0	0	0	0	0	0	0	0	0	0	0	0	0	0	0	0	0	0	0	0	0.43006114	0	0	0.19654531	0	0	0.20499984	0
ENSG00000199315	0	0	0	0	0	0	0	0	0	0	0	0	0	0	0	0	0	0	0	0	0	0	0	0	0	0	0	0	0	0	0	0	0	0	0	0	0	0	0	0	0	0	0	0	0	0	0	0
ENSG00000199318	0	0	0	0	0	0	0	0	0	0	0	0	0	0	0	0	0	0	0	0	0	0	0	0	0	0	0	0	0	0	0	0	0	0	0	0	0	0	0	0	0	0	0	0	0	0	0	0
ENSG00000199319	0.31392604	0.42054527	0.10233801	0.270524	0.17511463	0.15698915	0.16807073	0.51248842	0.15520233	0.28432993	0	0.0883701	0.17714008	0	0.0910068	0.20189144	0	0.25244739	0.11516032	0.09291414	0.08808678	0.08937249	0.1060019	0.09728709	0	0.25835136	0	0.2367077	0.30057361	0.16835029	0.1589889	0	0	0	0.40759513	0.74466521	0.29050188	0.1054451	0.10540208	0.16808583	0	0.37476105	0.11980943	0	0.38609917	0.57490471	0.46590872	0.48915742
ENSG00000199321	0	0	0	0	0	0	0	0	0	0	0	0	0	0	0	0	0	0	0	0	0	0	0	0	0	0	0	0	0	0	0	0	0	0	0	0	0	0	0	0	0	0	0	0	0	0	0	0
ENSG00000199322	0	0	0	0	0	0.20316243	0	0	0	0	0	0	0	0	0	0	0	0.32669662	0	0	0	0	0	0	0	0	0	0	0	0	0	0	0	0	0	0	0	0	0	0	0	0	0	0	0	0	0	0
ENSG00000199325	0	0.16998418	0	0	0.14156248	0.3807296	0	0	0	0.22985202	0	0.21431489	0.21479978	0	0.6621282	0	0.38177346	0	0	0.22533509	0	0.65023767	1.54245277	0	0.2422602	0.20885096	0.43671067	0.19135425	0	0	0	0	0	0	0	0	0	0	0	0.40764122	0	0.45443466	0	0.21666412	0	0	0.45196815	0.23726061
ENSG00000199326	0	0	0	0	0	0	0	0	0	0	0	0	0	0	0	0	0	0	0	0	0	0	0	0	0	0	0	0	0	0	0	0	0	0	0	0	0	0	0	0	0	0	0	0	0	0	0	0
ENSG00000199327	0	0	0	0	0.17286957	0	0	0	0	0.28068468	0	0	0	0	1.07808053	0	0	0.37381632	0.34105172	0	0	0	0.31392869	0	0.29583697	0.25503916	1.86651822	0	0	0	0	0	0	0	0	0.27566933	0	0	0	0	0	0	0.7096405	0.79374066	0.28586188	0	0	0.57946341
ENSG00000199331	0.31597784	0	0	0	0	0.23702284	0.25375385	0	0.31243344	0	0	0	0.53489358	1.02693357	0.27480484	0.30481649	0.95069077	1.14343817	0.34773901	0.56112855	0.53197507	0.26986988	0.96025246	0.29376887	0	0.78011978	0.54374761	0.71476442	0.68071083	0.25417593	0.48008414	0	0.51837102	0	0.61538873	0.28107461	0.58480116	0.31840285	0.9548188	0	1.18056	0	1.08533252	1.34884033	1.16586807	1.30199009	1.12548931	0.59082544
ENSG00000199332	0	0	0	0	0	0	0	0	0	0	0	0	0	0	0	0	0	0	0	0	0	0	0	0	0	0	0	0	0	0	0	0	0	0	0	0	0	0	0	0	0	0	0	0	0	0	0	0
ENSG00000199334	0	0	0	0	0	0	0	0	0	0	0	0	0	0	0	0	0	0	0	0	0	0	0	0	0	0	0	0	0	0	0	0	0	0	0	0	0	0	0	0	0	0	0	0	0	0	0	0
ENSG00000199335	0	0	0	0	0	0	0.48379239	0	0.14891687	0	0	0.25437374	0	0	0	0	0	0	0	0	0	0	0	0	0	0	0	0	0	0	0	0	0	0	0	0	0	0.60704843	0	0.48383584	0	0	0	0	0	0	0	0
ENSG00000199337	0	0	0	0	0	0	0	0	0	0	0	0	0	0	0	0	0	0	0	0	0	0	0	0	0	0	0	0	0	0	0	0	0	0	0	0	0	0	0	0	0	0	0	0	0	0	0	0
ENSG00000199347	19.8750062	13.8522939	15.2347182	38.188977	26.6680119	10.8793482	3.23536158	7.89232171	5.70972112	5.5949812	55.1534206	7.71176402	4.09193587	32.8789899	15.4165516	6.21825638	10.1010894	43.4125358	152.222749	5.48503156	11.7566491	36.9316933	22.3098655	35.457903	14.3579543	17.682715	27.2689424	40.9083504	17.9367303	14.6913689	26.5246486	5.25764159	5.28738441	18.599176	7.3231259	12.4234979	7.4562148	22.7339637	13.2560677	17.688232	33.616446	22.1233941	14.7605223	5.04466283	17.8377814	16.2314764	10.5233251	4.26871379
ENSG00000199348	0	0	0	0	0	0	0	0	0	0	0	0	0	0	0	0	0	0	0	0	0	0	0	0	0	0	0	0	0	0	0	0	0	0	0	0	0	0	0	0	0	0	0	0	0	0	0	0
ENSG00000199349	0	0	0	0	0	0	0	0	0	0	0	0	0	0	0	0	0	0	0	0	0	0	0	0	0	0	0	0	0	0	0	0	0	0	0	0	0	0	0	0	0	0	0	0	0	0	0	0
ENSG00000199350	0	0	0	0	0	0	0	0	0	0	0	0	0	0	0	0	0	0	0	0	0	0	0	0	0	0	0	0	0	0	0	0	0	0	0	0	0	0	0	0	0	0	0	0	0	0	0	0
ENSG00000199352	0	0	0	0	0	0	0	0	0	0	0	0	0	0	0	0	0	0	0	0	0	0	0	0	0	0	0	0	0	0	0	0	0	0	0	0	0	0	0	0	0	0	0	0	0	0	0	0
ENSG00000199354	0	0	0	0	0	0	0	0	0	0	0	0	0	0	0	0	0	0	0	0	0	0	0	0	0	0	0	0	0	0	0	0	0	0	0	0	0	0	0	0	0	0	0	0	0	0	0	0
ENSG00000199357	0	0.2225566	0	0	0	0	0.26683394	0	0	0	0	0.28059784	0	0	0	0	0	0	0	0	0	0	0	0	0.31718603	0	0	0	0	0	0	0	0	0	0	0.295563	0	0	0.33467875	0	0	0	0	0.28367364	0.61298218	0	0.29587606	0.62128036
ENSG00000199360	0	0	0	0	0	0	0	0	0	0	0	0.2567735	0	0	0	0	0	0	0	0	0	0	0	0	0	0	0	0	0	0	0	0	0	0	0	0	0	0	0	0	0	0	0	0	0	0	0	0
ENSG00000199361	0	0	0	0	0	0	0	0	0	0	0	0	0	0	0	0	0	0	0	0	0	0	0	0	0	0	0	0	0	0	0	0	0	0	0	0	0	0	0	0	0	0	0	0	0	0	0	0
ENSG00000199362	0	0	0	0	0	0	0	0	0	0	0	0	0	0	0	0	0	0	0	0	0	0	0	0	0	0	0	0	0	0	0	0	0	0	0	0	0	0	0	0	0	0	0	0	0	0	0	0
ENSG00000199363	0	0	0	0	0	0	0	0	0	0	0	0	0	0	0	0	0	0	0	0	0	0	0	0	0	0	0	0	0	0	0	0	0	0	0	0	0	0	0	0	0	0	0	0	0	0	0	0
ENSG00000199364	0.31910633	0	0.31208026	0	0	0.4787392	0	0	0	0	0	0	0	0	0	0	0	0	0	0	0	0	0	0	0	0	0	0	0	0	0	0	0	0	0	0	0	0	0	0	0	0	0	0	0	0	0	0
ENSG00000199366	0.30405415	0.20366029	0.2973595	0.786051	0	0.22807858	2.19760409	0.59564692	1.05225225	1.92772117	0.3830542	1.79741448	1.8014812	3.29393788	1.58660908	2.05319786	2.28703912	2.5673423	0.33461678	0.26997694	1.02380108	0.25968611	3.08005507	0.84804977	0.58051028	0	2.87775855	0.68779218	0.21834121	1.71209072	0	0.74400589	0	0	0	1.40683298	2.45110121	1.53131317	0.48840033	0	0	1.63339251	2.08875316	0.51917628	2.80468262	0.83523892	0.81226351	4.5482411
ENSG00000199368	0	0	0	0	0	0	0	0	0	0	0	0	0	0	0	0	0	0	0	0	0	0	0	0	0	0	0	0	0	0	0	0	0	0	0	0	0	0	0	0	0	0	0	0	0	0	0	0
ENSG00000199370	0	0	0	0	0	0	0	0	0	0	0	0	0	0	0	0	0	0	0	0	0	0	0	0	0	0	0	0	0	0	0	0	0	0	0	0	0	0	0	0	0	0	0	0	0	0	0	0
ENSG00000199373	0	0	0	0	0	0	0	0	0	0	0	0	0	0	0	0	0	0	0	0	0	0	0	0.25180189	0	0	0	0	0	0	0	0	0	0	0	0	0	0	0	0	0	0	0	0	0	0	0	0
ENSG00000199377	1.10187145	0.9225637	0.80820786	0.3560744	0.61464735	1.23981176	14.8218274	14.840263	25.331142	3.74246234	46.5034347	16.0516355	4.43001604	9.25117937	16.7701416	5.5804865	16.9905504	13.2912471	8.48839828	4.15810642	9.73923596	24.9387449	15.0685771	17.4152214	7.10008728	7.02774572	43.3743282	7.47753549	21.3638475	22.602106	1.25560467	0	0.9038264	0.43354723	0	0.73511822	2.54913326	7.49471332	13.5959668	13.2744705	23.1571385	4.43947708	1.57697888	3.76291866	2.54099451	9.45890235	2.69828847	0.51507859
ENSG00000199378	0	0	0	0	0	0	0	0	0	0	0	0	0	0	0	0	0	0	0	0	0	0	0	0	0	0	0	0	0	0	0	0	0	0	0	0	0	0	0	0	0	0	0	0	0	0	0	0
ENSG00000199381	0	0	0	0	0	0	0	0	0	0	0	0	0	0	0	0	0	0	0	0	0	0	0	0	0	0	0	0	0	0	0	0	0	0	0	0	0	0	0	0	0	0	0	0	0	0	0	0
ENSG00000199385	0	0	0	0	0	0	0	0	0	0	0	0	0	0	0	0	0	0	0	0	0	0	0	0	0	0	0	0	0	0	0	0	0	0	0	0	0	0	0	0	0	0	0	0	0	0	0	0
ENSG00000199390	0.8371361	0.56072703	0	0	0.23348617	0.3139783	0	0	0	0	0	0.70856032	0	0	0	0	0.40378288	3.14839151	0.50489478	1.84256512	2.22993943	1.40938851	2.14493984	2.54404548	0	0	3.60144518	1.26244106	0.90172084	2.69360469	0.63595561	0	0	0	0.81519027	0	0	0	0	1.91695095	0	0.77219833	0	0	0	0	0.74545396	0.39132594
ENSG00000199392	0	0	0	0	0	0	0	0	0	0	0	0	0	0	0	0	0	0	0	0	0	0	0	0	0	0	0	0	0	0	0	0	0	0	0	0	0	0	0	0	0	0	0	0	0	0	0	0
ENSG00000199394	0	0	0	0	0	0	0	0	0	0	0	0	0	0	0	0	0	0	0	0	0	0.27804776	0	0	0	0	0	0	0	0	0	0	0	0	0	0	0	0	0	0	0	0	0	0	0	0	0	0
ENSG00000199395	0	0	0	0	0	0	0	0	0	0	0	0	0	0	0	0	0	0	0	0	0	0	0	0	0	0	0	0	0	0	0	0	0	0	0	0	0	0	0	0	0	0	0	0	0	0	0	0
ENSG00000199396	0	0	0	0	0	0	0	0	0	0	0	0	0	0	0	0	0	0	0	0	0	0	0	0	0	0	0	0	0	0	0	0	0	0	0	0	0	0	0	0	0	0	0	0	0	0	0	0
ENSG00000199398	0	0	0	0	0	0	0	0	0	0	0	0	0	0	0	0	0	0	0	0	0	0	0	0	0	0	0	0	0	0	0	0	0	0	0	0	0	0	0	0	0	0	0	0	0	0	0	0
ENSG00000199400	0	0.4497498	0.98500333	0	0.18727536	0.50367353	0	0	0	0	0	0	0	0	0	0	0	0	0	0	0	0	0	0	0	0	0	0	0	0	0	0	0	0	0	0	0	0	0	0	0	0	0	0	0	0	0	0
ENSG00000199402	0	0	0	0	0	0	0	0	0	0	0	0	0	0	0	0	0	0	0	0	0	0	0	0	0	0	0	0	0	0	0	0	0	0	0	0	0	0	0	0	0	0	0	0	0	0	0	0
ENSG00000199404	0	0	0	0	0	0	0	0	0	0	0	0	0	0	0	0	0	0	0	0	0	0	0	0	0	0	0	0	0	0	0	0	0	0	0	0	0	0	0	0	0	0	0	0	0	0	0	0
ENSG00000199405	0	0	0	0	0	0	0	0	0	0	0	0	0	0	0	0	0	0	0	0	0	0	0	0	0	0	0	0	0	0	0	0	0	0	0	0	0	0	0	0	0	0	0	0	0	0	0	0
ENSG00000199407	0	0	0	0	0	0	0	0	0	0	0	0.22494207	0	0	0	0	0	0	0	0	0	0	0	0	0	0	0	0	0	0	0	0	0	0	0	0	0	0	0	0	0	0	0	0	0	0	0	0
ENSG00000199409	0	0	0	0	0	0	0	0	0	0	0	0	0	0	0	0	0	0	0	0	0	0	0	0	0	0	0	0	0	0	0	0	0	0	0	0	0	0	0	0	0	0	0	0	0	0	0	0
ENSG00000199410	0	0	0	0	0	0	0	0	0	0	0	0	0	0	0	0	0	0	0	0	0	0	0	0	0	0	0	0	0	0	0	0	0	0	0	0	0	0	0	0	0	0	0	0	0	0	0	0
ENSG00000199411	0	0.25102315	0.36651287	0.4844268	0.41810314	0	0.9028916	0	0.1852803	0	0	0	0	0.81199399	0.32593132	0.72305307	0.56378174	1.35617085	0.82486927	0.66552456	0.31547359	0	0.37963469	0.34842355	0	0.30841945	0.32245498	0.56516257	0.26911823	0.30146448	0	0	0.61481214	0	0	0.33336756	0	0	0	0	0	1.34216749	1.28725485	0.31995747	0	0	0	0.35037322
ENSG00000199415	0	0	0	0	0	0	0	0	0	0	0	0	0	0	0	0	0	0	0	0	0	0	0	0	0	0	0	0	0	0	0	0	0	0	0	0	0	0	0	0	0	0	0	0	0	0	0	0
ENSG00000199420	0	0.13163409	0	0.1270143	0.1096246	0.22112496	0.15782252	0.09624783	0.19431836	0	0.12379191	0.08298168	0.08316943	0.10645043	0	0	0.44346246	0.35558138	0.10813835	0.08724865	0	0.08392295	0.09953836	0	0.2814059	0.16173215	0.16909224	0.07409143	0.14112298	0.15808503	0.44788337	0.36066139	0	0.30929894	0.19137089	0.1748147	0.54557669	0.19803104	0.09897512	0.15783669	0	0	0.45001592	0.08389129	0.09063913	0	0.52499959	0.1837323
ENSG00000199422	0	0	0	0	0	0	0	0	0	0	0	0	0	0	0	0	0	0	0	0	0	0	0	0	0	0	0	0	0	0	0	0	0	0	0	0	0	0	0	0	0	0	0	0	0	0	0	0
ENSG00000199424	0	0	0	0	0	0	0	0	0	0	0	0	0	0	0	0	0	0	0	0	0	0	0	0	0	0	0	0	0	0	0	0	0	0	0	0	0	0	0	0	0	0	0	0	0	0	0	0
ENSG00000199426	0	0	0	0	0	0.30032707	0	0	0	0	0	0	0	0	0	0	0	0	0	0	0	0	0	0	0	0	0	0	0	0	0	0	0	0	0	0	0	0	0	0	0	0	0	0	0	0	0	0
ENSG00000199432	0	0	0	0	0	0	0	0	0	0	0	0	0	0	0	0	0	0	0	0	0	0	0	0	0	0	0	0	0	0	0	0	0	0	0	0	0	0	0	0	0	0	0	0	0	0	0	0
ENSG00000199436	0.61980269	1.66061466	1.21231179	0.4005837	0.69147827	1.16232353	0.24887397	0.60710167	0.91927532	0.56136935	0.78084125	1.04684579	0.26230358	2.0143697	0.53904027	2.09268244	0.46620413	0.74763265	0.34105172	2.20135046	1.56523435	0.79404023	0.94178607	0.86435841	0.59167394	0.76511748	2.93310006	0.70101895	0.89016031	0.7478638	3.29596226	1.13747054	0.50840235	1.46322189	0	0.27566933	4.01488489	4.68419583	1.8729138	2.48896322	1.73678539	0	1.77410124	0	1.71517129	1.70260242	0.27596132	1.44865852
ENSG00000199440	0	0	0	0	0	0	0	0	0	0	0	0	0	0	0	0	0	0	0	0	0	0	0	0	0	0	0	0	0	0	0	0	0	0	0	0	0	0	0	0	0	0	0	0	0	0	0	0
ENSG00000199442	0	0	0	0	0	0	0	0.32545657	0	0	0	0	0	0	0	0	0	0	0	0	0	0	0	0	0	0	0	0	0	0	0	0	0	0	0	0	0	0	0	0	0	0	0	0	0	0	0	0
ENSG00000199444	0	0	0	0	0	0	0	0	0	0	0	0	0	0	0	0	0	0	0	0	0	0	0	0	0	0	0	0	0	0	0	0	0	0	0	0	0	0	0	0	0	0	0	0	0	0	0	0.29541272
ENSG00000199446	0	0	0	0	0	0	0	0	0	0	0	0	0	0	0	0	0	0	0	0	0	0	0	0	0	0	0	0	0	0	0	0	0	0	0	0	0	0	0	0	0	0	0	0	0	0	0	0
ENSG00000199448	0	0	0	0	0	0	0	0	0	0	0	0	0	0	0	0	0	0	0	0	0	0	0	0	0	0	0	0	0	0	0	0	0	0	0	0	0	0	0	0	0	0	0	0	0	0	0	0
ENSG00000199450	0	0	0	0	0	0	0	0	0	0	0	0	0	0	0	0	0	0	0	0	0	0	0	0	0	0	0	0	0	0	0	0	0	0	0	0	0	0	0	0	0	0	0	0	0	0	0	0
ENSG00000199453	0	0	0	0	0	0	0	0	0	0	0	0	0.33267771	0	0	0	0	0	0	0	0	0	0	0	0	0	0	0	0	0	0	0	0	0	0	0	0	0	0	0	0	0	0	0	0	0	0	0
ENSG00000199454	0	0	0	0	0	0	0	0	0	0	0	0	0	0	0	0	0	0	0	0	0	0	0	0	0	0	0	0	0	0	0	0	0	0	0	0	0	0	0	0	0	0	0	0	0	0	0	0
ENSG00000199455	0	0	0	0	0	0	0	0	0	0	0	0	0	0	0	0	0	0	0.34773901	0	0	0	0	0.29376887	0	0	0	0	0	0	0	0	0	0	0	0	0	0	0	0	0	0	0	0	0	0	0	0
ENSG00000199458	0	0	0	0	0	0	0	0	0	0	0	0	0	0	0	0	0	0	0	0	0	0	0	0	0	0	0	0	0	0	0	0	0	0	0	0	0	0	0	0	0	0	0	0	0	0	0	0
ENSG00000199459	0	0	0.57309285	0	0	0.21978481	0	0	0.1448555	0	0.36912496	0	0	0.31741583	0.25481903	0	0	0	0.3224489	0	0	0	0	0.27240386	0	0	0	0	0	0	0	0	0	0	0	0.26063282	0	0.29524628	0	0	0	0	0	0	0	0	0	0.27392816
ENSG00000199460	0.30121252	0	0	0	0	0	0.24189619	0	0.14891687	0	0	0.25437374	0	0	0	0	0	0	0	0	0	0	0	0	0	0	0	0.22712141	0	0	0	0	0	0	0	0	0	0	0.30340037	0	0	0	0	0	0	0	0.53644818	0.28160839
ENSG00000199461	0	0	0	0	0	0	0	0	0	0	0	0	0	0	0	0	0	0	0	0	0	0	0	0	0	0	0	0	0	0	0	0	0	0	0	0	0	0	0	0	0	0	0	0	0	0	0	0
ENSG00000199466	0	0	0	0	0	0	0	0	0	0	0	0	0	0	0	0	0	0	0	0	0	0	0	0	0	0	0	0	0	0	0	0	0	0	0	0	0	0	0	0	0	0	0	0	0	0	0	0
ENSG00000199468	0	0	0	0	0	0	0	0	0	0	0	0	0	0	0	0	0	0	0	0	0	0	0	0	0	0	0	0	0	0	0	0	0	0	0	0	0	0	0	0	0	0	0	0	0	0	0	0
ENSG00000199469	0	0	0	0	0	0	0	0	0	0	0	0	0	0	0	0	0	0	0	0	0	0	0	0	0	0	0	0	0	0	0	0	0	0	0	0	0	0	0	0	0	0	0	0	0	0	0	0
ENSG00000199470	0	0	0	0	0	0	0	0	0	0	0	0	0	0	0	0	0	0	0	0	0	0	0	0	0	0	0	0	0	0	0	0	0	0	0	0	0	0	0	0	0	0	0	0	0	0	0	0
ENSG00000199471	0.31910633	0.42748496	0.62416053	0.8249644	0	0.4787392	1.79386385	0.62513439	0.15776342	0.57804369	0	0.26948506	0	0.69140082	0.27752568	0	0	0	0.70236393	0	0.26862108	0.54508372	0.3232533	0.29667748	0.30462421	0	0.27456562	0.7218413	0	0.25669253	1.93934979	0	0	2.0089119	0	0.28385753	0.59059127	0.32155536	0.32142415	1.53773569	0.59612436	1.71425353	0.36535946	0	0.29435283	0	0	0.2983376
ENSG00000199472	0	0	0	0	0	0	0	0	0	0	0	0	0	0.31174769	0	0	0	0	0	0	0	0	0	0.26753951	0.27470576	0	0	0	0	0	0	0	0	0	0	0	0	0	0	0	0	0	0	0	0	0	0	0
ENSG00000199473	0	0	0	0	0	0	0	0	0	0	0	0	0	0	0	0	0	0	0	0	0	0	0	0	0	0	0	0	0	0	0	0	0	0	0	0	0	0	0	0	0	0	0	0	0	0	0	0
ENSG00000199474	0	0	0	0	0	0	0	0	0	0	0	0	0	0	0	0	0	0	0	0	0	0	0	0	0	0	0	0	0	0	0	0	0	0	0	0	0	0	0	0	0	0	0	0	0	0	0	0
ENSG00000199475	0	0	0	0	0	0	0	0	0	0	0	0	0	0	0	0	0	0	0	0	0	0	0	0	0	0	0	0	0	0	0	0	0	0	0	0	0	0	0	0	0	0	0	0	0	0	0	0
ENSG00000199476	0.59684703	0.3997776	1.16741136	0.7714945	0.49940097	0	0	0.29230821	0	0.54057789	0	0	0	0	0	0	0.89787462	0	0	0	0	0	0.3023017	0	0	0.24559326	0.2567697	0	0	0.24005505	0	0.730228	0	0	0	0	0.2761561	0.60142761	0	0.47935588	0	0	0	0	0.2752744	0	0	0
ENSG00000199477	0	0.4981844	0.72738708	1.2818678	0.41488696	1.11583059	0.7963967	1.69988468	0.73542025	0.67364322	0.624673	1.04684579	1.46890006	2.14866102	2.80300938	0.95665483	2.23777981	2.69147754	0.81852412	1.76108037	0.62609374	2.32918467	0.5022859	0	1.42001746	2.04031327	2.55979642	1.30856871	1.24622444	1.99430347	3.39013261	0.30332548	1.62688751	0	1.93137386	1.54374826	1.37653196	1.74876644	1.74805288	0.79646823	0.92628554	1.33184313	2.55470579	2.5399701	1.60082654	1.36208194	4.85691925	6.02641946
ENSG00000199480	0	0	0	0	0	0	0	0	0	0	0	0	0	0	0	0	0	0	0	0	0	0	0	0	0	0	0	0	0	0	0	0	0	0	0	0	0	0	0	0	0	0	0	0	0	0	0	0
ENSG00000199482	0	0	0	0	0	0	0	0	0	0	0	0	0	0	0	0	0	0	0	0	0	0	0	0	0	0	0	0	0	0	0	0	0	0	0	0	0	0	0	0	0	0	0	0	0	0	0	0
ENSG00000199483	0	0	0	0	0.12750663	0	0	0	0	0	0	0	0	0	0	0	0	0	0	0	0	0	0	0	0	0	0	0	0	0	0	0	0	0	0.44517483	0	0	0	0	0	0	0	0	0	0	0	0	0
ENSG00000199487	0	0	0	0	0	0	0	0	0	0	0	0	0	0	0	0	0	0	0	0	0	0	0	0	0	0	0	0	0	0	0	0	0	0	0	0	0	0	0	0	0	0	0	0	0	0	0	0
ENSG00000199488	0	0	0	0	0	0	0	0	0	0	0	0	0	0	0	0	0	0	0	0	0	0	0	0	0	0	0	0	0	0	0	0	0	0	0	0	0	0	0	0	0	0	0	0	0	0	0	0
ENSG00000199489	0	0	0	0	0	0	0	0	0	0	0	0	0	0	0	0	0	0	0	0	0	0	0	0	0	0	0	0	0	0	0	0	0	0	0	0	0	0	0	0	0	0	0	0	0	0	0	0
ENSG00000199490	0	0	0	0	0	0	0	0	0	0	0	0	0	0	0	0	0	0	0	0	0	0	0	0	0	0	0	0	0	0	0	0	0	0	0	0	0	0	0	0	0	0	0	0	0	0	0	0
ENSG00000199492	0	0	0	0	0	0	0	0	0	0	0	0	0	0	0	0	0	0	0	0	0	0	0	0	0	0	0	0	0	0	0	0	0	0	0	0	0	0	0	0	0	0	0	0	0	0	0	0
ENSG00000199497	0	0	0	0.2408133	0	0	0	0	0.09210466	0	0	0	0	0.2018251	0	0	0	0	0	0	0	0	0	0	0	0	0	0	0	0	0	0	0.61125831	0	0	0.16572029	0	0	0	0	0	0	0	0	0	0	0	0
ENSG00000199506	0	0	0	0	0	0	0	0	0	0	0	0	0	0	0	0	0	0	0	0	0.25355821	0	0	0	0	0	0	0	0	0	0	0	0	0	0	0	0	0	0	0	0	0	0	0	0	0	0	0
ENSG00000199508	0	0	0	0	0	0	0	0	0	0	0	0	0	0	0	0	0	0	0	0	0	0	0	0	0	0	0	0	0	0	0	0	0	0	0	0	0	0	0	0	0	0	0	0	0	0	0	0
ENSG00000199509	0	0	0	0	0	0	0	0	0	0	0	0	0	0	0	0	0	0	0	0	0	0	0	0	0	0	0	0	0	0	0	0	0	0	0	0	0	0	0	0	0	0	0	0	0	0	0	0
ENSG00000199512	0	0	0	0	0	0	0.49774794	0.30355083	0.15321255	0	0	0	0	0.33572828	0	0	0.46620413	0	0	0	0	0	0	0	0	0	0	0	0	0	0	0	0	0	0	0	0.28677749	0	0	0.49779264	0	0.55493464	0	0.26458022	0	0	0	0
ENSG00000199514	0	0	0	0	0	0	0	0	0	0	0	0	0	0	0	0	0	0	0	0	0	0	0	0	0	0	0	0	0	0	0	0	0	0	0	0	0	0	0	0	0	0	0	0	0	0	0	0
ENSG00000199515	0	0	0	0	0	0	0	0	0	0	0	0	0	0	0	0	0	0	0	0	0	0	0	0	0	0	0	0	0	0	0	0	0	0	0	0	0	0	0	0	0	0	0	0	0	0	0	0
ENSG00000199516	0	0	0	0	0	0	0	0	0	0	0	0	0	0	0	0	0	0	0	0	0	0	0	0	0	0	0	0	0	0	0	0	0	0	0	0	0	0	0	0	0	0	0	0	0	0	0.28137233	0
ENSG00000199520	0	0	0.58916087	0	0	0.225947	0	0	0	0	0	0	0	0	0	0	0	0	0	0	0	0	0	0	0	0	0	0	0	0	0	0	0	0	0	0	0	0	0	0	0	0	0	0	0	0	0	0
ENSG00000199523	0	0	0	0	0	0	0	0	0	0	0	0	0	0	0	0	0	0	0	0	0	0	0	0	0	0	0	0	0	0	0	0	0	0	0	0	0	0	0	0	0	0	0	0	0	0	0	0
ENSG00000199525	0	0	0	0	0	0	0	0	0	0	0	0	0	0	0	0	0	0	0	0	0	0	0	0	0	0	0.22918287	0	0	0	0	0	0	0	0	0	0	0	0	0	0	0	0	0	0	0	0	0
ENSG00000199529	0	0	0	0	0	0	0	0	0	0	0	0	0	0	0	0	0	0	0	0	0.25838789	0	0	0	0	0	0	0	0	0	0	0	0	0	0	0	0	0	0	0	0	0	0	0	0	0	0	0
ENSG00000199530	0	0.3997776	0.29185284	0.3857472	0	0.47931283	0	0.14753801	0	0	0	0	0	0	0	0	0	0	0	0	0	0	0.3023017	0	0.28488005	0	0.2567697	0	0	0	0	0	0	0	0	0.26545936	0	0.30071381	0.3005911	0.95871176	0.55748667	0	0	0	0	0	0	0.2790009
ENSG00000199535	0	0	0	0	0	0	0	0	0	0	0	0	0	0	0	0	0	0	0	0	0	0	0	0	0	0	0	0	0	0	0	0	0	0	0	0	0	0	0	0	0	0	0	0	0	0	0	0
ENSG00000199536	0	0	0	0	0	0	0	0	0	0	0	0	0	0	0	0	0	0	0	0	0	0	0	0	0	0	0	0	0	0	0	0	0	0	0	0	0	0	0	0	0	0	0	0	0	0	0	0
ENSG00000199540	0	0	0	0	0	0	0	0	0	0	0	0	0	0	0	0	0	0	0	0	0	0	0	0	0	0	0	0	0	0	0	0	0	0	0	0	0	0	0	0	0	0	0	0	0	0	0	0
ENSG00000199545	0.55093572	0.9225637	0.80820786	0.7121488	0.15366184	2.06635294	0	1.07929186	0.40856681	0.74849247	1.73520279	0.4652648	0.69947622	1.19370056	0.4791469	0.26573745	1.65761468	1.66140589	0.60631416	0.48918899	0.92754628	0.94108471	1.67428634	1.02442479	0.78889859	0.9068059	0.71105456	1.24625591	1.97813403	1.10794637	0	0.67405661	0	0	0.53649274	0.49007881	0.25491333	0	0.83240613	2.21241175	1.02920615	0	0	0.23518242	1.5245967	0.37835609	1.22649476	0.51507859
ENSG00000199546	0	0	0	0	0	0	0	0	0	0	0	0	0	0	0	0	0.44893731	0	0	0	0	0	0	0	0	0	0	0	0	0	0	0.365114	0	0	0	0	0	0	0	0	0	0	0	0	0	0	0	0
ENSG00000199550	0	0	0	0	0	0	0	0	0	0	0	0	0	0	0	0	0	0	0	0	0	0	0	0	0	0	0	0	0	0	0	0	0	0	0	0	0	0	0	0	0	0	0	0	0	0	0	0
ENSG00000199551	0	0	0	0	0	0	0	0	0	0	0	0	0	0	0	0	0	0	0	0	0	0	0	0	0	0	0	0	0	0	0	0	0	0	0	0	0	0	0	0	0	0	0	0	0	0	0	0
ENSG00000199552	0	0	0	0	0	0	0	0	0	0	0	0	0	0	0	0	0	0	0	0	0	0	0	0	0	0	0	0	0	0	0	0	0	0	0	0	0	0	0	0	0	0	0	0	0	0	0	0
ENSG00000199556	0	0	0	0	0	0	0	0	0	0	0	0	0	0	0	0	0	0	0	0	0	0	0	0	0	0	0	0	0	0	0	0	0	0	0	0	0	0	0	0	0	0	0	0	0	0	0	0
ENSG00000199562	0	1.00878461	0.29458044	0	0.50406827	0.225947	0	0.29504006	0	0.54563002	0	0.50874749	0.76484783	0.32631534	0	0.29057273	0	0.36333549	0.33148952	0	0.25355821	0.25725914	0.30512695	0	0	0.24788853	0	0.22712141	0.21630064	0.48459711	0	0	0	0	0	0.53588057	0.557474	0	0	0.48383584	0	0	0	0	0	0	0	0.28160839
ENSG00000199564	0	0	0	0	0	0	0	0	0	0	0	0	0	0	0	0	0	0	0	0	0	0	0	0	0	0	0	0	0	0	0	0	0	0	0	0	0	0	0	0	0	0	0	0	0	0	0	0
ENSG00000199565	0.63195568	0.21164697	0	0	0.35251833	0.23702284	0	0.30950281	0	0	0.39807593	0	0	0	0	0	0	0	0	0	0	0	0	0	0.30163769	0.52007985	0.2718738	0.47650961	0.22690361	0.25417593	2.88050483	0.38659129	0	0	0	1.68644768	0.58480116	0	0.31827293	0	0	0	0	0	0.29146702	0	0	0
ENSG00000199566	0	0	0	0	0	0	0	0	0	0	0	0	0	0	0	0	0	0	0	0	0	0	0	0	0	0	0	0	0	0	0	0	0	0	0	0	0	0	0	0	0	0	0	0	0	0	0	0
ENSG00000199567	0	0	0	0	0	0	0	0	0	0	0	0	0	0	0	0	0	0	0	0	0	0	0	0	0	0	0	0	0	0	0	0	0	0	0	0	0	0	0	0	0	0	0	0	0	0	0	0
ENSG00000199568	4.16763876	3.90817071	5.16277609	10.415176	4.18463574	1.87574969	2.67754062	3.53793732	2.47253361	4.27802161	26.952486	3.51956776	5.40888075	12.9429042	6.76588471	2.94831122	8.77749841	19.7735945	28.1308864	3.9472491	5.14548304	8.06817887	4.50325292	10.8491884	4.77419662	10.0608551	7.17184341	11.5224954	7.18267286	6.92848534	51.0792968	18.6963548	11.395225	25.7997974	9.74011819	13.346198	7.97043651	15.1186458	7.27637775	9.81853077	28.5471621	19.4035766	5.7260647	7.82792508	16.4025576	11.0669158	8.16464875	5.71470811
ENSG00000199570	0	0	0	0.3930255	0	0	0.48835647	0	0.15032175	0	0	0	0	0	0	0	0	0	0.33461678	0	0	0	0	0.28268326	0.58051028	0.7506813	0.52322883	0.45852812	0.43668242	0	0	0	0	0	0	0	0.30638765	0	0.48840033	0	0	0	0	0	0	0	0	0
ENSG00000199571	0	0.32220881	0.23522468	0	0.13416743	0	0.19315592	0.23559169	0	0.21784482	0	0	0	0.52113047	0	0	0	0	0	0	0	0	0	0	0	0	0	0	0	0	0	0	0	0	0	0	0.24236635	0.24226746	0	0	0	0	0	0	0	0	0	0
ENSG00000199572	0.27313339	0	0	0	0.20488415	0.21934655	0	0.27006958	0.2473831	1.03229861	0.23066094	0	0	0	0	0	0	0	0	0	0	0	0	0	0	0	0	0	0.19613702	0	0	0	0	0	0	0	0.55045917	0	0	0	0	0.48909493	0	0	0	0	0	0
ENSG00000199574	0	0	0	0	0	0	0	0	0	0	0	0	0	0	0	0	0	0	0	0	0	0	0	0	0	0	0	0	0	0	0	0	0	0	0	0	0	0	0	0	0	0	0	0	0	0	0	0
ENSG00000199575	2.23817637	1.49916601	2.62667555	1.7358626	0.99880195	3.02204117	0	0	0	0	0	0	2.65218066	2.90964513	2.33584115	3.02276352	6.06065366	1.61987074	8.37471437	7.94932111	4.8986038	6.49936631	21.7657225	4.57789827	7.26444115	6.63101814	23.8795823	6.07549758	9.3219566	13.3230551	1.36023839	0	2.93743578	0	0	0.39818903	0	0	0	0	0	0	2.56259068	3.82171427	2.47746964	0.61482865	3.58749718	3.76651216
ENSG00000199577	0	0	0	0	0	0	0	0	0	0	0	0	0	0	0	0	0	0	0	0	0	0	0	0	0	0	0	0	0	0	0	0	0	0	0	0	0	0	0	0	0	0	0	0	0	0	0	0
ENSG00000199580	0	0	0	0	0	0	0	0	0	0	0	0	0	0	0	0	0	0	0	0	0	0	0	0	0	0	0	0	0	0	0	0	0	0	0	0	0	0	0	0	0	0	0	0	0	0	0	0
ENSG00000199584	0	0	0	0	0	0	0	0	0	0	0	0	0	0	0	0	0	0	0	0	0	0	0	0	0	0	0	0	0	0	0	0	0	0	0	0	0	0	0	0	0	0	0	0	0	0	0	0
ENSG00000199585	0	0	0	0	0	0	0	0	0	0	0	0	0	0	0	0	0	0	0	0	0	0	0	0	0	0	0	0	0	0	0	0	0	0	0	0	0	0	0	0	0	0	0	0	0	0	0	0
ENSG00000199591	0	0.20175692	0	0	0	0.225947	0.24189619	0.29504006	0	0.27281501	0	0	0	0	0	0	0.45313298	0	0.33148952	0	0	0	0	0	0	0	0.25916942	0.45424281	0	0.24229855	0	0	0	0	0	0	0	0	0	0	0	0	0	0	0	0	0	0
ENSG00000199592	0	0	0	0	0	0	0	0	0	0	0	0	0	0	0	0	0	0	0	0	0	0	0	0	0	0	0	0	0	0	0	0	0	0	0	0	0	0	0	0	0	0	0	0	0	0	0	0
ENSG00000199593	0.42972986	0.57567975	3.36214471	1.6664281	0.23971247	1.93410635	0	0	0	0	0	0	2.54609343	1.86217288	1.49493834	5.38915553	5.17175779	6.22030364	5.67510056	6.48664602	13.7462359	11.7447372	22.2010369	5.19383367	11.8965907	16.9754064	28.1008762	15.2292473	12.0349674	19.0123598	1.30582886	1.05152832	0	0.67633367	0	0.38226147	0	0	0	0	0	0	6.88824376	2.56819199	4.75674172	1.18047101	3.44399729	5.62465816
ENSG00000199594	0	0	0	0	0	0	0	0	0	0	0	0	0	0	0	0	0	0	0	0	0	0	0	0	0	0	0	0	0	0	0	0	0	0	0	0	0	0	0	0	0	0	0	0	0	0	0	0
ENSG00000199595	0	0	0	0	0	0	0	0	0	0	0	0	0	0	0	0	0	0	0	0	0	0	0	0	0	0	0	0	0	0	0	0	0	0	0	0	0	0	0	0	0	0	0	0	0	0	0	0
ENSG00000199598	0	0	0	0	0	0	0	0	0	0	0	0	0	0	0	0	0	0	0	0	0	0	0	0	0	0	0	0	0	0	0	0	0	0	0	0	0	0	0	0	0	0	0	0	0	0	0	0
ENSG00000199601	0	0	0	0	0	0	0	0	0	0	0	0	0	0	0	0	0	0	0	0	0	0	0	0	0	0	0	0	0	0	0	0	0	0	0	0	0	0	0	0	0	0	0	0	0	0	0	0
ENSG00000199603	0	0	0	0	0	0	0.72568858	1.77024038	0.29783375	0.81844504	0.37947425	0.25437374	0	0	0	0	0	0	0	0.26745379	0	0.51451828	0	0	0	0	0.25916942	0	0.21630064	0	0	0	0	0	0	0	0.83621101	0	0.30340037	0.48383584	0.56269682	0	0	0	0	0	0	0.28160839
ENSG00000199605	0	0	0	0	0	0	0	0	0	0	0	0	0	0	0	0	0	0	0	0	0	0	0	0	0	0	0	0	0	0	0	0	0	0	0	0	0	0	0	0	0	0	0	0	0	0	0	0
ENSG00000199609	0	0	0	0	0	0	0	0	0	0	0	0	0	0	0	0	0	0	0	0	0	0	0	0	0	0	0	0	0	0	0	0	0	0	0	0	0	0	0	0	0	0	0	0	0	0	0	0
ENSG00000199620	0	0	0	0	0	0	0	0	0	0	0	0	0	0	0	0	0	0	0	0	0	0	0	0	0	0	0	0	0	0	0	0	0	0	0	0	0	0	0	0	0	0	0	0	0	0	0	0
ENSG00000199622	0	0	0	0	0	0	0	0	0	0	0	0	0	0	0	0	0	0	0	0	0	0	0	0	0	0	0	0	0	0	0	0	0	0	0	0	0	0	0	0	0	0	0	0	0	0	0	0
ENSG00000199626	0	0	0	0	0	0	0	0	0	0	0	0	0	0	0	0	0	0	0	0	0	0	0	0	0	0	0	0	0	0	0	0	0	0	0	0	0	0	0	0	0	0	0	0	0	0	0	0
ENSG00000199627	0	0.20175692	0.58916087	0.3893524	0.33604551	0.45189401	0	0	0.14891687	0	0	0.50874749	0	0.32631534	0	0	0.90626597	0	0.33148952	0	0	0.25725914	0.30512695	0	0	0	0	0.22712141	0	0	0	0	0.49414808	0	0	0	0	0	0.30340037	0	0	0	0.34487202	0.51432416	1.38923531	0	0.26822409	0.28160839
ENSG00000199629	0	0	0	0	0	0	0	0	0	0	0	0	0	0	0	0	0	0	0	0	0	0	0	0	0	0	0	0.15988152	0	0	0	0	0	0	0	0	0	0	0	0	0	0	0	0	0	0	0	0
ENSG00000199630	0.33572646	0.4497498	0	0	0	0.75551029	0.26961347	0.65769348	0.99588159	0	0.84591136	0.28352074	0.28416221	0	0	0	0	0	0.73894539	0.29809954	0	0	1.02026824	0.31212943	0.32049005	0.27629242	0	0	0.24108508	0.27006193	0	0	0	0	0	0.29864178	0	0.33830303	0	1.61782609	0.6271725	0.60117919	0	0.85988571	0	0	0	0.94162804
ENSG00000199631	0	0	0	0	0	0	0	0	0	0	0	0	0	0	0	0	0	0	0	0	0	0	0	0	0	0	0	0	0	0	0	0	0	0	0	0	0	0	0	0	0	0	0	0	0	0	0	0
ENSG00000199633	0	0	0	0	0	0	0	0	0	0	0	0	0	0	0	0	0	0	0	0	0	0	0	0	0	0	0	0	0	0.20576147	0	0	0	0	0	0	0	0	0	0	0	0	0	0	0	0	0	0
ENSG00000199634	0	0	0	0	0	0	0	0	0	0	0	0	0	0	0	0	0	0	0	0	0	0	0	0	0	0	0	0	0	0	0	0	0	0	0	0	0	0	0	0	0	0	0	0	0	0	0	0

ENSG00000199635	0	0	0	0	0	0	0	0	0	0	0	0	0	0	0	0	0	0	0	0	0	0	0	0	0	0	0	0	0	0	0	0	0	0	0	0	0	0	0	0	0	0	0	0	0	0	0	0
ENSG00000199636	0	0	0	0	0	0	0	0	0.15175339	0.55602298	0	0.25921896	0.25980545	0	0.80085982	0.29610745	0	0	0.3378036	0	0.25838789	0	0	0	0	0	0.26410598	0.23144753	0.22042065	0.24691376	0	0	0.50356042	0	0	0	0.85213883	0.30930563	0	0	0	0	0	0.52412081	0.56627878	0.42159679	0	0
ENSG00000199638	0	0	0	0	0	0	0	0	0	0	0	0	0	0	0	0	0	0	0	0	0	0	0	0	0	0	0	0	0	0	0	0	0	0	0	0	0	0	0	0	0	0	0	0	0	0	0	0
ENSG00000199640	0	0	0	0	0	0	0	0	0	0	0	0	0	0	0	0	0	0	0	0	0	0	0	0	0	0	0	0	0	0	0	0	0	0	0	0	0	0	0	0	0	0	0	0	0	0	0	0
ENSG00000199641	0	0	0	0	0	0	0	0	0	0	0	0	0	0	0	0	0	0	0	0	0	0	0	0	0	0	0	0	0	0	0	0	0	0	0	0	0	0	0	0	0	0	0	0	0	0	0	0
ENSG00000199643	0	0	0	0	0	0	0	0	0	0	0	0	0	0	0	0	0	0	0	0	0	0	0	0	0	0	0	0	0	0	0	0	0	0	0	0	0	0	0	0	0	0	0	0	0	0	0	0
ENSG00000199645	0.61389981	2.0559991	3.00191492	0	2.22590148	2.0722568	0.49300748	0.30065987	0.45526016	1.11204595	0	0.51843792	0.5196109	0	0	0.88832234	0.92352818	0.37025617	0.3378036	0.27254815	0.25838789	0	0.93281668	1.14150191	0.29301948	0.25261021	0	0	0.22042065	0.49382753	0	0.75109166	1.00712084	0	0	0.81913173	0	0.30930563	0.30917942	0.49305176	0	2.19859817	0.70288202	0.26206041	0.28313939	0.84319358	0.27333312	0.28697236
ENSG00000199646	0	0.40351384	0.88374131	0.3893524	0.33604551	1.35568202	0.24189619	0.59008013	0	0.27281501	0.37947425	0	0	0	0	0.29057273	0	0	0	0	0	0	0	0	0	0	0.25916942	0	0.21630064	0.24229855	0.4576503	0.73705256	1.48244423	0.47406566	0.58663225	0	0	0	0.30340037	0	0	0.53937572	0	0.25716208	0	0	0	0
ENSG00000199652	0	0	0	0	0	0	0	0	0	0	0	0	0	0	0	0	0	0	0	0	0	0	0	0	0	0	0	0	0	0	0	0	0	0	0	0	0	0	0	0	0	0	0	0	0	0	0	0
ENSG00000199664	0.3069499	0.20559991	0.30019149	0	0	0.23025076	0.24650374	0.30065987	0.45526016	0.55602298	0	0.77765688	0.25980545	0	0.26695327	0	0	0.37025617	0.3378036	0	0.25838789	0.26215931	0.31093889	0	0	0	0.26410598	0.23144753	0.22042065	0	0	0	0	0	1.19561239	0.81913173	0	0	0.30917942	0.49305176	0	0.54964954	0	0	0.28313939	0.42159679	0	0
ENSG00000199666	0	0	0	0	0	0	0	0	0	0	0	0	0	0	0	0	0	0	0	0	0	0	0	0	0	0	0	0	0	0	0	0	0	0	0	0	0	0	0	0	0	0	0	0	0	0	0	0
ENSG00000199667	0.31597784	0.21164697	0	0	0	0.23702284	0	0.30950281	0.15621672	0	0	0	0.26744679	0.34231119	0	0	0.47534539	0	0	0	0.26598754	0	0	0	0	0	0	0	0	0.25417593	0.48008414	0	0.51837102	0.99460834	0	0.28107461	0	0.31840285	0	0	0	0	0	0	0	0	0	0
ENSG00000199668	0	0	0	0	0	0	0	0	0	0	0	0	0	0.31174769	0	0	0	0	0	0	0	0	0	0	0	0	0	0	0.20664436	0	0	0	0	0.45290201	0	0	0	0	0	0	0	0	0	0	0	0	0	0
ENSG00000199672	0.51158317	0.17133326	0	1.322562	0.14268599	0.57562689	0.41083957	0.2505499	0.50584462	0.46335248	0.32225195	0	0	0.27710906	0.22246106	0	0.76960681	0.61709361	0	0.22712346	0.21532324	0.43693219	0.25911574	0	0	0.21050851	0.22008832	0.38574588	0.36736775	0	0	0	0	0.40257957	0	0	0.47341046	0	0	0	0	0.45804129	0	0.21838367	0	0	0.6833328	0.71743089
ENSG00000199673	0	0	0	0	0	0	0	0	0	0	0	0	0	0	0	0	0	0	0	0	0	0	0	0	0	0	0	0	0	0	0	0	0	0	0	0	0	0	0	0	0	0	0	0	0	0	0	0
ENSG00000199674	0.3069499	0.41119982	0	0	0	0	0	0.30065987	0	0	0.38670234	0	0	0.33253087	0.53390655	0.29610745	0	0.37025617	0.67560721	0	0	0.26215931	0	0	0.29301948	0	0	0	0	0.24691376	0	0	0.50356042	0	0	0	0	0.30930563	0.30917942	0	0	0	0.35144101	0	0.28313939	0	0	0.57394471
ENSG00000199676	0	0	0	0	0	0	0	0	0	0	0	0	0	0	0	0	0	0	0	0	0	0	0	0	0	0	0	0	0	0	0	0	0	0	0	0	0	0	0	0	0	0	0	0	0	0	0	0
ENSG00000199677	0.31597784	0	0	0	0	0	0	0	0	0.2861883	0	0	0	0	0	0	0	0	0	0	0	0.26986988	0	0	0	0.26003993	0	0	0	0	0.48008414	0	0	0	0	0	0.58480116	0	0.31827293	0	0	0	0	0	0	0	0	0
ENSG00000199678	0	0	0	0	0	0	0	0	0	0	0	0	0	0	0	0	0	0	0	0	0	0	0	0	0	0	0	0.25314573	0	0	0	0	0	0	0	0	0	0	0	0	0	0	0	0	0	0	0	0
ENSG00000199683	21.9478596	11.7873077	20.4977381	68.11397	11.1951605	61.8498733	17.6257735	19.8518522	11.2907312	12.715188	8.22039013	8.26558612	6.35965493	7.92565912	8.34024263	3.62413716	3.56946473	1.9080686	0.97921597	9.74401446	8.90487109	8.6126572	0.60089418	9.19154142	75.0300635	94.3801354	37.8538402	67.3150223	45.6493868	61.3951829	66.6934063	59.753258	40.3852368	69.396814	56.2231226	51.0952261	145.098855	90.9557796	112.528032	46.6886745	43.7712538	59.8376144	34.8638104	85.2500188	72.6825757	41.4160651	85.0434913	57.1215832
ENSG00000199687	0	0	0	0	0	0	0	0	0	0	0	0	0	0	0	0	0	0	0	0	0	0	0	0	0	0	0	0	0	0	0	0	0	0	0	0	0	0	0	0	0	0	0	0	0	0	0	0
ENSG00000199691	15.2451785	18.5725252	14.6093193	20.631967	13.7549296	24.9438319	12.2430191	19.4426719	11.8367641	13.6225629	18.9484144	14.1706364	9.09319082	12.081955	13.7925858	7.10657872	11.2362595	12.0950349	5.63006008	14.3542027	10.5939036	8.38909803	8.0844112	9.7027662	11.9161253	12.5463073	11.0924511	13.8868516	10.6536647	9.62963676	10.2600839	11.6419207	10.5747688	7.56849586	11.159049	12.013932	8.71075252	14.9497721	9.58456204	10.1897364	12.6151269	10.6265578	13.1204643	18.256875	18.6871996	15.1774845	23.5977592	18.844518
ENSG00000199695	0	0	0	0	0	0	0	0	0	0	0	0	0	0	0	0	0	0	0	0	0	0	0	0	0	0	0	0	0	0	0	0	0	0	0	0	0	0	0	0	0	0	0	0	0	0	0	0
ENSG00000199697	0	0	1.18943799	0.3930255	0.16960788	0.22807858	0.7325347	0.29782346	0.9019305	0.82616621	0.3830542	1.02709399	0	0	1.05773939	0	0	0	0.33461678	1.8898386	0.51190054	0.25968611	0.92401652	0.28268326	0.58051028	0.2502271	0.52322883	0.45852812	0	1.22292194	0.92393551	0	1.49642955	0.95707595	0.59216651	0.54093605	0.56273319	0	0.30626263	0.97680066	0.56800528	0.54446417	0	0.51917628	0.84140478	0.83523892	0	0.56853014
ENSG00000199698	0	0	0	0	0	0	0	0	0	0	0	0	0	0	0	0	0	0	0	0	0	0	0	0	0	0	0	0	0	0	0	0	0	0	0	0	0	0	0	0	0	0	0	0	0	0	0	0
ENSG00000199700	0	0	0	0	0	0	0	0	0	0	0	0	0	0	0	0	0	0	0	0	0	0	0	0	0	0	0	0	0	0	0	0	0	0	0	0	0	0	0	0	0	0	0	0	0	0	0	0
ENSG00000199701	0	0	0	0	0	0	0	0	0.16259291	0	0	0	0	0	0	0	0	0	0	0	0	0	0	0	0	0	0	0	0	0	0	0	0	0	0	0	0	0.33139889	0	0	0	0	0	0	0	0	0	0
ENSG00000199702	0	0	0	0	0	0	0	0	0	0	0	0	0	0	0	0	0	0	0	0	0	0	0	0	0	0	0	0	0.22254008	0	0	0	0	0	0	0	0	0	0	0.99558529	0	0	0	0	0	0	0	0
ENSG00000199704	0	0	0	0	0	0	0	0	0	0	0	0	0	0	0	0	0	0	0	0	0	0	0	0	0	0	0	0	0	0	0	0	0	0	0	0	0	0	0	0	0	0	0	0	0	0	0	0
ENSG00000199705	0	0	0	0	0	0	0	0	0	0	0	0	0	0	0	0	0	0	0	0	0	0	0	0	0	0	0	0	0	0	0	0	0	0	0	0	0	0	0	0	0	0	0	0	0	0	0	0
ENSG00000199709	0	0.4625998	0.22514362	0.2975764	0.51366957	0.17268807	0	0.45098981	0.45526016	0	0.29002675	0.38882844	0	0.24939815	0	0	0	0	0.2533527	0	0.19379092	0	0	0.64209482	0	0.18945766	0.39615897	0.34717129	0	0	0	0.84497811	0	0.36232161	0.89670929	0	0.42606942	0.46395844	0.6956537	0.36978882	0	0.41223716	0	0	0	0	0.20499984	0
ENSG00000199710	0	0	0	0	0	0	0	0	0	0	0	0	0	0	0	0	0	0	0	0	0	0	0	0	0	0	0	0	0	0	0	0	0	0	0	0	0	0	0	0	0	0	0	0	0	0	0	0
ENSG00000199711	0	0	0	0	0	0	0	0	0	0	0	0	0	0	0	0	0	0	0	0	0	0	0	0	0	0	0	0	0	0	0	0	0	0	0	0	0	0	0	0	0	0	0	0	0	0	0	0
ENSG00000199712	0	0	0	0	0	0	0	0	0	0	0	0	0	0	0.34183041	0	0	0	0	0	0	0	0	0	0	0	0	0	0	0	0	0	0	0	0	0	0	0	0	0	0	0	0	0	0	0	0	0
ENSG00000199713	0	0	0	0	0	0	0	0	0	0	0	0	0	0	0	0	0	0	0	0	0	0	0	0	0	0	0	0	0	0	0	0	0	0	0	0	0	0	0	0	0	0	0	0	0	0	0	0
ENSG00000199715	0	0	0	0	0	0	0	0	0	0	0	0	0	0	0	0	0	0	0	0	0	0	0	0	0	0	0	0	0	0	0	0	0	0	0	0	0	0	0	0	0	0	0	0	0	0	0	0
ENSG00000199716	0	0	0	0	0	0	0	0	0	0	0	0	0	0	0	0	0	0	0	0	0	0	0	0	0	0	0	0	0	0	0	0	0	0	0	0	0	0	0	0	0	0	0	0	0.29729636	0	0	0
ENSG00000199719	1.98176393	2.23564951	2.24414999	2.8313099	1.3963833	3.12962193	1.42397791	1.73682161	1.49543385	1.41704885	2.36526671	1.49742991	0.70626725	0.90396742	2.35851922	1.10680939	1.09837089	2.01304325	1.72181449	1.6670421	1.05362053	1.42533219	1.69054155	0.67880575	2.98709174	1.88844529	2.15387401	1.73023879	1.12350331	1.84586017	0.95084625	1.78657724	0.85556382	2.29822122	2.23451831	1.94842013	2.21997334	2.62759636	2.62652421	1.67541861	3.31244505	2.98838587	1.79132553	3.29483715	2.11667309	1.86239359	3.06504613	3.90059512
ENSG00000199727	0	0	0	0	0	0	0	0	0	0	0	0	0	0	0	0	0	0	0	0	0	0	0	0	0	0	0	0	0	0	0	0	0	0	0	0	0	0	0	0	0	0	0	0	0	0	0	0
ENSG00000199728	0	0	0	0	0	0	0	0	0	0	0	0	0	0	0	0	0	0	0	0	0	0	0	0	0	0	0	0	0	0	0	0	0	0	0	0	0	0	0	0	0	0	0	0	0	0	0	0
ENSG00000199730	0.21273756	0.07124749	0.20805351	0	0.11866954	0.07978987	0.08542209	0	0.05258781	0.09634061	0	0	0.09003159	0.11523347	0	0	0.16001726	0.12830659	0	0	0	0	0	0	0	0.08753819	0.09152187	0	0.22915018	0.17112835	0	0	0	0.50222797	0	0	0	0	0.10714138	0.17085952	0.39741624	0	0.24357298	0.09081301	0.09811761	0.1460979	0	0
ENSG00000199731	0.29299763	0.39250892	0.57309285	0.7574673	0.16344032	0.21978481	0	0.28699352	0.57942202	0	0	0.24743628	0.24799611	0	0	0.28264802	0	0.70685269	0.3224489	0.2601596	0	0.25024298	0.59361061	0	0.27970041	1.20563966	0.50420232	0	0.42080306	0.23569041	0	0.71695113	0.48067131	0.4611366	0.57063319	0.26063282	0	0	0	0	0	1.04933095	0	0	0	0.4024333	0	0
ENSG00000199732	0	0	0	0	0	0	0	0	0	0	0	0	0	0	0	0	0	0	0	0	0	0	0	0	0	0	0	0	0.22690361	0	0.48008414	0	0	0	0	0	0	0	0	0	0	0	0	0	0.29146702	0.4339967	0	0.29541272
ENSG00000199733	0	0	0	0	0	0	0	0	0	0	0	0	0	0.30099777	0	0	0	0	0	0	0	0	0	0	0	0	0	0	0	0	0	0	0	0	0	0	0	0	0	0	0.51903931	0	0	0	0	0	0	0
ENSG00000199735	0	0	0	0	0	0	0	0	0	0	0	0	0	0	0	0	0	0	0	0	0	0	0	0	0	0	0	0	0	0	0	0	0	0	0	0	0	0	0	0	0	0	0	0	0	0	0	0
ENSG00000199739	0	0	0	0	0	0	0	0	0	0	0	0	0	0	0	0	0	0	0	0	0	0	0	0	0	0	0	0	0	0	0	0	0	0	0	0	0	0	0	0	0	0	0	0	0	0	0	0
ENSG00000199740	0	0	0	0	0	0	0	0	0	0.29191206	0.40603745	0	0	0	0	0	0	0.38876898	0	0	0	0	0	0	0	0.26524073	0	0.2430199	0	0	0.48968582	0.39432312	0	1.52175076	0	0	0	0	0	0	0	0	0.36901306	0	0.29729636	0	0	0
ENSG00000199744	0	0	0	0	0	0	0	0	0	0	0	0	0	0	0	0	0	0	0	0	0	0	0	0	0	0	0	0	0	0	0	0	0	0	0	0	0	0	0	0	0	0	0	0	0	0	0	0
ENSG00000199751	0	0	0	0.4084383	0	0	0	0	0	0.57237659	0	0	0	0	0.27480484	0.30481649	0	0	0	0	0.26598754	0	0	0	0	0	0	0	0	0	0	0	0	0	0	0	0	0	0	0	0	0	0	0	0	0	0	0
ENSG00000199753	7.36679766	6.78479704	9.45603199	13.093364	6.67770443	15.1965499	24.4038702	41.0400729	35.055032	21.6848961	34.8032102	45.1040988	19.0957007	11.9711114	16.4176264	9.32738458	5.54116906	17.2169119	38.509611	30.6616671	42.2464205	16.9092757	8.3953501	26.9679825	24.1741067	34.102379	38.0312611	24.3019903	23.4747991	22.96298	4.89685821	5.63318742	12.0854501	4.34785933	3.58683718	14.7443711	35.789831	49.6435535	56.579834	33.2809939	65.3692938	32.9789726	17.9234914	23.5854366	25.0578358	18.9718556	15.9899874	24.1056778
ENSG00000199755	0	0	0	0	0	0	0	0	0	0	0	0	0	0	0	0.28524112	0	0	0.32540714	0	0	0	0	0	0	0	0	0	0	0	0	0	0	0	0	0	0	0	0	0	0	0	0	0	0	0	0	0
ENSG00000199756	0	0	0	0	0	0	0	0	0	0	0	0	0	0	0	0	0	0	0	0	0	0	0	0	0	0	0	0	0	0	0	0	0	0	0	0	0	0	0	0	0	0	0	0	0	0	0	0
ENSG00000199762	0	0	0	0	0	0	0.24887397	0	0.30642511	0.28068468	0	0.26171145	0	0	0	0	0	0	0	0.27516881	0.26087239	0.26468008	0.31392869	0	0	0	0	0.23367298	0	0	0	0	0	0	0	0	0	0	0	0	0	0.55493464	0	0	0	0.42565061	0	0
ENSG00000199764	0	0	0	0	0	0	0	0	0	0	0	0	0	0	0	0	0	0	0	0	0	0	0	0	0	0	0	0	0	0	0	0	0	0	0	0	0	0	0	0	0	0	0	0	0	0	0	0
ENSG00000199765	0	0	0	0	0	0	0	0	0	0	0	0	0	0	0	0	0	0	0	0	0	0	0	0	0	0	0	0	0	0	0	0	0	0	0	0	0	0	0	0	0	0	0	0	0	0	0	0
ENSG00000199767	0	0	0	0	0	0	0	0	0	0	0	0	0	0	0	0	0	0	0	0	0	0	0	0	0	0	0	0	0	0	0	0	0	0	0	0	0	0	0	0	0	0	0	0	0	0	0	0
ENSG00000199769	0	0	0	0	0	0	0	0	0	0	0.37770926	0	0	0	0	0	0	0	0	0	0	0	0	0	0	0	0	0	0	0	0	0	0	0	0	0	0	0	0	0	0	0	0	0	0	0	0	0
ENSG00000199771	0	0	0	0	0	0	0	0	0	0	0	0	0	0	0	0	0	0	0	0	0	0	0	0	0	0	0	0	0	0	0	0	0	0	0	0	0	0	0	0	0	0	0	0	0	0	0	0
ENSG00000199773	0	0	0	0	0	0	0	0	0	0	0	0	0	0	0	0	0	0	0	0	0	0	0	0	0	0	0	0	0	0	0	0	0	0	0	0	0	0	0	0	0	0	0	0	0	0	0	0
ENSG00000199780	0	0	0	0	0	0	0	0	0	0	0	0	0	0	0	0	0	0	0	0	0	0	0	0	0	0	0	0	0	0	0	0	0	0	0	0	0	0	0	0	0	0	0	0	0	0	0	0
ENSG00000199781	0.97665878	0.21806051	0.63676983	0.4208152	0.18160035	0.24420535	0.26144336	0	0.16095056	0.58972134	0	0	0	0	0	0.31405335	0	0	0.35827655	0	0.8221433	0	0	0	0	0	0	0	0.23377948	0	0.49463214	0.79661236	0	0	0	0	0	0	0.32791757	0	0	0	0.74548093	0	0	0	0	0.30436462
ENSG00000199782	0	0	0	0	0	0	0	0	0	0	0	0	0	0	0	0	0	0	0	0	0	0	0	0	0	0	0	0	0	0	0	0	0	0	0	0	0	0	0	0	0	0	0	0	0	0	0	0
ENSG00000199783	0	0	0	0	0	0	0	0	0	0	0	0	0	0	0	0	0	0	0	0	0	0	0	0	0	0	0	0	0	0	0	0	0	0	0	0	0	0	0	0	0	0	0	0	0	0	0	0
ENSG00000199784	0	0	0	0	0	0	0	0	0	0	0	0	0	0	0	0	0	0	0	0	0	0	0	0	0	0	0	0	0	0	0	0	0	0	0	0	0	0	0	0	0	0	0	0	0	0	0	0
ENSG00000199785	0	0	0	0	0	0	0	0	0	0	0	0	0	0	0	0	0	0	0	0	0	0	0	0	0	0	0	0	0	0	0	0	0	0	0	0	0	0	0	0	0	0	0	0	0	0	0	0
ENSG00000199786	0	0	0	0	0	0	0	0	0	0	0	0	0	0	0	0	0	0	0	0	0	0	0	0	0	0	0	0	0	0	0	0	0	0	0	0	0	0	0	0	0	0	0	0	0.23046229	0	0	0
ENSG00000199787	0	0.15991104	0	0	0.13317359	0.35816784	0	0.23384657	0.23606082	0.21623116	0.30076848	0.40322949	0	0	0	0	0	0	0	0	0	0.20096836	0.20390169	0	0	0	0.61624729	0.18001474	0.17143828	0	0	0	0	0	0	0.21236749	0	0.24057104	0.24047288	0	0.44598933	0.4275052	0	0	0	0	0	0
ENSG00000199788	0.31597784	0.21164697	0	0	0.17625917	0	0.25375385	0	0	0	0	0	0	0	0	0	0	0.38114606	0.34773901	0	0.26598754	0	0	0	0	0	0	0.23825481	0	0	0	0	0	0.99460834	0	0	0.29240058	0.31840285	0.31827293	0	0	0.56581571	0	0	0	0	0	0
ENSG00000199790	0	0	0	0	0	0	0	0	0	0	0	0	0	0	0	0	0	0	0	0	0	0	0	0	0	0	0	0	0	0	0	0	0	0	0	0	0	0	0	0	0	0	0	0	0	0	0	0
ENSG00000199791	0	0	0	0	0	0	0	0	0	0	0	0	0	0	0	0	0	0	0	0	0	0	0	0	0	0	0	0	0	0	0	0	0	0	0	0	0	0	0	0	0	0	0	0	0	0	0	0
ENSG00000199792	0	0	0	0	0	0	0	0	0	0	0	0	0	0	0	0	0	0	0	0	0	0	0	0	0	0	0	0	0	0	0	0	0	0	0	0	0	0	0	0	0	0	0	0	0	0	0	0
ENSG00000199796	0	0	0	0	0	0	0	0	0	0	0	0	0	0	0	0	0	0	0	0	0	0	0	0	0	0	0	0	0	0	0	0	0	0	0	0	0	0	0	0	0	0	0	0	0	0	0	0
ENSG00000199797	0	0	0	0	0	0	0	0	0	0	0	0	0	0	0	0	0	0	0	0	0	0	0	0	0	0	0	0	0	0	0	0	0	0	0	0	0	0	0	0	0	0	0	0	0	0	0	0
ENSG00000199798	0	0	0	0.5951529	0.25683479	0	0	0	0	0	0	0	0	0	0.80085982	0	0	0	0.50670541	0.40882223	0.38758184	0.78647794	0.93281668	0	1.75811685	0	0.79231794	1.04151387	1.32252389	2.22222387	0	0	0	0	0	0	0	0	0	0	0	0	0	0	0.42470908	0	0	0
ENSG00000199801	0	0	0	0	0	0	0	0	0	0	0	0	0	0	0	0	0	0	0	0	0	0	0	0	0	0	0	0	0	0	0	0	0	0	0	0	0	0	0	0	0	0	0	0	0	0	0	0
ENSG00000199803	0	0	0	0	0	0	0	0	0	0	0	0	0	0	0	0	0	0	0	0	0	0	0	0	0	0	0	0	0	0	0	0	0	0	0	0	0	0	0	0	0	0	0	0	0	0	0	0
ENSG00000199804	1.07432466	0.89949961	0	0	0	0.40293882	1.5098354	1.05230956	0.53113685	0.4865201	0.67672909	0.45363318	0.68198931	0.87289354	0.70075234	0.51818803	0	0.32397415	0.88673446	0.7154389	0	1.146947	0	0.49940708	0.51278408	0.66310181	0.23109273	1.01258293	0.19286807	0	0	0.3286026	0.88123074	0	0	0.47782684	0	0	0.27053199	0.43142029	0.501738	0.48094335	1.23004353	0.91721142	1.48648179	0	0.23916648	1.50660487
ENSG00000199805	0	0.13083631	0	0	0	0.29304642	0.15686602	0.38265802	0.38628134	0.1769164	0.2460833	0	0	0.21161055	0.16987936	0.37686402	0.29384987	0	0.42993186	0.17343973	0.32885732	0.33365731	0.39574041	0.18160258	0.37293388	0.48225586	0.33613488	0.29456958	0.42080306	0.15712694	0	0.47796742	0.64089508	0.3074244	0	0.17375522	0.18075672	0.19683085	0.39350108	1.56880106	0.72980073	0.34977698	0	0.16676571	0.54053883	0	0.52181777	0.18261877
ENSG00000199806	0	0	0	0	0	0	0	0	0	0	0	0	0	0	0	0	0	0	0	0	0	0	0	0	0	0	0	0	0	0	0	0	0	0	0	0	0	0	0	0	0	0	0	0	0	0	0	0
ENSG00000199809	0	0	0	0	0	0	0	0	0	0	0	0	0	0	0	0	0	0	0	0	0	0	0	0	0	0	0	0	0	0	0	0	0	0	0	0	0	0	0	0	0	0	0	0	0	0	0	0
ENSG00000199812	0	0	0	0	0	0	0	0	0	0	0	0	0	0	0	0	0	0	0	0	0	0	0	0	0	0	0	0	0	0	0	0	0	0	0	0	0	0	0	0	0	0	0	0	0	0	0	0
ENSG00000199814	0	0	0	0	0	0	0	0	0	0	0	0	0	0	0	0	0	0	0	0	0	0	0	0	0.28754248	0	0	0	0	0	0	0	0	0	0	0	0	0.30352422	0	0	0	0	0	0	0	0	0	0
ENSG00000199815	0	0	0	0	0	0	0	0	0	0	0	0	0	0	0	0	0	0	0	0	0	0	0	0	0	0	0	0	0	0	0	0	0	0	0	0	0	0	0	0	0	0	0	0	0	0	0	0
ENSG00000199824	0	0	0	0	0	0	0	0	0	0	0	0	0	0	0	0	0	0	0	0	0	0	0	0	0	0	0	0	0	0	0	0	0	0	0	0	0	0	0	0	0	0	0	0	0	0	0	0
ENSG00000199827	0	0	0	0	0	0	0	0	0	0	0	0	0	0	0	0	0	0	0	0	0	0	0	0	0	0	0	0	0	0	0	0	0	0	0	0	0	0	0	0	0	0	0	0	0	0	0	0
ENSG00000199831	4.53384719	5.21544726	4.24123759	6.7523462	3.46373519	5.6189634	2.92864535	4.24785511	3.36224244	3.03517129	6.82937611	3.6623596	3.33695076	2.56262323	3.77163341	1.14096447	2.66891171	5.3500318	2.92866428	3.41310301	2.3231205	2.44120828	3.49449673	2.65739549	3.3871976	4.2990087	2.54414017	4.60771682	3.32653181	2.37852708	5.39103656	2.77352653	3.07218055	2.01659123	1.15173671	3.6823353	3.64830082	5.95909928	6.35377892	3.64134558	5.5237211	4.41232432	1.80556848	3.78665267	4.00031796	3.11362767	4.21284072	3.59373638
ENSG00000199832	0	0	0	0	0	0	0	0	0	0	0	0	0	0	0	0	0	0	0	0	0	0	0	0	0	0	0	0	0	0	0	0	0	0	0	0	0	0	0	0	0	0	0	0	0	0	0	0
ENSG00000199833	0	0	0	0	0	0	0	0	0	0	0	0	0	0	0	0	0	0	0	0	0	0	0	0	0	0	0	0	0	0	0	0	0	0	0	0	0	0	0	0	0	0	0	0	0	0	0	0
ENSG00000199836	0	0	0	0	0	0	0	0	0.09775525	0.17908715	0	0	0.16735934	0	0	0	0.29745539	0	0	0	0	0	0.20029806	0.18383083	0	0	0	0	0	0.15905488	0	0	0.32437941	0	0.38508988	0	0.1829746	0	0	0	0	0	0	0	0	0	0.17607348	0.18485949
ENSG00000199837	0	0	0	0	0	0	0	0	0	0	0	0	0	0	0	0	0	0.30854681	0	0	0	0	0	0	0	0	0	0	0	0	0	0	0	0	0	0	0	0	0	0	0	0	0	0	0	0	0	0
ENSG00000199839	0	0	0	0	0	0	0	0	0	0	0	0	0	0	0	0	0	0	0	0	0	0	0	0	0	0	0	0	0	0	0	0	0	0	0	0	0	0	0	0	0	0	0	0	0	0	0	0
ENSG00000199840	0	0	0	0	0	0	0	0	0	0	0	0	0	0	0	0	0	0	0	0	0	0	0	0	0	0	0	0	0	0	0	0	0	0	0	0	0	0	0	0	0	0	0	0	0	0	0	0
ENSG00000199843	0	0	0.26940262	0	0	0	0	0	0	0	0	0	0	0	0	0	0	0	0	0	0	0	0	0	0	0	0	0	0	0	0	0	0	0	0	0	0	0	0	0	0	0	0	0	0	0	0	0
ENSG00000199845	0	0	0	0	0	0	0	0	0.13390005	0	0	0	0	0	0	0	0	0	0	0	0	0	0	0	0	0	0	0	0	0	0	0	0	0	0	0	0	0	0	0	0	0	0	0	0	0	0	0
ENSG00000199846	0	0	0	0	0	0	0.33833847	1.23801125	0.5207224	0	0	0.71158146	0.71319144	0.45641492	0.73281291	1.21926596	1.58448462	0	0.231826	0	0.17732502	0.53973976	0.64016831	0.19584592	0.2010918	0.52007985	1.08749521	0.31767308	0.30253815	0	0	0.25772753	0	0	0	0	0.58480116	1.69814855	0.42436391	0.33836886	0	0.75442094	1.20592503	0.17984538	0	0	0.3751631	0.78776725
ENSG00000199849	0	0	0	0	0	0	0	0	0	0	0	0	0	0	0	0	0	0	0	0	0	0	0	0	0	0	0	0	0	0	0	0	0	0	0	0	0	0	0	0	0	0	0	0	0	0	0	0
ENSG00000199851	0	0	0	0	0	0	0	0	0.07660628	0	0	0	0	0	0	0	0	0	0	0	0	0	0	0	0	0	0	0	0	0	0	0	0	0	0	0	0.14338875	0	0	0	0	0	0	0	0	0	0	0
ENSG00000199855	0	0.80702768	0.29458044	0	0.33604551	0.67784101	0.48379239	0	0.14891687	0.81844504	1.13842276	0.50874749	0	0	0.26196349	0.29057273	0.45313298	0	0.66297904	0.80236138	0	0	0.6102539	0	0.28754248	0.24788853	0.25916942	0	0.21630064	0	0	0	0	0.47406566	0	0	0	0.30352422	0.30340037	0.96767168	0.56269682	0.53937572	0.68974403	0	0	0	0	0.56321677
ENSG00000199856	0	0.20081852	0	0	0	0	0.12038555	0.29366778	0	0	0.18885463	0	0	0	0.13037253	0.14461061	0	0	0	0	0	0	0	0	0	0.12336778	0	0	0	0	0	0	0	0	0	0	0.13872028	0.15105624	0	0	0	0.2684335	0.17163398	0	0.13827738	0	0.13348827	0
ENSG00000199857	0	0	0	0	0	0	0	0	0	0	0	0	0	0	0	0	0	0	0	0	0	0	0	0	0	0	0	0	0	0	0	0	0	0	0	0	0	0	0	0	0	0	0	0	0	0	0	0
ENSG00000199858	0	0	0	0	0	0	0	0	0	0	0	0	0	0	0	0	0	0	0	0	0	0	0	0	0	0	0	0	0	0	0	0	0	0	0	0	0	0	0	0	0	0	0	0	0	0	0	0
ENSG00000199859	0	0	0	0	0	0	0	0	0	0	0	0	0	0	0	0	0	0	0	0	0	0	0	0	0	0	0	0	0	0	0	0	0	0	0	0	0	0	0	0	0	0	0	0	0	0	0	0
ENSG00000199862	0	0	0	0	0	0	0	0	0	0	0	0	0	0	0	0	0	0	0	0	0	0	0	0	0	0	0	0	0	0	0	0	0	0	0	0	0	0	0	0	0	0	0	0	0	0	0	0
ENSG00000199865	0.30121252	0	0	0	0	0.225947	0.24189619	0	0.14891687	0	0	0	0	0	0.26196349	0	0	0	0	0.26745379	0	0.25725914	0	0	0	0	0	0	0.22712141	0	0	0	0	0	0	0	0	0	0	0	0	0.53937572	0	0	0	0	0	0.28160839
ENSG00000199866	0	0	0	0.4084383	0	0	0	0	0	0	0	0	0	0	0	0	0	0	0	0	0	0	0	0	0	0.26003993	0	0	0.22690361	0	0	0	0.51837102	0	0	0	0	0.31840285	0	0	0	0	0	0	0	0	0	0
ENSG00000199867	0	0	0	0	0	0	0	0	0	0.2861883	0	0	0.26744679	0	0	0	0	0	0	0	0	0	0	0	0	0	0	0	0	0	0	0	0	0	0	0	0	0	0	0	0	0	0	0	0	0	0	0
ENSG00000199870	0	0	0	0	0	0	0	0	0	0	0	0	0	0	0	0	0	0	0	0	0	0	0	0	0	0	0	0	0	0	0	0	0	0	0	0	0	0	0	0	0	0	0	0	0	0	0	0
ENSG00000199872	0	0.80702768	0.58916087	0.3893524	0.16802276	0.90378801	0.24189619	0	0.14891687	0.81844504	0	0.50874749	0	0	0	0.29057273	0	0.36333549	0	0	0.25355821	0	0	0.28004136	0.28754248	0	0	1.13560703	0.43260127	0.24229855	0	0.36852628	0	0	0.58663225	0	0	0.30352422	0.30340037	0	0	0	0	0	0.25716208	0	0	0.84482516
ENSG00000199874	0	0.54423506	0	0	0.15107929	0.20316243	0	0	0	0.24530425	0	0	0	0	0	0	0	0	0	0	0	0	0	0	0	0.22289137	0	0	0	0	0	0	0	0	0	0	0	0	0.27280537	0	0	0	0	0	0	0	0	0
ENSG00000199875	0.28521894	0.57313249	0	0	0.15910119	0	0	0	0.42302935	0	0.35932518	0.24086717	0	0	0.49610785	0	0	0	0	0	0.72028483	0.48719872	1.15570208	0	0	0.23472631	0	0.21506186	0.20481565	0.45886629	0.43335028	0	0.93582025	0	0	0	0	0	0	0	0	1.0214726	0.32656023	0.24350746	0.52618824	0	0.2539821	0
ENSG00000199876	0	0	0	0	0	0	0	0	0	0	0	0	0	0	0	0	0	0	0	0	0	0	0	0	0	0	0	0	0	0	0	0	0	0	0	0	0	0	0	0	0	0	0	0	0	0	0	0
ENSG00000199878	0	0	0	0	0	0	0	0	0	0	0	0	0	0	0	0	0	0	0	0	0	0	0	0	0	0	0	0	0	0	0	0	0	0	0	0	0	0	0	0	0	0	0	0	0	0	0	0
ENSG00000199879	0	0	0	0	0	0	0	0.40215652	0	0	0	0	0	0	0	0	0	0	0	0	0	0	0	0	0	0	0	0	0	0	0	0	0.33677608	0	0	0.18260898	0	0.20686045	0	0	0.38349401	0	0	0	0	0	0	0
ENSG00000199880	0	0	0	0	0	0	0.51253253	0	0	0	0	0	0.27009478	0	0	0	0	0	0	0	0	0	0	0	0	0	0	0	0	0	0	0	0	0	0	0.28385753	0	0	0	0	0	0	0	0	0	0	0	0
ENSG00000199881	0	0	0	0	0	0	0	0	0	0	0.39042063	0	0	0	0	0	0	0	0	0	0	0	0	0	0	0	0	0	0	0	0	0	0	0	0	0	0	0.62455944	0	0	0	0.55493464	0	0	0	0	0	0
ENSG00000199883	4.81040892	5.34866632	4.89267327	4.9744122	2.84434942	4.47442514	2.78144518	3.76946708	2.23553121	2.87555166	4.24218234	1.9499456	2.19865211	2.18874798	3.93258032	1.67057634	2.89464056	4.29386632	1.2705449	4.27127701	2.51060475	4.60148287	1.07204305	2.05725903	2.75525775	3.32540611	3.47673842	2.53901392	3.45435345	2.55389907	4.6775959	2.23645948	4.261474	1.51417986	1.12423255	2.39626595	4.62953038	4.36259432	6.49276784	2.16354057	3.23508681	2.58417323	1.98275673	3.03911845	3.99353316	3.03927239	3.94089242	3.32802269
ENSG00000199884	0	0	0	0	0	0	0	0	0	0	0	0	0	0	0	0	0	0	0	0	0	0	0	0	0	0	0	0	0	0	0	0	0	0	0	0	0	0	0	0	0	0	0	0	0	0	0	0
ENSG00000199885	0	0	0	0	0	0	0	0	0	0	0	0	0	0	0	0	0	0	0	0	0	0	0	0	0	0	0	0	0	0	0	0	0	0	0	0	0	0	0	0	0	0	0	0	0	0	0	0
ENSG00000199886	0	0	0	0	0	0.23702284	0	0	0	0.2861883	0.79615187	0	0.26744679	0	0.54960968	0.91444947	0	0	0	1.1222571	0	0	0	0.29376887	0	0	0	0	0	0.50835187	0	0	0	0	0	0.28107461	0.58480116	0	0	0	0	0.56581571	0	0	0.58293403	0	0	0
ENSG00000199890	0	0	0	0	0	0	0	0	0	0	0	0	0	0	0	0	0	0	0	0	0	0	0	0	0	0	0	0	0	0	0	0	0	0	0	0	0	0	0	0	0	0	0	0	0	0	0	0
ENSG00000199892	0	0	0	0	0	0	0	0	0	0	0	0	0	0	0	0	0	0	0	0	0	0	0	0	0	0	0	0	0	0	0	0	0	0	0	0	0	0	0	0	0	0	0	0	0	0	0	0
ENSG00000199894	0	0	0	0	0	0	0	0	0	0	0	0	0	0	0	0	0	0	0	0	0	0	0	0	0	0	0	0	0	0	0	0	0	0	0	0	0	0	0	0	0	0	0	0	0	0	0	0
ENSG00000199895	0	0	0	0	0	0	0	0	0	0	0	0	0	0	0	0	0	0	0	0	0	0	0	0	0	0	0	0	0	0	0	0	0	0	0	0	0	0	0	0	0	0	0	0	0	0	0	0
ENSG00000199899	0.33226536	0	0	0.4294918	0	0	0	0	0.16426913	0	0	0	0	0	0.28897004	0.32052868	0	0	0	0	0	0	0	0.6178232	0	0.27344405	0	0	0	0.26727778	0	0	0	0	0	0	0	0	0	0	0	0	0	0.56734727	0.30649109	0	0.29587606	0.31064018
ENSG00000199900	0	0	0	0	0	0	0	0	0	0	0	0	0	0	0	0	0	0	0	0	0	0	0	0	0	0	0	0	0	0	0	0	0	0	0	0	0	0	0	0	0	0	0	0	0	0	0	0
ENSG00000199901	0	0	0	0	0	0	0	0	0	0	0	0	0	0	0	0	0	0	0	0	0	0	0	0	0	0	0	0	0	0	0	0	0	0	0	0	0	0	0	0	0	0	0	0	0	0	0	0
ENSG00000199903	0	0	0	0	0	0	0	0	0	0	0	0	0	0	0	0	0	0	0	0	0	0	0	0	0	0	0	0	0	0	0	0	0	0	0	0	0	0	0	0	0	0	0	0	0	0	0	0
ENSG00000199905	0	0	0	0	0	0	0	0	0	0	0	0	0	0	0	0	0	0	0	0	0	0	0	0	0	0	0	0	0	0	0	0	0	0	0	0	0	0	0	0	0	0	0	0	0	0	0	0
ENSG00000199906	0	0	0	0	0	0	0	0	0	0	0	0	0	0	0	0	0	0	0	0	0	0	0	0	0	0	0	0	0	0	0	0	0	0	0	0	0	0	0	0	0	0	0	0	0	0	0	0
ENSG00000199910	0	0	0	0	0	0	0	0	0	0	0	0	0	0	0	0	0	0	0	0	0	0	0	0	0	0	0	0	0	0	0	0	0	0	0	0	0	0	0	0	0	0	0	0	0	0	0	0
ENSG00000199911	0	0	0	0	0	0	0	0	0	0	0	0	0	0	0	0	0	0	0	0	0	0	0	0	0	0	0	0	0	0	0	0	0	0	0	0	0	0	0	0	0	0	0	0	0	0	0	0
ENSG00000199912	0	0	0	0	0	0	0	0	0	0	0.37947425	0	0	0	0	0	0	0	0	0	0	0	0	0	0	0	0	0	0	0	0	0	0	0	0	0	0	0	0	0	0	0	0	0	0	0	0	0
ENSG00000199913	0	0	0	0	0	0	0	0	0	0	0	0	0	0	0	0	0	0	0	0	0	0	0	0	0	0	0	0	0	0	0	0	0	0	0	0	0	0	0	0	0	0	0	0	0	0	0	0
ENSG00000199914	0	0	0.45028724	0	0.25683479	0.34537613	0	0	0	0	0	0	0	0	0	0	0	0.55538425	0	0	0.38758184	0.39323897	1.39922502	0	0.43952921	0.37891532	1.18847691	0.69434258	0	0	0	0	0	0.72464322	0	0	0	0	0	0	0	0	1.05432302	0.39309061	0	0	0	0.43045853
ENSG00000199920	0	0	0	0	0	0	0	0	0	0	0	0	0	0	0	0	0	0	0	0	0	0	0	0	0	0	0	0	0	0	0	0	0	0	0	0	0	0	0	0	0	0	0	0	0	0	0	0
ENSG00000199921	0	0	0	0	0	0	0	0	0	0	0	0	0	0	0	0	0	0	0	0	0	0	0	0	0	0	0	0	0	0	0	0	0	0	0	0	0	0	0	0	0	0	0	0	0	0	0	0
ENSG00000199924	0	0	0	0	0	0	0	0	0	0	0	0	0	0	0	0	0	0	0	0	0	0	0	0	0	0	0	0	0	0	0	0	0	0	0	0	0	0	0	0	0	0	0	0	0	0	0	0
ENSG00000199927	0	0	0	0	0	0	0	0	0	0	0	0	0	0	0	0	0	0	0	0	0	0	0	0	0	0	0	0	0	0	0	0	0	0	0	0	0	0	0	0	0	0	0	0	0	0	0	0
ENSG00000199929	0	0	0	0	0	0	0	0	0	0	0	0	0	0	0	0	0	0	0	0	0	0	0	0	0	0	0	0	0	0	0	0	0	0	0	0	0	0	0	0	0	0	0	0	0	0	0	0
ENSG00000199932	0	0	0	0	0	0	0	0	0	0	0	0	0	0	0.17091521	0	0	0	0	0	0.16543127	0.1678459	0	0	0.18760393	0	0	0.29636574	0.14112298	0.15808503	1.49294458	0.72132278	0	0.30929894	0.38274177	0.1748147	0	0	0	0	0	0	0	0	0	0	0	0
ENSG00000199933	0.29035802	0.77794561	0.28396492	0.3753216	0.16196788	0.21780477	0.69953764	0.28440799	0.7177525	0.26298384	0.36579951	0.24520712	0.24576191	0.94366869	0	0.84030492	0.87360774	0.70048464	0.31954395	0.77344746	0.48884196	0	1.17652554	0.26994978	0.27718058	0	0	0	0	0.70070122	0.4411584	0.35524605	0.47634094	0.45698221	0	0.51656956	0	0.58517281	0.58493404	0	0	0	0.3324442	0.24789498	0.80350367	0	0	0.54292067
ENSG00000199934	0	0	0	0	0	0	0	0	0	0	0.52732137	0	0	0	0	0	0	0	0	0	0	0	0	0	0	0	0	0	0	0	0	0	0	0	0	0	0	0	0	0	0.78192935	0	0	0.3573551	0	0	0	0
ENSG00000199936	0	0	0	0	0	0	0	0	0	0	0	0	0	0	0	0	0	0	0	0	0	0	0	0	0	0	0	0	0	0	0	0	0	0	0	0	0	0	0	0	0	0	0	0	0	0	0	0
ENSG00000199938	0	0	0	0	0	0	0	0	0	0	0	0	0	0	0	0	0	0	0	0	0	0	0	0	0	0	0	0	0	0	0	0	0	0	0	0	0	0	0	0	0	0	0	0	0	0	0	0
ENSG00000199940	0	0	0	0	0	0	0	0	0	0	0	0	0	0	0.09469626	0	0	0	0	0	0	0	0	0	0	0	0	0	0	0	0	0.13321727	0	0	0	0	0	0	0	0	0	0	0	0	0	0	0	0
ENSG00000199942	0	0.28783987	0	0	0	0	0	0	0	0	0	0	0.36372763	0	0.37373458	0	0	0.51835864	0	0.38156741	0	0	0.43531445	0.79905133	0	0	0.36974837	0.64805308	0.61717782	0.34567927	0	0	0	0	0	0	0	0	0	0	0	0	0	0.36688457	0.39639514	0	0	0
ENSG00000199944	0	0	0.2973595	0	0	0.22807858	0	0	0.15032175	0	0	0.2567735	0	0	0	0	0	0.36676319	0	0	0	0.25968611	0	0	0	0	0	0.45852812	0	0	0	0	0	0.47853798	0	0	0	0	0	0	0	0	0	0	0	0	0	0
ENSG00000199949	0	0	0	0	0	0	0	0	0	0	0	0	0	0	0	0	0	0	0	0	0	0	0	0	0	0	0	0	0	0	0	0	0	0	0	0	0	0	0	0	0	0	0	0	0	0	0	0
ENSG00000199953	0	0	0	0	0	0	0	0	0	0	0	0	0	0	0	0	0	0	0	0	0	0	0	0	0	0	0	0	0	0	0	0	0	0	0	0	0	0	0	0	0	0	0	0	0	0	0	0
ENSG00000199959	0	0	0	0	0	0	0	0	0	0	0	0	0	0	0	0	0	0	0	0	0	0	0	0	0	0	0	0	0	0	0	0	0	0	0	0	0	0	0	0	0	0	0	0	0	0	0	0
ENSG00000199960	0	0	0	0	0	0	0	0	0	0	0	0	0	0	0	0	0	0	0	0	0	0	0	0	0	0	0	0	0	0	0.6045504	0	0	0	0	0	0	0	0	0	0	0	0	0	0	0	0.35432071	0
ENSG00000199961	0	0	0.75047873	0.4959607	0	0	1.84877805	2.6307739	1.32784212	0.69502872	2.4168896	1.62011849	0.32475682	0	1.00107478	2.22080585	1.15441022	1.38846063	0	1.02205557	1.61492433	1.31079657	1.94336808	1.78359673	3.2964691	3.47339045	3.96158969	1.44654704	1.65315486	1.54321102	1.74887793	1.40829685	0.62945053	1.2077387	2.98903098	2.04782932	2.48540493	1.54652815	4.63769131	1.2326294	3.58384286	1.37412386	1.31790378	0.65515102	1.0617727	1.58098796	0.6833328	2.86972355
ENSG00000199962	0	0.19805496	0.28917529	0	0.65975908	0	0	0.28962648	0	0	0.74502285	0.24970634	0	0	0	0	0	0	0	0	0.24890577	0.25253879	0	0	0	0	0	0	0	0.47570542	0.89850609	0	0	0	0	0.26302395	0	0	0	0	0	0	0	0	0	0	0	0
ENSG00000199963	0.29842352	0	0.29185284	0	0	0	0	0	0.14753801	0	0	0.50403686	0.25258863	0	0	0	0.44893731	0	0.32842017	0	0	0	0	0	0	0	0	0	0	0	0	0	0	0.46967616	0	0	0	0	0	0	0	0	0	0.25478095	0	0	0	0
ENSG00000199964	0	0	0	0	0	0	0	0	0	0	0	0	0	0	0	0	0	0	0	0	0	0	0	0	0	0	0	0	0	0	0	0	0	0	0	0	0	0	0	0	0	0	0	0	0	0	0	0
ENSG00000199968	0	0	0	0	0	0	0	0	0	0	0	0	0	0	0	0	0	0	0	0	0	0	0	0	0	0	0	0	0	0	0	0	0	0	0	0	0	0	0	0	0	0	0	0	0	0	0	0
ENSG00000199970	0	0	0	0	0	0	0	0	0	0	0	0	0	0	0	0	0	0	0	0	0	0	0	0	0	0	0	0	0	0	0	0	0	0	0	0	0	0	0	0	0	0	0	0	0	0	0	0
ENSG00000199971	0	0	0	0	0	0	0	0	0	0	0	0	0	0	0	0	0	0	0	0	0	0	0	0	0	0	0	0	0	0	0	0	0	0	0	0	0	0	0	0	0	0	0	0	0	0	0	0
ENSG00000199975	2.39897739	3.21374617	3.06020453	4.1795527	2.09457495	3.52082467	1.08892429	2.6563154	2.21736743	1.60598869	4.07351489	1.67359813	1.05940087	3.16388596	2.17709466	1.00619035	2.03983165	3.27119528	1.95138976	3.14885729	2.19504278	2.04891502	0.84527078	1.35761149	2.68838256	3.51937532	2.33336351	2.28076932	2.39680705	2.68488752	5.2296544	1.02090128	2.22446593	1.47742793	1.01569014	2.31954778	3.86082319	5.04498502	6.30365809	2.68066977	3.50729476	3.54870822	1.07479532	2.49339028	2.8863724	3.86804822	3.71520743	2.24284219
ENSG00000199977	0.47165473	0.21061454	0.46126985	0.2032229	0.35079873	0.23586663	0.25251603	0	0	0.14239613	0.19806705	0.26554137	0	0.51096207	0	0.15166479	0.23651331	0.1896434	0	0	0.26469004	0	0	0	0.45024944	0.25877144	0	0	0.5644919	0	0	0	0	0.4948783	0	0	0.29097424	0	0	0	1.17480117	0.56305563	0	0	0	0.21593982	0.41999967	0.14698584
ENSG00000199979	0	0	0	0	0	0	0	0	0	0	0	0	0	0	0	0	0	0	0	0	0	0	0	0	0	0	0	0	0	0	0	0	0	0	0	0	0	0	0	0	0	0	0	0	0	0	0	0
ENSG00000199980	0	0	0	0	0	0	0	0	0	0	0	0	0	0	0	0	0	0	0	0	0.27130729	0	0	0	0.30767045	0	0	0	0	0	0	0	0	0	0	0	0	0	0	0	0	0	0	0	0.29729636	0	0	0
ENSG00000199985	0	0	0	0	0	0	0	0	0	0	0	0	0	0	0	0	0	0	0	0	0	0	0	0	0	0	0	0	0	0	0	0	0	0	0	0	0	0	0	0	0	0	0	0	0	0	0	0
ENSG00000199986	0	0	0	0	0	0	0	0	0	0	0	0	0	0	0	0	0	0	0	0	0	0	0	0	0	0	0	0	0	0	0	0	0	0	0	0	0	0	0	0	0	0	0	0	0	0	0	0
ENSG00000199990	0.97665878	0	0	0	0.54480106	0.24420535	0	0	0	0.29486067	0	0.2749292	0.27555124	0	0	0	0.48974979	0.39269594	1.07482965	0	1.37023883	0.83414327	0.32978367	0	0.93233469	0.53583985	1.12044961	0.73642395	1.16889738	0.26187823	0.49463214	0	0.53407923	2.56186997	0	0.86877608	0.60252241	0.65610285	0.65583513	0	0	0	0.37274046	0.55588571	1.8017961	0.89429622	0.57979752	0.30436462
ENSG00000199994	0	0	0.25216085	0	0.14382748	0.19341064	0	0	0	0	0.32482996	0	0	0	0	0	0	0	0	0	0	0	0	0	0	0	0	0	0	0	0	0	0	0	0	0	0	0	0	0	0	0	0	0	0.3541413	0	0	0
ENSG00000200003	0.31597784	0.42329393	0.92706196	0	0	0.23702284	0.5075077	0	0.15621672	0	0	0	0	0.68462238	0.54960968	0.30481649	0	0	0.34773901	0.56112855	0.26598754	0.26986988	1.28033662	0	0	0.26003993	0.2718738	0.23825481	0	0	0	0	0	0.49730417	0	0	0	0	0.31827293	0	0.59028	0.56581571	0	0	0	0	0	0.29541272
ENSG00000200008	0	0	0	0	0	0	0	0	0	0	0	0	0	0	0.26695327	0	0	0	0	0	0	0	0	0	0	0	0	0	0	0	0	0	0	0	0	0	0	0	0	0	0	0	0	0	0	0	0	0
ENSG00000200011	0	0	0	0	0	0	0	0	0	0	0.39807593	0	0	0	0	0	0	0	0	0	0	0	0	0	0	0	0	0	0	0	0	0	0	0	0	0	0	0	0	0	0	0	0	0	0.86799339	0	0	0
ENSG00000200013	0	0	0	0	0	0	0	0	0	0	0	0	0	0	0	0	0	0	0	0	0	0	0	0	0	0	0	0	0	0	0	0	0	0	0	0	0	0	0	0	0	0	0	0	0	0	0	0
ENSG00000200021	0	0	0	0	0	0	0	0	0	0	0	0	0.49152383	0	0	0	0	0	0	0.51563164	0	0	0	0	0	0	0	0	0	0	0	0	0	0	0	0	0	0	0	0	0	0	0	0	0	0	0	0
ENSG00000200024	0	0	0	0	0	0	0	0	0	0	0	0	0	0	0	0	0	0	0	0	0	0	0	0	0	0	0	0	0	0	0	0	0	0	0	0	0	0	0	0	0	0	0	0	0	0	0	0
ENSG00000200026	0	0	0	0	0	0	0	0	0	0	0	0	0	0	0	0	0	0	0	0	0	0	0	0	0	0	0	0	0	0	0	0	0	0	0	0	0	0	0	0	0	0	0	0	0	0	0	0
ENSG00000200028	0	0	0	0	0	0	0	0	0	0	0	0	0	0	0	0	0	0	0	0	0	0	0	0	0	0	0	0	0	0	0	0	0	0	0	0	0	0	0	0	0	0	0	0	0	0	0	0
ENSG00000200029	0	0	0	0	0	0	0	0	0	0	0	0	0	0	0	0	0	0	0	0	0	0	0	0	0	0	0	0	0	0	0	0	0	0	0	0	0	0	0	0	0	0	0	0	0	0	0	0
ENSG00000200033	0	0	0	0	0	0	0	0	0	0	0	0	1.31151791	0	0.8085604	0	0	0	0	0.27516881	0	0	0	0	0	0	0.26664546	0	0	0	0	0	0	0	0	0	0	0	0	0	0	0	0.35482025	0.26458022	0.57172376	0	0	0.57946341
ENSG00000200034	0	0	0	0	0	0	0	0	0	0	0	0	0	0	0	0	0	0	0	0	0	0	0	0	0	0	0	0	0	0	0	0	0	0	0	0	0	0	0	0	0	0	0	0	0	0	0	0
ENSG00000200036	0	0.18141169	0	0	0	0	0	0	0	0	0	0	0	0	0	0	0	0	0	0	0	0	0	0	0	0	0	0	0	0	0	0	0	0	0	0	0	0	0	0	0	0	0	0	0	0	0	0
ENSG00000200037	0	0	0	0	0	0	0	0	0	0	0	0	0	0	0	0	0	0	0	0	0	0	0	0	0	0	0	0	0	0	0	0	0	0	0	0	0	0	0	0	0	0	0	0	0	0	0	0
ENSG00000200040	0	0	0	0	0	0	0.22905215	0	0	0	0	0	0	0	0	0	0	0.34404334	0	0	0	0	0	0	0	0	0	0	0	0	0	0	0.46791012	0	0	0	0	0	0	0	0	0	0	0	0	0	0	0
ENSG00000200041	0	0	0	0	0	0	0	0	0	0	0	0	0	0	0	0	0	0	0	0	0	0	0	0	0	0	0	0	0	0	0	0	0	0	0	0	0	0	0	0	0	0	0	0	0	0	0	0

ENSG00000200042	0	0	0	0	0	0	0	0	0	0	0	0	0	0	0	0	0	[illegible]	0	0	0	0	0	0	0	0	0	0	0	0	0	0	0	0	0	0	0	0	0	0	0	0	0	0	0
ENSG00000200047	0	0.34050458	0.39773005	0.1314218	0.05671431	0	0	0.09958766	0	0	0	0	0.08605543	0	0	0.09807975	0	[illegible]	0	0	0.094525	0	0.08367215	0	0	0	0	0	0	0	0.16001585	0	0	0	0	0	0	0	0	0.23281581	0.08680234	0	0	0	0
ENSG00000200048	0	0	0	0	0	0	0	0	0	0	0	0	0	0	0	0	0	[illegible]	0	0	0	0	0	0	0	0	0	0	0	0	0	0	0	0	0	0	0	0	0	0	0	0	0	0	0
ENSG00000200049	0	0	0	0	0	0	0	0	0	0	0	0	0	0	0	0	0	[illegible]	0	0	0	0	0	0	0	0	0	0	0	0	0	0	0	0	0	0	0	0	0	0	0	0	0	0	0
ENSG00000200051	0	0	0	0	0.23348617	0	0	0	0	0	0.52732137	0	0	0	0	0	0	[illegible]	0	0	0	0	0	0	0	0	0	0	0	0	0	0	0	0	0	0	0	0	0	0	0	0	0	0	0
ENSG00000200052	0	0	0	0	0	0	0	0	0	0	0	0	0	0	0	0	0	[illegible]	0	0	0	0	0	0	0	0	0	0	0	0	0	0	0	0	0	0	0	0	0	0	0	0	0	0	0
ENSG00000200057	0	0	0	0	0	0	0	0	0	0	0	0	0	0	0	0	0	[illegible]	0	0	0	0	0	0	0	0	0	0	0	0	0	0	0	0	0	0	0	0	0	0	0	0	0	0	0
ENSG00000200058	0.59137137	0	0	0	0	0	0	0	0	0	0	0	0	0	0	0	0	[illegible]	0	0	0	0	0	0	0	0	0	0	0	0	0	0	0	0	0	0	0	0	0	0	0	0	0	0	0
ENSG00000200059	0	0	0	0	0	0	0	0	0	0	0	0	0	0	0	0.31091282	0	[illegible]	0	0	0	0	0	0	0	0	0	0	0	0	0	0	0	0	0	0	0	0	0	0	0	0	0	0	0.30132097
ENSG00000200060	0	0	0	0	0	0	0	0	0	0	0	0	0	0	0	0	0	[illegible]	0	0	0	0	0	0	0	0	0	0	0	0	0	0	0	0	0	0	0	0	0	0	0	0	0	0	0
ENSG00000200062	0	0	0	0	0	0	0	0	0	0	0	0	0	0	0	0	0	[illegible]	0	0	0	0	0	0	0	0	0	0	0	0	0	0	0	0	0	0	0	0	0	0.26171139	0	0	0	0	0
ENSG00000200063	0.74952883	0	0.24434191	0	0.13936771	0.56224022	0	0.48944631	0.49408079	0.22628842	0	0.21099218	0	0.27066466	0.4345751	0.24101769	0	[illegible]	[illegible]	0.50617959	0.69684709	0.71551267	0.20561297	0.85987993	0.37677504	0.53823647	0.60292896	0	0	0	0	1.45975932	0	0.46240092	0	0.50331534	0.4013212	0.93346605	0.44738916	0.28605663	0	0.46092458	1.02948054	0.22248045	0.70074645
ENSG00000200064	0	0.2225566	0	0.4294918	0	0.24924051	0	0	0	0	0	0	0	0	0	0	0	[illegible]	0	0	0	0	0	0	0	0	0.26727778	0	0	0	0	0	0	0	0	0	0	0	0	0	0	0	0	0	0
ENSG00000200065	0	0	0	0	0	0	0	0	0	0	0	0	0	0	0	0	0	[illegible]	0	0	0	0	0	0	0	0	0	0	0	0	0	0	0	0	0	0	0	0	0	0	0	0	0	0	0
ENSG00000200066	0	0	0	0	0	0	0	0	0	0	0	0	0	0	0	0	0	[illegible]	0	0	0	0	0	0	0.23594165	0	0	0	0	0	0	0	0	0	0	0	0	0	0	0	0	0	0	0	0
ENSG00000200070	0	0	0	0	0	0.17146333	0	0.89558261	0	0	0	0	0	0	0	0	0.34386687	[illegible]	[illegible]	0.23155024	0.21251365	0	0.18811399	0.19667467	0	0.16414304	0	0.34729491	0.27966179	0	0	0	0.20333057	0.21152382	0	0	0.36716621	0	0	0.26171139	0	0.21084848	0.94186517	0	0
ENSG00000200072	0	0	0	0	0	0	0	0	0	0	0	0	0	0	0	0	0	[illegible]	0	0	0	0	0	0	0	0	0	0	0	0	0	0	0	0	0	0	0	0	0	0	0	0	0	0	0
ENSG00000200075	0	0	0	0	0	0	0.2038023	0.49715412	0	0.22985202	0	0	0	0.8247813	0	0	0	[illegible]	[illegible]	0	0.23594035	0.2422602	0.20885096	0	0.19135425	0.36447509	0	0	0.31049065	0	0.39940965	0	0	0.23484141	0	0.25562078	0	0	0	0	0	0	0	0	0
ENSG00000200079	0	0	0	0	0	0	0	0	0	0	0	0	0	0	0	0	0	[illegible]	0	0	0	0	0	0	0	0	0	0	0	0	0	0	0	0	0	0	0	0	0	0	0	0	0	0	0
ENSG00000200084	0	0	0	0	0	0	0	0	0	0	0	0	0	0	0	0	0	[illegible]	0	0	0	0	0	0	0	0	0	0	0	0	0	0	0	0	0	0	0	0	0	0	0	0	0	0	0
ENSG00000200085	0	0	0	0	0	0	0	0	0	0	0	0	0	0	0	0	0	[illegible]	0	0	0	0	0	0	0	0	0	0	0	0	0	0	0	0	0	0	0	0	0.56581571	0	0	0	0	0	0
ENSG00000200086	0.30990134	3.11365249	1.81846769	0.4005837	1.21008697	1.62725294	0.49774794	0.30355083	0	0.28068468	0.39042063	1.04684579	0.26230358	0	0	0	0	[illegible]	[illegible]	0.31392869	0.57623894	0.59167394	0.25503916	1.06658184	1.16836492	0.44508016	0	0.9417035	0.37915685	1.52520704	0.48774063	0	0.27566933	0.57355498	0	0.9364569	0.49779264	0	0	0.35482025	0.52916044	0	0.42565061	0.82788396	0
ENSG00000200087	191.324583	127.623121	165.78958	286.111	239.976856	185.233347	1081.75266	1707.52701	1260.20028	1185.9643	5024.51443	2069.2344	410.798268	1614.16842	1014.30467	387.26935	979.449167	[illegible]	[illegible]	369.85724	674.199563	920.145789	905.849085	935.925565	1696.37423	1228.11578	1259.69592	1581.39715	1145.47	988.015166	962.034919	1042.46851	1621.80052	2614.79219	3136.26811	2958.82432	3742.44414	3575.03082	3560.39533	1622.21035	1456.3429	1746.47037	2816.85556	1691.89181	1043.84084
ENSG00000200089	0	0	0.42026809	0	0	0.64470212	0	0	0	0	0	0	0	0	0	0	0	[illegible]	[illegible]	0.43531445	0	0	0	1.10924511	0.32402654	0.61717782	1.38271707	0	0	0	0	0	0	0	0	0	0	0	0	0.49201741	0	0.79279029	0	0	0.4017613
ENSG00000200090	2.27887049	4.1431497	1.59192458	3.7873366	2.7240053	2.93046417	8.62763088	5.42098865	4.18471457	7.6663774	3.28111072	7.69801756	3.85771732	5.29026386	4.24698391	3.14053353	6.85649707	[illegible]	[illegible]	7.5850245	3.32938056	0.62155646	3.48295902	2.52101162	3.68211975	4.67558952	6.28507761	2.96779285	3.58475563	4.8067131	8.19798391	8.24247937	3.76469633	2.41008963	2.95246282	3.93501081	4.70640318	4.25717091	5.24665474	2.9819237	5.28091426	5.70568766	4.91862922	3.18888638	14.0007725
ENSG00000200091	0.09766588	0	0.09551547	0	0	0.0732616	0	0	0	0	0	0	0	0.10580528	0	0	0	[illegible]	[illegible]	0.0989351	0.09080129	0.09323347	0.24112793	0.08403372	0.36821197	0.07013384	0.07856347	0	0	0.32044754	0.1537122	0.19021106	0.08687761	0	0.09841543	0	0	0	0.17488849	0.11182214	0.16676571	0	0.13414443	0.17393926	0

[illegible]

ENSG00000200411	0	0	0	0	0	0	0	0	0	0	0	0
ENSG00000200413	0.44763527	0.89949961	1.31333778	0	0.24970049	0.33578235	0	0	0	0	0	0
ENSG00000200418	0	0	0	0	0	0	0	0	0	0	0	0
ENSG00000200419	0	0	0	0	0	0	0	0.30950281	0.31243344	0.85856489	0	0
ENSG00000200421	0	0	0	0	0	0	0	0	0	0	0	0
ENSG00000200422	0	0	0	0	0	0	0	0	0	0	0	0
ENSG00000200424	0	0	0	0	0	0	0	0	0	0	0	0
ENSG00000200427	0	0	0	0	0	0	0	0	0	0	0	0
ENSG00000200428	0.27546786	0	0.26940262	0	0	1.03317647	0.88488522	0	0.13618894	0.24949749	0.34704056	0
ENSG00000200431	0	0.1499166	0	0	0	0	0	0	0	0	0	0
ENSG00000200432	0	0	0	0	0	0	0	0	0	0	0	0
ENSG00000200434	0.63611328	3.6926826	2.48842947	3.2890028	2.1290252	1.74960278	0	0.41538535	2.41108175	1.9204741	29.9185491	3.58131456
ENSG00000200436	0	0	0	0	0	0	0	0	0	0	0	0
ENSG00000200437	0	0	0	0	0	0	0	0	0	0	0	0
ENSG00000200443	0	0	0	0	0	0	0	0	0	0	0	0
ENSG00000200444	0	0	0	0	0	0	0	0	0	0	0	0
ENSG00000200446	0	0	0	0	0	0	0	0	0	0	0	0
ENSG00000200448	0	0	0	0	0	0	0.25882893	0	0	0	0	0
ENSG00000200455	0	0.21806051	0	0	0	0.24420535	0	0	0	0	0.41013884	0
ENSG00000200456	0	0	0	0	0	0	0	0	0	0	0	0
ENSG00000200462	0	0	0	0	0	0	0	0	0	0	0	0
ENSG00000200463	0	0	0	0	0	0	0	0	0	0	0	0
ENSG00000200468	0	0	0	0	0	0	0	0	0	0	0	0
ENSG00000200469	0	0	0	0	0	0	0	0	0	0	0	0
ENSG00000200471	0	0	0	0	0	0	0	0	0	0	0	0
ENSG00000200472	0.10199285	0	0	0	0	0	0	0	0.05042438	0	0	0
ENSG00000200473	0	0	0	0	0	0	0	0	0	0	0	0
ENSG00000200475	0	0	0	0.1207557	0	0	0	0	0	0	0	0
ENSG00000200478	0	0	0	0	0	0	0	0	0	0	0	0

[illegible]

ENSG00000200806	0	0	0	0	0	0	0	0	0	0	0	0	0	0	0	0	0	0	0	0	0	0	0	0
ENSG00000200807	0	0	0	0	0	0	0	0	0	0	0	0	0	0	0	0	0	0	0	0	0	0	0	0
ENSG00000200812	0	0	0	0	0	0	0	0	0	0	0	0	0	0	0	0	0	0	0	0	0	0	0	0
ENSG00000200814	0	0	0	0	0	0	0	0	0	0	0	0	0	0	0	0	0	0	0	0	0	0	0	0
ENSG00000200815	0	0	0	0	0	0	0	0	0	0	0	0	0	0	0	0	0	0	0	0	0	0	0	0
ENSG00000200816	4.63912921	3.43445304	4.05940767	15.464958	4.49460875	4.21254224	7.8433008	11.4797407	6.39778477	5.08634654	35.9896833	8.45407286	1.23998057	9.25796176	4.24698391	1.6487801	4.04043578	9.42470248	13.4353707	3.68559433	4.72732395	11.46947	5.44143062	10.8961546
ENSG00000200817	0	0	0	0	0	0	0	0	0	0	0	0	0	0	0	0	0	0	0	0	0	0	0	0
ENSG00000200818	0	0	0	0	0	0	0	0	0	0	0	0	0	0	0	0	0	0	0	0	0	0	0	0
ENSG00000200822	0	0	0	0	0	0	0	0	0	0	0	0	0	0	0	0	0	0	0	0	0	0	0	0
ENSG00000200823	0	0.55353822	0	0.5341116	0	0.61990588	0	0	0	0	0	0	0.69947622	0	1.43744071	0.79721236	1.86481651	1.99368706	4.54735622	5.13648441	1.04348957	4.58778798	6.69714538	1.53663718
ENSG00000200827	0	0	0	0	0	0	0	0	0	0	0	0	0	0	0	0	0	0	0	0	0	0	0	0
ENSG00000200829	0	0	0	0	0	0	0	0	0.1422688	0	0	0	0	0	0	0	0	0	0	0	0	0	0	0
ENSG00000200830	0	0	0	0	0	0	0	0	0	0.1085175	0.15094329	0	0	0	0	0	0	0	0	0	0	0	0	0
ENSG00000200831	0	0	0	0	0	0	0	0	0	0	0	0	0	0	0	0	0	0	0	0	0	0	0	0
ENSG00000200832	0	0.86351962	0	0	0	0.32235106	0	0	0	0	0	0	0.36372763	0.93108644	0.74746917	0	0.64646972	0.51835864	0.94585009	1.14470224	2.1704583	0.73404608	1.30594335	0.39952567
ENSG00000200833	0	0	0	0	0	0	0	0.28699352	0	0	0	0	0	0	0	0	0	0	0	0	0	0	0.29680531	0
ENSG00000200834	0	0	0	0.4124822	0.35600861	0.2393696	0.25626626	0.3125672	0	0.57804369	0.80403456	0.26948506	0.27009478	0	0.27752568	0	0	0	0	0.56668428	0	0.27254186	0	0.29667748
ENSG00000200839	0	0	0	0	0	0	0	0	0	0	0	0	0	0	0	0	0	0	0	0	0	0	0	0
ENSG00000200840	0.90363756	0.60527076	0	0.3893524	0.33604551	0	0.24189619	0	0.29783375	0.27281501	0	0.50874749	0.25494928	1.30526136	0.26196349	0.29057273	0.90626597	1.45334197	0	0.26745379	1.01423285	0	0	0
ENSG00000200842	0	0	0	0	0	0	0	0	0	0	0	0	0	0	0	0	0	0	0	0	0	0	0	0
ENSG00000200843	0	0	0	0	0	0	0	0	0	0	0	0	0	0	0	0	0	0	0	0	0	0	0	0
ENSG00000200847	0	0.84658787	0.30902065	0	0	1.42213702	0	0	0	0.57237659	0	0	0.26744679	1.02693357	0.27480484	0.30481649	0	0	0	0	0	0	0	0
ENSG00000200849	0	0	0	0	0	0	0	0	0	0	0	0	0	0	0	0	0	0	0	0	0	0	0	0
ENSG00000200852	0	0	0	0	0	0	0	0	0	0	0	0	0	0	0	0	0	0	0	0	0	0	0	0
ENSG00000200855	0	0.42329393	0	0	0	0.23702284	0.25375385	0	0.31243344	0.57237659	0	0.26684305	0	0.68462238	0	0	0	0	0.69547801	0	0	0.26986988	0	0.29376887
ENSG00000200857	0	0	0	0	0	0	0	0	0	0	0	0	0	0	0	0	0	0	0	0	0	0	0	0
ENSG00000200860	0	0	0	0	0	0	0	0	0	0	0	0	0	0	0	0	0	0	0	0	0	0	0	0
ENSG00000200867	0.51650224	0.27676911	0.20205197	0.4005837	0.23049276	0.77488235	0.33183196	0.20236722	0.25535425	0.18712312	0	0.61066005	0	0.11190943	0.26952013	0.09965154	0.46620413	0.37381632	0.11368391	0	0.34782986	0.17645338	0.41857159	0.48019912
ENSG00000200869	0	0	0	0	0	0	0	0	0	0	0	0	0	0	0	0	0	0	0	0	0	0	0	0
ENSG00000200871	0	0	0.29458044	0	0	0	0	0	0	0	0	0	0	0	0	0	0	0.36333549	0	0	0	0	0	0
ENSG00000200872	0	0	0	0	0	0	0	0	0	0	0	0	0	0	0	0	0	0	0	0	0	0	0	0
ENSG00000200873	0	0	0	0	0	0	0	0	0	0	0	0	0	0	0	0	0	0	0	0	0	0	0	0
ENSG00000200874	0	0	0	0	0	0.47404567	0.25375385	0	0	0.57237659	0	0.26684305	0.26744679	0	0.27480484	0.30481649	0	0	0	0	0	0	0	0
ENSG00000200875	0	0	0	0	0	0	0	0	0	0	0	0	0	0	0	0	0	0	0	0	0	0.25725914	0	0.28004136
ENSG00000200877	0	0	0	0	0	0	0	0	0	0	0	0	0	0	0	0	0	0	0	0	0	0	0	0
ENSG00000200879	1.51669364	0.50795272	1.11247435	1.4703777	2.11511	1.70656443	8.22162472	7.05666412	8.99808308	6.52509316	6.68767568	7.04465641	2.56748917	1.23232029	4.94648714	5.48669681	3.42248678	2.28687634	3.33829445	3.03009416	2.23429531	3.56228243	5.76151477	6.34540765
ENSG00000200882	0.92970403	0.83030733	0	0.4005837	0.17286957	0.23246471	0	0.30355083	0	0	0.39042063	0.26171145	0.26230358	0.33572828	0.26952013	0	0	0.37381632	0.34105172	0.27516881	0.26087239	0	0	0.28811947
ENSG00000200883	0	0	0	0	0	0	0	0	0	0	0	0	0	0	0	0	0	0	0	0	0	0	0	0
ENSG00000200884	0	0	0	0	0	0	0	0	0	0	0	0	0	0	0	0	0	0	0	0	0	0	0	0
ENSG00000200885	0	0	0	0	0	0	0	0	0	0	0	0	0	0	0	0	0	0	0	0	0	0	0	0
ENSG00000200887	0	0	0	0	0	0	0	0	0	0.27801149	0	0	0	0.33253087	0	0	0	0	0.3378036	0	0.25838789	0	0	0
ENSG00000200888	0	0.42329393	0.61804131	0	0.17625917	0	0	0.92850844	0.7810836	0.2861883	0	1.06737218	0.26744679	0.34231119	0.27480484	0.60963298	1.90138154	0.76229211	0.34773901	0.28056427	0.79796261	0.26986988	1.28033662	0.29376887
ENSG00000200889	0	0	0	0	0	0	0	0	0	0	0	0	0	0	0	0	0	0	0	0	0	0	0	0
ENSG00000200890	0	0	0	0	0	0	0	0	0	0	0	0	0	0	0	0	0	0	0	0	0	0	0	0
ENSG00000200891	0	0	0	0	0	0	0	0	0	0	0	0	0	0	0	0	0	0	0	0	0	0	0	0
ENSG00000200893	0	0	0	0	0	0	0	0	0.14891687	0.27281501	0	0	0	0	0	0	0	0.36333549	0	0	0	0	0	0
ENSG00000200895	0	0	0.09850033	0	0	0.07555103	0.08088404	0	0.14938224	0	0.38066011	0.08505622	0.08524866	0	0	0	0.45454902	0	0.11084181	0.08942986	0	0.17204205	0.20405365	0.18727766
ENSG00000200897	0	0	0	0	0	0	0	0	0	0	0	0	0	0	0	0	0	0	0	0	0	0	0	0
ENSG00000200898	0	0	0	0	0	0	0.24189619	0	0	0	0	0	0	0	0	0	0	0	0	0	0	0	0	0
ENSG00000200902	0	0	0	0	0	0	0	0	0	0	0	0	0	0	0	0	0	0	0	0	0	0	0	0
ENSG00000200903	0	0	0	0.2603794	0.22473044	0	0.16176808	0	0.09958816	0	0.76132022	0	0	0.21822338	0.17518809	0	0	0.24298061	1.77346893	0	0.50870116	0	0.20405365	0

ENSG00000201218	0	0	0	0	0	0	0	0	0	0	0	0	0	0	0	0	0	0	0	0	0	0.25253879	0	0
ENSG00000201221	0.2253828	0	0	0	0.12572332	0.50719572	0.18099925	0	0	0	0.56788455	0.1903356	0.19076624	0.24416602	0.19601464	0.65226466	0	0	0	0.20012277	0.37945075	0.1924946	0	0.20954143
ENSG00000201223	0.30990134	0.41515366	0.6061559	0.8011674	0	0	1.24436984	0.30355083	0.45963766	0.28068468	0	0.5234229	0	0	0	0	0	0.37381632	0	1.10067523	0	0.52936015	0.62785738	0.86435841
ENSG00000201227	0	0	0	0	0	0	0.16076331	0.19608253	0.0989696	0	0	0	0	0.21686796	0	0	0.30115049	0.24147141	0	0	0	0	0	0.18611444
ENSG00000201228	0.31597784	0	0	0.4084383	0	0	0	0.30950281	0.31243344	0.2861883	0.39807593	0	0	0.34231119	0	0	0	0	0	0	0	0	0	0.29376887
ENSG00000201229	6.10411738	6.70536071	8.8351814	16.09618	5.03940981	11.5387027	0.58824756	1.43496758	1.68998088	0.442291	1.53802065	0.8247876	0.41332686	2.38061874	1.91114276	0.70662004	1.10193703	3.23974148	4.29931861	1.73439733	1.64428659	2.71096563	2.2260398	2.04302898
ENSG00000201231	0	0.61242526	0	0	0.12750663	0.34292666	0	0	0.11300784	0	0	0.19303539	0	0	0	0	0	0	0	0	0	0.19241652	0	0.21251365
ENSG00000201239	0	0	0	0	0	0	0	0	0	0	0	0	0	0	0	0	0	0	0	0	0	0	0	0
ENSG00000201240	0	0	0	0.5786209	0	0.33578235	0	0	0	0	0	0	0	0.96988171	0	0.43182336	0.67340596	0	0.98526051	0.39746606	0	0	0	0.41617257
ENSG00000201241	0	0	0	0	0	0	0	0	0	0	0	0	0	0	0	0	0	0	0	0	0	0	0	0
ENSG00000201242	0	0	0	0	0	0	0	0	0	0.26063577	0	0	0	0	0	0	0	0	0	0	0	0	0	0
ENSG00000201243	0	0	0	0	0	0	0	0	0	0	0	0	0	0	0	0	0	0	0	0	0	0	0	0
ENSG00000201245	0	0	0	0	0	0	0	0	0	0	0	0	0	0	0	0	0	0	0	0	0	0	0	0
ENSG00000201247	0	0.2917296	0	0.5629825	0	0.65341431	0	0	0	0	0	0	0	0	0.7575701	0.42015246	0.6552058	0	1.43794778	3.09378984	1.09989441	1.11594843	3.08837954	0
ENSG00000201253	0	0	0	0	0	0	0	0	0	0	0	0	0	0	0	0	0	0	0	0	0	0	0	0
ENSG00000201255	0	0	0	0	0	0	0	0	0	0	0	0	0	0	0	0	0	0	0	0	0	0	0	0
ENSG00000201260	0	0	0	0	0	0	0	0	0	0	0	0	0	0	0	0	0	0	0	0	0	0	0	0
ENSG00000201263	0	0	0	0	0	0	0	0	0	0	0	0	0.37888295	0	0.38930686	0	0	0	0.49263026	0.79493211	0.37681568	1.52926266	3.17416786	0.41617257
ENSG00000201264	1.34290582	0.2998332	0	0	0.24970049	0.67156471	3.5948462	5.70001012	2.87699126	10.5412689	6.20334996	4.53633178	0.37888295	3.87952683	3.11445486	3.02276352	5.3872477	2.15982765	1.97052103	4.37212661	2.26089406	3.44084099	4.53452551	1.66469028
ENSG00000201270	0	0	0	0	0	0	0	0	0	0	0	0	0	0	0	0	0	0	0	0	0	0	0	0
ENSG00000201271	0	0	0	0	0	0	0	0	0	0	0	0	0	0	0	0	0	0	0	0	0	0	0	0
ENSG00000201273	0	0	0	0	0	0	0	0	0	0	0	0	0	0	0	0	0	0	0	0	0	0	0	0
ENSG00000201274	0	0	0	0	0	0	0	0	0	0	0	0	0	0	0	0	0	0	0	0	0	0	0	0
ENSG00000201277	0	0	0	0	0	0	0	0	0	0	0	0	0	0	0	0	0	0	0	0	0	0	0	0
ENSG00000201279	0	0.2225566	0	0	0.18534469	0	0	0.32545657	0.16426913	0	0	0	0	0.3599561	0	0	0	0.40079276	0	0.29502635	0	0	0	0
ENSG00000201282	0	0	0	0	0	0	0	0	0	0	0	0	0	0	0	0	0	0	0	0	0	0	0	0
ENSG00000201283	0	0	0	0	0	0	0	0	0	0	0	0	0	0	0	0	0	0	0	0	0	0	0	0
ENSG00000201285	0	0	0	0	0	0	0	0	0	0	0	0	0	0	0	0	0	0	0	0	0	0	0	0
ENSG00000201287	0	0	0	0	0	0	0	0	0.05258781	0	0	0	0	0	0	0	0	0	0	0	0	0	0.1077511	0
ENSG00000201288	0	0	0	0	0	0	0	0	0	0	0	0	0	0	0	0	0	0	0	0	0	0	0	0
ENSG00000201289	0	0	0.09758547	0.1289805	0	0.07484932	0	0.09773773	0	0	0	0	0	0	0	0	0	0	0	0	0	0	0	0
ENSG00000201291	0	0	0	0	0	0	0	0	0	0	0	0	0	0	0	0	0	0	0	0.18227743	0	0	0	0
ENSG00000201292	0	0	0	0	0	0	0	0	0	0	0	0	0	0	0	0	0	0	0	0	0	0	0	0
ENSG00000201294	0	0	0	0	0	0	0	0	0	0	0	0	0	0	0	0	0	0	0	0	0	0	0	0
ENSG00000201296	0	0	0	0	0	0	0	0	0	0	0	0	0	0	0	0	0	0	0	0	0	0	0	0
ENSG00000201297	0	0.3889728	0	0	0.16196788	0	0.23317921	0	0	0	0	0.24520712	0	0	0	0.28010164	0	0.35024232	0	0	0	0	0	0
ENSG00000201298	0	0	0	0	0	0	0	0	0	0	0	0	0	0	0	0	0	0	0	0	0	0	0	0
ENSG00000201300	0	0	0	0	0	0	0	0	0	0	0	0	0	0	0	0	0	0	0	0	0	0	0	0
ENSG00000201301	0	0	0	0	0	0.21207306	0	0	0	0	0	0	0	0	0	0	0	0	0	0	0	0	0	0
ENSG00000201302	0.9916843	0.83030733	1.45477415	0.6409339	0.55318262	1.30180235	13.737843	14.5704401	14.0955548	15.4937941	13.7428061	18.424486	7.76418601	5.64023517	6.89971539	9.56654828	8.57815596	6.87822037	15.0062755	9.68594203	12.5218748	11.2224352	7.7854315	12.6772567
ENSG00000201308	0	0	0	0	0	0.23472164	0	0	0	0	0	0	0	0	0	0	0	0	0	0	0	0	0	0
ENSG00000201309	0	0	0	0	0	0	0	0	0	0	0	0	0	0	0	0	0	0	0	0	0	0	0	0
ENSG00000201311	0	0	0	0	0	0	0	0	0	0	0	0	0	0	0	0	0	0	0	0	0	0	0	0
ENSG00000201312	0	0	0	0	0	0	0	0	0	0	0	0	0	0	0	0	0	0	0	0	0	0	0	0
ENSG00000201314	0	0	0	0	0	0	0	0	0	0	0	0	0	0	0	0	0	0	0	0	0	0	0	0
ENSG00000201315	13.8685547	17.2049743	16.8107235	15.780569	11.4408223	14.066228	11.6865182	12.6277147	10.7675925	9.99577669	19.6866643	15.423528	9.17585619	14.7069335	12.9957708	9.61003259	11.3132202	21.3233894	12.468024	11.7939019	9.29021923	9.00874731	9.2009645	9.44333395
ENSG00000201316	0	0	0	0	0	0	0	0	0	0	0	0	0	0	0	0	0	0	0	0	0	0	0	0
ENSG00000201317	0	0	0	0	0	0	0	0	0	0	0	0	0	0	0	0	0.41797611	0.33514567	0	0	0	0	0	0
ENSG00000201318	0	0	0	0	0	0	0	0	0	0	0	0	0	0	0	0	0	0.53995691	0	0.79493211	0	0.76463133	0	0.41617257
ENSG00000201321	0	0	0.26487485	2.8007195	1.20863429	0.40632486	0	0.53057625	0	0.24530425	1.02362383	0.22872261	0	0	0.94218803	0	0	0.65339324	0	0	0.22798932	0.23131704	0	0.50360378
ENSG00000201324	0	0	0	0	0	0	0	0	0	0	0	0	0	0	0	0	0	0	0	0	0	0	0	0

ENSG00000201579	0	0	0.30307795	0	0	0.69739412	0.49774794	0	0.15321255	0.56136935	0.78084125	0.5234229	0.52460716	0.33572828	0.53904027	0	1.86481651	0	0.34105172	0.82550642	0.26087239	0.52936015	0.62785738	0.86435841
ENSG00000201581	0.10135138	0.40732058	0.19823966	0.262017	0.16960788	0.15205239	0.08139274	0.09927449	0.15032175	0.18359249	0.12768473	0.42795583	0	0.10979793	0.08814495	0.19554265	0.30493855	0.36676319	0.11153893	0.44996157	0.25595027	0.08656204	0.61601101	0.28268326
ENSG00000201583	0	0	0	0.2722922	0	0	0	0	0.05207224	0	0	0	0	0	0	0	0	0	0	0	0	0	0	0
ENSG00000201584	0	0	0	0	0	0	0	0	0	0	0	0	0	0	0	0	0	0	0	0	0	0	0	0
ENSG00000201586	0.57553107	0.57824975	0.28142952	0.3719706	0	0	0.69329177	0.56373726	0.2845376	0.78190731	0	0	0.24356761	0.93524308	0.25026869	0	0	0	0	0.25551389	0	0	0	0.80261853
ENSG00000201588	0	0	0	0	0	0	0	0	0	0	0	0	0	0	0	0	0	0	0	0	0	0	0	0
ENSG00000201591	0	0	0	0	0	0	0	0	0	0	0	0	0	0	0	0	0	0	0	0	0	0	0	0
ENSG00000201592	0.40287175	0	0	0	0.22473044	0	0.32353616	0	0.59752895	0.36489008	1.52264045	1.02067465	1.36397862	1.74578708	1.75188086	2.72048717	1.8181961	3.40172855	0	0.35771945	0.67826822	0.3440841	0.81621459	1.12366594
ENSG00000201594	0	0	0	0	0	0	0	0	0	0	0	0	0	0	0	0	0	0	0	0	0	0	0	0
ENSG00000201595	0	0	0	0	0	0	0	0	0	0	0	0	0	0	0	0	0	0	0	0	0	0	0	0
ENSG00000201596	0	0	0	0	0	0	0	0	0	0	0	0	0	0	0	0	0	0	0	0	0	0	0	0
ENSG00000201598	0	0	0	0	0	0	0	0	0	0	0	0	0	0	0	0	0	0	0	0	0	0	0	0
ENSG00000201600	0	0	0	0.4886886	0	0.14179665	0	0.18515711	0.18691033	0.08560471	0.11907257	0.07981815	0.07999875	0.20478441	0.08219969	0.09117678	0	0.1140085	0.10401577	0	0.07956225	0.08072354	0.09574365	0.08787221
ENSG00000201602	0	0	0	0	0	0	0	0	0	0	0	0	0	0	0	0	0	0	0	0	0	0	0	0
ENSG00000201604	0	0	0	0	0	0	0	0	0	0	0	0	0	0	0	0	0	0	0	0	0	0	0	0
ENSG00000201607	0	0	0	0	0.12750663	0.17146333	0	0	0	0	0	0	0	0	0	0	0	0	0	0	0	0	0	0
ENSG00000201608	0	0	0	0	0	0	0	0	0	0	0	0	0	0	0	0	0	0	0	0	0	0	0	0
ENSG00000201609	0	0	0	0	0	0	0	0	0	0	0	0	0	0	0	0	0	0	0	0	0	0	0	0
ENSG00000201610	0	0	0	0	0	0	0	0	0.14891687	0	0	0	0	0	0	0	0	0	0	0	0	0	0	0
ENSG00000201612	0	0	0	0	0	0	0	0	0	0	0	0	0	0	0	0	0	0	0	0	0	0	0	0
ENSG00000201613	0	0	0	0	0	0	0	0	0	0	0	0	0	0	0	0	0	0	0	0	0	0	0	0
ENSG00000201614	0.30405415	1.01830144	0.2973595	0	0.33921575	0.45615716	0.24417823	0	0	0	0	0	0	0	0	0	0	0	0	0	0.25595027	0	0	0
ENSG00000201616	0.39545693	0.26488332	0.38674977	0.2555871	0.11029715	0.29664208	0.79395376	0.96838303	0.29326574	1.07452293	0.24910273	0.66792615	0.8367967	0.214207	0.17196377	0.57223218	1.78473237	0.71552573	0.21760355	0.70227131	0.49933857	0.16887563	1.20178836	0.18383083
ENSG00000201618	0	0	0	0	0	0	0	0	0	0	0	0	0	0	0	0	0	0	0	0	0	0	0	0
ENSG00000201619	0	0	0	0	0	0	0	0	0	0	0	0	0	0	0	0	0	0	0	0	0	0	0	0
ENSG00000201620	0	0	0	0	0	0	0	0	0	0	0	0	0	0	0	0	0	0	0	0	0	0	0	0
ENSG00000201622	0	0	0.28917529	0	0	0.44360237	0.23745773	0	0	0	0.37251142	0	0.50054261	0	0.51431365	0	1.33445585	0	0.65081428	0.26254639	0.49781154	0	0	0
ENSG00000201623	0	0	0.29458044	0	0.16802276	0	0.48379239	0	0.74458437	0.27281501	0	0	0	0	0.52392699	0	0	0	0	0.26745379	0	0.25725914	0.30512695	0
ENSG00000201624	0.31597784	0	0	0	0	0	0	0	0	0	0	0	0	0	0	0	0	0	0	0	0	0	0	0
ENSG00000201627	0	0	0	0	0	0	0	0	0	0	0.35932518	0	0	0	0	0	0	0.34404334	0	0	0	0	0	0.2651719
ENSG00000201628	0	0	0	0.295466	0.12750663	0	0	0	0	0	0.28796982	0	0	0	0	0	0	0	0	0	0	0	0	0
ENSG00000201633	0	0	0	0	0	0	0	0	0	0	0	0	0	0	0	0	0	0	0	0	0	0	0	0
ENSG00000201634	0	0	0	0	0	0	0	0	0	0	0	0	0	0	0	0	0	0	0	0	0	0	0	0
ENSG00000201635	0	0	0	0	0	0	0	0	0	0	0	0	0	0	0	0	0	0	0	0	0	0	0	0
ENSG00000201638	0	0	0	0	0	0	0	0	0	0	0	0	0	0	0	0	0	0	0	0	0	0	0	0
ENSG00000201640	0.10707555	0	0.10471796	0.4152229	0	0	0	0	0.05293723	0.09698075	0	0	0	0	0	0.1032933	0	0.12915913	0	0.09507494	0	0	0	0
ENSG00000201641	0	0	0	0	0	0	0	0	0	0	0	0	0	0	0	0	0	0	0	0	0	0	0	0
ENSG00000201642	0	0	0	0	0	0	0	0	0	0	0	0	0	0	0	0	0	0	0	0	0	0	0	0
ENSG00000201643	1.19369407	1.27928833	0.93392909	4.3203691	1.8644303	0.89541961	1.15035078	0.70153971	1.0622737	0.86492463	7.21844359	1.20968847	0.80828363	2.84498635	1.03815162	0.46061158	2.15489908	2.8797702	2.62736137	0	0.40193672	1.22341013	1.45104816	1.33175222
ENSG00000201644	0	0	0	0	0.38663301	0	1.39155337	0	0	0	0.87319882	0	0	0	0.30139886	0	0	0.41803116	0	0.30771566	0	0.59197264	0.35106004	1.28879247
ENSG00000201648	0.23873881	0	0	0	0.13317359	0	0	0	0	0	0	0	0	0	0	0	0	0	0	0	0	0	0	0
ENSG00000201649	0	0.45448401	0	0	0.18924668	0.25448768	0	0	0	0	0	0	0	0	0	0	0	0	0	0.60247486	0.28558662	0.28975503	0.6873386	0
ENSG00000201654	0.30121252	0.20175692	0	0	0.16802276	0.45189401	2.90275432	2.06528045	0.59566749	0.81844504	3.79474254	0.50874749	0	0	0	0	0	0	1.65744759	1.33726897	3.54981498	0.25725914	0.6102539	1.96028949
ENSG00000201658	0	0	0	0	0	0	0	0	0	0	0	0	0	0	0	0	0	0	0	0	0	0	0	0
ENSG00000201659	0	0.13838455	0.20205197	0	0.11524638	0.46492941	0.33183196	0.40473445	0.1021417	0.37424623	0	0.5234229	0.17486905	0	0	0	0	0.24921088	0	0.55033762	0.17391493	0.17645338	0.41857159	0.19207965
ENSG00000201660	0	0	0	0	0	0	0	0	0	0	0	0	0	0	0	0	0	0	0	0	0	0	0	0
ENSG00000201662	0.31291009	0.41918428	0	0.4044728	0.52364374	0.70416493	0.75387066	0.30649793	0	1.13363908	0	0.26425234	0	1.01696334	0.27213683	0.90557132	0.47073038	1.13233683	0.3443629	1.1113614	0	0.53449957	1.26790616	0.29091675
ENSG00000201663	0	0	0	0	0	0.23702284	0	0	0	0	0	0	0	0	0	0	0	0	0	0	0	0	0	0
ENSG00000201665	0	0	0	0	0	0	0	0	0	0	0	0	0	0	0	0	0	0	0	0	0	0	0	0
ENSG00000201666	0	0	0	0	0	0	0	0	0	0	0	0	0	0	0	0	0	0	0	0	0	0	0	0
ENSG00000201668	0	0	0	0	0	0	0	0	0	0	0	0	0	0	0	0	0	0	0	0	0	0	0	0
ENSG00000201671	0	0	0	0	0	0	0	0	0	0	0	0	0	0	0	0	0	0	0	0	0	0	0	0
ENSG00000201672	2.57837918	1.1513595	3.7824128	2.2219041	2.63683713	3.22351058	0	0	0	0	0	0	6.91082502	6.51760508	9.71709918	4.56005468	9.05057614	6.73866228	19.862852	14.8811291	17.3636664	16.1490137	16.9772635	10.787193
ENSG00000201674	0	0	0	0	0	0	0	0	0	0	0	0	0	0	0	0	0	0	0	0	0	0	0	0
ENSG00000201675	0	0	0	0	0	0	0	0	0	0	0	0	0	0	0	0	0	0	0	0	0	0	0	0
ENSG00000201676	0	0	0	0	0	0	0	0	0	0	0	0	0	0	0	0	0	0	0	0	0	0	0	0
ENSG00000201678	2.03556251	0.68172602	1.99074358	1.7541348	0.94623342	1.27243839	1.63470901	3.32308282	2.01272911	2.15093099	3.8466706	3.15155681	1.14861358	1.10260236	1.77032171	0.6545533	1.5311125	2.04615251	0.74672376	0.90371229	0.85675985	0.57951006	0.6873386	1.26166
ENSG00000201679	0	0	0	0	0	0	0	0	0	0	0	0	0	0	0	0	0	0	0	0	0	0	0	0
ENSG00000201680	0	0	0	0	0	0	0	0	0	0	0	0	0	0	0	0	0	0	0	0	0	0	0	0
ENSG00000201683	0	0	0	0	0	0	0	0	0	0	0	0	0	0	0.30481649	0	0	0	0	0	0	0	0	0
ENSG00000201684	0.32776007	0.36589815	0.21369564	0	0	0.08195366	0.08773862	0	0.05401392	0.09895324	0	0.09226438	0	0.11835845	0.09501727	0	0	0	0	0	0	0.09331094	0	0.10157432
ENSG00000201687	0	0	0	0	0	0	0	0	0	0	0	0	0	0	0	0	0	0	0	0	0	0	0	0
ENSG00000201689	0	0	0	0	0.34537613	0	0	0	0	0	0	0.38970818	0	0	0	0	0	0.55538425	0.50670541	0	0.38758184	0	0	0
ENSG00000201690	0	0	0.26940262	0	0	0	0	0	0	0	0	0	0	0	0	0	0	0	0	0	0	0	0	0
ENSG00000201695	0	0	0	0	0	0	0	0	0	0	0	0	0	0	0	0	0	0	0	0	0	0	0	0
ENSG00000201699	0.79579604	1.19933281	0.58370568	0.7714945	0.99880195	0.89541961	4.1540445	8.5743742	7.77033538	4.50481578	25.3146807	10.9207987	21.8910149	54.5289049	41.8721154	12.2829756	21.8482823	27.1178361	55.3935356	7.59601795	34.4995686	44.8583714	10.2782578	18.1266275
ENSG00000201700	0.88300657	0.2957259	1.29534685	0.5706946	0.73883979	1.32473038	0	0	0	0	0	0	2.61584941	0.95659566	1.53589555	2.12953986	1.32836245	2.13024097	4.85881898	5.88031972	2.97323055	2.26247079	4.47240873	0.82094315
ENSG00000201701	0	0	0	0	0	0	0	0	0	0	0	0	0	0	0	0	0	0	0	0	0	0	0	0
ENSG00000201704	0	0	0	0	0	0	0	0	0	0	0	0	0	0	0	0	0	0	0	0	0	0	0	0
ENSG00000201707	0	0	0	0	0	0	0	0	0	0	0	0	0	0	0	0	0	0	0	0	0	0	0	0
ENSG00000201708	0	0	0	0	0	0	0	0	0	0	0	0	0	0	0	0	0	0	0	0	0	0	0	0
ENSG00000201709	0	0	0	0	0	0	0	0	0	0	0	0	0	0	0	0	0	0	0	0	0	0	0	0
ENSG00000201710	0.42972986	0.57567975	0.84053618	1.1109521	0	0	0	0	0	0	0	0	0.36372763	0	1.12120375	0.82910085	0	0.51835864	0.47292505	1.90783707	1.08522915	2.56916127	1.7412578	0.39952567
ENSG00000201711	0	0	0	0	0	0	0	0	0	0	0	0	0	0	0	0	0	0	0	0	0	0	0	0
ENSG00000201713	0	0	0	0	0	0	0	0	0	0	0	0	0	0	0	0	0	0	0	0	0	0	0	0
ENSG00000201715	0.1895867	0.06349409	0.0927062	0.245063	0	0	0	0	0.09373003	0.34342596	0.11942278	0.08005291	0.24070211	0.20538671	0.08244145	0.09144495	0.14260362	0.34303145	0	0.33667713	0.15959252	0.24288289	0.09602525	0.17626132
ENSG00000201723	0	0	0	0	0	0	0	0	0	0	0	0	0	0	0	0	0	0	0	0	0	0	0	0
ENSG00000201724	0	0	0	0	0	0	0	0	0	0	0	0	0	0	0	0	0	0	0	0	0.29489923	0	0	0
ENSG00000201725	0	0	0	0	0	0	0	0	0	0	0	0	0	0	0	0	0	0	0	0	0	0	0	0
ENSG00000201727	0	0	0	0	0	0	0	0	0	0	0	0	0	0	0	0	0	0	0	0	0	0	0	0
ENSG00000201728	0	0	0	0	0	0	0	0.26982296	0.54475574	0	0	0.6978972	0	0.29842514	0.9582938	0.26573745	0	0	0.30315708	0	0	0.47054236	0.55809545	0.2561062
ENSG00000201733	0	0	0	0	0	0	0	0	0	0	0	0	0	0	0	0	0	0	0	0	0	0	0	0
ENSG00000201736	0	0	0	0	0	0	0	0	0	0	0	0	0	0	0	0	0	0	0	0	0	0	0	0
ENSG00000201737	0	0	0	0	0	0	0	0	0	0	0	0	0	0	0	0	0	0	0	0	0	0	0	0
ENSG00000201741	0	0	0	0	0	0	0	0	0	0	0	0	0	0	0	0	0	0	0	0	0	0	0	0
ENSG00000201742	0	0	0	0	0	0	0	0	0	0	0	0	0	0	0	0	0	0	0	0	0	0	0	0
ENSG00000201744	0	0	0	0	0	0	0	0	0	0	0	0	0	0	0	0	0	0	0	0	0	0	0	0
ENSG00000201746	0	1.00878461	0	0	0.16802276	0	0.48379239	0.29504006	0.14891687	0.81844504	0	0	0	0	0	0	0	0	0	0.26745379	0.50711643	0	0.30512695	0
ENSG00000201747	0.30405415	0	0.2973595	0	0	0	0	0	0	0	0	0	0	0	0	0	0	0	0	0	0.25595027	0.25968611	0	0
ENSG00000201749	0	0	0	0	0	0	0	0	0	0	0	0	0	0	0	0	0	0	0	0	0	0	0	0
ENSG00000201752	0	0	0	0	0	0	0	0	0	0	0	0	0	0	0	0	0	0	0	0	0	0	0	0
ENSG00000201754	0	0	0	0	0	0	0	0	0	0	0	0	0	0	0	0	0	0	0	0	0	0	0	0
ENSG00000201756	0	0	0	0	0	0	0	0	0	0	0	0	0	0	0	0	0	0	0	0	0	0	0	0
ENSG00000201758	1.25979439	2.03925644	1.02671357	2.0355392	1.0541102	1.81125595	1.60187283	1.13114709	0.8823446	1.14102435	1.98389635	1.4185272	1.33287813	2.16090908	1.7347615	0.91147081	1.26345874	2.40606208	1.50196065	1.58468551	0.70698967	0.80697248	0.53173589	0.9760399
ENSG00000201761	0	0	0	0	0.16802276	0	0	0	0	0.27281501	0.37947425	0	0	0	0.26196349	0	0	0.36333549	0.33148952	0	0	0	0	0
ENSG00000201763	0	0	0	0	0	0	0	0	0	0	0	0	0	0	0	0	0	0	0	0	0	0	0	0
ENSG00000201766	0	0	0	0	0	0	0	0.31569287	0	0	0	0	0	0	0	0	0	0	0	0	0	0	0	0.29964425
ENSG00000201770	0	0	0.30307795	0.4005837	0	0.23246471	0.24887397	0	0	0.28068468	0.39042063	0.26171145	0.52460716	0	0.53904027	0	0	0	0	0.27516881	0	0.26468008	0.62785738	0.28811947
ENSG00000201772	48.69749	35.7698822	38.192246	69.333139	36.7442467	55.0585019	11.7134259	19.3563511	12.2122706	18.5374813	17.4862844	15.8937172	2.78769354	4.33261026	3.68278604	2.72332396	2.1234407	4.25659464	4.40130974	6.68439264	11.882071	5.42497557	3.33635162	8.53001881
ENSG00000201774	0	0	0	0	0	0	0	0	0	0	0	0	0	0	0	0	0	0	0	0	0	0	0	0
ENSG00000201775	0	0	0	0	0	0	0	0	0	0	0	0	0	0	0	0	0	0	0	0	0	0	0	0
ENSG00000201778	0	0	0	0	0	0	0	0	0	0	0	0	0	0	0	0	0	0	0	0	0	0	0	0
ENSG00000201780	0	0	0	0	0	0	0	0	0	0	0	0	0	0	0	0	0	0	0	0	0	0	0	0
ENSG00000201782	0	0	0	0	0.05992812	0	0	0	0	0	0	0	0	0	0	0	0	0	0	0	0	0	0	0
ENSG00000201785	2.12037762	2.55647257	2.07369123	3.2890028	0.94623342	5.40786315	0.68112875	2.90769747	1.04829641	1.9204741	1.60277942	1.43252582	2.5125922	5.05359417	1.47526809	2.8636707	1.27592709	2.55769064	4.66702349	6.40129542	3.56983273	3.25974409	3.43669302	1.97134375
ENSG00000201786	0	0	0	0	0	0	0	0	0	0	0	0	0	0	0	0	0	0	0	0	0	0	0	0
ENSG00000201788	0	0	0	0	0	0	0	0	0	0	0	0	0	0	0	0	0	0	0	0	0	0	0	0
ENSG00000201789	0	0	0	0	0	0	0	0	0	0	0	0	0	0	0	0	0	0	0	0	0	0	0	0
ENSG00000201790	0	0	0	0	0	0	0	0	0	0	0	0	0	0	0	0	0	0	0	0	0	0	0	0
ENSG00000201791	0	0	0	0	0	0	0	0	0	0	0	0	0	0	0	0	0	0	0	0	0	0	0	0
ENSG00000201792	0	0	0	0	0	0	0	0	0	0	0	0	0	0	0	0	0	0	0	0	0	0	0	0
ENSG00000201793	18.0012259	22.5987995	24.1408175	28.842025	12.5668659	24.5806158	14.7160259	18.4770073	14.2287831	15.327824	22.9499429	15.5661418	9.76225503	14.1297817	15.2806197	8.31873764	9.72947745	16.7729759	12.4558018	14.739477	10.4348957	12.8887689	8.18944407	11.6249943
ENSG00000201794	0	0	0.10471796	0	0	0.08032003	0	0	0	0	0.13489616	0	0	0	0	0	0.1610805	0	0	0	0	0	0	0
ENSG00000201796	0	0	0	0	0	0	0	0	0	0	0	0	0	0	0	0	0	0	0	0	0	0	0	0
ENSG00000201800	0	0	0	0	0	0	0	0	0	0	0	0	0	0	0	0	0	0	0	0	0	0	0	0
ENSG00000201801	1.07432466	1.97889914	1.31333778	1.3886901	0.89892175	1.41028588	0.64707232	0.52615478	2.39011582	0.97304021	4.39873906	1.36089953	3.40994656	4.6554322	2.56942526	1.5545641	4.04043578	3.23974148	3.54693785	0.7154389	2.48698347	2.75267279	1.63242919	2.49703542
ENSG00000201805	0	0	0	0	0	0	0	0	0	0	0	0	0	0	0	0	0	0	0	0	0	0	0	0
ENSG00000201806	0	0	0.44709371	0	0	0	0	0	0	0	0	0	0	0	0	0	0	0	0	0.20296139	0	0	0	0
ENSG00000201807	0	0	0	0	0	0	0	0	0	0	0	0	0	0	0	0	0	0	0	0	0	0	0	0
ENSG00000201809	0	0	0	0	0	0	0	0	0	0	0	0	0	0	0	0	0	0	0	0	0	0	0	0
ENSG00000201810	0	0	0	0	0	0	0	0	0	0	0	0	0	0	0	0	0	0	0	0	0	0	0	0
ENSG00000201811	0	0	0	0	0	0	0	0	0	0	0	0	0	0	0	0	0	0	0	0	0	0	0	0
ENSG00000201812	0	0	0.54345011	0	0.15498651	0	0	0	0.13736298	0	0	0	0.23516873	0	0	0	0	0	0	0	0	0	0	0
ENSG00000201813	0	0	0	0	0	0	0	0	0	0	0	0	0	0	0	0	0	0	0	0	0	0	0	0
ENSG00000201815	0	0	0	0	0	0	0	0	0	0	0	0	0	0	0	0	0	0	0	0	0	0	0	0
ENSG00000201816	0	0	0	0	0	0	0	0	0	0	0	0	0	0	0	0	0	0	0	0	0	0	0	0
ENSG00000201818	0	0	0	0	0	0	0	0	0	0	0	0	0	0	0	0	0	0	0	0	0	0	0	0
ENSG00000201820	0	0	0	0	0	0	0	0	0	0	0	0	0	0	0	0	0	0	0	0	0	0	0	0
ENSG00000201821	0	0	0	0	0	0	0	0	0	0	0	0.39162576	0.19625592	0	0	0	0.3488146	0.83906974	0	0	0	0.19803401	0	0
ENSG00000201822	0	0	0	0	0	0	0	0.34314442	0	0	0	0	0	0	0	0	0	0	0	0	0	0	0	0
ENSG00000201823	1.00717937	0	0.98500333	1.3018969	1.40456524	2.26653088	7.68398375	9.86540214	6.47323034	2.73667559	6.34433519	4.67809214	3.83618988	3.81890923	3.06579151	3.88641024	5.30307196	4.85961222	8.31313559	3.5771945	10.1740233	4.73115636	2.04053648	5.61832969
ENSG00000201825	0	0	0	0	0	0	0	0	0	0	0	0	0	0	0	0	0	0	0	0	0	0	0	0
ENSG00000201826	0	0	0	0	0	0	0	0	0	0	0	0	0	0	0	0	0	0	0	0	0	0	0	0
ENSG00000201827	0	0	0	0	0	0	0	0	0	0	0	0	0	0	0	0	0	0	0	0	0	0	0	0
ENSG00000201830	0	0	0	0	0	0	0	0	0	0	0	0	0	0	0	0	0	0	0	0	0	0	0	0
ENSG00000201831	0	0	0	0	0	0	0	0	0	0	0	0	0	0	0	0	0	0	0	0	0	0	0	0
ENSG00000201839	1.28918959	0.86351962	2.52160853	3.8883322	1.4382748	2.57880847	0.69021047	0	0.42490948	0.38921608	0	0.72581308	3.2735487	2.3277161	5.97975334	1.24365128	4.52528807	4.14686909	8.51265085	14.4995617	11.9375206	5.87236862	4.78845894	2.397154
ENSG00000201843	0	0	0	0	0	0	0	0	0	0	0	0	0	0	0	0	0	0	0	0	0	0	0	0
ENSG00000201846	0	0	0	0	0	0	0	0	0	0	0	0	0	0	0	0	0	0	0	0	0	0	0	0
ENSG00000201847	0	0	0.45681314	0	0.26055703	0	0	0	0	0	0.58846008	0	0.79071225	0.50602524	0.40623324	1.35179487	0.70268448	1.1268666	0	0	0	0	0	0
ENSG00000201850	0	0	0	0	0	0	0	0	0	0	0	0	0	0	0	0	0	0	0	0	0	0	0	0
ENSG00000201852	0	0	0	0	0	0	0	0	0	0	0	0	0	0	0	0	0	0	0	0	0	0	0	0
ENSG00000201853	0	0	0	0	0	0	0	0	0	0	0	0	0	0	0	0	0	0	0	0	0	0	0	0
ENSG00000201856	0	0	0	0	0	0	0	0	0	0	0	0	0	0	0	0	0	0	0	0	0	0	0	0
ENSG00000201860	0	0	0	0	0	0	0	0	0	0	0	0	0	0	0	0	0	0	0	0	0	0	0	0
ENSG00000201861	0	0	0	0	0	0	0	0	0	0	0	0	0	0	0	0	0	0	0	0	0	0	0	0
ENSG00000201862	0	0	0	0	0	0	0	0	0	0	0	0	0	0	0	0	0	0	0	0	0	0	0	0
ENSG00000201863	0	0.1740967	0.25419441	0	0.57994952	0.1949704	0.41746601	0	0.64250425	0.23541295	0	0.65849977	0.21999655	0	0	0	0	0	0	0.46157348	0	0	0	0
ENSG00000201867	0	0	0	0	0	0	0	0	0	0	0	0	0	0	0	0	0	0	0	0	0	0	0	0
ENSG00000201868	0	0	0	0	0	0	0.27534992	0.33584348	0	0.31054475	0	0.28955309	0	0	0	0	0	0	0	0	0	0	0	0.31877048
ENSG00000201869	0	0.21164697	0	0	0	0	0	0	0	0	0	0	0	0	0	0	0	0	0	0	0.26598754	0	0	0
ENSG00000201875	0.09796273	0	0	0.2532565	0.0546457	0.14696857	0	0.09595528	0	0	0.12341564	0	0.08291663	0.10612687	0	0	0	0.23633372	0	0.08698345	0.08246422	0	0	0
ENSG00000201876	0	0	0	0	0	0	0	0	0	0	0	0	0	0	0	0	0	0	0	0	0	0	0.38867362	0
ENSG00000201881	0	0	0	0	0	0	0	0	0	0	0	0	0	0	0	0	0	0	0	0	0	0	0	0
ENSG00000201882	9.20849708	6.1679973	7.2045958	10.712752	7.96187836	10.0159079	12.2019351	22.5494906	19.1209266	22.5189305	20.8819261	20.9967356	35.4634442	42.8964824	46.0494398	34.6445713	35.3249528	36.6553607	14.1877514	11.0382002	28.2934743	21.6281434	20.0555586	23.1154136
ENSG00000201884	0	0	0	0	0	0	0	0	0	0	0	0	0	0	0	0	0	0	0	0	0	0	0	0
ENSG00000201885	0	0	0	0	0	0	0	0	0.15621672	0	0	0	0	0	0	0	0	0	0	0	0	0	0	0
ENSG00000201892	0	0	0	0	0	0	0	0	0	0	0	0	0	0	0	0	0	0	0	0	0	0	0	0
ENSG00000201894	0	0	0	0	0	0	0	0	0	0	0	0	0	0	0	0	0	0	0	0	0	0	0	0
ENSG00000201896	0	0	0	0	0.05689378	0	0.08190789	0	0	0	0	0	0	0	0	0	0	0	0	0	0	0	0	0.09482413
ENSG00000201898	0	0	0	0	0	0	0	0	0	0	0	0.42862977	0	0	0	0	0	0	0	0	0	0	0	0
ENSG00000201899	0	1.19933281	0.87555852	0	0	0.33578235	0	0	0	0	0	0	0.37888295	0	1.16792057	0.86364672	1.34681193	0	1.47789077	1.58986422	2.63770974	0.38231567	2.72071531	0.41617257
ENSG00000201900	0	0	0	0	0	0	0	0	0	0	0	0	0	0	0	0	0	0	0	0	0	0	0	0
ENSG00000201901	15.5253015	18.0996872	17.682011	22.735566	11.7846449	25.4293708	10.7319311	17.6133521	9.23012207	9.70073622	15.845364	11.9493618	6.73672369	12.5611509	14.4423349	6.44575357	5.91283284	15.2899994	7.78596114	13.8725348	9.18143564	9.0636787	7.36583901	9.31820535
ENSG00000201907	0	0	0	0	0	0	0	0	0	0	0	0	0	0	0	0	0	0	0	0	0	0	0	0
ENSG00000201909	0	0	0	0	0	0	0	0	0	0	0	0.25437374	0	0	0	0	0	0	0	0	0	0	0	0
ENSG00000201910	0	0.13083631	0.19103095	0	0	0	0	0	0	0	0	0	0.16533074	0	0	0	0	0	0.64489779	0	0.16442866	0	0	0
ENSG00000201912	2.05098344	2.42047167	1.43273212	3.4086029	1.19856233	2.34437133	1.9608252	1.62629659	1.40026987	2.29991322	1.84562478	1.48461767	0.49599223	1.58707916	1.61385388	1.03637606	1.91002419	2.12055806	0.42993186	0.95391853	1.3976436	1.33462923	0.69254571	1.18041674
ENSG00000201913	0	0	0.31208026	0	0	0.2393696	0	0	0	0	0	0	0	0	0	0	0	0	0	0.28334214	0	0	0	0.29667748
ENSG00000201916	0	0	0	0	0	0	0	0	0	0	0	0	0	0	0	0	0	0	0	0	0	0	0	0
ENSG00000201919	0	0	0	0	0	0	0	0	0	0	0	0	0	0	0	0	0	0	0	0	0	0	0	0
ENSG00000201920	0	0	0	0	0	0	0	0	0	0	0	0	0	0	0	0	0	0	0	0	0	0	0	0
ENSG00000201922	0	0	0	0	0	0	0	0	0	0	0	0	0	0	0	0	0	0	0	0	0	0	0	0
ENSG00000201923	0	0	0	0	0	0	0	0	0	0	0	0	0	0	0	0	0	0	0	0	0	0	0	0
ENSG00000201925	0	0	0	0	0	0	0	0	0	0	0	0	0	0	0	0	0	0	0	0	0	0	0	0
ENSG00000201931	0	0	0	0	0.15235962	0	0	0	0	0	0	0	0	0	0	0	0	0	0	0	0	0.23327736	0	0
ENSG00000201933	0	0	0	0	0	0	0	0	0	0	0	0	0	0	0	0	0	0	0	0	0	0	0	0
ENSG00000201938	0	0.2248749	0	0	0	0	0	0	0	0	0	0	0	0	0	0	0	0	0	0	0	0	0	0
ENSG00000201939	0	0	0	0	0	0	0	0	0	0	0	0	0	0	0	0	0	0	0	0	0	0	0	0
ENSG00000201942	0	0.18772166	0	0	0	0.21022895	0	0	0.13855744	0	0	0	0	0	0	0	0.42161069	0	0	0	0.47183876	0	0	0
ENSG00000201943	0	0	0	0	0	0	0	0	0	0	0	0	0	0	0	0	0	0	0	0	0	0	0	0

ENSG00000201579	0	0	0	0	0.44508016	0.24928793	0	0	0	0	0	0.27566933	0	0	0	0.49779264	0	0	0.35482025	0.26458022	0.28586188	0	1.10384529	0.2897317
ENSG00000201581	0.19350343	0.2502271	0.61043363	0.22926406	0.29112161	0.40764065	0.3079785	0	0.3325399	0	0.19738884	0	0.18757773	0	0.40835018	0	0.18933509	0.36297611	0.11604184	0	0.28046826	0	0.45125751	0.47377511
ENSG00000201583	0	0.08667998	0	0	0.07563454	0	0	0	0	0	0	0.28107461	0	0	0	0	0	0	0	0	0	0.14466557	0.09379078	0
ENSG00000201584	0	0	0	0	0	0	0	0	0	0	0	0	0	0	0	0	0	0	0	0	0	0	0	0
ENSG00000201586	0	0.23682208	0.49519871	0.21698206	0.20664436	0.23148165	0	0	0	0.45290201	0	0.25597867	0.26629339	0.28997403	0.28985571	0	0	0	0	0.49136326	0	0.39524699	0.2562498	0.53807317
ENSG00000201588	0	0	0	0	0	0	0	0	0	0	0	0	0	0	0	0	0	0	0	0	0	0	0	0
ENSG00000201591	0	0	0	0	0	0	0	0	0	0	0	0	0	0	0	0	0	0	0	0	0	0	0	0
ENSG00000201592	1.15376418	2.65240725	3.11975188	1.5188744	1.15720841	0.97222294	1.22421455	0	0	0	0	0	1.8640537	0	2.02898995	1.29426087	0	0.72141503	3.69013058	2.75163427	2.22972268	1.66003736	1.79374859	3.01320973
ENSG00000201594	0	0	0	0	0	0	0	0	0	0	0	0	0	0	0	0	0	0	0	0	0	0	0	0
ENSG00000201595	0	0	0	0	0	0	0	0	0	0	0	0	0	0	0	0	0	0	0	0	0	0	0	0
ENSG00000201596	0	0	0	0	0	0	0	0	0	0	0	0	0	0	0	0	0	0	0	0	0	0	0	0
ENSG00000201598	0	0	0	0	0	0	0	0	0	0	0	0	0	0	0	0	0	0	0	0	0	0	0	0
ENSG00000201600	0	0.0777832	0.16264591	0	0.20361438	0.15205833	0	0	0	0	0	0.08746293	0.09524074	0	0	0	0.17656469	0	0.10821497	0.08069309	0.08718368	0.12981719	0.08416416	0.08836392
ENSG00000201602	0	0	0	0	0	0	0	0	0	0	0	0	0	0	0	0	0	0	0	0	0	0	0	0
ENSG00000201604	0	0	0	0	0	0	0	0	0	0	0	0	0	0	0	0	0	0	0	0	0	0	0	0
ENSG00000201607	0	0	0	0	0	0	0	0	0	0.35975195	0	0	0	0	0	0	0	0	0	0	0	0	0	0
ENSG00000201608	0	0	0	0	0	0	0.34484917	0	0	0.35721849	0	0	0	0	0	0	0	0	0	0	0	0	0	0
ENSG00000201609	0	0	0	0	0	0	0	0	0	0	0	0	0	0	0	0	0	0	0	0	0	0	0	0.21370282
ENSG00000201610	0	0	0	0	0	0	0	0	0	0	0	0	0	0	0	0	0	0	0	0	0	0	0	0
ENSG00000201612	0	0	0	0.07864722	0	0	0	0	0	0	0	0	0	0	0	0	0	0	0	0	0	0	0	0
ENSG00000201613	0	0	0	0	0	0	0	0	0	0	0	0	0	0	0	0	0	0	0	0	0	0	0	0
ENSG00000201614	0.29025514	0.2502271	0.26161441	0	0	0	0	0	0	0	0	0.27046802	0	0	0	0.48840033	0	0	0	0	0	0	0	0
ENSG00000201616	0.37750975	0.32544874	0.17012962	0.74545983	1.13591009	1.4314939	0.30042075	0.9676641	0.64875882	0	0	0.35177436	1.28082217	0.39849191	1.19498795	0.95283009	1.10813301	1.06220617	0.45277676	0.50643576	0	0.54316151	1.23251437	1.10915696
ENSG00000201618	0	0	0	0	0	0	0	0	0	0	0	0	0	0	0	0	0	0	0	0	0	0.40988577	0	0
ENSG00000201619	0	0	0	0	0	0	0	0	0	0	0	0	0	0	0	0	0	0	0	0	0	0	0	0
ENSG00000201620	0	0	0	0.20421841	0	0	0	0	0	0	0	0	0	0	0.27280537	0	0	0	0	0	0	0	0	0
ENSG00000201622	0	0	0	0	0	0	0	0	0	0	0	0	0	0	0	0.94991624	0	0	1.01563227	0	0	0	0.26330255	0
ENSG00000201623	0	0	0	0	0	0.24229855	0	0	0	0	0	0	0	0	0	0.48383584	0	0	0	0	0	0	0	0
ENSG00000201624	0	0	0	0	0	0	0	0	0	0	0	0	0	0	0	0	0	0	0	0	0.29146702	0	0	0
ENSG00000201627	0	0	0.24540821	0	0	0	0	0	0	0	0	0	0	0.28740789	0	0	0	0	0	0	0	0	0	0
ENSG00000201628	0	0.18811399	0	0.34470908	0.32828607	0	1.04188473	0.27966179	0	0	0	0	0	0.23033398	0.23023999	0	0	0.81862698	0	0	0.21084848	0.31395506	0.20354594	0
ENSG00000201633	0	0	0	0	0	0	0	0	0	0	0	0	0	0	0	0	0	0	0	0	0	0	0	0
ENSG00000201634	0	0	0	0	0	0	0	0	0	0	0	0	0	0	0	0	0	0	0	0	0	0	0	0
ENSG00000201635	0	0	0	0	0	0	0	0	0	0	0	0	0	0	0	0	0	0	0	0	0	0	0	0
ENSG00000201638	0	0	0	0	0	0	0	0	0	0	0	0	0	0	0	0	0	0	0	0	0	0	0	0
ENSG00000201640	0	0	0	0	0	0.17226542	0.16268632	0.39301308	0	0	0.20853705	0	0	0.10789731	0	0	0	0.19173821	0.2451914	0.09141642	0	0	0.19069752	0
ENSG00000201641	0	0	0	0	0	0	0	0	0	0	0	0	0	0	0	0	0	0	0	0	0	0	0	0
ENSG00000201642	0	0	0	0	0	0	0	0	0	0	0	0	0	0	0	0	0	0	0	0	0	0	0	0
ENSG00000201643	0.68371211	0.78589845	1.6433261	0.72005897	1.20006798	1.15226423	12.3328281	9.93110078	2.34994863	3.38166836	2.32480187	1.4865724	0.66277465	2.1651394	1.20236441	1.91742352	2.22994667	2.5650312	1.91340104	0.81529904	1.10109762	0.65581723	0.42518485	0.44640144
ENSG00000201644	0	0.28520508	0	0	0.24886202	0.2787736	0	0	0	0	0	0	0	0.34921603	0.34907354	0.55667134	0	0	0.39678823	0.29587465	0	0	0	0.32400105
ENSG00000201648	0	0	0	0	0	0	0	0	0	0	0	0	0	0	0	0	0	0	0	0	0	0	0	0
ENSG00000201649	0.32386363	0	0	0	0	0	0	0	0	2.13579055	0	0	0.62789177	0	0	0	0	0	0	0	0.31294353	0	0	0
ENSG00000201654	1.72525485	1.98310823	1.81418593	1.36272843	0.64890191	0.72689566	0	0	0	0	0	0	0.278737	1.51762108	2.7306033	1.93534336	1.12539365	1.61812716	0	0	0	0	0	0
ENSG00000201658	0	0	0	0	0	0	0	0	0	0	0	0	0	0	0	0	0	0	0	0	0	0	0	0
ENSG00000201659	0	0	0.35552728	0	0.14836005	0.16619196	0	0.25277123	0	0	0	0.18377955	0	0.41637296	0.20810153	0.33186176	0.38595231	0	0	0.17638681	0.19057459	0.28376707	0.18397421	0
ENSG00000201660	0	0	0	0	0	0	0	0	0	0	0	0	0	0	0	0	0	0	0	0	0	0	0	0
ENSG00000201662	0	0.25751527	0.26923425	0	0	0.50341641	0	0	0.51333829	0	0.60941408	0	0	0	0	0	0	0	0	0.80144688	0.28863724	0	0.27864056	0
ENSG00000201663	0	0	0	0.07255225	0	0	0	0	0	0	0	0	0	0	0	0	0	0	0	0	0	0	0	0
ENSG00000201665	0	0	0	0	0	0	0	0	0	0	0	0	0	0	0	0	0	0	0	0	0	0	0	0
ENSG00000201666	0	0	0	0	0	0	0	0	0	0	0	0	0	0	0	0	0	0	0	0	0	0	0	0
ENSG00000201668	0	0	0	0	0	0	0	0	0	0	0	0	0	0	0	0	0	0	0	0	0	0	0	0
ENSG00000201671	0	0	0	0	0	0	0	0	0	0	0	0	0	0	0	0	0	0	0	0	0	0	0	0
ENSG00000201672	23.3829541	24.4021467	30.6891148	18.7935392	16.3552121	21.4321146	2.61165771	2.10305664	0.70498459	1.35266735	2.51078602	1.52904589	0	0	0	0	0	0	16.728592	9.53899881	11.099064	5.31211956	6.88799458	7.63346465
ENSG00000201674	0	0	0	0	0	0	0	0	0	0	0	0	0	0	0	0	0	0	0	0	0	0	0	0
ENSG00000201675	0	0	0	0	0	0	0	0	0	0	0	0	0	0	0	0	0	0	0	0	0	0	0	0
ENSG00000201676	0	0	0	0	0	0	0	0	0	0	0	0	0	0	0	0	0	0	0	0	0	0	0	0
ENSG00000201678	0.97159089	1.39600382	0.87571983	0.51162085	0.97449129	1.09161874	2.06183504	1.2452309	4.45253424	0.53394764	1.32146633	1.2071415	1.88367532	1.02559235	2.05034774	0	1.26754863	0	1.16530439	1.15858285	0.31294353	0.9319508	1.2084201	1.58589986
ENSG00000201679	0	0	0	0	0	0	0	0	0	0	0	0	0	0	0	0	0	0	0	0	0	0	0	0
ENSG00000201680	0	0	0	0	0	0	0	0	0	0	0.28107461	0	0	0	0	0	0	0	0	0	0	0	0	0
ENSG00000201683	0	0	0	0	0	0	0	0	0	0	0	0	0	0	0	0	0	0	0	0	0	0	0	0
ENSG00000201684	0.10429507	0.08991211	0.28201147	0.08237963	0.07845481	0	0.33199039	0.13366885	0	0.68779696	0.21277848	0.09718512	0.10110122	0.11009183	0.22009382	0.350986	0	0	0	0	0.10077843	0.15005987	0.09728806	0.1021427
ENSG00000201687	0	0	0	0	0	0	0	0	0	0	0	0	0	0	0	0	0	0	0	0	0	0	0	0
ENSG00000201689	0	0.37891532	0.79231794	0	0.33063097	0.37037064	0	0	0	0	0	0	0	0	0	0	0	0	0	0	0	0.63239519	0	0.86091707
ENSG00000201690	0	0	0	0	0	0	0	0	0	0	0	0	0	0	0	0	0	0	0	0	0	0.37835609	0	0
ENSG00000201695	0	0	0	0	0	0	0	0	0	0	0	0	0	0	0	0	0	0	0	0	0	0	0	0
ENSG00000201699	9.11616145	30.6172936	16.433261	13.8011303	14.4293888	9.7622386	0	0	0	0	0	0	16.2011581	20.0475871	18.0354662	11.1849705	36.4224622	15.3189364	14.122722	13.2486095	37.9878679	33.0641187	15.7672715	9.67203123
ENSG00000201700	0.84293274	1.45337384	5.31829849	1.99742386	3.17043399	2.84119947	0	0	0.72429923	1.38972673	0.85985823	0	0	0	0	0	0	0	2.02198936	0.3769362	1.62902114	0	2.35890225	2.88937919
ENSG00000201701	0	0	0	0	0	0	0	0	0	0	0	0	0	0	0	0	0	0	0	0	0	0	0	0
ENSG00000201704	0	0	0	0	0	0	0	0	0	0	0	0	0	0	0	0	0	0	0	0	0	0	0	0
ENSG00000201707	0	0	0	0	0	0	0	0	0	0	0	0	0	0	0	0	0	0	0	0	0	0	0	0
ENSG00000201708	0	0	0	0	0	0	0	0	0	0	0	0	0	0	0	0	0	0	0	0	0	0	0	0
ENSG00000201709	0	0	0	0	0	0	0	0	0	0	0	0	0	0	0	0	0	0	0	0	0	0	0	0
ENSG00000201710	0.41022727	0.3536543	1.10924511	0.97207961	0.30858891	2.07407561	0.65291443	0	0	0	0	0	0	0	0	0	0	0	0.98403482	1.10065371	1.58558057	0	1.1479991	1.20528389
ENSG00000201711	0	0	0	0	0	0	0	0	0	0	0	0	0	0	0	0	0	0	0	0	0	0	0	0
ENSG00000201713	0	0	0	0	0	0	0	0	0	0	0	0	0	0	0	0	0	0	0	0	0	0	0	0
ENSG00000201715	0.18098262	0.39005989	0.32624856	0	0.06807108	0	0.43207572	0.46390955	0	0.14919125	0	0.16864477	0.17544035	0.19104171	0.19096376	0.76132993	0	0.84872356	0	0.64744336	0	0.13019901	0.50647019	0.44311908
ENSG00000201723	0	0	0	0	0	0	0	0	0	0	0	0	0	0	0	0	0	0	0	0	0	0	0	0
ENSG00000201724	0	0	0	0	0	0	0	0	0	0	0	0	0	0	0	0	0	0	0	0	0	0	0	0
ENSG00000201725	0	0	0	0	0	0	0	0	0	0	0	0	0	0	0	0	0	0	0	0	0	0	0	0
ENSG00000201727	0	0	0	0	0	0	0	0	0	0	0	0	0	0	0	0	0	0	0	0	0	0	0	0
ENSG00000201728	0.2629662	0	0.47403637	0.41541864	0.1978134	0	0	0	0.4519132	0.86709445	0	0	0	0.55516395	0.55493742	0.44248235	0	0	0.31539578	0	0	0	0	0
ENSG00000201733	0	0	0	0	0	0	0	0	0	0	0	0	0	0	0	0	0	0	0	0	0	0	0	0
ENSG00000201736	0	0	0	0	0	0	0	0	0	0	0	0	0	0	0	0	0	0	0	0	0	0	0	0
ENSG00000201737	0	0	0	0	0	0	0	0	0	0	0	0	0	0	0	0	0	0	0	0	0	0	0	0
ENSG00000201741	0	0	0	0	0	0	0	0	0	0	0	0	0	0	0	0	0	0	0	0	0	0	0	0
ENSG00000201742	0	0	0	0	0	0	0	0	0	0	0	0	0	0	0	0	0	0	0	0	0	0	0	0
ENSG00000201744	0	0	0	0	0	0	0	0	0	0	0	0	0	0	0	0	0	0	0	0	0	0	0	0
ENSG00000201746	0	0.74366559	0	0.45424281	0.64890191	0.24229855	0	0	0	0	0	0.26794029	0	0	0.30340037	0.48383584	0	0	0	0.25716208	0.83354119	0	0.26822409	0.56321677
ENSG00000201747	0	0	0	0	0	0	0	0	0.49880985	0	0	0	0	0	0	0	0	0.56800528	0	0	0.25958814	0	0	0
ENSG00000201749	0	0	0	0	0	0	0	0	0	0	0	0	0	0	0	0	0	0	0	0	0	0	0	0
ENSG00000201752	0	0	0	0	0	0	0	0	0	0	0	0	0	0	0	0	0	0	0	0	0	0	0	0
ENSG00000201754	0	0	0	0	0	0	0	0	0	0	0	0	0	0	0	0	0	0	0	0	0	0	0	0
ENSG00000201756	0	0	0	0	0	0	0	0	0	0	0	0	0	0	0	0	0	0	0	0	0	0	0	0
ENSG00000201758	1.80393097	0.77757867	1.89691754	1.4248724	2.03548058	1.01339199	0.95704069	0.64222006	1.20559254	0.82614048	0.40892281	1.30740895	0.97149378	1.37525141	1.05745404	1.18043337	2.15730997	0.56397266	1.20199693	1.34444671	2.03362329	1.44194342	2.33713171	1.86485293
ENSG00000201761	0	0	0	0	0	0	0	0	0.49414808	0.47406566	0	0	0	0	0	0	0	0	0	0	0	0	0	0
ENSG00000201763	0	0	0	0	0	0	0	0	0	0	0	0	0	0	0	0	0	0	0	0	0	0	0	0
ENSG00000201766	0	0	0	0	0	0	0	0	0	0	0	0	0	0	0	0	0	0	0	0	0	0	0	0
ENSG00000201770	0	0	0.26664546	0.46734597	0	0.49857587	0	0	0	0.48774063	0.60355433	0.27566933	0.57355498	0	0	0	0	0	0	0.52916044	0	0	0.27596132	0.57946341
ENSG00000201772	22.23312	20.5222751	21.8610351	21.9959621	14.0216493	16.0854404	8.22100283	7.19567736	9.6485117	5.18357925	2.74903579	10.881896	45.0638383	41.7223944	45.970692	19.2722057	28.5661051	18.9568912	4.57899415	7.02972259	5.85912529	4.84682442	6.28466659	5.93844253
ENSG00000201774	0.31718603	0	0	0	0	0	0	0	0	0	0	0	0	0	0	0	0	0	0	0	0	0	0	0
ENSG00000201775	0	0	0	0	0	0	0	0	0	0	0	0	0	0	0	0	0	0	0	0	0	0	0	0
ENSG00000201778	0	0	0	0	0	0	0	0	0	0	0	0	0	0	0	0	0	0	0	0	0	0	0	0
ENSG00000201780	0	0	0	0	0	0	0	0	0	0	0	0	0	0	0	0	0	0	0	0	0	0	0	0
ENSG00000201782	0	0.08841358	0	0.08100663	0	0.08641982	0	0	0	0.16908342	0	0	0	0	0	0	0	0	0	0	0	0	0	0
ENSG00000201785	1.21448861	0.69800191	4.37859914	4.1569194	1.21811411	5.45809371	3.22161724	1.55653863	1.39141695	1.33486909	0.82591645	1.88615859	2.7470265	2.13665073	2.56293467	1.36237987	3.96108948	4.55630543	0.971087	2.53439999	2.73825592	2.91234625	3.02105025	7.92949929
ENSG00000201786	0	0	0	0	0	0	0	0	0	0	0	0	0	0	0	0	0	0	0	0	0	0	0	0
ENSG00000201788	0	0	0	0	0	0	0	0	0	0	0	0	0	0	0	0	0	0	0	0	0	0	0	0
ENSG00000201789	0	0	0	0	0	0	0	0	0	0	0	0	0	0	0	0	0	0	0	0	0	0	0	0
ENSG00000201790	0	0	0	0	0	0	0	0	0	0	0	0	0	0	0	0	0	0	0	0	0	0	0	0
ENSG00000201791	0	0	0	0	0	0	0	0	0	0	0	0	0	0	0	0	0	0	0	0	0	0	0	0
ENSG00000201792	0	0	0	0	0	0	0	0	0	0	0	0	0	0	0	0	0	0	0	0	0	0	0	0
ENSG00000201793	14.8175735	14.63703	14.653907	13.65463	13.1588916	16.387972	22.7646586	22.5515898	16.7993819	16.1166469	19.3137386	21.6700066	20.9472255	24.4392826	18.5662759	19.2191247	20.7407414	20.267178	15.0567201	23.8352266	22.6700901	16.2857623	25.2444617	18.8451578
ENSG00000201794	0.1022161	0.08811984	0	0.16147502	0	0.08613271	0	0.52401743	0	0.33704336	0	0	0	0	0	0.20002844	0.19173821	0	0	0.09876955	0.14706865	0	0	0
ENSG00000201796	0	0	0	0	0	0	0	0	0	0	0	0	0	0	0	0	0	0	0	0	0	0	0	0
ENSG00000201800	0	0	0	0	0	0	0	0	0	0	0	0	0	0	0	0	0	0	0	0	0	0	0	0
ENSG00000201801	0.51278408	0.66310181	1.61764913	1.21509952	2.50728488	1.94444588	2.04035759	0.6572052	3.08430757	0.84541709	0.52308042	1.91130737	0	1.0825697	0.54106399	0.43142029	5.519118	0.9618867	2.46008706	2.0637257	2.97296357	2.95117753	4.06583013	0.75330243
ENSG00000201805	0	0	0	0	0	0	0	0	0	0	0	0	0	0	0	0	0	0	0	0	0	0	0	0
ENSG00000201806	0	0	0	0	0	0	0	0	0	0	0	0	0	0	0	0	0	0	0	0	0	0	0	0
ENSG00000201807	0	0	0	0	0	0	0	0	0	0	0	0	0	0	0	0	0	0	0	0	0	0	0	0
ENSG00000201809	0	0	0	0	0	0	0	0	0	0	0	0	0	0	0	0	0	0	0	0.20382476	0	0	0	0
ENSG00000201810	0	0	0	0	0	0	0	0	0	0	0	0	0	0	0	0	0	0	0	0	0	0	0	0
ENSG00000201811	0	0	0	0	0	0	0	0	0	0	0	0	0	0	0.24226746	0	0	0	0	0	0	0	0	0
ENSG00000201812	0	0	0	0.20949992	0.39903738	0	0	0	0	0	0	0	0	0	0	0	0	0	0	0	0	0	0	0
ENSG00000201813	0	0	0	0	0	0	0	0	0	0	0	0	0	0	0	0	0	0	0	0	0	0	0	0
ENSG00000201815	0	0	0	0	0	0	0	0	0	0	0	0	0	0	0	0	0	0	0	0	0	0	0	0
ENSG00000201816	0	0	0	0	0	0	0	0	0	0	0	0	0	0	0	0	0	0	0	0	0	0	0	0
ENSG00000201818	0	0	0	0	0	0	0	0	0	0	0	0	0	0	0	0	0	0	0	0	0	0	0	0
ENSG00000201820	0	0	0	0	0	0	0	0	0	0	0	0	0	0	0	0	0	0	0	0	0	0	0	0
ENSG00000201821	0	0	0	0.17483446	0	0	0	0	0.38038737	0	0.45158022	0	0.21456733	0.23364814	0.2335528	0	0	0.41520289	0	0	0	0	0	0
ENSG00000201822	0	0	0	0	0	0	0	0	0	0	0	0	0	0	0	0	0	0	0	0	0	0	0	0
ENSG00000201823	4.32661569	1.65775453	3.03309211	2.27831159	3.25464864	3.24074314	2.29540229	2.4645195	0.82615381	0	0.98077579	2.23981332	2.79608055	8.62672731	5.57972236	5.66239133	5.6445525	4.50884391	4.03608033	5.15931426	3.71620446	4.84177564	3.13906003	8.00383835
ENSG00000201825	0	0	0	0	0	0	0	0	0	0	0	0	0	0	0	0	0	0	0	0	0	0	0	0
ENSG00000201826	0	0	0	0	0	0	0	0	0	0	0	0	0	0	0	0	0	0	0	0	0	0	0	0
ENSG00000201827	0	0	0	0	0	0	0	0	0	0	0	0	0	0	0	0	0	0	0	0	0	0	0	0
ENSG00000201830	0	0	0	0	0	0	0	0	0.49414808	0	0	0	0	0	0	0	0	0	0	0	0	0	0	0
ENSG00000201831	0	0	0	0	0	0	0	0	0	0	0	0	0	0	0	0	0	0	0	0	0	0	0	0
ENSG00000201839	20.101136	12.7315548	15.1596832	9.3967696	11.1092007	18.6666805	0	0	1.40996918	0.67633367	0	1.91130737	0	0.43302788	0	0.69027247	0	0	6.39622634	7.70457596	8.324298	1.18047101	5.35732912	4.82113557
ENSG00000201843	0	0	0	0	0	0	0	0	0	0	0	0	0	0	0	0	0	0	0	0	0	0	0	0
ENSG00000201846	0	0	0	0	0	0	0	0	0	0	0	0	0	0	0	0	0	0	0	0	0	0	0	0
ENSG00000201847	0	0	0	0.35220276	0	0.37573834	0	0	0	0	0	0	0	0	0	0	0	0	0	0	0	0.64156033	0	0
ENSG00000201850	0	0	0	0	0	0	0	0	0	0	0	0	0	0	0	0	0	0	0	0	0	0	0	0
ENSG00000201852	0	0	0	0	0	0	0	0	0	0	0	0	0	0	0	0	0	0	0	0	0	0	0	0
ENSG00000201853	0	0	0	0	0	0	0	0	0	0	0	0	0	0	0	0	0	0	0	0	0	0	0	0
ENSG00000201856	0	0	0	0	0	0	0	0	0	0	0	0	0	0	0	0	0	0	0	0	0	0	0	0
ENSG00000201860	0.29870917	0	0	0	0	0	0	0	0	0	0	0	0	0	0	0	0	0	0	0	0	0	0	0
ENSG00000201861	0	0	0	0	0	0.23356707	0	0	0	0	0	0	0	0	0	0	0	0.51993876	0	0	0	0	0	0
ENSG00000201862	0	0	0	0	0	0	0	0	0	0	0	0	0	0	0	0	0	0	0	0	0	0	0	0
ENSG00000201863	0	0	0	0	0	0	0	0	0	0	0	0	0	0	0	0	0	0	0	0	0	0	0	0
ENSG00000201867	0	0	0	0	0	0	0	0	0	0	0	0	0	0	0	0	0	0	0	0	0	0	0	0
ENSG00000201868	0	0	0	0	0	0	0	0	0	0	0	0	0	0	0	0	0	0	0	0	0	0	0	0
ENSG00000201869	0	0	0	0	0	0	0	0	0	0	0	0	0	0	0	0	0	0	0	0	0	0	0	0
ENSG00000201875	0.18703371	0.40310141	0.33715657	0.14773246	0.14069403	0	0	0	0.16071077	0	0	0.08714167	0	0.09871456	0	0	0.18300474	0	0.22432405	0.0836363	0	0.13455217	0.17446795	0
ENSG00000201876	0	0	0	0	0	0	0	0	0	0	0	0	0	0	0	0	0	0	0	0	0	0	0	0
ENSG00000201881	0	0	0	0	0	0	0	0	0	0	0	0	0	0	0	0	0	0	0	0	0	0	0	0
ENSG00000201882	26.3717528	42.0596008	40.8043738	38.5360132	31.0793115	25.9259451	3.49775586	3.37991245	3.77670315	2.89857288	1.79341859	5.32435624	19.1731238	32.4770911	35.246454	23.6664846	32.6846469	22.2608065	76.4384192	76.2595784	74.3240893	56.9155667	75.8499403	46.9199801
ENSG00000201884	0	0	0	0	0	0	0	0	0	0	0	0	0	0	0	0	0	0	0	0	0	0	0	0
ENSG00000201885	0	0	0	0	0	0	0	0	0	0	0	0	0	0	0	0	0	0	0	0	0	0	0	0
ENSG00000201892	0	0	0	0	0	0	0	0	0	0	0	0	0	0	0	0	0	0	0	0	0	0	0	0
ENSG00000201894	0	0	0	0	0	0	0	0	0	0	0	0	0	0	0	0	0	0	0	0	0	0	0	0
ENSG00000201896	0	0	0	0	0	0.08204413	0	0	0	0	0	0	0	0	0	0	0	0	0	0	0	0	0.09082271	0
ENSG00000201898	0	0	0	0	0	0	0	0	0	0	0	0	0	0	0	0	0	0	0	0	0	0	0	0
ENSG00000201899	0.85464014	2.21033938	3.46639098	1.35011057	2.57157423	0.36008257	0	0	0	0	0	0	0	0	0	0	0	0	1.02503627	0	0.41291161	0	0.79722159	0
ENSG00000201900	0	0	0	0	0	0	0	0	0	0	0	0	0	0	0	0	0	0	0	0	0	0	0	0
ENSG00000201901	20.2612247	21.7529741	16.4019476	16.7446641	15.1001585	17.784566	18.0646294	16.8308648	16.4424759	17.9393383	16.0751544	17.0444331	23.1870094	22.2784923	23.0612028	20.8344433	17.9891429	18.4752629	15.7505574	27.6841253	27.19174	16.3304489	30.7999758	24.8038606
ENSG00000201907	0	0	0	0	0	0	0	0	0	0	0	0	0	0	0	0	0	0	0	0	0	0	0	0
ENSG00000201909	0	0	0	0	0	0	0	0	0	0	0	0	0	0	0	0	0	0	0	0	0	0	0	0
ENSG00000201910	0	0.16075195	0.16806744	0	0	0	0	0	0	0	0	0	0	0	0	0	0.36490036	0	0	0	0	0	0	0
ENSG00000201912	0.74586775	1.52714357	2.26891046	2.43019903	1.75334607	2.12121369	1.92906536	2.50932894	1.28179016	1.84454638	1.5216885	1.73755215	1.71718886	2.75563197	2.55775703	0.94128064	2.006952	1.74888491	1.56550994	2.91839998	2.07206552	2.14631093	3.043937	2.10011587
ENSG00000201913	0	0	0.27456562	0	0	0	0	0	0	0	0	0	0	0	0	0	0	0	0	0	0	0	0	0
ENSG00000201916	0	0	0	0	0	0	0	0	0	0	0	0	0	0	0	0	0	0	0	0	0	0	0	0
ENSG00000201919	0	0	0	0	0	0	0	0	0	0	0	0	0	0	0	0	0	0	0	0	0	0	0	0
ENSG00000201920	0	0	0	0	0	0	0	0	0	0	0	0	0	0	0	0	0	0	0	0	0	0	0	0
ENSG00000201922	0	0	0	0	0	0	0	0	0	0	0	0	0	0	0	0	0	0	0	0	0	0	0	0
ENSG00000201923	0	0	0	0	0	0	0	0	0	0	0	0	0	0	0	0	0	0	0	0	0	0	0	0
ENSG00000201925	0	0	0	0	0	0	0	0	0	0	0	0	0	0	0	0	0	0	0	0	0	0	0	0
ENSG00000201931	0	0.22478028	0	0	0	0	0.41498798	0	0	0	0	0	0.25275304	0	0	0.877465	0.51024203	0	0	0	0	0	0	0
ENSG00000201933	0	0	0	0	0	0	0	0	0	0.44889403	0	0	0	0	0	0	0	0	0	0	0	0	0	0
ENSG00000201938	0	0	0	0	0	0	0	0	0	0	0	0	0	0	0	0	0	0	0	0	0	0	0	0
ENSG00000201939	0	0	0	0	0	0	0	0	0	0	0	0	0	0	0	0	0	0	0	0	0	0	0	0
ENSG00000201942	0	0	0	0	0	0	0	0	0	0	0	0.24930096	0	0	0	0	0	0	0	0	0	0	0	0
ENSG00000201943	0	0	0	0	0	0	0	0	0	0	0	0	0	0	0	0	0	0	0	0	0	0	0	0

ENSG00000201944	1.46498817	2.12608998	1.43273212	2.2092797	1.36200265	2.74731016	0.58824756	0.95664506	1.68998088	0.88458201	1.84562478	1.23718139	0.61999028	0.79353958	0.42469839	0.23554001	1.46924937	0.29452195	0.80612224	0.650399	0.41107165	1.45975072	2.2260398	2.04302898	0.23308367	0.40187989	0.2100843	0.92052994	1.22734225	0.39281735	0.74194821	0.29872964
ENSG00000201945	0	0	0	0	0	0	0	0	0	0	0	0	0	0	0	0	0	0	0	0	0	0	0	0	0	0	0	0	0	0	0	0
ENSG00000201950	0	0.5996664	0	0.5786209	0.24970049	0.67156471	0	0	0	0	0	0	0	0	0	0.43182336	0	0.53995691	1.47789077	2.38479633	2.63770974	1.146947	2.26726276	0	2.56392041	0.3683899	2.69608188	1.35011057	2.89302101	2.16049543	0	0.547671
ENSG00000201954	0	0	0	0	0	0	0	0	0	0	0	0	0	0	0	0	0	0	0	0	0	0	0	0	0	0	0	0	0	0	0	0
ENSG00000201955	0	0	0	0	0.17625917	0	0	0	0	0	0	0	0	0	0	0	0.47534539	0.38114606	0	0	0	0	0	0	0.30163769	0.78011978	0	0.23825481	0	0.25417593	0.48008414	0
ENSG00000201957	0	0	0	0	0	0	0	0	0	0	0	0	0	0	0	0	0	0.30372576	0	0	0	0	0	0	0	0	0	0	0	0	0	0
ENSG00000201959	0	0	0	0	0	0	0	0	0	0	0	0	0	0	0	0	0	0	0	0	0	0	0	0	0	0	0	0	0	0	0	0
ENSG00000201962	0	0	0	0	0	0	0	0	0	0	0	0	0	0	0	0	0	0	0	0	0	0	0	0	0	0	0	0	0	0	0	0
ENSG00000201965	0	0	0	0	0	0	0	0	0	0	0	0	0	0	0	0	0	0	0	0	0	0	0	0	0	0	0	0	0	0	0	0
ENSG00000201966	0	0	0	0	0	0	0	0	0	0	0	0	0	0	0	0	0	0	0	0	0	0	0	0	0	0	0	0	0	0	0	0
ENSG00000201967	0	0	0	0	0	0	0.08833752	0	0.05438261	0	0	0	0	0.11916635	0	0	0.1654786	0	0	0	0.09259634	0.09394788	0	0	0.10500698	0.09052584	0.18929098	0.16588389	0	0	0	0
ENSG00000201968	0	0	0	0	0	0	0	0	0	0	0	0	0	0	0	0	0	0	0	0	0	0	0	0	0	0	0	0	0	0	0	0
ENSG00000201969	0.39304561	0	0	0	0	0	0	0	0	0	0	0	0	0	0	0	0	0	0	0.34899459	0	0	0	0	0	0	0	0	0	0	0	0
ENSG00000201980	0	0	0	0	0	0	0	0	0	0	0	0	0	0	0	0	0	0	0	0	0	0	0	0	0	0	0	0	0	0	0	0
ENSG00000201984	0	0	0	0	0	0	0	0	0	0	0	0	0	0	0	0	0	0	0	0	0	0	0	0	0	0	0	0	0	0	0	0
ENSG00000201987	0	0.21164697	0.61804131	0	0	0	0	0	0	0	0	0	0	0	0	0	0	0	0	0	0	0	0	0	0	0	0	0	0	0	0	0
ENSG00000201988	0	0.3889728	0	0	0	0	1.63225449	0.85322397	0.7177525	1.57790304	1.09739852	1.47124274	1.47457148	0.31455623	2.52523367	2.24081311	2.62082321	3.1521809	0.6390879	0.25781582	0.48884196	1.2399427	2.35305108	0.80984933	0	1.91164487	1.49897988	0.21893685	1.25103611	0.23356707	0	0
ENSG00000201990	0.31291009	0	0	0.4044728	0	0.70416493	0	0.30649793	0.15470005	0.56681954	0	0.26425234	0	0	0	0	0	0	0	0	0	0	0.31697654	0	0	0	0	0	0	0.25170821	0.47542313	0
ENSG00000201992	0.39304561	0	0	0	0.21924921	0	0	0	0	0	0	0	0	0	0	0	0	0	0	0.34899459	0	0	0	0	0	0	0	0	0	0	0	0
ENSG00000201998	5.13550799	2.72815265	2.94418578	5.7226239	5.9269566	2.52390252	6.68404371	10.9278301	4.64015159	4.49095481	21.4173601	6.28107477	1.94854089	11.510684	7.70057521	0.68332488	5.06164482	8.54437313	17.929576	2.04411114	4.47209814	4.53737273	5.5610225	7.07950701	11.15728	11.658933	8.07554822	21.2308597	11.3177525	7.69231339	13.183849	7.79979796
ENSG00000201999	0	0	0	0	0	0	0	0	0	0	0	0.2061969	0	0	0	0	0	0	0	0	0	0	0	0	0	0	0	0	0	0	0	0
ENSG00000202000	0	0	0	0	0	0	0	0	0	0	0	0	0	0	0	0	0	0	0	0	0	0	0	0	0	0.20093994	0	0	0	0	0	0
ENSG00000202001	0	0	0	0	0	0	0	0	0	0	0	0	0	0	0	0	0	0	0	0	0	0	0	0	0	0	0	0	0	0	0	0
ENSG00000202008	0	0	0	0	0	0	0	0	0	0	0	0	0	0	0	0	0	0	0	0	0	0	0	0	0	0	0	0	0	0	0	0
ENSG00000202012	0	0	0	0	0	0	0	0	0	0	0	0	0	0	0	0	0	0	0	0	0	0	0	0	0	0	0	0	0	0	0	0
ENSG00000202014	0	0	0	0	0	0	0	0	0	0	0	0	0	0	0	0	0	0	0	0	0	0	0	0	0	0	0	0	0	0	0	0
ENSG00000202016	0	0	0	0	0	0	0	0	0	0	0	0	0	0	0	0	0	0	0	0	0	0	0	0	0	0	0	0	0	0	0	0
ENSG00000202017	0	0	0	0	0	0	0	0	0	0	0	0	0	0	0	0	0	0	0	0	0	0	0	0	0	0	0	0	0	0	0	0.37554583
ENSG00000202019	0	0	0	0	0	0	0	0	0	0	0	0	0	0	0	0	0	0	0	0	0	0	0	0	0	0	0	0	0	0	0	0
ENSG00000202021	0	0	0	0	0	0	0	0	0	0	0	0	0	0	0	0	0	0	0	0	0	0	0	0	0	0	0	0	0	0	0	0
ENSG00000202023	0	0	0	0	0	0	0	0	0	0	0	0	0	0	0	0	0	0	0	0	0	0	0	0	0	0	0	0	0	0	0	0
ENSG00000202024	0	0	0	0	0	0	0	0	0	0	0	0	0	0	0	0	0	0	0	0	0	0	0	0	0	0	0	0	0	0	0	0
ENSG00000202025	0	0	0	0	0	0	0	0	0	0	0	0	0	0	0	0	0	0	0	0	0	0	0	0	0	0	0	0	0	0	0	0
ENSG00000202026	0	0	0	0	0	0	0	0	0	0	0	0	0	0	0	0	0	0	0	0	0	0	0	0	0	0	0	0	0	0	0	0
ENSG00000202027	0	0	0	0	0	0	0	0	0	0	0	0	0	0	0	0	0	0	0	0	0	0	0	0	0	0	0	0	0	0	0	0
ENSG00000202029	0	0	0	0	0	0	0	0	0	0	0	0	0	0	0	0	0	0	0	0	0	0	0	0	0	0	0	0	0	0	0	0
ENSG00000202031	1.84169942	0.61679973	1.35086171	1.1903058	0.77050436	4.48988974	4.80682292	4.96088793	2.50393086	4.58718955	4.06037452	3.88828438	2.33824907	1.49638892	2.00214956	1.33248351	2.0779384	3.33230552	4.05364326	2.04411114	5.81372759	3.53915073	1.86563335	1.71225286	2.19764606	2.65240725	3.56543072	1.04151387	2.64504778	2.22222387	2.79820469	2.25327497
ENSG00000202034	0	0	0	0	0	0	0	0	0	0	0	0	0	0	0	0	0	0	0	0	0	0	0	0	0	0	0	0	0	0	0	0
ENSG00000202035	0	0	0	0	0	0	0.25375385	0	0	0	0	0	0	0	0	0.30481649	0	0	0	0	0	0.53973976	0	0.29376887	0	0	0.2718738	0	0.22690361	0.50835187	0.48008414	0
ENSG00000202039	0	0	0	0	0	0	0	0	0	0	0	0	0	0	0	0	0	0	0	0	0	0	0	0	0	0	0	0	0	0	0	0
ENSG00000202041	0	0	0	0	0	0	0	0	0	0	0	0	0	0	0	0	0	0	0	0	0	0	0	0	0	0	0	0	0	0	0	0
ENSG00000202044	0	0	0	0	0	0	0	0	0	0.24326005	0	0	0	0	0	0	0	0	0	0	0	0	0	0	0	0	0	0	0	0	0	0
ENSG00000202046	0	0	0	0	0	0	0	0	0	0	0	0	0	0	0	0	0	0	0	0	0	0	0	0	0	0	0	0	0	0	0	0
ENSG00000202047	0	0	0	0	0	0	0	0	0	0	0	0	0	0	0	0	0	0	0	0	0	0	0	0	0	0	0	0	0	0	0	0
ENSG00000202048	0.89527055	0.89949961	1.75111703	0.5786209	0	2.01469412	0	0	0	0	0	0	4.54659541	2.42470427	4.28237544	5.61370368	2.02021789	8.0993537	7.38945386	9.53918533	8.66676057	4.20547232	7.70869337	4.57789827	6.83712109	11.4200868	22.3389641	10.8008846	7.7147227	12.9629726	2.04035759	0
ENSG00000202050	0	0	0	0	0	0	0	0	0	0	0	0	0	0	0	0	0	0	0	0	0	0	0	0	0	0	0	0	0	0	0	0
ENSG00000202051	0	0	0	0	0	0	0	0	0	0	0	0	0	0	0	0	0	0	0	0	0	0	0	0	0	0	0	0	0	0	0	0
ENSG00000202054	0	0	0	0	0	0	0	0	0	0	0	0	0	0	0	0	0	0	0	0.24664299	0.25024298	0	0.27240386	0.55940082	0.48225586	0	0	0	0	0	0	0
ENSG00000202056	0	0	0	0	0	0	0	0	0	0	0	0	0	0	0	0	0	0	0	0	0	0	0	0	0	0	0	0	0	0	0.44925305	0
ENSG00000202058	1.48051808	0.83910917	1.11378469	2.7970083	0.95292058	3.50257775	1.37188477	1.89638826	0.84456389	1.3409388	1.14780905	0.67323652	0.67475974	0.86364025	1.68378655	0.54931593	0.85662949	1.09899358	1.25333493	1.7190758	1.34215619	0.87540831	0.34609806	0.84705088	3.15280672	3.84271016	3.42964479	2.40443721	2.20810084	2.56511118	4.49888033	3.06540941
ENSG00000202059	0	0	0	0	0	0	0	0	0	0	0	0	0	0	0	0	0	0	0	0	0	0	0	0	0	0	0	0	0	0	0	0
ENSG00000202060	0	0	0	0	0	0	0	0	0	0	0	0	0	0	0	0	0	0	0	0	0	0	0	0	0	0	0	0	0	0	0	0
ENSG00000202063	0	0	0	0	0	0	0	0	0	0	0	0	0	0	0	0	0	0	0	0	0	0	0	0	0	0	0	0	0	0	0	0
ENSG00000202064	0	0	0	0	0	0	0	0	0	0	0	0	0	0	0	0	0	0	0	0	0	0	0	0	0	0	0	0	0	0	0	0
ENSG00000202067	0	0	0	0	0.14498738	0	0	0	0	0	0	0	0	0	0	0	0	0	0	0	0	0	0.52659006	0	0	0	0.22363813	0	0	0	0	0
ENSG00000202069	0	0	0	0	0	0	0	0	0	0	0	0	0	0	0	0	0	0	0	0	0	0	0	0	0	0	0	0	0	0	0	0
ENSG00000202070	0	0	0	0	0	0	0	0	0	0	0	0	0	0	0	0	0	0	0	0	0	0	0	0	0	0	0	0	0	0	0	0
ENSG00000202071	0	0.21164697	0	0	0	0	0.25375385	0.30950281	0.31243344	0.2861883	0	0	0	0	0	0	0	0	0	0.28056427	0	0	0	0	0	0	0	0	0.22690361	0	0	0
ENSG00000202072	0	0	0	0	0	0	0	0	0	0	0	0	0	0	0	0	0	0	0	0	0	0	0	0	0	0	0	0	0	0	0	0
ENSG00000202074	0	0	0	0	0	0	0	0	0	0	0	0	0	0	0	0	0	0	0	0	0	0	0	0	0	0	0	0	0	0	0	0
ENSG00000202077	0	0	0.20076501	0	0.11451232	0	1.15401432	0.80431304	1.92833123	1.1155875	6.98280969	3.64062295	1.91130762	3.11350561	4.99899762	0.99016821	3.7058774	0.24762355	2.03327648	1.09366456	1.72807189	5.61054327	3.3272442	5.72568631	1.76371595	6.25089608	4.59241608	1.70268722	3.68537709	1.32106727	2.49521437	1.00464489
ENSG00000202078	1.71131362	2.6746183	0.55787799	2.5807515	1.11370836	2.13949818	1.14526074	2.7937422	1.83312718	1.54997555	1.43730072	0.9634687	1.20706073	1.23595545	1.98443142	0.27514409	4.29072826	1.03213003	0.62777661	2.27927437	1.92075956	1.46159617	1.15570208	1.32585951	0.54454947	0.70417892	1.47244927	1.72049489	1.43370953	2.29433143	0	0.34895851
ENSG00000202079	0	0	0	0	0	0	0	0	0	0	0	0	0	0	0	0	0	0	0	0	0	0	0	0	0	0	0	0	0	0	0	0
ENSG00000202081	0	0	0	0	0	0	0	0	0	0	0	0	0	0	0	0	0	0	0	0	0	0	0	0	0	0	0	0	0	0	0	0
ENSG00000202082	0	0	0	0	0	0	0	0	0	0	0	0	0	0	0	0	0	0	0	0	0	0	0	0	0	0	0	0	0	0	0	0
ENSG00000202089	0	0	0	0	0	0	0	0	0	0	0.41013884	0	0	0	0	0	0	0	0	0	0	0	0	0	0	0	0	0	0	0	0	0
ENSG00000202092	0	0	0	0	0	0	0	0	0	0	0	0	0	0	0	0	0	0	0	0	0	0	0	0	0	0	0	0	0	0	0	0
ENSG00000202093	0	0	0	0	0	0	0	0	0	0	0	0	0	0	0	0	0	0	0	0	0	0	0	0	0	0	0	0	0	0	0	0
ENSG00000202095	0	0	0	0	0	0	0	0	0	0	0	0	0	0	0	0	0	0	0	0	0	0	0	0	0	0	0	0	0	0	0	0
ENSG00000202099	0	0	0	0	0	0	0	0.30355083	0	0	0	0	0	0	0	0	0	0	0	0	0	0	0	0	0	0	0.26664546	0	0	0	0	0
ENSG00000202100	0	0	0	0	0	0	0	0	0	0	0	0	0	0	0	0	0	0	0	0	0	0	0	0	0	0	0	0	0	0	0	0
ENSG00000202103	0	0	0	0	0	0	0	0	0	0	0	0	0	0	0	0	0	0	0	0	0	0	0	0	0	0	0	0	0	0	0	0
ENSG00000202105	0	0.21164697	0	0.8168765	0.17625917	0	0	0	0	0	0.39807593	0	0	0	0	0	0	0	0	0	0	0.26986988	0	0	0	0	0	0	0	0	0	0
ENSG00000202107	0	0	0	0	0	0	0	0	0	0	0	0	0	0	0	0	0	0	0	0	0	0	0	0	0	0	0	0	0	0	0	0
ENSG00000202111	0	0	0	0	0	0	0	0	0	0	0.45622186	0	0	0	0	0	0	0	0.79706469	0	0.30483965	0	0	0	1.38278853	0	0.31158571	0.27305607	0.52009367	0.87390826	0	0
ENSG00000202112	0	0	0	0	0	0	0	0	0	0	0	0	0	0	0	0	0	0	0	0	0	0	0	0	0	0	0	0	0	0	0	0
ENSG00000202119	0	0	0	0	0	0	0	0	0.14891687	0	0	0	0	0	0	0	0	0	0.33148952	0.26745379	0.25355821	0	0	0.28004136	0	0	0	0	0.64890191	0.24229855	0	0
ENSG00000202124	0	0	0	0	0	0	0	0	0.16772743	0	0	0.28650516	0	0	0	0	0	0	0	0	0	0	0	0	0	0	0	0	0	0	0	0
ENSG00000202125	0.19533176	1.9625446	0.3820619	0.2524891	0.54480106	0.87913925	0.15686602	0.57398703	0.38628134	0.1769164	0.49216661	0.16495752	0.16533074	0.42322111	0.16987936	0.37686402	0	1.17808781	1.07482965	1.21407813	0.49328598	0.16682865	0.59361061	0.18160258	0	0.64300782	0.84033721	0.44185437	0.42080306	0.94276164	0.59355857	0.47796742
ENSG00000202129	0	0	0	0	0	0	0	0	0.2042834	0	0	0	0.34973811	0	0	0	0	0.49842177	0	0.36689174	0.34782986	0	0	0	0	0	0	0	0	0	0	0
ENSG00000202137	0.31597784	0	0	0.4084383	0	0	0	0	0.15621672	0	0	0	0	0.34231119	0	0	0	0.38114606	0	0	0	0	0	0	0	0	0	0	0	0	0.48008414	0
ENSG00000202140	0	0	0	0	0	0.24176329	0	0	0	0	0	0	0	0	0	0	0	0	0	0	0	0	0	0	0	0.26524073	0	0	0	0	0	0
ENSG00000202141	0.57043787	0.95522082	1.39469498	0.3686788	0.15910119	1.71159854	0.45810429	1.3968711	0.56403913	0.77498778	0.35932518	0.24086717	0	0.30898886	0.49610785	0.27514409	2.14536413	0.68808669	0.31388831	0.75975812	0.72028483	1.21799681	1.15570208	0.2651719	0	0.23472631	0.24540821	0.21506186	0.20481565	0.22943314	1.30005085	0
ENSG00000202142	0	0	0.43777926	0.5786209	0	0	0	0	0	0	0	0	0	0	0	0	0	0.53995691	0	0.79493211	0.37681568	0	1.81381021	0	0	0.3683899	0.38515455	0.33752764	0.32144678	0.72016514	0	0
ENSG00000202144	0	0	0	0	0	0	0	0	0	0	0	0	0	0	0	0	0	0	0	0	0	0	0	0	0	0	0	0	0	0	0	0
ENSG00000202146	0	0	0	0	0	0	0	0	0	0	0	0	0	0	0	0	0	0	0	0	0	0	0	0	0	0	0	0	0	0	0	0
ENSG00000202147	0	0	0	0	0	0	0	0	0	0	0	0	0	0	0	0	0	0	0	0	0	0	0	0	0	0	0	0	0	0	0	0
ENSG00000202150	0	0.41515366	0.6061559	0	0	0.46492941	1.24436984	0	1.22570042	1.1227387	1.17126188	0.78513435	0.52460716	0.33572828	0.8085604	0	0.46620413	0	1.02315515	0	0.26087239	0.79404023	0.94178607	0.28811947	0.88751091	0.25503916	0.53329092	0	0.22254008	0.49857587	0	0.75831369
ENSG00000202151	0	0	0	0	0	0	0	0	0	0	0	0	0	0	0	0	0	0	0	0	0	0	0	0	0	0	0	0	0	0	0	0
ENSG00000202157	0	0	0	0	0	0	0	0	0	0	0	0	0	0	0	0	0	0	0	0	0	0	0	0	0	0	0	0	0	0	0	0
ENSG00000202159	0	0	0	0	0	0	0	0	0	0	0	0	0	0	0	0	0	0	0	0	0	0	0	0	0	0	0.25916942	0	0	0	0	0
ENSG00000202160	0	0	0	0	0	0	0	0	0	0	0	0	0	0	0	0	0	0	0	0	0	0	0	0	0	0	0	0	0	0	0	0
ENSG00000202164	0	0	0	0	0	0	0	0	0	0	0	0	0	0	0	0	0	0	0	0	0	0	0	0	0	0	0	0	0	0	0	0
ENSG00000202167	0	0	0	0	0	0	0	0	0	0	0	0	0	0	0	0	0	0	0	0	0	0	0	0	0	0	0	0	0	0	0	0
ENSG00000202169	0	0	0.32833444	0	0	0.25183676	0	0	0	0	0	0	0	0	0	0	0	0	0	0	0	0	0	0	0	0	0	0	0	0	0	0
ENSG00000202172	0.92084971	0.41119982	1.20076597	0.3967686	0.17122319	0.23025076	0	0	0	0	0	0	0	0	0	0	0	0	0	0	0	0	0	0	0	0	0	0	0	0	0	0
ENSG00000202174	0.21203776	0.14202625	0.20736912	0.5481671	0.59139589	0	0.17028219	0.20769268	0.10482964	1.15228446	2.40416912	0.17906573	0	1.83767061	0	0	0	1.02307626	2.33351174	0	0.35698327	0.36219379	0	0	0	0.69800191	0.36488326	0	0.15226426	0.51169629	0	0
ENSG00000202175	0	0.18610337	0	0.359144	0	0	0	0	0	0	0	0	0	0	0	0	0	0	0	0	0	0	0	0	0	0	0	0	0	0	0	0
ENSG00000202177	0	0	0	0	0	0	0	0	0	0	0	0	0	0	0	0	0.44481862	0	0	0	0	0	0	0	0	0	0	0.22295404	0	0	0	0
ENSG00000202178	0	0	0	0	0	0	0	0	0	0	0	0	0	0	0	0	0	0	0	0	0	0	0	0	0	0	0	0	0	0	0	0
ENSG00000202182	0	0	0	0	0	0	0	0	0	0	0	0	0	0	0	0	0	0	0	0	0	0	0	0	0	0	0	0	0	0	0	0
ENSG00000202183	0	0	0	0	0	0	0	0	0	0	0	0	0	0	0	0	0	0	0	0	0	0	0	0	0	0	0	0	0	0	0	0
ENSG00000202184	0	0.41119982	0.60038298	0.3967686	0	0.23025076	0.49300748	0.30065987	0.45526016	0.55602298	0.38670234	0	0.25980545	0	0	0	0	0	0.3378036	0.27254815	0	0.26215931	0.31093889	0.28537548	0	0.26410598	0	0	0	0	0.46636745	0
ENSG00000202186	0	0	0	0	0	0	0	0	0	0	0	0	0	0	0	0	0	0	0	0	0	0	0	0	0	0	0	0	0	0	0	0
ENSG00000202187	0	0	0.46696454	0.3085978	0.26634719	0	0.19172513	0.23384657	0.23606082	0	0	0	0	0	0.20763032	0	0	0	0	0	0	0	0	0	0	0	0	0	0	0	0	0
ENSG00000202188	0	0	0	0	0	0	0	0	0	0	0	0	0	0	0	0	0	0	0	0	0	0	0	0	0	0	0	0	0	0	0	0
ENSG00000202189	0	0	0	0	0	0.37482681	0.20064258	0	0.61760099	0	0.94427314	1.47694523	0.21146955	0.27066466	0	0	0.37585449	0	0	0	0.21031573	0.21338549	0	0.46456473	0	0.41122593	0	0.18838752	0	0	0.37960141	0.30567684
ENSG00000202190	0	0	0	0	0	0	0	0	0	0	0	0	0	0	0	0	0	0	0	0	0	0	0	0	0	0	0	0	0	0	0	0
ENSG00000202191	0	0.93860829	0.45681314	0	0	1.40152634	0	0	0	0	0	0	0.39535612	1.51807572	0.81246649	1.35179487	0	0	2.05619586	1.24424156	1.96599484	1.59575234	0.94633576	0	0	0.7688137	3.61710363	1.05660828	2.01253636	3.38164502	0	0
ENSG00000202193	0	0	0	0	0	0	0	0	0	0	0	0.22872261	0	0	0	0	0	0	0	0	0	0	0	0	0	0	0	0	0	0	0	0
ENSG00000202195	0	0	0	0	0	0	0	0	0	0	0	0	0	0	0	0	0	0	0	0	0	0	0	0	0	0	0	0	0	0	0	0
ENSG00000202198	41905.0882	50941.2009	42765.1665	52790.655	34949.4259	46350.8441	36735.4879	41130.4888	36099.8472	39699.8641	63481.0729	56041.8428	28468.5003	50320.0181	44783.5369	27510.8061	41701.1057	59393.4464	39479.24	33396.6878	28346.9395	37050.6431	28046.317	29728.1497	27847.243	25810.7272	27818.6778	29655.4765	27603.3711	27747.889	43122.2364	35240.2164
ENSG00000202199	0	0	0	0	0	0	0	0	0	0	0	0	0	0	0	0	0	0	0	0	0	0	0	0	0	0	0	0	0	0	0	0
ENSG00000202200	0	0	0	0	0	0	0	0	0	0	0	0	0	0	0	0	0	0	0	0	0	0	0	0	0	0	0	0	0	0	0	0
ENSG00000202205	0	0	0	0	0	0	0	0	0	0	0	0	0	0	0	0	0	0	0	0	0	0	0	0	0	0	0	0	0	0	0	0
ENSG00000202206	0	0	0	0	0	0	0	0	0	0	0	0	0	0	0	0	0	0	0	0	0	0	0	0	0	0	0	0	0	0	0	0
ENSG00000202211	0	0	0	0	0	0	0	0	0	0	0	0	0	0	0	0	0	0	0	0	0	0	0	0	0	0	0	0	0	0	0	0
ENSG00000202215	0	0.36797711	0.17909151	0.2367085	0.4086008	0.41209652	0.14706189	0	0	0	0	0	0	0	0	0	0	0	0	0	0	0	0	0	0	0	0	0	0.26300191	0.14730651	0	0
ENSG00000202216	0	0	0	0	0	0.35038159	0.37511439	0.22876295	0.23092906	0	0.88269011	0	0	0	0.20311662	0	0	0	0.77107345	0	0.58979845	0.39893809	0.23658394	0.43426703	0	0.7688137	0	0.35220276	0.67084545	0.37573834	0	0.57148278
ENSG00000202217	0	0	0	0.1270143	0	0.07370832	0	0	0	0	0.12379191	0	0	0	0	0	0	0	0	0	0	0	0	0.09135495	0.09380197	0.08086607	0	0.14818287	0.14112298	0.07904252	0.14929446	0
ENSG00000202220	0	0	0	0	0	0	0	0	0	0	0	0	0	0	0	0	0	0	0	0	0	0	0	0	0	0	0	0	0	0	0	0
ENSG00000202222	0	0.95522082	0.278939	0	0.31820239	0.42789964	0	0.27937422	0.98706848	2.06663407	1.07797554	0.48173435	0.72423644	0.92696659	0	0	0.85814565	0	0.62777661	0.50650542	0	0.48719872	1.73355312	0.2651719	0.54454947	0	0.73622463	0.86024745	0.20481565	0.22943314	0	0
ENSG00000202224	0	0	0.28142952	0	0	0	0	0	0	0	0	0	0	0	0	0	0	0	0	0	0	0	0	0	0	0	0	0	0	0	0	0
ENSG00000202225	0	0	0	0	0	0	0	0	0	0	0	0	0	0	0	0	0	0	0	0	0	0	0	0	0	0	0	0	0	0	0	0
ENSG00000202227	0	0	0	0	0	0	0	0	0	0	0	0	0	0	0	0	0	0	0	0	0	0	0	0	0	0	0	0	0	0	0	0
ENSG00000202229	0	0	0	0	0	0	0	0	0.15032175	0	0	0	0	0	0	0	0	0	0	0	0	0	0	0	0	0	0	0	0	0	0	0
ENSG00000202231	0	0	0	0	0	0	0	0	0	0	0	0	0	0	0	0	0	0	0	0	0	0	0	0	0	0	0	0	0	0.19493192	0	0
ENSG00000202233	0	0	0	0	0	0	0	0	0	0	0	0	0	0	0	0	0	0	0	0	0	0	0	0	0	0	0	0	0	0	0	0
ENSG00000202237	0	0	0	0	0	0	0	0	0	0	0	0	0.25735446	0	0	0	0.45740782	0	0	0	0	0	0	0	0	0	0	0	0.21834121	0	0	0.37200294
ENSG00000202239	0	0	0	0	0	0.23025076	0	0	0	0	0	0.25921896	0	0	0	0	0	0	0	0	0	0	0	0	0	0	0	0	0	0	0	0
ENSG00000202240	0	0	0	0	0	0	0	0	0	0	0	0	0	0	0	0	0	0	0	0	0	0	0	0	0	0	0	0	0	0	0	0
ENSG00000202241	3.93564479	6.12728445	4.26509694	4.6747983	3.67875568	6.30339941	3.50230561	5.00107514	3.8914977	3.66094336	4.55619583	4.40158925	2.25078981	4.37887187	4.25539378	2.66789878	4.80051775	3.33597142	1.99003114	4.91126373	2.32804933	2.45287674	3.23253304	2.4723123	2.74161786	2.5386076	3.47783122	3.04777437	2.2151184	1.88241186	4.20192454	5.07544609
ENSG00000202242	0	0	0	0	0	0	0	0	0.30642511	0	0	0	0	0	0	0	0	0	0	0.27516881	0	0	0	0	0	0	0	0.23367298	0	0	0	0
ENSG00000202245	0	0	0	0	0	0	0	0	0	0	0	0	0	0	0	0	0	0	0	0	0	0	0	0	0	0	0	0	0	0	0	0
ENSG00000202248	0	0	0	0	0	0	0	0	0	0	0	0	0	0	0	0	0	0	0	0	0	0	0	0	0	0	0	0	0	0	0	0
ENSG00000202249	0	0	0	0	0	0	0	0	0	0	0	0	0	0	0	0	0	0	0	0	0	0	0	0	0	0	0	0	0	0	0	0
ENSG00000202250	0	0	0	0	0	0	0	0	0	0	0	0	0	0	0	0	0	0	0	0	0	0	0	0	0	0	0	0	0	0	0	0
ENSG00000202251	0	0	0	0	0	0	0	0	0	0	0	0	0	0	0	0	0	0	0	0	0	0	0	0	0	0	0	0	0	0	0	0
ENSG00000202252	0	0	0	0	0	0	0	0	0	0	0	0	0	0	0	0	0	0	0	0	0	0	0	0	0	0	0	0	0	0	0	0
ENSG00000202254	0	0	0	0	0	0	0	0	0	0	0	0	0	0	0	0	0	0	0	0	0	0	0	0	0	0	0	0	0	0	0	0
ENSG00000202255	0	0.38549983	0	0	0.16052174	0	0.23109726	0.28186863	0	0.26063577	0	0	0.24356761	0.31174769	0	0.27760073	0	0.69423032	0.31669088	0.25551389	0	0	0.29150521	0	0.27470576	0	0.49519871	0	0	0	0	0
ENSG00000202257	0	0	0	0	0	0	0	0	0	0	0	0	0	0	0	0	0	0	0	0	0	0	0	0	0	0	0	0	0	0	0	0
ENSG00000202259	0	0	0	0	0	0	0	0	0	0	0	0	0	0	0	0	0	0	0	0	0	0	0	0	0	0	0	0	0	0	0	0
ENSG00000202260	0.10567128	0	0	0	0	0	0	0	0	0	0	0	0	0	0	0	0	0	0	0	0.08895321	0	0	0	0	0	0	0	0	0	0	0
ENSG00000202261	0	0	0	0	0	0	0	0	0	0	0	0	0	0	0	0	0	0	0	0	0	0	0	0	0	0	0	0	0	0	0	0
ENSG00000202263	0	0	0	0	0	0.23745773	0	0	0	0	0	0	0.25027131	0	0	0	0	0.35666879	0	0	0	0.25253879	0	0	0.28226647	0.24334012	0	0.22295404	0.21233182	0.23785271	0	0.36176433
ENSG00000202264	0	0	0	0	0	0	0	0	0	0	0	0	0	0	0	0	0	0	0	0	0	0	0	0	0	0	0	0	0	0	0	0
ENSG00000202265	0	0	0	0	0	0	0	0	0	0	0	0	0	0	0	0.31725798	0	0.39670304	0.36193243	0	0	0	0.33314881	0	0	0	0	0	0	0	0	0
ENSG00000202268	0	0	0	0	0	0	0	0	0	0	0	0	0	0	0	0	0	0	0	0	0	0	0	0	0	0	0	0	0	0	0	0
ENSG00000202269	0	0	0	0	0	0	0	0	0	0	0	0	0	0	0	0	0	0	0	0	0	0	0	0	0	0	0	0	0	0	0	0
ENSG00000202270	0	0	0.84053618	0	0	0	0	0	0	0	0	0	0	0	0.74746917	0	0	0	0	1.14470224	0	0.73404608	0	0.39952567	0.41022727	0.3536543	0.73949674	0.64805308	0.61717782	0.69135854	0	0
ENSG00000202272	0	0	0	0	0	0	0	0	0	0	0	0	0	0	0	0	0	0	0	0	0	0	0	0	0	0	0.27456562	0	0	0	0	0
ENSG00000202273	0.31291009	0	0	0	0.17454791	0.23472164	0	0	0	0	0	0	0	0	0	0	0	0	0	0	0	0	0	0	0	0	0	0	0	0	0	0.76567596
ENSG00000202275	0	0	0	0	0	0	0	0	0	0	0	0	0	0	0	0	0	0	0	0	0	0	0	0	0	0	0	0	0	0	0	0
ENSG00000202276	0.63195568	0	0	0	0	0	0	0	0	0	0	0	0	0	0	0	0	0	0	0	0	0	0.32008415	0	0	0	0	0	0	0	0	0
ENSG00000202279	0.3288749	0.44057124	0	0.8502184	0	0	0	0	0	0	0	0	0.27836298	0	0.28602137	0	0	0	0	0	0	0.28088498	0	0	0	0	0	0	0.23616498	0	0	0
ENSG00000202281	0	0	0	0	0	0	0	0	0	0	0	0	0	0	0	0	0	0	0	0	0	0	0	0	0	0	0	0	0	0	0	0
ENSG00000202283	0	0	0	0	0	0	0	0	0	0	0	0	0	0	0	0	0	0	0	0	0	0	0	0	0	0	0	0	0	0	0	0
ENSG00000202285	0	0	0	0	0	0	0	0	0	0	0	0	0	0	0	0	0	0	0	0	0	0	0	0	0	0	0	0	0	0	0	0
ENSG00000202290	0	0	0	0	0	0	0	0	0	0	0	0	0	0	0	0	0	0	0	0	0.20869791	0	0	0	0	0	0	0	0	0	0	0
ENSG00000202293	0	0	0	0.5786209	0	0.33578235	0	0	0	0	0	0	0.37888295	0.96988171	0	0	0	0	0	1.19239817	1.13044703	0.76463133	1.36035765	0	0.85464014	0	0.38515455	0.67505529	0.64289356	0.36008257	0	0
ENSG00000202296	0	0	0	0	0	0	0	0	0	0	0	0	0	0	0	0	0	0	0	0	0	0	0	0	0	0	0	0	0	0	0	0
ENSG00000202297	0	0.21164697	0	0	0.17625917	0.47404567	0	0.61900562	0.31243344	0.2861883	0.39807593	0	0.26744679	0.34231119	0	0	0	0	0	0	0	0	0	0	0	0.26003993	0	0	0	0.25417593	0	0
ENSG00000202300	0	0	0	0	0	0	0	0	0	0	0	0	0	0	0	0	0	0	0	0	0	0	0	0	0	0	0	0	0	0	0	0
ENSG00000202304	0	0	0	0	0	0	0	0	0	0	0	0	0	0	0	0	0	0	0	0	0	0	0	0	0	0	0	0	0	0	0	0
ENSG00000202306	0	0	0	0	0	0	0	0	0	0	0	0	0	0	0	0	0	0	0	0	0	0	0	0	0	0	0	0	0	0	0	0
ENSG00000202308	0	0	0	0	0	0	0	0	0	0	0	0	0	0	0	0	0	0	0	0	0	0	0	0	0	0	0	0	0	0	0	0
ENSG00000202309	0.31597784	0	0	0	0	0	0	0	0.15621672	0	0	0	0	0	0	0	0	0	0	0	0	0	0	0	0	0	0	0.23825481	0	0	0.48008414	0
ENSG00000202310	0	0	0	0	0	0	0.47931283	1.16923285	0.29507603	0.27028895	0	0	0	0	0	0.28788224	0	0	0	0	0	0	0	0	0	0	0	0	0	0.4801101	0	0
ENSG00000202313	0	0	0	0	0	0	0	0	0	0	0	0	0	0	0	0	0	0	0	0	0	0	0	0	0	0	0	0	0	0	0	0
ENSG00000202314	0.88300657	0.5914518	0.86356457	2.2827782	1.23139966	2.31827816	4.96384243	7.78420771	4.58378376	7.19783168	7.23080394	4.47419024	1.86846387	1.91319132	5.37563442	5.11089566	3.98508734	3.19536146	3.40117328	4.31223446	5.20315346	4.14786311	5.81413134	5.33613049	2.10733184	4.36012151	5.31829849	0.99871193	4.75565098	2.84119947	3.35401247	4.86151791
ENSG00000202317	0	0	0	0	0	0	0	0	0	0	0	0	0	0	0	0	0	0	0	0	0	0	0	0	0	0	0	0	0	0	0	0
ENSG00000202318	0	0	0	0	0	0	0	0	0	0	0	0	0	0	0	0	0	0	0	0	0	0	0	0	0	0	0	0	0	0	0	0
ENSG00000202322	0	0	0	0	0	0	0	0	0	0	0	0	0	0	0	0	0	0	0	0	0	0	0	0	0	0	0	0	0.19448881	0	0	0
ENSG00000202324	0	0	0	0	0	0	0	0	0	0	0	0	0	0	0	0	0	0	0	0	0	0	0	0	0	0	0	0	0	0	0	0
ENSG00000202329	0	0	0	0	0	0	0	0	0	0	0	0	0	0	0	0	0	0	0	0	0	0	0	0	0	0	0	0	0	0	0	0
ENSG00000202331	0	0	0	0	0	0	0	0	0	0	0	0	0	0	0	0	0	0	0	0	0	0	0	0	0	0	0	0	0	0	0	0
ENSG00000202332	0	0	0	0	0	0	0	0	0	0	0	0	0	0	0	0	0	0	0	0	0	0	0	0	0	0	0	0	0	0	0	0
ENSG00000202334	0	0	0	0	0	0	0	0	0	0	0	0	0	0	0	0	0	0	0	0	0	0	0	0	0	0	0	0	0	0	0	0
ENSG00000202335	0	0	0	0	0	0	0	0	0	0	0	0	0	0	0	0	0	0	0	0	0	0	0	0	0	0	0	0	0	0	0	0
ENSG00000202336	0	0	0	0	0	0.4477098	0	0	0	0	0	0	0	0	0	0	0	0	0	0	0	0	0	0	0	0	0	0	0	0	0	0

ENSG00000201944	0.80111885	0.3842805	0.47552766	0.21719402	0.2259459	0.49207714	2.45938176	0.78440053	1.36837636	1.31166368	0.27955535	0.41691428	0.22522451	0	0.21742407	0
ENSG00000201945	0	0	0	0	0	0	0	0	0	0	0	0	0	0	0	0
ENSG00000201950	0	0	0	0	0	0	0	0	0	0	0	1.14651428	1.65164643	0	0.3986108	1.25550405
ENSG00000201954	0	0	0	0	0	0	0	0	0	0	0	0	0	0	0	0
ENSG00000201955	0	0	0	0.28107461	0.29240058	0.95520856	0	0.50755328	0	0.56581571	0	0.26976807	0.29146702	0.4339967	0	0
ENSG00000201957	0	0	0	0	0	0	0	0	0	0	0	0	0	0	0	0
ENSG00000201959	0	0	0	0	0	0	0	0	0	0	0	0	0	0	0	0
ENSG00000201962	0	0	0	0	0	0	0	0	0	0	0	0	0	0	0	0
ENSG00000201965	0	0	0	0	0	0	0	0	0	0	0	0	0	0	0	0
ENSG00000201966	0	0	0	0	0	0	0	0	0	0	0	0	0	0	0	0
ENSG00000201967	0.18045681	0.17312295	0	0	0	0.11084331	0.11079809	0	0	0.19697339	0	0.09391243	0	0	0	0.10283992
ENSG00000201968	0	0	0	0	0.26162157	0	0	0	0	0	0	0	0	0	0	0
ENSG00000201969	0	0	0	0	0	0	0	0	0	0	0	0	0	0	0	0
ENSG00000201980	0	0	0	0	0	0	0	0	0	0	0	0	0	0	0	0
ENSG00000201984	0	0	0	0	0	0	0	0	0	0	0	0	0	0	0	0
ENSG00000201987	0	0	0	0	0	0	0	0	0	0	0	0	0	0	0	0
ENSG00000201988	0	0	0	0.25828478	0.80607728	0.58517281	0.87740106	0.46640032	0	1.03987752	0.3324442	0.99157992	1.87484189	1.99403887	1.55135013	5.15774639
ENSG00000201990	0	0	0	0	0.28956174	0	0.3151829	0	0.58454913	0	0	0	0.28863724	0	0.27864056	0
ENSG00000201992	0	0	0	0	0	0	0	0	0	0	0	0	0	0	0	0
ENSG00000201998	2.32412502	9.47610366	4.82843466	5.8284373	16.3872853	24.6254866	10.5239918	15.3604587	15.5483644	11.7329037	13.1790378	4.38447219	8.16748234	13.1343616	8.98845446	3.80790241
ENSG00000201999	0	0	0	0	0	0	0	0	0	0	0	0	0	0.33536108	0	0
ENSG00000202000	0	0	0	0	0	0	0	0	0	0	0	0	0	0	0	0
ENSG00000202001	0	0	0	0	0	0	0	0	0	0	0	0	0	0	0	0
ENSG00000202008	0	0	0	0	0	0	0	0	0	0	0	0	0	0	0	0
ENSG00000202012	0	0	0	0	0	0	0	0	0	0	0	0	0	0	0	0
ENSG00000202014	0	0	0	0	0	0	0	0	0	0	0	0	0	0	0	0
ENSG00000202016	0	0	0.58120047	0	0	0	0	0	0	0	0	0	0	0	0	0
ENSG00000202017	0	0	0	0	0	0	0	0	0	0	0	0	0	0	0	0
ENSG00000202019	0	0	0	0	0	0	0	0	0	0	0	0	0	0	0	0
ENSG00000202021	0	0	0	0	0	0	0	0	0	0	0	0	0	0	0	0
ENSG00000202023	0	0	0	0	0	0	0	0	0	0	0	0	0	0	0	0
ENSG00000202024	0	0	0	0	0	0	0	0	0	0	0	0	0	0	0	0
ENSG00000202025	0	0	0	0	0	0	0	0	0	0	0	0	0	0	0	0
ENSG00000202026	0	0	0	0	0	0	0	0	0	0	0	0	0	0	0	0
ENSG00000202027	0	0	0	0	0	0	0	0	0	0	0	0	0	0	0	0
ENSG00000202029	0	0	0	0	0	0	0	0	0	0	0	0	0	0	0	0
ENSG00000202031	5.28738441	1.44928644	0.89670929	2.86696105	2.13034708	3.71166755	5.10146044	0	3.44048915	5.77132021	3.16296907	2.75163427	3.82238174	3.79437111	2.45999806	5.1655024
ENSG00000202034	0	0	0	0	0	0	0	0	0	0	0	0	0	0	0	0
ENSG00000202035	0.51837102	0	0	0	0	0	0	0	0	0	0	0	0.29146702	0	0	0
ENSG00000202039	0	0	0	0	0	0	0	0	0	0	0	0	0	0	0	0
ENSG00000202041	0	0	0	0	0	0	0	0	0	0	0	0	0	0	0	0
ENSG00000202044	0	0	0	0	0	0	0	0	0	0	0	0	0	0	0	0
ENSG00000202046	0	0	0	0	0	0	0	0	0	0	0	0	0	0	0	0
ENSG00000202047	0	0	0	0	0	0	0	0	0	0	0	0	0	0	0	0
ENSG00000202048	0.73435895	0.70451424	0	0	0	0	0	0	0	0	4.10014509	8.40777138	10.3227902	0.61482865	4.38471877	8.37002703
ENSG00000202050	0	0	0	0	0	0	0	0	0	0	0	0	0	0	0	0
ENSG00000202051	0	0	0	0	0	0	0	0	0	0	0	0	0	0	0	0
ENSG00000202054	0	0	0	0	0	0	0	0	0	0	0	0	0	0	0	0.27392816
ENSG00000202056	0	0	0	0	0	0	0	0	0	0	0	0	0	0	0	0
ENSG00000202058	2.24200046	1.43392298	1.77440708	1.92481484	2.1077639	4.81992164	4.35910208	1.46347519	1.70200876	2.03933576	2.21668622	2.8196959	1.78587918	2.5027654	3.04240043	2.44889837
ENSG00000202059	0	0	0	0	0	0	0	0	0	0	0	0	0	0	0	0
ENSG00000202060	0	0	0	0	0	0	0	0	0	0	0	0	0	0	0	0
ENSG00000202063	0	0	0	0	0	0	0	0	0	0	0	0	0	0	0	0
ENSG00000202064	0	0	0	0	0	0	0	0	0	0	0	0	0	0	0	0
ENSG00000202067	0	0	0	0	0	0	0	0	0	0	0	0	0	0	0.23145143	0
ENSG00000202069	0	0	0	0	0	0	0	0	0	0	0	0	0	0	0	0
ENSG00000202070	0	0	0	0	0	0	0	0	0	0	0	0	0	0	0	0
ENSG00000202071	0	0	0	0	0.29240058	0.31840285	0.31827293	0	1.18056	0	0	0.53953613	0	0	0	0
ENSG00000202072	0	0	0	0	0	0	0	0	0	0	0	0	0	0	0	0
ENSG00000202074	0	0	0	0	0	0	0	0	0	0	0	0	0	0	0	0
ENSG00000202077	7.4090737	5.16942935	3.19845353	12.2348019	2.27960707	3.5166277	2.48131255	1.31899197	3.83494013	2.57320009	3.9956828	2.27842328	6.24890432	2.81959637	3.65604808	0.57577256
ENSG00000202078	1.8716405	0.89778806	1.6664509	1.26856684	0	0.86222366	1.14916245	1.37443633	1.59845735	1.53220891	1.30624091	1.7045522	1.57856473	0.39174923	1.77787471	4.79980311
ENSG00000202079	0	0	0	0	0	0	0	0	0	0	0	0	0	0	0	0
ENSG00000202081	0	0	0	0	0	0	0	0	0	0	0	0	0	0	0	0
ENSG00000202082	0	0	0	0	0	0	0	0	0	0	0	0	0	0	0	0
ENSG00000202089	0	0	0	0	0	0	0	0	0	0	0	0	0	0	0	0
ENSG00000202092	0	0	0	0	0	0	0	0	0	0	0	0	0	0	0	0
ENSG00000202093	0	0	0	0	0	0	0	0	0	0	0	0	0	0	0	0
ENSG00000202095	0	0	0	0	0	0	0	0	0	0	0	0	0	0	0	0
ENSG00000202099	0	0	0.60355433	0	0	0	0	0	0	0	0	0	0	0	0	0
ENSG00000202100	0	0	0	0	0	0	0	0	0	0	0	0	0	0	0	0
ENSG00000202103	0	0	0	0	0	0	0	0	0	0	0	0	0	0	0	0
ENSG00000202105	0	0	0	0.28107461	0	0	0	0	0	0	0	0	0	0.4339967	0	0
ENSG00000202107	0	0	0	0	0	0	0	0	0	0	0	0	0	0	0	0
ENSG00000202111	0	0.56994411	0	0.32213046	0	0	0	0	0	0	1.24386424	0.30917239	1.00212255	1.49216842	0.32247166	2.70850313
ENSG00000202112	0	0	0	0	0	0	0	0	0	0	0	0	0	0	0	0
ENSG00000202119	0.49414808	0	0.58663225	0	0	0.30352422	0.30340037	0	0	0	0	0	0	0	0	0.28160839
ENSG00000202124	0	0	0	0.30178537	0.31394589	0.34186412	0	0	0.63377432	0	0	0.28964571	0	0	0	0.31717997
ENSG00000202125	0.64089508	0.61484879	0	0.34751043	0.54227017	0	0.19675054	0	0	0	0	0.16676571	0.36035922	0	0.17393926	0.54785631
ENSG00000202129	0	0.65032084	0	0	0	0	0	0	0.77190462	0	0	0.35277362	0	0	0	0
ENSG00000202137	0	0.49730417	0	0	0.29240058	0	0	0.50755328	0.59028	0	0	0	0.58293403	0	0.28137233	0
ENSG00000202140	0	0	0.62769651	0	0	0	0	0	0.6020856	0	0	0	0	0	0	0
ENSG00000202141	2.33955062	0.44889403	0	0.50742673	0.26393681	0.28740789	1.43645306	0	0	0	0.65312046	0.24350746	0.26309412	0.39174923	0.2539821	0.26665573
ENSG00000202142	0	0	0	0	0	0	0	0	0	0	0.51251814	0	0.41291161	0	0	0.41850135
ENSG00000202144	0	0	0	0	0	0	0	0	0	0	0	0	0	0	0	0
ENSG00000202146	0	0	0	0	0	0	0	0	0	0	0	0	0	0	0	0
ENSG00000202147	0	0	0	0	0	0	0	0	0	0	0	0	0	0	0	0
ENSG00000202150	0.50840235	0	0	0.27566933	0.28677749	0.62455944	1.2486092	1.49337793	1.73678539	0.55493464	0.35482025	0.79374066	0.28586188	0	0.82788396	0.57946341
ENSG00000202151	0	0	0	0	0	0	0	0	0	0	0	0	0	0	0	0
ENSG00000202157	0	0	0	0	0	0	0	0	0	0	0	0	0	0	0	0
ENSG00000202159	0	0	0	0	0	0	0	0	0	0	0	0	0	0	0	0
ENSG00000202160	0	0	0	0	0	0	0	0	0	0	0	0	0	0	0	0
ENSG00000202164	0	0	0	0	0	0	0	0	0	0	0	0	0	0	0	0
ENSG00000202167	0	0	0	0	0	0	0	0	0	0	0	0	0	0	0	0
ENSG00000202169	0	0	0	0	0	0	0	0	0	0	0	0	0	0	0	0
ENSG00000202172	0	0	1.19561239	0.27304391	0	0	0	0	0	0	0	0	0	0	0	0
ENSG00000202174	0.34785424	0	0	0.37723172	0	0.42733015	0	0	1.58443579	0	0.24277175	0.36205714	0	0.29123463	0.18881564	0.19823748
ENSG00000202175	0	0	0	0	0	0	0	0	0	0	0	0	0	0	0	0
ENSG00000202177	0	0	0	0	0	0	0	0	0	0	0	0.25244351	0	0	0	0
ENSG00000202178	0	0	0	0	0	0	0	0	0	0	0	0	0	0	0	0
ENSG00000202182	0	0	0	0	0	0	0	0	0	0	0	0	0	0	0	0
ENSG00000202183	0	0	0	0	0	0	0	0	0	0	0	0	0	0	0	0
ENSG00000202184	0	0.48309548	0.5978062	0	0	0	0	0	0.57341486	0	0	0	0	0.42159679	0	0
ENSG00000202186	0	0	0	0	0	0	0	0	0	0	0	0	0	0	0	0
ENSG00000202187	0.3916581	0	0	0	0.22092488	0.24057104	0.24047288	0	0	0.4275052	0	0	0	0	0	0
ENSG00000202188	0	0	0	0	0	0	0	0	0	0	0	0	0	0	0	0
ENSG00000202189	0	0	0	0.44449009	0.46240092	0.75528119	1.50994601	0.4013212	0.46673302	0.44738916	0	0.21330498	0	0	0.22248045	0
ENSG00000202190	0	0	0	0	0	0	0	0	0	0	0	0	0	0	0	0
ENSG00000202191	0	0.7351453	0	0	0	0	0	0	0	0	0.53480153	2.39272545	1.29259286	0.64156033	0.8318834	2.18348531
ENSG00000202193	0	0	0	0	0	0	0	0	0	0	0	0	0	0	0	0
ENSG00000202195	0	0	0	0	0	0	0	0	0	0	0	0	0	0	0	0
ENSG00000202198	42406.2606	31336.7254	32543.3141	39321.1071	35085.2976	44769.621	41420.8184	42001.4634	44698.3984	42642.9076	28976.2209	41449.0059	42979.3529	34051.997	44905.8402	40814.7906
ENSG00000202199	0	0	0	0	0	0	0	0	0	0	0	0	0	0	0	0
ENSG00000202200	0	0	0	0	0	0	0	0	0	0	0	0	0	0	0	0
ENSG00000202205	0	0	0	0	0	0	0	0	0	0	0	0	0	0	0	0
ENSG00000202206	0	0	0	0	0	0	0	0	0	0	0	0	0	0	0	0
ENSG00000202211	0	0	0	0	0	0	0	0	0	0	0	0	0	0	0	0
ENSG00000202215	0	0	0	0	0	0	0	0.5883004	0	0	0	0	0	0	0	0
ENSG00000202216	0	0	0	0	0	0	0.23524521	0	0	0.41821161	0	0.59818136	0.43086429	0	0	0
ENSG00000202217	0	0.15464947	0	0	0.09092945	0	0	0	0	0	0	0	0	0	0	0
ENSG00000202220	0	0	0	0	0	0	0	0	0	0	0	0	0	0	0	0
ENSG00000202222	0	0	1.6664509	0.25371337	1.31968403	0.28740789	0	0.45814544	0	0	0.97968069	0	0.26309412	0	0.5079642	0.53331146
ENSG00000202224	0	0	0	0	0	0	0	0	0	0	0	0	0	0	0	0
ENSG00000202225	0	0	0	0	0	0	0	0	0	0	0	0	0	0	0	0
ENSG00000202227	0	0	0	0	0	0	0	0	0	0	0	0	0	0	0	0
ENSG00000202229	0	0	0	0	0	0	0	0	0	0	0	0	0	0	0	0
ENSG00000202231	0	0	0	0	0	0	0	0	0	0	0	0	0	0	0	0
ENSG00000202233	0	0	0	0	0	0	0	0	0	0	0	0	0	0	0	0
ENSG00000202237	0	0	0	0	0	0	0	0	0	0	0	0	0	0	0	0.28426507
ENSG00000202239	0	0.48309548	0	0.27304391	0	0	0	0	0	0	0	0	0	0	0.27333312	0
ENSG00000202240	0	0	0	0	0	0	0	0	0	0	0	0	0	0	0	0
ENSG00000202241	4.01352612	3.01336785	2.90024788	4.0686246	4.03570702	5.25207083	3.53566565	4.7840666	3.57674614	5.52370581	3.41002166	4.99471567	5.00399809	4.52903483	4.83068927	4.17672636
ENSG00000202242	0	0	0	0	0	0	0	0	0	0	0	0.26458022	0	0	0	0
ENSG00000202245	0	0	0	0	0	0	0	0	0	0	0	0	0	0	0	0
ENSG00000202248	0	0	0	0	0	0	0	0	0	0	0	0	0	0	0	0
ENSG00000202249	0	0	0	0	0	0	0	0	0	0	0	0	0	0	0	0
ENSG00000202250	0	0	0	0	0	0	0	0	0	0	0	0	0	0	0	0
ENSG00000202251	0	0	0	0	0	0	0	0	0	0.51529645	0	0	0	0	0	0
ENSG00000202252	0	0	0	0	0	0	0	0	0	0	0	0	0	0	0	0
ENSG00000202254	0	0	0	0	0	0	0	0	0	0	0	0	0	0	0	0
ENSG00000202255	0.47208789	0	0	0	0.53258677	0.28997403	0	0	0	0.51529645	0	0.24568163	0	0	0	0
ENSG00000202257	0	0	0	0	0	0	0	0	0	0	0	0	0	0	0	0
ENSG00000202259	0	0	0	0	0	0	0	0	0	0	0	0	0	0	0	0
ENSG00000202260	0	0	0	0	0	0	0	0	0	0	0	0	0	0	0	0
ENSG00000202261	0	0.61859787	0	0	0	0	0	0	0	0	0	0	0	0	0	0
ENSG00000202263	0	0	0	0	0	0	0	0	0.55237211	0	0.67708818	0	0	0.40612535	0.26330255	0
ENSG00000202264	0	0	0	0	0	0	0	0	0	0	0	0	0	0	0	0
ENSG00000202265	0	0	0	0	0	0	0	0	0	0	0	0	0	0	0	0
ENSG00000202268	0	0	0	0	0	0	0	0	0	0	0	0	0	0	0	0
ENSG00000202269	0	0	0	0	0	0	0	0	0	0	0	0	0	0	0	0
ENSG00000202270	0	0	0	0	0	0	0	0	0	0	0	0.36688457	0	0.59023551	0.38266637	0
ENSG00000202272	0	0	0	0	0	0	0	0	0	0	0	0	0	0	0.28415819	0
ENSG00000202273	0	0	0	0	0.57912348	0	0	0	0	0	0	0	0	0	0	0
ENSG00000202275	0	0	0	0	0	0	0	0	0	0	0	0	0	0	0	0
ENSG00000202276	0	0	0	0	0	0	0	0	0	0	0	0	0	0	0	0
ENSG00000202279	0	0	0	0	0	0	0	0	0	0	0	0	0	0	0.29285691	0
ENSG00000202281	0	0	0	0	0	0	0	0	0	0	0	0	0	0	0	0
ENSG00000202283	0	0	0	0	0	0	0	0	0	0	0	0	0	0	0	0
ENSG00000202285	0	0	0	0	0	0	0	0	0	0	0	0	0	0	0	0
ENSG00000202290	0	0	0	0	0	0	0	0	0	0	0	0	0	0	0	0
ENSG00000202293	0	0	0	0	0	0	0	0	0	0	0	0	0.41291161	0	0	0
ENSG00000202296	0	0	0	0	0	0	0	0	0	0	0	0.25716208	0	0	0	0
ENSG00000202297	0	0	0	0.28107461	0	0.31840285	0	0	0	0	0	0	0	0	0	0
ENSG00000202300	0	0	0	0	0	0	0	0	0	0	0	0	0	0	0	0
ENSG00000202304	0	0	0	0	0	0	0	0	0	0	0	0	0	0	0	0
ENSG00000202306	0	0	0	0	0	0	0	0	0	0	0	0	0	0	0	0
ENSG00000202308	0	0	0	0	0	0	0	0	0	0	0	0	0	0	0	0
ENSG00000202309	1.03674204	0	0	0	0.29240058	0.31840285	0.31827293	0	0	0	0	0	0.86799339	0	0	0
ENSG00000202310	0	0	0	0.53091871	0.2761561	0	0	0	0	0	0	0	0	0	0	0
ENSG00000202313	0	0	0	0	0	0	0	0	0	0	0	0	0	0	0	0
ENSG00000202314	3.62149617	4.86404354	4.29929114	3.92734391	6.53695544	4.00402493	3.55768101	4.25510425	6.59819836	2.37177543	3.53848138	1.1308086	2.4435317	3.03203171	1.17945113	5.77875839
ENSG00000202317	0	0	0	0	0	0	0	0	0	0	0	0	0	0	0	0
ENSG00000202318	0	0	0	0	0	0	0	0	0	0	0	0	0	0	0	0
ENSG00000202322	0	0	0	0	0	0	0	0	0	0	0	0	0	0	0	0
ENSG00000202324	0	0	0	0	0	0	0	0	0	0	0	0	0	0	0	0
ENSG00000202329	0	0	0	0	0	0	0	0	0	0	0	0	0	0	0	0
ENSG00000202331	0	0	0	0	0	0	0	0	0	0	0	0	0	0	0	0
ENSG00000202332	0	0	0	0	0	0	0	0	0	0	0	0	0	0	0	0
ENSG00000202334	0	0	0	0	0	0	0	0	0	0	0	0	0	0	0	0
ENSG00000202335	0	0	0	0	0	0	0	0	0	0	0	0	0	0	0	0
ENSG00000202336	0	0	0	0	0	0	0	0	0	0	0	0	0	0	0	0

ENSG00000202337	0.60242504	1.21054153	2.06206305	2.3361141	0.84011379	2.03352303	1.20948096	1.77024038	0.74458437	2.45533511	[illegible]
ENSG00000202339	0.22857971	0.9186379	0.67064057	0.295466	0.25501326	0.68585332	0	0	0	0	[illegible]
ENSG00000202341	0	0	0	0	0	0.71106851	0	0	0	0	[illegible]
ENSG00000202343	0.71621644	0.63964416	0.93392909	0.9257934	0.39952078	0.89541961	0.19172513	0.70153971	0.82621288	0.43246231	[illegible]
ENSG00000202344	0	0	0	0	0	0	0	0	0	0	[illegible]
ENSG00000202345	0	0	0	0	0	0	0	0	0	0	[illegible]
ENSG00000202347	0.1965228	0.13163409	0.76878309	0.762086	0.21924921	0.88449986	0.15782252	0.3849913	0.29147754	0.8899758	[illegible]
ENSG00000202350	0	0	0	0	0	0	0	0	0	0	[illegible]
ENSG00000202351	0.1039669	0.06963868	0	0	0.05799495	0.07798816	0.0834932	0	0	0	[illegible]
ENSG00000202354	2.84380057	2.32811663	4.6353098	6.5350121	2.11511	3.79236539	0.76126155	1.54751406	2.03081736	0.57237659	[illegible]
ENSG00000202356	0	0	0	0	0	0	0	0	0	0	[illegible]
ENSG00000202357	0.59137137	0	0.28917529	0.3822083	0	0.22180119	0	0	0	0	[illegible]
ENSG00000202358	0.30121252	0.20175692	0	0	0.16802276	1.35568202	1.93516954	1.18016026	0.74458437	1.63689007	[illegible]
ENSG00000202360	0	0	0	0	0	0	0	0	0	0	[illegible]
ENSG00000202361	0	0	0.31838492	0	0	0	0	0	0.16095056	0	[illegible]
ENSG00000202363	0	0	0	0	0	0	0	0	0	0	[illegible]
ENSG00000202368	0	0	0	0	0	0	0	0	0	0	[illegible]
ENSG00000202373	0	0	0	0	0	0	0	0	0	0	[illegible]
ENSG00000202374	0	0.14296682	0	0.2758987	0	0	0.34281977	0.20906812	0.10552388	0.19331925	[illegible]
ENSG00000202377	0	0	0	0	0	0	0	0	0	0	[illegible]
ENSG00000202379	0	0	0	0	0	0	0	0	0	0	[illegible]
ENSG00000202380	0	0	0	0	0	0	0	0	0	0	[illegible]
ENSG00000202382	0	0.21164697	0.61804131	0.8168765	0.70503667	0.47404567	0.76126155	0.30950281	0.62486688	0.85856489	[illegible]
ENSG00000202383	0	0	0	0	0	0	0	0	0	0	[illegible]
ENSG00000202385	0	0	0	0	0	0	0	0	0.39835264	0.24326005	[illegible]
ENSG00000202386	0	0	0	0	0	0	0	0	0	0	[illegible]
ENSG00000202388	0	0	0	0	0	0	0	0	0	0	[illegible]
ENSG00000202389	0	0	0	0	0	0	0	0	0	0	[illegible]
ENSG00000202392	0.1016711	0.27240367	0	0.1314218	0.05671431	0.07626602	0.16329901	0.49793828	0.25132658	0.46042912	[illegible]
ENSG00000202395	0	0	0.20534271	0	0	0.15750052	0	0.30849466	0.05190262	0	[illegible]
[illegible]	[illegible]	[illegible]	[illegible]	[illegible]	[illegible]	[illegible]	[illegible]	[illegible]	[illegible]	[illegible]	[illegible]

ENSG00000203442	0.32555293	1.09030255	1.27353966	1.0520379	0.99880195	0.97682139	0.78433008	1.75384927	0.8047528	1.17944268	0.41013884	0.8247876	0.96442933	0.70536852	0.84939678	0.62810671	0.73462469	[illegible]
ENSG00000203443	1.30221171	1.63545383	1.27353966	2.1040759	1.63440318	1.95364278	1.56866016	2.86993517	0.96570336	1.47430335	1.0253471	1.51211059	0.55110247	1.05805277	1.13252904	1.25621341	0.73462469	[illegible]
ENSG00000203446	0.07279444	0.08532803	0.14238332	0.0705715	0.1116673	0.16381477	2.13376755	1.67561432	0.54883141	0.46152105	1.23805886	0.44569268	0.84719169	0.67031915	0.74388165	0.91290043	0.52016903	[illegible]
ENSG00000203457	0.29433552	0.1971506	0.14392743	0	0.24627993	0.2207884	0.70912035	0.28830399	0.21827542	0.93305225	0.55621569	0.24856612	0.37369277	0.15943261	0.1279913	0	0.22139374	[illegible]
ENSG00000203462	0	0.06349409	0	0	0	0	0	0	0	0	0	0	0	0	0	0	0	[illegible]
ENSG00000203465	0.21486493	0.14391994	0	0	0.23971247	0.16117553	0	0	0.10622737	0	0	0.36290654	0	0	0	0	0	[illegible]
ENSG00000203469	0.89718762	1.5254897	1.3498975	1.248929	0.76995439	1.44954437	1.66271259	1.48720409	1.50128617	2.31279364	1.82586435	0.81595689	0.75938853	0.52336229	0.90032421	0.93207269	0.5191138	[illegible]
ENSG00000203471	0	0	0	0	0	0	0	0	0.09657034	0	0.2460833	0	0	0	0	0	0	[illegible]
ENSG00000203472	0.05624736	0.22605226	0.16502674	0.0727063	0.09412793	0.25315528	0.3161959	0.55094741	0.13904106	0.30566708	0.28344674	0.19000343	0.38086663	0.18280493	0.09783628	0.05426053	0.16923291	[illegible]
ENSG00000203473	0.1761188	0.35390148	0.17224102	0	0.29472844	0.13211109	0.28287314	0.34501953	0	0.63805915	0.66563517	0.14873219	0.44720611	0.3815928	0.30633982	0.33979543	0.79483982	[illegible]
ENSG00000203477	0	0.14685708	0.21442249	0	0	0	0	0	0	0	0.27621595	0.1851564	0	0	0.19068091	0	0	[illegible]
ENSG00000203478	0.21486493	0.14391994	0.42026809	0.277738	0.11985623	0	0.17255262	0	0.21245474	0	0	0	0.18186382	0	0	0	0	[illegible]
ENSG00000203479	0	0	0	0	0	0.17146333	0	0	0	0	0	0	0	0	0	0	0	[illegible]
ENSG00000203480	0	0	0	0	0	0	0.17255262	0.21046191	0	0	0	0	0	0	0	0	0	[illegible]
ENSG00000203482	0.85945973	0.28783987	0.84053618	0.555476	0.3595687	0.48352659	0.34510524	0.21046191	0.53113685	0	0.54138327	0.18145327	0	0.23277161	0.18686729	0.20727521	0	[illegible]
ENSG00000203483	0	0.14109798	0.20601377	0	0	0	0	0	0.10414448	0	0	0.17789536	0	0	0.18320323	0	0	[illegible]
ENSG00000203484	0	0	0	0	0	0	0	0.21046191	0	0	0	0	0	0	0	0	0	[illegible]
ENSG00000203485	0.55542722	0.57120365	0.5340531	0.4955578	0.67181804	0.62145259	2.63124219	2.68359088	2.25596016	2.7372049	2.42199432	1.85412098	5.39766404	4.04588231	4.42392103	4.12770413	3.48011075	[illegible]
ENSG00000203489	0.0366247	0.17172265	0.14327321	0.0946834	0.06129012	0.16483861	0.05882476	0.10762257	0.05432081	0.03317183	0.04614062	0.12371814	0.06199903	0.07935396	0.06370476	0.10599301	0	[illegible]
ENSG00000203492	0	0	0	0	0	0	0	0	0	0	0	0	0	0	0	0	0	[illegible]
ENSG00000203496	0	0	0	0	0	0	0	0	0.02191761	0	0	0	0	0	0	0	0	[illegible]
ENSG00000203497	1.49135043	2.16434867	1.49902821	1.6600023	1.20164347	1.61589862	3.79260895	2.39407188	2.60106863	5.17787463	3.70548317	3.25356444	0.28050974	0.44878845	0.39631238	0.43959396	0.37392208	[illegible]
ENSG00000203498	0	0	0	0	0	0.09352545	0.05006362	0	0.03082032	0	0	0	0.10553026	0.06753528	0	0.12027575	0	[illegible]
ENSG00000203499	0.03389433	0.07819908	0.02578181	0.0438124	0.03151163	0.03389997	0.04839054	0.0295109	0.03909981	0.0886856	0.02846722	0.04134539	0.04781416	0.04895874	0.03275309	0.02906406	0.01699646	[illegible]
ENSG00000203504	0.14005319	0.38696561	0.37666613	0.2489233	0.17578046	0.380833	0.0281183	0.20577482	0.19041299	0.09513701	0.0661658	0.17741222	0.0148178	0.13275947	0.1065783	0.16888257	0.13168177	[illegible]
ENSG00000203505	0.45715943	0.30621263	0.22354686	0	0.19125995	0.25719499	0.18356661	0.11194783	0.2825196	0.20702983	0.14398491	0.19303539	0.09673607	0.12381469	0	0	0.17193344	[illegible]
ENSG00000203506	0.13364094	0.23870618	0.2396138	0.1151643	0.17394478	0.15037109	0.26830918	0.19635355	0.23124825	0.28243047	0.1403032	0.15047956	0.09426252	0.09651898	0.09685589	0.15040703	0.10052224	[illegible]
ENSG00000203507	0.04225927	0.04717655	0.08265762	0	0.03928854	0.05283289	0.02262491	0.05519106	0.02785683	0.02551679	0.05323918	0.03568792	0	0	0	0	0.04238219	[illegible]
ENSG00000203511	0	0	0.26599246	0	0.07585838	0	0	0	0.13446503	0.12316965	0	0	0	0.1473238	0	0	0	[illegible]
ENSG00000203512	0.13429058	0.08994996	0	0.3471725	0.37455073	0.30220412	0.32353616	0.1315387	0.26556842	0.4865201	0.33836454	0	0.11366489	0	0	0.12954701	0.20202179	[illegible]
ENSG00000203513	1.31015202	1.05307271	1.79382721	0.6774098	0.9500799	2.063833	1.36779514	1.66829565	0.77727344	0.71198064	0.82527937	0.66385343	0.88714057	0.85160345	0.68366082	0.63193662	0.19709443	[illegible]
ENSG00000203518	0.14324329	0.57567975	0.42026809	0.3703174	0.07990416	1.07450353	0.34510524	0.42092382	0.35409123	0.51895478	0.18046109	0.48387539	0.24248509	0.15518107	0.62289097	0.5527339	0.43097982	[illegible]
ENSG00000203520	0	0	0	0	0	0	0	0	0	0.05094451	0	0	0	0	0	0.05426053	0	[illegible]
ENSG00000203523	0	0	0	0	0	0	0	0	0	0	0	0	0	0	0	0	0	[illegible]
ENSG00000203527	0.0330392	0.02213018	0.04846762	0	0.02764495	0.04956705	0.09286532	0.06472432	0.06533718	0.04488653	0.08324704	0.06975395	0.04194706	0.01789633	0.05746816	0.04780822	0.04970295	[illegible]
ENSG00000203531	0.03738949	0.05008815	0.10969875	0	0	0	0.06005312	0	0.14788033	0.10159353	0	0.15787698	0.03164684	0.081011	0.06503502	0.07213755	0.11249473	[illegible]
ENSG00000203542	0.2441647	0.24531807	0.23878869	0.3156114	0.13620027	0.82419305	0.29412378	0.23916126	0.36213876	0.2211455	0.15380207	0.2061969	0.20666343	0.1322566	0.42469839	0.23554001	0.36731234	[illegible]
ENSG00000203543	0.60355318	0.48512338	0.82636984	0.6241304	0.40400978	1.4487688	0.48469836	0.70942218	0.29839149	0.87464288	0.3041479	0.40776016	0.40868273	0.3923117	0.4199265	0.34934025	0.18159262	[illegible]
ENSG00000203546	0.03804415	0.15714256	0.15502708	0.2049021	0.0919613	0.18549613	0.08656486	0.06210759	0.06269567	0.04594327	0.05591702	0.03212826	0.11807015	0.10990593	0.07168822	0.07951735	0.08584833	[illegible]
ENSG00000203547	0.15131333	0.10135207	0.29596344	0.1955902	0.1688116	0.34051168	0.24303186	0.44463784	0.14961601	0	0.19062791	0.12778399	0.12807311	0.16392367	0	0	0.22763018	[illegible]
ENSG00000203560	0	0	0	0	0	0	0	0	0	0	0	0	0	0	0	0	0	[illegible]
ENSG00000203562	0	0	0	0	0	0	0	0.09437754	0	0.04363409	0	0	0	0	0	0	0	[illegible]
ENSG00000203563	1.74686936	2.2231535	2.39176961	1.9193277	1.07188501	1.76899971	1.47301015	1.8821797	0.99318273	2.84792256	1.65055875	1.77027582	1.10892571	1.7032069	1.21539702	1.76942255	2.10234057	[illegible]
ENSG00000203565	0.28521894	0.42984937	0.1394695	0.0921697	0.35797769	0.37441218	1.0879977	0.62859199	0.31727201	1.03331704	0.53898777	0.30108397	0.18105911	0.23174165	0.06201348	0.27514409	0.32180462	[illegible]
ENSG00000203572	0	0	0	0	0	0	0	0.18143268	0	0	0	0	0	0	0	0	0.27865074	[illegible]
ENSG00000203573	0	0.73179629	0.3561594	0	0.5078654	0.54635773	0.14623103	0	0.36009278	0.16492207	0.22939969	0.61509583	0.15412188	0	0	0.35131392	0	[illegible]
ENSG00000203574	0	0	0	0	0	0	0	0	0	0	0	0	0	0	0	0	0	[illegible]
ENSG00000203575	1.66705551	1.36475802	1.99265042	1.6760053	1.75651377	1.80627748	0.89251354	2.54005756	1.37362978	2.5164833	0.93341943	1.25140187	0.78389576	0.40133036	1.12764745	0.17868553	0.83595223	[illegible]
ENSG00000203576	0.20270277	0.13577353	0	0	0.33921575	0.45615716	0.32557098	0.39709795	0	0	0	0.34236466	0	0.43919172	0.1762899	0.39108531	0	[illegible]
ENSG00000203578	0	0.36589815	0.1780797	0.7061136	0.20314616	0.27317886	0.14623103	0	0.36009278	0.98953242	0	0.46132188	0.30824376	0	0.15836211	0	0	[illegible]
ENSG00000203581	0	0	0	0	0	0	0.02701763	0	0	0	0.02119193	0	0.05695109	0	0	0	0	[illegible]
ENSG00000203583	0	0	0	0	0	0	0	0	0	0	0	0	0	0	0	0	0	[illegible]
ENSG00000203585	0.01386225	0.03714063	0.02711407	0.0179186	0.00773266	0.02079684	0	0	0.00685338	0	0	0	0	0	0	0.01337259	0.02085386	[illegible]
ENSG00000203588	0	0.11949072	0.05815518	0	0.09951163	0	0.09550883	0.11649183	0.05879744	0.10771663	0	0	0.05033131	0	0	0.05736399	0.08945614	[illegible]
ENSG00000203589	0.17052772	0.3997776	0.3335461	0.3306405	0.28537198	0.51166835	0.38801515	0.47326091	0.29507603	0.66928692	0.35805772	0.45603335	0.16839242	0.18473937	0.19774317	0.08225207	0.21377967	[illegible]
ENSG00000203593	0.1024793	0.37753243	0.20044583	0.0662332	0.11433027	0.15374454	0.24689564	0.15056894	0.10132976	0.13922674	0.06455285	0.08654369	0.13010925	0.16652977	0.08912589	0.0988594	0.15416607	[illegible]
ENSG00000203594	0	0.25249112	0	0	0.10513705	0.14138204	0.15136195	0	0.0931819	0.17070881	0.2374488	0	0	0	0.16391868	0.18182036	0	[illegible]
ENSG00000203601	0.05709431	0.11472803	0.13959303	0.0738011	0.04777264	0.19272539	0.18340402	0.22369734	0.11290774	0.3619813	0.17982172	0.09643221	0.12081299	0.06185251	0.04965473	0.1376939	0.12883586	[illegible]
ENSG00000203602	1.10898029	1.0213673	0.61006658	0.4479645	0.65727612	0.46792896	0.44529708	0.3394547	0.30840204	0.37666073	0.43659941	0.46826651	0.23466299	0.22526285	0.24111909	0.40117783	0.31280793	[illegible]
ENSG00000203605	0.3132903	0.31477021	0.30639229	0.2531027	0.17476	0.35251027	0.22014611	0.30687035	0.32913705	0.3192234	0.29601758	0.19843007	0.13258601	0.12727488	0.17029219	0.18888993	0.29456397	[illegible]
ENSG00000203606	0	0	0	0	0	0	0	0	0	0	0	0	0	0	0	0	0	[illegible]
ENSG00000203614	0.05641145	0.06297547	0.03677959	0.0972245	0.01048917	0.02821042	0	0.03683697	0.06507511	0.05109313	0	0.01587981	0	0	0	0	0	[illegible]
ENSG00000203615	0.2620304	0	0.76878309	0.3387049	0	0	0.42086004	0.51332174	0.38863672	0.71198064	0	0.22128448	0.22178514	0	0	0.25277465	0.39418886	[illegible]
ENSG00000203616	0	0	0	0	0	0	0	0	0	0	0	0	0	0	0	0	0	[illegible]
ENSG00000203618	0	0	0	0	0	0	0	0	0	0	0	0	0	0	0	0	0	[illegible]
ENSG00000203620	0.08567941	0.200863	0.30724074	0.2768766	0.16727831	0.31063959	0.25229226	0.1258855	0.1976761	0.27160626	0.12593098	0.1447124	0.33842624	0.18563974	0.19870691	0.11020392	0.21482157	[illegible]
ENSG00000203630	0.04853876	0.08128008	0.07120506	0.062742	0.05415191	0.07282027	0.07796052	0.09508821	0.10798716	0.13188798	0.12230044	0.04099095	0.02054185	0.1051679	0.12664199	0.09364844	0.03650996	[illegible]
ENSG00000203632	0.17468694	0.14040969	0.08542034	0.067741	0.12667732	0.26207403	0.23848736	0.25666087	0.31090937	0.55376272	0.1320447	0.29504597	0.10349973	0.22709425	0.13673216	0.08425822	0.23651331	[illegible]
ENSG00000203635	0.21604335	0.38150623	0.26891011	0.1777117	0.2191156	0.23572289	0.22081688	0.15390268	0.24274993	0.2312527	0.22268964	0.23220711	0.11636624	0.12766267	0.11956774	0.07578618	0.17727689	[illegible]
ENSG00000203643	0	0	0.0196387	0.0519136	0	0	0	0.01966934	0.00992779	0	0	0	0	0.02175436	0	0	0	[illegible]
ENSG00000203644	14.727497	17.1798468	15.7252629	11.495779	15.3590604	11.2075699	5.48511632	5.26154781	6.48971845	3.83416506	6.67765788	6.66930897	5.17890338	4.39337145	3.49602715	4.56417273	4.17422504	[illegible]
ENSG00000203645	0.01575256	0.02110263	0.06162289	0.020362	0.02636134	0.05908194	0.06325243	0.04628928	0.04672758	0.01426745	0.09922714	0.07981815	0.09333187	0.03413073	0	0.01519613	0.02369757	[illegible]
ENSG00000203647	0	0	0.14694688	0	0	0.05635508	0.18099925	0.07358808	0.03714244	0	0.09464742	0	0	0.16277735	0.26135286	0.07247385	0.11301918	[illegible]
ENSG00000203648	0	0	0	0	0	0	0	0.04312744	0.04353581	0.0398787	0.0554696	0	0.03726718	0	0	0	0	[illegible]
ENSG00000203650	0.13550667	0.20945657	0.16310534	0.1077897	0.069774	0.20329384	0.19253122	0.24503974	0.18551999	0.16993587	0.24950555	0.15844885	0.0882263	0.09033827	0.10878432	0.14077553	0.06272345	[illegible]
ENSG00000203652	0	0.02705262	0	0	0	0	0.0324347	0.13846178	0.05990265	0.10974138	0.07632283	0.05116164	0.10255478	0.08750812	0.01756272	0.11688452	0.03037922	[illegible]
ENSG00000203659	0.21168959	0.1772413	0	0.2052251	0.23616992	0.27788884	0.29750451	0.20735164	0.31397252	0.38346412	0.06667282	0.04469292	0.17917617	0.11466582	0.23013213	0.15315902	0.0796145	[illegible]
ENSG00000203661	0	0	0	0	0	0	0	0	0	0	0	0	0	0	0	0	0	[illegible]
ENSG00000203663	0.02842129	0.05711109	0	0	0.015854	0.02131951	0.02282442	0.05567775	0.01405124	0.10296722	0.03580577	0.02400176	0	0	0	0	0	[illegible]
ENSG00000203664	0	0	0	0	0	0	0	0	0	0	0	0	0	0	0	0	0	[illegible]
ENSG00000203666	2.3783235	2.74308013	2.48674657	1.7629304	1.91758857	1.79665262	0.68266861	0.72101901	0.73893017	0.44503433	0.6708056	0.81570351	0.32575456	0.36431705	0.2973455	0.35144629	0.337269	[illegible]
ENSG00000203667	16.9356492	16.341469	18.1681756	10.97995	17.7633253	12.4092773	6.24769275	5.97078828	6.12527303	5.32234836	6.15518904	6.95537676	1.79368429	1.1459722	1.26766176	1.51190317	1.64455937	[illegible]
ENSG00000203668	7.49963004	6.71002815	6.26572337	3.8317487	8.12814242	4.39600677	11.9358239	14.5714694	17.0799743	8.30617256	10.4348986	14.8395346	7.11088036	4.70114463	5.78977071	7.15565107	6.33637145	[illegible]
ENSG00000203684	0.34089148	0.56045745	0.30307795	0.3204669	0.32845218	0.65090118	0.54752273	0.51603642	0.49028017	0.84205403	0.3123365	0.60193633	0.15738215	0.20143697	0.29647215	0.17937278	0.09324083	[illegible]
ENSG00000203685	0.27534492	1.0955716	0.35971304	1.0040003	0.32667223	0.67203568	0	0	0	0	0	0	0.00347843	0.00667818	0.00536119	0	0.00309118	[illegible]
ENSG00000203688	0	0	0	0	0	0	0	0	0	0	0.01587945	0	0	0	0	0	0.01896176	[illegible]
ENSG00000203690	0	0.0024149	0	0	0	0.00135222	0	0.00353144	0	0	0	0	0	0	0	0	0.00271185	[illegible]
ENSG00000203691	0	0	0	0	0	0	0	0	0	0	0	0	0	0	0	0	0	[illegible]
ENSG00000203697	0.0057064	0.00382224	0.00558076	0.0036881	0.00318315	0.01070128	0	0	0	0.00516841	0	0	0.00241498	0.00309098	0	0.00275242	0	[illegible]
ENSG00000203705	1.05393981	1.09689254	0.99801249	1.3575349	0.92385868	1.12383191	0.75831994	0.74649101	0.68831219	0.69699287	0.81024833	0.67813438	0.35399411	0.40274227	0.37181623	0.60070243	0.53968794	[illegible]
ENSG00000203706	0	0	0	0	0	0	0	3.60565152	4.17882467	3.6657653	2.24417944	4.06037452	2.64313435	0.48882471	0.50456274	0.51847572	0.44929598	[illegible]
ENSG00000203709	0.20591926	0.33916717	0.29217381	0.2749017	0.16005938	0.36210684	0.10030553	0.1256489	0.059247	0.12382758	0.11269958	0.07982233	0.17286349	0.16639604	0.11743428	0.1123487	0.11680129	[illegible]
ENSG00000203710	0.00060778	0.0008142	0.00297197	0.0007856	0.00067806	0.00045591	0.00097618	0.00178596	0.00180287	0.00220191	0.00076569	0.0015398	0	0.00197528	0.00158574	0.00058631	0	[illegible]
ENSG00000203711	0	0	0	0	0	0	0	0.05602358	0.05466543	0.01379576	0.01263689	0.07030952	0.07069608	0.01180934	0.03023008	0.02426848	0.04037829	[illegible]
ENSG00000203721	0.04443438	0.07936761	0.2027948	0.0765822	0.0165243	0.17776713	0.02378942	0.02901589	0.04393595	0.12073569	0.01865981	0	0	0.11232086	0.01288148	0.05715309	0.02228181	[illegible]
ENSG00000203722	0	0.01397733	0.02040797	0.0134868	0	0.00782659	0.24299252	0.25549763	0.30434193	0.37800202	0.40748336	0.46699693	0.45922233	0.38431052	0.4083374	0.32208515	0.45518668	[illegible]
ENSG00000203724	0.0243611	0	0	0	0.01358914	0	1.07600839	0.64427116	0.73467909	0.44128808	0.3989786	0.47317746	0.47424805	0.34308741	0.46610889	0.44651123	0.40312738	[illegible]
ENSG00000203727	0.00994438	0.01998271	0.05348984	0.0257085	0.02496234	0.02237858	3.57776486	3.55044895	2.05506204	0.53140425	2.88147528	1.57882821	0.06733619	0.03770598	0.08216164	0.05276213	0.06731982	[illegible]
ENSG00000203729	0	0	0	0	0	0	0.03497688	0	0	0	0.10973985	0	0	0	0	0	0	[illegible]
ENSG00000203730	0	0.0086352	0.01260804	0.0333286	0.02157412	0.0290116	0.02070631	0	0.01274728	0	0	0.03266159	0.03273549	0	0.03363611	0	0	[illegible]
ENSG00000203733	0	0	0	0	0	0	0	0	0	0	0	0	0	0	0	0	0	[illegible]
ENSG00000203734	0	0.00351453	0.00769722	0.0033912	0.00292689	0.00787182	0.00421374	0.00513949	0.00389111	0.01188083	0.00991544	0.00443109	0.00666168	0.01421072	0.01368991	0.00253083	0	[illegible]
ENSG00000203737	0.13137122	0.20532056	0.14989181	0.1698126	0.18320416	0.29563446	0.01758349	0.15012568	0.0974232	0.07932393	0.11033626	0.1109429	0.01853232	0	0.01904218	0.02112179	0.06587667	[illegible]
ENSG00000203739	0.36430649	0.51853881	0.4453565	0.7799425	0.2540224	0.4355326	0.20113869	0.25647955	0.28705029	0.18560287	0.25816581	0.22112815	0.15417632	0.19733376	0.20792369	0.2306312	0.30827769	[illegible]
ENSG00000203740	0	0	0	0	0	0	0	0	0	0	0	0	0	0	0	0	0	[illegible]
ENSG00000203747	0	0	0	0	0	0	0	0	0	0	0	0	0	0	0	0	0	[illegible]
ENSG00000203756	0	0	0.0566399	0	0.06461252	0.02172177	0	0	0.02863271	0.0262275	0	0.02445462	0.02450995	0	0.05036854	0.02793466	0.04356265	[illegible]
ENSG00000203757	0	0	0	0	0	0	0	0	0	0	0	0	0	0	0	0	0	[illegible]
ENSG00000203758	0	0	0	0	0	0	0	0	0	0	0	0	0	0	0	0	0	[illegible]
ENSG00000203760	7.94038911	4.3276332	7.21610808	4.5267585	8.77506415	4.9026955	3.00788991	3.85215005	7.42542277	3.13792746	3.58615298	8.9988592	9.4154945	7.14139513	9.41394201	11.2008105	10.1703473	[illegible]
ENSG00000203761	0.25349234	0.51423079	0.47457239	0.5898032	0.27068655	0.49439235	0.20357331	0.24120354	0.29003653	0.3935893	0.2463598	0.19572488	0.20842819	0.19615585	0.18266803	0.23755137	0.16343336	[illegible]
ENSG00000203772	0.64971063	0.65791971	0.66042128	0.2909636	0.44803402	0.36072618	0.0041084	0.005011	0	0	0.00644504	0.00864063	0.06062127	0.12192799	0.09788287	0.12831323	0.10774495	[illegible]
ENSG00000203778	9.83001933	9.08606289	10.0149845	11.831056	7.70994968	9.58278576	2.93355931	2.72374299	2.46544898	2.59291517	3.83932261	2.72959792	2.37100391	3.27925621	3.06091123	2.05889419	2.26912726	[illegible]
ENSG00000203780	0.03255803	0.0381638	0.04510833	0.0245496	0.05902508	0.03052824	0.1590581	0.13553612	0.09791983	0.13515585	0.21192291	0.13976744	0.09185814	0.12932845	0.14393768	0.09684127	0.12652935	[illegible]
ENSG00000203781	0	0	0	0	0	0	0	0	0	0	0	0	0	0	0	0	0	[illegible]
ENSG00000203782	0	0	0	0	0	0	0	0	0	0	0	0	0	0	0	0	0	[illegible]
ENSG00000203783	0	0	0	0.033898	0	0	0	0	0	0	0	0	0.93225553	0.02840988	0.04561447	0.17708623	0	[illegible]
ENSG00000203784	0	0	0	0	0	0	0	0	0	0	0	0	0	0	0	0	0	[illegible]
ENSG00000203785	0	0	0	0	0	0	0	0	0	0	0	0	0	0	0	0	0	[illegible]
ENSG00000203786	0.00642667	0	0	0	0	0	0	0	0	0	0	0	0.01087919	0.01392452	0.0111785	0.06819623	0.10634846	[illegible]
ENSG00000203791	1.82151932	1.45524581	1.64811015	1.9540968	1.67734214	1.31679354	1.39647543	1.63449851	1.7091947	1.54878372	1.68596866	1.7998825	0.86701704	0.92625381	0.95553056	1.54201283	1.44157032	[illegible]
ENSG00000203795	0	0	0	0	0	0	0	0	0	0	0	0	0	0	0	0	0	[illegible]
ENSG00000203797	1.10354122	1.01503167	1.14637347	1.3104284	0.83942562	1.08524152	0.53003958	0.52753395	0.23232886	0.05738769	0.37916341	0.20957496	0.37987609	0.62349538	0.53727408	0.29033471	0.36538672	[illegible]
ENSG00000203799	0.02256458	0.00755706	0.01838979	0.0243061	0.025174	0.00846313	0.00302017	0.0036837	0.00185929	0	0.00473789	0	0.00318315	0.00814836	0.00981217	0.00362792	0.01131511	[illegible]
ENSG00000203801	0.00475646	0.01592975	0.00930346	0.0061483	0.00265325	0.02140761	0.00381979	0.00465899	0.0094062	0	0.00599229	0	0.00402591	0.00515285	0	0	0	[illegible]
ENSG00000203804	0.3288749	0.52868548	0.57894073	0.6801747	0.33021615	0.39471558	0.15846669	0.45098981	0.42274157	0.41701723	0.91151265	0.38882844	0.33403558	0.14251323	0.11440855	0	0	[illegible]
ENSG00000203805	0.01670151	0	0.036751	0.0053972	0.0326076	0.00313205	0.36549233	0.45805935	1.0919992	0.32522914	0.35243567	0.69816844	0.01060224	0.0180934	0.02541918	0.14500404	0.10678182	[illegible]
ENSG00000203808	0.13126421	0.12895351	0.17116539	0.1583627	0.06834051	0.06564303	0.10541498	0.12857434	0.14277097	0.1743705	0.09922175	0.13302303	0.05925511	0.0853222	0.08371736	0.05909285	0.15797522	[illegible]
ENSG00000203809	0	0	0.05481758	0	0	0	0.27008236	0.10980622	0.05542298	0.05076732	0.14123042	0.14200691	0	0.06072303	0	0	0	[illegible]
ENSG00000203811	0	0	0.01903388	0.0251574	0	0.02919847	0	0	0	0	0	0	0	0	0	0	0	[illegible]
ENSG00000203812	0.11741253	0.11796716	0.05741367	0.1517694	0.06549521	0.22018515	0	0	0	0.0531716	0	0.09915479	0	0	0.10211327	0	0	[illegible]
ENSG00000203813	377.898809	255.359	322.931156	262.207	310.23253	215.389116	62.8740881	105.186461	176.454217	31.3512321	59.4892081	153.583123	299.844603	124.23508	142.580139	214.483833	122.699407	[illegible]
ENSG00000203814	39.8690338	29.6819136	34.2402091	23.466028	33.556251	19.2564816	11.6586207	18.1776506	31.3293316	8.65523521	10.8434492	27.7813951	17.2823647	6.44363393	7.33031899	15.4459313	7.23744531	[illegible]
ENSG00000203815	0	0	0	0	0	0	2.37532876	3.84625805	1.84049003	2.17086502	2.12013227	1.50732543	0.77694985	0.77344997	1.06443394	0.34436546	0.76717135	[illegible]
ENSG00000203817	0.57996961	0.2774806	0.52668559	0.6158073	0.71636438	0.4661246	0.066537	0.12173247	0.31745326	0.13132291	0.15656971	0.34984564	0.10519115	0.08975769	0.19815619	0.25976007	0.3427619	[illegible]
ENSG00000203818	0	0	0	0	0	0	0	0	0	0	0	0	0	0	0	0	0	[illegible]
ENSG00000203819	1.16693885	1.1166202	1.14124524	1.2929183	0.68194064	1.29218312	0.62475948	0.43543844	0.27472596	0.20131866	0.4900452	0	0.23516873	0.30099777	0.24163874	0.16081698	0.50157134	[illegible]
ENSG00000203825	0.01242473	0.04161139	0	0.0160604	0	0.0093201	0.00997798	0.01217012	0.00614268	0.03376007	0	0	0.02103282	0.0269203	0.02161148	0.07191507	0.0186913	[illegible]
ENSG00000203827	0.02524523	0.0281276	0.04114896	0.0978972	0.04694108	0.05049886	0	0	0	0	0	0.00710652	0.01424521	0.00911638	0.00731856	0	0.01265933	[illegible]
ENSG00000203832	0.02888487	0.07255329	0.06497244	0.076541	0.04108712	0.06608514	0.05451228	0.07780565	0.07211618	0.08371739	0.08915502	0.09513368	0.07456775	0.07823029	0.07536322	0.08916661	0.07604333	[illegible]
ENSG00000203835	0	0	0	0	0	0	0	0	0	0	0	0	0	0	0	0	0	[illegible]
ENSG00000203836	0.17201864	0.25238844	0.15701573	0.1842363	0.10143884	0.25192637	0.15656302	0.18293594	0.14255083	0.20328345	0.16098674	0.16878945	0.14005169	0.12246155	0.11968321	0.15171875	0.11583418	[illegible]
ENSG00000203837	0	0	0	0	0	0	0.19791507	0.12069821	0.04061369	0.12400682	0.25873245	0.30062352	1.61081588	2.78851292	2.32194915	2.61515455	1.23581723	[illegible]
ENSG00000203843	0	0.05164591	0	0	0.04301061	0.11567622	0	0	0	0	0	0	0	0	0	0	0	[illegible]
ENSG00000203849	0.38662405	0.67204298	0.53121169	0.1563656	0.13892668	0.2633277	0.00190483	0.00464664	0.00351798	0	0.00298821	0.00400618	0.00401524	0	0.00412571	0	0	[illegible]
ENSG00000203852	0.23354884	0.11732604	0.17130493	0.2012594	0.23884394	0.10219463	0	0.03812716	0.01924409	0	0	0.01643598	0.01647317	0	0.01692639	0	0	[illegible]
ENSG00000203855	0	0	0	0	0	0	0	0	0.00822618	0	0	0	0	0	0	0	0	[illegible]
ENSG00000203857	0	0	0	0	0.00275321	0	0	0	0	0	0	0	0	0	0	0	0	[illegible]
ENSG00000203858	0	0	0	0	0	0.02710351	0	0	0	0	0	0	0	0	0	0	0	[illegible]
ENSG00000203859	0.00532283	0	0	0	0	0	0	0	0	0	0	0	0	0.00576643	0	0	0	[illegible]
ENSG00000203863	0.05283564	0.06193276	0.05167231	0.1024443	0.05157748	0.11889998	0.1485084	0.16819702	0.0914252	0.11963609	0.06656352	0.0780844	0.04472061	0.04292919	0.03446323	0.0891963	0.05961299	[illegible]
ENSG00000203864	0	0	0	0	0	0	0	0	0.04128007	0	0	0	0.07067247	0	0	0	0	[illegible]
ENSG00000203865	0.2307195	0.51116938	0.24299642	0.3097024	0.13860027	0.37276279	0.31355927	0.30421945	0.28955148	0.44204745	0.32420391	0.34472125	0.30043582	0.49028161	0.40131192	0.23113012	0.29368806	[illegible]
ENSG00000203867	2.51077072	2.83267508	2.72641549	2.6834007	1.98738875	2.78300038	3.00760846	3.29397738	2.81720394	2.93150728	2.81853873	2.68300506	0.09794646	0.11776607	0.11588984	0.07780432	0.06330353	[illegible]
ENSG00000203870	0	0	0	0	0	0	0	0	0	0	0	0	0	0	0	0	0	[illegible]
ENSG00000203871	0.06166404	0.09637496	0.10051054	0.079708	0.05732919	0.06167431	0.03301389	0.14093432	0.04064823	0.05585052	0.05179049	0	0.05219306	0.02226769	0.01787634	0	0.0309217	[illegible]
ENSG00000203872	0.1832778	0.27735203	0.23235125	0.166713	0.10602278	0.24441108	0.19624771	0.24601171	0.15437423	0.2090356	0.15393164	0.19490558	0.13789169	0.1397218	0.04132491	0.05238632	0.11232888	[illegible]
ENSG00000203875	2.89182561	2.81860246	2.97739644	4.610885	2.58567691	4.64753302	10.6282853	10.8519423	11.9392623	5.02760477	10.4393152	9.97477498	5.76720128	6.71202228	7.18669308	8.40583427	7.41511793	[illegible]
ENSG00000203876	0.01535481	0.03085468	0.0600669	0.0595436	0.03426095	0.03455407	0.2712833	0.24064249	0.08350413	0.08344318	0.2708206	0.15560547	0.01299646	0.01663446	0.05341609	0.02962485	0.0230992	[illegible]
ENSG00000203877	0.08159428	0.10930628	0.07979774	0	0.0606867	0.0612059	0.34947366	0.34632973	0.28237655	0.27097322	0.68529528	1.10250089	0.57551841	0.20625333	0.07096226	0.4197979	0.12274742	[illegible]
ENSG00000203878	0.00543595	0.02184651	0.00531626	0.0140532	0	0.00815528	0.02182737	0	0.01343743	0.00492346	0	0.00459065	0.02300521	0.01177795	0.00945525	0.0157318	0.00817764	[illegible]
ENSG00000203879	17.2779365	17.3806606	16.021538	15.693119	15.6485679	16.0693225	4.73609697	5.11251842	4.58119695	5.28783583	4.88178473	4.44655133	8.1631809	7.27031972	8.0173775	7.75439711	6.94123691	[illegible]
ENSG00000203880	6.56162018	6.94968136	6.47753389	4.7835713	6.53510774	4.76609801	4.20004025	4.0542714	3.48110191	4.11051854	4.09434279	3.19475489	1.35883942	1.35123184	1.45708515	1.60628693	1.89804238	[illegible]
ENSG00000203883	0.18705595	0.21299817	0.21952483	0.2417917	0.21912196	0.12628379	0.09013195	0.03664456	0.02774365	0.01694208	0.04713145	0	0	0	0	0.03608971	0	[illegible]
ENSG00000203886	0.19692305	0.17586958	0.06419574	0.5090921	0.10984787	0.64010648	0.47443184	0.1928877	0.12980941	0.41616791	0.24808806	0.05543379	0.22223684	0	0	0.06332237	0	[illegible]
ENSG00000203896	0.13905615	0.14669867	0.07139707	0.1348097	0.06981163	0.17732611	0.45506542	0.42224049	0.40561416	0.4848933	0.34161278	0.37578501	0.45313927	0.22220135	0.39606755	0.20792358	0.13074434	[illegible]
ENSG00000203897	0.1320891	0.13271306	0.03229519	0	0.03684106	0.02477083	0.05303871	0	0.01632593	0.08972707	0.0416022	0.02788729	0	0	0	0	0	[illegible]
ENSG00000203900	0	0	0	0	0	0	0	0	0	0	0	0	0	0	0	0	0	[illegible]
ENSG00000203902	0	0	0	0	0	0	0	0	0	0	0	0	0	0	0	0	0	[illegible]
ENSG00000203907	0	0	0	0	0	0	0	0	0	0	0	0.01111392	0.01113907	0	0.01144553	0	0.01979797	[illegible]
ENSG00000203908	0	0	0.06192555	0	0	0.02374885	0.02542524	0	0	0	0	0	0	0.10289511	0	0	0	[illegible]
ENSG00000203909	0	0	0	0	0	0	0	0	0	0	0	0	0	0	0	0	0	[illegible]
ENSG00000203910	0	0	0	0	0	0.01421301	0.01521628	0	0	0.0171612	0	0	0	0.0205266	0	0	0.02850396	[illegible]
ENSG00000203914	0.02200733	0.02211129	0.04304555	0.0142235	0.03682848	0.0330165	0	0.01077818	0	0	0.02772533	0.01858518	0.00931361	0.02384141	0.0191397	0.01061498	0	[illegible]
ENSG00000203923	0	0	0.02470228	0	0	0	0	0	0	0	0	0	0.02137898	0	0	0.02436621	0	[illegible]
ENSG00000203924	0	0	0	0	0	0	0	0	0	0	0	0	0	0	0	0	0	[illegible]
ENSG00000203926	0	0	0	0	0	0	0	0	0	0	0	0	0	0	0	0	0	[illegible]
ENSG00000203930	0.77131001	0.90147653	0.91596891	1.1903058	0.53562126	1.73573647	1.06186226	0.90197962	1.28406711	1.40431444	1.94342712	1.68159991	0.05329343	0.0511586	0.10267434	0.015185	0.08288073	[illegible]
ENSG00000203933	0	0	0	0	0	0	0	0	0	0	0	0	0	0	0	0	0	[illegible]
ENSG00000203942	0.02597078	0.01739564	0	0	0	0.01948133	0	0.05087717	0.01283973	0.04704465	0.03271857	0	0	0.02813517	0	0	0	[illegible]
ENSG00000203943	0.44901092	0.51305202	0.36808975	0.5249197	0.22652607	0.50769796	0.10075291	0.11318634	0.09956776	0.14054361	0.09150608	0.10594997	0.03632805	0.0178835	0.02297078	0.02229451	0.02483366	[illegible]

ENSG00000203950	2.39876003	2.48721416	2.00812826	3.1874964	2.73503432	2.00809643	7.28943627	8.17662386	7.00645245	8.03866204	6.51545658	6.74169938	4.30430884	4.61523943	4.90672675	3.64690833	4.08494192
ENSG00000203952	0.38604738	0.62897998	0.66325896	0.3776302	0.17460442	0.28175716	0.14244389	0.08175924	0.13927512	0.12285066	0.14459087	0.11454642	0.0088312	0.01130325	0	0.04026064	0.01569609
ENSG00000203963	0	0	0	0	0	0	0	0	0	0	0	0	0	0	0	0	0
ENSG00000203965	1.25321151	1.68136265	1.41700619	1.2696688	0.86071443	1.31270355	1.26331727	1.33443272	1.12193666	1.18619101	1.24693893	1.21088912	1.45571749	1.31688282	1.5645008	1.4667069	1.80036232
ENSG00000203970	0	0	0	0	0	0	0	0	0	0	0	0	0	0	0	0	0
ENSG00000203971	0	0	0	0	0	0	0	0	0	0	0	0	0	0	0	0	0
ENSG00000203972	0	0	0.0187174	0	0	0	0	0	0	0	0	0	0	0	0	0	0
ENSG00000203985	0	0	0	0	0	0	0	0	0	0	0	0	0	0	0	0	0
ENSG00000203987	0	0	0	0	0	0	0.01405152	0	0	0.00792378	0.01102165	0	0	0	0	0	0
ENSG00000203989	0	0	0	0	0	0	0	0	0	0	0	0	0	0	0	0	0
ENSG00000203993	3.99316578	3.8939157	3.82736205	4.7204679	3.73783912	3.56713932	4.80564826	5.18168668	3.85838205	4.84287573	4.47171497	4.28493605	4.08278811	3.6850712	3.720196	3.35825354	3.09771497
ENSG00000203995	0.29122105	0.22586413	0.34476873	0.3615778	0.50872281	0.46277363	1.19397959	1.33618667	1.61027103	1.17306238	1.23582913	1.65359568	0.11675955	0.0788728	0.12663697	0.09610906	0.12681905
ENSG00000203999	0.02999045	0	0.06843696	0.0775323	0.14498738	0.0974852	0.39338143	0.44063831	0.39538723	0.22635861	0.42820327	0.32924989	0.56691419	0.48734751	0.55642866	0.27966724	0.4210876
ENSG00000204001	0	0	0	0	0	0	0	0	0	0	0	0	0	0	0	0	0
ENSG00000204003	0	0	0	0	0	0	0	0	0	0	0	0	0	0	0	0	0
ENSG00000204006	0	0	0	0	0	0	0.02501971	0.01525823	0.01540271	0	0.03924963	0	0	0	0	0	0
ENSG00000204007	0.02112041	0	0	0	0	0	0	0	0	0	0.01912923	0	0	0	0	0	0
ENSG00000204010	0.04903104	0.01094726	0.03196765	0.0211261	0.00911685	0	0.0525008	0.03201753	0.00808018	0	0	0.02760445	0.01383345	0	0.01421404	0.01576637	0
ENSG00000204011	0	0	0	0	0	0	0	0	0	0	0	0	0	0	0	0	0

ENSG00000204438	1.81997626	1.760257	1.79285828	1.6984801	1.38466165	1.3393758	2.06578076	2.25754394	2.061506	1.85474293	2.20273482	2.16562674	1.9261181	1.62151849	1.91920665	2.10069322	1.8810643	[illegible]
ENSG00000204439	14.0378422	11.2780896	13.3976372	11.900653	14.1067155	10.1784789	11.1374871	12.2960778	9.76004197	8.20892103	15.142326	11.7669697	13.5571209	10.7357088	10.1248096	10.4140092	8.81549624	[illegible]
ENSG00000204442	4.85577448	2.904882	3.8516519	1.8686226	7.32473905	2.91255952	1.97210079	2.38721174	2.1166063	4.28048165	1.91461018	2.18338683	5.39630416	5.92302688	6.60859025	7.56274425	9.78626538	[illegible]
ENSG00000204444	1.2136243	1.29604633	0.95176166	1.9091405	1.20069122	1.33978948	0.1655034	0.22429333	0.10754814	0.20739756	0.25963318	0.32874305	0.38763158	0.32248997	0.36842397	0.25403179	0.24113436	[illegible]
ENSG00000204446	0.07960417	0.0399901	0.04865716	0.0128622	0.02220245	0.0447848	0.02397304	0.04873308	0.00491945	0	0.02507178	0.00840321	0.00842222	0.01077979	0.00865393	0.04799519	0.0149692	[illegible]
ENSG00000204449	0	0	0	0	0	0	0	0	0	0	0	0	0	0	0	0	0	[illegible]
ENSG00000204450	0	0	0	0	0	0	0	0	0	0	0	0	0	0	0	0	0	[illegible]
ENSG00000204455	0	0	0	0	0	0	0	0	0	0	0	0	0	0	0	0	0	[illegible]
ENSG00000204456	0	0	0	0	0	0	0	0	0	0	0	0	0	0	0	0	0	[illegible]
ENSG00000204460	0.08738294	0.03902032	0.04272947	0.0376509	0.04062005	0.04369874	0	0.01426538	0	0	0	0	0	0	0.02533221	0	0	[illegible]
ENSG00000204463	5.72150303	5.17991583	4.56267839	5.6846234	5.36354453	5.31903201	6.6977152	7.12727535	6.7410487	5.29434508	6.21858298	6.20076283	5.88851089	5.98663826	6.47768994	5.835529	5.09684545	[illegible]
ENSG00000204464	0	0.03285843	0	0	0	0	0	0	0.02425282	0	0	0	0.04152142	0.10628841	0	0	0	[illegible]
ENSG00000204466	0.01992829	0.02669657	0.01113686	0.0036799	0.02064479	0.00213553	0	0	0	0	0	0	0	0	0.00247594	0	0	[illegible]
ENSG00000204469	18.0727774	17.986096	15.4904115	15.553064	17.7720728	16.4805766	15.306412	17.0105223	16.0852541	14.7504921	12.6874613	14.9335248	14.5443001	12.9986918	15.4555851	12.6401339	11.195684	[illegible]
ENSG00000204471	0	0	0	0	0	0	0	0	0	0	0	0	0	0	0	0	0	[illegible]
ENSG00000204472	0	0	0	0	0	0	0.00675441	0	0	0	0	0	0	0	0	0.00811359	0	[illegible]
ENSG00000204475	0	0	0	0	0	0	0.00514264	0	0	0	0	0.00540791	0	0	0	0	0	[illegible]
ENSG00000204478	0	0	0	0	0	0	0	0	0	0	0	0	0	0	0	0	0	[illegible]
ENSG00000204479	0	0	0	0	0	0	0	0	0	0	0	0	0	0	0	0	0	[illegible]
ENSG00000204480	0	0	0	0	0	0	0	0	0	0	0	0	0	0	0	0	0	[illegible]
[illegible]																		

ENSG00000204850	0	0.01084279	0.01583129	0	0	0	0	0	0	0	0	0	0	0	0	0	0	0	0	0	0	0	0	0	0	0	0	0	0	0
ENSG00000204851	0.33985666	0.5749404	0.39203053	0.6589566	0.18228777	0.42162316	0	0	0	0	0	0	0.08113432	0.09440514	0.06441957	0.02521937	0.03277358	0.02627883	0.07192655	0.08511373	0.04034582	0.0409347	0.00882752	0	0	0	0.00374897	0	0.00312886	0.00350493
ENSG00000204852	1.10891128	1.11235829	1.18300655	0.9678889	0.89852395	0.88197197	0.85331364	0.86790218	0.72539931	0.65316091	0.90627344	0.73924291	0.71299911	0.70689022	0.67447122	0.71373393	0.75900652	0.55268076	0.34237231	0.40366616	0.458482	0.42330072	0.37293575	0.52708746	0.64927689	0.48417656	0.37735687	0.33741562	0.41671793	0.33558309
ENSG00000204856	0.93785716	0.7228506	0.90464298	1.1624698	0.88148319	1.0151103	2.80976924	3.28864216	3.79615348	3.1572737	2.93495049	3.8841512	1.46800782	1.21087012	1.16947242	2.23083872	1.95203621	1.06412981	1.68250972	1.22820497	2.02597257	2.38106562	3.42710352	3.11747871	3.33589007	3.67234701	2.51665697	3.84905295	3.33662269	3.12103459
ENSG00000204859	0.88033103	1.19448745	0.98275407	1.2659936	0.80844535	1.09139586	1.60944036	1.76894476	1.48481345	1.59723533	1.44784126	1.23826797	0.99908499	0.85556753	0.92317628	0.57616902	0.61319805	0.59752829	1.14015392	1.43012378	1.37488411	1.10242297	0.95485494	0.96846201	1.38892158	1.3208457	1.18611095	1.14402484	1.19725233	1.20453407
ENSG00000204860	0.01629136	0	0.00531089	0.0070195	0.00605844	0.00407352	0.00436106	0.00531917	0.00268477	0	0	0	0.02298195	0.01176605	0.00472285	0.03667043	0.00816937	0	0.00597631	0	0.01828524	0.00463803	0	0.00504877	0	0.00893819	0	0.00409469	0.00389961	0
ENSG00000204866	0	0.01434418	0	0	0.00398193	0.01606401	0.01719794	0	0	0	0	0	0	0	0	0	0	0	0	0	0.00600902	0	0	0	0	0	0.0053825	0	0	0
ENSG00000204869	0.06937094	0.11616439	0.04749047	0.053802	0.03869659	0.05203687	0.02785503	0.0339747	0.02400748	0.0376985	0.02621852	0.01171674	0.07045951	0.04509136	0.06636484	0.04684438	0.04174363	0.05857475	0.01526878	0.01847884	0.02919794	0.02962412	0.02810898	0.03869706	0.03311133	0.03996309	0.02387527	0.02092294	0.02988915	0.01116054
ENSG00000204872	0	0	0	0	0	0	0	0	0	0	0	0	0	0	0	0	0	0	0	0	0	0	0	0	0	0	0	0	0	0
ENSG00000204873	0	0	0	0	0	0	0	0	0	0	0	0	0	0	0	0	0	0	0	0	0	0	0	0	0	0	0	0	0	0
ENSG00000204876	0	0	0	0	0	0	0.24494851	0.29876297	0.15079595	0.25323602	0.20814223	0.21465292	0.46254796	0.13768037	0.14368739	0.56395858	0.38237563	0.19929009	0	0	0.03209471	0	0.01287405	0.01181563	0.01213212	0	0.01093499	0	0	0
ENSG00000204880	0	0	0	0	0	0	0	0	0	0	0	0	0	0	0	0	0.04176161	0	0	0	0.02336841	0	0	0	0	0	0	0	0	0
ENSG00000204882	0	0	0	0	0	0	0.10250651	0.18754032	0.05258781	0.19268123	0.0536023	0.07186268	0	0	0	0	0	0.02566132	3.55864392	4.87348478	1.57591032	1.29003147	0.99131013	1.06803891	1.15757199	1.36559581	1.51926311	2.19760573	3.72750958	2.84073062
ENSG00000204887	0	0	0	0	0	0	0	0	0	0	0	0	0	0	0	0	0	0	0	0	0	0	0	0	0	0	0	0	0	0
ENSG00000204889	0	0	0	0	0	0	0	0	0	0	0	0	0	0	0	0	0	0	0	0	0	0.00484114	0	0	0	0	0	0	0	0
ENSG00000204894	0	0	0.35022341	0	0	0	3.16346466	4.20923824	2.65568424	2.59477389	0.90230545	2.72179907	0.90931908	1.16385805	0.62289097	0.34545869	0.53872477	0.43196553	0.39410421	1.90783707	1.80871525	0.30585253	0	0.99881417	4.78598476	3.53654301	3.08123643	2.70022115	1.80010196	6.62551931
ENSG00000204897	0	0	0	0	0	0	0	0	0	0	0	0	0	0	0	0	0	0	0	0	0	0	0	0	0	0	0	0	0	0
ENSG00000204899	10.2759457	6.69518803	7.81490247	6.5961268	11.135838	6.48838418	5.9894297	7.19543554	9.34976436	5.57413638	6.23450285	9.41204984	14.5356574	11.3601456	17.8373324	24.30232	22.5659832	10.4167764	5.16843924	2.80076124	7.04525666	14.1165777	28.0615926	17.0871515	8.0029112	10.2681273	11.9175095	16.9025152	19.6206975	14.6714461
ENSG00000204904	0	0.04143568	0.12099849	0	0.03450755	0	0.24839628	0.12118728	0.33642065	0.50426268	0.31173701	0.05224182	0.05236002	0.06701678	0.05380056	0.11935233	0.18612372	0	0.20423826	0.16478439	0.36452035	0.10566882	0.25066091	0.11502658	0.4133768	0.96728863	0.37258713	0.69967343	0.26653553	0.34833324
ENSG00000204909	0.42688397	0.71483412	0.26838312	0.2364846	0.25513389	0.32021628	0.3673069	0.41813625	0.22612259	0.19331925	0.30731311	0.07725068	0.38712733	0.33032868	0.26518537	0.26473182	0.59631786	0.40458456	0.30200985	0.73100663	0.48768576	0.41667705	0.21621579	0.42522836	0.29107895	0.27603103	0.3935354	0.22991476	0.28464918	0.22075072
ENSG00000204913	0.03617255	0	0.0353761	0.0467572	0	0.02713393	0	0	0	0	0	0	0	0	0	0	0	0	0.03980851	0	0.03044975	0	0	0	0	0	0.0622472	0	0	0.02909758
ENSG00000204915	0	0	0	0	0	0	0	0	0	0	0	0	0.11645495	0.18631666	0.08974416	0.26545385	0.31047105	0.16596328	0	0	0	0.02937751	0.03484374	0.06395822	0	0.05661488	0.02959565	0.02593596	0	0
ENSG00000204918	0	0	0	0	0	0	0	0	0	0	0	0	0	0	0	0	0	0	0	0	0	0	0	0	0	0	0	0	0	0
ENSG00000204919	0	0	0	0	0	0	0	0	0	0	0	0	0	0	0	0	0	0	0	0	0	0	0	0	0	0	0	0	0	0
ENSG00000204920	1.20466139	1.07511709	1.14522835	1.170049	1.11684786	1.31508327	1.05120539	1.15360727	0.94826061	1.62139504	1.04710013	0.91787544	0.79463361	0.87489851	0.98038019	0.70116067	0.835254	0.76714697	1.08504154	0.98598804	0.93759357	1.02600145	1.04647267	0.81965748	0.83518811	0.78370354	0.82805414	1.5807204	1.31934807	1.49416598
ENSG00000204922	3.88887769	2.78324506	3.41622879	3.8266855	4.2876551	3.12265695	3.58754616	3.93591109	3.92802978	3.97027976	2.93382433	3.16204283	1.95820308	1.97869609	1.85322934	1.62981454	1.32804878	1.42288528	3.14911129	2.946223	4.42035486	3.95188915	2.82157772	5.87967821	3.95939539	4.07725413	2.68471368	7.19591402	5.91856766	5.16682354
ENSG00000204923	0.87030536	0.68581686	0.71763038	0.7940974	0.58383905	0.76377744	0.22383723	0.2730136	0.29809657	0.31942372	0.25798356	0.31704684	0.68848903	0.57925868	0.60354244	1.009671	0.77870735	0.45285479	0.49454305	0.50002436	0.48841058	0.65586097	1.37140186	0.79326928	0.88510913	1.17031736	1.10121846	1.06798369	1.12330502	1.07071499
ENSG00000204928	0	0	0	0	0	0	0	0	0	0	0	0	0	0	0	0	0	0.04748243	0	0	0	0	0	0	0	0	0	0	0	0
ENSG00000204929	0.09888319	0.0952107	0.13901485	0.1437953	0.09997596	0.09735435	0.04466846	0.03632133	0.01527719	0.01679264	0.04671572	0.02609587	0.05230982	0.0133905	0.01612469	0.01788568	0.04648632	0.02981929	0.03400707	0.03292528	0.05202441	0.06334051	0.06886566	0.05745815	0.02359888	0.08137779	0.1063514	0.07922029	0.12870199	0.11931403
ENSG00000204930	0.01502552	0.04277341	0.01836836	0.0534112	0.00209539	0.03381305	0.01809992	0.02575583	0.01114273	0.0442291	0.01892948	0.0317226	0.02225606	0.0122083	0.02286837	0.02174216	0.03390575	0.00453111	0.01240188	0.02668304	0.01264836	0.02887419	0.02663637	0.02095414	0.0035859	0.00927415	0.02585615	0.0198268	0.02697455	0.01813003
ENSG00000204933	0	0	0	0	0	0	0	0	0	0	0	0	0	0	0	0	0	0	0	0	0	0	0	0	0	0	0	0	0	0
ENSG00000204934	2.59925428	2.88134004	2.40823327	3.0913145	2.75569472	3.08237748	0.68359157	0.5757015	0.52103348	1.08761451	0.40214368	0.45784075	0.3602396	0.2305394	0.31286538	0.26882888	0.1981789	0.14668221	0.09479318	0.19345306	0.27723587	0.10385812	0.22070258	0.32032399	0.45949839	0.27520654	0.20925863	0.15663915	0.17464551	0.10189414
ENSG00000204936	0	0	0	0	0	0	0	0	0.00220297	0	0	0	0.07543087	0.06758197	0.07750614	0.06877651	0.00670334	0.03762454	0	0	0	0	0	0	0	0	0	0	0.0035844	0.03385081
ENSG00000204941	0	0.0015856	0.0023151	0	0.00132049	0	0.00760423	0.00695614	0.003511	0.00643214	0	0.00199912	0.00601092	0.01538703	0.01029383	0.00913442	0.00356116	0.00856634	0	0	0.00199271	0.00202179	0	0.00220084	0	0.00194815	0.00611042	0.00178494	0.0033998	0
ENSG00000204946	1.04857445	1.46958595	1.12495684	1.3460141	0.96855796	1.29882602	1.29715902	1.40423907	1.19963736	1.65378086	1.10440723	1.00208997	0.68255298	0.57716306	0.6212997	0.57701906	0.50273962	0.54916649	0.66360172	1.04931755	0.59321076	0.55223055	0.44646797	0.56060875	0.33520336	0.31887081	0.42297836	0.31406885	0.42083467	0.49868825
ENSG00000204947	0.61775882	1.03668627	0.87050583	0.9530787	0.60026926	0.8719822	1.56833686	1.46395085	1.20852243	2.2380727	1.48121659	0.99851656	0.36545182	0.50373081	0.38128322	0.24350138	0.21984234	0.32851459	0.81143879	1.05575897	0.32431621	0.27231718	0.29607125	0.2099733	0.35510192	0.18586531	0.25719307	0.38566638	0.44838248	0.47555835
ENSG00000204949	0	0	0	0	0	0.00727326	0	0.00949738	0	0.02634585	0	0	0	0	0	0	0	0	0	0.00860937	0	0.01656241	0	0	0	0	0.0083427	0.00731107	0.01392549	0.00779962
ENSG00000204950	0	0.01952781	0	0.0188425	0.00813136	0	0	0	0.02882697	0	0.09182213	0.03693079	0.11104304	0.06316733	0.07606538	0.1406209	0.28507824	0.1230838	0.06416893	0.02588653	0.08589557	0.1120491	0.19196363	0.23039133	0.12523899	0.23992829	0.23830458	0.16487103	0.19888702	0.1289848
ENSG00000204952	0	0	0	0	0	0	0	0	0	0	0	0.01182363	0	0	0	0	0	0	0	0	0	0	0	0	0	0	0	0	0	0
ENSG00000204954	0.67921273	0.52203648	0.56018059	0.7525387	0.6372855	0.62219358	0.56054045	0.63156969	0.52922833	0.72574039	0.69007045	0.65553673	0.445078	0.37977693	0.38926905	0.33817846	0.37669402	0.29449335	0.47191462	0.4502325	0.51115484	0.4892096	0.5421878	0.59364307	0.48106188	0.49199746	0.45513845	0.43897933	0.47875185	0.40033265
ENSG00000204956	0.42371583	0.34179459	0.33418264	0.2885743	0.264314	0.24606951	0.0622009	0.02677632	0.02702987	0.13204956	0.05165871	0.03462849	0.05398841	0.10365148	0.1109475	0.04395149	0.11651808	0.06594894	0.33594124	0.6351366	0.66350241	0.19845393	0.32768581	0.33886825	0.50452039	0.45744089	0.39985511	0.29200865	0.36316125	0.33351159
ENSG00000204957	0.07328272	0.0687203	0	0.0757812	0.06540586	0.12093662	0.45904175	0.24405542	0.30433489	0.41151769	0.4062221	0.21041648	0.17367622	0.09526805	0.12746746	0.16966593	0.13229258	0.08839677	0.2742062	0.50754192	0.1727286	0.16273191	0.22270521	0.25890135	0.16789656	0.15680443	0.13871869	0.12156521	0.19997235	0.14147855
ENSG00000204959	0	0	0	0.0043501	0.00187725	0	0.13783309	0.10218731	0.09150838	0.11887408	0.16958858	0.09662856	0.01993913	0.02187475	0.02926814	0.00324645	0	0.01217821	0.0222216	0.04183416	0.01699743	0.01724552	0.01363625	0.01877274	0.00642519	0.01661736	0.02026918	0.04060059	0.02416641	0.02165684

ENSG00000205452	0	0	0	0	0	0	0	0	[illegible]
ENSG00000205456	0.06275719	0.06042641	0.06137541	0.0354904	0.02844343	0.08532476	0.03464912	0.02305169	[illegible]
ENSG00000205457	0	0.00196881	0.00287461	0	0.00327924	0	0.0023605	0	[illegible]
ENSG00000205464	0.48223253	0.57328393	0.43101216	0.7141599	0.35629082	0.60368956	0.2154343	0.23148308	[illegible]
ENSG00000205476	3.54412811	3.50169599	3.38477765	4.4770813	3.16405382	4.13983583	0.97029548	1.35907765	[illegible]
ENSG00000205482	0	0.04813376	0	0	0	0	0	0	[illegible]
ENSG00000205485	0.76507723	0.98798759	0.84934419	0.7929432	0.69206978	1.02888998	0.87457165	0.93843688	[illegible]
ENSG00000205488	0.01136851	0.00380741	0.01667731	0	0.0031708	0.01705561	0.00912977	0.0222711	[illegible]
ENSG00000205494	0	0	0	0	0	0	0	0	[illegible]
ENSG00000205495	0	0	0	0	0	0	0	0	[illegible]
ENSG00000205496	0	0	0	0	0	0	0	0	[illegible]
ENSG00000205497	0	0	0	0	0	0	0	0	[illegible]
ENSG00000205500	0.05915523	0.09245392	0.04821063	0.0509767	0.02749837	0.09614325	0.07917679	0.06760019	[illegible]
ENSG00000205502	0	0	0.01996207	0	0.03415789	0.01531116	0	0	[illegible]
ENSG00000205505	0	0	0	0	0	0	0	0	[illegible]
ENSG00000205506	0	0	0	0	0	0	0	0	[illegible]
ENSG00000205508	0	0	0	0	0	0	0	0	[illegible]
ENSG00000205509	0	0	0	0	0	0	0	0	[illegible]
ENSG00000205517	0	0.00221745	0.00161882	0	0	0	0.00797583	0.0064854	[illegible]
ENSG00000205531	8.71117585	7.37422835	8.08351488	6.9453784	8.22293501	6.68215646	3.34765685	4.0971524	[illegible]
ENSG00000205534	0.0826555	0.0632731	0.03464382	0.0610525	0.06586714	0.08857421	0.10430915	0.10987662	[illegible]
ENSG00000205537	0	0	0	0	0	0	0	0	[illegible]
ENSG00000205542	227.58216	166.201083	210.479198	146.8563	239.835927	170.492929	53.8626455	55.088054	[illegible]
ENSG00000205544	12.5005348	10.0384156	13.2159304	17.55701	11.7373497	14.1431527	4.84379848	5.5246252	[illegible]
ENSG00000205549	0	0	0	0	0	0	0.02704586	0	[illegible]
ENSG00000205559	0.33456477	0.44819358	0.16359917	0.0720773	0.37325471	0.25096536	0.76126155	0.60079958	[illegible]
ENSG00000205560	0.0176922	0.02005472	0.01064779	0.0281467	0.01290573	0.02041747	0.14426745	0.13197194	[illegible]
ENSG00000205562	0.20293482	0.18535768	0.14433935	0.214623	0.2778579	0.31829169	0.08889374	0.12649399	[illegible]
ENSG00000205565	0	0	0	0	0	0	0	0	[illegible]
ENSG00000205571	0.10331885	0.10957395	0.11788464	0.0445172	0.11046412	0.04951519	0.11984955	0.11806857	[illegible]
ENSG00000205572	0.10556594	0.08915576	0.0897753	0.0771275	0.05120602	0.1101741	0.06266152	0.02697461	[illegible]
ENSG00000205578	0	0.00388693	0.00567521	0	0.00647405	0	0.0372818	0.09662907	[illegible]
ENSG00000205579	0	0	0	0	0	0	0	0	[illegible]
ENSG00000205580	0	0	0	0	0	0	0	0	[illegible]
ENSG00000205581	6.12936734	5.58574806	5.41685902	4.7073944	5.85893611	5.15405683	5.36736525	6.3009148	[illegible]
ENSG00000205583	0.10354936	0.21385693	0.12658677	0.2063515	0.11311733	0.16505928	0.2009649	0.09297514	[illegible]
ENSG00000205584	0	0	0	0	0	0	0	0	[illegible]
ENSG00000205592	0.01752699	0.02828238	0.02882818	0.0164769	0.00977693	0.03406379	0.00063979	0.00078036	[illegible]
ENSG00000205593	0.95757876	1.33991394	1.0262957	0.9749656	0.90184864	0.97167787	0.25282435	0.23127683	[illegible]
ENSG00000205595	0	0	0	0	0	0	0	0	[illegible]
ENSG00000205596	0	0	0	0	0	0	0	0	[illegible]
ENSG00000205609	0	0	0	0.0045117	0.00194698	0	0.002803	0.00341881	[illegible]
ENSG00000205611	0.08715975	0.11676197	0.09471186	0.0375547	0.09183696	0.09443879	0.00777731	0	[illegible]
ENSG00000205622	0	0	0	0	0	0	0	0	[illegible]
ENSG00000205625	0.11181176	0.03744664	0.08201242	0.0361324	0.03118549	0	0.08979321	0.08214038	[illegible]
ENSG00000205628	0	0	0	0	0	0	0	0	[illegible]
ENSG00000205629	2.09221968	1.93864408	1.79580826	2.205904	2.22564763	1.79216674	1.99816041	2.03263014	[illegible]
ENSG00000205632	0	0	0	0	0	0	0	0	[illegible]
ENSG00000205634	0	0	0	0	0	0	0	0	[illegible]
ENSG00000205636	0	0	0	0	0	0	0	0	[illegible]
ENSG00000205639	0.04758508	0.0588429	0.02505857	0.0378519	0.06942269	0.0604065	0.08230789	0.12190298	[illegible]
ENSG00000205642	0	0	0	0	0	0	0	0	[illegible]
ENSG00000205643	1.02518363	1.12650266	0.97775304	1.3361214	0.80817886	0.87070377	0.60556715	0.64317028	[illegible]
ENSG00000205644	0	0	0	0	0	0	0	0	[illegible]
ENSG00000205649	0.1351706	0.35649893	0.23960238	0.1310428	0.16022721	0.22180119	0.16961266	0.19032598	[illegible]
ENSG00000205653	0	0	0	0	0	0	0	0	[illegible]
ENSG00000205659	4.17728991	3.86502662	4.05155158	4.1692577	3.40035894	3.48864248	0.70103627	1.0676071	[illegible]
ENSG00000205662	0	0	0	0	0	0	0	0	[illegible]
ENSG00000205663	0.00851962	0	0	0	0	0	0	0	[illegible]
ENSG00000205664	0.69516224	0.75852741	0.76757957	0.905825	0.75053477	0.86629394	1.2786122	1.3947815	[illegible]
ENSG00000205667	0	0	0	0	0	0	0	0.01683695	[illegible]
ENSG00000205669	0	0.02303948	0.03363939	0.0444618	0	0.03870277	0.01381158	0.01684594	[illegible]
ENSG00000205670	1.56566758	1.51388433	1.87681879	2.1459803	1.47852742	1.85876917	1.63356265	1.92804901	[illegible]
ENSG00000205678	0	0.0072362	0	0	0	0	0	0	[illegible]
ENSG00000205682	0.0775126	0.10383834	0.07580593	0.0601165	0.06918109	0.15117474	0.06224842	0.07592421	[illegible]
ENSG00000205683	1.79855654	1.1530873	1.47514997	1.0193711	2.68515033	1.32946445	0.20276556	0.25534218	[illegible]
ENSG00000205693	0	0.02110263	0	0	0.01757423	0	0.17710679	0.18515711	[illegible]
ENSG00000205695	0	0	0	0	0	0	0.0163196	0	[illegible]
ENSG00000205696	0	0	0	0	0	0	0	0	[illegible]
ENSG00000205702	0.06171915	0.1157533	0.07243226	0.0877571	0.04475673	0.09259414	0.21312991	0.16927232	[illegible]
ENSG00000205704	0.61866959	1.04585329	0.66267135	1.4280405	0.4683596	0.91710939	0.13012423	0.17314051	[illegible]
ENSG00000205707	1.76773558	2.29627851	2.14112486	2.136814	1.76174223	1.73381593	2.70263136	2.5393081	[illegible]
ENSG00000205710	0.03601893	0.06755294	0.04227104	0.0372469	0.0321474	0.01621122	0.26611825	0.19757265	[illegible]
ENSG00000205716	0	0	0	0	0	0	0	0	[illegible]
ENSG00000205718	0	0	0	0	0	0	0	0	[illegible]
ENSG00000205726	0.66055894	0.8554427	0.71350886	0.6254374	0.65381896	0.68039037	0.48875866	0.51628106	[illegible]
ENSG00000205730	3.28244335	1.99390161	2.84301549	2.1944767	3.83994612	2.94073484	7.90547479	9.3851998	[illegible]
ENSG00000205740	0.06875678	0.09210876	0.05043217	0.0666571	0.019177	0.07736425	0.06902105	0.11785867	[illegible]
ENSG00000205744	0	0.00230161	0	0	0	0	0.00137976	0.00504866	[illegible]
ENSG00000205745	0	0	0	0	0	0	0	0	[illegible]
ENSG00000205746	0.16253993	0.16607566	0.14818411	0.2350292	0.12908698	0.27484843	0.23451463	0.25365527	[illegible]
ENSG00000205754	0	0	0	0	0	0	0	0	[illegible]
ENSG00000205755	0	0.00658572	0	0	0.00548457	0	0.54481989	0.32744227	[illegible]
ENSG00000205758	0.66030599	1.04688942	0.78823567	0.8588021	0.61363475	1.00798535	0.66257056	0.75479307	[illegible]
ENSG00000205763	2.40018636	2.05808429	2.0824643	2.4264854	2.53188044	2.16473074	0.97501479	0.9788217	[illegible]
ENSG00000205765	8.40645285	9.79017551	8.40242557	6.759111	8.99898688	7.93115727	5.85891282	5.80804299	[illegible]
ENSG00000205767	0	0	0	0	0	0	0	0	[illegible]
ENSG00000205771	0.09578769	0.07925661	0.09918915	0.1602335	0.09743557	0.14793209	0.15384936	0.16557318	[illegible]
ENSG00000205775	0	0	0	0	0	0	0	0	[illegible]
ENSG00000205777	0	0	0	0.0106905	0	0	0	0	[illegible]
ENSG00000205784	0	0	0	0	0	0	0	0.01927307	[illegible]
ENSG00000205786	0.02809566	0.06989885	0.04317823	0.0466932	0.02910581	0.04516126	0.04512584	0.03145134	[illegible]
ENSG00000205790	0	0.00702963	0	0.0271317	0	0.00787246	0	0.01027981	[illegible]
ENSG00000205791	1.02682526	1.22116722	1.25014727	0.8938902	0.65461142	0.91172113	1.02656466	0.90315255	[illegible]
ENSG00000205794	0.06319557	0.08465879	0.07416496	0.0816877	0.02820147	0.07584731	0.05075077	0.16094146	[illegible]
ENSG00000205795	0	0	0.00971344	0.0385153	0	0	0.21535843	0.13620031	[illegible]
ENSG00000205808	1.78630528	1.83851761	1.25696944	2.9848154	1.7376926	2.03443934	3.73504399	3.68077187	[illegible]
ENSG00000205809	0.00758347	0.01015905	0.01483299	0.0098025	0	0.0113771	0.15225231	0.16341748	[illegible]
ENSG00000205810	0.01151473	0.06170201	0.04504481	0.0148841	0.01284633	0	0.07397754	0.01127877	[illegible]
ENSG00000205821	0	0	0	0	0	0	0	0	[illegible]
ENSG00000205822	0.03298847	0.03682701	0.02150809	0	0	0.02474548	0.00883074	0.06462495	[illegible]
ENSG00000205830	0	0	0	0	0	0	0	0	[illegible]
ENSG00000205832	0.02529145	0.02823436	0.03297945	0.0435895	0.00940541	0.02529566	0.04062186	0.04128863	[illegible]
ENSG00000205835	0	0	0	0	0	0	0	0.04444102	[illegible]
ENSG00000205837	0	0	0	0	0	0	0	0	[illegible]
ENSG00000205838	0.09650778	0.08274233	0.07928162	0.0848284	0.18303593	0.08976718	0.02480095	0.04537447	[illegible]
ENSG00000205846	0	0	0	0	0	0	0	0	[illegible]
ENSG00000205847	0	0	0	0	0	0	0	0	[illegible]
ENSG00000205850	0.10636878	0	0	0	0	0	0	0	[illegible]
ENSG00000205853	0.04367173	0.06435444	0.0256261	0.1241918	0.02436102	0.11138146	0.01402867	0.05988754	[illegible]
ENSG00000205856	0	0	0	0	0	0.01246203	0	0	[illegible]
ENSG00000205857	0	0.02380153	0.03475205	0	0.01982187	0.02665527	0.08561045	0.24364389	[illegible]
ENSG00000205858	0	0	0	0	0	0	0	0	[illegible]
ENSG00000205861	0.21225259	0.07655316	0.0958058	0.0844188	0.1639371	0.0612369	0.0262238	0.14393292	[illegible]
ENSG00000205863	0.00774753	0.01556826	0.00757695	0	0.00432174	0	0	0	[illegible]
ENSG00000205864	0	0	0	0	0	0	0	0	[illegible]
ENSG00000205865	0	0	0	0	0	0	0	0	[illegible]
ENSG00000205866	0	0	0	0	0	0	0	0	[illegible]
ENSG00000205867	0	0	0	0	0	0	0	0	[illegible]
ENSG00000205869	0	0	0	0	0	0	0	0	[illegible]
ENSG00000205871	0.11030027	0.20686644	0.1725947	0.2281216	0.20919466	0.11583457	0.19487462	0.38894399	[illegible]
ENSG00000205879	0.01330158	0	0.01300871	0.0343877	0.02225972	0.0399114	0	0	[illegible]
ENSG00000205882	0	0	0	0	0	0	0	0	[illegible]
ENSG00000205883	0	0	0	0	0	0	0	0	[illegible]
ENSG00000205884	0	0	0	0	0	0	0	0	[illegible]
ENSG00000205885	0.05996559	0.0921453	0.04484638	0.1094294	0.06493251	0.06879551	1.93760518	1.81392969	[illegible]
ENSG00000205890	0	0	0	0	0	0.00789818	0.0084557	0.03094017	[illegible]
ENSG00000205897	0	0	0	0	0	0	0	0	[illegible]
ENSG00000205898	0	0	0	0	0	0	0	0	[illegible]
ENSG00000205899	0	0	0	0	0	0.02680303	0	0	[illegible]
ENSG00000205903	6.38852303	7.28632927	5.96067617	6.840824	5.86055484	7.33268706	6.00038714	6.49410719	[illegible]
ENSG00000205913	0.07126969	0.08751888	0.06195598	0.0716523	0.04417306	0.06534143	0.31797165	0.2947501	[illegible]
ENSG00000205916	0	0	0	0	0	0	0	0	[illegible]
ENSG00000205918	0.01626943	0.0508551	0.07425231	0.0560804	0.03630174	0.06508855	0.05661747	0.03718408	[illegible]
ENSG00000205922	0.02324819	0.04152535	0.04926201	0.0250425	0.01512972	0.02906507	0.01244669	0.04554357	[illegible]
ENSG00000205923	0.00863374	0.01734904	0	0	0	0.01942914	0.00693354	0.00845681	[illegible]
ENSG00000205927	29.8784509	36.0955241	31.2989991	18.541123	12.5050315	19.1413214	0	0.029527	[illegible]
ENSG00000205929	8.51975515	6.70745293	10.5319875	1.7835741	9.39114199	2.34567046	0.04589747	0.04798372	[illegible]
ENSG00000205930	0.12098251	0.14910624	0.10885322	0.1313626	0.08098394	0.14883326	0.15545281	0.14220399	[illegible]
ENSG00000205936	0	0	0	0	0	0	0	0	[illegible]
ENSG00000205937	3.77153527	3.02467541	3.12910134	3.0255357	3.70599574	2.92509663	3.09002104	3.34224009	[illegible]
ENSG00000205940	0.39893489	0.3375321	0.32170359	0.2714052	0.3630824	0.16275053	0.02248243	0.02742175	[illegible]
ENSG00000205944	0	0	0	0	0	0	0	0	[illegible]
ENSG00000205946	0	0	0	0	0	0	0	0	[illegible]
ENSG00000205955	0.0204309	0.00684247	0.01998105	0	0.00569839	0.00766286	0	0	[illegible]
ENSG00000205959	0.05981129	0.13735731	0.08356338	0.0994025	0.05719545	0.14100722	0.05489479	0.05021626	[illegible]
ENSG00000205971	0	0.01517076	0	0	0	0	0	0	[illegible]
ENSG00000205976	0	0	0	0	0	0	0	0	[illegible]
ENSG00000205978	2.30768361	3.12533213	2.40149569	2.3139245	0.94212036	2.03464158	0.62816487	0.79285338	[illegible]
ENSG00000205981	3.53582426	3.68206381	3.59187281	4.0262573	3.3394523	3.52785435	1.80322617	1.83729494	[illegible]
ENSG00000205989	0	0	0	0	0	0	0	0	[illegible]
ENSG00000206013	0	0	0	0	0.04885444	0	0	0	[illegible]
ENSG00000206014	0	0	0	0.0223142	0	0	0	0.0169091	[illegible]
ENSG00000206026	0	0	0	0	0	0	0	0	[illegible]
ENSG00000206028	0.09867505	0.14045	0.15681645	0.1753799	0.08944495	0.20355118	0.24763579	0.096653	[illegible]
ENSG00000206034	0	0	0	0	0	0	0	0	[illegible]
ENSG00000206042	0	0	0	0	0	0	0	0	[illegible]
ENSG00000206043	0	0.00421065	0	0	0	0	0	0	[illegible]
ENSG00000206044	0	0	0	0.0887342	0	0	0	0	[illegible]
ENSG00000206047	0	0	0	0	0	0	0	0	[illegible]
ENSG00000206052	0.50526151	0.70871677	0.65400443	0.6761604	0.50566421	0.60864422	0.0954736	0.12227131	[illegible]
ENSG00000206053	6.66779065	4.18967342	5.27306222	4.4010821	7.07832549	4.43394319	4.51585984	5.47122004	[illegible]
ENSG00000206062	0	0	0.04253726	0	0	0.03262663	0.03492968	0	[illegible]
ENSG00000206066	0	0.09948383	0	0.1919848	0	0.11141166	0	0	[illegible]
ENSG00000206069	0	0	0	0	0	0	0	0	[illegible]
ENSG00000206072	0	0	0	0	0	0	0.00991175	0.00402978	[illegible]
ENSG00000206073	0	0	0	0	0	0	0.00908969	0.01663	[illegible]
ENSG00000206075	0	0	0	0	0	0	0.00426126	0	[illegible]
ENSG00000206077	0.56096066	1.07788139	0.6372736	0.8056746	0.66692155	1.15186098	0	0	[illegible]
ENSG00000206082	0.06723868	0.21017515	0.21919407	0.202799	0.10001911	0.13449975	0.08999615	0.08781443	[illegible]
ENSG00000206090	0	0	0	0	0	0	0	0	[illegible]
ENSG00000206102	0	0	0	0	0	0	0	0	[illegible]
ENSG00000206104	0	0	0	0	0	0	0	0	[illegible]
ENSG00000206105	0	0	0	0	0	0	0	0	[illegible]
ENSG00000206106	0	0	0	0	0	0	0.08833752	0	[illegible]
ENSG00000206107	0	0	0	0	0	0	0	0	[illegible]
ENSG00000206113	0	0	0	0	0	0	0.04014408	0.01836135	[illegible]
ENSG00000206114	0.03857539	0.03875761	0.05658906	0.0249316	0.05379544	0.08680908	0.04646839	0.09446226	[illegible]
ENSG00000206120	0.55965543	0.96449917	0.69271827	0.7460269	0.29592793	0.96425443	0.09831613	0.14561216	[illegible]
ENSG00000206127	0	0.0122035	0	0	0	0	0	0	[illegible]
ENSG00000206129	0.05176637	0.02080436	0.01012532	0.0133828	0.01155055	0	0.00831445	0.01014111	[illegible]

ENSG00000206140	0.01265897	0.03532991	0.0082535	0.0245448	0.00588454	0.0316527	0.033887	0.01239956	0.01877546	0.02866379	0.01063204	0.01425399	0.04643028	0.08228376	0.07706624	0.05088256	0.05395712	0.03562952	0.04875995	0.08617489	0.05505712	0.0396431	0.05343117	0.03138458	0.04632378	0.06424396	0.05809087	0.0286355	0.03030135	0.02545753	0.11219563	0.16004211
ENSG00000206142	0	0.00464958	0	0.0029909	0.00129072	0	0	0	0	0	0	0	0	0	0	0	0	0	0.01273221	0.00616359	0	0.00790487	0.00703179	0.01505858	0.01987963	0.02856351	0.00995446	0.0017447	0	0	0	0
ENSG00000206144	0.01494886	0.01001298	0	0	0	0	0	0	0	0.01353952	0	0	0	0.01619469	0	0	0	0	0	0	0	0	0.01514313	0.01389816	0.04281129	0	0	0.02254359	0.02146954	0	0.0227127	0.01828957
ENSG00000206145	0	0	0	0	0.01892467	0	0	0.06646166	0.03354549	0.15363793	0.38466706	0.28650516	0.20100738	0.11026024	0.14752681	0.03272767	0.10207417	0.0818461	0.07467238	0.06024749	0.02855866	0.0289755	0	0.0315415	0.06477273	0	0	0.12790521	0.14617369	0.08187141	0.15463763	0.33206157
ENSG00000206147	0.34581266	0.27795696	0.40583828	0.4470032	0.231482	0.20752214	0.22217075	0.20323575	0.13677344	0.12528415	0.0871325	0.17522312	0.11707971	0.14985297	0.18045125	0	0.41618223	0.33370728	0	0	0.23288179	0.05907023	0.07006134	0.38580805	0.39614221	0.17075583	0.29754429	0.41720155	0.24832798	0.33381045	0.10508279	0.42309348
ENSG00000206149	0.17775199	0.22863946	0.17691504	0.1768989	0.17812594	0.25959258	0.27286372	0.22033325	0.19208961	0.30916549	0.22988113	0.25505658	0.18639954	0.16359565	0.15048613	0.21547962	0.21297626	0.15179617	0.27525166	0.32264412	0.36811668	0.27541506	0.34737621	0.31004237	0.29131762	0.2498485	0.29370173	0.1838454	0.1796048	0.19359996	0.32742914	0.29830682
ENSG00000206150	0	0	0.01961425	0	0.01118758	0	0	0	0	0	0	0	0	0	0	0	0	0	0	0.01780806	0	0.01712926	0.02031648	0	0	0	0	0	0	0.01613313	0	0
ENSG00000206159	0.03171178	0.03540176	0	0.0136637	0.04127551	0.03964633	0.01697796	0.04141592	0.02090404	0.03829611	0.01331707	0.00892686	0.03578822	0.03435462	0.00919321	0.02039441	0	0.0127507	0.03489936	0.01877177	0.02669472	0.01805623	0	0	0.04036346	0	0.00909516	0.03985239	0.01518148	0.0085031	0	0.06466434
ENSG00000206168	0	0.12736278	0.09297967	0	0.05303373	0	0	0	0.04700326	0	0	0	0	0	0	0	0	0	0	0	0	0	0	0.08839063	0	0	0	0	0.06827188	0	0	0
ENSG00000206172	0	0	0	0.0192428	0	0	0	0	0	0	0	0	0.01260026	0	0	0	0	0	0	0	0	0	0	0.01384038	0.01421111	0	0.01280884	0	0	0	0	0
ENSG00000206176	0.01120255	0	0	0	0	0	0.00899649	0	0	0	0	0	0.06637366	0.07281698	0.08768538	0.06484105	0.05055811	0.054052	0.0369858	0.14920519	0.05658129	0.00956786	0.02269627	0.01041516	0.01069414	0.0368774	0.02891671	0.03378796	0.04022275	0.0360458	0.05106213	0.08223631
ENSG00000206177	0	0	0	0	0	0	0	0	0	0	0	0	0	0	0	0	0	0	0	0	0	0	0	0	0	0	0	0	0	0	0	0
ENSG00000206178	0	0	0	0	0	0	0	0	0	0	0	0	0	0	0	0	0	0	0	0	0	0	0	0	0	0	0	0	0	0	0	0
ENSG00000206181	0	0	0.01034803	0	0	0	0	0	0	0	0	0	0	0	0	0	0	0	0	0	0	0	0	0	0	0	0	0	0	0	0	0
ENSG00000206187	0	0.00868732	0	0	0.00723478	0	0	0	0	0.01174696	0	0	0	0	0	0	0	0	0	0	0	0	0	0	0	0	0	0	0	0	0.0129456	0
ENSG00000206190	0	0	0	0	0	0	0.70212726	0.85063499	0.94088388	0.64306396	0.55935491	0.58968459	0	0	0	0	0.00294242	0	1.55197366	1.33032212	2.2194576	1.78076781	1.12144061	1.18017428	0.16617714	0.14165059	0.21709646	0.25514288	0.3286646	0.41379558	0.42496099	0.48339161
ENSG00000206192	0.02164522	0.03624579	0.03175296	0	0.01811125	0.01623662	0	0	0	0	0.02726914	0	0	0	0	0	0	0	0	0.00960966	0	0	0.01096326	0	0.01033145	0	0	0.01632101	0.00777172	0.00870582	0	0
ENSG00000206195	0.55824177	0.55503629	0.64831613	0.5524694	0.3470803	0.46891392	1.4710169	1.4011808	1.48488326	0.99279069	1.48715567	1.27434706	0.33961037	0.42837536	0.37171166	0.28609028	0.19245377	0.23147273	0.35197399	0.53181926	0.74649276	0.57859518	0.40055998	0.94339958	1.34059384	0.86618983	0.49533273	0.87035545	0.96460132	0.83496278	0.55661176	0.53714742
ENSG00000206199	0.12617123	0.07981641	0.1005426	0.0422829	0.02215698	0.04907476	0	0.00457725	0	0.00211622	0.00883074	0.00394635	0.00593292	0.00253123	0.0040641	0.00225397	0	0	0	0.00414928	0.0039337	0	0.00473374	0	0.00446093	0.00192287	0.00402075	0.00528534	0	0	0.01419997	0.02286925
ENSG00000206203	0	0	0	0	0.00991093	0	0	0.01740313	0.00878396	0	0	0.0150044	0	0	0	0	0	0	0.01955313	0	0	0	0	0	0	0	0.01528728	0	0	0	0	0

ENSG00000206741	0	0	0	0	0	0	0	0	0	0	0	0	0	0	0	0	0	0	0	0	0	0	0	0	0	0	0	0	0	0	0	0	0	0	0	0	0	0	0	0	0	0	0	0	0	0	0	0
ENSG00000206743	0	0	0	0	0	0	0	0	0	0	0	0	0	0	0	0	0	0	0	0	0	0	0	0	0	0	0	0	0	0	0	0	0	0	0	0	0	0	0	0	0	0	0	0	0	0	0	0
ENSG00000206744	0	0	0	0	0	0	0	0	0	0	0	0	0	0	0	0	0	0	0	0	0	0	0	0	0	0	0	0	0	0	0	0	0	0	0	0	0	0	0	0	0	0	0	0	0	0	0	0
ENSG00000206745	0	0	0	0	0	0	0	0	0	0	0	0	0	0	0	0	0	0	0	0	0	0	0	0	0	0	0	0	0	0	0	0	0	0	0	0	0	0	0	0	0	0	0	0	0	0	0	0
ENSG00000206746	0	0	0	0	0	0	0	0	0	0	0	0	0	0	0	0	0	0	0	0	0	0	0	0	0	0	0	0	0	0	0	0	0	0	0	0	0	0	0	0	0	0	0	0	0	0	0	0
ENSG00000206747	0	0	0	0	0	0	0	0	0	0	0	0	0	0	0	0	0	0	0	0	0	0	0	0	0	0	0	0	0	0	0	0	0	0	0	0	0	0	0	0	0	0	0	0	0	0	0	0
ENSG00000206749	0	0	0	0	0	0	0	0	0	0	0	0	0	0	0	0	0	0	0	0	0	0	0	0	0	0	0	0	0	0	0	0	0	0	0	0	0	0	0	0	0	0	0	0	0	0	0	0
ENSG00000206751	0.59684703	0.5996664	0.29185284	0.3857472	0	0.89541961	0	0	0	0	0	0.50403686	0.25258863	0.3232939	0	0.28788224	0.44893731	0	0	0	0	0.25487711	0	0	0.28488005	0.49118653	0.2567697	0	0	0	0	0	0	0	0	0	0	0	0	0	0	0	0	0	0	0	0	0.2790009
ENSG00000206752	0	0	0	0	0	0	0	0	0	0	0	0	0	0	0	0	0	0	0	0	0	0	0	0	0	0	0	0	0	0	0	0	0	0	0	0	0	0	0	0	0	0	0	0	0	0	0	0
ENSG00000206754	4.85653613	2.36580719	1.29534685	2.8534728	1.72395952	3.97419113	19.5008095	19.8929753	18.9899613	10.7967475	22.8048432	26.8451415	8.59493379	3.3480848	5.37563442	6.38861957	9.96271835	5.85816267	7.28822846	12.1526608	14.4944989	11.3123539	6.70861309	14.7769767	10.9581256	11.2636472	13.2957462	7.65679148	3.80452078	4.97209907	2.68320998	3.7811806	5.79439388	4.16918018	0.85985823	7.46195342	7.76263458	7.56315819	8.00478226	7.80102445	12.3716219	8.6965099	3.53848138	3.39242581	4.07255284	8.4896888	1.96575188	9.49367449
ENSG00000206755	0.24984294	0.50204629	0.48868382	1.2918047	0.27873543	0.18741341	2.40771094	2.69195469	0.74112118	0.90515368	0.94427314	0.21099218	0.21146955	0.54132933	0.21728755	1.20508845	0.37585449	0.6027426	0	0.88736608	0.63094718	1.06692744	0.2530898	1.39369419	0.47700845	0.82245186	1.07484992	1.6954877	0.71764862	0.40195264	0	0.30567684	1.63949904	0.39321725	0.97317288	0.88898017	1.15600229	1.00704158	0.50331534	0.8026424	0.93346605	1.34216749	0.28605663	0.42660996	0.69138688	0.68632036	0.88992178	2.3358215
ENSG00000206756	0	0.21164697	0.30902065	0	0	0	0	0	0	0	0	0.26684305	0	0	0.27480484	0	0	0	0	0	0	0.53973976	0	0	0	0	0	0.23825481	0	0	0	0	0	0	0	0	0.29240058	0	0	0	0.59028	0	0	0	0.29146702	0	0	0
ENSG00000206758	0	0	0	0	0	0	0	0	0	0.27281501	0	0	0	0	0	0	0	0	0.33148952	0	0	0	0	0	0	0	0	0	0	0	0	0	0	0	0	0	0	0	0	0	0	0	0	0	0	0	0	0.28160839
ENSG00000206759	0	0	0	0	0	0	0	0	0	0	0	0	0	0	0	0	0	0	0	0	0	0	0	0	0	0	0	0	0	0	0	0	0	0	0	0	0	0	0	0	0	0	0	0	0	0	0	0
ENSG00000206760	0	0	0	0	0	0	0	0	0	0	0	0	0	0	0	0	0	0	0	0	0	0	0	0	0	0	0	0	0	0	0	0	0	0	0	0	0	0	0	0	0	0	0	0	0	0	0	0
ENSG00000206761	0	0	0	0	0	0	0	0	0	0	0.18885463	0.12659531	0	0	0	0	0	0	0	0	0	0	0	0	0	0	0	0	0	0	0	0	0	0	0	0	0	0	0	0	0	0	0	0	0	0	0	0
ENSG00000206762	0	0.20175692	0	0	0.33604551	0	0	0	0	0	0	0.25437374	0	0	0	0	0	0	0	0	0	0	0	0	0	0	0	0	0	0	0	0	0	0	0	0	0	0	0	0	0	0	0	0	0	0	0	0
ENSG00000206763	0.90363756	0.40351384	0.88374131	0.3893524	0.16802276	0.225947	0.24189619	0.59008013	0	0.27281501	0	0	0.25494928	0	0	0	0	0	0	0	0	0	0	0.56008271	0	0	0	0.22712141	0.21630064	0.48459711	0.4576503	0	0.49414808	0	0	0	0	0.30352422	0	0	0	0	0.34487202	0	0.27784706	0.41371648	0	0
ENSG00000206764	0	0	0	0	0	0	0	0	0	0	0	0	0	0	0	0	0	0	0	0	0	0	0	0	0	0	0	0	0	0	0	0	0	0	0	0	0	0	0	0	0	0	0	0	0	0	0	0
ENSG00000206765	0	0	0	0	0	0	0	0	0	0	0	0	0	0	0	0	0	0	0	0	0	0	0	0	0	0	0	0	0	0	0	0	0	0	0	0	0	0	0	0	0	0	0	0	0	0	0	0
ENSG00000206766	0	0	0	0	0	0	0	0	0	0	0	0	0	0	0	0	0	0	0	0	0	0	0	0	0	0	0	0	0	0	0	0	0	0	0	0	0	0	0	0	0	0	0	0	0	0	0	0
ENSG00000206767	0	0	0	0	0	0	0	0	0	0	0	0	0	0	0	0	0	0	0	0	0	0	0	0	0	0	0	0	0	0	0	0	0	0	0	0	0	0	0	0	0	0	0	0	0	0	0	0
ENSG00000206768	0	0	0	0	0	0	0.5075077	0	0.31243344	0	0	0.26684305	0	0.34231119	0	0.30481649	0.47534539	1.14343817	0	0	0.26598754	0	0	0	0	0	0.2718738	0	0	0	0	0	0	0	0	0.28107461	0.58480116	0.63680571	0	0	0	0	0	0	0	0.4339967	0.28137233	0.59082544
ENSG00000206769	0	0.59416488	1.15670116	0.7644166	0	0.22180119	0	0	0	0	0	0	0	0	0	0	0	0	0	0	0	0	0	0	0	0.24334012	0	0	0	0	0	0	0	0	0	0	0	0	0	0	0	0	0	0	0	0	0	0
ENSG00000206770	0	0	0	0	0	0	0	0	0	0	0	0	0	0	0	0	0	0	0	0	0	0	0	0	0	0	0	0	0	0	0	0	0	0	0	0	0	0	0	0	0	0	0	0	0	0	0	0
ENSG00000206772	0	0	0	0	0	0	0	0	0	0	0	0	0	0	0	0	0	0	0	0	0	0	0	0	0	0	0	0	0	0	0	0	0	0	0	0	0	0	0	0	0	0	0	0	0	0	0	0
ENSG00000206774	0	0	0	0	0	0	0	0	0	0	0	0	0	0	0	0	0	0	0	0	0	0	0	0	0	0	0	0	0	0	0	0	0	0	0	0	0	0	0	0	0	0	0	0	0	0	0	0
ENSG00000206775	0.48832939	0.32709077	0.95515475	1.2624455	0	1.8315401	4.31381544	6.69651539	3.62138761	6.19207405	4.92166609	4.53633178	4.13326855	0.52902639	2.54819034	1.41324009	3.67312343	0.58904391	1.61224448	1.300798	2.05535824	1.66828654	1.48402653	1.81602576	0.93233469	2.00939944	2.94118023	2.20927185	3.15602292	1.96408675	3.70974107	2.98729636	1.6022377	2.30568298	1.90211062	3.90949234	2.71135083	3.44453996	4.91876351	6.27520424	5.47350546	2.62332737	0.55911069	3.75222855	3.60359221	3.35361083	4.78332957	6.84820393
ENSG00000206776	0	0	0	0	0	0	0	0	0	0	0	0	0	0	0	0	0	0	0	0	0	0	0	0	0	0	0	0	0	0	0	0	0	0	0	0	0	0	0	0	0	0	0	0	0	0	0	0
ENSG00000206777	0	0	0	0	0	0	0	0	0	0	0	0	0	0	0	0	0	0	0	0	0	0	0	0	0	0	0	0	0	0	0	0	0	0	0	0	0	0	0	0	0	0	0	0	0	0	0	0
ENSG00000206778	0.30990134	0	0	0	0	0	0.49774794	0.60710167	0.15321255	0	0.39042063	0.26171145	0	0	0	0	0.59790927	0.93240826	0	0	0	0.26468008	0	0.28811947	0.59167394	0.25503916	0.53329092	0.23367298	0	0.24928793	0.47085175	0.37915685	0.50840235	0	0	0	0	0	0	0.49779264	0	0.55493464	0	0	0.28586188	0.42565061	0.55192264	0.57946341
ENSG00000206779	0.31910633	0	0	0	0	0	0	0	0	0	0	0	0	0	0	0	0	0	0	0	0	0	0	0	0	0	0	0	0	0	0	0	0	0	0	0	0	0	0	0	0	0	0	0	0	0	0	0
ENSG00000206780	0	0	0	0	0	0	0	0	0	0	0	0	0	0	0	0	0	0	0	0	0	0	0	0	0	0	0	0	0	0	0	0	0	0	0	0	0	0	0	0	0	0	0	0	0	0	0	0
ENSG00000206781	0	0	0	0	0	0	0	0	0	0	0	0	0	0	0	0	0	0	0	0	0	0	0	0	0	0	0	0	0	0	0	0	0	0	0	0	0	0	0	0	0	0	0	0	0	0	0	0
ENSG00000206782	0	0	0	0	0	0	0	0	0	0	0	0	0	0	0	0	0	0	0	0	0	0	0	0	0	0	0	0	0	0	0	0	0	0	0	0	0	0	0	0	0	0	0	0	0	0	0	0
ENSG00000206783	0	0	0	0	0	0.30602949	0	0	0	0	0	0.68906305	0	0	0	0	0	0	0	0	0	0	0	0	0	0	0	0	0	0.32817652	0	0.49914319	0	0	0	0	0	0	0	0	0	0	0	0	0	0	0	0.38141895
ENSG00000206784	0	0	0	0	0	0.21394982	0.22905215	0	0	0	0.35932518	0	0	0	0.24805393	0	0	0.34404334	0	0	0.24009494	0	0.57785104	0	0	0.23472631	0.24540821	0	0.40963129	0	0	0	0.46791012	0	0	0	0.28740789	0.57458122	0	0	0	0.5107363	0	0	0.52618824	0	0.2539821	0
ENSG00000206785	0	0.15991104	0.23348227	0	0	0	0.19172513	0	0.11803041	0	0	0	0	0	0	0	0	0	0	0	0.20096836	0.20390169	0.24184136	0.2219587	0	0	0	0.18001474	0.17143828	0	0	0	0	0.37574093	0	0.21236749	0	0.48114209	0	0	0	0	0	0	0.44043905	0	0	0.22320072
ENSG00000206786	0	0.40351384	0	0	0	0.45189401	0	0.59008013	0	0	0	0.50874749	0	0.65263068	0	0	0.45313298	0	0.66297904	0	0	0	0.30512695	0.28004136	0.28754248	0	0.25916942	0	0.21630064	0	0	0	0	0	0.58663225	0	0	0.30352422	0	0	0	0	0	0	0	0.41371648	0.26822409	0
ENSG00000206787	0	0	0	0	0	0	0	0	0	0	0	0	0	0	0	0	0	0	0	0	0	0	0	0	0	0	0	0	0	0	0	0	0	0	0	0	0	0	0	0	0	0	0	0	0	0	0	0
ENSG00000206788	0	0	0	0	0	0	0	0	0	0	0	0	0	0	0	0	0	0	0	0	0	0	0	0	0	0	0	0	0	0	0	0	0	0	0	0	0	0	0	0	0	0	0	0	0	0	0	0
ENSG00000206790	0	0	0	0	0.17454791	0	0	0	0.15470005	0	0	0	0	0	0	0	0.47073038	0	0	0	0	0	0	0	0	0.25751527	0	0.47188331	0	0	0	0	0	0.49247598	0	0	0.28956174	0	0	0	0	0	0	0	0.28863724	0	0	0
ENSG00000206791	0	0	0	0	0	0	0	0	0	0	0	0	0	0	0	0	0	0	0	0	0	0	0	0	0	0	0	0	0	0	0	0	0	0	0	0	0	0	0	0	0	0	0	0	0	0	0	0
ENSG00000206792	0	0	0	0	0	0	0	0	0	0	0	0	0	0	0	0	0	0	0	0	0	0	0	0	0	0	0	0	0	0	0	0	0	0	0	0	0	0	0	0	0	0	0	0	0	0	0	0
ENSG00000206795	0	0	0	0	0	0	0	0	0	0	0.35932518	0	0	0	0	0	0	0	0	0	0	0	0	0	0	0	0	0	0	0	0	0	0	0	0	0	0	0	0	0	0	0	0	0	0	0	0	0
ENSG00000206796	0	0	0	0	0	0	0	0	0	0	0	0	0	0	0	0	0	0	0	0	0	0	0	0	0	0	0	0	0	0	0	0	0	0	0	0	0	0	0	0	0	0	0	0	0	0	0	0
ENSG00000206797	0	0	0	0	0	0	0	0	0	0	0	0	0	0	0	0	0	0	0	0	0	0	0	0	0	0	0	0	0	0	0	0	0	0	0	0	0	0	0	0	0	0	0	0	0	0	0	0
ENSG00000206799	0	0	0	0	0	0	0	0	0	0	0	0	0	0	0	0	0	0	0	0	0	0	0	0	0	0	0	0	0	0	0	0	0	0	0	0	0	0	0	0	0	0	0	0	0	0	0	0
ENSG00000206800	0	0	0	0	0	0	0	0	0	0	0	0	0	0	0	0	0	0	0	0	0	0	0	0	0	0	0	0	0	0	0	0	0	0	0	0	0	0	0	0	0	0	0	0	0	0	0	0
ENSG00000206801	0	0	0	0	0	0	0	0	0	0	0	0	0	0	0	0	0	0	0	0	0	0	0	0	0	0	0	0	0	0	0	0	0	0	0	0	0	0	0	0	0	0	0	0	0	0	0	0
ENSG00000206802	0	0	0	0	0	0.225947	0	0.29504006	0	0	0	0	0	0	0	0	0	0	0	0	0	0	0	0	0	0	0	0	0	0	0	0	0	0	0	0	0	0	0	0	0	0	0	0	0	0	0	0
ENSG00000206803	0	0	0	0	0	0	0	0	0	0	0	0	0.25494928	0.32631534	0	0.29057273	0	0	0	0	0	0	0	0	0	0	0	0	0	0	0	0	0	0	0	0	0	0	0	0	0.56269682	0	0	0.25716208	0	0	0	0
ENSG00000206804	0	0	0	0	0	0	0	0	0	0	0	0	0	0	0	0	0	0	0	0	0	0	0	0	0	0	0	0	0	0	0	0	0	0	0	0	0	0	0	0	0	0	0	0	0	0	0	0
ENSG00000206805	0	0	0	0	0.17625917	0	0	0	0	0	0	0	0	0	0	0	0	0	0	0	0	0.26986988	0	0.29376887	0.60327539	0.26003993	0.2718738	0.71476442	0.22690361	0	1.44025241	0.38659129	0	0.49730417	0.61538873	1.12429845	0.29240058	0.63680571	1.27309173	1.52265985	0.59028	0	0.36177751	0	0	0	0	0.29541272
ENSG00000206806	0	0	0	0	0	0	0	0	0	0	0	0	0	0	0	0.28524112	0	0	0	0	0	0	0	0	0	0	0	0	0	0	0	0	0	0	0	0	0	0	0	0	0	0	0	0	0	0	0	0
ENSG00000206807	0	0	0	0	0	0	0	0	0	0	0	0	0	0	0	0	0	0	0	0	0	0	0	0	0	0	0	0	0	0	0	0	0	0	0	0	0	0	0	0	0	0	0	0	0	0	0	0
ENSG00000206808	0	0	0	0	0	0	0	0	0	0	0	0	0	0	0	0	0	0	0	0	0	0	0	0	0	0	0	0	0	0	0	0	0	0	0	0	0	0	0	0	0	0	0	0	0	0	0	0
ENSG00000206811	0	0	0	0	0	0	0	0	0	0	0	0	0	0	0	0	0	0	0	0	0	0	0	0	0	0	0	0	0	0	0	0	0	0	0	0	0	0	0	0	0	0	0	0	0	0	0	0
ENSG00000206812	0	0	0	0	0	0	0	0	0	0	0	0	0	0	0	0	0	0	0	0	0	0	0	0	0	0	0	0	0	0	0	0	0	0	0	0	0	0	0	0	0	0	0	0	0	0	0	0
ENSG00000206813	0	0	0	0	0	0	0	0	0	0.2861883	0	0	0	0	0	0	0	0	0	0	0	0	0	0	0	0	0	0	0.22690361	0	0	0	0	0	0	0	0	0	0	0	0	0	0	0	0	0	0	0
ENSG00000206814	0	0	0	0	0	0	0	0	0.287101	0	0	0	0.24576191	0	0	0	0	0	0	0	0.24442098	0.24798854	1.17652554	0.26994978	0.27718058	0.95582243	0.74948994	0	0	0	0	0	0	0	0	0	0	0	0	0	0	0	0	0	0	0	0	0
ENSG00000206815	0	0	0	0	0	0	0	0	0	0	0	0	0	0	0	0	0	0	0	0	0	0	0	0	0	0	0	0	0	0	0	0	0	0	0	0	0	0	0	0	0	0	0	0	0	0	0	0
ENSG00000206816	0	0	0	0	0	0	0	0	0	0	0	0	0	0	0	0	0	0	0	0	0	0	0	0	0	0	0	0	0	0	0	0	0	0	0	0	0	0	0	0	0	0	0	0	0	0	0	0
ENSG00000206817	0	0	0	0	0	0	0	0	0	0	0	0	0	0	0	0	0	0	0	0	0	0	0	0	0	0	0	0	0	0	0	0	0	0	0	0	0	0	0	0	0	0	0	0	0	0	0.28699977	0
ENSG00000206818	0	0	0	0	0	0	0	0	0	0	0	0	0	0	0	0	0	0	0	0	0	0	0	0	0	0	0	0	0	0	0	0	0	0	0	0	0	0	0	0	0	0	0	0	0	0	0	0
ENSG00000206819	0	0	0	0	0	0	0	0	0	0	0	0	0	0	0	0	0	0	0	0	0	0	0	0	0	0	0	0	0	0	0	0	0	0	0	0	0	0	0	0	0	0	0	0	0	0	0	0
ENSG00000206820	0	0	0	0.2540287	0	0	0	0	0	0	0	0	0	0	0	0	0	0	0	0	0	0	0	0	0.18760393	0	0	0	0	0	0	0	0	0	0	0	0	0	0	0	0	0	0	0	0	0	0	0
ENSG00000206822	0	0	0	0	0	0	0	0	0	0	0	0	0	0	0	0	0	0	0	0	0	0	0	0	0	0	0	0	0	0	0	0	0	0	0	0	0	0	0	0	0	0	0	0	0	0	0	0
ENSG00000206824	0	0	0	0	0	0.71106851	0	0.30950281	0.31243344	1.14475319	0.39807593	0.53368609	0.80234037	0.68462238	0	0.30481649	0.95069077	1.14343817	1.04321702	0.56112855	0.53197507	1.07947953	0.96025246	0.88130662	0.30163769	1.04015971	1.08749521	0.71476442	1.13451804	0.7625278	0.96016828	0.38659129	1.55511306	0	1.23077746	0.84322384	1.4620029	0	0	1.52265985	0	0.56581571	0.72355502	0.53953613	1.16586807	0.86799339	0.56274466	0.88623816
ENSG00000206826	0	0	0	0	0	0	0	0	0	0	0	0	0	0	0	0	0	0	0	0	0	0	0	0	0	0	0	0	0	0	0	0	0	0	0	0	0	0	0	0	0	0	0	0	0	0	0	0
ENSG00000206827	0	0	0	0	0	0	0	0	0	0	0	0	0	0	0	0	0	0	0	0	0	0	0	0	0	0	0	0	0	0	0	0	0	0	0	0	0	0	0	0	0	0	0	0	0	0	0	0
ENSG00000206828	0	0	0	0	0	0	0	0	0	0	0	0	0	0	0	0	0	0	0	0	0	0	0	0	0	0	0	0	0	0	0	0	0	0	0	0	0	0	0	0	0	0	0	0	0	0	0	0
ENSG00000206832	0.30121252	0.20175692	0.29458044	0	0.33604551	0	0.24189619	1.47520032	0.59566749	0	1.51789702	0.76312123	1.27474638	5.5473608	2.88159843	1.45286364	2.26566492	4.72336141	0.99446856	0.53490759	0.50711643	1.02903656	0.30512695	0.84012407	0.57508495	0	0.25916942	0	0.21630064	0.24229855	0	0.36852628	1.48244423	0.47406566	0	0	0.557474	0.60704843	0.60680073	1.45150752	0	0.53937572	2.0692321	3.08594498	3.88985888	1.65486591	2.41401679	1.40804193
ENSG00000206833	0	0	0	0	0	0	0	0	0	0	0	0	0	0	0	0	0	0	0	0	0	0	0	0	0	0	0	0	0	0	0	0	0	0	0	0	0	0	0	0	0	0	0	0	0	0	0	0
ENSG00000206834	0	0	0	0	0	0	0	0	0	0	0	0	0	0	0	0	0	0	0	0	0	0	0	0	0	0	0	0	0	0	0	0	0	0	0	0	0	0	0	0	0	0	0	0	0	0	0	0
ENSG00000206835	0	0	0	0	0	0	0	0	0	0	0	0	0	0	0	0	0	0	0	0	0	0	0	0	0	0	0	0	0	0	0	0	0	0	0	0	0	0	0	0	0	0	0	0	0	0	0	0
ENSG00000206836	0	0	0	0	0	0	0	0	0	0	0	0	0	0	0	0	0	0	0	0.26745379	0	0	0	0	0	0	0	0	0	0	0	0	0	0	0	0	0	0	0	0	0	0	0	0	0	0	0	0
ENSG00000206838	58.4464684	48.0091133	49.1619574	79.590595	46.4219292	66.9359567	16.9977205	19.3185188	18.1934189	27.8841373	21.8169377	17.671382	4.27515688	3.90847853	5.0203153	5.33656331	1.80915035	3.77163933	5.55863395	11.1053202	14.3752369	9.03862707	9.50219973	10.06268	22.7308764	27.3158359	23.5921536	23.3951997	22.9714504	26.5063767	8.03961796	10.5937554	6.31329482	10.5992591	7.96331388	12.837139	57.6465562	40.4751807	36.8246534	32.066762	31.4522329	21.5347769	4.40612607	7.80314196	6.65588859	6.27675819	9.42387318	6.07139274
ENSG00000206839	0	0	0	0	0	0	0	0	0	0	0	0	0	0	0	0	0	0	0	0	0	0	0	0	0	0	0	0	0	0	0	0	0	0	0	0	0	0	0	0	0	0	0	0	0	0	0	0
ENSG00000206840	0	0	0	0	0	0	0	0	0	0	0	0	0	0	0	0	0	0	0	0	0	0	0	0	0	0	0	0	0	0	0	0	0	0	0	0	0	0	0	0	0	0	0	0	0	0	0	0
ENSG00000206841	0	0	0	0	0	0	0	0	0	0	0	0	0	0	0	0	0	0	0	0	0	0	0	0	0	0	0	0	0	0	0	0	0	0	0	0	0	0	0	0	0	0	0	0	0	0	0	0
ENSG00000206842	0	0	0	0	0	0	0	0	0	0	0	0	0	0	0	0	0	0	0	0	0	0	0	0	0	0	0	0	0	0	0	0	0	0	0	0	0	0	0	0	0	0	0	0	0	0	0	0
ENSG00000206843	0	0	0	0	0	0	0	0	0	0	0	0	0	0	0	0	0	0	0	0	0	0	0	0	0	0	0	0	0	0	0	0	0	0	0	0	0	0	0	0	0	0	0	0	0	0	0	0
ENSG00000206844	0	0	0	0	0	0	0	0	0	0	0	0	0	0	0	0	0	0	0	0	0	0	0	0	0	0	0	0	0	0	0	0	0	0	0	0	0	0	0	0	0	0	0	0	0	0	0	0
ENSG00000206845	0	0	0	0	0	0	0	0	0	0	0	0	0	0	0	0	0	0	0	0	0	0	0	0	0	0	0	0	0	0	0	0	0	0	0	0	0	0	0	0	0	0	0	0	0	0	0	0
ENSG00000206846	0.68573914	0.68897842	0.33532028	0	0.19125995	1.02877997	0	1.34337391	0.16951176	0.31054475	0	0	0	0	0.29819249	0.33075832	0.51580031	1.24075206	0.37733381	0	0	0	0.69465072	0.63754096	0	0	0	1.55119087	0.49242911	0	0	0	0	0	2.67104896	0.60999171	0	0	1.03607997	0	0.6405166	0.61397024	0.39256708	0	0	0	0.30531891	0
ENSG00000206847	0	0	0	0	0	0	0	0	0	0	0	0	0	0	0	0	0	0	0	0	0	0	0	0	0	0	0	0	0	0	0	0	0	0	0	0	0	0	0	0	0	0	0	0	0	0	0	0
ENSG00000206848	0	0.20175692	0	0.7787047	0.33604551	0	1.20948096	0.59008013	0.89350124	0.81844504	0.75894851	0.76312123	0.50989855	0.32631534	0.78589048	0.29057273	0	0.36333549	0.33148952	0	0.25355821	0.25725914	1.8307617	0.28004136	0.28754248	0.24788853	1.2958471	0.45424281	1.08150318	0.72689566	0	0.36852628	1.48244423	0.47406566	0	0.26794029	0.557474	0.30352422	0.30340037	0	0	0	0.34487202	0.51432416	0.83354119	0	0.53644818	0.28160839
ENSG00000206849	0	0	0	0	0	0	0	0	0	0	0	0	0	0	0	0	0	0	0	0	0	0	0	0	0	0	0	0	0	0	0	0	0	0	0	0	0	0	0	0	0	0	0	0	0	0	0	0
ENSG00000206850	0	0	0	0	0	0	0	0	0	0	0	0	0	0	0	0	0	0	0	0	0	0	0	0	0	0	0	0	0	0	0	0	0	0	0	0	0	0	0	0	0	0	0	0	0	0	0	0
ENSG00000206852	0	0	0	0	0	0	0	0	0	0	0	0	0	0	0	0	0	0	0	0	0	0	0	0	0	0	0	0	0	0	0	0	0	0	0	0	0	0	0	0	0	0	0	0	0	0	0	0
ENSG00000206853	0	0	0	0	0	0	0	0	0	0	0	0	0	0	0	0	0	0	0	0	0	0	0	0	0	0	0	0	0	0	0	0	0	0	0	0	0	0	0	0	0	0	0	0	0	0	0	0
ENSG00000206854	0	0	0	0	0	0	0	0	0	0	0	0	0	0	0	0	0	0	0	0	0	0	0	0	0	0	0	0	0	0	0	0	0	0	0	0	0	0	0	0	0	0	0	0	0	0	0	0
ENSG00000206855	0	0	0	0	0	0	0	0	0	0	0	0	0	0	0	0	0	0	0	0	0	0	0	0	0	0	0	0	0	0	0	0	0	0	0	0	0	0	0	0	0	0	0	0	0	0	0	0
ENSG00000206856	0	0	0	0	0	0	0	0	0	0	0	0	0	0	0	0	0	0	0	0	0	0	0	0	0	0	0	0	0	0	0	0	0	0	0	0	0	0	0	0	0	0	0	0	0	0	0	0
ENSG00000206857	0	0	0	0	0	0	0	0	0	0	0	0	0	0	0	0	0	0	0	0	0	0	0	0	0	0	0	0	0	0	0	0	0	0	0	0	0	0	0	0	0	0	0	0	0	0	0	0
ENSG00000206858	0	0	0	0	0	0	0	0	0	0	0	0	0	0	0	0	0	0	0	0	0	0	0	0	0	0	0	0	0	0	0	0	0	0	0	0	0	0	0	0	0	0	0	0	0	0	0	0
ENSG00000206859	0	0.80702768	0.88374131	0.3893524	0	0	0.96758477	0.59008013	0	1.63689007	0.37947425	1.01749498	0.25494928	0	0.78589048	0.29057273	0.90626597	0.36333549	0.33148952	0	0.50711643	0.51451828	0.91538085	0.84012407	0.57508495	0.74366559	0	0	0.64890191	0.48459711	0	0	0	0	0	0.53588057	0.278737	0.91057265	0.60680073	0.96767168	0	0.53937572	0	0.51432416	0	0.41371648	0.26822409	0
ENSG00000206862	0	0	0	0	0	0	0	0	0	0	0	0	0	0	0	0	0	0	0	0	0	0	0	0	0	0	0	0	0	0	0	0	0	0	0	0	0	0	0	0	0	0	0	0	0	0	0	0
ENSG00000206863	0	0	0	0	0	0	0	0	0	0	0	0	0	0	0	0	0	0	0	0	0	0	0	0	0	0	0	0	0	0	0	0	0	0	0	0	0	0	0	0	0	0	0	0	0	0	0	0
ENSG00000206864	0	0.40351384	0	0	0	0	0	0	0	0	0	0	0	0	0	0	0	0	0	0	0	0	0	0	0	0	0	0	0	0	0	0	0	0	0	0	0	0	0	0	0	0	0	0	0	0	0	0
ENSG00000206865	0.31910633	0	0	0	0	0	0	0	0	0	0	0	0	0.34570041	0	0	0	0	0	0	0	0	0	0	0	0	0	0	0.45830036	0	0	0	0	0	0	0.28385753	0.59059127	0	0	0	0	0	0.36535946	0.27243904	0	0	0	0
ENSG00000206866	0	0	0	0	0	0	0	0	0	0	0	0	0	0	0	0	0	0	0	0	0	0	0	0	0	0	0	0	0	0	0	0	0	0	0	0	0	0	0	0	0	0	0	0	0	0	0	0
ENSG00000206867	0	0	0	0	0	0	0	0	0	0	0	0	0	0	0	0	0	0	0	0	0	0	0	0	0	0	0	0	0	0	0	0	0	0	0	0	0	0	0	0	0	0	0	0	0	0	0	0
ENSG00000206869	0.23873881	0.15991104	0	0	0.26634719	0.17908392	0	0.46769314	0.35409123	0	0	0	0	0	0	0	0	0	0	0.2119819	0	0.20390169	0.24184136	0.2219587	0	0.19647461	0.20541576	0	0	0.19204404	0.36273024	0	0.3916581	0.75148186	0	0.21236749	0	0	0	0	0	0	0	0	0	0	0	0
ENSG00000206870	0	0	0	0	0.16493977	0	0	0	0	0	0	0	0	0	0	0	0	0.35666879	0	0	0	0	0	0	0	0	0	0	0	0	0	0	0	0	0.57586835	0	0	0.29795496	0	0	0	0	0	0	0.27274895	0	0	0
ENSG00000206871	0	0	0	0	0	0	0	0	0	0	0	0	0	0	0	0	0	0	0	0	0	0	0	0	0	0	0	0	0	0	0	0	0	0	0	0	0	0	0	0	0	0	0	0	0	0	0	0
ENSG00000206875	0	0	0	0	0	0	0.24189619	0.29504006	0	0	0.37947425	0.25437374	0	0	0	0	0	0	0	0	0	0.25725914	0	0.28004136	0	0	0.25916942	0.22712141	0	0.24229855	0	0	0	0	0	0	0	0.30352422	0	0.48383584	0	0	0	0	0.27784706	0	0.26822409	0
ENSG00000206877	0	0	0	0	0	0	0	0	0	0	0	0	0	0	0	0	0	0	0	0	0	0	0	0	0	0	0	0	0	0	0	0	0	0	0	0	0	0	0	0	0	0	0	0	0	0	0	0
ENSG00000206878	0.51567584	0.17270392	0.75648256	0.9998569	0.28765496	0.96705318	1.86356827	2.27298865	1.65714697	1.8682372	0.64965992	0.65323178	0	0.27932593	0	0.49746051	0	0.31101518	0.85126508	0.91576179	0.21704583	0.22021382	0	0	0	0	0.22184902	0.19441592	0.37030669	0.41481512	0	0	0	0	0.5021572	0	0.23859887	0.77945019	1.55826428	0.41416348	0.48166848	1.38511685	0	0	0	0.3541413	0	0.24105678
ENSG00000206880	0	0	0	0	0	0	0	0	0	0	0	0	0	0	0	0	0	0	0	0	0	0	0	0	0	0	0	0	0	0	0	0	0	0	0	0	0	0	0	0	0	0	0	0	0	0	0	0
ENSG00000206881	0	0	0	0	0	0	0	0	0	0	0	0	0	0	0	0	0	0	0	0	0	0	0	0	0	0	0	0	0	0	0	0	0	0	0	0	0	0	0	0	0	0	0	0	0	0	0	0
ENSG00000206882	0	0	0	0	0	0	0	0	0	0	0	0	0	0	0	0	0	0	0	0	0	0	0	0	0	0	0	0	0	0	0	0	0	0	0	0	0	0	0	0	0	0	0	0	0	0	0	0
ENSG00000206885	0.47050715	2.04849546	3.22103279	0.3040927	2.09967124	2.11763469	3.02281958	1.843462	3.2566055	2.13074498	5.03842094	4.17210076	1.7920887	0.76457828	2.04599225	0.68083099	4.60078818	2.27018381	0.51780115	0.41777454	3.16855226	2.61202528	1.42986498	3.93693176	3.14407758	2.71048917	2.4290039	0.70954716	0.5068066	1.13544285	1.42973962	0.57565419	2.70158328	0.74051132	0.45817263	2.72047399	3.91859464	7.58588988	4.97620893	3.02309109	2.63687124	2.94884974	1.61611558	1.00424608	1.30202784	1.61560814	1.88539998	2.19942316
ENSG00000206886	0	0	0	0	0	0	0	0	0	0	0	0	0	0	0	0	0	0	0	0	0	0	0	0	0	0	0	0	0	0	0	0	0	0	0	0	0	0	0	0	0	0	0	0	0	0	0	0
ENSG00000206887	0	0	0	0	0	0	0	0	0	0	0	0	0	0	0	0	0	0	0	0	0	0	0	0	0	0	0	0	0	0	0	0	0	0	0	0	0	0	0	0	0	0	0	0	0	0	0	0
ENSG00000206888	0	0	0.29458044	0	0.16802276	0	0.48379239	0.59008013	0.29783375	1.09126005	0.75894851	0	0.25494928	0	0.52392699	0	0	0	0.33148952	0	0	0	0	0.28004136	0	0	0.25916942	0.45424281	0.21630064	0	0	0.73705256	0	0.94813132	1.1732645	0.26794029	0.557474	0	0.30340037	0	0	0	0.34487202	0	0	0	0	0.28160839
ENSG00000206889	0	0	0	0	0	0	0	0	0	0	0	0	0	0	0	0	0	0	0	0	0	0	0	0	0	0	0	0	0	0	0	0	0	0	0	0	0	0	0	0	0	0	0	0	0	0	0	0
ENSG00000206891	0	0	0	0	0	0	0	0	0	0	0	0	0	0	0	0	0	0	0	0	0	0	0	0	0	0	0	0	0	0	0	0	0	0	0	0	0	0	0	0	0	0	0	0	0	0	0	0
ENSG00000206892	0	0.40351384	0.29458044	0.3893524	0	0	0	0.59008013	0	0	0	0.50874749	0.25494928	1.30526136	0.52392699	0.58114546	0	1.45334197	0.33148952	0.26745379	0.25355821	0	0	0.28004136	1.1501699	0.74366559	0	1.36272843	0	0.24229855	1.3729509	1.47410512	1.48244423	0.47406566	1.75989675	0.53588057	1.11494801	0.30352422	0.60680073	0	0.56269682	0	1.72436009	0.25716208	0.83354119	0.41371648	0.53644818	0.56321677
ENSG00000206895	0	0	0	0	0	0	0	0	0	0	0	0	0	0	0	0	0	0	0	0	0	0	0	0	0	0	0	0	0	0	0	0.38659129	0	0	0	0	0	0	0	0	0	0	0	0	0	0	0	0
ENSG00000206896	0	0	0	0	0	0	0	0	0	0	0	0	0	0	0	0	0	0	0	0	0	0	0	0	0	0	0	0	0	0	0	0	0	0	0	0	0	0	0	0	0	0	0	0	0	0	0	0
ENSG00000206897	0.72698661	0.32463144	0.23699328	0	0.1351762	0.54533074	1.36225751	1.66154141	1.91688487	1.75586203	2.44233054	1.43252582	0.82043827	0.26252437	0.63225775	0.46753807	0.72910119	0.584615	0.53337411	0.64550878	1.83591397	1.65574303	1.47286844	0.90118572	1.85064932	1.1965747	1.87654249	0.73088693	0.69606521	1.75438726	0.36818483	0.29648355	0.3975477	1.14417351	0.47195226	1.07780491	2.01822355	1.70932058	1.22044508	1.94625695	1.35808782	0	0.27745343	1.24133877	0.67059329	1.33135829	0.43157861	0.22655712
ENSG00000206898	0	0	0	0	0	0	0	0	0	0	0	0	0	0	0	0	0	0	0	0	0	0	0	0	0.22960481	0	0.20694872	0	0.17271767	0	0	0	0	0	0	0	0	0	0	0	0	0	0	0	0	0	0	0
ENSG00000206899	0	0.40351384	0.58916087	0	1.00813654	0	0.24189619	0	0.44675062	0.27281501	0.37947425	0.50874749	1.78464493	13.378929	9.43068576	2.90572728	3.62506387	7.99338084	1.32595808	1.06981518	1.01423285	1.80081397	0	0.56008271	0.28754248	0.99155411	0.51833884	0.90848562	0.43260127	0.72689566	1.8306012	0	0	0	0	1.875582	0.557474	0.30352422	1.21360146	1.93534336	1.68809047	0.53937572	13.7948807	8.22918661	11.3917296	3.30973181	4.82803358	4.78734256
ENSG00000206900	0	0	0	0	0	0	0	0	0	0	0	0	0	0.32631534	0	0	0	0	0	0	0	0	0	0	0	0	0.25916942	0	0	0	0	0	0	0	0	0	0	0	0	0	0	0	0	0	0	0	0	0
ENSG00000206901	0	0	0	0	0	0	0	0	0	0	0	0	0	0	0	0	0	0	0	0	0	0	0	0	0	0	0	0	0	0	0	0	0	0	0	0	0	0	0	0	0	0	0	0	0	0	0	0
ENSG00000206902	0	0	0	0	0	0	0	0	0	0	0	0	0	0	0	0	0	0	0	0	0	0	0	0	0	0	0	0	0	0	0	0	0	0	0	0	0	0	0	0	0	0	0	0	0	0	0	0
ENSG00000206903	0	0.49438146	0.2406115	1.2720825	0.13723996	0	0.19757933	0.96394769	0.24326879	0.44566727	0.30995225	0.41554184	0	0	0	0.94935212	0	0	0.27075861	0.43690925	0.2071048	0.42025539	0.24922583	0.45747214	0.23486294	0.40494767	0.635064	0.37102275	0.53001912	0.19790798	1.49522388	0.30101001	0	0.38721393	0.95831528	0.43770398	0.22767068	0.49583345	0.49563113	0	0.45960733	0.44055879	0.28168936	0.42009684	0.22694378	1.01376327	0	0
ENSG00000206905	0.30990134	0	0	0	0	0.23246471	0	0.60710167	0.45963766	0	0	0	0	0.33572828	0.26952013	0	0	0	0	0.27516881	0.52174478	0.52936015	0	0.28811947	0	0.51007832	0	0	0	0	1.883407	0.37915685	1.52520704	0	0.60355433	0.27566933	0.86033248	0.93683917	1.5607615	0.49779264	0	0	0	0	0.85758565	0.42565061	0.27596132	0
ENSG00000206906	0	0	0	0	0	0	0	0	0	0	0	0	0	0	0	0	0	0	0	0	0	0	0	0	0	0	0	0	0	0	0	0	0	0	0	0	0	0	0	0	0	0	0	0	0	0	0	0
ENSG00000206907	0	0.20175692	0	0	0	0	0	0	0	0	0	0	0	0	0.26196349	0	0.45313298	0	0	0	0	0	0	0	0	0	0	0	0	0	0.9153006	0	0	0	0	0	0	0	0	0	0	0	0	0	0	0	0	0
ENSG00000206908	0	0.13163409	0	0	0.1096246	0.29483329	0	0	0.09715918	0	0	0	0	0.21290086	0	0	0	0	0.2162767	0	0.16543127	0	0	0	0.18760393	0	0	0.14818287	0	0	0.29858892	0.24044093	0	0	0	0.1748147	0.1818589	0.19803104	0	0.94702015	1.46850146	0	0	0	0.18127827	0	0.34999972	0.1837323
ENSG00000206909	0	0	0	0	0	0	0	0	0	0	0	0	0	0	0	0	0	0	0	0	0	0	0	0	0	0	0	0	0	0	0	0	0	0	0	0	0	0	0	0	0	0	0	0	0	0	0	0
ENSG00000206910	0	0	0	0	0	0	0	0	0	0	0	0	0	0	0	0	0	0	0	0	0	0	0	0	0	0	0	0	0	0	0	0	0	0	0	0	0	0	0	0	0	0	0	0	0	0	0	0
ENSG00000206911	0	0.19104416	0	0	0	0	0	0	0	0	0	0	0	0	0	0	0	0	0	0	0	0	0	0	0	0	0	0	0	0	0	0	0	0	0	0	0	0	0	0	0	0	0	0	0	0	0	0
ENSG00000206912	0	0	0	0	0	0	0	0	0	0	0	0	0	0	0	0	0	0	0	0	0	0	0	0	0	0	0	0	0	0	0	0	0	0	0	0	0	0	0	0	0	0	0	0	0	0	0	0
ENSG00000206913	0	0	0	0	0	0	0	0	0	0	0	0	0	0	0	0	0	0	0	0	0	0	0	0	0	0	0	0	0	0	0	0	0	0	0	0	0	0	0	0	0	0	0	0	0	0	0	0
ENSG00000206914	0.31597784	0.42329393	0.30902065	0.4084383	0.17625917	0.23702284	0.25375385	0.92850844	0.93730032	1.71712978	0.39807593	0.53368609	0.26744679	0.68462238	0.54960968	0.30481649	0.47534539	0.76229211	0	0.28056427	1.06395015	0.80960964	0	0.88130662	0.60327539	0.78011978	0.81562141	0.23825481	0.90761444	0.50835187	0.96016828	0	1.03674204	0.99460834	0	0.28107461	1.16960232	0.63680571	0.9548188	0	1.18056	0.56581571	0	0.53953613	0	0.4339967	0.56274466	0.59082544
ENSG00000206915	0	0	0	0	0	0	0	0	0	0	0	0	0	0	0	0	0	0	0	0	0	0	0	0	0	0	0	0	0	0	0	0	0	0	0	0	0	0	0	0	0	0	0	0	0	0	0	0
ENSG00000206917	0	0	0	0	0	0	0	0	0	0.17799516	0	0.16596336	0	0	0	0	0	0.23705425	0.4325534	0	0.33086255	0	0.19907673	0	0	0	0	0	0	0	0	0	0	0	0	0	0	0.19803104	0	0	0	0	0	0	0	0	0	0
ENSG00000206918	0	0	0	0	0	0	0	0	0	0	0	0	0	0	0	0	0	0	0	0	0	0	0	0	0	0	0	0	0	0	0	0	0	0	0	0	0	0	0	0	0	0	0	0	0	0	0	0
ENSG00000206920	0	0	0	0	0	0	0	0	0	0	0	0	0	0	0	0	0	0	0	0	0	0	0	0	0	0	0	0	0	0	0	0	0	0	0	0	0	0	0	0	0	0	0	0	0	0	0	0
ENSG00000206921	0.30121252	0.40351384	1.47290218	1.5574094	0.33604551	0.225947	0.24189619	0.59008013	0.74458437	0.27281501	0.37947425	0.76312123	0	0	0	0	0	0	0.66297904	0	0.25355821	1.02903656	0.30512695	0.28004136	0.28754248	0.24788853	0.25916942	0	0.43260127	0.48459711	0	0	0	0	0	0	0.278737	0.30352422	0.60680073	0	0.56269682	0.53937572	0	0	0	0	0.26822409	0
ENSG00000206922	0	0.20175692	0.29458044	0	0.16802276	0.225947	0	0.29504006	0	0	0.37947425	0	0.25494928	0.32631534	0.78589048	0	0	0	0	0	0.25355821	0.25725914	0	0.28004136	0	0	0	0	0.43260127	0	0	0	0.49414808	0.47406566	0	0.53588057	0	0	0.30340037	0	0	0.53937572	0.34487202	0.77148624	0	0	0.26822409	0
ENSG00000206923	0	0	0	0	0	0	0	0	0	0	0	0	0	0	0	0	0	0	0	0	0	0	0	0	0	0	0	0	0	0	0	0	0	0	0	0	0	0	0	0	0	0	0	0	0	0	0	0
ENSG00000206924	0	0	0	0.3893524	0	0.225947	0	0	0	0	0	0	0	0	0	0	0	0	0	0	0	0	0	0	0	0	0	0	0	0	0	0	0	0	0	0	0	0	0	0	0	0	0	0	0.27784706	0	0	0
ENSG00000206925	0	0	0	0	0	0	0	0	0	0	0	0	0	0	0	0	0	0	0	0	0	0	0	0	0	0	0	0	0	0	0	0	0	0	0	0	0	0	0	0	0	0	0	0	0	0	0	0
ENSG00000206926	0	0	0	0	0	0	0	0	0	0	0	0	0	0	0	0	0	0	0	0.26745379	0	0	0	0	0	0	0	0	0	0	0	0	0	0	0	0	0	0	0	0	0	0	0	0	0	0	0	0
ENSG00000206927	0	0	0	0	0	0	0	0	0	0	0	0	0	0	0	0	0	0	0	0	0	0	0	0	0	0	0	0	0	0	0	0	0	0	0	0	0	0	0	0	0	0	0	0	0	0	0	0
ENSG00000206929	0	0	0	0	0	0	0	0	0	0	0	0	0	0	0	0	0	0	0	0	0	0	0	0	0	0	0	0	0	0	0	0	0	0	0	0	0	0	0	0	0	0	0	0	0	0	0	0
ENSG00000206931	0	0	0	0	0	0	0	0	0	0	0	0	0	0	0	0	0	0	0	0	0	0	0	0	0	0	0	0	0	0	0	0	0	0	0	0	0	0	0	0	0	0	0	0	0	0	0	0
ENSG00000206932	0	0.80702768	0	0	0.50406827	0.225947	0	0	0	0	0	0	0	0.32631534	0.26196349	0	0	0	0	0	0	0	0.30512695	0	0	0.24788853	0	0	0	0	0	0	0	0	0	0.26794029	0	0	0.30340037	0	0	0	0.68974403	0	0.27784706	0	0	0
ENSG00000206935	0	0	0	0	0	0	0	0	0	0	0	0	0	0	0	0	0	0	0	0	0	0	0	0	0	0	0	0	0	0	0	0	0	0	0	0	0	0	0	0	0	0	0	0	0	0	0	0
ENSG00000206936	0	0	0	0	0	0	0	0	0	0	0.29423004	0	0	0.25301262	0	0	0	0	0	0	0.19659948	0	0	0	0	0.19220342	0	0	0	0	0	0	0	0	0	0	0	0	0	0	0	0	0.26740077	0	0	0	0.20797085	0
ENSG00000206937	0	0.15991104	0.23348227	0	0.13317359	0.89541961	0	0	0.35409123	0	0.30076848	0	0	0	0	0	0	0.28797702	0	0	0	0.20390169	0.24184136	0	0.22790404	0.19647461	0.41083152	0.36002949	0.34287656	0.38408808	0	0.2920912	0.3916581	0.37574093	0	0	0.22092488	0.72171313	0.24047288	0	0	0	0	0	0	0	0	0.44640144
ENSG00000206938	0	0	0	0	0	0	0	0	0	0	0	0	0	0	0	0	0	0	0	0	0	0	0	0	0	0	0	0	0	0	0	0	0	0	0	0	0	0	0	0	0	0	0	0	0	0	0	0
ENSG00000206939	0	0	0	0	0	0	0	0	0	0	0	0	0	0	0	0	0	0	0	0	0	0	0	0	0	0	0	0	0	0	0	0	0	0	0	0	0	0	0	0	0	0	0	0	0	0	0	0
ENSG00000206941	0.21776851	0.72932401	0.21297369	0.2814912	0.48590365	1.63353577	3.14791938	1.27983595	1.722606	2.9585682	2.46914667	2.57467479	1.29025005	1.41550303	3.03028041	0.84030492	2.94842611	1.31340871	2.15692167	4.44732289	1.83315735	2.60387967	2.42658392	2.02462331	1.66308351	3.58433413	2.24846983	0.98521582	2.03293368	1.22622713	1.32347519	0.53286908	1.07176711	1.02820998	0.42411926	2.13084943	2.61975114	1.97495824	3.94830476	1.74900118	2.03407297	1.94977034	1.49599889	0.92960617	1.00437959	1.19642332	0.96959383	3.05392878
ENSG00000206944	0	0	0	0	0	0	0	0	0	0	0	0	0	0	0	0	0	0	0.33148952	0	0	0	0	0	0	0	0	0	0	0	0	0	0	0	0	0	0	0	0	0	0	0	0	0	0	0	0	0
ENSG00000206946	0	0	0	0	0	0	0	0	0	0	0	0	0	0	0	0	0	0	0	0	0	0	0	0	0	0	0	0	0	0	0	0	0	0	0	0	0	0	0	0	0	0	0	0	0	0	0	0
ENSG00000206947	0	0	0	0	0	0	0	0	0	0	0	0	0	0	0	0	0	0	0	0	0	0	0	0	0	0	0	0	0	0	0	0	0	0	0	0	0	0	0	0	0	0	0	0	0	0	0	0
ENSG00000206948	0	0	0	0	0	0	0.19608252	0	0	0.2211455	0	0.2061969	0	0	0	0	0	0	0	0	0	0	0	0	0.46616735	0	0.2100843	0	0	0.19640868	0	0.29872964	0	0	0	0	0	0	0	0	0.45612545	0	0	0	0.22522451	0.33536108	0	0
ENSG00000206949	0	0	0	0	0	0	0	0	0.14891687	0	0	0	0	0	0	0	0	0	0	0	0	0	0	0	0	0	0	0	0	0	0	0	0	0	0	0	0	0	0	0	0	0	0	0	0	0	0	0
ENSG00000206950	0	0	0	0	0	0	0	0	0	0	0	0	0	0	0	0	0	0	0	0	0	0	0	0	0	0	0	0	0	0	0	0	0	0	0	0	0	0	0	0	0	0	0	0	0	0	0	0
ENSG00000206951	0	0	0	0	0	0	0	0.27937422	0	0	0	0	0	0	0	0	0	0	0	0	0	0	0	0	0	0	0.24540821	0	0	0	0	0.34895851	0	0	0.55548363	0	0	0	0	0	0	0	0	0	0	0.39174923	0	0
ENSG00000206952	4.26570085	2.69849882	2.08588941	4.9012591	3.04047063	4.79971245	0.38063077	0.46425422	1.52311302	0.85856489	3.88124035	1.20079371	1.20351055	5.13466787	3.09155446	2.7433484	4.99112655	11.1485221	2.60804254	0.21042321	0	0.60720723	1.20031558	0.88130662	2.48851098	0.97514973	2.44686422	2.68036658	2.38248789	2.09695144	9.00157759	6.37875634	5.05411745	8.57849695	4.61541548	6.74579071	2.85090566	5.01484494	1.6709329	3.0453197	3.09897	2.1218089	8.13999393	5.26047728	8.96261077	3.90597027	5.48676039	2.88027401
ENSG00000206954	0	0.80702768	0.58916087	0.3893524	0.33604551	0.67784101	0.24189619	0	0.59566749	2.18252009	0.75894851	1.78061621	0.50989855	0.65263068	1.04785397	0.58114546	0.45313298	0.36333549	0.33148952	0.26745379	1.26779106	1.54355484	2.13588865	0.28004136	0.57508495	0	0.51833884	0	0.21630064	0.48459711	0	0	1.48244423	0.94813132	1.75989675	0	0.557474	0.60704843	0.9102011	0.48383584	1.12539365	0	0.34487202	0.51432416	0.27784706	0	0.26822409	0.56321677
ENSG00000206957	0	0	0	0	0	0	0	0.32213558	0	0	0	0	0	0	0.28602137	0	0	0.39670304	0	0	0	0	0	0	0	0	0.28297069	0	0	0	0	0	0.53952902	1.0352046	0.64050664	0	0.3043353	0	0	0	0	0	0	0	0	0	0	0
ENSG00000206958	0.23698338	0	0.23176549	0.3063287	0	0	0	0	0	0	0	0	0	0	0	0	0	0	0	0	0	0	0	0	0	0	0	0	0	0	0	0	0	0	0.46154155	0	0	0	0	0	0	0	0	0	0	0	0	0
ENSG00000206959	0	0	0	0	0	0	0	0	0	0	0	0	0	0	0	0	0	0	0	0	0	0	0	0	0	0	0	0	0	0	0	0	0	0	0	0	0	0	0	0	0	0	0	0	0	0	0	0
ENSG00000206960	0	0	0	0	0	0	0	0	0	0	0	0	0	0	0	0	0	0	0	0	0	0	0	0	0	0	0	0	0	0	0	0	0	0	0	0	0	0	0	0	0	0	0	0	0	0	0	0
ENSG00000206961	0	0	0	0.3156114	0	0.18315401	0	0	0	0	0	0	0	0	0	0	0	0	0	0	0	0.20853582	0	0	0	0	0.2100843	0	0.17533461	0	0	0	0	0.3842805	0	0	0	0	0	0.39220027	0	0	0	0	0	0	0	0
ENSG00000206962	0	0	0	0	0	0	0	0	0	0	0	0	0	0	0	0	0	0	0	0	0	0	0	0	0	0	0	0	0	0	0	0	0	0	0	0	0	0	0	0	0	0	0	0	0	0	0	0
ENSG00000206963	0	0	0	0	0	0	0	0	0	0	0	0	0	0	0	0	0	0	0	0	0	0	0	0	0	0	0	0	0	0	0	0	0	0	0	0	0	0	0	0	0	0	0	0	0	0	0	0
ENSG00000206964	0	0	0	0	0	0	0	0	0	0	0	0	0	0	0	0	0	0	0	0	0.24442098	0	0	0	0	0	0	0	0	0	0	0	0	0	1.1309847	0	0.26869243	0	0.29246702	0	0	0	0	0.24789498	0	0	0	0
ENSG00000206965	0	1.41229845	0	0	0	0.45189401	0	0	0.14891687	0	0	0	0.25494928	0.32631534	0.26196349	0	0	0	0.66297904	0.26745379	0	0	0.30512695	0	0	0	0.25916942	0.68136422	0.21630064	0.48459711	0	0.36852628	0	0.47406566	0	0	0	0.30352422	0	0	0	0.53937572	0.34487202	0	0.55569413	0	0	0.28160839
ENSG00000206967	0.63195568	0	0.30902065	0	0	0	0	0	0	0	0	0	0	0	0	0	0	0	0	0	0	0	0	0	0	0	0	0	0	0	0.48008414	0	0	0	0	0	0	0	0	0	0	0	0	0	0	0	0	0
ENSG00000206968	0	0	0	0	0	0	0	0	0	0	0	0	0	0	0	0	0	0	0	0	0	0	0	0	0	0	0	0	0	0	0	0	0	0	0	0	0	0	0	0	0	0	0	0	0	0	0	0
ENSG00000206969	0	0	0	0	0	0	0	0	0	0	0	0.50403686	0	0	0	0	0	0.71994255	0	0	0	0.25487711	0	0	0	0	0.2567697	0	0	0	0	0	0	0	0	0	0	0	0.90177331	0	0	0	0	0	0	0	0	0.2790009
ENSG00000206970	0	0	0	0	0	0	0	0	0	0	0	0	0	0	0	0	0	0	0	0	0	0	0	0	0	0	0	0	0	0	0	0	0	0	0	0	0	0	0	0	0	0	0	0	0	0	0	0

ENSG00000206972	0	0	0	0	0	0	0	0	0	0	0	0	0	0	0	0	0	0	0	0	0	0	0	0	0	0	0	0	0	0	0	0	0	0	0	0	0	0	0	0	0	0	0	0	0	0	0	0
ENSG00000206973	0	0	0	0	0	0	0	0	0	0	0	0	0	0	0	0	0	0	0	0	0	0	0	0	0	0	0	0	0	0	0	0	0	0	0	0	0	0	0	0	0	0	0	0	0	0	0	0
ENSG00000206974	0	0	0	0	0	0	0	0	0	0	0	0	0	0	0	0	0	0	0	0	0	0	0	0	0	0	0	0	0	0	0	0	0	0	0	0	0	0	0	0	0	0	0	0	0	0	0	0
ENSG00000206975	0	0	0	0	0	0	0	0	0	0	0	0	0.25494928	0	0	0	0	0	0	0	0	0.25725914	0	0	0	0	0.25916942	0	0	0	0	0	0	0	0	0	0	0.30352422	0	0	0	0	0	0	0.27784706	0	0.26822409	0
ENSG00000206976	0	0	0	0.3040927	0	0	0	0	0	0	0	0.19867146	0	0	0	0	0	0	0	0	0	0	0	0	0.22457697	0	0	0	0.16893553	0	0	0.28782709	0	0	0.45817263	0	0	0	0	0	0	0	0	0	0	0	0	0
ENSG00000206977	0	0	0	0	0	0	0	0	0	0	0	0	0	0	0	0	0	0	0	0	0	0	0	0	0	0	0	0.17358565	0	0	0	0	0	0	0	0	0	0	0	0	0	0	0	0	0	0	0	0
ENSG00000206978	0	0	0	0	0	0	0	0	0	0	0	0	0	0	0	0	0	0	0	0	0	0	0	0	0	0	0	0	0	0	0	0	0	0	0	0	0	0	0	0	0	0	0	0	0	0	0	0
ENSG00000206979	0	0	0	0	0	0	0	0	0	0	0	0	0	0	0	0	0	0	0	0	0	0	0	0	0	0	0	0	0	0	0	0	0	0	0	0	0	0	0	0	0	0	0	0	0	0	0	0
ENSG00000206980	0	0	0	0	0	0	0	0	0	0	0	0	0	0	0	0	0	0	0	0	0	0	0	0	0	0	0	0	0	0	0.47085175	0	0	0	0	0	0	0	0	0	0	0	0	0	0	0	0	0
ENSG00000206981	0	0	0	0	0	0	0	0	0	0	0	0	0	0	0	0	0	0	0	0	0	0	0	0	0	0	0	0	0	0	0	0	0	0	0	0	0	0	0	0	0	0	0	0	0	0	0	0
ENSG00000206982	0	0	0	0	0	0	0	0	0	0	0	0	0	0	0	0	0	0	0	0	0	0	0	0	0	0	0	0	0	0	0	0	0	0	0	0	0	0	0	0	0	0	0	0	0	0	0	0
ENSG00000206983	0	0	0	0	0	0	0	0	0	0	0	0	0	0	0	0	0	0.36333549	0	0	0	0	0	0	0	0	0	0	0	0	0	0	0	0	0	0	0	0	0	0	0	0	0	0	0	0	0	0
ENSG00000206985	0	0	0	0	0	0	0	0	0	0	0	0	0	0	0	0	0	0	0	0	0	0	0	0	0	0	0	0	0	0	0	0	0	0	0	0	0	0	0	0	0	0	0	0	0	0	0	0
ENSG00000206987	0	0	0	0	0	0.18177691	0	0	0.1198053	0	0	0	0	0	0	0	0	0	0	0	0	0	0.24547807	0	0	0	0	0.18272173	0.1740163	0.19493192	0	0	0	0	0	0	0	0.24418865	0	0	0	0	0	0	0	0	0	0
ENSG00000206989	0	1.2698818	0	0	0.5287775	1.42213702	1.14189232	1.85701687	1.87460064	3.00497711	2.3884556	1.20079371	1.20351055	1.54040036	2.47324357	2.7433484	0	2.85859542	1.56482552	2.94592488	1.59592522	2.02402411	2.40063115	3.52522647	1.80982617	2.73041923	1.63124282	0	3.06319872	2.28758339	0.72012621	2.8994347	2.33266959	0	0.9230831	1.68644768	3.07020609	2.3880214	3.3418658	3.0453197	0.88542	2.54617068	1.08533252	0.8093042	1.31160158	1.30199009	0.84411698	4.87430986
ENSG00000206990	0	0	0	0	0	0	0	0	0	0	0	0	0	0	0	0	0	0	0	0	0	0	0	0	0	0	0	0	0	0	0	0	0	0	0	0	0	0	0	0	0	0	0	0	0	0	0	0
ENSG00000206991	0	0	0	0	0	0	0	0	0.14891687	0	0	0	0	0	0	0	0	0	0	0	0	0	0	0	0	0	0	0	0	0	0	0	0	0	0.58663225	0	0	0	0	0	0	0	0	0	0	0	0	0
ENSG00000206992	0.90363756	0.20175692	0.58916087	1.1680571	0.16802276	1.12973502	0	0	0.44675062	0.54563002	0	0	0.50989855	0.65263068	0.52392699	0.29057273	0	0.72667099	0	0	0.25355821	0.25725914	0	0.28004136	0.28754248	0.49577706	0.51833884	0.22712141	0.86520255	0.48459711	0	1.10557884	0.49414808	0	0	0	0.557474	0.30352422	0	0.48383584	1.12539365	0.53937572	0.68974403	0	0	0	0.26822409	0.56321677
ENSG00000206995	0	0	0.30902065	0	0.35251833	0	0	0	0	0	0	0	0	0	0	0	0	0	0	0	0	0	0	0	0	0	0	0.23825481	0.22690361	0	0.48008414	0	0	0.49730417	1.23077746	0	0	0.31840285	0	0	0	0	0	0	0.29146702	0.4339967	0.28137233	0
ENSG00000206996	0	0	0	0	0	0	0	0	0	0	0	0	0	0	0	0	0	0	0	0	0	0	0	0	0	0	0	0	0	0	0	0	0	0	0	0	0	0	0	0	0	0	0	0	0	0	0	0
ENSG00000206997	0	0	0	0	0	0	0	0	0	0	0	0	0	0	0	0	0	0	0	0	0	0	0	0	0	0	0	0	0	0	0	0	0	0	0	0	0	0	0	0	0	0	0	0	0	0	0	0
ENSG00000206998	0	0	0	0	0	0	0.25375385	0	0	0	0	0	0	0	0	0	0	0	0	0	0	0	0	0	0	0	0	0	0	0	0	0	0	0	0	0	0	0	0	0	0	0	0	0	0	0	0	0
ENSG00000206999	0	0	0	0	0	0	0	0	0	0	0	0	0	0	0	0	0	0	0	0	0	0	0	0	0	0	0	0	0	0	0	0	0	0	0	0	0	0	0	0	0	0	0	0	0	0	0	0
ENSG00000207000	0	0	0	0	0	0	0	0	0	0	0	0	0	0	0	0	0	0	0	0	0	0	0	0	0	0	0	0	0	0	0	0	0	0	0	0	0	0	0	0	0	0	0	0	0	0	0	0
ENSG00000207001	0.33926042	0.90896802	0	0	0.37849337	0.25448768	0	0	0.16772743	0.30727586	0.42740784	0	0	0	0	0	0	0.4092305	0.37336188	0.60247486	0.28558662	0	0	0.63083	0.32386363	0.27920076	0.87571983	0.51162085	0.48724564	0.27290469	0	0.41507697	0	0	0	0	1.25578354	0.34186412	1.02517387	0.54495195	0	0	0	0.28964571	0.31294353	0.4659754	0	0.31717997
ENSG00000207002	0	0	0	0	0	0	0	0	0	0	0	0	0	0	0	0	0	0	0.27075861	0	0	0	0	0	0	0.20247384	0	0	0	0	0	0	0	0	0	0	0	0	0	0	0	0	0.84506807	0	0	0	0	0
ENSG00000207003	0.61980269	0.41515366	0.6061559	0.4005837	0.34573913	0.69739412	1.99099174	1.21420334	0.91927532	3.36821611	0.39042063	1.04684579	0.78691074	0	0.53904027	0.29895463	0.46620413	0.74763265	2.38736202	2.20135046	0.78261718	0.26468008	1.25571476	1.44059736	0	0.76511748	0	1.4020379	0.22254008	0.99715174	0	0.75831369	1.01680469	1.46322189	1.20710866	0	0.57355498	0.62455944	0.9364569	1.49337793	1.15785692	0.55493464	0.35482025	0.52916044	1.14344753	0	0	0.57946341
ENSG00000207004	0.60242504	0.20175692	0	0.3893524	0	0.225947	0.24189619	0.59008013	0.29783375	0	0.37947425	0.50874749	0.50989855	0	0	0	0.90626597	0	0	0.80236138	0.25355821	0.25725914	0	0.28004136	0	0	0	0	0.21630064	0	0	0	0	0	0	0	0.30352422	0.30340037	0	0	0	0.53937572	0	0.51432416	0	0	0.26822409	0
ENSG00000207005	1.76870523	1.71124315	1.72976195	1.7782007	1.64436906	2.063833	0.63129006	0.57748695	1.06875098	0.53398548	1.9806705	1.65963358	5.32284341	2.98061208	3.76013453	3.98120073	5.32154956	3.31875956	1.08138349	0.69798917	0.99258764	1.17492131	1.39353711	1.82709909	1.50083146	4.20503589	2.02910692	2.07456015	3.24582845	2.21319044	0.59717783	0.48088185	1.93440893	3.71158723	5.74112658	4.54518216	1.45487118	2.37637252	1.78155215	2.52538707	2.2027522	4.22291723	3.37511944	12.4159107	13.0520352	5.12857071	7.69999394	5.87943362
ENSG00000207007	0	0	0	0	0	0	0	0	0.28440799	0.1435505	0	0.36579951	0	0	0	0	0	0	0	0	0.48884196	0	0	0	0	0	0	0	0.41701204	0.23356707	0	0	0	0	0	0	0	0	0	0	0	0	0	0	0	0	0	0
ENSG00000207008	47.1654728	32.4697419	58.4275147	132.09491	20.4632594	107.712427	8.41720086	14.3730086	9.84546354	9.25574832	22.7777107	10.1790859	13.3071085	18.7352759	19.598277	9.85821134	13.4024211	24.3375701	17.5905048	23.0336426	22.2780781	13.6514667	7.16676228	17.296538	101.055985	125.935434	119.717308	77.6478228	53.626731	83.6796766	90.7710302	61.5528772	57.6023993	50.3126269	24.4954734	64.7979815	114.207388	110.10526	107.68493	53.4540264	34.2650342	54.8979239	67.8023993	131.988961	181.278267	53.6250552	106.633249	103.380041
ENSG00000207009	0	0	0	0	0	0.21978481	0	1.43496758	1.01398853	2.12299682	0.36912496	1.48461767	0	0	0.25481903	0	0.44077481	0	0	0	0.49328598	0.25024298	1.78083184	0.81721159	0	2.17015139	0.75630349	1.10463592	0	0.47138082	0	0	0	0	0	0	0.54227017	0.59049256	0	0.94128064	0.54735055	0	0	0	0	0.4024333	0	0.27392816
ENSG00000207010	0	0	0	0	0	0	0	0	0	0	0	0	0	0	0	0	0	0	0	0	0	0	0	0	0	0	0	0	0	0	0	0	0	0	0	0	0	0	0	0	0	0	0	0	0	0	0	0
ENSG00000207011	0	0	0	0	0	0	0	0	0	0	0	0	0	0	0	0	0	0	0	0	0	0	0	0	0	0	0	0	0	0	0	0	0	0	0	0	0	0	0	0	0	0	0	0	0	0	0	0
ENSG00000207012	0	0	0	0	0	0	0	0	0	0	0	0	0	0	0	0	0	0	0	0	0	0	0	0	0	0	0	0	0	0	0	0	0	0	0	0	0	0	0	0	0	0	0	0	0	0	0	0
ENSG00000207013	0	0	0	0	0	0	0	0	0	0	0	0	0	0	0	0	0	0	0	0	0	0	0	0	0	0	0	0	0	0	0	0	0	0	0	0	0	0	0	0	0	0	0	0	0	0	0	0
ENSG00000207014	0.67852084	0.68172602	0	0	0.18924668	0.76346303	0.2724515	0	0	0.61455171	0	0	0.57430679	0	0	0	0.51037083	0	0.37336188	0.30123743	0	0	0.3436693	0.315415	0.32386363	0	0	0.25581042	0	0	0	0.41507697	0	0	0	0	0.31394589	0	0.68344925	0	0	0	0	0	0	0	0	0.31717997
ENSG00000207016	0	0.32709077	0	0	0	0	0	0	0	0	0	0.2061969	0.20666343	0	0	0	0	0	0	0	0	0	0	0	0	0	0.2100843	0	0.17533461	0.19640868	0	0	0	0.3842805	0	0	0	0	0	0	0	0	0	0	0	0	0	0
ENSG00000207019	0	0	0	0	0	0	0	0	0	0	0	0	0	0	0	0	0	0	0	0	0	0	0	0	0	0	0	0	0	0	0	0	0	0	0	0	0	0	0	0	0	0	0	0	0	0	0	0
ENSG00000207020	0	0	0	0	0	0	0	0	0	0.2861883	0	0	0	0	0.27480484	0	0	0	0.34773901	0	0	0	0	0	0	0	0	0	0	0	0	0	0	0	0	0	0	0	0	0	0	0	0	0	0	0	0	0
ENSG00000207021	0	0	0	0	0	0	0	0	0	0	0	0	0	0	0	0	0	0	0	0	0	0	0	0	0	0	0	0	0	0	0	0	0	0	0	0	0	0	0	0	0	0	0	0	0	0	0	0
ENSG00000207022	0	0	0	0	0	0.1949704	0	0	0.12850085	0	0	0	0	0	0	0	0.39100991	0	0	0	0	0	0	0	0	0	0	0	0	0	0	0	0	0	0.50620686	0	0	0	0	0	0	0	0	0	0	0	0	0.24300078
ENSG00000207023	0	0.41119982	0.30019149	0.3967686	0	0.23025076	0.24650374	0.30065987	0	0	0	0.25921896	0	0.33253087	0	0.88832234	0.46176409	0	0	0	0	0	0.62187778	0	0	0	0.26410598	0	0	0.74074129	0	0.37554583	0.50356042	0.48309548	0	0.27304391	0	0	0	0	0	0	0	0.26206041	0	0	0	0.86091707
ENSG00000207024	0	0	0	0	0.17625917	0	0	0	0	0	0	0	0	0	0	0	0	0	0	0	0	0	0	0	0	0	0	0	0	0	0	0	0	0	0	0	0	0	0	0	0	0	0	0	0	0	0	0
ENSG00000207025	0	0	0	0	0	0	0	0	0	0	0	0	0	0	0	0	0	0	0	0	0	0	0	0	0	0	0	0	0	0	0	0	0	0	0	0	0	0	0	0	0	0	0	0	0	0	0	0
ENSG00000207026	0	0	0	0	0	0	0	0	0	0	0	0	0	0	0	0	0	0	0	0	0	0	0	0	0	0	0	0	0	0	0	0	0	0	0	0	0	0	0	0	0	0	0	0	0	0	0	0
ENSG00000207027	0	0	0	0	0	0	0	0	0	0	0	0	0	0	0	0	0	0	0	0	0	0	0	0	0	0	0	0	0	0	0	0	0	0	0	0	0	0	0	0	0	0	0	0	0	0	0	0
ENSG00000207029	0	0	0.58916087	0	0	0	0	0	0.14891687	0	0	0	0	0	0	0	0	0	0	0	0	0	0	0	0	0	0	0	0	0	0	0	0	0	0	0	0	0	0	0	0	0	0	0	0	0	0	0
ENSG00000207031	1.28918959	4.31759811	1.68107235	6.6657124	1.67798727	10.959936	2.76084188	0.84184765	1.48718318	3.89216083	3.24829962	2.17743925	2.54609343	0.93108644	1.86867292	2.90185298	0.64646972	0	1.89170019	3.0525393	0	1.10106912	2.6118867	1.59810267	1.64090906	2.1219258	2.5882386	2.5922123	2.16012236	3.45679268	1.30582886	2.10305664	3.52492294	1.35266735	0	2.29356884	2.78365352	3.46422305	3.46280951	1.38054493	1.6055616	4.61705617	0.98403482	1.10065371	1.58558057	2.36094203	1.1479991	5.22289687
ENSG00000207032	0	0.19805496	0	0	0	0	0	0	0	0	0	0	0	0	0	0	0	0	0	0	0	0	0	0	0	0	0	0	0	0	0	0	0	0	0	0	0	0	0	0	0	0	0	0	0	0	0	0
ENSG00000207033	0	0	0	0	0	0	0	0	0	0	0	0	0	0	0	0	0	0	0	0	0	0	0	0	0	0	0	0	0	0	0	0	0	0	0	0	0	0	0	0	0	0	0	0	0	0	0	0
ENSG00000207034	0.27546786	0.73805096	0	0	0.46098551	0.41327059	0.66366391	0.26982296	0.40856681	0.24949749	0	0.6978972	0.23315874	1.19370056	0.71872035	0.26573745	0.41440367	0	0.30315708	0.2445945	0	0	0.27904772	0.2561062	0	0.68010442	0.71105456	0	0	0.22158927	0.41853489	0.33702831	0.4519132	0	0.53649274	0.24503941	0	0.27758197	0	0.44248235	0	0	1.26158311	0.94072967	0	0.37835609	0.24529895	0.77261788
ENSG00000207036	0	0	0	0	0	0	0	0	0	0	0	0	0	0	0	0	0	0	0	0	0	0	0	0	0	0	0	0	0	0	0	0	0	0	0	0	0	0	0	0	0	0	0	0	0	0	0	0
ENSG00000207037	0	0	0	0	0	0	0	0	0	0	0	0	0	0	0	0	0	0	0	0	0	0	0	0	0	0	0	0	0	0	0	0	0	0	0	0	0	0	0	0	0	0	0	0	0	0	0	0
ENSG00000207039	0.31597784	0	0	0	0.17625917	0	0	0	0.15621672	0	0.39807593	0	0	0	0	0	0	0	0	0	0	0	0	0.29376887	0.30163769	0.26003993	0	0	0	0	0.96016828	0.38659129	0	0	0	0.29240058	0	0	0	0	0	0.56581571	0	0	0	0.4339967	0	0.29541272
ENSG00000207041	0	0.20175692	0	0	0.16802276	0	0	0.29504006	0	0	0.37947425	0	0.25494928	0	0.78589048	0	0.90626597	0	0.66297904	0	0	0.25725914	0	0.28004136	0	0.74366559	0.25916942	0.22712141	0	0	0	0	0	0	0	0.26794029	0	0.60704843	0	0	0.56269682	0	0.68974403	0	0.55569413	0.41371648	0.26822409	0.28160839
ENSG00000207042	0	0	0	0	0	0	0	0	0	0	0	0	0.25494928	0.32631534	0	0.29057273	0	0	0.33148952	0	0	0.25725914	0	0	0	0	0.25916942	0	0.21630064	0.24229855	0	0	0	0.47406566	0	0	0	0	0.60680073	0	0	0.53937572	0	0.25716208	0	0	0.53644818	0
ENSG00000207044	0.30121252	0	0	0	0.33604551	0	0.48379239	0.29504006	0.59566749	0	0.75894851	0.25437374	0.25494928	1.30526136	0.26196349	0.29057273	0.45313298	0.36333549	0.66297904	1.60472277	0.76067464	0.25725914	0.91538085	0.84012407	0.86262743	1.48733117	1.81418593	0.22712141	0.21630064	0.96919421	0	0	0	0	0	0.53588057	0.278737	0	0.30340037	0.48383584	0	1.07875144	2.0692321	0.25716208	0.27784706	0.41371648	1.07289635	0.28160839
ENSG00000207045	0	0	0	0	0	0	0	0	0	0	0	0	0	0	0	0	0	0	0	0	0	0	0	0	0	0	0	0	0	0	0	0	0	0	0	0	0	0	0	0	0	0	0	0	0	0	0	0
ENSG00000207046	0	0	0	0	0	0	0	0	0	0	0	0	0	0	0	0	0	0	0	0	0	0	0	0	0	0	0	0	0	0	0	0	0	0	0	0	0	0	0	0	0	0	0	0	0	0	0	0
ENSG00000207047	1.61148699	1.07939953	1.97000666	0	1.7978435	0.90661235	3.23536158	3.55154477	1.99176318	3.64890078	3.04528089	2.38157418	3.0689519	0.43644677	0.70075234	1.94320512	2.42426147	3.88768977	1.77346893	0.35771945	2.71307287	2.75267279	0.81621459	2.62188719	0.38458806	2.32085635	1.73319549	0.91132464	0.5786042	0	3.67264366	1.9716156	1.3218461	2.53625127	1.56924126	1.79185066	3.35529666	5.68349094	3.65218191	0	1.505214	2.8856601	1.38379897	1.37581714	2.60134313	0.55334579	4.30499661	1.88325608
ENSG00000207049	0	0	0	0	0	0	0	0	0	0	0	0	0	0	0	0	0	0	0	0	0	0	0	0	0	0	0	0	0	0	0	0	0	0	0	0	0	0	0	0	0	0	0	0	0	0	0	0
ENSG00000207051	0	0	0	0	0	0	0	0	0	0	0	0	0	0	0	0	0	0	0	0	0	0	0	0	0	0	0	0	0	0	0	0	0	0	0	0	0	0	0	0	0	0	0	0	0	0	0	0
ENSG00000207052	0	0	0	0	0	0	0	0	0	0	0	0	0	0	0	0	0	0	0	0	0	0	0	0	0	0	0	0	0	0	0	0	0	0	0	0	0	0	0	0	0	0	0	0	0	0	0	0
ENSG00000207053	0	0	0	0	0	0	0	0	0	0	0	0	0	0	0	0	0	0	0	0	0	0	0	0	0	0	0	0	0	0	0	0	0	0	0	0	0	0	0	0	0	0	0	0	0	0	0	0
ENSG00000207056	0	0	0.3820619	0	0	0	0	0	0	0	0	0	0	0	0	0	0	0	0	0	0	0	0	0	0	0	0	0	0	0	0	0	0	0	0	0	0	0	0	0	0	0	0	0	0	0	0	0
ENSG00000207058	0	0	0	0	0	0	0	0	0	0	0	0	0	0	0	0	0	0	0	0	0	0	0.30800551	0	0	0	0.26161441	0	0	0	0	0	0	0	0	0	0	0	0	0	0	0	0	0	0	0	0	0
ENSG00000207060	0	0	0	0	0	0	0	0	0	0	0	0	0	0	0	0	0	0	0	0	0	0	0	0	0	0	0	0	0	0	0	0	0	0	0	0	0	0	0	0	0	0	0	0	0	0	0	0
ENSG00000207061	0	0	0	0.4084383	0	0	0	0	0	0	0	0	0	0	0	0	0	0	0	0	0.26598754	0.26986988	0.32008415	0	0.30163769	1.30019963	0.2718738	0.23825481	0	0	0.96016828	0.38659129	0	0.49730417	0.61538873	0.56214923	0.29240058	0	0.31827293	0	0	0	0.72355502	0.26976807	0.29146702	0.86799339	0	0
ENSG00000207062	0.23873881	0	0	0.3085978	0	0	0	0	0	0	0	0	0	0	0	0.23030579	0	0	0	0	0	0	0	0	0	0	0	0	0	0	0	0	0	0.37574093	0	0	0	0	0	0	0	0	0	0	0	0	0	0.22320072
ENSG00000207063	1.01778126	1.81793605	2.98611536	6.5780056	0.37849337	4.5807782	0	0	0	1.22910342	0.85481569	0.28650516	0.28715339	0.36753412	0.29505362	0	0	1.63692201	3.36025691	2.10866202	1.14234647	1.73853018	1.71834651	0.63083	1.61931815	1.95440535	1.16762644	0.76743127	0.24362282	0.54580937	1.54637628	3.32061574	0.55656678	0.53394764	1.98219949	1.2071415	0.31394589	2.0511847	1.36689849	2.17980779	0.63377432	1.21501478	1.55373919	1.15858285	1.56471767	0	1.2084201	0.31717997
ENSG00000207065	0	0	0	0	0	0	0	0	0	0	0	0	0	0	0	0	0	0	0	0	0	0	0	0	0	0	0	0	0	0	0	0	0	0	0	0	0	0	0	0	0	0	0	0	0	0	0	0
ENSG00000207067	2.19748226	1.9625446	1.43273212	3.4717252	1.22580239	3.11361818	5.68639308	5.9790316	5.79422017	3.98061903	3.99885369	3.29915038	2.47996113	1.32256597	3.39758713	1.88432012	3.67312343	1.47260976	3.76190378	2.16799667	4.52178812	4.37925217	4.69941735	4.5400644	3.26317143	2.00939944	5.46219185	3.31390777	2.27934989	4.71380821	2.22584464	2.09110745	2.80391598	2.30568298	1.42658297	1.30316411	2.48540493	3.19850139	4.18094899	3.52980239	4.56125455	3.49776982	1.39777674	2.91839998	1.35134708	2.68288867	1.5219685	3.8806489
ENSG00000207068	0	0	0	0	0	0	0	0	0	0	0	0	0	0	0	0	0	0	0	0	0	0	0	0	0	0	0	0	0	0	0	0	0	0	0	0	0	0	0	0	0	0	0	0	0	0	0	0
ENSG00000207069	0	0	0	0	0	0	0	0	0	0	0	0	0	0	0	0	0	0	0	0	0	0.26986988	0	0	0	0	0	0	0	0	0	0	0	0	0	0	0	0	0	0	0	0	0	0	0	0	0	0
ENSG00000207071	0	0	0	0	0	0	0	0	0	0	0	0	0	0	0	0	0	0	0	0	0	0	0	0	0	0	0	0	0	0	0	0	0	0	0	0	0	0	0	0	0	0	0	0	0	0	0	0
ENSG00000207072	0	0.20175692	0	0	0	0	0	0	0.14891687	0.27281501	0.37947425	0	0	0	0.26196349	0	0	0.36333549	0.33148952	0	0	0	0.30512695	0	0	0	0	0.22712141	0	0	0	0	0	0	0	0.26794029	0	0.30352422	0	0	0	0	0.34487202	0.25716208	0	0	0	0
ENSG00000207073	0	0	0	0	0	0	0	0	0	0.28902184	0	0	0	0	0	0	0	0	0	0	0	0	0.3232533	0	0	0	0	0	0	0	0	0	0	0	0	0	0	0	0	0	0	0	0	0	0	0	0	0.2983376
ENSG00000207075	0	0	0	0	0	0	0	0	0	0	0	0	0	0	0	0	0	0	0	0	0	0	0	0	0	0	0	0	0	0	0	0	0	0	0	0	0	0	0	0	0	0	0	0	0	0	0	0
ENSG00000207076	0	0	0	0	0	0	0	0	0	0	0	0	0	0	0	0	0	0	0	0.26745379	0	0	0	0	0	0	0.25916942	0	0	0	0	0	0	0	0	0	0	0	0	0	0	0	0	0	0	0	0	0
ENSG00000207077	0	0	0	0	0	0	0	0	0	0	0	0	0	0	0	0	0	0	0	0	0	0	0	0	0	0	0	0	0	0	0	0	0	0	0	0	0	0	0	0	0	0	0	0	0	0	0	0
ENSG00000207080	0	0	0	0	0	0	0	0	0	0	0	0	0	0	0	0	0	0	0	0	0	0	0	0	0	0	0	0	0	0	0	0	0	0	0	0	0	0	0	0	0	0	0	0	0	0	0	0
ENSG00000207081	0	0	0	0	0	0	0	0	0	0	0	0	0	0	0	0	0	0	0	0	0	0	0	0	0	0	0	0	0	0	0	0	0	0	0	0	0	0	0	0	0	0	0	0	0	0	0	0
ENSG00000207082	2.71091269	4.64040919	5.59702828	1.9467618	1.34418206	4.51894007	0	0.88512019	0.89350124	0.27281501	0.75894851	1.01749498	0.25494928	0	0	0	0	0	0	0.26745379	0.25355821	0.77177742	0	0.56008271	0	0	0.25916942	0.22712141	0	0.24229855	0.4576503	0.73705256	0.49414808	0.47406566	1.1732645	1.07176114	0.278737	0.60704843	0	0	0.56269682	1.07875144	0	0.27784706	0	0	0	0
ENSG00000207083	0.30121252	0	0	0	0	0	0	0	0	0	0	0	0	0	0	0	0.45313298	0	0.66297904	0.26745379	0	0	0	0.28004136	0	0.74366559	0	0	0	0	0	0	0	0	0	0.26794029	0	0	0	0	0	0	0.34487202	0	0	0	0	0
ENSG00000207084	0	0	0	0	0	0	0	0	0	0	0	0	0	0	0	0	0	0	0	0	0	0	0	0	0	0	0	0	0	0	0	0	0	0	0	0	0	0	0	0	0	0	0	0	0	0	0	0
ENSG00000207086	0	0	0	0	0	0	0	0	0	0	0	0	0	0	0	0	0	0	0	0	0	0	0	0	0	0	0	0	0	0	0	0	0	0	0	0	0	0	0	0	0	0	0	0	0	0	0	0
ENSG00000207087	0	0	0.29458044	0	0	0.225947	0	0	0.14891687	0	0	0	0	0	0	0	0	0	0	0	0	0	0	0	0	0	0	0.22712141	0	0	0	0.36852628	0	0	0	0.26794029	0	0	0	0	0	0	0	0	0	0	0	0
ENSG00000207088	14.6077238	14.754382	12.0184579	28.772859	10.8646658	20.34986	12.1035109	17.7151394	13.0682592	9.87040787	45.2775576	16.8399079	7.65398076	12.5596192	10.4860782	3.13149602	5.58103359	11.4672864	18.3726277	8.23526791	11.906291	11.2879388	3.75811035	6.8982849	21.6918734	18.8912459	21.5464878	22.0291423	14.8189278	15.1079249	30.6494003	24.6806557	23.2036294	16.0568426	19.4179495	26.1945362	31.1122632	42.2903128	30.8289696	44.3214515	32.053478	30.3098112	18.3179144	22.7653195	34.8628102	25.1593193	23.538111	17.1254366
ENSG00000207089	0	0	0	0	0	0	0	0	0	0	0	0	0	0	0	0	0	0	0	0	0	0	0	0	0	0	0	0	0	0	0	0	0	0	0	0	0	0	0	0	0	0	0	0	0	0	0	0
ENSG00000207090	0	0	0	0	0	0	0	0.28699352	0	0	0	0	0	0	0	0	0	0	0	0	0	0	0	0	0	0	0	0	0	0	0	0	0	0	0	0	0	0	0	0	0	0	0	0	0	0	0	0
ENSG00000207091	0	0	0	0	0	0	0	0	0	0	0	0	0	0	0	0	0	0	0	0	0	0	0	0	0	0	0	0	0	0	0	0	0	0	0	0	0	0	0	0	0	0	0	0	0	0	0	0
ENSG00000207092	0	0	0.30902065	0.4084383	0	0	0.25375385	0	0.15621672	0	0	0.53368609	0	0	0.27480484	0	0	0	0	0	0	0	0	0	0	0	0.2718738	0	0	0.25417593	0	0	0	0	0	0.28107461	0	0	0	0	0	0	0	0	0	0	0.28137233	0
ENSG00000207093	0	0.45448401	1.65895298	1.3156011	0	0.50897536	0.2724515	0	0	0	0	0.28650516	0	0	0.29505362	0	0	0	0	0.90371229	1.42793309	0.28975503	0	0	0	0.27920076	0	0.25581042	0	0	0	0	0	0	0	0	0.31394589	0	0.68344925	0	0	0	0.3884348	0.28964571	0.31294353	0	1.2084201	0.31717997
ENSG00000207094	0	0	0	0	0	0	0	0	0	0	0	0	0	0	0	0	0	0	0	0	0	0	0	0	0	0	0	0	0	0	0	0	0	0	0	0	0	0	0	0	0	0	0	0	0	0	0	0
ENSG00000207095	0	0	0	0	0	0	0	0	0	0	0	0	0	0	0	0	0	0	0	0	0	0	0	0	0	0	0	0	0	0	0	0	0	0	0	0	0	0	0	0	0	0	0	0	0	0	0	0
ENSG00000207097	0	0	0	0	0	0	0	0	0	0	0	0	0	0	0	0	0	0	0	0	0	0	0	0	0	0	0	0	0	0	0	0	0	0	0	0	0	0	0	0	0	0	0	0	0	0	0	0
ENSG00000207098	0	0	0	0	0	0	0	0	0	0	0	0	0	0	0	0	0	0	0	0	0	0	0	0	0	0	0	0	0	0	0	0	0	0	0	0	0	0	0	0	0	0	0	0	0	0	0	0
ENSG00000207099	0	0	0	0	0	0	0	0	0	0	0	0	0	0	0	0	0	0	0	0	0	0	0	0	0	0	0	0	0	0	0	0	0	0	0	0	0	0	0	0	0	0	0	0	0	0	0	0
ENSG00000207100	0	0	0	0	0	0	0	0	0	0	0	0	0	0	0	0	0	0	0	0	0	0	0	0	0	0	0	0	0	0	0	0	0	0	0	0	0	0	0	0	0	0	0	0	0	0	0	0
ENSG00000207101	0	0	0	0	0	0	0	0	0	0	0	0	0	0	0	0	0	0	0	0	0	0	0	0	0	0	0	0	0	0	0	0	0	0	0	0	0	0	0.31827293	0	0	0	0	0	0	0	0	0
ENSG00000207104	0	0	0	0	0	0	0	0	0	0	0	0	0	0	0	0	0	0	0	0	0	0	0	0	0	0	0	0	0	0	0	0	0	0	0	0	0	0	0	0	0	0	0	0	0	0	0	0
ENSG00000207105	0	0.3889728	0.28396492	0	0	0	0.46635843	0	0.4306515	0.26298384	0.36579951	0.49041425	0	0	0.25252337	0.56020328	0	0	0	0	0	0.24798854	0.29413138	0.26994978	0	0	0	0	0.41701204	0.46713415	0	0	0	0	0.56549235	0	0	0	0	0	0	0	0.3324442	0.24789498	0	0.39880777	0.25855836	0
ENSG00000207106	0	0	0	0	0	0	0	0	0	0	0	0	0	0	0	0	0	0	0	0	0	0	0	0	0	0	0	0	0	0	0	0	0	0	0	0	0	0	0	0	0	0	0	0	0	0	0	0
ENSG00000207108	0	0	0	0	0	0	0	0	0	0	0	0	0	0	0	0	0	0	0	0	0	0	0	0	0	0	0	0	0	0	0	0	0	0	0	0	0	0	0	0	0	0	0	0	0	0	0	0
ENSG00000207109	0	0	0	0	0	0.70076317	0.75022877	0.4575259	0.69278719	0.84612192	1.76538023	1.97231816	0.39535612	0	0.81246649	0	0	1.1268666	0.51404896	0	0	0.39893809	0	0	0.4458992	0	0.4019004	0	0.67084545	0	0	0	0	0	0	0	0	0	0.47049042	0	0	0	0	1.29259286	0	0	0	0.87339412
ENSG00000207110	0	0.26326818	0	0	0	0	0	0.3849913	0.29147754	0.71198064	0.24758381	0	0	0	0	0	0	0	0	0.17449729	0.16543127	0.1678459	0	0	0.37520786	0.6469286	1.01455346	0.4445486	0.28224595	0.47425509	1.19435566	0.48088185	0	0	0.76548354	0.3496294	1.45487118	0.59409313	0.98975119	0.31567338	0	1.05572931	0.22500796	0	0	0	0	0
ENSG00000207112	0	0	0	0	0	0	0	0	0	0	0	0	0	0	0	0	0	0	0	0	0	0	0	0	0	0	0	0	0	0	0	0	0	0	0	0	0	0	0	0	0	0	0	0	0	0	0	0
ENSG00000207113	0	0	0	0	0	0	0	0	0	0	0	0	0	0	0.26196349	0	0	0	0	0	0	0	0	0	0	0	0	0	0	0	0	0	0	0	0	0	0	0	0	0	0	0	0	0.25716208	0	0	0	0
ENSG00000207114	0	0	0	0	0	0	0	0	0	0	0	0	0	0	0	0	0	0	0	0	0	0	0	0	0	0	0	0	0	0	0	0	0	0	0	0	0	0	0	0	0	0	0	0	0	0	0	0
ENSG00000207115	0	0	0	0	0	0	0	0	0	0	0	0	0	0	0	0	0	0	0	0	0	0	0	0	0	0	0	0	0	0	0	0	0	0	0	0	0	0	0	0	0	0	0	0	0	0	0	0
ENSG00000207116	0	0.1998888	0	0	0	0.2238549	0.23965641	0	0.14753801	0	0.3759606	0	0.25258863	0	0	0	0.89787462	0	0.32842017	0	0	0.25487711	0.3023017	0	0	0	0	0	0.21429785	0	0	0.365114	0	0	0	0.26545936	0	0	0.3005911	0	0	0	0	0	0.2752744	0	0	0
ENSG00000207117	0.31291009	0.41918428	0.61204091	0	0	0.93888658	0	0	0	0	0	0	0.26485022	0	0.27213683	0	0.47073038	0	0.3443629	0	0	0.26724979	0	0	0	0.25751527	0	0.47188331	0	0	0	0	0	0	0	0.27834573	0	0	0.3151829	0	0	0	0	0	0	0	0	0
ENSG00000207118	0	0	0	0	0	0	0	0	0	0	0	0	0	0	0	0	0	0	0	0	0	0	0	0	0	0	0	0	0	0	0	0	0	0	0	0	0	0	0	0	0	0	0	0	0	0	0	0
ENSG00000207119	0.30990134	0	0.15153897	0	0.08643478	0.34869706	0.37331095	0.45532625	0.22981883	0	0	0.13085572	0	0	0.13476007	0.14947732	0	0.18690816	0.17052586	0.1375844	0	0.26468008	0.15696434	0.14405974	0.14791849	0.25503916	0	0.11683649	0	0	0.47085175	0.18957842	0.25420117	0.24387031	0	0	0.28677749	0.15613986	0.15607615	0	0	0.83240195	0	0	0	0	0.13798066	0.72432926
ENSG00000207121	0	0	0	0	0	0	0	0	0	0	0	0	0	0	0	0	0	0	0	0	0	0	0	0	0	0	0	0	0	0	0	0	0	0	0	0	0	0	0	0	0	0	0	0	0	0	0	0
ENSG00000207122	0	0	0	0	0	0	0	0	0	0	0	0	0	0	0	0	0	0	0	0	0	0	0	0	0	0	0	0	0	0	0	0	0	0	0	0	0	0	0	0	0	0	0	0	0	0	0	0
ENSG00000207123	0.31597784	0	0	0	0	0	0	0	0	0	0	0	0	0	0	0	0	0	0	0	0	0	0	0	0	0	0	0	0	0	0	0	0	0	0	0	0	0	0	0	0	0	0	0	0	0	0	0
ENSG00000207124	0	0	0	0	0	0	0	0	0	0	0	0	0	0	0	0	0	0	0	0	0	0	0	0	0	0	0	0	0	0	0	0	0	0	0	0	0	0	0	0	0	0	0	0	0.28046826	0	0	0
ENSG00000207127	0	0	0.29458044	0	0	0	0	0	0	0	0	0	0	0	0	0	0	0	0	0	0	0	0	0	0	0	0	0	0	0	0	0	0	0	0	0	0	0	0	0	0	0	0	0	0	0	0	0
ENSG00000207128	0.30121252	2.42108305	0.88374131	0	0.33604551	0.90378801	0	0	0.14891687	0.54563002	0	0	0	0	0	0	0.45313298	0.36333549	0	0.80236138	0	0.51451828	0	0.28004136	0	0	0.25916942	0.68136422	0.21630064	0	0	0	0	0	0	0	0	0	0	0	0	0	0.34487202	0.51432416	0.27784706	0	0	0
ENSG00000207129	0	0	0	0	0	0	0	0	0	0	0	0	0	0	0	0	0	0	0	0	0	0	0	0	0	0	0	0	0	0	0	0	0	0	0	0	0	0	0	0	0	0	0	0	0	0	0	0
ENSG00000207130	0	0	0	0	0	0	0	0	0	0	0	0	0	0	0	0	0	0	0	0	0	0	0	0	0	0	0.20541576	0	0	0	0	0	0	0	0	0	0	0	0	0	0	0	0	0	0	0	0	0
ENSG00000207131	0	0	0	0	0	0	0	0	0	0	0	0	0	0	0	0	0	0	0	0	0	0	0	0	0	0	0	0	0	0	0	0	0	0	0	0	0	0	0	0	0	0	0	0	0	0	0	0
ENSG00000207132	0	0	0	0	0	0	0	0	0	0	0	0	0	0	0	0	0	0	0	0	0	0	0	0	0	0	0	0	0	0	0	0	0	0	0	0	0	0	0	0	0	0	0	0	0	0	0	0
ENSG00000207133	4.07112502	4.77208213	0.66358119	7.8936067	2.64945358	3.56282749	1.08980601	0	0	0.61455171	2.56444707	0.28650516	1.14861358	0.73506824	0.29505362	0	0	0	1.12008564	1.80742459	2.28469295	0.86926509	1.37467721	2.207905	2.59090904	3.35040916	2.33525287	0.51162085	1.46173693	0.81871406	0.51545876	0.41507697	1.66970034	0	0	0.30178537	0.62789177	0.68372823	0	0	0.63377432	0.60750739	0.3884348	2.60681141	1.56471767	0	1.2084201	1.26871989
ENSG00000207134	0	0.40351384	0.29458044	0	0	0	0.48379239	0.59008013	0.59566749	0.54563002	0	0	0	0	0	0	0	0	0	0.26745379	0	0.25725914	0.30512695	0.56008271	0.28754248	0.24788853	0	0.68136422	0.21630064	0.24229855	0	0.36852628	0	0.94813132	0	0.26794029	0	0.30352422	0	0	0	0.53937572	0	0.25716208	0	0	0.26822409	0.56321677
ENSG00000207135	0	0	0	0	0	0	0	0	0	0	0	0	0	0	0	0	0	0	0	0	0	0	0	0	0	0	0	0	0	0	0	0	0	0	0	0	0	0	0	0	0	0	0	0	0	0	0	0
ENSG00000207136	0	0.20757683	0	0	0	0	0.24887397	0.30355083	0	0	0	0	0	0	0	0	0.46620413	0	0.34105172	0	0	0	0	0	0	0	0	0	0	0	0	0	0	0	0	0	0	0	0.3121523	0	0	0	0	0	0	0	0	0
ENSG00000207137	3.15290933	3.285129	5.82436753	3.169836	2.54043103	2.3650757	0.28133579	1.02943327	0.1731968	2.22107004	1.32403517	0.29584772	0.59303418	0.75903786	0	0	1.05402672	0.84514995	2.31322034	1.55530196	0.88469768	1.79522138	5.32313865	3.58270299	2.00654641	2.59474623	3.31567833	1.05660828	1.76096931	1.12721501	0.5322672	0	1.72414709	0	0	1.86975721	2.59346602	2.47108302	3.88154599	0.56272212	0	5.64585673	3.60991035	0.59818136	2.58518571	0	1.55978138	1.96513678
ENSG00000207138	0	0	0	0	0	0	0	0	0	0	0	0	0	0	0	0	0	0	0	0	0	0	0	0	0	0	0	0	0	0	0	0	0	0	0	0	0	0	0	0	0	0	0	0	0	0	0	0
ENSG00000207139	0	0.21374248	0.62416053	0	0	0	0	0.3125672	0	0	0	0.26948506	0	0.34570041	0.27752568	0	0.96010355	0	0	0	0	0.27254186	0	0	0	0	0	0	0	0.51338505	0.48483745	0	0	0	0	0	0	0	0	0	0.59612436	0	0	0.27243904	0	0	0	0
ENSG00000207141	0	0	0	0	0	0	0	0	0	0	0	0	0	0	0	0	0	0	0	0	0	0	0	0	0	0	0	0	0	0	0	0	0	0	0	0	0	0	0	0	0	0	0	0	0	0	0.28415819	0
ENSG00000207142	0	0	0	0	0	0	0	0	0.15621672	0	0	0	0	0	0	0	0	0	0.34773901	0	0	0.26986988	0	0	0	0	0	0.23825481	0	0	0	0	0	0	0	0	0	0	0.31827293	0	0	0	0	0.26976807	0	0	0	0
ENSG00000207144	0	0	0	0	0	0	0	0	0	0	0	0	0	0	0	0	0	0	0	0	0	0	0	0	0	0	0	0	0	0	0	0	0	0	0	0	0	0	0	0	0	0	0	0	0	0	0	0
ENSG00000207145	0	0	0	0	0	0	0	0	0	0	0	0	0	0	0	0	0	0	0	0	0	0	0	0	0	0	0	0	0	0	0	0	0	0	0	0	0	0	0	0	0	0	0	0	0	0	0	0
ENSG00000207146	0	0	0	0	0	0	0	0	0	0	0	0	0	0	0	0	0	0	0	0	0	0	0	0	0	0	0	0	0	0	0	0	0	0	0	0	0	0	0	0	0	0	0	0	0	0	0	0
ENSG00000207147	0	0	0	0	0	0	0	0	0.11716254	0	0	0	0.20058509	0.25673339	0	0	0.35650904	0	0	0	0	0	0	0	0	0	0	0	0	0.19063195	0	0	0	0	0	0	0	0	0	0	0	0	0	0	0	0	0	0
ENSG00000207148	0	0	0	0	0	0	0.5075077	0	0.46865016	0	0	0.26684305	0.26744679	0	0	0	0	0	0	0.56112855	1.59592522	1.07947953	0.96025246	3.81899535	2.71473925	2.34035934	1.63124282	0.71476442	0.90761444	0.50835187	0	0	0	0	0	0	0.58480116	0.63680571	0.63654587	0.50755328	1.18056	1.13163141	0	0	0	0.86799339	0	0
ENSG00000207149	0	0	0	0	0	0	0	0	0	0	0	0	0	0	0	0	0	0	0	0	0	0	0	0	0	0	0	0	0	0	0	0	0	0	0	0	0	0	0	0	0	0	0	0	0	0	0	0
ENSG00000207150	0	0	0	0	0	0	0	0	0	0	0	0	0	0	0	0	0	0	0	0	0	0	0	0	0	0	0	0	0	0	0	0	0	0	0	0	0	0	0	0	0	0	0	0	0	0	0	0
ENSG00000207151	0	0	0	1.2253148	0.5287775	0	0.25375385	0	0.15621672	0	0	0	0	0	0	0	0	0	0.34773901	0	0	0.26986988	0	0	0	0.26003993	0	0.23825481	0	0.25417593	2.88050483	0	0	0.49730417	0	0	0.58480116	0.31840285	0.31827293	0	0.59028	0.56581571	0	0	0	0.4339967	0	0
ENSG00000207153	0	0	0	0	0	0	0	0	0	0	0	0	0	0	0	0	0	0	0	0	0	0	0	0	0	0	0	0	0	0	0	0	0	0	0	0	0	0	0	0	0	0	0	0	0	0	0	0
ENSG00000207154	0	0	0	0	0	0	0	0	0	0	0	0	0	0	0	0	0	0	0	0	0	0	0	0	0	0	0	0	0	0	0	0	0	0	0	0	0	0	0	0	0	0	0	0	0	0	0	0
ENSG00000207155	0	0	0	0.3857472	0	0	0	0.58461642	0.14753801	0	0	0	0	0.3232939	0	0	0.44893731	0	0	0	0.25121045	0	0	0	0	0	0	0.22501843	0	0	0	0	0	0	0	0	0	0	0	0	1.11497333	0	0.34167876	0	0	0	0	0
ENSG00000207156	0	0	0	0	0	0	0	0	0	0.27281501	0	0	0	0	0	0	0	0	0	0	0	0	0	0	0	0	0	0	0	0	0	0	0	0	0	0	0	0	0	0	0	0	0	0	0	0	0	0
ENSG00000207157	0	0	0	0	0	0	0	0	0	0	0	0	0	0	0	0	0	0	0	0	0	0	0	0	0	0	0	0	0	0	0	0	0	0	0	0	0	0	0	0	0	0	0	0	0	0	0	0
ENSG00000207158	0	0	0	0	0	0	0	0	0	0	0	0	0	0	0	0	0	0	0	0	0	0	0	0	0	0	0	0	0	0	0	0	0	0	0	0	0	0	0	0	0	0	0	0	0	0	0	0
ENSG00000207160	0	0	0	0	0	0	0	0	0	0	0	0	0	0	0	0	0	0	0	0	0	0	0	0	0	0	0	0	0	0	0	0	0	0	0	0	0	0	0	0	0	0	0	0	0	0	0	0
ENSG00000207161	0	0	0	0	0	0	0	0	0	0	0	0	0	0	0	0	0	0	0	0	0	0	0	0	0	0	0	0	0	0	0	0	0	0	0	0	0	0	0	0	0	0	0	0	0	0	0	0
ENSG00000207162	0	0.20175692	0	0	0.16802276	0	0	0	0.14891687	0.27281501	0.37947425	0.25437374	0.25494928	0	0	0	0	0	0	0	0	0	0	0	0	0	0.25916942	0.22712141	0.21630064	0	0	0	0	0	0	0	0	0	0	0	0	0.53937572	0.34487202	0.25716208	0	0	0	0
ENSG00000207163	0.90363756	0.80702768	0.58916087	0.7787047	1.00813654	0.225947	0	0	0	0	0	0	0	0	0	0	0	0	0	0	0	0	0	0	0	0	0	0	0	0	0	0	0	0	0	0	0	0	0	0	0	0	0	0	0	0	0	0
ENSG00000207164	0	0	0	0	0	0	0	0	0	0	0	0	0	0	0	0	0	0	0	0	0	0	0	0	0	0	0	0	0	0	0	0	0	0	0	0	0	0	0	0	0	0	0	0	0	0	0	0
ENSG00000207165	0	0	0	0	0	0	0	0	0	0	0	0	0	0	0	0	0	0	0	0	0	0	0	0	0	0	0	0	0	0	0	0	0	0	0	0	0	0	0	0	0	0	0	0	0	0	0	0
ENSG00000207166	0	0	0	0	0	0	0	0	0	0	0	0	0	0	0	0	0	0	0	0	0	0	0	0	0	0	0	0	0	0	0	0	0	0	0	0	0	0	0	0	0	0	0	0	0	0	0	0
ENSG00000207167	0	0	0	0	0.33604551	0	0	0	0	0.27281501	0	0	0	0	0	0	0	0	0	0	0	0	0.30512695	0	0.28754248	0.24788853	0.25916942	0	0	0	0	0	0	0	0	0	0.278737	0	0	0.48383584	0	0	0	0	0	0	0	0
ENSG00000207168	0.48465774	0.16231572	0.47398657	0.6264767	0.1351762	0.72710765	0.19460822	0.23736306	0.23961061	1.53637928	0	1.02323273	0.41021913	0	0.21075258	0.46753807	0.3645506	0	0.26668706	0	0.20399044	0.41393576	0	0	0.46266233	0	0.41700944	0	0	0	0	0	0	0	0.47195226	0	0.44849412	0.73256596	1.4645341	0.38925139	0	0	0.27745343	0.20688979	0.2235311	0	0	0.22655712
ENSG00000207169	0	0	0	0	0	0	0	0	0	0	0	0	0	0	0	0	0	0	0	0	0	0	0	0	0	0	0	0	0	0	0	0	0	0	0	0	0	0	0	0	0	0	0	0	0	0	0	0
ENSG00000207170	0	0	0	0	0	0	0	0	0	0	0	0	0	0	0	0	0	0	0	0	0	0	0	0	0	0	0	0	0	0	0	0	0	0	0	0	0	0	0	0	0	0	0	0	0	0	0	0
ENSG00000207171	0	0	0	0	0	0	0	0	0	0	0	0	0	0	0	0	0	0	0	0	0	0	0	0	0	0	0	0	0	0	0	0	0	0	0	0	0	0	0	0	0	0	0	0	0	0	0	0
ENSG00000207172	0	0	0	0	0	0	0	0	0	0	0	0	0	0	0	0	0	0	0	0	0	0	0	0	0	0	0	0	0	0	0	0	0	0	0	0	0	0	0	0	0	0	0	0	0	0	0	0
ENSG00000207173	0	0	0	0	0	0	0	0	0	0	0	0	0	0	0	0	0	0	0	0	0	0	0	0	0	0	0	0	0	0	0	0	0	0	0	0	0	0	0	0	0	0	0	0	0	0	0	0
ENSG00000207174	4.55420236	4.45838936	6.50958724	7.2453395	3.12668435	6.30686853	0.56267158	1.02943327	0.69278719	1.5864786	3.97210551	0.59169545	0.88955128	0.75903786	0.60934986	0	0	0.84514995	1.54214689	1.24424156	2.06429458	1.49601782	0.70975182	1.95420163	1.0032732	0	3.31567833	2.64152069	2.76723749	1.12721501	10.1130767	4.71473295	4.02300988	6.61630767	2.04683643	3.11626201	1.62091626	7.06023719	4.23441381	2.8136106	4.58108609	1.88195224	6.81871956	1.49545341	7.10926072	0.48117025	2.18369393	0.3275228
ENSG00000207175	0.1965228	0.13163409	0.19219577	0.762086	0.32887381	0.88449986	0	0.3849913	0.4857959	0.17799516	0.74275144	0.33192672	1.66338856	24.4835992	8.71667551	0.75832395	2.36513314	18.7272861	4.10925727	0.34899459	0.33086255	1.51061312	0.79630692	0.73083964	1.87603932	2.2642501	2.02910692	8.74278921	4.79818119	2.05510541	9.8534342	5.04925945	1.93440893	9.27896807	5.3583848	7.34221733	0.54557669	0.59409313	0.39590048	0	0.73425073	0	6.97524683	2.34895609	9.42646986	8.63759278	5.77499545	1.65359071
ENSG00000207176	0	0	0	0	0	0	0	0	0	0	0	0	0	0	0	0	0	0	0	0	0	0	0	0	0	0	0	0	0	0	0	0	0	0	0	0	0	0	0	0	0	0	0	0	0	0	0	0
ENSG00000207177	0	0.16110441	0	0	0	0	0	0	0	0	0	0	0	0	0	0	0	0	0	0	0	0	0	0	0	0	0	0	0	0	0	0	0	0.37854497	0	0.21395232	0	0	0	0	0	0	0	0	0	0	0	0
ENSG00000207178	0	0	0	0	0	0	0	0	0	0	0	0	0	0	0	0	0	0	0	0	0	0	0	0	0	0	0	0	0	0	0	0	0	0	0	0	0	0	0	0	0	0	0	0	0	0	0	0
ENSG00000207180	0	0	0	0	0	0	0	0	0	0	0	0	0	0	0	0	0	0	0	0	0	0	0	0	0	0	0	0	0	0	0	0	0	0	0	0	0	0	0	0	0	0	0	0	0	0	0	0
ENSG00000207181	3.34234338	3.35813187	1.86785817	3.7031735	2.26395108	2.5071749	5.36830366	10.289249	7.90803753	8.43301514	16.843035	8.66943406	1.61656726	2.84498635	1.45341227	1.38183475	1.79574923	3.74370126	11.0349178	1.48387327	3.6174305	3.87413208	5.80419266	2.88546315	3.64646458	3.73301762	5.34080981	5.94048653	2.57157423	3.84088076	12.6955583	10.8073744	5.87487157	9.39352324	2.78976225	5.30918713	6.62774648	11.5474102	8.89749666	16.873327	19.6235307	16.2451976	3.82680209	3.05737141	3.08307333	3.60699476	2.12592425	5.80321874
ENSG00000207182	0	0	0.19337489	0	0	0	0	0	0	0	0	0	0	0	0	0	0	0	0	0	0	0	0	0	0	0	0	0	0	0	0	0	0	0	0	0	0	0	0	0	0	0	0	0	0	0	0	0
ENSG00000207183	0	0	0	0	0	0	0	0	0	0	0	0	0	0	0	0	0	0	0	0	0	0	0	0	0	0	0	0	0	0	0	0	0	0	0	0	0	0	0	0	0	0	0	0	0	0	0	0
ENSG00000207184	0	0	0	0	0	0	0	0	0	0	0	0	0	0	0	0	0	0	0	0	0	0	0	0	0	0	0	0	0	0	0	0	0	0	0	0	0	0	0	0	0	0	0	0	0	0	0	0
ENSG00000207185	0.30405415	0.20366029	0	0	0	0.22807858	0.48835647	1.4891173	0.601287	0.82616621	0.3830542	0.77032049	0.51470891	0	0.79330454	0.58662796	0	1.10028956	0	0.53995389	0.51190054	0	0.61601101	0.28268326	0.29025514	0.7506813	0.26161441	1.60484842	0.21834121	0.73375316	0	0.37200294	0.49880985	0	0	1.0818721	0.2813666	0.6127753	0.30626263	1.46520099	0	2.17785668	1.04437658	0.25958814	0.56093652	0	0	2.55838562
ENSG00000207186	0	0	0	0	0	0	0	0	0	0	0	0	0	0	0	0	0	0	0	0	0	0	0	0	0	0	0	0	0	0	0	0	0	0	0	0.26545936	0	0	0	0	0	0	0	0	0	0	0	0
ENSG00000207187	0	0	0	0	0	0	0	0	0.1198053	0	0	0	0	0.26252437	0	0	0.3645506	0	0	0	0	0	0	0	0	0	0	0.18272173	0	0	0	0	0	0	0	0	0	0	0	0	0	0	0	0	0	0	0	0
ENSG00000207188	0	0	0	0	0	0	0	0	0	0	0	0	0	0	0	0	0	0	0	0	0	0	0	0	0	0	0	0	0	0	0	0	0	0	0	0	0	0	0	0	0	0	0	0	0	0	0	0
ENSG00000207189	0.63195568	0.21164697	0	0	0	0	0.25375385	0.61900562	0	0.57237659	0	0.26684305	0	0	0.82441452	0	0	0	0.34773901	0.28056427	0.53197507	0	1.28033662	0	0	0	0.2718738	0.23825481	0.22690361	0	0	0	0	0	0	0.28107461	0	0	0.31827293	0	0	0	0	0.26976807	0.58293403	0	0	0.59082544
ENSG00000207190	0	0	0	0	0	0	0	0	0	0	0	0	0	0	0	0	0	0	0	0	0	0	0	0	0	0	0	0	0	0	0	0	0	0	0	0	0	0	0	0	0	0	0	0	0	0	0	0
ENSG00000207191	0.33926042	0	0.3317906	0	0.37849337	0.25448768	0.2724515	0	0	0	0	0.28650516	0	0	0	0	0	0	0.74672376	0.30123743	0	0.28975503	0	0	0	0	0.87571983	0	0	0	0	0	0	0	0	0	0.62789177	0	0	0	0	0	0	0.28964571	0	0	0.30210503	0
ENSG00000207192	0	0	0	0	0	0	0	0	0	0	0	0	0	0	0	0	0	0	0	0	0	0	0	0	0	0	0	0	0	0	0	0	0	0	0	0	0	0	0	0	0	0	0	0	0	0	0	0
ENSG00000207193	0	0	0	0	0	0	0	0	0	0	0	0	0	0	0	0	0	0	0	0	0	0	0	0	0	0	0	0	0	0	0	0	0	0	0	0	0	0	0	0	0	0	0	0	0	0	0	0

ENSG00000207194	0	0	0	0	0	0	0	0	0	0	0	0	0	0	0	0	0	0	0	0	0	0	0	0	0	0	0	0	0	0	0	0	0	0	0	0	0	0	0	0	0	0	0	0	0	0	0	0
ENSG00000207195	1.45179008	0.77794561	1.41982462	0.3753216	0.64787153	2.61365723	0	0	0.287101	1.05193536	0.73159901	0.49041425	0.24576191	0	0.25252337	0.28010164	1.3104116	0.35024232	0.31954395	0	0	0.49597708	0.29413138	0.26994978	0.27718058	0	0.24982998	0.4378737	0	0	0	0	0	0	0	0.25828478	0	0.29258641	0.29246702	0.46640032	0.54241946	0	0.66488839	0.49578996	0.80350367	0	0.25855836	0.54292067
ENSG00000207196	0	0	0	0	0	0	0	0	0	0	0	0	0	0	0	0	0	0	0	0	0	0	0	0	0	0	0	0	0	0	0	0	0	0	0	0	0	0	0	0	0	0	0	0	0	0	0	0
ENSG00000207197	0.35032326	0.93860829	1.37043942	0.9056674	0.39083554	1.05114476	0.56267158	0.34314442	0	0	0.44134506	0.29584772	0.29651709	0.37951893	0.30467493	0.33794872	0	0.84514995	0.77107345	2.48848313	0.88469768	0.89761069	2.48413137	1.30280109	3.00981961	1.44152568	0.3014253	0.26415207	1.00626818	1.12721501	0	0.85722417	0	0	0	0	0.97254976	0.70602372	1.41147127	0	0.65444087	0	0	0.29909068	0	0	0	0.65504559
ENSG00000207198	0	0	0	0	0	0	0	0	0	0	0	0	0	0	0	0	0	0	0	0	0	0	0	0	0	0	0	0	0	0	0	0	0	0	0	0	0	0	0	0	0	0	0	0	0	0	0	0
ENSG00000207199	0	0	0	0	0	0	0	0	0	0	0	0	0	0	0	0	0	0	0	0	0	0	0	0	0	0	0	0	0	0	0	0	0	0	0	0	0	0	0	0	0	0	0	0	0	0	0	0
ENSG00000207200	0	0.40351384	0	0	0	0.45189401	0.72568858	0	0	0.54563002	0.75894851	0.25437374	0	0	0.52392699	0.29057273	0.45313298	0.72667099	0	0.53490759	0	0	0.91538085	0	0	0.24788853	0.77750826	0	0	0.24229855	0.4576503	0.36852628	0	0.47406566	0.58663225	0	0.278737	0	0.60680073	0	0.56269682	0.53937572	0.34487202	0.25716208	0	0	0.26822409	0.56321677
ENSG00000207201	0	0	0	0	0	0	0	0	0	0	0	0	0	0	0	0	0	0	0	0	0	0	0	0	0	0	0	0	0	0.1600367	0	0	0	0	0	0	0	0	0	0	0	0	0	0	0	0	0	0
ENSG00000207202	0.31291009	0	0	0	0	0	0	0	0	0	0	0	0	0	0	0	0	0	0	0	0	0	0	0	0	0	0	0	0	0	0	0	0	0	0	0	0	0	0	0	0	0	0	0	0	0	0	0
ENSG00000207203	0	0	0	0	0	0	0	0	0	0	0	0	0	0	0	0	0	0	0	0	0	0	0	0	0	0	0	0	0	0	0	0	0	0	0	0	0	0	0	0	0	0	0	0	0	0	0	0
ENSG00000207204	0	0	0	0	0	0	0	0	0	0	0	0	0	0	0	0	0	0	0	0	0	0	0	0	0	0	0	0	0	0	0	0	0	0	0	0	0	0	0	0	0	0	0	0	0	0	0	0.28160839
ENSG00000207205	1.56265405	3.66341658	1.91030949	1.2624455	1.30752255	1.46523208	1.25492813	2.29594813	5.31136849	1.06149841	9.84333217	2.30940527	1.32264594	3.59737943	3.39758713	3.2033442	3.82004837	2.59179318	4.72925047	1.73439733	2.7952872	6.00583154	3.95740409	2.90564122	0.74586775	2.57203128	4.20168604	1.4728479	3.22615677	1.41414246	0.59355857	0.71695113	0.96134262	3.38166836	1.14126637	2.2588178	0.72302689	2.95246282	1.57400432	0.94128064	7.29800728	3.14799284	1.11822139	1.66765713	4.50449026	5.09748847	3.13090663	1.2783314
ENSG00000207206	0	0	0	0	0	0	0	0.29504006	0	0	0	0	0	0	0	0	0	0	0	0	0	0	0	0	0	0	0	0	0	0	0	0	0	0	0	0	0	0	0	0	0	0	0	0	0	0	0	0
ENSG00000207207	0	0	0	0	0	0	0	0.27937422	0.14100978	0	0.35932518	0	0	0.30898886	0.49610785	0	0.42907283	0	0.62777661	0	0	0	0.28892552	0	0	0	0	0	0	0	0	0	0	0	0	0	0	0	0.57458122	0	0	0	0	0	0.52618824	0	0	0.26665573
ENSG00000207208	0	0	0	0	0	0.23025076	0.24650374	0	0	0	0	0	0	0	0.26695327	0	0	0.37025617	0	0	0	0	0	0	0	0.25261021	0	0	0	0	0.46636745	0.37554583	0	0	0	0	0	0	0	0	0	0	0	0.52412081	0	0	0	0
ENSG00000207209	0	0	0	0	0	0	0	0	0	0	0	0	0	0	0	0	0	0	0	0	0	0	0	0	0	0	0	0	0	0	0	0	0	0	0	0	0	0	0	0	0	0	0	0	0	0	0	0
ENSG00000207210	0	0	0	0	0	0	0	0	0	0	0	0	0	0	0	0	0	0	0	0	0	0	0	0	0	0	0	0	0	0	0	0	0	0	0	0	0	0	0	0	0	0	0	0	0	0	0	0
ENSG00000207212	0	0	0	0	0	0.47404567	0.76126155	0	0	0.2861883	0	0	0.26744679	0	0.27480484	0.60963298	0	0	0	0	0	0.26986988	0	0	0	0	0.2718738	0	0	0	0	0	0	0	0	0	0	0.63680571	0	0	0	0	0.36177751	0	0	0	0	0
ENSG00000207213	0	0	0	0	0	0	0	0	0	0	0	0	0	0	0	0	0	0	0	0	0	0	0	0	0	0	0	0	0	0	0	0	0	0	0	0	0	0	0	0	0	0	0	0	0	0	0	0
ENSG00000207214	0	0	0	0	0	0	0	0	0	0	0	0	0	0	0	0	0	0	0	0	0	0	0	0	0	0	0	0	0	0	0	0	0	0	0	0	0	0	0	0	0	0	0	0	0	0	0	0
ENSG00000207215	0	0	0	0	0	0	0	0	0	0	0	0	0	0	0	0	0	0	0	0	0	0	0	0	0	0	0	0	0	0	0	0	0	0	0	0	0	0	0	0	0	0	0	0	0	0	0	0
ENSG00000207217	1.67117169	2.07884354	4.66964543	8.3321404	2.39712467	5.0143498	10.7366073	14.9661804	13.219406	13.8387941	42.1075876	24.7986137	2.62692179	8.27632391	6.43654005	4.14550426	6.82384709	10.0791957	13.9250153	5.08756551	5.62711411	11.2145928	6.52971674	9.98814168	13.9021462	17.2897658	13.1466088	28.0822999	21.4297853	28.4225176	22.1265445	12.2678304	7.83316209	9.01778231	9.76416787	9.98127181	55.2312207	55.0907693	52.4230885	51.0034656	38.801072	36.3379421	11.2070633	7.54151615	13.2131714	8.19771537	9.99184399	8.25842667
ENSG00000207218	0	0	0	0	0	0	0	0	0	0	0	0	0	0	0	0	0	0	0	0	0	0	0	0	0	0	0	0	0	0	0	0	0	0	0	0	0	0	0	0	0	0	0	0	0	0	0	0
ENSG00000207220	0	0	0	0	0	0	0	0	0	0	0	0	0	0	0	0	0	0.23419818	0	0	0	0	0	0	0	0	0	0	0	0	0	0	0	0	0	0	0	0	0	0	0	0	0	0	0	0	0	0
ENSG00000207221	0	0	0	0	0	0	0.19172513	0	0.11803041	0	0	0	0.20207091	0	0	0	0	0	0	0.2119819	0	0	0	0	0	0	0.20541576	0	0.17143828	0	0	0.2920912	0	0	0	0	0	0.24057104	0	0	0	0.4275052	0	0	0	0	0	0
ENSG00000207222	0	0	0	0	0	0	0	0	0	0	0	0	0	0	0	0	0	0	0	0	0	0	0	0	0	0	0	0	0	0	0	0	0	0	0	0	0	0	0	0	0	0	0	0	0	0	0	0
ENSG00000207223	0	0.42329393	0	0.4084383	0	0	0	0.30950281	0.31243344	0.2861883	0	0	0	0	0	0	0	0	0	0	0	0	0	0	0	0	0	0	0.68071083	0.25417593	0	0.38659129	0	0	0	0	0	0	0	0	0.59028	0	0	0	0	0	0	0
ENSG00000207225	0	0	0	0	0	0	0	0	0	0	0	0	0	0	0	0	0	0	0	0	0	0	0	0	0	0	0	0	0	0	0	0	0	0	0	0	0	0	0	0	0	0	0	0	0	0	0	0
ENSG00000207227	0	0	0.30902065	0	0.35251833	0.23702284	0	0.92850844	0.46865016	0.2861883	0.79615187	0.26684305	0	0.34231119	0.27480484	0.30481649	0.47534539	0	0	0	0	0	0	0.29376887	0	0.52007985	0	0.23825481	0	0.50835187	0	0	0	0	0	0.28107461	0	0	0	0.50755328	0	0	0	0.26976807	0.29146702	0.4339967	0.28137233	0
ENSG00000207229	0	0	0	0	0	0	0	0	0	0	0	0	0	0	0	0	0	0	0	0	0	0	0	0	0	0	0	0	0	0	0	0	0	0	0	0	0	0	0	0	0	0	0	0	0	0	0	0
ENSG00000207231	0.28271702	0.75747335	0.55298433	0	0.94623342	0.84829226	1.13521459	0.55384714	0.27954571	1.28031606	0	0.47750861	0.2392945	0.61255687	0.24587802	0.27273054	0.85061806	0.68205084	0.6222698	1.00412477	0.23798885	0.48292505	2.29112868	0.26284583	0.26988636	0	0	0	0	0.22742057	0	0	0	0	0.55061097	0	0	0	0	0	0.52814526	0.50625616	0	0.48274285	0	0	0.25175419	0.52863329
ENSG00000207233	2.99811533	3.68167281	4.1538125	7.4278771	3.90229597	2.62378768	2.00642579	5.38390938	3.70560592	3.62061473	16.9969166	5.27480439	1.69175643	18.4051971	17.1657163	2.65119458	6.76538083	22.0001049	15.6725161	3.54946431	5.04757744	6.8283356	0.75926939	3.2519531	7.15512672	5.962776	6.66406949	7.15872584	7.89413486	9.6468633	12.906448	7.64192092	6.96787093	11.010083	7.29879658	10.0010269	10.1728202	13.0915406	11.5762527	3.21056961	3.26713117	3.13172414	13.7307184	7.46567438	25.5813145	10.6379655	14.2387485	10.5111967
ENSG00000207234	0.30121252	0.60527076	0.58916087	0.7787047	0	0	0.48379239	0.29504006	0	0.27281501	0	0.50874749	0	0	0.26196349	0	0.45313298	1.09000648	0.33148952	0.26745379	0.25355821	0	0.6102539	0.56008271	0	0.49577706	0.51833884	0.22712141	0.21630064	0	0	0.36852628	0	0.47406566	0	0	0.278737	0	0.30340037	0.96767168	0	0.53937572	0.34487202	0.25716208	0	0	0	0.56321677
ENSG00000207235	0	0	0	0	0	0	0.26144336	0	0	0	0	0	0.27555124	0	0	0	0	0	0	0	0	0	0.32978367	0	0	0	0	0	0	0	0	0	0	0	0	0	0	0	0	0	0	0	0	0	0	0	0	0
ENSG00000207237	0	0	0	0	0	0	0	0	0	0	0	0	0	0	0	0	0	0	0	0	0	0	0	0	0	0	0	0	0	0	0	0	0	0	0	0	0	0	0	0	0	0	0	0	0	0	0	0
ENSG00000207240	0	0	0	0	0	0	0	0	0	0	0	0	0	0	0	0	0	0	0	0	0	0	0	0	0	0	0	0	0	0	0	0	0	0	0	0	0	0	0	0	0	0	0	0	0	0	0	0
ENSG00000207241	0	0	0	0	0	0	0	0	0	0	0	0	0	0	0	0	0	0	0	0	0	0	0	0	0	0	0	0	0	0	0	0	0	0	0	0	0	0	0	0	0	0	0	0	0	0	0	0
ENSG00000207242	0	0	0	0	0	0	0	0	0	0	0	0	0	0	0	0	0	0	0	0	0	0	0	0	0	0	0	0	0	0	0	0	0	0	0	0	0	0	0	0	0	0	0	0	0	0	0	0
ENSG00000207243	0	0	0	0	0	0	0	0	0	0	0	0	0	0	0	0	0	0	0	0	0	0	0	0	0	0	0	0	0	0	0	0	0	0	0	0	0	0	0	0	0	0	0	0	0	0	0	0
ENSG00000207244	0.24052045	0	0	0	0	0	0	0	0	0	0	0	0	0	0	0	0	0	0	0	0	0	0	0	0	0	0	0	0	0	0	0	0	0	0	0	0	0	0	0	0	0	0	0	0	0	0	0
ENSG00000207245	0.77662024	0.26009627	0.75952064	0.5019362	0.21660765	0.29128108	0	0	0.19197717	0.70340256	0	0	0	0.84134317	0	0	0	0.46839636	0.42734191	0.34478983	0	0.66329465	0	0	0	0.63913428	0.33410997	0	0.2788454	0	1.17996583	0.4750881	0.63703427	0	0	0.69083399	1.79667826	1.17387076	1.56452237	0	0	0	0	0	0	0	0	0
ENSG00000207247	0	0	0	0	0	0	0	0	0	0	0	0	0	0	0	0	0	0	0	0	0	0	0	0	0.30163769	0	0	0	0	0	0	0	0	0	0	0	0	0	0	0	0	0	0	0	0	0	0	0
ENSG00000207248	0	0.60527076	0.29458044	0	0.33604551	0.45189401	0	0.29504006	0.89350124	0	0	0	0	0	0.52392699	0.58114546	1.35939895	0.36333549	0	0.53490759	0	0.25725914	0.91538085	0.28004136	0	0	0.25916942	0.22712141	0.43260127	0	0	0	0.49414808	0	0	0	0	0	0.60680073	0	0	1.07875144	0	0	0.27784706	0.82743295	0	0
ENSG00000207249	0	0	0	0	0	0	0	0	0	0	0	0	0	0	0	0	0	0	0	0	0	0	0	0	0	0	0	0	0	0	0	0	0	0	0	0	0	0	0	0	0	0	0	0	0	0	0	0
ENSG00000207251	0	0	0	0	0	0.23246471	0	0	0	0	0	0	0	0	0	0	0	0	0	0	0	0	0	0	0	0	0	0	0	0	0	0	0	0	0	0	0	0	0	0	0	0	0	0	0	0	0	0
ENSG00000207252	0	0	0	0	0	0	0	0	0	0	0	0	0	0	0	0	0	0	0	0	0	0	0	0	0	0	0	0	0	0	0	0	0	0	0	0	0	0	0	0	0	0	0	0	0	0	0	0
ENSG00000207255	0	0	0	0	0	0	0	0	0	0	0	0	0	0	0	0	0	0	0	0	0	0	0	0	0	0	0	0	0	0	0	0	0	0	0	0	0	0	0	0	0	0	0	0	0	0	0	0
ENSG00000207256	0	0.60527076	0	0	0	0.225947	0	0	0	0	0	0	0	0	0	0	0	0	0	0	0	0.25725914	0	0	0	0	0	0	0	0	0	0	0	0	0	0	0	0	0	0	0	0	0	0	0	0	0	0
ENSG00000207257	0	0	0	0	0	0	0	0	0	0	0	0	0	0	0	0	0	0	0	0	0	0	0	0	0	0	0	0	0	0	0	0	0	0	0	0	0	0	0	0	0	0	0	0	0	0	0	0
ENSG00000207258	0	0	0	0	0	0	0	0	0	0	0	0	0	0	0	0	0	0	0	0	0	0	0	0	0	0	0	0	0	0	0	0	0	0	0	0	0	0	0	0	0	0	0	0	0	0	0	0
ENSG00000207260	0.30121252	0	0.29458044	0	0	0.225947	0.24189619	0	0	0.27281501	0.75894851	0.25437374	0.25494928	0	0	0	0	0	0	0	0	0	0.30512695	0	0.57508495	0	0	0	0	0.24229855	0	0	0	0.47406566	0	0	0	0	0	0	0	0.53937572	0	0	0	0	0	0
ENSG00000207261	0	0	0	0	0	0	0	0	0	0	0	0	0	0	0	0	0	0.36676319	0	0	0	0	0	0	0	0	0	0	0	0	0	0	0	0	0	0	0	0	0	0	0	0	0	0	0	0	0	0
ENSG00000207263	0	0.46930414	0	0	0	0	0	0.34314442	0	0	0.44134506	0	0	0	0	0	0	0	0	0.62212078	0	0.29920356	0.35487591	0	0.3344244	0.28830514	0	0.26415207	0	0	0	0.42861209	0	0.55135897	0	0	0	0.35301186	0	0	0	0	0	0	0	0	0	0
ENSG00000207264	0	0	0	0	0	0	0	0	0	0	0	0	0	0	0	0	0	0	0	0	0	0	0	0	0	0	0	0	0	0	0	0	0	0	0	0	0	0	0	0	0	0	0	0	0	0	0	0
ENSG00000207265	0	0	0	0	0	0	0	0	0	0	0	0	0	0	0	0	0	0	0	0	0	0	0	0	0	0	0	0	0	0	0	0	0	0	0	0	0	0	0	0	0	0	0	0	0	0	0	0
ENSG00000207266	0	0	0	0	0	0	0	0	0	0	0	0	0	0	0	0	0	0	0	0	0	0	0	0	0	0	0	0	0	0	0	0	0	0	0	0	0	0	0	0	0	0	0	0	0	0	0	0
ENSG00000207267	0	0	0	0	0	0	0	0	0	0	0	0	0	0	0	0	0	0	0	0	0	0	0	0	0	0	0	0	0	0	0	0	0	0	0	0	0	0	0	0	0	0	0	0	0	0	0	0
ENSG00000207268	0	0	0	0	0	0	0	0	0	0	0	0	0	0	0	0	0	0	0	0	0	0	0	0	0	0	0	0	0	0	0	0	0	0	0	0	0	0	0	0	0	0	0	0	0	0	0	0
ENSG00000207269	0.19592547	0.13123399	0.0958058	0.1266283	0.0546457	0.22045285	0	0.19191056	0.09686386	0	0.12341564	0	0.08291663	0.10612687	0.08519785	0	0	0	0	0.17396691	0	0.08366787	0	0.18215456	0.28055056	0.08062028	0.16857828	0.14773246	0.14069403	0.15760453	0.29768135	0.2397101	0	0	0	0.08714167	0	0.29614369	0	0.15735695	0.18300474	0.35084013	0.33648607	0.3345452	0.54218181	0.26910433	0.08723397	0
ENSG00000207270	0	0	0	0	0	0	0	0	0	0	0	0	0	0	0	0	0	0	0	0	0	0	0	0	0	0	0	0	0	0	0	0	0	0	0	0	0	0	0	0	0	0	0	0	0	0	0	0
ENSG00000207271	0	0	0	0	0	0	0	0	0	0	0	0	0	0	0	0	0	0	0	0	0	0	0	0	0	0	0	0	0	0	0	0	0	0	0	0	0	0	0	0	0	0	0	0	0	0	0	0
ENSG00000207274	0.23873881	0.15991104	0	0.3085978	0	0.17908392	0.76690052	0.93538628	0.35409123	0.43246231	0.90230545	1.00807373	1.01035454	0.25863512	0.20763032	0	0.71829969	0.86393106	1.05094455	0.42396379	1.00484181	1.83511519	0.48368272	0.66587611	2.05113633	1.96474611	1.84874186	0.72005897	1.02862969	0.57613211	9.79371642	5.84182399	8.6164783	3.75740929	4.64960375	9.55653684	1.10462441	1.20285522	2.64520171	0.76696941	0	1.2825156	1.36671503	1.22294857	0.66065857	0.32790861	0.21259243	0
ENSG00000207275	0	0	0	0	0	0	0	0	0	0	0	0	0	0	0	0	0	0	0	0	0	0	0	0	0	0	0	0	0	0	0	0	0	0	0	0	0	0	0	0	0	0	0	0	0	0	0	0
ENSG00000207276	0	0	0	0	0	0	0	0	0	0	0	0	0.26230358	0	0	0	0	0	0	0	0	0	0.62785738	0	0.29583697	0	0	0	0.22254008	0.24928793	0	0	0.50840235	0	0	0.55133866	0	0	0	0	0	0	0	0	0	0	0	0
ENSG00000207277	0	0	0	0.3530568	0	0	0.4386931	0	0	0.2473831	0	0	0	0	0	0.52697088	0.41089177	0	0.30058795	0.24252166	0	0.23327736	0.55336583	0.25393581	0.52147534	0.67434083	0.70502867	1.02974535	0.39227404	0.4394228	0.41498798	0.33417214	0	0.85974619	0	0	0.25275304	0	0.82535184	0	0	0	0.93816879	0.46637869	0.50389213	0.37514969	0.72966044	0.76607027
ENSG00000207278	0	0	0	0	0	0	0	0	0	0	0	0	0	0	0	0	0	0	0	0	0	0	0	0	0	0	0	0	0	0	0	0	0	0	0	0	0	0	0	0	0	0	0	0	0	0	0	0
ENSG00000207279	6.30581865	7.74351836	8.22263651	5.8868384	1.17250663	5.78129616	0.28133579	2.40201095	0.3463936	1.5864786	6.17883079	1.47923862	0.59303418	2.27711358	1.21869973	0.33794872	1.05402672	2.53544985	5.78305085	5.28802665	1.47449613	4.48805346	3.19388319	5.8626049	1.672122	0.57661027	3.31567833	2.11321655	1.76096931	1.40901876	6.38720636	3.42889669	4.02300988	8.27038459	3.41139405	5.92089782	2.59346602	7.06023719	1.41147127	1.68816636	3.27220435	1.88195224	5.61541611	1.19636273	6.7861125	0.9623405	2.18369393	1.31009119
ENSG00000207280	0.80574349	1.61909929	1.182004	1.0415176	2.02257394	3.32424529	3.55889774	4.34077694	3.78435005	2.91912063	3.04528089	6.1240479	3.40994656	4.36446769	2.45263321	4.66369229	8.48491513	1.94384489	3.10357062	1.4308778	3.05220698	3.78492509	3.67296567	2.9964425	2.30752837	2.65240725	1.73319549	1.5188744	1.15720841	2.59259451	0	0.4929039	4.62646136	1.26812564	1.56924126	0.71674026	0.74562148	3.24770911	4.86957588	2.58852175	0.752607	1.44283005	0.46126632	1.03186285	0.37162045	1.66003736	1.07624915	3.76651216
ENSG00000207281	0.30405415	0.81464115	0	0	0	0.22807858	0.24417823	0.59564692	0.601287	0	0.7661084	0.51354699	0	0.32939379	0.26443485	0.58662796	0.45740782	0.36676319	0.33461678	0	0.51190054	0.25968611	0.30800551	0.28268326	0	0	0	0.22926406	0.21834121	0.24458439	0.46196776	0	0	0	0.59216651	0.27046802	0.2813666	0.6127753	1.53131317	0.48840033	0.56800528	0	0	0	0	0	0	0.28426507
ENSG00000207282	0	0	0	0	0	0	0	0	0	0	0	0	0	0	0	0	0	0	0	0	0	0	0	0	0	0	0	0	0	0	0	0	0	0	0	0	0	0	0	0	0	0	0	0	0	0	0	0
ENSG00000207283	0	0	0	0	0	0	0	0	0	0	0	0	0	0	0	0	0	0	0	0	0	0	0	0	0	0	0	0	0	0	0	0	0	0	0	0	0	0	0	0	0	0	0	0	0	0	0	0
ENSG00000207286	0	0	0	0	0	0	0	0	0	0	0	0	0	0	0	0	0	0	0	0	0	0	0	0	0	0	0	0	0	0	0	0	0	0	0	0	0	0	0	0	0	0	0	0	0	0	0	0
ENSG00000207287	0	0	0	0	0	0	0	0	0	0	0	0	0	0	0	0	0	0	0	0	0	0	0	0	0	0	0	0	0	0	0	0	0	0	0	0	0	0	0	0	0	0	0	0	0	0	0	0
ENSG00000207289	0	0	0	0	0	0	0	0	0	0	0	0	0	0	0	0	0	0	0	0	0	0	0	0	0	0	0	0	0	0	0	0	0	0	0	0	0	0	0	0	0	0	0	0	0	0	0	0
ENSG00000207290	0	0.18936834	0.82947649	0.3654448	0.94623342	0.42414613	0	0	0.27954571	0	0	0	0	0	0	0	0	0	0.3111349	0	0	0	0.28639109	0	0	0	0	0	0	0	0	0	0	0	0	0	0	0	0	0	0.52814526	0.50625616	0.32369567	0.24137143	0.26078628	0	0	0.26431664
ENSG00000207291	0.30121252	0	0	0	0	0	0	0	0	0	0	0	0	0	0	0	0	0	0	0	0	0	0	0	0	0	0	0	0	0	0.4576503	0.36852628	0	0	0	0	0	0	0	0	0	0	0	0	0	0	0	0
ENSG00000207292	0	0	0	0	0	0	0	0	0	0	0	0	0	0	0	0	0	0	0	0	0	0	0	0	0	0	0	0	0	0	0	0	0	0	0	0	0	0	0	0	0	0	0	0	0.55569413	0	0	0
ENSG00000207293	0	0	0	0	0.17625917	0	0	0	0.15621672	0	0	0	0	0	0	0	0	1.14343817	0	0	0	0	0	0	0	0.26003993	0	0	0	0	0	0	0	0	0	0.28107461	0	0	0	0	0	0	0	0.8093042	0	0	0	0.29541272
ENSG00000207294	0	0	0	0	0	0	0	0	0	0	0	0	0	0	0	0	0	0	0	0	0	0	0	0	0	0	0	0	0	0	0	0	0	0	0	0	0	0	0	0	0	0	0	0	0	0	0	0
ENSG00000207295	0	0	0	0	0	0	0	0	0	0	0	0	0.25494928	0	0	0	0.45313298	0	0	0	0	0	0	0	0	0	0	0	0	0	0	0	0	0	0	0	0	0	0	0	0	0	0	0	0	0	0	0
ENSG00000207296	0	0	0	0	0	0	0	0	0	0	0	0	0	0	0	0	0	0	0	0	0	0	0	0	0	0	0	0	0	0	0	0	0	0	0	0	0	0	0	0	0	0	0	0	0	0	0	0
ENSG00000207297	1.32906143	0.89022642	0	0	0	0.99696204	2.4015055	0.65091313	1.64269128	2.10658189	1.67438125	0.56119568	1.12493082	0.3599561	0.86691012	0.64105736	0.99969545	0.80158552	0.3656637	0.29502635	1.11879294	1.9864649	3.70241671	1.85346959	0.95155809	2.73444047	3.14476708	2.00428786	2.14739704	5.3455557	0	1.21955604	2.18036471	0.52293841	0.6471098	0.88668899	2.4597822	1.33926149	3.6814663	1.60114747	1.86212041	0.59498146	0.76085167	0	0.91947327	2.2818383	1.77525633	1.55320089
ENSG00000207298	0	0	0	0	0	0	0	0	0	0	0	0	0	0	0	0	0	0	0	0	0	0	0	0	0	0	0	0	0	0	0	0	0	0	0	0	0	0	0	0	0	0	0	0	0	0	0	0
ENSG00000207299	0	0	0	0	0	0	0	0	0	0	0	0	0	0	0	0	0	0	0	0	0	0	0	0	0	0	0	0	0	0	0	0	0	0	0	0	0	0	0	0	0	0	0	0	0	0	0	0
ENSG00000207300	0	0	0	0	0	0	0	0	0	0	0	0	0	0	0	0	0	0	0	0	0	0	0	0	0	0	0	0	0	0	0	0	0	0	0	0	0	0	0	0	0	0	0	0	0	0	0	0
ENSG00000207302	0	0	0	0	0	0	0	0	0	0	0	0	0	0	0	0	0	0	0	0	0	0.25024298	0	0	0	0	0.25210116	0	0	0	0	0	0	0	0	0	0	0	0	0	0	0	0	0	0	0	0	0
ENSG00000207303	0	0	0	0	0	0	0	0	0	0	0	0	0	0	0	0	0	0	0	0	0	0	0	0	0	0	0	0	0	0	0	0	0	0	0	0	0	0	0	0	0	0	0	0	0	0	0	0
ENSG00000207304	0	0	0	0	0	0	0	0	0	0	0	0	0	0	0	0	0	0	0	0	0	0	0	0	0	0	0	0	0	0	0	0	0	0	0	0	0	0	0	0	0	0	0	0	0	0	0	0
ENSG00000207305	0	0	0	0	0	0.2238549	0	0	0.14753801	0	0	0	0	0	0	0	0	0.35997128	0	0	0	0	0	0	0	0	0	0	0	0	0	0.365114	0	0	0	0	0	0.30071381	0	0	0	0	0	0	0.2752744	0	0	0
ENSG00000207306	0	0	0	0	0	0	0	0	0	0	0	0	0	0	0	0	0	0	0	0	0	0	0	0	0	0	0	0	0	0	0	0	0	0	0	0	0	0	0	0	0	0	0	0	0	0	0	0
ENSG00000207307	0	0	0	0.3930255	0	0	0	0	0	0	0	0	0	0	0	0	0	0	0	0	0	0	0	0	0	0	0.26161441	0	0	0	0.92393551	0	0	0	0	0	0	0	0	0	0	0	0	0	0	0	0	0
ENSG00000207308	0	0	0	0	0	0	0	0	0	0	0	0	0	0	0	0	0	0	0	0	0	0	0	0	0	0	0	0	0	0	0	0	0	0	0	0	0	0	0	0	0	0	0	0	0	0	0	0
ENSG00000207309	0	0	0	0	0	0	0	0	0	0	0	0	0	0	0	0	0	0	0	0	0	0	0	0	0	0	0	0	0	0	0	0	0	0	0	0	0	0	0	0	0	0	0	0	0	0	0	0
ENSG00000207310	0	0	0	0	0	0	0	0	0	0	0	0	0	0	0	0	0	0	0	0	0	0	0	0	0	0	0	0	0	0	0	0	0	0	0	0	0	0	0	0	0	0	0	0	0	0	0	0
ENSG00000207312	0	0	0	0	0	0	0	0	0	0	0	0	0	0	0	0	0	0	0	0	0	0	0	0	0	0	0	0	0	0	0	0	0	0	0	0	0	0	0	0	0	0	0	0	0	0	0	0
ENSG00000207313	1.17626787	1.10303601	0.69022131	0.6081854	0.65614726	0.70587823	1.70033601	1.15216375	1.39568807	0.85229799	2.07464392	0.99335732	0.79648387	0.25485943	0.40919845	0.68083099	0.35390678	1.1350919	1.03560229	0	1.1882071	0.60277506	1.19155415	1.09359215	0.89830788	1.35524458	0.40483398	0.70954716	0.33787107	0.7569619	0.35743491	0.57565419	0.77188094	0.37025566	0	1.25560338	0	0.71117718	0.23696233	0	0.43947854	0.8425285	0.2693526	0.60254765	0.43400928	1.29248651	1.4664222	1.09971158
ENSG00000207316	0	0	0	0	0	0	0	0	0	0	0	0	0	0	0	0	0	0	0	0	0	0	0	0	0	0	0	0	0	0	0	0	0	0	0	0	0	0	0	0	0	0	0	0	0	0	0	0
ENSG00000207317	0	0	0	0	0	0	0	0	0	0	0	0	0	0	0	0	0	0	0	0	0	0	0	0	0	0	0	0	0	0	0	0	0	0	0	0	0	0	0	0	0	0	0	0	0	0	0	0
ENSG00000207318	0	0	0	0	0	0	0	0	0	0	0	0	0	0	0	0	0	0	0	0	0	0	0	0	0	0	0	0	0	0	0	0	0	0	0	0	0	0	0	0	0	0	0	0	0	0	0	0
ENSG00000207319	0	0	0	0	0	0	0	0	0	0	0	0	0	0	0	0	0	0	0	0	0	0	0	0	0	0	0	0	0	0	0	0	0	0	0	0	0	0	0	0	0	0	0	0	0	0	0	0
ENSG00000207320	0	0	0	0	0	0	0	0	0	0	0	0	0	0	0	0	0	0	0.31388831	0	0	0	0	0.2651719	0	0	0	0	0	0	0	0	0	0	0	0	0	0	0	0	0	0	0	0.24350746	0	0.39174923	0	0
ENSG00000207321	0	0	0	0	0	0	0	0	0	0	0	0	0	0	0	0	0	0	0	0	0	0	0	0	0	0	0	0	0	0	0	0	0	0	0	0	0	0	0	0	0	0	0	0	0	0	0	0
ENSG00000207322	0.1965228	0	0.57658732	0.5080573	0.32887381	0.44224993	0.15782252	0	0.09715918	0	0.74275144	0.49789007	0.66535543	0.63870259	0.34183041	0	0	0.23705425	3.67670387	0.17449729	0	0.1678459	0.19907673	0.18270991	0	0.3234643	0	0.88909721	0.28224595	0	0.59717783	0.24044093	0	0.61859787	0	0.3496294	0.36371779	0.39606209	0	0.31567338	0	0.35190977	0	0.16778258	0	0	0	0
ENSG00000207323	0	0	0	0	0	0	0	0	0	0	0	0	0	0	0	0	0	0	0	0	0	0	0	0	0	0	0	0	0	0	0	0	0	0	0	0	0	0	0	0	0	0	0	0	0	0	0	0
ENSG00000207325	0	0	0	0	0	0	0	0	0	0	0	0	0	0	0	0	0	0	0	0	0	0	0	0	0	0	0	0	0	0	0	0	0	0	0	0	0	0	0	0	0	0	0	0	0	0	0	0
ENSG00000207326	0	0.18936834	0	0.3654448	0	0.63621919	0	0	0.13977285	0	0	0.2387543	0	0	0	0	0	0	0	0.25103119	0	0.24146253	0.28639109	0	0	0	0	0	0	0	0	0	0	0	0	0	0	0	0	0	0.52814526	0	0	0.24137143	0	0	0	0
ENSG00000207327	0	0.20175692	0.58916087	0	0.16802276	0.45189401	0	0.59008013	0.14891687	0	0	0.25437374	0.25494928	0	0.26196349	0	0.90626597	0.36333549	0	0	0.25355821	0	0	0	0	0.24788853	0	0	0.86520255	0	1.3729509	0	0.98829615	0	0.58663225	0	0	0	0	0	0	0	0	0	0	0	0	0
ENSG00000207328	0	0	0	0	0	0	0	0	0	0	0	0	0	0	0	0	0	0	0	0	0	0	0	0	0	0	0	0	0	0	0	0	0	0	0	0	0	0	0	0	0	0	0	0	0	0	0	0
ENSG00000207329	0	0	0	0	0	0	0	0	0	0	0	0	0	0	0	0	0	0	0	0	0	0	0	0	0	0	0	0	0	0	0	0	0	0	0	0	0	0	0	0	0	0	0	0	0	0	0	0
ENSG00000207330	0	0	0	0	0	0	0	0	0	0	0	0	0	0	0	0	0	0	0	0	0	0	0	0	0	0	0	0	0	0	0	0	0	0	0	0	0	0	0	0	0	0	0	0	0	0	0	0
ENSG00000207331	0	0.61098087	0	0	0	0	0.48835647	2.38258769	0	0.27538874	0.3830542	0	0	0	0	0.87994194	0.45740782	0.73352637	0	0	0	0	0	0.28268326	0	0	0	0	0	0	0	0	0	0	0	0	0.2813666	0	0	0	0	0	0.34812553	0	0	0	0.2707545	0
ENSG00000207332	0	0	0	0	0	0	0	0	0	0	0	0	0	0	0	0	0	0	0	0	0	0	0	0	0	0	0	0	0	0	0	0	0	0	0	0	0	0	0	0	0	0	0	0	0	0	0	0
ENSG00000207333	0	0.20559991	0.30019149	0	0.17122319	0	0	0.60131975	0.30350677	0.27801149	0	0	0	0	0.26695327	0.29610745	0	0.74051234	0	0	0.25838789	0.26215931	0.62187778	0.28537548	0	0	0	0	0.22042065	0	0	0	0	0	0	0	0.28404628	0	0	0	0	0	0	0.26206041	0	0	0.27333312	0
ENSG00000207334	0	0	0	0	0	0	0	0	0	0	0	0	0	0	0	0.29057273	0	0	0	0	0.25355821	0	0	0	0	0	0	0	0	0	0.4576503	0	0	0	0	0.26794029	0	0	0	0	0	0	0	0	0	0	0	0.28160839
ENSG00000207336	0	0	0	0	0	0.22807858	0	0.29782346	0.15032175	0.55077748	0	0.51354699	0	0	0	0	0	0	0	0.26997694	0.25595027	0.51937222	2.15603855	0	0	0	0	0	0.21834121	0.24458439	0	0	0	0	0	0.27046802	0.2813666	0	0.30626263	0	0	0	0	0	0	0	0.2707545	0
ENSG00000207338	0.30121252	0	0	0	0	0	0	0	0	0	0	0	0	0	0	0	0	0	0	0	0	0	0	0	0	0	0	0	0	0	0	0	0	0	0	0	0	0	0	0	0	0	0	0	0	0	0	0
ENSG00000207339	0	0	0	0	0	0	0	0	0	0	0	0	0	0	0	0	0	0	0	0	0	0	0	0	0	0	0	0	0	0	0	0	0	0	0	0	0	0	0	0	0	0	0	0	0	0	0	0
ENSG00000207340	0	0	0	0	0	0	0	0	0	0	0	0	0	0	0	0	0	0	0	0	0	0	0	0	0	0	0	0	0	0	0	0	0	0	0	0	0	0	0	0	0	0	0	0	0	0	0	0
ENSG00000207341	0	0	0	0	0	0	0	0	0	0	0	0	0	0	0	0	0	0	0	0	0	0	0	0	0	0	0	0	0	0	0	0	0	0	0	0	0	0	0	0	0	0	0	0	0	0	0	0
ENSG00000207342	0	0.43612102	0.31838492	1.2624455	0.18160035	0.24420535	0.26144336	0.63776337	0.48285168	0.58972134	0.41013884	0	1.10220495	0.35268426	1.13252904	0.31405335	0.97949958	1.17808781	1.07482965	0.57813244	0.8221433	0	0.32978367	0	0	0.26791992	1.12044961	0	0.46755895	0.26187823	0	0	0	0	0	0	1.20504482	0.32805142	0.65583513	0	0	1.74888491	0.74548093	1.66765713	0	0	0.28989876	1.21745848
ENSG00000207343	0	0	0	0	0	0	0	0	0	0	0	0	0	0	0	0	0	0	0	0	0	0	0	0	0	0	0	0	0.22470066	0	0	0	0	0	0	0	0	0	0	0	0	0	0	0	0	0	0	0
ENSG00000207344	4.08884758	3.06098374	3.29314547	0.9327023	1.87834396	0.90210184	0.96577958	2.82710031	2.85386964	1.74275858	11.2114819	3.85926733	2.23936789	9.6409137	4.39277589	1.16012246	2.89464056	14.216179	4.49984653	0.4271277	1.01234062	3.69762017	1.46187688	2.01253601	1.37762888	1.18764504	0.82779486	2.90173019	1.38174138	1.74129482	25.2151654	11.4765684	12.6265896	11.356349	9.83703479	7.70228342	2.67088291	3.63549527	2.18040711	3.09077224	3.5945409	3.01486877	2.75382879	1.64276673	4.65912202	2.64284555	3.64104191	0.67459919
ENSG00000207345	0	0	0	0	0	0	0	0.29504006	0.44675062	1.09126005	0.37947425	0	0	0.32631534	0	0.29057273	0.45313298	0	0	0	0	0	0	0	0	0.24788853	0.51833884	0.22712141	0	0	0	0	0	0	0	0	0	0	0	0	0	0.53937572	0	0	0	0	0	0
ENSG00000207347	0	0	0	0	0	0	0	0	0	0	0	0	0	0	0	0	0	0	0	0	0	0	0	0	0	0	0	0	0	0	0	0	0	0	0	0	0	0	0	0	0	0	0	0	0	0	0	0
ENSG00000207349	0	0	0	0.2540287	0	0.29483329	0	0.19249565	0	0	0.49516762	0	0	0	0	0	0	0	0.4325534	0	0.16543127	0	0	0	0.18760393	0	0	0.14818287	0	0	0	0	0	0	0	0	0	0	0	0	0.36712537	0	0	0	0	0	0.17499986	0
ENSG00000207351	0	0	0	0	0	0	0	0	0	0	0	0	0	0	0	0	0	0	0	0	0	0	0	0	0	0	0	0	0	0	0	0	0	0	0	0	0	0	0	0	0	0	0	0	0	0	0	0.26665573
ENSG00000207352	0	0.21374248	0	0	0	0	0	0.3125672	0	0.28902184	0.40201728	0	0	0	0.27752568	0.30783447	0	0.38491978	0.35118197	0.28334214	0.80586323	0	0.3232533	0.59335495	0	0.26261458	0.27456562	0	0	0	0	0	0	0	0	0	0	0	0.32142415	0	1.19224871	0	0	0.54487807	0	0	0.28415819	0
ENSG00000207355	0	0	0	0	0	0	0	0	0	0	0	0	0	0	0	0	0	0	0	0	0	0	0	0	0	0	0	0	0	0	0	0	0	0	0	0	0	0	0	0	0	0	0	0	0	0	0	0
ENSG00000207356	0	0	0	0	0	0	0	0	0	0	0	0	0	0	0	0	0	0	0	0	0	0	0	0	0	0	0	0	0	0	0	0	0	0	0	0	0	0	0	0	0	0	0	0	0	0	0	0
ENSG00000207357	21.0848765	15.7370399	13.5507	13.23798	24.0272543	19.8833363	1.69327335	0.88512019	1.63808561	0.54563002	3.03579403	1.27186872	2.29454348	4.56841478	2.61963493	0.87171818	2.71879791	4.36002591	0.99446856	0.26745379	1.52134928	1.2862957	1.52563475	0.56008271	2.3003398	0.74366559	0.51833884	0.68136422	0.86520255	0	0.9153006	2.21115768	1.48244423	0	1.75989675	1.07176114	0	1.21409686	1.8204022	0	2.25078729	0	0.68974403	0.51432416	1.66708238	1.24114943	1.07289635	1.68965032
ENSG00000207359	0	0.6227305	0.30307795	1.201751	0.5186087	0.23246471	0.24887397	0.60710167	0.15321255	0	0	0	0.52460716	0	0.26952013	0.59790927	0.46620413	1.12144897	0.68210343	1.10067523	0.26087239	0	0.94178607	0	0.29583697	0.25503916	0.26664546	0.46734597	0.22254008	0.49857587	0	0.75831369	0.50840235	1.46322189	1.810663	0	0	0.31227972	0.6243046	0	0	0	0	0	0	1.27695182	1.65576793	0.86919511
ENSG00000207360	0	0	0	0	0	0	0	0	0	0	0	0	0	0	0	0	0	0	0	0	0	0	0	0	0	0	0	0	0	0	0	0	0	0	0	0	0	0	0	0	0	0	0	0	0	0	0	0
ENSG00000207361	0	0	0	0	0	0	0	0	0	0	0	0	0	0	0	0	0	0	0	0	0	0.26468008	0	0	0	0	0	0	0	0	0	0	0	0	0	0	0	0	0	0	0	0	0	0	0	0	0	0
ENSG00000207362	0	0	0	0	0	0	0	0	0	0	0	0	0	0	0	0	0	0	0	0	0	0	0	0	0	0	0	0	0	0	0	0	0	0	0	0	0	0	0	0	0	0	0	0	0	0	0	0
ENSG00000207363	0	0	0	0	0	0	0	0	0	0	0	0	0	0	0	0	0	0	0	0	0	0	0	0	0	0	0	0	0	0	0	0	0	0	0	0	0	0	0	0	0	0	0	0	0	0	0	0
ENSG00000207364	0	0	0	0	0	0	0	0	0	0	0	0	0	0	0	0	0	0	0	0	0	0	0	0	0	0	0	0	0	0	0	0	0	0	0	0	0	0	0	0	0	0	0	0	0	0	0	0
ENSG00000207365	0	0	0	0	0	0	0	0	0	0	0	0	0	0	0	0	0	0	0	0	0	0	0	0	0	0	0	0	0	0	0	0	0	0	0	0	0	0	0	0	0	0	0	0	0	0	0	0
ENSG00000207366	0	0	0	0	0	0	0	0	0	0	0	0	0	0	0	0	0	0	0	0	0	0	0	0	0	0	0	0	0	0	0	0	0	0	0	0	0	0	0	0	0	0	0	0	0	0	0	0
ENSG00000207367	0	0	0	0	0	0	0	0	0	0	0	0	0	0	0	0	0	0	0	0	0	0	0	0	0	0	0	0	0	0	0	0	0	0	0	0	0	0	0	0	0	0	0	0	0	0	0	0
ENSG00000207368	0	0	0.28396492	0.3753216	0.16196788	0.87121908	0.23317921	0	0	0	0	0.24520712	0	0.62911246	0	0	0.43680387	0	0.31954395	0.25781582	0	0.24798854	0	0.53989955	0.55436117	0.23895561	0	0.21893685	0.83402408	0.46713415	0	0	0	0	0	0	0	0.29258641	0	0	0	0	0	0	0	0	0	0.54292067
ENSG00000207369	0.30121252	0	0	0	0	0	0	0	0	0	0	0	0	0	0	0	0	0	0	0	0	0.25725914	0	0	0	0	0.25916942	0	0	0	0	0	0	0	0	0	0	0	0	0	0	0	0	0	0	0	0	0
ENSG00000207370	1.42609468	2.6746183	1.39469498	1.8433939	1.11370836	2.78134763	0.68715644	1.11749688	0.98706848	1.54997555	2.87460143	0.48173435	1.20706073	0.61797773	1.24026964	1.37572044	2.14536413	0.68808669	0.31388831	1.51951625	2.40094945	1.9487949	2.02247864	2.38654713	1.08909893	1.40835783	1.71785748	1.07530931	3.0722347	0.68829943	0.43335028	0.69791703	1.40373037	1.34668209	0.55548363	0	0.26393681	1.14963154	0.57458122	0	1.06563823	1.0214726	0.32656023	0.73052237	0.26309412	1.17524769	0.7619463	0.53331146
ENSG00000207371	0	0.1944864	0	0	0	0	0.69953764	0.56881598	0.4306515	0.52596768	0	0.24520712	0.24576191	0	0.25252337	0.56020328	0.43680387	0.35024232	0	0	0.24442098	0.24798854	0	0	0	0.23895561	0	0.21893685	0.20850602	0.23356707	0	0	0	0	0.56549235	0	0	0.58517281	0.29246702	0	0	0	0	0	0	0	0	0.27146034
ENSG00000207375	1.40129303	1.40791243	2.39826898	2.2641686	0.58625332	2.62786189	1.40667895	0.34314442	0	1.26918288	0.88269011	0.29584772	0.59303418	0.37951893	0	0.33794872	0.52701336	0	1.15661017	0.62212078	1.1795969	1.19681426	1.06462773	0.65140054	0.6688488	0.57661027	0.90427591	0.52830414	0.25156704	1.12721501	1.59680159	0.85722417	2.29886279	0	0	0.6232524	1.29673301	2.11807116	0.70573563	0.56272212	2.61776348	0.62731741	1.20330345	0.89727204	2.2620375	0	0.31195628	0.98256839
ENSG00000207378	0	0.20175692	0.29458044	0.3893524	0	0.225947	0	0	0.14891687	0	0	0	0	0	0	0	0	0	0	0	0.25355821	0	0	0	0	0	0	0	0.21630064	0.24229855	0	0.36852628	0	0	0	0	0	0	0	0	0	0.53937572	0	0	0	0	0	0
ENSG00000207379	0.30121252	0.20175692	0	0	0	0.225947	0	0	0.14891687	0	0	0	0	0	0	0	0.45313298	0	0	0	0.25355821	0.25725914	0	0	0	0	0	0	0	0	0	0	0.49414808	0	0	0	0	0	0	0	0	0	0	0	0	0	0	0
ENSG00000207380	0	0	0	0	0	0	0.91620859	0.27937422	0	0	0	0	0	0	0	0	0	0	0	0	0.24009494	0.48719872	0.28892552	0	0	0.46945261	0.24540821	0	0	0.22943314	0	0	0	0.44889403	0	0	0	0	0	0.45814544	0	0	0	0	0	0	0	0
ENSG00000207381	0	0	0	0	0	0	0	0	0	0	0	0	0	0	0	0	0	0	0	0	0	0	0	0	0	0	0	0	0	0	0	0	0	0	0	0	0	0	0	0	0	0	0	0	0	0	0	0
ENSG00000207382	0	0	0	0	0	0	0	0	0	0	0	0	0	0	0	0	0	0	0	0	0	0	0	0	0	0	0	0	0	0	0	0	0	0	0	0	0	0	0	0	0	0	0	0	0	0	0	0
ENSG00000207383	0.957319	1.28245489	0.93624079	0	0.17800431	1.19684799	0.76879879	0	0	0.28902184	0.40201728	0.26948506	0.54018955	0.34570041	0.55505136	0.30783447	0.96010355	0	0.35118197	0.85002642	0.26862108	0.54508372	0.64650661	0.29667748	0.60924841	1.05045832	0.27456562	0.24061377	0	1.0267701	2.42418723	0.78083786	2.09401363	1.50668392	1.24296338	1.98700271	0.29529564	0	0.6428483	0.51257856	0.59612436	0	0.36535946	0.81731711	2.0604698	0	0	0.2983376
ENSG00000207384	0	0	0	0	0	0	0	0	0	0	0	0	0	0	0	0	0	0	0	0	0	0	0	0	0	0	0	0	0	0	0	0	0	0	0	0	0	0	0	0	0	0	0	0	0	0	0	0
ENSG00000207385	0	0	0	0	0	0	0	0	0	0	0	0	0	0	0	0	0.45313298	0.36333549	0	0	0	0	0	0	0	0	0	0	0	0	0	0	0	0	0	0	0	0	0	0	0	0	0	0	0	0	0	0
ENSG00000207386	0.3069499	0	0	0	0	0	0	0	0	0	0	0	0	0	0	0	0	0	0	0	0	0	0	0	0.58603895	0.25261021	0.26410598	0.23144753	0.22042065	0	0	0.37554583	0.50356042	0.48309548	0	0	0	0	0	0	0	0	0	0	0	0	0	0
ENSG00000207387	0	0	0	0	0	0	0	0	0	0	0	0	0	0	0	0	0	0	0	0	0	0	0	0	0	0	0	0	0	0	0	0	0	0	0	0	0	0	0	0	0	0	0	0	0	0	0	0
ENSG00000207389	0	0	0	0	0	0	0	0	0	0	0	0	0	0	0	0	0	0	0	0	0	0	0	0	0	0	0	0	0	0	0	0	0	0	0	0	0	0	0	0	0	0	0	0	0	0	0	0
ENSG00000207390	0	0	0.32163374	0	0	0	0	0	0	0	0	0	0	0.35628308	0	0	0.49474724	0	0	0	0	0	0	0	0	0	0	0	0	0	0	0	0	0	0	0	0	0	0	0	0	0	0	0	0	0	0	0
ENSG00000207391	0	0	0	0	0	0	0	0	0	0	0	0	0	0	0	0	0	0	0	0	0	0	0	0	0	0	0	0	0	0	0	0.38659129	0	0	0	0	0	0	0	0	0	0	0	0	0	0	0	0
ENSG00000207392	0.73249409	0.49063615	0.71636606	1.2624455	1.36200265	1.28207807	2.15690772	3.34825769	1.32784212	1.32687301	5.22927022	2.26816589	0.41332686	2.11610555	1.48644437	0.70662004	1.83656172	2.06165367	5.10544085	0.650399	2.46642989	1.2512149	0.98935102	1.81602576	8.39101224	7.8366578	9.66387789	7.73245147	3.33135753	5.10662556	6.67753392	4.48094454	2.40335655	2.68996347	1.42658297	5.21265646	3.38918854	7.38115706	1.9675054	2.74540186	2.28062727	1.74888491	3.63421951	3.12685713	2.92791867	1.34144433	2.82651293	0.91309386
ENSG00000207393	0	0	0	0	0	0	0	0	0	0	0	0	0	0	0	0	0	0	0	0	0	0	0	0	0	0	0	0	0	0	0	0	0	0	0	0	0	0	0	0	0	0	0	0	0	0	0	0
ENSG00000207394	0	0	0	0	0	0	0	0	0	0	0	0	0	0	0	0	0	0	0	0	0	0	0	0	0	0	0	0	0	0	0	0	0	0	0	0	0	0	0	0	0	0	0	0	0	0	0	0
ENSG00000207395	0	0	0	0	0	0	0	0	0	0	0	0	0	0	0	0	0	0	0	0	0	0	0	0	0	0	0	0	0	0	0	0	0	0	0	0	0	0	0	0	0	0	0	0	0	0	0	0
ENSG00000207397	0	0	0	0	0	0	0	0	0	0	0	0	0	0	0	0	0	0	0	0	0	0	0	0	0	0	0	0	0	0	0	0	0	0	0	0	0	0	0	0	0	0	0	0	0	0	0	0
ENSG00000207399	0	0	0	0	0	0	0	0	0	0	0	0	0	0	0	0	0	0	0	0	0.25355821	0	0	0	0	0	0	0	0	0	0	0	0	0	0	0	0	0	0	0	0	0	0	0	0	0	0	0
ENSG00000207401	0	0	0	0	0	0	0	0	0	0	0	0	0	0	0	0	0	0	0	0	0	0	0	0	0	0	0	0	0	0	0	0	0	0	0	0	0	0	0	0	0	0	0	0	0	0	0	0
ENSG00000207402	0	0	0	0	0	0	0	0	0	0	0	0	0	0	0	0	0	0	0	0	0	0	0	0	0	0	0	0	0	0	0	0	0	0	0	0	0	0	0	0	0	0	0	0	0	0	0	0
ENSG00000207403	0	0	0	0	0	0	0	0	0	0	0	0	0	0	0	0	0	0	0	0	0	0	0	0	0	0	0	0	0	0	0	0	0	0	0	0	0	0	0	0	0	0	0	0	0	0	0	0
ENSG00000207404	0	0	0	0	0	0	0	0	0.1448555	0	0	0	0	0	0	0	0	0	0	0.2601596	0	0	0	0.27240386	0	0	0.50420232	0	0	0	0	0	0	0	0	0.52126565	0	0.29524628	0	0	0.54735055	0.52466547	0.33546642	0	0	0	0	0
ENSG00000207405	0	0	0	0	0	0	0	0	0	0	0	0	0	0	0	0	0	0	0	0	0	0	0	0	0	0	0	0	0	0	0	0	0	0	0	0	0	0	0	0	0	0	0	0	0	0	0	0
ENSG00000207406	0	0	0	0	0	0	0	0	0	0	0	0	0	0	0	0	0	0	0	0	0	0	0	0	0	0	0	0	0	0	0	0	0	0	0	0	0	0	0	0	0	0	0	0	0	0	0	0
ENSG00000207407	0	0	0	0	0	0	0	0	0	0	0	0	0	0	0	0	0	0	0	0	0	0	0	0	0	0	0	0	0	0	0	0	0	0	0	0	0	0	0	0	0	0	0	0	0	0	0	0
ENSG00000207408	0	0	0	0	0	0	0	0	0	0	0	0	0	0	0	0	0	0	0	0	0	0	0	0	0	0	0	0	0	0	0	0	0	0	0	0	0	0	0	0	0	0	0	0	0	0	0	0
ENSG00000207410	0	0	0	0	0	0	0	0	0	0	0	0	0	0	0	0	0	0	0	0	0	0	0	0	0	0	0	0	0	0	0	0	0	0	0	0.20625619	0	0.23364814	0	0	0	0	0	0	0	0	0	0
ENSG00000207411	0	0	0	0	0	0	0	0	0	0	0	0	0	0	0	0	0	0	0	0	0	0	0	0	0	0	0	0	0	0	0	0	0	0	0	0	0	0	0	0	0	0	0	0	0	0	0	0
ENSG00000207412	0	0	0	0	0	0	0	0	0	0	0	0	0	0	0	0	0	0	0	0	0	0	0	0	0	0	0	0	0	0	0	0	0	0	0	0	0	0	0	0	0	0	0	0	0	0	0	0
ENSG00000207414	0	0	0	0	0	0	0	0	0	0	0	0	0	0	0	0	0	0	0	0	0	0	0	0	0	0	0	0	0	0	0	0	0	0	0	0	0	0	0	0	0	0	0	0	0	0	0	0
ENSG00000207415	0	0	0	0	0	0	0	0	0	0	0	0	0	0	0	0	0	0	0	0	0	0	0	0	0	0	0	0	0	0	0	0	0	0	0	0	0	0	0	0	0	0	0	0	0	0	0	0
ENSG00000207416	0	0	0.55787799	0	0.15910119	0.21394982	0.22905215	0	0.42302935	0.25832926	0.35932518	0	0.24141215	0	0	0	0	0.34404334	0.31388831	0	0	0	0	0	0	0	0	0	0	0	0	0	0	0	0	0	0.26393681	0	0.28729061	0	0	0.5107363	0	0.24350746	0	0.39174923	0	0
ENSG00000207417	0	0	0	0.3893524	0	0	0	0	0	0	0	0	0	0	0	0	0	0	0	0	0	0	0	0.56008271	0	0	0	0	0.21630064	0	0	0	0	0.47406566	0	0	0	0	0	0	0	0	0	0	0	0	0	0
ENSG00000207418	0	0	0	0	0	0	0	0	0	0	0	0	0	0	0	0	0	0	0	0	0	0	0	0	0	0	0	0	0	0	0	0	0	0	0	0	0	0	0	0	0	0	0	0	0	0	0	0
ENSG00000207419	0	0	0	0	0	0	0	0	0	0	0	0	0	0	0	0	0	0	0	0	0	0	0	0	0	0	0	0	0	0	0	0	0	0	0	0	0	0	0	0	0	0	0	0	0	0	0	0
ENSG00000207420	0	0	0	0	0	0	0	0	0	0	0	0	0	0	0	0	0	0	0	0	0	0	0	0	0	0	0	0	0	0	0	0	0	0	0	0	0	0	0	0	0	0	0	0	0	0	0	0

ENSG00000207421	[illegible]
ENSG00000207422	[illegible]
ENSG00000207425	[illegible]
ENSG00000207426	[illegible]
[illegible]	[illegible]

ENSG00000207699	[illegible]
ENSG00000207701	[illegible]
ENSG00000207702	[illegible]
ENSG00000207703	[illegible]
ENSG00000207704	[illegible]
ENSG00000207705	[illegible]
ENSG00000207706	[illegible]
ENSG00000207707	[illegible]
ENSG00000207708	[illegible]
ENSG00000207709	[illegible]
ENSG00000207710	[illegible]
ENSG00000207711	[illegible]
ENSG00000207712	[illegible]
ENSG00000207713	[illegible]
ENSG00000207714	[illegible]
ENSG00000207715	[illegible]
ENSG00000207716	[illegible]
ENSG00000207717	[illegible]
ENSG00000207719	[illegible]
ENSG00000207720	[illegible]
ENSG00000207721	[illegible]
ENSG00000207722	[illegible]
ENSG00000207725	[illegible]
ENSG00000207726	[illegible]
ENSG00000207728	[illegible]
ENSG00000207729	[illegible]
ENSG00000207730	[illegible]
ENSG00000207731	[illegible]
ENSG00000207732	[illegible]
ENSG00000207733	[illegible]
ENSG00000207734	[illegible]
ENSG00000207735	[illegible]
ENSG00000207736	[illegible]
ENSG00000207737	[illegible]
ENSG00000207738	[illegible]
ENSG00000207739	[illegible]
ENSG00000207741	[illegible]
ENSG00000207742	[illegible]
ENSG00000207743	[illegible]
ENSG00000207744	[illegible]
ENSG00000207746	[illegible]
ENSG00000207747	[illegible]
ENSG00000207749	[illegible]
ENSG00000207750	[illegible]
ENSG00000207751	[illegible]
ENSG00000207752	[illegible]
ENSG00000207753	[illegible]
ENSG00000207754	[illegible]
ENSG00000207755	[illegible]
ENSG00000207756	[illegible]
ENSG00000207757	[illegible]
ENSG00000207758	[illegible]
ENSG00000207759	[illegible]
ENSG00000207761	[illegible]
ENSG00000207762	[illegible]
ENSG00000207763	[illegible]
ENSG00000207764	[illegible]
ENSG00000207765	[illegible]
ENSG00000207766	[illegible]
ENSG00000207767	[illegible]
ENSG00000207768	[illegible]
ENSG00000207769	[illegible]
ENSG00000207770	[illegible]
ENSG00000207771	[illegible]
ENSG00000207773	[illegible]
ENSG00000207775	[illegible]
ENSG00000207776	[illegible]
ENSG00000207778	[illegible]
ENSG00000207779	[illegible]
ENSG00000207780	[illegible]
ENSG00000207781	[illegible]
ENSG00000207782	[illegible]
ENSG00000207783	[illegible]
ENSG00000207784	[illegible]
ENSG00000207785	[illegible]
ENSG00000207788	[illegible]
ENSG00000207789	[illegible]
ENSG00000207797	[illegible]
ENSG00000207798	[illegible]
ENSG00000207799	[illegible]
ENSG00000207800	[illegible]
ENSG00000207802	[illegible]
ENSG00000207803	[illegible]
ENSG00000207804	[illegible]
ENSG00000207805	[illegible]
ENSG00000207806	[illegible]
ENSG00000207807	[illegible]
ENSG00000207808	[illegible]
ENSG00000207810	[illegible]
ENSG00000207811	[illegible]
ENSG00000207812	[illegible]
ENSG00000207813	[illegible]
ENSG00000207814	[illegible]
ENSG00000207815	[illegible]
ENSG00000207816	[illegible]
ENSG00000207817	[illegible]
ENSG00000207818	[illegible]
ENSG00000207819	[illegible]
ENSG00000207820	[illegible]
ENSG00000207821	[illegible]
ENSG00000207825	[illegible]
ENSG00000207826	[illegible]
ENSG00000207827	[illegible]
ENSG00000207830	[illegible]
ENSG00000207832	[illegible]
ENSG00000207833	[illegible]
ENSG00000207834	[illegible]
ENSG00000207835	[illegible]
ENSG00000207836	[illegible]
ENSG00000207837	[illegible]
ENSG00000207838	[illegible]
ENSG00000207839	[illegible]
ENSG00000207844	[illegible]
ENSG00000207846	[illegible]
ENSG00000207849	[illegible]
ENSG00000207857	[illegible]
ENSG00000207858	[illegible]
ENSG00000207861	[illegible]
ENSG00000207862	[illegible]
ENSG00000207863	[illegible]
ENSG00000207864	[illegible]
ENSG00000207865	[illegible]
ENSG00000207866	[illegible]
ENSG00000207867	[illegible]
ENSG00000207868	[illegible]
ENSG00000207869	[illegible]
ENSG00000207870	[illegible]
ENSG00000207871	[illegible]
ENSG00000207873	[illegible]
ENSG00000207874	[illegible]
ENSG00000207875	[illegible]
ENSG00000207895	[illegible]
ENSG00000207922	[illegible]
ENSG00000207923	[illegible]
ENSG00000207924	[illegible]
ENSG00000207925	[illegible]
ENSG00000207926	[illegible]
ENSG00000207927	[illegible]
ENSG00000207928	[illegible]
ENSG00000207930	[illegible]
ENSG00000207931	[illegible]
ENSG00000207932	[illegible]
ENSG00000207933	[illegible]
ENSG00000207934	[illegible]
ENSG00000207935	[illegible]
ENSG00000207937	[illegible]
ENSG00000207938	[illegible]
ENSG00000207939	[illegible]
ENSG00000207940	[illegible]
ENSG00000207941	[illegible]
ENSG00000207942	[illegible]
ENSG00000207943	[illegible]
ENSG00000207944	[illegible]
ENSG00000207945	[illegible]
ENSG00000207946	[illegible]
ENSG00000207947	[illegible]
ENSG00000207948	[illegible]
ENSG00000207949	[illegible]
ENSG00000207951	[illegible]
ENSG00000207952	[illegible]
ENSG00000207953	[illegible]
ENSG00000207954	[illegible]
ENSG00000207955	[illegible]
ENSG00000207956	[illegible]
ENSG00000207957	[illegible]
ENSG00000207959	[illegible]
ENSG00000207960	[illegible]
ENSG00000207961	[illegible]
ENSG00000207962	[illegible]
ENSG00000207963	[illegible]
ENSG00000207964	[illegible]
ENSG00000207965	[illegible]
ENSG00000207966	[illegible]
ENSG00000207967	[illegible]
ENSG00000207969	[illegible]
ENSG00000207970	[illegible]
ENSG00000207971	[illegible]
ENSG00000207972	[illegible]
ENSG00000207973	[illegible]
ENSG00000207974	[illegible]
ENSG00000207975	[illegible]
ENSG00000207976	[illegible]
ENSG00000207977	[illegible]

ENSG00000207978	0	0.25699989	0	0	[illegible]
ENSG00000207979	0	0	0	0	[illegible]
ENSG00000207980	0.44150328	0.2957259	0	0	[illegible]
ENSG00000207981	0	0	0	0	[illegible]
ENSG00000207982	0	0	0	0	[illegible]
ENSG00000207983	0.33926042	1.13621003	0	0	[illegible]
ENSG00000207984	0	0	0	0	[illegible]
ENSG00000207986	7.28672377	5.44392806	4.38540614	9.0566744	[illegible]
ENSG00000207987	0	0	0	0	[illegible]
ENSG00000207988	0	0.22028562	0.32163374	0.4251092	[illegible]
ENSG00000207989	0	0	0.3541585	0	[illegible]
ENSG00000207990	0	0	0	0	[illegible]
ENSG00000207991	0	0	0	0.5273507	[illegible]
ENSG00000207992	0	0	0	0	[illegible]
ENSG00000207993	0.31291009	0	0.30602045	0	[illegible]
ENSG00000207994	0	0.26984988	0	0	[illegible]
ENSG00000207995	0	0	0	0	[illegible]
ENSG00000207996	1.12429325	2.76125461	6.23071875	4.3598409	[illegible]
ENSG00000207997	0	0	0	0	[illegible]
ENSG00000208000	0	0	0	0	[illegible]
ENSG00000208001	0	0	0	0	[illegible]
ENSG00000208002	0	0.2225566	0.32494955	0	[illegible]
ENSG00000208003	0	0	0	0.4339656	[illegible]
ENSG00000208004	0	0.26326818	0	0	[illegible]
ENSG00000208005	0	0.60811241	0	2.3470818	[illegible]
ENSG00000208006	0	0.24256169	0	0.4680978	[illegible]
ENSG00000208008	0.37476442	0	0	0	[illegible]
ENSG00000208009	0	0	0.3541585	0	[illegible]
ENSG00000208012	0	0	0	0	[illegible]
ENSG00000208013	0	0	0	0	[illegible]
ENSG00000208014	0	0	0	0	[illegible]
ENSG00000208015	0.49584215	0	0	0	[illegible]
ENSG00000208016	0	0	0	0	[illegible]
ENSG00000208017	0	0	0	0	[illegible]
ENSG00000208018	0	0.45931895	0	0	[illegible]
ENSG00000208022	0	0	0.32163374	0	[illegible]
ENSG00000208023	0	0	0	0	[illegible]
ENSG00000208024	0	0	0	0	[illegible]
ENSG00000208025	0	0	0.3317906	0.4385337	[illegible]
ENSG00000208027	0	0	0	0	[illegible]
ENSG00000208028	0	1.11278302	0.64989911	0.4294918	[illegible]
ENSG00000208032	0.33226536	1.11278302	0	0	[illegible]
ENSG00000208033	0	0.22724201	0	0	[illegible]
ENSG00000208034	0	0	0	0	[illegible]
ENSG00000208035	0	0	0	0	[illegible]
ENSG00000208036	1.17913682	1.05307271	1.92195772	1.524172	[illegible]
ENSG00000208037	0	0	0	0	[illegible]
ENSG00000208038	0	0	0	0	[illegible]
ENSG00000208308	0	1.01193706	0	0	[illegible]
ENSG00000208317	1.48752645	1.32849173	2.9095483	3.8456033	[illegible]
ENSG00000208772	5.6460858	5.67275665	5.06162296	12.163709	[illegible]
ENSG00000208797	0	0	0	0	[illegible]
ENSG00000208839	0	0	0	0	[illegible]
ENSG00000208883	0	0	0	0	[illegible]
ENSG00000208892	24.4092882	26.826253	30.1295137	49.625248	[illegible]
ENSG00000209042	3.25918717	2.66817861	1.77079251	2.8085867	[illegible]
ENSG00000209082	2.14864932	6.90815698	4.62294897	3.8883322	[illegible]
ENSG00000209480	0	0	0	0	[illegible]
ENSG00000209482	1.69630209	1.36345204	1.32716238	0.8770674	[illegible]
ENSG00000209582	0	0	0	0	[illegible]
ENSG00000209645	0.37917341	0.76192908	0.37082478	0.4901259	[illegible]
ENSG00000209702	0.46042485	1.23359946	0.90057448	1.1903058	[illegible]
ENSG00000210049	1.36181999	2.73650585	3.55156131	6.454475	[illegible]
ENSG00000210077	3.73678142	6.25738857	4.5681314	4.2264481	[illegible]
ENSG00000210082	51178.0359	69477.0773	75108.4311	62170.915	[illegible]
ENSG00000210100	1.40129303	1.56434714	2.74087884	1.8113349	[illegible]
ENSG00000210107	0.44763527	0	0	0	[illegible]
ENSG00000210112	1.42190028	0.6349409	0.46353098	6.1265739	[illegible]
ENSG00000210117	0	0	0	0	[illegible]
ENSG00000210127	0.93419536	1.25147771	4.11131826	1.8113349	[illegible]
ENSG00000210135	0.44150328	0.88717769	0	0.5706946	[illegible]
ENSG00000210140	1.95331756	1.30836306	1.91030949	3.1561138	[illegible]
ENSG00000210144	0	0	0	0	[illegible]
ENSG00000210151	0	0	0	0.6037783	[illegible]
ENSG00000210154	0	0	0.92706196	0	[illegible]
ENSG00000210156	0.46042485	0.61679973	0.45028724	1.7854587	[illegible]
ENSG00000210164	0	0	0	0	[illegible]
ENSG00000210174	0	0	0	0	[illegible]
ENSG00000210176	0	0.62573886	0.91362628	0.6037783	[illegible]
ENSG00000210181	0	0	0.25015958	0.3306405	[illegible]
ENSG00000210184	2.73133388	2.92718516	4.80815186	2.8244544	[illegible]
ENSG00000210191	0	0.30405621	0.44394516	0	[illegible]
ENSG00000210194	0.46709768	0	0	0.6037783	[illegible]
ENSG00000210195	1.46498817	2.28963536	3.82061899	1.2624455	[illegible]
ENSG00000210196	1.42190028	0.6349409	1.39059294	0	[illegible]
ENSG00000210678	0	0	0	0	[illegible]
ENSG00000210709	0	0	0	0	[illegible]
ENSG00000210741	0	0	0	0	[illegible]
ENSG00000210825	0	0	0	0	[illegible]
ENSG00000210839	0	0	0	0	[illegible]
ENSG00000210841	0	0.17133326	0	0	[illegible]
ENSG00000211137	0	0	0	0	[illegible]
ENSG00000211445	0.42116951	0.74998915	1.39642863	0.4182668	[illegible]
ENSG00000211448	0.00507955	0.00453648	0.00496771	0.0043773	[illegible]
ENSG00000211450	8.11158202	7.01504398	8.03547483	7.6313231	[illegible]
ENSG00000211451	0.65352632	0.62743061	0.62315858	0.7391642	[illegible]
ENSG00000211452	0	0.00197367	0	0	[illegible]
ENSG00000211454	0.03787278	0.07972734	0.04233018	0.048955	[illegible]
ENSG00000211455	4.78546896	4.83151418	4.43198025	3.4321453	[illegible]
ENSG00000211456	1.8904084	2.13731052	2.0419615	2.4829861	[illegible]
ENSG00000211459	21533.9256	36097.5415	34407.5334	33044.011	[illegible]
ENSG00000211460	8.48321787	7.31052801	7.6876858	6.8875393	[illegible]
ENSG00000211482	0	0	0	0	[illegible]
ENSG00000211483	0	0	0	0	[illegible]
ENSG00000211491	0	0.89949961	0	0	[illegible]
ENSG00000211495	0	0	0	0	[illegible]
ENSG00000211498	0	0	0	0	[illegible]
ENSG00000211499	0	0	0	0	[illegible]
ENSG00000211508	0	0	0	0	[illegible]
ENSG00000211510	0	0	0	0	[illegible]
ENSG00000211513	0	0	0.59471899	0.786051	[illegible]
ENSG00000211514	0	0.56316497	0.54817577	0.724534	[illegible]
ENSG00000211515	0	0	0	0	[illegible]
ENSG00000211517	0	0	0	0	[illegible]
ENSG00000211518	0	0	0.29458044	0	[illegible]
ENSG00000211519	0	0	0	0	[illegible]
ENSG00000211520	0	0	0	0	[illegible]
ENSG00000211524	0	0	0	0	[illegible]
ENSG00000211525	0	0	0	0	[illegible]
ENSG00000211526	0	0	0	0	[illegible]
ENSG00000211528	0	0	0	0	[illegible]
ENSG00000211530	0	0	0	0	[illegible]
ENSG00000211532	0	0	0	0	[illegible]
ENSG00000211534	0	0	0	0	[illegible]
ENSG00000211535	0	0	0	0	[illegible]
ENSG00000211538	0	0	0	0	[illegible]
ENSG00000211542	0	0	0	0	[illegible]
ENSG00000211543	0	0	0	0	[illegible]
ENSG00000211544	0.38831012	0	0	0	[illegible]
ENSG00000211553	2.38738813	3.59799843	3.21038123	3.0859779	[illegible]
ENSG00000211554	0	0	0	0	[illegible]
ENSG00000211556	0	0	0	0	[illegible]
ENSG00000211562	0	0	0	0	[illegible]
ENSG00000211563	0	0	0.39898869	0	[illegible]
ENSG00000211566	0	0	0	0	[illegible]
ENSG00000211568	0	0	0	0	[illegible]
ENSG00000211571	0	0	0	0.2873152	[illegible]
ENSG00000211573	0	0	0	0	[illegible]
ENSG00000211574	0.3288749	0.22028562	0	0	[illegible]
ENSG00000211575	0	0	0	0	[illegible]
ENSG00000211577	0	0.35979984	0.26266756	0	[illegible]
ENSG00000211578	0	0	0	0.3753216	[illegible]
ENSG00000211579	0	0	0	0	[illegible]
ENSG00000211580	0	0.18294907	0	0	[illegible]
ENSG00000211581	0.28271702	0.94684169	0.82947649	0.7308895	[illegible]
ENSG00000211582	0	0.49063615	0	0	[illegible]
ENSG00000211583	0	0	0	0	[illegible]
ENSG00000211584	1.04338296	1.3182218	1.06263365	1.7114466	[illegible]
ENSG00000211588	0	0	0	0	[illegible]
ENSG00000211590	0	0	0	0	[illegible]
ENSG00000211591	3.10648094	1.30048136	1.13928096	0	[illegible]
ENSG00000211592	0	0	0	0	[illegible]
ENSG00000211593	0	0	0	0	[illegible]
ENSG00000211594	0	0	0	0	[illegible]
ENSG00000211595	0	0	0	0	[illegible]
ENSG00000211596	0	0	0	0	[illegible]
ENSG00000211597	0	0	0	0	[illegible]
ENSG00000211598	0	0	0	0	[illegible]
ENSG00000211599	0	0	0	0	[illegible]
ENSG00000211611	0	0	0	0	[illegible]
ENSG00000211619	0	0	0	0	[illegible]
ENSG00000211623	0	0	0	0	[illegible]
ENSG00000211625	0	0	0	0	[illegible]
ENSG00000211626	0	0	0	0	[illegible]
ENSG00000211630	0	0	0	0	[illegible]
ENSG00000211632	0	0	0	0	[illegible]
ENSG00000211633	0	0	0	0	[illegible]
ENSG00000211637	0	0	0	0	[illegible]
ENSG00000211638	0	0	0	0	[illegible]
ENSG00000211639	0	0	0	0	[illegible]
ENSG00000211640	0	0	0	0	[illegible]
ENSG00000211641	0	0	0	0	[illegible]
ENSG00000211642	0	0	0	0	[illegible]
ENSG00000211643	0.17007778	0.0569604	0.08316651	0	[illegible]
ENSG00000211644	0	0	0	0	[illegible]
ENSG00000211645	0	0	0	0	[illegible]
ENSG00000211647	0	0	0	0	[illegible]
ENSG00000211648	0	0	0	0	[illegible]
ENSG00000211649	0	0	0	0	[illegible]
ENSG00000211650	0	0	0	0	[illegible]
ENSG00000211651	0	0	0	0	[illegible]

ENSG00000211652	0	0	0	0	0	0	0	0	0	0	0	0	0	0	0	0	0	0	0	0	0	0	0	0	0	0	0	0	0	0	0	0	0	0	0	0	0	0	0	0	0	0	0	0	0	0	0	0
ENSG00000211653	0	0	0	0	0	0	0	0	0	0	0	0	0	0	0	0	0	0	0	0	0	0	0	0	0	0	0	0	0	0	0	0	0	0	0	0	0	0	0	0	0	0	0	0	0	0	0	0
ENSG00000211654	0	0	0	0	0	0	0	0	0	0	0	0	0	0	0	0	0	0	0	0	0	0	0	0	0	0	0	0	0	0	0	0	0	0	0	0	0	0	0	0	0	0	0	0	0	0	0	0
ENSG00000211655	0	0	0	0	0	0	0	0	0	0	0	0	0	0	0	0	0	0	0	0	0	0	0	0	0	0	0	0	0	0	0	0	0	0	0	0	0	0	0	0	0	0	0	0	0	0	0	0
ENSG00000211656	0	0	0	0	0	0	0	0	0	0	0	0	0	0	0	0	0	0	0	0	0	0	0	0	0	0	0	0	0	0	0	0	0	0	0	0	0	0	0	0	0	0	0	0	0	0	0	0
ENSG00000211657	0	0	0	0	0	0	0	0	0	0	0	0	0	0	0	0	0	0	0	0	0	0	0	0	0	0	0	0	0	0	0	0	0	0	0	0	0	0	0	0	0	0	0	0	0	0	0	0
ENSG00000211658	0	0	0	0	0	0	0	0	0	0	0	0	0	0	0	0	0	0	0	0	0	0	0	0	0	0	0	0	0	0	0	0	0	0	0	0	0	0	0	0	0	0	0	0	0	0	0	0
ENSG00000211659	0	0	0	0	0	0	0	0	0	0	0	0	0	0	0	0	0	0	0	0	0	0	0	0	0	0	0	0	0	0	0	0	0	0	0	0	0	0	0	0	0	0	0	0	0	0	0	0
ENSG00000211660	0	0	0	0	0	0	0	0	0	0	0	0	0	0	0	0	0	0	0	0	0	0	0	0	0	0	0	0	0	0	0	0	0	0	0	0	0	0	0	0	0	0	0	0	0	0	0	0
ENSG00000211661	0	0	0	0	0	0	0	0	0	0	0	0	0	0	0	0	0	0	0	0	0	0	0	0	0	0	0	0	0	0	0	0	0	0	0	0	0	0	0	0	0	0	0	0.06727712	0	0	0	0
ENSG00000211662	0	0	0	0	0	0	0	0	0	0	0	0	0	0	0	0	0	0	0	0	0	0	0	0	0	0	0	0	0	0	0	0	0	0	0	0	0	0	0	0	0	0	0	0	0	0	0	0
ENSG00000211663	0	0	0	0	0	0	0	0	0	0	0	0	0	0	0	0	0	0	0	0	0	0	0	0	0	0	0	0	0	0	0	0	0	0	0	0	0	0	0	0	0	0	0	0	0	0	0	0
ENSG00000211664	0	0	0	0	0	0	0	0	0	0	0	0	0	0	0	0	0	0	0	0	0	0	0	0	0	0	0	0	0	0	0	0	0	0	0	0	0	0	0	0	0	0	0	0	0	0	0	0
ENSG00000211665	0	0	0	0	0	0	0	0	0	0	0	0	0	0	0	0	0	0	0	0	0	0	0	0	0	0	0	0	0	0	0	0	0	0	0	0	0	0	0	0	0	0	0	0	0	0	0	0
ENSG00000211666	0	0	0	0	0	0	0	0	0	0	0	0	0	0	0	0	0	0	0	0	0	0	0	0	0	0	0	0	0	0	0	0	0	0	0	0	0	0	0	0	0	0	0	0	0	0	0	0
ENSG00000211667	0	0	0	0	0	0	0	0	0	0	0	0	0	0	0	0	0	0	0	0	0	0	0	0	0	0	0	0	0	0	0	0	0	0	0	0	0	0	0	0	0	0	0	0	0	0	0	0
ENSG00000211668	0	0	0	0	0	0	0	0	0	0	0	0	0	0	0	0	0	0	0	0	0	0	0	0	0	0	0	0	0	0	0	0	0	0	0	0	0	0	0	0	0	0	0	0	0	0	0	0
ENSG00000211669	0	0	0	0	0	0	0	0	0	0	0	0	0	0	0	0	0	0	0	0	0	0	0	0	0	0	0	0	0	0	0	0	0	0	0	0	0	0	0	0	0	0	0	0	0	0	0	0
ENSG00000211670	0	0	0	0	0	0	0	0	0	0	0	0	0	0	0	0	0	0	0	0	0	0	0	0	0	0	0	0	0	0	0	0	0	0	0	0	0	0	0	0	0	0	0	0	0	0	0	0
ENSG00000211671	0	0	0	0	0	0	0	0	0	0	0	0	0	0	0	0	0	0	0	0	0	0	0	0	0	0	0	0	0	0	0	0	0	0	0	0	0	0	0	0	0	0	0	0	0	0	0	0
ENSG00000211672	0	0	0	0	0	0	0	0	0	0	0	0	0	0	0	0	0	0	0	0	0	0	0	0	0	0	0	0	0	0	0	0	0	0	0	0	0	0	0	0	0	0	0	0	0	0	0	0
ENSG00000211673	0	0	0	0	0	0	0	0	0	0	0	0	0	0	0	0	0	0	0	0	0	0	0	0	0	0	0	0	0	0	0	0	0	0	0	0	0	0	0	0	0	0	0	0	0	0	0	0
ENSG00000211674	0	0	0	0	0	0	0	0	0	0	0	0	0	0	0	0	0	0	0	0	0	0	0	0	0	0	0	0	0	0	0	0	0	0	0	0	0	0	0	0	0	0	0	0	0	0	0	0
ENSG00000211675	0	0	0	0	0	0	0	0	0	0	0	0	0	0	0	0	0	0	0	0	0	0	0	0	0	0	0	0	0	0	0	0	0	0	0	0	0	0	0	0	0	0	0	0	0	0	0	0
ENSG00000211676	0	0	0	0	0	0	0	0	0	0	0	0	0	0	0	0	0	0	0	0	0	0	0	0	0	0	0	0	0	0	0	0	0	0	0	0	0	0	0	0	0	0	0	0	0	0	0	0
ENSG00000211677	0	0	0	0	0	0	0	0	0	0	0	0	0	0	0	0	0	0	0	0	0	0	0	0	0	0	0	0	0	0	0	0	0	0	0	0	0	0	0	0	0	0	0	0	0	0	0	0
ENSG00000211678	0	0	0	0	0	0	0	0	0	0	0	0	0	0	0	0	0	0	0	0	0	0	0	0	0	0	0	0	0	0	0	0	0	0	0	0	0	0	0	0	0	0	0	0	0	0	0	0
ENSG00000211679	0	0	0	0	0	0	0	0	0	0	0	0	0	0	0	0	0	0	0	0	0	0	0	0	0	0	0	0	0	0	0	0	0	0	0	0	0	0	0	0	0	0	0	0	0	0	0	0
ENSG00000211680	0	0	0	0	0	0	0	0	0	0	0	0	0	0	0	0	0	0	0	0	0	0	0	0	0	0	0	0	0	0	0	0	0	0	0	0	0	0	0	0	0	0	0	0	0	0	0	0
ENSG00000211681	0	0	0	0	0	0	0	0	0	0	0	0	0	0	0	0	0	0	0	0	0	0	0	0	0	0	0	0	0	0	0	0	0	0	0	0	0	0	0	0	0	0	0	0	0	0	0	0
ENSG00000211682	0	0	0	0	0	0	0	0	0	0	0	0	0	0	0	0	0	0	0	0	0	0	0	0	0	0	0	0	0	0	0	0	0	0	0	0	0	0	0	0	0	0	0	0	0	0	0	0
ENSG00000211683	0	0.03972031	0	0.0383263	0	0	0.04762262	0.23234066	0.02931758	0.16112901	0.03735395	0.12519775	0.0250962	0.06424239	0.02578665	0.05720567	0.22302313	0	0	0.0789813	0.02495927	0.05064715	0	0.05513234	0.02830455	0	0.07653485	0.08942775	0.02129178	0.07155275	0.45049294	0.32648648	0.04864199	0.18666063	0.17323731	0.21099989	0.10975109	0	0.05973107	0	0	0.10618804	0	0.05062805	0	0.04072462	0.05280585	0.08316126
ENSG00000211684	0	0	0	0	0	0	0	0	0	0	0	0	0	0	0	0	0	0	0	0	0	0	0	0	0	0	0	0	0	0	0	0	0	0	0	0	0	0	0	0	0	0	0	0	0	0	0	0
ENSG00000211685	0	0	0	0	0	0	0	0	0	0	0	0	0	0	0	0	0	0	0	0	0	0	0	0	0	0	0	0	0	0	0	0	0	0	0	0	0	0	0	0	0	0	0	0	0	0	0	0
ENSG00000211687	0	0	0	0	0	0	0	0	0	0	0	0	0	0	0	0	0	0	0	0	0	0	0	0	0	0	0	0	0	0	0	0	0	0	0	0	0	0	0	0	0	0	0	0	0	0	0	0
ENSG00000211688	0	0	0	0	0	0	0	0	0	0	0	0	0	0	0	0	0	0	0	0	0	0	0	0	0	0	0	0	0	0	0	0	0	0	0	0	0	0	0	0	0	0	0	0	0	0	0	0
ENSG00000211689	0	0	0	0	0	0	0	0	0	0	0	0	0	0	0	0	0	0	0	0	0	0	0	0	0	0	0	0	0	0	0	0	0	0	0	0	0	0	0	0	0	0	0	0	0	0	0	0
ENSG00000211690	0	0	0	0	0	0	0	0	0	0	0	0	0	0	0	0	0	0	0	0	0	0	0	0	0	0	0	0	0	0	0	0	0	0	0	0	0	0	0	0	0	0	0	0	0	0	0	0
ENSG00000211691	0	0	0	0	0	0	0	0	0	0	0	0	0	0	0	0	0	0	0	0	0	0	0	0	0	0	0	0	0	0	0	0	0	0	0	0	0	0	0	0	0	0	0	0	0	0	0	0
ENSG00000211692	0	0	0	0	0	0	0	0	0	0	0	0	0	0	0	0	0	0	0	0	0	0	0	0	0	0	0	0	0	0	0	0	0	0	0	0	0	0	0	0	0	0	0	0	0	0	0	0
ENSG00000211693	0	0	0	0	0	0	0	0	0	0	0	0	0	0	0	0	0	0	0	0	0	0	0	0	0	0	0	0	0	0	0	0	0	0	0	0	0	0	0	0	0	0	0	0	0	0	0	0
ENSG00000211694	0	0	0	0	0	0	0	0	0	0	0	0	0	0	0	0	0	0	0	0	0	0	0	0	0	0	0	0	0	0	0	0	0	0	0	0	0	0	0	0	0	0	0	0	0	0	0	0
ENSG00000211695	0	0	0	0	0	0	0.04475428	0	0.00918392	0.06729961	0	0.0156876	0	0	0	0	0	0	0	0	0.03127462	0	0.01881763	0.01727056	0	0.01528765	0	0.04202073	0	0.01494291	0.02822397	0	0	0.02923633	0.03617847	0.01652427	0	0	0	0.02983887	0	0	0	0	0	0	0.01654177	0
ENSG00000211696	0	0	0	0	0	0	0.60835859	0.33727871	0.27237787	0.56136935	0.17352028	0.4071067	0	0	0.05989336	0.13286873	0	0	0	0	0.05797164	0.41172456	0.06976193	0.19207965	0.19722465	0.11335074	0	0.15578199	0.34617345	0.05539732	0.10463372	0.16851415	0.1129783	0	0	0.06125985	0.19118499	0.06939549	0.13873436	0.33186176	0.12865077	0.24663762	0.07884894	0.11759121	0.06352486	0.09458902	0	0.06438482
ENSG00000211697	0	0	0	0	0	0	0	0	0	0	0	0	0.04941952	0	0	0	0	0	0	0	0	0	0	0	0	0	0	0	0.04192784	0.04696729	0	0	0	0	0	0	0	0	0	0	0	0	0	0	0	0	0	0
ENSG00000211698	0	0	0	0	0	0	0	0	0	0	0	0	0	0	0	0	0	0	0	0	0	0	0	0	0	0	0	0	0	0	0	0	0	0	0	0	0	0	0	0	0	0	0	0	0	0	0	0
ENSG00000211699	0	0.0402011	0	0	0	0	0	0	0	0	0	0.05068527	0	0	0	0	0	0	0	0.05329154	0	0	0.06079811	0	0	0	0	0	0	0	0	0	0	0	0	0	0	0	0	0	0	0.10747337	0	0	0	0	0	0.05611191
ENSG00000211701	0	0	0	0	0	0	0	0	0.03426689	0.06277679	0.08731988	0	0	0	0	0	0	0	0.07627823	0	0	0	0	0	0.06616569	0	0	0	0	0	0	0	0	0	0	0	0	0	0	0	0	0	0	0	0	0	0	0
ENSG00000211706	0	0	0	0	0	0	0	0	0	0	0	0	0	0	0	0	0	0	0	0	0	0	0	0	0	0	0	0	0	0	0	0	0	0	0	0	0	0	0	0	0	0	0	0	0	0	0	0
ENSG00000211707	0.0848151	0.113621	0.1658953	0	0.04731167	0	0	0	0	0	0	0	0	0	0	0	0	0	0	0	0	0	0	0	0	0	0	0	0	0	0	0	0	0	0	0	0	0	0	0	0	0	0	0	0	0	0	0
ENSG00000211710	0	0	0	0	0	0	0	0	0	0	0	0	0	0	0	0	0	0	0	0	0	0	0	0	0	0	0	0	0	0	0	0	0	0	0	0	0	0	0	0	0	0	0	0	0	0	0	0
ENSG00000211713	0	0	0	0	0	0	0	0	0	0	0	0	0	0	0	0	0	0	0	0	0	0	0	0	0	0	0	0	0	0	0	0	0	0	0	0	0	0	0.08894203	0	0	0	0	0	0	0	0	0
ENSG00000211714	0	0	0	0	0	0.06089756	0	0	0.04013629	0	0	0.13711834	0.06871429	0	0	0.15663114	0	0	0	0	0	0.20801054	0	0.07547714	0.23249656	0.4008676	0	0.06121408	0	0.1306093	0	0.09932572	0	0	0	0	0.15025118	0	0	0	0	0.43611992	0.09295039	0.06931069	0.07488573	0	0.07229213	0.07589949
ENSG00000211715	0	0	0	0	0	0	0	0	0	0	0	0	0	0	0	0.09064514	0	0	0	0	0	0.08025285	0	0	0	0.07732966	0	0	0	0	0	0	0	0	0	0	0	0	0	0	0	0	0	0	0	0	0	0
ENSG00000211716	0	0	0	0	0.04609855	0.06199059	0	0	0	0	0	0	0	0	0	0	0	0	0	0	0	0	0	0	0	0	0	0	0	0	0	0	0	0	0	0	0	0	0	0	0	0	0	0	0	0	0	0
ENSG00000211717	0	0	0	0	0	0	0	0	0	0	0	0	0	0	0	0	0	0	0	0	0	0	0	0	0	0	0	0	0	0	0	0	0	0	0	0	0	0	0	0	0	0	0	0	0	0	0	0
ENSG00000211720	0	0	0	0	0	0	0	0	0	0	0	0	0	0	0	0	0	0	0	0	0	0	0	0	0	0	0	0	0	0	0	0	0	0	0	0	0	0	0	0	0	0	0	0	0	0	0	0
ENSG00000211721	0	0	0	0	0	0	0	0	0	0	0	0	0	0	0	0	0	0	0	0	0	0	0	0	0	0	0	0	0	0	0	0	0	0	0	0	0	0	0	0	0	0	0	0	0	0	0	0
ENSG00000211724	0	0	0	0	0	0	0	0	0	0	0	0	0	0	0	0	0	0	0	0	0	0	0	0	0	0	0	0	0	0	0	0	0	0	0	0	0	0	0	0	0	0	0	0	0	0	0	0
ENSG00000211725	0	0	0	0	0	0	0	0	0	0	0	0	0	0	0	0	0	0	0	0	0	0	0	0	0	0	0	0	0	0	0	0	0	0	0	0	0	0	0	0	0	0	0	0	0	0	0	0
ENSG00000211727	0	0	0	0	0	0	0	0	0	0	0	0	0	0	0	0	0	0	0	0	0	0	0	0	0	0	0	0	0	0	0	0	0	0	0	0	0	0	0	0	0	0	0	0	0	0	0	0
ENSG00000211728	0	0	0	0	0	0	0	0	0	0	0	0	0	0	0	0	0	0	0	0	0	0	0	0	0	0	0	0	0	0	0	0	0	0	0	0	0	0	0	0	0	0	0	0	0	0	0	0
ENSG00000211731	0	0	0	0	0	0	0	0	0	0	0	0	0	0	0	0	0	0	0	0	0	0	0	0	0	0	0	0	0	0	0	0	0	0	0	0	0	0	0	0	0	0	0	0	0	0	0	0
ENSG00000211732	0	0	0	0	0	0	0	0	0	0	0	0	0	0	0	0	0	0	0	0	0	0	0	0	0	0	0	0	0	0	0	0	0	0	0	0	0	0	0	0	0	0	0	0	0	0	0	0
ENSG00000211734	0	0	0	0	0	0	0	0	0	0	0	0	0	0	0	0	0	0	0	0	0	0	0	0	0	0	0	0	0	0	0	0	0	0	0	0	0	0	0	0	0	0	0	0	0	0	0	0
ENSG00000211739	0	0	0	0	0.05226289	0	0	0	0	0	0	0	0	0	0	0	0	0	0	0	0	0	0	0	0	0	0	0	0	0	0	0	0	0	0	0	0	0	0	0	0	0	0	0.07998937	0	0	0	0
ENSG00000211745	0	0	0	0	0	0	0	0	0	0	0	0	0	0	0	0	0	0	0	0	0	0	0	0	0	0	0	0	0	0	0	0	0	0	0	0	0	0	0	0	0	0	0	0	0	0	0	0.06622439
ENSG00000211746	0	0	0	0	0	0	0	0	0	0	0	0	0	0	0	0	0	0	0	0	0	0	0	0	0	0	0	0	0	0	0	0	0	0	0	0	0	0	0	0	0	0	0	0	0	0	0	0
ENSG00000211747	0	0	0	0	0	0	0	0	0	0	0	0	0	0	0	0	0	0	0	0	0	0	0	0	0	0	0	0	0	0	0	0	0	0	0	0	0	0	0	0	0	0	0	0	0	0	0	0
ENSG00000211749	0	0	0	0	0	0	0	0	0	0	0	0	0	0	0	0	0	0	0	0	0	0	0	0	0	0	0	0	0	0	0	0	0	0	0	0	0	0	0	0	0	0	0	0	0	0	0	0
ENSG00000211750	0	0	0	0	0	0	0	0	0	0	0	0	0	0	0	0	0	0	0	0	0	0	0	0	0	0	0	0	0	0	0	0	0	0	0	0	0	0	0	0	0	0	0	0	0	0	0	0
ENSG00000211751	0	0	0	0	0	0	0	0	0	0	0	0	0	0	0	0	0	0	0	0	0	0	0	0	0	0	0	0	0	0	0	0	0	0	0	0	0	0	0	0	0	0	0	0	0	0	0	0
ENSG00000211752	0	0	0	0	0	0	0	0	0	0	0	0	0	0	0	0	0	0	0	0	0	0	0	0	0	0	0	0	0	0	0	0	0	0	0	0	0	0	0	0	0	0	0	0	0	0	0	0
ENSG00000211753	0	0	0	0	0	0	0	0	0	0	0	0	0	0	0	0	0	0	0	0	0	0	0	0	0	0	0	0	0	0	0	0	0	0	0	0	0	0	0	0	0	0	0	0	0	0	0	0
ENSG00000211764	0	0	0	0	0	0	0	0	0	0	0	0	0	0.69831483	2.2424075	0	0	0	0	0	0	0	0	0	0	0	0	0	0	0	0	0	0	0	0	0	0	0	0	0	0	0	0	0	0	0	0	0
ENSG00000211765	0	0	0	0	0	0	0	0	0.31243344	0	0	0	0.53489358	0	0	0	0	0	0	0	0	0	0	0	0	0	0	0	0	0	0	0	0	0	0	0	0	0	0	0	0	0	0	0	0	0	0	0
ENSG00000211766	0	0	0	0	0	0	0	0	0	0	0	0	0.59303418	0	0.60934986	0	0	0	0	0	0	0	0	0	0	0	0	0	0	0	0	0	0	0	0	0	0	0	0	0	0	0	0	0	0	0	0	0
ENSG00000211767	0	0	0	0	0	0	0	0	0.32518583	0	0	0	0.55672597	0.71256615	1.14408546	0	0	0	0	0	0	0	0	0	0	0	0	0	0	0	0	0	0	0	0	0	0	0	0	0	0	0	0	0	0	0	0	0
ENSG00000211768	0	0	0	0	0	0	0	0	0	0	0	0	0	0	1.12120375	0	0	0	0	0	0	0	0	0	0	0	0	0	0	0	0	0	0	0	0	0	0	0	0	0	0	0	0	0	0	0	0	0
ENSG00000211769	0	0	0	0	0	0	0	0	0	0	0	0	0	0	0.58396029	0	0	0	0	0	0	0	0	0	0	0	0	0	0	0	0	0	0	0	0	0	0	0	0	0	0	0	0	0	0	0	0	0
ENSG00000211770	0	0	0	0	0	0	0	0	0	0	0	0	0.51470891	0.65878758	0	0	0	0	0	0	0	0	0	0	0	0	0	0	0	0	0	0	0	0	0	0	0	0	0	0	0	0	0	0	0	0	0	0
ENSG00000211771	0	0	0	0	0	0	0	0	0	0	0	0	0	0	0	0	0	0	0	0	0	0	0	0	0	0	0	0	0	0	0	0	0	0	0	0	0	0	0	0	0	0	0	0	0	0	0	0
ENSG00000211772	0	0	0	0	0.02371825	0	0.10243889	0.04164814	1.42944482	0.38510826	0.10713389	0.1795382	6.44200986	3.0862199	5.4728943	0.28712266	0.31982341	0.25644392	0	0	0	0	0	0	0.04058977	0	0	0	0	0	0	0	0	0	0	0	0.11804034	0.17138307	0.04282828	0.54638982	0.71487736	0.07613879	1.31442646	0.5808199	0.54909617	0	0.07572553	0.07950421
ENSG00000211776	0	0	0	0	0	0	0	0	0	0	0	0	0	0	0	0	0	0	0	0	0	0	0	0	0	0	0	0	0	0	0	0	0	0	0	0	0	0	0	0	0	0	0	0	0	0	0	0
ENSG00000211777	0	0	0	0	0	0	0	0	0	0	0	0	0	0	0	0	0	0	0	0	0	0	0	0	0	0	0	0	0	0	0	0	0	0	0	0	0	0	0	0	0	0	0	0	0	0	0	0
ENSG00000211778	0	0	0	0	0	0	0	0	0	0	0	0	0	0	0	0	0	0	0	0	0	0	0	0	0	0	0	0	0	0	0	0	0	0	0	0	0	0	0	0	0	0	0	0	0	0	0	0
ENSG00000211779	0	0	0	0	0	0	0	0	0	0	0	0	0	0	0	0	0	0	0	0	0.07707593	0	0	0	0	0	0	0	0	0	0	0	0	0	0	0	0	0	0	0	0	0	0	0	0	0	0	0
ENSG00000211780	0	0	0	0	0	0	0	0	0	0	0	0	0	0	0	0	0	0	0	0	0	0	0	0	0	0	0	0	0	0	0	0	0	0	0	0	0	0	0	0	0	0	0	0	0	0	0	0
ENSG00000211781	0	0	0	0	0	0	0	0	0	0	0	0	0	0	0	0	0	0	0	0	0	0	0	0	0	0	0	0	0	0	0	0	0	0	0	0	0	0	0	0	0	0	0	0	0	0	0	0
ENSG00000211782	0	0	0	0	0	0	0	0	0	0	0	0	0	0	0	0	0	0	0	0	0	0	0	0	0	0	0	0	0	0	0	0	0	0	0	0	0	0	0	0	0	0	0	0	0	0	0	0
ENSG00000211783	0	0	0	0	0	0	0	0	0	0	0	0	0	0	0	0	0	0	0	0	0	0	0	0	0	0	0	0	0	0	0	0	0	0	0	0	0	0	0	0	0	0	0	0	0	0	0	0
ENSG00000211784	0	0	0	0	0	0	0	0	0	0	0	0	0	0	0	0	0	0	0	0	0	0	0	0	0	0	0	0	0	0	0	0	0	0	0	0	0	0	0	0	0	0	0	0	0	0	0	0
ENSG00000211785	0	0	0	0	0	0	0	0	0	0	0	0	0	0	0	0	0	0	0	0	0	0	0	0	0	0	0	0	0	0	0	0	0	0	0	0	0	0	0	0	0	0	0	0	0	0	0	0
ENSG00000211786	0	0	0	0	0	0	0	0	0	0	0	0	0	0	0	0	0	0	0	0	0	0	0	0	0	0	0	0	0	0	0	0	0	0	0	0	0	0	0	0	0	0	0	0	0	0	0	0
ENSG00000211787	0	0	0	0	0	0	0	0	0	0	0	0	0	0	0	0	0	0	0	0	0	0	0	0	0	0	0	0	0	0	0	0	0	0	0	0	0	0	0	0	0	0	0	0	0	0	0	0
ENSG00000211788	0	0	0	0	0	0	0	0	0	0	0	0	0	0	0	0	0	0	0	0	0	0	0	0	0	0	0	0.06750553	0	0	0	0	0	0	0	0	0	0	0	0	0	0	0	0	0	0	0	0
ENSG00000211789	0	0	0	0	0	0	0	0	0	0	0	0	0	0	0	0	0	0	0	0	0	0	0	0	0	0	0	0	0	0	0	0	0	0	0	0	0	0	0	0	0	0	0	0	0	0	0	0
ENSG00000211790	0	0	0	0	0	0	0	0	0	0	0	0	0	0	0	0	0	0	0	0	0	0	0	0	0	0	0	0	0	0	0	0	0	0	0	0	0	0	0	0	0	0	0	0	0	0	0	0
ENSG00000211791	0	0	0	0	0	0	0	0	0	0	0	0	0	0	0	0	0	0	0	0	0	0	0	0	0	0	0	0	0.05644919	0	0	0	0	0	0	0	0	0	0	0	0	0	0	0	0	0	0	0
ENSG00000211792	0	0	0	0	0	0	0	0	0	0	0	0	0	0	0	0	0	0	0	0	0	0	0	0	0	0	0	0	0	0	0	0	0	0	0	0	0	0	0	0	0	0	0	0	0	0	0	0
ENSG00000211793	0	0	0	0	0	0	0	0	0	0	0	0	0	0	0	0	0	0	0	0	0	0	0	0	0	0	0	0	0	0	0	0	0	0	0	0	0	0	0	0	0	0	0	0	0	0	0	0
ENSG00000211794	0	0	0	0	0	0	0	0	0	0	0	0	0	0	0	0	0	0	0	0	0	0	0	0	0	0	0.06950157	0	0	0	0	0	0	0	0	0	0	0	0	0	0	0	0	0	0	0	0	0
ENSG00000211795	0	0	0	0	0	0	0	0	0	0	0	0	0	0	0	0	0	0	0	0	0	0	0	0	0	0	0	0	0	0	0	0	0	0	0	0	0	0	0	0	0	0	0	0	0	0	0	0
ENSG00000211796	0	0	0	0	0	0	0	0	0	0	0	0	0	0	0	0	0	0	0	0	0	0	0	0	0	0	0	0	0	0	0	0	0	0	0	0	0	0	0	0	0	0	0	0	0	0	0	0
ENSG00000211797	0	0	0	0	0	0.04995109	0	0	0	0	0	0	0	0	0	0	0	0	0	0	0	0	0	0	0.06356827	0	0	0	0	0	0	0	0	0	0	0	0	0	0	0	0	0	0	0	0	0	0	0
ENSG00000211798	0	0	0	0	0	0	0	0	0	0	0	0	0	0	0	0	0	0	0	0	0	0.12512149	0.07420133	0.06810097	0	0.12056397	0	0.11046359	0	0	0	0	0	0	0	0	0	0	0	0	0	0	0	0	0	0	0	0
ENSG00000211799	0	0	0	0	0	0	0	0	0	0	0	0	0	0	0	0	0	0	0	0	0	0	0	0	0	0	0.05938143	0	0.04955925	0	0	0	0	0	0	0	0	0	0	0	0	0	0	0	0	0	0	0
ENSG00000211800	0	0	0	0	0	0	0	0	0	0	0	0	0	0	0	0	0	0	0	0	0	0.06779982	0	0	0	0.13066046	0	0	0	0	0	0	0	0	0	0	0	0	0	0	0	0	0	0	0	0	0	0
ENSG00000211801	0	0	0	0	0	0	0	0	0	0	0	0	0	0	0	0	0	0	0	0	0	0	0	0	0	0	0	0	0	0	0	0	0	0	0	0	0	0	0	0	0	0	0	0	0	0	0	0
ENSG00000211802	0	0	0	0	0	0	0	0	0	0	0	0	0	0	0	0	0	0	0	0	0	0.05932484	0.07036333	0.129157	0	0.05716395	0.05976536	0	0.14963902	0.05587488	0	0	0	0	0	0	0	0	0	0	0	0	0	0	0	0	0	0
ENSG00000211803	0	0	0	0	0	0	0	0	0	0	0	0	0	0	0	0	0	0	0	0	0	0	0	0	0	0	0	0	0	0	0	0	0	0	0	0	0	0	0	0	0	0	0	0	0	0	0	0
ENSG00000211804	0	0	0	0	0	0	0	0	0	0	0	0	0	0	0	0	0	0	0	0	0	0	0	0	0	0	0	0	0	0	0	0	0	0	0	0	0	0	0	0	0	0	0	0	0	0	0	0
ENSG00000211805	0	0	0	0	0	0	0	0	0	0	0	0	0	0	0	0	0	0	0	0	0	0	0	0	0	0	0	0	0	0	0	0	0	0	0	0	0	0	0	0	0	0	0	0	0	0	0	0
ENSG00000211806	0	0	0	0	0	0	0	0	0	0	0	0	0	0	0	0	0	0	0.08801335	0	0	0	0	0	0	0	0	0	0	0	0	0	0	0	0	0	0	0	0	0	0	0	0	0	0	0	0	0
ENSG00000211807	0	0	0	0	0	0	0	0	0	0	0	0	0	0	0	0	0	0	0	0	0	0	0	0	0	0	0	0	0	0	0	0	0	0	0	0	0	0	0	0	0	0	0	0	0	0	0	0
ENSG00000211808	0	0	0	0	0	0	0	0	0	0	0	0	0	0	0	0	0	0	0	0	0	0	0	0	0	0	0	0	0	0	0	0	0	0	0	0	0	0	0	0	0	0	0	0	0	0	0	0
ENSG00000211809	0	0	0	0	0	0	0	0	0	0	0	0	0	0	0	0	0	0	0	0	0	0	0	0	0	0	0	0.05718115	0	0	0	0	0	0	0	0	0	0	0	0	0	0	0	0	0	0	0	0
ENSG00000211810	0	0	0	0	0	0	0	0	0	0	0	0	0	0	0	0	0	0	0	0	0	0	0	0	0	0	0	0	0	0	0	0	0	0	0	0	0	0	0	0	0	0	0	0	0	0	0	0
ENSG00000211812	0	0	0	0	0	0	0	0	0	0	0	0	0	0	0	0	0	0	0	0	0	0	0	0	0	0	0	0	0	0	0	0	0	0	0	0	0	0	0	0	0	0	0	0	0	0	0	0
ENSG00000211813	0	0	0	0	0	0	0	0	0	0	0	0	0	0	0	0	0	0	0	0	0	0	0	0	0	0	0	0	0	0	0	0	0	0	0	0	0	0	0	0	0	0	0	0	0	0	0	0
ENSG00000211814	0	0	0	0	0	0	0	0	0	0	0	0	0	0	0	0	0	0	0	0	0	0	0	0	0	0	0	0	0	0	0	0	0	0	0	0	0	0	0	0	0	0	0	0	0	0	0	0
ENSG00000211815	0	0	0	0	0	0	0	0	0	0	0	0	0	0	0	0	0	0	0	0	0	0	0	0	0	0	0	0	0	0	0	0	0	0	0	0	0	0	0	0	0	0	0	0	0	0	0	0
ENSG00000211816	0	0	0	0	0	0	0	0	0	0	0	0	0	0	0	0	0	0	0	0	0	0	0	0	0	0	0	0	0	0	0	0	0	0	0	0	0	0	0	0	0	0	0	0	0	0	0	0
ENSG00000211817	0	0	0	0	0	0	0	0	0	0	0	0	0	0	0	0	0	0	0	0	0	0	0	0	0	0	0	0	0	0	0	0	0	0	0	0	0	0	0	0	0	0	0	0	0	0	0	0
ENSG00000211818	0	0	0	0	0	0	0	0	0.48139291	0.08819096	0	0.16445916	0	0	0	0	0.14648106	0	0.64294946	0.25937362	0.32786379	0.16632464	1.77545168	0	0	0.1602663	0	0.58735928	0.13984392	0.46995671	0	0	0	0	0	0	0	0.1962362	0	0.15640615	0.36379795	0.52308038	0	0	0	0	0	0
ENSG00000211819	0	0	0	0	0	0	0	0	0	0	0	0	0	0	0	0	0	0	0	0	0	0	0	0	0	0	0	0	0	0	0	0	0	0	0	0	0	0	0	0	0	0	0	0	0	0	0	0
ENSG00000211820	0	0	0	0	0	0	0	0	0	0	0	0.08100592	0	0	0	0	0	0	0	0.0851713	0	0	0	0	0	0	0	0.07232735	0	0	0	0	0	0	0	0	0	0	0	0	0	0	0	0	0	0	0	0
ENSG00000211821	0	0	0	0	0	0	0	0	0	0	0	0	0	0	0	0	0	0	0	0	0	0	0	0	0	0	0	0	0	0	0	0	0	0	0	0	0	0	0	0	0	0	0	0	0	0	0	0
ENSG00000211825	0	0	0	0	0	0	0	0.61900562	0	0	0	0	0	0	0	0	0	0	0.69547801	1.68338565	0	0	0.64016831	0	0	0.52007985	1.08749521	0.47650961	0.90761444	0	0	0	0	0	0	1.12429845	0	0	0	0	0	0	0	0	0	0	0	0
ENSG00000211826	0	0	0	0	0	0	0	0	0	0	0	0	0	0	0	0	0	0	0	0	0	0	0	0	0	0	0	0	0	0	0	0	0	0	0	0	0	0	0	0	0	0	0	0	0	0	0	0
ENSG00000211827	0	0	0	0	0	0	0	0	0	0	0	0	0	0	0	0	0	0	0	0	0	0	0	0	0	0	0	0	0	0	0	0	0	0	0	0	0	0	0	0	0	0	0	0	0	0	0	0
ENSG00000211828	0	0	0	0	0	0	0	0	0	0	0	0	0	0	0	0	0	0	0	0	0	0	0	0	0	0	0	0	0	0	0	0	0	0	0	0	0	0	0	0	0	0	0	0	0	0	0	0
ENSG00000211829	0	0	0	0	0	0	0.10784539	0.1315387	0.11065351	0.08108668	0	0.07560553	0.0378883	0.14548226	0.03893069	0.17272934	1.01010894	0.26997846	0.59115631	1.58986422	0.71594979	0.03823157	1.67777444	0.33293806	0.34185605	0.03683899	0.73179365	0.67505529	1.70366793	0.57613211	0	0	0	0	0	0.19909452	0	0.04510707	0.04508867	0.28761353	0	0	0.46126632	0.22930286	0.41291161	0.24593146	0.15944432	0.16740054
ENSG00000211831	0	0	0	0	0	0	0	0	0	0.4865201	0	0	0	0	0	0	0	0	0	0.47695927	0	0	0	0	0	0.44206788	0.92437093	0	0	0	0	0	0	0	0	0	0	0	0	0	0	0	0	0	0.49549393	0	0.47833296	0.50220162
ENSG00000211832	0	0	0	0	0	0	0	0	0	0	0	0	0	0.64658781	0	0.57576448	0	0	0	0	0	0	0.6046034	0	0	0	0	0	0	0	0	0	0	0	0	0	0	0	0	0	0	0	0	0	0	0	0	0
ENSG00000211833	0	0	0	0	0	0	0	0	0	0	0	0	0	0	0.44492212	0	0	0	0	0	0	0	0	0	0	0	0	0	0	0	0	0	0	0	0	0	0	0	0	0	0	0	0	0	0	0	0	0
ENSG00000211834	0	0	0	0	0	0	0	0	0	0	0	0	0	0	0	0	0	0	0	0	0	0	0	0	0	0	0	0	0	0	0	0	0	0	0	0	0	0	0	0	0	0	0	0	0	0	0	0
ENSG00000211835	0	0	0	0	0	0	0	0	0	0	0	0	0	0	0	0	0	0	0	0.92314697	0	0	0.52659006	0	0	0	0	0.39196759	0.37329303	0.41816041	0	0	0	0	0	0	0	0	0	0	0	0	0	0.44381198	0	0	0	0
ENSG00000211836	0	0	0	0	0.59928117	0	0	0	0	0	0	0	0	0	0	0	0	0	0	0	0	0	0	0	0	0	0	0	0	0	0	0	0	0	0	0	0	0	0	0	0	0	0	0	0	0	0	0
ENSG00000211837	0	0	0	0	0	0	0	0	0	0	0	0	0	0	0	0	0	0	0	0	0	0	0	0	0	0	0	0	0	0.39281735	0	0	0	0	0	0	0	0	0	0	0	0	0	0	0	0	0	0
ENSG00000211838	0	0	0	0	0	0	0	0	0	0	0	0	0	0	0	0	0	0	0	0	0	0	0	0	0	0	0	0	0	0	0	0	0	0	0	0	0	0	0	0	0	0	0	0	0	0	0.4159417	0
ENSG00000211839	0.53716233	0	0	0	0	0.40293882	0	0	0	0	0	0	0	0	0	0	0	0	0	0	0	0	0	0	0	0	0	0	0	0	0	0	0	0	0	0	0	0	0	0	0	0	0	0	0	0	0	0
ENSG00000211840	0	0	0	0	0	0	0	0	0	0	0	0	0	0	0	0	0	0	0	0	0	0	0	0	0	0	0	0	0	0	0	0	0	0	0	0	0	0	0	0	0	0	0	0	0	0	0	0
ENSG00000211841	0	0	0	0	0.28537198	0	0	0	0	0	0	0	0	0	0	0	0	0	0	0	0	0	0	0	0	0	0	0	0	0	0	0	0	0	0	0	0	0	0	0	0	0	0	0	0	0	0	0
ENSG00000211842	0	0	0	0	0	0	0	0	0	0	0	0	0	0	0	0	0	0	0	0	0	0	0	0	0	0	0	0	0	0	0	0	0	0	0	0	0	0	0	0	0	0	0	0	0	0	0	0
ENSG00000211843	0	0	0	0	0	0	0	0	0	0	0	0	0	0	0	0	0	0	0	0	0	0	0	0	0	0	0	0	0	0	0	0	0	0	0	0	0	0	0	0	0	0	0	0	0	0	0	0
ENSG00000211844	0	0	0	0	0	0	0	0	0	0	0	0	0	0	0	0	0	0	0	0	0	0	0	0	0	0	0	0	0	0	0	0	0	0	0	0	0	0	0	0	0	0	0	0	0	0	0	0
ENSG00000211845	0	0	0	0	0	0	0	0	0	0	0	0	0	0	0	0	0	0	0	0	0	0	0	0	0	0	0	0	0.35066921	0	0	0	0	0	0	0	0	0	0	0	0	0	0	0	0	0	0	0
ENSG00000211846	0	0	0	0	0	0	0	0	0	0	0	0	0	0	0	0	0	0	0	0	0	0	0	0	0	0	0	0	0	0	0	0	0	0	0	0	0	0	0	0	0	0	0	0	0	0	0	0
ENSG00000211847	0	0	0	0	0	0	0	0	0	0	0	0	0	0	0.42469839	0	0	0	0	0	0	0	0	0	0	0	0	0	0	0	0	0	0	0	0	0	0	0	0	0	0	0	0	0	0	0	0	0.45654693
ENSG00000211848	0	0	0	0	0	0	0	0	0	0	0	0	0	0	0	0	0	0	0	0	0	0	0	0	0	0	0	0	0	0	0	0	0	0	0	0	0	0	0	0	0	0	0	0	0	0	0	0
ENSG00000211849	0	0	0	0	0.29472844	0	0	0	0	0	0	0	0	0	0	0	0	0	0	0	0	0	0	0	0	0	0	0	0	0	0	0	0	0	0	0	0	0	0	0	0	0	0	0	0	0	0	0
ENSG00000211850	0	0	0	0	0	0	0	0	0	0	0	0	0	0	0	0	0	0	0	0	0	0	0	0	0	0	0	0	0	0	0	0	0	0	0	0	0	0	0	0	0	0	0	0	0	0	0	0
ENSG00000211851	0	0	0	0	0	0.3899408	0	0	0	0	0	0	0	0	0	0	0	0	0	0	0	0	0	0	0	0	0.44727626	0	0	0	0	0	0	0	0	0	0	0	0	0	0	0	0	0	0	0	0	0
ENSG00000211852	0	0	0	0	0	0	0	0	0	0	0	0	0	0	0	0	0	0	0	0	0	0	0	0	0	0.42101702	0	0	0	0	0	0	0	0	0	0	0	0	0	0	0	0	0	0	0	0	0	0
ENSG00000211853	0	0	0	0	0	0	0	0	0	0	0	0	0	0	0	0	0	0	0	0	0	0	0	0	0	0	0	0	0	0	0	0	0	0	0	0	0	0	0	0	0	0	0	0	0	0	0	0
ENSG00000211854	0	0	0	0	0.30471924	0	0	0	0	0	0	0	0	0	0	0	0	0	0.60117591	0	0	0	0	0	0	0	0	0	0	0	0	0	0	0	0	0	0	0	0	0	0	0	0	0	0	0	0	0
ENSG00000211855	0.55568517	0	0	0	0	0	0	0	0	0	0	0	0	0	0	0	0	0	0	0	0	0	0	0	0	0	0	0	1.19711214	0	0	0	0	0	0	0	0	0	0	0	0	0	0	0	0	0	0	0
ENSG00000211856	0	0	0	0	0	0	0	0	0	0	0	0	0	0	0	0	0	0	0	0	0	0	0	0	0	0	0	0.42635071	0	0	0	0	0	0	0	0	0	0	0	0	0	0	0	0	0	0	0	0
ENSG00000211857	0.48832939	0	0.47757737	0	0	0	0	0	0	0	0	0	0	0	0	0	0	0	0	0	0	0	0	0	0	0	0	0	0	0	0	0	0	0	0	0	0	0	0	0	0	0	0	0	0	0	0	0
ENSG00000211858	0	0	0	0	0	0	0	0	0	0	0	0	0	0	0	0	0	0	0	0	0	0	0	0	0	0	0	0	0	0	0	0	0	0	0	0	0	0	0	0	0	0	0	0	0	0	0	0
ENSG00000211859	0	0	0	0	0	0	0	0	0	0	0	0	0	0	0	0	0	0	0	0	0	0	0	0	0	0	0	0	0	0	0	0	0	0	0	0	0	0	0	0	0	0	0	0	0	0	0	0
ENSG00000211860	0	0	0	0	0	0	0	0	0	0	0	0	0	0	0	0	0	0	0	0	0	0	0	0	0	0	0	0	0	0	0	0	0	0	0	0	0	0	0	0	0	0	0	0	0	0	0	0
ENSG00000211861	0	0	0	0	0.27240053	0	0	0	0	0	0	0	0	0	0	0	0	0	0	0	0	0	0	0	0	0	0	0	0	0	0	0	0	0	0	0	0	0	0	0	0	0	0	0	0	0	0	0
ENSG00000211862	0	0	0	0	0.30471924	0	0	0	0	0	0	0	0	0	0	0	0	0	0	0	0	0	0	0	0	0	0	0	0	0	0	0	0	0	0	0	0	0	0	0	0	0	0	0	0	0	0	0
ENSG00000211863	0	0	0	0	0	0	0	0	0	0	0	0	0	0	0	0	0	0	0	0	0	0	0	0	0	0	0	0	0	0	0	0	0	0	0	0	0	0	0	0	0	0	0	0	0	0	0	0
ENSG00000211864	0	0	0	0	0	0	0	0	0	0	0	0	0	0	0	0	0	0	0	0	0	0	0	0	0	0	0	0	0	0	0	0	0	0	0	0	0	0	0	0	0	0	0	0	0	0	0	0
ENSG00000211865	0	0	0	0	0	0.38375126	0	0	0	0	0	0	0	0	0	0	0	0	0	0	0	0	0	0	0	0	0	0	0	0	0	0	0	0	0	0	0	0	0	0	0	0	0	0	0	0	0	0
ENSG00000211866	0	0	0	0	0	0	0.41083957	0	0	0	0	0.4320316	0	0	0	0	0	0.61709361	0	0	0	0	0	0	0.48836579	0.42101702	1.76070653	0.77149176	0	0	0	0	0	0	0	0	0.94682093	0	0.51529903	0	0	0	0	0	0	0	0	0
ENSG00000211867	0	0	0	0	0	0	0	0	0.25292231	0	0	0	0.43300909	0	0	0.49351241	0	0	0	0	0.43064649	0	0.51823149	0	0	0	2.20088316	0	0	0	0	0	0	0	0	0	0	0	0	0	0	0	0.58573501	0	0.94379796	0.70266132	0	0.47828726
ENSG00000211868	0	0	0	0	0.32688064	0	0	0	0	0	0	0	0	0	0.50963807	0	0	0.70685269	0	0	0	0	0	0	0.55940082	0	0.50420232	0	0.42080306	0.47138082	0	0	0	0	0	0	0	0	0	0	0	0	0	0	0	0	0.52181777	0
ENSG00000211869	0	0	0	0	0.31541114	0	0	0	0	0	0	0	0	0	0	0	0.85061806	0	0	0	0	0	0	0.52569167	0	0	0.97302203	0	0	0	0	0	0	0	0	0	0	0	0	0.90825325	0	0	0	0	1.04314511	0.77662567	0	0
ENSG00000211870	0	0	0	0	0	0	0	0	0	0	0	0.45363318	0.45465954	0.58192903	0	1.03637606	0.80808716	0	0	0	0.45217881	0	0.54414306	0	0	0	2.31092732	0	0.38573614	0	0	0	0	0	0	0	0	0	0	0	0	0	0	0	0	0	0.47833296	0
ENSG00000211871	0	0	0	0	0	0	0	0	0	0	0	0	0	0	0	0	0	0	1.07482965	0	0.8221433	0	0.49467551	0	0	0	2.10084302	0.36821197	0	0	0	0	0	0	0	0	0	0	0	0	0	0	0	0	0.45044903	0	0	0
ENSG00000211872	0	0	0	0	0	0	0	0	0	0	0	0	0	0	0	0	0	0	0	0.45424692	0	0	0	0.47562579	0	0.84203405	0	0	0	0	0	0	0	0	0	0	0	0	0	0	0	0	0	0	0.47189898	0.70266132	0	0
ENSG00000211873	0.53716233	0	0	0	0	0	0	0	0	0	0	0	0	0	0	0.51818803	0.80808716	0.6479483	0	0	0.90435762	0	0	0	0	0.44206788	0.46218546	0	0.38573614	0	0	0	0	0	0	0	0	0	0	0	0	0	0	0	0.49549393	0	0	0
ENSG00000211874	0	0	0	0	0	0	0	0	0	0	0	0	0.4399931	0	0	0	0.78201983	0	0	0	0	0	0	0	0	0	0.89455251	0	0	0	0	0	0	0	0	0	0	0	0	0	0	0	0	0.44381198	0	0	0	0
ENSG00000211875	0.61980269	0	0	0	0	0	0	0	0	0.56136935	0	0.5234229	0	0	0	0	0	0	0	0	0	0	0	0	0	0	0	0	0	0	0	0	0	0	0	0	0	0	0	0	0	0	0	0	0.57172376	0	0	0
ENSG00000211876	0	0	0	0	0	0	0	0	0	0	0	0	0	0	0	0	0	0	0	0	0	0	0.51823149	0	0	0	0	0	0	0	0	0	0	0	0	0	0	0	0	0	0	0	0	0	0	0	0	0
ENSG00000211877	0	0	0	0	0.29964058	0	0	0.52615478	0	0	0	0	0	0	0.46716823	0	0	0	0	0	0.45217881	0	0	0	0	0	0	0	0	0	0	0	0	0	0	0	0	0	0	0	0	0	0	0	0	0.73779438	0.47833296	0
ENSG00000211878	0	0	0	0	0.29964058	0	0	0	0	0	0	0	0	0	0.46716823	0.51818803	0	0	0	0	0	0	0	0	0	0.44206788	0	0	0	0	0	0	0	0	0	0	0	0	0	0	0	0	0	0.45860571	0	0	0	0

ENSG00000211879	0	0	0	0	0	0	0	0	0	0	0	0	0	0.54555846	0	0	0.75758171	0.60745153	1.10841808	0	0	0	2.04053648	0.46819414	0	0.41443863	0.86659775	0	0	0	0	0	0	0	0	0	0.46601342	0	0	0	0	0	0	0.42994285	0	0	0.89687429	0
ENSG00000211880	0	0	0	0	0	0	0	0	0	0	0	0	0	0	0	0	0	0	0	0	0	0	0	0	0	0	0.45460865	0	0	0	0	0	0	0	0	0	0	0	0	0	0	0	0	0	0	0	0	0
ENSG00000211881	0	0	0	0	0.29964058	0	0	0	0	0	0	0	0	0	0	0	0	0	0	0	0	0	0	0	0	0	0	0	0	0	0	0	0	0	0	0	0	0	0	0	0	0	0	0	0	0	0	0
ENSG00000211882	0	0	0	0	0	0	0	0	0	0	0	0	1.84946254	0	0.95017267	0	0	0	0	0	0	0	0	0	0	0	0.47001912	0	0.39227404	0	0	0	0	0	0	0	0	0	0.55023456	0	0	0	0	0	0.50389213	0	0	0.51071351
ENSG00000211883	0	0	0	0	0	0	0	0	0	0	0	0	0	1.68947136	0	0	0	0	0	0	0	0	0	0.48329718	0	0.85561524	0	0	0	0	0	0	0	0	0	0	0	0	0	0	0	0	0	0	0.47951025	0	0	0
ENSG00000211884	0	0	0	0	0	0	0	0	0	0	0	0	0	0	0	0	0	0	0	0	0	0	0	0	0	0	0	0	0	0	0	0	0	0	0	0	0	0	0	0	0	0	0	0	0	0	0	0
ENSG00000211885	0	0	0	0	0	0.76750252	0	0	0	0	0	0	0.43300909	0	0	0	0	0	0	0	0	0	0	0	0	0	0.44017663	0	0	0	0	0	0	0	0	0	0	0	0	0.82175294	0	0	0	0	0	0	0	0
ENSG00000211886	0	0	0	0	0	0	0	0	0	0	0	0	0	0	0	0	0	0	0	0.46157348	0	0	0	0	0	0.42780762	0	0	0	0	0	0	0	0	0	0	0	0.52382405	0	0	0	0	0	0	0	0	0	0
ENSG00000211887	0	0	0	0	0	0	0	0	0	0	0	0	0	0	0	0	0	0	0	0	0	0	0	0	0	0	0	0	0	0	0	0	0	0	0	0	0	0	0	0	0	0	0	0	0	0	0	0
ENSG00000211888	0	0	0	0	0	0	0	0	0	0	0	0	0	0	0	0	0	0	0	0	0	0	0	0	0	0	0	0	0	0	0	0	0	0	0	0	0	0	0	0	0	0	0	0	0	0	0	0
ENSG00000211890	0	0	0	0	0	0	0	0	0	0	0	0	0	0	0	0.02903014	0	0	0	0	0	0	0	0	0	0	0	0	0	0	0	0	0	0	0	0	0	0	0	0	0	0	0	0	0	0	0	0
ENSG00000211891	0	0	0	0	0	0.01337186	0	0	0	0	0	0	0	0	0	0	0	0	0	0	0	0	0	0	0	0	0	0	0	0	0	0	0	0	0	0	0	0	0	0	0	0	0	0	0.01644338	0	0	0
ENSG00000211892	0	0	0	0	0	0	0	0	0	0	0	0	0.09830477	0.09436687	0.02525234	0	0	0.07004846	0	0	0	0	0	0	0	0	0	0	0	0.04671341	0	0	0	0	0	0	0.02686924	0	0	0	0	0	0.06648884	0.07436849	0.05356691	0	0	0
ENSG00000211893	0	0	0	0	0	0	0	0	0	0	0	0	0	0	0	0	0	0	0	0	0	0	0	0	0	0	0	0	0	0	0	0	0	0	0	0	0	0	0	0	0	0	0	0	0	0	0	0
ENSG00000211895	0	0	0	0	0	0	0.04663584	0	0	0	0	0	0	0	0	0	0	0	0	0	0	0	0	0	0	0	0	0	0	0	0	0	0	0	0	0	0	0	0	0	0	0	0	0	0	0	0	0
ENSG00000211896	0	0	0	0	0	0	0	0	0	0	0	0	0	0	0	0.00651946	0	0	0	0	0	0	0	0.00628317	0	0	0	0	0	0	0	0	0	0	0	0	0	0	0	0	0	0	0	0.00576983	0	0.00928238	0	0
ENSG00000211897	0	0	0	0	0	0	0	0	0	0	0	0	0	0	0	0	0	0	0	0	0	0	0	0	0	0	0	0	0	0	0	0	0	0	0	0	0	0	0	0	0	0	0	0	0	0	0	0
ENSG00000211898	0	0	0	0	0	0	0	0	0	0	0	0	0	0	0	0	0	0	0	0	0	0	0	0	0	0	0	0	0	0	0	0	0	0	0	0	0	0	0	0	0	0	0	0	0	0	0	0
ENSG00000211899	0.02206005	0	0	0	0	0	0	0	0	0	0	0	0.01867185	0	0	0	0	0	0	0	0	0	0	0	0	0	0	0	0	0	0	0	0	0	0	0	0	0	0	0	0	0	0	0.01883391	0	0	0	0
ENSG00000211900	0	0	0	0	0	0	0	0	0	0	0	0	0	0	0	0	0	0	0	0	0	0	0	0	0	0	0	0	0	0	0	0	0	0	0	0	0	0	0	0	0	0	0	0	0	0	0	0
ENSG00000211904	0	0	0	0	0	0	0	0	0	0	0	0	0	0	0	0	0	0	0	0	0	0	0	0	0	0	0	0	0	0	0	0	0	0	0	0	0	0	0	0	0	0	0	0	0	0	0	0
ENSG00000211905	0	0	0	0	0	0	0	0	0	0	0	0	0	0	0	0	0	0	0	0	0	0	0	0	0	0	0	0	0	0	0	0	0	0	0	0	0	0	0	0	0	0	0	0	0	0	0	0
ENSG00000211907	0	0	0	0	0	0	0	0	0	0	0	0	0	0	0	0	0	0	0	0	0	0	0	0	0	0	0	0	0	0	0	0	0	0	0	0	0	0	0	0	0	0	0	0	0	0	0	0
ENSG00000211909	0	0	0	0	0	0	0	0	0	0	0	0	0	0	0	0	0	0	0	0	0	0	0	0	0	0	0	0	0	0	0	0	0	0	0	0	0	0	0	0	0	0	0	0	0	0	0	0
ENSG00000211911	0	0	0	0	0	0	0	0	0	0	0	0	0	0	0	0	0	0	0	0	0	0	0	0	0	0	0	0	0	0	0	0	0	0	0	0	0	0	0	0	0	0	0	0	0	0	0	0
ENSG00000211912	0	0	0	0	0	0	0	0	0	0	0	0	0	0	0	0	0	0	0	0	0	0	0	0	0	0	0	0	0	0	0	0	0	0	0	0	0	0	0	0	0	0	0	0	0	0	0	0
ENSG00000211914	0	0	0	0	0	0	0	0	0	0	0	0	0	0	0	0	0	0	0	0	0	0	0	0	0	0	0	0	0	0	0	0	0	0	0	0	0	0	0	0	0	0	0	0	0	0	0	0
ENSG00000211915	0	0	0	0	0	0	0	0	0	0	0	0	0	0	0	0	0	0	0	0	0	0	0	0	0	0	0	0	0	0	0	0	0	0	0	0	0	0	0	0	0	0	0	0	0	0	0	0
ENSG00000211917	0	0	0	0	0	0	0	0	0	0	0	0	0	0	0	0	0	0	0	0	0	0	0	0	0	0	0	0	0	0	0	0	0	0	0	0	0	0	0	0	0	0	0	0	0	0	0	0
ENSG00000211918	0	0	0	0	0	0	0	0	0	0	0	0	0	0	0	0	0	0	0	0	0	0	0	0	0	0	0	0	0	0	0	0	0	0	0	0	0	0	0	0	0	0	0	0	0	0	0	0
ENSG00000211920	0	0	0	0	0	0	0	0	0	0	0	0	0	0	0	0	0	0	0	0	0	0	0	0	0	0	0	0	0	0	0	0	0	0	0	0	0	0	0	0	0	0	0	0	0	0	0	0
ENSG00000211921	0	0	0	0	0	0	0	0	0	0	0	0	0	0	0	0	0	0	0	0	0	0	0	0	0	0	0	0	0	0	0	0	0	0	0	0	0	0	0	0	0	0	0	0	0	0	0	0
ENSG00000211923	0	0	0	0	0	0	0	0	0	0	0	0	0	0	0	0	0	0	0	0	0	0	0	0	0	0	0	0	0	0	0	0	0	0	0	0	0	0	0	0	0	0	0	0	0	0	0	0
ENSG00000211924	0	0	0	0	0	0	0	0	0	0	0	0	0	0	0	0	0	0	0	0	0	0	0	0	0	0	0	0	0	0	0	0	0	0	0	0	0	0	0	0	0	0	0	0	0	0	0	0
ENSG00000211925	0	0	0	0	0	0	0	0	0	0	0	0	0	0	0	0	0	0	0	0	0	0	0	0	0	0	0	0	0	0	0	0	0	0	0	0	0	0	0	0	0	0	0	0	0	0	0	0
ENSG00000211928	0	0	0	0	0	0	0	0	0	0	0	0	0	0	0	0	0	0	0	0	0	0	0	0	0	0	0	0	0	0	0	0	0	0	0	0	0	0	0	0	0	0	0	0	0	0	0	0
ENSG00000211930	0	0	0	0	0	0	0	0	0	0	0	0	0	0	0	0	0	0	0	0	0	0	0	0	0	0	0	0	0	0	0	0	0	0	0	0	0	0	0	0	0	0	0	0	0	0	0	0
ENSG00000211931	0	0	0	0	0	0	0	0	0	0	0	0	0	0	0	0	0	0	0	0	0	0	0	0	0	0	0	0	0	0	0	0	0	0	0	0	0	0	0	0	0	0	0	0	0	0	0	0
ENSG00000211933	0	0	0	0	0	0	0	0	0	0	0	0	0	0	0	0	0	0	0	0	0	0	0	0	0	0	0	0	0	0	0	0	0	0	0	0	0	0	0	0	0	0	0	0	0	0	0	0
ENSG00000211934	0	0	0	0	0	0	0	0	0.07679087	0.07034026	0	0	0	0	0	0	0	0.18735854	0.08546838	0	0	0	0.31468514	0.07220343	0	0	0.06682199	0.11711803	0.33461448	0.74966588	0	0	0	0	0	0	0	0	0	0.12474804	0.29016174	0.13906796	0	0.13260888	0.07163768	0	0.06915657	0.21782239
ENSG00000211935	0	0	0	0	0	0	0	0	0	0	0	0	0.48589569	0.3553765	0.92720412	0.55378874	0.12337208	0.29677021	0	0	0	0	0	0	0	0	0	0.18551138	0.23556405	0.2638773	0	0	0	0	0	0	0	0	0	0	0	0.14685293	0.65727517	0.21004842	0.15129586	0.56320182	0.65725139	0
ENSG00000211936	0	0	0	0	0	0	0	0	0	0	0	0	0	0	0	0	0	0	0	0	0	0	0	0	0	0	0	0	0	0	0	0	0	0	0	0	0	0	0	0	0	0	0	0	0	0	0	0
ENSG00000211937	0	0	0	0	0	0	0	0	0	0	0	0	0	0	0	0	0	0	0	0	0	0	0	0	0	0	0	0	0	0	0	0	0	0	0	0	0	0	0	0	0	0	0	0	0	0	0	0.07992599
ENSG00000211938	0	0	0	0	0	0	0	0	0	0	0	0	0	0	0	0	0	0	0	0	0	0	0	0	0	0	0	0	0	0	0	0	0	0	0	0	0	0	0	0	0	0	0	0	0	0	0	0
ENSG00000211939	0	0	0	0	0	0	0	0	0	0	0	0	0	0	0	0	0	0	0	0	0	0	0	0	0	0	0	0	0	0	0	0	0	0	0	0	0	0	0	0	0	0	0	0	0	0	0	0
ENSG00000211940	0	0	0	0	0	0	0	0	0	0	0	0	0	0	0	0	0	0	0	0	0	0	0	0	0	0	0	0	0	0	0	0	0	0	0	0	0	0	0	0	0	0	0	0	0	0	0	0
ENSG00000211941	0	0	0	0	0	0	0	0	0	0	0	0	0	0	0	0	0	0	0	0	0	0	0	0	0	0	0	0	0	0	0	0	0	0	0	0	0	0	0	0	0	0	0	0	0	0	0	0
ENSG00000211942	0	0	0	0	0	0	0	0	0	0	0	0	0	0	0	0	0	0	0	0	0	0	0	0	0	0	0	0	0	0	0	0	0	0	0	0	0	0	0	0	0	0	0	0	0	0	0	0
ENSG00000211943	0	0	0	0	0	0	0	0	0	0	0	0	0	0	0	0	0	0	0	0	0	0	0	0	0	0	0	0	0	0	0	0	0	0	0	0	0	0	0	0	0	0	0	0	0	0	0	0
ENSG00000211944	0	0	0	0	0	0	0	0	0	0	0	0	0	0	0	0	0	0	0	0	0	0	0	0	0	0	0	0	0	0	0	0	0	0	0	0	0	0	0	0	0	0	0	0	0	0	0	0
ENSG00000211945	0	0	0	0	0	0	0	0	0	0	0	0	0	0	0	0	0	0	0	0	0	0	0	0	0	0	0	0	0	0	0	0	0	0	0	0	0	0	0	0	0	0	0	0	0	0	0	0
ENSG00000211946	0	0	0	0	0	0	0	0	0	0	0	0	0	0	0	0	0	0	0	0	0	0	0	0	0	0	0	0	0	0	0	0	0	0	0	0	0	0	0	0	0	0	0	0	0	0	0	0
ENSG00000211947	0	0	0	0	0	0	0	0	0	0	0	0	0	0	0	0	0	0	0	0	0	0	0	0	0	0	0	0	0	0	0	0	0	0	0	0	0	0	0	0	0	0	0	0	0	0	0	0
ENSG00000211949	0	0	0	0	0	0	0	0	0	0	0	0	0	0	0	0	0	0	0	0	0	0	0	0	0	0	0	0	0	0	0	0	0	0	0	0	0	0	0	0	0	0	0	0	0	0	0	0
ENSG00000211950	0	0	0	0	0	0.05882319	0	0	0	0	0	0	0	0	0	0	0	0	0	0	0	0	0	0	0	0	0	0	0	0	0	0	0	0	0	0	0	0	0	0	0	0	0	0	0	0	0	0
ENSG00000211951	0	0	0	0	0	0	0	0	0	0	0	0	0	0	0	0	0	0	0	0	0	0	0	0	0	0	0	0	0	0	0	0	0	0	0	0	0	0	0	0	0	0	0	0	0	0	0	0
ENSG00000211952	0	0	0	0	0	0	0	0	0	0	0	0	0	0	0	0	0	0	0	0	0	0	0	0	0	0	0	0	0	0	0	0	0	0	0	0	0	0	0	0	0	0	0	0	0	0	0	0
ENSG00000211953	0	0	0	0	0	0	0	0	0	0	0	0	0	0	0	0	0	0	0	0	0	0	0	0	0	0	0	0	0	0	0	0	0	0	0	0	0	0	0	0	0	0	0	0	0	0	0	0
ENSG00000211955	0	0	0	0	0	0	0	0	0	0	0	0	0	0	0	0	0	0	0	0	0	0	0	0	0	0	0	0	0	0	0	0	0	0	0	0	0	0	0	0	0	0	0	0	0	0	0	0
ENSG00000211956	0	0	0	0	0	0	0	0	0	0	0	0	0	0	0	0	0	0	0	0	0	0	0	0	0	0	0	0	0	0	0	0	0	0	0	0	0	0	0	0	0	0	0	0	0	0	0	0
ENSG00000211957	0	0	0	0	0	0	0	0	0	0	0	0	0	0	0	0	0	0	0	0	0	0	0	0	0	0	0	0	0	0	0	0	0	0	0	0	0	0	0	0	0	0	0	0	0	0	0	0
ENSG00000211958	0	0	0	0	0	0	0	0	0	0	0	0	0	0	0	0	0	0	0	0	0	0	0	0	0	0	0	0	0	0	0	0	0	0	0	0	0	0	0	0	0	0	0	0	0	0	0	0
ENSG00000211959	0	0	0	0	0	0	0	0	0	0	0	0	0	0	0	0	0	0	0	0	0	0	0	0	0	0	0	0	0	0	0	0	0	0	0	0	0	0	0	0	0	0	0	0	0	0	0	0
ENSG00000211961	0	0	0	0	0	0	0	0	0	0	0	0	0	0	0	0	0	0	0	0	0	0	0	0	0	0	0	0	0	0	0	0	0	0	0	0	0	0	0	0	0	0	0	0	0	0	0	0
ENSG00000211962	0	0	0	0	0	0	0	0	0	0	0	0	0	0	0	0	0	0	0	0	0	0	0	0	0	0	0	0	0	0	0	0	0	0	0	0	0	0	0	0	0	0	0	0	0	0	0	0
ENSG00000211964	0	0	0	0	0	0	0	0	0	0	0	0	0	0	0	0	0	0	0	0	0	0	0	0	0	0	0	0	0	0	0	0	0	0	0	0	0	0	0	0	0	0	0	0	0	0	0	0
ENSG00000211965	0	0	0	0	0	0	0	0	0	0	0	0	0	0	0	0	0	0	0	0	0	0	0	0	0	0	0	0	0	0	0	0	0	0	0	0	0	0	0	0	0	0	0	0	0	0	0	0
ENSG00000211966	0	0	0	0	0	0	0	0	0	0	0	0	0	0	0	0	0	0	0	0	0	0	0	0	0	0	0	0	0	0	0	0	0	0	0	0	0	0	0	0	0	0	0	0	0	0	0	0
ENSG00000211967	0	0	0	0	0	0	0	0	0	0	0	0	0	0	0	0	0	0	0	0	0	0	0	0	0	0	0	0	0	0	0	0	0	0	0	0	0	0	0	0	0	0	0	0	0	0	0	0
ENSG00000211968	0	0	0	0	0	0	0	0	0	0	0	0	0	0	0	0	0	0	0	0	0	0	0	0	0	0	0	0	0	0	0	0	0	0	0	0	0	0	0	0	0	0	0	0	0	0	0	0
ENSG00000211970	0	0	0	0	0	0	0	0	0	0	0	0	0	0	0	0	0	0	0	0	0	0	0	0	0	0	0	0	0	0	0	0	0	0	0	0	0	0	0	0	0	0	0	0	0	0	0	0
ENSG00000211972	0	0	0	0	0	0	0	0	0	0	0	0	0	0	0	0	0	0	0	0	0	0	0	0	0	0	0	0	0	0	0	0	0	0	0	0	0	0	0	0	0	0	0	0	0	0	0	0
ENSG00000211973	0	0	0	0	0	0	0	0	0	0	0	0	0	0	0	0	0	0	0	0	0	0	0	0	0	0	0	0	0	0	0	0	0	0	0	0	0	0	0	0	0	0	0	0	0	0	0	0
ENSG00000211974	0	0	0	0	0	0	0	0	0	0	0	0	0	0	0	0	0	0	0	0	0	0	0	0	0	0	0	0	0	0	0	0	0	0	0	0	0	0	0	0	0	0	0	0	0	0	0	0
ENSG00000211976	0	0	0	0	0	0	0	0	0	0	0	0	0	0	0	0	0	0	0	0	0	0	0	0	0	0	0	0	0	0	0	0	0	0	0	0	0	0	0	0	0	0	0	0	0	0	0	0
ENSG00000211978	0	0	0	0	0	0	0	0	0	0	0	0	0	0	0	0	0	0	0	0	0	0	0	0	0	0	0	0	0	0	0	0	0	0	0	0	0	0	0	0	0	0	0	0	0	0	0	0
ENSG00000211979	0	0	0	0	0	0	0	0	0	0	0	0	0	0	0	0	0	0	0	0	0	0	0	0	0	0	0	0	0	0	0	0	0	0	0	0	0	0	0	0	0	0	0	0	0	0	0	0
ENSG00000211983	0	0	0	0	0	0	0	0	0	0	0	0	0	0	0	0	0	0	0	0	0	0	0	0	0	0	0	0	0	0	0	0	0	0	0	0	0	0	0	0	0	0	0	0	0	0	0	0
ENSG00000211984	0.74952883	0.50204629	0	0	0	0	0	0	0	0	0	0	0	0	0	0	0	0	0	0	0	0	0	0	0	0	0	0	0	0	0	0	0	0	0	0	0	0	0	0	0	0	0	0	0	0	0	0
ENSG00000211987	0	0	0	0	0	0	0	0	0	0	0	0	0	0	0	0	0	0	0	0	0	0	0	0	0	0	0	0	0.24362282	0	0	0	0	0	0	0	0	0	0	0	0	0	0	0	0	0	0	0
ENSG00000211990	0	0	0	0	0.30471924	0	0	0	0.13503479	0.2473831	0	0	0	0	0	0	0	0	0	0	0	0	0	0	0	0	0	0	0	0	0	0	0	0	0	0	0	0	0	0	0	0	0	0	0	0	0.24322015	0
ENSG00000211991	0	0	0	0	0	0	0	0	0	0	0	0	0	0	0	0	0	0	0	0	0	0	0	0	0	0	0	0	0	0	0	0	0	0	0	0	0	0	0	0	0	0	0	0	0	0	0	0
ENSG00000211995	0	0	0	0	0	0	0	0	0	0	0	0	0	0	0	0	0	0	0	0	0	0	0	0	0	0	0	0	0	0	0	0	0	0	0	0	0	0	0	0	0	0	0	0	0	0	0	0
ENSG00000211996	0	0	0	0	0	0	0	0	0	0	0	0	0	0	0	0	0	0	0	0	0	0	0	0	0	0	0	0	0	0	0	0	0	0	0	0	0	0	0	0	0	0	0	0	0	0	0	0
ENSG00000211997	0	0.24531807	0	0	1.02150199	0.54946203	0	0	0.36213876	0.33171825	0	0.30929535	0	0	0	0	0	0	0	1.300798	0	0	0	1.02151449	0.34962551	0	0.63025291	0	0.78900573	0	0	0	0	0	0	0	0	0.36905785	0	1.1766008	0	0	0	0	0	0	0	0
ENSG00000212001	0	0	0	0	0	0	0	0	0	0	0	0	0	0	0	0	0	0	0	0	0	0	0	0	0	0	0	0	0	0	0	0	0	0	0	0	0.26629339	0	0	0	0	0	0	0	0	0	0	0
ENSG00000212007	0	0	0	0	0	0	0	0	0	0	0	0	0	0	0	0	0	0	0	0	0	0	0	0	0	0	0	0	0	0	0	0	0	0	0	0	0	0	0	0	0	0	0	0	0	0	0	0
ENSG00000212013	0	0	0	0	0	0	0	0	0	0	0	0	0	0	0	0	0	0	0	0	0	0	0	0	0	0	0	0	0	0	0	0	0	0	0	0	0	0	0	0	0	0	0	0	0	0	0	0
ENSG00000212014	0	0	0	0	0	0	0	0	0	0	0	0	0	0	0	0	0	0	0	0	0	0	0	0	0	0	0	0	0	0	0	0	0	0	0	0	0	0	0	0	0	0	0	0	0	0	0	0
ENSG00000212017	0.39789802	0.2665184	0.38913712	0	0	0	0	0	0.3934347	0	0.50128081	0.67204915	0.33678485	0.43105854	0.34605054	0	0	0.4799617	0	0	0.33494727	0	0	0.73986235	0.37984006	0	0.3423596	0	0.28573047	0.3200734	0	0	0.65276351	0	0	0.35394581	0.36820814	0.80190348	0	0.63914117	0	0	0	0	0	0.54651436	0	0
ENSG00000212022	0	0	0	0	0	0	0	0	0	0	0	0	0	0	0	0	0	0	0	0	0	0	0	0	0	0	0	0	0	0	0	0	0	0	0	0	0	0	0	0	0	0	0	0	0	0	0	0
ENSG00000212024	0	0	0.3317906	0.4385337	0	0.25448768	0	0	0.16772743	0	0.42740784	0	0	0	0	0	0	0	0	0	0	0	0	0	0	0	0	0	0.24362282	0	0	0.83015394	0.55656678	0	0	0.60357075	0.31394589	0	0	0	0.63377432	0	0	0	0	0	0	0
ENSG00000212025	0	0	0	0	0	0	0	0	0	0	0	0	0	0	0	0	0	0	0	0	0	0	0	0	0	0	0	0	0	0	0	0	0	0	0	0	0	0	0	0	0	0	0	0	0	0	0	0
ENSG00000212027	1.34290582	0	0.87555852	2.3144835	0.74910146	2.68625882	0.71896924	0.87692463	0.44261404	0	0.56394091	0.7560553	0.37888295	0	0.38930686	0	0	0.53995691	0	0	0	0	0	0.41617257	0.85464014	0.73677979	0.38515455	0	0.32144678	0	0.6801192	0.547671	2.93743578	2.11354273	1.7436014	0.39818903	2.48540493	2.25535355	3.15620659	0.71903382	0.83623	0	1.02503627	1.14651428	2.06455804	0	0.79722159	0
ENSG00000212030	0	0	0	0	0	0	0	0	0	0	0	0	0	0	0	0	0	0	0	0	0	0	0	0	0	0	0	0	0	0	0	0	0	0	0	0	0	0	0	0	0	0	0	0	0	0	0	0
ENSG00000212031	0	0	0	0	0	0	0	0	0	0	0	0	0	0	0	0	0	0	0	0	0	0	0	0	0	0	0	0	0	0	0	0	0	0	0	0	0	0	0	0	0	0	0	0	0	0	0	0
ENSG00000212032	0	0	0	0	0	0	0	0	0	0	0	0	0	0	0	0	0	0	0	0	0	0	0	0	0	0	0	0	0	0	0	0	0	0	0	0	0	0	0	0	0	0	0	0	0	0	0	0
ENSG00000212034	0	0	0	0	0	0	0	0	0	0	0	0	0	0	0	0	0	0	0	0	0	0	0	0	0	0	0	0	0	0	0	0	0	0	0	0	0	0	0	0	0	0	0	0	0	0	0	0
ENSG00000212036	0	0	0	0	0	0	0	0	0	0	0	0	0	0	0	0	0	0	0	0	0	0	0	0	0	0	0	0	0	0	0	0	0	0	0	0	0	0	0	0	0	0	0	0	0	0	0	0
ENSG00000212037	0	0	0	0	0	0	0	0	0	0	0	0	0	0	0	0	0	0	0	0	0	0	0	0	0	0	0	0	0	0	0	0	0.56853596	0	0	0	0	0	0	0	0	0	0	0	0	0	0	0
ENSG00000212039	0	0	0	0	0	0	0	0	0	0	0	0	0	0	0	0	0	0	0	0	0	0	0	0	0	0	0	0	0	0	0	0	0	0	0	0	0	0	0	0	0	0	0	0	0	0	0	0
ENSG00000212040	0	0	0.40410393	0	0	0	0	0	0	0	0	0	0.34973811	0	0	0	0	0	0	0.36689174	0	0	0	0	0	0	0.35552728	0	0.2967201	0.66476782	0	0	0	0	0	0	0	0	0	0	0	0	0	0	0	0	0	0
ENSG00000212044	0	0	0	0	0	0	0	0	0	0	0	0	0	0	0	0	0	0	0	0	0	0	0	0	0	0	0	0	0	0	0	0	0	0	0	0	0	0	0	0	0	0	0	0	0	0	0	0
ENSG00000212045	0	0	0	0	0.15633422	0	0	0	0	0	0	0	0	0	0	0	0	0	0	0	0	0	0	0	0	0	0	0	0	0	0	0	0	0	0	0	0	0	0	0	0	0	0	0	0	0	0	0
ENSG00000212049	0.29035802	0	0.85189477	0	0.32393577	0	0	0	0	0	0	0	0	0	0	0	0	0	0	0	0	0	0	0	0	0	0	0	0	0	0	0	0	0	0	0	0	0	0	0	0	0	0	0	0	0	0	0
ENSG00000212051	0	0	0	0	0	0	0	0	0	0	0	0	0	0	0	0	0	0	0	0	0	0	0	0	0	0	0	0	0	0	0	0	0	0	0	0	0	0	0	0	0	0	0	0	0	0	0	0
ENSG00000212054	0	0	0	0	0	0	0	0	0	0	0	0	0	0	0	0	0	0	0	0	0	0	0	0	0	0	0	0	0	0	0	0	0	0	0	0	0	0	0	0	0	0	0	0	0	0	0	0
ENSG00000212055	0	0	0	0	0	0	0	0	0	0	0	0	0	0	0	0	0	0	0	0	0	0	0	0	0	0	0	0	0	0	0	0	0	0	0	0	0	0	0	0	0	0	0	0	0	0	0	0
ENSG00000212057	0	0	0	0	0	0	0	0	0	0	0	0	0	0	0	0	0	0	0	0	0	0	0	0	0	0	0	0	0	0	0	0	0	0	0	0	0	0	0	0	0.66163253	0	0	0	0	0	0	0
ENSG00000212061	0	0	0	0	0	0	0	0	0	0	0	0	0	0	0	0	0	0	0	0	0	0	0	0	0	0	0	0	0	0	0	0	0	0	0	0	0	0	0	0	0	0	0	0	0	0	0	0
ENSG00000212063	0	0	0	0	0	0	0	0	0	0	0	0	0	0	0	0	0	0	0	0	0	0	0	0	0	0	0	0	0	0	0	0	0	0	0	0	0	0	0	0	0	0	0	0	0	0	0	0
ENSG00000212065	0	0	0	0	0	0	0	0	0	0	0	0	0	0	0	0	0	0	0	0	0	0	0	0	0	0	0	0	0	0	0	0	0	0	0	0	0	0	0	0	0	0	0	0	0	0	0	0
ENSG00000212066	0	0	0	0	0	0	0	0	0	0	0	0	0	0	0	0	0	0	0	0	0	0	0	0	0	0	0	0	0	0	0	0	0	0	0	0	0	0	0	0	0	0	0	0	0	0	0	0
ENSG00000212067	0	0	0	0	0	0	0	0	0	0	0	0	0	0	0	0	0	0	0	0	0	0	0	0	0	0	0	0	0	0	0	0	0	0	0	0	0	0	0	0	0	0	0	0	0	0	0	0
ENSG00000212070	0	0	0	0	0	0	0	0	0	0	0	0	0	0	0	0	0	0	0	0	0	0	0	0	0	0	0	0	0	0	0	0	0	0	0	0	0	0	0	0	0	0	0	0	0	0	0	0
ENSG00000212071	0	0	0	0	0	0	0	0	0	0	0	0	0	0	0	0	0	0	0	0	0	0	0	0	0	0	0	0	0	0	0	0	0	0	0	0	0	0	0	0	0	0	0	0	0	0	0	0
ENSG00000212072	0	0	0	0	0	0	0	0	0	0	0	0	0	0	0	0	0	0	0	0	0	0	0	0	0	0	0	0	0	0	0	0	0	0	0	0	0	0	0	0	0	0	0	0	0	0	0	0
ENSG00000212073	0	0	0	0	0	0	0	0	0	0	0	0	0	0	0	0	0	0	0	0	0	0	0	0	0	0	0	0	0	0	0	0	0	0	0	0	0	0	0	0	0	0	0	0	0	0	0	0
ENSG00000212075	0	0	0	0	0	0	0	0	0	0	0	0	0	0	0	0	0	0	0	0	0	0	0	0	0	0	0	0	0	0	0	0	0	0	0	0	0	0	0	0	0	0	0	0	0	0	0	0
ENSG00000212076	0	0	0	0	0	0	0.36975561	0	0	0	0	0	0	0	0	0	0	0	0	0	0	0	0	0	0.43952921	0	0	0.34717129	0	0	0	0	0	0	0	0	0	0	0	0	0	0	0	0	0	0	0	0
ENSG00000212082	0	0	0	0	0	0	0	0	0	0	0	0	0	0	0	0	0	0	0	0	0	0	0	0	0	0	0	0	0	0	0	0	0	0	0	0	0	0	0	0	0	0	0	0	0	0	0	0
ENSG00000212084	0	0	0	0	0	0	0	0	0	0	0	0	0	0	0	0	0	0	0	0	0	0	0	0	0	0	0	0	0	0	0	0	0	0	0	0	0	0	0	0	0	0	0	0	0	0	0	0
ENSG00000212085	0	0	0	0	0	0	0	0	0	0	0	0	0	0	0	0	0	0	0	0	0	0	0	0	0	0	0	0	0	0	0	0	0	0	0	0	0	0	0	0	0	0	0	0	0	0	0	0
ENSG00000212086	0	0	0	0	0	0	0	0	0	0	0	0	0	0	0	0	0	0	0	0	0	0	0	0	0	0	0	0	0	0	0	0	0	0	0	0	0	0	0	0	0	0	0	0	0	0	0	0
ENSG00000212089	0	0.23723067	0	0	0	0	0	0	0.17510006	0	0	0	0	0.38368947	0	0.34166244	0	0	0	0	0	0.30249152	0	0.3292794	0	0	0	0.26705484	0	0	0	0	0	0	0	0	0	0	0	0	0	0	0	0	0	0	0	0.33112195
ENSG00000212091	0	0	0	0	0	0	0	0	0	0	0	0	0	0	0	0	0	0	0	0	0	0	0	0	0	0	0	0	0	0	0	0	0	0	0	0	0	0	0	0	0	0	0	0	0	0	0	0
ENSG00000212093	0	0	0	0	0	0	0	0	0	0	0	0	0	0	0	0	0	0	0	0	0	0	0	0	0	0	0	0	0	0	0	0	0	0	0	0	0	0	0	0	0	0	0	0	0	0	0	0
ENSG00000212094	0	0	0	0	0	0	0	0	0	0	0	0	0	0	0	0	0	0	0	0	0	0	0	0	0	0	0	0	0	0	0	0	0	0	0	0	0	0	0	0	0	0	0.43930126	0	0	0	0	0
ENSG00000212095	0	0	0	0	0	0	0	0	0	0	0	0	0	0	0	0	0	0	0	0	0	0	0	0	0	0	0	0	0	0	0	0	0	0	0	0	0	0	0	0	0	0	0	0	0	0	0	0
ENSG00000212098	0	0	0	0	0	0	0	0	0	0	0	0	0	0	0	0	0	0	0	0	0	0	0	0	0	0	0	0	0	0	0	0	0	0	0	0	0	0	0	0	0	0	0	0	0	0	0	0
ENSG00000212099	0	0	0	0	0	0	0	0	0	0	0	0	0	0	0	0	0	0	0	0	0	0	0	0	0	0	0	0	0	0	0	0	0	0	0	0	0	0	0	0	0	0	0	0	0	0	0	0
ENSG00000212100	0	0	0	0	0	0	0	0	0	0	0	0	0	0	0	0	0	0	0	0	0	0	0	0	0	0	0	0	0	0	0	0	0	0	0	0	0	0	0	0	0	0	0	0	0	0	0	0
ENSG00000212101	0	0	0	0	0	0	0	0	0	0	0	0	0	0	0	0	0	0	0	0	0	0	0	0	0	0	0	0	0	0	0	0	0	0	0	0	0	0	0	0	0	0	0	0	0	0	0	0
ENSG00000212102	0.41320179	0.83030733	0.80820786	0	0	0	0	0.40473445	0	0	0	0.3489486	1.04921433	0	0	0.39860618	0	0	0.45473562	0	0.69565971	1.0587203	0.83714317	0	0	0.34005221	0	0.62312796	1.18688042	0.33238391	0.62780233	0	0	0.65032084	0	0.36755911	0	0	0.41620307	0	0	0	0	0	0.38114918	0	0	0.38630894
ENSG00000212104	0	0	0	0	0	0	0	0	0	0	0	0	0	0	0	0	0	0	0	0	0	0	0	0	0	0	0	0	0	0	0	0	0	0	0	0	0	0	0	0	0	0	0	0	0	0	0	0
ENSG00000212111	0	0	0	0	0	0	0	0	0	0	0	0	0	0	0	0	0	0	0	0	0	0	0	0	0	0	0	0	0	0	0	0	0	0	0	0	0	0	0	0	0	0	0	0	0	0	0	0
ENSG00000212114	0	0	0	0	0	0	0	0	0	0	0	0	0	0	0	0	0	0	0	0	0	0	0	0	0	0	0	0	0	0	0	0	0	0	0	0	0	0	0	0	0	0	0	0	0	0	0	0
ENSG00000212122	0	0.00871186	0	0	0	0.00975639	0	0	0	0	0	0	0.01100871	0	0.02262316	0	0	0	0.01431371	0	0	0	0	0.01209218	0	0	0	0	0	0	0	0	0	0	0	0.01156966	0	0	0	0	0	0	0.01489157	0.01110425	0	0	0.01158191	0
ENSG00000212123	0.09566875	0.0712005	0.04158325	0.082442	0.04150694	0.03986862	1.83536343	1.95746238	1.5976148	1.08793084	0.64280335	1.07722918	0.16194997	0.05757873	0.02773426	0.13330695	0.03198234	0.06411098	0.09358675	0.11326209	0.49214712	0.20881093	0.02153601	0.20753724	0.31457071	0.19245699	0.0548769	0.04809101	0.07633301	0.04275387	0.37146352	0.35114526	0.45340368	0.3178679	0.28983348	0.34040435	1.02301628	0.57841785	0.68525254	0.23904554	0.4368695	0.38069395	0.04868246	0.07260249	0.05883173	0.2774029	0.03786277	0.06956619
ENSG00000212124	0.51464654	0.81870623	0.56622946	0.8731285	0.41267865	0.82035449	0.56828706	0.69313803	0.50887363	0.55352587	0.2836589	0.35312762	0.19057586	0.2090763	0.3077156	0.18617534	0.14516536	0.42679229	0.63717446	0.5712087	0.37907206	0.21977427	0.391001	0.65790155	0.39917324	0.42353808	0.38746087	0.53357663	0.43886147	0.56923233	0.39096672	0.47224326	0.42214646	0.30374267	0.31322181	0.2002867	0.4167146	0.51859626	0.58318274	0.56833811	0.66097222	0.46078405	0.66289771	0.24715278	0.35604354	0.13253791	0.22914154	0.30071953
ENSG00000212125	0.27784258	0.72115055	0.78120954	0.718288	0.60057272	0.989979	0.44625677	0.74840982	0.41208893	0.25164833	0.26252421	0.35195678	0.35275309	0.15049889	0.12081937	0.33503537	0.41797611	0.20946604	0.45865576	0.15418942	0.43853549	0.29662422	0.17590832	0.48433877	0.39784972	0.4573116	0.1494134	0.28806239	0.29927804	0.36318673	0.47491082	0.5523923	0.455809	0.49194529	0.87931623	0.18536386	0.51422171	0.66494044	0.27986068	0.66944528	0.58391922	0.18657285	0.35787904	0.35581478	0.19221747	0.19080889	0.3711204	0.29222939
ENSG00000212126	0.09668922	0.2374679	0.25216085	0.1666428	0.10787061	0.45935026	0.02588289	0.03156929	0.14340695	0.05838241	0.12181124	0.10887196	0.05455914	0.10474722	0.05606019	0.06218256	0.04848523	0.19438449	0	0.08585267	0	0.05505346	0.06529717	0.05992885	0.09230113	0	0	0.17011393	0.18515334	0.12962973	0.04896858	0.11829694	0	0.15217508	0	0.08600883	0.05964972	0.06495418	0.06492768	0.05177043	0.24083424	0	0.25830914	0.05503269	0.08918891	0	0.14349989	0.15066049
ENSG00000212127	0.20143587	0.51516796	0.25072812	0.165696	0.24005297	0.22665309	0.19118046	0.26008787	0.23086346	0.27366756	0.21916794	0.30929535	0.20149684	0.18846565	0.12740952	0.23848426	0.22038741	0.09940116	0.16122445	0.15446976	0.19268983	0.12512149	0.12985232	0.44265628	0.18355339	0.12809921	0.17331955	0.15188744	0.23012667	0.09574923	0.31996517	0.33607084	0.31544055	0.44672608	0.26748431	0.15475074	0.22877023	0.24911405	0.23056704	0.10295257	0.22236116	0.26233274	0.14676656	0.09380571	0.24493166	0.21379269	0.13860785	0.19688586
ENSG00000212128	0.15750636	0.35606338	0.17329319	0.1526965	0.12080805	0.25106756	0.01581117	0.07713937	0.04866862	0.10699281	0.04960751	0.01662675	0.08332185	0	0.08561421	0.05697853	0.11847338	0.02374887	0.06500192	0.01748171	0	0.0336307	0.01994416	0.0732179	0.09397387	0.04860856	0.06776085	0.04453633	0.05655264	0.0633499	0.11965445	0.02408816	0.06459847	0.06197315	0.03834432	0.07005403	0.16397296	0.13887577	0.0594939	0	0.11033945	0.17627734	0.09016813	0.01680901	0.0363221	0.05408389	0.08766029	0.0184069
ENSG00000212133	0	0	0	0	0	0	0	0	0	0	0	0	0	0	0	0	0	0	0	0	0	0	0	0	0	0	0	0	0	0	0	0	0	0	0	0	0	0	0	0	0	0	0	0	0	0	0	0
ENSG00000212134	0	0	0	0	0	0	0	0	0	0	0	0	0	0	0	0	0	0	0	0	0	0	0	0	0	0	0	0	0	0	0	0	0	0	0	0	0	0	0	0	0	0	0	0	0	0	0	0
ENSG00000212135	1.16143206	0.97243201	1.1358597	0.7506433	1.13377518	1.08902385	0.23317921	1.13763196	1.0048535	0.26298384	1.46319803	0.98082849	0	0.62911246	0.7575701	0.28010164	1.3104116	0.70048464	2.55635161	0.77344746	0.73326294	1.48793124	1.76478831	1.88964843	0.83154175	0.47791122	0.49965996	2.40830535	1.66804815	2.33567073	1.32347519	1.06573816	0.95268188	0	1.69647704	2.06627824	0.53738485	1.17034562	1.4623351	1.39920095	1.62725838	1.03987752	0.99733259	0.49578996	0.26783456	0.79761555	0.51711671	0.27146034
ENSG00000212136	0	0	0	0	0	0	0	0	0	0	0	0	0	0	0	0	0	0	0	0	0	0	0	0	0	0	0	0	0	0	0	0	0	0	0	0	0	0	0	0	0	0	0	0	0	0	0	0
ENSG00000212138	0	0.56316497	0	0.362267	0	0	0	0	0	0.50767315	0	0.47335636	0.23721367	0	0	0	0.42161069	0	0	0	0.23591938	0.4787257	0.28390073	0.26056022	0	0	0	0	0.20125364	0.450886	0	0	0	0	0	0	0	0	0	0	0	0	0	0	0.25851857	0	0.24956502	0
ENSG00000212140	0.30121252	0.20175692	0	0	0.16802276	0.225947	0	0	0	0	0	0	0	0	0	0	0	0	0	0	0	0	0.30512695	0	0	0	0	0	0	0	0	0	0	0	0	0	0	0	0	0	0	0	0	0	0	0	0	0
ENSG00000212144	0	0	0	0	0	0	0	0	0	0	0	0	0	0	0	0	0	0	0	0	0	0	0	0	0	0	0	0	0	0	0	0	0	0	0	0	0	0	0	0	0	0	0	0	0	0	0	0
ENSG00000212145	0	0	0.44084065	0	0.12572332	0	0	0.44152849	0.22285462	0	0	0.1903356	0	0	0	0	0	0.27186642	0	0	0	0	0	0	0	0	0	0	0	0	0	0	0	0	0	0.20048679	0	0	0	0	0	0	0	0	0	0	0	0.21071397
ENSG00000212146	0	0.20175692	0	0	0	0	0	0	0	0	0	0	0	0	0.26196349	0	0	0	0	0	0	0	0	0	0	0.24788853	0	0	0	0	0	0	0	0	0	0	0	0	0	0	0	0	0	0	0	0	0	0
ENSG00000212147	0	0	0	0	0	0	0	0	0	0	0	0	0	0	0	0	0	0	0	0	0	0	0	0	0	0	0	0	0	0	0	0	0	0	0	0	0	0	0	0	0	0	0	0	0	0	0	0
ENSG00000212149	0	0	0	0	0	0	0	0	0	0	0	0	0	0	0	0	0	0	0	0	0	0	0	0	0	0	0	0	0	0	0	0	0.41307691	0	0	0	0	0	0	0	0	0	0	0	0	0	0	0
ENSG00000212153	0	0	0	0	0	0	0	0	0	0	0	0	0	0	0	0	0	0	0	0	0	0	0	0.36320515	0	0	0	0	0	0	0	0	0	0	0	0	0	0	0	0	0	0	0	0	0	0	0	0
ENSG00000212154	0	0	0	0	0	0	0	0	0	0	0	0	0	0	0	0	0	0	0	0	0	0	0	0	0	0	0	0	0	0	0	0	0	0	0	0	0	0	0	0	0	0	0	0	0	0	0	0
ENSG00000212156	0	0	0	0	0	0	0	0	0	0	0	0	0	0	0	0	0	0	0	0	0	0	0	0	0	0	0	0	0	0	0	0	0	0	0	0	0	0	0	0	0	0	0	0	0	0	0	0
ENSG00000212157	0	0	0	0	0	0	0	0	0	0	0	0	0	0	0	0	0	0	0	0	0	0	0	0	0	0	0	0	0	0	0	0	0	0	0	0	0	0	0	0	0	0	0	0	0	0	0	0
ENSG00000212158	2.54445314	0.28405251	0.41473825	1.6445014	0.23655836	2.22676718	2.04338626	1.66154141	1.25795569	2.68866373	2.13703922	1.43252582	2.5125922	1.83767061	1.47526809	0	0.63796354	1.02307626	2.80021409	1.50618716	1.78491636	1.08658136	1.71834651	2.3656125	2.83380677	2.79200764	1.09464978	1.27905212	2.13169969	0.68226171	3.86594069	2.07538484	0.69570848	2.00230364	1.65183291	1.88615859	1.17729707	0.42733015	2.13577889	2.0435698	1.58443579	0	0.4855435	1.44822856	1.95589709	1.1649385	1.13289385	1.98237482
ENSG00000212160	0	0	0	0	0	0	0	0	0	0	0	0	0	0	0	0	0	0	0	0	0	0	0	0	0	0	0	0	0	0	0	0	0	0	0	0	0	0	0	0	0	0	0	0	0	0	0	0
ENSG00000212161	0	0	0	0	0	0	0	0	0	0	0.60602605	0	0	0	0	0	0	0	0	0	0	0	0	0	0	0	0	0	0	0	0	0	0	0	0	0	0	0	0	0	0.89863522	0	0	0	0	0	0	0.4497328
ENSG00000212163	0.17235155	0.2308876	0.33711344	0	0.19228273	1.03428147	0.83046714	0.33763943	0.34083648	1.24882166	1.08566164	0.58220301	0.29176013	0.1867152	0.14989355	1.16384478	0	0.4157957	0	0.30607012	0.29016822	0.58880701	1.04754814	0.64095027	0.49358896	1.27655964	1.33465321	0.51982867	0.24753121	1.10913134	0	0	0	0	0.67133316	0.30662685	0.47847368	0.52102285	1.04162051	0	0.32197091	0	0.19733319	0	0.15898201	0	0	0.48340263
ENSG00000212165	0	0	0	0	0	0	0	0	0	0	0	0	0	0	0	0	0	0	0	0	0	0	0	0	0	0	0	0	0	0	0	0	0	0	0	0	0	0	0	0	0	0	0	0	0	0	0	0
ENSG00000212167	0	0	0	0	0	0	0	0	0	0	0	0	0	0	0	0	0	0	0	0	0	0	0	0	0	0	0	0	0	0	0	0	0	0	0	0	0	0	0	0	0	0	0	0	0	0	0	0
ENSG00000212168	0	0	0	0	0	0	0	0	0	0	0	0	0	0	0	0	0	0	0	0	0	0	0	0	0	0	0	0	0	0	0	0	0	0	0	0	0	0	0	0	0	0	0	0	0	0	0	0
ENSG00000212170	0	0	0.19456856	0	0	0	0	0	0	0.36038526	0	0	0	0	0	0	0	0	0	0	0	0	0	0	0.18992003	0	0	0.15001229	0	0	0	0	0	0	0.77493396	0	0.18410407	0	0.20039407	0	0	0	0	0	0	0	0	0
ENSG00000212171	0	0	0	0	0	0	0	0	0	0	0	0	0	0	0	0	0	0	0	0	0	0	0	0	0	0	0	0	0	0	0	0	0	0	0	0	0	0	0	0	0	0	0	0	0	0	0	0
ENSG00000212172	0	0	0	0	0	0	0	0	0	0	0	0	0	0	0	0	0	0	0	0	0	0	0	0	0	0	0	0	0	0	0	0	0	0	0	0	0	0	0	0	0	0	0	0	0	0	0	0
ENSG00000212174	0	0	0	0	0	0	0	0	0	0	0	0	0	0	0	0	0	0	0	0	0	0	0	0	0	0	0	0	0	0	0	0	0.6866733	0	0	0	0	0	0	0.67234331	0	0	0	0	0	0	0	0
ENSG00000212175	0	0	0	0	0	0	0	0	0	0	0	0	0	0	0	0	0	0	0	0	0	0	0	0	0	0	0	0	0	0	0	0	0	0	0	0	0	0	0	0	0	0	0	0	0	0	0	0
ENSG00000212176	0	0	0	0	0	0	0	0	0	0	0	0	0	0	0	0	0	0	0	0	0	0	0	0	0	0	0	0	0	0	0	0	0	0	0	0	0	0	0	0	0	0	0	0	0	0	0	0
ENSG00000212181	0	0	0	0	0	0	0	0	0	0	0	0	0	0	0	0	0	0	0	0	0	0	0	0	0	0	0	0	0	0	0	0	0	0	0	0	0	0	0	0	0	0	0	0	0	0	0	0
ENSG00000212182	0	0	0	0	0	0	0	0	0	0	0	0	0	0	0	0	0	0	0	0	0	0	0	0	0	0	0	0	0	0	0	0	0	0	0	0	0	0	0	0	0	0	0	0	0	0	0	0
ENSG00000212184	0	0	0	0	0	0	0	0	0	0	0	0	0	0	0	0	0	0	0	0	0	0	0	0	0	0	0	0	0	0	0	0	0	0	0	0	0	0	0	0	0	0	0	0	0	0	0	0
ENSG00000212186	0	0	0	0	0	0	0	0	0	0	0	0	0	0	0	0	0	0	0	0	0	0	0	0	0	0	0	0	0	0	0	0	0	0	0	0	0	0	0	0	0	0	0	0	0	0	0	0
ENSG00000212187	0	0	0	0	0	0	0	0	0	0	0	0	0	0	0	0	0	0	0	0	0	0	0	0	0	0	0	0	0	0	0	0	0	0	0	0	0	0	0	0	0	0	0	0	0	0	0	0
ENSG00000212189	0	0	0	0	0	0	0	0	0	0	0	0	0	0	0	0	0	0	0	0	0	0	0	0	0	0	0	0	0	0	0	0	0	0	0	0	0	0	0	0	0	0	0	0	0	0	0	0
ENSG00000212190	0	0.2248749	0.32833444	0	0.37455073	0	0	0	0	0.60815013	0	0.28352074	0	0	0	0.32386752	0	0.40496768	0.73894539	0	0.28261176	0.28673675	0.34008941	0.62425885	0	0	0	0.50629147	0.48217017	0	0	0	0	0	0	0	0	0	0	0	0.6271725	0	0	0	0	0	0	0
ENSG00000212191	0	0	0	0	0	0	0	0	0	0	0	0	0	0	0	0	0	0	0	0	0	0	0	0	0	0	0	0	0	0	0	0	0	0	0	0	0	0	0	0	0	0	0	0	0	0	0	0
ENSG00000212195	3.30561434	4.87113633	3.71775617	2.1364463	2.30492757	2.72758588	15.1315372	20.5605099	22.6346011	16.9159298	36.4392585	18.424486	9.51287655	18.4426737	15.8118478	9.24766334	21.8805137	12.3608598	13.0963859	5.4299978	15.7219095	18.7746401	9.04114626	13.5224072	6.46896841	11.4257543	10.9502402	6.10665398	9.61373137	5.98291041	4.26905588	2.42660381	12.7439522	3.12154003	8.69118239	27.1993741	4.89433587	7.99436088	8.98998623	11.681534	22.2308529	12.726501	4.73093665	5.64437799	15.5508864	13.6208194	7.2117892	3.70856582

ENSG00000212197	0	0	0	0	0	0	0	0	0	0	0	0.3489486	0	0	0	0	0	0	0	0	0	0	0	0	0	0	0	0	0	0	0	0	0	0	0	0	0	0	0	0	0	0	0	0	0	0	0	0
ENSG00000212199	0	0	0	0	0	0	0	0	0	0	0	0	0	0	0	0	0	0	0	0	0	0	0	0	0	0	0	0	0	0	0	0	0	0	0	0	0	0	0	0	0	0	0	0	0	0	0	0
ENSG00000212204	0	0	0	0	0	0	0	0	0	0	0	0	0	0	0	0	0	0	0	0	0	0	0	0	0	0	0	0	0	0	0	0	0	0	0	0	0	0	0	0	0	0	0	0	0	0	0	0
ENSG00000212205	0	0	0	0	0	0	0	0	0	0	0	0	0	0	0	0	0	0	0	0	0	0	0	0	0	0	0	0	0	0	0	0	0	0	0	0	0	0	0	0	0	0	0	0	0	0	0	0
ENSG00000212206	0	0	0	0	0	0	0	0	0	0	0	0	0	0	0	0	0	0	0	0	0	0	0	0	0	0	0	0	0	0	0	0	0	0	0	0	0.2176997	0	0	0	0	0	0	0	0	0	0	0
ENSG00000212207	0	0	0	0	0.16802276	0	0	0	0.14891687	0	0	0.25437374	0	0	0	0	0	0	0	0.26745379	0.25355821	0	0.6102539	0	0.28754248	0	0.25916942	0	0	0	0	0	0	0	0	0.26794029	0	0	0	0	0	0	0	0	0	0	0	0
ENSG00000212211	0	0	0	0	0	0	0	0	0	0	0.1879803	0	0	0	0	0	0	0	0	0	0	0	0	0	0	0	0	0	0	0	0	0	0	0.23483808	0	0	0	0	0	0	0.27874333	0	0	0	0	0	0	0
ENSG00000212214	0	0	0	0	0	0	0	0	0	0	0	0	0	0	0	0	0	0	0.26273614	0.2119819	0	0	0	0	0	0	0.20541576	0	0	0	0	0	0	0	0	0	0.22092488	0	0	0	0	0	0	0	0	0	0.42518485	0
ENSG00000212215	0	0	0	0	0	0	0	0	0	0	0	0	0	0	0	0	0	0	0	0	0	0	0	0	0	0	0	0	0	0	0	0	0	0	0	0	0	0	0	0	0	0	0	0	0	0	0	0
ENSG00000212216	0	0	0	0	0	0	0	0	0	0	0	0	0	0	0	0	0	0	0	0	0	0	0	0	0	0	0	0	0	0	0	0	0	0	0	0	0	0	0	0	0	0	0	0	0	0	0	0
ENSG00000212219	0	0	0	0	0	0	0	0	0	0	0	0	0	0	0	0	0	0	0	0	0	0	0	0	0	0	0	0	0	0	0	0	0	0	0	0	0	0	0	0	0	0	0	0	0	0	0	0
ENSG00000212221	0	0	0	0	0	0	0	0	0	0	0	0	0	0	0	0	0	0	0	0	0	0	0	0	0	0	0	0	0	0	0	0	0	0	0	0	0	0	0	0	0	0	0	0	0	0	0	0
ENSG00000212224	0	0	0	0	0	0	0	0	0	0	0	0	0	0	0	0	0	0	0	0	0	0	0	0	0	0	0	0	0	0	0	0	0	0	0	0	0	0	0	0	0	0	0	0	0	0	0	0
ENSG00000212226	0	0	0.30902065	0	0	0	0	0	0.15621672	0.2861883	0	0.26684305	0	0	0	0	0	0	0	0	0	0	0	0	0	0	0	0	0	0	0	0	0	0	0	0	0	0	0	0	0.59028	0	0	0	0	0	0	0
ENSG00000212228	0	0	0	0	0	0	0	0	0	0	0	0	0	0	0	0	0	0	0	0	0	0	0	0	0	0	0	0	0	0	0	0	0	0	0	0	0	0	0	0	0	0	0	0	0	0	0	0
ENSG00000212229	0	0	0	0	0	0	0	0	0	0	0	0	0	0	0	0	0	0	0	0	0	0	0	0	0	0	0	0	0	0	0	0	0	0	0	0	0	0	0	0	0	0	0	0	0	0	0	0
ENSG00000212230	0	0	0	0	0	0	0	0	0	0	0	0	0	0	0	0	0	0	0	0	0	0	0	0	0	0	0	0	0	0	0	0	0	0	0	0	0	0	0	0	0	0	0	0	0	0	0	0
ENSG00000212232	610.59718	322.726796	376.778321	560.92532	517.733413	334.082189	319.549974	563.185421	213.933855	264.198892	624.81797	332.702612	102.672484	502.079523	533.636216	64.9374876	269.225999	360.55452	580.829781	277.602368	284.586463	294.896043	168.202197	271.323444	298.19368	256.958948	480.789892	647.745455	365.619263	357.274839	453.734204	303.312678	165.760617	323.82685	241.279121	331.091662	783.751996	1025.5635	718.450787	471.831812	436.194505	400.826712	619.537114	363.982002	430.891556	367.776491	427.472237	183.335377
ENSG00000212237	0	0	0	0	0	0	0	0	0	0	0	0	0	0	0	0	0	0	0	0	0	0	0	0	0	0	0	0	0	0	0	0	0	0	0	0	0	0	0	0	0	0	0	0	0	0	0	0
ENSG00000212238	0	0	0	0	0	0	0	0	0	0	0	0	0	0	0	0	0	0	0	0	0	0	0	0	0	0	0	0	0	0	0	0	0	0	0	0	0	0	0	0	0	0	0	0	0	0	0	0
ENSG00000212240	0	0	0	0	0	0	0	0	0	0	0	0	0	0	0	0	0	0	0	0	0	0	0	0	0	0	0	0	0	0	0	0	0	0	0	0	0	0	0	0	0	0	0	0	0	0	0	0
ENSG00000212241	0	0.45931895	0	0	0	0	0	0	0.16951176	0.31054475	0	0	0	0	0	0	0	0	0	0	0	0	0	0	0	0	0	0	0.24621455	0	0	0	0	0	0	0	0	0	0	0	0	0	0.39256708	0	0	0	0	0
ENSG00000212242	0	0	0	0	0	0	0	0	0	0	0	0	0	0.30898886	0	0	0	0	0	0	0	0	0	0	0	0	0	0	0	0	0	0	0	0	0	0	0	0	0	0	0	0	0	0	0	0	0	0
ENSG00000212246	0	0	0	0	0	0	0.24887397	0	0.15321255	0.84205403	0	0.26171145	0	0	0.26952013	0	0	0	0	0	0	0	0	0	0	0	0	0	0	0	0	0	0	0	0	0	0	0.31227972	0.6243046	0	0	0	0	0	0	0	0	0
ENSG00000212247	0	0	0	0	0	0	0	0	0	0	0	0	0	0	0	0	0	0	0	0	0	0	0	0	0	0	0	0	0	0	0	0	0	0	0	0	0	0	0	0	0	0	0	0	0	0	0	0
ENSG00000212248	0	0.20175692	0	0	0.16802276	0.225947	0	0	0.14891687	0.27281501	0	0	0	0	0	0	0	0	0	0.53490759	0	0	0	0	0	0	0	0	0	0.24229855	0	0.36852628	0.49414808	0	0	0.26794029	0	0	0	0	0	0	0	0	0.55569413	0	0.26822409	0.28160839
ENSG00000212249	0	0	0	0	0	0	0	0	0	0	0	0	0	0	0	0	0	0	0	0	0	0	0	0	0	0	0	0	0	0	0	0	0	0	0	0	0	0	0	0	0	0	0	0	0	0	0	0
ENSG00000212251	0	0	0	0	0	0	0	0	0	0	0	0	0	0	0	0	0	0	0	0	0	0	0	0	0	0	0	0	0	0	0	0	0	0	0	0	0	0	0	0	0	0	0	0	0	0	0	0
ENSG00000212254	0	0	0.28917529	0	0	0	0	0	0	0	0	0	0	0.3203279	0	0.28524112	0	0	0	0	0	0	0	0.27490298	0.28226647	0	0.25441402	0	0	0	0	0	0	0	0	0	0	0	0	0	0	0	0	0	0.27274895	0	0	0
ENSG00000212257	0	0	0.29458044	0	0	0	0	0	0	0	0	0	0	0	0	0	0	0	0	0.26745379	0	0	0	0	0.28754248	0	0	0	0	0	0	0	0	0	0	0	0	0	0	0	0	0	0	0	0.27784706	0	0	0
ENSG00000212258	0	0	0	0	0	0	0	0	0	0	0	0	0	0	0	0	0	0	0	0	0	0	0	0	0	0	0	0	0	0	0	0	0	0	0	0	0	0	0	0	0	0	0	0	0	0	0	0
ENSG00000212259	0	0	0	0	0	0	0	0	0	0	0	0	0	0	0	0	0	0	0	0	0	0	0	0	0	0	0	0	0	0	0	0	0	0	0	0	0	0	0	0	0	0	0	0	0	0	0	0
ENSG00000212260	0	0	0	0	0	0	0	0	0	0	0	0	0	0	0	0	0	0	0	0	0	0	0	0	0	0	0	0	0	0	0	0	0	0	0	0	0	0	0	0	0	0	0	0	0	0	0	0
ENSG00000212264	0	0	0	0	0	0	0	0	0	0	0	0	0	0	0	0	0	0	0	0	0	0	0	0	0	0	0	0	0	0	0	0	0	0	0	0	0	0	0	0	0	0	0	0	0	0	0	0
ENSG00000212265	0	0	0	0	0	0	0	0	0	0	0	0	0	0	0	0	0	0	0	0	0	0	0	0	0	0	0	0	0	0	0	0	0	0	0	0	0	0	0	0	0	0	0	0	0	0	0	0
ENSG00000212266	0	0	0	0	0	0	0	0	0	0	0	0	0	0	0	0	0	0	0	0	0	0	0	0	0	0	0	0	0	0	0	0	0	0	0	0	0	0	0	0	0	0	0	0	0	0	0	0
ENSG00000212269	0	0	0	0	0	0	0	0	0	0	0	0	0	0	0	0	0	0	0	0	0	0	0	0	0	0	0	0	0	0	0	0	0	0	0	0	0	0	0	0	0	0	0	0	0	0	0	0
ENSG00000212270	0	0	0	0	0	0	0	0	0	0	0	0	0	0	0	0	0	0	0	0	0	0	0	0	0	0	0	0	0	0	0	0	0	0	0	0	0	0	0	0	0	0	0	0	0	0	0	0
ENSG00000212273	0	0	0	0	0	0	0	0	0	0	0	0	0	0	0	0	0	0	0	0	0	0	0	0	0	0	0	0	0	0	0	0	0	0	0	0	0	0	0	0	0	0	0	0	0	0	0	0
ENSG00000212276	0	0	0	0	0	0	0	0	0	0	0	0	0	0	0	0	0	0	0	0	0	0	0	0	0	0	0	0	0	0	0	0	0	0	0	0	0	0	0	0	0	0	0	0	0	0	0	0
ENSG00000212277	0	0	0	0	0	0	0	0	0	0	0	0	0	0	0	0	0	0	0	0	0	0	0	0	0	0	0	0	0	0	0	0	0	0	0	0	0	0	0	0	0	0	0	0	0	0	0	0
ENSG00000212278	0	0	0	0	0	0	0	0	0	0	0	0	0	0	0	0	0	0	0	0	0	0	0	0	0	0	0	0	0	0	0	0	0	0	0	0	0	0	0	0	0	0	0	0	0	0	0	0
ENSG00000212279	0	0	0	0	0	0	0	0	0	0	0	0	0	0	0	0	0	0	0	0	0	0	0	0	0	0	0	0	0	0	0	0	0	0	0	0	0	0	0	0	0	0	0	0	0	0	0	0
ENSG00000212280	0	0	0	0	0	0	0	0	0	0	0	0	0	0	0	0	0	0	0	0	0	0	0	0	0	0	0	0	0	0	0	0	0	0	0	0	0	0	0	0	0	0	0	0	0	0	0	0
ENSG00000212282	0	0	0	0	0	0	0	0	0	0	0	0	0	0	0	0	0	0	0	0	0	0	0	0	0	0	0	0	0	0	0	0	0	0	0	0	0	0	0	0	0	0	0	0	0	0	0	0
ENSG00000212283	4.24075523	2.4617884	3.0414138	7.3088951	1.89246684	7.63463033	2.04338626	2.76923569	2.37613853	2.81669534	11.0413693	2.38754304	4.30730091	6.43184712	5.16343833	2.72730543	3.82778126	10.2307626	4.97815839	4.76959266	3.56983273	3.38047536	2.86391085	4.20553334	11.0653407	14.1927055	10.4599868	8.52701415	4.46641841	6.82261714	1.2886469	0.69179495	1.39141695	0.44495636	2.20244388	0.50297562	14.9124296	13.1047911	19.0796248	3.17888636	2.64072632	5.56881775	4.85543498	4.34468569	8.08437463	6.60131817	7.30087145	2.90748307
ENSG00000212289	0	0	0	0	0	0	0	0	0	0	0	0	0	0	0	0	0	0	0	0	0	0	0	0	0	0	0	0	0	0	0	0	0	0	0	0	0	0	0	0	0	0	0	0	0	0	0	0
ENSG00000212290	0	0.37544331	0	0	0	0	0	0	0	0	0	0	0	0.30361514	0	0	0	0	0	0	0	0.23936285	0	0	0	0	0	0	0	0.225443	0	0	0	0	0	0	0	0	0	0	0	0	0	0	0	0	0	0
ENSG00000212292	0	0	0	0	0	0	0	0	0	0	0	0	0	0	0	0	0	0	0	0	0	0	0	0	0	0	0	0	0	0	0	0	0	0	0	0	0	0	0	0	0	0	0	0	0	0	0	0
ENSG00000212293	0	0	0	0.328037	0	0	0.2038023	0	0.1254654	0.22985202	0.31971453	0	0	0	0.2207094	0	0	0	0.27928645	0.45067017	0	0.43349178	0.25707546	0.23594035	0	0	0.21835534	0.19135425	0.18223754	0.4082826	0	0	0.83265896	0	0	0	0	0	0.51124156	0	0	0	0	0	0	0	0.22598407	0
ENSG00000212295	0	0	0	0	0	0	0	0	0	0	0	0	0	0	0	0	0	0	0	0	0	0	0	0	0	0	0	0	0	0	0	0	0	0	0	0	0	0	0	0	0	0	0	0	0	0	0	0
ENSG00000212296	0	0	0.78800267	1.0415176	0.44946088	0.90661235	7.76486779	6.70847345	4.18270268	4.74357102	5.58301497	5.10337325	3.40994656	1.30934031	3.50376172	4.27505126	4.24245757	0.97192244	3.10357062	3.21947505	3.7304752	5.50534558	3.67296567	5.61832969	2.69211643	3.31550907	6.23950377	1.21509952	3.76092732	1.29629726	1.22421455	0.9858078	5.28738441	2.53625127	3.13848253	1.79185066	1.49124296	2.43578183	3.65218191	3.88278262	5.268249	3.60707513	3.69013058	2.40767999	3.71620446	2.76672894	2.51124802	2.63655851
ENSG00000212297	0	0	0	0	0	0	0	0	0	0	0	0	0	0	0.52392699	0	0.45313298	0.36333549	0	0	0	0	0	0	0	0	0	0	0	0	0	0	0	0	0	0	0	0	0	0	0	0	0	0	0.27784706	0.41371648	0.26822409	0.28160839
ENSG00000212298	0	0	0	0	0	0	0	0	0	0	0	0	0	0	0	0	0	0	0	0	0	0	0	0	0	0	0	0	0	0	0	0	0	0	0	0	0	0	0	0	0	0	0	0	0	0	0	0
ENSG00000212302	0	1.25147771	0	0	0	0	0.37511439	0.4575259	0	0	0	0.39446363	0	0	0	0	0	0	0	0	0.39319897	0	0.47316788	0	0.4458992	0	0.4019004	0	0.67084545	0.37573834	0	0	0	0	0	0	0	0	0	0.75029616	0	0	0	0	0	0	0	0
ENSG00000212303	0	0	0	0	0	0	0	0	0	0	0	0	0	0	0	0	0	0	0	0	0	0	0	0	0	0	0	0	0	0	0	0	0	0	0	0	0	0	0	0	0	0	0	0	0	0	0	0
ENSG00000212304	0.71621644	2.63853218	1.40089363	3.2402768	1.19856233	3.49213647	24.7325419	26.307739	26.9109337	21.7312313	27.0691635	21.4719704	7.88076538	4.26747952	7.16324619	5.18188032	8.61959632	3.02375871	11.0349178	6.04148404	20.1973203	11.6223962	7.25524082	11.3198939	16.4090906	24.755801	20.6442841	9.45077402	10.8006118	10.3703781	4.89685821	2.6288208	4.1124101	0.56361139	3.48720281	4.14116596	26.8423733	18.4036849	33.5459672	21.283401	30.773264	23.0852808	9.0203192	8.25490281	7.59757357	13.2802989	6.05888412	6.02641946
ENSG00000212305	0	0	0	0	0	0	0	0	0	0	0	0	0	0	0	0	0	0	0	0	0	0	0	0	0	0	0	0	0	0	0	0	0	0	0	0	0	0	0	0	0	0	0	0	0	0	0	0
ENSG00000212306	0.33926042	0.90896802	0	0	0	0.25448768	0	0.99692485	0	0.30727586	0	0.28650516	0	0	0	0	1.02074167	0.4092305	0.37336188	0.60247486	0.28558662	0.57951006	0	0.63083	0	0.55840153	0.87571983	0.25581042	0	1.09161874	0	0.41507697	0.55656678	0.53394764	0	0	0	0	0	0	0	0.60750739	0	0.28964571	0	0	0	0
ENSG00000212308	0	0	0	0	0	0	0	0	0	0	0	0	0	0	0	0	0	0	0	0	0	0	0	0	0	0	0	0	0	0	0	0	0	0	0	0	0	0	0	0	0	0	0	0	0	0	0	0
ENSG00000212309	0.37917341	0.76192908	0	0	0.423022	0.2844274	1.5225231	0.74280675	0.56238019	0.68685191	0.47769112	0.64042331	0.96280844	0.41077343	1.31906324	0.36577979	1.71124339	0	0	0	1.27674018	0.32384386	3.45690886	0	0	0.31204791	0.65249713	0.57181154	0	0.61002224	2.30440386	0.46390955	0	0	0	0.33728954	0.3508807	0.76416685	1.14578256	0	0.708336	0	0.43413301	0	0	1.56238811	0.67529359	0
ENSG00000212312	0	0	0	0	0	0	0	0	0	0	0	0	0	0	0	0	0	0	0	0	0	0	0	0	0	0	0	0	0	0	0	0	0	0	0	0	0	0	0	0	0	0	0	0	0	0	0	0
ENSG00000212314	0	0	0	0	0	0	0	0	0	0	0	0	0	0	0	0	0	0	0	0	0	0	0	0	0	0	0	0	0	0	0	0	0	0	0	0	0	0	0	0	0	0	0	0	0	0	0	0
ENSG00000212316	0	0	0	0	0	0	0	0	0	0	0	0	0	0	0	0	0	0	0	0	0	0	0	0	0	0	0	0	0	0	0	0	0	0	0	0	0	0	0	0	0	0	0	0.25716208	0	0.41371648	0	0.28160839
ENSG00000212319	0	0	0	0	0	0	0	0	0	0	0	0	0	0	0	0	0	0	0	0	0	0	0	0	0	0	0	0	0	0	0	0	0	0	0	0	0	0	0	0	0	0	0	0	0	0	0	0
ENSG00000212321	0	0	0	0	0	0	0	0	0	0	0	0	0	0	0	0	0	0	0	0	0	0	0	0	0	0	0	0	0	0	0	0	0	0	0	0	0	0	0	0	0	0	0	0	0	0	0	0
ENSG00000212324	0	0	0	0	0	0	0	0	0	0	0	0	0	0	0	0	0	0	0	0	0	0	0	0	0	0	0	0	0	0	0	0	0	0	0	0	0	0	0	0	0	0	0	0	0	0	0	0
ENSG00000212325	0	0	0	0	0	0	0	0	0	0	0	0	0	0	0	0	0	0	0	0	0	0	0	0	0	0	0	0	0	0	0	0	0	0	0	0	0	0	0	0	0	0	0	0	0	0	0	0
ENSG00000212327	0	0	0	0	0	0	0	0	0	0	0	0	0	0	0	0	0	0	0	0	0	0	0	0	0	0	0	0	0	0	0	0	0	0	0	0	0	0	0	0	0	0	0	0	0	0	0	0
ENSG00000212329	0	0	0	0	0	0	0	0.29504006	0	0	0	0	0	0	0	0	0	0	0	0	0	0	0	0	0	0	0	0	0	0	0	0	0	0	0	0	0	0	0	0	0	0	0.34487202	0	0	0	0.26822409	0
ENSG00000212330	0	0	0	0	0	0	0	0	0	0	0	0	0	0	0	0	0	0	0	0	0	0	0	0	0	0	0	0	0	0	0	0	0	0	0	0	0	0	0	0	0	0	0	0	0	0	0	0
ENSG00000212331	0	0	0	0	0	0	0	0	0	0	0	0	0	0	0	0	0	0	0	0	0	0	0	0	0	0	0	0	0	0	0	0	0	0	0	0	0	0	0	0	0	0	0	0	0	0	0	0
ENSG00000212332	0	0	0	0	0	0	0	0	0	0	0	0	0	0	0	0	0	0	0	0	0	0	0	0	0	0	0	0	0	0	0	0	0	0	0	0	0	0	0	0	0	0	0	0	0	0	0	0
ENSG00000212333	0	0.20366029	0	0	0.16960788	0	0	0	0	0	0	0	0	0	0	0	0	0	0	0	0	0	0	0.28268326	0	0	0	0	0	0	0	0	0.49880985	0	0	0	0	0	0	0	0	0	0	0	0	0	0	0
ENSG00000212335	0	0	0	0	0	0	0	0	0	0	0	0	0	0	0	0.27514409	0	0	0	0.25325271	0	0	0.28892552	0	0	0	0	0	0	0.22943314	0	0	0	0	0	0	0	0	0	0	0	0	0	0	0	0	0	0
ENSG00000212336	0	0	0	0	0	0	0	0	0	0	0	0	0	0	0	0	0	0	0	0	0	0	0	0	0	0	0	0	0	0	0	0	0	0	0	0	0	0	0	0	0	0	0	0	0	0	0	0
ENSG00000212338	0	0	0	0	0	0	0	0	0	0	0	0	0	0	0	0	0	0	0	0	0	0	0	0	0	0	0	0	0	0	0	0	0	0	0	0	0	0	0	0	0	0	0	0	0	0	0	0
ENSG00000212340	0	0	0	0	0	0	0	0	0	0	0	0	0	0	0	0	0	0	0	0	0	0	0	0	0	0	0	0	0	0	0	0	0	0	0	0	0	0	0	0	0	0	0	0	0	0	0	0
ENSG00000212342	0	0	0	0	0	0	0	0	0	0	0	0	0	0	0	0	0	0	0	0	0	0	0	0	0	0	0	0	0	0	0	0	0	0	0	0	0	0	0	0	0	0	0	0	0	0	0	0
ENSG00000212344	0	0	0	0	0	0	0	0	0.15470005	0.28340977	0.39421112	0	0	0	0	0	0	0	0	0	0	0	0	0	0	0	0	0	0	0	0	0	0	0	0	0	0	0	0	0	0.58454913	1.1206447	0	0	0	0	0	0
ENSG00000212345	0	0	0	0	0	0	0	0	0	0	0	0	0	0	0	0	0	0	0	0	0	0	0	0	0	0	0	0	0	0	0	0	0	0	0	0	0	0	0	0	0	0	0	0	0	0	0	0
ENSG00000212347	0	0	0	0	0	0	0	0	0	0	0	0	0	0.51346679	0	0	0	0	0	0	0	0	0	0	0	0	0	0	0	0	0	0	0	0	0	0	0	0	0	0	0	0	0	0	0	0	0	0
ENSG00000212348	0	0	0	0	0	0	0	0	0	0	0	0	0	0	0	0	0	0.35997128	0	0	0	0	0	0	0	0	0.2567697	0	0	0.24005505	0.4534128	0	0.46967616	0	0	0	0	0	0	0	0	0	0	0.25478095	0	0	0	0
ENSG00000212354	0	0	0	0	0	0	0	0.30649793	0.15470005	0	0	0	0	0	0	0	0	0	0	0	0	0	0	0	0.29870917	0	0.26923425	0	0	0	0	0	0	0	0	0	0	0	0	0	0	0	0	0	0	0	0	0
ENSG00000212358	0	0	0	0	0	0	0	0	0	0	0	0	0	0	0	0	0	0	0	0	0	0	0	0	0	0	0	0	0	0	0	0	0	0	0	0	0	0	0	0	0	0	0	0	0	0	0	0
ENSG00000212359	0	0	0	0	0	0	0	0	0	0	0	0	0	0	0	0	0	0	0	0	0	0	0	0	0	0	0	0	0	0	0	0	0	0	0	0	0	0	0	0	0	0	0	0	0	0	0	0
ENSG00000212360	0	0.20757683	0	0	0	0	0	0	0	0	0	0	0	0	0	0	0	0	0	0	0	0	0	0	0	0	0	0	0	0	0.47085175	0	0	0	0	0	0	0	0	0	0	0	0.7096405	0	0	0	0	0
ENSG00000212363	0	0	0	0	0	0	0	0	0	0	0	0	0	0	0	0	0	0	0	0	0	0	0	0	0	0	0	0	0	0	0	0	0	0	0	0	0	0	0	0	0	0	0	0	0	0	0	0
ENSG00000212365	0	0	0	0	0	0	0	0	0	0	0	0	0	0	0	0	0	0	0	0	0	0	0	0	0	0	0	0	0	0	0	0	0	0	0	0	0	0	0	0	0	0	0	0	0	0	0	0
ENSG00000212366	0	0	0	0	0	0	0	0	0	0	0	0	0	0	0	0	0	0	0	0	0	0	0	0	0	0	0	0	0	0	0	0	0	0	0	0	0	0	0	0	0	0	0	0	0	0	0	0
ENSG00000212368	0	0	0	0	0	0	0	0	0	0	0	0	0	0	0	0	0	0	0	0	0	0	0	0	0	0	0	0	0	0	0	0	0	0	0	0	0	0	0	0	0	0	0	0	0	0	0	0
ENSG00000212370	0	1.00878461	0.58916087	0	0.33604551	0.45189401	0	0	0	0	0	0	0.25494928	0	0	0	0	0	0	0.26745379	0	0	0.6102539	0.28004136	0	0	0.22712141	0	0	0	0	0	0	0	0	0	0.30352422	0.30340037	0	0	0	0	0	0	0.27784706	0.41371648	0	0.28160839
ENSG00000212371	0.63195568	0.28219596	0	0	0	0.47404567	1.18418463	0.61900562	1.14558928	1.52633758	1.06153582	1.06737218	2.13957431	0.22820746	0.91601614	1.21926596	1.90138154	0.76229211	0.46365201	1.1222571	0.88662512	0.35982651	0.64016831	0.58753775	1.40764258	1.38687961	1.26874441	0.47650961	0.75634536	0.67780249	0	0.77318259	0	0	0	0.37476615	3.70374068	1.27361141	2.54618347	1.01510657	0.78704	0	0.72355502	0.17984538	0.19431134	0.57866226	0.75032621	1.18165087
ENSG00000212373	0	0	0	0	0	0	0	0	0	0	0	0	0	0	0	0	0	0	0	0	0	0	0	0	0	0	0	0	0	0	0	0	0	0	0	0	0	0	0	0	0	0	0	0	0	0	0	0
ENSG00000212374	0	0	0	0	0	0	0	0	0	0	0	0	0	0	0	0	0	0	0	0	0	0	0	0	0	0	0	0	0	0	0	0	0	0	0	0	0	0	0	0	0	0	0	0	0	0	0	0
ENSG00000212377	0	0	0	0	0	0	0	0	0	0	0	0	0	0	0	0	0	0	0	0	0	0	0	0	0	0	0	0	0	0	0	0	0	0	0	0	0	0	0	0	0	0	0	0	0	0	0	0
ENSG00000212378	0	0	0	0	0	0	0	0	0	0	0	0	0	0	0	0	0	0	0	0	0	0	0	0	0	0	0	0	0	0	0	0	0	0	0	0	0	0	0	0	0	0	0	0	0	0	0	0
ENSG00000212379	0	0	0	0	0	0	0	0	0	0	0	0	0	0	0	0	0	0	0	0	0	0	0	0	0	0	0	0	0	0	0	0	0	0	0	0	0	0	0	0	0	0	0	0	0	0	0	0
ENSG00000212380	0	0	0.38913712	0	0	0	0	0	0	0	0	0	0	0	0	0	0	0	0	0	0	0	0	0	0	0	0	0	0	0	0	0	0	0	0	0	0	0	0	0	0	0	0	0	0	0	0	0
ENSG00000212382	0	0	0	0	0	0	0	0	0	0	0	0	0	0	0	0	0	0	0	0	0	0	0	0	0	0	0	0	0	0	0	0	0	0	0	0	0	0	0	0	0	0	0	0	0	0	0	0
ENSG00000212383	0	0	0	0	0	0	0	0	0	0	0	0	0	0	0	0	0	0	0	0	0	0	0	0	0	0	0	0	0	0	0	0	0	0	0	0	0	0	0	0	0	0	0	0	0	0	0	0
ENSG00000212384	0	1.49916601	0	0.5786209	0	0.67156471	0	0	0	0	0	0	1.5155318	1.93976342	1.16792057	1.29547008	2.02021789	2.15982765	3.94104206	1.98733028	2.26089406	0.76463133	6.34833572	0.83234514	0.42732007	0.3683899	3.46639098	0.33752764	0.96434034	0.72016514	0	0	0	0	0	0	0	0	0	0	0	0	0.51251814	0	0	0	0.3986108	0
ENSG00000212385	1.23960538	0.20757683	0.6061559	0.8011674	0.69147827	0.69739412	0.49774794	1.21420334	1.22570042	0.28068468	0.39042063	0.78513435	0.52460716	0.33572828	0.26952013	0.59790927	0.46620413	1.12144897	0.68210343	0	0.26087239	0.52936015	0.62785738	0.28811947	0	1.02015664	0.26664546	0.46734597	1.11270039	0.49857587	2.82511051	1.13747054	1.01680469	1.46322189	1.20710866	2.48102399	2.29421994	0.62455944	2.4972184	0.99558529	2.89464231	0	1.06446075	0.79374066	0.57172376	0.85130121	0.27596132	1.73839023
ENSG00000212387	0	0	0	0	0	0	0	0	0	0	0	0	0	0	0	0	0	0	0	0	0	0	0	0	0	0	0	0	0	0	0	0	0	0	0	0	0	0	0	0	0	0	0	0	0	0	0	0
ENSG00000212388	0	0	0	0	0	0	0	0	0	0	0	0	0	0	0	0	0	0	0	0	0	0	0	0	0	0	0	0	0	0	0	0	0	0	0	0	0	0	0	0	0	0	0	0	0	0	0	0
ENSG00000212389	0	1.61405537	0.58916087	1.1680571	0.67209103	1.12973502	0	0.29504006	0.29783375	0	0.75894851	0.25437374	0.25494928	0	0.26196349	0	0	0	0.33148952	0	0	0.25725914	0.30512695	0.28004136	0.28754248	0.24788853	0.51833884	0	0	0	0	0	0	0	1.1732645	0.26794029	0.278737	0	0.60680073	0	0	0	0	0	0	0	0	0
ENSG00000212391	0	0	0	0	0	0	0	0	0	0	0	0	0	0	0	0	0	0	0	0	0	0	0	0	0	0	0	0	0	0	0.73087436	0.88281295	0.39458093	0	0.93686046	0.85580927	0	0	0	0	0	0	0	0.41069168	0	0	0.21417894	0
ENSG00000212392	0	0	0	0	0	0	0	0	0	0	0	0	0	0	0	0	0	0	0	0	0	0	0	0	0	0	0	0	0	0	0	0	0	0	0	0	0	0	0	0	0	0	0	0	0	0	0	0
ENSG00000212395	0	0	0	0	0	0	0	0	0	0	0	0	0	0	0	0	0	0	0	0	0	0	0	0	0	0	0	0	0	0	0	0	0	0	0	0	0	0	0	0	0	0	0	0	0	0	0	0
ENSG00000212396	0	0	0	0	0	0	0	0	0	0	0	0	0	0	0	0	0	0	0	0	0	0	0	0	0	0	0	0	0	0	0	0	0	0	0	0	0	0	0	0	0	0	0	0	0	0	0	0
ENSG00000212397	0	0	0	0	0	0	0	0	0	0	0	0	0	0	0	0	0	0	0	0	0	0	0	0	0	0	0	0	0	0	0	0	0	0	0	0	0	0	0	0	0	0	0	0	0	0	0	0
ENSG00000212398	0	0	0	0	0	0	0	0	0	0	0	0	0	0	0	0	0	0	0	0	0	0	0	0	0	0	0	0	0	0	0	0	0	0	0	0	0	0	0	0	0	0	0	0	0	0	0	0
ENSG00000212402	1.28277571	0.42961175	0.47044935	0.4145344	0.5366697	0.84196172	0.77262366	1.25648903	0.63419325	0.43568965	2.62611287	0.81247733	0.67859633	1.2159711	1.95234484	0.30936599	1.44732028	0.3868348	1.7646457	0.8542554	1.3497875	0.82169337	0.16243076	0.74538371	1.68376863	0.6598028	0.96576067	1.69267594	0.8060158	1.03187841	1.94899829	0.78472263	0.78916185	1.00945324	0.62457364	1.2837139	1.63220622	1.93893081	1.2920931	1.28782177	0.89863522	0.57426072	0.73435434	0.95828059	0.73954318	2.64284555	0.9995017	4.19750609
ENSG00000212404	0	0	0	0	0	0	0	0	0	0	0	0	0	0	0	0	0	0	0	0	0	0	0	0	0	0	0	0	0	0	0	0	0	0	0	0	0	0	0	0	0	0	0	0	0	0	0	0
ENSG00000212407	0	0	0	0	0	0	0	0	0	0	0	0	0	0	0	0	0	0	0	0	0	0	0	0	0	0	0	0	0	0	0	0	0	0	0	0	0	0	0	0	0	0	0	0	0	0	0	0
ENSG00000212409	0	0	0	0	0	0	0	0	0	0	0	0	0	0	0	0	0	0	0	0	0	0	0	0	0	0	0	0	0	0	0	0	0	0	0	0	0	0	0	0	0	0	0	0	0	0	0	0
ENSG00000212410	0	0	0	0	0	0	0	0	0	0	0	0	0	0	0	0	0	0	0	0	0	0	0	0	0	0	0	0	0	0	0	0	0	0	0	0	0	0	0	0	0	0	0	0	0	0	0	0
ENSG00000212411	0	0	0	0	0	0	0	0	0	0	0	0	0	0	0	0	0	0	0	0	0	0	0	0	0	0	0	0	0	0	0	0	0	0	0	0	0	0	0	0	0	0	0	0	0	0	0	0
ENSG00000212413	0	0.64926287	0	0	0.1351762	0.54533074	0.38921643	0	0.47922122	0.21948275	0.30529132	0.40929309	0.20510957	0.26252437	0.21075258	0.70130711	0	0.2923075	0	0	0	0	0	0.22529643	0	0.19942912	0.41700944	0.18272173	0.3480326	0.19493192	0	0	0.3975477	0.38139117	0	0.21556098	0	0.24418865	0.24408902	0	0.45269594	0.43393385	0	0	0.2235311	0	0	0.22655712
ENSG00000212414	0	0	0	0	0	0	0	0	0	0	0	0	0	0	0	0	0	0	0	0	0	0	0	0	0	0	0	0	0	0	0	0	0	0	0	0	0	0	0	0	0	0	0	0	0	0	0	0
ENSG00000212415	0	0	0	0	0	0	0	0	0	0	0	0	0	0	0	0	0	0	0	0	0	0	0	0	0	0	0	0	0	0	0	0	0	0	0	0	0	0	0	0	0	0	0	0	0	0	0	0
ENSG00000212418	0.33572646	0	0.32833444	0	0	0.25183676	0.53922693	0.32884674	0.33196053	0.30407507	0.42295568	0.28352074	0.28416221	0	0.58396029	0.64773504	1.51516342	0.40496768	0	0	0	0	0.68017883	0.62425885	0	0	0.28886592	0.25314573	0.72325525	0.54012386	0.5100894	0.41075325	0.55076921	0	0	0	0	0.33830303	0	1.61782609	0	0.60117919	0.3843886	0.57325714	0.30968371	0.92224298	0.5979162	0.31387601
ENSG00000212420	0	0	0	0.7787047	0.16802276	0.225947	0	0.29504006	0.14891687	0	0	0.25437374	0.25494928	0.32631534	1.04785397	0.29057273	0.45313298	0	0.33148952	0.53490759	0.50711643	0.51451828	0.6102539	0	0	0.24788853	0	0	0.64890191	0	0	0	0	0	0	0	0	0	0	0.48383584	0.56269682	0.53937572	0	0	0	0	0	0.28160839
ENSG00000212421	0	0	0	0	0	0	0	0	0	0	0	0	0	0	0	0	0	0	0	0	0	0	0	0	0	0	0.20390535	0	0	0	0	0	0	0	0	0	0	0	0	0	0	0	0	0	0	0	0	0
ENSG00000212422	0	0	0	0	0	0	0	0	0	0	0	0	0	0	0	0	0	0	0	0	0	0	0	0	0	0	0	0	0	0	0	0	0	0	0	0	0	0	0	0	0	0	0	0	0	0	0	0
ENSG00000212424	0	0	0	0	0	0	0	0	0	0	0	0	0	0	0	0	0	0	0	0	0	0	0	0	0	0	0	0	0	0	0	0	0	0	0	0	0	0	0	0	0	0	0	0	0	0	0	0
ENSG00000212425	0	0	0	0	0	0	0	0	0	0	0	0	0	0	0	0	0	0	0	0	0	0	0	0	0	0	0	0	0	0	0	0	0	0	0	0	0	0	0	0	0	0	0	0	0	0	0	0
ENSG00000212428	0	0	0	0	0	0	0	0	0	0	0	0	0	0	0	0	0	0	0	0	0	0	0	0	0	0	0	0	0	0	0	0	0	0	0	0	0	0	0	0	0	0	0	0	0	0	0	0
ENSG00000212429	0	0.81157859	0	0	0.18177691	0	0	0	0	0.21948275	0	0	0	0	0	0	0	0	0	0	0	0	0	0	0	0	0	0	0	0	0	0	0	0	0	0	0	0	0	0	0	0	0	0	0	0	0	0
ENSG00000212432	0	0	0	0	0	0	0	0	0	0	0	0	0	0	0	0	0	0	0	0	0	0	0	0	0	0	0	0	0	0	0	0	0	0	0	0	0	0	0	0	0	0	0	0	0	0	0	0
ENSG00000212433	0	0	0	0	0	0	0	0	0	0	0	0	0	0	0	0	0	0	0	0	0	0	0	0	0	0	0	0	0	0	0	0	0	0	0	0	0	0	0	0	0	0	0	0	0	0	0	0
ENSG00000212434	0	0	0	0	0.12148909	0	0	0	0	0	0	0	0	0	0	0	0	0	0	0	0	0	0	0	0	0	0	0	0	0	0	0	0	0	0	0	0	0	0	0	0	0	0	0	0	0	0	0
ENSG00000212440	0	0	0	0	0	0	0	0	0	0	0	0	0	0	0	0	0	0	0	0	0	0	0	0	0	0	0	0	0	0	0	0	0	0	0	0	0.19883239	0	0	0	0	0	0	0	0	0	0	0
ENSG00000212441	0	0	0	0	0	0	0	0	0	0	0	0	0	0	0	0	0	0	0	0	0	0	0	0	0	0	0	0	0	0	0	0	0	0	0	0	0	0	0	0	0	0	0	0	0	0	0	0
ENSG00000212442	0	0	0	0	0	0	0	0	0	0	0	0	0	0	0	0	0	0	0	0	0	0	0	0	0	0	0	0	0	0	0	0	0	0	0	0	0	0	0	0	0	0	0	0	0	0	0	0
ENSG00000212443	1.29436706	0.95368633	0.75952064	1.1711844	1.66065866	2.524436	1.76710512	1.39462713	0.7679087	1.52403888	1.46760525	0.4372368	0.87645213	1.26201475	0.7879946	0.37459376	0.97359898	0.7806606	0.99713112	0.80450961	0.76271125	0.77384376	1.83566334	1.56440773	1.23562429	0.95870142	1.78191986	1.75677039	0.74358773	0.52060131	1.96660972	1.26690159	1.27406853	0.40742992	1.0083478	1.49680697	1.19778551	1.43473093	1.17339178	1.66330715	1.20900723	0.92711971	1.9265742	1.65761101	0.83577289	0.71112712	1.38313144	2.66227366
ENSG00000212445	0	0.31982208	0.23348227	0	0	0	0	0	0.23606082	0	0	0	0.20207091	0	0.20763032	0.46061158	0	0.28797702	0	0	0.20096836	0.20390169	0	0	0	0	0.20541576	0.18001474	0.17143828	0.19204404	0	0	0	0	0	0	0	0	0	0	0	0.4275052	0	0	0	0.32790861	0	0
ENSG00000212446	0	0	0	0	0	0	0	0	0.14891687	0	0	0	0	0	0	0	0.45313298	0	0	0.26745379	0	0	0	0	0	0	0	0	0	0	0	0	0	0	0	0	0	0	0	0	0	0	0	0	0	0	0.26822409	0
ENSG00000212447	0	0.1944864	0.56792985	0	0	0.21780477	0	0	0	0.26298384	0	0	0	0.62911246	0	0	0	0.35024232	0	0	0	0.74396562	0.29413138	0	0.27718058	0.23895561	0	0.21893685	0	0.23356707	0	0	0	0.45698221	0	0	0	0	0.29246702	0.93280063	0	0	0.66488839	0.74368494	0	0	0	0.27146034
ENSG00000212448	0	0	0	0	0	0	0	0	0	0	0	0	0	0	0	0	0	0	0	0	0	0	0	0	0	0	0	0	0	0	0	0	0	0	0	0	0	0	0	0	0	0	0	0	0	0	0	0
ENSG00000212450	0	0	0.30307795	0	0	0	0	0	0	0	0	0	0	0	0	0	0	0	0	0	0	0	0	0	0	0	0	0	0.22254008	0	0	0	0	0	0	0	0	0	0	0	0	0	0	0	0	0	0	0
ENSG00000212451	0	0	0	0	0	0	0	0	0	0	0	0	0	0	0	0	0	0	0	0	0	0	0	0	0	0	0	0	0	0	0	0	0	0	0	0	0	0	0	0	0	0	0	0	0	0	0	0
ENSG00000212452	2.92997634	1.12145406	0.40935203	1.0820962	0.70045851	3.76773964	4.36983902	4.09990738	5.79422017	4.92838547	4.21857093	5.30220597	3.54280162	0.45345119	1.45610877	5.65296035	6.92646133	4.03915821	2.3032064	1.11496971	7.39928966	1.78744986	4.24007581	3.50233539	0.39957201	4.1336217	2.52101162	1.26244106	1.20229445	2.69360469	1.27191122	0	2.7466932	0.65876656	0.81519027	0.3723326	3.87335834	2.53068242	6.74573282	3.36171656	3.90964676	2.99808842	2.39618869	2.8588408	1.54439666	5.74904714	2.23636188	5.86988909
ENSG00000212454	0	0	0	0	0	0	0	0	0	0	0	0	0	0	0	0	0	0	0	0	0	0	0	0	0	0	0	0	0	0	0	0	0	0	0	0	0	0	0	0	0	0	0	0	0	0	0	0
ENSG00000212455	0	0	0	0	0	0	0	0	0	0	0	0	0	0	0	0	0	0	0	0	0	0	0	0	0	0	0	0	0	0	0.38256705	0	0	0	0	0	0	0	0	0	0	0	0	0	0	0	0	0
ENSG00000212456	0	0	0	0	0.10701449	0	0	0	0.18969173	0	0.24168896	0.16201185	3.89708178	6.65061743	7.00752345	2.59094016	5.194846	4.85961222	7.60058111	0.68137038	5.49074272	3.76854013	0.58301042	3.03211444	2.19764606	3.9470346	1.48559614	2.60378468	2.4797323	2.16049543	1.45739828	0.46943228	1.88835158	0.60386935	0.37362887	4.43696353	0.35505785	0.19331602	0.38647428	0	0.35838429	0	4.3930126	3.9309061	8.49418163	10.2764218	2.56249798	0.89678861
ENSG00000212457	0	0	0	0	0	0	0	0	0	0	0	0	0	0	0	0	0	0	0	0	0	0	0	0	0	0	0	0	0	0	0	0	0	0	0	0	0	0	0	0	0	0	0	0	0	0	0	0
ENSG00000212458	0	0	0	0	0	0	0	0	0	0	0	0	0	0	0	0	0	0	0	0	0	0	0	0	0	0	0	0	0	0	0	0	0	0	0	0	0	0	0	0	0	0	0	0	0	0	0	0
ENSG00000212459	0	0	0	0	0	0	0	0	0	0	0	0	0	0	0	0	0	0	0	0	0	0	0	0	0	0	0	0	0	0	0	0	0	0	0	0	0	0	0	0	0	0	0	0	0	0	0	0
ENSG00000212460	0	0	0	0	0	0	0	0	0	0	0	0	0	0	0	0	0	0	0	0	0	0	0	0	0	0	0	0	0	0	0	0	0	0	0	0	0	0	0	0	0	0	0	0	0	0	0	0
ENSG00000212461	0	0	0	0	0	0	0	0	0	0	0	0	0	0	0	0	0	0	0	0	0	0	0	0	0	0	0	0	0	0	0	0	0	0	0	0	0	0	0	0	0	0	0	0	0	0	0	0
ENSG00000212464	45.6039855	50.2251209	40.9546964	68.017473	37.7914042	62.8255634	35.2148199	40.159569	25.7980755	27.8011488	59.1102141	32.032057	18.371957	29.9277784	38.7082247	22.2080585	44.8570829	44.1662716	39.0887029	34.2631963	25.2851009	30.8973476	28.4286987	28.9452269	36.6274344	43.1241724	47.9163706	43.1484318	32.433324	38.2716333	85.2786192	55.5271331	49.996356	63.4925489	59.3536152	75.0870752	53.9687928	58.547137	53.2230289	69.0272466	51.1977551	76.1657225	39.9136573	36.5012709	49.5493929	59.0235507	51.9332925	22.3428477
ENSG00000212466	0	0	0	0	0	0	0	0	0	0	0	0	0	0	0	0	0	0	0	0	0	0	0	0	0	0	0	0	0	0	0	0	0	0	0	0	0	0	0	0	0	0	0	0	0	0	0	0
ENSG00000212468	0	0	0	0	0	0	0	0	0	0	0	0	0	0	0	0	0	0	0	0	0	0	0	0	0	0	0	0	0	0	0	0	0	0	0	0	0	0	0	0	0	0	0	0	0	0	0	0
ENSG00000212469	0	0	0	0	0	0	0	0	0	0	0	0	0	0	0	0	0	0	0.33148952	0	0	0	0	0	0	0	0	0	0	0.24229855	0	0	0	0.47406566	0	0	0	0.30352422	0	0	0	0	0	0	0	0	0	0
ENSG00000212473	0	0	0	0	0	0	0	0	0	0	0	0	0	0	0	0	0	0	0	0	0	0	0	0	0	0	0	0	0	0	0	0	0	0	0	0	0	0	0	0	0	0	0	0	0	0	0	0
ENSG00000212475	0	0	0	0	0	0	0	0	0	0	0	0	0	0	0	0	0	0	0	0	0	0	0	0	0	0	0	0	0	0	0	0	0	0	0	0	0	0	0	0	0	0	0	0	0	0	0	0
ENSG00000212479	0	0	0	0	0	0	0	0	0	0	0	0	0	0	0	0	0	0	0	0	0	0	0	0	0	0	0.12958471	0	0	0	0	0	0	0	0	0	0	0	0	0	0	0	0	0	0	0	0	0
ENSG00000212482	0.30121252	0.40351384	0.58916087	0.3893524	0.33604551	0	0	0	0	0	0	0	0.25494928	0	0	0	0	0.36333549	0	0	0	0.25725914	0	0	0	0	0	0	0	0.24229855	0	0	0	0	0.58663225	0	0	0.30352422	0.30340037	0	0	0	0	0	0	0	0	0.28160839
ENSG00000212485	0	0	0	0	0	0	0	0	0	0	0	0	0	0	0	0.29057273	0	0	0	0	0	0	0	0	0	0	0	0	0	0	0	0	0	0	0	0	0	0	0	0	0	0	0	0	0	0	0	0
ENSG00000212489	0	0	0	0	0	0	0	0	0	0	0	0	0	0	0	0	0	0	0	0	0	0	0	0	0	0	0	0	0	0	0	0	0	0	0	0	0	0	0	0	0	0	0	0	0	0	0	0
ENSG00000212490	0	0	0	0	0	0	0	0	0	0	0	0	0	0	0	0	0	0	0	0	0	0	0	0	0	0	0	0	0	0	0	0	0	0	0	0	0	0	0	0	0	0	0	0	0	0	0	0
ENSG00000212493	0.94793352	2.85723405	1.39059294	3.0632869	1.32194375	4.26641107	9.51576935	8.35657593	7.26407749	7.29780156	9.5538224	8.80582051	4.01170183	3.08080072	5.3586944	4.11502261	1.42603616	3.43031451	3.65125955	6.31269617	6.38370088	2.83363375	5.76151477	5.72849302	2.26228271	3.12047912	6.93278196	3.57382211	3.06319872	2.66884729	0.72012621	1.73966082	4.66533919	2.23786877	0.9230831	1.68644768	4.82460957	0.95520856	7.161141	6.09063941	7.08336001	6.78978848	2.71333131	1.2139563	4.37200525	1.30199009	1.68823397	11.521096
ENSG00000212495	0	0	0	0	0	0	0	0	0	0	0	0	0	0	0	0	0	0	0	0	0	0	0	0	0	0	0	0	0	0	0	0	0	0	0	0	0	0	0	0	0	0	0	0	0	0	0	0
ENSG00000212496	0	0	0	0	0	0	0	0	0	0	0	0	0	0	0	0	0	0	0	0	0	0	0	0	0	0	0	0	0	0	0	0.36852628	0	0	0	0	0	0	0	0	0	0	0	0	0	0	0	0
ENSG00000212497	0	0.19805496	0.28917529	0.3822083	0	0.22180119	0.23745773	0.28962648	0.14618445	0	0	0.24970634	0.25027131	0	0.25715682	0	0	0	0	0	0	0	0	0	0.28226647	0	0	0	0	0	0	0	0.48508114	0	0	0	0	0	0	0.47495812	0	0	0.33854409	0	0	0	0	0.27644126
ENSG00000212498	0	0	0	0	0	0	0	0	0	0	0	0	0	0	0	0	0	0	0	0	0	0	0	0	0	0	0	0	0	0	0	0	0	0	0	0	0	0	0	0	0	0	0	0	0	0	0	0
ENSG00000212499	0	0	0	0	0	0	0	0	0	0	0	0.25437374	0	0	0	0	0	0	0.33148952	0	0	0.25725914	0	0	0	0	0	0	0	0	0	0	0	0	0	0	0	0	0	0	0	0	0	0	0	0	0	0
ENSG00000212505	0	0	0	0	0	0	0	0	0	0	0	0	0	0	0	0	0	0	0	0	0	0	0	0	0.26523315	0	0	0	0	0	0	0	0	0	0	0	0	0	0	0	0.51903931	0	0	0	0	0	0	0
ENSG00000212510	0	0	0	0	0	0	0	0	0	0	0	0	0	0	0	0	0	0	0	0	0	0	0	0	0	0	0	0	0	0	0	0	0	0	0	0	0	0	0	0	0	0	0	0	0	0	0	0
ENSG00000212511	0	0	0	0	0	0	0	0	0	0	0	0	0	0	0	0	0	0	0	0	0	0	0	0	0	0	0	0	0	0	0	0	0	0	0	0	0	0	0	0	0	0	0	0	0	0	0	0
ENSG00000212512	0	0	0	0	0.211511	0	0	0	0	0	0	0	0	0	0	0	0	0	0	0	0	0	0	0	0	0	0	0	0	0	0	0	0	0	0	0	0	0	0	0	0	0	0	0	0	0	0	0
ENSG00000212516	0	0	0	0	0	0	0	0	0	0	0	0	0	0	0	0	0	0	0	0	0	0	0	0	0	0	0	0	0	0	0	0	0	0	0	0	0	0	0	0	0	0	0	0	0	0	0	0
ENSG00000212517	0	0.35102424	0	0	0	0.39311105	0.21043002	1.02664347	0	0.47465376	0	0	0.22178514	0	0	0	0	0	0	0.23266306	0	0.22379454	0	0.24361321	0	0.21564287	0.45091265	0	0	0	0	0	0	0	0	0	0	0	0	0.42089785	0.48950049	0	0	0	0	0	0.3598997	0
ENSG00000212518	0	0	0	0	0	0	0	0	0	0	0	0	0	0	0	0	0	0	0	0	0	0	0	0	0	0	0	0	0	0	0	0	0	0	0	0	0	0	0	0	0	0	0	0	0	0	0	0
ENSG00000212520	0	0	0	0	0	0	0	0	0	0	0	0	0	0	0	0	0	0	0	0	0	0	0	0	0	0	0	0	0	0	0	0	0	0	0	0	0	0	0	0	0	0	0	0	0	0	0	0
ENSG00000212521	0	0	0	0	0	0	0.24417823	0	0	0.27538874	0	0	0	0	0	0	0	0	0	0	0.25595027	0	0	0	0	0	0	0	0	0	0	0.37200294	0	0	0	0.27046802	0	0	0	0	0	0	0	0	0	0	0	0
ENSG00000212522	0	0	0	0	0	0	0	0	0	0	0	0	0	0	0	0	0	0	0	0	0	0	0	0	0	0	0	0	0	0	0	0	0	0	0	0	0	0	0	0	0	0	0	0	0	0	0	0
ENSG00000212525	0	0	0	0	0	0	0	0	0	0	0	0	0	0	0	0	0	0	0	0	0	0	0	0	0	0	0	0	0	0	0	0	0	0	0	0	0	0	0	0	0	0	0	0	0	0	0	0
ENSG00000212526	0	0	0	0	0.17454791	0	0	0	0.15470005	0.28340977	0.39421112	0	0	0	0.27213683	0	0	0	0	0	0	0	0	0	0.29870917	0.25751527	0	0	0	0.25170821	0	0	0.51333829	0	0	0	0	0	0	0	0	0	0	0	0	0	0.27864056	0.29254463
ENSG00000212527	0	0	0	0	0	0	0	0	0	0	0	0	0	0	0	0	0	0	0	0	0	0	0	0	0	0	0	0	0	0	0	0	0	0	0	0	0	0	0	0	0	0	0	0	0	0	0	0

ENSG00000212528	0	0	0	0	0	0	0	0	0	0	0	0	0	0	0	0	0	[illegible]
ENSG00000212529	0	0	0.43476009	0	0	0.16673331	0	0	0	0	0	0.18771028	0	0.24079822	0	0	0	[illegible]
ENSG00000212532	0	0	0	0	0	0	0	0	0	0	0	0	0	0	0	0	0	[illegible]
ENSG00000212533	0	0	0	0	0	0	0	0	0	0	0	0	0	0	0	0	0	[illegible]
ENSG00000212534	0	0.9812723	1.07454909	0.9468341	0.61290119	1.09892406	2.64711402	4.66364465	2.53497132	4.64405554	1.84562478	2.16506744	0.92998542	1.19030937	0.95557138	1.05993007	3.30581109	[illegible]
ENSG00000212535	0	0	0.29185284	0	0.16646699	0.2238549	0	0	0.59015205	0	0	0	0	0.96988171	0	0	0	[illegible]
ENSG00000212536	0.35810822	0.95946625	0	1.3886901	0.39952078	0	0.2875877	0.70153971	0	0	1.35345817	1.20968847	0.90931908	1.16385805	0	0	0.53872477	[illegible]
ENSG00000212538	0	0	0	0	0	0	0	0	0	0	0	0	0	0	0	0	0	[illegible]
ENSG00000212539	0	0	0	0	0	0	0	0	0	0	0	0.13677382	0	0	0	0	0	[illegible]
ENSG00000212541	0.59684703	0.79955521	0.58370568	0	0	0.89541961	0	0	0	0	0	0.25201843	0	0	0	0	0.89787462	[illegible]
ENSG00000212542	0	0	0	0.3822083	0	0	0	0.28962648	0.14618445	0	0	0	0	0	0	0	0	[illegible]
ENSG00000212544	0.19299245	0.25853881	0.56622946	0.4989306	0.4306212	0.57907376	0.15498738	0.18903765	1.24037947	0.17479764	0.9725448	0.48894594	0	0.62722889	1.17491411	0	0	[illegible]
ENSG00000212545	0	0	0	0	0	0	0	0	0	0	0	0	0	0	0	0	0	[illegible]
ENSG00000212546	0	0	0	0	0	0	0	0	0	0	0	0	0	0	0	0	0	[illegible]
ENSG00000212549	0.23698338	0	0	0	0.13219438	0	0	0	0.23432508	0	0	0	0	0	0	0	0	[illegible]
ENSG00000212550	0	0	0	0	0	0	0	0	0	0	0	0	0	0	0	0	0	[illegible]
ENSG00000212551	0	0	0	0	0	0.23135243	0	0	0	0	0	0	0	0	0	0	0	[illegible]
ENSG00000212552	0	0.715624	0.17414424	0.4603393	0.39731348	0.53428352	1.00099585	0.52324785	0.88033732	0.9676643	1.3459805	0.75187819	0.30143174	0.57871395	0.30972479	0.68710015	0.80362259	[illegible]
ENSG00000212553	0	0	0	0	0	0	0	0	0	0	0	0	0	0	0	0	0	[illegible]
ENSG00000212555	0	0	0	0	0	0	0	0	0	0	0	0	0	0	0	0	0	[illegible]
ENSG00000212556	0	0	0	0	0	0	0	0	0	0	0	0	0	0	0	0	0	[illegible]
ENSG00000212558	0	0	0.54817577	0	0	0	0	0	0	0	0	0	0	0	0	0	0	[illegible]
ENSG00000212559	0	0	0	0	0	0	0	0	0	0	0	0	0	0	0	0	0	[illegible]
ENSG00000212560	0	0	0	0	0	0	0	0	0	0	0	0	0	0	0	0	0	[illegible]
ENSG00000212561	0	0	0	0	0	0	0	0	0	0	0	0	0	0	0	0	0	[illegible]
ENSG00000212564	0	0	0	0	0	0	0	0	0	0	0	0	0	0	0	0	0	[illegible]
[illegible]																		

ENSG00000213096	4.07125672	5.365152	[illegible]
ENSG00000213100	0	0	[illegible]
ENSG00000213104	0.03725981	0.02495721	[illegible]
ENSG00000213107	0	0	[illegible]
ENSG00000213108	0	0.04535292	[illegible]
ENSG00000213109	0.0310349	0.02078767	[illegible]
ENSG00000213110	0	0.04906361	[illegible]
ENSG00000213111	0	0	[illegible]
ENSG00000213113	0	0	[illegible]
ENSG00000213115	0	0	[illegible]
ENSG00000213117	0	0	[illegible]
ENSG00000213118	0	0	[illegible]
ENSG00000213120	0	0	[illegible]
ENSG00000213121	0.74458145	2.35116729	[illegible]
ENSG00000213122	0.06577498	0	[illegible]
ENSG00000213123	3.13158951	2.83659627	[illegible]
ENSG00000213126	0.15166936	0	[illegible]
ENSG00000213128	0.07997454	0.05356821	[illegible]
ENSG00000213130	0	0	[illegible]
ENSG00000213131	0.09104446	0.2439321	[illegible]
ENSG00000213132	0.3268448	0.61870343	[illegible]
ENSG00000213133	0	0	[illegible]
ENSG00000213135	0	0.17551212	[illegible]
ENSG00000213137	0.11981316	0.44139014	[illegible]
ENSG00000213139	0.19749768	0.40316061	[illegible]
ENSG00000213140	0	0	[illegible]
ENSG00000213144	0.05913714	0.03961099	[illegible]
ENSG00000213145	0.00474316	0.00953112	[illegible]
ENSG00000213147	0	0	[illegible]
ENSG00000213148	0	0	[illegible]
ENSG00000213149	0.28365008	0.23749165	[illegible]
ENSG00000213150	0	0	[illegible]
ENSG00000213152	0.16236645	0.10875562	[illegible]
ENSG00000213153	0	0.02874566	[illegible]
ENSG00000213155	0	0	[illegible]
ENSG00000213157	0.16155258	0.16231572	[illegible]
ENSG00000213158	0	0	[illegible]
ENSG00000213159	0	0	[illegible]
ENSG00000213160	10.7046653	10.2911763	[illegible]
ENSG00000213165	0	0	[illegible]
ENSG00000213167	0.11677442	0	[illegible]
ENSG00000213169	0	0.0283307	[illegible]
ENSG00000213170	0	0	[illegible]
ENSG00000213171	0	0.00638887	[illegible]
ENSG00000213172	0	0	[illegible]
ENSG00000213174	0	0	[illegible]
ENSG00000213176	0.05148521	0.10345682	[illegible]
ENSG00000213177	0	0	[illegible]
ENSG00000213178	0.07860912	0	[illegible]
ENSG00000213179	0	0.07980773	[illegible]
ENSG00000213180	0.10264248	0.06875156	[illegible]
ENSG00000213181	0	0	[illegible]
ENSG00000213182	0	0	[illegible]
ENSG00000213183	0	0	[illegible]
ENSG00000213184	0	0	[illegible]
ENSG00000213185	0.22496879	0.22999696	[illegible]
ENSG00000213186	0.01315822	0.01938988	[illegible]
ENSG00000213187	0	0	[illegible]
ENSG00000213188	0.03988829	0.10687124	[illegible]
ENSG00000213189	0	0.06121358	[illegible]
ENSG00000213190	47.5293682	30.6536742	[illegible]
ENSG00000213194	0	0	[illegible]
ENSG00000213197	0.51084602	0.8398786	[illegible]
ENSG00000213199	0.11530733	0.20655786	[illegible]
ENSG00000213201	0	0	[illegible]
ENSG00000213203	0.02588734	0.06068913	[illegible]
ENSG00000213204	0.00681967	0.05481504	[illegible]
ENSG00000213205	0	0	[illegible]
ENSG00000213209	0.07880132	0	[illegible]
ENSG00000213210	0	0	[illegible]
ENSG00000213211	0	0	[illegible]
ENSG00000213212	0.03283723	0.01099745	[illegible]
ENSG00000213213	0.03655202	0.11425456	[illegible]
ENSG00000213214	0.00776807	0	[illegible]
ENSG00000213215	0	0	[illegible]
ENSG00000213216	0.76133244	0.62327532	[illegible]
ENSG00000213218	0	0	[illegible]
ENSG00000213221	0.04405377	0.05901583	[illegible]
ENSG00000213222	0	0.02102044	[illegible]
ENSG00000213225	0.03669977	0.01966567	[illegible]
ENSG00000213226	0	0	[illegible]
ENSG00000213228	25.3684424	19.4053671	[illegible]
ENSG00000213231	0	0	[illegible]
ENSG00000213232	0	0.03628234	[illegible]
ENSG00000213233	0	0	[illegible]
ENSG00000213234	0	0	[illegible]
ENSG00000213235	1.48156512	2.35116729	[illegible]
ENSG00000213236	0.04427162	0.02965383	[illegible]
ENSG00000213237	0.09796273	0	[illegible]
ENSG00000213238	0	0	[illegible]
ENSG00000213239	0	0.03574171	[illegible]
ENSG00000213240	3.67040358	5.78135881	[illegible]
ENSG00000213244	0.46485202	0.51894208	[illegible]
ENSG00000213246	4.56049116	5.23335357	[illegible]
ENSG00000213247	0	0	[illegible]
ENSG00000213250	0	0.01760848	[illegible]
ENSG00000213252	0	0	[illegible]
ENSG00000213253	0	0.04326251	[illegible]
ENSG00000213260	0.08830066	0.17743554	[illegible]
ENSG00000213261	0.23944829	0.19246351	[illegible]
ENSG00000213262	0	0	[illegible]
ENSG00000213264	0	0	[illegible]
ENSG00000213265	0	0.04286166	[illegible]
ENSG00000213269	0.04235183	0.02836792	[illegible]
ENSG00000213270	0.07495288	0.02510231	[illegible]
ENSG00000213272	0	0	[illegible]
ENSG00000213275	0	0	[illegible]
ENSG00000213277	0	0	[illegible]
ENSG00000213279	0.0541904	0.13611596	[illegible]
ENSG00000213280	0.07194138	0.04818748	[illegible]
ENSG00000213281	41.9370563	41.1525619	[illegible]
ENSG00000213285	0	0	[illegible]
ENSG00000213287	0	0	[illegible]
ENSG00000213290	0.07964516	0.07113012	[illegible]
ENSG00000213291	0	0	[illegible]
ENSG00000213293	0.07822752	0.15719411	[illegible]
ENSG00000213295	0	0	[illegible]
ENSG00000213296	0.03696071	0	[illegible]
ENSG00000213297	0	0	[illegible]
ENSG00000213300	0.02690295	0.18020026	[illegible]
ENSG00000213301	0.0533605	0	[illegible]
ENSG00000213302	0	0	[illegible]
ENSG00000213303	0	0	[illegible]
ENSG00000213304	0.06069631	0.0813107	[illegible]
ENSG00000213305	0.14801258	0.09914117	[illegible]
ENSG00000213307	0	0	[illegible]
ENSG00000213309	0	0.08070277	[illegible]
ENSG00000213310	0	0	[illegible]
ENSG00000213312	0	0	[illegible]
ENSG00000213315	0.07358388	0.04928765	[illegible]
ENSG00000213316	0	0.00463262	[illegible]
ENSG00000213318	0	0	[illegible]
ENSG00000213326	1.09999112	1.21570595	[illegible]
ENSG00000213328	0	0	[illegible]
ENSG00000213331	0.14345582	0	[illegible]
ENSG00000213332	0	0.02436568	[illegible]
ENSG00000213333	0.03717386	0	[illegible]
ENSG00000213335	0	0.03165395	[illegible]
ENSG00000213337	1.44304171	1.03924681	[illegible]
ENSG00000213338	0	0	[illegible]
ENSG00000213339	1.35579809	1.6655423	[illegible]
ENSG00000213341	3.15594797	2.49968581	[illegible]
ENSG00000213343	0	0	[illegible]
ENSG00000213344	0	0	[illegible]
ENSG00000213347	3.72216246	2.63157132	[illegible]
ENSG00000213352	0.0960171	0.02143793	[illegible]
ENSG00000213355	1.27083311	2.08077018	[illegible]
ENSG00000213356	0	0	[illegible]
ENSG00000213358	0.3069499	0.15991104	[illegible]
ENSG00000213361	0	0	[illegible]
ENSG00000213362	0.11698635	0.11753897	[illegible]
ENSG00000213363	0.15981689	0.14273052	[illegible]
ENSG00000213365	0	0	[illegible]
ENSG00000213366	0.58975246	1.15241705	[illegible]
ENSG00000213368	0	0.08994996	[illegible]
ENSG00000213370	0.15013854	0.06704345	[illegible]
ENSG00000213371	0.32962234	0.41704073	[illegible]
ENSG00000213373	0.02460285	0.04943815	[illegible]
ENSG00000213375	0	0	[illegible]
ENSG00000213376	0	0	[illegible]
ENSG00000213380	2.04608664	2.55152035	[illegible]
ENSG00000213383	0	0	[illegible]
ENSG00000213384	0	0	[illegible]
ENSG00000213385	0.48709934	0.35345576	[illegible]
ENSG00000213386	0	0	[illegible]
ENSG00000213390	5.36359551	4.70308679	[illegible]
ENSG00000213391	0	0	[illegible]
ENSG00000213393	0	0	[illegible]
ENSG00000213394	0.03654166	0.07342854	[illegible]
ENSG00000213397	0.17240349	0.17728403	[illegible]
ENSG00000213398	0.80079739	0.88030537	[illegible]
ENSG00000213399	0	0.05173874	[illegible]
ENSG00000213400	0	0	[illegible]
ENSG00000213401	0.00610643	0.00409018	[illegible]
ENSG00000213402	0.47396676	0.55139604	[illegible]
ENSG00000213403	0.0985619	0	[illegible]
ENSG00000213406	0	0	[illegible]
ENSG00000213409	0	0	[illegible]
ENSG00000213410	0.84260758	0.56439191	[illegible]
ENSG00000213411	0.17767221	0.26181686	[illegible]
ENSG00000213412	0.05745052	0.03848127	[illegible]

ENSG00000213413	0.0155624	0.03127184	0	0	0	0.03502124	0.02499555	0.0152435	0	0	0.01960586	0	0	0	0.01353457	0	0.04682301	[illegible]
ENSG00000213414	0	0	0	0	0	0	0	0.02022376	0	0.01870032	0	0.01743625	0	0	0.0179565	0	0	[illegible]
ENSG00000213416	0	0	0	0	0	0	0	0	0	0	0	0	0	0	0	0	0	[illegible]
ENSG00000213417	0	0	0	0	0	0	0	0	0	0	0	0	0	0	0	0	0	[illegible]
ENSG00000213420	4.66975	3.31686789	3.34241446	4.6274472	3.98602641	4.54265239	0.26624994	0.30401589	0.37838705	0.59097977	0.35102822	0.37529731	0.11642628	0.07641878	0.116562	0.09526766	0.05305891	[illegible]
ENSG00000213421	0	0	0	0	0	0	0	0	0	0	0.05185664	0	0	0	0	0	0	[illegible]
ENSG00000213423	0	0	0	0	0	0	0	0	0	0	0	0	0	0	0	0	0	[illegible]
ENSG00000213424	0	0	0	0	0	0	0.14190182	0.15576951	0.1601564	0.29340576	0.16324605	0.14424739	0	0	0	0	0	[illegible]
ENSG00000213430	0.17305925	0.10909913	0.16924884	0.0921115	0.21578665	0.12217981	0.08175266	0.17948426	0.17615088	0.11986282	0.16672416	0.25790894	0.11201341	0.07719842	0.0973882	0.10802404	0.13782914	[illegible]
ENSG00000213431	0	0.01797501	0	0	0	0.02013017	0.02155112	0.02628583	0	0.02430575	0	0	0.06814215	0.20350557	0.35008444	0.07766349	0.12111215	[illegible]
ENSG00000213432	0.23228641	0.15558912	0.28396492	0.3753216	0.12957431	0.34848763	0.04663584	0.56881598	0.2583909	0.10519354	0.1463198	0.09808285	0.34406668	0.25164498	0.20201869	0.44816262	0.26208232	[illegible]
ENSG00000213433	0.37346164	0.52531611	0.62090592	0.2413714	0.2916548	0.22411429	0.89975293	0.8413599	0.86779022	0.87945697	0.7527925	0.63077614	0.28449149	0.32366852	0.4222378	0.43232373	0.56182189	[illegible]
ENSG00000213434	0	0	0	0	0	0	0.03718806	0.04535817	0.02289383	0	0	0	0	0	0	0.04467138	0	[illegible]
ENSG00000213435	0	0	0	0	0	0	0	0	0	0	0	0	0	0	0	0	0	[illegible]
ENSG00000213438	0	0	0	0	0	0	0	0	0	0	0	0	0	0	0	0	0	[illegible]
ENSG00000213439	0	0	0	0	0	0	0	0	0	0	0	0	0	0	0	0	0	[illegible]
ENSG00000213440	0	0.05465314	0	0	0	0	0	0	0	0	0	0	0	0	0	0	0	[illegible]
ENSG00000213442	0.0886244	0	0.08667307	0	0.01647886	0.04431958	0.02372401	0.0289361	0.02921009	0.1070255	0.037217	0.04989549	0	0	0	0	0	[illegible]
ENSG00000213443	0.30549516	0.66503288	0.29876878	0.5923323	0.21301463	0.45831904	0.55200482	0.37404368	0.4719818	0.58797453	0.24054351	0.32248804	0.16160884	0.16547745	0.23247708	0.22102807	0.28723477	[illegible]
ENSG00000213445	1.53798056	0.89129365	1.13914771	0.6358739	1.21328152	0.68004763	0.97779817	1.00984795	0.93647813	1.06693005	1.3083429	0.95660891	2.08983859	2.45431119	2.33256277	2.39821058	2.46679237	[illegible]
[illegible]																		

ENSG00000213752	0	0	0	0	0	0	0	0	0	0	[illegible]
ENSG00000213753	6.64952177	5.49060335	6.1318561	6.6594573	6.92981872	5.78592196	3.13904613	3.42416029	3.61126309	3.32491089	[illegible]
ENSG00000213754	0.02244411	0.01503342	0.02194993	0	0.0125198	0.01683588	0.09012149	0.08793673	0.11096174	0.06098441	[illegible]
ENSG00000213755	0	0	0	0	0	0	0.05325698	0	0	0	[illegible]
ENSG00000213757	0.81905311	0.63090697	1.041325	1.2704661	0.54826231	0.70655092	0.69064898	1.28363047	0.64789247	0.85311022	[illegible]
ENSG00000213759	0	0	0.01190336	0	0	0	0	0	0	0	[illegible]
ENSG00000213760	1.92534952	1.93649147	1.95468896	2.4729376	1.18651534	1.87982079	0.68719988	0.79626686	0.51068914	0.49823792	[illegible]
ENSG00000213761	0	0	0	0	0	0	0	0.17250976	0	0	[illegible]
ENSG00000213762	8.89911718	9.18708847	9.06834502	9.2427393	8.10574353	10.0271781	6.04717129	5.99200791	6.12258542	8.5674133	[illegible]
ENSG00000213763	0.1434984	0.038447	0.14033885	0.1483907	0.16009292	0.02152834	0.04609598	0.08433469	0.08513324	0.1039758	[illegible]
ENSG00000213770	0.05614937	0	0	0	0	0	0	0	0	0	[illegible]
ENSG00000213771	0	0	0	0	0	0	0	0	0	0	[illegible]
ENSG00000213772	0	0	0	0	0	0	0	0	0	0	[illegible]
ENSG00000213774	0	0	0	0	0	0	0	0	0	0	[illegible]
ENSG00000213777	0.28776553	0.32124986	0.14071476	0.5786209	0.08026087	0.41972794	0	0	0	0.0434393	[illegible]
ENSG00000213778	0	0.02241744	0	0.0432614	0.03733839	0.05021045	0	0.06556446	0.01654632	0	[illegible]
ENSG00000213779	0	0	0	0	0	0	0.05325183	0.05701078	0.06953587	0	[illegible]
ENSG00000213780	0.05964788	0.14915823	0.07389044	0.1490636	0.06876391	0.25056282	0.13731825	0.12074619	0.09240012	0.17647984	[illegible]
ENSG00000213781	0.02676889	0	0	0	0.01493225	0	0	0	0.01323431	0	[illegible]
ENSG00000213782	0.10611342	0.13622986	0.11458712	0.0971578	0.08755531	0.21392047	0.33021593	0.318313	0.27760908	0.42648515	[illegible]
[illegible]	[illegible]	[illegible]	[illegible]	[illegible]	[illegible]	[illegible]	[illegible]	[illegible]	[illegible]	[illegible]	[illegible]

ENSG00000214111	0	0	0	0	0	0	0	0	0	0	0	0	0	0	[illegible]
ENSG00000214113	1.88791715	1.75338882	1.87278358	1.7845756	1.44514096	1.58149784	0.99183525	1.13234781	1.20371831	0.74009058	1.011099	1.1501054	1.17558573	1.17129124	[illegible]
ENSG00000214114	0.69267821	0.44656809	0.51371536	0.3469549	0.63754457	0.37021155	1.11718047	1.32304345	1.24282313	0.85218351	1.25443647	1.23086642	0.82080562	0.68787169	[illegible]
ENSG00000214121	0.27038372	0.10866438	0.05288608	0.139801	0.09049548	0.12169293	0.08685534	0.15890581	0.05347015	0.04897853	0	0.13700331	0	0	[illegible]
ENSG00000214124	0	0.09070584	0.13243742	0.175045	0.07553964	0.20316243	0	0	0	0	0	0	0	0	[illegible]
ENSG00000214125	0	0	0	0	0	0	0	0	0.02336379	0	0	0	0	0	[illegible]
ENSG00000214128	0.01183467	0.02906584	0.01157409	0.0356946	0	0.0147958	0.00633608	0.01159215	0.02145351	0.0142919	0.03478901	0.00666291	0.01001698	0.00427365	[illegible]
ENSG00000214132	0	0.01537606	0.02245022	0.0296729	0.02561031	0	0	0	0.01134908	0	0.02892005	0	0	0.02486876	[illegible]
ENSG00000214135	3.85382265	4.58724247	4.04504034	3.2945694	3.69929464	4.18896796	1.96470802	1.85937409	1.48054902	1.84529331	1.50498776	1.38197465	2.33389589	1.87919198	[illegible]
ENSG00000214140	0	0	0	0	0	0	0.04468024	0.06812045	0.05615847	0.2309597	0.10513809	0.03523871	0.01765922	0.02009105	[illegible]
ENSG00000214141	0	0	0	0	0	0	0	0	0	0	0	0	0	0	[illegible]
ENSG00000214142	0	0	0	0.0566812	0	0	0	0	0	0	0	0	0	0	[illegible]
ENSG00000214144	0.06641884	0.0111221	0.14615196	0.0429271	0.12967444	0.04982242	0.08000894	0.03252889	0.09030147	0.13535335	0.08367593	0.11218131	0.11243513	0.07195413	[illegible]
ENSG00000214145	0.04993418	0.05574451	0.04340865	0.0430305	0.04332899	0.033295	0.04455654	0.05434548	0.03017303	0.03015101	0.08387759	0.02811292	0.01878435	0.01803189	[illegible]
ENSG00000214146	0	0	0	0	0	0	0	0	0	0	0	0	0.02285678	0.02925492	[illegible]
ENSG00000214147	0	0.01785607	0	0	0	0	0	0	0	0	0	0	0	0	[illegible]
ENSG00000214160	1.54085581	1.60701207	1.36736136	2.5665324	1.52175806	1.59024178	3.61260935	3.62632506	3.4285475	3.13211172	2.26690999	2.60075647	2.35980644	1.60429127	[illegible]
ENSG00000214161	0	0	0	0	0	0	0	0	0	0	0	0	0	0	[illegible]
ENSG00000214164	0	0	0	0	0	0	0	0	0	0	0	0	0	0	[illegible]
ENSG00000214167	0	0	0	0	0	0	0	0	0	0	0	0	0	0	[illegible]
[illegible]															

ENSG00000214578	0.24142127	0.0808539	0.11805283	0.0780163	0.10100244	0.1810961	0.43622853	0.23647406	0.89517446	0.5466518	[illegible]	[illegible]
ENSG00000214581	0	0	0	0	0	0	0	0	0	0	[illegible]	[illegible]
ENSG00000214584	0	0	0	0	0	0	0	0	0	0	[illegible]	[illegible]
ENSG00000214593	0	0	0.12813051	0	0	0	0	0.12833043	0.06477279	0	[illegible]	[illegible]
ENSG00000214595	0.41059932	0.34816017	0.26018601	0.5903821	0.65967251	0.52256309	0.23711783	0.26962985	0.25317574	0.51395911	[illegible]	[illegible]
ENSG00000214597	0	0.03116949	0.0273059	0.0240605	0.02595789	0.02094398	0.5231887	0.46492452	0.47392806	0.50576736	[illegible]	[illegible]
ENSG00000214602	0	0	0	0	0	0	0	0	0	0	[illegible]	[illegible]
ENSG00000214604	0	0	0	0	0	0	0	0	0	0	[illegible]	[illegible]
ENSG00000214607	0	0	0	0	0	0	0	0	0	0	[illegible]	[illegible]
ENSG00000214612	0.51983451	0.29845148	0.2905079	0.3839696	0.16569986	0.11141166	0.29819001	0.29096117	0.22028717	0.26904338	[illegible]	[illegible]
ENSG00000214614	0	0	0	0	0.00467094	0	0	0	0	0	[illegible]	[illegible]
ENSG00000214617	0.21877639	0.26976662	0.21395938	0.2570855	0.14145328	0.38416568	0.09982603	0.14123871	0.1499507	0.11708907	[illegible]	[illegible]
ENSG00000214626	0.05481248	0.03671427	0.05360562	0	0.04586335	0.04111621	0.04401852	0.10737853	0.05419764	0.07446736	[illegible]	[illegible]
ENSG00000214628	0	0	0	0	0	0	0	0	0	0	[illegible]	[illegible]
ENSG00000214629	0.14616662	0.07342854	0.10721125	0.1417031	0.12230228	0.19187563	0.20541978	0.14317137	0.25292231	0.13238642	[illegible]	[illegible]
ENSG00000214641	0	0	0.08102855	0	0	0	0	0	0	0	[illegible]	[illegible]
ENSG00000214642	0	0	0	0	0	0	0	0	0	0	[illegible]	[illegible]
ENSG00000214643	0	0	0	0	0	0	0	0	0	0	[illegible]	[illegible]
ENSG00000214645	0.70972225	0.7550204	0.6124373	0.9713635	0.46576257	1.25265955	0.46937888	1.59482149	0.88752142	1.43687285	[illegible]	[illegible]
ENSG00000214646	0	0.05918572	0.02160391	0	0.01232244	0	0.05322048	0	0.01092125	0.02000768	[illegible]	[illegible]
[illegible]	[illegible]	[illegible]	[illegible]	[illegible]	[illegible]	[illegible]	[illegible]	[illegible]	[illegible]	[illegible]	[illegible]	[illegible]

ENSG00000215030	0.25647008	0.19087525	0.08360771	[illegible]
ENSG00000215032	0.11769838	0.11825436	0.1151069	[illegible]
ENSG00000215033	0	0	0	[illegible]
ENSG00000215034	0.03378379	0	0.03303994	[illegible]
ENSG00000215035	0.09147514	0.10211916	0.11928139	[illegible]
ENSG00000215037	0	0	0	[illegible]
ENSG00000215039	1.15106213	1.55658581	1.21699253	[illegible]
ENSG00000215041	0.79334744	0.94774874	0.72188793	[illegible]
ENSG00000215043	0	0	0	[illegible]
ENSG00000215044	0	0	0	[illegible]
ENSG00000215045	0.04389691	0.03570346	0.04293039	[illegible]
ENSG00000215046	0	0	0	[illegible]
ENSG00000215049	0	0	0.05351461	[illegible]
ENSG00000215054	0	0	0	[illegible]
ENSG00000215057	0.04026201	0.0404522	0.11812657	[illegible]
ENSG00000215063	0.0670057	0.04488148	0	[illegible]
ENSG00000215065	0	0	0	[illegible]
ENSG00000215066	0.04674364	0.08610149	0.09142888	[illegible]
ENSG00000215067	0.29308795	0.33939196	0.37408265	[illegible]
ENSG00000215068	0.09253664	0.13429543	0.04902038	[illegible]
ENSG00000215070	0	0	0	[illegible]
ENSG00000215085	0.10071794	0.10119371	0	[illegible]
ENSG00000215086	0.03605116	0.02414764	0.03525739	[illegible]
ENSG00000215088	0.05232101	0	0	[illegible]
ENSG00000215089	0	0	0	[illegible]
ENSG00000215093	0.29406697	0.29545608	0.20131455	[illegible]
ENSG00000215094	0	0	0	[illegible]
ENSG00000215096	0	0	0	[illegible]
ENSG00000215097	0	0	0	[illegible]
ENSG00000215102	0	0	0	[illegible]
ENSG00000215104	0.06445948	0	0	[illegible]
ENSG00000215105	1.66562727	1.86670526	1.9616883	[illegible]
ENSG00000215110	0	0	0	[illegible]
ENSG00000215112	0	0	0	[illegible]
ENSG00000215113	0	0	0	[illegible]
ENSG00000215114	9.9380201	9.83302843	8.96836528	[illegible]
ENSG00000215115	0	0	0	[illegible]
ENSG00000215117	0	0	0	[illegible]
ENSG00000215120	0	0	0	[illegible]
ENSG00000215124	0.11981316	0.14445496	0.25778526	[illegible]
ENSG00000215126	0.04494545	0.06537124	0.07409699	[illegible]
ENSG00000215127	0	0	0.00663721	[illegible]
ENSG00000215131	0	0	0	[illegible]
ENSG00000215142	0.15274758	0.01461611	0.02134063	[illegible]
ENSG00000215146	0.79803885	0.74096676	0.77729503	[illegible]
ENSG00000215148	0.01711617	0	0	[illegible]
ENSG00000215149	0	0	0	[illegible]
ENSG00000215151	0	0	0	[illegible]
ENSG00000215154	0.71195687	0.95987384	0.64272095	[illegible]
ENSG00000215156	0	0	0	[illegible]
ENSG00000215158	1.30755271	2.3144698	1.87605366	[illegible]
ENSG00000215159	0.32831653	0.10995581	0.05351461	[illegible]
ENSG00000215160	0	0	0	[illegible]
ENSG00000215162	0.02966382	0.09934648	0.02901068	[illegible]
ENSG00000215165	0	0	0	[illegible]
ENSG00000215168	0	0	0	[illegible]
ENSG00000215174	0	0.00675892	0.01973707	[illegible]
ENSG00000215177	0	0	0	[illegible]
ENSG00000215179	0	0	0.02943054	[illegible]
ENSG00000215182	0	0	0	[illegible]
ENSG00000215183	1.19671608	0.47366055	0.55858417	[illegible]
ENSG00000215184	0.07918855	0.26520873	0.46466988	[illegible]
ENSG00000215186	0	0	0	[illegible]
ENSG00000215187	0.00781138	0.00523218	0	[illegible]
ENSG00000215190	0.51130331	0.66133888	0.5517743	[illegible]
ENSG00000215193	1.61890932	1.84887153	1.62298281	[illegible]
ENSG00000215196	0.08767015	0.19818964	0.12860975	[illegible]
ENSG00000215197	0	0	0	[illegible]
ENSG00000215198	0	0	0.01775781	[illegible]
ENSG00000215199	0.2620304	0.11700808	0.08542034	[illegible]
ENSG00000215203	0.03252244	0	0.06361273	[illegible]
ENSG00000215206	0	0	0	[illegible]
ENSG00000215208	0	0	0	[illegible]
ENSG00000215210	0.27593955	0.25876016	0.10794557	[illegible]
ENSG00000215217	0	0	0	[illegible]
ENSG00000215218	0.65698798	0.68009421	0.87616697	[illegible]
ENSG00000215221	0.08437105	0.05651306	0	[illegible]
ENSG00000215223	0.11284923	0.24188225	0.13243742	[illegible]
ENSG00000215227	0	0	0	[illegible]
ENSG00000215231	0	0	0	[illegible]
ENSG00000215236	0	0.05818865	0.04247993	[illegible]
ENSG00000215237	0.30418459	0.1296576	0.10817711	[illegible]
ENSG00000215241	0.47531097	0.83266214	0.607875	[illegible]
ENSG00000215244	0.37597882	0.18188197	0.19406417	[illegible]
ENSG00000215246	0.02326721	0.04363729	0.0273059	[illegible]
ENSG00000215251	7.06497427	7.66922003	7.4029478	[illegible]
ENSG00000215252	0.03993896	0.13375873	0.07030725	[illegible]
ENSG00000215256	1.91985063	2.1238001	2.07888691	[illegible]
ENSG00000215262	0	0	0	[illegible]
ENSG00000215263	0	0	0	[illegible]
ENSG00000215264	0	0	0	[illegible]
ENSG00000215267	0	0	0	[illegible]
ENSG00000215268	0	0	0	[illegible]
ENSG00000215269	0	0	0	[illegible]
ENSG00000215270	0	0	0	[illegible]
ENSG00000215271	1.20462492	1.35555314	1.23111611	[illegible]
ENSG00000215274	0	0	0	[illegible]
ENSG00000215277	0.01065798	0	0	[illegible]
ENSG00000215278	0	0	0	[illegible]
ENSG00000215283	0	0	0.05067541	[illegible]
ENSG00000215284	0	0.09887629	0.1443669	[illegible]
ENSG00000215286	0	0	0.08360771	[illegible]
ENSG00000215288	0	0.01762285	0	[illegible]
ENSG00000215296	0	0	0	[illegible]
ENSG00000215297	0.06946065	0.13957753	0.06793126	[illegible]
ENSG00000215298	0	0	0	[illegible]
ENSG00000215301	29.1017652	36.6441625	29.7390194	[illegible]
ENSG00000215302	0.08395591	0.04998666	0.0456152	[illegible]
ENSG00000215304	0.05875978	0.07871647	0.14366503	[illegible]
ENSG00000215305	3.21915215	3.41453759	3.08502001	[illegible]
ENSG00000215306	0	0	0	[illegible]
ENSG00000215307	0	0.06735722	0	[illegible]
ENSG00000215310	0	0	0	[illegible]
ENSG00000215311	0.07728954	0.02588488	0	[illegible]
ENSG00000215313	0	0	0.03656625	[illegible]
ENSG00000215317	0	0	0	[illegible]
ENSG00000215319	0	0	0	[illegible]
ENSG00000215323	0	0	0	[illegible]
ENSG00000215325	0	0	0	[illegible]
ENSG00000215326	0.05318439	0.03562375	0	[illegible]
ENSG00000215333	0	0	0	[illegible]
ENSG00000215339	0	0	0	[illegible]
ENSG00000215343	0	0	0	[illegible]
ENSG00000215346	0.22186604	0.74304694	0.795597	[illegible]
ENSG00000215347	0.25123405	0.21636071	0.14040137	[illegible]
ENSG00000215349	0	0	0	[illegible]
ENSG00000215351	0	0	0.1270972	[illegible]
ENSG00000215353	0	0	0	[illegible]
ENSG00000215354	0	0	0	[illegible]
ENSG00000215356	0	0	0	[illegible]
ENSG00000215357	0	0	0	[illegible]
ENSG00000215365	0	0	0	[illegible]
ENSG00000215367	0	0	0	[illegible]
ENSG00000215368	0	0	0	[illegible]
ENSG00000215369	0	0	0	[illegible]
ENSG00000215371	0	0	0	[illegible]
ENSG00000215372	0	0	0.00689869	[illegible]
ENSG00000215373	0	0	0	[illegible]
ENSG00000215374	0.12695122	0.36139423	0.24831202	[illegible]
ENSG00000215375	0.18497247	0.37398687	0.28474961	[illegible]
ENSG00000215378	0	0	0	[illegible]
ENSG00000215380	0	0	0	[illegible]
ENSG00000215381	0	0	0	[illegible]
ENSG00000215386	2.52304748	4.72573739	3.45354047	[illegible]
ENSG00000215388	0.02893154	0.17440926	0.2829453	[illegible]
ENSG00000215394	0	0	0	[illegible]
ENSG00000215397	0	0.01207044	0	[illegible]
ENSG00000215398	0	0	0	[illegible]
ENSG00000215399	0	0	0	[illegible]
ENSG00000215403	0	0.10881044	0.0317743	[illegible]
ENSG00000215405	0	0	0	[illegible]
ENSG00000215409	0.03988829	0	0	[illegible]
ENSG00000215414	0	0.01471574	0.0429722	[illegible]
ENSG00000215417	0.41411944	0.7122941	0.54500184	[illegible]
ENSG00000215418	0	0	0	[illegible]
ENSG00000215421	0.55959709	0.86959864	0.69255277	[illegible]
ENSG00000215424	0.31291009	0.47787008	0.35090345	[illegible]
ENSG00000215428	0	0	0	[illegible]
ENSG00000215430	0	0	0	[illegible]
ENSG00000215431	0	0	0.38913712	[illegible]
ENSG00000215432	0	0	0	[illegible]
ENSG00000215440	0.0662973	0.10740294	0.08443963	[illegible]
ENSG00000215441	0.01363356	0	0	[illegible]
ENSG00000215444	0	0	0	[illegible]
ENSG00000215447	3.88709138	5.5458602	3.83559978	[illegible]
ENSG00000215448	0	0	0	[illegible]
ENSG00000215450	0	0	0	[illegible]
ENSG00000215452	0.02290671	0.09717409	0.0746745	[illegible]
ENSG00000215454	0	0	0	[illegible]
ENSG00000215455	0	0	0	[illegible]
ENSG00000215456	0	0	0	[illegible]
ENSG00000215457	0	0	0.05067541	[illegible]
ENSG00000215458	0.02425721	0.05145156	0.03953852	[illegible]
ENSG00000215462	0	0	0	[illegible]
ENSG00000215464	0	0	0.06912304	[illegible]
ENSG00000215467	0	0	0	[illegible]
ENSG00000215472	0.0381417	0.05109584	0.02797643	[illegible]
ENSG00000215474	0.0082704	0	0	[illegible]
ENSG00000215475	0	0.00535682	0.00782137	[illegible]
ENSG00000215477	0	0	0	[illegible]
ENSG00000215478	0	0	0	[illegible]

ENSG00000215480	0	0	0	0	0	0	0	0	[illegible]
ENSG00000215481	0.04242616	0.07578057	0.04149202	0.0182803	0.02366622	0.04243322	0.01135713	0.04155676	[illegible]
ENSG00000215482	0	0.09616031	0	0.0927855	0.04004106	0	0.05764564	0.07031022	[illegible]
ENSG00000215483	0.12521266	0.03354777	0.09796459	0.0971112	0.11175406	0.05635508	0	0.02452936	[illegible]
ENSG00000215486	0	0	0	0	0	0	0	0	[illegible]
ENSG00000215492	0.11598067	0.12947615	0.08822101	0.0832881	0.12220448	0.24166663	0.04139607	0.10098133	[illegible]
ENSG00000215493	0.03785055	0	0	0	0.03167076	0.05678527	0.06079364	0.05561237	[illegible]
ENSG00000215498	0.00335028	0.01122037	0.00327652	0	0	0.00251313	0	0	[illegible]
ENSG00000215504	0.62110098	0.87726648	0.75267955	0.8028455	0.48956777	0.95206324	0.8024022	0.88610901	[illegible]
ENSG00000215506	0	0	0	0.0259568	0	0.01506313	0	0	[illegible]
ENSG00000215507	0	0	0	0	0	0	0	0	[illegible]
ENSG00000215512	0.01925601	0.02579596	0.02354003	0.037336	0.02416817	0.02166661	0.01933002	0.00943071	[illegible]
ENSG00000215513	0.04219402	0.0760906	0.04602634	0.052443	0.03439983	0.07790962	0.03388496	0.04132938	[illegible]
ENSG00000215515	0.08897099	0.04469563	0.04350601	0	0.06203739	0.01668484	0.03572518	0.06536084	[illegible]
ENSG00000215520	0	0	0	0	0	0	0	0	[illegible]
ENSG00000215527	0.31968182	0.36985737	0.39790937	0.6761881	0.25938229	0.45780245	0.23338947	0.17079867	[illegible]
ENSG00000215529	0.00825768	0.01659338	0	0	0.00460631	0	0.01326308	0.03235387	[illegible]
ENSG00000215533	0	0	0	0	0	0.00881062	0	0	[illegible]
ENSG00000215537	0	0	0	0	0	0	0	0	[illegible]
ENSG00000215540	0	0	0	0	0	0	0	0	[illegible]
ENSG00000215544	0	0	0	0	0	0.04444178	0.19031539	0.11606355	[illegible]
ENSG00000215545	0	0	0	0	0	0	0	0	[illegible]
ENSG00000215546	0	0	0	0	0	0	0	0	[illegible]
ENSG00000215547	0	0	0	0	0	0.09054805	0	0	[illegible]
ENSG00000215548	0.76160502	1.13621003	1.2188226	1.4766951	0.52139393	1.32437465	1.08424577	0.94945224	[illegible]
ENSG00000215549	0	0	0	0	0	0	0	0	[illegible]
ENSG00000215553	0	0	0	0	0	0	0	0	[illegible]
ENSG00000215559	0.05074218	0.08213746	0.02894788	0.0327951	0.0212288	0.03806297	0	0	[illegible]
ENSG00000215560	0	0	0	0	0	0	0	0	[illegible]
ENSG00000215562	0	0	0	0	0	0	0	0	[illegible]
ENSG00000215565	0.49394237	0.08271261	0.48306677	0.6384782	0.34441446	0.09262961	0.49584086	0.84668585	[illegible]
ENSG00000215567	0	0.00833835	0.01217463	0	0	0	0	0	[illegible]
ENSG00000215568	0.010581	0.00354366	0	0	0	0	0	0	[illegible]
ENSG00000215571	0.02997186	0.04015125	0.05862388	0.0258281	0.02229192	0.02997685	0.01604643	0.02935768	[illegible]
ENSG00000215572	0	0	0	0	0	0.01923336	0	0.02511479	[illegible]
ENSG00000215580	0.00284614	0	0	0.003679	0	0	0.00457133	0	[illegible]
ENSG00000215583	0	0	0	0	0	0	0	0	[illegible]
ENSG00000215586	0	0	0	0	0	0	0	0	[illegible]
ENSG00000215589	0	0	0	0	0	0	0	0	[illegible]
ENSG00000215595	0.14065348	0.10767078	0.01965094	0.103892	0.022417	0.16579777	0	0.0393632	[illegible]
ENSG00000215601	0	0	0	0	0	0	0	0	[illegible]
ENSG00000215603	0.02706107	0.05437781	0	0.0349796	0.01509524	0.02029919	0	0	[illegible]
ENSG00000215604	0.0205285	0.02750062	0.0200765	0.0265355	0.06298178	0.06159574	0.02472888	0.03016174	[illegible]
ENSG00000215606	0	0	0	0	0	0	0	0	[illegible]
ENSG00000215612	0	0.00848585	0	0	0	0	0.02034819	0	[illegible]
ENSG00000215621	0	0	0	0	0	0	0	0	[illegible]
ENSG00000215630	0.8651295	1.51253437	1.34799364	1.5731748	0.85884244	1.63888675	0.71831504	0.76122484	[illegible]
ENSG00000215644	0	0	0	0	0	0	0	0	[illegible]
ENSG00000215692	0	0	0	0	0.07617981	0.05122104	0	0.20065225	[illegible]
ENSG00000215695	0	0	0	0	0	0	0	0	[illegible]
ENSG00000215704	0	0	0	0	0	0	0	0	[illegible]
ENSG00000215712	2.51586378	2.50221535	2.66550872	2.5704317	2.22559771	2.43526598	1.80613917	2.05981351	[illegible]
ENSG00000215717	7.75911818	7.73399125	7.76868305	9.9699552	5.62730482	10.0795965	3.31926919	3.72665522	[illegible]
ENSG00000215720	0	0.03040562	0	0	0.1266087	0	0	0	[illegible]
ENSG00000215734	0	0	0	0	0	0	0	0	[illegible]
ENSG00000215749	0.03440198	0.03950227	0.03845088	0.0254106	0.00274145	0.04055194	0.00394677	0.00481386	[illegible]
ENSG00000215760	0	0	0	0	0	0	0	0	[illegible]
ENSG00000215765	0	0	0	0	0	0	0	0	[illegible]
ENSG00000215769	0.30313024	0.21866001	0.20067786	0.3315495	0.22632381	0.35682037	0.34081077	0.28778253	[illegible]
ENSG00000215771	0	0	0	0	0.03891436	0.05232972	0	0.06833179	[illegible]
ENSG00000215784	1.27949239	0.57736373	0.71127696	1.253477	0.96165469	0.97998494	0.16224128	0.30342399	[illegible]
ENSG00000215785	0	0	0	0	0	0	0	0	[illegible]
ENSG00000215788	0.02812368	0.06996856	0.03143366	0.0605886	0.02166437	0.10146303	0.35276277	0.35549226	[illegible]
ENSG00000215790	0.30051039	0.52418392	0.39185836	0.5179264	0.2409697	0.43675187	0.46255364	0.50898423	[illegible]
ENSG00000215791	0	0	0	0	0	0	0	0	[illegible]
ENSG00000215795	0.09649623	0.03878082	0.07549726	0.0748396	0.08612424	0.07238422	0.06199495	0.11342259	[illegible]
ENSG00000215796	0	0	0	0	0.01460474	0	0	0	[illegible]
ENSG00000215800	0	0	0	0	0	0	0	0	[illegible]
ENSG00000215802	0	0	0	0	0	0	0	0	[illegible]
ENSG00000215805	0	0	0	0	0	0.01657048	0	0	[illegible]
ENSG00000215807	0	0	0	0	0	0	0	0	[illegible]
ENSG00000215808	0	0	0	0	0	0	0	0	[illegible]
ENSG00000215811	0	0	0	0	0	0	0	0	[illegible]
ENSG00000215812	0	0	0	0	0	0	0	0	[illegible]
ENSG00000215817	0.0113625	0.02663775	0.01111232	0.022031	0.00950737	0	0.00912494	0.01112966	[illegible]
ENSG00000215819	0	0	0	0	0	0	0.0203322	0	[illegible]
ENSG00000215833	0	0	0	0	0	0	0	0.02049954	[illegible]
ENSG00000215834	0	0.00687516	0.03011475	0.0132677	0	0.0230984	0.01648592	0.03016174	[illegible]
ENSG00000215835	0.03273717	0.04385575	0.04802454	0.0211583	0.00913074	0.03683544	0.02629039	0.01603316	[illegible]
ENSG00000215837	0	0	0	0	0	0	0	0	[illegible]
ENSG00000215838	0.04384999	0.05874283	0.02858967	0.1133625	0.04076743	0.07675025	0.01173827	0.01431714	[illegible]
ENSG00000215840	0.69272065	0.51283688	0.74878081	0.9425498	0.65080308	0.4922782	0.73198226	0.78566098	[illegible]
ENSG00000215841	0.24602855	0.32958764	0	0	0.20585994	0.36910427	0.19757933	0.24098692	[illegible]
ENSG00000215845	0.00780192	0.03135511	0	0.0201698	0	0.02926208	0.05012422	0.04585227	[illegible]
ENSG00000215846	0.02659219	0	0	0	0	0	0	0	[illegible]
ENSG00000215853	0	0	0	0	0	0	0	0	[illegible]
ENSG00000215859	0	0	0	0	0	0	0	0	[illegible]
ENSG00000215860	0	0	0	0	0	0	0	0	[illegible]
ENSG00000215861	0.51294016	0.34357545	0.41803855	0.5525292	0.23844078	0.89779473	0.06865489	0.16747632	[illegible]
ENSG00000215863	0.03990475	0.00890961	0	0	0	0	0	0	[illegible]
ENSG00000215864	0.28086919	0.45688869	0.2844952	0.3371236	0.13988823	0.35365312	0.17722491	0.20633521	[illegible]
ENSG00000215866	0.01960446	0.05252552	0.04793204	0.0126705	0.00546789	0.04411739	0.04723156	0.04800682	[illegible]
ENSG00000215867	0	0	0	0	0	0	0	0	[illegible]
ENSG00000215869	0.02630999	0.01762285	0.0514614	0.0340087	0.04402882	0.05920734	0.04225778	0	[illegible]
ENSG00000215871	0	0	0	0	0	0	0	0	[illegible]
ENSG00000215873	0	0	0	0.0363849	0.04710507	0	0	0	[illegible]
ENSG00000215874	0	0	0	0	0	0	0	0	[illegible]
ENSG00000215875	0	0	0	0	0	0	0.02430319	0	[illegible]
ENSG00000215878	0.09906682	0.11059421	0.06459038	0.1280554	0.07368211	0.04954166	0.10607743	0.06469116	[illegible]
ENSG00000215881	0	0	0	0	0	0	0	0	[illegible]
ENSG00000215883	0.178052	0.21903684	0.17413166	0.185025	0.17267607	0.18157344	0.15887676	0.20404053	[illegible]
ENSG00000215887	0	0	0	0	0	0	0	0	[illegible]
ENSG00000215893	0	0	0	0	0	0	0	0	[illegible]
ENSG00000215895	0	0.0550714	0.08040844	0.0637664	0.08255404	0.03700459	0.01320556	0.01610678	[illegible]
ENSG00000215899	0.38425919	0.33781505	0.82205941	0.8692248	0.2545382	0.73862109	0.34716249	0.65867364	[illegible]
ENSG00000215900	0	0	0	0	0	0	0	0	[illegible]
ENSG00000215902	0.97665878	1.63545383	1.91030949	3.1561138	0.81720159	4.02938823	0.78433008	0.47832253	[illegible]
ENSG00000215905	0	0	0	0	0	0	0	0	[illegible]
ENSG00000215906	0	0	0	0	0	0	0	0	[illegible]
ENSG00000215908	0.85035	1.40583681	0.84124122	1.2770781	0.69643473	1.20199533	1.8197367	2.46988625	[illegible]
ENSG00000215909	0	0	0	0	0	0	0	0	[illegible]
ENSG00000215910	0.01877218	0.0055884	0.00815949	0.0134807	0.00349051	0.01721069	0.01675051	0.0183875	[illegible]
ENSG00000215912	0.04967828	0.15101867	0.056059	0.0839734	0.04050157	0.13759353	0.27926763	0.28447543	[illegible]
ENSG00000215914	0	0	0	0	0	0	0	0	[illegible]
ENSG00000215915	0.21091036	0.27812749	0.39964143	0.298185	0.25000687	0.30158611	0.39168386	0.38089733	[illegible]
ENSG00000215921	0.34655634	0.23212893	0.33892588	0	0.57994952	0	0	0	[illegible]
ENSG00000215922	0	0	0	0	0	0	0	0	[illegible]
ENSG00000215923	0	0	0	0	0	0	0	0	[illegible]
ENSG00000215924	0	0	0	0	0	0	0	0	[illegible]
ENSG00000215925	0	0	0	0	0	0	0	0	[illegible]
ENSG00000215930	0	0	0	0.4844268	0	0	0	0.36708473	[illegible]
ENSG00000215933	0	0	0	0	0	0	0	0	[illegible]
ENSG00000215934	0	0	0	0	0.23348617	0	0	0	[illegible]
ENSG00000215938	0	0	0	0	0	0	0	0	[illegible]
ENSG00000215939	0	0	0	0	0	0.3139783	0	0	[illegible]
ENSG00000215941	0	0	0	0	0	0	0	0	[illegible]
ENSG00000215942	0	0	0	0	0	0	0	0	[illegible]
ENSG00000215943	0	0	0	0	0	0	0	0	[illegible]
ENSG00000215945	0	0	0	0	0	0	0	0	[illegible]
ENSG00000215946	0.27546786	0.55353822	0.80820786	1.0682231	0.46098551	1.23981176	1.54854913	0.26982296	[illegible]
ENSG00000215948	0	0	0	0	0	0	0	0	[illegible]
ENSG00000215952	0	0	0	0	0	0	0	0.56373726	[illegible]
ENSG00000215953	0	0	0	0	0	0	0.28442739	0	[illegible]
ENSG00000215954	0	0.45448401	0	0	0	0	0	0.33230828	[illegible]
ENSG00000215957	0	0	0	0	0	0.58256215	0	0	[illegible]
ENSG00000215958	0	0	0	0	0	0	0	0	[illegible]
ENSG00000215960	0	0	0	0	0	0	0	0	[illegible]
ENSG00000215961	0	0	0	0	0	0	0	0	[illegible]
ENSG00000215964	0	0	0	0	0	0	0	0	[illegible]
ENSG00000215966	0	0	0	0	0	0	0	0	[illegible]
ENSG00000215968	0	0.23465207	0	0	0	0	0	0	[illegible]
ENSG00000215972	0	0.25102315	0	0	0	0	0.30096387	0	[illegible]
ENSG00000215973	0	0.56072703	0.40935203	0.5410481	0.46697234	0.62795661	0.33614146	0	[illegible]
ENSG00000215976	0	0	0	0.443199	0	0	0.27534992	0	[illegible]
ENSG00000215977	0	0	0	0	0	0	0	0	[illegible]
ENSG00000215979	0	0	0	0	0	0	0	0	[illegible]
ENSG00000215983	0	0	0.38913712	0	0.22195599	0	0	0	[illegible]
ENSG00000215984	0	0	0	0	0	0	0	0	[illegible]
ENSG00000215991	0	0	0	0	0	0	0	0	[illegible]
ENSG00000215993	0	0	0	0	0	0	0	0	[illegible]
ENSG00000215996	0	0	0	0	0	0	0	0	[illegible]
ENSG00000215998	0	0	0	0	0	0	0	0	[illegible]
ENSG00000215999	0	0	0	0	0	0	0	0	[illegible]
ENSG00000216001	0	0	0	0	0	0	0	0.40473445	[illegible]
ENSG00000216002	0	0	0	0	0	0	0	0	[illegible]
ENSG00000216004	0	0	0	0	0	0	0.35948462	0	[illegible]
ENSG00000216005	0	0	0	0	0	0	0	0	[illegible]
ENSG00000216009	0	0	0	0	0	0	0	0	[illegible]
ENSG00000216011	0	0	0	0	0	0	0	0	[illegible]
ENSG00000216014	0	0	0	0	0	0	0	0	[illegible]
ENSG00000216015	0	0	0	0	0	0	0	0	[illegible]
ENSG00000216017	0	0	0	0	0	0	0	0	[illegible]
ENSG00000216020	0	0	0	0	0	0	0	0	[illegible]
ENSG00000216022	0	0	0	0	0	0	0	0	[illegible]
ENSG00000216027	0	0	0	0	0	0	0	0	[illegible]
ENSG00000216031	0	0	0	0.4734171	0	0.27473102	0.29412378	0.3587419	[illegible]
ENSG00000216032	0	0	0	0	0	0	0	0	[illegible]
ENSG00000216035	0	0.26009627	0	0	0	0	0.31184208	0	[illegible]
ENSG00000216037	0	0	0	0	0	0	0	0	[illegible]
ENSG00000216042	0	0	0	0	0	0	0	0	[illegible]
ENSG00000216045	0	0	0	0	0	0	0	0	[illegible]
ENSG00000216047	0	0	0	0	0	0	0	0	[illegible]
ENSG00000216054	0	0	0	0	0	0	0	0	[illegible]
ENSG00000216055	0	0	0	0	0	0	0	0	[illegible]
ENSG00000216056	0	0	0	0	0	0	0	0	[illegible]
ENSG00000216058	0	0	0	0	0	0	0	0	[illegible]
ENSG00000216060	0	0	0	0	0	0	0	0	[illegible]
ENSG00000216064	0	0	0	0	0.22757513	0	0	0	[illegible]
ENSG00000216067	0	0	0	0	0	0	0	0	[illegible]
ENSG00000216069	0	0	0	0	0	0	0	0	[illegible]
ENSG00000216070	0	0	0	0	0	0	0	0	[illegible]

ENSG00000216072	0	0	0	0	0	0	0	0	0	0	0	0	0	0	0	0	0
ENSG00000216073	0	0	0	0	0	0	0	0	0	0	0	0	0	0	0	0	0
ENSG00000216075	0	0	0	0	0	0	0	0	0	0	0	0	0	0	0	0	0
ENSG00000216076	0	0	0	0	0	0	0	0	0	0	0	0	0	0	0	0	0
ENSG00000216077	0	0.2732657	0	0	0	0	0	0	0	0	0	0	0	0	0	0	0
ENSG00000216081	0	0	0	0	0	0	0	0	0	0	0	0	0	0	0	0	0
ENSG00000216083	0	0	0	0	0	0	0	0	0	0	0	0	0	0	0	0	0
ENSG00000216084	0	0	0	0	0	0	0	0	0	0	0	0	0	0	0	0	0
ENSG00000216085	0	0	0	0	0	0	0	0	0	0	0	0	0	0	0	0	0
ENSG00000216088	0	0	0	0	0	0	0	0	0	0	0	0	0	0	0	0	0
ENSG00000216089	0	0	0	0	0	0	0	0	0	0	0	0.38882844	0	0	0	0	0
ENSG00000216090	0.37476442	0.25102315	0	0.4844268	0.20905157	0.56224022	0.60192774	1.10125419	0.92640148	1.35773052	0.94427314	0.63297653	0.95161299	0.81199399	0	0	0
ENSG00000216093	0	0	0	0	0	0	0	0	0	0	0	0	0	0	0	0	0
ENSG00000216097	0	0	0	0	0	0	0	0	0	0	0	0	0	0	0	0	0
ENSG00000216098	0	0	0	0	0	0	0	0	0	0	0	0	0	0	0	0	0
ENSG00000216099	0	0	0	0	0	0	0	0	0	0	0	0	0	0.44197141	0.35481131	0.78712106	1.22747416
ENSG00000216100	0	0	0	0	0	0	0	0	0	0	0	0	0	0	0	0	0
ENSG00000216101	0	0	0	0	0	0	0	0	0	0	0	0	0	0	0	0	0
ENSG00000216102	0	0	0	0	0.23049276	0	0	0	0	0	0	0	0	0	0	0	0
ENSG00000216105	0	0	0	0	0	0	0	0	0	0	0	0	0	0	0	0	0
ENSG00000216109	1.08639572	0.48512338	0	0.4680978	0.40400978	1.0865766	0.87245706	1.06413326	0.71613957	0.98397324	0.45622186	0.91746036	0	0.3923117	0.31494487	0	1.08955571
ENSG00000216112	0	0	0	0	0	0	0	0	0	0	0	0	0	0	0	0	0
ENSG00000216113	0	0	0	0	0.39952078	0	0	0	0	0	0	0	0	0	0	0	0
ENSG00000216114	0	0	0	0	0	0	0	0	0	0	0	0	0	0	0	0	0
ENSG00000216115	0	0	0	0	0.19976039	0	0	0	0.17704562	0.32434674	0	0	0	0	0	0	0
ENSG00000216116	0	0.46425786	0	0	0.19331651	0.25996053	0	0	0	0	0	0	0	0	0	0	0
ENSG00000216123	0	0	0	0	0	0	0	0	0	0	0	0	0	0	0	0	0
ENSG00000216125	0	0	0	0	0	0	0	0	0	0	0	0	0	0	0	0	0
ENSG00000216127	0	0	0	0	0	0.78835857	0	0	0	0.31729572	0.44134506	0.29584772	0.59303418	0	0	0	0
ENSG00000216131	0	0	0	0	0	0	0	0	0	0	0	0	0	0	0	0	0
ENSG00000216133	0	0	0	0	0	0	0	0	0	0	0	0	0	0	0	0	0
ENSG00000216135	0	0.2917296	0	0	0	0.32670715	0	0	0	0	0	0	0	0	0	0	0
ENSG00000216136	0	0	0	0	0	0	0	0	0	0	0	0	0	0	0.87594043	0.64773504	1.51516342
ENSG00000216141	0	0	0	0	0	0	0	0	0	0	0	0	0	0	0	0	0
ENSG00000216154	0	0.23465207	0	0	0	0	0	0	0	0	0	0	0	0	0	0	0
ENSG00000216157	0	0	0	0	0	0	0	0	0	0	0	0	0	0	0	0	0
ENSG00000216162	0	0.25699989	0	0	0	0	0	0	0	0	0	0	0	0	0	0	0
ENSG00000216166	0	0	0	0	0	0	0	0	0	0	0	0	0	0	0	0	0
ENSG00000216168	0	0	0	0	0	0	0	0	0	0	0	0	0	0	0	0	0
ENSG00000216169	0	0	0	0	0.211511	0	0	0	0	0	0	0	0	0	0	0	0
ENSG00000216171	0	0	0	0	0	0	0	0	0	0	0	0	0	0	0	0	0
ENSG00000216173	0	0	0	0	0	0	0	0	0	0	0	0	0	0	0	0	0
ENSG00000216179	0	0.25699989	0.75047873	0	0	0.28781345	0	0	0	0	0	0	0.64951363	0.83132718	1.00107478	1.48053723	0
ENSG00000216181	0	0	0	0	0	0	0	0	0	0	0	0	0	0	0	0	0
ENSG00000216182	0	0	0	0	0.2043004	0	0	0	0	0	0.4614062	0	0	0	0	0	0
ENSG00000216184	0	0	0	0	0	0	0	0.3394547	0	0	0	0	0	0	0	0	0
ENSG00000216191	0	0	0	0	0	0	0	0	0	0	0	0	0	0	0	0	0
ENSG00000216192	0	0	0	0	0	0	0	0	0	0	0	0	0	0	0	0	0
ENSG00000216193	0	0.24531807	0	0	0	0	0	0	0	0	0	0	0	0	0	0	0
ENSG00000216194	0	0	0	0	0	0	0	0	0	0	0	0	0	0	0	0	0
ENSG00000216195	0	0	0	0	0	0	0	0	0	0	0	0	0	0	0	0	0
ENSG00000216197	0	0	0	0	0	0	0	0	0	0	0	0	0	0	0	0	0
ENSG00000216204	0	0.23465207	0.34260985	0	0	0	0	0	0	0	0	0	0	0	0	0	0
ENSG00000216205	0	0.23723067	0	0	0	0	0	0	0	0	0	0	0	0	0.30802301	0	0
ENSG00000216206	0	0	0	0	0	0	0	0	0	0	0	0	0	0	0	0	0
ENSG00000216265	0	0	0	0	0	0	0	0	0	0	0	0	0	0	0	0	0
ENSG00000216285	0.02685812	0	0.05253351	0.0694345	0.01498203	0.04029388	0	0	0.01327842	0	0.03383645	0	0.02273298	0	0	0	0.04040436
ENSG00000216306	0	0	0	0	0	0	0	0	0	0	0	0	0	0	0	0	0
ENSG00000216307	0	0	0.0456152	0	0	0.03498745	0.07491431	0	0.02305949	0	0	0	0	0	0	0	0.07016676
ENSG00000216316	0	0	0	0	0	0	0.08894465	0.21697104	0.10951275	0.05015671	0	0.14029892	0.04687212	0.05999268	0	0.05342145	0.16661591
ENSG00000216324	0	0	0	0	0	0	0	0	0	0	0	0	0	0	0	0	0
ENSG00000216331	0.15645505	0	0	0	0.08727396	0.35208247	0	0.15324897	0.15470005	0	0.39421112	0.13212617	0.26485022	0.16949389	0.40820525	0.15092855	0.23536519
ENSG00000216347	0	0	0	0	0	0	0.07796052	0.04754411	0	0	0	0	0	0.05258395	0	0	0
ENSG00000216352	0	0	0	0	0	0	0	0	0	0	0	0	0	0	0.02077842	0	0
ENSG00000216359	0	0	0	0	0	0	0	0	0	0	0	0	0	0	0	0	0
ENSG00000216360	0	0	0	0	0	0	0	0	0	0	0	0	0	0	0	0	0
ENSG00000216364	0	0	0	0	0	0	0	0	0.037404	0.06852396	0.09531396	0	0	0	0	0	0
ENSG00000216365	0	0	0	0	0	0	0	0	0	0	0	0	0	0	0	0	0
ENSG00000216368	0.04018671	0.05383539	0.15720751	0	0.067251	0.03014505	0.03227293	0.0393632	0.13907573	0.10919404	0.15188433	0	0.03401443	0	0.06990048	0	0
ENSG00000216378	0	0	0	0	0	0	0	0	0	0	0	0	0	0	0	0	0
ENSG00000216412	0	0	0	0	0	0	0	0	0	0	0	0.02754857	0	0.03533982	0	0	0
ENSG00000216425	0	0	0	0.051946	0	0	0	0	0	0	0	0	0	0	0	0	0
ENSG00000216436	0	0.06522052	0.09522691	0	0	0	0	0	0	0	0	0	0.08241563	0	0	0	0
ENSG00000216439	0	0	0	0	0	0	0	0	0	0.06224138	0	0	0	0	0	0	0
ENSG00000216444	0	0	0	0	0	0	0	0	0	0	0	0	0	0	0	0	0
ENSG00000216471	0.10313517	0.06908157	0.10086434	0.0666571	0.08629649	0.07736425	0.08282526	0	0	0.04670593	0	0.04354879	0	0	0	0	0.07757637
ENSG00000216475	0.10906849	0.0365279	0	0.0704919	0.12168146	0	0	0	0.08088378	0	0	0	0.04615833	0	0	0	0.08203931
ENSG00000216480	0	0.04632616	0.13527943	0.0894006	0.115741	0.15564161	0	0.1354905	0.10258008	0.12528415	0	0	0.05853986	0.14985297	0.24060166	0.26687796	0.10404556
ENSG00000216490	0.06162474	0.06486422	0.07748729	0.0796572	0.03928639	0.10566	0.19088722	0.17246264	0.13927653	0.09568273	0.05545445	0.09664952	0.06706259	0.05722329	0.06125123	0.0254777	0
ENSG00000216516	0	0	0	0	0	0	0	0	0	0	0	0	0	0	0	0	0
ENSG00000216518	0	0	0	0	0	0	0	0	0	0	0	0	0	0	0	0	0
ENSG00000216519	0	0	0	0	0	0	0	0	0	0	0	0	0	0	0	0	0
ENSG00000216522	0	0.02163125	0.06316655	0	0	0	0	0	0	0	0	0	0	0	0.02808627	0	0
ENSG00000216523	0	0	0	0	0	0	0	0	0	0	0	0	0	0	0	0	0
ENSG00000216548	0	0	0	0	0	0	0	0	0	0	0	0	0	0	0	0	0
ENSG00000216560	0	0	0	0	0	0	0	0	0	0	0	0	0	0	0	0	0
ENSG00000216588	0	0	0	0	0	0	0.02112027	0.01288017	0.03250532	0.11909917	0.03313239	0.0222097	0.02225995	0.01424551	0.01143619	0	0
ENSG00000216613	0	0	0	0	0	0	0	0.05452381	0	0	0	0	0	0	0	0	0
ENSG00000216616	0	0	0	0	0	0	0	0	0	0	0	0	0	0	0	0	0
ENSG00000216621	0	0	0	0	0	0	0	0	0.00779555	0.01428141	0.01986485	0	0	0.01708207	0	0	0
ENSG00000216624	0.03191063	0.0427485	0.18724816	0.0824964	0.28480689	0.14362176	0.05125325	0.03125672	0.11043439	0.05780437	0.24121037	0.05389701	0.02700948	0	0.13876284	0.06156689	0.19202071
ENSG00000216629	0	0	0	0	0	0	0	0	0	0	0	0	0	0	0	0	0
ENSG00000216636	0	0	0	0	0.02493542	0	0	0	0.02210001	0	0	0	0	0	0	0	0
ENSG00000216639	0	0	0	0	0	0	0	0	0	0	0	0	0	0	0	0	0
ENSG00000216642	0	0	0	0	0	0	0	0	0	0.07661734	0	0	0	0	0.0735698	0	0
ENSG00000216649	0	0	0	0	0	0	0	0	0	0	0	0	0	0	0	0	0
ENSG00000216657	0	0.02079768	0	0	0	0	0	0	0.01535078	0	0	0	0	0	0.05400789	0.02995307	0
ENSG00000216663	0	0	0	0	0.0540976	0	0	0	0	0	0	0	0	0	0	0	0
ENSG00000216671	0	0	0	0	0	0	0	0	0	0	0	0	0	0	0	0	0
ENSG00000216676	0	0	0	0	0	0	0	0	0	0	0	0	0	0	0	0	0
ENSG00000216687	0	0	0	0	0	0	0	0	0	0	0	0	0	0	0	0	0
ENSG00000216708	0	0	0	0	0	0	0	0	0	0	0	0	0	0	0	0	0
ENSG00000216710	0	0	0	0	0	0	0	0	0.05438261	0	0	0	0	0	0	0	0
ENSG00000216713	0	0	0	0	0	0	0	0	0	0	0	0	0	0	0	0	0
ENSG00000216718	0.09937227	0	0.03239477	0.0428168	0.05543197	0.04969441	0.07980337	0.06489062	0.08188132	0	0	0.05594654	0	0.07176925	0.08642372	0.03195404	0.14949197
ENSG00000216721	0	0	0	0	0	0	0	0	0	0	0	0	0	0	0	0	0
ENSG00000216723	0	0	0	0	0	0	0	0	0	0	0	0	0	0	0	0	0
ENSG00000216740	0	0	0	0	0	0	0	0.03131874	0.01580764	0	0	0.05400395	0.05412614	0	0.0834229	0.03084453	0.04810043
ENSG00000216753	0	0	0	0	0	0	0.05155955	0	0	0	0	0	0	0	0	0	0
ENSG00000216754	0	0	0	0	0	0	0	0	0	0	0	0	0	0	0	0	0
ENSG00000216762	0	0	0	0	0	0	0	0	0	0	0	0	0	0	0	0	0
ENSG00000216775	12.0942678	10.5299695	11.510508	8.5599256	7.10342195	7.93122748	7.25465826	9.14827967	6.3919836	10.3156263	5.79552737	5.19295764	1.9468587	1.37653427	1.3793226	1.26154554	1.42316357
ENSG00000216777	0	0	0	0	0.11307192	0	0	0	0	0.18359249	0	0	0	0	0	0	0
ENSG00000216781	0	0	0	0	0	0	0	0	0	0	0	0	0	0	0	0	0
ENSG00000216802	0.08617577	0.14430475	0.2106959	0.1670884	0.16824739	0.06464259	0.0346028	0.16881972	0.02130228	0.07805135	0.16284925	0.07277538	0.10941005	0.23339399	0.03747339	0	0.06481983
ENSG00000216809	0.2426169	0.37633581	0.13736972	0.2145757	0.19232082	0.23946444	0.12305654	0.10006113	0.07575644	0.13878545	0.04826119	0.12940408	0.17292914	0.17983544	0.05552713	0.1724556	0.09604839
ENSG00000216811	0	0	0	0	0	0	0	0	0	0	0	0	0	0	0	0	0
ENSG00000216813	0.08118322	0.16313343	0	0	0.13585719	0.12179511	0.32598102	0.31807846	0.36122657	0.22058846	0.40910575	0.20567751	0.06871429	0.26384691	0.07060477	0.07831557	0
ENSG00000216817	0	0.01020226	0	0	0	0.01142549	0	0	0	0	0	0	0	0	0	0	0
ENSG00000216819	0.8140692	0.49570587	1.04946394	0.860956	0.90821026	0.77719543	0	0.03624488	0	0	0	0.06249826	0.03131983	0.08017392	0.06436302	0.03569608	0
ENSG00000216824	0	0	0	0	0	0	0	0	0	0	0	0	0	0	0	0	0
ENSG00000216829	0	0	0	0	0	0	0	0	0	0	0	0	0	0	0	0	0
ENSG00000216835	0	0	0.02646524	0.0349796	0	0	0	0	0.01337876	0	0	0	0	0	0	0	0
ENSG00000216844	0	0	0	0	0	0	0	0	0	0	0	0	0	0	0	0	0
ENSG00000216853	0	0	0	0	0	0	0	0	0	0	0	0	0	0	0	0	0
ENSG00000216854	0.11656325	0.11711387	0	0.0753358	0.06502146	0.087437	0	0.17126195	0.02881393	0.05278699	0	0.04921879	0.04933015	0.12627755	0	0.05622293	0
ENSG00000216859	0	0	0	0	0.2231366	0.04286583	0	0	0	0	0	0	0	0	0	0	0
ENSG00000216863	0	0.00288147	0	0	0	0	0	0	0	0	0	0	0.00364116	0.0046604	0.00374134	0	0
ENSG00000216866	0.12823504	0.14315644	0.16721542	0.1105058	0.17883059	0.09619229	0.12014605	0.16747632	0.21132766	0.13550295	0.10770224	0.19853972	0.09044951	0.02315367	0.07435038	0.08247024	0.06430402
ENSG00000216867	0	0	0	0	0	0	0	0	0	0	0	0	0	0	0	0	0
ENSG00000216895	0.80156866	0.93212395	0.90368527	0.7627002	0.68311843	0.93531913	0.52749246	0.59976193	0.49536079	1.09908169	0.54699346	0.68632584	0.60307172	0.67539949	0.57125234	0.69807714	0.58617721
ENSG00000216901	0.13236033	0.17731409	0.45306108	0.4277279	0.18458352	0.19857355	0	0	0.0654378	0.11988175	0	0.0558891	0.05601555	0.14339114	0.23022664	0	0
ENSG00000216902	0	0	0	0	0	0	0	0	0	0	0	0	0	0	0	0	0
ENSG00000216904	0	0	0	0	0	0.01524359	0	0	0.01004672	0	0	0	0	0	0	0	0
ENSG00000216906	0.01481146	0.0198419	0.02897069	0.0191455	0.0165243	0.03333134	0.19031539	0.14507944	0.06590393	0.10732061	0.14927848	0.23765709	0.02507314	0.03209167	0.02576295	0	0.04456363
ENSG00000216913	0	0	0	0	0	0	0	0	0	0	0	0	0	0	0	0	0
ENSG00000216915	0.02155835	0.04332038	0.04216737	0	0	0	0.36357241	0.29563212	0.28777314	0.11715534	0.38023574	0.32770825	0.07298882	0.07006503	0.05624768	0.08318738	0.09729477
ENSG00000216917	0	0	0.05324342	0	0	0	0	0	0	0	0	0	0	0	0	0	0
ENSG00000216921	0	0	0	0	0	0	0	0	0	0	0	0	0	0	0	0	0
ENSG00000216937	0.41001689	0.40710721	0.43637055	0.4894657	0.32423878	0.45953661	0.13558351	0.20080741	0.13593415	0.34077888	0.21573493	0.21793946	0.29192388	0.27957677	0.28107705	0.25593362	0.30115049
ENSG00000216938	0	0.0569604	0.04158325	0.164884	0.02371825	0.15947447	0.06829259	0.04164814	0	0	0.16070084	0.03590764	0	0.04606298	0	0	0
ENSG00000216966	0	0	0	0	0	0	0	0	0	0	0	0	0	0	0	0	0
ENSG00000216977	0.27665013	0.32428312	0.13527943	0.6258045	0.231482	0.41504428	0.61096957	0.4064715	0.30774023	0.25056829	0.522795	0.35044623	0.35123913	0.14985297	0.12030083	0.46703642	0.41618223
ENSG00000216990	0.03846031	0.03864199	0.03761349	0.0994289	0.05363495	0.10097512	0	0.03767218	0.03802889	0.05225156	0.04845316	0.0324797	0.01627659	0	0.0334488	0.03710177	0.0867874
ENSG00000216998	0	0	0	0	0	0	0	0	0	0	0	0	0	0	0	0	0
ENSG00000217004	0	0	0	0	0	0	0	0	0	0	0	0	0	0	0	0	0
ENSG00000217026	0	0.1340869	0.04894426	0.3234527	0.05583365	0.18770442	0.08038165	0.09804126	0.123712	0.09065592	0.12609859	0.12679188	0	0.16265097	0.08704998	0.09655678	0.07528762
ENSG00000217027	0.31049846	0.2911675	0.42512668	0.0802711	0.06928106	0.32607766	0.0997414	0.18248143	0.09210466	0.0562451	0.07823458	0.05244314	0.15768539	0.33637516	0.05400789	0.11981226	0
ENSG00000217030	0.07619324	0.05103544	0	0.295466	0.04250221	0.11430889	0.12237774	0.4477913	0.07533856	0.06900994	0.38395977	0.06434513	0	0.16508625	0.066265	0.07350185	0
ENSG00000217041	0	0	0	0	0	0	0	0	0	0	0	0	0	0	0	0	0
ENSG00000217044	0	0	0	0	0	0	0	0	0	0	0	0	0	0	0	0	0
ENSG00000217060	0	0.02715471	0	0	0.01130719	0	0.01627855	0	0	0	0	0.01711823	0	0	0	0	0
ENSG00000217067	0	0	0	0	0	0	0	0	0	0	0	0	0	0	0	0	0
ENSG00000217075	0.14990577	0.05020463	0.07330257	0	0	0	0.12038555	0.07341695	0.07411212	0.06788653	0.18885463	0.12659531	0.25376346	0	0.06518626	0.07230531	0
ENSG00000217078	0	0	0	0	0	0	0	0	0	0	0	0	0	0	0	0	0
ENSG00000217083	0	0	0	0	0	0	0	0	0	0	0	0	0	0	0	0	0
ENSG00000217085	0	0	0	0	0	0	0	0	0	0	0	0	0	0	0	0	0
ENSG00000217089	0	0	0	0	0	0	0	0	0	0	0	0	0	0	0	0	0
ENSG00000217094	0.26044234	0.26167261	0.12735397	0.2524891	0.43584085	0.14652321	0.10457734	0.19132901	0.09657034	0.23588854	0.49216661	0.10997168	0.11022049	0	0.05662645	0.06281067	0.09794996
ENSG00000217120	0.18629907	0.32756344	0.18219715	0.18061	0.14289219	0.15721601	0.1496121	0.22810178	0.20723548	0.10545956	0.1760278	0.07866471	0.03942135	0.25228137	0.02025296	0.0898592	0.21019608
ENSG00000217128	2.66719174	3.11228141	3.06959278	3.677349	2.40739675	3.18585398	2.68168187	2.67974458	2.55290133	2.26913465	2.77725273	2.67886004	4.36913945	4.07118423	4.17567148	4.88458482	3.78480692
ENSG00000217130	0.32359176	0.04334938	0.18988016	0.5019362	0.1444051	0.24273423	0.2598684	0.38035285	0.28796576	0.05861688	0.16306725	0.1639638	0.10955652	0.21033579	0.11257066	0.31216147	0.1947198
ENSG00000217135	0	0	0	0	0	0	0	0	0	0	0	0	0	0	0	0	0
ENSG00000217139	0	0.01601483	0	0	0	0	0	0	0.01182055	0	0	0	0	0	0	0	0
ENSG00000217159	0.11037582	0.07393147	0.21589114	0.1426736	0.06156998	0.33118259	0	0.108114	0.05456885	0.39987954	0.13905392	0.0932123	0.37369277	0	0.19198694	0.53238496	0
ENSG00000217160	0	0	0	0	0	0	0	0	0	0	0	0	0	0	0	0	0
ENSG00000217165	0	0	0	0	0	0	0	0	0	0	0	0	0	0	0	0	0
ENSG00000217169	0	0	0	0	0	0	0	0	0	0	0	0	0	0	0	0	0
ENSG00000217178	0	0	0	0	0	0	0	0	0	0	0	0	0	0	0	0	0
ENSG00000217179	0	0	0	0	0	0	0	0	0	0	0	0	0	0	0	0	0
ENSG00000217181	0	0	0	0	0	0	0	0	0	0	0	0	0	0	0	0	0
ENSG00000217195	0	0	0	0	0	0	0	0	0	0	0	0	0	0	0	0	0
ENSG00000217227	0	0.01788566	0	0	0	0	0	0	0	0	0	0	0	0	0	0	0
ENSG00000217228	0	0	0	0	0	0	0	0	0	0	0	0	0	0	0	0	0
ENSG00000217231	0	0	0	0	0	0	0	0	0	0	0	0	0	0	0	0	0
ENSG00000217236	0	0.03381048	0.01234147	0	0	0	0	0	0.01871665	0.29716968	0	0.010657	0.0640867	0.10936802	0.16462467	0.06086782	0.03796807
ENSG00000217239	0.09635199	0.70991897	0.28269154	0.5604579	0.34935673	0.28910409	0.88984534	0.80220908	0.71453388	0.78541362	0.7283183	0.44753049	0.12232992	0.15657283	0.37708646	1.06890805	0.79721603
ENSG00000217241	0.05838721	0	0.05710164	0.0754723	0.2279874	0.17519079	0	0	0	0	0.07355751	0	0	0	0.05077916	0	0
ENSG00000217258	0.26096955	0.26220231	0.12761177	0.1686668	0.07278719	0.04893994	0.05239452	0.25562176	0.16127637	0.94546417	0.24658145	0.16529144	0.11044361	0.07067964	0.11348216	0.18881345	0
ENSG00000217261	0	0	0	0	0	0	0	0	0	0	0	0	0	0	0	0	0
ENSG00000217268	0	0	0	0	0	0	0	0	0	0	0	0	0	0	0	0	0

ENSG00000217272	0	0	[illegible]
ENSG00000217275	0.06458866	0.30283754	[illegible]
ENSG00000217289	0.0279772	0.01873958	[illegible]
ENSG00000217314	0	0	[illegible]
ENSG00000217315	0	0.02374916	[illegible]
ENSG00000217325	0.16496348	0.08287136	[illegible]
ENSG00000217327	0.05547287	0	[illegible]
ENSG00000217330	0	0	[illegible]
ENSG00000217331	0	0	[illegible]
ENSG00000217334	0	0	[illegible]
ENSG00000217372	0	0	[illegible]
ENSG00000217377	0	0	[illegible]
ENSG00000217379	0	0.04632616	[illegible]
ENSG00000217385	0	0	[illegible]
ENSG00000217404	0	0.08303073	[illegible]
ENSG00000217408	0	0	[illegible]
ENSG00000217414	0	0.01075635	[illegible]
ENSG00000217416	0	0	[illegible]
ENSG00000217442	0.16318856	0.08197971	[illegible]
ENSG00000217447	0	0	[illegible]
ENSG00000217455	0	0.0147308	[illegible]
ENSG00000217477	0	0	[illegible]
ENSG00000217482	0	0	[illegible]
ENSG00000217483	0	0	[illegible]
ENSG00000217488	0	0	[illegible]
ENSG00000217495	0	0	[illegible]
ENSG00000217512	0.36051163	0.77272449	[illegible]
ENSG00000217514	0	0	[illegible]
ENSG00000217527	0.15955317	0.21374248	[illegible]
ENSG00000217539	0	0.01321984	[illegible]
ENSG00000217555	3.86333498	2.25856059	[illegible]
ENSG00000217557	0	0	[illegible]
ENSG00000217566	0	0	[illegible]
ENSG00000217576	0	0	[illegible]
ENSG00000217585	0	0	[illegible]
ENSG00000217612	0	0.03446725	[illegible]
ENSG00000217624	0.04384999	0.05874283	[illegible]
ENSG00000217631	0	0	[illegible]
ENSG00000217643	0	0	[illegible]
ENSG00000217644	0	0	[illegible]
ENSG00000217646	0.1895867	0.06349409	[illegible]
ENSG00000217648	0.59474915	0.70189425	[illegible]
ENSG00000217653	0	0	[illegible]
ENSG00000217680	0	0	[illegible]
ENSG00000217684	0	0	[illegible]
ENSG00000217686	0	0	[illegible]
ENSG00000217702	0.10475538	0.04677788	[illegible]
ENSG00000217707	0	0	[illegible]
ENSG00000217716	0.06524239	0.08740077	[illegible]
ENSG00000217718	0	0	[illegible]
ENSG00000217733	0	0	[illegible]
ENSG00000217746	0	0	[illegible]
ENSG00000217767	0.1401293	0.18772166	[illegible]
ENSG00000217769	0	0	[illegible]
ENSG00000217770	0	0	[illegible]
ENSG00000217776	0	0	[illegible]
ENSG00000217783	0	0.01995193	[illegible]
ENSG00000217786	0	0	[illegible]
ENSG00000217791	0	0	[illegible]
ENSG00000217801	0.36954063	0.49019493	[illegible]
ENSG00000217805	0	0	[illegible]
ENSG00000217809	0	0	[illegible]
ENSG00000217811	0	0	[illegible]
ENSG00000217824	0	0	[illegible]
ENSG00000217825	0	0	[illegible]
ENSG00000217835	0.03549531	0.14265192	[illegible]
ENSG00000217862	0	0.24439235	[illegible]
ENSG00000217874	0	0	[illegible]
ENSG00000217878	0	0	[illegible]
ENSG00000217889	0	0	[illegible]
ENSG00000217896	0	0	[illegible]
ENSG00000217897	0	0	[illegible]
ENSG00000217929	0	0.00767709	[illegible]
ENSG00000217930	0.06987278	0.03448561	[illegible]
ENSG00000217950	0.0151848	0.03051306	[illegible]
ENSG00000218014	0	0	[illegible]
ENSG00000218016	0	0.01560954	[illegible]
ENSG00000218018	0.0209284	0.01401818	[illegible]
ENSG00000218020	0	0	[illegible]
ENSG00000218027	0	0	[illegible]
ENSG00000218029	0	0	[illegible]
ENSG00000218048	0	0.02230164	[illegible]
ENSG00000218049	0	0	[illegible]
ENSG00000218052	0.09142506	0.15792946	[illegible]
ENSG00000218069	0.13074945	0.04378903	[illegible]
ENSG00000218073	0	0	[illegible]
ENSG00000218089	0	0	[illegible]
ENSG00000218107	0	0	[illegible]
ENSG00000218109	0	0.0122104	[illegible]
ENSG00000218125	0	0	[illegible]
ENSG00000218143	0	0	[illegible]
ENSG00000218153	0	0.01602672	[illegible]
ENSG00000218173	0	0	[illegible]
ENSG00000218175	0.56051721	0.75088663	[illegible]
ENSG00000218180	0	0.02409374	[illegible]
ENSG00000218186	0	0	[illegible]
ENSG00000218187	0	0	[illegible]
ENSG00000218189	0.02068661	0	[illegible]
ENSG00000218194	0	0	[illegible]
ENSG00000218198	0	0.03275871	[illegible]
ENSG00000218208	0.07210233	0.14488584	[illegible]
ENSG00000218213	0	0	[illegible]
ENSG00000218226	0	0.00969811	[illegible]
ENSG00000218227	0.27267123	0.32875113	[illegible]
ENSG00000218233	0	0.03149233	[illegible]
ENSG00000218261	0	0	[illegible]
ENSG00000218265	0	0	[illegible]
ENSG00000218274	0	0	[illegible]
ENSG00000218281	0	0	[illegible]
ENSG00000218283	0.03332962	0.02232471	[illegible]
ENSG00000218300	0	0	[illegible]
ENSG00000218305	0	0	[illegible]
ENSG00000218313	0	0	[illegible]
ENSG00000218336	4.57477905	4.41903799	[illegible]
ENSG00000218337	0	0	[illegible]
ENSG00000218346	0.06938588	0.09295152	[illegible]
ENSG00000218347	1.05839188	1.48646121	[illegible]
ENSG00000218350	0	0	[illegible]
ENSG00000218351	0	0	[illegible]
ENSG00000218357	0	0	[illegible]
ENSG00000218358	0.04833857	0.03237794	[illegible]
ENSG00000218359	0	0	[illegible]
ENSG00000218363	0	0	[illegible]
ENSG00000218410	0	0	[illegible]
ENSG00000218416	0.02152955	0.00961389	[illegible]
ENSG00000218418	0.04359789	0.02555224	[illegible]
ENSG00000218422	0.72637	0.76119051	[illegible]
ENSG00000218424	0	0	[illegible]
ENSG00000218426	0.21630698	0.38636225	[illegible]
ENSG00000218428	0	0	[illegible]
ENSG00000218454	0.0871074	0	[illegible]
ENSG00000218459	0.12789579	0.08566663	[illegible]
ENSG00000218472	0	0	[illegible]
ENSG00000218475	0	0	[illegible]
ENSG00000218476	0	0	[illegible]
ENSG00000218483	0	0	[illegible]
ENSG00000218490	0	0	[illegible]
ENSG00000218499	0	0	[illegible]
ENSG00000218502	0.08328098	0.11156584	[illegible]
ENSG00000218510	1.01786496	1.14518085	[illegible]
ENSG00000218512	0	0	[illegible]
ENSG00000218520	0	0	[illegible]
ENSG00000218521	0	0	[illegible]
ENSG00000218536	0	0.03156139	[illegible]
ENSG00000218537	0.34937387	0.71667448	[illegible]
ENSG00000218549	0	0	[illegible]
ENSG00000218561	0	0	[illegible]
ENSG00000218565	0	0	[illegible]
ENSG00000218574	0.09978248	0	[illegible]
ENSG00000218577	0	0	[illegible]
ENSG00000218582	0.1280228	0.15006548	[illegible]
ENSG00000218586	0.08621419	0.12511987	[illegible]
ENSG00000218596	0.42631931	0.3997776	[illegible]
ENSG00000218617	0	0	[illegible]
ENSG00000218631	0.05232101	0.14018176	[illegible]
ENSG00000218632	0	0.02917296	[illegible]
ENSG00000218643	0	0	[illegible]
ENSG00000218672	0.02077997	0	[illegible]
ENSG00000218676	0	0	[illegible]
ENSG00000218682	0.12739028	0.08532803	[illegible]
ENSG00000218689	0	0	[illegible]
ENSG00000218690	0	0	[illegible]
ENSG00000218698	0	0	[illegible]
ENSG00000218713	0.09208497	0.2364399	[illegible]
ENSG00000218716	0	0	[illegible]
ENSG00000218725	0	0	[illegible]
ENSG00000218728	0	0	[illegible]
ENSG00000218730	0.15876719	0.10634478	[illegible]
ENSG00000218732	0	0	[illegible]
ENSG00000218739	8.28752892	9.08617252	[illegible]
ENSG00000218748	0	0.08303073	[illegible]
ENSG00000218749	0	0	[illegible]
ENSG00000218754	0	0	[illegible]
ENSG00000218757	0	0	[illegible]
ENSG00000218766	0	0.04216404	[illegible]
ENSG00000218772	0	0	[illegible]
ENSG00000218776	0	0	[illegible]
ENSG00000218792	0	0.05479185	[illegible]
ENSG00000218793	0	0	[illegible]
ENSG00000218803	0	0	[illegible]
ENSG00000218806	0	0	[illegible]

ENSG00000218809	0	0	0	0	0	0	0	0	[illegible]
ENSG00000218813	0	0	0	0	0	0	0	0	[illegible]
ENSG00000218819	0	0	0	0	0	0	0	0	[illegible]
ENSG00000218823	0.03781058	0.04558703	0.01479123	0	0.02530986	0.01134506	0	0	[illegible]
ENSG00000218834	0	0	0	0.2192669	0	0	0	0	[illegible]
ENSG00000218839	0	0	0	0	0	0	0	0	[illegible]
ENSG00000218857	0	0	0	0	0	0	0	0	[illegible]
ENSG00000218868	0	0	0	0	0	0	0	0	[illegible]
ENSG00000218870	0	0	0	0	0	0	0.0332685	0	[illegible]
ENSG00000218872	0	0	0	0	0	0	0	0	[illegible]
ENSG00000218890	0	0	0	0	0	0	0	0	[illegible]
ENSG00000218891	3.62324458	3.64035997	3.24418872	3.9239856	2.73807537	3.70954693	3.97139712	4.30435776	[illegible]
ENSG00000218893	0	0	0	0	0	0	0	0	[illegible]
ENSG00000218896	0	0	0	0	0	0	0	0	[illegible]
ENSG00000218902	0.09766588	0	0	0	0.05448011	0	0	0.09566451	[illegible]
ENSG00000218965	0	0	0	0	0	0.03969841	0	0	[illegible]
ENSG00000218976	0	0	0	0	0	0	0	0	[illegible]
ENSG00000218980	0.11510621	0	0	0	0	0	0.27731671	0.11274745	[illegible]
ENSG00000218986	0	0	0	0	0	0	0	0	[illegible]
ENSG00000218991	0	0	0	0	0	0	0	0	[illegible]
ENSG00000218996	0.16141773	0.10812015	0.26310607	0.2086513	0.15007041	0.24216691	0.21605086	0.42162654	[illegible]
ENSG00000219016	0	0	0	0	0	0	0	0	[illegible]
ENSG00000219023	0.08221872	0.0550714	0	0.1062773	0.04586335	0	0	0	[illegible]
ENSG00000219027	0.04274501	0.05726257	0.16721542	0.1105058	0	0.25651278	0.27461955	0.29308356	[illegible]
ENSG00000219039	0	0	0.06580398	0	0	0	0	0	[illegible]
ENSG00000219041	0	0	0	0	0.03001408	0	0	0	[illegible]
ENSG00000219061	0.02382095	0	0	0	0	0	0	0	[illegible]
ENSG00000219073	0.02343981	0	0	0	0.00653761	0	0	0	[illegible]
ENSG00000219074	0	0	0	0	0	0	0	0	[illegible]
ENSG00000219085	0.07375226	0.22230196	0.0360642	0.0953334	0.08228117	0.11064682	0.11845717	0.18060233	[illegible]
ENSG00000219087	0	0	0	0	0	0	0	0	[illegible]
ENSG00000219088	0	0.01290376	0	0	0	0	0	0	[illegible]
ENSG00000219095	0	0	0	0	0	0	0	0	[illegible]
ENSG00000219102	0.06063921	0.0406171	0	0	0.01691292	0	0	0.02969829	[illegible]
ENSG00000219133	0.20018472	0.1340869	0.06525902	0.4312702	0.14888973	0.45049061	0.16076331	0.06536084	[illegible]
ENSG00000219135	0.66044549	0.53085223	0.90426535	0.6829623	0.69998005	0.39633327	0.31823229	0.45283813	[illegible]
ENSG00000219139	0	0	0	0	0	0	0	0	[illegible]
ENSG00000219146	0	0	0	0.0540346	0	0.03135711	0.03357055	0.08189179	[illegible]
ENSG00000219149	0.06577498	0	0.06432675	0.0850218	0.07338137	0.14801834	0	0.06442712	[illegible]
ENSG00000219150	0	0.10582348	0.30902065	0	0.26438875	0	0	0	[illegible]
ENSG00000219159	0	0	0.007251	0	0	0	0	0	[illegible]
ENSG00000219163	0.99974082	0.70152997	1.21052108	0.3692234	1.06224136	0.46424266	0.15292699	0.18652459	[illegible]
ENSG00000219186	0.06104117	0.08177269	0.05969717	0.1578057	0.03405007	0.0457885	0	0	[illegible]
ENSG00000219188	0	0	0	0	0	0	0	0	[illegible]
ENSG00000219190	0	0	0	0	0	0	0	0	[illegible]
ENSG00000219200	0.15823386	0.14224645	0.17103934	0.2314483	0.08594342	0.19678408	0.28089961	0.27735291	[illegible]
ENSG00000219201	0	0	0	0	0	0	0	0	[illegible]
ENSG00000219222	0	0.33996836	0	0	0.14156248	0.3807296	0	0	[illegible]
ENSG00000219240	0	0	0	0	0	0	0	0	[illegible]
ENSG00000219249	0	0	0	0	0	0	0	0	[illegible]
ENSG00000219253	0	0	0	0	0	0	0	0	[illegible]
ENSG00000219257	0	0	0	0	0.02078432	0	0	0	[illegible]
ENSG00000219262	0	0	0	0	0	0	0	0	[illegible]
ENSG00000219273	0	0	0	0	0.01004382	0.01350633	0.01445972	0.01763647	[illegible]
ENSG00000219274	0	0.05549612	0.1620571	0.1070969	0	0.12429989	0.133074	0	[illegible]
ENSG00000219280	0	0	0	0	0	0	0	0	[illegible]
ENSG00000219284	0	0	0	0	0	0	0	0	[illegible]
ENSG00000219294	0	0.00667326	0.00974346	0.0128781	0	0.00747336	0.00800089	0.00975867	[illegible]
ENSG00000219297	0	0	0	0	0	0	0	0	[illegible]
ENSG00000219298	0	0	0	0	0	0	0	0	[illegible]
ENSG00000219302	0	0	0	0	0	0	0	0	[illegible]
ENSG00000219314	0	0	0	0	0	0	0	0	[illegible]
ENSG00000219329	0.23785786	0.07966048	0.23262071	0.2305943	0.19902327	0.13381732	0.14326324	0.05824592	[illegible]
ENSG00000219355	0	0.11764573	0	0	0.04898756	0.26350223	0.07052559	0.08601986	[illegible]
ENSG00000219361	0	0	0	0	0	0	0	0	[illegible]
ENSG00000219368	0	0	0	0	0	0	0	0	[illegible]
ENSG00000219374	0	0	0	0	0	0	0	0	[illegible]
ENSG00000219375	0	0	0	0	0	0	0	0	[illegible]
ENSG00000219384	0	0	0	0	0	0	0	0	[illegible]
ENSG00000219387	0	0	0.03400227	0	0	0	0	0	[illegible]
ENSG00000219391	0	0	0	0	0.06888289	0	0	0	[illegible]
ENSG00000219392	0	0	0	0	0	0	0	0	[illegible]
ENSG00000219395	0	0.08239691	0.16040767	0	0.09149331	0.09227607	0.592738	0.68279628	[illegible]
ENSG00000219404	0	0	0	0	0	0	0	0	[illegible]
ENSG00000219409	0	0.05437781	0.07939573	0	0.02264287	0	0	0	[illegible]
ENSG00000219410	0.05071556	0.03397009	0	0.0655558	0.02829022	0.09510751	0	0.0496763	[illegible]
ENSG00000219430	0	0	0	0.1293811	0	0	0	0	[illegible]
ENSG00000219433	0	0.15967449	0.09325475	0.1848848	0.07978595	0.21458281	0.34459472	0.46700128	[illegible]
ENSG00000219435	0.0157065	0	0	0.0203025	0.00876142	0.02356367	0.0126135	0.01538464	[illegible]
ENSG00000219438	0	0	0	0	0	0	0.05239452	0.02457901	[illegible]
ENSG00000219445	0	0.01125547	0.01643384	0	0.01874706	0.05041987	0	0	[illegible]
ENSG00000219448	0.08071405	0.18714305	0.20037825	0.1123579	0.0727311	0.15369271	0.08974996	0.15203856	[illegible]
ENSG00000219451	0.15060626	0.15131769	0	0.1946762	0.08401138	0.39540726	0.42331834	0.73760016	[illegible]
ENSG00000219453	0	0	0	0	0	0	0	0	[illegible]
ENSG00000219463	0	0	0	0	0	0	0	0	[illegible]
ENSG00000219470	0.18270828	0.19580944	0.10721125	0.1417031	0.22422084	0.21928643	0.55756798	0.28634274	[illegible]
ENSG00000219481	1.18682768	1.78520158	1.40554261	1.2944722	0.8096483	1.24209755	1.52192427	1.70107858	[illegible]
ENSG00000219487	0	0	0	0	0	0	0	0.04754411	[illegible]
ENSG00000219491	0	0	0	0	0	0	0	0	[illegible]
ENSG00000219492	0	0	0	0	0	0	0	0	[illegible]
ENSG00000219500	0	0	0	0	0	0	0	0	[illegible]
ENSG00000219507	0.87785283	0.4619998	0.61323165	1.0536753	0.7345275	0.61146358	0.75533733	0.85986384	[illegible]
ENSG00000219529	0	0.2412066	0	0.1163707	0	0.13506329	0.4337915	0.44091183	[illegible]
ENSG00000219532	0	0	0	0	0	0	0	0	[illegible]
ENSG00000219545	3.02040771	2.69345112	2.70037576	2.4313766	2.93587238	2.24307134	2.44497957	2.57467435	[illegible]
ENSG00000219547	0	0.11669184	0	0.0750643	0.03239358	0.08712191	0	0.1137632	[illegible]
ENSG00000219549	0	0	0	0	0.03602893	0	0	0	[illegible]
ENSG00000219553	0	0.05971782	0.08719255	0	0.02486644	0.03343891	0	0	[illegible]
ENSG00000219559	0.07308331	0	0	0	0	0	0	0	[illegible]
ENSG00000219565	0	0	0	0	0.00572835	0	0	0.00502936	[illegible]
ENSG00000219575	0	0	0	0	0	0	0	0	[illegible]
ENSG00000219582	0	0.03556506	0	0	0	0.03982921	0	0	[illegible]
ENSG00000219592	0	0	0	0	0	0	0	0	[illegible]
ENSG00000219604	0	0	0	0	0	0	0	0	[illegible]
ENSG00000219607	2.15360012	0.99483827	1.57963668	1.8238556	1.61557365	2.57639455	0.23855201	0.2000358	[illegible]
ENSG00000219608	0.12492147	0.16734876	0.24434191	0	0	0.37482681	0.20064258	0.61180788	[illegible]
ENSG00000219619	0	0	0	0	0	0	0	0	[illegible]
ENSG00000219622	0	0	0	0	0	0	0	0	[illegible]
ENSG00000219626	1.20026115	1.79245186	1.5846757	1.5303212	0.9099538	1.50603288	0.85874684	0.96992392	[illegible]
ENSG00000219627	0	0	0	0	0	0	0	0	[illegible]
ENSG00000219642	0	0.02701876	0	0	0	0.01512912	0	0	[illegible]
ENSG00000219653	0	0	0	0	0	0	0	0	[illegible]
ENSG00000219665	0.25521287	0.32968085	0.32803731	0.3157542	0.3070977	0.36100401	0.53288308	0.67495421	[illegible]
ENSG00000219666	0	0	0	0	0	0	0	0	[illegible]
ENSG00000219669	0.0396918	0	0	0	0	0	0	0	[illegible]
ENSG00000219681	0	0	0	0	0	0	0	0	[illegible]
ENSG00000219682	0	0.01520817	0	0	0	0	0.0091169	0	[illegible]
ENSG00000219693	0	0.07393147	0	0	0	0	0	0.108114	[illegible]
ENSG00000219699	0	0	0	0	0.04260293	0	0	0	[illegible]
ENSG00000219700	0	0	0	0	0	0	0	0	[illegible]
ENSG00000219702	0	0	0	0	0	0	0	0	[illegible]
ENSG00000219703	0	0	0	0	0.0327476	0	0	0	[illegible]
ENSG00000219712	0.07880132	0.0703765	0.10275503	0.1358132	0.13187116	0.13792527	0.08437781	0.10291536	[illegible]
ENSG00000219722	0	0	0	0	0	0	0	0	[illegible]
ENSG00000219736	0	0	0	0	0	0	0	0	[illegible]
ENSG00000219738	0	0	0	0	0	0	0	0	[illegible]
ENSG00000219747	0.0795796	0.63964416	0.38913712	0.8229275	0.44391198	0.65664104	0.44735864	0.38974428	[illegible]
ENSG00000219755	0	0	0	0.0559204	0	0	0.34742138	0.33899905	[illegible]
ENSG00000219757	0	0	0	0	0	0	0	0	[illegible]
ENSG00000219758	0	0.03137789	0	0	0	0.03514001	0	0.04588559	[illegible]
ENSG00000219770	0	0	0	0	0	0	0	0	[illegible]
ENSG00000219773	0.07375226	0.07410065	0	0	0.02057029	0.11064682	0	0.03612047	[illegible]
ENSG00000219776	0.06742623	0.09032632	0	0.0871563	0.03761179	0.15173428	0	0	[illegible]
ENSG00000219784	0	0	0	0	0	0	0	0	[illegible]
ENSG00000219790	0	0	0	0	0	0	0	0	[illegible]
ENSG00000219797	7.18373718	2.86105899	4.68371062	4.8520497	7.1119512	6.01980892	2.75460504	4.69101853	[illegible]
ENSG00000219806	0	0	0	0	0	0	0	0	[illegible]
ENSG00000219807	0	0	0	0	0	0	0	0	[illegible]
ENSG00000219814	0	0	0	0	0	0	0	0	[illegible]
ENSG00000219863	0	0	0	0	0	0	0	0.04330492	[illegible]
ENSG00000219867	0	0	0	0	0	0	0	0	[illegible]
ENSG00000219870	0	0	0	0	0	0	0	0	[illegible]
ENSG00000219881	0	0	0	0	0	0	0	0	[illegible]
ENSG00000219891	0.5433723	0.86310367	0.63769002	0.4013555	0.43300662	0.57063591	0.02493535	0.07603393	[illegible]
ENSG00000219902	0.08687261	0	0.08495986	0	0.04845939	0	0.06976521	0.76583176	[illegible]
ENSG00000219926	0	0	0	0	0	0	0	0	[illegible]
ENSG00000219928	0	0.12406891	0.36230008	0	0.10332434	0.27788884	0	0	[illegible]
ENSG00000219930	0	0	0	0	0	0	0	0	[illegible]
ENSG00000219932	0	0	0	0	0	0	0.02407711	0	[illegible]
ENSG00000219940	0	0	0	0	0	0.0933449	0	0	[illegible]
ENSG00000219941	0	0	0	0	0	0	0	0	[illegible]
ENSG00000219951	0.16212143	0.0434366	0.06342074	0.0838244	0.07234783	0.02432226	0	0	[illegible]
ENSG00000219986	0	0	0	0	0	0	0	0.06469116	[illegible]
ENSG00000219992	0.73921421	0.7427061	1.15670116	0.5733124	0.2886446	0.55450297	0.53427989	0.65165959	[illegible]
ENSG00000219993	0	0	0.09083604	0	0.05181105	0	0	0	[illegible]
ENSG00000220008	0	0	0	0	0	0	0	0	[illegible]
ENSG00000220030	0	0	0	0	0	0	0	0	[illegible]
ENSG00000220032	0.13965703	0.17669516	0.1062305	0.2607555	0.11252752	0.11640024	0.06230836	0.03039893	[illegible]
ENSG00000220069	0	0.02998332	0	0	0	0	0	0	[illegible]
ENSG00000220076	0	0	0	0	0	0	0	0	[illegible]
ENSG00000220091	0	0	0	0	0	0	0.1760741	0.10737853	[illegible]
ENSG00000220105	0.0533605	0.03574171	0	0	0	0	0.08570494	0.10453406	[illegible]
ENSG00000220110	0	0	0	0	0	0	0	0	[illegible]
ENSG00000220113	0	0	0	0	0	0	0	0	[illegible]
ENSG00000220125	0	0	0	0	0	0	0	0	[illegible]
ENSG00000220130	0	0	0	0.1560326	0.06733496	0	0	0	[illegible]
ENSG00000220131	0	0	0	0	0	0	0	0	[illegible]
ENSG00000220132	0	0	0	0	0	0	0	0	[illegible]
ENSG00000220139	0	0	0	0	0	0	0	0	[illegible]
ENSG00000220154	0	0	0	0	0	0	0	0	[illegible]
ENSG00000220157	0.20122626	0.20217681	0.29519351	0.1734056	0.18708049	0.30188965	0.16159975	0.16425227	[illegible]
ENSG00000220161	0.01722594	0.01153821	0.03369333	0.0445331	0.019218	0.05168643	0.11066977	0.10123769	[illegible]
ENSG00000220181	0	0	0	0	0	0	0	0	[illegible]
ENSG00000220184	0	0	0	0	0	0	0	0.05350727	[illegible]
ENSG00000220201	0.04070439	0.07088757	0.03184653	0.0526152	0.05449387	0.10992016	0.19613205	0.24719573	[illegible]
ENSG00000220204	0	0	0	0	0	0	0	0	[illegible]
ENSG00000220205	3.71122621	4.408845	3.51122657	4.6490809	3.35919406	4.67004743	1.04799388	0.94152919	[illegible]
ENSG00000220212	0	0	0	0	0	0	0	0	[illegible]
ENSG00000220237	0	0	0	0	0	0	0	0	[illegible]
ENSG00000220240	0	0	0	0	0.13122945	0	0	0	[illegible]
ENSG00000220248	0	0	0	0	0	0	0.01669864	0	[illegible]

ENSG00000220256	0.01990719	0.03333538	0.02920331	0.0385986	0.02776164	0.08959727	0.00799348	0.01949925	0.0344468	0.04507598	0.02507952	[illegible]
ENSG00000220267	0.02908821	0.19483746	0.02844775	0.0751998	0.30829446	0.13091875	0.16352008	0.08547641	0.05752385	0.02634585	0.14658392	[illegible]
ENSG00000220291	0	0	0	0	0	0	0	0	0	0	0	[illegible]
ENSG00000220305	0.02719809	0.21861256	0.13299623	0.0703134	0.10620173	0.10200983	0.10921052	0.13320374	0.17480453	0.24633929	0.47970669	[illegible]
ENSG00000220311	0	0	0	0	0	0	0	0	0	0	0	[illegible]
ENSG00000220323	0.06537473	0.26273417	0.38361185	0.1690089	0.21880448	0.29423525	0.0525008	0	0	0.05921137	0.08236054	[illegible]
ENSG00000220326	0	0	0	0	0	0	0	0	0	0	0	[illegible]
ENSG00000220340	0	0	0	0	0	0	0	0	0	0	0	[illegible]
ENSG00000220343	0.15332892	0.06162129	0.32989645	0.0396391	0.13684822	0.02300317	0.09850768	0.03003738	0.1212872	0.13887348	0.11590032	[illegible]
ENSG00000220347	0	0	0	0	0	0	0	0	0	0	0	[illegible]
ENSG00000220349	0	0	0	0	0	0	0	0	0	0	0	[illegible]
ENSG00000220370	4.70611244	3.24775079	3.62620696	3.1337696	3.1024733	5.34874544	3.66483436	3.49217775	3.38423479	5.03742055	5.0305525	[illegible]
ENSG00000220377	0	0	0	0	0	0	0	0	0	0	0	[illegible]
ENSG00000220378	0	0	0	0	0	0	0.018501	0	0	0	0.02902341	[illegible]
ENSG00000220392	0	0	0	0.086433	0	0	0	0	0	0	0.08424014	[illegible]
ENSG00000220412	0.10870064	0.06067451	0.03543576	0	0.02021184	0.01358984	0.02909825	0.10647314	0.04478388	0	0.04564783	[illegible]
ENSG00000220418	0	0	0	0	0.01349732	0.0181504	0	0	0	0	0.03048329	[illegible]
ENSG00000220446	0	0	0	0	0	0	0	0	0	0	0	[illegible]
ENSG00000220447	0	0	0	0	0	0	0	0	0	0	0	[illegible]
ENSG00000220472	0.04415033	0.05914518	0	0.1141389	0.02462799	0.09935478	0.07091203	0.25947359	0.06548263	0.07997591	0.11124314	[illegible]
ENSG00000220483	0	0	0	0	0	0	0	0.07248975	0	0	0	[illegible]
ENSG00000220494	0	0	0	0	0	0.01735558	0.01858068	0.04532561	0.04575479	0.23051204	0.02914842	[illegible]
ENSG00000220505	0	0	0	0	0	0	0	0	0	0	0	[illegible]
ENSG00000220506	0	0	0	0	0	0	0	0	0	0	0	[illegible]
ENSG00000220514	0	0	0	0	0	0	0	0	0	0	0	[illegible]
ENSG00000220515	0	0.08510377	0	0.0547447	0.02362475	0.03176916	0.30610517	0.04148395	0.12563027	0.23015406	0.2134231	[illegible]
ENSG00000220517	0	0	0	0	0	0	0	0.02552085	0	0	0	[illegible]
ENSG00000220522	0	0	0	0	0	0	0	0	0	0	0	[illegible]
ENSG00000220537	0	0	0	0	0	0	0	0	0	0	0	[illegible]
ENSG00000220540	0	0	0	0	0	0	0	0	0	0	0	[illegible]
ENSG00000220541	0	0.02133201	0	0	0	0	0	0	0.01574516	0	0	[illegible]
ENSG00000220548	0	0	0	0	0	0	0	0	0	0	0	[illegible]
ENSG00000220553	0	0	0	0	0	0	0	0	0	0	0	[illegible]
ENSG00000220556	0	0	0	0	0	0	0	0	0	0	0	[illegible]
ENSG00000220557	0	0	0	0	0	0	0	0	0	0	0	[illegible]
ENSG00000220563	0.54091871	1.14733376	0.92576537	0.8740007	0.50289329	0.71007401	0.03619985	0.13245855	0.1782837	0	0.17036536	[illegible]
ENSG00000220575	0.00691774	0.02316805	0.01353085	0	0.00771772	0.00518917	0.01666638	0.00677598	0.00342007	0.0125311	0.00871512	[illegible]
ENSG00000220581	0	0	0	0	0	0	0	0	0	0	0	[illegible]
ENSG00000220583	0.08878716	0.05947105	0.26049675	0.2295355	0.09905474	0.13320292	0	0.17393546	0.08779121	0.24124964	0.33556814	[illegible]
ENSG00000220585	0	0	0	0	0.01519732	0	0	0	0	0	0	[illegible]
ENSG00000220586	0	0	0	0	0	0	0.02355131	0	0	0	0	[illegible]
ENSG00000220598	0	0	0	0	0	0	0	0	0	0	0	[illegible]
ENSG00000220600	0	0.05493127	0	0	0	0	0.06585978	0.04016449	0.0202724	0.14855576	0	[illegible]
ENSG00000220614	0	0.06293875	0	0.1214598	0.10483052	0.07048493	0.07546033	0.18407747	0.04645512	0	0.11837827	[illegible]
ENSG00000220635	0	0	0	0	0	0	0	0	0	0	0	[illegible]
ENSG00000220643	0	0	0	0	0	0	0	0	0	0	0	[illegible]
ENSG00000220660	0	0	0	0	0	0	0	0	0	0	0	[illegible]
ENSG00000220666	0	0.01707911	0	0	0	0	0.02047697	0.0249757	0.02521219	0	0.03212322	[illegible]
ENSG00000220685	0.10097036	0.09468417	0.05924832	0.0522064	0.0675881	0.0605923	0.08108676	0.11868153	0.17970796	0.10974138	0.10176377	[illegible]
ENSG00000220693	0.15713308	0.24558386	0.13659851	0.1354086	0.17530435	0.36670489	0.37856885	0.34202911	0.29347757	0.34789087	0.19795975	[illegible]
ENSG00000220694	0	0	0	0	0	0	0.04672002	0	0	0	0.14658392	[illegible]
ENSG00000220695	0	0.02778377	0.08113284	0	0	0	0	0	0.04101443	0.03756912	0	[illegible]
ENSG00000220702	0	0.03562375	0	0	0	0	0	0	0	0	0	[illegible]
ENSG00000220721	0	0	0	0	0	0	0	0	0	0	0	[illegible]
ENSG00000220725	0	0	0	0	0	0	0	0	0	0	0	[illegible]
ENSG00000220730	0	0	0	0	0	0	0	0	0	0	0	[illegible]
ENSG00000220733	0	0	0	0	0	0	0	0	0	0	0	[illegible]
ENSG00000220734	0	0	0	0	0	0	0	0	0	0	0	[illegible]
ENSG00000220739	0	0	0	0	0	0	0	0	0	0	0	[illegible]
ENSG00000220744	0.10851764	0.2665184	0.14150441	0.0467572	0.1210669	0.10853571	0.52288672	0.56690077	0.3934347	0.45867215	0.54685179	[illegible]
ENSG00000220745	0	0	0	0	0	0	0	0	0	0	0	[illegible]
ENSG00000220748	0	0.02030855	0	0	0.01691292	0	0	0	0	0	0	[illegible]
ENSG00000220749	0.18847801	0.02104093	0.12288541	0.2030249	0.07009136	0.23563674	0.17658894	0.12307714	0.0931819	0.02845147	0.2374488	[illegible]
ENSG00000220758	0	0	0	0	0	0	0	0	0	0	0	[illegible]
ENSG00000220771	0.25179484	1.01193706	0.61562708	0.3254742	0.42136957	0.75551029	0.2022101	0	0.0622426	0.2280563	0	[illegible]
ENSG00000220773	0	0	0.03565623	0	0	0.02734879	0.02927929	0.03571186	0	0	0	[illegible]
ENSG00000220785	0.05359713	0.11667567	0.06988937	0.1789747	0.06726964	0.17757005	0.10760626	0.03937411	0.03753877	0.04854413	0.04501524	[illegible]
ENSG00000220793	0.06301024	0	0	0.040724	0	0.0945311	0	0	0	0.0285349	0.03969086	[illegible]
ENSG00000220804	0.25857342	0.30790502	0.35403222	0.2748165	0.28847551	0.24568564	0.2399555	0.2082481	0.1903343	0.10929138	0.44882015	[illegible]
ENSG00000220831	0	0	0	0	0	0	0	0	0	0	0	[illegible]
ENSG00000220842	0	0	0.03075132	0	0	0	0	0	0	0	0	[illegible]
ENSG00000220848	1.40129303	0.89167787	1.57600533	1.9924684	1.21159019	2.3650757	0.95654168	1.85297988	1.62804991	1.26918288	0.97095912	[illegible]
ENSG00000220867	0	0.15700357	0.34385571	0	0.26150451	0.26374178	1.60003336	1.26277147	1.50649724	0.74304889	1.18119986	[illegible]
ENSG00000220868	0.0563457	0	0.05510508	0	0.03143083	0.04226631	0.09049962	0.2759553	0.02785683	0	0	[illegible]
ENSG00000220871	0	0	0	0	0	0	0	0	0	0	0	[illegible]
ENSG00000220875	0.54102681	0.15530928	0.5291145	0.4995288	0.43113753	0.40583766	0.37241572	0.45423434	0.34390156	0.14000579	0.09737109	[illegible]
ENSG00000220884	0	0	0	0	0	0.02432226	0.02603913	0.06351969	0.01603029	0	0	[illegible]
ENSG00000220891	0.02071986	0.02775698	0.03039547	0.0535657	0.02889495	0.06216992	0.04159899	0.08118107	0.03073116	0.05629934	0.07831002	[illegible]
ENSG00000220908	0	0	0	0	0	0	0	0	0	0	0	[illegible]
ENSG00000220913	0	0	0	0	0	0	0	0	0	0	0	[illegible]
ENSG00000220918	0	0	0.03321402	0	0	0	0	0	0	0	0	[illegible]
ENSG00000220920	0	0	0	0	0	0	0	0	0	0	0	[illegible]
ENSG00000220924	0	0	0	0	0	0	0	0.07031022	0	0	0	[illegible]
ENSG00000220925	0.12565201	0.12624556	0.0614427	0	0	0.0942547	0.15136195	0.18461571	0.03106063	0.17070881	0.0791496	[illegible]
ENSG00000220937	0.10419097	0.16284045	0.1018969	0.179572	0.03874663	0.10420832	0.11156419	0.03401863	0.08585186	0.03145604	0.08750807	[illegible]
ENSG00000220948	0	0.0159911	0	0	0	0	0	0	0	0	0	[illegible]
ENSG00000220949	0	0	0	0	0	0	0	0	0.01152141	0	0	[illegible]
ENSG00000220960	0	0	0	0	0	0	0	0	0	0	0	[illegible]
ENSG00000220981	0	0	0.38439154	0	0	0.29483329	0.31564503	0	0.19431836	0	0.49516762	[illegible]
ENSG00000220982	0	0	0	0	0	0	0	0	0	0	0	[illegible]
ENSG00000220984	0.37045678	0.49627565	0.36230008	0	0	0.55577769	0	0.36286537	0.73260255	0	0	[illegible]
ENSG00000220986	0	0	0	0	0	0	0	0	0	0	0	[illegible]
ENSG00000220987	0.31910633	0.21374248	0	0	0.17800431	0.2393696	0	0	0	0	0	[illegible]
ENSG00000220988	0	0	0	0	0	0	0	0	0	0	0	[illegible]
ENSG00000220989	0	0	0	0	0	0	0	0	0	0	0	[illegible]
ENSG00000220990	0	0	0	0	0	0	0	0	0	0	0	[illegible]
ENSG00000220992	0	0	0	0	0	0	0	0	0	0	0	[illegible]
ENSG00000220993	0	0	0	0	0	0	0	0	0	0	0	[illegible]
ENSG00000220996	0	0	0	0	0	0	0	0	0	0	0	[illegible]
ENSG00000220997	0	0	0	0	0	0	0	0	0	0	0	[illegible]
ENSG00000220999	0	0	0	0	0	0	0	0	0	0	0	[illegible]
ENSG00000221000	0	0	0	0	0	0	0	0	0	0	0	[illegible]
ENSG00000221002	0	0	0	0	0	0	0	0	0	0	0	[illegible]
ENSG00000221003	0	0	0	0	0	0	0	0	0	0	0	[illegible]
ENSG00000221005	0	0	0	0	0	0	0	0	0	0	0	[illegible]
ENSG00000221007	0	0	0	0	0	0	0	0	0	0	0	[illegible]
ENSG00000221008	0	0	0	0	0	0	0	0	0	0	0	[illegible]
ENSG00000221010	0	0	0	0	0	0	0	0	0	0	0	[illegible]
ENSG00000221011	0	0	0	0	0	0	0	0	0	0	0	[illegible]
ENSG00000221015	0	0	0	0	0	0	0	0	0	0	0	[illegible]
ENSG00000221016	0	0	0	0	0	0	0	0	0	0	0	[illegible]
ENSG00000221017	0	0	0	0	0	0	0	0	0	0	0	[illegible]
ENSG00000221018	0	0	0	0	0	0	0	0	0	0	0	[illegible]
ENSG00000221019	0	0	0	0	0	0	0	0	0	0	0	[illegible]
ENSG00000221020	0	0	0	0	0	0	0	0	0	0	0	[illegible]
ENSG00000221021	0	0	0	0	0	0	0	0	0	0	0	[illegible]
ENSG00000221023	0	0	0	0	0	0	0	0	0	0	0	[illegible]
ENSG00000221025	0	0	0	0	0	0	0	0	0	0	0	[illegible]
ENSG00000221026	0	0	0	0	0	0	0	0	0	0	0	[illegible]
ENSG00000221027	0	0	0	0	0.17286957	0	0	0	0	0	0	[illegible]
ENSG00000221028	0	0	0	0	0	0	0	0	0	0	0	[illegible]
ENSG00000221029	0	0	0	0	0	0	0	0	0	0	0	[illegible]
ENSG00000221030	0	0	0	0	0	0	0	0	0	0	0	[illegible]
ENSG00000221031	0	0	0	0	0	0	0	0	0	0	0	[illegible]
ENSG00000221032	0	0	0	0	0	0	0	0	0	0	0	[illegible]
ENSG00000221033	0	0	0	0	0	0	0	0	0	0.22628842	0.31475771	[illegible]
ENSG00000221035	0	0	0	0	0	0	0	0	0	0	0	[illegible]
ENSG00000221036	0	0	0.40410393	0	0.23049276	0	0	0	0	0	0	[illegible]
ENSG00000221037	0	0	0	0	0	0	0	0	0	0	0	[illegible]
ENSG00000221038	0	0	0	0	0	0	0	0	0	0	0	[illegible]
ENSG00000221039	0	0	0	0	0	0	0	0	0	0	0	[illegible]
ENSG00000221040	0	0	0	0	0	0	0	0	0	0	0	[illegible]
ENSG00000221041	0	0	0	0	0	0	0	0	0	0	0	[illegible]
ENSG00000221042	0	0	0	1.0682231	0.23049276	0	0	0	0	0	0	[illegible]
ENSG00000221043	0	0	0	0	0	0	0	0	0	0	0	[illegible]
ENSG00000221044	0	0.21268956	0.15527146	0.2052251	0	0.11909522	0.25500387	0.31102746	0.54945191	0.43139714	0.20001845	[illegible]
ENSG00000221046	0	0	0	0	0	0	0	0	0	0	0	[illegible]
ENSG00000221048	0	0	0	0	0	0	0	0	0	0	0	[illegible]
ENSG00000221050	0	0	0	0	0	0	0	0	0	0	0	[illegible]
ENSG00000221051	1.46498817	1.47190845	1.07454909	0	0.61290119	1.09892406	2.05886646	0.71748379	2.53497132	2.98546428	2.76843717	[illegible]
ENSG00000221052	0	0	0	0.4959607	0	0	0	0	0	0	0	[illegible]
ENSG00000221053	0	0	0	0	0	0	0	0	0	0	0	[illegible]
ENSG00000221055	0	0	0	0	0	0	0	0	0	0	0	[illegible]
ENSG00000221058	0	0	0	0	0	0	0	0	0	0	0	[illegible]
ENSG00000221059	0	0	0	0	0	0	0	0	0	0	0	[illegible]
ENSG00000221060	0	0	0	0	0	0	0	0	0	0	0	[illegible]
ENSG00000221062	0	0	0	0	0	0	0	0	0	0	0	[illegible]
ENSG00000221063	0	0.93860829	0	0	0	0.52557238	0.28133579	0.34314442	0.1731968	0.31729572	0	[illegible]
ENSG00000221065	0	0	0	0	0	0	0	0	0	0	0	[illegible]
ENSG00000221066	0.68573914	0.68897842	0.67064057	1.7727958	0.19125995	2.05755995	0.27534992	1.00753043	1.01707056	1.55272374	1.72781895	[illegible]
ENSG00000221067	0	0.22965947	0.33532028	0	0.38251989	0	0	0	0.16951176	0	0	[illegible]
ENSG00000221069	0	0	0	0	0	0	0	0	0	0	0	[illegible]
ENSG00000221070	0	0	0	0	0	0	0	0	0	0	0	[illegible]
ENSG00000221072	0	0	0	0	0	0	0	0	0	0	0	[illegible]
ENSG00000221073	0	0	0	0	0	0	0	0	0	0	0	[illegible]
ENSG00000221074	0	0	0	0	0	0	0	0	0	0	0	[illegible]
ENSG00000221075	0	0	0	0	0	0	0	0	0	0	0	[illegible]
ENSG00000221077	0	0.17989992	0	0.3471725	0	0	0	0	0	0	0	[illegible]
ENSG00000221079	0	0	0	0	0	0	0.60900924	0	0.18746006	0	0	[illegible]
ENSG00000221081	0	0	0	0	0	0	0	0	0	0	0	[illegible]
ENSG00000221082	0	0	0	0	0	0	0	0	0	0	0	[illegible]
ENSG00000221083	0.25783792	0.17270392	0	0	0	0.19341064	0	0	0.12747284	0	0.32482996	[illegible]
ENSG00000221084	0	0	0	0	0	0	0	0	0	0	0	[illegible]
ENSG00000221085	0	0	0	0	0	0	0	0	0	0	0	[illegible]
ENSG00000221087	0	0	0	0	0	0	0	0	0	0	0	[illegible]
ENSG00000221091	0	0	0	0	0	0	0	0	0	0	0	[illegible]
ENSG00000221093	0	0	0	0	0	0	0	0	0	0	0	[illegible]
ENSG00000221094	0	0.24813782	0	0	0	0	0	0	0	0	0	[illegible]
ENSG00000221095	0	0	0	0	0	0	0	0	0	0	0	[illegible]
ENSG00000221096	0	0	0	0	0	0	0	0	0	0	0	[illegible]
ENSG00000221100	0	0.18610337	0	0	0.15498651	0.41683327	0.22312838	0.27214902	0.41208893	0.50329666	0	[illegible]
ENSG00000221101	0	0	0	0	0	0	0	0	0	0	0	[illegible]
ENSG00000221102	0.24984294	0	0	0.3229512	0.27873543	0.56224022	0.20064258	0.24472315	0.37056059	0.22628842	0.31475771	[illegible]
ENSG00000221104	0	0	0	0	0	0	0	0	0	0	0	[illegible]
ENSG00000221105	0	0	0	0	0	0	0	0	0	0	0	[illegible]
ENSG00000221108	0	0	0	0	0	0	0	0	0	0	0	[illegible]
ENSG00000221110	0	0	0	0	0	0	0	0	0	0	0	[illegible]
ENSG00000221111	0	0	0	0	0	0	0	0	0	0	0	[illegible]
ENSG00000221112	0	0	0	0	0	0	0	0	0	0	0	[illegible]
ENSG00000221113	0	0	0	0	0	0	0	0	0	0	0	[illegible]
ENSG00000221114	0	0	0	0	0	0	0	0	0	0	0	[illegible]

ENSG00000221115	0	0	0.31208026	0	0	0	0	0	0	0	0	0	0	0.34570041	0	0	0	0	0	0	0	0	0	0	0	0	0.27456562	0	0	0	0	0	0	0	0	0	0	0	0	0	0	0	0	0	0	0	0	0
ENSG00000221116	0	0	0	0	0	0	0	0	0	0	0	0	0	0	0	0	0	0	0	0	0	0	0	0	0	0	0	0	0	0	0	0	0	0	0	0	0	0	0	0	0	0	0	0	0	0	0	0
ENSG00000221118	0	0	0	0	0	0	0	0	0	0	0	0	0	0	0	0	0	0	0	0	0	0	0	0	0	0	0	0	0	0	0	0	0	0	0	0	0	0	0	0	0	0	0	0	0	0	0	0
ENSG00000221120	0	0	0	0	0	0	0	0	0	0	0	0	0	0	0	0	0	0	0	0	0	0	0	0	0	0	0	0	0	0	0	0	0	0	0	0	0	0	0	0	0	0	0	0	0	0	0	0
ENSG00000221121	0	0	0	0	0	0	0	0	0	0	0	0	0	0	0	0	0	0	0.30842938	0	0.23591938	0	0	0	0	0	0	0	0	0	0	0	0	0	0	0	0	0	0	0	0	0	0	0	0	0	0	0
ENSG00000221122	0	0	0	0	0	0	0	0	0	0	0	0	0	0	0	0	0	0	0	0	0	0	0	0	0	0	0	0	0	0	0	0	0	0	0	0	0	0	0	0	0	0	0	0	0	0	0	0
ENSG00000221123	0	0	0	0	0	0	0	0	0	0	0	0	0	0	0	0	0	0	0	0	0	0	0	0	0	0	0	0	0	0	0	0	0	0	0	0	0	0	0	0	0	0	0	0	0	0	0	0
ENSG00000221125	0	0	0	0	0	0	0	0	0	0	0	0	0	0	0	0	0	0	0	0	0	0	0	0	0	0	0	0	0	0	0	0	0	0	0	0	0	0	0	0	0	0	0	0	0	0	0	0
ENSG00000221126	0	0	0	0	0	0	0	0	0	0	0	0	0	0	0	0	0	0	0	0	0	0	0	0	0	0	0	0	0	0	0	0	0	0	0	0	0	0	0	0	0	0	0	0	0	0	0	0
ENSG00000221128	0	0	0	0	0	0	0	0	0	0	0	0	0	0	0	0	0	0	0	0	0	0	0	0	0	0	0	0	0	0	0	0	0	0	0	0	0	0	0	0	0	0	0	0	0	0	0	0
ENSG00000221130	0	0	0	0	0	0	0	0	0	0	0	0	0	0	0	0	0	0	0	0	0	0	0	0	0	0	0	0	0	0	0	0	0	0	0	0	0	0	0	0	0	0	0	0	0	0	0	0
ENSG00000221131	0	0	0	0	0	0	0	0	0	0	0	0	0	0	0	0	0	0	0	0	0	0	0	0	0	0	0	0	0	0	0	0	0	0	0	0	0	0	0	0	0	0	0	0	0	0	0	0
ENSG00000221132	0.93873028	0.62877642	0.30602045	1.6178913	0.69819165	0.93888658	1.50774132	0.61299586	1.54700053	2.55068793	1.18263336	0.52850467	1.32425109	1.35595113	2.72136833	1.81114264	1.41219115	1.50978244	1.37745159	0.5556807	1.84383593	0.80174936	1.26790616	2.03641724	0.59741835	0.7725458	0	2.12347488	0.89880264	1.51024923	0.47542313	1.14851394	0.51333829	0.98495195	1.21882817	1.6700744	1.4478087	0.94593469	0.94554871	0	1.16909825	1.68096705	2.14959063	0.80144688	0.28863724	0.85956627	2.22912446	2.34035707
ENSG00000221134	0	0	0	0	0	0	0	0	0	0	0	0	0	0	0	0	0	0	0	0	0	0	0	0	0	0	0	0	0	0	0	0	0	0	0	0	0	0	0	0	0	0	0	0	0	0	0	0
ENSG00000221135	0	0	0	0	0	0	0	0	0	0	0	0	0	0	0	0	0	0	0	0	0	0	0	0	0	0	0	0	0	0	0	0	0	0	0	0	0	0	0	0	0	0	0	0	0	0	0	0
ENSG00000221136	0	0	0	0	0	0	0	0	0	0	0	0	0	0	0	0	0	0	0	0	0	0	0	0	0	0	0	0	0	0	0	0	0	0	0	0	0	0	0	0	0	0	0	0	0	0	0	0
ENSG00000221137	0	0	0	0	0	0	0	0	0	0	0	0	0	0	0	0	0	0	0	0	0	0	0	0	0	0	0	0	0	0	0	0	0	0	0	0	0	0	0	0	0	0	0	0	0	0	0	0
ENSG00000221139	0	0	0	0	0	0	0	0	0	0	0	0	0	0	0	0	0	0	0	0	0	0	0	0	0	0	0	0	0	0	0	0	0	0	0	0	0	0	0	0	0	0	0	0	0	0	0	0
ENSG00000221141	0	0	0	0	0	0	0	0	0	0	0	0	0	0	0	0	0	0	0	0	0	0	0	0	0	0	0	0	0	0	0	0	0	0	0	0	0	0	0	0	0	0	0	0	0	0	0	0
ENSG00000221142	0	0.3997776	0.58370568	0	0	0	0	0	0	0	0	0	0.25258863	0	0	0	0	0.35997128	0	0	0	0	0	0	0	0	0	0	0	0.24005505	0	0	0	0	0.58120047	0	0	0.30071381	0.3005911	0	0	0	0.34167876	0.5095619	0.2752744	0.40988577	0.26574053	0
ENSG00000221145	0	0	0	0	0.19331651	0	0	0	0	0.31388394	0	0	0	0	0	0	0	0	0	0	0.29172827	0	0	0	0	0	0	0	0	0	0	0	0	0	0	0	0	0	0	0	0.64740387	0	0	0	0	0	0	0
ENSG00000221147	0	0	0	0	0	0	0	0	0	0	0	0	0	0	0	0	0	0	0	0	0	0	0	0	0	0	0	0	0	0	0	0	0	0	0	0	0	0	0	0	0	0	0	0	0	0	0	0
ENSG00000221148	0	0	0	0	0	0	0	0	0	0	0	0	0	0	0	0	0	0	0	0	0	0	0	0	0	0	0	0	0	0	0	0	0	0	0	0	0	0	0	0	0	0	0	0	0	0	0	0
ENSG00000221156	0	0.25397636	0	0	0	0	0.60900924	0	0	0	0.47769112	0.32021165	0.32093615	0	0	0	0.57041446	0	0.41728681	0	0	0	0	0	0	0	0	0	0	0	0	0	0	0	0	0.33728954	0	0	0	0	0.708336	0	0	0	0	0	0	0
ENSG00000221157	0	0	0	0	0	0	0	0	0	0	0	0	0	0	0	0	0	0	0	0	0	0	0	0	0	0	0	0	0	0	0	0	0	0	0	0	0	0	0	0	0	0	0	0	0	0	0	0
ENSG00000221159	0.33226536	0.2225566	0	0	0	0	0	0	0.16426913	0	0	0.56119568	0	0	0	0	0	0	0	0	0	0.5675614	0	0	0	0	0.28588792	0	0	0	0	0	0	0	0	0	0	0	0	0	0	0	0	0.28367364	0	0	0.29587606	0
ENSG00000221160	0	0	0	0	0	0	0	0	0	0	0	0	0	0	0	0	0	0	0	0	0	0	0	0	0	0	0	0	0	0	0	0	0	0	0	0	0	0	0	0	0	0	0	0	0	0	0	0
ENSG00000221163	0	0	0	0	0	0	0	0.36286537	0	0	0	0	0	0	0	0	0	0	0	0	0	0	0	0	0	0	0	0	0	0	0	0	0	0	0	0	0	0	0	0	0	0	0	0	0	0	0	0
ENSG00000221164	0.75538453	3.87909205	2.2162575	3.2547424	1.12365219	2.64428603	6.06630296	8.63222687	6.59771554	7.06974526	14.9091877	9.35618429	0.85248664	3.27335077	2.40883618	0.97160256	2.65153598	3.03725764	6.92761299	1.78859725	4.23917637	5.59136661	4.33614002	5.61832969	7.45139368	5.59492155	11.2657707	5.69577898	6.87092491	7.49421851	2.67796933	2.15645456	1.65230763	2.37773557	1.47116369	3.80768265	7.92222822	10.149091	10.6521972	7.68467394	8.46682876	4.50884391	6.63070339	1.93474285	4.4129928	3.45841117	4.93280862	1.64784907
ENSG00000221165	0	0	0	0	0	0	0	0	0	0	0	0	0	0	0	0	0	0	0	0	0	0	0	0	0	0	0	0	0	0	0	0	0	0	0	0	0	0	0	0	0	0.67108375	0	0	0	0	0	0
ENSG00000221167	0	0	0	0	0	0	0	0	0	0	0	0	0	0	0	0	0	0	0	0	0.30483965	0	0	0	0	0	0.31158571	0	0	0	0	0	0	0	0	0	0	0	0	0	0	0	0	0	0	0	0	0
ENSG00000221169	0	0	0	0	0	0	0	0	0	0	0	0	0	0	0	0	0	0	0	0	0	0	0	0	0	0	0	0	0	0	0	0	0	0	0	0	0	0	0	0	0	0	0	0	0	0	0	0
ENSG00000221170	0	0	0	0	0	0	0	0	0	0	0	0	0	0	0	0	0	0	0	0	0	0	0	0	0	0	0	0	0	0	0	0	0	0	0	0	0	0	0	0	0	0	0	0	0	0	0	0
ENSG00000221172	0	0	0	0	0	0	0	0	0	0	0	0	0	0	0	0	0	0	0	0	0	0	0	0	0	0	0	0	0	0	0	0	0	0	0	0	0	0	0	0	0	0	0	0	0	0	0	0
ENSG00000221173	0	0	0	0	0	0	0	0	0	0	0	0	0	0	0	0	0	0	0	0	0	0	0	0	0	0	0	0	0	0	0	0	0	0	0	0	0	0	0	0	0	0	0	0	0	0	0	0
ENSG00000221174	0	0	0	0	0	0.35948462	0.43846232	0.55326755	0.20271671	0.84591136	0.7560553	0.94720738	0.24247043	0.58396029	0	0.33670298	0.80993537	0.24631513		0	0.18840784	0.19115783	0	0	0.42732007	0.55258484	0.19257728	0.16876382	0	0	0	0	0	0	0	0	0.41423416	0.67660606	0.67632998	0	0.418115	0	0	0	0.2064558	0.61482865	0	0.20925068
ENSG00000221175	0	0	0	0	0	0.60440823	0.32353616	0.39461609	0.19917632	0	0	0.34022488	0.34099466	0	0	0	0	0	0	0	0.33913411	0	0	0	0	0	0	0	0	0	0.61210728	0	0	0	0	0	0	0	0	0	0	0	0	0.34395428	0	0	0	0
ENSG00000221176	0	0.24813782	0	0	0.20664868	0	0	0	0	0	0.46670972	0	0	0	0.32218499	0	0	0	0	0	0.31184746	0	0	0	0	0	0	0.27933322	0.26602492	0	0	0	0	0	0	0	0	0.3732999	0.37314758	0	1.38410483	0	0	0	0	0.50882371	0	0
ENSG00000221177	0	0	0	0	0	0	0	0	0	0	0	0	0	0	0	0	0	0	0	0	0	0	0	0	0	0	0	0	0	0	0	0	0	0	0	0	0	0	0	0	0	0	0	0	0	0	0	0
ENSG00000221178	0	0	0	0	0	0	0	0	0	0	0	0	0	0	0	0	0	0	0	0	0	0	0	0	0	0	0	0	0	0	0	0	0	0	0	0	0	0	0	0	0	0	0	0	0	0	0	0
ENSG00000221179	0	0	0	0	0	0	0	0	0	0	0	0	0	0	0	0	0	0	0	0	0	0	0	0	0	0	0	0	0	0	0	0	0	0	0	0	0	0	0	0	0	0	0	0	0	0	0	0
ENSG00000221180	0	0	0	0	0	0.44360237	0	0	0	0	0.37251142	0	0.25027131	0	0	0	0	0	0	0	0	0	0.29952829	0	0	0	0.25441402	0.44590808	0.21233182	0	0	0	0	0	0	0	0.27362256	0	0.29783339	0.94991624	0.55237211	1.05895784	0	0	0	0	0.26330255	0
ENSG00000221181	0	0	0	0	0	0	0	0	0	0	0	0	0	0	0	0	0	0	0	0	0	0	0	0	0	0	0	0	0	0	0	0	0	0	0	0	0	0	0	0	0	0	0.34812553	0	0	0.41761946	0	0
ENSG00000221182	0	0	0.47044935	0	0	0.72168147	1.15893549	0.94236677	0.23782247	0.43568965	0	0	0	0	0	0.46404898	0	0	0	0	0	0	0	0	0	0	0.41389743	0	0	0	0	0	0.78916185	0	0	0	0	0	0	0	0	0	0.55076576	0	0	0	0.42835787	0.4497328
ENSG00000221183	0	0	0	0	0	0	0	0	0	0	0	0	0	0	0	0	0	0	0.40306112	0	0	0	0	0	0	0	0.31512645	0	0	0	0	0	0	0	0	0	0	0	0	0	0	0	0	0	0	0	0	0
ENSG00000221184	0.66453072	0.89022642	0	0	0.37068938	0.49848102	1.86783761	1.62728283	1.31415303	3.00940271	2.51157187	2.80597842	1.68739623	0	1.15588016	0.32052868	0.49984772	0.80158552	0	1.1801054	0.8390947	1.7026842	1.34633335	0.3089116	1.26874412	0	0.28588792	1.25267991	0.47719934	1.60366671	1.00966149	0.40651868	0.54509118	0	0.6471098	0.295563	0.92241833	1.00444612	0.66935751	2.1348633	1.24141361	0	1.1412775	0.28367364	0.61298218	0.45636766	0.59175211	2.17448125
ENSG00000221185	0	0	0	0	0	0	0	0	0	0	0	0	0	0	0	0	0	0	0	0	0	0	0	0	0	0	0	0	0	0	0	0	0	0	0	0	0	0	0	0	0	0	0	0	0	0	0	0
ENSG00000221186	0	0	0	0	0	0	0	0	0	0	0	0	0	0	0	0	0	0	0	0	0	0	0	0	0	0	0	0	0	0	0	0	0	0	0	0	0	0	0	0	0	0	0	0	0	0	0	0
ENSG00000221187	0	0	0	0	0	0	0	0	0	0	0	0	0	0	0	0	0	0	0	0	0	0	0	0	0	0	0	0	0	0	0	0	0	0	0	0	0	0	0	0	0	0	0	0	0	0	0	0
ENSG00000221189	0	0	0	0	0	0	0	0	0	0	0	0	0	0	0	0	0	0	0	0	0	0	0	0	0	0	0	0.28258128	0	0	0	0	0	0	0	0	0	0	0	0	0	0	0	0	0	0	0	0
ENSG00000221190	0	0	0	0	0	0	0	0	0	0	0	0	0	0	0	0	0	0	0	0	0	0	0	0	0	0	0	0	0	0	0	0	0	0	0	0	0	0	0	0	0	0	0	0	0	0	0	0
ENSG00000221191	0	0	0	0	0	0	0	0	0	0	0	0	0	0	0	0	0	0	0	0	0	0	0	0	0	0	0	0	0	0	0	0	0	0	0	0	0	0	0	0	0	0	0	0	0	0	0	0
ENSG00000221192	0	0	0	0	0	0	0	0	0	0	0	0	0	0	0	0	0	0	0	0	0	0	0	0	0	0	0	0	0	0	0	0	0	0	0	0	0	0	0	0	0	0	0	0	0	0	0	0
ENSG00000221195	0	0	0	0	0	0	0	0	0	0	0	0	0	0	0	0	0	0	0	0	0	0	0	0	0	0	0	0	0	0	0	0	0	0	0	0	0	0	0	0	0	0	0	0	0	0	0	0
ENSG00000221198	0	0	0	0	0	0	0	0	0	0	0	0	0	0	0	0	0	0	0	0	0	0	0	0	0	0	0	0	0	0	0	0	0	0	0	0	0	0	0	0	0	0	0	0	0	0	0	0
ENSG00000221199	0	0	0	0	0	0	0	0	0	0	0	0	0	0	0	0	0	0	0	0	0	0	0	0	0	0	0	0	0	0	0	0	0	0	0	0	0	0	0	0	0	0	0	0	0	0	0	0
ENSG00000221200	0	0	0	0	0	0	0	0	0	0	0	0	0	0	0	0	0	0	0	0	0	0	0	0	0	0	0	0	0	0	0	0	0	0	0	0	0	0	0	0	0	0	0	0	0	0	0	0
ENSG00000221201	0	0	0	0	0	0	0	0	0	0	0	0	0	0	0	0	0	0	0	0	0	0	0	0	0	0	0	0	0	0	0	0	0	0	0	0	0	0	0	0	0	0	0	0	0	0	0	0
ENSG00000221202	0	0	0	0	0	0	0	0	0	0	0	0	0	0	0	0	0	0.40079276	0	0	0	0	0	0	0	0	0	0	0	0	0	0	0	0	0	0	0	0	0	0	0	0	0	0	0	0	0	0
ENSG00000221203	0.34655634	0.23212893	0	0.4479645	0	0	0	0.67890939	0.17133447	0.31388394	0	0.29266657	0	0.75087616	0	0	0	0	0	0.30771566	0	0.59197264	0.35106004	0.32219812	0.33082844	0	0	0.26131172	0	0	0	0	0	0	0	0	0	0.34921603	0.34907354	0	1.29480774	0	0	0	0	0	0	0
ENSG00000221204	0	0	0	0	0	0	0	0	0	0	0	0	0	0	0	0	0	0	0	0	0	0	0	0	0	0	0	0	0	0	0	0	0	0	0	0	0	0	0	0	0	0	0	0	0	0	0	0
ENSG00000221206	0	0	0	0	0	0	0	0	0	0	0	0	0	0	0	0	0	0	0	0	0	0	0	0	0	0	0	0	0	0	0	0	0	0	0	0	0	0	0	0	0	0	0	0	0	0	0	0
ENSG00000221210	0	0	0	0	0	0	0	0	0	0	0	0	0	0	0	0	0	0	0	0	0	0	0	0	0	0	0	0	0	0	0	0	0	0	0	0	0	0	0	0	0	0	0	0	0	0	0	0
ENSG00000221211	1.53474951	5.48266427	4.2527128	2.9757644	1.42685992	5.18064201	0	0	0	0	0	0	0	0	0	0	0	0	0	0	0	0	0	0	0	0	0	0	0	0	0	0	0	0.40257957	0	0.45507318	0	0	0	0	0	0	0	0	0	0	0	0
ENSG00000221214	0	0.24531807	0	0	0	0.27473102	0.29412378	0.3587419	0	0.33171825	0.4614062	0	0	0	0.63704759	0	0	0	0	0	0	0	0	0	0	0	0.31512645	0	0	0.29461301	1.11292232	0	0.60083914	0.57642074	0	0	0	0.36905785	0	0.5883004	0	0	0	0.31268571	0.33783677	0	0	0
ENSG00000221216	0.27083815	0.36282337	0.79462454	0	0	0.40632486	0	0	0.26780009	0.24530425	0	0.22872261	0	0	0	0.26127128	0.4074389	0.32669662	0	0.48096733	0.22798932	0.23131704	0.54871569	0	0	0.22289137	0.46606938	0.40843681	0.19448881	0	0	0	0	0	0	0.48184219	0.25062907	0	0.27280537	0.87009134	0	0.96996978	0	0.23122977	0	0	0.24117628	0
ENSG00000221217	0	0	0.35022341	0	0.19976039	0.26862588	0	0	0	0	0	0	0	0	0	0	0	0	0	0	0	0	0	0	0	0	0	0	0	0	0	0	0	0	0	0	0	0	0	0	0.668984	0	0	0	0	0.49186292	0	0
ENSG00000221218	0	0	0	0	0	0	0	0	0	0	0	0	0	0	0	0	0	0	0	0	0	0	0	0	0	0	0	0	0	0	0	0	0	0	0	0	0	0	0	0	0	0	0	0	0	0	0	0
ENSG00000221219	0	0	0	0	0	0	0	0	0	0	0	0	0	0	0	0	0	0	0	0	0	0	0	0	0	0.34446847	0	0	0	0	0	0	0	0	0	0	0	0	0	0	0	0	0	0	0	0	0	0
ENSG00000221220	0	0	0	0	0	0	0.17728009	0	0	0	0	0	0	0	0	0	0	0	0	0	0	0	0	0	0	0	0	0	0	0	0	0	0	0	0	0	0	0	0	0	0	0	0	0	0	0	0	0
ENSG00000221221	0.33572646	0	0	0	0	0	0	0.32884674	0.33196053	0.30407507	0.42295568	0	0.28416221	0	0.87594043	0.32386752	0	0	0.36947269	0.29809954	0	0	0.68017883	0	0	0	0.28886592	0	0	0.54012386	0.5100894	0	0.55076921	0	0	0	0	0.33830303	0.33816499	0	0	0.60117919	0	0	0	0.46112149	0	0
ENSG00000221222	0	0.1458648	0	0	0	0.16335358	0	0	0	0	0	0	0	0	0	0	0	0	0	0	0	0	0	0	0	0	0	0	0	0	0	0	0	0	0	0	0	0	0	0	0	0	0	0	0	0	0	0
ENSG00000221225	0	0	0	0	0	0	0	0	0	0	0	0	0	0	0	0	0	0	0	0	0	0	0	0	0	0	0	0	0	0	0	0	0	0	0	0	0	0	0	0	0	0	0	0	0	0	0	0
ENSG00000221226	0	0	0	0	0	0	0	0	0	0	0	0	0	0	0	0	0	0	0	0	0	0	0	0	0	0	0	0	0	0	0	0	0	0	0	0	0	0	0	0	0	0	0	0	0	0	0	0
ENSG00000221227	0	0.25102315	0.36651287	0	0.41810314	0.56224022	0	0	0.37056059	0	0	0	0	0	0	0	0	0	0	0.33276228	0	0.32007823	0	0.69684709	0	0	0	0.28258128	0.26911823	0	0	0	0	0	0	0	0	0	0	0.6019818	0	0	0.42908495	0	0	0	0.33372067	0
ENSG00000221230	0	0	0	0	0	0	0	0	0	0	0.47213657	0.31648826	0.63440866	0.40599699	0	0.36152653	0	0	0	0.33276228	0	0	0	0.69684709	0	0	0.32245498	0	0.26911823	0	0	0	0	0	0	0	0.34680069	0	0.3774865	0	0	0	0.8581699	0.31995747	0	0	0.33372067	0
ENSG00000221232	0	0	0	0	0	0	0	0	0	0	0	0	0	0	0	0	0	0	0	0	0	0	0	0	0	0	0	0	0	0	0	0	0	0	0	0	0	0	0	0	0	0	0	0	0	0	0	0
ENSG00000221233	0	0	0	0	0	0	0	0	0	0	0	0	0	0	0	0	0	0	0	0	0	0	0	0	0	0	0	0	0	0	0	0	0	0	0	0	0	0	0	0	0	0	0	0	0	0	0	0
ENSG00000221234	0	0	0	0	0	0	0	0	0	0	0	0	0	0	0	0	0	0	0	0	0	0	0	0	0	0	0	0	0	0	0	0	0	0	0	0	0	0	0	0	0	0	0	0	0	0	0	0
ENSG00000221235	0	0	0	0	0	0.21780477	0	0	0	0	1.09739852	0	0	0	0	0	0	0.35024232	0	0	0	0	0	0.26994978	0.55436117	0	0.24982998	0.21893685	0.20850602	0.23356707	0	0.35524605	0	0.45698221	0.56549235	0	0.26869243	0	0	0	0.54241946	0.51993876	0	0	0	0	0	0
ENSG00000221236	0	0	0	0	0	0	0	0	0	0	0	0	0	0	0	0	0	0	0	0	0	0	0	0	0	0	0	0	0	0	0	0	0	0	0	0	0	0	0	0	0	0	0	0	0	0	0.30860191	0.32400105
ENSG00000221237	0	0	0	0	0	0	0	0	0	0	0	0	0	0	0	0	0	0	0	0	0	0	0	0	0	0	0	0	0	0	0	0	0	0	0	0	0	0	0	0	0	0	0	0	0	0	0	0
ENSG00000221238	0	0	0	0	0	0	0	0	0	0	0	0	0	0	0	0	0	0	0	0	0	0	0	0	0	0	0.31512645	0	0	0	0	0	0	0	0	0	0	0	0	0	0	0	0	0	0	0	0	0
ENSG00000221240	0	0	0	0	0	0	0	0	0	0	0	0	0	0	0	0	0	0	0	0	0	0	0	0	0	0	0	0	0	0	0	0	0	0	0	0	0	0	0	0	0	0	0	0	0	0	0	0
ENSG00000221241	2.47921075	0	1.38549919	1.8312397	1.77808698	3.71943529	1.13770957	0.34691524	1.57590054	1.92469492	1.33858501	2.09369159	2.39820417	0	0.61604602	0.68332488	1.06560944	0.85443731	0	1.25791455	5.96279752	2.11744061	1.07632694	1.97567638	2.02859637	3.4976799	1.82842601	2.93760323	1.27165759	1.70940298	0	0	0.58103125	1.67225359	0	2.83545599	4.58843987	4.28269333	6.06467325	1.70671764	3.96979517	4.43947708	1.62203542	0.60475478	0.32669929	4.37812052	0.9461531	1.32448779
ENSG00000221244	0	0	0	0	0	0	0	0	0	0	0	0	0	0	0	0	0	0	0	0	0	0	0	0	0	0	0	0	0	0	0	0	0	0	0	0	0	0	0	0	0	0	0	0	0	0	0	0
ENSG00000221245	0	0	0	0	0	0	0	0	0	0	0	0	0	0	0	0	0	0	0.27928645	0.22533509	0	0	0	0	0	0	0.21835534	0	0	0	0	0	0	0	0	0	0.23484141	0	0	0	0	0	0	0	0	0	0	0
ENSG00000221246	0	2.15879906	1.05067022	1.8515868	0.39952078	0.26862588	0.2875877	0.70153971	0.35409123	0.64869347	1.35345817	0.30242212	0.90931908	0.38795268	0	0.34545869	1.07744954	0	1.18231262	0	0.30145254	0	0.72552408	0	0.34185605	0	0	0.54004423	0.25715742	0	0	0.4381368	2.34994863	0	2.09232169	0	0.33138732	0	0.36070932	0	0	0.6412578	0	0.30573714	0.66065857	0	0.31888864	0
ENSG00000221247	0	0	0	0	0	0	0	0	0	0	0	0	0	0	0	0	0	0	0	0	0	0	0	0	0	0	0	0	0	0	0	0	0	0	0	0	0	0	0	0	0	0	0	0	0	0	0	0
ENSG00000221249	0	0	0	0	0	0	0	0	0	0	0	0	0	0	0	0	0	0	0	0	0	0	0	0	0	0	0	0	0	0	0	0	0	0	0	0	0	0	0	0	0	0	0	0	0	0	0	0
ENSG00000221250	0	0	0	0	0	0	0	0	0	0	0	0	0	0	0	0	0	0	0	0	0	0	0	0	0	0	0	0	0	0	0	0	0	0	0	0	0	0	0	0	0	0	0	0	0	0	0	0
ENSG00000221251	0	0	0	0	0	0	0	0	0	0	0	0	0	0	0	0	0	0	0	0	0	0	0	0	0	0	0	0	0	0	0	0	0	0	0	0	0	0	0	0	0	0	0	0	0	0	0	0
ENSG00000221252	0	0	0.16000054	0.6344269	0.18252218	0.61361242	0.39415573	0.48075056	0	0	0.20611038	0.13816239	0.13847499	0.35447453	0	0	0.24611792	0	0.18004761	0	0.13771944	0	0	0	0	0	0.14076715	0.24672071	0	0.39481135	0	0.40032804	0	0	0	0.14553102	0	0.32971666	0	0	0	0.29296042	0	0	0.45273557	0.22470895	0.29137033	0.15295481
ENSG00000221254	0	0	0	0	0	0	0	0	0	0	0	0	0	0	0	0	0	0	0	0	0	0	0	0	0	0	0	0	0	0	0	0	0	0	0	0	0	0	0	0	0	0	0	0	0	0	0	0
ENSG00000221255	0	0	0	0	0	0	0	0	0	0	0	0	0	0	0	0	0	0	0	0	0	0	0	0	0	0	0	0	0	0	0	0	0	0	0	0	0	0	0	0	0	0	0	0	0	0	0	0
ENSG00000221257	0	0	0	0	0	0	0	0	0	0	0	0	0	0	0	0	0	0	0	0	0	0	0	0	0	0	0	0	0	0	0	0	0	0	0	0	0	0	0	0	0	0	0	0	0	0	0	0
ENSG00000221258	0	0	0	0	0	0	0	0	0	0	0	0	0	0	0	0	0	0	0	0	0	0	0	0	0	0	0	0	0	0	0	0	0	0	0	0	0	0	0	0	0	0	0	0	0	0	0	0
ENSG00000221259	0	0	0	0	0	0	0	0	0	0	0	0	0	0	0	0	0	0	0	0	0	0	0	0	0	0	0	0	0	0	0	0	0	0	0	0	0	0	0	0	0	0	0	0	0	0	0	0
ENSG00000221260	0	0	0	0	0	0	0	0	0	0	0	0	0	0	0	0	0	0	0	0	0	0	0	0	0	0	0	0	0	0	0	0	0	0	0	0	0	0	0	0	0	0	0	0	0	0	0	0
ENSG00000221261	0	0	0	0	0.24627993	0	0	0	0	0	0.55621569	0	0.37369277	0	0.38397389	0	0	0	0	0	0.37165382	0	0.44724087	0	0	0	0	0	0	0.35514993	0	0	0.72429923	0	0	0	0	0	0	0	0	0.79059181	0	0	0	0	0	0
ENSG00000221262	0	0	0	0	0	0	0	0	0	0	0	0	0	0	0	0	0	0	0	0	0	0	0	0	0	0	0	0	0	0	0	0	0	0	0	0	0	0	0	0	0	0	0	0	0	0	0	0
ENSG00000221263	0	0.25699989	0	0	0	0	0	0	0	0	0.48337792	0	0	0	0	0	0	0	0	0	0	0	0	0	0	0	0	0	0	0	0	0	0	0	0	0	0	0	0	0	0	0	0.43930126	0	0	0	0	0
ENSG00000221264	0	0	0	0	0	0	0	0.52615478	0	0	0	0	0	0	0	0	0	0	0	0	0	0.2293894	0	0	0	0	0	0	0	0	0	0	0	0	0	0	0	0	0	0	0	0	0	0	0	0	0	0
ENSG00000221265	0	0	0	0	0.15910119	0.42789964	0	0.27937422	0.14100978	0	0	0	0	0	0	0.27514409	0	0	0.31388831	0.25325271	0	0	0	0	0	0	0	0	0.20481565	0	0	0	0.46791012	0	0	0	0	0	0	0	0	0	0	0	0	0	0.2539821	0
ENSG00000221266	0	0	0	0	0	0	0	0	0	0	0	0	0	0	0	0	0	0	0	0	0	0	0	0	0	0	0	0	0	0	0	0	0	0	0	0	0	0	0	0	0	0	0	0	0	0	0	0
ENSG00000221267	0	0	0	0	0	0	0	0	0	0	0	0	0	0	0	0	0	0	0	0	0	0	0	0	0	0	0	0	0	0	0	0	0	0	0	0	0	0	0	0	0	0	0	0	0	0	0	0
ENSG00000221269	0	0	0	0	0	0	0	0	0	0	0	0	0	0	0	0	0	0	0	0	0	0	0	0	0	0	0	0	0	0	0	0	0	0	0	0	0	0	0	0	0	0	0	0	0	0	0	0
ENSG00000221270	0	0	0	0	0	0	0	0	0	0	0	0	0	0	0	0	0	0	0	0	0	0	0	0	0	0	0	0	0	0	0	0	0	0	0	0	0	0	0	0	0	0	0	0	0	0	0	0
ENSG00000221272	0	0	0	0	0	0	0	0	0	0	0	0	0	0	0	0	0	0	0	0	0	0	0	0	0	0	0	0	0	0	0	0	0	0	0	0	0	0	0	0	0	0	0	0	0	0	0	0
ENSG00000221273	0	0	0	0	0	0	0	0	0	0	0	0	0	0	0	0	0	0	0	0	0	0	0	0	0	0	0	0	0	0	0	0	0	0	0	0	0	0	0	0	0	0	0	0	0	0	0	0
ENSG00000221275	0	0	0	0	0	0	0	0	0	0	0	0	0	0	0	0	0	0	0	0	0	0	0	0	0	0	0	0	0	0	0	0	0	0	0	0	0	0	0	0	0	0	0	0	0	0	0	0
ENSG00000221278	0	0	0	0	0	0	0	0	0	0	0	0	0	0	0	0	0	0	0	0	0	0	0	0	0	0	0	0	0	0	0	0	0	0	0	0	0	0	0	0	0	0	0	0	0	0	0	0
ENSG00000221279	0	0.26984988	0	0	0	0	0	0	0	0	0	0	0	0	0	0	0	0	0	0	0	0	0	0	0	0	0	0	0	0	0	0	0	0	0	0	0	0	0	0	0	0	0	0	0	0	0	0
ENSG00000221280	0	0	0.43178228	0	0	0	0	0	0	0	0	0	0	0	0	0	0.66418122	0	0	0.39202131	0	0	0	0	0	0	0	0	0	0	0	0	0	0	0	0	0	0	0	0	0	0	0	0	0	0	0	0
ENSG00000221281	0	0	0	0	0	0	0	0	0	0	0	0	0	0	0	0	0	0	0	0	0	0	0	0	0	0	0	0	0	0	0	0	0	0	0	0	0	0	0	0	0	0	0	0	0	0	0	0
ENSG00000221286	0.33572646	0.2248749	0	0	0	0	0	0	0	0	0.42295568	0	0	0	0	0	0	0	0	0	0	0	0	0	0	0	0	0.25314573	0	0	0	0	0	0	0	0	0	0	0	0	0	0	0	0	0	0.46112149	0	0
ENSG00000221287	0	0	0	0	0	0	0	0	0	0	0	0	0	0	0	0	0	0	0	0	0	0	0	0	0	0	0	0	0	0	0	0	0	0	0	0	0	0	0	0	0	0	0	0	0	0	0	0
ENSG00000221288	0.84077582	0.18772166	0.27408788	1.0868009	0.46900265	0.4204579	0	0.27451554	0.83134463	0.76150973	0.70615209	0.23667818	0	0.60723029	0.24373995	0	0.84322138	0.67611996	0.30842938	0	0.70775814	0.4787257	0.28390073	0.26056022	0.80261856	1.61450876	1.92912194	0	1.40877545	0.676329	0	0	0	0.44108718	0.54582305	0.49860192	0.5186932	1.41204744	1.41147127	0.90035539	0	0	0.32088092	0.47854509	0.51703714	1.1548086	1.2478251	0.26201824
ENSG00000221289	0	0	0	0	0	0	0	0	0	0	0	0	0	0	0	0	0	0	0	0	0	0	0	0	0	0	0	0	0	0	0	0	0	0	0	0	0	0	0	0	0	0	0	0	0	0	0	0
ENSG00000221290	0	0	0	0	0	0	0	0	0	0	0	0	0	0	0	0	0	0	0	0	0	0	0	0	0	0	0	0	0	0	0	0	0	0	0	0	0	0	0	0	0	0	0	0	0	0	0	0
ENSG00000221291	0	0	0	0	0	0	0	0	0	0	0	0	0	0	0	0	0	0	0	0	0	0	0	0	0	0	0	0	0	0	0	0	0	0	0	0	0	0	0	0	0	0	0	0	0	0	0	0
ENSG00000221293	0	0	0	0	0	0	0	0	0	0	0	0	0	0	0	0	0	0	0	0	0	0	0	0	0	0	0	0	0	0	0	0	0	0	0	0	0	0	0	0	0	0	0	0	0	0	0	0
ENSG00000221294	0	0	0	0	0	0.2984732	0	0.38974428	0	0	0	0	0.33678485	0	0.69210108	0	0	0	0	0	0	0	0	0	0	0	0	0.30002457	0	0	0	0	0	0	0	0	0	0	0	0	0	0	0	0	0	0	0	0
ENSG00000221295	0	0	0	0	0	0.45615716	0	0	0	0.27538874	0	0	0	0	0	0.29331398	0	0	0	0	0	0	0.30800551	0	0	0	0	0	0	0.24458439	0	0.37200294	0	0	0	0	0	0	0	0	0	0	0	0	0	0	0	0
ENSG00000221296	0	0	0	0	0	0	0	0	0.21532575	0	0	0.73562137	0	0	0	0	0	0	0	0	0	0	0.44119708	0	0	0	0	0	0	0	0	0	0	0	0	0	0	0	0	0	0	0.77990814	0	0	0	0.59821166	0	0
ENSG00000221297	0.38831012	0.26009627	0	0	0	0	0	0.38035285	0	0	0	0	0.32866955	0.84134317	0.33771197	0	0	0	0	0	0	0	0	0	0	0.31956714	0	0.29279506	0	0.31236078	0	0	0	0	0	0	0	0	0	0	0	0	0	0.3315222	0	0	0.34578286	0.36303732
ENSG00000221299	0	0	0	0	0	0	0	0	0	0	0	0	0	0	0	0	0	0	0	0	0	0	0	0	0	0	0	0	0	0	0	0	0	0	0	0	0	0	0	0	0	0	0	0	0	0	0	0
ENSG00000221300	0	0	0	0	0	0	0	0	0	0	0	0	0	0	0	0	0	0	0	0	0	0	0	0	0	0	0	0	0	0	0	0	0	0	0	0	0	0	0	0	0	0	0	0	0	0	0	0
ENSG00000221301	0	0	0	0	0	0	0	0	0	0	0	0	0	0	0	0	0	0	0	0	0	0	0	0	0	0	0	0	0	0	0	0	0	0	0	0	0	0	0	0	0	0	0	0	0	0	0	0
ENSG00000221302	0	0	0	0	0	0	0	0	0	0	0	0	0	0	0	0	0	0	0	0	0	0	0	0	0	0	0	0	0	0	0	0	0	0	0	0	0	0	0	0	0	0	0	0	0	0	0	0
ENSG00000221303	0	0.4625998	0	0	0.38525218	0.17268807	0.1848778	0.22549491	0.11381504	0	0	0.19441422	0	0	0.20021496	0.22208059	0.34632307	0	0	0	0.38758184	0	0.23320417	0.21403161	0	0	0	0	0.33063097	0	0	0.28165937	0	0	0	0	0	0	0	0.36978882	0	0.41223716	0.26358076	0	0.21235454	0	0	0
ENSG00000221304	0	0	0	0	0	0	0	0	0	0	0	0	0	0	0	0	0	0	0	0	0	0	0	0	0	0	0	0	0	0	0	0	0	0	0	0	0	0	0	0	0	0	0	0	0	0	0	0
ENSG00000221305	0	0	0	0	0	0	0	0	0	0	0	0	0	0	0	0	0	0	0	0	0	0	0	0	0	0	0	0	0	0	0	0	0	0	0	0	0	0	0	0	0	0	0	0	0	0	0	0
ENSG00000221307	0	0	0	0.4124822	0	0	0	0	0	0	0	0	0	0	0	0	0	0	0	0	0	0	0	0	0	0	0	0	0	0	0	0	0	0	0	0	0	0	0	0	0	0	0	0	0	0	0	0
ENSG00000221309	0	0	0	0	0	0	0	0	0.19197717	0	0	0	0	0	0	0	0	0	0	0.34478983	0	0	0	0	0	0	0	0	0	0	0	0	0	0	0	0	0.35933565	0	0	0	0	0	0	0	0	0	0	0
ENSG00000221310	0	0	0	0	0	0	0	0	0	0	0	0	0	0	0	0	0	0	0	0	0	0	0	0	0	0	0	0	0	0	0	0	0	0	0	0	0	0	0	0	0	0	0	0	0	0	0	0
ENSG00000221312	0	0	0	0	0	0	0	0	0	0	0	0	0	0	0	0	0	0	0	0	0	0	0	0	0	0	0	0	0	0	0	0	0	0	0	0	0	0	0	0	0	0	0	0	0	0	0	0
ENSG00000221313	0	0	0	0	0	0	0	0	0	0	0	0	0	0	0	0	0	0	0	0	0	0	0	0	0	0	0	0	0	0	0	0	0	0	0	0	0	0	0	0	0	0	0	0	0	0	0	0
ENSG00000221314	0	0	0	0	0	0	0	0	0	0	0	0	0	0	0	0	0	0	0	0	0	0	0	0	0	0	0	0	0	0	0	0	0	0	0	0	0	0.22397994	0	0	0	0	0	0	0	0	0	0
ENSG00000221316	0	0	0	0	0	0	0	0	0	0	0	0	0	0	0	0	0	0	0	0	0	0	0	0	0	0	0	0	0	0	0	0	0	0	0	0	0	0	0	0	0	0	0	0	0	0	0	0
ENSG00000221317	0	0	0	0	0	0	0	0	0	0	0	0	0	0	0	0	0	0	0	0	0	0	0	0	0	0	0	0	0	0	0	0	0	0	0	0	0	0	0	0	0	0	0	0	0	0	0	0
ENSG00000221318	0	0	0	0	0	0	0	0	0	0	0	0	0	0	0	0	0	0	0	0	0	0	0	0	0	0	0	0	0	0	0	0	0	0	0	0	0	0	0	0	0	0	0	0	0	0	0	0
ENSG00000221319	0	0	0	0	0.12934126	0.17393043	0.18620786	0.22711717	0.34390156	0.21000868	0	0.39162576	0	0	0	0	0.6976292	0	0	0.4117634	0.1951851	0.19803401	0.2348819	0.64671421	0.44269129	0	0.59851355	0	0	0	1.05687587	0.2836857	0	1.09478472	0.45158022	0.41251238	0.643702	0.46729627	0	0.74489835	0.43315511	0	0	0.1979593	0	0	0	0
ENSG00000221320	0	0.1944864	0	0	0.16196788	0.21780477	0	0.28440799	0.287101	0	0	0.24520712	0	0	0	0	0	0	0	0	0	0	0	0	0	0	0	0	0	0.23356707	0	0	0	0	0	0	0.26869243	0	0.29246702	0	0.54241946	0	0	0	0	0	0	0
ENSG00000221321	0	0	0	0	0	0	0	0	0	0	0	0	0	0	0	0	0	0	0	0	0	0	0	0	0	0	0	0	0	0	0	0	0	0	0	0	0	0	0	0	0	0	0	0	0	0	0	0
ENSG00000221322	0	0	0	0	0	0	0	0	0	0	0	0	0	0	0	0	0	0	0	0	0	0	0	0	0	0	0	0	0	0	0	0	0.62945053	0	0	0	0	0	0	0	0	0	0	0	0	0	0	0
ENSG00000221323	0	0	0	0	0	0	0	0	0	0	0	0	0	0	0	0	0	0	0	0	0	0	0	0	0	0	0	0	0	0	0	0	0	0	0	0	0	0	0	0	0	0	0	0	0	0	0	0
ENSG00000221324	0	0	0	0	0	0	0	0	0	0	0	0	0	0	0	0	0	0	0	0	0	0	0	0	0	0	0	0	0	0	0	0	0	0	0	0	0	0	0	0	0	0	0	0	0	0	0	0
ENSG00000221325	0	0	0	0	0	0	0	0	0	0	0	0	0	0	0	0	0	0	0	0	0	0	0	0	0	0	0	0	0	0	0	0	0	0	0	0	0	0	0	0	0	0	0	0	0	0	0	0
ENSG00000221326	0	0.24531807	0.35818303	0.4734171	0.2043004	0.54946203	0	0.3587419	0.18106938	0.66343651	0.4614062	0.30929535	0	0	0	0.70662004	0	0	0	0.3251995	0	0	0	0.34050483	0	0.60281983	0	0.27615898	0.52600382	0	0	0	0	0	0	0.32579103	0.33891885	0	0	0	0	0	0	0	0	0	0	0
ENSG00000221328	0	0	0	0	0	0	0	0	0	0	0	0	0	0	0	0	0	0	0	0	0	0	0	0	0	0	0	0	0	0	0	0	0	0	0	0	0	0	0	0	0	0	0	0	0	0	0	0
ENSG00000221330	0	0	0	0	0	0	0	0	0	0	0	0	0	0	0	0	0	0	0	0	0	0	0	0	0	0	0	0	0	0	0	0	0	0	0	0	0	0	0	0	0	0	0	0	0	0	0	0
ENSG00000221331	0	0	0	0	0	0	0	0	0	0	0	0	0	0	0	0	0	0	0	0	0	0	0	0	0	0	0	0	0	0	0	0	0	0	0	0	0	0	0	0	0	0	0	0	0	0	0	0
ENSG00000221332	0	0.10428981	0	0	0	0	0	0	0	0	0	0	0	0	0	0	0	0	0	0	0	0	0	0	0	0	0	0	0	0	0	0	0	0	0	0	0	0	0	0	0	0	0	0	0	0	0	0
ENSG00000221333	0	0	0	0	0	0	0	0.27214902	0.41208893	0	0.35003229	0	0	0	0	0	0	0	0	0	0	0	0	0	0	0	0.23906145	0	0	0	0.42214295	1.01980117	0.455809	0	0	0	0	0	0	0	0.51903931	0	0	0	0.25628996	0	0	0
ENSG00000221334	0	0	0	0	0	0	0	0	0	0	0	0	0	0	0	0	0	0	0	0	0	0	0	0	0	0	0	0	0	0	0	0	0	0	0	0	0	0	0	0	0	0	0	0	0	0	0	0
ENSG00000221335	0	0	0	0	0	0	0	0	0	0	0	0	0	0	0	0	0	0	0	0	0	0	0	0	0	0	0	0	0	0	0	0	0	0	0	0	0	0	0	0	0	0	0	0	0	0	0	0
ENSG00000221336	0	0	0	0	0	0	0	0.34691524	0	0	0	0	0	0	0	0	0	0	0	0	0	0	0	0	0	0	0.30473767	0	0	0	0	0	0	0.55741786	0	0	0	0	0	0	0	0	0	0	0	0	0	0
ENSG00000221337	0	0	0	0	0	0	0	0	0	0	0	0	0	0	0	0	0	0	0	0	0	0	0	0	0	0	0	0	0	0	0	0	0	0	0	0	0	0	0	0	0	0	0	0	0	0	0	0
ENSG00000221338	0	0.68897842	0	0.443199	0	0	0	0	0	0	0.43195474	0	0	0	0	0.33075832	0	0	0	0.30444208	0	0.29283753	0	0.31877048	0	0	0	0	0.24621455	0.27580793	0	0	0	0	0	0	0	0	0	0	0	0	0	0	0.63254544	0	0.30531891	0
ENSG00000221340	0	0.21374248	0	0	0	0	0	0	0	0.28902184	0	0.26948506	0.27009478	0	0	0	0	0.38491978	0	0.28334214	0	0	0	0	0	0	0	0	0	0	0	0	0	0	0	0	0.29529564	0	0	0	0	0.57141784	0	0	0.58870566	0	0	0
ENSG00000221343	0	0	0	0	0	0	0	0	0	0	0	0	0	0	0	0	0	0	0	0	0	0	0	0	0	0	0	0	0	0	0	0	0	0	0	0	0	0	0	0	0	0	0	0	0	0	0	0
ENSG00000221344	0.38831012	0	0	0	0	0	0	0	0	0	0	0.3279276	0	0	0	0	0	0	0	0	0	0	0	0	0	0.31956714	0	0	0	0.31236078	0	0	0.63703427	0	0	0	0	0	0	0	0	0	0	0.3315222	0.35818838	0	0	0.36303732
ENSG00000221345	0	0	0	0	0	0	0	0	0	0	0	0	0	0	0	0	0	0	0	0	0	0	0	0	0	0	0	0	0	0	0	0	0	0	0	0	0	0	0	0	0	0	0	0	0	0	0	0
ENSG00000221346	0	0	0	0	0	0	0	0	0	0	0	0	0	0	0	0	0	0	0	0	0	0	0	0	0	0	0	0	0	0	0	0	0	0	0	0	0	0	0	0	0	0	0	0	0	0	0	0
ENSG00000221347	0	0	0	0	0	0	0	0	0	0	0	0	0	0	0	0	0	0	0	0	0	0	0	0	0	0	0	0	0	0	0	0	0	0	0	0	0	0	0	0	0	0	0	0	0	0	0	0
ENSG00000221348	0	0	0	0	0	0	0	0	0	0	0	0	0	0	0	0	0	0	0	0	0	0	0	0	0	0	0	0	0	0	0	0	0	0	0	0	0	0	0	0	0	0	0	0	0	0	0	0
ENSG00000221351	0	0	0	0	0	0	0	0	0	0	0	0	0	0	0	0	0	0	0	0	0	0	0	0	0	0	0	0	0	0	0	0	0	0	0	0	0	0	0	0	0	0	0	0	0	0	0	0
ENSG00000221352	0	0	0	0	0	0	0	0	0	0	0	0	0	0	0	0	0	0	0	0	0	0	0	0	0	0	0	0	0	0	0	0	0	0	0	0	0	0	0	0	0	0	0	0	0	0	0	0
ENSG00000221354	0	0	0	0	0	0	0	0	0	0	0	0	0	0	0	0	0	0	0	0	0	0	0	0	0	0	0	0	0	0	0	0	0	0	0	0	0	0	0	0	0	0	0	0	0	0	0	0
ENSG00000221355	0	0	0	1.1109521	0	0	0	0	0.21245474	0	0	0	0	0	0	0	0	0	0.47292505	0	0	0	0	0	0	0	0	0	0	0	0	0	0	0	0.83692867	0	0	0	0	0	0	0	0	0	0.39639514	0	0	0.4017613
ENSG00000221356	0	0	0	0	0	0	0	0	0	0	0	0	0	0	0	0	0	0	0	0	0	0	0	0	0	0	0	0	0	0	0	0	0	0	0	0	0	0	0	0	0	0	0	0	0	0	0	0
ENSG00000221357	0	0	0	0	0	0	0	0	0	0	0	0	0	0	0	0	0	0	0	0	0	0	0	0	0	0	0	0	0	0	0	0	0	0	0	0	0	0	0	0	0	0	0	0	0	0	0	0
ENSG00000221358	0	0	0	0	0	0	0	0	0	0	0	0	0	0	0	0	0	0	0	0	0	0	0	0	0	0	0	0	0	0	0	0	0	0	0	0	0	0	0	0	0	0	0	0	0	0	0	0
ENSG00000221359	0	0	0	0	0	0	0	0	0	0	0	0	0	0	0	0	0	0	0	0	0	0	0	0	0	0	0	0	0	0	0	0	0	0	0	0	0	0	0	0	0	0	0	0	0	0	0	0
ENSG00000221362	0	0	0	0	0	0	0	0	0	0	0	0	0	0	0	0	0	0	0	0	0	0	0	0	0	0	0	0	0	0	0	0	0	0	0	0	0	0	0	0	0	0	0	0	0	0	0	0
ENSG00000221363	0	0	0	0	0	0	0	0	0	0	0	0	0	0	0	0	0	0	0	0	0	0	0	0	0	0	0	0	0	0	0	0	0	0	0	0	0	0	0	0	0	0	0	0	0	0	0	0
ENSG00000221365	0	0	0	0	0	0	0	0	0	0	0	0	0	0	0	0	0	0	0	0	0	0	0	0	0	0	0	0	0	0	0	0	0	0	0	0	0	0	0	0	0	0	0	0	0	0	0	0
ENSG00000221366	0	0	0	0	0	0	0	0	0	0	0	0	0	0	0	0	0	0	0	0	0	0	0	0	0	0	0	0	0	0	0	0	0	0	0	0	0	0	0	0	0	0	0	0	0	0	0	0
ENSG00000221369	0	0	0	0	0	0	0	0	0	0	0	0	0	0	0	0	0	0	0	0	0	0	0	0	0	0	0	0	0	0	0	0	0	0	0	0	0	0	0	0	0	0	0	0	0	0	0	0
ENSG00000221371	0	0	0	0	0	0	0	0	0	0	0	0	0	0	0	0	0	0	0	0	0	0	0	0	0	0	0	0	0	0	0	0	0	0	0	0	0	0	0	0	0	0	0	0	0	0	0	0

ENSG00000221372	0	0	0	0	0	0	0	0	0	0	0	0	0	0	0	0	0	0	0	0	0	0	0	0	0	0	0	0	0	0	0	0	0	0	0	0	0	0	0	0	0	0	0	0	0	0	0	0
ENSG00000221373	0	0	0	0	0	0	0	0	0	0	0	0	0	0	0	0	0	0	0	0	0	0	0	0	0	0	0	0	0	0	0	0	0	0	0	0	0	0	0	0	0	0	0	0	0	0	0	0
ENSG00000221374	0	0	0	0	0	0	0	0	0	0	0	0	0	0	0	0	0	0	0	0	0	0	0	0	0	0	0	0	0	0	0	0	0	0	0	0	0	0	0	0	0	0	0	0	0	0	0	0
ENSG00000221375	0	0	0	0	0	0	0	0	0	0	0	0	0	0	0	0	0	0	0	0	0	0	0	0	0	0	0	0	0	0	0	0	0	0	0	0	0	0	0	0	0	0	0	0	0	0	0	0
ENSG00000221376	0	0	0	0	0	0	0	0	0	0	0	0	0	0	0	0	0	0	0	0	0	0	0	0	0	0	0	0	0	0	0	0	0	0	0	0	0	0	0	0	0	0	0	0	0	0	0	0
ENSG00000221377	0	0	0	0	0	0	0	0	0	0	0	0	0	0	0	0	0	0	0	0	0	0	0	0	0	0	0	0	0	0	0	0	0	0	0	0	0	0	0	0	0	0	0	0	0	0	0	0
ENSG00000221378	0	0	0	0	0	0	0	0	0	0	0	0	0	0	0	0	0	0	0	0	0	0	0	0	0	0	0	0	0	0	0	0	0	0	0	0	0	0	0	0	0	0	0	0	0	0	0	0
ENSG00000221379	0	0	0	0	0	0	0	0	0	0	0	0	0	0	0	0	0	0	0	0	0	0	0	0	0	0	0	0	0	0	0	0	0	0	0	0	0	0	0	0	0	0	0	0	0	0	0	0
ENSG00000221380	0	0	0	0	0	0	0	0	0	0	0	0	0	0	0	0	0	0	0	0	0	0	0	0	0	0	0	0	0	0	0	0	0	0	0	0	0	0	0	0	0	0	0	0	0	0	0	0
ENSG00000221381	0	0	0	0	0	0	0	0	0	0	0	0	0	0	0	0	0	0	0	0	0	0	0	0	0	0	0	0	0	0	0	0	0	0	0	0	0	0	0	0	0	0	0	0	0	0	0	0
ENSG00000221382	0	0	0	0	0	0	0	0	0	0	0	0	0	0	0	0	0	0	0	0	0	0	0	0	0	0	0	0	0	0	0	0	0	0	0	0	0	0	0	0	0	0	0	0	0	0	0	0
ENSG00000221383	0	0	0	0	0	0	0	0	0	0	0	0	0	0	0	0	0	0	0	0	0	0	0	0	0	0	0	0	0	0	0	0	0	0	0	0	0	0	0	0	0	0	0	0	0	0	0	0
ENSG00000221384	0	0	0	0	0	0	0	0	0	0	0	0	0	0	0	0	0	0	0	0	0	0	0	0	0	0	0	0	0	0	0	0	0	0	0	0	0	0	0	0	0	0	0	0	0	0	0	0
ENSG00000221385	0	0	0	0	0	0	0	0	0	0	0	0	0	0	0	0	0	0	0	0	0	0	0	0	0	0	0	0	0	0	0	0	0	0	0	0	0	0	0	0	0	0	0	0	0	0	0	0
ENSG00000221386	0	0	0	0	0	0.24176329	0	0	0.31868211	0.58382413	0.40603745	0	0	0	0.56060188	0	0.96970459	0	0	0.85852668	0.27130729	0	0.32648584	0	0.30767045	0	0	0	0.23144168	0	0	0	0.52873844	0	0	0	0	0	0	0.51770435	0.6020856	0	0	0.82549028	0	0	0	0.30132097
ENSG00000221387	0	0	0	0	0	0	0.20541978	0.2505499	0.37938346	0.23167624	0	0	0	0	0	0	0	0	0	0	0.21532324	0	0.25911574	0	0	0.21050851	0	0.19287294	0	0	0	0	0	0.40257957	0	0	0	0	0.25764952	0	0	0	0	0	0	0	0	0
ENSG00000221388	0	0	0	0	0	0	0	0.29782346	0	0.27538874	0	0.51354699	0	0	0	0	0.45740782	0	0	0	0	0	0	0	0	0	0	0	0	0	0	0	0	0	0	0	0	0	0	0	0	0	0	0	0	0	0	0
ENSG00000221389	0	0	0	0	0	0	0	0	0	0	0	0	0	0	0	0	0	0	0	0	0	0	0	0	0	0	0	0	0	0	0	0	0	0	0	0	0	0	0	0	0	0	0	0	0	0	0	0
ENSG00000221390	0	0	0	0	0	0	0	0	0	0	0	0	0	0	0	0	0	0	0	0	0	0	0	0	0	0	0	0	0	0	0	0	0	0	0	0	0	0	0	0	0.76213367	0	0	0	0	0	0	0
ENSG00000221391	0	0.20757683	0.30307795	0	0.17286957	0.46492941	0.49774794	0	0.30642511	0.28068468	0.39042063	0	0	0	0	0	0	0	0.68210343	0.27516881	0	0.79404023	0	0	0	0.25503916	0.26664546	0.23367298	0.22254008	0	0	0	0.50840235	0	0.60355433	0.27566933	0.28677749	0	0	0.49779264	0	0.55493464	0	0	0	0	0.27596132	0
ENSG00000221392	0	0	0	0	0	0	0	0	0	0	0	0	0	0	0	0	0	0	0	0	0	0	0	0	0	0	0	0	0	0	0	0	0	0	0	0	0	0	0	0	0	0	0	0	0	0	0	0
ENSG00000221393	0	0	0	0	0	0	0	0	0	0	0	0	0	0	0	0	0	0	0	0	0	0	0	0	0	0	0	0	0	0	0	0	0	0	0	0	0	0	0	0	0	0	0	0	0	0	0	0
ENSG00000221394	0	0	0	0	0	0	0.75022877	0.91505179	0	0	0	0	0.39535612	0	0	0	0	0	0	0	0	0	0	0	0	0.7688137	0.4019004	0	0.33542273	0.37573834	0	0	0	0	0	0	0	0	0	0	0	0	0	0	0	0	0.4159417	0.87339412
ENSG00000221395	0	0	0	0	0	0	0	0.27451554	0	0	0	0.23667818	0	0	0	0	0	0	0	0	0	0	0	0	0	0	0	0	0	0	0	0	0	0	0	0	0	0	0	0	0	0	0	0.23927255	0	0	0	0
ENSG00000221396	0	0	0	0	0	0	0	0	0	0	0	0	0	0	0	0	0	0	0	0	0	0	0	0	0	0	0	0	0	0	0	0	0	0	0	0	0	0	0	0	0	0	0	0	0	0	0	0
ENSG00000221398	0	0	0	0	0	0	0	0	0	0	0	0	0	0	0	0	0	0	0	0	0	0	0	0	0	0	0	0	0	0	0	0	0	0	0	0	0	0	0	0	0	0	0	0	0	0	0	0
ENSG00000221400	0	0	0	0	0	0	0	0	0	0	0	0	0	0	0	0	0	0	0	0	0	0	0	0	0	0	0	0	0	0	0	0	0	0	0	0	0	0	0	0	0	0	0	0	0	0	0	0
ENSG00000221401	0	0	0	0	0	0	0	0	0	0	0	0	0	0	0	0	0	0	0	0	0	0	0	0	0	0	0	0	0	0	0	0	0	0	0	0	0	0	0	0	0	0	0	0	0	0	0	0
ENSG00000221402	0	0	0	0	0	0	0	0	0	0	0	0	0	0	0	0	0	0	0	0	0	0	0	0	0	0	0	0	0	0	0	0	0	0	0	0	0	0	0	0	0	0	0	0	0	0	0	0
ENSG00000221403	0	0	0	0	0	0	0	0	0	0	0	0	0	0	0	0	0	0	0	0	0	0	0	0	0	0	0	0	0	0	0	0	0	0	0	0	0	0	0	0	0	0	0	0	0	0	0	0
ENSG00000221405	0	0	0	0	0	0	0	0	0	0	0	0	0	0	0	0	0	0	0	0	0	0	0	0	0	0	0	0	0	0	0	0	0	0	0	0	0	0	0	0	0	0	0	0	0	0	0	0
ENSG00000221406	0.23354884	0.15643471	0	0	0	0.52557238	0.37511439	0.22876295	0.57732266	0	0	0.39446363	0	0	0.40623324	0	0	0.28171665	0	0	0	0.19946904	0	0.43426703	0	0.19220342	0.2009502	0.17610158	0.50113409	0	0	0.28574139	0.7662876	0	0.45485254	0	0	0	0	0	0	0	0.26740077	0.19939379	0	0	0	0
ENSG00000221407	0	0	0	0	0	0	0	0	0	0	0	0	0	0	0	0	0	0	0	0	0	0	0	0	0	0	0	0	0	0	0	0	0	0	0	0	0	0	0	0	0	0	0	0	0	0	0	0
ENSG00000221409	0	0	0	0	0	0	0	0	0	0	0	0	0	0	0	0	0	0	0	0	0	0	0	0	0	0	0	0	0	0	0	0	0	0	0	0	0	0	0	0	0	0	0	0	0	0	0	0
ENSG00000221410	0	0	0	0	0	0	0	0	0	0	0	0	0	0.42067158	0	0	0	0	0	0	0	0	0.39335643	0	0	0	0	0	0	0	0	0	0	0.61114489	0	0	0	0	0.39113059	0	0	0	0	0	0	0	0.34579286	0
ENSG00000221411	0	0	0	0	0	0	0.29412378	1.43496758	0.18106938	0	0	0	0	0	0	0	0	0	0	0	0	0.31280373	0.37100663	0.34050483	0	0.30140992	0	0	0	0.29461301	0	0	0	0	0	0	0	0	0	0.5883004	0.68418818	0	0	0	0	0	0	0
ENSG00000221412	0	0	0	0	0	0	0	0	0	0	0	0	0	0	0	0	0	0	0	0	0	0	0	0	0	0	0	0	0	0	0	0	0	0	0	0	0	0	0	0	0	0	0	0	0	0	0	0.3424102
ENSG00000221413	0	0.20175692	0.29458044	0	0	0.90378801	0	0.29504006	0.14891687	0	0	0	0	0	0	0	0	0	0	0	0	0	0	0	0	0.49577706	0	0.22712141	0	0	0	0	0.49414808	0	0	0.26794029	0	0	0	0	0	0	0	0	0	0	0	0
ENSG00000221415	0	0	0	0	0	0	0.31184208	0.38035285	0.19197717	0.35170128	0	0	0	0	0	0	0	0	0	0	0	0	0	0	0	0	0	0	0	0	0	0	0	0	0	0.34541699	0	0	0	0	0	0	0	0	0	0	0	0
ENSG00000221416	0	0	0	0	0	0	0	0	0	0	0	0	0	0	0	0	0	0	0	0	0	0	0	0	0	0	0	0	0	0	0	0	0	0	0	0	0	0	0	0	0	0	0	0	0	0	0	0
ENSG00000221417	0	0	0	0	0	0	0	0	0	0	0	0	0	0	0	0	0	0	0	0	0	0	0	0	0	0	0	0	0	0	0	0	0	0	0	0	0	0	0	0	0	0	0	0	0	0	0	0
ENSG00000221418	0	0	0	0	0	0	0	0	0	0	0	0	0	0	0	0	0	0	0	0	0	0	0	0	0	0	0	0	0	0	0	0	0	0	0	0	0	0	0	0	0	0	0	0	0	0	0	0
ENSG00000221419	0	0	0.35022341	0	0	0	0.2875877	0	0	0	0	0.30242212	0	0	0	0	0	0	0	0.31797284	0.30145254	0.30585253	0	0	0.34185605	0.29471192	0	0	0.51431485	0.86419817	0	0	0.58748716	0	0.69744056	0	0	0	0.72141865	0	0	0	0	0	0	0	0	0
ENSG00000221420	0	0	0	0	0	0	0	0	0	0	0	0	0	0	0	0	0	0	0	0	0	0	0	0	0	0	0	0	0	0	0	0	0	0	0	0	0	0	0	0	0	0	0	0	0	0	0	0
ENSG00000221421	0	0	0	0	0	0	0	0	0	0	0	0	0	0	0	0	0	0	0	0	0	0	0	0	0	0	0	0	0	0	0	0	0	0	0	0	0	0	0	0	0	0	0	0	0	0	0	0
ENSG00000221423	0	0	0	0	0	0	0	0	0	0	0	0	0	0	0	0	0	0	0	0	0	0	0	0	0	0	0	0	0	0	0	0	0	0	0	0	0	0	0	0	0	0	0	0	0	0	0	0
ENSG00000221424	0	0	0	0	0	0	0	0	0	0	0	0	0	0	0	0	0	0	0	0	0	0	0	0	0	0	0	0	0	0	0	0	0	0	0	0	0	0	0	0	0	0	0	0	0	0	0	0
ENSG00000221425	0	0	0	0	0	0	0	0	0	0	0	0	0	0	0	0	0	0	0	0	0	0	0	0	0	0	0	0	0	0	0	0	0	0	0	0	0	0	0	0	0	0	0	0	0	0	0	0
ENSG00000221427	0	0	0	0	0	0	0	0	0	0	0	0	0	0	0	0	0	0	0	0	0	0	0	0	0	0	0	0	0	0	0	0	0	0	0	0	0	0	0	0	0	0	0	0	0	0	0	0
ENSG00000221428	0	0	0	0	0	0	0	0	0	0	0	0	0	0	0	0	0	0	0	0	0	0	0	0	0	0	0	0	0	0	0	0	0	0	0	0	0	0	0	0	0	0	0	0	0	0	0	0
ENSG00000221429	0	0	0	0	0	0	1.17649512	0.71748379	0.36213876	0	0.4614062	0	1.23998057	0	0.31852379	0	0.55096851	0.88356586	1.20918336	1.300798	0.61660747	0.62560745	0.37100663	0.34050483	0.34962551	0	0.94537936	0	0	0.29461301	0	0	0.60083914	0	0	0.32579103	0	0	0	0	0	0.65583184	0.41933302	0.31268571	0.33783677	1.00608325	0	0.68482039
ENSG00000221430	0	0	0	0	0	0	0	0	0	0	0	0	0	0	0	0	0	0	0	0	0	0	0	0	0	0	0.19528963	0	0	0	0	0	0	0	0	0	0	0	0	0	0	0	0	0	0	0	0	0
ENSG00000221431	0	0	0	0	0	0	0	0	0	0	0	0	0	0	0	0	0	0	0	0	0	0	0	0	0	0	0	0	0	0	0	0	0	0	0	0	0	0	0	0	0	0	0	0	0	0	0	0
ENSG00000221432	0.34286957	0.22965947	0.33532028	0.443199	0	0	0	0	0	0	0	0	0	0	0	0	0	0	0	0	0	0	0	0	0	0	0	0	0	0	0	0	0	0	0	0	0	0.34550097	0	0	0	0	0	0	0	0	0	0
ENSG00000221434	0	0	0	0	0	0	0	0	0	0	0	0	0	0	0	0	0	0	0	0	0	0	0	0	0	0	0	0	0	0	0	0	0	0	0	0	0	0	0	0	0	0	0	0	0	0	0	0
ENSG00000221436	0	0	0	0	0	0	0	0	0	0	0	0	0	0	0	0	0	0	0	0	0	0	0	0	0	0	0	0	0	0	0	0	0	0	0	0	0	0	0	0	0	0	0	0	0	0	0	0
ENSG00000221437	0	0	0	0	0	0	0	0	0	0	0	0	0	0	0	0	0	0	0	0	0	0	0	0	0	0	0	0	0	0	0	0	0	0	0	0	0	0	0	0	0	0	0	0	0	0	0	0
ENSG00000221438	0	0	0	0	0	0	0	0	0	0	0	0	0	0	0	0	0	0	0	0	0	0	0	0	0	0	0	0	0	0	0	0	0	0	0	0	0	0	0	0	0	0	0	0	0	0	0	0
ENSG00000221439	0.51158317	0.68533303	0.25015958	0	0.14268599	0.38375126	0.20541978	0	0.37938346	0	0.64450389	0.6480474	0.64951363	0	1.11230531	0.24675621	0.76960681	0.30854681	0.281503	0	0.43064649	0.21846609	0	0.2378129	0	0	0	0.19287294	0.18368387	0	0.38863954	0	0	0.40257957	0	0.22753659	0.23670523	0	0	0	0.95569143	0	0	0	0	0.35133066	0.2277776	0.23914363
ENSG00000221440	0	0.34266652	0	0	0	0	0	0	0	0	0	0	0	0	0	0	0	0	0	0	0	0	0	0	0	0	0	0	0	0	0	0	0	0	0	0	0	0	0	0	0	0	0	0	0	0	0	0
ENSG00000221442	0	0	0	0	0	0	0	0	0	0	0	0	0	0	0	0	0	0	0	0	0	0	0	0	0	0	0	0	0	0	0	0	0	0	0	0	0	0	0	0	0	0	0	0	0	0	0	0
ENSG00000221443	0	0	0	0	0	0	0	0	0	0	0	0	0	0	0	0	0	0.75489122	0	0	0	0	0	0	0	0	0	0	0	0	0	0	0	0	0	0	0	0	0	0	0	0	0	0	0	0	0	0
ENSG00000221444	0	0	0	0	0	0	0	0	0	0	0	0	0	0	0	0	0	0	0	0	0	0	0	0	0	0	0	0	0	0	0	0	0	0	0	0	0	0	0	0	0	0	0	0	0	0	0	0
ENSG00000221445	0.39304561	0.52653636	0	0	0.21924921	0.58966657	0	0	0.38863672	0.35599032	0.99033525	0	0.33267771	0	0	0	0	0	0.4325534	0	0	0.67138361	0	0	0	0	0.33818449	0	0	0	0	0.48088185	0	0	0.76548354	0.3496294	0	0.79212417	0.79180096	0	0	0	0	0	0	0	0	0.3674646
ENSG00000221446	0	0	0	0	0	0	0	0	0	0	0	0	0	0	0	0	0	0	0	0	0	0	0	0	0	0	0	0	0	0	0	0	0	0	0	0	0	0	0	0	0	0	0	0	0	0	0	0
ENSG00000221447	0	0	0	0	0	0	0	0	0	0	0	0	0	0	0	0	0	0	0	0	0	0	0	0	0	0	0	0	0	0	0	0	0	0	0	0	0	0	0	0	0	0	0	0	0	0	0	0
ENSG00000221448	0	0.45448401	0	0	0	0.25448768	0.2724515	0	0	0	0	0	0	0	0	0	0	0	0	0	0	0	0	0	0	0	0.29190661	0	0	0	0	0	0	0	0	0	0	0	0	0	0.63377432	0	0	0.28964571	0	0	0	0
ENSG00000221449	0	0	0	0	0	0	0	0	0	0	0	0	0	0	0	0	0	0	0	0	0	0	0	0	0	0	0	0	0	0	0	0	0	0	0	0	0	0	0	0	0	0	0	0	0	0	0	0
ENSG00000221450	0	0	0	0	0	0	0	0	0	0	0	0	0	0	0	0	0	0	0	0	0	0	0	0	0	0	0	0	0	0	0	0	0	0	0	0	0	0	0	0	0	0	0	0	0	0	0	0
ENSG00000221451	0	0	0	0	0	0	0	0	0	0	0	0	0	0	0	0	0	0	0	0	0	0	0	0	0	0	0	0	0	0	0	0	0	0	0	0	0	0	0	0	0	0	0	0	0	0	0	0
ENSG00000221454	0	0.24531807	0	0.4734171	0	0	0	0.71748379	0	0.33171825	0	0	0	0.39676979	0	0.35331002	0	0	0	0	0	0	0.37100663	0	0.34962551	0	0	0.27615898	0	0.29461301	0	0	0	0	0	0	0.33891885	0	0	0	0	0	0.41933302	0	0	0	0	0
ENSG00000221455	0	0	0	0	0	0	0	0	0	0	0	0	0	0	0	0	0	0	0	0	0	0	0	0	0	0	0	0	0	0	0	0	0	0	0	0	0	0	0	0	0	0	0	0	0	0	0	0
ENSG00000221456	0	0	0	0	0	0	0	0	0	0	0	0	0	0	0	0	0	0	0	0	0	0	0	0	0	0	0	0	0	0	0	0	0	0	0	0	0	0	0	0	0	0	0	0	0	0	0	0
ENSG00000221457	0	0	0	0	0	0	0	0	0	0	0	0	0	0	0	0	0	0	0	0	0	0	0	0	0	0	0	0	0	0	0	0	0	0	0	0	0	0	0	0	0	0	0	0	0	0	0	0
ENSG00000221458	0	0	0	0	0	0	0	0	0	0	0	0	0	0	0	0	0	0	0	0	0	0	0	0	0	0	0	0	0	0	0	0	0	0	0	0	0	0	0	0	0	0	0	0	0	0	0	0
ENSG00000221459	0	0.16734876	0	0	0	0	0	0	0	0	0	0	0	0	0	0	0	0	0	0	0	0	0	0	0	0	0	0	0	0	0	0	0	0	0	0	0.23120046	0	0	0	0	0	0	0	0	0	0	0
ENSG00000221461	0	0	0	0	0	0	0	0	0	0	0	0	0	0	0	0	0	0	0	0	0	0	0	0	0	0	0	0	0	0	0	0	0	0	0	0	0	0	0	0	0	0	0	0	0	0	0	0
ENSG00000221463	0	0	0	0	0	0	0	0	0.40856681	0.37424623	0	0	0	0	0	0	0	0.49842177	0	0	0	0	0	0	0	0	0	0	0	0	1.25560467	0	0.6778698	0.65032084	0	0	0	0	0	0	0	0	0	0	0	0	0	0
ENSG00000221464	0.37476442	0.50204629	0.36651287	0	0	0	0	0	0	0	0.47213657	0	0	0	0	0	0	0	0	0.33276228	0.63094718	0	0.75926939	0	0	0	0.32245498	0.28258128	0.26911823	0.30146448	0	0.45851526	0.61481214	0	0.72987966	0	0	0	0	0	0	0.67108375	0	0	0	0	0	0
ENSG00000221465	0.35810822	0.23986656	0	0	0.19976039	0.26862588	0	0	0	0	0	0	0.30310636	0	0	0	0	0	0	0	0	0	0	0	0	0	0	0	0	0	0	0	0	0	0	0	0	0	0	0	0	0	0	0	0	0	0	0
ENSG00000221466	0	0	0	0	0	0	0	0	0	0	0	0	0	0	0	0	0	0	0	0	0	0	0	0	0	0	0	0	0	0	0	0	0	0	0	0	0	0	0	0	0	0	0	0	0	0	0	0
ENSG00000221467	0	0	0	0	0	0	0	0	0	0	0	0	0	0	0	0	0	0	0	0	0	0	0	0	0	0	0	0	0	0	0	0	0	0	0	0	0	0	0	0	0	0	0	0	0	0	0	0
ENSG00000221468	0	0	0	0	0	0	0	0	0	0	0	0	0	0	0	0	0	0	0	0	0	0	0	0	0	0	0	0	0	0	0	0	0	0	0	0	0	0	0	0	0	0	0	0	0	0	0	0
ENSG00000221469	0	0	0	0	0	0	0	0	0	0	0	0	0	0	0	0	0	0	0	0	0	0	0	0	0	0	0	0	0	0	0	0	0	0	0	0	0	0	0	0	0	0	0	0	0	0.79049398	0	0
ENSG00000221470	0	0	0	0	0	0.25996053	0	0	0	0	0	0	0	0	0	0	0	0	0	0	0	0	0	0	0	0	0	0	0	0	0	0	0	0	0	0	0	0	0	0	0	0	0	0	0	0	0	0
ENSG00000221471	0	0	0	0	0	0	0	0	0	0	0	0	0	0	0	0	0	0	0	0	0	0	0	0	0	0	0	0	0	0	0	0	0	0	0	0	0	0	0	0	0	0	0	0	0	0	0	0
ENSG00000221473	0	0	0	0	0	0	0	0	0	0	0	0	0	0	0	0	0	0	0	0	0	0	0	0	0	0	0	0	0	0	0	0	0	0	0	0	0	0	0	0	0	0	0	0	0	0	0	0
ENSG00000221474	0	0	0	0	0	0	0	0	0	0	0	0	0	0	0	0	0	0	0	0	0	0	0	0	0	0	0	0	0	0	0	0	0	0	0	0	0	0	0	0	0	0	0	0	0	0	0	0
ENSG00000221475	0	0	0	0	0	0	0	0	0	0	0	0	0	0	0	0	0	0	0	0	0	0	0	0	0	0	0	0	0	0	0	0	0	0	0	0	0	0	0	0	0	0	0	0	0	0	0	0
ENSG00000221476	0	0	0	0	0	0	0	0	0	0	0	0	0	0	0	0	0	0	0	0	0	0	0	0	0	0	0	0	0	0	0	0	0	0	0	0	0	0	0	0	0	0	0	0	0	0	0	0
ENSG00000221477	0	0	0	0	0	0	0	0	0	0	0	0	0	0	0	0	0	0	0	0	0	0	0	0	0	0	0	0	0	0	0	0	0	0	0	0	0	0	0	0	0	0	0	0	0	0	0	0
ENSG00000221478	0	0	0	0	0	0	0	0	0	0	0	0	0	0	0	0	0	0	0	0	0	0	0	0	0	0	0	0	0	0	0	0	0	0	0	0	0	0	0	0	0	0	0	0	0	0	0	0
ENSG00000221479	0.38368738	2.56999888	0.75047873	0.4959607	0	2.87813445	0	0	0	0	0	0	0	0	0	0	0	0	0	0	0	0	0	0	0	0	0	0	0	0	0	0.46943228	0	0.60386935	0	0.34130489	0	0	0	0	0	0	0	0	0	0	0	0
ENSG00000221484	0	0	0	0	0	0	0	0	0	0	0	0	0	0	0	0	0	0	0	0	0	0	0	0	0	0	0	0	0	0	0	0	0	0	0	0	0	0	0	0	0	0	0	0	0	0	0	0
ENSG00000221486	0	0	0	0	0	0	0	0	0	0	0	0	0	0	0	0	0	0	0	0	0	0	0	0	0	0	0	0	0	0	0	0	0	0	0	0	0	0	0	0	0	0	0	0	0	0	0	0
ENSG00000221487	0.27784258	0.93051683	0.27172506	0.718288	0	0.41683327	0	0.27214902	0	0	0	0.23463785	0	0	0	0.26802829	0.41797611	0	0.3057705	0	0	0.23729938	0	0	0	0	0	0.20949992	0.19951869	0.22349953	0	0	0.455809	0.4372847	0.54111768	0	0	0	0.55972137	0	0	0	0	0	0	0	0	0.25975946
ENSG00000221488	0	0	0	0	0	0	0	0	0	0	0	0	0	0	0	0	0	0	0	0	0	0	0	0	0	0	0	0	0	0	0	0.41949268	0	0	0	0	0	0	0	0	0	0	0	0	0	0	0	0
ENSG00000221489	0	0	0	0	0	0	0	0	0	0	0	0	0	0	0	0	0	0	0	0	0	0	0	0	0	0	0	0	0	0	0	0.4929039	0	0.63406282	0	0	0	0	0	0	0	0	0	0	0	0	0	0
ENSG00000221491	14.3504681	10.8727836	13.5743525	45.918	12.5980275	39.7056504	16.6255077	19.5867838	18.6091743	17.6851834	51.5697202	15.4963742	12.3454999	18.0950193	21.2783194	7.03525357	17.3414324	23.83693	19.9353441	26.9464578	20.0014862	19.8915771	10.2473657	17.7161929	56.5933964	77.8297603	77.9305418	45.2336316	42.5717545	75.3177092	73.2741557	77.7133155	58.6629511	39.2471	33.9047748	69.4767204	106.890554	91.7418558	153.077665	39.6780706	31.6424549	41.2838964	41.4803	77.3269485	104.162227	40.3902035	80.8627101	78.9592915
ENSG00000221492	0	0	0	0	0	0	0	0	0	0	0	0	0	0	0	0	0	0	0	0	0	0	0	0	0	0	0	0	0	0	0	0	0	0	0	0	0	0	0	0	0	0	0	0	0	0	0	0
ENSG00000221493	0	0	0	0	0	0	0	0	0	0	0	0	0	0	0	0	0	0	0	0	0	0	0	0	0	0	0	0	0	0	0	0	0	0	0	0	0	0	0	0	0	0	0	0	0	0	0	0
ENSG00000221494	0	0	0	0	0	0	0	0	0	0	0	0	0	0	0	0	0	0	0	0	0	0	0	0	0	0	0	0	0	0	0	0	0	0	0	0	0	0	0	0	0	0	0	0	0	0	0	0
ENSG00000221495	0	0	0	0	0	0	0	0	0	0	0	0	0	0	0	0	0	0	0	0	0	0	0	0	0	0	0	0	0	0	0	0	0	0	0	0	0	0	0	0	0	0	0	0	0	0	0	0
ENSG00000221496	0	0	0	0	0	0	0	0	0	0	0	0	0	0	0	0	0	0	0	0	0	0	0	0	0	0	0	0	0	0	0	0	0	0	0	0	0	0	0	0	0	0	0	0	0	0	0	0
ENSG00000221497	0	0	0	0	0	0	0	0	0	0	0	0	0	0	0	0	0	0	0	0	0	0	0	0	0	0	0	0	0	0	0	0	0	0	0	0	0	0	0	0	0	0	0	0	0	0	0	0
ENSG00000221498	0	0	0	0	0	0	0	0	0	0	0	0	0	0	0	0	0	0	0	0	0	0	0	0	0	0	0	0	0	0	0	0	0	0	0	0	0	0	0	0	0	0	0	0	0	0	0	0
ENSG00000221499	0	0	0	0	0	0	0	0	0	0	0	0	0	0	0	0	0	0	0	0	0	0	0	0	0	0	0	0	0	0	0	0	0	0	0	0	0	0	0	0	0	0	0	0	0	0	0	0
ENSG00000221500	1.69630209	2.55647257	2.07369123	2.1926685	2.83870026	3.81731517	18.049912	23.6769651	15.7244462	9.21827566	12.2879755	17.5484413	7.89671834	6.89126477	7.74515749	11.4546828	7.65556252	2.55769064	6.06713054	12.8025908	9.99553164	9.77923228	5.58462616	7.88537501	11.3352271	14.6580401	15.325097	5.43597152	7.61321319	7.163748	2.57729379	8.82038556	9.73991865	4.00460727	7.43324809	4.14954889	7.84864715	7.26461248	10.6788945	12.2614188	14.2599221	7.59384238	5.34097848	5.79291426	8.60594718	10.4844465	5.66446923	6.7400744
ENSG00000221502	0	0	0	0	0	0	0	0.90197962	0	0	0	0	0	0	0	0	0	0	0.50670541	0.40882223	0	0	0	0	0	0	0	0	0.33063097	0	0	0	0	0	0	0	0	0	0	0	0	0	0	0	0	0	0	0
ENSG00000221504	0	0	0	0	0	0	0	0	0	0	0	0	0	0	0	0	0	0	0	0	0	0	0	0	0	0	0	0	0	0	0	0	0	0	0	0	0	0	0	0	0	0	0	0	0	0	0	0
ENSG00000221505	0	0	0	0	0	0	0	0	0	0	0	0	0	0	0	0	0	0	0	0	0	0	0	0	0	0	0	0	0	0	0	0	0	0	0	0	0	0	0	0	0	0	0	0	0	0	0	0
ENSG00000221506	0	0	0	0	0	0	0	0	0	0	0	0	0	0	0	0	0	0	0	0	0	0	0	0	0	0	0	0	0	0	0	0	0	0	0	0	0	0	0	0	0	0	0	0	0	0	0	0
ENSG00000221507	0	0	0	0	0	0	0	0	0	0	0	0	0	0	0	0	0	0	0	0	0	0	0	0	0	0	0	0	0	0	0	0	0	0	0	0	0	0	0	0	0	0	0	0	0	0	0	0
ENSG00000221510	0	0	0	0	0	0	0	0	0	0	0	0	0	0	0	0	0	0	0	0	0	0	0	0	0	0	0	0	0	0	0	0	0	0	0	0	0	0	0	0	0	0	0	0	0	0	0	0
ENSG00000221511	0	0	0	0	0	0	0	0	0	0	0	0	0	0	0	0	0	0	0	0	0	0	0	0	0	0	0	0	0	0	0	0	0	0	0	0	0	0	0	0	0	0	0	0	0	0	0	0
ENSG00000221514	0.40287175	0.26984988	1.182004	1.5622763	0.22473044	0.30220412	0.64707232	0.78923217	0.59752895	1.45956031	1.52264045	0.68044977	1.36397862	0	1.05112852	1.94320512	1.21213073	0.48596122	0.44336723	0.7154389	0.33913411	0	0	0.74911063	0	0.99465272	0.6932782	0.30377488	0	0	0.61210728	0.4929039	1.3218461	1.26812564	0	0.35837013	0.37281074	0	0	0.64713044	0.752607	1.44283005	0.46126632	0.34395428	1.11486134	1.10669158	0.71749944	0.75330243
ENSG00000221515	0	0	0	0	0	0	0	0	0	0	0	0	0	0	0	0	0	0	0	0	0	0	0	0	0	0	0	0	0	0	0	0	0	0	0	0	0	0	0	0	0	0	0	0	0	0	0	0
ENSG00000221516	0	0	0	0	0	0	0	0	0	0	0	0	0	0	0	0	0	0	0	0	0	0	0	0	0	0	0	0	0	0	0	0	0	0	0	0	0	0	0	0	0	0	0	0	0	0	0	0
ENSG00000221517	0	0	0	0	0	0	0	0	0	0	0	0	0	0	0	0	0	0	0	0	0	0	0	0	0	0	0	0	0	0	0	0	0	0	0	0	0	0	0	0	0	0	0	0	0	0	0	0
ENSG00000221518	0	0	0.22840657	0	0	0	0	0	0.23092906	0	0	0.19723182	0	0	0.20311662	0	0	0	0	0	0	0	0	0	0	0	0	0	0.16771136	0	0	0.57148278	0	0.36757265	0.45485254	0	0	0.23534124	0	0	0	0	0.26740077	0	0	0	0	0
ENSG00000221519	0	0	0	0	0	0	0	0	0	0	0	0	0	0	0	0	0	0	0	0	0	0	0	0	0	0	0	0	0	0	0	0	0	0	0	0	0	0	0	0	0	0	0	0	0	0	0	0
ENSG00000221520	0.38368738	0	0.37523936	0.9919215	0.21402899	0.28781345	0	1.12747453	0.18969173	0.34751436	0.48337792	0	0	0	0	0.37013431	0	0	0.42225451	0	0	0	0	0	0.36627434	0	0	0.86792823	0.27552581	0.3086422	1.16591862	0	0	0.60386935	0	0.34130489	0	0	0.38647428	0.6163147	0	0	0.43930126	0	0.35392423	0	0.6833328	0.35871544
ENSG00000221521	0	0.84658787	0	0.8168765	0.17625917	0.47404567	0.25375385	0.30950281	0.7810836	0.2861883	0	0	0	0.68462238	0.54960968	0.30481649	0.95069077	0.38114606	0.69547801	0.56112855	0	0.26986988	0.64016831	0.29376887	0.30163769	0.26003993	0.2718738	0.23825481	0.22690361	0.25417593	0.48008414	0	0	0	0	0	0	0	0	0	0.59028	0	0.36177751	0.26976807	0	0	0	1.47706359
ENSG00000221523	0	0	0	0	0	0	0	0	0	0	0	0	0	0	0	0	0	0	0	0	0.7234861	0	0	0	0	0	0	0	0	0	0	0	0	0	0	0	0	0	0	0	0	0	0	0.36688457	0	0	0	0
ENSG00000221524	0	0	0	0	0	0	0	0	0	0	0	0	0	0	0	0	0	0	0	0	0	0	0	0	0	0	0	0	0	0	0	0	0	0	0	0	0	0	0	0	0	0	0	0	0	0	0	0
ENSG00000221525	0	0.50204629	0	0	0.20905157	0.28112011	0	0	0	0	0	0	0	0	0.65186265	0	0.56378174	0	0.41243463	1.99657367	0.63094718	0.64015646	1.51853878	0	0.35775634	0.30841945	1.2898199	0.84774385	0.53823647	4.2205027	0	0	0	0	0	0	0	0	0	0	0	0	0.42908495	1.27982989	0	0	0.66744134	1.05111967
ENSG00000221526	0	0	0	0	0	0	0	0	0	0	0	0	0	0	0	0	0	0	0	0	0	0	0	0	0	0	0	0	0	0	0	0	0	0	0	0	0	0	0	0	0	0	0	0	0	0	0	0
ENSG00000221527	0	0	0	0	0	0	0	0	0	0	0	0	0	0	0	0	0	0	0	0	0	0	0	0	0	0	0	0	0	0	0	0	0	0	0	0	0	0	0	0	0	0	0	0	0	0	0	0
ENSG00000221528	0	0	0	0	0	0	0	0	0	0	0	0	0	0	0	0	0	0	0	0	0	0	0	0	0	0	0	0	0	0	0	0	0	0	0	0.2409211	0	0	0	0	0	0	0	0	0	0	0	0
ENSG00000221529	0	0	0	0	0	0	0	0	0	0	0	0	0	0	0	0	0	0	0	0	0	0	0	0	0	0	0	0	0	0	0	0	0	0	0	0	0	0	0	0	0	0	0	0	0	0	0	0
ENSG00000221530	0	0.56810501	0	0	0	0	0	0	0	0	0	0	0	0	0.24587802	0	0	0	0	0	0.23798885	0	0.28639109	0	0	0	0.24325551	0	0	0	0	0.34589747	0	0	0.55061097	0	0.26162157	0	0	0.52814526	0	0	0	0	0	0	0	0
ENSG00000221531	0.55568517	0.37220673	0.54345011	0	0	0.83366653	0	1.0885961	0.54945191	0.50329666	2.10019372	0.9385514	0.47033746	0.60199554	0	0	0	0.67029134	0.61154101	0	0.46777119	0.47459876	0.56290662	1.03325604	1.06093258	0.4573116	0.95624579	0.83799967	1.19711214	1.34099716	0	0	1.823236	1.74913881	0	0.49430363	1.02844342	0	1.11944273	0	1.03807862	0	0	0	0	0	0	0
ENSG00000221532	0	0	0	0	0	0	0	0	0	0	0	0	0	0	0	0	0	0	0	0	0	0	0	0	0	0	0	0	0	0	0	0	0	0	0	0	0	0	0	0	0	0	0	0	0	0	0	0
ENSG00000221533	0	0	0	0	0	0	0	0	0	0	0	0	0	0	0	0	0	0	0	0	0	0	0	0	0	0	0	0	0	0	0	0	0	0	0	0	0	0	0	0	0	0	0	0	0	0	0	0
ENSG00000221534	0	0	0	0	0	0	0	0	0	0	0	0	0	0	0	0	0	0	0	0	0	0	0	0	0	0	0	0	0	0	0	0	0	0	0	0	0	0	0	0	0	0	0	0	0	0	0	0
ENSG00000221535	0	0	0	0	0	0	0	0	0	0	0	0	0	0	0	0	0	0	0	0	0	0	0	0	0	0	0	0	0	0	0	0	0	0	0	0	0	0	0	0	0	0	0	0	0	0	0	0
ENSG00000221536	0	0	0	0	0	0	0	0	0	0	0	0	0	0	0	0	0	0	0	0	0	0	0	0	0	0	0	0	0	0	0	0	0	0	0	0	0	0	0	0	0	0	0	0	0	0	0	0
ENSG00000221537	0	0	0	0	0	0	0	0	0	0	0	0	0	0	0	0	0	0	0	0	0	0	0	0	0	0	0	0	0	0	0	0	0	0	0	0	0	0	0	0	0	0	0	0	0	0	0	0
ENSG00000221538	0.35810822	0	0	0	0	0	0	0.35076985	0	0	0	0.60484424	0	0	0	0	0	1.29589659	0	0	0	0	0	0	0	0	0	0	0	0	0	0	0	0.56361139	0	0.31855123	0	0	0	0	0	0	0	0	0	0	0	0
ENSG00000221539	0.43553702	1.45864801	2.98163171	0.5629825	2.42951824	1.63353577	10.4930646	13.6515835	14.8574767	15.3845546	27.434963	14.7124274	10.6906433	4.2465091	8.71205618	9.24335408	8.51767542	16.286268	13.4208459	7.73447459	8.06589233	6.69569057	6.17675908	8.09849325	4.98925052	9.31926873	7.12015445	3.9408633	5.31690348	6.30631098	3.97042558	1.06573816	5.71609126	7.54020649	2.54471556	6.97368905	10.8820432	14.0441475	13.1610159	12.5928085	9.76355028	10.1388058	5.98399554	8.18053432	6.02627751	6.58032828	6.59323805	9.3653816
ENSG00000221540	0	0	0	0	0	0	0.37511439	0	0	0	0	0	0	0.50602524	0.40623324	0	0	0	0	0	0	0	0	0	0	0	0	0	0	0	0	0	0	0	0	0	0	0	0	0.75029616	0	0	0	0.39878758	0	0	0	0
ENSG00000221541	0	0	0	0	0	0	0	0	0.15776342	0	0	0.26948506	0	0	0	0	0	0	0	0	0	0	0	0	0	0	0	0.24061377	0	0	0	0	0	0	0	0	0	0	0	0	0	0	0	0	0	0	0	0
ENSG00000221542	0	0	0	0	0	0	0	0	0	0	0	0	0	0	0	0	0	0	0	0	0	0	0	0	0	0	0	0	0	0	0	0	0	0	0	0	0	0	0	0	0	0	0	0	0	0	0	0
ENSG00000221544	0	0	0	0	0	0	0	0	0	0	0	0	0	0	0	0	0	0	0	0	0	0	0	0	0	0	0	0	0	0	0	0	0	0	0	0	0	0	0	0	0	0	0	0	0	0	0	0
ENSG00000221545	0	0	0	0	0.26833485	0	0	0.47118339	0	0	0	0.40623867	0	0	0	0	0	0	0	0.4271277	0	0.41084669	0	0	0	0	0.41389743	0.36271627	0.34543534	0.3869544	0	0	0	0	1.87372091	0	0	0.4847327	0	0	0.89863522	0.86139108	0	0	0	0	0	0
ENSG00000221547	0	0	0	0	0	0	0	0	0	0	0	0	0	0	0	0	0	0	0	0	0	0	0	0	0	0	0	0	0	0	0	0	0	0	0	0	0	0	0	0	0	0	0	0	0	0	0	0
ENSG00000221548	0	0	0	0	0	0	0	0	0	0	0	0	0	0	0	0	0	0	0	0	0	0	0	0	0	0	0	0	0	0	0	0	0	0	0	0	0	0	0	0	0	0	0	0	0	0	0	0
ENSG00000221550	0	0	0	0	0	0	0	0	0	0	0	0	0	0	0	0	0	0	0	0	0	0	0	0	0	0	0	0	0	0	0	0	0	0	0	0	0	0	0	0	0	0	0	0	0	0	0	0
ENSG00000221551	0	0.14296682	0	0	0	0	0	0	0	0	0	0	0	0	0	0	0	0	0	0	0	0	0	0	0	0	0	0	0	0	0	0	0	0	0	0	0	0	0	0	0	0	0	0	0	0	0	0
ENSG00000221552	0	0	0	0	0	0	0	0	0	0	0	0	0	0	0	0	0	0	0	0	0	0.32007823	0	0	0	0.30841945	0	0	0	0.30146448	0	0	0	0	0	0	0	0	0	0	0	0	0	0.31995747	0	0	0	0
ENSG00000221553	0	0	0	0	0	0	0	0	0	0	0	0	0	0	0	0	0	0	0	0	0	0	0	0	0	0	0	0	0	0	0	0	0	0	0	0	0	0	0	0	0	0	0	0	0	0	0	0
ENSG00000221555	0	0	0	0	0	0	0	0	0.18315064	0	0	0.9385514	0.3135583	0.40133036	0	0.71474211	0.55730149	0	0	0.32893743	0	0.63279834	0.37527108	0	0	0	0.3187486	0	0.26602492	0.59599874	0	0	0	0	0	0	0.68562895	0.74659979	0	0	0	0	0	0	1.3668798	0	0	1.03903784
ENSG00000221556	0	0	0	0	0	0	0	0	0	0	0	0	0	0	0	0	0	0	0	0	0	0	0	0	0	0	0	0	0	0	0	0	0	0	0	0	0	0	0	0	0	0	0	0	0	0	0	0
ENSG00000221558	0	0	0	0	0	0	0	0	0	0	0	0	0	0	0	0	0	0	0	0	0	0	0	0	0	0	0	0	0	0	0	0	0	0	0	0	0	0	0	0	0	0	0	0	0	0	0	0
ENSG00000221559	0	0	0	0	0	0	0	0	0	0	0	0	0	0	0	0	0	0	0	0	0	0	0	0	0	0	0	0	0	0	0	0	0	0	0	0	0	0	0	0	0	0	0	0	0	0	0	0
ENSG00000221560	0	0	0	0	0	0	0	0	0	0	0	0	0	0	0	0	0	0	0	0	0	0	0	0	0	0	0	0	0	0	0	0	0	0	0	0	0	0	0	0	0	0	0	0	0	0	0	0
ENSG00000221561	0	0	0	0	0	0	0	0	0	0	0	0	0	0	0	0	0	0	0	0	0	0	0	0	0	0	0	0	0	0	0	0	0	0	0	0	0	0	0	0	0	0	0	0	0	0	0	0
ENSG00000221562	0	0	0.28396492	0	0	0	0	0	0	0	0	0.24520712	0	0	0	0.28010164	0	0	0.31954395	0.25781582	0.24442098	0	0.88239415	0	0	0	0.24982998	0	0.20850602	0	0	0	0	0	0	0	0.26869243	0	0.58493404	0	0	0	0	0	0	0	0	0
ENSG00000221563	0	0	0	0	0	0	0	0	0	0	0	0	0	0	0	0	0	0	0	0	0	0	0	0	0	0	0	0	0	0	0	0	0	0	0	0	0	0	0	0	0	0	0	0	0	0	0	0
ENSG00000221564	0	0	0	0	0	0	0	0	0	0	0	0	0	0	0	0	0	0	0	0	0	0	0	0	0	0	0	0	0	0	0	0	0	0	0	0	0	0	0	0	0	0	0	0	0	0	0	0
ENSG00000221565	0	0	0	0	0	0	0	0	0	0	0	0	0	0	0	0	0	0	0	0	0	0	0	0	0	0	0	0	0	0	0	0	0	0	0	0	0	0	0	0	0	0	0	0	0	0	0	0
ENSG00000221566	0	0	0	0	0	0	0	0	0	0	0	0	0	0	0	0	0	0	0	0	0	0	0	0	0	0	0	0	0	0	0	0	0	0	0	0	0	0	0	0	0	0	0	0	0	0	0	0
ENSG00000221567	0	0	0	0	0	0	0	0	0	0	0	0	0	0	0	0	0	0	0	0	0	0	0	0	0	0	0	0	0	0	0	0	0	0	0	0	0	0	0	0	0	0	0	0	0	0	0	0
ENSG00000221568	0	0	0	0	0	0	0	0	0	0	0	0	0	0	0	0	0	0	0	0	0	0	0	0	0	0	0	0	0	0	0	0	0	0	0	0	0	0	0	0	0	0	0	0	0	0	0	0
ENSG00000221571	0	0	0.11545827	0	0	0.08855798	0	0	0	0	0	0	0	0	0	0	0	0	0	0	0	0	0	0	0	0	0	0	0	0	0	0.07222035	0	0	0	0.05250844	0	0	0	0	0	0	0	0	0	0	0	0
ENSG00000221573	0	0	0	0	0	0	0.27831067	0	0	0.31388394	0	0	0	0	0	0	0	0	0	0	0	0	0	0	0	0	0	0	0	0	0	0	0	0	0	0	0	0	0	0	0	0	0	0.29587465	0.3196735	0	0	0
ENSG00000221574	0	0	0	0	0	0	0	0	0	0	0	0	0	0	0	0	0	0	0	0	0	0	0	0	0	0	0	0	0	0	0	0	0	0	0	0	0	0	0	0	0	0	0	0	0	0	0	0
ENSG00000221576	0	0	0	0	0	0	0	0	0	0	0	0	0	0	0	0	0	0	0	0	0	0	0	0	0	0	0	0	0	0	0	0	0	0	0	0	0	0	0	0	0	0	0	0	0	0	0	0
ENSG00000221578	0	0	0	0	0	0	0	0	0	0	0	0	0	0	0	0	0	0	0	0	0	0	0	0	0	0	0	0	0	0	0	0	0	0	0	0	0	0	0	0	0	0	0	0	0	0	0	0
ENSG00000221579	0	0	0	0	0	0	0	0	0	0	0	0	0	0	0	0	0	0	0	0	0	0	0	0	0	0	0	0	0.54456866	0	0	0	0	0	0	0	0	0	0	0	0	0	0	0	0	0	0	0
ENSG00000221580	0	0.19625446	0	0	0	0	0	0	0	0	0	0	0	0	0	0	0	0	0	0	0	0	0	0	0	0	0	0	0	0	0	0	0	0	0	0	0	0	0	0	0	0	0	0	0	0	0	0
ENSG00000221581	0	0	0	0	0	0	0	0	0	0	0	0	0	0	0	0	0	0	0	0	0	0	0	0	0	0	0	0	0	0	0	0	0	0	0	0	0	0	0	0	0	0	0	0	0	0	0	0
ENSG00000221583	0	0	0	0	0	0	0	0	0	0	0	0	0	0	0	0	0	0	0	0	0	0	0	0	0	0	0	0	0	0	0	0	0	0	0	0	0	0	0	0	0	0	0	0	0	0	0	0
ENSG00000221584	0	0	0	0	0	0	0	0	0	0	0	0	0	0	0	0	0	0	0	0	0	0	0	0	0	0	0	0	0	0	0	0	0	0	0	0	0	0	0	0	0	0	0	0	0	0	0	0
ENSG00000221585	0	0	0	0	0	0.32235106	0	0.21245474	0	0	0	0	0	0	0.41455043	0	0	0	0	0	0	0	0	0.39952567	0	0	0	0	0.30858891	0	0	0	0	0	0	0	0	0	0	0	0	0	0	0	0	0	0	0
ENSG00000221586	0	0	0	0	0	0	0	0	0	0	0	0	0	0	0	0	0	0	0	0	0	0	0	0	0	0	0	0	0	0	0	0	0	0	0	0	0	0	0	0	0	0	0	0	0	0	0	0
ENSG00000221589	0	0	0	0	0	0	0	0	0	0	0	0	0	0	0	0	0	0	0	0	0	0	0	0	0	0	0	0	0	0	0	0	0	0	0	0	0	0	0	0	0	0	0	0	0	0	0	0
ENSG00000221590	0	0	0	0	0	0	0	0	0	0	0	0	0	0	0	0	0	0	0	0	0	0	0	0	0	0	0	0	0	0	0	0	0	0	0	0	0	0	0	0	0	0	0	0	0	0	0	0
ENSG00000221591	0	0	0	0	0	0	0	0	0	0	0	0	0	0	0	0	0	0	0	0	0	0	0	0	0	0	0	0	0	0	0	0	0	0	0	0	0	0	0	0	0	0	0	0	0	0	0	0
ENSG00000221593	0	0	0	0	0	0	0	0	0	0	0	0	0	0	0	0	0	0	0	0	0	0	0	0	0	0	0	0	0	0	0	0	0	0	0	0	0	0	0	0	0	0	0	0	0	0	0	0
ENSG00000221594	0	0	0	0	0	0	0	0	0	0	0	0	0	0	0	0	0	0	0	0	0	0	0	0	0	0	0	0	0	0	0	0	0	0	0	0	0	0	0	0	0	0	0	0	0	0	0	0

ENSG00000221595	0	0	0	0	0	0	0	0	0	0	0	0	0	0	0	0	0	0	0	0	0	0	0	0	0	0	0	0	0	0	0	0	0	0	0	0	0	0	0	0	0	0	0.8581699	0	0	0	0	0
ENSG00000221598	0	0.65418153	0	0	0	0.36630802	1.17649512	1.91329011	0.96570336	0	1.23041652	1.23718139	0	0	0	0	0	0	1.07482965	1.300798	0.41107165	0	0	0.45400644	1.86466939	0	0.4201686	0.36821197	0.70133843	1.5712694	0	0	0	0.76856099	1.90211062	0	0.90378361	0.98415427	1.9675054	0.78440053	0	0	0	0.41691428	0.45044903	0	0.43484814	0
ENSG00000221599	0	0	0	0	0	0	0	0	0	0	0	0	0	0	0	0	0	0	0	0	0	0	0	0	0	0	0	0	0	0	0	0	0	0	0	0	0	0	0	0	0	0	0	0	0	0	0	0
ENSG00000221601	0	0	0	0	0	0	0	0	0	0	0	0	0	0	0	0	0	0	0	0	0	0	0	0	0	0	0	0	0	0	0	0	0	0	0	0	0	0	0	0	0	0	0	0	0	0	0	0
ENSG00000221602	0	0	0	0	0	0	0	0	0	0	0	0	0	0	0	0	0	0	0	0	0	0	0	0	0	0	0	0	0	0	0	0	0	0	0	0	0	0	0	0	0	0	0	0	0	0	0	0
ENSG00000221603	0	0	0	0	0	0	0	0	0	0	0	0	0	0	0	0	0	0	0	0	0	0	0	0	0	0	0	0	0	0	0	0	0	0	0	0	0	0	0	0	0	0	0	0	0	0	0	0
ENSG00000221604	0	0	0	0	0	0	0	0	0	0	0	0	0	0	0	0	0	0	0	0	0	0	0	0	0	0	0	0	0	0	0	0	0	0	0	0	0	0	0	0	0	0	0	0	0	0	0	0
ENSG00000221606	0	0	0.278939	0	0	0	0	0	0.14100978	0	0	0	0	0	0	0	0	0	0	0	0	0	0	0	0	0	0	0	0	0	0	0	0	0	0	0	0	0	0	0	0	0	0	0	0	0	0	0
ENSG00000221607	0	0	0	0	0	0	0	0	0	0	0	0	0	0	0	0	0	0	0	0	0.27684417	0	0	0	0	0	0	0	0	0	0	0	0	0	0	0	0	0	0	0	0	0	0	0	0	0	0	0
ENSG00000221608	0	0	0	0	0	0	0	0	0	0	0	0	0	0	0	0	0	0	0	0	0	0	0	0	0	0	0	0	0	0	0	0	0	0	0	0	0	0	0	0	0	0	0	0	0	0	0	0
ENSG00000221610	0.47396676	0.31747045	0.46353098	0	0	0.35553426	0	0	0	0	0	0	0	0	0	0	0	0	0	0	0	0	0	0	0	0	0	0	0	0	0	0	0	0	0	0	0	0	0	0	0	0	0	0.4046521	0	0	0	0
ENSG00000221611	0	0	0	0	0	0	0	0	0	0	0	0	0	0	0	0	0	0	0	0	0	0	0	0	0	0	0	0	0	0	0	0	0	0	0	0	0	0	0	0	0	0	0	0	0	0	0	0
ENSG00000221612	0	0	0	0	0	0	0	0	0	0	0	0	0	0	0	0	0	0	0	0	0	0	0	0	0	0	0	0	0	0	0	0	0	0	0	0	0	0	0	0	0	0	0	0	0	0	0	0
ENSG00000221613	0	0	0	0	0	0	0	0	0	0	0	0	0	0	0	0	0	0	0	0	0	0	0	0	0	0	0	0	0	0	0	0	0	0	0	0	0	0	0	0	0	0	0	0	0	0	0	0
ENSG00000221614	0	0	0	0	0	0	0	0	0	0	0	0	0.63440866	0	0.65186265	0.36152653	0.56378174	0	0.82486927	3.32762279	2.52378872	3.20078231	2.65744286	0.34842355	0.71551267	1.85051669	1.2898199	1.13032513	1.34559117	4.52196717	0	0	0	0	0	0	0	0	0	0	0	0	0	0.31995747	0.69138688	0	0.33372067	0.70074645
ENSG00000221615	0	0	0	0	0	0	0	0	0	0	0	0	0	0	0	0	0	0	0	0	0	0	0	0	0	0	0	0	0	0	0	0	0	0	0	0	0	0	0	0	0	0	0	0	0	0	0	0
ENSG00000221616	0	0	0	0	0	0	0	0	0	0	0	0	0	0	0	0	0	0	0	0	0	0	0	0	0	0	0	0	0	0	0	0	0	0	0	0	0	0	0	0	0	0	0	0	0	0	0	0
ENSG00000221617	0	0	0	0	0	0	0	0	0	0	0	0	0	0	0	0	0	0	0	0	0	0	0	0	0	0	0	0	0	0	0	0	0	0	0	0	0	0	0	0	0	0	0	0	0	0	0	0
ENSG00000221618	0	0	0	0	0	0	0	0	0	0	0	0	0	0	0	0	0	0	0	0	0	0	0	0	0	0	0	0	0	0	0	0	0	0	0	0	0	0	0	0	0	0	0	0	0	0	0	0
ENSG00000221619	0	0	0	0	0	0	0	0	0	0	0	0	0	0	0	0	0	0	0	0	0	0	0	0	0	0	0	0	0	0	0	0	0	0	0	0	0	0	0	0	0	0	0	0	0	0	0	0
ENSG00000221621	0	0	0	0	0	0	0	0	0	0	0	0	0	0	0	0	0	0	0	0	0	0	0	0	0	0	0	0	0	0	0	0	0	0	0	0	0	0	0	0	0	0	0	0	0	0	0	0
ENSG00000221623	0	0	0	0	0	0	0	0	0	0	0	0	0	0	0	0	0	0	0	0	0	0	0	0	0	0	0	0	0	0	0	0	0	0	0	0	0	0	0	0	0	0	0	0	0	0	0	0
ENSG00000221625	4.81593813	3.22579169	4.70990099	2.8731519	4.33962224	4.72411034	3.27254964	4.35438439	5.31136849	5.3684977	3.26696801	3.12850467	3.13558304	4.41463398	5.15495978	1.07211317	0.55730149	1.34058268	0.40769401	1.64468713	1.87108474	1.58199586	1.12581323	4.13302414	0.70728839	1.82924638	0.95624579	2.513999	3.72434889	1.48999685	1.12571453	3.17271475	3.64647201	4.66437016	2.88596095	6.59071506	4.79940263	10.0790972	5.97036123	2.38024988	6.22847173	3.98022083	2.1207647	3.47907781	4.78407931	8.1411794	3.29884798	2.42442162
ENSG00000221628	0	0	0	0	0	0	0	0	0	0	0	0	0	0	0	0	0	0	0	0	0	0	0	0	0	0	0	0	0	0	0	0	0	0	0	0	0	0	0	0	0	0	0	0	0	0	0	0
ENSG00000221629	0	0	0	0	0	0	0	0	0	0	0	0	0	0	0	0	0	0	0	0	0	0	0	0	0	0	0	0	0	0	0	0	0	0	0	0	0	0	0	0	0	0	0	0	0	0	0	0
ENSG00000221630	0	0	0	0.4578099	0	0	0	0	0	0	0	0	0	0	0	0	0	0	0	0	0	0	0	0	0	0	0	0	0.25433152	0	0	0	0	0	0	0	0	0	0	0	0	0	0	0	0	0	0	0
ENSG00000221631	0	0	0	0	0	0	0	0	0	0.25383658	0	0	0	0	0	0	0	0	0	0	0	0	0	0	0	0	0	0	0	0	0	0	0	0	0	0	0	0	0	0	0	0	0	0	0	0	0	0
ENSG00000221633	0	0	0	0	0	0	0	0	0	0	0	0	0	0	0	0	0	0	0	0	0	0	0	0	0	0	0	0	0	0	0	0	0	0	0	0	0	0	0	0	0	0	0	0	0	0	0	0
ENSG00000221634	0	0	0.75952064	0	0.8664306	0.87384323	0	0	0	0	0.48920175	0	0	0	0	0	0	0	0	0	0	0	0	0	0	0	0	0	0	0.31236078	0	0	0	0	0	0	0	0.39129025	0	0	0	0	0	0	0	0.53334534	0	0
ENSG00000221635	0	0	0	0	0	0	0.1848778	0	0	0	0	0	0	0	0	0	0	0	0	0	0	0	0	0	0	0	0	0	0	0	0	0.28165937	0	0	0	0	0	0	0	0	0	0	0	0	0	0	0	0
ENSG00000221637	0	0	0	0	0	0	0	0	0	0	0	0	0	0	0	0	0	0	0	0	0	0	0	0	0	0	0	0	0	0	0	0	0	0	0	0	0	0	0	0	0	0	0	0	0	0	0	0
ENSG00000221638	0	0	0	0	0	0	0.12038555	0	0	0	0	0	0	0	0	0	0	0	0	0	0	0	0	0	0	0	0	0	0	0	0	0	0	0	0	0	0	0	0	0	0	0	0	0	0	0	0	0
ENSG00000221639	1.03135167	1.03622355	1.00864341	0	0.28765496	2.51433826	1.24237885	1.26277147	1.01978275	0.9341186	1.29931985	0.8709757	0.43647316	0	0.896963	0.74619077	0	0	0.56751006	0.22894045	0.43409166	0.44042765	0.52237734	0.2397154	0.73840908	0.84877032	0.88739609	0.38883185	1.6663801	1.24444537	0.39174866	0.63091699	0.42299075	0.4058002	0.5021572	0	3.10178535	2.07853383	1.29855357	1.24249044	1.44500544	0.46170562	0	0.66039223	1.90269669	0.3541413	0.45919964	0.96422711
ENSG00000221641	0	0	0	0	0	0	0	0	0	0	0	0	0	0	0	0	0	0	0	0	0	0	0	0	0	0	0	0	0	0	0	0	0	0	0	0	0	0	0	0	0	0	0	0	0	0	0	0
ENSG00000221643	0.25783792	0.51811177	0	0	0	0	0.41412628	0.25255429	0	0	0.32482996	1.52420748	0	0	0	0	0	0	0.56751006	0.4578809	0	0	0	0.2397154	0.98454544	1.27315548	0.22184902	0.58324777	0.55546003	0.82963024	0	0	0	0.4058002	0	0.91742754	0.23859887	0.51963346	0	0.82832696	0.48166848	1.38511685	0.29521045	0.22013074	0	0.3541413	0	0.24105678
ENSG00000221644	0	0	0	0	0	0	0	0	0	0	0	0	0	0	0	0	0	0	0	0	0	0	0	0	0	0	0	0	0	0	0	0	0	0	0	0	0	0	0	0	0	0	0	0	0	0	0	0
ENSG00000221645	0	0	0	0	0	0	0	0	0	0	0	0	0	0	0	0	0	0	0	0	0	0	0	0	0	0	0	0	0	0	0	0	0	0	0	0	0	0	0	0	0	0	0	0	0	0	0	0
ENSG00000221649	0	0	0	0	0	0	0	0	0	0	0	0	0	0	0	0	0	0	0	0	0	0	0	0	0	0	0	0	0	0	0	0	0	0	0	0	0	0	0	0	0	0	0	0	0	0	0	0
ENSG00000221650	0	0	0	0.5341116	0.23049276	0	0	0	0	0.37424623	0	0	0.34973811	0	0.71872035	0	0	0	0.45473562	0	0.34782986	0.35290677	0	0.76831859	0	0	1.06658184	0.62312796	0.89016031	0.33238391	0	0	0	0	0	0	1.52947996	0	0	0	0.77190462	0	0.47309366	1.05832087	0	0	0	0.38630894
ENSG00000221653	0	0	0	0	0	0	0	0	0	0	0	0	0	0	0	0	0	0	0	0	0	0	0	0	0	0	0	0	0	0	0	0	0	0	0	0	0	0	0	0	0	0	0	0	0	0	0	0
ENSG00000221654	0	0	0	0	0	0	0	0	0	0	0	0	0	0	0	0	0	0	0	0	0	0	0	0	0	0	0	0	0	0	0	0	0	0	0	0	0	0	0	0	0	0	0	0	0	0	0	0
ENSG00000221655	0	0	0	0	0	0	0	0	0	0	0	0	0	0	0	0	0	0	0	0	0	0	0	0	0	0	0	0	0	0	0	0	0	0	0	0	0	0	0	0	0	0	0	0	0	0	0	0
ENSG00000221656	0	0.23986656	0	0	0	0	0	0	0	0	0	0	0.30310636	0	0	0	0	0	0	0	0	0	0	0	0	0	0	0	0	0	0	0	0	0.56361139	0	0	0	0	0.36070932	0	0	0	0	0	0	0	0	0
ENSG00000221657	0	0	0	0	0	0	0	0	0	0	0	0	0	0	0	0	0	0	0	0	0	0	0	0	0	0	0	0	0	0	0	0	0	0	0	0	0	0	0	0	0	0	0	0	0	0	0	0
ENSG00000221659	0	0	0	0	0	0	0	0	0	0	0	0	0	0	0	0	0	0	0	0	0	0	0	0	0	0	0	0	0	0	0	0	0	0	0	0	0	0	0	0	0	0	0	0	0	0	0	0
ENSG00000221660	0	0	0	0	0	0	0	0	0	0	0	0	0	0	0	0	0	0	0	0	0	0	0	0	0	0	0	0.26415207	0	0	0	0	0	0	0	0	0	0	0	0	0	0	0	0	0	0	0	0
ENSG00000221662	0	0	0	0	0	0	0	0.80946889	0	0	0	0	0	0	0	0	0	0	0	0	0	0	0	0	0	0	0	0	0	0	0	0	0	0	0	0	0	0	0.41620307	0	0	0.73991285	0	0	0	0	0	0
ENSG00000221664	0	0	0	0	0	0	0	0	0	0	0	0	0	0	0	0	0	0	0	0	0	0.25725914	0	0.28004136	0	0	0	0	0	0	0	0	0	0	0	0	0.278737	0	0.30340037	0	0	0	0	0	0	0	0	0
ENSG00000221665	0	0	0	0	0	0	0	0	0	0	0	0	0	0	0	0	0	0	0	0	0	0	0	0	0	0	0	0	0	0	0	0	0	0	0	0	0	0	0	0	0	0	0	0	0	0	0	0
ENSG00000221667	0	0	0	0	0	0	0	0	0	0	0	0	0	0	0	0	0	0	0	0	0	0	0	0	0	0	0	0	0	0	0	0	0	0	0	0	0	0	0	0	0	0	0	0	0	0	0	0
ENSG00000221669	0.42972986	0.28783987	0.42026809	0	0.23971247	0.32235106	0	0	0	0.38921608	0	0	0.36372763	0	0	0	0.64646972	0	0	0	0	0.36702304	0	0	0	0	0.73949674	0	0	0	0	0	0	0.67633367	0	0.38226147	0	0.43302788	0	0	0	0.76950936	0	0	0	0	0	0.80352259
ENSG00000221670	0	0	0	0	0	0	0	0	0	0	0	0	0	0	0	0	0	0	0	0	0	0	0	0	0	0	0	0	0	0	0	0	0	0	0	0	0	0	0	0	0	0	0	0	0	0	0	0
ENSG00000221672	0	0	0	0	0	0.33118259	0.35456017	0	0	0	0	0	0	0	0	0	0	0	0	0	0	0	0	0	0.42146637	0	0	0	0	0	0	0	0.72429923	0	0	0	0	0	0	0	0	0	0	0	0	0	0	0
ENSG00000221673	0	0	0	0	0	0	0	0	0	0	0	0	0	0	0	0	0	0	0	0	0	0	0	0	0	0	0	0	0	0	0	0	0	0	0	0	0	0	0	0	0	0	0	0	0	0	0	0
ENSG00000221676	7.16216439	2.0559991	6.75430856	10.580496	4.99400973	4.98876638	2.05419783	6.51429728	4.80552387	1.62173368	11.923322	6.91250556	1.5155318	3.04819966	5.33906548	0.74026862	3.07842726	7.40512338	18.2976953	3.86109882	6.02905083	8.95710987	2.59115744	9.75032878	10.2556816	12.2094937	13.205299	14.2725975	7.16367108	7.20165143	4.27503495	2.81659371	0.83926737	4.02579567	0.99634366	3.41304887	12.308672	12.1144705	22.6731575	6.98489996	16.7246	10.5349496	5.56448263	1.74706938	5.19088878	2.45931461	3.18888638	4.0654417
ENSG00000221677	0	0	0	0	0	0	0	0	0	0	0	0	0	0	0	0	0	0	0	0	0	0	0	0	0	0	0	0	0	0	0	0	0	0	0	0	0	0	0	0	0	0	0	0	0	0	0	0
ENSG00000221679	0	0	0	0	0	0	0	0	0	0	0	0	0	0	0	0	0	0	0	0	0	0	0	0	0	0.22670147	0	0	0	0	0	0	0	0	0	0	0	0	0	0	0	0	0	0	0	0	0	0
ENSG00000221680	0	0	0.38913712	0.5143297	0	0	0	0	0	0	1.00256161	0	0	0	0.34605054	0.38384299	0	0	0	0	0	0.33983615	0	0.36993117	0	0	0	0	0	0	0	0	0	0	0	0	0	0.40095174	0	0	0	0	0	0	0	0	0	0.3720012
ENSG00000221681	0	0.21587991	0	0	0	0.48352659	0	0	0	0	0	0	0	0	0.28030094	0.31091282	0	0	0	0	0	0	0	0	0	0	0.27731128	0	0	0	0	0.39432312	0	0	0	0	0	0.32477091	0	0	0	0	0	0	0.29729636	0	0	0
ENSG00000221683	0	0	0	0	0	0	0	0	0	0	0	0	0	0	0	0	0	0	0	0	0	0	0	0	0	0	0	0	0	0	0	0	0	0	0	0	0	0	0	0	0	0	0	0	0	0	0	0
ENSG00000221684	0	0	0	0	0	0	0	0	0	0	0	0	0	0	0	0	0	0	0	0	0	0	0	0	0	0	0	0	0	0	0	0	0	0	0	0	0	0	0	0	0	0	0	0.38755412	0	0	0.40422503	0
ENSG00000221685	0	0	0	0	0	0	0	0	0	0	0	0	0	0	0	0	0	0	0	0	0	0	0	0	0	0	0	0	0	0	0	0	0	0	0	0	0	0	0	0	0	0	0	0	0	0	0	0
ENSG00000221686	0	0	0	0	0	0	0	0	0	0	0	0	0	0	0	0	0	0	0	0	0	0	0	0	0	0	0	0	0	0	0	0	0	0	0	0	0	0	0	0	0	0	0	0	0	0	0	0
ENSG00000221689	0	0	0	0	0	0	0	0	0	0	0	0	0	0	0	0	0	0	0	0	0	0	0	0	0	0	0	0	0	0	0	0	0	0	0	0	0	0	0	0	0	0	0	0	0	0	0	0
ENSG00000221690	0	0	0	0	0	0	0	0	0	0	0	0	0	0	0	0	0	0	0	0	0	0	0	0	0	0	0	0	0	0	0	0	0	0	0	0	0	0	0	0	0	0	0	0	0	0	0	0
ENSG00000221691	0	0	0	0	0	0	0	0	0	0	0	0	0	0	0	0	0	0	0	0	0	0	0	0	0	0	0	0	0	0	0	0	0	0	0	0	0	0	0	0	0	0	0	0	0	0	0	0
ENSG00000221693	0	0	0	0	0	0	0	0	0	0	0	0	0	0	0	0	0	0	0	0	0	0	0	0	0	0	0	0	0	0	0	0	0	0	0	0	0	0	0	0	0	0	0	0	0	0	0	0
ENSG00000221695	0	0	0	0	0	0	0	0	0	0	0	0	0	0	0	0	0	0	0	0	0	0	0	0	0	0	0	0	0	0	0	0	0	0	0	0	0	0	0	0	0	0	0	0	0	0	0	0
ENSG00000221696	0	0	0	0	0	0	0	0	0	0	0	0	0	0	0	0	0	0	0	0	0	0	0	0	0	0	0	0	0	0	0	0	0	0	0	0	0	0	0	0	0	0	0	0	0	0	0	0
ENSG00000221697	0	0	0	0	0	0	0	0	0	0	0	0	0	0	0	0	0	0	0	0	0	0	0	0	0	0	0	0	0	0	0	0	0	0	0	0	0	0	0	0	0	0	0	0	0	0	0	0
ENSG00000221698	0	0	0	0	0	0	0	0	0	0	0	0.23066094	0.23118282	0	0	0	0.41089177	0	0	0	0.22992143	0	0	0	0	0	0	0.20594907	0	0	0	0	0	0	0	0	0	0	0	0	0	0	0	0	0	0	0	0.25535676
ENSG00000221699	0	0	0	0	0	0	0	0	0	0	0	0	0	0	0	0	0	0	0	0	0	0	0	0	0	0	0	0	0	0	0	0	0	0	0	0	0	0	0	0	0	0	0	0	0	0	0	0
ENSG00000221701	0	0	0	0	0	0	0	0	0	0	0	0	0	0	0	0	0	0	0	0	0	0	0	0	0	0	0	0	0	0	0	0	0	0	0	0	0	0	0	0	0	0	0	0	0	0	0	0
ENSG00000221702	0	0	0	0	0	0	0	0	0	0	0	0	0	0	0	0	0	0	0	0	0	0	0	0	0	0	0	0	0	0	0	0	0	0	0	0	0	0	0	0	0	0	0	0	0	0	0	0
ENSG00000221703	0	0	0	0	0	0	0	0	0	0	0	0	0	0	0	0	0	0	0	0	0	0	0	0	0	0	0	0	0	0	0	0	0	0	0	0	0	0	0	0	0	0	0	0	0	0	0	0
ENSG00000221704	0	0	0.86356457	0	0	0	0	0	0	0	0	0	0	0	0	0	0	0	0	0	0	0	0	0	0.42146637	0	0	0	0	0	0	0	0	0	0	0.39273439	0	0	0	0	0	0	0	0	0	0	0	0
ENSG00000221705	0	0	0	0	0	0	0	0	0	0	0	0	0	0	0	0	0	0	0	0	0	0	0	0	0	0	0	0	0	0	0	0	0	0	0	0	0	0	0	0	0	0	0	0	0	0	0	0
ENSG00000221706	0	0	0	0	0	0	0	0	0	0	0	0	0	0	0	0	0	0	0	0	0	0	0	0	0	0	0	0	0	0	0	0	0	0	0	0	0	0	0	0	0	0	0	0	0	0	0	0
ENSG00000221707	0	0	0	0	0	0	0	0	0	0	0	0	0	0	0	0	0	0	0	0	0	0	0	0	0	0	0	0	0	0	0	0	0	0	0	0	0	0	0	0	0	0	0	0	0	0	0	0
ENSG00000221708	0	0	0	0	0	0	0	0	0	0	0	0	0	0	0	0	0	0	0	0	0	0	0	0	0	0	0	0	0	0	0	0	0	0	0	0	0	0	0	0	0	0	0	0	0	0	0	0
ENSG00000221710	0	0	0	0	0	0	0	0	0	0	0	0	0	0	0	0	0	0	0	0	0	0	0	0	0	0	0	0	0	0	0	0	0	0	0	0	0	0	0	0	0	0	0	0	0	0	0	0
ENSG00000221711	0	0	0	0	0	0	0	0	0	0	0	0	0	0	0	0	0	0	0	0	0	0	0	0	0	0	0	0	0	0	0	0	0	0.8745694	0	0	0	0	0	0	0	0	0	0	0	0	0	0
ENSG00000221714	0	0	0	0	0	0	0	0	0	0	0	0	0	0	0	0	0	0	0	0	0	0	0	0	0	0	0	0	0	0	0	0	0	0	0	0	0	0	0	0	0	0	0	0	0	0	0	0
ENSG00000221716	2.01435874	1.01193706	0	2.2783197	0.28091305	2.26653088	0.4044202	0	0.62242599	1.36833779	0.95165028	0.21264055	0.85248664	0.54555846	1.97086597	0	0.37879085	0.30372576	3.04814972	3.5771945	2.331547	1.93547306	3.8260059	0.46819414	1.92294031	0.82887727	2.16649436	1.5188744	3.07383483	3.24074314	1.14770114	0.30806494	0.82615381	0	0.4903879	0.44796266	1.39804027	0.76118182	0.76087123	0.40445652	0	0.90176878	0.28829145	1.28982856	0.46452556	0.34584112	0.44843715	2.11866309
ENSG00000221717	0	0	0.30902065	0	0.17625917	0.47404567	0	0	0	0	0	0	0	0	0	0	0	0	0	0	0	0	0	0	0	0	0	0	0	0	0	0	0	0	0	0	0	0	0	0	0	0	0	0	0	0	0	0
ENSG00000221718	0	0	0	0	0	0	0	0	0	0	0	0	0	0.67145657	0.53904027	0	0	0	0	0	0	0	0	0	0	0	0	0	0	0	0	0	0	0	0	0	0	0	0	0	0	1.10986927	0	0	0	0	0	0
ENSG00000221719	0	0	0	0	0	0	0	0	0	0	0	0	0	0	0	0	0	0	0	0	0	0	0	0	0	0	0	0	0	0	0	0	0	0	0	0	0	0	0	0	0	0	0	0	0	0	0	0
ENSG00000221720	0	0.2998332	0.43777926	0	0.24970049	0.33578235	0	0	0	0	0	0	0	0	0	0	0	0	0	0	0	0	0	0	0	0	0	0	0	0	0	0	0	0	0	0	0	0	0	0	0	0	0	0	0	0	0	0
ENSG00000221721	0	0	0	0	0	0	0	0	0	0	0	0	0	0	0	0	0	0	0	0	0	0	0	0	0	0	0	0	0	0	0	0	0	0	0	0	0	0	0	0	0	0	0	0	0	0	0	0
ENSG00000221723	0	0	0	0	0	0	0	0	0	0	0	0	0	0	0	0	0	0	0	0	0	0	0	0	0	0	0	0	0	0	0	0	0	0	0	0	0	0	0	0	0	0	0	0	0	0	0	0
ENSG00000221724	0	0	0	0	0	0	0	0	0	0	0	0	0	0	0	0	0	0	0	0	0	0	0	0	0	0	0	0	0	0	0	0	0	0	0	0	0	0	0	0	0	0	0	0	0	0	0	0
ENSG00000221725	0	0	0	0	0	0	0	0	0	0	0	0	0	0	0	0	0	0	0	0	0	0	0	0	0	0	0	0	0	0	0	0	0	0	0	0	0	0	0	0	0	0	0	0	0	0	0	0
ENSG00000221726	0	0	0	0	0	0	0	0	0	0	0	0	0	0	0	0	0	0	0	0	0	0	0	0	0	0	0	0	0	0	0	0	0	0	0	0	0	0	0	0	0	0	0	0	0	0	0	0.3275228
ENSG00000221729	0	0	0	0	0	0	0	0	0	0	0	0	0	0	0	0	0	0	0	0	0	0	0	0	0	0	0	0	0	0	0	0	0	0	0	0	0	0	0	0	0	0	0	0	0	0	0	0
ENSG00000221730	0	0	0	0	0	0	0	0	0	0	0	0	0	0	0	0	0	0	0	0	0	0	0	0	0	0	0	0	0	0	0	0	0	0	0	0	0	0	0	0	0	0	0	0	0	0	0	0
ENSG00000221731	0	0	0	0	0	0	0	0	0	0	0	0	0	0	0	0	0	0	0	0	0	0	0	0	0	0	0	0	0	0	0	0	0	0	0	0	0	0	0	0	0	0	0	0	0	0	0	0
ENSG00000221732	0	0	0	0	0	0	0	0	0	0	0	0	0	0	0	0	0	0	0	0	0	0	0	0	0	0	0	0	0	0	0	0	0	0	0	0	0	0	0	0	0	0	0	0	0	0	0	0
ENSG00000221733	0	0	0	0	0	0	0	0	0	0	0	0	0	0	0	0	0	0	0	0	0	0	0	0	0	0	0	0	0	0	0	0	0	0	0	0	0	0	0	0	0	0	0	0	0	0	0	0
ENSG00000221734	0	0.2957259	0	0	0.49255986	0.33118259	0	0	0.21827542	0.39987954	0	0	0	0	0	0	0	0	0	0	0	0	0	0	0	0	0	0	0	0	0	0	0	0	0	0	0	0	0	0	0	0	0	0	0	0	0.39315038	0
ENSG00000221736	0.36624704	0	0	0	0.4086008	0	0	0	0	0	0	0	0	0	0	0	0	0	0	0	0	0	0	0	0	0	0	0	0	0	0	0	0	0	0	0	0	0	0	0	0	0	0	0	0	0	0	0
ENSG00000221737	0	0	0	0	0	0	0	0	0	0	0	0	0	0	0	0	0	0	0	0	0	0	0	0	0	0	0	0	0	0	0	0	0	0	0	0.19241349	0	0	0	0	0	0	0	0	0	0	0	0
ENSG00000221739	0	0	0	0	0	0	0	0	0	0	0	0	0	0	0	0	0	0	0	0	0	0	0	0	0	0	0	0	0	0	0	0	0	0	0	0	0	0	0	0	0	0	0	0	0	0	0	0
ENSG00000221740	0	0	0	0	0	0	4.54699465	5.97256778	3.44521199	5.9171364	7.68178963	6.62059232	3.31778584	1.41550303	1.89392526	2.94106721	1.9656174	0.52536348	0.47931593	1.54689492	1.46652588	1.85991405	1.32359123	3.2393973	2.49462526	3.22590071	0.74948994	0.32840527	0.62551806	1.75175305	0	1.06573816	1.42902281	0.68547332	2.54471556	0.38742717	8.06077275	12.7275087	12.2836148	2.79840189	8.1362919	4.67944882	2.49333148	2.23105481	2.81226284	1.19642332	3.87837533	4.07190504
ENSG00000221741	0	0	0	0	0	0	0	0	0	0	0	0	0	0	0	0	0	0	0	0	0	0.22749362	0	0	0	0	0	0	0	0	0	0	0	0	0	0	0	0	0	0	0	0	0	0	0	0	0	0
ENSG00000221743	0	0	0.3463748	0	0	0	0	0	0	0	0	0	0	0	0	0	0	0	0	0.94343591	0	0	0	0	0	0	0	0	0	0	0	0	0	0	0	0	0	0	0	0	0	0	0	0	0	0	0	0
ENSG00000221744	0	0	0	0	0	0	0	0	0	0	0	0	0	0	0	0	0	0	0	0	0	0	0	0	0	0	0	0	0	0	0	0	0	0	0	0	0	0	0	0	0	0	0	0	0	0	0	0
ENSG00000221745	0	0	0	0	0	0	0	0	0	0	0	0	0	0	0.31852379	0.35331002	0	0	0.80612224	0.650399	0	0.62560745	0.37100663	0.34050483	0.34962551	0.60281983	1.26050581	0.55231796	0.52600382	1.17845205	0	0	0	0	0	0	0	0	0	0	0	0	0	0.93805714	0	0	0.32613611	0
ENSG00000221746	0	0	0	0	0	0	0	0	0	0	0	0	0	0	0	0	0	0	0	0	0	0	0	0	0	0	0	0	0	0	0	0	0	0	0	0	0	0	0	0	0	0	0	0	0	0	0	0
ENSG00000221747	0	0	0	0	0	0	0	0	0	0	0	0	0	0	0	0	0	0	0	0.34478983	0	0	0	0.36101717	0.37068729	0	0	0	0	0	0	0	0	0	0	0	0	0	0	0	0	0	0	0	0	0	0	0
ENSG00000221748	0	0	0	0	0	0	0	0	0	0	0	0	0	0.45345119	0	0	0	0	0	0	0	0	0	0	0	0	0	0	0	0	0	0	0	0	0	0	0	0	0	0	0	0	0	0	0	0	0	0
ENSG00000221749	0	0	0	0	0	0	0	0	0	0	0	0	0	0	0	0	0	0	0	0	0	0	0	0	0	0	0	0	0	0	0	0	0	0	0	0	0	0	0	0	0	0	0	0	0	0	0	0
ENSG00000221750	0	0	0	0	0	0	0	0	0	0	0	0	0	0	0	0	0	0	0	0	0	0	0	0	0	0	0	0	0	0	0	0	0	0	0	0	0	0	0	0	0	0	0	0	0	0	0	0
ENSG00000221751	0	0	0	0	0	0	0	0	0	0	0	0	0	0	0	0	0	0	0	0	0	0	0	0	0	0	0	0	0	0	0	0	0	0	0	0	0	0	0	0	0	0	0	0	0	0	0	0
ENSG00000221753	0.31291009	0.20959214	0	0.4044728	0.17454791	0	0	0	0.15470005	0.28340977	0.39421112	0	0.26485022	0	0.27213683	0	0	0	0	0.27784035	0	0	0	0	0.29870917	0	0	0	0.22470066	0	0	0	0	0	0	0	0.28956174	0	0.3151829	0	0	0	0.35826511	0.26714896	0	0	0	0.58508927
ENSG00000221754	0	0	0	0	0	0	0	0	0	0	0	0	0	0	0	0	0	0	0	0	0	0	0	0	0	0	0	0	0	0	0	0	0	0	0	0	0	0	0	0	0	0	0	0	0	0	0	0
ENSG00000221755	0	0	0	0	0	0	0	0	0	0	0	0	0.24799611	0	0	0	0	0	0	0	0	0	0	0	0	0	0	0	0	0	0	0	0	0	0	0	0	0	0	0	0	0	0	0	0	0	0	0
ENSG00000221756	0	0	0	0	0	0	0	0	0	0	0	0	0	0	0	0	0	0	0	0	0	0	0	0	0	0	0	0	0	0	0	0	0	0	0	0	0	0	0	0	0	0	0	0	0	0	0	0
ENSG00000221760	0	0	0	0.3719706	0	0	0	0	0	0	0	0	0.24356761	0	0	0	0	0	0	0	0	0	0	0	0.27470576	0	0	0	0	0.46296331	0	0	0	0	0	0	0	0	0	0	0	0	0	0.24568163	0	0	0	0.80710975
ENSG00000221762	0	0	0	0	0.211511	0	0	0	0	0	0	0	0	0	0	0	0	0	0	0	0	0	0	0	0	0	0	0	0	0	0	0	0	0	0	0	0	0	0	0	0	0	0	0	0	0	0	0
ENSG00000221763	0.22381764	0	0.21888963	0	0.12485024	0.33578235	0.35948462	0.43846232	0.11065351	0.60815013	0	0.18901382	0.18944148	0.48494085	0.19465343	0.43182336	0.67340596	0.26997846	0.98526051	1.19239817	0.18840784	0.38231567	0.9069051	0.20808628	0	0	0.57773183	0.67505529	0.48217017	0.18004129	0	0	0	0	0	0	0	0	0.45088666	0.71903382	0	0	0.25625907	0.76434285	0.41291161	0.30741433	0.1993054	0
ENSG00000221764	0	0	0	0	0	0	0	0	0	0	0	0	0	0	0	0	0	0	0	0	0	0	0	0	0	0	0	0	0	0	0	0	0	0	0	0	0	0	0	0	0	0	0	0	0	0	0	0
ENSG00000221766	0	0	0	0	0	0	0	0	0	0	0	0	0	0	0	0	0	0	0	0	0	0	0	0	0	0	0	0	0	0	0	0	0	0	0	0	0	0	0	0	0	0	0	0	0	0	0	0
ENSG00000221767	0.43261396	0.72442921	0.63463302	0.279602	0.12066064	0.64902898	5.73245273	5.93248343	3.10126885	6.85699476	2.18006686	3.65342156	1.09850627	0.93733534	0.5643643	2.29532954	1.95242534	0.52183755	1.42829712	4.03334682	1.63876885	1.66268826	2.19118011	1.20662114	0.41298047	0.71205564	0.55834486	0.48930182	1.08730991	0.5219989	0	0.26464639	0.35485801	1.02130924	0.84254565	0.19241349	2.0016684	0.65390116	3.26817173	2.77962067	1.61633718	1.54934771	0.74297931	1.29271409	1.3966943	0.89129523	0.57785189	2.62897493
ENSG00000221768	0	0	0	0	0	0	0	0	0	0	0	0	0	0	0	0	0	0	0	0	0	0	0	0	0	0	0	0	0	0	0	0	0	0	0	0	0	0	0	0	0	0	0	0	0	0	0	0
ENSG00000221770	0	0	0	0	0	0	0	0	0	0	0	0	0	0	0	0	0	0	0	0	0	0	0	0	0	0	0	0	0	0	0	0	0	0	0	0	0	0	0	0	0	0	0	0	0	0	0	0
ENSG00000221771	0	0	0	0	0	0	0.82167913	0	0.25292231	0	0	0	0	0	0	0	0	0	0	0	0.43064649	0	0	0	0	0	0.44017663	0	0.36736775	0.41152294	0	0	2.5178021	0	0	0	0	0.51550938	0.51529903	0	0	0	0	0.43676734	0	0	0.4555552	0
ENSG00000221772	0	0	0	0	0	0	0	0	0	0	0	0	0	0	0	0	0	0	0	0	0	0	0	0	0	0	0	0	0	0	0	0	0	0	0	0	0	0	0	0	0	0	0	0	0	0	0	0
ENSG00000221774	0	0.24813782	0	0	0	0.55577769	0	0	0	0	0	0	0	0	0	0	0	0	0	0.32893743	0	0	0	0	0	0	0	0	0.26602492	0	0	0	0	0	0	0	0	0	0.37314758	0	0	0	0	0	0.34171995	0	0	0
ENSG00000221777	0	0	0	0	0	0	0	0	0	0	0	0	0	0	0	0	0	0	0	0	0	0	0	0	0	0	0	0	0	0	0	0	0	0	0	0	0	0	0	0	0	0	0	0	0	0	0	0
ENSG00000221778	0	0	0	0	0	0	0	0	0	0	0	0	0	0	0	0	0	0	0	0	0	0	0	0	0	0	0	0	0	0	0	0	0	0	0.66776224	0	0	0	0	0	0	0	0	0	0	0	0	0.32055423
ENSG00000221779	0	0	0	0	0	0	0	0	0	0	0	0	0	0	0	0	0	0	0	0	0	0	0	0	0	0	0	0	0	0	0	0	0	0	0	0	0	0	0	0	0	0	0	0	0	0	0	0
ENSG00000221781	0	0	0	0	0	0	0	0	0	0	0	0	0	0	0	0	0	0	0	0	0	0	0	0	0	0	0	0	0	0	0	0	0	0	0	0	0	0	0	0	0	0	0	0	0	0	0	0
ENSG00000221782	0	0.22028562	0.32163374	0	0	0	0	0	0	0.29786945	0	0	0	0	0	0	0	0	0	0	0	0	0	0.61151888	0	0	0	0	0	0	0	0	0.53952902	0	0	0	0	0	0	0	0.61437306	0	0	0	0	0	0	0
ENSG00000221783	0	0.24256169	0.3541585	0.4680978	0.20200489	0	0	0	0.17903489	0.32799108	0	0	0	0	0	0	0	0	0	0	0	0	0	0	0.34569713	0	0	0	0	0	0	0	0	0	0	0	0	0.36491114	0	0	0	0	0	0	0	0	0	0
ENSG00000221785	0	0	0	0	0	0	0	0	0	0	0	0	0	0	0	0	0	0	0	0	0	0	0	0	0	0	0	0	0	0	0	0	0	0	0	0	0	0	0	0	0	0	0	0	0	0	0	0
ENSG00000221786	0	0	0	0	0	0	0	0	0	0	0	0	0	0	0	0	0	0	0	0	0	0	0	0	0	0	0	0	0	0	0	0	0	0	0	0	0	0	0	0	0	0	0	0	0	0	0	0
ENSG00000221788	0	0	0.48492472	0	0.27659131	0	0	0	0	0	0	0	0	0	0	0	0	0	0	0	0	0	0	0	0	0	0	0	0	0	0	0	0.81344376	0	0	0	0	0	0	0	0	0	0	0	0	0	0	0
ENSG00000221789	0	0	0	0	0	0	0	0	0	0	0	0	0	0	0	0	0	0	0	0	0	0	0	0	0	0	0	0	0	0	0	0	0	0	0	0	0	0	0	0	0	0	0	0	0	0	0	0
ENSG00000221790	0	0	0	0	0	0	0	0	0	0	0	0	0	0	0	0	0	0	0	0	0	0	0	0	0	0	0	0	0	0	0	0	0	0	0	0	0	0	0	0	0	0	0	0	0	0	0	0
ENSG00000221792	2.55285068	0.85496992	1.24832105	1.2374466	0.53401292	0.71810879	0.51253253	2.18797037	0.47329026	0.86706553	1.60806912	0.53897011	0.81028433	0.34570041	1.11010272	0.92350342	1.9202071	1.53967912	1.75590983	0.85002642	1.61172646	1.09016744	1.93951982	2.07674233	2.13236945	3.15137496	3.29478747	2.40613766	1.1457509	1.0267701	4.36353702	3.12335144	0.52350341	2.51113987	0.62148169	1.41928765	2.06706945	2.2508875	3.2142415	0	0	0.57141784	0.36535946	0.81731711	2.0604698	0.43829369	0.56831638	0.89501279
ENSG00000221793	0	0	0	0	0	0	0	0	0	0	0	0	0	0	0	0	0	0	0	0	0	0	0	0	0	0	0	0	0	0	0	0	0	0	0	0	0	0	0	0	0	0	0	0	0	0	0	0
ENSG00000221795	0	0	0	0	0	0.25183676	0	0	0	0	0	0	0	0	0	0	0	0	0	0	0	0	0	0	0	0	0	0	0	0	0	0.41075325	0	0	0	0	0	0	0	0	0	0	0	0	0	0	0	0
ENSG00000221796	0	0	0	0	0	0	0	0	0	0	0	0	0	0	0	0	0	0	0	0	0	0	0	0	0	0	0	0	0	0	0	0	0	0	0	0	0	0	0	0	0	0	0	0	0	0	0	0
ENSG00000221798	0	0	0	0	0	0	0	0	0	0	0	0	0	0	0	0	0	0	0	0	0	0	0	0	0	0	0	0	0	0	0	0	0	0	0	0	0	0	0	0	0	0	0	0	0	0	0	0
ENSG00000221799	0	0	0	0	0	0	0	0	0	0	0	0	0	0	0	0	0	0	0	0	0	0	0	0	0	0	0	0	0	0	0	0	0	0	0	0	0	0	0	0	0	0	0	0	0	0	0	0
ENSG00000221801	0	0	0	0	0	0	0	0	0	0	0	0	0	0	0	0	0	0	0	0	0	0	0	0	0	0	0	0	0	0	0	0	0	0	0	0	0	0	0	0	0	0	0	0	0	0	0	0
ENSG00000221802	0	0	0	0	0	0	0	0	0	0	0	0	0	0	0	0	0	0	0	0	0	0	0	0	0	0	0	0	0	0	0	0	0	0	0	0	0	0	0	0	0	0	0	0	0	0	0	0
ENSG00000221803	0	0	0	0	0	0	0	0	0	0	0	0	0	0	0	0	0	0	0	0	0	0	0	0	0	0	0	0	0	0	0	0	0	0	0	0	0	0	0	0	0	0	0	0	0	0	0	0
ENSG00000221804	0	0	0	0	0	0	0	0	0	0	0	0	0	0	0	0	0	0	0	0	0	0	0	0	0	0	0	0	0	0	0	0	0	0	0	0	0	0	0	0	0	0	0	0	0	0	0	0
ENSG00000221806	0	0	0	0	0	0	0	0	0	0	0	0	0	0	0	0	0	0	0	0	0	0	0	0	0	0	0	0	0	0	0	0	0	0	0	0	0	0	0	0	0	0	0	0	0	0	0	0
ENSG00000221807	0	0	0	0	0	0	0	0	0	0	0	0	0	0	0	0	0	0	0	0	0	0	0	0	0	0	0	0	0	0	0	0	0	0	0	0	0	0	0	0	0	0	0	0	0	0	0	0
ENSG00000221808	0	0.18141169	0.26487485	0.3500899	0	0.20316243	0.2175033	0	0	0	0	0	0	0	0	0	0	0	0	0	0	0.46263408	0	0	0	0	0	0	0	0	0	0	0.44431802	0.85252144	0	0	0.25062907	0	0	0	0	0	0	0	0	0	0.24117628	0
ENSG00000221809	0	0	0	0	0	0	0	0	0	0	0	0	0	0	0	0	0	0	0	0	0	0	0	0	0	0	0	0	0	0	0	0	0	0	0	0	0	0	0	0	0	0	0	0	0	0	0	0
ENSG00000221810	0	0	0	0	0	0	0	0	0	0	0	0.33192672	0	0	0.34183041	0.37916197	0.59128328	0	0	0	0	0	0	0	0	0	0	0	0	0	0	0	0	0	0	0	0	0	0	0	0	0	0	0	0	0	0	0
ENSG00000221813	0	0	0	0	0	0	0	0.03015214	0	0	0	0	0	0	0	0	0	0	0	0	0	0	0.03118298	0	0	0	0	0	0	0	0	0	0	0	0	0	0	0	0	0	0	0	0	0	0	0	0	0
ENSG00000221817	0.17025056	0.30482839	0.24655102	0.1565874	0.15158575	0.18665187	0.63366285	0.49387141	0.38524148	0.43887632	0.60220914	0.35667623	0.41290698	0.39370655	0.2818955	0.33794872	0.3447751	0.31989321	0.48282169	0.41280912	0.49333609	0.3355554	0.27196098	0.35309562	0.58446134	0.40416608	0.48171707	0.2715582	0.29388673	0.35818047	0.77601573	0.99341867	0.67139684	1.03057752	0.92458343	0.55335494	0.83015151	0.64993772	0.48478102	0.22614066	0.31804603	0.2872762	0.14994436	0.19007632	0.15704399	0.57560553	0.22157642	0.33058376
ENSG00000221818	0.00610701	0.01227171	0.00895882	0.015788	0.03917613	0.01374306	0.00245219	0.0089728	0.00452888	0.00553126	0.00384687	0	0.01809162	0.01984789	0.01327811	0.10898886	0.08727801	0.01841634	0.19490516	0.30637459	0.2776048	0.01825553	0.04949098	0.02554996	0.04663882	0.07790111	0.06042785	0.05065313	0.1052506	0.03193153	0	0.00373589	0	0.00961156	0	0.00271621	0	0.00615388	0.00615137	0	0.00570427	0	0.11187511	0.08863625	0.09576576	0.04613399	0.14954986	0.10848126
ENSG00000221819	0	0.0217183	0	0	0.03617391	0.02432226	0	0	0.01603029	0	0	0	0	0	0	0	0	0	0	0	0	0	0	0	0	0	0	0	0	0	0	0	0	0	0	0	0	0	0	0	0	0	0	0	0	0	0	0.03031398
ENSG00000221821	7.73513755	6.20164093	6.07478419	9.0896078	6.63567692	6.72541526	2.44710985	2.52554295	2.17283256	2.38837142	4.2080245	2.5733373	2.92635414	3.87247315	3.61843029	2.14812493	2.38018398	3.53426343	8.12571217	4.99506432	2.2197869	6.00583154	4.57080172	3.75917332	3.30046482	4.14740043	2.92437348	8.52778934	10.8567189	9.23906408	2.76004735	3.29797518	3.07629639	2.95127421	4.33681222	3.96161891	2.11485365	2.42101952	2.47905681	2.91796997	1.97046197	2.41346118	3.75722387	3.35199084	3.40539464	2.17313982	4.01799684	2.41056778
ENSG00000221823	13.0743284	10.6471704	11.9151259	11.543981	14.9136601	10.9424676	9.59677239	11.234178	11.4768908	10.5800511	9.55853853	11.3816345	8.66626172	7.26964429	7.68705951	9.27008603	8.83967213	6.08144539	6.90244378	5.97088542	13.595126	11.8247769	17.2483084	18.0917283	17.6650629	16.0087151	13.1902337	13.2439982	14.0043036	11.7855547	9.23935511	9.76724006	9.68619128	12.2973131	10.4937415	9.31542193	9.0233613	11.2935913	8.6537	15.356215	16.4061061	13.3457032	8.85696108	6.441408	6.49642764	10.731849	6.64293027	5.98278808
ENSG00000221826	0	0	0	0	0	0	0	0	0	0	0	0	0.00336121	0.01720835	0.00690737	0.00383086	0.01792209	0.00479015	0	0	0	0	0	0	0	0	0	0	0	0	0	0	0.00651477	0	0	0	0.00367482	0	0	0	0	0	0.00454674	0.02712306	0.00366309	0.01090874	0.00707244	0.0334141
ENSG00000221829	6.6269659	5.30788246	5.65287678	6.3768943	6.12137868	6.08414797	1.99027185	2.66723756	3.26095108	2.45576256	1.87432017	3.1853283	4.56358281	2.26823969	3.19591042	4.26441832	3.19650722	1.83209196	1.20126496	1.11062469	2.85793141	1.96074439	1.77467895	3.12753912	2.64766422	2.52549323	1.5006962	2.86164211	2.28456956	1.98108343	2.93917728	2.42857797	3.51770061	2.35375856	2.6932621	3.70420905	2.80383152	3.3623986	3.41971742	4.41767723	4.68054974	3.38752916	3.29118553	1.29340408	1.2075313	6.95200252	1.82639365	1.39093567
ENSG00000221836	0	0	0	0	0	0	0	0	0	0	0	0	0	0	0	0	0.04786301	0	0	0	0	0	0	0	0	0	0	0	0	0	0	0	0	0	0	0	0	0	0	0	0	0	0	0	0	0	0	0
ENSG00000221837	0	0	0	0	0	0	0	0	0	0	0	0	0	0	0	0	0	0	0	0	0	0	0	0	0	0	0	0	0	0	0	0	0	0	0	0	0	0	0	0	0	0	0	0	0	0	0	0
ENSG00000221838	1.22503871	1.31747396	1.15233815	1.3014562	1.04241712	1.14808044	0.78228192	0.85949555	0.84066488	1.05872647	0.86591478	0.76077173	0.70840613	0.66863164	0.76449407	0.57735153	0.48768933	0.4324042	0.50256434	0.54387057	0.70978888	0.50849703	0.4515448	0.61438736	0.7870674	0.62080619	0.55384476	0.79091056	0.72524885	0.72967262	1.77602575	1.65707622	1.65686208	1.40310047	1.32041192	1.32402331	0.71392743	0.78683798	0.81006533	0.79111453	0.81233078	0.74238173	0.61386185	0.53358738	0.55062868	0.83487541	0.60650371	0.64259659
ENSG00000221840	0	0	0	0	0	0	0	0	0	0	0	0	0	0	0	0	0	0	0	0	0	0	0	0	0	0	0	0	0	0	0	0	0	0	0	0	0	0	0	0	0	0	0	0	0	0	0	0
ENSG00000221843	0.01039669	0.00348193	0	0	0	0	0.01252398	0	0.00771005	0.00941652	0	0.00878	0.00879986	0	0.00452098	0	0.02346059	0	0.01144174	0.00461573	0.00875185	0.00887959	0.0157977	0.01933189	0.02481213	0.03850269	0.00894553	0.02743773	0.01493172	0.01672642	0.03159263	0.0254402	0.01705608	0.04090728	0.01012414	0.01849652	0.02405231	0	0.0052361	0	0	0.00930858	0.00595182	0.00887624	0.0047951	0	0	0
ENSG00000221844	0.01900338	0.05091507	0.09292484	0.0245641	0.02120098	0.02850982	0	0	0	0	0	0	0	0	0	0	0	0	0	0	0	0	0	0	0.01814095	0	0	0	0	0	0	0.02325018	0	0.02990862	0	0	0	0	0	0	0	0	0	0	0	0	0	0
ENSG00000221845	0	0.04449603	0.04331172	0	0.0185281	0.00830516	0.03556564	0.05422413	0.05473757	0	0.04184515	0.0187001	0.01874241	0.01199441	0.01925805	0	0.01665587	0	0.02436921	0.0294925	0.00932007	0.01891221	0.01121559	0.02058703	0.01056924	0.01822334	0.02857897	0.02504499	0.00795059	0.0178124	0.11775338	0.13545968	0.14530771	0.05227588	0.08625167	0.05909229	0.06147343	0.05578339	0.05576063	0.01778442	0.1034156	0.07930361	0.02535301	0.00945254	0	0	0	0
ENSG00000221849	0	0	0	0	0	0	0	0	0	0	0	0	0	0	0	0	0	0	0	0	0	0	0	0	0	0	0	0	0	0	0	0	0	0	0	0	0	0	0	0	0	0	0	0	0	0	0	0
ENSG00000221852	0	0	0	0	0	0	0.02199056	0.02682182	0.0270758	0	0	0.02312489	0.02317721	0.05933006	0.02381486	0.29057273	0.24716345	0.066061	0	0	0	0	0	0	0	0	0	0	0	0	0	0	0	0	0	0	0.05067945	0.02759311	0	0	0	0	0.56433603	0.21040534	0.20207059	0.15044236	0.09753603	0.12800381
ENSG00000221855	0	0.02336363	0	0	0	0	0	0	0	0	0	0	0	0	0.0303356	0	0	0	0	0	0	0	0	0	0	0	0	0	0	0	0	0	0	0.05489721	0	0	0	0	0	0	0	0	0	0	0	0	0	0
ENSG00000221857	0	0	0	0	0	0	0	0	0	0	0	0	0	0	0	0	0	0	0	0	0	0	0	0	0	0.02015507	0	0	0	0	0	0	0	0	0	0	0	0	0	0	0	0	0	0	0	0	0	0
ENSG00000221858	0	0	0	0	0	0	0	0	0	0	0	0	0	0	0	0	0	0	0	0	0	0	0	0	0	0	0	0	0	0	0	0	0	0	0	0	0	0	0	0	0	0	0	0	0	0	0	0
ENSG00000221859	0	0	0	0	0	0	0	0	0	0	0	0	0	0	0	0	0	0	0	0	0	0	0	0	0	0	0	0	0	0	0	0	0	0	0	0	0	0	0	0	0	0	0	0	0	0	0	0
ENSG00000221864	0	0	0	0	0	0	0	0	0	0	0	0	0	0	0	0	0	0	0	0	0	0	0	0	0	0	0	0	0	0	0	0	0	0	0	0	0	0	0	0	0	0	0	0	0	0	0	0
ENSG00000221866	0.01082129	0.01713228	0.01346931	0.0305188	0.04280318	0.01254495	1.13052986	1.04550016	1.53251835	2.34691525	0.70643229	1.1223828	0.05995144	0.02238052	0.03251155	0.04745022	0.05623706	0.01779969	0.86611021	0.79401008	1.29682929	0.77634749	1.33735423	1.55666478	2.8839996	1.85074262	1.58284626	1.0666706	1.13594476	1.0429887	0.01046273	0.01083239	0.02420816	0.07431784	0.02299114	0.0481298	1.22897137	1.40467731	1.3456411	0.81222159	1.29194242	1.15560285	0.03604303	0.00923875	0.0063521	0.04458925	0.00876014	0.00735782
ENSG00000221867	0.00691774	0	0	0	0	0	0	0	0	0	0	0	0.07611815	0.06744831	0.15642465	0.02002014	0.07284752	0.01668895	0	0	0	0	0	0	0	0	0	0.01564842	0	0.0111294	0	0.00846369	0.03404626	0	0.02694555	0.01846079	0	0	0	0	0	0	0.06336348	0.02362425	0.02552448	0.01900307	0.03696069	0.01293501
ENSG00000221869	1.46498817	0.88215388	1.99714174	1.3772133	2.05538582	1.74273816	7.59374123	6.18920362	4.25787391	2.94860669	7.06557366	3.49909889	17.7354796	27.1727189	17.8244628	9.70710363	14.0691758	37.3596634	10.7971524	3.53449153	1.38269554	5.71261754	7.19528016	2.65524979	2.59923612	2.77662467	7.28292247	1.94148132	2.77347469	2.51165033	4.49665584	1.68374886	5.94770056	6.47454411	7.29142406	10.4253129	2.49225178	3.16122282	2.90654208	3.77938437	2.51560099	4.6636931	10.6061604	24.3705349	25.0886458	3.71945929	18.5798752	22.3292952
ENSG00000221870	0.91104139	1.83953381	1.21380173	0.9728226	0.99430099	0.90128881	0	0	0	0	0	0	0	0	0	0	0	0	0	0	0	0	0.02675017	0	0	0	0	0.00995575	0.06637	0.06372621	0	0	0	0	0	0	0	0	0	0	0	0	0	0	0	0	0	0
ENSG00000221874	0	0	0	0	0	0	0	0	0	0	0	0	0	0	0	0	0	0	0	0	0	0	0	0	0	0	0	0	0	0	0	0	0	0	0	0	0	0	0	0	0	0	0	0	0	0	0	0

ENSG00000221878	0.01623869	0	[illegible]
ENSG00000221879	0.24602855	0.16479382	[illegible]
ENSG00000221880	0	0	[illegible]
ENSG00000221882	0	0	[illegible]
ENSG00000221883	1.35130949	1.5535788	[illegible]
ENSG00000221886	3.35285953	2.89728233	[illegible]
ENSG00000221887	0.01560007	0.01044917	[illegible]
ENSG00000221888	0	0	[illegible]
ENSG00000221890	1.82211703	1.64223995	[illegible]
ENSG00000221891	0	0	[illegible]
ENSG00000221900	0	0	[illegible]
ENSG00000221909	5.91692261	5.7594006	[illegible]
ENSG00000221910	0	0	[illegible]
ENSG00000221914	1.33341011	1.48192847	[illegible]
ENSG00000221916	0.73742339	1.59803429	[illegible]
ENSG00000221923	5.28716701	5.87648086	[illegible]
ENSG00000221926	0.27843695	0.22380178	[illegible]
ENSG00000221930	0.03628454	0.02430396	[illegible]
ENSG00000221931	0	0	[illegible]
ENSG00000221932	0	0	[illegible]
ENSG00000221933	0	0	[illegible]
ENSG00000221937	0	0	[illegible]
ENSG00000221938	0	0	[illegible]
ENSG00000221944	8.55773319	10.4412503	[illegible]
ENSG00000221946	0	0	[illegible]
ENSG00000221947	0.08935439	0.17174631	[illegible]
ENSG00000221949	1.16746682	0.89736303	[illegible]
ENSG00000221953	0.58521671	0.63100415	[illegible]
ENSG00000221954	0	0	[illegible]
ENSG00000221955	0.22054601	0.14772521	[illegible]
ENSG00000221957	0	0	[illegible]
ENSG00000221961	0	0	[illegible]
ENSG00000221962	0	0	[illegible]
ENSG00000221963	2.78957139	2.77715282	[illegible]
ENSG00000221968	0.5547453	0.45426923	[illegible]
ENSG00000221970	0	0	[illegible]
ENSG00000221971	0	0	[illegible]
ENSG00000221972	0	0	[illegible]
ENSG00000221977	0	0	[illegible]
ENSG00000221978	1.50100566	3.20256792	[illegible]
ENSG00000221983	21.8429998	21.0415325	[illegible]
ENSG00000221986	0.04785411	0.03205344	[illegible]
ENSG00000221988	0.4606696	0.57122555	[illegible]
ENSG00000221989	0	0	[illegible]
ENSG00000221990	3.47458161	2.78209544	[illegible]
ENSG00000221994	0.70660552	0.76502518	[illegible]
ENSG00000221995	0.05638795	0.07014343	[illegible]
ENSG00000221996	0	0	[illegible]
ENSG00000222000	0.17194289	0.4606803	[illegible]
ENSG00000222001	0.02738296	0	[illegible]
ENSG00000222004	0	0	[illegible]
ENSG00000222005	0.01871102	0.02506588	[illegible]
ENSG00000222007	0	0	[illegible]
ENSG00000222009	0.14565139	0.20877757	[illegible]
ENSG00000222011	0.53174931	0.74007437	[illegible]
ENSG00000222012	0	0	[illegible]
ENSG00000222014	0.07332082	0.09120698	[illegible]
ENSG00000222017	0	0	[illegible]
ENSG00000222018	0	0	[illegible]
ENSG00000222019	0.07945476	0.0387055	[illegible]
ENSG00000222020	0.30866471	0.43072607	[illegible]
ENSG00000222022	0	0	[illegible]
ENSG00000222024	0	0	[illegible]
ENSG00000222028	0	0	[illegible]
ENSG00000222030	0	0	[illegible]
ENSG00000222031	0	0	[illegible]
ENSG00000222032	0	0	[illegible]
ENSG00000222033	0.04567275	0.091777	[illegible]
ENSG00000222035	0.01014471	0.01359017	[illegible]
ENSG00000222036	0	0.00194311	[illegible]
ENSG00000222037	0	0	[illegible]
ENSG00000222038	0	0	[illegible]
ENSG00000222040	0	0	[illegible]
ENSG00000222041	1.5587332	0.49149541	[illegible]
ENSG00000222042	0.04224081	0	[illegible]
ENSG00000222043	0.9042412	0.60567509	[illegible]
ENSG00000222044	0	0	[illegible]
ENSG00000222046	0	0	[illegible]
ENSG00000222047	0	0	[illegible]
ENSG00000222051	0	0	[illegible]
ENSG00000222054	0	0	[illegible]
ENSG00000222055	0	0	[illegible]
ENSG00000222057	0	0	[illegible]
ENSG00000222064	0	0	[illegible]
ENSG00000222066	0	0	[illegible]
ENSG00000222067	0	0.0985753	[illegible]
ENSG00000222068	0	0.96773751	[illegible]
ENSG00000222069	0	0	[illegible]
ENSG00000222070	0	0	[illegible]
ENSG00000222071	0	0	[illegible]
ENSG00000222072	0	0	[illegible]
ENSG00000222076	0.50622628	0.22605226	[illegible]
ENSG00000222078	0	0.22724201	[illegible]
ENSG00000222079	0	0	[illegible]
ENSG00000222080	0	0	[illegible]
ENSG00000222084	0	0	[illegible]
ENSG00000222085	0	0.87756059	[illegible]
ENSG00000222086	0	0	[illegible]
ENSG00000222087	0	0	[illegible]
ENSG00000222092	0	0	[illegible]
ENSG00000222094	0.17421481	0.11669184	[illegible]
ENSG00000222095	0	0.2917296	[illegible]
ENSG00000222096	0	0	[illegible]
ENSG00000222099	0	0	[illegible]
ENSG00000222100	0	0	[illegible]
ENSG00000222101	0	0	[illegible]
ENSG00000222102	0	0	[illegible]
ENSG00000222107	0	0	[illegible]
ENSG00000222108	0	0	[illegible]
ENSG00000222109	0	0	[illegible]
ENSG00000222111	0.11719905	0	[illegible]
ENSG00000222112	0.64675063	0.5776051	[illegible]
ENSG00000222114	0	0	[illegible]
ENSG00000222117	0	0	[illegible]
ENSG00000222118	0	0	[illegible]
ENSG00000222120	0	0	[illegible]
ENSG00000222121	0	0	[illegible]
ENSG00000222122	0	0	[illegible]
ENSG00000222123	0	0	[illegible]
ENSG00000222126	0	0	[illegible]
ENSG00000222129	0	0	[illegible]
ENSG00000222133	0	0	[illegible]
ENSG00000222139	0	0	[illegible]
ENSG00000222145	0	0.34266652	[illegible]
ENSG00000222146	0	0	[illegible]
ENSG00000222148	0	0	[illegible]
ENSG00000222150	0	0	[illegible]
ENSG00000222152	0	0	[illegible]
ENSG00000222154	0	0	[illegible]
ENSG00000222158	0	0	[illegible]
ENSG00000222160	0	0	[illegible]
ENSG00000222161	0	0	[illegible]
ENSG00000222162	0.10199285	0	[illegible]
ENSG00000222164	0	0	[illegible]
ENSG00000222168	0	0	[illegible]
ENSG00000222169	0	0	[illegible]
ENSG00000222170	0	0	[illegible]
ENSG00000222174	0	0.0646347	[illegible]
ENSG00000222177	0	0	[illegible]
ENSG00000222178	0	0	[illegible]
ENSG00000222179	0	0	[illegible]
ENSG00000222182	0	0	[illegible]
ENSG00000222185	0	0.28783987	[illegible]
ENSG00000222190	0	0	[illegible]
ENSG00000222192	0	0	[illegible]
ENSG00000222196	0	0	[illegible]
ENSG00000222197	0	0	[illegible]
ENSG00000222202	0	0	[illegible]
ENSG00000222203	0	0	[illegible]
ENSG00000222205	0	0	[illegible]
ENSG00000222206	0	0	[illegible]
ENSG00000222207	0	0	[illegible]
ENSG00000222208	0	0.36589815	[illegible]
ENSG00000222209	0	0.18294907	[illegible]
ENSG00000222210	0.10297041	0.06897122	[illegible]
ENSG00000222213	0	0	[illegible]
ENSG00000222219	0	0	[illegible]
ENSG00000222220	0	0	[illegible]
ENSG00000222222	0.16963021	0.34086301	[illegible]
ENSG00000222224	0	0	[illegible]
ENSG00000222225	0	0	[illegible]
ENSG00000222226	0	0	[illegible]
ENSG00000222230	0	0	[illegible]
ENSG00000222231	0	0	[illegible]
ENSG00000222232	0	0	[illegible]
ENSG00000222235	0	0	[illegible]
ENSG00000222236	0	0	[illegible]
ENSG00000222238	0	0	[illegible]
ENSG00000222240	0	0	[illegible]
ENSG00000222244	0	0	[illegible]
ENSG00000222246	0	0	[illegible]
ENSG00000222248	0	0	[illegible]
ENSG00000222249	0	0	[illegible]
ENSG00000222251	0	0	[illegible]
ENSG00000222255	0	0	[illegible]
ENSG00000222257	0	0	[illegible]
ENSG00000222259	0.23103756	0.07737631	[illegible]
ENSG00000222262	0	0	[illegible]
ENSG00000222266	0	0	[illegible]
ENSG00000222267	0.97665878	0	[illegible]
ENSG00000222268	0	0	[illegible]

ENSG00000222274	0	0	0	0	0	0	0	0	0	0	0	0	0	0	0	0	0	0	0	0	0	0	0	0	0	0	0	0	0	0	0	0	0	0	0	0	0	0	0	0	0	0	0	0	0	0	0	0
ENSG00000222276	0	0.2248749	0	0	0	0.12591838	0.13480673	0.16442337	0.2489704	0.15203753	0	0	0	0.18185282	0.14599007	0	0.25252724	0	0.18473635	0.29809954	0	0.14336837	0	0	0.16024503	0	0	0.12657287	0.12054254	0.40509289	0.2550447	0	0	0	0.32692526	0	0.31067562	0.33830303	0.84541248	0.26963768	0.94075875	0.30058959	0	0.14331428	0.15484185	0	0.44843715	0
ENSG00000222281	0	0	0	0	0	0	0	0	0	0	0	0	0	0	0	0	0	0	0	0	0	0	0	0	0	0	0	0	0	0	0	0	0	0	0	0	0	0	0	0	0	0	0	0.10153632	0	0	0	0
ENSG00000222282	0	0	0	0	0	0	0	0	0	0	0	0	0	0	0	0	0	0	0	0	0	0	0	0	0	0	0	0	0	0	0	0	0	0	0	0	0	0	0	0	0	0	0	0	0	0	0	0
ENSG00000222285	0	0	0	0	0	0	0	0	0	0	0	0	0	0	0	0	0	0	0	0	0	0	0	0.56008271	0	0	0	0	0	0	0	0	0	0	0	0	0	0	0	0	0.56269682	0	0	0	0	0	0	0
ENSG00000222287	0	0	0	0	0	0	0	0	0	0	0	0	0	0	0	0	0	0	0	0	0	0	0	0	0	0	0	0	0	0	0	0	0	0	0	0	0	0	0	0	0	0	0	0	0	0	0	0
ENSG00000222293	0	0	0	0	0	0	0	0	0	0	0	0	0	0.36560986	0	0	0	0	0	0	0	0	0	0	0	0	0	0	0	0	0	0	0	0	0	0	0	0.17003713	0	0	0	0	0	0	0	0.23176787	0	0
ENSG00000222296	0	0	0	0	0.19541777	0	0.56267158	0	0	0.31729572	0.44134506	0	0	0	0	0	0	0	0	0	0	0	0.70975182	0	0	0.28830514	0	0	0	0	0.5322672	0	0	0	0	0	0	0	0.35286782	0	0	0	0.40110115	0	0	0	0	0.65504559
ENSG00000222297	0	0	0.31520107	0.416607	0	0	0.77648678	0	0.47802316	1.16764825	0	0.81653972	1.0911829	0	0.28030094	1.24365128	0.48485229	0.77753795	0.35469379	0.28617556	0.81392186	0	0.97945751	0.5992885	0.92301135	0.53048145	0	1.21509952	0	0	0	0.39432312	0	0	0.62769651	0	2.08774014	0.32477091	0.64927678	0.51770435	0.6020856	0	0.36901306	0.27516343	0	0.44267663	0	0.30132097
ENSG00000222298	0	0	0	0	0	0	0	0	0	0	0	0	0	0	0	0	0	0	0	0	0	0	0	0	0	0	0	0	0	0	0	0	0	0	0	0	0	0	0	0	0	0	0	0	0	0	0	0
ENSG00000222299	0	0	0	0	0	0	0	0	0	0	0	0	0	0	0	0	0	0	0	0	0	0	0	0	0	0	0	0	0	0	0	0	0	0	0	0	0	0	0	0	0	0	0	0	0	0	0	0
ENSG00000222300	0	0	0	0	0	0	0	0	0	0	0	0	0	0	0	0	0	0	0.18866691	0	0	0	0	0	0	0	0	0	0	0	0	0	0	0	0	0	0	0	0	0	0	0	0	0	0	0	0	0
ENSG00000222301	0.41856805	1.12145406	1.2280561	0.5410481	0.23348617	0.94193491	1.34456585	0.40999074	2.06936435	4.5492789	3.69124956	1.06044119	2.12568097	1.36035357	3.64027192	2.82648017	4.40774812	2.52447388	0.92128256	2.22993943	0.35234713	1.42995989	1.27202274	0	0	0.68893695	0.72028904	0.63122053	0.60114722	1.34680234	0.63595561	0	1.3733466	0.65876656	0.81519027	0.74466521	1.1620075	0.4217804	2.95125811	0	0	3.74761053	0.95847548	1.7867755	0.38609917	2.29961886	2.98181583	1.56530376
ENSG00000222302	0	0	0	0	0	0	0	0	0	0	0	0	0	0	0	0	0	0	0	0	0	0	0	0	0	0	0	0	0	0	0	0	0	0	0	0	0	0	0	0	0	0	0	0	0	0	0	0
ENSG00000222303	0	0	0	0	0	0	0	0	0	0	0	0	0	0	0	0	0	0	0	0	0	0	0	0	0	0	0	0	0	0	0	0	0	0	0	0	0	0	0	0	0	0	0	0	0	0	0	0
ENSG00000222305	0	0	0	0	0	0	0	0	0	0	0	0	0	0	0	0	0	0	0	0	0	0	0	0	0	0	0	0	0	0	0	0	0	0	0	0	0	0	0	0	0	0	0	0	0	0	0	0
ENSG00000222306	0	0	0	0	0	0	0	0	0.38863672	0	0	0	0	0	0.34183041	0	0	0	0	0	0	0	0	0	0	0	0	0	0	0	0	0	0	0	0	0	0	0	0	0	0	0	0	0	0	0	0	0
ENSG00000222308	0	0	0	0	0	0	0	0	0	0	0	0	0	0	0	0	0	0	0	0	0	0	0	0	0	0	0	0	0	0	0	0	0	0	0	0	0	0	0	0	0	0	0	0	0	0	0	0
ENSG00000222309	0	0	0	0	0	0	0	0	0	0	0	0	0	0	0	0	0	0	0	0	0	0	0	0	0	0	0	0	0	0	0	0	0	0	0	0	0	0	0	0	0	0	0	0	0	0	0	0
ENSG00000222311	0	0.28405251	0	0.5481671	0	0.3181096	0	0	0	0	0	0	0	0	0	0	0	0	0	0	0	0	0	0	0	0	0	0	0	0	0	0	0	0	0	0	0	0	0	0	0	0	0	0	0	0	0	0
ENSG00000222312	0	0	0	0	0	0	0	0	0	0	0	0	0	0	0	0	0	0	0	0	0	0	0	0	0	0	0	0	0	0	0	0	0	0	0	0	0	0	0	0	0	0	0	0	0	0	0	0
ENSG00000222313	0	0	0	0	0	0	0	0	0	0	0	0	0	0	0	0	0	0	0	0	0	0	0	0	0	0	0	0	0	0	0	0	0	0	0	0	0	0	0	0	0	0	0	0	0	0	0	0
ENSG00000222314	0	0	0	0	0	0	0	0	0	0	0	0	0	0	0	0	0	0	0	0	0	0	0	0	0	0	0	0	0	0	0	0	0	0	0	0	0	0	0	0	0	0	0	0	0	0	0	0
ENSG00000222316	0.29299763	0	0	0	0	0	0	0	0.1448555	0	0	0.24743628	0	0	0	0	0	0	0	0	0	0	0	0	0	0	0	0.22092718	0	0	0	0	0	0	0	0	0	0.29524628	0	0	0	0	0	0	0	0	0	0
ENSG00000222317	0	0	0	0	0	0	0	0	0	0	0	0	0	0	0	0	0	0	0	0	0	0	0	0	0	0	0	0	0	0	0	0	0	0	0	0	0	0	0	0	0	0	0	0	0	0	0	0
ENSG00000222320	0	0	0	0	0	0	0	0	0	0	0	0	0	0	0	0	0	0	0	0	0	0	0	0	0	0	0	0	0	0	0	0	0	0	0	0	0	0	0	0	0	0	0	0	0	0	0	0
ENSG00000222321	0	0	0	0	0	0	0	0	0	0	0	0	0	0	0	0	0	0	0	0	0	0	0	0	0	0	0	0	0	0	0	0	0	0	0	0	0	0	0	0	0	0	0	0	0	0	0	0
ENSG00000222326	0	0	0	0	0	0	0	0	0	0	0	0	0	0	0	0	0	0	0	0	0	0	0	0	0	0	0	0	0	0	0	0	0	0	0	0	0	0	0	0	0	0	0	0	0	0	0	0
ENSG00000222327	0	0	0	0	0	0	0	0	0	0	0	0	0	0	0	0	0	0	0	0	0	0	0	0	0	0	0	0	0	0	0	0	0	0	0	0	0	0	0	0	0	0	0	0	0	0	0	0
ENSG00000222328	0	0	0	0	0	0	0	0	0	0	0	0	0	0	0	0	0	0	0	0	0	0	0	0	0	0	0	0	0	0	0	0	0	0	0	0	0	0	0	0	0	0	0	0	0	0	0	0
ENSG00000222329	0	0	0	0	0	0	0	0	0	0	0	0	0	0	0	0	0	0	0	0	0	0	0	0	0	0	0	0	0	0	0	0	0	0	0	0	0	0	0	0	0	0	0	0	0	0	0	0
ENSG00000222331	0	0	0	0	0	0	0	0	0	0	0	0	0	0	0	0	0	0	0	0	0	0	0	0	0	0	0	0	0	0	0	0	0	0	0	0	0	0	0	0	0	0	0	0	0	0	0	0
ENSG00000222333	0	0	0	0	0	0	0	0	0	0	0	0	0	0	0	0	0	0	0	0	0	0	0	0	0	0	0	0	0	0	0	0	0	0	0	0	0	0	0	0	0	0	0	0	0	0	0	0
ENSG00000222334	0	0	0	0	0	0	0	0	0	0	0	0	0	0	0	0	0	0	0	0	0	0	0	0	0	0	0	0	0	0	0	0	0	0	0	0	0	0	0	0	0	0	0	0	0	0	0	0
ENSG00000222335	0	0	0	0	0	0	0	0	0	0	0	0	0	0	0	0	0	0	0	0	0	0	0	0	0	0	0	0	0	0	0	0	0	0	0	0	0	0	0	0	0	0	0	0	0	0	0	0
ENSG00000222337	0	0	0	0	0	0.06868275	0	0	0	0	0	0	0	0	0	0	0	0	0	0	0	0	0.18550332	0	0	0	0	0	0	0	0	0	0	0	0	0	0	0	0	0	0	0	0.10483326	0	0	0	0	0
ENSG00000222338	0	0	0	0	0	0	0	0	0	0	0	0	0	0	0	0	0	0	0	0	0	0	0	0	0	0	0	0	0	0	0	0	0	0	0	0	0	0	0	0	0	0	0	0	0	0	0	0
ENSG00000222339	0	0	0	0	0	0	0	0	0	0	0	0	0	0	0	0	0	0	0	0	0	0	0	0	0	0	0	0	0	0	0	0	0	0	0	0	0	0	0	0	0	0	0	0	0	0	0	0
ENSG00000222343	0	0.29471659	0.10757716	0	0.06135985	0.24753921	0	0	0	0	0	0	0	0	0	0	0	0	0	0	0	0	0	0	0	0	0	0.08294195	0	0	0	0	0	0	0	0	0	0	0.11079809	0	0	0	0	0	0	0	0	0
ENSG00000222344	0	0	0	0	0	0	0	0	0	0	0	0	0	0	0	0	0	0	0	0	0	0	0	0	0	0	0	0	0	0	0	0	0	0	0	0	0	0	0	0	0	0	0	0	0	0	0	0
ENSG00000222345	0.84815105	1.13621003	0.41473825	2.7408357	1.18279178	2.86298637	7.83298067	5.40000959	9.01534914	8.45008602	6.41111766	3.93944602	3.58941743	1.37825295	4.7946213	3.68186233	5.74167189	5.11538128	1.8668094	1.88273395	1.42793309	5.07071303	3.86627965	5.12549376	1.21448861	4.88601336	5.47324892	2.55810425	1.21811411	2.387916	0	1.03769242	3.47854238	0	0.82591645	1.88615859	2.7470265	2.56398087	5.55302512	6.81189934	9.50661474	3.03753695	4.85543498	3.25851427	4.69415301	4.07728475	3.02105025	7.13654936
ENSG00000222346	0	0.38208833	0.55787799	0.3686788	0	0.42789964	0	0	0.14100978	0	0.71865036	0	0.24141215	0	0	0	0	0	0.62777661	0	0	0	0	1.06068761	0	0.23472631	0	0.21506186	0.40963129	0.22943314	0	0	0	0	0	0	0	0.28740789	0	0.91629088	0	0	0.32656023	0.24350746	0	0	0	0
ENSG00000222348	0.29842352	0	0	0	0	0	0.47931283	0.29230821	0.14753801	0.54057789	0.3759606	0.25201843	0	0.64658781	0	0.28788224	0.44893731	0.35997128	0	0	0	0	0.3023017	1.66469028	0.56976009	0	0.5135394	0.22501843	0.42859571	0.4801101	0	0.365114	0	0	0	0.53091871	0.2761561	0	0.3005911	0	0	0.5343815	0	0.25478095	0.2752744	0	0	0.5580018
ENSG00000222351	0	0	0	0	0	0	0	0	0	0	0	0	0	0	0	0	0	0	0	0	0	0	0	0	0	0	0	0	0	0	0	0	0	0	0	0	0	0	0	0	0	0	0	0	0	0	0	0
ENSG00000222352	0	0	0	0	0	0	0	0	0	0	0	0	0	0	0	0	0	0	0	0	0	0	0	0	0	0	0	0	0	0	0	0	0	0	0	0	0	0	0	0	0	0	0	0	0	0	0	0
ENSG00000222355	0	0	0	0	0	0	0	0.16703326	0.16861487	0	0	0	0	0	0	0	0	0	0	0	0	0	0.17274383	0.15854193	0	0.28067802	0	0.12858196	0.36736775	0	0	0	0	0	0	0.15169106	0.31560698	0.34367292	0.17176634	0	0	0	0.39049001	0	0.31459932	0	0	0
ENSG00000222356	0	0	0	0	0	0	0	0	0	0	0	0	0	0	0	0	0	0	0	0	0	0	0	0	0	0	0	0	0	0	0	0	0	0	0	0	0	0	0	0	0	0	0	0	0	0	0	0.57394471
ENSG00000222357	0	0.22605226	0.49508021	0.2181189	0.09412793	0.37973292	0	0	0.08342464	0	0.21258505	0	0	0	0	0	0	0	0	0	0	0	0	0	0.161084	0	0	0	0.12117365	0	0.25638001	0.20645189	0	0	0	0	0	0	0.16996774	0	0	0	0	0	0	0	0	0
ENSG00000222359	0	0	0	0	0	0	0	0	0	0	0	0	0	0	0	0	0	0	0	0	0	0	0	0	0	0	0	0	0	0	0	0	0	0	0	0	0	0	0	0	0	0	0	0	0	0	0	0
ENSG00000222361	0	0	0	0	0	0	0	0	0	0	0	0	0	0	0	0	0	0	0	0	0	0	0	0	0	0	0	0	0	0	0	0	0	0	0	0	0	0	0	0	0	0	0	0	0	0	0	0
ENSG00000222363	0	0	0	0	0	0.1949704	0	0	0	0	0	0	0	0	0	0	0	0	0	0	0	0	0	0	0	0	0	0	0	0	0	0	0	0	0	0	0	0	0	0	0	0	0	0	0	0	0	0
ENSG00000222364	0	0.2248749	0	0	0.18727536	0	0	0	0	0	0	0	0	0	0	0	0	0	0	0	0	0	0	0	0	0	0	0	0	0	0	0	0	0	0	0	0	0	0	0.53927536	0	0	0	0	0	0	0	0
ENSG00000222365	3.54172965	5.69353597	2.07824879	5.0359091	3.35860874	6.11050083	38.6821253	34.3446088	41.1485141	34.0029435	35.6956002	46.3603138	13.1901229	12.2780629	14.7851044	9.56654828	12.7873132	5.55384253	10.9136549	13.2081028	27.7270085	30.8541346	10.4044937	25.6837929	21.6383612	28.2729125	33.5211436	14.955071	14.242565	17.3789303	5.38116287	4.33322109	4.64825003	3.34450717	1.37955276	8.50636796	33.1023162	28.5512888	37.1015305	28.4452939	32.4199939	26.0026515	7.70466825	7.55943481	7.18738446	19.4583134	7.88460918	11.9203901
ENSG00000222370	0.4920571	0.32958764	0.962446	1.2720825	0.96067973	0.36910427	0.98789667	0.72296077	0.24326879	1.11416818	0.30995225	0.83108368	0.62472303	0.53306476	0.42794036	0.23733803	0.37011625	0	0.81227584	0.43690925	0.4142096	0.84051078	0.49845166	0.45747214	0.46972588	0.20247384	0	0.37102275	0.53001912	0.79163191	1.12141791	0.60202003	0.80723426	0.77442787	0.47915764	0.21885199	0.45534136	0.24791673	0.24781557	0.79038832	1.37882199	0.88111759	0.28168936	0.21004842	0	0	0.65725139	0.46003202
ENSG00000222371	0	0	0	0	0	0	0	0	0	0	0	0	0	0	0	0	0	0	0	0	0	0	0	0	0	0	0	0	0	0	0	0	0	0	0	0	0	0	0	0	0	0	0	0	0	0	0.08696963	0
ENSG00000222373	0	0	0	0	0	0	0	0	0	0	0	0	0	0	0	0	0	0	0	0	0	0	0	0	0	0	0	0	0	0	0	0	0	0	0	0	0	0	0	0	0	0	0	0	0	0	0	0
ENSG00000222374	0	0	0	0	0	0	0	0	0	0	0	0	0	0	0	0	0	0	0	0	0	0	0	0	0	0	0	0	0	0	0	0	0	0	0	0	0	0	0	0	0	0	0	0	0	0	0	0
ENSG00000222375	0	0	0	0	0	0	0	0	0	0	0	0	0	0	0	0	0	0	0	0	0	0	0	0	0	0	0	0.06864969	0	0	0	0	0	0	0	0	0	0	0	0	0	0	0	0	0	0	0	0
ENSG00000222376	0	0	0	0	0	0	0	0	0	0	0	0	0	0	0	0	0	0	0	0	0	0	0	0	0	0	0	0	0	0	0	0	0	0	0	0	0	0	0	0	0	0	0	0	0	0	0	0
ENSG00000222378	0	0	0	0	0	0	0	0	0	0	0	0	0	0	0	0	0	0	0	0	0	0	0	0	0	0	0	0	0	0	0	0	0	0	0	0	0	0	0	0	0	0	0	0	0	0	0	0
ENSG00000222383	0	0	0	0	0	0	0	0	0	0	0	0	0	0	0	0	0	0	0	0	0	0	0	0	0	0	0	0	0	0	0	0	0	0	0	0	0	0	0	0	0	0	0	0	0	0	0	0
ENSG00000222385	0	0	0	0	0	0	0	0	0	0	0	0	0	0	0	0	0	0	0	0	0	0	0	0	0	0	0	0	0	0	0	0	0	0	0	0	0	0	0	0	0	0	0	0	0	0	0	0
ENSG00000222386	1.06785282	1.30048136	1.51904128	1.7567766	1.24549399	2.76717023	1.09144728	1.61649963	0.7679087	0.79132788	2.44600875	0.4918914	0.16433477	0.52583948	1.51970388	0.65553908	0.73019924	1.1709909	0.53417739	1.12056695	0.89890969	0.49747099	0.98339108	0.81228863	2.78015466	2.47664533	2.33876982	3.29394447	1.46393834	1.71798432	2.21243594	0.83140417	2.3888785	0.45835866	0.75626085	1.55437647	1.43734261	2.05427383	3.12904474	1.55935045	2.72026627	2.2598543	2.00067321	1.98913321	3.67143092	3.20007202	2.67981717	2.54126122
ENSG00000222389	0	0	0	0	0	0	0	0	0	0	0	0	0	0	0	0	0	0	0	0	0	0	0	0	0	0	0	0	0	0	0	0	0	0	0	0	0	0	0	0	0	0	0	0	0	0	0	0
ENSG00000222391	0	0	0	0	0	0	0	0	0	0	0	0	0	0	0	0	0	0	0	0	0	0	0	0	0	0	0	0	0	0	0	0	0	0	0	0	0	0	0	0	0	0	0	0	0	0	0	0
ENSG00000222394	0	0	0	0	0	0	0	0	0	0	0	0	0	0	0	0	0	0	0	0	0	0	0	0	0	0	0	0	0	0	0	0	0	0	0	0	0	0	0	0	0	0	0	0	0	0	0	0
ENSG00000222395	0.30990134	0.20757683	0	0	0.34573913	0.23246471	0	0	0	0	0	0	0	0.33572828	0.53904027	0	0	0	0	0	0	0	0	0	0.29583697	0	0	0	0.44508016	0	0	0	0	0	0	0	0	0	0	0	0	0	0	0	0	0	0	0
ENSG00000222397	0	0	0	0	0	0	0	0	0	0	0	0	0	0	0	0	0	0	0	0	0.08983685	0	0	0	0	0	0	0	0	0	0	0	0	0	0	0	0	0	0	0	0	0	0	0	0	0	0	0
ENSG00000222398	0	0	0	0	0	0	0	0	0	0	0	0	0	0	0	0	0	0	0	0	0	0	0	0	0	0	0	0	0	0	0	0	0	0	0	0	0	0	0	0	0	0	0	0	0	0	0	0
ENSG00000222399	0	0	0	0	0	0	0	0	0	0	0	0	0	0	0	0	0	0	0	0	0	0	0	0	0	0	0	0	0	0	0	0	0	0	0	0	0	0	0	0	0	0	0	0	0	0	0	0
ENSG00000222404	0	0	0	0	0.1021502	0	0	0	0	0	0.11535155	0	0	0	0	0	0	0	0	0	0	0	0	0.08512621	0	0	0.07878161	0	0	0	0	0	0	0	0	0	0	0	0	0	0	0	0	0.07817143	0	0.12576041	0	0
ENSG00000222405	0	0	0	0	0	0	0	0	0	0	0	0	0	0	0	0	0	0	0	0	0	0	0	0	0	0	0	0	0	0	0	0	0	0	0	0	0	0	0	0	0	0	0	0	0	0	0	0
ENSG00000222406	0	0	0	0	0	0	0	0	0	0	0	0	0	0	0	0	0	0	0	0	0	0	0	0	0	0	0	0	0	0	0	0	0	0	0	0	0	0	0	0	0	0	0	0	0	0	0	0
ENSG00000222407	0	0	0	0	0	0	0	0	0	0	0	0	0	0	0	0	0	0	0	0	0	0	0	0	0	0	0	0	0	0	0	0	0	0	0	0	0	0	0	0	0	0	0	0	0	0	0	0
ENSG00000222408	0	0	0	0	0	0	0	0	0	0	0	0	0	0	0	0	0	0	0	0	0	0	0	0	0	0	0	0	0	0	0	0	0	0	0	0	0	0	0	0	0	0	0	0	0	0	0	0
ENSG00000222409	0	0	0	0	0	0	0	0	0	0	0	0	0	0	0	0	0	0	0	0	0	0	0	0	0	0	0	0	0	0	0	0	0	0	0	0	0	0	0	0	0	0	0	0	0	0	0	0
ENSG00000222411	0	0	0	0	0	0	0	0	0	0	0	0	0	0	0	0	0	0	0	0	0	0	0	0	0	0	0	0	0	0	0	0	0	0	0	0	0	0	0	0	0	0	0	0	0	0	0	0
ENSG00000222412	0	0	0	0.4385337	0	0	0	0	0	0.30727586	0	0	0	0	0	0	0	0	0	0	0	0.28975503	0.3436693	0	0	0.27920076	0	0	0	0	0	0	0	0	0	0	0	0	0	0	0	0	0	0	0	0	0	0
ENSG00000222413	0	0	0	0	0	0	0	0	0	0	0	0	0	0	0	0	0	0	0	0	0	0	0	0	0	0	0	0	0	0	0	0	0	0	0	0	0	0	0	0	0	0	0	0	0	0	0	0
ENSG00000222414	39.1481656	14.8064228	17.9879143	58.892092	46.0285587	17.5942921	208.418266	265.777033	187.038034	182.483247	247.236417	194.516007	58.9867195	284.992885	311.706383	59.7408401	203.079495	177.083251	209.845014	94.8424761	187.074187	264.890711	324.776487	267.954125	240.015167	377.863882	267.438416	879.324896	475.121901	344.367135	533.014043	167.638939	152.254525	278.854852	159.71754	314.014792	332.289531	627.947106	331.267134	600.645466	628.879986	349.60301	373.07027	149.106883	246.553628	452.410881	282.942709	132.991403
ENSG00000222416	0	0	0	0	0	0	0	0	0	0	0	0	0	0	0	0	0	0	0	0	0	0	0	0	0	0	0	0	0	0	0	0	0	0	0	0	0	0	0	0	0	0	0	0	0	0	0	0
ENSG00000222418	0	0	0	0	0	0	0	0	0	0	0	0	0	0	0	0	0	0	0	0	0	0	0	0	0	0	0	0	0	0	0	0	0	0	0	0	0	0	0	0	0	0	0	0	0	0	0	0
ENSG00000222419	0	0	0	0	0	0	0	0	0	0	0	0	0	0	0	0	0	0	0	0	0	0	0	0	0	0	0	0	0	0	0	0	0	0	0	0	0	0	0	0	0	0	0	0	0	0	0	0
ENSG00000222421	0	0.19805496	0	0	0	0	0	0.86887945	0.14618445	0.53561846	0.37251142	0.24970634	0.25027131	0.64065581	0.25715682	0.57048224	0	0.35666879	0	0	0	0.50507758	0	0	0.28226647	0	0.25441402	0	0	0	0	0	0	0	0	0	0.27362256	0	0.29783339	0	0.55237211	0	0	0	0	0	0	0
ENSG00000222426	0	0.30192994	0	0	0	0	0	0	0	0	0.28394227	0	0.19076624	0	0	0.21742155	0	0	0	0	0	0	0	0	0	0	0	0	0	0	0	0	0	0	0	0	0	0	0	0	0	0	0	0	0	0	0	0
ENSG00000222427	0	0	0	0	0	0	0	0	0	0	0	0	0	0	0	0	0	0	0	0	0	0	0	0	0	0	0	0	0	0	0	0	0	0	0	0	0	0	0	0	0	0	0	0	0	0	0	0
ENSG00000222428	0	0	0	0	0	0	0	0	0	0	0	0	0	0	0	0	0	0	0	0	0	0	0	0	0	0	0	0	0	0	0	0	0	0	0	0	0	0	0	0	0	0	0	0	0	0	0	0
ENSG00000222429	0	0	0	0	0	0	0	0	0	0	0	0	0	0	0	0	0	0	0	0	0	0	0	0	0	0	0	0	0	0	0	0	0	0	0	0	0	0	0	0	0	0	0	0	0	0	0	0
ENSG00000222430	0	0	0	0	0	0	0	0	0.15934105	0.29191206	0.40603745	0.27217991	0	0	0.28030094	0	0	0	0	0	0	0	0	0	0	0	0	0	0	0.25925945	0	0	0	0	0.62769651	0	0	0	0	0	0	0	0	0	0	0.44267663	0	0.30132097
ENSG00000222431	0	0	0	0	0	0	0	0	0	0	0	0	0	0	0	0	0	0	0	0	0	0	0	0	0	0	0	0	0	0	0	0	0	0	0	0	0	0	0	0	0	0	0	0	0	0	0	0
ENSG00000222432	0.85565681	0.38208833	0	0.3686788	0.47730358	0.42789964	0.22905215	0.55874844	0.14100978	0	0	0.24086717	0	0.30898886	0	0	0	0	0	0.50650542	0	0.24359936	0.28892552	0	0.27227473	0	0.24540821	0	0.20481565	0	0	0	0.93582025	0	0	0	0.26393681	0	0	0	0	0	0	0.24350746	0	0.78349846	0	0.26665573
ENSG00000222436	0	0	0	0	0	0	0	0	0	0	0	0	0.07976483	0	0	0	0	0	0	0	0	0	0	0	0	0	0	0	0	0	0	0.11529916	0.30920377	0.14831879	0	0.08382927	0	0.09496225	0	0	0	0	0	0	0.08692876	0	0	0
ENSG00000222437	0	0	0	0	0	0	0	0	0	0	0	0	0	0	0	0	0	0	0	0	0	0	0	0	0	0	0	0	0	0	0	0	0	0	0	0	0	0	0	0	0	0	0	0	0	0	0	0
ENSG00000222438	0	0	0	0	0	0.23702284	0.25375385	0	0	0	0	0	0	0	0	0.30481649	0.47534539	0	0	0	0.26598754	0.26986988	0.32008415	0	0	0	0	0.23825481	0	0	0	0	0	0	0	0	0	0	0	0	0	0	0	0	0	0	0	0
ENSG00000222439	0	0.1944864	0.28396492	0	0	0	0	0	0.1435505	0	0	0	0	0	0	0	0	0	0	0	0	0	0	0	0	0	0	0	0	0	0	0	0	0	0	0	0	0	0	0	0	0	0	0	0	0	0	0
ENSG00000222440	0	0	0	0	0	0	0	0	0	0	0	0	0	0	0	0	0	0	0	0	0	0	0	0	0	0	0	0	0	0	0	0	0	0	0	0	0	0	0	0	0	0	0	0	0	0	0	0
ENSG00000222445	0	0	0	0	0	0	0.23458815	0.09537549	0.33697504	0.17638191	0	0.08222958	0.08241563	0.73839937	0.59278144	0.18786273	0.14648106	0.23490573	0	0.17291575	0	0.08316232	0.29590861	0.18105393	0.0929518	0.08013315	0	0.14683982	0.20976587	0.15665224	0	0.35739256	0.79869855	0	0	0.25984541	0	0.0981181	0	0	0.18189897	0	0.11148431	0.16626189	0.35927052	0	0.08670688	0.36413411
ENSG00000222448	0	0.06725231	0	0	0	0.07531567	0	0	0	0.09093834	0	0	0	0	0.17464233	0	0	0.12111183	0	0	0.16903881	0.08575305	0	0	0	0	0	0	0.07210021	0	0	0.36852628	0	0	0	0.08931343	0	0	0.10113346	0	0	0	0.11495734	0	0.09261569	0	0	0.09386946
ENSG00000222449	0	0	0	0	0	0	0	0	0	0	0	0	0	0	0	0	0	0	0	0	0	0	0	0	0	0	0	0	0	0	0	0	0	0	0	0	0	0	0	0	0	0	0	0	0	0	0	0
ENSG00000222451	0	0	0	0	0	0	0	0	0	0	0.13097982	0	0	0	0	0	0	0	0	0	0	0	0	0	0	0	0	0	0	0	0	0	0	0	0	0	0	0	0	0	0	0	0	0	0.09590205	0	0	0
ENSG00000222452	0	0	0	0	0	0	0	0	0	0	0	0	0	0	0	0	0	0	0	0	0	0.08766474	0	0	0	0	0	0	0	0	0	0	0	0	0	0	0	0	0	0	0	0	0	0	0	0	0	0
ENSG00000222455	0	0.3889728	0	0	0	0	0	0	0.287101	0	0.73159901	0	0	0.62911246	0.25252337	1.12040656	0.43680387	0.35024232	0	0.51563164	0.24442098	0.49597708	0.58826277	0	0	0	0.24982998	0	0.20850602	0	0	0	0	0	0	0	0.26869243	0.29258641	0.87740106	0	0	0	0.66488839	0.24789498	0	0.39880777	0	0.27146034
ENSG00000222457	0	0	0	0	0	0	0	0	0	0	0	0	0	0	0	0	0	0	0	0	0	0	0	0	0	0	0	0	0	0	0	0	0	0	0	0	0	0	0	0	0	0	0	0	0	0	0	0
ENSG00000222459	0	0	0	0	0	0	0	0	0	0	0	0	0	0	0	0	0	0	0	0	0	0	0	0	0	0	0	0	0	0	0	0	0	0	0	0	0	0	0	0	0	0	0	0	0	0	0	0
ENSG00000222460	0	0	0	0	0	0	0	0	0	0	0	0	0	0	0	0	0	0	0	0	0	0	0	0	0	0	0.08665977	0	0	0	0	0	0	0	0	0	0	0	0	0	0	0.18035376	0	0	0	0	0	0
ENSG00000222463	0	0	0	0	0	0	0	0	0	0	0	0	0	0	0	0	0	0	0	0	0	0	0	0	0	0	0	0	0	0	0	0	0	0	0	0	0	0	0	0	0	0	0	0	0	0	0	0
ENSG00000222465	0	0	0	0	0	0	0	0	0	0	0	0	0	0	0	0	0	0	0.1970521	0	0	0	0	0	0	0	0.15406182	0	0	0	0	0	0	0	0	0	0	0	0	0	0	0	0	0	0.16516464	0	0	0
ENSG00000222467	0.35032326	0.70395621	0	0	0.19541777	0	0	0	0.1731968	0	0.88269011	0	0	0.37951893	0.30467493	0.33794872	0.52701336	0	0.38553672	0	0	0	0	0	0	0	0	0	0	0.28180375	0	0	0	0	0	0	0	0	0	0	0.65444087	0.62731741	0	0	0	0	0.31195628	0
ENSG00000222468	0	0	0	0	0	0	0	0	0	0	0	0	0	0	0	0	0	0	0	0	0	0	0	0	0	0	0	0	0	0	0	0	0	0	0	0	0.2813666	0	0	0	0	0	0	0	0	0	0	0
ENSG00000222472	0	0	0	0	0	0	0	0	0	0	0	0	0	0	0	0	0	0	0	0	0	0	0	0	0	0	0	0	0	0	0	0	0	0	0	0	0	0	0	0	0	0	0	0	0	0	0	0
ENSG00000222477	0	0	0	0	0	0	0	0	0	0	0	0	0	0	0	0	0	0	0	0	0	0	0	0	0.15777972	0	0	0	0	0	0	0	0	0	0	0	0	0	0	0	0	0	0	0	0	0	0	0
ENSG00000222481	0	0	0	0	0	0	0	0	0	0	0	0	0	0	0	0	0	0	0	0	0	0	0	0	0	0	0	0	0	0	0	0	0	0	0	0	0	0	0	0	0	0	0	0	0	0	0	0
ENSG00000222482	4.11443486	7.57876265	6.70640567	5.3183875	1.72133952	4.11511989	2.4781493	3.35843477	2.37316464	1.55272374	1.72781895	0.86865928	0.87062465	1.85722029	0.89457746	0.66151664	1.03160062	0.41358402	0.37733381	1.82665251	0.57724955	1.75702519	0.69465072	0.95631144	0.32730899	0.84651295	0	1.03412725	0.98485822	0.82742378	5.20942363	1.67797072	8.43731555	4.85665137	3.3388112	4.26994199	2.22100015	1.03650291	1.72679996	1.65224792	1.28103319	1.84191071	0	0.87818115	0.94881816	0.47093259	1.83191345	1.92332536
ENSG00000222486	0	0	0	0	0	0	0	0	0	0	0	0	0	0	0	0	0	0	0	0	0	0	0	0	0	0	0	0	0	0	0	0	0	0	0	0	0	0	0	0	0	0	0	0	0	0	0	0
ENSG00000222488	0	0	0	0	0	0	0	0	0	0	0	0	0	0	0	0	0	0	0	0	0	0	0	0	0	0	0	0	0	0	0	0	0	0	0	0	0	0	0	0	0	0	0	0	0	0	0	0
ENSG00000222489	43.1181654	45.6556828	49.8358443	121.32272	53.3279255	82.3302028	48.4429815	69.1111414	71.4881488	57.7906989	177.77856	87.1711322	36.1270014	79.9759214	72.5373373	38.4439499	61.2617424	105.072697	108.085741	105.188854	85.6084481	90.9497969	48.3110799	77.3406106	132.007253	117.207726	141.091482	194.908531	178.741785	164.489611	112.495391	89.2555709	76.0954648	62.3780719	36.0501372	92.0139527	146.907583	216.587087	184.034872	159.858708	160.28495	179.768825	154.835885	111.366819	211.723217	137.289576	156.686363	108.312674
ENSG00000222490	0	0	0	0	0	0	0	0	0	0	0	0	0	0	0	0	0	0	0	0	0	0	0	0	0	0	0	0	0	0	0	0	0	0	0	0	0	0	0	0	0	0	0	0	0	0	0	0
ENSG00000222492	0.69311268	0	0.67785176	0.8959291	0.57994952	1.03984212	0.55662135	0	0.34266893	0	0.43659941	0	0	0.75087616	0	1.00294458	0	0.41803116	0.38139117	0.30771566	0.58345653	0	0	0	0.33082844	0.28520508	0.29818417	0.52262345	0.74658607	0.83632081	1.05308779	0	0.56853596	0.54543038	0	0.30827538	0.32069741	1.0476481	0.69814708	0.55667134	1.29480774	0	0	0	0.3196735	0	2.16021335	0.32400105
ENSG00000222493	0	0	0	0	0	0.2844274	0	0	0.18746006	0	0	0	0	0	0	0	0.57041446	0	0	0	0	0.32384386	0.38410098	0	0	0	0	0	0.54456866	0.30501112	0	0	0	0	0	0	0	0	0	0	0	0	0	0.69952084	0	0	0	0
ENSG00000222494	0	0	0	0	0	0	0	0	0	0	0	0	0	0	0	0	0	0	0	0	0	0	0	0	0	0	0	0	0	0	0	0	0	0	0	0	0	0	0	0	0	0	0	0	0	0	0	0
ENSG00000222496	0	0.06746247	0	0	0	0	0.08088404	0	0	0	0.1268867	0	0	0	0	0.09716026	0	0	0	0	0	0	0	0	0	0	0	0.07594372	0	0	0	0	0	0	0	0	0	0	0	0	0	0	0	0.09290511	0.13833645	0	0	0
ENSG00000222499	0	0	0	0	0	0	0	0	0	0	0	0	0	0	0	0	0	0	0	0	0	0	0	0	0	0	0	0	0	0	0	0	0	0	0	0	0	0	0	0	0	0	0	0	0	0	0	0
ENSG00000222500	0	0	0	0	0	0	0	0	0	0	0	0	0	0	0	0	0	0	0	0	0	0	0	0	0	0	0	0	0	0	0	0	0	0	0	0	0	0	0	0	0	0	0	0	0	0	0	0
ENSG00000222501	0	0	0	0	0	0	0	0	0	0	0	0	0	0	0	0	0	0	0	0	0	0	0	0	0	0	0	0	0	0	0	0	0	0	0	0	0	0	0	0	0	0	0	0	0	0	0	0
ENSG00000222503	0.29842352	0.79955521	0.29185284	0.7714945	0	0.67156471	0.47931283	0.58461642	0.59015205	0.81086684	0	0.50403686	0	0.3232939	0.25953791	0	0	0.35997128	0	1.05990948	0	0	0	0	0.85464014	0	0	0.22501843	0	0.24005505	0	0	0	0	0	0	0.2761561	0.30071381	0	0	0	0	0	0.25478095	0.55054881	0	0.26574053	1.1160036
ENSG00000222506	0	0	0	0	0	0	0	0	0	0	0	0	0	0	0	0	0	0	0	0	0	0	0	0	0	0	0	0	0	0	0	0	0	0	0	0	0	0	0	0	0	0	0	0	0	0	0	0
ENSG00000222509	0	0	0	0	0	0	0	0	0	0	0	0	0	0	0	0	0	0	0	0	0	0	0	0	0	0	0	0	0	0	0	0	0.50356042	0	0	0	0	0	0	0	0	0	0	0	0	0	0	0
ENSG00000222511	0.97665878	0.21806051	0.95515475	0	0.18160035	0	0.78433008	0.31888169	0.32190112	0.29486067	0.41013884	0.2749292	0.27555124	0	0	0	0	0	0	0.57813244	0	0	0.65956735	0	0	0	0	0	0.23377948	0	0	0	0	0	0	0	0	0	0	0	0	0	0	0	0	0.44714811	0	0
ENSG00000222512	0	0	0	0	0	0	0	0	0	0	0	0	0	0	0	0	0	0	0	0	0	0	0	0	0	0	0	0	0	0	0	0	0	0	0	0	0	0	0	0	0	0	0	0	0	0	0	0
ENSG00000222514	0	0	0	0	0	0	0	0	0	0	0	0	0	0	0	0	0	0	0	0	0	0	0	0	0	0	0	0	0	0	0	0	0	0	0	0	0	0	0	0	0	0	0	0	0	0	0	0
ENSG00000222515	0.08594597	0.11513595	0	0.2221904	0	0.45129148	0	0	0	0	0	0	0	0	0	0	0	0	0	0	0	0.07340461	0	0	0	0	0	0	0	0	0	0	0.14099692	0	0.16738573	0.15290459	0	0	0	0	0	0	0	0	0	0	0	0.08035226
ENSG00000222518	0	0	0	0	0	0	0	0	0	0	0	0	0	0	0	0	0	0	0	0	0	0	0	0	0	0	0	0	0	0	0	0	0	0	0	0	0	0	0	0	0	0	0	0	0	0	0	0
ENSG00000222520	0	0	0	0	0	0	0	0	0	0	0	0	0	0	0	0	0	0	0	0	0	0	0	0	0	0	0	0	0	0	0	0	0	0	0	0	0	0	0	0	0	0	0	0	0	0	0	0
ENSG00000222521	0.38831012	0	0	0	0	0.29128108	0	0	0.19197717	0	0	0	0	0	0	0	0	0	0.42734191	0	0.32687625	0	0	0.36101717	0	0	0	0	0	0	0	0	0	0	0	0	0	0	0	0	0	0	0	0	0	0	0	0
ENSG00000222522	0	0.1998888	0.29185284	0.3857472	0	0.4477098	0	0	0	0	0	0	0	0	0	0	0	0	0	0	0.75363135	0	0	0.27744838	0.85464014	0	0	0	0	0	0	0	0	0	0.58120047	0	0	0.30071381	0.3005911	0	0	0	0	0	0	0.40988577	0	0
ENSG00000222524	0	0	0	0	0	0	0	0	0	0	0	0	0	0	0	0	0	0	0	0	0	0	0	0	0	0	0	0	0	0	0	0	0	0	0	0	0	0	0	0	0	0	0	0	0	0	0	0
ENSG00000222526	0	0	0	0	0	0	0	0	0	0	0	0	0	0	0	0	0	0	0	0	0	0	0	0	0	0	0	0	0	0	0	0	0	0	0	0	0	0	0	0	0	0	0	0	0	0	0	0
ENSG00000222529	0	0	0	0	0	0	0	0	0	0	0	0	0	0	0	0	0	0	0	0	0	0	0	0	0	0	0	0	0	0	0	0	0	0	0	0	0	0	0	0	0	0	0	0	0	0	0	0
ENSG00000222532	0	0	0	0	0	0	0	0	0	0	0	0	0	0	0	0	0	0	0	0	0	0	0	0	0	0	0	0	0	0	0	0	0	0	0	0	0	0	0	0	0	0	0	0	0	0	0	0
ENSG00000222533	0	0	0.30307795	0	0	0	0.24887397	0	0	0	0	0	0	0	0	0	0	0	0.34105172	0	0	0	0	0	0	0	0	0	0	0	0	0	0	0	0	0	0.57355498	0	0	0	0	0	0	0.26458022	0	0	0	0
ENSG00000222536	0	0	0	0	0	0	0	0	0	0	0	0	0	0	0	0	0	0	0	0	0	0	0	0	0	0	0	0	0	0	0	0	0	0	0	0	0	0	0	0	0	0	0	0	0	0	0	0
ENSG00000222543	0	0	0	0	0	0	0	0	0	0	0	0	0	0	0	0	0	0	0	0	0	0	0	0	0	0	0	0	0	0	0	0	0	0	0	0	0	0	0	0	0	0	0	0	0	0	0	0
ENSG00000222544	0	0	0	0	0	0	0	0	0	0	0	0	0	0	0	0	0	0	0	0	0	0	0	0	0	0	0	0	0	0	0	0	0	0	0	0	0	0	0	0	0	0	0	0	0	0	0	0
ENSG00000222545	0	0	0	0	0	0	0	0	0	0	0	0	0	0	0	0	0	0	0	0.32154557	0.6096793	0	0	0	0	0	0	0	0.26004683	0	0	0	0	0	0	0	0.33511078	0	0	0	0	0	0.41462141	0.30917239	0	0	0	0
ENSG00000222546	0	0	0	0	0	0	0	0	0	0	0	0	0	0	0	0	0	0	0	0	0	0	0	0	0	0	0	0	0	0	0	0	0	0	0	0	0	0	0	0	0	0	0	0	0	0	0	0
ENSG00000222548	0	0	0	0	0	0	0	0	0	0	0	0	0	0	0	0	0	0	0	0	0	0	0	0	0	0	0	0	0	0	0	0	0	0	0	0	0	0	0	0	0	0	0	0	0	0	0	0
ENSG00000222551	0	0	0	0	0	0	0	0	0	0	0	0	0	0	0	0	0	0	0	0	0	0	0	0	0	0	0	0	0	0	0	0	0	0	0	0	0	0	0	0	0	0	0	0	0	0	0	0
ENSG00000222552	0	0	0	0	0	0	0	0	0	0	0	0	0	0	0	0	0	0	0	0.32893743	0.31184746	0	0	0	0	0	0.3187486	0.27933322	0	0	0	0	0	0	0	0	0	0	0	0	0	0	0	0	0	0	0	0
ENSG00000222558	0.30990134	0	0	0	0	0	0	0	0	0	0	0	0	0	0	0	0	0	0	0	0	0	0	0	0	0	0	0	0	0	0	0	0	0	0	0	0	0	0	0	0	0	0	0	0	0	0	0
ENSG00000222560	0	0	0	0	0	0	0	0	0	0	0	0	0	0	0	0	0	0	0	0	0	0	0	0	0	0	0	0	0	0	0	0	0	0	0	0	0	0	0	0	0	0	0	0	0	0	0	0
ENSG00000222561	0	0.6227305	0	0	0	0	0.24887397	0	0.15321255	0.28068468	0	0	0	0.33572828	0.26952013	0.29895463	0	0	0.34105172	0	0.26087239	0	0	0	0	0.25503916	0	0.23367298	0.22254008	0	0.47085175	0	0	0	0	0.27566933	0.28677749	0	0	0	0	0	0	0	0.28586188	0	0.27596132	0
ENSG00000222563	0	0	0	0	0	0	0	0	0	0	0	0	0	0	0	0	0	0	0	0	0	0	0	0	0	0	0	0	0	0	0	0	0	0	0	0	0	0	0	0	0	0	0	0	0	0	0	0
ENSG00000222570	0	0	0	0	0	0	0	0	0	0	0	0	0.26485022	0	0	0	0	0	0	0	0	0	0	0	0	0	0	0	0	0	0	0	0	0	0	0	0	0	0	0	0	0	0	0	0	0	0	0
ENSG00000222574	0	0	0	0	0	0	0	0	0	0	0	0	0	0	0	0	0	0	0	0	0	0	0	0	0	0	0	0	0	0	0	0	0	0	0	0	0	0	0	0	0	0	0	0	0	0	0	0
ENSG00000222578	0.29842352	0.5996664	0	0	0	0.2238549	0.23965641	0	0	0	0.3759606	0	0.25258863	0.3232939	0	0	0	0.35997128	0.32842017	0.26497737	0	0.25487711	0	0	0	0	0	0	0.42859571	0.96022019	0	0	0	0	0	0	0	0.30071381	0	0	0	0	0	0	0.2752744	0	0	0
ENSG00000222579	0	0	0	0	0	0	0	0	0	0	0	0	0	0	0	0	0	0	0	0	0	0	0	0	0	0	0	0	0	0	0	0	0	0	0	0	0	0	0	0	0	0	0	0	0	0	0	0
ENSG00000222581	0	0	0	0	0	0	0	0	0	0	0	0	0	0	0	0	0	0	0	0	0	0	0	0	0	0	0	0	0	0	0	0	0	0	0	0	0	0	0	0	0	0	0	0	0	0	0	0
ENSG00000222582	0	0	0	0	0	0	0	0	0	0	0	0	0	0	0	0	0	0	0	0	0	0	0	0	0	0	0	0	0	0	0	0	0	0	0	0	0	0	0	0	0	0	0	0	0	0	0	0
ENSG00000222583	0	0	0	0	0	0	0	0	0	0	0	0	0	0	0	0	0	0	0	0	0	0	0	0	0	0	0	0	0	0	0	0	0	0	0	0	0	0	0	0	0	0	0	0	0	0	0	0
ENSG00000222585	0	0	0	0	0	0	0	0	0	0	0	0	0	0	0	0	0	0	0	0	0	0	0	0	0	0	0	0	0	0	0	0	0	0	0	0	0	0	0	0	0	0	0	0	0	0	0	0
ENSG00000222586	0	0	0	0	0	0	0	0	0	0	0	0	0	0	0	0	0	0	0	0	0	0	0	0	0	0	0	0	0	0	0	0	0	0	0	0	0	0	0	0	0	0	0	0	0	0	0	0
ENSG00000222588	0	0.16734876	0.97736765	0	0.27873543	0.37482681	0.40128516	0.73416946	0.86464138	1.35773052	0.31475771	0.42198435	0.63440866	0.27066466	0.21728755	0.48203538	0.75170898	0.3013713	0.27495642	0.22184152	0.21031573	0.42677098	0.75926939	0	0	0.82245186	0.21496998	0.18838752	0.17941216	0.40195264	0.37960141	0.91703051	0.81974952	0	0	1.11122521	0	1.00704158	0.50331534	1.2039636	0.46673302	2.23694582	0	0.21330498	0.23046229	0.68632036	0.22248045	1.4014929
ENSG00000222589	0	0.07393147	0	0	0.06156998	0	0	0	0	0	0	0	0	0	0	0.10647699	0	0	0	0	0	0.09426962	0	0.10261789	0	0	0.09496962	0.24967798	0.1585217	0	0.50310187	0	0	0.17371584	0	0	0.20427986	0	0	0	0.2061937	0.19764795	0	0	0.10181382	0.15160159	0.09828759	0
ENSG00000222591	0	0	0	0	0	0	0	0	0	0	0	0	0	0	0	0	0	0	0	0	0	0	0	0	0	0	0	0	0	0	0	0	0	0	0	0	0	0	0	0	0	0	0	0	0	0	0	0
ENSG00000222592	0	0	0	0	0	0	0	0	0	0	0	0	0	0	0	0	0	0	0	0	0	0	0	0	0	0	0	0	0	0	0	0	0	0	0	0	0	0	0	0	0	0	0	0	0	0	0	0
ENSG00000222594	0	0	0	0	0	0	0	0	0	0	0	0	0	0	0	0	0	0	0	0	0	0	0	0	0	0	0	0	0	0	0	0	0	0	0	0	0	0	0	0	0	0	0	0	0	0	0	0
ENSG00000222596	0	0	0.2973595	0.3930255	0	0.22807858	0	0	0	0	0	0	0	0	0.26443485	0	0	0	0	0	0	0	0	0	0	0	0	0	0	0	0	0	0	0	0	0	0	0	0	0	0	0	0.34812553	0	0	0	0	0
ENSG00000222598	0	0	0	0	0	0	0	0	0	0	0	0	0	0	0	0	0	0	0	0	0	0	0	0	0	0	0	0	0	0	0	0	0	0	0	0	0	0	0	0	0	0	0	0	0	0	0	0
ENSG00000222601	0	0.24256169	0	0	0	0	0	1.06413326	0	0.32799108	0	0	0	0	0	0.69868049	0	0	0	0	0	0	0	0	0	0	0	0	0	0	0	0	0	0	0	0	0	0.36491114	0	0	0	0	0	0	0	0.49738947	0	0
ENSG00000222602	0	0	0	0	0	0	0	0	0	0	0	0	0	0	0	0	0	0	0	0	0	0	0	0	0	0	0	0	0	0	0	0	0	0	0	0	0	0	0	0	0	0	0	0	0	0	0	0
ENSG00000222604	0	0	0	0	0	0	0	0	0	0	0	0	0	0	0	0	0	0	0	0	0	0	0	0	0	0	0	0	0	0	0	0	0	0	0	0	0	0	0	0	0	0	0.3884348	0	0.31294353	0	0	0
ENSG00000222607	0	0	0	0	0	0	0	0	0	0	0	0	0	0	0	0	0	0	0	0	0	0	0	0	0	0	0	0	0	0	0	0	0	0	0	0	0	0	0	0	0	0	0	0	0	0	0	0
ENSG00000222608	0	0	0	0	0	0	0	0	0	0	0	0	0	0	0	0	0	0	0	0	0	0	0	0	0	0	0	0	0	0	0	0	0	0	0	0	0	0	0	0	0	0	0	0	0	0	0	0
ENSG00000222609	0	0	0	0	0	0	0	0	0	0	0	0	0	0	0	0	0	0	0	0	0	0	0	0	0	0	0	0	0	0	0	0	0	0	0	0	0	0	0	0	0	0	0	0	0	0	0	0
ENSG00000222610	0	0	0	0	0	0	0	0	0	0	0	0	0	0	0	0	0	0	0	0	0	0	0	0	0	0	0	0	0	0	0	0	0	0	0	0	0	0	0	0	0	0	0	0	0	0	0	0
ENSG00000222611	0	0.26326818	0.38439154	0	0	0	0	0	0.19431836	0	0	0	0	0	0.34183041	0	0	0	0	0	0	0.3356918	0.39815346	0	0	0.6469286	0	0	0	0	0	0	0	0	0	0	0.36371779	0	0	0	0	0	0	0	0	0	0	0
ENSG00000222612	0	0	0	0	0	0	0	0	0	0	0	0	0	0.18376706	0	0	0	0	0	0	0	0	0	0	0	0	0	0	0.12181141	0	0	0	0	0	0	0	0	0	0	0	0	0	0	0	0	0	0	0
ENSG00000222613	0	0	0	0	0	0	0	0	0	0	0	0	0	0	0	0	0	0	0	0	0	0	0	0	0	0	0	0	0	0	0	0	0	0	0	0	0	0	0	0	0	0	0	0	0	0	0	0
ENSG00000222614	0	0	0	0	0	0	0	0	0	0	0	0	0	0	0	0	0	0	0	0	0	0	0	0	0	0	0	0	0	0	0	0	0	0	0	0	0	0	0	0	0	0	0	0	0	0	0	0
ENSG00000222615	0	0	0	0	0	0	0	0	0	0	0	0	0	0	0	0	0	0	0	0	0	0	0	0	0	0	0	0	0	0	0	0	0	0	0	0	0	0	0	0	0	0	0	0	0	0	0	0
ENSG00000222616	0	0	0	0	0	0	0	0	0	0	0	0	0	0	0	0	0	0	0	0	0	0	0	0	0	0	0	0	0	0	0	0	0	0	0	0	0	0	0	0	0	0	0	0	0	0	0	0

ENSG00000222617	0	0	0	0	0	0	0	0	0	0	0	0	0	0	0	0	0	0	0	0	0	0	0	0	0	0	0	0	0	0	0.15796317	0	0	0	0	0	0	0	0	0	0	0	0	0	0	0	0	0
ENSG00000222623	0	0	0	0	0	0	0	0	0	0	0	0	0	0	0	0	0	0	0	0	0	0	0	0	0	0	0	0	0	0	0	0	0	0	0	0	0	0	0	0	0	0	0	0	0	0	0	0
ENSG00000222624	0	0	0	0	0	0	0.13551253	0.16528422	0	0.15283354	0	0.28500514	0	0	0	0	0	0	0	0	0	0	0	0	0	0	0.14518915	0	0	0.13573793	0	0	0	0	0	0	0	0.17003713	0.16996774	0	0	0	0	0	0	0	0	0
ENSG00000222626	0	0	0	0	0	0	0	0	0	0	0	0	0	0	0	0	0	0	0	0	0	0	0	0	0	0	0	0	0	0	0	0	0	0	0	0	0	0	0	0	0	0	0	0	0	0	0	0
ENSG00000222627	0	0.2439321	0	0.2353712	0	0.27317886	0.58492413	0.17835755	0.27006958	0.32984414	0.22939969	0	0.15412188	0.39452815	0.15836211	0.17565696	0	0	0.20039197	0.16168111	0	0.15551824	0.18445528	0.50787161	0.17382511	0.29970703	0.15667304	0	0.13075801	0	0.27665866	0	0.29872228	0	0	0	0.16850203	0.36697278	0.18341152	0.58497667	0	0	0	0.15545956	0.16796404	0.25009979	0	0
ENSG00000222629	0.57043787	0.95522082	0.278939	0	0	0.21394982	0.22905215	0.27937422	0.8460587	0.51665852	0	0.9634687	0.24141215	0.61797773	0.24805393	0	0	0.68808669	0	0.25325271	0.24009494	0.24359936	0	0.53034381	0	0.23472631	0.24540821	0.21506186	0	0.22943314	0.43335028	0	0.46791012	0	0	0	0	0.28740789	0	0	0	0	0	0.24350746	0.26309412	0	0	0
ENSG00000222630	0	0	0	0	0	0	0	0	0.05224297	0	0	0	0	0	0	0	0	0	0	0	0	0	0	0	0	0	0	0	0	0	0	0	0	0	0	0	0	0	0	0	0	0	0	0	0	0	0	0
ENSG00000222631	0	0	0	0	0	0	0	0	0	0	0	0	0	0	0	0	0	0.53280472	0	0	0	0	0	0	0	0	0	0	0	0	0	0	0	0	0	0	0.32774571	0.35689111	0	0	0	0	0.40550886	0	0	0	0	0.99336585
ENSG00000222635	0	0	0	0	0	0	0	0	0	0	0	0	0	0	0	0	0	0	0	0	0	0	0	0	0	0	0	0	0	0	0	0	0	0	0	0	0	0	0	0	0	0	0	0	0	0	0	0
ENSG00000222636	0	0	0	0	0	0	0	0	0	0	0	0	0	0	0	0	0	0	0	0	0	0	0	0	0	0	0	0	0	0	0	0	0	0	0	0	0	0	0	0	0	0	0	0	0	0	0	0
ENSG00000222640	0	0	0	0	0	0	0	0	0	0	0	0	0	0	0	0	0	0	0	0	0	0	0	0	0	0	0	0	0	0	0	0	0	0	0	0	0	0	0	0	0	0	0	0	0	0	0	0
ENSG00000222643	0	0	0	0	0	0	0	0	0	0	0	0	0	0	0	0	0	0	0	0	0	0	0	0	0	0	0	0	0	0	0	0	0	0	0	0	0	0	0	0	0	0	0	0	0	0	0	0
ENSG00000222644	0	0	0	0	0	0	0	0	0	0	0	0	0	0	0	0	0	0	0	0	0	0	0	0	0	0	0	0	0	0	0	0	0	0	0	0	0	0	0	0	0	0	0	0	0	0	0	0
ENSG00000222647	0	0	0	0	0	0	0	0	0	0	0	0	0	0	0	0	0	0	0	0	0	0	0	0	0	0	0	0	0	0	0	0	0	0	0	0	0	0	0	0	0	0	0	0	0	0	0	0
ENSG00000222649	0	0	0	0	0	0	0	0	0	0	0	0	0	0	0	0	0	0	0	0	0	0	0	0	0	0	0	0	0	0	0	0	0	0	0	0	0	0	0	0	0	0	0	0	0	0	0	0
ENSG00000222650	0	0.1206033	0	0.2327414	0	0	0	0	0	0	0	0	0	0	0.1565927	0	0	0	0	0	0	0	0.18239432	0	0	0.14817918	0.1549225	0	0	0.14483768	0	0	0	0	0	0	0	0	0.18136223	0.28922031	0	0	0	0	0.15372258	0	0	0
ENSG00000222651	0	0	0	0	0	0	0	0	0	0	0	0	0	0	0	0	0	0	0	0	0	0	0	0	0	0	0	0	0	0	0	0	0	0	0	0	0	0	0	0	0	0	0	0	0	0	0	0
ENSG00000222652	0	0	0	0	0	0	0	0	0	0	0	0	0	0	0	0	0	0	0	0	0	0	0	0	0	0	0	0	0	0	0	0	0	0	0	0	0	0	0	0	0	0	0	0	0	0	0	0
ENSG00000222658	0	0	0	0	0	0	0	0	0	0	0	0	0	0	0	0	0	0	0	0	0	0	0	0	0	0	0	0	0	0	0	0	0	0	0	0	0	0	0	0	0	0	0	0	0	0	0	0
ENSG00000222659	0	0	0	0	0	0	0	0	0	0	0	0	0	0	0	0	0	0	0	0	0	0	0	0	0	0	0	0	0	0	0	0	0	0	0	0	0	0	0	0	0	0	0	0	0	0	0	0
ENSG00000222663	0.23525357	0.47272972	0	0.3040927	0.13122945	0	0	0	0	0	0	0	0	0	0	0	0	0	0	0	0	0	0	0	0	0	0	0	0	0	0	0	0	0	0	0	0	0	0	0	0	0	0	0	0	0	0	0
ENSG00000222664	0	0	0	0	0	0	0	0	0	0	0	0	0	0	0	0	0	0	0	0	0	0	0	0	0	0	0	0	0	0	0	0	0	0	0	0	0	0	0	0	0	0	0	0	0	0	0	0
ENSG00000222666	0	0	0	0	0	0	0	0	0	0	0	0	0	0	0	0	0	0	0	0	0	0	0	0	0	0	0	0	0	0	0	0	0	0	0	0	0	0	0	0	0	0	0	0	0	0	0	0
ENSG00000222667	0	0	0	0	0	0	0	0	0	0	0	0	0	0	0	0	0	0	0	0	0	0	0	0	0	0	0	0	0	0	0	0	0	0	0	0	0	0	0	0	0	0	0.34487202	0	0	0	0	0
ENSG00000222675	0.2620304	0	0	0	0.14616614	0.19655552	0.21043002	0	0.12954557	0	0.33011175	0.22128448	0.22178514	0	0	0.25277465	0	0	0.86510679	0.23266306	0	0.44758907	0.26543564	0	0	0.43128573	0.22545632	0	0.18816397	0.21078004	0	0	0	0	0	0	0	0	0	0	0	0	0	0	0	0	0	0
ENSG00000222677	0	0	0	0	0	0	0	0	0	0	0	0	0	0	0	0	0	0	0	0	0	0	0	0	0	0	0	0	0	0	0	0	0	0	0	0	0	0	0	0	0	0	0	0	0	0	0	0
ENSG00000222678	0	0	0.10577217	0	0.06033032	0	0	0	0	0	0	0	0	0	0	0	0	0	0	0	0.13045939	0	0	0	0	0.08900695	0	0	0	0	0	0	0	0	0	0	0	0	0	0	0	0	0	0	0	0	0.09630865	0
ENSG00000222679	0	0.20175692	0	0	0	0.45189401	0.24189619	0.29504006	0.89350124	0.54563002	0	0	0	0.65263068	0	0.58114546	0.90626597	0.36333549	0	0.26745379	0.25355821	0.25725914	0	0.84012407	0	0.25916942	0.45424281	0	0.48459711	0	0	0	0	0	0	0	0	0	0.30340037	0.48383584	0.56269682	1.61812716	0.34487202	0	0	0	0	0
ENSG00000222682	0	0	0	0	0	0	0	0.26528812	0	0	0	0	0	0.29340959	0	0	0	0	0	0	0	0	0	0	0	0	0	0	0	0	0	0	0	0	0	0	0	0	0	0	0	0	0	0	0	0	0	0
ENSG00000222683	0	0	0	0	0	0	0	0	0	0	0	0	0	0	0	0	0	0	0	0	0	0	0	0	0	0	0	0	0	0	0	0	0	0	0	0	0	0	0	0	0	0	0	0	0	0	0	0
ENSG00000222685	0	0.15815378	0	0	0	0	0	0.11563841	0.11673337	0	0.14873167	0	0	0	0	0	0	0	0	0	0.09937996	0	0	0	0	0.09715777	0	0.08901828	0	0.09496683	0	0.1444407	0	0	0	0	0.21849714	0	0	0	0	0	0.21140367	0	0.10079246	0	0	0
ENSG00000222686	0.18629907	0	0	0	0	0.13974757	0	0	0.09210466	0	0.23470373	0.15732943	0	0.2018251	0	0	0	0	0	0	0	0	0	0	0	0.30663668	0	0.14047393	0	0	0	0	0	0	0	0	0	0	0	0	0	0	0	0	0	0	0	0
ENSG00000222691	0	0	0	0	0	0	0	0	0	0	0	0	0	0	0	0	0	0	0	0	0	0	0	0	0	0	0	0	0	0	0	0	0	0	0	0	0	0	0	0	0	0	0	0	0	0	0	0
ENSG00000222692	0	0	0	0	0	0	0	0	0	0	0	0	0	0	0	0	0	0	0	0	0	0	0	0	0	0	0	0	0	0	0	0	0	0	0	0	0	0	0	0	0	0	0	0	0	0	0	0
ENSG00000222693	0	0	0	0.1221721	0	0	0	0	0.04672758	0	0	0	0	0	0	0	0	0	0	0	0	0	0	0	0	0	0	0	0	0	0	0	0	0	0	0	0	0	0	0.15181946	0	0	0	0	0	0	0	0.08836392
ENSG00000222698	0	0	0	0	0	0	0	0	0	0	0	0	0	0	0	0	0	0	0	0	0	0	0	0	0	0	0	0	0	0	0	0	0	0	0	0	0	0	0	0	0	0	0	0	0	0	0	0
ENSG00000222701	0	0	0	0	0	0	0	0	0	0	0	0	0	0	0	0	0	0	0	0	0	0	0	0	0	0	0.23701819	0	0	0	0	0	0	0	0	0	0	0	0	0	0	0	0	0	0	0	0	0
ENSG00000222704	0	0	0	0	0	0	0	0	0	0	0	0	0	0	0	0	0	0	0	0	0	0	0	0	0	0	0	0	0	0	0	0	0	0	0	0	0	0	0	0	0	0	0	0	0	0	0	0
ENSG00000222705	0	0	0	0	0	0	0	0	0	0	0	0	0	0	0	0	0	0	0	0	0	0	0	0	0	0	0	0	0	0	0	0	0	0	0	0	0	0	0	0	0	0	0	0	0	0	0	0
ENSG00000222706	0	0.08920657	0.13024837	0	0	0.09990219	0	0	0	0	0	0	0	0.14427992	0	0.12847637	0.40070437	0	0	0	0	0	0.1349115	0.12381994	0	0	0.10042145	0	0.42852802	0.20234951	0	0	0	0	0	0	0	0	0	0	0	0	0.15248473	0.1137039	0.12284973	0	0	0
ENSG00000222713	0	0	0	0	0	0	0	0	0	0	0	0	0	0	0	0	0	0	0	0	0	0	0	0	0	0	0	0	0	0	0	0	0	0	0	0	0	0	0	0	0	0	0	0	0	0	0	0
ENSG00000222714	0.10430336	0.20959214	0.40802727	0	0	0.1564811	0	0.10216598	0.05156668	0	0.26280741	0	0	0	0	0.10061904	0	0.1258152	0	0.09261345	0	0.08908326	0.10565885	0.1939445	0.09956972	0.08583842	0.08974475	0	0.25170821	0.15847438	0	0	0	0.40627606	0	0	0.09652058	0.42041542	0	0	0.74709647	0.2388434	0.08904965	0	0	0	0.14326105	0.27864056
ENSG00000222715	0	0	0	0	0	0	0	0	0	0	0	0	0	0	0	0	0	0	0	0	0	0	0	0	0	0	0	0	0	0	0	0	0	0	0	0	0	0	0	0	0	0	0	0	0	0	0	0
ENSG00000222717	0	0	0	0	0	0	0	0	0	0	0	0	0	0	0	0	0	0	0	0	0	0	0	0	0	0	0	0	0	0	0	0	0	0	0	0	0	0	0	0	0	0	0	0	0	0	0	0
ENSG00000222719	0	0	0	0	0	0	0	0	0	0	0	0	0	0	0	0	0	0	0	0	0	0	0	0	0	0	0	0	0	0	0	0	0	0	0	0	0	0	0	0	0	0	0	0	0	0	0	0
ENSG00000222720	0.78609121	0	1.92195772	0.5080573	0.65774762	0.58966657	0	0	0.19431836	0.35599032	0.49516762	0	0	0	0.75832395	0	0	0	0	0	0	0.67138361	0.79630692	0.73083964	1.50083146	0.9703929	0	0.29636574	0	0.31617006	0.59717783	0	0	0	0	0	0.72743559	0	1.18770143	0	0.73425073	0.70381954	0	0	0	0	0	0
ENSG00000222721	0	0	0	0	0	0	0	0	0	0	0	0	0	0	0	0	0	0	0	0	0	0	0	0	0	0	0	0	0	0	0	0	0	0	0	0	0	0	0	0	0	0	0	0	0	0	0	0
ENSG00000222722	0	0	0	0	0	0	0	0	0	0	0	0	0	0	0	0	0	0	0	0	0.36663147	0	0	0	0	0	0	0	0	0	0	0.53286908	0	0	0	0	0	0	0	0	0	0	0	0.37184247	0	0	0	0
ENSG00000222724	0	0	0	0	0	0.40025092	0	0.57494195	0.45141041	1.46508359	0.98209245	0.14061635	0.17997805	1.01139514	0.16026434	0.74977159	0.40079276	0	0	0	0	0	0.1544558	0	0	0.13672202	0	0	0	0	0	0	0	0	0	0	0	0.50222306	0.16733938	0.26685791	2.48282722	0.59498146	0	0.56734727	0.45973663	1.14091915	0.29587606	0
ENSG00000222726	0.18106595	0.36384254	0.708317	0.4680978	0.50501222	0.95075453	0.14540951	0	0.26855234	0.49198662	0.22811093	0.30582012	0.30651205	0	0.15747244	0	0.27238893	0	0.39853234	0.64309115	0.30483965	0.15464454	0.55025703	0	0.17284857	0.59604657	0.15579285	0	0.26004683	0	0.55020879	0.44305968	0.8911322	0	0.70527697	0	0.16755539	0.36491114	0.18238112	0.58169028	1.01475101	0.32423147	0.41462141	0	0.24869474	0	0	0
ENSG00000222727	0	0	0	0	0	0	0	0	0	0	0	0	0	0	0	0	0	0	0	0	0	0	0	0	0	0	0	0	0	0	0	0	0	0	0	0	0	0	0	0	0	0	0	0	0	0	0	0
ENSG00000222729	0	0	0	0	0	0	0	0	0	0	0	0	0	0	0	0	0	0	0	0	0	0	0	0	0	0	0	0	0	0	0	0	0	0	0	0	0	0	0	0	0	0	0	0	0	0	0	0
ENSG00000222730	0	0	0	0	0	0	0	0	0	0	0.41013884	0	0	0.70536852	0	0	0	0	0	0	0	0	0	0	0	0.26791992	0	0	0	0	0.98926428	0	0	0	0	0	0.3012612	0	0	0	0.60816727	0	0	0	0.30029935	0	0	0
ENSG00000222732	0	0	0	0	0	0	0	0	0	0	0	0	0	0	0	0	0	0	0	0	0	0	0	0	0	0	0	0	0	0	0	0	0	0	0	0	0	0	0	0	0	0	0	0	0	0	0	0
ENSG00000222733	0	0	0	0	0	0	0	0	0	0	0	0	0	0	0	0	0	0	0	0	0	0	0	0	0	0	0	0	0	0	0	0	0	0	0	0	0	0	0	0	0	0	0	0	0	0	0	0
ENSG00000222736	0	0	0	0	0	0	0	0	0	0	0	0	0	0	0	0	0	0	0	0	0	0	0	0	0	0	0	0	0	0	0	0	0	0	0	0	0	0	0	0	0	0	0	0	0	0	0	0
ENSG00000222740	0	0.19805496	0	0	0.16493977	0	0	0	0	0	0	0	0	0	0	0	0.44481862	0	0.32540714	0.26254639	0	0.25253879	0	0	0	0	0	0.22295404	0	0	0	0	0	0	0	0	0	0	0	0	0	0	0	0	0	0	0.26330255	0
ENSG00000222741	0	0	0	0.8416303	0	0	0	0.31888169	0	0	0	0	0	0	0	0	0	0	0	0.28906622	0	0	0	0	0	0	0	0	0	0	0	0	0	0	0	0	0	0	0.32791757	0	0	0	0	0.44714811	0	0	0	0
ENSG00000222743	0	0	0	0	0	0	0	0	0	0	0	0	0	0	0	0	0	0	0	0	0	0	0	0	0	0	0	0	0	0	0	0	0	0	0	0	0	0	0	0	0	0	0	0	0	0	0	0
ENSG00000222744	0	0	0	0.1365925	0	0	0	0	0	0	0	0	0	0	0	0	0	0	0	0	0	0	0	0	0	0	0	0	0	0.0850031	0.16055273	0.12928627	0	0	0.20580213	0	0.09778642	0.10648227	0	0	0	0	0	0	0	0	0.09409829	0
ENSG00000222747	0	0	0	0	0	0	0	0	0	0	0	0	0	0	0	0	0	0	0	0	0	0	0	0	0	0	0	0	0	0	0	0	0	0.4611366	0	0	0	0	0	0	0	0	0	0	0	0	0	0
ENSG00000222749	0	0	0	0	0	0	0	0	0	0	0	0	0	0	0	0	0	0	0	0	0	0	0	0	0	0	0	0	0	0	0	0	0	0	0	0	0	0	0	0	0	0	0	0	0	0	0	0
ENSG00000222750	0	0	0	0	0	0	0	0	0	0	0	0	0	0	0	0	0	0	0	0	0	0	0	0	0	0	0	0	0	0	0	0	0	0	0	0	0	0	0	0	0	0	0	0	0	0	0	0
ENSG00000222755	0	0	0	0	0	0	0	0	0	0	0	0	0	0	0	0	0	0	0	0	0	0	0	0	0	0	0	0	0	0	0	0	0	0	0	0	0	0	0	0	0	0	0	0	0	0	0	0
ENSG00000222760	0	0	0	0	0	0	0	0	0.22763008	0	0	0	0	0.19485409	0	0	0	0	0	0	0	0	0	0	0	0	0	0	0	0.37037064	0	0	0	0	0	0	0	0	0	0	0	0	0	0	0	0	0	0
ENSG00000222761	0	0	0	0	0	0	0	0	0	0	0	0	0	0	0	0	0	0	0	0	0	0	0	0	0	0	0	0	0	0	0	0	0	0	0	0	0	0	0	0	0	0	0	0	0	0	0	0
ENSG00000222764	0	0	0	0	0	0.19031539	0.11606355	0.05858127	0.21464122	0	0	0	0	0.10305182	0	0.35650904	0.28585954	0.26080425	0.52605801	0	0.40480482	0.36009467	0.33048998	0.33934241	0.39005989	1.01952676	0.35738221	0.17017771	0.09531597	0.90015776	0.14497174	0.38877827	0.74595626	0.23077077	0.31620894	0.21930044	0.11940107	0.11935235	0.38066496	0	0	0	0	0.20232605	0.43720053	0	0.10551462	0.55389885
ENSG00000222765	0	0	0	0	0	0	0	0	0	0	0	0	0	0	0	0	0	0	0	0	0	0	0	0	0	0	0	0	0	0	0	0	0	0	0	0	0	0	0	0	0	0	0	0	0	0	0	0
ENSG00000222767	0	0.22724201	0.3317906	0.4385337	0.37849337	0	0	0	0.16772743	0	0	0	0	0	0.29505362	0	0	0	0	0.30123743	0	0	0.3436693	0	0.32386363	0.27920076	0	0	0	0	0.51545876	0	0	0.53394764	0	0	0	0.34186412	0.34172462	0	0	0	0	0.31294353	0	0	0	0
ENSG00000222770	0	0	0	0	0	0	0	0	0	0	0	0	0	0	0	0	0	0	0	0	0	0	0	0	0	0	0	0	0	0	0	0	0	0	0	0	0	0	0	0	0	0	0	0	0	0	0	0
ENSG00000222774	0	0	0	0	0	0	0	0	0	0	0	0	0	0	0	0	0	0	0	0	0	0	0	0	0	0	0	0	0	0	0	0	0	0	0	0	0	0	0	0	0	0	0	0	0	0	0	0
ENSG00000222776	0	0	0	0	0	0	0	0	0	0	0	0	0	0	0	0	0	0	0	0	0	0	0	0	0	0	0	0	0	0	0	0	0	0	0	0	0	0	0	0	0	0	0	0	0	0	0	0
ENSG00000222777	0	0	0	0	0	0	0	0	0	0	0	0	0	0	0	0	0	0	0	0	0	0	0	0	0	0	0	0	0	0	0	0	0	0	0	0	0	0	0	0	0	0	0	0	0	0	0	0
ENSG00000222778	0	0	0	0	0	0	0	0	0	0	0	0	0	0	0	0	0	0	0	0	0	0	0	0	0	0	0	0	0	0	0	0	0	0	0	0	0	0	0	0	0	0	0	0	0	0	0	0
ENSG00000222783	0	0	0	0	0	0	0	0	0	0	0	0	0	0	0	0	0	0	0	0	0	0	0	0	0	0	0	0	0	0	0	0	0	0	0	0	0	0	0	0	0	0	0	0	0	0	0	0
ENSG00000222784	0.67145291	0.5996664	0.7661137	1.1572417	0.06242512	0.92340147	0.26961347	0.43846232	0.77457457	0.70950849	0.98689658	0.18901382	0.09472074	0	0	0.21591168	0.16835149	0.40496768	0.49263026	0.19873303	0	0.38231567	0.34008941	0.20808628	0.6409801	0.73677979	0.77030911	0.92820102	0.32144678	0.6301445	1.02017879	0.13691775	0.18358974	0	0.8718007	0.39818903	0.31067562	0.56383839	1.69082496	0.17975845	0	0.20039306	0.51251814	0.47771428	0.82582321	0.46112149	0.5979162	0.31387601
ENSG00000222787	0	0	0	0	0	0	0	0	0	0	0	0	0	0	0	0	0	0	0	0	0	0	0	0	0	0	0	0	0	0	0	0	0	0	0	0	0	0	0	0	0	0	0	0	0	0	0	0
ENSG00000222788	0	0	0	0	0	0	0	0	0	0	0	0	0	0	0	0	0	0	0	0	0	0	0	0	0	0	0	0	0	0	0	0	0	0	0	0	0	0	0	0	0.46710514	0	0	0	0	0	0	0.76283791
ENSG00000222790	0	0.35390148	0	0.6829623	0	0.39633327	0.21215486	0	0	0	0	0	0	0	0	0	0.39741991	0	0.29073261	0.46914026	0	0	0	0	0	0	0.19919664	0	0.21250775	0.40138182	0	0	0.83155779	0	0	0	0	0	0	0	0.60493944	0	0	0	0	0	0.47049143	0.74095321
ENSG00000222791	0	0	0	0	0	0	0	0	0	0	0	0	0	0	0	0	0	0	0	0	0	0	0	0	0	0	0	0	0	0	0	0	0	0	0	0	0	0	0	0	0	0	0	0	0	0	0	0
ENSG00000222792	0	0	0	0	0	0	0	0	0	0	0	0	0	0	0	0	0	0	0	0	0	0	0	0	0	0	0	0	0	0	0	0	0	0	0	0	0	0	0	0	0	0	0	0	0	0	0	0
ENSG00000222795	0	0	0	0	0	0	0	0	0	0	0	0	0	0	0	0	0	0	0	0	0	0	0	0	0	0	0	0	0	0	0	0	0	0	0	0	0	0	0	0	0	0	0	0	0	0	0	0
ENSG00000222796	0	0	0	0	0	0	0	0	0	0	0	0	0	0	0	0	0	0	0	0	0	0	0	0	0	0	0	0	0	0	0	0	0	0	0	0	0	0	0	0	0	0	0	0	0	0	0	0
ENSG00000222800	0	0	0	0	0.09412793	0	0	0	0	0	0	0	0	0	0	0	0.25384937	0	0.14983014	0.1420457	0.14411899	0	0.15688181	0	0	0	0.14518915	0	0.12117365	0	0	0.20645189	0	0	0	0	0	0	0	0	0.14406462	0	0	0	0	0	0	0.15775967
ENSG00000222805	0	0	0	0	0	0	0	0	0	0	0	0	0	0	0	0	0	0	0	0	0	0	0	0	0	0	0	0	0	0	0	0	0	0	0	0	0	0	0	0	0	0	0	0	0	0	0	0
ENSG00000222806	0	0	0	0	0	0	0	0	0	0	0	0	0	0	0	0	0	0	0	0	0	0	0	0	0	0	0	0	0	0	0	0	0	0	0	0	0	0	0	0	0	0	0	0	0	0	0	0
ENSG00000222808	0	0	0	0	0	0	0	0	0	0	0	0	0	0	0	0	0	0	0	0	0	0	0	0	0	0	0	0	0	0	0	0	0	0	0	0	0	0	0	0	0	0	0	0	0	0	0	0
ENSG00000222810	0	0	0	0.2181189	0	0.12657764	0	0	0	0	0	0	0	0	0	0	0	0	0	0.14983014	0	0	0	0	0.161084	0	0	0	0	0	0	0.20645189	0	0	0	0	0.31230219	0.17003713	0	0	0	0	0	0	0	0	0	0.15775967
ENSG00000222821	0	0	0	0	0	0	0	0	0	0	0	0	0	0	0	0	0	0	0	0	0	0	0	0	0	0	0	0	0	0	0	0	0	0	0	0	0	0	0	0	0	0	0	0	0	0	0	0
ENSG00000222826	0	0	0	0	0	0	0	0	0	0	0	0	0	0	0	0	0	0	0	0	0	0	0	0	0	0	0	0	0	0	0	0	0	0	0	0	0	0	0	0	0	0	0	0	0	0	0	0
ENSG00000222831	0	0.35390148	0	0.6829623	0	0.39633327	0	0.51752929	0	0	0	0	0	0	0	0	0	0	0	0	0	0	0	0	0	0	0	0	0	0.42501549	0	1.93929403	0	0	0	0	0.97786424	0	0	0	0.60493944	0	0.48737108	0	0	0	0.47049143	0
ENSG00000222832	0	0	0	0	0	0	0	0	0	0	0	0	0	0	0	0	0	0	0	0	0	0	0	0	0	0	0	0	0	0	0	0	0	0	0	0	0	0	0	0	0	0	0	0	0	0	0	0
ENSG00000222833	0	0	0	0	0	0	0	0	0	0	0	0	0	0	0	0	0	0	0	0	0	0	0	0	0	0	0	0	0	0	0	0	0	0	0	0	0	0	0	0	0	0	0	0	0	0	0	0
ENSG00000222834	0	0	0	0	0	0	0	0	0	0	0	0	0	0	0	0	0	0	0	0	0	0	0	0	0	0	0	0	0	0	0	0	0	0	0	0	0	0	0	0	0	0	0	0	0	0	0	0
ENSG00000222835	0	0	0	0	0	0	0	0	0	0	0	0	0	0	0	0	0	0	0	0	0	0	0	0	0	0	0	0	0	0	0	0	0	0	0	0	0	0	0	0	0	0	0	0	0	0	0	0
ENSG00000222838	0	0	0	0	0	0	0	0	0	0	0	0	0	0	0	0	0	0	0	0	0	0	0	0	0	0	0	0	0	0	0	0	0	0	0	0	0	0	0	0	0	0	0	0	0	0	0	0
ENSG00000222842	0	0	0	0	0	0	0	0	0	0	0	0	0	0	0	0	0	0	0	0	0	0	0	0	0	0	0	0	0	0	0	0	0	0	0	0	0	0	0	0	0	0	0	0	0	0	0	0
ENSG00000222844	0	0	0	0	0	0.68423574	0	0	0	0	0	0.2567735	0	0	0	0	0	0	0.66923356	0.26997694	0	0	0	0	0	0	0	0	0	0.21834121	0.24458439	0	0	0	0	0	0	0	0	0.48840033	0.56800528	0.54446417	0	0	0.28046826	0	0	0.28426507
ENSG00000222845	0	0	0	0	0	0	0	0	0	0	0	0	0	0	0	0	0	0	0	0	0	0	0	0	0	0	0	0	0	0	0	0	0	0	0	0	0	0	0	0	0	0	0	0	0	0	0	0
ENSG00000222849	0	0	0	0	0	0	0	0	0	0	0	0	0	0	0	0.29198014	0	0	0	0	0	0	0	0	0	0	0	0	0	0.48217017	0	0	0	0	0	0	0	0	0	0	0	0.60117919	0	0	0.30968371	0	0	0
ENSG00000222852	0	0	0	0	0	0	0	0	0	0	0	0	0	0	0	0	0	0	0	0	0	0	0	0	0	0	0	0	0	0	0	0	0	0	0	0	0	0	0	0	0	0	0	0	0	0	0	0
ENSG00000222854	0	0	0	0	0	0	0	0	0	0	0	0	0	0	0	0	0	0	0	0	0	0	0	0	0	0	0	0	0	0	0	0	0	0	0	0	0	0	0	0	0	0	0	0	0	0	0	0
ENSG00000222858	0	0	0	0	0.39083554	0	0	0	0.3463936	0	0	0.29584772	0	0.37951893	0	0	0	0	0.77107345	0.31106039	0.29489923	0	0.70975182	0	0	0.3344244	0.28830514	0	0	0	0	0.5636075	0	0	0	0	0	0	0	0	0	0	0.65444087	0	0	0	0	0.3275228
ENSG00000222859	0	0	0	0	0	0	0	0	0	0	0	0	0	0	0	0	0	0	0	0	0	0	0	0	0	0	0	0	0	0	0	0	0	0	0	0	0	0	0	0	0	0	0	0	0	0	0	0
ENSG00000222861	0	0	0	0	0	0	0	0	0	0	0	0	0	0	0	0	0	0	0	0	0	0	0	0	0	0	0	0	0	0	0	0	0	0	0	0	0	0	0	0	0	0	0	0	0	0	0	0
ENSG00000222862	0.30990134	0.41515366	0	0.4005837	0.17286957	0	0	0	0	0	0	0.26171145	0	0	0	0	0	0	0.34105172	0	0	0	0.62785738	0	0	0.25503916	0	0.23367298	0.22254008	0.24928793	0	0.37915685	0	0	0	0	0	0	0	0	0	0	0	0	0	0	0	0
ENSG00000222864	0	0	0	0	0	0	0	0	0	0	0	0	0	0	0	0	0	0	0	0	0	0	0	0	0	0	0	0	0	0	0	0	0	0	0	0	0	0	0	0	0	0	0	0	0	0	0	0
ENSG00000222869	0	0	0	0	0	0	0	0	0	0	0	0	0	0	0	0	0	0	0	0	0	0	0	0	0	0	0	0	0	0	0	0	0	0	0	0	0	0	0	0	0	0	0	0	0	0	0	0
ENSG00000222870	0	0	0	0	0	0	0	0	0	0	0	0	0	0	0	0	0	0	0	0	0	0	0	0	0	0	0	0	0	0	0	0	0	0	0	0	0	0	0	0	0	0	0	0	0	0	0	0
ENSG00000222872	0.25579159	0.17133326	0.25015958	0.9919215	0.14268599	0.57562689	0.20541978	0.50109979	0.25292231	0.23167624	0.32225195	0.2160158	0	0	0	0.24675621	0	0	0	0.22712346	0.21532324	0	0.25911574	0.2378129	0	0	0	0.38574588	0	0	0.77727908	0.31295486	0.41963368	0	0	0	0.23670523	0.25775469	0	0.41087647	1.43353714	0	0	0	0	0	0.4555552	0.47828726
ENSG00000222874	0	0	0	0	0	0	0	0	0	0	0	0	0	0	0	0	0	0	0	0	0	0	0	0	0	0	0	0	0	0	0	0	0	0	0	0	0	0	0	0	0	0	0	0	0	0	0	0
ENSG00000222880	0	0	0	0	0	0	0	0	0	0	0	0	0	0	0	0	0	0	0.12758769	0	0	0	0	0	0	0.09541033	0	0	0	0	0	0	0	0	0	0	0	0	0	0	0	0	0.09897965	0	0	0	0	0
ENSG00000222881	1.50606261	3.02635382	1.76748261	0.7787047	1.84825033	1.58162902	1.45137716	2.36032051	2.38266998	0.81844504	1.89737127	2.79811119	3.0593913	0.65263068	1.30981747	4.35859092	4.53132984	2.18001296	0.66297904	0.80236138	1.52134928	4.37340537	7.01791986	3.08045491	0	0.24788853	3.62837187	1.13560703	4.32601273	1.69608987	0	0	1.9765923	0	0	1.07176114	0.278737	0.30352422	0.30340037	2.4191792	1.68809047	0	0.34487202	0	0.55569413	0	0.26822409	0.56321677
ENSG00000222883	0	0	0	0	0	0	0	0	0	0	0	0	0	0	0	0	0	0	0	0	0	0	0	0	0	0	0	0	0	0	0	0	0	0	0	0	0	0	0	0	0	0	0	0	0	0	0	0
ENSG00000222884	0	0	0	0	0	0	0	0	0	0	0	0	0	0	0	0	0	0	0	0	0	0	0	0	0	0	0	0	0	0	0	0	0	0	0	0	0	0	0	0	0	0	0	0	0	0	0	0
ENSG00000222888	0	0	0	0	0	0.21402899	0	0	0	0	0	0	0	0	0	0.33369159	0	0	0	0	0	0	0	0	0	0	0	0	0	0	0	0	0	0	0	0	0	0	0	0	0	0	0	0	0	0	0	0
ENSG00000222889	0	0	0	0	0	0	0	0	0	0	0	0	0	0	0	0	0	0	0	0	0	0	0	0	0	0	0	0	0	0	0	0	0	0	0	0	0	0	0	0	0	0	0	0	0	0	0	0
ENSG00000222890	0	0	0	0	0	0.17454791	0	0	0.30649793	0	0	0	0	0.26485022	0	0	0	0	0	0	0	0	0	0	0	0	0	0	0	0	0	0	0	0	0	0	0	0	0	0	0	0	0.71653021	0	0	0	0	0
ENSG00000222894	10.4642012	9.81272298	8.59639272	12.985154	3.7357787	16.0128935	18.151639	13.9396851	15.1063597	15.5433696	17.4016051	14.4926963	13.8169263	25.3932666	21.4776043	17.7664468	20.779384	12.6223694	22.1107814	21.9277377	14.0938851	23.5943382	23.7444245	11.2853029	14.7841644	23.0793878	20.888382	12.3088003	21.9418737	14.4781252	6.99551173	5.12107947	11.6734461	5.27013252	11.4126637	10.0529803	17.8174483	10.1227297	12.2266407	14.1192095	9.38315221	6.74569895	15.3356076	20.0118856	27.0269416	12.072999	20.1272569	30.9147492
ENSG00000222895	0	0	0	0	0	0	0	0	0	0	0	0	0	0	0	0	0	0	0	0	0	0	0	0	0	0	0	0	0	0	0	0	0	0	0	0	0	0	0	0	0	0	0	0	0	0	0	0
ENSG00000222898	0.19357201	0.2593152	0.09465497	0	0	0	1.39907528	0.47401332	0.52635183	1.40258048	1.09739852	0.73562137	1.31073021	0.52426038	1.34679129	0.7469377	1.01920902	0.70048464	0.74560255	1.46095631	1.30357856	1.32260554	3.72566421	1.79966517	2.77180585	2.8674673	4.91332295	3.13809485	1.39004013	1.94639228	0.4411584	0.2368307	0.31756063	0.30465481	0.3769949	0.17218985	2.05997526	1.17034562	0.97489007	1.24373417	2.53129081	1.21319043	1.32977679	0.90894826	0.80350367	0.79761555	1.37897789	0.72389423
ENSG00000222899	0	0	0	0	0	0	0	0	0	0	0	0	0	0	0	0	0	0	0	0	0	0	0	0	0	0	0	0	0	0	0	0	0	0	0	0	0	0	0	0	0	0	0	0	0	0	0	0
ENSG00000222909	0	0	0	0	0	0	0	0	0	0	0	0	0	0	0	0	0	0	0	0	0	0	0	0	0	0	0	0	0	0	0	0	0	0	0	0	0	0	0	0	0	0	0	0	0	0	0	0
ENSG00000222915	0	0.20757683	0.30307795	0	0.17286957	0.69739412	0	0	0.15321255	0.28068468	0	0	0.78691074	0.67145657	0	0.59790927	2.33102064	0.37381632	0.34105172	0.82550642	0	0	0.28811947	0	0	0.25503916	0	0.23367298	0	0	1.41255525	0	2.03360939	0.97548126	0	1.65401599	0	0	0	0	0	0.57892846	0	0	0.85758565	0.85130121	0.27596132	1.15892682
ENSG00000222920	0	0	0	0	0	0	0	0	0	0	0	0	0	0.25735446	0	0	0	0	0	0	0	0	0	0	0	0.2502271	0	0	0	0	0	0	0	0.47853798	0	0	0	0	0	0	0	0	0	0	0	0	0	0
ENSG00000222921	0	0.54884722	0	0	0	0	0	0	0	0	0	0	0	0	0	0	0	0	0.60117591	0	0	0	0	0	0	0	0	0	0	0	0	0	0	0	0	0	0	0	0	0	0	0	0	0	0	0	0	0
ENSG00000222922	0	0	0	0	0	0	0	0	0	0	0	0	0	0	0	0	0	0	0	0	0	0	0	0	0	0	0	0	0	0	0	0	0	0	0	0	0	0	0	0	0	0	0	0	0	0	0	0
ENSG00000222923	0	0	0	0	0	0	0	0	0	0	0	0	0	0	0	0	0	0	0	0	0	0	0	0	0	0	0	0	0	0	0	0	0	0	0	0	0	0	0	0	0	0	0	0	0	0	0	0
ENSG00000222924	0	0	0	0	0	0	0	0	0	0	0	0	0	0	0	0	0	0	0	0	0	0	0	0	0	0	0	0	0	0	0	0	0	0	0	0	0	0	0	0	0	0	0	0	0	0	0	0
ENSG00000222931	0	0	0	0	0	0	0	0	0	0	0	0	0	0.17771708	0	0	0	0	0.11553543	0	0	0.08966361	0	0	0.30065516	0	0	0	0	0.07538817	0	0	0	0	0	0	0	0	0	0	0	0	0.26888934	0	0	0	0	0
ENSG00000222932	0	0	0	0	0	0	0	0	0.34266893	0	0	0.29266657	0	0	0	0	0	0	0	0	0.29172827	0	0	0	0	0	0	0	0	0	0	0	0	0	0	0	0	0	0	0	0	0	0	0	0	0.47599638	0	0
ENSG00000222934	0	0	0	0	0	0	0	0	0	0	0	0	0	0	0	0	0	0	0	0	0	0	0	0	0	0	0	0	0	0	0	0	0	0	0	0	0	0	0	0	0	0	0	0	0	0	0	0
ENSG00000222937	4.60424854	3.08399865	2.70172343	1.7854587	1.54100872	4.14451361	10.3531571	6.31385737	6.82890234	7.50631018	4.64042802	8.1653972	9.74270445	8.47953722	9.61031787	6.66241755	13.8529227	7.21999529	4.56034867	5.7235112	9.30196414	5.50534558	11.6602085	8.13320108	3.95576291	2.27349193	4.35774866	2.43019903	1.98378584	2.22222387	4.89685821	3.37991245	4.53204378	0.72464322	4.48354647	2.86696105	4.26069417	3.71166755	4.63769131	2.95831057	7.74110058	2.47342295	3.69013058	3.53781549	2.54825449	6.95634704	1.22999903	9.03962919
ENSG00000222939	0	0	0	0	0	0	0	0	0	0	0	0	0	0	0	0	0	0	0	0	0	0	0	0	0	0	0	0	0	0	0	0	0	0	0	0	0	0	0	0	0	0	0	0	0	0	0	0
ENSG00000222940	0	0	0	0	0	0	0	0	0	0	0	0	0	0	0	0	0	0	0	0	0	0	0	0	0	0	0	0	0	0	0	0	0	0	0	0	0	0	0	0	0	0	0	0	0	0	0	0
ENSG00000222941	0	0	0	0	0	0	0	0	0	0	0	0	0	0	0	0	0	0	0	0	0	0	0	0	0	0	0	0	0	0	0	0	0	0	0	0	0	0	0	0	0	0	0	0	0	0	0	0
ENSG00000222942	0	0	0	0	0	0.07926665	0	0	0.10350586	0	0	0	0	0	0	0	0	0	0	0	0	0	0.10704454	0	0	0	0	0	0	0	0	0	0	0	0	0	0.19557285	0.21296453	0	0	0	0	0.12098789	0	0	0	0	0
ENSG00000222945	0	0	0	0	0	0	0	0	0	0	0	0	0	0	0	0	0	0	0	0	0	0	0	0	0	0	0	0	0	0	0	0	0	0	0	0	0	0	0	0	0	0	0	0	0	0	0	0
ENSG00000222950	0	0	0	0	0	0	0	0	0	0	0	0	0	0	0	0	0	0	0	0	0	0	0	0	0.10871747	0.09372464	0	0	0	0	0	0	0	0	0	0	0	0	0	0	0	0	0	0	0	0.10141335	0	0
ENSG00000222952	0	0	0	0	0	0	0	0	0	0	0	0	0	0	0	0	0	0	0	0	0	0	0	0	0	0	0	0	0	0	0	0	0	0	0	0	0	0	0	0	0	0	0	0	0	0	0	0
ENSG00000222954	0	0	0	0	0	0	0	0	0	0	0	0	0	0	0	0	0	0	0	0	0	0	0	0	0	0	0	0	0	0	0	0	0	0	0	0	0	0	0	0	0	0	0	0	0	0	0	0
ENSG00000222955	0	0	0	0	0	0	0	0	0	0	0	0	0	0	0	0	0	0	0	0	0	0	0	0	0	0	0	0	0	0	0	0	0	0	0	0	0	0	0	0	0	0	0	0	0	0	0	0
ENSG00000222958	0.80574349	0.80954965	0	0.5207588	0.44946088	0.30220412	0.64707232	1.97308043	0.59752895	1.09467023	0.50754682	0	0	0.87289354	0	0	0	0	0.35771945	0.33913411	0.3440841	0.4081073	0	0.38458806	0	0	0.3466391	0.30377488	0	0.32407431	0	0	0	0	0	0.74562148	0.40596364	0.40579799	0	0	1.505214	0	0.46126632	0.34395428	0	0	0	0.37665122
ENSG00000222960	0	0	0.30307795	0	0	0	0	0	0.15321255	0	0	0	0	0	0	0	0	0	0	0	0	0	0	0	0	0	0	0	0	0	0	0	0	0	0	0	0	0	0	0	0	0	0	0	0	0	0	0
ENSG00000222961	0	0	0	0	0	0	0	0	0	0	0	0	0	0	0	0	0	0	0	0	0	0	0	0	0	0	0	0	0	0	0	0	0	0	0	0	0	0	0	0	0	0	0	0	0	0	0	0
ENSG00000222966	0	0	0	0	0	0	0	0	0	0	0	0	0	0	0	0	0	0	0	0	0	0	0	0	0	0	0	0	0	0	0	0	0	0	0	0	0	0	0	0	0	0	0	0	0	0	0	0
ENSG00000222969	3.78114265	4.16081382	3.43375463	5.2366804	2.61139279	3.44411396	2.24125607	2.91001806	2.18092505	2.03849206	3.17571192	2.43289302	1.82879815	2.43825011	2.50548324	0.95531872	2.16693762	1.6289203	2.27875896	1.51880884	0.98519406	1.46091573	1.45915458	1.42289169	0.85941466	1.92632929	1.47176377	1.15400513	1.42226731	2.10014639	3.96672872	2.53336082	3.39692295	2.26704024	2.10401063	1.36140609	1.83281258	2.35867142	1.8136223	2.31376246	2.01816402	1.77331068	1.03076273	3.22817428	1.9930482	1.60748497	2.40502604	2.27253248
ENSG00000222971	0	0	0	0	0	0	0	0	0	0	0.38670234	0	0	0	0	0	0	0	0	0	0	0	0	0	0	0	0	0	0.22042065	0	0	0	0	0	0	0	0	0	0	0	0	0	0	0	0	0	0	0
ENSG00000222972	0	0	0	0	0	0	0	0	0.15175339	0	0.38670234	0	0	0	0	0	0	0	0	0	0	0	0	0	0	0	0	0	0	0	0.24691376	0	0	0	0.5978062	0	0	0	0	0	0	0	0	0	0	0	0	0
ENSG00000222973	0	0	0	0	0	0	0	0	0	0	0	0	0	0	0	0	0	0	0	0	0	0	0	0	0	0	0	0	0	0	0	0	0	0	0	0	0	0	0	0	0	0	0	0	0	0	0	0
ENSG00000222974	0	0	0.47939326	0.3168114	0.27343627	0.18385041	0	0	0.06058595	0	0	0.31117383	0.26551895	0.1065783	0	0	0.36870897	0	0	0.217624	0	0.10466436	0	0.11393318	0	0.10085199	0.31632465	0	0	0	0.08800064	0.19715548	0.18619233	0.29986549	0	0.10900993	0	0.37046111	0.12343665	0.39369152	0	0.42092744	0.10462488	0.11304044	0	0	0.10912539	0.11457071
ENSG00000222976	0.113485	0.07601405	0	0.1466926	0.06330435	0	0	0	0	0	0	0	0	0	0	0	0	0	0.12489218	0	0	0	0	0	0	0	0	0	0	0	0	0	0	0	0	0	0	0	0.11430929	0	0	0	0	0	0	0	0	0
ENSG00000222978	0	0.90896802	0.3317906	0	0	0	0	0	0	0	0	0	0	0	0	0	0	0	0	0	0	0	0	0	0	0	0	0	0	0	0	0	0	0	0	0	0	0	0	0	0	0	0	0	0	0	0	0
ENSG00000222979	0	0	0	0	0	0	0	0	0	0	0	0	0	0	0	0	0	0	0	0	0	0	0	0	0	0	0	0	0	0	0	0	0	0	0	0	0	0	0	0	0	0	0	0	0.10431451	0	0	0
ENSG00000222982	0	0	0	0	0	0	0	0	0	0	0	0	0	0	0	0	0	0	0	0	0	0	0	0	0	0	0	0	0	0	0	0	0	0	0	0	0	0	0	0	0	0	0	0	0	0	0	0
ENSG00000222983	0	0	0	0	0	0	0	0	0	0	0	0	0	0	0	0	0	0	0	0	0	0	0	0	0	0	0	0	0	0	0	0	0	0	0	0	0	0	0	0	0	0	0	0	0	0	0	0
ENSG00000222985	0	0	0	0	0	0	0	0	0	0	0	0	0	0	0	0	0	0	0	0	0	0	0	0	0	0	0	0	0	0	0	0	0	0	0	0	0	0	0	0	0	0	0	0	0	0	0	0
ENSG00000222986	0	0.18610337	0	0	0	0.41683327	0	0	0.13736298	0	0.35003229	0.23463785	0	0	0	0	0	0	0	0	0	0	0.28145331	0	0	0	0.23906145	0	0	0.22349953	0	0	0.455809	0	0	0	0	0.27997492	0	0	0	0	0	0	0	0	0	0
ENSG00000222987	0	0	0	0	0	0	0	0	0	0	0	0	0	0	0	0	0	0	0	0	0	0	0	0	0	0	0	0	0	0	0	0	0	0	0	0	0	0	0	0	0	0	0.12019969	0	0	0	0	0
ENSG00000222990	0	0	0.18988016	0	0	0	0	0	0	0	0	0	0	0	0	0	0	0	0	0	0	0	0	0	0	0	0	0	0	0	0	0	0	0	0	0	0	0	0	0	0	0	0	0	0	0	0	0
ENSG00000222994	0	0	0	0.4084383	0	0	0	0	0	0	0	0	0	0	0	0	0	0	0	0	0	0	0	0	0	0	0	0	0	0	0	0	0	0	0	0	0	0	0	0	0	0	0	0	0	0	0	0
ENSG00000222997	0	0	0	0	0	0	0	0	0	0	0	0	0	0	0	0	0	0	0	0	0	0	0	0	0	0	0	0	0	0	0	0	0	0	0	0	0	0	0	0	0	0	0	0	0	0	0	0
ENSG00000222998	0	0	0	0	0	0	0	0	0	0	0	0	0	0	0	0	0	0	0	0	0	0	0	0	0.09239353	0	0	0	0	0	0	0	0	0	0	0	0	0	0	0	0	0	0	0	0	0	0.08618612	0.09048678
ENSG00000222999	0	0	0	0	0	0	1.32732783	0	0	0	0	0.3489486	0	0	0	0	0	0	0	0	0	0	0	0	0	0	0	0	0	0.33238391	0	0	0	0	0	0	0	0	0	0	0	0	0	0.35277362	0	0	0	0.77261788
ENSG00000223001	1.51867884	0.67815677	1.48524063	1.9630697	2.3531983	0.75946584	8.5372892	13.2227379	8.84301141	10.2398472	19.982995	13.3952415	2.71367475	17.7320781	16.1429859	3.25563161	14.7232634	17.3012372	16.1562091	4.64473422	8.23865061	14.9883754	30.7682988	18.668935	8.05420023	14.5812964	14.5189151	37.2800166	18.6607429	12.3521519	27.1762812	6.81291253	5.25970177	9.29516174	4.6009168	9.60657106	5.62143943	16.153527	9.85812917	17.0761121	24.903017	14.201678	16.2288465	5.76258486	12.607856	15.2966794	12.9225029	4.41727081
ENSG00000223003	0	0	0	0	0	0	0	0	0	0	0	0	0	0	0	0	0	0	0	0	0	0	0	0	0	0	0	0	0	0	0	0	0	0	0	0	0	0	0	0	0	0	0	0	0	0	0	0
ENSG00000223004	0	0	0	0	0	0	0	0	0	0	0	0	0	0	0	0	0	0	0	0	0	0	0	0	0	0	0	0	0	0	0	0	0	0	0	0	0	0	0	0	0	0	0	0	0	0	0	0
ENSG00000223006	0	0	0	0	0	0.07875026	0	0	0.05190262	0	0	0.08865795	0.08885854	0	0.09130324	0	0	0	0.3466063	0.27965038	0	0.08966361	0	0	0	0	0.17279526	0	0	0.16889867	0	0.25688803	0	0	0	0	0	0	0	0	0	0	0	0.17925956	0.19367841	0.14419434	0.09348527	0.29445046
ENSG00000223007	0	0	0	0	0	0	0	0	0	0	0	0	0	0	0	0	0	0	0	0	0	0	0	0	0	0	0	0	0	0	0	0	0	0	0	0	0	0	0	0	0	0	0	0	0	0	0	0
ENSG00000223009	0	0	0	0	0	0	0	0	0	0	0	0	0	0	0	0	0	0	0	0	0	0	0	0	0	0	0	0	0	0	0	0	0	0	0	0	0	0	0	0	0	0	0	0	0	0	0	0
ENSG00000223012	0	0	0.21813223	0	0	0.33462048	0.08956018	0.10923629	0.27567657	0	0.28099478	0.0941799	0	0.24163143	0.09698994	0.10758229	0	0.26904428	0	0	0.09387795	0	0	0.10368313	0.10646036	0.0917788	0.09595546	0.08408993	0	0.08970915	0.16944146	0	0.18295448	0	0	0	0	0.11237748	0.11233162	0	0	0.19969966	0.25537236	0.09521226	0.20574142	0.1531753	0.19861576	0
ENSG00000223013	0	0	0	0.4005837	0.17286957	0	0	0	0.30642511	0	0	0	0	0	0	0	0	0	0.34105172	0	0	0	0	0	0	0	0	0	0	0	0	0.37915685	0	0.48774063	0	0	0	0	0	0	0	0	0	0.35482025	0.26458022	0	0	0
ENSG00000223015	0	0	0	0	0	0	0	0	0	0	0	0	0	0	0	0	0	0	0	0	0	0	0	0	0	0	0	0	0	0	0	0	0	0	0	0	0	0	0	0	0	0	0	0	0	0	0	0
ENSG00000223018	0	0	0	0	0	0	0	0	0	0	0	0	0	0	0	0	0	0	0	0	0	0	0	0	0	0	0	0	0	0	0	0	0	0	0	0	0	0	0	0	0	0	0	0	0	0	0	0
ENSG00000223019	0	0	0	0	0	0	0	0	0	0	0	0	0	0	0	0	0	0	0	0	0	0	0	0	0	0	0	0	0	0	0	0	0	0	0	0	0	0	0	0	0	0	0	0	0	0	0	0
ENSG00000223023	0.29842352	0	0.29185284	0	0	0	0	0.29230821	0.14753801	0.27028895	0	0.25201843	0	0	0	0	0	0	0	0	0	0	0	0	0	0.28488005	0	0	0	0	0	0	0.48957263	0	0	0	0	0	0	0	0	0	0	0	0	0	0.79722159	0.2790009
ENSG00000223024	0	0	0	0	0	0	0	0	0	0	0	0	0	0	0	0	0	0	0	0	0	0	0	0	0	0	0	0	0	0	0	0	0	0	0	0	0	0	0	0	0	0	0	0	0	0	0	0
ENSG00000223026	0	0	0	0	0	0	0	0	0	0	0	0	0	0	0	0	0	0	0	0	0	0	0	0.1060019	0.09728709	0	0	0	0	0	0	0	0	0	0	0	0.09683396	0	0	0	0	0	0	0	0	0	0	0
ENSG00000223027	0	0	0	0	0	0	0	0	0	0	0	0	0	0	0	0	0	0	0	0	0	0	0	0	0	0	0	0	0	0	0	0	0	0	0	0	0	0	0	0	0	0	0	0	0	0	0	0
ENSG00000223029	0	0	0	0	0	0	0	0	0	0	0	0	0	0	0	0	0	0	0	0	0	0	0	0	0	0	0	0	0	0	0	0	0	0	0	0	0	0	0	0	0	0	0	0	0	0	0	0
ENSG00000223030	0	0.22028562	0.64326748	0	0.18345342	0	0	0	0	0	0	0	0	0	0	0	0	0	0	0	0	0	0	0	0	0	0	0	0	0	0	0	0	0	0	0	0	0	0	0	0	0	0	0	0	0	0	0

ENSG00000223036	0	0	0	0	0	0	0	0	0	0	0	0.22128448	0	0	0	0	0	[illegible]
ENSG00000223037	0	0	0	0	0	0	0	0	0	0	0	0	0	0	0	0	0	[illegible]
ENSG00000223039	0.50996424	0.54653141	0.29924152	1.3183767	0.34136269	0.22952211	0.08190789	0.39961123	0.05042438	0.64664064	0.25698573	0.60293017	0.17265552	0.11049285	0.88702828	0.19678027	0.15343427	[illegible]
ENSG00000223040	0	0.07737631	0.22595059	0.298643	0.19331651	0.08665351	0	0.11315157	0.11422298	0.10462798	0	0.09755552	0	0.25029205	0.10046629	0.11143829	0	[illegible]
ENSG00000223042	0	0	0	0	0	0	0	0	0	0	0	0	0	0	0	0	0	[illegible]
ENSG00000223044	0	1.04796071	0.30602045	0.4044728	0	0.93888658	0.50258044	0.30649793	0.46410016	0.28340977	1.57684447	1.05700935	0	0.67797556	0	1.20742842	1.88292153	[illegible]
ENSG00000223046	0	0	0	0	0	0	0	0	0	0	0	0	0	0	0	0	0	[illegible]
ENSG00000223047	0	0	0	0	0	0	0	0	0	0	0	0	0	0	0	0	0	[illegible]
ENSG00000223052	0	0	0	0	0	0	0	0.25459102	0	0.23541295	0	0	0	0	0	0	0	[illegible]
ENSG00000223053	0	0	0	0	0	0	0	0	0	0	0	0	0	0	0	0	0	[illegible]
ENSG00000223056	0	0	0	0	0	0	0	0	0	0	0	0	0	0	0	0	0	[illegible]
ENSG00000223059	0	0	0	0	0	0.3899408	0	0	0	0	0	0	0	0	0	0	0	[illegible]
ENSG00000223060	0	0	0	0	0	0	0	0	0	0	0	0	0	0	0	0	0	[illegible]
ENSG00000223062	0.6081083	0	0.89207849	0	0	0	0	0	0	0	0.3830542	0	0.25735446	0	0.26443485	0	0	[illegible]
ENSG00000223063	0	0	0	0	0	0	0	0	0	0	0	0	0	0	0	0	0	[illegible]
ENSG00000223064	0	0	0	0	0	0	0	0	0	0	0	0	0	0	0	0	0	[illegible]
ENSG00000223070	0	0	0	0	0	0	0	0	0	0	0	0	0	0	0	0	0	[illegible]
ENSG00000223075	0	0	0	0	0.05992812	0.08058776	0	0	0	0	0	0	0	0	0	0	0	[illegible]
ENSG00000223076	0	0	0	0	0	0	0	0	0	0	0	0	0	0	0	0	0	[illegible]
ENSG00000223078	0	0	0	0	0	0	0	0	0	0	0	0	0	0	0	0	0.27865074	[illegible]
ENSG00000223080	0	0	0	0	0	0	0	0	0	0	0	0	0	0	0	0	0	[illegible]
ENSG00000223082	0	0	0	0	0.22473044	0.30220412	0	0.39461609	0	0	0	0	0	0	0	0	0	[illegible]
ENSG00000223086	0	0.50204629	0.48868382	0.3229512	0.13936771	0	0.40128516	0.48944631	0.86464138	0.67886526	1.25903086	0.8439687	1.26881732	1.08265865	1.52101284	0.24101769	0.75170898	[illegible]
ENSG00000223087	0	0	0	0	0	0	0	0	0	0	0	0	0	0	0	0	0	[illegible]
ENSG00000223088	0	0	0	0	0	0	0	0	0	0	0	0	0	0	0	0	0	[illegible]
ENSG00000223091	0	0	0	0	0	0	0	0	0	0	0	0	0	0	0	0	0	[illegible]
ENSG00000223092	0	0	0	0	0	0	0	0	0	0	0	0	0	0	0.26196349	0	0	[illegible]
ENSG00000223096	0	0	0	0	0.34573913	0	0	0	0	0.28068468	0	0	0	0	0	0	0	[illegible]
ENSG00000223099	0	0	0	0	0	0	0	0	0	0	0	0	0	0	0	0	0	[illegible]
ENSG00000223101	0	0	0	0	0	0	0	0	0	0	0	0	0	0	0	0	0	[illegible]
ENSG00000223107	0.19772846	0	0	0	0	0.14832104	0	0	0	0.17908715	0.24910273	0	0.16735934	0	0	0.38148812	0	[illegible]
ENSG00000223109	0	0	0	0	0	0	0	0	0	0	0	0	0	0	0.45950973	0	0	[illegible]
ENSG00000223111	0	0	0	0	0	0	0	0	0	0	0	0	0	0	0	0	0	[illegible]
ENSG00000223113	0	0	0	0	0	0	0	0	0	0	0	0	0	0	0	0	0	[illegible]
ENSG00000223116	0	0	0	0	0	0	0	0	0	0	0	0	0	0	0	0	0	[illegible]
ENSG00000223117	0.37806146	1.01292624	0.73947464	0.4886886	0.21089073	0.42538996	0.37951455	0.37031422	0.37382066	0.17120942	0.23814513	0.15963631	0.39999373	0.40956882	0.41099844	0.36470712	0.42655627	[illegible]
ENSG00000223118	0.51158317	0.82239964	0.30019149	0.9257934	0.34244638	0.30700101	0.32867165	0.20043992	0.05058446	0.27801149	0.77340467	0.34562528	0.34640727	0.11084362	0.26695327	0.19740496	0.46176409	[illegible]
ENSG00000223119	0	0	0	0	0	0	0	0	0	0	0	0	0	0	0	0	0	[illegible]
ENSG00000223120	0	0	0	0	0	0.07650737	0.08190789	0.09990281	0.05042438	0	0	0	0	0	0	0	0	[illegible]
ENSG00000223123	0	0	0	0	0	0	0	0	0	0	0	0	0	0	0	0	0	[illegible]
ENSG00000223125	0	0	0	0	0	0	0	0	0	0	0	0	0	0	0	0	0	[illegible]
ENSG00000223126	0	0	0	0	0	0	0	0	0	0	0	0	0	0	0	0	0	[illegible]
ENSG00000223128	0	0	0	0	0	0	0	0	0	0	0	0	0	0	0	0	0	[illegible]
ENSG00000223131	0.61980269	0.41515366	0.30307795	0	0.69147827	1.16232353	0.24887397	0.30355083	1.22570042	0	3.12336502	1.83198014	4.45916088	5.70738082	2.69520133	1.19581854	0.46620413	[illegible]
ENSG00000223134	0	0	0	0	0	0	0	0	0	0	0	0	0	0	0	0	0	[illegible]
ENSG00000223136	0.21273756	0.35623747	0.31208026	0.1374941	0.11866954	0.31915946	0.17084418	0	0.10517561	0.09634061	0	0.08982835	0	0	0.09250856	0.20522298	0	[illegible]
ENSG00000223138	0	0	0	0	0	0.23702284	0.25375385	0	0.15621672	0	0	0	0.26744679	0.34231119	0	0	0.47534539	[illegible]
ENSG00000223142	0	0	0	0	0	0	0	0	0	0	0	0	0	0	0	0	0	[illegible]
ENSG00000223145	0.10135138	0	0	0	0	0	0	0	0	0	0	0	0	0	0	0	0	[illegible]
ENSG00000223148	0	0.21374248	0	0	0	0	0	0	0	0	0	0	0	0	0	0	0	[illegible]
ENSG00000223149	0	0	0	0	0	0	0	0	0	0	0	0	0	0	0	0	0	[illegible]
ENSG00000223152	0	0	0	0	0	0	0	0	0	0	0	0	0	0	0	0	0	[illegible]
ENSG00000223156	0	0	0	0	0	0	0.15977094	0.19487214	0	0	0	0	0	0	0	0	0	[illegible]
ENSG00000223158	0	0	0	0	0	0	0	0	0	0	0	0	0	0	0	0	0	[illegible]
ENSG00000223162	0	0	0	0	0	0	0	0	0	0	0	0	0	0	0	0	0	[illegible]
ENSG00000223164	0	0	0	0	0	0	0	0	0	0	0	0	0	0	0	0	0	[illegible]
ENSG00000223168	0	0	0	0	0	0	0	0	0	0	0	0	0	0	0	0	0	[illegible]
ENSG00000223169	0	0	0	0	0	0	0	0	0	0	0	0	0	0	0	0	0	[illegible]
ENSG00000223174	0	0	0	0	0	0	0	0	0	0	0	0	0	0.09947505	0	0	0	[illegible]
ENSG00000223175	0	0	0	0	0	0	0	0	0	0	0	0	0	0	0	0	0	[illegible]
ENSG00000223177	0	0	0	0	0	0	0	0	0	0	0	0	0	0	0	0	0	[illegible]
ENSG00000223179	0	0	0	0	0	0	0	0	0	0	0	0	0	0	0	0	0	[illegible]
ENSG00000223180	0	0	0	0	0	0	0	0	0	0	0	0	0	0	0	0	0	[illegible]
ENSG00000223181	0	0	0.30307795	0	0	0	0.7466219	0.30355083	0.45963766	0	0	0	0	0	0.26952013	0	1.39861238	[illegible]
ENSG00000223182	0	0	0	0	0	0	0	0	0	0	0	0	0	0	0	0	0	[illegible]
ENSG00000223187	0	0.2225566	0	0	0	0.24924051	0.53366789	0	0	0	0	0.28059784	0	0.3599561	0	0	0	[illegible]
ENSG00000223188	0	0.20175692	0	0	0	0.225947	0.48379239	0	0.14891687	0	0.37947425	1.27186872	0	0.32631534	0.78589048	0	0.45313298	[illegible]
ENSG00000223189	0	0.41515366	0	0	0.5186087	0.46492941	0	0	0.15321255	0.28068468	0	0	0	0.33572828	0	0.29895463	0.46620413	[illegible]
ENSG00000223190	0	0	0	0	0	0	0	0	0	0	0	0	0	0	0	0	0	[illegible]
ENSG00000223191	0	0	0	0	0	0	0	0	0	0	0	0	0	0	0	0	0	[illegible]
ENSG00000223192	0	0	0.41473825	0	0	0	0	0	0	0	0	0	0	0	0	0	0	[illegible]
ENSG00000223197	0	0	0	0	0	0	0	0	0	0	0	0	0	0	0	0	0	[illegible]
ENSG00000223198	0	0.50915072	0.2973595	0	0	0	0.24417823	0	0.15032175	0	0.3830542	0	0.38603169	0	0	0	0	[illegible]
ENSG00000223200	0	0	0	0	0	0	0	0	0	0	0	0	0	0	0	0	0	[illegible]
ENSG00000223201	0	0	0	0	0	0	0	0	0	0	0	0	0	0	0	0	0	[illegible]
ENSG00000223202	0.11428986	0.07655316	0.22354686	0.295466	0	0.25719499	0.18356661	0	0.11300784	0	0.43195474	0	0	0	0	0	0.17193344	[illegible]
ENSG00000223203	0.62582019	0.41918428	0.61204091	1.6178913	1.3963833	0.93888658	0	0	0.15470005	0	0.39421112	0.79275701	1.32425109	0.67797556	0.27213683	0	0	[illegible]
ENSG00000223208	0	0	0	0	0	0	0	0	0	0	0	0	0	0	0	0	0	[illegible]
ENSG00000223211	0	0	0	0	0	0	0	0	0	0	0	0	0	0	0	0	0	[illegible]
ENSG00000223212	0	0	0	0	0	0	0	0	0	0	0	0	0	0	0	0	0	[illegible]
ENSG00000223213	0	0	0	0	0	0	0	0	0	0	0	0	0	0	0	0	0	[illegible]
ENSG00000223214	0	0	0	0	0	0	0	0	0	0	0	0	0	0	0	0	0	[illegible]
ENSG00000223215	0	0	0	0	0	0	0	0	0	0	0	0	0	0	0	0	0	[illegible]
ENSG00000223217	0	0	0	0	0	0	0	0	0	0	0	0	0	0	0	0	0	[illegible]
ENSG00000223220	0	0	0	0	0	0	0	0	0	0	0	0	0	0	0	0	0	[illegible]
ENSG00000223223	0	0	0	0	0	0	0	0	0	0	0	0	0	0	0	0	0	[illegible]
ENSG00000223224	2.2485865	1.50613888	1.0995386	1.4532803	1.46336099	0.56224022	0.30096387	0.36708473	0.37056059	0.33943263	0.47213657	0.63297653	1.58602165	1.21799098	1.62965662	0.36152653	2.81890868	[illegible]
ENSG00000223225	0	0.20757683	0	0	0.17286957	0	0	0	0.15321255	0.28068468	0	0	0.26230358	0.33572828	0.26952013	0	0.46620413	[illegible]
ENSG00000223229	0	0	0	0	0	0	0	0	0	0	0	0	0	0	0	0	0	[illegible]
ENSG00000223231	0	0	0	0	0	0	0	0	0	0	0	0	0	0	0	0	0	[illegible]
ENSG00000223235	0	0	0	0	0	0	0	0	0	0	0	0	0	0	0	0	0	[illegible]
ENSG00000223238	0	0.43612102	0	0.4208152	0.36320071	0	0	1.27552674	0.64380224	0.29486067	0	0	0	0	0	0.31405335	0.48974979	[illegible]
ENSG00000223243	0	0	0	0	0	0	0	0	0	0	0	0	0	0	0	0	0	[illegible]
ENSG00000223244	0	0	0	0	0	0	0	0	0	0	0	0	0	0	0	0	0	[illegible]
ENSG00000223245	0	0	0	0	0	0	0	0	0	0	0	0	0	0	0	0	0	[illegible]
ENSG00000223247	4.55603651	1.24328741	2.14534757	4.3623772	4.7063966	1.89866461	22.9016171	39.3376454	21.77383	19.5626932	48.6819772	29.4980318	5.28452451	34.9157415	43.4393076	7.16238955	36.3004596	[illegible]
ENSG00000223249	0.33572646	0	0	0	0	0	0	0.26961347	0.32884674	0	0.30407507	0	0	0.28416221	0	0.58396029	0	[illegible]
ENSG00000223253	0	0	0	0	0	0	0	0	0	0	0	0	0	0	0	0	0	[illegible]
ENSG00000223254	0	0	0	0	0	0	0	0	0	0	0	0	0	0	0	0	0	[illegible]
ENSG00000223255	1.04387821	1.31101157	0.89328237	2.8673358	0.87344624	1.85971764	0.62873423	0.89467615	0.70961603	1.18183021	0.65755053	1.10194294	0.22088723	0.84815566	1.47526809	0.50350254	0.7851859	[illegible]
ENSG00000223256	0	0	0	0	0	0	0	0	0	0	0	0	0	0	0	0	0	[illegible]
ENSG00000223258	0	0	0	0	0	0	0	0	0	0	0	0	0	0	0	0	0	[illegible]
ENSG00000223259	0	0.18141169	0.26487485	0	0	0	0	0	0	0	0	0	0	0	0.23554701	0	0	[illegible]
ENSG00000223260	0	0	0	0	0	0	0	0	0	0	0	0	0	0	0	0	0	[illegible]
ENSG00000223262	0	0.18936834	0	0	0	0.21207306	0	0	0	0	0	0	0	0	0	0.27273054	0	[illegible]
ENSG00000223263	0	0	0	0	0	0	0	0	0	0	0	0	0	0	0	0	0	[illegible]
ENSG00000223265	0	0	0	0	0	0	0	0	0	0	0	0	0	0	0	0	0	[illegible]
ENSG00000223269	0.20463327	0.20559991	0.50031915	0	0.17122319	0.30700101	0	0	0	0	0	0	0	0	0	0	0	[illegible]
ENSG00000223271	0	0	0	0	0	0	0	0	0	0	0	0	0	0	0	0	0	[illegible]
ENSG00000223273	0	0	0	0	0	0	0	0	0	0	0	0	0	0	0	0	0	[illegible]
ENSG00000223274	0	0	0	0	0	0	0	0	0	0	0	0	0	0	0	0	0	[illegible]
ENSG00000223275	0	0	0	0	0	0	0	0	0	0	0	0	0	0	0	0	0	[illegible]
ENSG00000223280	0	0	0	0	0	0	0	0	0	0	0	0	0	0	0	0	0	[illegible]
ENSG00000223281	0	0	0	0	0	0	0	0	0	0	0	0	0	0	0	0	0	[illegible]
ENSG00000223282	0	0	0	0	0	0	0	0	0	0	0	0	0	0	0	0	0	[illegible]
ENSG00000223284	0.59137137	1.38638472	1.15670116	0.7644166	0.49481931	0.88720475	0.94983092	1.44813242	0.58473782	1.33904616	1.11753427	0.99882535	0.25027131	0.96098371	0.51431365	0	0	[illegible]
ENSG00000223286	0	0.24256169	0.3541585	0	0.20200489	0.27164415	0	0	0.35806979	0	0	0	0.6130241	0	0	0	0	[illegible]
ENSG00000223287	0	0	0	0	0	0	0	0	0	0	0	0	0	0	0	0	0	[illegible]
ENSG00000223290	0	0	0	0	0	0	0	0	0	0	0	0	0	0	0	0	0	[illegible]
ENSG00000223291	0	0	0	0	0.18534469	0.24924051	0	0	0	0	0	0	0.56246541	0.7199122	1.44485019	0.32052868	0.49984772	[illegible]
ENSG00000223293	0	0	0	0	0	0	0	0	0	0	0	0	0	0	0	0	0	[illegible]
ENSG00000223294	0	0	0	0	0	0	0	0	0	0	0	0	0	0	0	0	0	[illegible]
ENSG00000223297	0	0	0	0	0	0	0	0	0	0	0	0	0	0	0	0	0	[illegible]
ENSG00000223298	0	0	0	0	0	0	0	0	0	0	0	0	0	0	0	0	0	[illegible]
ENSG00000223299	0	0	0	0	0	0	0	0	0	0	0	0	0	0	0	0	0	[illegible]
ENSG00000223300	0	0	0	0	0	0	0	0	0	0	0	0	0	0	0	0	0	[illegible]
ENSG00000223302	0	0	0	0	0	0	0	0.29230821	0.14753801	0	1.12788181	0.25201843	0	0	0	0	0	[illegible]
ENSG00000223305	0.38598491	0.25853881	0.47185788	0.1247326	0.5382765	0.7238422	0.38746845	0.18903765	0.71560354	0.96138703	0.6078405	0.48894594	0.65340293	0.52269074	0.67137949	0.65161369	1.0161575	[illegible]
ENSG00000223306	0	0	0	0	0	0	0.47931283	0	0	0	0	0	0.25258863	0	0	0	0.44893731	[illegible]
ENSG00000223307	0	0	0	0	0	0	0	0	0	0	0	0	0	0	0	0	0	[illegible]
ENSG00000223308	0	0	0	0	0	0	0	0	0	0	0	0	0	0	0	0	0	[illegible]
ENSG00000223309	0	0	0	0	0	0	0	0	0	0	0	0	0	0	0	0	0	[illegible]
ENSG00000223313	0	0	0	0	0	0	0	0	0	0	0	0	0	0	0	0	0	[illegible]
ENSG00000223315	0	0	0	0	0	0	0	0	0	0	0	0	0	0	0	0	0	[illegible]
ENSG00000223316	0	0	0	0	0	0	0	0	0	0	0	0	0	0	0	0	0	[illegible]
ENSG00000223318	0	0	0	0	0	0	0.25626626	0	0	0	0	0	0	0	0	0	0	[illegible]
ENSG00000223321	0	0	0	0	0	0	0	0	0	0	0	0	0	0	0	0	0	[illegible]
ENSG00000223322	0	0	0	0	0	0	0	0	0	0	0	0	0	0	0	0	0	[illegible]
ENSG00000223324	0.10264248	0.06875156	0.10038251	0	0	0	0	0.10053913	0.05074556	0	0	0.0866815	0.08687762	0	0	0	0	[illegible]
ENSG00000223327	0	0	0	0	0	0	0	0	0	0	0	0	0	0	0	0	0	[illegible]
ENSG00000223330	0	0	0	0	0	0	0	0	0	0	0	0	0	0	0	0	0	[illegible]
ENSG00000223335	0	0	0	0	0	0	0	0	0	0	0	0	0	0	0.52392699	0	0	[illegible]
ENSG00000223336	6.10668754	1.47707304	3.31790596	8.1128736	4.16342706	3.30833981	16.4833158	34.8923697	18.1984257	25.6575339	44.8778237	28.0775062	10.7682523	43.7365604	44.7006232	4.4182348	23.9874292	[illegible]
ENSG00000223341	0	0.06030165	0	0	0.05021909	0	0	0	0	0	0	0.15205581	0	0	0	0	0.1354336	[illegible]
ENSG00000223342	0	0	0	0	0	0	0	0	0	0	0	0	0	0	0	0	0	[illegible]
ENSG00000223343	0.61821751	0.57972865	0.44337746	0.3729219	0.25289359	0.64923647	0.16549164	0	0.02037609	0.11198672	0.1038459	0.13922246	0.27907491	0.08929857	0.03584411	0.31806938	0.24800629	[illegible]
ENSG00000223344	0	0	0	0	0.03001408	0	0	0	0	0	0	0	0	0	0	0	0	[illegible]
ENSG00000223345	0.45514196	0.2476998	0.41730062	0.1838513	0.52364374	0.53345828	0.98231631	1.28171862	1.58919145	0.90175836	0.64507274	1.12907816	1.63725589	1.1710487	1.08854733	1.3446362	1.3266038	[illegible]
ENSG00000223349	0	0	0	0	0	0	0	0	0	0	0	0	0	0	0	0	0	[illegible]
ENSG00000223350	0	0	0	0	0	0	0	0.07718652	0	0	0	0	0	0	0	0	0	[illegible]
ENSG00000223351	0	0	0	0	0	0	0	0	0	0	0	0	0	0	0	0	0.06106452	[illegible]
ENSG00000223353	0	0	0	0	0	0	0	0.0399106	0	0	0	0	0	0	0	0	0	[illegible]
ENSG00000223356	0	0.39885433	0.14558941	0.3848564	0.04152064	0.22333792	0.5379816	0.36454142	0.22079592	0.80899417	0.5626385	0.56573191	0.06300132	0.24191045	0.12946926	0.07180435	0.11197513	[illegible]
ENSG00000223358	0	0.02174017	0.0317423	0.0209772	0.03621034	0.03652014	0.02606535	0.07947957	0.00802322	0.05879397	0	0.01370493	0.01373594	0.01758094	0	0.01565523	0	[illegible]
ENSG00000223360	0	0.21642096	0.3949888	0.1044128	0	0.12118461	0	0	0	0	0	0	0	0	0	0	0	[illegible]
ENSG00000223361	0.10888426	0.04375944	0.08518948	0.0281491	0.07288555	0.06534143	0.06995376	0.0639918	0.02153257	0.07889515	0.08230489	0.07356214	0.05529643	0.07077515	0.01893925	0.02100762	0.09828087	[illegible]
ENSG00000223362	0	0	0	0	0	0	0	0	0	0	0	0	0	0	0	0	0	[illegible]
ENSG00000223368	0	0	0	0	0	0	0	0	0	0	0	0	0	0	0	0	0	[illegible]
ENSG00000223373	0	0	0	0	0	0	0	0	0	0	0	0	0	0	0	0	0	[illegible]
ENSG00000223374	0	0.04884161	0	0	0	0	0	0	0	0	0	0	0.06171849	0	0	0	0.10969509	[illegible]
ENSG00000223375	0.14921176	0	0	0	0	0.11192745	0.23965641	0	0.22130702	0	0	0.12600922	0	0	0	0	0	[illegible]
ENSG00000223377	0	0	0	0	0	0	0	0	0	0	0	0	0	0	0	0	0	[illegible]
ENSG00000223379	0	0	0	0.0846762	0	0.04913888	0	0	0	0	0	0	0	0	0	0	0	[illegible]
ENSG00000223380	25.2490442	24.4552145	23.881601	35.412448	26.5451924	20.8467736	44.3087198	45.1671734	34.199396	38.901726	45.5153548	34.7037723	65.4509072	83.0443872	70.0122326	94.1251395	69.210832	[illegible]
ENSG00000223381	0	0.04497498	0	0	0	0.05036735	0	0	0	0	0.08459114	0	0	0	0	0	0.10101089	[illegible]
ENSG00000223382	0.06221957	0.13891886	0.18254888	0	0.06941481	0.10890238	0.04996697	0	0.01025361	0.01878456	0.02612854	0.03502959	0.05266327	0	0.01803738	0.02000726	0	[illegible]
ENSG00000223383	0	0	0	0	0	0	0	0	0	0	0	0	0	0	0	0	0	[illegible]
ENSG00000223387	0.01571033	0	0.02304663	0	0.00876356	0	0.00630828	0.01538839	0.01165058	0.0142292	0.00989611	0.01326736	0.01329738	0	0.01366322	0	0.02363404	[illegible]
ENSG00000223389	0	0.03515959	0	0	0	0	0	0	0	0.04754268	0	0	0	0	0	0	0	[illegible]
ENSG00000223390	0	0	0	0	0.02359375	0	0.10190115	0.04142951	0.1463763	0.03830867	0.05328575	0.0714383	0.03579996	0.04582118	0	0.08160441	0.06362891	[illegible]
ENSG00000223391	0	0	0	0	0	0	0	0	0.1435505	0	0	0	0	0	0.25252337	0	0	[illegible]
ENSG00000223392	0	0	0	0	0	0	0.20243603	0.07054589	0.05341041	0	0	0	0	0	0	0	0	[illegible]
ENSG00000223393	0.12177484	0.21751124	0.19848934	0.1574082	0.04528573	0.18269267	0.88014874	0.83495595	0.68231686	0.47794166	0.71593505	0.41135502	0.03435714	0.04397449	0	0.19578893	0.06106452	[illegible]
ENSG00000223394	0	0	0	0	0	0	0	0	0	0	0	0	0.18125962	0.23199828	0	0.1032933	0	[illegible]
ENSG00000223395	0	0	0	0	0	0	0	0	0	0	0	0	0	0	0	0	0	[illegible]
ENSG00000223396	0.08303067	0.17611499	0.06766876	0.0536634	0.09263255	0.11418618	0.52232544	0.43375577	0.34208041	0.48881797	0.66249133	0.3505967	0.09370398	0.2398677	0.28884596	0.05339851	0.06245414	[illegible]
ENSG00000223398	0	0	0	0	0	0.05188054	0	0	0	0	0	0	0	0	0	0	0	[illegible]
ENSG00000223400	0	0	0	0	0	0	0	0	0	0	0	0	0	0	0	0	0	[illegible]
ENSG00000223401	0.06013011	0.0402761	0	0	0	0.13531528	0.04828898	0	0	0	0	0.05077983	0	0	0	0	0	[illegible]
ENSG00000223402	0	0	0	0	0	0	0	0	0	0	0	0	0	0	0	0	0	[illegible]
ENSG00000223403	0.02568107	0.11467724	0.06697499	0.0885221	0.03820119	0.15411206	0.01374921	0.02515481	0	0.00775331	0.02156905	0	0.28982281	0.08346392	0.3722456	0.18993346	0.38633649	[illegible]

ENSG00000223404	0	0	0	[illegible]
ENSG00000223406	0	0	0	[illegible]
ENSG00000223407	0	0	0	[illegible]
ENSG00000223408	0	0	0	[illegible]
ENSG00000223409	0	0	0	[illegible]
ENSG00000223410	0	0	0	[illegible]
ENSG00000223414	0.41806429	0.16743828	0.28662306	[illegible]
ENSG00000223416	0	0.06203446	0.27172506	[illegible]
ENSG00000223417	0	0	0.0082904	[illegible]
ENSG00000223418	0	0	0	[illegible]
ENSG00000223419	0	0	0	[illegible]
ENSG00000223421	0	0	0	[illegible]
ENSG00000223422	0	0	0	[illegible]
ENSG00000223427	0	0	0	[illegible]
ENSG00000223428	0	0	0	[illegible]
ENSG00000223429	0	0	0	[illegible]
ENSG00000223430	0	0	0	[illegible]
ENSG00000223431	0	0	0	[illegible]
ENSG00000223432	0	0	0	[illegible]
ENSG00000223433	0	0	0	[illegible]
ENSG00000223436	0.73527923	1.27229602	0.77901404	[illegible]
ENSG00000223437	0	0	0	[illegible]
ENSG00000223438	0	0	0	[illegible]
ENSG00000223440	0	0	0	[illegible]
ENSG00000223442	0	0.01263934	0	[illegible]
ENSG00000223443	0	0	0	[illegible]
ENSG00000223445	0	0	0	[illegible]
ENSG00000223446	0.40625722	0	0	[illegible]
ENSG00000223449	0	0	0	[illegible]
ENSG00000223450	0	0	0	[illegible]
ENSG00000223452	0.11805765	0.11861533	0.05772913	[illegible]
ENSG00000223455	0	0	0	[illegible]
ENSG00000223457	0	0	0	[illegible]
ENSG00000223458	0.39789802	0.79955521	1.03769898	[illegible]
ENSG00000223459	0.63772697	0.89813051	0.7036452	[illegible]
ENSG00000223460	0.76737476	0.57824975	0.81301862	[illegible]
ENSG00000223461	0	0.07418553	0.10831652	[illegible]
ENSG00000223462	0	0	0	[illegible]
ENSG00000223466	0	0	0	[illegible]
ENSG00000223467	0	0	0.0727947	[illegible]
ENSG00000223469	0	0	0	[illegible]
ENSG00000223470	0	0.06941476	0.10135083	[illegible]
ENSG00000223473	0.53677336	0.5002286	0.68472353	[illegible]
ENSG00000223474	0.04655234	0.07275683	0.06070314	[illegible]
ENSG00000223475	0.05838721	0.07821736	0.05710164	[illegible]
ENSG00000223476	1.49905766	2.19645253	1.55767969	[illegible]
ENSG00000223477	0	0	0	[illegible]
ENSG00000223478	0.41472092	0.03968381	0.05794137	[illegible]
ENSG00000223479	0.18374994	0.14769435	0.21564497	[illegible]
ENSG00000223482	0.76348448	0.87752824	0.73795807	[illegible]
ENSG00000223484	0	0	0	[illegible]
ENSG00000223485	0.02578379	0.01727039	0	[illegible]
ENSG00000223486	0.01197686	0	0	[illegible]
ENSG00000223487	0	0	0	[illegible]
ENSG00000223488	0.0156455	0	0	[illegible]
ENSG00000223489	0	0	0	[illegible]
ENSG00000223490	0	0	0	[illegible]
ENSG00000223491	0	0	0	[illegible]
ENSG00000223492	0	0	0	[illegible]
ENSG00000223495	0	0	0	[illegible]
ENSG00000223496	11.735417	10.2733437	9.14083092	[illegible]
ENSG00000223497	0	0	0	[illegible]
ENSG00000223498	0	0	0	[illegible]
ENSG00000223500	0	0	0	[illegible]
ENSG00000223501	2.0162378	2.5323599	1.90017434	[illegible]
ENSG00000223502	0.0765552	0.25638944	0.14973922	[illegible]
ENSG00000223503	0	0.03261026	0	[illegible]
ENSG00000223504	0.45855321	0.92143862	0.57658732	[illegible]
ENSG00000223505	0	0	0	[illegible]
ENSG00000223506	0	0	0	[illegible]
ENSG00000223508	0.98978088	0.89777312	0.58482603	[illegible]
ENSG00000223509	0.62116012	0.57681447	0.73174142	[illegible]
ENSG00000223510	0	0	0	[illegible]
ENSG00000223511	0	0	0	[illegible]
ENSG00000223512	0	0	0	[illegible]
ENSG00000223513	0	0	0.1286535	[illegible]
ENSG00000223514	0	0	0	[illegible]
ENSG00000223516	0	0	0	[illegible]
ENSG00000223517	0	0	0	[illegible]
ENSG00000223518	0.05655994	0.07576949	0.05531461	[illegible]
ENSG00000223519	0	0	0	[illegible]
ENSG00000223522	0	0	0	[illegible]
ENSG00000223523	0	0	0	[illegible]
ENSG00000223524	0	0	0	[illegible]
ENSG00000223525	0.69969584	1.66962749	1.62518867	[illegible]
ENSG00000223528	0	0	0	[illegible]
ENSG00000223529	0.55728657	0.49770583	0.59043427	[illegible]
ENSG00000223530	0	0	0	[illegible]
ENSG00000223534	0	0	0	[illegible]
ENSG00000223535	0	0	0	[illegible]
ENSG00000223536	0	0	0	[illegible]
ENSG00000223537	0.16874209	0	0.08251337	[illegible]
ENSG00000223538	0	0	0	[illegible]
ENSG00000223539	0	0	0	[illegible]
ENSG00000223540	0	0	0	[illegible]
ENSG00000223542	0	0	0	[illegible]
ENSG00000223543	0	0.03671427	0.05360562	[illegible]
ENSG00000223544	0	0	0	[illegible]
ENSG00000223546	0.83224821	1.03679165	1.03684561	[illegible]
ENSG00000223547	3.63151997	4.77506449	3.69954303	[illegible]
ENSG00000223548	0.13967385	0.16372257	0.06829926	[illegible]
ENSG00000223549	0	0	0	[illegible]
ENSG00000223550	0	0	0	[illegible]
ENSG00000223551	3.285556	1.9212613	2.95819771	[illegible]
ENSG00000223552	0	0	0	[illegible]
ENSG00000223553	0.040677	0.0272461	0.03315114	[illegible]
ENSG00000223554	0	0	0	[illegible]
ENSG00000223555	0	0	0	[illegible]
ENSG00000223558	0	0.03002502	0	[illegible]
ENSG00000223559	0.01117536	0.07485434	0.0109293	[illegible]
ENSG00000223561	0	0	0	[illegible]
ENSG00000223562	0	0	0	[illegible]
ENSG00000223563	0	0	0	[illegible]
ENSG00000223564	0	0	0	[illegible]
ENSG00000223565	0	0	0	[illegible]
ENSG00000223566	0.04750146	0	0.02322779	[illegible]
ENSG00000223568	0	0	0	[illegible]
ENSG00000223569	0	0	0	[illegible]
ENSG00000223570	0	0	0	[illegible]
ENSG00000223571	0	0.23212893	0.14525395	[illegible]
ENSG00000223572	0	0.00288032	0	[illegible]
ENSG00000223573	0.04781861	0.04575666	0.05344656	[illegible]
ENSG00000223574	0	0	0	[illegible]
ENSG00000223575	0	0.04511597	0.03293637	[illegible]
ENSG00000223576	0.0725895	0.1458648	0.35495616	[illegible]
ENSG00000223579	0.00945847	0	0.00925021	[illegible]
ENSG00000223581	0	0	0	[illegible]
ENSG00000223583	0.18072751	0.32281107	0.17674826	[illegible]
ENSG00000223584	0.05043778	0.54054436	0.29596344	[illegible]
ENSG00000223586	0	0	0	[illegible]
ENSG00000223587	0	0	0	[illegible]
ENSG00000223588	0.06976134	0	0.13645068	[illegible]
ENSG00000223589	0	0	0	[illegible]
ENSG00000223591	1.22505439	1.03776813	0.77522901	[illegible]
ENSG00000223592	0	0	0	[illegible]
ENSG00000223593	0	0	0	[illegible]
ENSG00000223595	0	0	0	[illegible]
ENSG00000223597	0.05585743	0.11224259	0.1638827	[illegible]
ENSG00000223598	0	0	0	[illegible]
ENSG00000223599	0.27083815	0.54423506	0.60198829	[illegible]
ENSG00000223600	0	0	0	[illegible]
ENSG00000223601	0	0.01787085	0.0260928	[illegible]
ENSG00000223602	0.02297202	0	0	[illegible]
ENSG00000223603	0	0	0	[illegible]
ENSG00000223604	0	0.1626214	0.1780797	[illegible]
ENSG00000223605	0	0	0	[illegible]
ENSG00000223608	0	0	0	[illegible]
ENSG00000223609	0	0.02015685	0	[illegible]
ENSG00000223611	0	0	0	[illegible]
ENSG00000223612	0.02635302	0	0	[illegible]
ENSG00000223614	0	0	0	[illegible]
ENSG00000223615	0	0	0	[illegible]
ENSG00000223617	0	0	0	[illegible]
ENSG00000223619	0	0	0	[illegible]
ENSG00000223620	0	0	0	[illegible]
ENSG00000223621	0	0	0	[illegible]
ENSG00000223622	0	0	0	[illegible]
ENSG00000223623	0	0	0	[illegible]
ENSG00000223624	0	0	0	[illegible]
ENSG00000223625	0	0.20175692	0	[illegible]
ENSG00000223626	0.14716776	0.42715963	0.33583066	[illegible]
ENSG00000223628	0	0	0	[illegible]
ENSG00000223629	0	0	0	[illegible]
ENSG00000223631	0	0	0.01693719	[illegible]
ENSG00000223633	0.06484857	0	0	[illegible]
ENSG00000223634	0	0	0	[illegible]
ENSG00000223635	0	0	0	[illegible]
ENSG00000223636	0	0	0	[illegible]
ENSG00000223637	0	0	0	[illegible]
ENSG00000223638	1.80611102	0.85517644	2.04043202	[illegible]
ENSG00000223640	0	0	0	[illegible]
ENSG00000223641	0	0	0	[illegible]
ENSG00000223642	0.01408642	0.00943531	0.02755254	[illegible]
ENSG00000223643	0	0.03349572	0	[illegible]
ENSG00000223646	0	0	0	[illegible]
ENSG00000223647	0	0.05164591	0	[illegible]
ENSG00000223648	0	0	0	[illegible]
ENSG00000223649	0	0	0	[illegible]
ENSG00000223650	0	0.00904398	0	[illegible]
ENSG00000223651	0	0.00952271	0	[illegible]
ENSG00000223652	0	0.01628054	0	[illegible]

ENSG00000223653	0	0.00894283	0.01305721	0.017258	0.00744757	0	0.02144399	0.01307758	0.03300353	0.02418493	[illegible]
ENSG00000223655	0	0	0	0	0	0	0	0	0	0	[illegible]
ENSG00000223656	0	0	0.05510508	0	0	0	0.09049962	0	0	0.05103358	[illegible]
ENSG00000223657	0	0	0	0	0	0	0	0	0	0	[illegible]
ENSG00000223658	3.44719413	3.25914292	3.34145948	2.149085	2.14413896	2.57438434	1.46992291	1.43428847	1.05573818	1.42295042	[illegible]
ENSG00000223659	0	0	0	0	0	0	0	0	0	0	[illegible]
ENSG00000223660	0.07860912	0	0.07687831	0.1016115	0.04384984	0	0.18938702	0.15399652	0.07772734	0	[illegible]
ENSG00000223662	0	0	0	0	0	0	0	0	0	0	[illegible]
ENSG00000223663	0	0	0	0	0	0	0	0	0.13025154	0	[illegible]
ENSG00000223664	0	0	0	0	0	0	0	0	0	0	[illegible]
ENSG00000223665	0.66143123	0.84579814	0.47044935	0	0.33541856	0.45105092	0	0	0	0	[illegible]
ENSG00000223668	0.23388781	0.37598822	0.34310711	0.3023273	0.28702872	0.49124617	0.26296117	0.34364245	0.23126423	0.16946999	[illegible]
ENSG00000223669	0	0	0	0	0	0	0	0.08543785	0.02156171	0.07900191	[illegible]
ENSG00000223671	0	0	0	0	0	0	0	0	0	0	[illegible]
ENSG00000223672	0	0	0	0	0	0	0	0	0	0	[illegible]
ENSG00000223675	0.08907344	0.0298314	0.04355611	0.0191896	0.13249883	0.0334081	0.01192211	0	0	0	[illegible]
ENSG00000223676	0	0	0	0	0	0	0	0	0	0	[illegible]
ENSG00000223677	0	0	0	0	0	0	0	0	0	0	[illegible]
ENSG00000223678	0	0	0	0	0	0	0	0	0	0	[illegible]
ENSG00000223679	0	0	0	0	0	0	0	0	0	0	[illegible]
ENSG00000223683	0	0	0	0	0	0	0	0	0	0	[illegible]
ENSG00000223684	0	0	0	0	0	0	0	0	0	0	[illegible]
ENSG00000223685	0.08349674	0.11185487	0.10207288	0.0269823	0.02328813	0	0.21792591	0.18401786	0.19608031	0.20796844	[illegible]
ENSG00000223688	0	0	0	0	0	0	0	0	0	0	[illegible]
ENSG00000223691	0	0.01091405	0.01593534	0	0	0	0	0	0	0	[illegible]
ENSG00000223692	0.30121252	0.55483153	0	0	0.29403982	0.33892051	0.30237024	0.29504006	0.18614609	0.34101876	[illegible]
ENSG00000223694	0.11338519	0.0759472	0.08316651	0.0732818	0.03162434	0.08505305	0.0227642	0.05553085	0	0	[illegible]
ENSG00000223695	0.31597784	0.21164697	0.30902065	0.245063	0.1057555	0.23702284	0.10150154	0	0.37492013	0.11447532	[illegible]
ENSG00000223697	0.0196164	0	0	0	0	0.04414424	0	0	0.00969818	0	[illegible]
ENSG00000223698	0	0	0	0	0	0	0	0	0	0	[illegible]
ENSG00000223700	0	0	0	0	0	0	0	0	0	0	[illegible]
ENSG00000223701	0	0	0	0	0	0	0	0.03412896	0.01722606	0.03155806	[illegible]
ENSG00000223702	0	0	0	0	0	0	0	0	0	0	[illegible]
ENSG00000223703	0.60556922	0.99151522	0.85545175	0.382687	0.12761311	0.25236252	0.02161411	0	0.01330614	0.03656519	[illegible]
ENSG00000223704	0.30400383	0.48584589	0.34946999	0.2895498	0.20230574	0.32805875	0.23557159	0.29255007	0.19775905	0.17390095	[illegible]
ENSG00000223705	0.79582665	1.99439821	1.05228664	1.3555752	0.80764662	1.93038692	1.27025273	1.33805208	0.83837909	2.81358319	[illegible]
ENSG00000223707	0	0	0.04317823	0.0570695	0	0.06623652	0	0	0	0	[illegible]
ENSG00000223709	0.03012829	0.0100902	0.00736623	0.0097361	0	0.03389997	0.01814645	0.0295109	0.01861896	0.00682197	[illegible]
ENSG00000223710	0	0	0	0	0	0	0	0	0	0	[illegible]
ENSG00000223711	0.04164049	0.06972865	0.04072365	0.1076504	0.05806988	0.03123557	0.08360107	0.06118079	0.0411734	0.03771474	[illegible]
ENSG00000223714	0.07719698	0.1292694	0	0.0997861	0.08612424	0.05790738	0.03099748	0.07561506	0.01908276	0.20975717	[illegible]
ENSG00000223715	0.30748234	0.28084983	0.13668737	0	0.14033471	0.12580917	0.0224483	0	0.02763939	0	[illegible]
ENSG00000223716	0	0	0	0	0	0	0	0	0	0	[illegible]
ENSG00000223717	0	0	0	0	0	0	0	0	0	0	[illegible]
ENSG00000223718	0.05266297	0.05291174	0	0.0340365	0	0.03950381	0	0.0257919	0	0	[illegible]
ENSG00000223719	0	0	0	0	0	0	0	0	0	0	[illegible]
ENSG00000223720	0	0	0	0	0	0	0	0	0	0	[illegible]
ENSG00000223721	0	0	0	0	0	0	0	0	0	0	[illegible]
ENSG00000223722	0	0	0.03905837	0	0	0	0.67353252	0.31295452	0.09872432	0.25320749	[illegible]
ENSG00000223723	0.02620304	0.01755121	0	0	0.01461661	0.13758887	0.021043	0.10266435	0.15545469	0.07119806	[illegible]
ENSG00000223724	0	0	0	0	0	0	0	0.01596828	0	0.01476541	[illegible]
ENSG00000223725	0	0.01190733	0.01738561	0.1148944	0.0099164	0.05333994	0.08565767	0.0522382	0.06152164	0.24151577	[illegible]
ENSG00000223726	0.11152159	0.07469893	0	0	0.03110456	0.04182756	0	0	0	0	[illegible]
ENSG00000223727	0.03397969	0.02276014	0.03323153	0	0.0094773	0	0	0.03328338	0	0.01538809	[illegible]
ENSG00000223728	0	0	0	0	0	0	0	0	0	0	[illegible]
ENSG00000223729	0	0	0	0	0	0	0.03483566	0	0	0	[illegible]
ENSG00000223730	0	0	0	0	0.01019186	0	0	0	0	0.0165483	[illegible]
ENSG00000223731	0	0	0	0	0	0	0	0	0	0	[illegible]
ENSG00000223732	0	0	0	0	0	0.1535005	0	0	0.05058446	0	[illegible]
ENSG00000223733	0	0	0	0	0	0	0	0	0.03029298	0	[illegible]
ENSG00000223734	0.22498946	0.1507015	0.05500891	0.0727063	0.18825586	0.16877019	0	0.11018948	0.02780821	0.05094451	[illegible]
ENSG00000223735	0	0.09458046	0	0.0456306	0	0.07944029	0	0	0	0	[illegible]
ENSG00000223738	0	0	0	0	0	0	0	0	0	0	[illegible]
ENSG00000223739	0.10532595	0	0	0	0	0	0	0	0	0	[illegible]
ENSG00000223740	0	0	0	0	0	0	0	0	0	0	[illegible]
ENSG00000223741	0.02906198	0	0	0	0.01621139	0.04360023	0	0	0.02873599	0.0526442	[illegible]
ENSG00000223742	0	0	0	0	0	0	0.03423663	0	0.02107686	0	[illegible]
ENSG00000223744	0	0	0	0	0	0	0	0	0	0	[illegible]
ENSG00000223745	0.20741413	0.49367101	0.26389842	0.4373007	0.1898379	0.54983967	0.79565031	0.58779428	0.35641423	0.99583871	[illegible]
ENSG00000223746	0	0	0	0	0	0	0	0	0	0	[illegible]
ENSG00000223749	0.43241149	1.17201571	0.51140267	0.5589423	0.36461725	0.75433165	0.74297227	0.87665102	0.80540564	2.29522121	[illegible]
ENSG00000223750	0	0	0	0	0	0	0	0	0.01310371	0	[illegible]
ENSG00000223751	0	0	0	0	0.03360455	0.0451894	0	0	0	0	[illegible]
ENSG00000223753	0	0	0.04704494	0	0.02683349	0.18042037	0.07726237	0.04711834	0.09512899	0.04356896	[illegible]
ENSG00000223754	0.05935495	0.07951378	0	0	0.03310946	0	0	0	0.05868915	0	[illegible]
ENSG00000223756	0.18681057	0.28977169	0.20192869	0.1588648	0.06581489	0.1880709	0.36321326	0.28410432	0.14096677	0.16919857	[illegible]
ENSG00000223758	0	0	0	0	0	0	0	0	0	0	[illegible]
ENSG00000223759	0	0	0	0	0	0	0.19509718	0.25382341	0.2001772	0.24937211	[illegible]
ENSG00000223760	0.0407284	0.04546755	0.07966329	0.1228412	0.03029222	0.05091898	0.08722121	0.05319172	0.03355962	0.07377727	[illegible]
ENSG00000223761	0	0	0	0	0	0	0	0	0	0	[illegible]
ENSG00000223764	0	0	0	0.006751	0	0.00391773	0.34393084	0.2609032	0.22206013	0.37370042	[illegible]
ENSG00000223765	0	0	0	0	0	0	0.11452607	0	0	0	[illegible]
ENSG00000223768	3.38284832	4.05065819	2.92314432	2.0665625	2.42986756	2.57231973	6.17765662	5.26533037	5.04026341	6.0439018	[illegible]
ENSG00000223770	0.00897514	0.05410524	0.05266517	0.0232028	0.01001305	0.05385983	0.15136195	0.06153857	0.0665585	0.0894189	[illegible]
ENSG00000223772	0	0	0	0	0	0	0	0	0	0	[illegible]
ENSG00000223773	0.11674525	0.15639563	0.15748242	0.1457032	0.12126349	0.1660877	0.27803257	0.2287058	0.23485192	0.37920128	[illegible]
ENSG00000223774	0.53028822	1.30238334	0.60504775	1.8278919	0.23007138	1.56903052	0.33122532	0.49056479	0.23303993	0.48029407	[illegible]
ENSG00000223776	0.16844812	0.1880487	0.13179139	0.1016115	0.1691351	0.15162855	0.06312901	0.06599851	0.0943832	0.10171152	[illegible]
ENSG00000223777	0	0	0	0	0	0	0	0	0	0	[illegible]
ENSG00000223779	0.02157038	0.01685621	0.01406363	0.0232352	0.00601621	0.02696746	0.14146812	0.12677014	0.09775525	0.07489099	[illegible]
ENSG00000223780	0	0	0	0	0	0	0	0	0	0	[illegible]
ENSG00000223781	0	0	0	0	0	0	0	0	0	0	[illegible]
ENSG00000223782	0	0	0	0	0	0	0	0	0	0	[illegible]
ENSG00000223783	0	0.01438241	0	0	0.01197764	0	0.03448753	0.02103217	0.03184698	0.03889568	[illegible]
ENSG00000223784	0	0	0	0	0	0	0	0.0344267	0	0	[illegible]
ENSG00000223786	0	0	0	0	0	0	0.11606678	0	0	0	[illegible]
ENSG00000223787	0.09314954	0.06239304	0.09109857	0.1204067	0.05196079	0.13974757	0.07480605	0	0.04605233	0	[illegible]
ENSG00000223788	0	0	0	0	0.03216178	0	0	0	0.02850466	0	[illegible]
ENSG00000223791	0.41426401	0.72144956	0.20257138	0.5354846	0.30041087	0.40397466	0.266148	0.16230996	0.14336599	0.33768748	[illegible]
ENSG00000223794	0	0	0	0	0	0	0	0	0	0	[illegible]
ENSG00000223795	0	0	0	0	0	0	0	0	0	0	[illegible]
ENSG00000223797	0.79589333	0.83923973	0.98644832	1.110273	0.6505644	1.00488411	0.31641678	0.23155956	0.19479347	0.30690021	[illegible]
ENSG00000223799	0.04339732	0.09689403	0.02829453	0.0560961	0.01613863	0.07595795	0.03485129	0.07084669	0.05006227	0	[illegible]
ENSG00000223800	0	0	0	0	0	0	0	0	0	0	[illegible]
ENSG00000223802	0.96934846	0.96364465	1.26543705	1.593176	1.69067754	1.84250741	0.01408976	0.04725941	0.04336991	0.14301625	[illegible]
ENSG00000223803	0.44763527	0	0.61289096	0.8100692	0.39952078	0.47009529	0.35948462	0.43846232	0.35409123	1.13521358	[illegible]
ENSG00000223804	0.02830481	0.05055735	0.02768159	0.0121958	0.02631504	0.01415476	0.09092351	0.12938232	0.06996826	0.11963609	[illegible]
ENSG00000223806	0.01954502	0.03272891	0.00955734	0	0.01090263	0	0	0.00957225	0	0	[illegible]
ENSG00000223807	0	0	0	0	0	0	0	0	0	0	[illegible]
ENSG00000223808	0	0	0	0	0	0	0	0	0	0	[illegible]
ENSG00000223809	0	0	0	0	0	0	0	0	0	0	[illegible]
ENSG00000223810	0	0	0	0	0	0	0	0	0	0	[illegible]
ENSG00000223811	0	0	0	0	0	0	0.10210214	0	0.03142822	0.11515269	[illegible]
ENSG00000223812	0.06931127	0.06190105	0.06778518	0.1493215	0.06443884	0.10398421	11.3179674	14.0081638	12.7130175	14.4177356	[illegible]
ENSG00000223813	0.11383573	0.27640253	0.12524546	0.2575054	0.04762499	0.2241514	0.02285465	0.02787575	0.0281397	0.0257759	[illegible]
ENSG00000223814	0	0	0	0	0	0	0	0	0	0	[illegible]
ENSG00000223815	0	0	0	0	0	0	0	0	0	0	[illegible]
ENSG00000223816	0	0	0	0	0	0	0	0	0	0	[illegible]
ENSG00000223817	0	0	0	0	0	0	0	0	0	0	[illegible]
ENSG00000223819	0.13373336	0	0.06539441	0	0.03729966	0.15047508	0	0	0.03305831	0.06056267	[illegible]
ENSG00000223820	0.131742	0.25212252	0.21166787	0.2676016	0.13122945	0.33882155	0.30983901	0.20278082	0.19539633	0.45171794	[illegible]
ENSG00000223821	0	0	0	0	0	0	0	0	0	0	[illegible]
ENSG00000223822	6.71634631	6.42673603	5.80072328	10.711141	3.94114543	7.98244164	6.7246487	7.47581217	6.55476057	3.67358888	[illegible]
ENSG00000223823	0	0	0	0	0	0	0	0	0	0	[illegible]
ENSG00000223824	0	0	0	0	0	0	0	0	0	0	[illegible]
ENSG00000223825	0.39113762	0.28818919	0.15301023	0.3033546	0.24000338	0.23472164	0.06282255	0.03831224	0.11602504	0.03542622	[illegible]
ENSG00000223826	0	0	0	0	0	0	0.06636639	0	0	0	[illegible]
ENSG00000223828	0	0.07995552	0	0	0	0	0	0	0	0	[illegible]
ENSG00000223829	0	0.01005964	0.01468784	0.0194132	0	0	0.01206099	0.04413227	0	0	[illegible]
ENSG00000223831	0	0	0	0	0	0.19418738	0	0	0	0	[illegible]
ENSG00000223834	0.0765552	0.20511155	0.07486961	0	0.04270412	0.05742596	0	0	0.03784823	0.13867556	[illegible]
ENSG00000223836	0	0.0569604	0	0.1099227	0	0	0	0	0.04204249	0	[illegible]
ENSG00000223837	0.28606278	0.19160938	0.27976426	0.7395391	0.21276254	0.28611041	0.91891927	0.37360103	0.32999627	0.43182258	[illegible]
ENSG00000223838	0.11388601	0	0	0.0736055	0	0.04271436	0	0.05577613	0	0	[illegible]
ENSG00000223839	0.21854199	0.2881187	0.3155064	0.1322775	0.07062888	0.16783697	0.00975031	0.01698918	0.01457754	0.0078547	[illegible]
ENSG00000223841	0	0	0	0	0	0	0	0	0	0	[illegible]
ENSG00000223842	0	0	0	0	0	0	0	0	0	0	[illegible]
ENSG00000223843	0	0.02703004	0	0.0173876	0	0	0.01080254	0.02635166	0.01330059	0.02436662	[illegible]
ENSG00000223845	0	0	0	0	0	0	0	0	0	0	[illegible]
ENSG00000223847	0	0	0	0	0	0	0	0	0	0	[illegible]
ENSG00000223849	0	0	0	0	0	0	0.22312838	0	0.09157532	0	[illegible]
ENSG00000223850	0	0	0	0	0	0	0	0	0	0	[illegible]
ENSG00000223855	0	0	0	0	0	0	0.00738246	0.02701308	0.04090329	0.36634714	[illegible]
ENSG00000223856	0	0	0	0	0	0	0	0	0	0	[illegible]
ENSG00000223859	0	0.03807406	0	0	0	0	0	0	0	0	[illegible]
ENSG00000223861	0.13022117	0.13083631	0.25470793	0.1683261	0.14528028	0.53725176	0.3660207	0.44643436	0.22533078	0.5307492	[illegible]
ENSG00000223863	0	0	0	0	0.06286166	0	0	0	0	0	[illegible]
ENSG00000223864	0	0	0	0	0.05050122	0.03395552	0	0.08867777	0.04475872	0	[illegible]
ENSG00000223865	0.00395457	0.00529767	0.01933749	0.0102235	0.00220594	0.00296642	0.59387741	0.61976514	0.81527877	0.74142082	[illegible]
ENSG00000223866	0	0	0	0	0	0	0	0	0.04075219	0	[illegible]
ENSG00000223867	0	0	0	0	0	0	0	0	0	0	[illegible]
ENSG00000223869	0	0	0	0	0	0	0	0	0	0	[illegible]
ENSG00000223870	0	0	0	0	0	0	0	0	0	0	[illegible]
ENSG00000223872	0	0	0	0	0	0.04427899	0.04740457	0	0.02918334	0.05346375	[illegible]
ENSG00000223873	0	0	0	0	0	0	0	0	0	0	[illegible]
ENSG00000223874	0	0	0	0	0	0	0	0	0	0	[illegible]
ENSG00000223875	0.04705071	0.06303063	0.04601475	0.1216371	0.02624589	0.07058782	0	0.04608655	0	0.0426149	[illegible]
ENSG00000223876	0	0	0	0	0	0	0	0	0	0	[illegible]
ENSG00000223877	0.35910574	0.33073523	0.39509883	0.4061628	0.17527722	0.33671768	0.25234018	0.30777856	0.28850052	0.32525021	[illegible]
ENSG00000223878	0.79091386	0.57391386	0.90241614	0.6815657	0.84561146	0.59328416	0.3175815	0.25823548	0.22809558	0.29847859	[illegible]
ENSG00000223880	0	0	0	0	0	0	0	0	0	0	[illegible]
ENSG00000223881	0	0	0	0	0	0	0	0	0	0	[illegible]
ENSG00000223882	0	0	0	0	0	0	0	0	0	0	[illegible]
ENSG00000223883	0.10280619	0.10329182	0.20108521	0	0.1433687	0.1927937	0	0	0	0	[illegible]
ENSG00000223884	0	0	0	0	0	0	0	0	0	0	[illegible]
ENSG00000223885	0	0	0	0	0.00551486	0	0	0	0	0	[illegible]
ENSG00000223886	0	0	0.06807798	0	0.03883031	0	0	0	0	0.06304796	[illegible]
ENSG00000223889	0	0	0	0	0	0	0	0	0	0	[illegible]
ENSG00000223890	0	0	0	0	0	0	0	0	0	0	[illegible]
ENSG00000223891	1.86622697	2.09588096	1.86163919	2.5088089	0.81546656	2.38916824	1.68855775	1.28568221	1.40857369	1.88184962	[illegible]
ENSG00000223893	0	0	0	0	0	0	0	0	0	0	[illegible]
ENSG00000223896	0	0	0	0	0	0	0	0	0	0	[illegible]
ENSG00000223897	0	0	0	0	0	0	0	0	0	0	[illegible]
ENSG00000223899	0	0	0	0	0	0	0	0	0	0	[illegible]
ENSG00000223901	0	0	0	0	0	0	0	0	0	0	[illegible]
ENSG00000223903	0	0	0	0	0	0	0	0	0.03830314	0	[illegible]
ENSG00000223904	0	0	0	0	0	0	0	0	0	0	[illegible]
ENSG00000223905	0	0	0	0	0	0	0	0	0	0	[illegible]
ENSG00000223906	0	0	0	0	0	0	0	0	0	0	[illegible]
ENSG00000223907	0.03337276	0.055884	0.01631898	0.0539227	0.04654009	0.04380904	0.04690144	0.07354998	0.05362241	0.02266985	[illegible]
ENSG00000223908	0.10532595	0.07054899	0	0	0	0.07900761	0	0	0	0	[illegible]

ENSG00000223910	0	0	0	0	0	0.01251363	0.01339694	0.01634021	0	0	0.02101643	0	0	0.01807233	0	0	0	[illegible]
ENSG00000223911	0.07822752	0	0	0	0	0.05868041	0	0	0.03867501	0	0	0	0	0	0	0	0	[illegible]
ENSG00000223912	0	0	0	0	0.0133075	0	0	0	0	0	0.03005459	0.02014655	0	0	0	0	0	[illegible]
ENSG00000223914	0	0	0	0	0	0	0	0	0	0	0	0	0	0	0	0	0	[illegible]
ENSG00000223915	0	0	0	0	0	0	0	0	0	0	0	0	0	0	0	0	0	[illegible]
ENSG00000223916	0.25032808	0.08383686	0.06120409	0	0.10472875	0.23472164	0.25129022	0.30649793	0.15470005	0.11336391	0	0.21140187	0.10594009	0.06779756	0.1632821	0	0.09414608	[illegible]
ENSG00000223917	0	0	0	0	0	0	0	0	0	0	0	0	0	0	0	0	0	[illegible]
ENSG00000223918	0	0	0	0.1859853	0	0	0	0	0	0	0	0	0	0.15587385	0	0.13880037	0.21645192	[illegible]
ENSG00000223920	0	0	0	0	0	0	0	0	0	0	0	0	0	0	0	0	0	[illegible]
ENSG00000223921	0.07146284	0.11966735	0.03494469	0.046187	0	0.02680303	0	0	0	0	0	0	0	0	0	0	0	[illegible]
ENSG00000223922	0.02635302	0.10591001	0.15463666	0.2043861	0.02940055	0.09884027	0.12698067	0.12906495	0.10422964	0.31029083	0.16600059	0.22255103	0.06691637	0.02854926	0.02291913	0.05084429	0.0396445	[illegible]
ENSG00000223923	0.0567425	0.19003513	0.33295887	0.2200389	0.1266087	0.25538376	0	0.11115946	0.14026501	0.30835781	0	0.047919	0.14408225	0	0	0.05473817	0	[illegible]
ENSG00000223925	0	0	0	0	0	0	0	0	0	0	0.14098523	0	0	0	0	0	0	[illegible]
ENSG00000223928	0	0	0	0	0.01898462	0.02552939	0	0	0	0	0	0	0	0.07373969	0	0	0	[illegible]
ENSG00000223929	0	0	0	0	0	0	0	0	0	0	0	0	0	0	0	0	0	[illegible]
ENSG00000223930	0	0.00486107	0	0	0	0	0	0.0071086	0	0	0	0.0122576	0	0	0	0	0	[illegible]
ENSG00000223931	0	0	0	0	0	0	0	0	0	0	0	0	0	0	0	0	0	[illegible]
ENSG00000223935	0.08915557	0.11943563	0.13078882	0.115244	0.22379795	0.20063344	0.35799298	0.08732859	0.11019437	0.28262579	0.05616009	0.15058363	0.15092433	0.24146433	0.11630744	0.12900947	0.06706117	[illegible]
ENSG00000223940	0	0	0	0	0	0	0	0	0	0	0	0	0	0	0	0	0	[illegible]
ENSG00000223941	0	0	0	0	0	0	0	0.02221625	0.05606652	0.04108544	0.05714813	0.03830822	0	0	0	0	0	[illegible]
ENSG00000223942	0.08977643	0	0	0	0.20031682	0.26937414	0	0	0	0	0	0	0	0	0	0	0	[illegible]
ENSG00000223944	0	0	0	0	0	0	0	0	0	0	0	0	0	0	0	0	0	[illegible]
ENSG00000223945	0	0	0	0	0	0	0	0.03628654	0	0	0	0.03128505	0	0	0	0.07147421	0	[illegible]
ENSG00000223946	0	0	0	0	0	0	0	0	0	0	0	0	0	0	0	0	0	[illegible]
ENSG00000223947	0.05548879	0.01238909	0.02713352	0.0717257	0.04642924	0.10405881	0.02970777	0.05435171	0.02743318	0.06700994	0.04660401	0.0390502	0.01565542	0.04007546	0.03217227	0.02676437	0.04173764	[illegible]
ENSG00000223948	0	0	0	0	0	0	0	0	0	0	0	0	0	0	0	0	0	[illegible]
ENSG00000223949	0.49676452	0.6023755	0.50257943	0.2878497	0.25799508	0.45615716	0.41957386	0.44463784	0.36839415	0.27926745	0.46398114	0.60034367	0.50021007	0.5474432	0.4320344	0.35528172	0.283464	[illegible]
ENSG00000223951	0	0	0	0	0	0	0	0	0.0617601	0	0.15737886	0	0.10573478	0	0	0	0	[illegible]
ENSG00000223953	0	0	0	0	0	0	0	0	0	0	0	0	0	0	0	0	0	[illegible]
ENSG00000223955	0	0	0	0	0	0	0	0	0	0	0	0	0	0	0	0	0	[illegible]
ENSG00000223956	0	0	0	0	0	0	0	0	0	0	0	0	0	0	0	0	0	[illegible]
ENSG00000223958	0	0	0	0	0	0	0	0	0	0	0	0	0	0	0	0	0	[illegible]
ENSG00000223959	0.25882749	0.35369603	0.27041051	0.2942558	0.25628808	0.30487469	0.22622279	0.20566974	0.22354821	0.21653801	0.23440851	0.30811826	0.44518686	0.29165894	0.39144135	0.39006994	0.42377273	[illegible]
ENSG00000223960	1.56654512	2.39102047	1.80330172	1.1419348	1.1718215	1.2791301	1.80252177	1.6300317	1.5438942	1.70727852	1.44944047	1.54416011	1.39549685	1.14623789	1.26861301	1.78867773	1.65352017	[illegible]
ENSG00000223962	0	0	0	0	0	0	0	0	0	0	0	0	0	0	0	0	0.20118352	[illegible]
ENSG00000223963	0	0	0.02980625	0	0	0	0.01223777	0	0.00753386	0.01380199	0	0	0.01289814	0.01650862	0.026506	0.05880148	0	[illegible]
ENSG00000223965	0	0	0	0	0	0	0	0	0	0	0	0	0	0	0	0	0	[illegible]
ENSG00000223966	0	0	0	0	0	0	0	0	0	0	0	0	0	0	0	0	0	[illegible]
ENSG00000223967	0	0	0	0	0	0	0	0	0	0	0	0	0	0	0	0	0	[illegible]
ENSG00000223968	0	0.16931757	0.12360826	0.1633753	0.07050367	0	0.10150154	0.12380112	0.18746006	0.11447532	0.15923037	0.10673722	0.10697872	0	0	0	0	[illegible]
ENSG00000223969	0.71562745	0.88766409	0.59618623	1.1991156	1.16800688	0.77539214	0.14899691	0.15576951	0.20965928	0.40810075	0.20034743	0.26859859	0.04486772	0.0287136	0.20745958	0.28125337	0.35885449	[illegible]
ENSG00000223970	0	0	0	0	0	0	0	0	0	0	0	0	0	0	0	0	0	[illegible]
ENSG00000223972	0	0	0.00581016	0	0	0	0	0	0	0	0	0	0	0	0	0	0	[illegible]
ENSG00000223973	0	0	0	0	0	0	0	0	0	0	0	0	0	0	0	0	0	[illegible]
ENSG00000223974	0	0	0	0	0	0.04248916	0	0	0	0	0	0	0	0	0	0	0	[illegible]
ENSG00000223975	0	0	0	0	0	0	0	0	0	0.10425431	0	0	0	0	0	0	0	[illegible]
ENSG00000223976	0	0	0	0	0	0.05811618	0	0	0	0	0	0	0	0	0	0	0	[illegible]
ENSG00000223977	0	0.1998888	0	0	0	0	0	0	0	0	0	0	0	0	0	0	0	[illegible]
ENSG00000223978	0	0	0	0	0	0	0	0	0	0	0	0	0	0	0	0	0	[illegible]
ENSG00000223979	0	0.07655316	0.11177343	0	0.06375332	0.04286583	0.04589165	0.16792174	0	0.05175746	0.50394719	0.19303539	0.04836804	0.12381469	0	0.05512639	0.08596672	[illegible]
ENSG00000223981	0	0	0	0	0	0	0	0	0	0	0	0	0	0	0	0	0	[illegible]
ENSG00000223982	0.09678601	0	0	0	0	0	0	0	0	0	0	0	0	0	0.08417446	0.09336721	0	[illegible]
ENSG00000223983	0	0	0	0	0	0	0	0	0	0	0	0	0	0	0	0	0	[illegible]
ENSG00000223984	0.0190146	0.01273628	0.01859593	0.0491572	0.02121349	0.01426332	0.01527014	0	0.03760261	0.0344439	0	0.01605781	0.01609414	0	0	0.05502882	0	[illegible]
ENSG00000223985	0	0	0	0	0	0	0	0	0	0	0	0	0	0	0	0	0	[illegible]
ENSG00000223986	0	0	0	0	0	0	0	0	0	0	0	0	0	0	0	0	0	[illegible]
ENSG00000223987	0	0	0	0	0	0	0	0	0	0	0	0	0	0	0	0	0	[illegible]
ENSG00000223989	0	0	0	0	0	0	0	0	0	0	0	0	0	0	0	0	0	[illegible]
ENSG00000223991	0	0	0	0	0	0	0.04414992	0.01346238	0.00679493	0.02489655	0.01731503	0.01160682	0	0	0	0	0	[illegible]
ENSG00000223993	0	0	0	0	0	0	0	0	0	0	0	0	0	0	0	0	0	[illegible]
ENSG00000223995	0	0	0.08000027	0	0	0	0	0	0	0	0	0	0	0	0	0	0	[illegible]
ENSG00000223997	0	0	0	0	0	0	0	0	0	0	0	0	0	0	0	0	0	[illegible]
ENSG00000223999	0	0	0	0	0	0	0	0	0	0	0	0	0	0	0	0	0	[illegible]
ENSG00000224000	0	0	0	0	0	0	0	0	0.02421597	0	0	0	0	0	0.04259893	0	0.07368576	[illegible]
ENSG00000224001	0	0	0	0	0	0	0	0	0	0	0	0	0	0	0	0	0	[illegible]
ENSG00000224002	0	0	0	0	0	0	0	0	0	0	0	0	0	0	0	0	0	[illegible]
ENSG00000224003	0.03959427	0.05304175	0.01936124	0.0255901	0.02208653	0.04455098	0.07949291	0.01939145	0.06851274	0	0.02494088	0.050156	0	0	0.034435	0	0.02978208	[illegible]
ENSG00000224004	0.03601088	0.07236198	0	0.0465483	0.08035055	0.05402532	0	0.07054589	0	0	0	0	0	0	0.03131854	0	0	[illegible]
ENSG00000224005	0.51158317	0.42833315	0.62539894	0.661281	0.42805798	0.28781345	1.54064837	2.12967411	1.58076443	2.43260052	1.61125973	0.7560553	0.7577659	1.10843624	1.11230531	1.23378103	1.53921363	[illegible]
ENSG00000224007	0	0	0	0	0	0	0	0	0	0	0	0	0	0	0	0	0	[illegible]
ENSG00000224008	0.04222237	0.05656242	0.04129272	0.0181924	0.02355253	0.03167205	0.01130257	0.04135714	0.04870687	0.08923076	0.05319268	0.01188559	0.02382495	0.01524705	0	0.05430792	0	[illegible]
ENSG00000224011	0.10135138	0	0	0	0	0.07602619	0.08139274	0	0.15032175	0	0	0.08559117	0	0	0	0	0	[illegible]
ENSG00000224012	0	0	0	0	0	0	0	0	0	0	0	0	0	0	0	0	0	[illegible]
ENSG00000224014	1.31306347	1.11937729	0.46696454	0.7714945	1.19856233	0.62679372	0.09586257	0	0	0	0.15038424	0.10080737	0	0.25863512	0	0	0	[illegible]
ENSG00000224015	0	0	0	0	0	0.06345493	0	0	0	0	0	0	0	0	0	0	0	[illegible]
ENSG00000224016	1.77208191	0.96441195	1.40811473	0.4294918	1.66810222	1.41236288	0	0	0	0	0	0	0	0	0.10684289	0.16661591		[illegible]
ENSG00000224017	0.07925346	0.03539015	0.02583615	0	0.04420927	0.03963333	0	0.07762939	0	0.04785444	0.06656352	0	0	0.02861946	0.02297549	0.02548466	0	[illegible]
ENSG00000224018	0	0	0	0	0	0	0	0	0	0	0	0	0	0	0	0	0	[illegible]
ENSG00000224019	0.33783794	0.36206273	0.46255922	0.174678	0.37690639	0.25342064	0.10852366	0	0.06680967	0.30598749	0.08512316	0	0	0	0.0587633	0	0	[illegible]
ENSG00000224020	0.68159571	1.0761385	0.90465563	0.2517263	0.35305084	0.6208423	0.35188223	0.33381421	0.86650725	0.66143217	0.36800978	0.86341058	1.11261096	1.16034186	0.71980603	0.51662251	0.73240528	[illegible]
ENSG00000224023	0.21893079	0.233931	0.18352318	0.2088762	0.14829374	0.23851789	0.20512077	0.16849207	0.22678332	0.31159948	0.21671092	0.24651585	0.04412029	0.0508235	0.04080072	0.03519958	0.0705753	[illegible]
ENSG00000224025	0	0	0	0	0	0	0.00904996	0	0.01114273	0.01020672	0	0.01903356	0.00953831	0	0.00980073	0	0.01695288	[illegible]
ENSG00000224027	0	0	0	0	0	0	0	0	0	0	0	0	0	0	0	0	0	[illegible]
ENSG00000224028	0	0.0486216	0	0	0	0	0.0582948	0	0	0	0	0	0	0	0	0	0	[illegible]
ENSG00000224029	0.10087555	0.03378402	0.09865448	0	0.02813527	0.07566926	0	0	0	0	0	0	0	0	0	0	0	[illegible]
ENSG00000224031	0	0.23920211	0.34925326	0.1154036	0.19920704	0.33485221	0.28679105	0.34979819	0.13241639	0.56603447	0	0.22618829	0.30226673	0.19343901	0.31058276	0.08612543	0.13430811	[illegible]
ENSG00000224032	2.24729073	3.35886907	2.60952093	5.4336567	1.33992178	4.7832538	4.80767522	4.56620687	3.61777039	3.35872696	4.85123502	4.21392633	2.44185186	2.34738946	2.28289661	2.17985361	1.99305399	[illegible]
ENSG00000224033	0	0	0	0	0	0	0	0	0	0	0	0	0	0	0	0	0	[illegible]
ENSG00000224034	0	0.03119652	0	0	0.0259804	0	0.07480605	0.04562036	0.06907849	0.04218382	0.23470373	0.03933236	0.70958425	0.45410646	0.97214198	0.53915518	0.3503268	[illegible]
ENSG00000224035	0	0	0	0	0	0	0	0	0	0	0	0	0	0	0	0	0	[illegible]
ENSG00000224036	0	0	0	0	0	0	0	0	0	0	0	0	0	0	0	0	0	[illegible]
ENSG00000224037	0	0	0	0	0	0	0	0	0	0	0	0	0.02706307	0	0	0	0	[illegible]
ENSG00000224038	0	0	0	0	0	0	0	0	0	0	0	0	0	0	0	0	0	[illegible]
ENSG00000224039	0	0.02961316	0	0	0	0.03316369	0	0.02165246	0	0	0.02784893	0	0.11226162	0.07184309	0.21147533	0.10662305	0.03325462	[illegible]
ENSG00000224040	0	0	0	0	0	0	0	0	0	0	0	0	0	0	0	0	0	[illegible]
ENSG00000224041	0	0	0	0	0	0	0	0	0	0	0	0	0	0	0	0	0	[illegible]
ENSG00000224042	0	0	0	0	0	0	0	0	0	0	0	0	0	0	0	0	0	[illegible]
ENSG00000224043	1.41069105	2.04221019	1.45751287	1.4117287	0.98364187	1.3056754	0.23754155	0.35658919	0.46683048	0.28851175	0.34397807	0.47076652	0.34665182	0.12324653	0.26714173	0.25241775	0.25671671	[illegible]
ENSG00000224045	0	0	0	0	0	0	0	0	0	0	0	0	0	0	0	0	0	[illegible]
ENSG00000224046	1.42660998	0.81905655	1.33825204	1.241918	1.0881257	1.15749364	0.44424116	0.48480386	0.31666696	0.63287168	0.36679083	0.51633045	0.19714235	0.15770434	0.55705697	0.44937716	0.74457895	[illegible]
ENSG00000224048	0	0	0	0	0	0	0.00858186	0.01046727	0	0.00967878	0.01346278	0	0.00904495	0.02315367	0.0092938	0.03092634	0.016076	[illegible]
ENSG00000224049	0	0	0	0	0	0	0	0	0	0	0	0	0	0	0	0	0	[illegible]
ENSG00000224050	0.10925336	0.14635926	0.21369564	0.2824454	0.03047192	0.12293049	0.17547724	0.37455086	0.3240835	0.59371945	0.48173935	0.04613219	0	0	0	0	0	[illegible]
ENSG00000224051	4.20869454	3.77286433	3.38102273	4.1210758	3.79956616	3.44782704	5.11431728	5.21505401	4.38833243	5.3094462	5.61117048	3.74798546	3.40157654	2.86250802	3.44011951	3.3579195	3.24901021	[illegible]
ENSG00000224054	0	0	0	0	0	0	0	0	0	0	0	0	0	0	0	0	0	[illegible]
ENSG00000224055	0	0	0	0	0	0	0	0	0	0	0	0	0	0	0	0	0	[illegible]
ENSG00000224057	0.01148601	0	0.01123311	0	0	0.01723188	0.00922412	0	0	0.01040314	0	0	0.00972187	0	0	0	0	[illegible]
ENSG00000224058	0.20334221	0.23835321	0.54687882	0.3285544	0.05671431	0.26693108	0.24494851	0.39835062	0.25132658	0.50647203	0.06404376	0.12879175	0.2581663	0.11014429	0.08842301	0.19615951	0.3059005	[illegible]
ENSG00000224059	0	0	0.01690086	0	0	0	0.01387823	0	0	0	0.02177145	0	0	0	0	0	0	[illegible]
ENSG00000224060	0	0	0	0	0	0	0	0	0	0	0	0	0	0	0	0	0	[illegible]
ENSG00000224061	0	0	0	0	0	0	0	0	0	0	0	0	0	0	0	0	0	[illegible]
ENSG00000224062	0	0	0	0	0	0	0	0	0	0	0	0	0	0	0	0	0	[illegible]
ENSG00000224063	0.05145781	0.01148908	0.01677494	0.0221717	0.01913617	0.06433297	0.04132447	0.03360222	0.0084801	0.0155355	0.10804616	0.0724268	0.05807253	0.13007461	0.0745878	0.08273359	0.05160748	[illegible]
ENSG00000224064	0	0	0	0	0	0	0	0	0	0	0	0	0	0	0	0	0	[illegible]
ENSG00000224065	0.19415506	0	0.56964048	0	0	0	0	0	0	0	0	0	0	0.21033579	0	0	0	[illegible]
ENSG00000224066	0.31597784	0.42329393	0.26487485	0.4084383	0.17625917	0.32167385	0.725011	0.53057625	0.36822513	0.75635478	0.5686799	0.41932479	0.38206684	0.46456519	0.39257834	0.28304388	0.4074389	[illegible]
ENSG00000224067	0	0.10454233	0	0	0.08706264	0.23415331	0.06267044	0.1528779	0	0.07068089	0	0	0	0	0	0.07528155	0	[illegible]
ENSG00000224069	0	0	0	0	0	0	0	0	0	0	0	0	0	0	0	0	0	[illegible]
ENSG00000224070	0	0	0	0	0	0	0	0	0	0	0	0	0	0	0	0	0	[illegible]
ENSG00000224071	0	0	0	0	0	0	0	0	0	0	0	0	0	0	0	0	0	[illegible]
ENSG00000224072	0.02803805	0.01878033	0.04113107	0.0362425	0.03128044	0.0525801	0.02251665	0.01373175	0	0	0	0	0.02373169	0.03037472	0	0.01352383	0	[illegible]
ENSG00000224074	0	0	0.01213245	0	0	0.00465287	0	0.00607569	0.00306661	0	0.01562885	0	0.00525011	0.00671973	0	0	0	[illegible]
ENSG00000224075	0	0	0	0	0	0	0	0	0	0	0	0	0	0	0	0	0	[illegible]
ENSG00000224076	0	0	0	0	0	0	0	0	0	0	0	0	0	0	0	0	0	[illegible]
ENSG00000224077	0	0	0	0	0	0	0	0	0.03298987	0	0	0	0	0	0	0	0	[illegible]
ENSG00000224078	1.23854316	1.84892201	1.55408607	1.2046115	1.0764229	1.34658218	0.27581577	0.23236682	0.24962137	0.72347858	0.37202034	0.31575811	0.26432649	0.26620443	0.2488115	0.27530127	0.30787316	[illegible]
ENSG00000224079	0	0	0	0	0	0	0	0	0	0	0	0	0	0	0	0	0	[illegible]
ENSG00000224080	0.64892094	0.33806697	0.28205912	0.1864014	0.52286276	0.21634299	0.75274632	0.84749763	1.31893043	0.78365654	0.63585283	0.85246503	1.58673128	0.85922406	1.12872861	2.08666322	1.62702112	[illegible]
ENSG00000224081	0.03533317	0.05916679	0.0172776	0.0304482	0.02299453	0.03092167	0.03310438	0.06345006	0.02037982	0.03200205	0.08160811	0.00994628	0.01993756	0.03189817	0.02048609	0.02840424	0.00885899	[illegible]
ENSG00000224082	0	0	0	0	0	0	0	0	0	0	0	0	0	0	0	0	0	[illegible]
ENSG00000224083	0	0.02825653	0.02062834	0.0272649	0.02353198	0.03164441	0.0508172	0.04132106	0.01042808	0.01910419	0.05314626	0.01781282	0.01785312	0.02285062	0.0366886	0	0.03173117	[illegible]
ENSG00000224085	0	0	0	0	0	0	0	0	0	0	0	0	0	0	0	0	0	[illegible]
ENSG00000224086	0.06686668	0.20358346	0.10700904	0.0628604	0.0610358	0.06383791	0.12692478	0.12503867	0.15627565	0.11011394	0.12253111	0.14373892	0.21095105	0.27000102	0.15860106	0.11728134	0.15546	[illegible]
ENSG00000224087	0	0	0.05453306	0	0.03110456	0	0	0	0	0	0	0	0	0	0	0	0	[illegible]
ENSG00000224089	0	0	0	0	0	0	0	0	0	0	0	0	0	0	0	0	0	[illegible]
ENSG00000224090	0	0	0	0	0	0	0	0	0	0	0	0	0	0	0	0	0	[illegible]
ENSG00000224091	0	0	0	0	0	0	0	0	0	0	0	0	0	0	0	0	0	[illegible]
ENSG00000224092	0	0	0	0	0	0	0	0	0	0	0	0	0	0	0	0	0	[illegible]
ENSG00000224093	0.02343129	0.03923662	0.01145769	0.0302877	0.00653524	0.0087882	0	0.01147557	0.00579211	0	0	0	0	0	0.01018906	0	0	[illegible]
ENSG00000224094	0.72156134	0.75182057	0.86249048	0.6218015	0.5366697	0.66154135	0.70823836	0.39265282	0.71346741	1.08922411	0.30301302	0.94789022	0.4071578	0.43427539	0.41835961	0.69607348	0.24122005	[illegible]
ENSG00000224095	0	0	0	0	0.04114058	0	0	0	0	0	0	0	0	0	0	0	0	[illegible]
ENSG00000224097	0.04107792	0.01650879	0.05624286	0.0212392	0.01374848	0.06162715	0.02639092	0.03218892	0.028432	0.01488208	0.04140071	0.02775222	0.01390751	0.0267008	0.03572533	0.03962692	0	[illegible]
ENSG00000224099	0.09776463	0.1091405	0.06374137	0.0842481	0.03635679	0.04889045	0.10468308	0.09576123	0.06444532	0.11806352	0.08211071	0.05504144	0.05516597	0.10591226	0.08502556	0.06287418	0.0490245	[illegible]
ENSG00000224100	0	0	0	0	0	0	0.05922859	0.07224093	0.03646248	0	0	0	0	0	0	0	0	[illegible]
ENSG00000224101	0	0	0	0	0	0	0	0	0	0	0	0	0	0	0	0	0	[illegible]
ENSG00000224104	0	0	0	0	0	0	0	0	0	0	0	0	0	0	0	0	0	[illegible]
ENSG00000224106	0	0	0	0	0	0	0	0	0	0	0	0	0	0	0	0	0	[illegible]
ENSG00000224107	0	0	0	0	0	0	0	0	0	0	0	0	0	0	0	0	0	[illegible]
ENSG00000224109	0	0.17767893	0.25942475	0.0857216	0.11097799	0	0	0	0.09835868	0	0.0835468	0	0	0	0	0	0	[illegible]
ENSG00000224110	0.05745052	0	0	0	0.01602356	0.02154753	0	0.05627324	0.02840304	0.02601712	0.03618872	0.02425846	0.02431334	0.0622384	0	0	0	[illegible]
ENSG00000224113	2.97723403	3.77033657	2.94762809	3.6923138	2.62441801	3.14709794	1.48010676	1.70241674	1.73411249	1.62173368	1.82577935	1.88902965	1.6399743	1.48468696	1.83579304	2.30474638	2.21967244	[illegible]
ENSG00000224114	1.56310516	1.29627657	1.45589407	3.7523496	0.95497461	3.51757217	1.43461761	1.45816567	1.36157483	1.28090743	1.40659625	1.38290022	1.1340238	1.29018906	0.90628479	1.00526085	1.23172638	[illegible]
ENSG00000224116	0.00449195	0.03008779	0.00878609	0.0116127	0.00501141	0.01010857	0.09739904	0.08799801	0.03775328	0.17494381	0.096204	0.04552138	0.09505078	0.08272719	0.09766583	0.08666559	0.06081771	[illegible]
ENSG00000224117	0	0	0	0	0	0	0	0	0	0	0	0	0	0	0.02737314	0	0	[illegible]
ENSG00000224121	0	0.05189421	0	0	0	0	0	0	0	0	0	0	0	0	0	0	0	[illegible]
ENSG00000224122	0	0	0	0	0	0	0	0	0	0	0	0	0	0	0	0	0	[illegible]
ENSG00000224124	0.02082793	0.02790176	0.01745944	0.0115382	0.00497926	0.02455129	0.48745477	0.46048258	0.11915275	0.08893185	0.25864646	0.08794587	0.00755527	0.01611694	0.01035085	0.00861095	0.01790444	[illegible]
ENSG00000224126	0.06484857	0.0434366	0.12684148	0.0838244	0.07234783	0.04864453	0.05207825	0	0.03206057	0.11746964	0.08169768	0	0	0	0.11279716	0	0	[illegible]
ENSG00000224127	0	0	0	0	0	0	0	0	0	0	0	0	0	0	0	0	0	[illegible]
ENSG00000224128	0.27546786	0.23064092	0.06735066	0.1780372	0.03841546	0.30995294	0.11061065	0	0.03404723	0.06237437	0.08676014	0.1744743	0	0	0	0	0	[illegible]
ENSG00000224129	0	0	0	0	0	0	0	0	0	0	0	0	0	0	0	0	0	[illegible]
ENSG00000224130	0	0	0	0	0	0	0	0	0	0	0	0	0	0	0	0	0	[illegible]
ENSG00000224132	0	0.06167997	0	0	0.05136696	0	0.44370673	0.09019796	0.18210406	0.08340345	0.1160107	0.07776569	0.23382491	0.49879631	0.48051589	0	0.27705845	[illegible]
ENSG00000224134	0	0	0	0	0	0	0	0	0	0	0	0	0	0	0	0	0	[illegible]
ENSG00000224136	0	0	0	0	0	0	0	0	0	0	0	0	0	0	0	0	0	[illegible]
ENSG00000224137	0	0	0	0	0	0	0	0	0	0	0	0	0	0	0	0	0	[illegible]
ENSG00000224138	0	0	0	0	0	0	0	0	0	0	0	0	0	0	0	0	0	[illegible]
ENSG00000224141	0.03010718	0.09074821	0.02944428	0.1556682	0.05038328	0.09033659	0.14506993	0.07372556	0.07442366	0.16361255	0.1137891	0.08898923	0.06370755	0.01630815	0.10473646	0.0871311	0.09058427	[illegible]
ENSG00000224142	0.3447031	0.15392507	0.05618557	0.0742615	0.03204712	0.38785555	0.18454825	0.05627324	0.11361216	0.26017118	0	0.2911015	0.09725338	0.0622384	0.04996452	0.2771059	0	[illegible]
ENSG00000224144	0	0	0	0	0	0	0	0	0	0	0	0	0	0	0	0	0	[illegible]
ENSG00000224149	0	0	0	0	0	0	0	0	0	0	0	0	0	0	0	0	0	[illegible]
ENSG00000224151	0	0	0	0	0	0	0	0	0	0	0	0	0	0	0	0	0	[illegible]
ENSG00000224152	15.2519585	18.9292287	16.203107	22.140962	10.6258086	20.3734931	0.6063596	1.03540499	0.85323189	1.03556488	0.67944687	0.8016008	1.07730574	0.95819639	0.99437414	0.56189064	0.84378578	[illegible]
ENSG00000224153	0	0	0	0	0	0	0	0	0	0	0	0	0	0	0	0	0	[illegible]
ENSG00000224155	0	0	0	0	0	0	0	0	0	0	0	0	0	0	0	0	0	[illegible]
ENSG00000224157	0	0.20908465	0.22895961	0.5043669	0.08706264	0.64392161	0.06267044	0.07643895	0.03858137	0.07068089	0.09831415	0.39541875	0.1981567	0	0	0.22584466	0.2347953	[illegible]
ENSG00000224159	0.34923867	0.13367177	0.24396367	0.1289805	0.13915197	0.11227398	0	0	0	0	0.06285409	0	0	0.05404914	0	0	0	[illegible]
ENSG00000224160	0	0	0	0	0	0	0	0	0	0	0	0	0	0	0	0	0	[illegible]
ENSG00000224161	0	0	0	0	0	0	0	0	0	0	0	0	0	0	0	0	0	[illegible]
ENSG00000224162	0	0	0	0	0	0	0	0	0	0	0	0	0	0	0	0	0	[illegible]
ENSG00000224163	0	0	0	0	0	0	0	0	0	0.02646528	0	0	0	0	0	0	0	[illegible]

ENSG00000224164	0.08437105	0	[illegible]
ENSG00000224165	0.19141326	0.2279318	[illegible]
ENSG00000224166	0	0	[illegible]
ENSG00000224167	0.14962739	0.12026736	[illegible]
ENSG00000224169	0	0	[illegible]
ENSG00000224172	0	0	[illegible]
ENSG00000224173	0	0	[illegible]
ENSG00000224174	0	0.06601832	[illegible]
ENSG00000224177	0	0	[illegible]
ENSG00000224183	0	0	[illegible]
ENSG00000224184	0.39797479	0.36653351	[illegible]
ENSG00000224185	0.15439396	0.18097717	[illegible]
ENSG00000224186	0.01006079	0.00673888	[illegible]
ENSG00000224187	0	0	[illegible]
ENSG00000224188	0	0	[illegible]
ENSG00000224189	0.47833288	0.35353925	[illegible]
ENSG00000224190	0	0	[illegible]
ENSG00000224192	0	0	[illegible]
ENSG00000224194	0	0	[illegible]
ENSG00000224195	0	0	[illegible]
ENSG00000224196	0	0	[illegible]
ENSG00000224198	0	0.09265232	[illegible]
ENSG00000224199	0	0	[illegible]
ENSG00000224201	0	0	[illegible]
ENSG00000224203	0	0	[illegible]
ENSG00000224204	0.10145773	0.16989499	[illegible]
ENSG00000224205	0	0	[illegible]
ENSG00000224207	0	0	[illegible]
ENSG00000224208	0.30215381	0.50596853	[illegible]
ENSG00000224209	0	0	[illegible]
ENSG00000224210	0	0	[illegible]
ENSG00000224215	0	0	[illegible]
ENSG00000224216	0.10815349	0.20525494	[illegible]
ENSG00000224217	0	0	[illegible]
ENSG00000224218	0	0	[illegible]
ENSG00000224219	0	0	[illegible]
ENSG00000224220	0	0	[illegible]
ENSG00000224221	0	0	[illegible]
ENSG00000224222	0	0	[illegible]
ENSG00000224223	0	0	[illegible]
ENSG00000224224	0	0	[illegible]
ENSG00000224226	0.01064215	0	[illegible]
ENSG00000224227	0	0.04602983	[illegible]
ENSG00000224228	0	0	[illegible]
ENSG00000224231	0	0	[illegible]
ENSG00000224232	0	0.09552208	[illegible]
ENSG00000224233	0	0	[illegible]
ENSG00000224235	0	0	[illegible]
ENSG00000224236	0	0	[illegible]
ENSG00000224237	0	0	[illegible]
ENSG00000224238	1.56455047	3.72026051	[illegible]
ENSG00000224239	0	0	[illegible]
ENSG00000224240	0	0	[illegible]
ENSG00000224243	0.05913714	0	[illegible]
ENSG00000224244	0	0	[illegible]
ENSG00000224245	0	0	[illegible]
ENSG00000224247	0	0	[illegible]
ENSG00000224250	0	0	[illegible]
ENSG00000224251	0	0	[illegible]
ENSG00000224252	0	0	[illegible]
ENSG00000224254	0	0	[illegible]
ENSG00000224255	0	0.06115578	[illegible]
ENSG00000224256	0	0	[illegible]
ENSG00000224257	0	0	[illegible]
ENSG00000224259	0.03362518	0.0563067	[illegible]
ENSG00000224260	0	0.01435372	[illegible]
ENSG00000224261	0.07275336	0.07309703	[illegible]
ENSG00000224263	0	0	[illegible]
ENSG00000224265	0	0	[illegible]
ENSG00000224267	0	0	[illegible]
ENSG00000224269	0	0	[illegible]
ENSG00000224271	0.01059492	0.00709664	[illegible]
ENSG00000224272	0.01171139	0.03922237	[illegible]
ENSG00000224273	0	0	[illegible]
ENSG00000224274	0	0	[illegible]
ENSG00000224276	0	0	[illegible]
ENSG00000224277	0	0.0215449	[illegible]
ENSG00000224278	0	0	[illegible]
ENSG00000224279	0	0	[illegible]
ENSG00000224280	0	0.01450806	[illegible]
ENSG00000224281	0.19046701	0.18832917	[illegible]
ENSG00000224282	0.05595441	0	[illegible]
ENSG00000224286	0	0	[illegible]
ENSG00000224287	0	0	[illegible]
ENSG00000224288	0	0	[illegible]
ENSG00000224289	0	0	[illegible]
ENSG00000224291	0	0	[illegible]
ENSG00000224292	0	0.03305971	[illegible]
ENSG00000224294	0.01446577	0.02906821	[illegible]
ENSG00000224295	0.04361264	0.10224353	[illegible]
ENSG00000224296	0	0	[illegible]
ENSG00000224297	0	0	[illegible]
ENSG00000224298	0.02461225	0.03297135	[illegible]
ENSG00000224299	0	0	[illegible]
ENSG00000224300	0	0	[illegible]
ENSG00000224301	0	0	[illegible]
ENSG00000224302	0	0	[illegible]
ENSG00000224303	0	0	[illegible]
ENSG00000224307	0	0	[illegible]
ENSG00000224308	0.01613908	0.07567147	[illegible]
ENSG00000224309	0.00467844	0.01566845	[illegible]
ENSG00000224310	0.04434777	0.08911451	[illegible]
ENSG00000224311	0	0	[illegible]
ENSG00000224312	0.09620818	0.06444176	[illegible]
ENSG00000224314	0	0	[illegible]
ENSG00000224315	0	0.1535419	[illegible]
ENSG00000224316	0	0.02953213	[illegible]
ENSG00000224318	4.7539802	4.51316876	[illegible]
ENSG00000224321	0	0	[illegible]
ENSG00000224322	0	0	[illegible]
ENSG00000224323	0.07006465	0.04693041	[illegible]
ENSG00000224324	0.12301427	0.32958764	[illegible]
ENSG00000224326	0	0	[illegible]
ENSG00000224328	0	0	[illegible]
ENSG00000224329	0	0	[illegible]
ENSG00000224330	0	0	[illegible]
ENSG00000224331	0.1039669	0.13927736	[illegible]
ENSG00000224333	0	0	[illegible]
ENSG00000224334	0.02766501	0.05559139	[illegible]
ENSG00000224335	0	0	[illegible]
ENSG00000224336	0	0	[illegible]
ENSG00000224337	0	0	[illegible]
ENSG00000224338	0	0	[illegible]
ENSG00000224339	0	0	[illegible]
ENSG00000224340	0	0	[illegible]
ENSG00000224341	0	0	[illegible]
ENSG00000224342	0	0	[illegible]
ENSG00000224344	0	0	[illegible]
ENSG00000224346	0	0	[illegible]
ENSG00000224347	0	0	[illegible]
ENSG00000224348	0	0.07124749	[illegible]
ENSG00000224349	0	0.18557012	[illegible]
ENSG00000224351	0	0	[illegible]
ENSG00000224352	0	0	[illegible]
ENSG00000224353	0	0	[illegible]
ENSG00000224354	0	0	[illegible]
ENSG00000224356	0.00982015	0.03946616	[illegible]
ENSG00000224357	0	0	[illegible]
ENSG00000224358	0.32979269	0.84343777	[illegible]
ENSG00000224359	0	0	[illegible]
ENSG00000224361	0	0	[illegible]
ENSG00000224363	0	0	[illegible]
ENSG00000224365	0	0	[illegible]
ENSG00000224366	0	0	[illegible]
ENSG00000224367	0.13688571	0.04584411	[illegible]
ENSG00000224368	0	0	[illegible]
ENSG00000224370	0	0	[illegible]
ENSG00000224371	0	0	[illegible]
ENSG00000224372	0	0	[illegible]
ENSG00000224373	0	0	[illegible]
ENSG00000224374	0	0	[illegible]
ENSG00000224375	0	0	[illegible]
ENSG00000224376	0.1418676	0.2505209	[illegible]
ENSG00000224382	0	0	[illegible]
ENSG00000224383	0	0.00550258	[illegible]
ENSG00000224384	0	0	[illegible]
ENSG00000224387	0.21250378	0.2846768	[illegible]
ENSG00000224388	0.16874209	0.39559145	[illegible]
ENSG00000224389	0.04828667	0.00754674	[illegible]
ENSG00000224391	0.05444213	0.0546993	[illegible]
ENSG00000224394	0	0	[illegible]
ENSG00000224396	0	0	[illegible]
ENSG00000224397	0.01501502	0.0100573	[illegible]
ENSG00000224400	0	0	[illegible]
ENSG00000224401	0	0	[illegible]
ENSG00000224402	0	0	[illegible]
ENSG00000224403	0	0	[illegible]
ENSG00000224404	0	0	[illegible]
ENSG00000224405	0	0	[illegible]
ENSG00000224406	0	0	[illegible]
ENSG00000224407	0	0	[illegible]
ENSG00000224408	0	0	[illegible]
ENSG00000224409	0	0	[illegible]
ENSG00000224410	0	0	[illegible]
ENSG00000224411	0.03993772	0.04681411	[illegible]
ENSG00000224412	0	0	[illegible]
ENSG00000224413	0.02932642	0.04910826	[illegible]
ENSG00000224414	0.03370137	0.02257371	[illegible]
ENSG00000224415	0.43627397	0.36527903	[illegible]
ENSG00000224416	0.11308681	0	[illegible]
ENSG00000224417	0	0	[illegible]

ENSG00000224418	0.40350222	[illegible]
ENSG00000224419	0	[illegible]
ENSG00000224420	0.54404101	[illegible]
ENSG00000224421	0	[illegible]
ENSG00000224424	0.3100078	[illegible]
ENSG00000224425	0	[illegible]
ENSG00000224426	0.05684257	[illegible]
ENSG00000224427	0.10199285	[illegible]
ENSG00000224429	0	[illegible]
ENSG00000224430	0.17575864	[illegible]
ENSG00000224431	0.01812696	[illegible]
ENSG00000224435	0	[illegible]
ENSG00000224436	0	[illegible]
ENSG00000224437	0	[illegible]
ENSG00000224438	0	[illegible]
ENSG00000224439	0	[illegible]
ENSG00000224440	0	[illegible]
ENSG00000224441	0.21813699	[illegible]
ENSG00000224442	0.05781119	[illegible]
ENSG00000224443	0	[illegible]
ENSG00000224445	0	[illegible]
ENSG00000224447	0	[illegible]
ENSG00000224448	0	[illegible]
ENSG00000224451	0.05664278	[illegible]
ENSG00000224452	0	[illegible]
ENSG00000224458	0	[illegible]
ENSG00000224459	0	[illegible]
ENSG00000224460	0.08017348	[illegible]
ENSG00000224462	0.01702575	[illegible]
ENSG00000224463	0	[illegible]
ENSG00000224464	0.34516455	[illegible]
ENSG00000224465	0	[illegible]
ENSG00000224466	0	[illegible]
ENSG00000224467	0	[illegible]
ENSG00000224468	0	[illegible]
ENSG00000224469	0	[illegible]
ENSG00000224470	5.92756238	[illegible]
ENSG00000224471	0	[illegible]
ENSG00000224473	0	[illegible]
ENSG00000224474	0.95596686	[illegible]
ENSG00000224475	0	[illegible]
ENSG00000224476	0	[illegible]
ENSG00000224477	0.07784961	[illegible]
ENSG00000224478	0	[illegible]
ENSG00000224479	0.01736048	[illegible]
ENSG00000224481	0	[illegible]
ENSG00000224482	0	[illegible]
ENSG00000224484	0	[illegible]
ENSG00000224485	0	[illegible]
ENSG00000224486	0	[illegible]
ENSG00000224488	0	[illegible]
ENSG00000224490	0	[illegible]
ENSG00000224493	0	[illegible]
ENSG00000224494	0	[illegible]
ENSG00000224497	0	[illegible]
ENSG00000224498	0	[illegible]
ENSG00000224500	0	[illegible]
ENSG00000224503	0.29568569	[illegible]
ENSG00000224504	0	[illegible]
ENSG00000224505	0.71047523	[illegible]
ENSG00000224506	0	[illegible]
ENSG00000224509	0	[illegible]
ENSG00000224510	0	[illegible]
ENSG00000224511	0.0104034	[illegible]
ENSG00000224512	0	[illegible]
ENSG00000224513	0	[illegible]
ENSG00000224514	0	[illegible]
ENSG00000224515	0	[illegible]
ENSG00000224516	0	[illegible]
ENSG00000224517	0	[illegible]
ENSG00000224518	0	[illegible]
ENSG00000224519	0	[illegible]
ENSG00000224520	0	[illegible]
ENSG00000224521	0	[illegible]
ENSG00000224523	0	[illegible]
ENSG00000224524	0	[illegible]
ENSG00000224525	0.9677337	[illegible]
ENSG00000224529	0	[illegible]
ENSG00000224530	0.14074122	[illegible]
ENSG00000224531	5.61319706	[illegible]
ENSG00000224532	0	[illegible]
ENSG00000224533	0.035781	[illegible]
ENSG00000224535	0	[illegible]
ENSG00000224536	0	[illegible]
ENSG00000224537	0	[illegible]
ENSG00000224539	0	[illegible]
ENSG00000224540	0	[illegible]
ENSG00000224541	0	[illegible]
ENSG00000224543	0.08364119	[illegible]
ENSG00000224545	0	[illegible]
ENSG00000224546	0	[illegible]
ENSG00000224547	0	[illegible]
ENSG00000224548	0	[illegible]
ENSG00000224549	0	[illegible]
ENSG00000224550	0.2620304	[illegible]
ENSG00000224551	0	[illegible]
ENSG00000224553	0.09261419	[illegible]
ENSG00000224555	0	[illegible]
ENSG00000224556	0	[illegible]
ENSG00000224557	0.02700062	[illegible]
ENSG00000224559	0.26583119	[illegible]
ENSG00000224560	0	[illegible]
ENSG00000224563	0	[illegible]
ENSG00000224565	0	[illegible]
ENSG00000224566	0.13513518	[illegible]
ENSG00000224567	0	[illegible]
ENSG00000224568	0	[illegible]
ENSG00000224569	0	[illegible]
ENSG00000224570	0	[illegible]
ENSG00000224571	0	[illegible]
ENSG00000224573	0	[illegible]
ENSG00000224574	0	[illegible]
ENSG00000224577	0.04610835	[illegible]
ENSG00000224578	0.71621644	[illegible]
ENSG00000224579	0	[illegible]
ENSG00000224581	0	[illegible]
ENSG00000224582	0	[illegible]
ENSG00000224583	0	[illegible]
ENSG00000224584	0	[illegible]
ENSG00000224585	0	[illegible]
ENSG00000224586	0	[illegible]
ENSG00000224589	0	[illegible]
ENSG00000224590	0	[illegible]
ENSG00000224592	0.05528257	[illegible]
ENSG00000224593	0.05985096	[illegible]
ENSG00000224594	0	[illegible]
ENSG00000224595	0.05537756	[illegible]
ENSG00000224596	0.00985167	[illegible]
ENSG00000224597	2.28359883	[illegible]
ENSG00000224598	0	[illegible]
ENSG00000224599	0	[illegible]
ENSG00000224600	0	[illegible]
ENSG00000224602	0	[illegible]
ENSG00000224603	0	[illegible]
ENSG00000224604	0	[illegible]
ENSG00000224605	0	[illegible]
ENSG00000224606	0	[illegible]
ENSG00000224607	0	[illegible]
ENSG00000224609	0	[illegible]
ENSG00000224610	0	[illegible]
ENSG00000224611	0	[illegible]
ENSG00000224612	0	[illegible]
ENSG00000224613	0	[illegible]
ENSG00000224614	0.03075357	[illegible]
ENSG00000224615	0.61980269	[illegible]
ENSG00000224616	0.07619324	[illegible]
ENSG00000224617	0	[illegible]
ENSG00000224618	0	[illegible]
ENSG00000224620	0	[illegible]
ENSG00000224621	0	[illegible]
ENSG00000224622	0	[illegible]
ENSG00000224623	0	[illegible]
ENSG00000224625	0	[illegible]
ENSG00000224626	0	[illegible]
ENSG00000224627	0	[illegible]
ENSG00000224628	0	[illegible]
ENSG00000224629	0	[illegible]
ENSG00000224630	0	[illegible]
ENSG00000224631	0.30001088	[illegible]
ENSG00000224632	0	[illegible]
ENSG00000224634	0	[illegible]
ENSG00000224635	0.09649623	[illegible]
ENSG00000224637	0	[illegible]
ENSG00000224638	0	[illegible]
ENSG00000224640	0	[illegible]
ENSG00000224643	0	[illegible]
ENSG00000224644	0	[illegible]
ENSG00000224645	0	[illegible]
ENSG00000224646	0	[illegible]
ENSG00000224647	0.04985265	[illegible]
ENSG00000224648	0	[illegible]
ENSG00000224649	0	[illegible]
ENSG00000224650	0	[illegible]
ENSG00000224652	0	[illegible]
ENSG00000224653	0	[illegible]
ENSG00000224655	0	[illegible]
ENSG00000224656	0	[illegible]
ENSG00000224657	0	[illegible]
ENSG00000224658	0	[illegible]
ENSG00000224659	0	[illegible]
ENSG00000224660	0.44086082	[illegible]

ENSG00000224661	0	0	0	0	0	0	0	0	[illegible]
ENSG00000224662	0	0	0	0	0	0	0	0	[illegible]
ENSG00000224664	0	0	0	0	0	0	0	0	[illegible]
ENSG00000224666	0.10103367	0	0.19761822	0	0	0	0	0	[illegible]
ENSG00000224667	0	0.18690901	0.06822534	0.0901747	0	0.05232972	0.05602358	0.06833179	[illegible]
ENSG00000224668	0.15549891	0.1597053	0.14193679	0.1206003	0.18504661	0.08553864	0.02497545	0	[illegible]
ENSG00000224669	0	0.0908968	0.3317906	0.1754135	0.03784934	0.05089754	0	0	[illegible]
ENSG00000224670	0	0	0	0	0	0	0	0	[illegible]
ENSG00000224671	0	0	0	0	0	0	0	0.04260363	[illegible]
ENSG00000224672	0	0.07779456	0	0.0750643	0	0.08712191	0	0.1137632	[illegible]
ENSG00000224674	0.05330166	0.01190077	0.01737602	0.0459324	0.01982187	0.0399829	0.02853682	0.01740313	[illegible]
ENSG00000224675	0.04093532	0.02741912	0.01334467	0.0705516	0.03805765	0.04094213	0.0547902	0.12028941	[illegible]
ENSG00000224676	0	0	0	0	0	0	0	0	[illegible]
ENSG00000224677	0	0	0	0	0	0	0	0	[illegible]
ENSG00000224678	0	0	0	0	0	0	0	0	[illegible]
ENSG00000224679	0	0	0	0	0	0	0	0	[illegible]
ENSG00000224680	0.0567425	0.11402108	0	0	0.09495652	0	0.31897931	0.38905811	[illegible]
ENSG00000224681	0	0	0	0	0	0	0	0	[illegible]
ENSG00000224682	0	0	0	0	0	0	0	0	[illegible]
ENSG00000224683	0	0	0	0	0	0	0	0	[illegible]
ENSG00000224685	0	0	0	0	0	0	0	0	[illegible]
ENSG00000224686	0	0	0	0	0	0	0	0	[illegible]
ENSG00000224687	0.11210344	0.21118686	0.15074834	0.1358501	0.11334231	0.16818316	0.40512354	0.26765265	[illegible]
ENSG00000224688	0	0	0	0	0	0	0	0	[illegible]
ENSG00000224689	2.13819869	2.18399905	1.87648884	1.8317741	1.692911	1.76383726	0	0.00614188	[illegible]
ENSG00000224690	0	0	0	0	0	0	0	0	[illegible]
ENSG00000224691	0.63911665	1.34542717	1.42867339	0.5900949	0.71302575	1.16429915	0.73322642	1.1626083	[illegible]
ENSG00000224692	0	0	0	0	0	0	0	0	[illegible]
ENSG00000224695	0	0	0	0	0	0	0	0	[illegible]
ENSG00000224697	0	0	0	0	0	0	0	0	[illegible]
ENSG00000224698	0	0	0	0	0	0	0	0	[illegible]
ENSG00000224699	0.0320459	0.03954051	0.04288674	0.0348826	0.0235209	0.05693311	0.04605256	0.04130159	[illegible]
ENSG00000224700	0	0	0	0	0	0	0	0	[illegible]
ENSG00000224701	0	0	0	0	0	0	0	0	[illegible]
ENSG00000224702	0	0	0	0	0	0	0	0	[illegible]
ENSG00000224706	0	0	0	0	0	0	0	0	[illegible]
ENSG00000224707	0.08057435	0.16190993	0.2364008	0	0.13483826	0.30220412	0.12941446	0.31569287	[illegible]
ENSG00000224709	0	0.02412066	0	0	0	0	0	0	[illegible]
ENSG00000224710	0	0	0	0	0	0	0	0	[illegible]
ENSG00000224711	0	0	0	0	0	0	0	0	[illegible]
ENSG00000224712	0.061157	0.06144589	0.05981045	0.0395263	0.0568578	0.06881308	0.06548486	0.04991981	[illegible]
ENSG00000224713	0.00624244	0	0	0	0	0.00936523	0	0.00611452	[illegible]
ENSG00000224714	0.05012401	0.06714772	0.06127548	0.0485934	0.02097018	0.03759927	0.02012667	0.01227422	[illegible]
ENSG00000224715	0	0	0	0	0	0.01661984	0	0	[illegible]
ENSG00000224717	0.03351446	0.05237987	0.06555308	0.0433214	0.04985355	0.05028006	0.05382924	0.03282768	[illegible]
ENSG00000224718	0	0	0	0	0	0	0	0	[illegible]
ENSG00000224719	0	0	0	0	0	0	0	0	[illegible]
ENSG00000224721	0	0	0	0	0	0	0	0	[illegible]
ENSG00000224722	0	0.01729807	0	0	0.02881159	0.03874412	0	0	[illegible]
ENSG00000224723	0	0	0	0	0	0	0	0	[illegible]
ENSG00000224725	0	0	0.01610634	0	0	0.01235377	0	0	[illegible]
ENSG00000224727	0	0	0	0	0	0	0.04638511	0.11315157	[illegible]
ENSG00000224728	0.1902083	0.24065301	0.24802707	0.2731849	0.17683707	0.25365329	1.06926048	1.36627733	[illegible]
ENSG00000224729	0.06951058	0.08277207	0.03776672	0.049917	0.02154138	0.09269621	0.11784686	0.12104208	[illegible]
ENSG00000224730	0	0	0	0	0	0	0	0	[illegible]
ENSG00000224731	0	0	0	0	0	0	0	0	[illegible]
ENSG00000224732	0	0	0	0	0	0	0	0	[illegible]
ENSG00000224733	0	0	0	0	0	0	0	0.05750325	[illegible]
ENSG00000224735	0	0	0	0	0	0	0	0	[illegible]
ENSG00000224738	0.36131995	0.54857375	0.56538308	0.4981848	0.29560954	0.27103508	0.23213357	0.25953823	[illegible]
ENSG00000224739	0	0	0	0	0	0	0	0	[illegible]
ENSG00000224743	0.00209583	0.00561529	0.00409938	0.0081273	0.0046764	0	0.01346489	0.01231732	[illegible]
ENSG00000224745	0.32294328	0.51915007	0.25266619	0.5844187	0.43234713	0.53294514	1.40047716	1.20203697	[illegible]
ENSG00000224746	0	0	0	0	0	0	0.03219265	0	[illegible]
ENSG00000224747	0	0	0	0	0	0	0	0	[illegible]
ENSG00000224750	0	0	0	0	0	0	0.09782951	0.04261513	[illegible]
ENSG00000224751	0	0	0	0	0	0	0	0	[illegible]
ENSG00000224752	0	0	0	0	0	0	0	0	[illegible]
ENSG00000224755	0	0	0	0	0	0	0	0	[illegible]
ENSG00000224758	0	0	0	0	0	0	0	0	[illegible]
ENSG00000224760	0	0	0	0	0	0	0	0	[illegible]
ENSG00000224761	0	0.10930628	0.11969661	0.1582052	0	0.18361769	0	0	[illegible]
ENSG00000224762	0	0	0	0	0	0	0	0	[illegible]
ENSG00000224763	0.50312725	0.6938289	0.37627308	0.3443033	0.41272808	0.48841069	0.07130273	0	[illegible]
ENSG00000224764	0	0	0	0	0	0	0	0	[illegible]
ENSG00000224765	0.03621319	0	0	0	0	0.02716442	0	0	[illegible]
ENSG00000224769	0.11510621	0.15419993	0.16885771	0.0991921	0.13911884	0.17268807	0.21569077	0.26307739	[illegible]
ENSG00000224771	0	0.04257986	0	0	0.03546042	0	0	0	[illegible]
ENSG00000224773	0.01654504	0	0.01618075	0	0	0.01241085	0	0	[illegible]
ENSG00000224775	0.07154215	0.19966695	0.12827272	0.0616511	0.09976934	0.03577703	0.02872685	0.0233587	[illegible]
ENSG00000224776	0	0	0	0	0	0	0	0	[illegible]
ENSG00000224777	0	0	0	0	0	0	0	0	[illegible]
ENSG00000224778	0	0	0	0	0	0	0	0	[illegible]
ENSG00000224781	0.05389589	0.01805016	0.05270921	0.1393335	0.04509641	0.08085729	0.06492364	0.05279145	[illegible]
ENSG00000224783	0	0	0	0	0	0	0	0	[illegible]
ENSG00000224784	0	0	0	0	0	0	0	0	[illegible]
ENSG00000224785	0.01787562	0	0	0	0	0.02681789	0	0	[illegible]
ENSG00000224786	0.41232076	0.32220881	1.07531281	1.2436031	0.72833745	0.82477883	0.33112443	0.40387147	[illegible]
ENSG00000224788	0	0	0	0	0	0	0	0	[illegible]
ENSG00000224789	0	0.03754433	0.05481758	0.1449068	0	0.12613737	0	0.05490311	[illegible]
ENSG00000224790	0.29433552	0.5914518	0.71963714	0.8560418	0.49255986	0.2207884	0.82730707	0.50453198	[illegible]
ENSG00000224791	0	0	0	0	0	0	0.01992524	0	[illegible]
ENSG00000224792	0	0	0	0	0	0	0	0	[illegible]
ENSG00000224794	0.01812696	0.04856691	0.01772784	0.0234312	0.02022321	0.04079246	0.04367192	0.03551101	[illegible]
ENSG00000224795	0	0	0	0	0.02557388	0.03439023	0	0	[illegible]
ENSG00000224796	0	0	0	0	0	0	0	0	[illegible]
ENSG00000224797	0	0	0	0	0.06784315	0	0	0	[illegible]
ENSG00000224799	0	0.06704345	0	0	0	0.03754088	0	0	[illegible]
ENSG00000224800	0.14388277	0.04818748	0.14071476	0.0929926	0.04013044	0	0.11554863	0.21140147	[illegible]
ENSG00000224802	0.04038815	0.05410524	0.11849664	0.0522064	0	0.09088846	0.0324347	0.03956051	[illegible]
ENSG00000224804	0	0	0	0	0	0	0	0	[illegible]
ENSG00000224805	0	0	0.04768549	0	0	0	0.19578587	0.14327967	[illegible]
ENSG00000224806	0	0	0	0	0	0	0	0	[illegible]
ENSG00000224807	0	0	0	0.0330903	0	0	0	0.02507489	[illegible]
ENSG00000224808	0	0	0	0	0	0	0	0	[illegible]
ENSG00000224809	0.12869018	0.04788818	0.06992038	0.036966	0.03988118	0.0643558	0	0	[illegible]
ENSG00000224810	0.23554012	0.48207043	0.42231568	0.4059508	0.29197621	0.3435532	0.13661291	0.24353084	[illegible]
ENSG00000224812	0.01953318	0.01308363	0.02865464	0.0757467	0.00544801	0.01465232	0.0156866	0	[illegible]
ENSG00000224813	0	0	0	0	0	0	0	0	[illegible]
ENSG00000224814	0	0.03256107	0	0	0	0	0	0	[illegible]
ENSG00000224815	0	0	0	0	0	0	0	0	[illegible]
ENSG00000224816	0	0	0	0	0	0	0	0	[illegible]
ENSG00000224817	0	0	0	0	0	0	0	0	[illegible]
ENSG00000224818	3.17985727	2.77972454	3.90048142	1.532668	1.41302081	2.70871918	2.20740389	2.11165798	[illegible]
ENSG00000224819	0	0	0	0	0	0	0	0	[illegible]
ENSG00000224820	0.13022117	0	0.06367698	0.2524891	0.07264014	0.29304642	0.05228867	0	[illegible]
ENSG00000224821	0	0	0	0	0	0	0	0	[illegible]
ENSG00000224822	0.20212494	0.14769435	0.19767456	0	0.22549918	0.16540248	0	0	[illegible]
ENSG00000224825	2.02461863	3.48193396	2.91654637	2.899981	2.48767819	3.28371197	0.43943791	0.42878488	[illegible]
ENSG00000224826	0.01719837	0.09215791	0.13455755	0	0.06715531	0.07740554	0.02762315	0.05053781	[illegible]
ENSG00000224827	0.0412145	0.13803063	0.08061408	0.1598237	0.18392261	0.15458011	0.19858997	0.04036993	[illegible]
ENSG00000224828	0	0	0	0	0	0	0	0	[illegible]
ENSG00000224829	0.30191794	0.15167206	0.14763516	0.4878302	0.25262438	0.28309519	0.06061567	0.22179827	[illegible]
ENSG00000224830	0	0	0	0	0.02616948	0.03519116	0.11302573	0.09190477	[illegible]
ENSG00000224831	0	0	0	0	0	0.01070223	0	0	[illegible]
ENSG00000224832	0	0	0	0	0	0	0	0	[illegible]
ENSG00000224834	0.11388601	0.07628265	0.11137847	0	0.12705608	0	0.09145898	0.11155225	[illegible]
ENSG00000224836	0	0	0	0	0	0	0	0	[illegible]
ENSG00000224837	0	0	0	0	0.03444145	0	0	0	[illegible]
ENSG00000224838	0.25783792	1.3816314	0.33621447	0.4443808	0.43148244	1.16046381	0.41412628	0.33673906	[illegible]
ENSG00000224839	0	0	0	0	0.03610128	0.04854685	0	0	[illegible]
ENSG00000224842	0	0	0.04574761	0.0604655	0	0.07017802	0	0	[illegible]
ENSG00000224843	0.02620304	0.05850404	0.06406526	0.0790311	0.03166933	0.0262074	0.13327235	0.12405275	[illegible]
ENSG00000224844	0	0	0	0	0	0	0	0.04718877	[illegible]
ENSG00000224846	0	0	0.03839233	0.0507439	0.06569465	0	0.18915634	0.03845224	[illegible]
ENSG00000224848	0	0	0	0	0	0	0	0	[illegible]
ENSG00000224849	0.96496227	4.05044134	1.32120207	1.8294121	0.2870808	1.68896513	0	0.1260251	[illegible]
ENSG00000224850	0.06742623	0	0.06594165	0	0.03761179	0.15173428	0	0	[illegible]
ENSG00000224851	0	0	0	0	0	0	0	0	[illegible]
ENSG00000224852	0	0	0	0	0	0	0	0	[illegible]
ENSG00000224853	0.04202052	0.02814601	0.12328595	0.0543164	0.02343994	0	0	0	[illegible]
ENSG00000224854	0	0	0	0	0	0	0	0	[illegible]
ENSG00000224855	0	0	0	0	0	0	0	0	[illegible]
ENSG00000224856	0	0.20113035	0	0.2587621	0.05583365	0.2252453	0.16076331	0.49020632	[illegible]
ENSG00000224857	0	0	0	0	0	0	0	0	[illegible]
ENSG00000224858	0.06714529	0	0	0.0867931	0.01872754	0.02518368	0.13480673	0.06576935	[illegible]
ENSG00000224860	0	0	0	0	0	0	0	0	[illegible]
ENSG00000224861	0.87288879	0.71959969	1.08350367	0.694345	0.99255943	0.90661235	0.48530424	0.55903945	[illegible]
ENSG00000224863	0	0	0	0	0	0	0	0	[illegible]
ENSG00000224864	0	0	0	0	0	0	0	0	[illegible]
ENSG00000224865	0.01802558	0.02414764	0.01762869	0.0466003	0	0.01352144	0	0.0176562	[illegible]
ENSG00000224866	0	0	0	0	0	0	0	0	[illegible]
ENSG00000224867	0	0	0	0	0	0	0	0	[illegible]
ENSG00000224869	0	0.1353479	0	0	0	0	0	0	[illegible]
ENSG00000224870	1.27793657	1.28497557	1.2293105	1.3965562	1.19700417	1.06637166	1.95401539	1.98755469	[illegible]
ENSG00000224871	0	0	0	0	0	0	0	0	[illegible]
ENSG00000224873	0	0	0	0	0	0	0	0	[illegible]
ENSG00000224875	0.05331636	0.07142429	0.02607122	0.1033764	0.0446115	0.01999696	0.12845108	0	[illegible]
ENSG00000224876	0	0	0	0	0	0	0	0	[illegible]
ENSG00000224877	3.50784689	2.70878772	2.80785699	2.3393531	3.22178541	2.51400306	4.94331849	4.69435842	[illegible]
ENSG00000224879	0	0	0	0	0	0	0	0	[illegible]
ENSG00000224880	0	0	0	0	0	0	0	0	[illegible]
ENSG00000224881	0.03629475	0.0243108	0	0.0469152	0.02024599	0	0	0	[illegible]
ENSG00000224882	0	0	0	0	0	0	0	0	[illegible]
ENSG00000224883	0	0	0	0	0	0.04694433	0	0	[illegible]
ENSG00000224884	0	0.1154438	0.39329901	0.0742615	0.16023561	0.34476049	0.13841119	0.22509295	[illegible]
ENSG00000224885	0.22444108	0.30066839	0.04389987	0.0580233	0.15023762	0.20203061	0.0360486	0.2638102	[illegible]
ENSG00000224886	0.39464987	0.48462836	0.38596049	0.4251092	0.29352547	0.39471558	1.69031136	1.6106779	[illegible]
ENSG00000224887	0	0	0	0	0	0	0	0	[illegible]
ENSG00000224888	0	0.00712475	0.02080535	0.0068747	0.00296674	0	0.0042711	0.01041891	[illegible]
ENSG00000224891	0	0.02785547	0.04067111	0	0	0	0	0	[illegible]
ENSG00000224892	0.29478421	0.31028035	0.28829366	0.3084634	0.12528526	0.263244	0.37201022	0.27499379	[illegible]
ENSG00000224893	0	0	0	0	0	0	0	0	[illegible]
ENSG00000224895	0.17075359	0.17156019	0.25049091	0	0.14287498	0.25617303	0.06856395	0.08362725	[illegible]
ENSG00000224896	0	0	0	0	0	0	0	0	[illegible]
ENSG00000224897	0.2710577	0.30405621	0.32257886	0.1730762	0.17670566	0.17393043	0.11277377	0.07357317	[illegible]
ENSG00000224899	0	0	0	0	0	0	0	0	[illegible]
ENSG00000224901	0	0	0	0	0	0	0	0	[illegible]
ENSG00000224902	0	0	0	0	0	0	0	0	[illegible]
ENSG00000224903	0.13030892	0.37822625	0.55223906	0.224586	0.24229697	0.68423574	0.31394344	0.08509242	[illegible]
ENSG00000224904	0	0	0	0.0157985	0	0.00916812	0	0	[illegible]

ENSG00000224905	0.67565474	0.50660178	0.60160404	0.5865869	0.46689928	0.46900264	0.10528085	0.15804399	0.23432508	0.09133669	0.11434096	0.19587415	0.29020822	0.26219581	0.35958506	0.3404865	0.43994732	0.23112048	0.27745133
ENSG00000224906	0	0.03151531	0	0	0	0	0	0	0.02326147	0.0426149	0.05927554	0.03973429	0	0	0	0	0	0	0
ENSG00000224907	0	0	0	0	0	0	0	0	0	0	0	0	0	0	0	0	0	0	0
ENSG00000224908	0.12840534	0	0	0	0.07162723	0	0	0	0	0	0	0	0	0	0	0	0	0	0
ENSG00000224910	0	0	0	0	0	0	0	0	0	0	0	0	0	0	0	0	0	0	0
ENSG00000224911	0	0	0	0	0	0	0	0	0	0.05791906	0	0	0	0	0	0	0	0.0771367	0
ENSG00000224914	3.84364405	4.52513706	3.76860417	3.6375484	2.87698717	3.32452111	5.20492608	5.58969423	4.41617587	5.95013939	5.50936123	3.88355267	1.88392545	2.15634181	2.06366982	2.1849973	2.28634334	2.21173711	2.98905319
ENSG00000224916	0	0	0	0	0	0	0	0	0	0	0	0	0	0	0	0	0	0	0
ENSG00000224917	0	0	0	0	0	0	0	0	0	0	0	0	0	0	0	0	0	0	0
ENSG00000224918	0	0	0	0	0	0	0	0	0	0	0	0	0	0	0	0	0	0	0
ENSG00000224919	0	0	0	0	0	0	0	0	0	0	0	0	0	0	0	0	0	0	0
ENSG00000224920	0.02216626	0	0	0	0	0	0	0	0	0	0	0	0	0	0.01927792	0	0	0	0
ENSG00000224922	0	0	0	0	0	0	0	0	0	0	0	0	0	0	0	0	0	0	0
ENSG00000224924	0.05518116	0.17424574	0.09636819	0.224174	0.04177452	0.24244332	0	0	0	0	0	0	0	0	0	0	0	0	0.0216885
ENSG00000224927	0	0	0	0	0	0	0	0	0	0	0	0	0	0	0	0	0	0	0
ENSG00000224928	0	0	0	0	0	0	0	0	0	0	0	0	0.04196857	0	0	0	0	0	0
ENSG00000224930	0	0	0	0	0	0	0.0352628	0	0	0	0	0	0	0	0	0	0	0	0
ENSG00000224931	0	0	0	0	0	0	0	0	0	0	0	0	0	0	0	0	0	0	0
ENSG00000224932	0	0	0	0	0	0	0	0	0	0	0	0	0	0	0	0	0	0	0
ENSG00000224933	0	0	0	0	0	0	0	0	0	0	0	0	0.09848221	0	0	0	0	0	0
ENSG00000224934	0.3977653	0.5400598	0.4626033	0.5280543	0.40778305	1.07253229	0.34533546	0.43173474	0.19133682	0.46737088	0.24378499	0.31775506	0.26387845	0.18634152	0.28983753	0.28000821	0.09703515	0.3501255	0.36676142
ENSG00000224935	0	0	0	0	0	0	0	0	0	0	0	0	0	0	0	0	0	0	0
ENSG00000224936	0	0	0	0.0304093	0	0	0	0	0.01163073	0	0.02963777	0	0.03982419	0.02548594	0	0.02269437	0	0	0
ENSG00000224937	0.14852415	0.09948383	0	0.1919848	0	0.11141166	0	0	0	0	0	0	0	0	0	0	0	0	0.16345336
ENSG00000224939	0.01419812	0.05706077	0.04165653	0.0183527	0.01584003	0	0.06841293	0.02781435	0.0701943	0.03857869	0.10732267	0.09592243	0.19227892	0.15381384	0.08643641	0.0136966	0.14951392	0.01712639	0
ENSG00000224940	0.00196451	0.00394758	0.00192125	0	0	0.00294725	0.01419883	0.01154552	0.00874113	0.0088965	0.00989973	0.00995416	0	0	0	0	0	0	0.01297186
ENSG00000224942	0	0	0	0	0	0	0	0	0	0	0	0	0	0	0	0	0	0	0
ENSG00000224943	0	0	0	0	0	0	0	0	0	0	0	0	0	0	0	0	0	0	0
ENSG00000224944	0	0.05890311	0.12900453	0.1136718	0.07358159	0.13193086	0.0353109	0	0.02173821	0.11947288	0	0.07426464	0.03721633	0.14290208	0.11472071	0	0	0.05303806	0
ENSG00000224945	2.29029829	2.68001595	3.10343516	2.4967887	3.10928414	2.25618142	0.66480032	0.51354148	0.68211068	2.94911159	0.90385049	1.04863834	5.6521032	5.91893563	6.95952671	7.10733927	7.8456407	9.45294431	0.4858819
ENSG00000224946	0	0	0	0	0	0	0	0	0	0	0	0	0	0	0	0	0	0	0
ENSG00000224947	0	0	0	0	0	0	0	0	0	0	0	0	0	0	0	0	0	0	0
ENSG00000224948	0	0	0	0	0	0	0	0	0	0	0	0	0	0	0	0	0	0	0
ENSG00000224949	0	0	0	0	0	0	0	0	0	0	0	0	0	0	0	0	0	0	0
ENSG00000224950	0.05617384	0.08465879	0.06867126	0.0907641	0.00783374	0.0210687	0.33833847	0.22009089	0.06248669	0.05087792	0.15923037	0.07115815	0.10697872	0.13692448	0.04885419	0.0270948	0.12675877	0.10163895	0.0463652
ENSG00000224953	0.14716776	0	0	0	0	0	0	0	0	0	0	0	0	0	0	0	0	0	0
ENSG00000224955	0	0	0	0	0	0	0.04141263	0.05051086	0	0.09341186	0	0.04354879	0	0	0	0	0	0	0
ENSG00000224956	0.13037921	0.15719411	0.10200682	0.1011182	0.08727396	0.05868041	0.14658596	0.05108299	0.0902417	0.23617481	0.19710556	0.19818925	0.15449596	0.14124491	0.13606842	0.15092855	0.15691013	0.157269	0.20087836
ENSG00000224957	0.00710846	0.01428407	0.01390388	0	0.00396525	0.00533223	0.00570862	0	0.00351436	0	0.02686617	0	0.00601667	0	0	0.00685736	0	0	0.03911489
ENSG00000224958	0	0	0	0	0	0	0	0	0	0	0	0	0	0	0	0	0	0	0
ENSG00000224959	0.54874699	0.26954358	1.25221763	0.0945759	0.79586716	0.57628027	1.55708662	1.57667267	3.90665923	1.9549162	2.02788285	4.17074772	4.48982748	3.48761096	3.30888735	2.64681742	1.92620094	2.07402292	1.73119555
ENSG00000224960	0	0	0	0	0	0.00969769	0	0	0	0	0	0	0.02188494	0.02801102	0.01124352	0.02494287	0	0.01559442	0
ENSG00000224961	0.22075164	0.0985753	0.14392743	0	0.24627993	0.2759855	0	0.072076	0.10913771	0.06664659	0	0	0.06228213	0	0	0	0	0	0
ENSG00000224962	0	0	0	0	0.01705734	0	0	0	0	0	0	0	0	0	0	0	0	0	0
ENSG00000224963	0	0	0.04502872	0	0	0	0.22185337	0.31569287	0.52354918	0.5421224	0.29002675	0.23329706	0	0	0.04004299	0	0	0	0.15201162
ENSG00000224964	0	0	0	0	0	0	0	0	0	0	0	0	0	0	0	0	0	0	0
ENSG00000224965	0	0	0	0	0	0	0	0.08440986	0.04260456	0.11707703	0	0.21832613	0.03647002	0.0466788	0.07494677	0.08313177	0	0	0.04741896
ENSG00000224966	0	0	0	0	0	0	0	0	0	0	0	0	0	0	0	0	0	0	0
ENSG00000224967	0.27468528	0.42930663	0.6268203	0.4734171	0.2043004	0.48077928	0.14706189	0.08968547	0.54320814	0.49757738	0.11535155	0.15464767	0.38749393	0.59515468	0.39815474	0.52996503	0.27548426	0.44178293	0
ENSG00000224968	0.06564102	0	0	0.0848487	0	0.04923896	0	0	0	0	0	0	0	0	0	0	0	0	0
ENSG00000224969	0	0.04583437	0	0	0.03817077	0.10265957	0.16485919	0.26810435	0.13532149	0.06197708	0.25862258	0.173363	0.28959206	0.07413109	0.11902375	0.19803364	0.20588208	0.08254118	0.15061307

ENSG00000225158	0	0	0	0	0	0	0	0	0	0	[illegible]
ENSG00000225159	0.18586932	0.02489964	0.07271074	0.0480516	0.04147275	0.05577008	0.11941358	0.18206048	0.1470275	0.10100763	[illegible]
ENSG00000225163	0.12909108	0.28822417	0.12624876	0	0.31204226	0.35505958	0.24189619	0.2107429	0.29783375	0.11692072	[illegible]
ENSG00000225165	0	0	0	0	0	0	0	0	0	0	[illegible]
ENSG00000225166	0	0	0	0	0	0	0.34510524	0.53572123	0.28971101	0.1769164	[illegible]
ENSG00000225167	0	0	0	0	0	0	0	0	0	0	[illegible]
ENSG00000225169	0	0	0	0.1102135	0	0	0	0	0	0	[illegible]
ENSG00000225170	0	0	0.04493244	0.029694	0	0	0.01844825	0	0.02271433	0	[illegible]
ENSG00000225171	0.34880671	0.56072703	0.47757737	0.9017468	0.5058867	0.94193491	0.05602358	0.27332716	0.03448941	0.31592215	[illegible]
ENSG00000225172	0.01839597	0.01232191	0	0	0.01026166	0.01379927	0.01477334	0	0	0.01666165	[illegible]
ENSG00000225173	0.04545803	0.12179402	0.26674279	0.1762794	0.10142982	0.23869437	0.0365062	0.13357949	0.08989622	0.20586182	[illegible]
ENSG00000225174	0	0.00532905	0	0	0	0	0	0.00779296	0	0	[illegible]
ENSG00000225175	0	0	0	0	0	0	0	0	0	0	[illegible]
ENSG00000225176	0	0	0	0	0	0	0	0	0	0	[illegible]
ENSG00000225177	4.08739506	3.68107832	3.01204571	3.2558986	4.92411133	2.79981648	1.26885939	1.24482801	1.27359635	2.63394898	[illegible]
ENSG00000225178	0.07275336	0.12182839	0.10672722	0.0940422	0.02029169	0.05457411	0.0292132	0.03563125	0.07193727	0.03294719	[illegible]
ENSG00000225179	0	0	0	0	0	0	0	0	0	0	[illegible]
ENSG00000225180	0	0	0.01028388	0.0271848	0	0	0	0	0	0	[illegible]
ENSG00000225181	0	0	0	0	0	0	0	0	0	0	[illegible]
ENSG00000225182	0	0	0	0	0	0	0	0	0	0	[illegible]
ENSG00000225183	0	0	0	0	0	0	0.2175033	0.39793219	0.06695002	0.12265213	[illegible]
ENSG00000225185	0.09916843	0.06642459	0.09698494	0.1281868	0.11063652	0.07438871	0.07963967	0.06475751	0.11439871	0.0898191	[illegible]
ENSG00000225187	0	0	0.03665129	0.0484427	0.04181031	0.05622402	0.18057832	0.03670847	0.03705606	0.13577305	[illegible]
ENSG00000225189	0	0	0	0	0	0	0	0	0	0	[illegible]
ENSG00000225190	0.31733208	0.36259222	0.32129843	0.3052287	0.28270272	0.2996487	0.58238757	0.52566721	0.41528712	0.57821108	[illegible]
ENSG00000225191	0	0	0	0	0	0	0.0208733	0	0	0	[illegible]
ENSG00000225192	0.07029387	0.20010678	0.03437307	0.1362946	0.02940856	0.17136984	0	0	0	0	[illegible]
ENSG00000225193	0.08138823	0.05451513	0.15919246	0.3156114	0.13620027	0.36630802	1.24185596	0.71748379	0.36213876	0.66343651	[illegible]
ENSG00000225194	0.02245975	0.08274143	0.06589569	0.0870955	0.02505705	0.04211904	0.08116587	0.10999752	0.03886367	0.08136922	[illegible]
ENSG00000225195	0	0	0.1121712	0	0.0639802	0	0	0.22469243	0.056705	0	[illegible]
ENSG00000225196	0	0	0	0	0	0	0	0	0	0	[illegible]
ENSG00000225198	0	0	0.02424624	0	0	0	0	0	0	0	[illegible]
ENSG00000225200	2.10752684	2.37571188	2.31247991	2.524891	1.31899204	2.00505443	2.51811236	2.92028491	2.94793658	2.0485057	[illegible]
ENSG00000225203	0	0	0	0	0	0	0	0	0	0	[illegible]
ENSG00000225205	0.08287411	0.01665312	0.06483951	0.0107124	0.13868682	0.0870323	0.03993246	0.06494068	0.05736114	0.03753048	[illegible]
ENSG00000225206	0.0249926	0.00478298	0.01047528	0.0092302	0.02389955	0.0107129	0.47023312	0.45113969	0.36891858	0.56590906	[illegible]
ENSG00000225208	0	0	0	0	0	0	0	0	0	0	[illegible]
ENSG00000225209	0	0	0	0	0	0	0	0	0	0	[illegible]
ENSG00000225210	0.63944802	0.98720789	0.75349299	0.7277771	0.45500709	0.68674112	4.12197434	4.20414601	3.13901492	2.67827375	[illegible]
ENSG00000225212	0	0	0	0	0	0	0	0.02619858	0	0	[illegible]
ENSG00000225213	0	0	0.1356676	0.1195429	0.02579402	0.10405881	0.22280826	0.58881023	0.36577574	0.25128728	[illegible]
ENSG00000225214	0.06356951	0.04257986	0	0	0.03546042	0	0	0	0	0	[illegible]
ENSG00000225215	0.05435032	0.10921412	0.05315364	0.0351271	0.01515888	0.02038476	0.10911843	0.15970971	0.12091648	0.12306579	[illegible]
ENSG00000225216	0.01962834	0	0.07678467	0	0.01094911	0.07361854	0	0	0	0.03555567	[illegible]
ENSG00000225217	0	0	0	0	0	0	0	0.01638261	0.00826887	0.01514852	[illegible]
ENSG00000225218	0.11308681	0.15149467	0	0	0.18924668	0.25448768	0.72653734	0.77538599	0.22363657	1.02425285	[illegible]
ENSG00000225221	0	0	0	0	0	0	0	0	0	0	[illegible]
ENSG00000225222	0	0	0	0	0	0	0	0	0	0	[illegible]
ENSG00000225224	0.90104649	0.97494151	1.15234798	1.0751149	0.34796971	1.24781055	0.44529708	0.3394547	0.41120272	0.18833036	[illegible]
ENSG00000225225	0	0	0	0	0	0	0	0	0	0	[illegible]
ENSG00000225226	0.11892893	0.18587446	0.23262071	0.1024864	0.06634109	0.20816028	0.12734511	0.15532244	0.23518975	0.17952771	[illegible]
ENSG00000225230	0	0	0	0	0	0	0	0.0345397	0	0	[illegible]
ENSG00000225231	0	0	0.03219623	0	0	0	0.02643809	0.03224646	0.0162759	0.11926948	[illegible]
ENSG00000225233	0	0.04906361	0	0.0946834	0	0	0	0	0	0	[illegible]
ENSG00000225234	0	0	0	0	0	0.02662591	0	0	0	0	[illegible]
ENSG00000225235	0	0.05424118	0.23758874	0	0.09034389	0.06074455	0.06503239	0	0.08007088	0.07334474	[illegible]
ENSG00000225236	0	0	0	0	0	0	0	0	0	0	[illegible]
ENSG00000225238	0.08264036	0.16606147	0	0	0.0921971	0	0	0	0	0	[illegible]
ENSG00000225239	0	0	0	0	0	0	0	0	0.03463936	0	[illegible]
ENSG00000225240	0.53716233	0.35979984	0	0	0	0.40293882	0	0	0	0	[illegible]
ENSG00000225241	0.73499512	0.83902235	0.74405681	0.9044076	0.5979423	0.91516013	1.12923298	1.19500989	1.1693832	1.24403783	[illegible]
ENSG00000225242	0	0	0	0	0	0	0	0	0	0	[illegible]
ENSG00000225243	0	0.01432514	0	0.0552896	0.01192995	0.01604269	0.05152533	0.04189686	0.02114679	0.01937041	[illegible]
ENSG00000225244	0	0	0	0	0	0	0	0	0	0	[illegible]
ENSG00000225246	0.03925669	0.0525895	0	0.0507439	0.02189822	0.02944742	0	0	0	0	[illegible]
ENSG00000225249	0	0	0	0	0	0	0	0	0	0	[illegible]
ENSG00000225251	0	0.02785547	0	0.0537557	0.02319798	0	0	0.04073456	0.06168041	0	[illegible]
ENSG00000225253	0	0	0	0	0	0	0	0	0	0	[illegible]
ENSG00000225254	0	0	0	0	0	0	0	0	0	0	[illegible]
ENSG00000225255	0	0	0	0	0	0	0.0452894	0	0	0	[illegible]
ENSG00000225256	0	0	0	0	0	0	0	0	0	0	[illegible]
ENSG00000225258	0	0.15365118	0.33651359	0.2965175	0.09597029	0.12905514	0.04605497	0.11234622	0	0.1038833	[illegible]
ENSG00000225259	0	0.05882286	0.04294292	0.0567584	0.03674067	0.03293778	0.0176314	0	0.04341718	0.01988502	[illegible]
ENSG00000225261	0	0	0	0	0	0	0	0	0	0	[illegible]
ENSG00000225263	0	0	0	0	0	0	0	0	0	0	[illegible]
ENSG00000225264	1.67602353	1.62587377	1.5825995	1.5688131	1.26805459	1.22832516	0.88184392	0.7359248	1.11433971	1.34352832	[illegible]
ENSG00000225265	0.93289889	0.85598694	0.57491075	0.7433512	0.67009234	0.78606622	0.5131422	0.56329021	0.32222021	0.74077605	[illegible]
ENSG00000225267	0	0	0	0	0	0	0	0	0	0.05814981	[illegible]
ENSG00000225269	0	0	0	0	0	0	0	0	0	0	[illegible]
ENSG00000225270	0	0	0	0	0	0	0	0	0	0	[illegible]
ENSG00000225271	0	0	0	0	0	0	0	0	0	0	[illegible]
ENSG00000225272	0.06828335	0	0	0	0	0	0.05483664	0	0	0	[illegible]
ENSG00000225273	0.01351352	0	0.01321598	0.0174678	0.00753813	0.02027365	0.01085237	0	0.00668097	0.024479	[illegible]
ENSG00000225275	0	0	0	0	0	0	0	0	0	0	[illegible]
ENSG00000225276	0	0	0	0	0	0	0	0	0	0	[illegible]
ENSG00000225278	0.01662184	0.11133569	0.08940721	0.0751998	0.01854403	0.03117113	0.02669716	0.04884366	0.01643538	0.00752739	[illegible]
ENSG00000225279	0.39974871	0.43510679	0.87963088	0.6459024	0.44597668	0.82461899	0.04012852	0	0	0	[illegible]
ENSG00000225280	0	0	0	0	0	0	0	0	0	0	[illegible]
ENSG00000225282	0.05484357	0.01224503	0	0	0	0	0.01468116	0.03581314	0.00903806	0.03311538	[illegible]
ENSG00000225284	0	0	0	0	0	0	0	0	0	0	[illegible]
ENSG00000225285	0.04805627	0	0.09399634	0.0414122	0.03574242	0.02403214	0.01286426	0.0156905	0.00791954	0.0290171	[illegible]
ENSG00000225286	0	0	0	0	0	0	0	0	0	0	[illegible]
ENSG00000225287	0	0	0	0	0	0	0	0	0	0	[illegible]
ENSG00000225289	0	0	0	0	0	0	0	0	0	0	[illegible]
ENSG00000225292	0.20463327	0.41119982	0.80051064	1.1903058	0.22829759	0.99775328	0.49300748	0.10021996	0.10116892	0.27801149	[illegible]
ENSG00000225293	0	0	0	0	0	0	0	0	0	0	[illegible]
ENSG00000225294	0	0	0	0	0	0	0	0	0	0.06574596	[illegible]
ENSG00000225295	0	0	0	0	0	0	0	0	0	0	[illegible]
ENSG00000225297	0	0	0	0	0	0	0	0	0	0	[illegible]
ENSG00000225298	0	0	0	0	0	0	0	0	0	0	[illegible]
ENSG00000225299	0.25528507	0.08549699	0.12483211	0.2474893	0.07120172	0.19149568	0	0	0	0	[illegible]
ENSG00000225300	0.04933123	0.24231418	0.17689856	0.2338101	0.13759006	0.17268807	0.33013894	0.25770846	0.26014866	0.65531279	[illegible]
ENSG00000225302	0	0.02642349	0	0	0	0.02959159	0.15840204	0	0.05850957	0	[illegible]
ENSG00000225303	0	0	0	0	0	0	0	0	0	0	[illegible]
ENSG00000225304	0	0	0	0	0	0	0	0	0	0	[illegible]
ENSG00000225308	0	0	0	0	0	0	0	0	0	0	[illegible]
ENSG00000225310	0	0	0	0	0	0	0	0	0	0	[illegible]
ENSG00000225311	0	0	0	0	0	0	0	0	0	0	[illegible]
ENSG00000225313	0	0	0	0	0	0	0.0116171	0	0	0.01310198	[illegible]
ENSG00000225314	0	0	0	0	0	0	0	0	0	0	[illegible]
ENSG00000225315	0	0	0	0	0	0	0	0	0.03509715	0	[illegible]
ENSG00000225316	0	0	0	0	0	0	0.06133387	0	0	0	[illegible]
ENSG00000225321	0	0	0	0	0	0	0	0	0	0	[illegible]
ENSG00000225322	0	0	0	0	0	0	0	0	0	0	[illegible]
ENSG00000225323	0	0	0	0	0	0	0	0	0	0	[illegible]
ENSG00000225325	0	0	0	0.051433	0	0	0	0	0.01967174	0	[illegible]
ENSG00000225326	0	0	0	0	0	0	0	0	0	0	[illegible]
ENSG00000225327	0	0	0	0	0	0	0	0	0	0	[illegible]
ENSG00000225328	0	0	0	0	0	0	0.04236153	0.05166823	0	0.04777611	[illegible]
ENSG00000225329	0.05035897	0	0	0	0	0	0.12132606	0.19730804	0.02489704	0.04561126	[illegible]
ENSG00000225330	0.05924585	0.19841903	0.17382412	0.1531643	0.06609719	0.13332535	0.04757885	0.05803178	0.05858127	0	[illegible]
ENSG00000225331	0	0	0	0	0	0	0	0	0	0	[illegible]
ENSG00000225333	0	0	0	0	0.02579402	0	0	0	0	0	[illegible]
ENSG00000225334	0.38096619	0.05103544	0.07451562	0	0.4250221	0	0.06118887	0.59705507	0.3766928	0.62108949	[illegible]
ENSG00000225335	0.0128713	0.05172841	0.0251758	0.049913	0.02871954	0.13517117	0.52716754	0.32779611	0.46453263	0.18652528	[illegible]
ENSG00000225336	0.05624736	0	0.05500891	0.0727063	0	0	0	0	0	0	[illegible]
ENSG00000225337	0	0	0	0	0	0	0	0	0	0	[illegible]
ENSG00000225338	0.06886697	0.04612818	0.06735066	0	0.23049276	0.15497647	0.05530533	0.26982296	0.06809447	0	[illegible]
ENSG00000225339	0.06030218	0.0689029	0.06244353	0.059607	0.0534248	0.07184249	0.23643849	0.13550541	0.07891644	0.05140428	[illegible]
ENSG00000225341	0	0	0	0	0	0	0	0	0	0	[illegible]
ENSG00000225342	0.05509357	0.40592803	0.43104419	0.2848595	0.12292947	0.28928941	0.04424426	0	0.02723779	0.14969849	[illegible]
ENSG00000225343	0	0	0	0	0	0	0	0	0	0	[illegible]
ENSG00000225344	0	0	0	0	0	0	0	0	0.01780347	0	[illegible]
ENSG00000225345	0.73561108	1.59508944	1.45102232	0.4673734	0.26428647	0.60791544	0.12015269	0.32973723	0.3082032	0.39523877	[illegible]
ENSG00000225347	0	0	0	0	0	0	0	0	0	0	[illegible]
ENSG00000225349	0.06046855	0.12150839	0.17741148	0.234488	0.10119194	0.36287174	0.04856077	0.11845886	0.0896854	0.10953548	[illegible]
ENSG00000225350	0	0	0	0	0	0	0	0	0	0	[illegible]
ENSG00000225352	0	0	0	0	0	0	0	0	0	0	[illegible]
ENSG00000225353	0	0	0	0	0	0.01661603	0	0	0	0	[illegible]
ENSG00000225354	0	0	0	0	0	0	0	0	0	0	[illegible]
ENSG00000225355	0	0	0.11197196	0	0.09579983	0	0	0	0.05660428	0	[illegible]
ENSG00000225356	0.09507298	0.19104416	0.1394695	0.1228929	0.10606746	0.07131661	0	0	0	0.04305488	[illegible]
ENSG00000225357	0	0	0	0	0	0	0	0	0.01758731	0	[illegible]
ENSG00000225358	0.07950108	0.04260087	0.09330076	0.1233173	0.07095584	0.10734433	0	0	0	0.01440119	[illegible]
ENSG00000225359	0	0	0	0	0	0	0	0	0	0	[illegible]
ENSG00000225360	0	0	0	0	0	0	0	0	0	0	[illegible]
ENSG00000225361	0.01464988	0.06868906	0.0358183	0.0852151	0.04494609	0.04395696	0.71177955	0.55963736	0.39110986	0.57718976	[illegible]
ENSG00000225362	0.00509803	0.00682948	0.00997156	0	0	0	0	0	0.00252042	0	[illegible]
ENSG00000225364	0	0	0	0	0	0	0	0	0	0	[illegible]
ENSG00000225365	0	0	0	0	0	0	0	0	0	0	[illegible]
ENSG00000225366	0	0.01976319	0.01923717	0.0127131	0.00548625	0.02951032	0.03949175	0.02890078	0.01458722	0.04453953	[illegible]
ENSG00000225369	0	0	0	0	0	0	0	0	0	0	[illegible]
ENSG00000225370	0	0	0	0	0	0	0	0	0	0	[illegible]
ENSG00000225371	0	0	0	0	0	0	0	0	0	0	[illegible]
ENSG00000225373	0.44036203	0.81911596	0.44230578	0.4384527	0.33692968	0.75439433	0.79091926	0.6674083	0.53692287	1.02675864	[illegible]
ENSG00000225376	0	0.03525529	0.05147541	0.0453573	0	0.01316077	0	0.05155572	0	0	[illegible]
ENSG00000225377	2.74975954	2.81272099	3.06445481	3.2512982	2.49700486	3.1659479	2.77316707	1.98352001	2.12876277	2.62566405	[illegible]
ENSG00000225378	0	0	0	0	0	0	0	0	0	0	[illegible]
ENSG00000225380	0	0	0	0	0	0	0	0	0	0	[illegible]
ENSG00000225381	0	0	0	0	0	0	0	0	0	0	[illegible]
ENSG00000225383	0.56828257	0.35345576	0.2381872	0.524694	1.16988137	1.03525844	0.11952637	0.10602615	0.07358319	0.02450983	[illegible]
ENSG00000225384	0	0	0	0	0	0	0	0	0	0	[illegible]
ENSG00000225385	0	0	0	0	0	0	0	0	0	0	[illegible]
ENSG00000225386	0	0	0	0	0.03960008	0	0	0	0	0	[illegible]
ENSG00000225387	0	0	0	0	0	0	0	0	0	0	[illegible]
ENSG00000225391	0.01191047	0	0	0	0	0.01786868	0	0	0	0.01078759	[illegible]
ENSG00000225392	0	0	0	0	0	0	0	0	0	0	[illegible]
ENSG00000225393	0.17484488	0.15615183	0.11399677	0.2260074	0.03251073	0.21859249	0.04680451	0.17126195	0.02881393	0	[illegible]
ENSG00000225394	0	0	0	0	0	0	0	0	0	0	[illegible]
ENSG00000225396	0	0	0	0	0	0	0	0	0	0	[illegible]
ENSG00000225397	0	0	0	0	0	0	0	0	0	0	[illegible]
ENSG00000225398	0	0	0	0	0	0	0	0	0	0	[illegible]
ENSG00000225399	0	0.05278237	0	0	0.0293047	0.03940722	0.08437781	0.02572884	0.05194492	0.02379071	[illegible]
ENSG00000225400	0	0	0	0	0	0	0	0	0	0	[illegible]
ENSG00000225401	0.09410143	0.25212252	0.04601475	0.1216371	0.05249178	0.21176347	0.26449671	0.23043275	0.18609174	0.0852298	[illegible]
ENSG00000225402	0.03078294	0	0.03010516	0.0795811	0.06868552	0.06927315	0.02472101	0.03015214	0.06087528	0.02788081	[illegible]
ENSG00000225404	0	0	0	0	0	0	0	0	0	0	[illegible]
ENSG00000225405	0	0.05535382	0	0	0.04609855	0	0	0.08094689	0	0	[illegible]
ENSG00000225406	0	0	0	0	0	0	0.03784049	0	0	0	[illegible]
ENSG00000225407	0.05371623	0.03597998	0	0	0	0.04029388	0.51765785	0.4735393	0.45146632	0.04865201	[illegible]
ENSG00000225408	0.11870991	0.07951378	0.05804808	0.0767232	0.06621891	0.04452363	0.09533294	0	0	0.21503651	[illegible]

ENSG00000225410	0	0	0	0	0	0	0	0	0.08707161	0	0	0	0.1490687	0
ENSG00000225411	0.04479463	0.03000416	0.02190417	0.0144756	0.01874055	0.03360157	0.28778755	0.18647598	0.16609561	0.31442925	0.26805808	0.24588873	0.04739328	0.01213195
ENSG00000225413	0	0	0	0	0	0	0	0	0	0	0	0	0	0
ENSG00000225414	0	0	0	0	0	0	0	0	0	0	0	0	0	0
ENSG00000225415	0	0	0	0	0	0	0	0	0	0	0	0	0.01452586	0.01859198
ENSG00000225416	0.20206733	0.40604371	0.88928201	0.7835869	0.28179365	0.07578787	0.64910076	0.89066953	1.09890383	1.18961029	0.63642234	0.76790569	0.42757951	0.32836121
ENSG00000225417	0.11428986	0	0	0	0.38251989	0	0	0	0	0	0	0	0	0
ENSG00000225418	0	0	0	0	0	0	0	0	0	0	0	0	0.04479404	0
ENSG00000225419	0	0	0	0	0.03761179	0.10115619	0.10829662	0.06604453	0	0	0.16989015	0.11388281	0	0.07304548
ENSG00000225420	0.01844862	0.04942871	0.0902121	0.119235	0.01029103	0.15222646	0	0	0	0	0	0	0	0
ENSG00000225421	0	0	0	0	0	0	0	0	0	0	0	0	0	0
ENSG00000225422	0.02462165	0.03298394	0	0.0318264	0.05493792	0.03693862	0.03954605	0.0482342	0	0	0	0.02079296	0	0.0800208
ENSG00000225423	0.22657111	0.22764138	0.24928028	0.1464348	0.15798273	0.29742409	0.15920936	0.27741025	0.0840111	0.20521059	0.07135983	0.21525652	0	0.24545337
ENSG00000225424	0	0	0	0	0	0	0	0	0	0	0	0	0	0
ENSG00000225427	0	0	0	0	0	0	0	0	0	0	0	0	0	0
ENSG00000225428	0	0	0	0	0	0	0	0	0	0	0	0	0	0
ENSG00000225431	0.0181576	0.25540721	0.05327342	0.0469416	0.03038609	0.0408614	0.01458191	0.03557103	0	0.04933725	0.02287535	0.04600224	0.01536877	0.01967084
ENSG00000225433	0	0	0	0	0	0	0	0	0	0	0	0	0	0
ENSG00000225434	0	0.00646928	0	0	0.0053876	0	0	0	0.01909992	0	0	0	0.00817488	0
ENSG00000225437	0	0	0	0	0	0	0	0	0	0	0	0	0	0
ENSG00000225438	0	0	0	0	0	0	0	0	0	0	0	0	0	0
ENSG00000225439	1.35821908	1.48236822	1.74243524	1.5387815	1.52865722	1.58818227	1.24474	1.35386381	1.27978438	2.05510723	1.4494148	1.39665941	0.13524825	0.08655365
ENSG00000225442	0	0.02040453	0	0.0787537	0.01699285	0.02285097	0.04892796	0.02983865	0.01506059	0	0.07675566	0.02572589	0.02578409	0.03300165
ENSG00000225443	0	0	0	0	0.05837154	0	0	0	0.05173411	0	0	0	0	0
ENSG00000225444	0	0	0	0	0	0	0	0	0	0	0	0	0	0
ENSG00000225446	0	0	0	0	0	0	0	0	0	0	0	0	0	0
ENSG00000225447	0.08437105	0.50861758	0.57759358	0	0.32944776	0.37973292	0.88083143	0.74377901	0.08342464	0.91700124	0.85034021	0.71251284	0.42847496	0.63981725
ENSG00000225448	0	0	0	0	0	0	0	0	0	0	0	0	0	0
ENSG00000225449	0.43773415	0.49425357	0.50148404	0.7598188	0.42556637	0.73176317	0.02008762	0.0245008	0.0185496	0.02265519	0.09453724	0	0.01058579	0.04064696

[illegible]

ENSG00000225667	0	0 0 0 0 0 0 0 0 0 0 0 0 0 0.07918106 0 0 [illegible]
ENSG00000225669	0	0 0 0 0 0 0 0 0 0 0 0 0 0 0 0 0 [illegible]
ENSG00000225670	0	0 0 0 0 0 0 0 0 0 0 0 0 0 0 0 0 [illegible]
ENSG00000225671	0	0.14512935 0 0 0 0 0 0 0 0 0 0 0 0 0 0 0 [illegible]
ENSG00000225672	0	0 0 0 0 0 0 0 0 0 0 0 0 0 0 0 0 [illegible]
ENSG00000225673	0.05462668	0.03658981 0.05342391 0 0 0.04097683 0.04386931 0.05350727 0.13503479 0 0 0.04613219 0 0 0 0 0 [illegible]
ENSG00000225674	0.03683399	0.01233599 0.01801149 0.0238061 0.03082017 0.05526018 0.02958045 0.07215837 0.02731561 0.03336138 0.02320214 0.04665941 0.04676498 0.09975926 0.0160172 0.03553289 0 [illegible]
ENSG00000225675	0.28421287	0.30459246 0.27795508 0.1469513 0.31707998 0.21319514 0.54778609 0.66813305 0.33722975 1.85340992 0.71611544 0.67204915 0.19244848 0.06157979 0 0 0.08551187 [illegible]
ENSG00000225676	0	0 0 0 0 0 0 0 0.05347015 0 0 0.09133554 0.09154219 0.35150075 0.37624287 0 0 [illegible]
ENSG00000225678	0	0 0 0 0 0 0 0 0 0 0.03509399 0 0 0 0 0 0 [illegible]
ENSG00000225679	0	0 0 0 0 0 0 0 0 0 0 0 0 0 0 0 0 [illegible]
ENSG00000225680	0	0 0 0 0 0 0 0 0 0 0 0 0 0 0 0 0 [illegible]
ENSG00000225681	0	0 0 0 0 0 0 0 0 0 0 0 0 0 0 0 0 [illegible]
ENSG00000225683	0	0 0 0 0 0 0 0 0 0 0 0 0 0 0 0 0 [illegible]
ENSG00000225684	0.01486497	0.02323252 0.03876714 0.0192147 0.017966 0.03531019 0.09550149 0.09949579 0.04776925 0.03365886 0.09675733 0.04602935 0.00629093 0.02147175 0.00861868 0.00477996 0.00372705 [illegible]
ENSG00000225685	0	0 0 0 0 0 0 0 0 0 0 0 0 0 0 0 0 [illegible]
ENSG00000225689	0	0 0 0 0 0 0 0 0 0 0 0 0 0 0 0 0 [illegible]
ENSG00000225690	0	0 0 0 0 0 0 0 0 0 0 0 0 0 0 0 0 [illegible]
ENSG00000225693	0.19977111	0.17841315 0.39074512 0.1721517 0.11143658 0.24975547 0.90910987 0.65225799 0.55966899 0.90468614 0.50335221 0.44988414 0.28181377 0.14427992 0.40539392 0.83509642 0.20035219 [illegible]
ENSG00000225695	0.16734029	0.11208718 0.09819348 0.0432614 0.13068437 0.25105223 0.02687735 0.09834669 0.13237055 0.09093834 0.08432761 0.0565275 0.0283277 0 0 0.03228586 0.10069622 [illegible]
ENSG00000225697	0.48888114	0.50369132 0.4157926 0.5024891 0.50529752 0.67334786 1.11275529 1.12180563 1.25485244 0.98370457 0.8487309 0.96180177 1.48410984 1.25058359 1.37054665 1.15136632 1.00525841 [illegible]
ENSG00000225698	0	0 0 0 0 0 0 0 0 0 0 0 0 0 0 0 0 [illegible]
ENSG00000225701	0	0 0 0 0 0 0 0 0 0 0.35307605 0 0.11860684 0 0 0 0.21080534 [illegible]
ENSG00000225703	0	0 0 0 0 0 0 0 0 0 0 0 0 0 0 0 0 [illegible]
ENSG00000225704	0	0 0 0 0 0 0 0.13607451 0 0.12582416 0 0 0 0 0 0 0 [illegible]
ENSG00000225705	0	0 0 0 0 0 0 0 0 0 0 0 0 0 0.01635361 0 0 [illegible]
ENSG00000225706	0.87083388	1.44038978 1.18189537 0.5628272 0.48576968 0.51992106 0 0 0.00439319 0.03219322 0 0 0 0 0 0.00857218 0 [illegible]
ENSG00000225708	0	0 0 0 0 0 0 0 0 0 0 0 0 0 0 0 0 [illegible]
ENSG00000225710	0.03654166	0.2447618 0.03573708 0.2834061 0.12230228 0.13705402 0.05869137 0.03579284 0.03613176 0.03309661 0.18414397 0.1234376 0.12371688 0.19793504 0.38136182 0.38775975 0.21988766 [illegible]
ENSG00000225711	0	[illegible]
ENSG00000225712	0	[illegible]
ENSG00000225713	0	[illegible]
ENSG00000225715	0	[illegible]
ENSG00000225716	0	[illegible]
ENSG00000225718	0	[illegible]
ENSG00000225719	0	[illegible]
ENSG00000225720	0	[illegible]
ENSG00000225721	0.44352853	[illegible]
ENSG00000225722	0	[illegible]
ENSG00000225723	0	[illegible]
ENSG00000225724	0	[illegible]
ENSG00000225725	0.21974823	[illegible]
ENSG00000225726	0.33090082	[illegible]
ENSG00000225727	0.05499956	[illegible]
ENSG00000225728	0	[illegible]
ENSG00000225730	0	[illegible]
ENSG00000225731	0	[illegible]
ENSG00000225733	37.669535	[illegible]
ENSG00000225735	0	[illegible]
ENSG00000225738	0	[illegible]
ENSG00000225739	0	[illegible]
ENSG00000225740	0	[illegible]
ENSG00000225741	0	[illegible]
ENSG00000225742	0	[illegible]
ENSG00000225744	0	[illegible]
ENSG00000225745	0	[illegible]
ENSG00000225746	1.27498429	[illegible]
ENSG00000225750	0.23103756	[illegible]
ENSG00000225751	0	[illegible]
ENSG00000225752	0	[illegible]
ENSG00000225755	0	[illegible]
ENSG00000225756	0.01506767	[illegible]
ENSG00000225758	0	[illegible]
ENSG00000225759	0	[illegible]
ENSG00000225760	0	[illegible]
ENSG00000225761	0.07291796	[illegible]
ENSG00000225762	0	[illegible]
ENSG00000225764	0.22075164	[illegible]
ENSG00000225765	0	[illegible]
ENSG00000225766	0.0271237	[illegible]
ENSG00000225767	0	[illegible]
ENSG00000225768	0	[illegible]
ENSG00000225769	0.0113585	[illegible]
ENSG00000225770	0	[illegible]
ENSG00000225774	0	[illegible]
ENSG00000225775	0	[illegible]
ENSG00000225777	0	[illegible]
ENSG00000225778	0.04456546	[illegible]
ENSG00000225779	0	[illegible]
ENSG00000225781	0	[illegible]
ENSG00000225782	0	[illegible]
ENSG00000225783	0.25908653	[illegible]
ENSG00000225784	0.10957838	[illegible]
ENSG00000225785	0	[illegible]
ENSG00000225786	0.27198093	[illegible]
ENSG00000225787	0	[illegible]
ENSG00000225790	0	[illegible]
ENSG00000225791	2.0028237	[illegible]
ENSG00000225792	31.2216603	[illegible]
ENSG00000225793	0.19885459	[illegible]
ENSG00000225794	0.04928095	[illegible]
ENSG00000225795	0	[illegible]
ENSG00000225796	0	[illegible]
ENSG00000225797	0	[illegible]
ENSG00000225798	0	[illegible]
ENSG00000225801	0	[illegible]
ENSG00000225802	0	[illegible]
ENSG00000225803	0	[illegible]
ENSG00000225805	0	[illegible]
ENSG00000225806	0.08772384	[illegible]
ENSG00000225807	0.0565434	[illegible]
ENSG00000225808	4.48768528	[illegible]
ENSG00000225809	0	[illegible]
ENSG00000225811	0	[illegible]
ENSG00000225812	0	[illegible]
ENSG00000225813	0	[illegible]
ENSG00000225814	0	[illegible]
ENSG00000225815	0	[illegible]
ENSG00000225816	0	[illegible]
ENSG00000225818	0.03245694	[illegible]
ENSG00000225819	0	[illegible]
ENSG00000225822	0.12116444	[illegible]
ENSG00000225823	0	[illegible]
ENSG00000225825	0	[illegible]
ENSG00000225826	0	[illegible]
ENSG00000225827	0	[illegible]
ENSG00000225828	0.77061262	[illegible]
ENSG00000225830	1.05434671	[illegible]
ENSG00000225831	0	[illegible]
ENSG00000225832	0	[illegible]
ENSG00000225833	0	[illegible]
ENSG00000225836	0	[illegible]
ENSG00000225839	0	[illegible]
ENSG00000225840	46.1654703	[illegible]
ENSG00000225842	0	[illegible]
ENSG00000225843	0	[illegible]
ENSG00000225846	0	[illegible]
ENSG00000225849	0	[illegible]
ENSG00000225850	0	[illegible]
ENSG00000225851	0	[illegible]
ENSG00000225854	0	[illegible]
ENSG00000225855	0.49152109	[illegible]
ENSG00000225856	0	[illegible]
ENSG00000225857	0.03265424	[illegible]
ENSG00000225858	0	[illegible]
ENSG00000225860	0.01218516	[illegible]
ENSG00000225864	0	[illegible]
ENSG00000225867	0	[illegible]
ENSG00000225868	0	[illegible]
ENSG00000225869	0.05240608	[illegible]
ENSG00000225870	0	[illegible]
ENSG00000225871	0	[illegible]
ENSG00000225872	0	[illegible]
ENSG00000225873	0	[illegible]
ENSG00000225876	0.14452798	[illegible]
ENSG00000225877	0	[illegible]
ENSG00000225878	0	[illegible]
ENSG00000225879	0	[illegible]
ENSG00000225880	0.25882391	[illegible]
ENSG00000225881	0	[illegible]
ENSG00000225882	0.09104446	[illegible]
ENSG00000225883	0	[illegible]
ENSG00000225884	0	[illegible]
ENSG00000225885	0.01192811	[illegible]
ENSG00000225886	0	[illegible]
ENSG00000225889	0.97456594	[illegible]
ENSG00000225891	0.30462892	[illegible]
ENSG00000225892	0.25928994	[illegible]
ENSG00000225893	0	[illegible]
ENSG00000225894	0	[illegible]
ENSG00000225895	0	[illegible]
ENSG00000225896	0	[illegible]
ENSG00000225898	0	[illegible]
ENSG00000225899	0	[illegible]
ENSG00000225900	0.10851764	[illegible]
ENSG00000225901	0	[illegible]
ENSG00000225903	0	[illegible]
ENSG00000225904	0	[illegible]
ENSG00000225905	0	[illegible]
ENSG00000225906	0	[illegible]
ENSG00000225907	0.48104089	[illegible]
ENSG00000225911	0	[illegible]
ENSG00000225912	0	[illegible]
ENSG00000225913	0	[illegible]
ENSG00000225914	0.64310354	[illegible]
ENSG00000225916	0.2339727	[illegible]
ENSG00000225918	0	[illegible]
ENSG00000225919	0	[illegible]
ENSG00000225920	0	[illegible]
ENSG00000225921	15.9039116	[illegible]
ENSG00000225922	0	[illegible]

ENSG00000225923	0	0	[illegible]
ENSG00000225924	0	0	[illegible]
ENSG00000225925	0	0	[illegible]
ENSG00000225928	0	0	[illegible]
ENSG00000225929	0	0	[illegible]
ENSG00000225930	0.40814652	0.66260628	[illegible]
ENSG00000225931	0	0	[illegible]
ENSG00000225932	0	0	[illegible]
ENSG00000225933	0	0	[illegible]
ENSG00000225934	0	0	[illegible]
ENSG00000225935	0	0	[illegible]
ENSG00000225936	0	0	[illegible]
ENSG00000225937	0	0.00550433	[illegible]
ENSG00000225938	0.3998727	1.66061466	[illegible]
ENSG00000225940	0.50556455	0.25397636	[illegible]
ENSG00000225942	0	0	[illegible]
ENSG00000225943	0	0	[illegible]
ENSG00000225944	0	0	[illegible]
ENSG00000225945	0.15384124	0.56674916	[illegible]
ENSG00000225946	0	0	[illegible]
ENSG00000225947	0	0	[illegible]
ENSG00000225948	0	0	[illegible]
ENSG00000225949	0	0	[illegible]
ENSG00000225950	0.00988946	0.0231844	[illegible]
ENSG00000225951	0	0.10608349	[illegible]
ENSG00000225952	0	0	[illegible]
ENSG00000225953	0	0	[illegible]
ENSG00000225956	0.07226399	0	[illegible]
ENSG00000225957	0	0	[illegible]
ENSG00000225959	0	0	[illegible]
ENSG00000225960	0	0	[illegible]
ENSG00000225962	0	0	[illegible]
ENSG00000225963	0	0.00532774	[illegible]
ENSG00000225964	0.02469712	0.03308504	[illegible]
ENSG00000225965	0.89527055	0.47973312	[illegible]
ENSG00000225968	5.25568898	4.48024145	[illegible]
ENSG00000225969	0.05775939	0	[illegible]
ENSG00000225970	0	0	[illegible]
ENSG00000225971	0.65358154	0.43777928	[illegible]
ENSG00000225972	0.34655634	0.29016116	[illegible]
ENSG00000225973	2.73589057	2.53166327	[illegible]
ENSG00000225974	0	0	[illegible]
ENSG00000225975	0.3359014	0.64122744	[illegible]
ENSG00000225976	0	0	[illegible]
ENSG00000225978	0	0	[illegible]
ENSG00000225979	0	0.04776104	[illegible]
ENSG00000225980	0	0	[illegible]
ENSG00000225981	0	0.04334938	[illegible]
ENSG00000225982	0	0	[illegible]
ENSG00000225984	0.05132124	0	[illegible]
ENSG00000225986	0	0	[illegible]
ENSG00000225988	0.01671667	0.01119709	[illegible]
ENSG00000225990	0	0	[illegible]
ENSG00000225991	0	0.21061454	[illegible]
ENSG00000225992	0	0	[illegible]
ENSG00000225995	0	0	[illegible]
ENSG00000225996	0	0	[illegible]
ENSG00000225997	0.03410554	0.04568887	[illegible]
ENSG00000225999	0	0	[illegible]
ENSG00000226002	0.10999911	0.36839574	[illegible]
ENSG00000226003	0	0	[illegible]
ENSG00000226004	0	0	[illegible]
ENSG00000226005	0	0	[illegible]
ENSG00000226007	0.02386799	0.02131621	[illegible]
ENSG00000226008	0	0	[illegible]
ENSG00000226009	0.07583468	0.12698818	[illegible]
ENSG00000226010	0.09099305	0.08532803	[illegible]
ENSG00000226011	0	0	[illegible]
ENSG00000226012	0	0	[illegible]
ENSG00000226013	0	0	[illegible]
ENSG00000226014	0	0	[illegible]
ENSG00000226015	0.03890132	0.20845374	[illegible]
ENSG00000226016	0	0	[illegible]
ENSG00000226017	0	0.05189421	[illegible]
ENSG00000226020	0	0	[illegible]
ENSG00000226022	0	0	[illegible]
ENSG00000226023	0	0	[illegible]
ENSG00000226024	0	0	[illegible]
ENSG00000226025	0.00614602	0	[illegible]
ENSG00000226026	0.336518	0.79982451	[illegible]
ENSG00000226028	0	0.04042695	[illegible]
ENSG00000226029	0.08938911	0.0997904	[illegible]
ENSG00000226030	0	0	[illegible]
ENSG00000226031	0.13494657	0.18077871	[illegible]
ENSG00000226032	0	0	[illegible]
ENSG00000226034	0	0	[illegible]
ENSG00000226036	0	0	[illegible]
ENSG00000226037	0.048393	0	[illegible]
ENSG00000226038	0.48393003	0.3025344	[illegible]
ENSG00000226040	0.06246074	0.04183719	[illegible]
ENSG00000226041	0	0	[illegible]
ENSG00000226042	0	0	[illegible]
ENSG00000226043	0.22227407	0.49627565	[illegible]
ENSG00000226044	0	0	[illegible]
ENSG00000226045	0.0871074	0.05834592	[illegible]
ENSG00000226046	0	0	[illegible]
ENSG00000226047	0	0	[illegible]
ENSG00000226048	0	0	[illegible]
ENSG00000226049	0.03029111	0	[illegible]
ENSG00000226051	0.01672534	0.0074686	[illegible]
ENSG00000226053	0.2458078	0.28086663	[illegible]
ENSG00000226054	0.02654839	0	[illegible]
ENSG00000226055	0.02648294	0.08869347	[illegible]
ENSG00000226056	0.02605476	0.10471135	[illegible]
ENSG00000226057	0	0.00677802	[illegible]
ENSG00000226058	0	0	[illegible]
ENSG00000226059	0	0	[illegible]
ENSG00000226061	0	0	[illegible]
ENSG00000226063	0	0	[illegible]
ENSG00000226064	0	0	[illegible]
ENSG00000226065	0	0	[illegible]
ENSG00000226066	0	0	[illegible]
ENSG00000226067	0	0	[illegible]
ENSG00000226068	0.04013666	0	[illegible]
ENSG00000226070	0	0.02619902	[illegible]
ENSG00000226072	0.02555887	0	[illegible]
ENSG00000226074	0	0	[illegible]
ENSG00000226075	0	0	[illegible]
ENSG00000226078	0	0.12736278	[illegible]
ENSG00000226079	0	0	[illegible]
ENSG00000226080	0.03765157	0.02521962	[illegible]
ENSG00000226081	0	0.02042383	[illegible]
ENSG00000226082	0	0.04216404	[illegible]
ENSG00000226083	0.03253338	0.02905517	[illegible]
ENSG00000226084	0.02749978	0.01841979	[illegible]
ENSG00000226085	0.07837019	0.0262468	[illegible]
ENSG00000226086	0.03920893	0.07878829	[illegible]
ENSG00000226087	0	0	[illegible]
ENSG00000226088	0	0	[illegible]
ENSG00000226089	0.02922007	0.03914414	[illegible]
ENSG00000226091	0.1979869	0.32166134	[illegible]
ENSG00000226092	0	0	[illegible]
ENSG00000226093	0	0	[illegible]
ENSG00000226094	0	0	[illegible]
ENSG00000226096	0	0	[illegible]
ENSG00000226097	0	0	[illegible]
ENSG00000226098	0	0	[illegible]
ENSG00000226101	0.03124551	0	[illegible]
ENSG00000226102	7.96140917	6.99329272	[illegible]
ENSG00000226104	0.39789802	0	[illegible]
ENSG00000226107	0.11152159	0	[illegible]
ENSG00000226108	0	0	[illegible]
ENSG00000226110	0	0	[illegible]
ENSG00000226112	0.1761188	0.23593432	[illegible]
ENSG00000226113	0	0.10159054	[illegible]
ENSG00000226114	0	0	[illegible]
ENSG00000226115	0	0	[illegible]
ENSG00000226116	0	0	[illegible]
ENSG00000226117	0	0	[illegible]
ENSG00000226118	0	0	[illegible]
ENSG00000226119	0	0	[illegible]
ENSG00000226121	0.12697696	0.10006021	[illegible]
ENSG00000226122	0	0	[illegible]
ENSG00000226124	0	0	[illegible]
ENSG00000226125	0.03705988	0.15721419	[illegible]
ENSG00000226126	0	0.01642922	[illegible]
ENSG00000226128	0	0	[illegible]
ENSG00000226129	0	0	[illegible]
ENSG00000226130	0	0	[illegible]
ENSG00000226131	0.32359176	0.04334938	[illegible]
ENSG00000226132	0	0	[illegible]
ENSG00000226133	0	0	[illegible]
ENSG00000226134	0	0	[illegible]
ENSG00000226137	4.22716468	4.7766899	[illegible]
ENSG00000226138	0.0440297	0.02949179	[illegible]
ENSG00000226140	0	0	[illegible]
ENSG00000226141	0	0	[illegible]
ENSG00000226142	0	0	[illegible]
ENSG00000226143	0	0	[illegible]
ENSG00000226144	0.07099062	0	[illegible]
ENSG00000226145	0	0	[illegible]
ENSG00000226147	0	0.01659338	[illegible]
ENSG00000226148	0	0	[illegible]
ENSG00000226149	0.11870991	0.09939222	[illegible]
ENSG00000226153	0	0	[illegible]
ENSG00000226155	0.15551141	0.18228701	[illegible]
ENSG00000226156	0	0	[illegible]
ENSG00000226157	0	0	[illegible]
ENSG00000226158	0	0.10255577	[illegible]
ENSG00000226159	0	0	[illegible]
ENSG00000226160	0	0	[illegible]

ENSG00000226161	0	0.01971506	0	0.0380463	0	0	0	0.0288304	0.07275847	0.02665864	0.03708105	0	0	0.03188652	0.07679478	0	0.13283624	0.03550402	0	0	0	0	0.02981606	0.02736477
ENSG00000226163	0	0	0	0	0	0	0	0	0	0	0	0	0	0	0	0	0	0	0	0	0	0	0	0
ENSG00000226164	0	0	0	0	0	0	0	0	0	0	0	0	0	0	0	0	0	0	0	0	0	0	0	0
ENSG00000226166	0	0	0	0	0.16052174	0	0	0	0	0	0	0	0	0	0	0	0	0	0	0	0	0	0	0
ENSG00000226167	0.0557608	0.12449822	0.03635537	0.0480516	0.12441824	0.09759764	0.08956018	0.18206048	0.03675688	0.13467685	0.09366493	0.10987655	0.03146433	0.02013595	0.06465996	0.05379115	0	0.11210178	0.10227618	0.09902269	0.09387795	0.0317494	0.07531392	0.01728052
ENSG00000226168	0	0	0	0	0	0	0	0	0	0	0	0	0	0	0	0	0	0	0	0	0	0	0	0
ENSG00000226169	0	0	0	0	0	0	0	0	0	0	0	0	0	0	0	0	0	0	0	0	0	0	0	0
ENSG00000226170	0	0	0	0	0	0	0	0	0	0	0	0	0	0	0	0	0	0	0	0	0	0	0	0
ENSG00000226172	0	0	0	0	0	0	0	0	0	0	0	0	0	0	0	0	0	0	0	0	0	0.04215425	0	0
ENSG00000226174	0.35589839	0.61700006	0.42995923	0.4600402	0.2919525	0.50252844	0.96672058	0.99455045	0.47093329	1.55484178	0.6725531	0.50387316	0.25693654	0.15875946	0.10924363	0.12117421	0.03149414	0.13889116	0.34559317	0.6970824	0.18504232	0.26820456	0.11663995	0.19463738
ENSG00000226179	0.67772817	0.75658845	0.51551576	0.7787047	0.3780512	0.84730126	0.90711072	1.03264022	0.4095214	1.56868632	1.13842276	0.82671467	1.52969565	1.06052486	2.09570795	0.87171818	0.79298272	1.99834521	1.07734094	1.33726897	0.82406419	1.41492527	1.52563475	0.98014474
ENSG00000226180	0.23462263	0.24813782	0.27776339	0.1596195	0.15843065	0.18525923	0.4065895	0.32657883	0.32356613	0.20131866	0.09334194	0.28156542	0.15677915	0.06688839	0.12887399	0.21442263	0.16719045	0.04468609	0.0815388	0.12061039	0.23908305	0.07382647	0.08756325	0.13776747
ENSG00000226181	0	0	0	0	0	0	0	0	0	0	0	0	0	0	0	0	0	0.12622369	0	0	0	0	0	0
ENSG00000226183	0	0	0	0	0	0	0	0	0	0	0	0	0	0	0	0	0	0	0	0	0	0	0	0
ENSG00000226185	0	0	0	0	0	0	0	0	0	0	0	0	0	0	0	0	0	0	0	0	0	0	0	0
ENSG00000226186	0	0	0.09380984	0	0	0	0	0	0	0	0	0	0	0	0	0	0	0	0	0	0	0	0	0
ENSG00000226188	0	0	0	0	0	0	0	0.05729453	0	0.0529786	0.07369101	0	0	0	0.05087131	0	0	0	0	0	0.04923907	0.04995776	0	0
ENSG00000226191	0	0.02971506	0.02169312	0	0.01237332	0	0.03562683	0.08690788	0.03289905	0.46207691	0.05588953	0.03746454	0	0.0240301	0	0	0	0	0	0	0	0.07577902	0.06740933	0.12373472
ENSG00000226193	0	0	0	0	0	0	0	0	0.08277457	0.37910657	0.31639282	0.28278432	0	0.09069024	0.07280544	0	0	0	0	0	0	0	0	0
ENSG00000226194	0	0	0	0	0	0	0.03607372	0.01099975	0.00555195	0	0	0.00948362	0	0	0	0	0	0	0.02471734	0	0.00945322	0	0	0.01044057
ENSG00000226196	0	0	0	0	0	0	0	0	0	0	0	0	0	0	0	0	0	0	0	0	0	0	0	0
ENSG00000226197	0.06294871	0.08432809	0.06156271	0	0.03511413	0	0	0	0	0	0	0	0	0	0	0	0	0	0	0	0	0	0	0
ENSG00000226199	0	0	0	0	0	0	0	0	0	0	0	0	0	0	0	0	0	0	0	0	0	0	0	0
ENSG00000226200	0.23212957	0.39842792	0.29796185	0.203162	0.17939319	0.21403007	0.51070604	0.47132479	0.28690714	0.83221985	0.618393	0.44720084	0.17396381	0.18074771	0.13669113	0.1632823	0.18915387	0.14291905	0.20490225	0.26193577	0.17505009	0.26227732	0.18370799	0.26077525
ENSG00000226202	0	0	0.05598598	0	0.03193328	0	0	0	0	0	0	0	0	0	0	0	0	0	0	0	0	0	0	0
ENSG00000226203	0.04200227	0.02344482	0	0.0180976	0.02733472	0.0367581	0.07308375	0.01371385	0.00692185	0.03170201	0.03527693	0.00591181	0.00592519	0.02275135	0.03044102	0	0.02106222	0.02533247	0.00770404	0	0.0294643	0.01793662	0	0.00650835
ENSG00000226204	0	0	0	0	0	0	0	0	0	0	0	0	0	0	0	0	0	0	0	0	0	0	0	0
ENSG00000226205	0	0	0	0	0	0	0	0	0	0	0	0	0	0	0	0	0	0	0	0	0	0	0	0
ENSG00000226206	0	0	0	0	0	0	0	0	0	0	0	0	0	0	0	0	0	0	0	0	0	0	0	0
ENSG00000226207	0	0	0	0	0	0	0	0	0	0	0	0	0	0	0	0	0	0	0	0	0	0	0	0

ENSG00000226419	0.43181593	0.35630664	0.34274291	0.4287412	0.3595687	0.47413772	1.13583179	1.09113263	0.91891832	1.24133479	1.11955958	0.83503738	1.01172783	1.05086212	1.05589091	0.83916275	0.93204616
ENSG00000226420	0	0	0	0	0	0	0	0	0	0	0	0	0	0	0	0	0
ENSG00000226421	0	0	0	0	0	0.02695243	0.02885495	0.0351943	0	0	0	0	0	0.03892502	0	0	0
ENSG00000226423	0	0	0	0	0	0	0	0	0	0	0	0	0	0	0.04961079	0	0
ENSG00000226425	0	0	0	0	0	0	0	0	0	0	0	0	0	0	0	0	0
ENSG00000226426	0.20726521	0.27765904	0.20270165	0.1339572	0.20232965	0.27208088	0	0	0	0	0	0	0	0	0	0	0
ENSG00000226427	0.82640358	0.07907689	0	0	0.32927537	0	0	0	0.23346675	0.1069275	0	0.0996996	0.09992517	0	0.20534867	0	0
ENSG00000226428	0	0.03346975	0	0	0	0	0	0	0	0.09051537	0.06295154	0	0.04229391	0	0	0	0
ENSG00000226429	0	0	0	0	0	0	0	0	0	0	0	0	0	0	0	0	0
ENSG00000226430	0	0	0	0	0	0	0	0	0	0	0	0	0	0	0	0	0
ENSG00000226431	0	0	0	0	0	0	0	0	0	0	0	0	0	0	0	0	0
ENSG00000226432	0	0	0.03594083	0	0	0	0	0	0	0	0	0	0.03110556	0	0	0	0
ENSG00000226433	0	0	0	0	0	0	0	0	0	0	0	0	0	0	0	0	0
ENSG00000226434	0	0	0	0	0	0	0.02947938	0	0	0	0	0.03099999	0.06214026	0	0.03192494	0.07082297	0
ENSG00000226435	0	0	0	0	0	0	0	0.00423181	0	0.00391303	0	0.00364852	0.05850847	0.14509222	0.16908234	0.09169011	0.07149297
ENSG00000226436	0.0795796	0.05330368	0.07782742	0	0.0887824	0.11938928	0	0	0	0.07207705	0	0.13440983	0	0	0	0	0
ENSG00000226438	0	0.03868816	0	0	0	0	0	0	0.02855574	0.10462798	0.07276657	0	0.04888812	0	0	0	0
ENSG00000226439	0	0.05370147	0	0.1036336	0.04472248	0.06014012	0.25754122	0.15706113	0	0.07261494	0	0	0	0	0.0697266	0	0
ENSG00000226440	0.00421634	0	0.00412351	0	0	0	0.00677208	0.00412994	0	0	0	0	0.00713751	0.00456773	0.00733388	0.01220223	0
ENSG00000226441	0	0	0.10135083	0	0	0	0	0	0	0	0	0	0	0	0	0	0.15590106
ENSG00000226442	0	0	0	0	0	0	0	0	0	0	0	0	0	0	0	0	0
ENSG00000226443	0	0	0	0	0	0	0.05186952	0.0632651	0.06386415	0.5264947	0.12205535	0.10909014	0	0.03498571	0.02808627	0.03115359	0
ENSG00000226444	0	0	0	0	0	0	0	0	0	0	0	0	0	0	0	0	0
ENSG00000226445	0	0	0	0	0	0	0.84515568	0.51541693	0.65037165	0.74467363	0.66291829	0.58324266	0.30619928	0.35628308	0.25741923	0.22208059	0.14842417
ENSG00000226446	0	0	0	0	0	0	0	0	0	0	0	0	0	0	0	0	0.07770069
ENSG00000226447	0.0725895	0.0486216	0	0	0	0	0	0	0	0	0	0	0	0	0	0	0
ENSG00000226448	0	0	0	0.0548167	0	0	0	0	0	0	0	0	0	0	0	0	0
ENSG00000226449	0	0	0	0	0	0	0	0	0	0	0	0	0	0	0	0	0
ENSG00000226450	0.15141488	0.28977169	0.16923547	0.2236816	0.19305702	0.22716014	0.5558742	0.33899905	0.35290301	0.74447372	0.3815117	0.34707505	0.21970125	0.21090045	0.26337001	0.14606643	0.39048507
ENSG00000226453	0	0	0	0.1013642	0.26245891	0.17646956	0	0.11521638	0.1356919	0	0	0.09933573	0.06637366	0	0	0	0
ENSG00000226454	0	0	0	0	0	0	0	0	0	0	0	0	0	0	0	0	0
ENSG00000226455	0	0	0	0	0	0	0	0	0	0	0	0	0.02108159	0.0539656	0	0.04805453	0.03746927
ENSG00000226457	0.16918499	0	0	0.2186914	0.04718749	0.12690987	0.0679341	0.24857706	0.0418218	0	0.31971453	0.14287659	0.14319986	0	0.1471396	0	0.25451564
ENSG00000226461	0	0	0	0	0	0	0	0	0	0	0	0	0	0	0	0	0
ENSG00000226465	0.60430762	0.94447459	0.39400133	1.0415176	0.44946088	1.20881647	0.08088404	0.09865402	0	0.09122252	0	0.08505622	0.08524866	0.10911169	0	0	0
ENSG00000226466	0	0	0	0	0	0	0.0325162	0	0	0	0	0	0	0	0	0	0
ENSG00000226468	0	0	0	0	0	0.03037227	0	0.03965991	0	0	0	0	0.03427082	0	0	0	0
ENSG00000226469	0	0	0	0.0416607	0.00898922	0.02417633	0.01294145	0.01578464	0.03186821	0.02919121	0.02030187	0.013609	0.01363979	0	0.01401505	0	0.04848523
ENSG00000226470	0	0	0	0	0	0	0	0	0	0	0	0	0	0	0	0	0
ENSG00000226471	0.06494658	0.2283868	0.15879147	0.4197552	0.14491434	0.23141071	0.22166709	0.17494315	0.21673594	0.23529436	0.36819517	0.10969467	0.24737144	0.43974486	0.59308007	0.4385672	0.46409036

[illegible]

ENSG00000226664	0	0	0	0	0	0	0	0	0	0	0	0	0	0.15247049	0	0	0	[illegible]
ENSG00000226665	0	0	0	0	0	0	0	0	0	0	0	0	0	0.04436562	0.03561638	0	0	[illegible]
ENSG00000226666	0	0.04255888	0.07767399	0.0615979	0.04430368	0.03574617	0.01275648	0.03111807	0.02355954	0.05754797	0	0	0.01344484	0.0688334	0	0.04597035	0.02389612	[illegible]
ENSG00000226667	0	0	0	0	0	0	0.28133579	0.51471663	0.3463936	0	0	0	0	0	0	0	0	[illegible]
ENSG00000226668	0	0	0	0	0	0	0	0	0	0	0	0	0	0	0	0	0	[illegible]
ENSG00000226669	0	0	0	0	0	0	0	0	0	0.04299147	0	0	0	0	0	0	0	[illegible]
ENSG00000226670	0	0	0	0	0	0	0	0	0	0	0	0	0	0	0	0	0	[illegible]
ENSG00000226671	0	0	0	0	0	0	0	0	0	0	0	0	0	0	0	0	0	[illegible]
ENSG00000226673	0	0	0	0	0	0	0	0	0.00505524	0	0.0257638	0.00863515	0	0	0	0	0	[illegible]
ENSG00000226674	0.00178425	0.00537803	0.00174496	0.0023063	0.00447881	0.00936885	0.00716442	0.01135994	0.00617481	0.00888818	0.0067435	0.00452038	0.00604082	0.00676531	0.01086227	0.00688489	0.00671039	[illegible]
ENSG00000226675	0.062826	0.04208185	0	0.0812099	0	0.04712735	0.15136195	0	0.12424254	0	0	0.10611302	0.05317655	0	0.05463956	0	0	[illegible]
ENSG00000226676	0.06504488	0.02178405	0.03180636	0.0840781	0.01814171	0.04879178	0.07835386	0.06371198	0.08039407	0.17673788	0.081945	0.02746518	0.08258195	0.10569851	0.14142328	0.09412093	0.09785112	[illegible]
ENSG00000226677	0	0	0	0	0	0	0	0	0.01011689	0	0	0	0	0.02216872	0	0	0	[illegible]
ENSG00000226679	0	0	0.0481223	0	0	0	0.07903173	0.04819738	0.07298064	0.08913345	0.06199045	0.12466255	0.16659281	0.05330648	0.04279404	0.09493521	0.07402325	[illegible]
ENSG00000226680	0	0	0	0	0	0	0	0	0	0	0	0	0	0	0	0	0	[illegible]
ENSG00000226681	0	0	0	0	0	0	0	0	0	0	0	0	0	0	0	0	0	[illegible]
ENSG00000226683	0	0	0.01386718	0.0183285	0.02372869	0.03190893	0	0	0	0	0	0	0	0	0	0	0	[illegible]
ENSG00000226684	0.78529315	2.06017372	1.37600465	1.1842636	0.12776553	1.25176934	0	0.03205004	0	0	0	0	0	0	0	0.03156475	0	[illegible]
ENSG00000226685	0	0	0	0	0	0	0	0	0	0	0	0	0	0	0	0	0	[illegible]
ENSG00000226686	0.43844931	0.83126457	0.58141769	0.8741351	0.56376923	0.95880301	0.52517744	0.55320862	0.37842123	0.93557244	0.53364387	0.42675199	0.06289964	0.11270933	0.07755617	0.11470152	0.12297384	[illegible]
[illegible]	[illegible]	[illegible]	[illegible]	[illegible]	[illegible]	[illegible]	[illegible]	[illegible]	[illegible]	[illegible]	[illegible]	[illegible]	[illegible]	[illegible]	[illegible]	[illegible]	[illegible]	[illegible]

ENSG00000226906	0	0	0	[illegible]
ENSG00000226907	0.01053259	0.0141098	0.01030069	[illegible]
ENSG00000226908	0.09451537	0.12661578	0	[illegible]
ENSG00000226912	0	0	0	[illegible]
ENSG00000226913	0.25425797	0.21117946	0.25860611	[illegible]
ENSG00000226915	0.05518791	0	0	[illegible]
ENSG00000226917	0	0	0	[illegible]
ENSG00000226918	0	0	0	[illegible]
ENSG00000226919	0	0	0	[illegible]
ENSG00000226920	1.14624598	1.76136325	1.64854114	[illegible]
ENSG00000226921	0	0	0	[illegible]
ENSG00000226925	0	0	0	[illegible]
ENSG00000226926	0.01286616	0	0	[illegible]
ENSG00000226927	0	0	0	[illegible]
ENSG00000226928	0	0.10329182	0	[illegible]
ENSG00000226929	0	0	0	[illegible]
ENSG00000226930	0	0	0	[illegible]
ENSG00000226933	0	0	0	[illegible]
ENSG00000226935	0	0	0	[illegible]
ENSG00000226937	0.00399674	0	0.00781749	[illegible]
ENSG00000226938	0	0	0	[illegible]
ENSG00000226939	0	0	0	[illegible]
ENSG00000226941	0	0	0	[illegible]
ENSG00000226942	0	0.07737631	0.01882922	[illegible]
ENSG00000226943	0	0	0	[illegible]
ENSG00000226944	0.17052772	0.62822195	0.3335461	[illegible]
ENSG00000226945	0.15824749	0	0.05158774	[illegible]
ENSG00000226946	0	0	0	[illegible]
ENSG00000226947	0.09916843	0	0	[illegible]
ENSG00000226948	0.0812856	0.10889276	0.07949586	[illegible]
ENSG00000226949	0	0	0	[illegible]
ENSG00000226950	4.11967905	4.51256613	4.30445739	[illegible]
ENSG00000226952	0	0	0	[illegible]
ENSG00000226953	0.02060725	0.08281838	0.02015352	[illegible]
ENSG00000226954	0	0.02859336	0	[illegible]
ENSG00000226955	0	0	0	[illegible]
ENSG00000226956	0	0	0	[illegible]
ENSG00000226957	0	0	0	[illegible]
ENSG00000226958	1216.67268	2594.00695	2141.76206	[illegible]
ENSG00000226960	0	0	0	[illegible]
ENSG00000226961	0.03255529	0.10903026	0.03183849	[illegible]
ENSG00000226962	0	0	0	[illegible]
ENSG00000226963	0.05775939	0.03868816	0.05648765	[illegible]
ENSG00000226964	0.10743247	0	0	[illegible]
ENSG00000226965	0.4557741	0.39250892	1.01883173	[illegible]
ENSG00000226967	0	0	0	[illegible]
ENSG00000226968	0	0	0	[illegible]
ENSG00000226969	0	0	0	[illegible]
ENSG00000226970	0.26417819	0.17695074	0.12918076	[illegible]
ENSG00000226971	0	0	0.02369933	[illegible]
ENSG00000226972	0.06631634	0.04441973	0	[illegible]
ENSG00000226973	0	0	0	[illegible]
ENSG00000226974	0	0	0	[illegible]
ENSG00000226975	0.122314	0.2867475	0	[illegible]
ENSG00000226976	0.05644438	0	0.11040318	[illegible]
ENSG00000226977	0.21630698	0.14488584	0.21154434	[illegible]
ENSG00000226978	0	0	0	[illegible]
ENSG00000226979	0	0.00744029	0	[illegible]
ENSG00000226981	0	0	0	[illegible]
ENSG00000226982	0	0.05024358	0.02445315	[illegible]
ENSG00000226983	0	0	0	[illegible]
ENSG00000226984	0	0	0	[illegible]
ENSG00000226985	0	0	0	[illegible]
ENSG00000226986	0.04139979	0.04159536	0.08097651	[illegible]
ENSG00000226987	0	0	0.0563866	[illegible]
ENSG00000226989	0.24354967	0.27188905	0.15879147	[illegible]
ENSG00000226990	0	0	0	[illegible]
ENSG00000226991	0	0	0	[illegible]
ENSG00000226992	0	0	0	[illegible]
ENSG00000226994	0	0.00856666	0.00416933	[illegible]
ENSG00000226995	0	0.00410965	0	[illegible]
ENSG00000226996	0.06058222	0.06086839	0.02962416	[illegible]
ENSG00000226998	0	0	0	[illegible]
ENSG00000226999	0	0	0	[illegible]
ENSG00000227000	0	0	0	[illegible]
ENSG00000227001	0.93705441	1.25530779	0.98877152	[illegible]
ENSG00000227002	0.11762679	0	0	[illegible]
ENSG00000227004	0	0	0	[illegible]
ENSG00000227005	0	0	0	[illegible]
ENSG00000227006	0	0	0	[illegible]
ENSG00000227007	0.06125871	0.11796716	0.14228606	[illegible]
ENSG00000227008	0	0.05189421	0.22730846	[illegible]
ENSG00000227009	0	0	0	[illegible]
ENSG00000227011	0	0	0	[illegible]
ENSG00000227012	0	0	0	[illegible]
ENSG00000227013	0	0	0.03229519	[illegible]
ENSG00000227014	0.02133007	0.05714888	0.04172086	[illegible]
ENSG00000227015	0	0.06581704	0.09609789	[illegible]
ENSG00000227016	0	0	0	[illegible]
ENSG00000227017	0	0	0	[illegible]
ENSG00000227018	0.03159778	0	0	[illegible]
ENSG00000227019	0	0	0	[illegible]
ENSG00000227021	0	0	0.05826267	[illegible]
ENSG00000227023	0	0	0	[illegible]
ENSG00000227028	0.03152978	0.0295668	0.03083556	[illegible]
ENSG00000227029	0	0	0	[illegible]
ENSG00000227032	0	0.02597833	0	[illegible]
ENSG00000227033	0	0	0	[illegible]
ENSG00000227034	0.2508151	0.50399978	0.12264633	[illegible]
ENSG00000227035	0	0	0	[illegible]
ENSG00000227036	2.63418564	3.23533098	2.7732177	[illegible]
ENSG00000227038	0.10723388	0.11971159	0.12817788	[illegible]
ENSG00000227039	0	0	0	[illegible]
ENSG00000227040	0	0.07614811	0	[illegible]
ENSG00000227042	0.10549833	0	0.20635094	[illegible]
ENSG00000227043	0	0	0	[illegible]
ENSG00000227045	0.16699347	0.22370975	0.16331661	[illegible]
ENSG00000227047	0	0	0	[illegible]
ENSG00000227048	0	0	0	[illegible]
ENSG00000227050	0	0	0	[illegible]
ENSG00000227051	6.09510193	5.95765762	5.50561648	[illegible]
ENSG00000227053	0	0	0	[illegible]
ENSG00000227054	0.42972986	0.28783987	0.08405362	[illegible]
ENSG00000227055	0	0	0	[illegible]
ENSG00000227056	0	0	0.04025556	[illegible]
ENSG00000227057	3.86564151	3.08328936	3.06646852	[illegible]
ENSG00000227058	0	0	0	[illegible]
ENSG00000227059	0	0	0	[illegible]
ENSG00000227060	0	0.0275357	0.06030633	[illegible]
ENSG00000227061	0	0	0	[illegible]
ENSG00000227062	0	0	0	[illegible]
ENSG00000227063	0	0	0	[illegible]
ENSG00000227064	0.10851764	0.43612102	0.21225661	[illegible]
ENSG00000227066	0.02703837	0.03018455	0.01762869	[illegible]
ENSG00000227067	0.03385477	0.1133823	0.06621871	[illegible]
ENSG00000227068	0.05091586	0.17052125	0.09958959	[illegible]
ENSG00000227069	0	0	0	[illegible]
ENSG00000227070	0	0	0	[illegible]
ENSG00000227071	0.05595441	0	0	[illegible]
ENSG00000227072	0	0	0	[illegible]
ENSG00000227073	0	0	0	[illegible]
ENSG00000227075	0.06081083	0.12219617	0.2973595	[illegible]
ENSG00000227076	0.17374523	0.23275462	0	[illegible]
ENSG00000227077	8.82018696	7.54279188	9.1142477	[illegible]
ENSG00000227078	0	0	0	[illegible]
ENSG00000227080	0.18820286	0.18909189	0.09202951	[illegible]
ENSG00000227081	0.22342974	0.22448517	0.05462757	[illegible]
ENSG00000227082	1.02973226	0.7074164	0.63694372	[illegible]
ENSG00000227083	0	0	0.05902642	[illegible]
ENSG00000227084	0	0	0	[illegible]
ENSG00000227087	0	0	0	[illegible]
ENSG00000227088	0	0.1520281	0	[illegible]
ENSG00000227089	0	0	0	[illegible]
ENSG00000227090	0	0	0	[illegible]
ENSG00000227091	0	0	0	[illegible]
ENSG00000227094	0.43637946	0.1252688	0.36580395	[illegible]
ENSG00000227096	0	0	0	[illegible]
ENSG00000227097	0.11037582	0	0.05397279	[illegible]
ENSG00000227098	0	0	0	[illegible]
ENSG00000227101	0	0	0	[illegible]
ENSG00000227102	0	0	0	[illegible]
ENSG00000227104	0	0	0	[illegible]
ENSG00000227105	0.03195282	0.03567084	0.02083285	[illegible]
ENSG00000227107	0	0	0	[illegible]
ENSG00000227108	0	0	0	[illegible]
ENSG00000227109	0	0	0	[illegible]
ENSG00000227110	0.01460507	0.02690245	0.01071262	[illegible]
ENSG00000227112	0	0	0	[illegible]
ENSG00000227113	0	0.37220673	0.27172506	[illegible]
ENSG00000227114	0	0	0	[illegible]
ENSG00000227115	0.01343839	0.03600499	0.02628501	[illegible]
ENSG00000227116	0	0.04593189	0.06706406	[illegible]
ENSG00000227117	0.02918039	0.03909097	0	[illegible]
ENSG00000227118	0	0	0	[illegible]
ENSG00000227120	0	0	0	[illegible]
ENSG00000227121	0	0	0	[illegible]
ENSG00000227123	0	0	0	[illegible]
ENSG00000227124	1.08350065	1.60637661	1.13071787	[illegible]
ENSG00000227125	0	0	0	[illegible]
ENSG00000227128	0	0	0	[illegible]
ENSG00000227131	0	0	0	[illegible]
ENSG00000227133	0	0.04974191	0.07262697	[illegible]
ENSG00000227134	0	0	0	[illegible]
ENSG00000227135	0	0	0	[illegible]
ENSG00000227136	0.06374553	0.16225151	0.13715236	[illegible]
ENSG00000227138	0	0	0	[illegible]
ENSG00000227139	0	0	0	[illegible]
ENSG00000227140	0	0	0	[illegible]
ENSG00000227141	0.0130432	0.01747308	0.02551202	[illegible]
ENSG00000227143	0	0	0	[illegible]
ENSG00000227145	0	0	0	[illegible]

ENSG00000227148	0	0	0	0	0	0	0	0	0	0	0	0	0	0	0	0	0	0	0	0	0	0	0
ENSG00000227149	0	0	0.12971237	0	0	0	0	0	0	0	0	0	0	0	0	0	0	0	0	0	0	0	0
ENSG00000227150	0	0	0	0	0	0	0.0924389	0	0.11381504	0.10425431	0	0	0.29228113	0	0	0	0	0	0	0.10220556	0.09689546	0.09830974	0
ENSG00000227151	0	0	0	0	0	0	0	0	0	0	0	0	0	0	0	0	0	0	0	0	0	0	0
ENSG00000227152	0	0	0	0	0	0	0	0	0	0	0	0	0	0	0	0	0	0	0	0	0	0	0
ENSG00000227154	0	0	0	0	0	0	0	0	0	0.04501343	0	0	0	0.05384077	0	0	0	0	0	0	0	0	0
ENSG00000227155	0.00507315	0.00339808	0.00496145	0.0032788	0.00282991	0	0	0	0	0.0068923	0.00319564	0	0	0	0	0	0	0	0	0	0	0	0
ENSG00000227157	0.47899826	0.45834375	0.86998171	0.7960644	0.38170775	0.92393615	0	0	0	0	0	0	0	0	0	0	0	0	0	0.06075914	0.0576024	0	0
ENSG00000227158	0	0	0	0	0	0	0.02527626	0	0	0	0	0.07974021	0.02664021	0	0.10949255	0	0.04734886	0	0.13855226	0.19562782	0.34443308	0.02688157	0.22318368
ENSG00000227159	0	0	0	0	0	0	0.01755367	0	0	0	0	0	0	0	0	0	0	0	0	0	0	0	0
ENSG00000227160	0	0	0.02201125	0.0581853	0	0.06753165	0	0	0	0	0	0	0	0.0731475	0.01957409	0	0	0.05429734	0	0	0	0	0
ENSG00000227161	0	0.89022642	0	0	0.74137876	0.49848102	1.33416972	0	1.1498839	2.10658189	0.83719062	1.68358705	0	1.07986829	0.57794008	0.64105736	1.9993909	0.40079276	0.73132739	0.88507905	0.55939647	1.1351228	1.68291669
ENSG00000227163	0.06198027	0.20757683	0.12123118	0.3204669	0	0.27895765	0.09954959	0	0.21449757	0.28068468	0.23425238	0.10468458	0.15738215	0.06714566	0.05390403	0.05979093	0	0.07476326	0.20463103	0.22013505	0.15652344	0.21174406	0.06278574
ENSG00000227164	0	0	0	0	0	0	0	0	0	0	0	0	0	0	0	0	0	0	0	0	0	0	0
ENSG00000227165	0.0270293	0.05069303	0.07401568	0.0279508	0.02412403	0.07299127	0.03038917	0.05295083	0.03741655	0.09792421	0.02043127	0.01826098	0.03660459	0.01756914	0.04701458	0.02085963	0.00813238	0.03912469	0.01784772	0.04319993	0.02275304	0.01385109	0.02738056
ENSG00000227166	0	0	0	0	0	0	0	0	0	0	0	0	0	0	0	0	0	0	0	0	0	0	0
ENSG00000227167	0	0	0	0	0	0	0	0	0	0	0	0	0	0	0	0	0	0	0	0	0	0	0
ENSG00000227169	0	0	0	0	0	0	0	0	0	0	0	0	0	0	0	0	0	0	0	0	0	0	0
ENSG00000227170	0	0	0	0	0	0	0.06438531	0	0	0	0	0	0.06785963	0.08685508	0	0	0	0	0	0.07118795	0.06748937	0	0
ENSG00000227172	0.23354884	0.15643471	0	0.2012594	0.08685234	0.29198465	0.18755719	0	0.07697635	0.3525508	0.58846008	0.32871969	0	0.25301262	0	0	0.11711408	0	0	0.13824906	0.19659948	0	0.07886131
ENSG00000227173	0	0.0999444	0	0	0.0832335	0	0	0.07307705	0.0368845	0	0.1879803	0.06300461	0	0	0	0.07197056	0	0	0.08210504	0	0.06280261	0	0
ENSG00000227175	0	0.1704315	0.08294765	0.1096334	0.04731167	0.25448768	0.06811288	0	0	0.15363793	0	0.07162629	0	0	0	0.08181916	0	0	0.09334047	0	0	0	0
ENSG00000227176	0	0	0.08165831	0.0539646	0	0.06263298	0	0	0	0	0	0	0	0	0	0.04027368	0	0.05035868	0	0	0	0	0
ENSG00000227177	0	0	0	0	0	0	0	0	0	0	0	0	0	0	0	0	0.15540138	0	0	0	0	0	0
ENSG00000227179	0	0.01098067	0	0.0211906	0.01828935	0.01229722	0	0	0.00810483	0	0	0	0	0	0	0	0	0	0.01804139	0.02911247	0	0.02800278	0
ENSG00000227180	0	0	0	0	0	0	0	0	0	0	0	0	0	0	0	0	0	0	0	0	0	0	0
ENSG00000227181	0	0	0	0	0	0	0	0	0	0	0	0	0	0	0	0	0	0	0	0	0	0	0
ENSG00000227182	0	0.02409374	0	0	0	0.02698251	0	0	0	0	0	0	0	0	0	0	0	0	0	0	0	0	0
ENSG00000227183	18.0333068	15.3343266	18.7619682	20.16907	22.6157298	17.4606823	0.10270989	0.12527495	0	0.11583812	0	0.1080079	0	0	0	0	0	0	0.1407515	0	0	0.32769914	0
ENSG00000227184	0.00806046	0.04319218	0.01182447	0.0104191	0.00674444	0.01511587	0.12946301	0.09474339	0.18729598	0.05475404	0.06600584	0.0544564	0.04093471	0.06112547	0.02453553	0.03110295	0.03031464	0.04861435	0.04878869	0.05367805	0.09838579	0.0103264	0.01224781
ENSG00000227185	0	0	0.07330257	0	0	0	0	0	0	0	0	0	0	0	0	0	0	0	0	0	0	0	0
ENSG00000227186	0	0.1704315	0.08294765	0	0.04731167	0.25448768	0	0	0	0.07681896	0	0.07162629	0	0	0.14752681	0.08181916	0	0	0.18668094	0.07530936	0.14279331	0	0
ENSG00000227187	0	0.15605776	0	0	0	0.23302486	0	0	0.07679087	0	0	0.06558552	0	0	0	0	0	0	0	0	0	0	0
ENSG00000227188	0	0	0	0	0	0	0	0	0	0.05861688	0	0	0	0	0	0	0	0	0	0	0	0	0
ENSG00000227189	0	0	0	0	0	0	0	0	0	0	0	0	0	0	0	0	0	0	0	0	0	0	0
ENSG00000227191	0	0	0	0	0	0	0.02555073	0	0	0	0	0	0	0	0	0	0	0	0	0	0	0	0
ENSG00000227192	0	0	0	0	0.03256963	0	0.0468893	0.05719074	0.02886613	0	0.14711502	0	0.04941952	0.06325315	0.05077916	0	0	0	0.19276836	0.0518434	0.04914987	0.04986726	0.05914598
ENSG00000227193	0	0	0	0	0	0	0	0	0	0	0	0	0	0	0.05674108	0	0	0	0	0	0	0	0.06609025
ENSG00000227194	0	0	0	0	0	0	0	0	0	0	0	0	0	0	0	0	0	0	0	0	0	0	0
ENSG00000227195	6.85215428	6.68209984	6.94327112	8.600672	9.31738559	7.3227916	10.0782762	11.4264938	13.7748555	13.2760518	32.7489362	13.2961011	5.84772634	10.5240057	4.82168273	8.29341204	8.98398787	22.955542	37.7967507	8.71834776	9.26777622	11.1905411	7.404645
ENSG00000227196	0	0	0	0	0	0	0	0	0	0	0	0	0	0	0	0	0	0	0	0	0	0	0
ENSG00000227197	0	0	0	0	0	0	0	0	0	0	0	0	0	0	0	0	0	0	0	0	0	0	0
ENSG00000227198	0	0	0.04683523	0	0	0.10776967	0.03845898	0.0469083	0	0	0	0	0.04053428	0	0.04164947	0.04619804	0	0	0.05270339	0	0	0	0
ENSG00000227199	4.50431514	7.07409537	5.18938759	4.6314174	3.91166585	4.72263925	1.4661035	1.05286663	0.94474315	1.5453259	0.98876246	0.61957311	1.21306728	1.58959967	1.54321321	0.75711962	1.48869418	1.52297005	1.44581374	1.43921007	0.38778702	1.1511972	0.58763994
ENSG00000227200	0.60484766	0.75239645	0.76053877	0.5584545	0.3373969	0.77779076	0.97147586	0.76172542	0.42718781	0.62608485	1.19742948	0.87564581	0.58508466	0.74886309	0.45088623	0.50012786	0.38996163	0.31268282	0.2852765	0.38361335	0.43641923	0.66418378	0.61270801
ENSG00000227201	0	0	0.03433563	0	0	0	0	0	0	0	0	0	0	0	0	0	0	0	0	0	0	0	0
ENSG00000227203	0.25245227	0.31001031	0.24689379	0.0543873	0.39899921	0.22093251	0.03378968	0	0.0208017	0.07621725	0	0.03553262	0	0	0.03659281	0.04058914	0	0.10150626	0	0.03735973	0	0.1437427	0
ENSG00000227204	0	0	0	0	0	0	0	0	0	0	0	0	0	0	0	0	0	0	0	0	0	0	0
ENSG00000227205	0	0	0	0	0	0	0	0.07290828	0	0	0	0	0	0	0	0	0	0	0	0	0	0	0
ENSG00000227206	0	0.05549612	0	0	0	0	0	0	0	0	0	0	0	0	0	0	0	0	0	0	0	0	0
ENSG00000227207	0.09002721	0	0	0	0	0	0	0	0	0	0	0	0	0.09753	0	0	0	0.10859469	0	0	0	0	0
ENSG00000227208	0	0	0	0	0	0	0.06026285	0.06125201	0.030916	0.06796556	0.04726862	0	0.02117157	0.02709798	0.02175405	0	0	0.04525832	0.02752765	0.01110499	0	0.01068169	0.02533844
ENSG00000227209	0	0	0	0	0	0	0	0	0	0	0	0	0	0	0	0	0	0	0	0	0	0	0
ENSG00000227210	0.11914876	0.23942319	0.11652535	0.3080274	0.09969557	0.1787529	0.9568537	0.46682864	0.53015508	1.40290455	0.75053133	0.55341571	0	0	0.05181163	0.05747002	0.0896215	0.07186118	0.5900636	0.37028261	0.25074611	0.45793078	0.30174292
ENSG00000227211	0	0	0	0	0	0	0	0	0	0	0	0	0	0	0	0	0	0	0	0	0	0	0
ENSG00000227212	0	0	0	0	0	0	0	0	0	0	0	0	0	0	0	0	0	0	0	0	0	0	0
ENSG00000227213	0	0	0	0	0	0	0	0	0	0	0	0	0	0	0	0	0	0	0	0	0	0	0

ENSG00000227388	0.22161904	0.44533183	0.21673944	0.3683166	0.22958709	0.2849862	0	0.03101109	0	0.02867506	0	0	0	0.03429837	0.02753447	0	0	0	0.24389553	0.44978477	0.13325505	0.13520004	0.0962139	0.14717301	0.06044606	0.07816524	0.16344476	0.2148506	0.1136747	0.12733765	0.19241093	0.69723145	0.98683992	1.19587486	1.10987594	0.92936851	0	0	0.03188982	0	0.05914397	0	0	0.02702981	0.02920396	0.04348493	0.02819251	0.05919862
ENSG00000227391	0	0	0	0.0108576	0.00468554	0	0	0	0	0	0	0	0	0	0	0.00810302	0	0	0	0	0	0	0	0	0	0	0	0	0	0	0	0	0	0	0	0	0	0	0	0	0	0	0	0	0	0	0	0
ENSG00000227392	0	0	0	0	0	0	0	0.02808655	0.01417625	0.05194165	0	0	0	0	0.04987561	0	0	0	0	0.02546046	0	0	0.02904678	0.02665874	0.02737282	0.04719586	0	0	0	0	0	0.03508213	0.04704079	0	0	0	0.02653457	0	0.02888242	0.04605911	0	0.10269253	0.03283034	0	0	0.03938404	0	0
ENSG00000227393	0	0	0	0.0493026	0	0	0	0	0	0	0	0	0	0	0	0	0	0.04600816	0	0	0	0	0	0	0	0	0	0	0	0	0	0	0	0	0	0	0	0	0	0	0	0	0	0	0	0.05238777	0	0
ENSG00000227394	0.11075512	0.1112783	0.16247478	0.1431639	0	0.12462025	0.04447232	0	0.08213456	0.15047014	0.06976589	0.09353261	0	0	0	0	0	0	0.2437758	0.09834212	0.04661637	0	0.05609722	0	0	0.04557401	0.09529597	0.08351199	0.07953322	0.08909259	0	0	0	0	0	0.0492605	0	0	0.05577979	0	0	0.19832715	0	0	0.05108185	0.07606128	0.04931268	0.05177336
ENSG00000227395	0	0	0	0	0	0	0	0	0	0	0	0	0	0	0	0	0	0	0	0	0	0	0	0	0	0	0	0	0	0	0	0	0	0	0	0	0	0	0	0	0	0	0	0	0	0	0	0
ENSG00000227397	0	0	0	0	0	0	0	0	0	0	0	0	0	0	0	0	0	0	0	0	0	0	0	0	0	0	0	0	0	0	0	0	0	0	0	0	0	0	0	0	0	0	0	0	0	0	0	0
ENSG00000227398	0.19082533	0.2335981	0.16731784	0.6549355	0.16150493	0.58244321	0.44388791	0.54140876	0.31555905	0.24435269	0.36475394	0.18337968	0.60150956	0.65582855	0.58372184	0.35547403	0.35636347	0.67467054	0.53587873	0.82382103	0.31019208	0.69687919	0.55991854	0.3425904	0.60931061	0.42780762	0.617128	0.47631504	0.63790582	0.55048965	1.04975523	0.9419315	1.00393375	1.79163524	1.78133553	0.80190622	0.31663795	0.33153421	0.33139893	0.49677633	0.46711419	0.45953754	1.00954981	1.18537124	1.24429876	0.70495666	1.08987256	1.71638529
ENSG00000227399	0	0.04469563	0	0	0	0.05005451	0.05358777	0.06536084	0	0.06043728	0.08406573	0.05635195	0	0	0.17409996	0	0	0.08049047	0	0	0	0	0.06759541	0	0	0.05491526	0.05741434	0.15094404	0	0	0	0	0	0.10502076	0	0	0	0	0	0	0	0	0	0	0	0	0	0
ENSG00000227400	0	0	0	0	0	0	0	0	0	0	0	0	0	0	0	0	0	0	0	0	0	0	0	0	0.03924368	0	0	0	0	0	0	0	0.06744113	0	0.04003166	0.01828419	0	0	0	0	0	0	0.023534	0	0	0	0	0
ENSG00000227401	0	0	0	0	0	0	0	0	0.16831802	0.10278594	0	0	0	0	0	0	0	0	0	0	0	0	0	0	0.10833467	0	0.09764482	0.17114078	0	0	0	0	0	0	0	0	0	0	0	0	0	0	0	0.09688853	0	0	0	0
ENSG00000227403	0	0	0	0	0	0	0	0	0.00514335	0.0094226	0	0	0	0	0	0	0	0	0.02289824	0.01847486	0.0087575	0	0.0105386	0.01934437	0	0	0.0179026	0	0.01494136	0	0	0	0	0	0.02026135	0	0	0	0	0	0	0	0.01191133	0	0.01919279	0	0	0.01945261
ENSG00000227404	0	0	0	0	0	0	0	0	0	0	0	0	0	0	0	0	0	0	0	0	0	0	0	0	0	0	0	0.01724769	0	0	0	0	0	0	0	0	0	0	0	0	0	0	0	0	0	0	0	0
ENSG00000227406	0	0	0	0	0	0	0	0	0	0	0	0	0	0	0	0	0	0	0	0	0	0	0	0	0	0	0	0	0	0	0	0	0	0	0	0	0	0	0	0	0	0	0	0	0	0	0	0
ENSG00000227407	0.18277735	0.12242717	0.11916865	0.0787537	0.0339857	0.13710584	0.44035167	0.29838645	0.30121182	0.60700051	0.15351132	0.25725889	0.05156819	0.13200658	0.21194778	0	0.0916545	0.1469826	0.13409973	0.2704873	0.10257364	0.1040708	0.18515265	0.05664353	0	0.05014002	0.36695254	0.04593949	0.08750158	0.24504674	0.09256821	0.07454123	0.09995056	0	0	0.05419586	0.33827818	0.12278673	0.18410495	0.19572943	0	0.10909868	0.06975672	0.26007885	0.22479876	0.33472713	0.10850653	0.45568389
ENSG00000227408	0	0	0	0	0	0	0	0	0	0	0	0	0	0	0	0	0	0	0	0.02797415	0	0	0	0	0	0	0	0	0	0	0	0	0	0	0	0	0	0	0	0	0	0	0	0	0	0	0	0
ENSG00000227409	0	0	0	0	0	0	0.05638974	0	0	0	0	0	0	0	0	0	0	0	0	0	0	0	0	0	0	0	0	0	0	0	0	0	0	0.11051204	0	0	0	0	0	0	0	0.12573682	0	0	0	0	0	0
ENSG00000227411	0	0	0	0	0	0	0.04225778	0	0	0	0	0	0	0	0	0	0	0	0	0.02336127	0	0	0	0	0	0	0	0	0	0.02116404	0.07994871	0.06437928	0.04316232	0.04140818	0	0	0	0	0.02650109	0	0	0	0.03012351	0	0	0.07227374	0	0
ENSG00000227412	0	0	0	0	0	0	0	0	0	0	0	0	0	0	0	0	0	0	0	0	0	0	0	0	0	0	0	0	0	0	0	0	0	0	0	0	0	0	0	0	0	0	0	0	0	0	0	0
ENSG00000227413	0	0	0	0	0	0	0	0	0	0	0	0	0.07684387	0	0	0	0	0	0	0	0	0	0	0	0	0	0	0	0	0	0	0	0	0	0	0	0	0	0	0	0	0	0	0	0	0	0	0
ENSG00000227415	0	0	0	0	0	0	0	0	0	0	0	0	0	0	0	0	0	0	0	0	0	0	0	0	0	0	0	0	0	0	0	0	0	0	0	0	0	0	0	0	0	0	0	0	0	0	0	0
ENSG00000227416	0	0	0.07347344	0	0	0.05635508	0	0	0	0	0	0	0	0	0	0	0	0	0	0	0	0	0	0	0	0	0	0	0	0	0	0	0	0	0	0	0	0	0	0	0	0	0	0	0	0	0	0
ENSG00000227417	0.10024802	0.03357386	0	0	0	0	0	0	0	0.04539845	0	0	0	0	0	0	0	0	0	0	0	0	0	0	0.04784921	0	0	0.0377947	0	0	0	0.06132552	0	0.07888806	0.09761999	0.08917453	0.09276784	0	0	0	0.09363695	0	0	0	0	0	0	0
ENSG00000227418	0	0	0	0	0	0	0	0	0	0.02485416	0	0	0	0	0	0	0	0	0	0	0	0	0	0	0	0	0	0	0	0.01103701	0.04169313	0	0	0	0	0	0.01269683	0	0.01382028	0	0	0	0	0	0	0	0	0
ENSG00000227421	0	0	0	0	0	0	0.08335875	0	0.05131757	0	0.13076891	0.08765859	0	0	0	0	0.07807605	0.1878111	0	0	0	0	0	0	0	0	0	0	0	0	0	0.12699617	0.08514307	0	0	0	0	0.15689416	0.15683014	0.25009872	0.19390841	0.27880774	0.05942239	0.04430973	0.04787381	0	0	0.19408758
ENSG00000227423	0	0	0	0	0	0	0	0	0	0	0	0	0	0	0	0	0	0	0	0	0	0	0	0	0	0	0	0	0	0	0	0	0	0	0	0	0	0	0	0	0	0	0	0	0	0	0	0
ENSG00000227425	0	0	0.11939434	0	0	0	0	0	0	0	0	0	0	0	0	0	0	0	0	0	0	0	0	0	0	0	0	0	0	0	0	0	0	0	0	0	0	0	0	0	0	0	0	0	0	0	0	0
ENSG00000227426	0	0	0	0	0	0	0	0	0	0	0	0	0	0	0	0	0	0	0	0	0	0	0	0	0	0	0	0	0	0	0	0	0	0	0	0	0	0	0	0	0	0	0	0	0	0	0	0
ENSG00000227427	0	0	0	0	0	0	0	0	0.01637626	0	0	0	0	0	0	0	0	0	0	0	0	0	0	0	0	0	0	0.02497635	0	0	0	0	0	0	0	0	0	0	0	0	0	0	0	0	0	0	0	0
ENSG00000227430	0	0	0	0	0	0	0	0	0	0	0	0	0	0	0	0	0	0	0	0	0	0	0	0	0	0	0	0	0	0	0	0	0	0	0	0	0	0	0	0	0	0	0	0	0	0	0	0
ENSG00000227431	0	0	0.03545569	0.0468624	0.04044642	0.02719497	0.08734384	0.14204404	0.01792363	0.032836	0	0	0	0.0392753	0.03152991	0.03497332	0	0.08746209	0	0.09657218	0.03051826	0	0.07345013	0.06741153	0.1384344	0.0596717	0.09358086	0.05467264	0	0.05832609	0.11016554	0	0.05947564	0.17117556	0	0.03224928	0.10064632	0.07306432	0.10955176	0.05823446	0	0	0	0	0.03344166	0	0.03228344	0.03389437
ENSG00000227432	0	0	0.06073238	0.0802711	0	0	0	0	0	0.0562451	0	0	0	0	0	0	0	0	0	0.1654194	0.05227501	0	0	0	0	0	0	0	0	0.04995365	0	0.07597748	0	0	0	0	0	0	0	0	0	0	0	0	0	0	0.05529861	0
ENSG00000227433	0	0	0	0	0	0	0	0	0	0	0.11063691	0	0	0	0	0	0	0	0	0	0	0	0	0	0	0	0	0	0	0	0	0	0	0	0	0	0	0	0	0	0	0	0	0	0	0	0	0
ENSG00000227434	0	0	0	0	0	0	0	0	0	0	0	0	0	0	0	0	0	0	0	0	0	0	0	0	0	0	0	0	0	0	0	0	0	0	0	0	0	0	0	0	0	0	0	0	0	0	0	0
ENSG00000227436	0.11288876	0.11342202	0.05520159	0.1459219	0	0.16936133	0.04532906	0.33172631	0.08371684	0.10224591	0.42665932	0.1430017	0	0.06114841	0.04908948	0.16335174	0.08491284	0.13617127	0.124236	0.05011831	0.04751441	0.04820793	0.17153371	0.20990841	0.05388274	0.23225983	0.24282949	0.12768121	0.08106539	0	0	0.13811668	0	0	0	0	0.31339607	0.28438784	0.11370872	0.18133252	0.31633219	0.50536955	0.45238028	0.04818974	0.10413182	0	0.20105063	0.05277075
ENSG00000227437	0	0	0	0	0	0	0	0	0.02541325	0.04655695	0	0	0	0	0	0	0	0	0	0	0	0	0	0.04779015	0	0.04230315	0	0	0	0	0	0	0	0	0	0	0.04756756	0	0	0	0	0	0	0	0	0	0	0.04805757
ENSG00000227438	0	0	0	0	0	0	0.96290523	0.51675916	0.18969173	1.34661815	0.24168896	0.28352074	0.36535142	0.46762154	0.33369159	0.37013431	0.43290383	0.57852526	1.47789077	2.93840977	0.56522352	0.40962393	0.68017883	0.80261853	0.50362722	0.94728831	2.88865915	1.15723764	1.06766252	1.23456882	0	0	0	0	0	0	0.44382231	0.43496104	0.67632998	0.30815735	0.44798036	0.17176548	0.05491266	0.69609796	0.48664582	0.39524699	0.5979162	0.71743089
ENSG00000227439	0	0	0	0	0	0	0	0	0	0	0	0	0	0	0	0	0	0	0	0	0	0	0	0	0	0	0	0	0	0	0	0	0	0	0	0	0	0	0	0	0	0	0	0	0	0	0	0
ENSG00000227440	0	0	0	0	0	0	0.11738273	0	0	0	0	0	0	0	0	0	0	0	0	0.06489242	0	0	0	0	0	0.12029058	0	0	0	0	0	0	0	0	0	0	0	0	0.07361415	0	0	0	0	0	0	0	0	0
ENSG00000227443	0	0	0	0	0	0	0	0	0	0	0	0	0	0	0	0	0	0	0	0	0	0	0	0	0	0	0	0	0	0	0	0	0	0	0	0	0	0	0	0	0	0	0	0	0	0	0	0
ENSG00000227444	0	0	0	0	0	0	0	0	0	0	0	0	0	0	0	0	0	0	0	0	0	0	0	0	0	0	0	0	0	0	0	0	0	0	0	0	0	0	0	0	0	0	0	0	0	0	0	0
ENSG00000227445	0	0	0	0	0	0	0	0	0	0	0	0	0	0	0	0	0	0	0	0	0	0	0	0	0	0	0	0	0	0	0	0	0	0	0	0	0	0	0	0	0	0	0	0	0	0	0	0
ENSG00000227447	0	0	0	0	0	0	0	0	0	0	0	0	0	0	0	0	0	0	0	0	0	0	0	0	0	0	0	0	0.05189275	0	0	0	0	0	0	0	0	0	0	0	0	0	0	0	0	0	0	0
ENSG00000227449	0.04846577	0.09738943	0.07109798	0.1148541	0.06308223	0.10906615	0.01297388	0.0079121	0.00399351	0.04389655	0.02035275	0.00682155	0.05469588	0.03500325	0.07727595	0.14805372	0.15797193	0.15589733	0	0.0358616	0.00679968	0	0	0	0.00771104	0.00664764	0.01390031	0	0	0	0.08590979	0.1284762	0.09276113	0.13984343	0.15731742	0.0431122	0.0074749	0.03255849	0	0	0	0.01446446	0.1942174	0.2344751	0.11921658	0.06656791	0.14385954	0.1812457
ENSG00000227450	0.23750729	0.19090338	0.06968336	0.0921018	0.06624331	0.19597614	0.24795697	0.27916834	0.2113588	0.53778936	0.32913869	0.10028736	0.16082283	0.12865048	0.3718067	0.13747066	0.25010804	0.0572983	0.33979508	0.29524376	0.31989069	0.22313486	0.1443563	0.11040687	0.45345681	0.25409944	0.42914789	0.21490338	0.18760932	0.22926407	0.3247732	0.40681825	0.46756531	0.26166189	0.09251238	0.211272	0.37363493	0.26326308	0.40669511	0.34335587	0.39931985	0.38276995	0.16315979	0.42582402	0.2848086	0.09786514	0.23264536	0.44409871
ENSG00000227453	0.06693612	0.02241744	0.03273116	0	0	0	0	0	0	0.03031278	0	0	0	0	0.02910705	0	0	0	0	0	0	0	0.03390299	0.03111571	0.06389833	0.02754317	0	0	0.12016702	0.02692206	0	0	0	0	0	0	0	0.03372491	0.03371115	0.05375954	0	0.17979191	0	0.05714713	0.0308719	0	0.02980268	0.03128982
ENSG00000227454	0	0.12006669	0.0701226	0.0463412	0.03999652	0.05378494	0.02879076	0	0.01772426	0.12988301	0	0	0	0	0.03117919	0	0.16179721	0	0	0	0	0	0	0	0	0.05900795	0.03084664	0	0.05148869	0	0.05447006	0	0	0.05642383	0	0	0.03317559	0	0.03611106	0	0.06697281	0	0	0.04104706	0	0	0.06384867	0.03351735
ENSG00000227455	0.20758659	0.11844556	0.18045863	0.0795052	0.09864122	0.12687959	0.00617435	0.01506168	0.00760215	0.0139271	0	0	0.00650753	0.00832914	0.00668657	0.00741681	0.01156613	0.00927407	0.01692241	0.03413354	0.0258881	0.00656649	0	0.007148	0.03669733	0.03796384	0.0396915	0.01739169	0.01104206	0.0247385	0.01168144	0.00940656	0.02522607	0.06050218	0.02994735	0.00683912	0	0.0077474	0.02323271	0.02469964	0	0	0.02640838	0.01312803	0.03545997	0	0.02053911	0.007188
ENSG00000227456	0.03935255	0.02635896	0.01924304	0.008478	0.04024477	0.04427899	0.14748087	0.08994099	0.08755003	0.12474874	0.10741732	0.0996996	0.0166542	0.01421072	0.03422478	0.01265416	0	0.02373437	0.01443605	0.03494207	0.02208444	0.01680508	0.01328799	0.01219553	0.0125222	0.03778358	0.02257316	0.01978184	0.01412953	0.00527594	0.13951163	0.1444407	0.13987789	0.16516085	0.15328364	0.10501689	0.06069365	0.07270004	0.03963839	0.03160588	0.07351473	0.0234893	0	0.03359749	0.01814996	0.04504239	0.0233618	0.00613189
ENSG00000227459	0	0	0	0	0	0	0	0	0	0	0	0	0	0	0	0	0	0	0	0	0	0	0	0	0	0	0	0	0	0	0	0	0	0	0	0	0	0	0	0	0	0	0	0	0	0	0	0
ENSG00000227462	0	0	0	0	0	0	0	0	0	0	0	0	0	0	0	0	0	0	0	0	0	0	0	0	0	0	0	0	0	0	0	0	0	0	0	0	0	0	0	0	0	0	0	0	0	0	0	0
ENSG00000227463	0	0	0.03141017	0	0	0.012046	0	0	0	0	0	0	0.01359221	0	0	0	0	0	0	0	0	0	0.01626736	0	0.01532987	0.06607891	0.17962365	0.09686892	0.05765862	0.07750656	0.02439889	0.13753173	0.02634472	0.15164432	0.09382608	0.04285443	0.07430209	0.06472764	0.09705184	0.02579494	0.02999928	0.02875596	0.11031781	0.04113056	0.08887783	0.0661699	0.05719976	0.07506751
ENSG00000227465	0	0	0	0	0	0	0	0	0	0	0	0	0	0	0	0	0	0	0	0	0	0	0	0	0	0	0	0	0	0	0	0	0	0	0	0	0	0	0	0	0	0	0	0	0	0	0	0
ENSG00000227466	0	0	0	0	0	0	0	0	0	0	0	0	0	0	0	0	0	0	0	0	0	0	0	0	0	0	0	0	0	0	0	0	0	0	0	0	0	0	0	0	0	0	0	0	0	0	0	0
ENSG00000227467	0.06577498	0.06167997	0.21871094	0.1360349	0.0807195	0.0986789	0.10564446	0.1675105	0.14958548	0.16680689	0.18230253	0.17775014	0.10021067	0.41328837	0.29746222	0.22842574	0.17810901	0.09520873	0.08686378	0.1985708	0.05536883	0.11235399	0.14658548	0.1100734	0.06278989	0.09743537	0.11318828	0.08927262	0.12280579	0.13756624	0.17988459	0.03218964	0.08632464	0.08281637	0.05124053	0.01170188	0.0973873	0.13255956	0.18550765	0.08452316	0.07372477	0.16489486	0.24098812	0.10108044	0.15774909	0.21682121	0.11714276	0.19678104
ENSG00000227468	0	0	0	0	0	0	0	0	0	0	0	0	0	0	0	0	0	0	0	0	0	0	0	0	0	0	0	0	0	0	0	0	0	0	0	0	0	0	0	0	0	0	0	0	0	0	0	0
ENSG00000227469	0	0	0	0	0	0	0	0	0	0	0	0	0	0	0	0	0	0	0	0	0	0	0	0	0	0	0	0	0	0	0	0	0	0	0	0	0	0	0	0	0	0	0	0	0	0	0	0
ENSG00000227470	1.89586705	2.11646966	2.47216523	0.5445843	1.76259167	1.73816747	1.18418463	0.20633521	0.72901136	0.76316879	0	0.71158146	1.60468073	0.45641492	0.18320323	1.21926596	0.63379385	0.25409737	0.69547801	0.56112855	0.88662512	1.07947953	0.85355774	2.74184281	2.01091797	2.25367937	1.08749521	0.79418269	0.75634536	0.16945062	1.60028046	0.51545506	1.03674204	1.65768057	1.23077746	1.12429845	1.36453604	2.12268569	1.48527369	1.01510657	1.57408	0.75442094	0.48237001	0.71938151	0.19431134	1.15732452	0.56274466	0.19694181
ENSG00000227471	0.17270677	0.14682681	0.10394099	0.1202081	0.08522341	0.1345344	0.1013551	0.05855803	0.12479308	0.13837546	0.10878992	0.12341216	0.12931372	0.12953078	0.08665528	0.09611897	0.07994267	0.10416316	0.5044079	0.49543996	0.12301649	0.25529735	0.5046669	0.17909487	0.30437307	0.20226519	1.30882693	0.56597793	0.72027399	1.05798375	0.12110944	0.50387538	0.15256262	0.19863468	0.12936861	0.10045	0.09835073	0.1137903	0.10036224	0.1920585	0.13649921	0.0475789	0.14450223	0.19848969	0.15318238	0.03649436	0.17153738	0.43471699
ENSG00000227473	0	0	0.03475205	0	0	0	0	0	0	0.03218435	0	0	0	0	0	0	0	0	0	0	0	0	0	0	0	0	0	0	0	0	0	0	0	0	0	0	0	0	0	0	0	0	0	0	0	0	0	0
ENSG00000227474	0	0	0	0	0	0.02798186	0	0	0	0	0	0	0	0	0	0	0	0.04499641	0	0	0	0	0	0	0	0	0.03209621	0	0	0	0	0.0912785	0	0	0	0	0	0	0	0	0	0	0.04270984	0	0.0344093	0	0	0
ENSG00000227475	0	0	0	0	0	0	0	0	0	0	0	0	0	0	0	0	0	0	0	0	0	0	0	0	0	0	0	0	0	0	0	0	0	0	0	0	0	0	0	0	0	0	0.39466637	0	0	0	0	0
ENSG00000227477	0.07386495	0.04947591	0.02407953	0.0318264	0.04807068	0.06464259	0.24716284	0.10852696	0.13390005	0.17840309	0.24815123	0.13515427	0.2084001	0.22672559	0.21413364	0.1068837	0.0740798	0.05939939	0.12193446	0.13117291	0.21762617	0.15771616	0.07482487	0.17168311	0.30555507	0.33433705	0.19066474	0.38058885	0.26521201	0.18815621	0.14963661	0.12049599	0.08078509	0.11625292	0.14385711	0.07665671	0.10253007	0.12405306	0.17360342	0.11864882	0.09199169	0.04408954	0.08457137	0.04204178	0.02271172	0.32127028	0.03288767	0.05754793
ENSG00000227479	0	0	0	0	0	0	0	0	0	0	0	0	0	0	0	0	0	0	0	0	0	0	0	0	0	0	0	0	0	0	0	0	0	0	0	0	0	0	0	0	0	0	0	0	0	0	0	0
ENSG00000227480	0	0	0	0	0	0	0	0	0	0	0	0	0	0	0	0	0	0	0	0	0	0	0	0	0	0	0	0	0	0	0	0	0	0	0	0	0	0	0	0	0	0	0	0	0	0	0	0
ENSG00000227481	0	0	0	0	0	0	0	0	0	0	0	0	0	0	0	0	0	0	0	0	0	0	0	0	0	0	0	0	0	0	0	0	0	0	0	0	0	0	0	0	0	0	0	0	0	0	0	0
ENSG00000227482	0	0	0	0	0	0	0.02116344	0.10325196	0.0130287	0.11934263	0.13280047	0.08902041	0.84760732	0.85647771	0.43546344	0.35591001	0.27751153	1.27152568	0.37702528	0.65518526	0.31057253	0.40513582	0.24025941	0.19600605	0.62892569	0.43375425	0.74826428	0.19870802	0.26493733	0.29678106	0	0.03224228	0	0.0414759	0.05132433	0.02344204	0.12193319	0.1593316	0.07963329	0.08466138	0.04923022	0.18875945	0.54310998	0.8774631	0.75357212	0.76011523	1.6661475	1.52754704
ENSG00000227484	0	0	0.0593599	0.0784571	0.03385769	0	0	0	0	0	0	0.05125799	0	0.06575469	0.05278737	0	0	0	0	0.0538937	0	0	0	0	0	0	0	0	0.043586	0	0	0	0	0	0	0	0	0	0	0	0	0	0	0	0	0.0833666	0	0
ENSG00000227485	0	0	0	0	0	0	0	0	0	0	0	0	0	0	0	0	0	0	0	0	0	0	0	0	0	0	0	0	0	0	0	0	0	0	0	0.07985964	0	0	0	0	0	0	0	0	0	0	0	0
ENSG00000227486	0.8321482	1.41490306	1.12683599	1.4066176	0.85696612	1.44049629	0.05140594	0.21944887	0.09494005	0.14494144	0.36289345	0.05405758	0.08126983	0.03467303	0.05567049	0.06175031	0.09629638	0.0772133	0.77490201	1.02307052	0.13471067	0.82006141	0.51874612	0.35707359	0.30553173	0.23705725	0.22030687	0.48266118	0.32176599	0.54066023	0.38902548	0.27410743	0.2625315	1.25931046	1.30899966	0.76869859	0.08885261	0.03225133	0.03223817	0.10282112	0.05979003	0.05731202	0	0.05465013	0.02952297	0.04395994	0.02850047	0.05984528
ENSG00000227487	0.09484915	0.02541258	0.05565646	0.0245207	0.0423271	0.09960818	0	0	0	0	0	0	0	0	0	0.01829975	0	0	0	0	0	0	0	0.01763651	0	0	0	0	0	0.01525953	0	0	0	0	0	0	0	0	0	0	0	0.03396892	0	0	0	0	0	0.0177352
ENSG00000227488	0	0	0	0	0	0	0	0	0	0	0	0	0	0	0	0	0	0	0	0	0	0	0	0	0	0	0	0	0	0	0	0	0	0	0	0	0	0	0	0	0	0	0	0	0	0	0	0
ENSG00000227489	0	0	0	0	0	0	0	0	0	0	0	0	0	0	0	0	0	0	0	0	0	0	0	0	0	0	0	0	0	0	0	0	0	0	0	0	0	0	0	0	0	0	0	0	0	0	0	0
ENSG00000227490	0.26562972	0.41515366	0.3463748	0	0.14817391	0.13283697	0	0	0	0.16039124	0	0	0	0.2877671	0	0	0.13320118	0	0.1948867	0	0.07453497	0	0	0	0.08452485	0	0.30473767	0	0	0	0.13452907	0.32499158	0.29051563	0.69677233	0.34488819	0.07876267	0	0	0.08918637	0.28445294	0	0	0	0	0.16334965	0	0	0.08278049
ENSG00000227491	0	0	0	0	0	0	0	0	0	0	0	0	0	0	0	0	0	0	0	0	0	0	0	0	0	0	0	0	0	0	0	0	0	0	0	0	0	0	0	0	0	0	0	0	0	0	0	0
ENSG00000227492	0.15238648	0.07655316	0.11177343	0.0492443	0.08500442	0.08573166	0.18356661	0.37315942	0.15067712	0.13801989	0.09598994	0.03217257	0.16122679	0.04127156	0.0331325	0.03675092	0	0.04595378	0.12577794	0.0676538	0	0.0325375	0.15436683	0.03541894	0.14547066	0.09405699	0.13111644	0.05745151	0.08207152	0.06129065	0.05788248	0.0466103	0.1874959	0.17987598	0.22258741	0.06777686	0.03525397	0.07677799	0.03837333	0.12238874	0.07116851	0.13643783	0.04361856	0.09757568	0.03514141	0.10465169	0	0.03561714
ENSG00000227493	0	0	0	0	0	0	0	0	0	0	0	0	0	0	0	0	0	0	0	0	0	0	0	0	0	0	0	0	0	0	0	0	0	0	0	0	0	0	0	0	0	0	0	0	0	0	0	0
ENSG00000227494	0	0	0	0	0	0	0	0	0	0	0	0	0	0	0	0	0	0	0	0	0	0	0	0	0	0	0	0	0	0	0	0	0	0	0	0	0	0	0	0	0	0	0	0	0	0	0	0
ENSG00000227495	0.05528257	0	0.05406536	0.0714592	0	0	0.13318813	0.21659888	0.08199368	0.5007068	0.20893865	0.18674436	0	0.11977956	0.19231625	0.15998944	0	0	0.18251824	0.24543358	0	0.09443131	0.112002	0.10279391	0.26386831	0.22747918	0.14269877	0.04168437	0.27788881	0.22234944	0.16798827	0.0676369	0.0906927	0.17401381	0.10766664	0.049176	0.30694538	0.33424107	0.16705235	0.08880006	0.20654738	0.29698046	0	0.14159353	0.05099423	0	0.04922809	0
ENSG00000227496	0	0	0	0	0	0	1.09960001	0.84597435	0.67693912	0.85856489	1.1676894	0.44473841	0.44574465	0.66180164	0.51296904	0.0812844	0.03168969	0.33032658	0.71866061	0.95391853	0.47877757	0.25187856	0.12803366	0.21543051	0.32174687	0.26003993	0.21749904	0.07941827	0.10588835	0.10167037	1.40824681	1.62368343	2.14260022	1.4587589	1.35385521	2.09869044	0.66277465	0.95520856	0.9548188	0.50755328	0.39352	0.37721047	0.24118501	0.23379899	0.36919155	0.2314649	0.35640495	0.39388362
ENSG00000227497	0	0	0	0	0	0	0	0	0	0	0	0	0	0	0	0	0	0	0	0	0	0	0	0	0	0	0	0	0	0	0	0	0.02890861	0	0	0	0	0	0	0	0	0	0	0.01504447	0	0	0	0
ENSG00000227498	0	0	0	0	0	0	0	0	0	0	0	0	0	0	0	0	0	0	0	0	0	0	0	0	0	0	0	0	0	0	0	0	0	0	0	0	0	0	0	0	0	0	0	0	0	0	0	0
ENSG00000227499	0.12739028	0.08532803	0.49834161	0.6586672	0.07106101	0.38223446	0.20460785	0.12477979	0.06298065	0.23076052	0	0	0.43032396	0	0	0.12289044	0.19164122	0	0.14019517	0.33933861	0.10723608	0.1088013	0	0.11843646	0.36482662	0.31451469	0.65765521	0.96055298	0.27443678	0	0.19355171	0.15585894	0.41797505	0.20049417	1.24050693	0.11331862	0	0.12836795	0.51326228	0.20462623	0.475957	0.22811542	0.87512978	0.32628074	0.11750844	0.349942	0.68063188	0
ENSG00000227500	1.2924594	1.03441222	0.97663067	1.0709325	1.2422719	1.11368888	2.2373408	2.48866739	1.73016336	2.41325711	1.60878563	1.59337565	2.52824115	2.00092952	2.31726714	1.77962849	1.40257518	1.20191122	0.81209024	0.963204	1.3669513	1.07555764	0.81464796	1.54464269	1.75685141	1.61457201	1.20520531	2.02072349	2.08627509	1.76832434	3.05466203	3.05446343	3.16779132	2.87562353	3.34759996	3.49035017	2.94813867	3.15242398	2.72163938	2.41675804	2.26999644	2.1007479	2.86599805	3.1179404	2.73696879	3.33797843	2.9726944	2.85870734
ENSG00000227502	6.46934522	7.50682213	6.17816972	3.5990008	3.7457792	3.87874323	0	0.01145891	0	0.01059572	0	0	0.13862578	0.03802077	0.04069705	0.0225708	0.01759899	0	0	0.0103875	0	0	0	0.01087638	0	0.01925523	0	0.00882105	0.00840079	0	0	0	0	0	0	0	0	0	0	0	0	0	0.0133943	0	0.03237347	0.01606812	0.01041741	0
ENSG00000227505	0	0	0	0	0	0	0	0	0	0	0	0	0	0	0	0	0	0	0	0	0	0	0	0	0	0	0	0	0	0	0	0	0	0	0	0	0	0	0	0	0	0	0	0	0	0	0	0
ENSG00000227507	0	0	0	0	0.0139152	0	0.00667773	0.00814481	0	0	0.01047568	0	0.00703807	0.01801638	0.00723171	0	0.02501818	0.04012064	0.46670235	0.28056427	0.05599738	0.05681471	0.03369307	0	0	0.00684316	0.00715457	0.00626986	0.01791344	0	0	0	0.01364134	0	0	0.0073967	0	0	0.0083756	0	0.03106737	0	0.00952046	0.04969412	0.06136148	0.06852579	0.08885442	0.06219215
ENSG00000227508	0	0	0	0	0	0	0	0	0	0	0	0	0	0	0	0	0	0	0	0	0	0	0	0	0	0	0	0	0	0	0	0	0	0	0	0	0	0	0	0	0	0	0	0	0	0	0	0
ENSG00000227509	0	0	0	0	0	0	0	0	0	0	0	0	0	0	0	0	0	0	0	0	0	0	0	0	0	0	0	0	0	0	0	0	0.07272881	0	0	0	0	0	0	0	0	0	0	0.03784916	0	0	0	0
ENSG00000227510	0	0	0	0	0	0	0	0	0	0	0	0	0	0	0	0	0	0	0	0	0	0	0	0	0	0	0	0	0	0	0	0	0	0	0	0	0	0	0	0	0	0	0	0	0	0	0	0
ENSG00000227511	0	0	0	0	0	0	0	0	0	0	0	0	0	0	0	0	0	0	0	0	0	0	0	0	0	0	0	0	0	0	0	0	0	0	0	0	0	0	0	0	0	0	0	0	0	0	0	0
ENSG00000227512	0.11190882	0.1998888	0.07296321	0.0482184	0.10404187	0.05596373	0	0	0	0	0	0	0.03157358	0.04041174	0.03244224	0	0.05611716	0	0	0	0	0	0	0	0	0	0	0	0	0	0	0	0	0	0	0.03318242	0	0	0	0	0	0	0.04270984	0	0.1032279	0	0.06643513	0
ENSG00000227513	0.07426207	0	0	0	0	0	0.029819	0	0	0	0	0.03135713	0	0	0	0	0	0	0	0	0	0	0	0	0	0	0.0319483	0	0	0	0	0	0	0	0	0	0	0	0	0	0	0	0	0	0	0	0	0
ENSG00000227514	0	0.04143568	0	0	0	0	0	0	0	0	0	0	0	0	0	0.05380056	0	0	0	0	0	0	0	0	0	0.05090993	0	0	0	0	0	0	0	0	0	0	0	0	0	0	0	0	0	0	0	0	0	0
ENSG00000227515	0	0	0	0	0.08480394	0	0	0	0	0	0	0	0	0	0	0	0	0	0	0	0	0	0	0	0.07256379	0	0	0.11463203	0	0	0	0	0	0.23926899	0	0	0	0	0	0	0	0	0	0	0	0	0	0
ENSG00000227516	0	0.10713643	0.15642733	0	0	0.05999089	0.19267662	0.0783357	0.03953872	0.28973902	0.20150742	0.06753844	0.06769125	0	0.06955358	0.07714958	0.12031074	0.19293746	0.17602669	0	0.06732191	0.13660907	0.08101386	0	0.07634502	0.13163311	0.06881173	0.18090812	0.17228909	0	0	0	0.13120061	0	0.15575596	0.07114047	0.07400709	0.16117663	0.2416663	0.12846262	0	0.28641788	0	0	0.14754162	0.21969064	0.21364747	0.14953894
ENSG00000227517	0.01933398	0.01726694	0	0.0249914	0.0107849	0.0145029	0.02070217	0.01893778	0.0191171	0.0233483	0	0.02721255	0.185464	0.37003285	0.34189876	0.31085065	0.15512171	0.26431004	0.01418491	0.02861183	0.02170024	0.01100849	0.01305682	0.01198337	0.00615218	0.00530375	0	0.00485943	0.03239536	0.00518415	0.00979176	0.02365466	0.09515389	0.03042893	0.05020568	0.03439665	0.00596378	0.01298824	0.01947441	0.01035202	0.02407861	0.01154033	0.21398478	0.30261925	0.39235272	1.63757602	0.68866173	0.48804237
ENSG00000227518	0.04211662	0.03526297	0.05148662	0.0952711	0.00587339	0.06318544	0.03382279	0.03094017	0.02082209	0.04768247	0	0.02667559	0.00891198	0.02281329	0.00915717	0.02031446	0	0.01270072	0.01158751	0.11218905	0.01772671	0.04496362	0.06399592	0.01957819	0	0.0259955	0.0634165	0.03969616	0.01512197	0.01693953	0.01599758	0.02576433	0	0.01657139	0	0	0.019487	0.02121992	0.01060563	0.01691292	0.03933914	0.01885436	0.01205531	0.00898933	0.00971239	0	0.00937601	0.05906324
ENSG00000227519	0	0	0	0	0	0	0	0	0	0	0	0	0	0	0	0	0	0	0	0	0	0	0	0	0	0	0	0	0	0	0	0	0	0	0	0.06061228	0	0	0	0	0	0	0	0	0	0	0	0
ENSG00000227521	0	0	0	0	0	0	0	0	0	0	0	0	0	0	0	0	0	0	0	0	0	0	0	0	0	0	0	0	0	0	0	0	0	0	0	0	0	0	0	0	0	0	0	0	0	0	0	0
ENSG00000227523	0	0	0	0	0.05780847	0	0	0	0	0	0	0	0	0	0	0	0	0	0	0	0	0	0	0	0	0	0	0	0	0	0	0	0	0	0	0	0	0	0	0	0	0	0	0	0	0	0	0
ENSG00000227525	0.10511983	0.0985753	0.10280531	0.0543519	0.09382093	0.1419354	0.16883818	0.16474514	0.17669915	0.09520941	0.10594585	0.10652834	0.14235915	0.09110435	0.05485341	0.02028133	0.06325535	0.10144005	0.04627447	0.11200609	0.12388461	0.14364894	0.06389155	0.25410145	0.38132671	0.20762483	0.30752066	0.2694937	0.3321407	0.35514993	0.09582893	0.10288927	0.10347132	0.03308873	0.04094563	0.01870164	0.29182837	0.27540912	0.16941338	0.20262401	0.23564994	0.22588337	0.26478432	0.16154409	0.09696554	0.14438246	0.16849302	0.07862256
ENSG00000227527	0	0	0	0	0	0	0	0	0	0	0	0	0	0	0	0	0	0	0	0	0	0	0	0	0	0	0	0	0	0	0	0	0	0	0	0	0	0.04913327	0	0	0	0	0	0	0	0	0	0
ENSG00000227528	0	0	0	0	0	0	0	0	0	0	0	0	0	0	0	0	0	0	0	0	0	0	0	0	0	0	0	0	0	0	0	0	0	0	0	0	0	0	0	0	0	0	0	0	0	0	0	0
ENSG00000227531	0.12995863	0	0	0	0	0	0	0	0	0	0	0	0.03666609	0	0	0	0	0	0	0	0	0	0.0438825	0	0	0	0	0	0	0	0.06581799	0	0	0	0	0	0	0.043652	0	0	0	0	0	0.03995919	0	0.03857524	0	0
ENSG00000227532	0	0	0	0	0	0	0	0	0	0	0	0	0	0	0	0	0	0	0	0	0	0	0	0	0	0	0	0	0	0	0	0	0	0	0	0	0	0	0	0	0	0	0	0	0	0	0	0
ENSG00000227533	0.23026725	0.27762598	0.26273011	0.3174905	0.33396474	0.31667019	0.06780467	0.0601463	0.04174212	0.14599079	0.10636847	0.11667631	0.16891376	0.2411423	0.16688553	0.10366229	0.08082796	0.07406887	0.21117753	0.29305904	0.10337977	0.08522207	0.18660777	0.0999052	0.10258124	0.1705525	0.39625332	0.20256482	0.29763874	0.30871571	0.314873	0.43198055	0.35257624	0.30200658	0.25412814	0.29359211	0.14915981	0.05414138	0.07731326	0.14795075	0.02867757	0.08246707	0.07909306	0.12450834	0.10620256	0.13705159	0.08201946	0.11481628
ENSG00000227534	0	0	0.06512419	0	0	0	0.0326129	0	0.0301562	0.08389204	0	0	0.03606998	0	0	0	0	0	0	0	0	0	0	0	0	0	0.02864786	0	0	0	0	0	0	0	0	0	0	0	0.06707405	0	0	0	0	0	0	0.09146211	0	0
ENSG00000227535	0	0.16998418	0	0	0.07078124	0.0951824	0	0.24857706	0	0.22985202	0	0.21431489	0	0	0	0	0.19088673	0	0	0	0	0	0	0	0	0	0.21835534	0.09567713	0.09111877	0	0	0	0	0	0	0.11287248	0	0	0	0	0	0	0	0	0	0	0	0
ENSG00000227536	0.0617428	0.05514174	0.06038335	0.0798098	0.03444145	0.13894442	0.06611211	0.06047756	0.07122525	0.1118437	0.07778495	0.03476116	0.03483981	0.11148066	0.17899166	0.05956184	0.15480597	0.07447682	0.02264967	0.0182743	0.01732486	0	0.02084839	0	0.0196469	0.0508124	0.03541651	0.06207405	0.01477916	0.03311104	0.25015878	0.1007211	0.16881815	0.09717438	0.40082791	0.14646033	0.07618099	0.1244333	0.20730421	0.1322361	0.07689471	0.11056169	0.11782026	0.0351422	0.09492221	0.02826798	0.01832693	0.17317297
ENSG00000227537	0	0	0	0.1243603	0	0	0	0	0	0	0	0	0	0	0	0	0	0	0	0	0	0	0	0	0	0	0	0	0	0	0	0	0	0	0	0	0	0	0	0	0	0	0	0	0	0	0	0
ENSG00000227538	0	0.07621532	0	0	0	0	0	0	0	0	0	0	0	0	0	0.05488311	0	0	0	0	0	0	0	0	0	0.02341048	0	0	0	0	0	0	0	0	0	0	0	0	0.02865299	0	0	0	0	0	0	0	0	0
ENSG00000227540	0.94452194	1.05442627	1.08578259	1.1994855	0.70249926	0.95710918	0.77182919	0.99009074	0.71273377	0.99054993	0.89766635	0.62972215	0.96775861	0.55649768	0.73497932	0.94312886	0.89741298	0.69958428	0.9300454	0.8092368	1.08801894	0.87745868	1.64501861	0.87813482	0.98074899	1.20006087	1.44002258	1.76173812	1.20183084	1.34628301	0.60423957	0.48656837	0.70679689	1.30398523	1.29089256	0.70752766	1.82476002	1.38591186	1.20174623	1.83658613	1.88828903	1.42428468	0.81581293	0.67906656	0.93239474	1.22902509	1.03290407	0.9760011
ENSG00000227541	0	0.06275579	0.09162822	0.18166	0.02613145	0.03514001	0	0	0	0	0.11803414	0.03956103	0.03965054	0	0	0	0	0	0	0	0	0	0	0	0	0	0.04030687	0	0.03363978	0.07536612	0	0	0.07685152	0.07372823	0	0	0.04335009	0	0	0.07524773	0	0	0	0	0	0.04321168	0.04171508	0
ENSG00000227542	0.07091252	0	0	0	0.03955651	0	1.08201311	1.04189065	1.05175614	1.15608738	0.9827089	1.16776858	0.7802738	0.53775619	0.58588755	0.75248427	0.80008629	0.81260842	0.81942459	0.50371936	0.80586323	1.05988501	0.89792584	1.51635154	1.55696817	1.42979049	1.22029165	1.92491013	1.52766786	1.28346263	0	0	0.05816705	0	0.20716056	0.03153973	1.5092888	1.50059167	1.3214104	1.48078252	1.05977663	0.76189046	1.50203335	0.99894313	0.91576436	0.53569229	0.97876711	1.0607559
ENSG00000227543	0.03823786	0.01920925	0.01402348	0.0494269	0.01866366	0.06812254	0.01535393	0.03277251	0.00945224	0.01298734	0.02408646	0.02421889	0.04450175	0.01553422	0.02909842	0.04610898	0.02876181	0.05765519	0.02104071	0.04668443	0.03621186	0.05306947	0.15493915	0.05332539	0.09125625	0.11800714	0.20562901	0.12974517	0.137293	0.09227683	0.01452427	0.052631	0.07057164	0.06770358	0.03723544	0.04676935	0.02653851	0.00481642	0.0192578	0.05374359	0.04464523	0.0427949	0.01641761	0.05713018	0.07495237	0.02625992	0.04681889	0.09831027
ENSG00000227544	0.02221592	0.04836186	0.04888522	0	0	0.01249853	0	0	0	0	0	0	0	0	0	0	0	0	0.00611225	0.00986302	0	0.00474353	0.00562616	0	0.02120768	0.00457075	0	0	0	0	0.11813892	0.01359032	0.11844907	0.30594105	0.4975709	0.46934568	0	0.0055966	0	0	0	0	0	0	0	0	0	0
ENSG00000227545	0	0	0	0	0	0	0	0	0	0	0	0	0	0	0	0	0	0	0	0	0	0	0	0	0	0	0	0	0	0	0	0	0	0	0	0	0	0	0	0	0	0	0	0	0	0	0	0
ENSG00000227547	0	0	0	0	0	0	0	0	0	0	0	0	0	0	0	0	0	0	0	0	0	0	0	0	0	0	0	0	0	0	0	0	0	0	0	0	0	0	0	0	0	0	0	0	0	0	0	0
ENSG00000227549	0	0	0	0	0	0	0	0	0	0	0	0	0	0	0	0	0	0	0	0	0	0	0	0	0	0	0	0	0	0	0	0	0	0	0	0	0	0	0	0	0	0	0	0	0	0	0	0
ENSG00000227550	0	0	0	0	0	0	0	0	0	0	0	0	0	0	0	0	0	0	0	0	0	0	0	0	0	0	0	0	0	0	0	0	0	0	0	0	0	0	0	0	0	0	0	0	0	0	0	0
ENSG00000227551	0	0	0	0	0	0	0	0	0	0	0	0	0	0	0	0	0	0	0	0	0	0	0	0	0	0	0	0	0	0	0	0	0	0	0	0	0	0	0	0	0	0	0	0	0	0	0	0
ENSG00000227552	0	0	0	0	0	0	0	0	0	0	0	0	0	0	0	0.03221894	0	0	0	0	0	0	0	0	0	0	0.02873692	0	0	0	0	0	0	0	0	0.02970944	0	0	0	0	0	0	0	0	0	0	0	0
ENSG00000227554	0	0	0	0	0	0	0	0	0	0	0	0	0	0	0	0	0	0	0	0	0	0	0	0	0	0	0	0	0	0	0	0	0	0	0	0	0	0	0	0	0	0	0	0	0	0	0	0
ENSG00000227555	0	0	0	0	0	0	0	0	0	0	0	0	0	0	0	0	0	0	0	0	0	0	0	0	0	0	0	0	0	0	0	0	0	0	0	0	0	0	0	0	0	0	0	0	0	0	0	0
ENSG00000227556	0	0	0	0	0	0	0	0	0.14891687	0	0	0	0	0	0	0	0	0	0	0	0	0	0.30512695	0	0	0	0	0	0	0	0	0	0	0	0	0	0	0	0	0	0	0	0	0	0	0	0	0
ENSG00000227557	0	0	0	0	0	0	0	0	0	0	0	0	0	0	0	0	0	0	0	0	0	0	0	0	0	0	0	0	0	0	0	0	0	0	0	0	0	0	0	0	0	0	0	0	0	0	0	0
ENSG00000227558	0.07913994	0.14577526	0.16769445	0.1108224	0.1030072	0.19293572	0.09003666	0.09689775	0.05868915	0.21503651	0.06646817	0.05569468	0.11164139	0.10002463	0.11471289	0.15268892	0.07937013	0.08750683	0.09435276	0.08198195	0.09437741	0.1295508	0.06680701	0.10423475	0.02518277	0.07598466	0.17023406	0.07956453	0.09945315	0.04244067	0.21042362	0.29047744	0.30293997	0.34252626	0.34679365	0.31092477	0.28683617	0.11297535	0.09964346	0.0317805	0.06160074	0.05904768	0.15101824	0.11261037	0.10341801	0.04529125	0.08221807	0.08632072
ENSG00000227560	0.24792108	0.94101497	0.96984943	0.4272893	0.23049276	1.05384	0.33183196	0.48568134	0.16342672	0.29939699	0.9370095	0.41873832	0.06994762	0.08952754	0.14374407	0	0	0.19936871	0.3637885	0.44027009	0.13913194	0.28232541	0	0.23049558	0.15777972	0.06801044	0.42663274	0.18693839	0.17803206	0.33238391	0.25112093	0	0	0	0	0	0.152948	0.08327459	0.41620307	0.26548941	0	0.73991285	0.18923747	0.07055472	0.15245967	0	0.07358969	0.07726179
ENSG00000227562	0	0	0	0	0	0	0	0	0	0	0	0	0	0	0	0	0	0	0	0	0	0	0	0	0	0	0	0	0	0	0	0	0	0	0	0	0	0	0	0	0	0	0	0	0	0	0	0
ENSG00000227563	0	0	0	0	0	0	0	0	0	0	0	0	0	0	0	0	0	0	0	0	0	0	0	0	0	0	0	0	0	0	0	0	0	0	0	0	0	0	0	0	0	0	0	0	0	0	0	0
ENSG00000227564	0	0	0	0	0	0	0	0	0	0	0	0	0	0	0	0	0	0	0	0	0	0	0	0	0	0	0	0	0	0	0	0	0	0	0	0	0	0	0	0	0	0	0	0	0	0	0	0
ENSG00000227568	1.26305737	1.86706945	0.97967899	0.7318772	0.53449401	0.75142645	0	0.0853224	0.02153257	0.03944758	0.05486993	0	0	0	0	0	0	0	0	0	0	0	0	0	0	0	0	0	0	0	0	0	0	0	0	0.07748543	0	0	0	0.06996005	0	0	0	0.03718425	0	0	0	0
ENSG00000227569	0	0	0	0	0	0	0	0	0	0	0	0	0	0	0	0	0	0	0	0	0	0	0	0	0	0	0	0	0	0	0	0	0	0	0	0	0	0	0	0	0	0	0	0	0	0	0	0
ENSG00000227573	0	0	0	0	0	0	0	0	0	0	0	0	0	0	0	0	0	0	0	0	0	0	0	0	0	0	0	0	0	0	0	0	0	0	0	0	0	0	0	0	0	0	0	0	0.04897798	0	0	0
ENSG00000227574	0	0	0	0	0	0	0	0	0	0	0	0	0	0.0733524	0	0	0	0	0	0	0	0	0	0	0	0	0	0	0	0	0	0	0	0	0	0	0	0	0	0	0	0	0	0	0.06245722	0	0	0
ENSG00000227578	0	0	0	0	0	0	0	0	0	0	0	0	0	0	0	0	0	0	0	0	0	0	0	0	0	0	0.03564412	0	0.02974829	0	0	0	0	0	0	0	0	0	0	0	0	0	0	0	0	0	0.03688943	0.0387302
ENSG00000227579	0	0.01606249	0	0	0	0	0	0	0	0	0	0	0	0	0	0	0	0	0	0	0	0	0	0	0	0	0	0	0	0.01929014	0	0	0	0	0	0	0	0	0	0	0	0	0	0	0.02212026	0.03293725	0	0
ENSG00000227581	0	0.03470738	0.20270165	0.0669786	0.02890424	0.07773739	0	0.05123507	0	0	0	0.04375883	0.04385783	0.05613463	0	0.09997197	0	0	0	0.04600893	0	0	0.05248968	0	0	0.0426432	0	0.07814145	0.11162782	0.04168158	0	0.190188	0.08500618	0.08155149	0.10091584	0.04609262	0.04794993	0.05221397	0.05219267	0	0	0	0	0	0	0.07116988	0	0.04844389
ENSG00000227582	0	0	0	0	0	0	0.09790219	0.05970551	0.0678047	0.04140597	0.03839598	0.11582124	0.14187957	0.04952587	0.053012	0.05880148	0.04584892	0.05514454	0	0	0	0	0.01543668	0	0.01454707	0	0.01311164	0	0	0.01225813	0.71774281	1.64068248	1.04997705	0.67153698	0.71227972	0.43377188	0.01410159	0.0460668	0.01534933	0.02447775	0	0.0818627	0.03489485	0.07806055	0.07028283	0.04186067	0.08141838	0.02849371
ENSG00000227583	0.25956837	0.28977169	0.08461774	0.5032837	0.21718915	0.51922318	0.24319496	0.29662417	0.32082092	0.39182827	0.32701003	0.25573951	0.1464675	0.37493414	0.07524857	0.12519979	0	0.10436751	0.09521981	0.03841283	0.10925126	0.07389726	0.52588323	0.32176564	0.33038437	0.17801391	0.29778392	0.32620121	0.24852798	0.31319934	0	0.05292928	0.35485801	0.27234913	0.33701826	0.1154481	0.16013347	0.43593411	0.30502936	0.55592413	0.32326744	0.23240216	0.44578759	0.18467344	0.19952776	0.05941968	0.15409384	0.04044577
ENSG00000227584	0	0	0	0	0	0	0	0	0	0	0	0	0	0	0	0	0	0	0	0	0	0	0	0	0	0	0	0	0	0	0	0	0	0	0	0	0	0	0	0	0	0	0	0	0	0	0	0
ENSG00000227585	0	0	0	0	0	0	0	0	0	0	0	0	0	0	0	0	0	0	0	0	0	0	0	0	0	0	0	0	0	0	0	0	0	0	0	0	0	0	0	0	0	0	0	0	0	0	0	0
ENSG00000227586	0	0	0.04574761	0.0604655	0.05218704	0.10526704	0.15026353	0.09163799	0.11563212	0.12710249	0.17679425	0.19751807	0	0	0.04068228	0.09025046	0.07037043	0	0.10295901	0.20767457	0.23626179	0	0.09477093	0.08697946	0.08930927	0.19248238	0.08049674	0.24689976	0.03359096	0.03762837	0	0.05723122	0.07673998	0	0	0.04161047	0.30301018	0.09427312	0.14135198	0.30055405	0.26215628	0.08376372	0.16067332	0.15974655	0.17259585	0.06424915	0.37489085	0.17493235
ENSG00000227588	0	0	0	0	0	0	0	0	0	0	0	0	0	0	0	0	0	0	0	0	0	0	0	0	0	0	0	0	0	0	0	0	0	0	0	0	0	0	0	0	0	0	0	0	0	0	0	0
ENSG00000227589	0	0	0	0	0.02384408	0	0	0	0	0	0	0	0	0	0	0	0	0	0	0	0	0	0	0	0	0	0	0	0	0	0	0	0	0	0	0.03802336	0	0	0	0	0	0	0	0	0	0	0	0
ENSG00000227590	0	0	0	0	0	0	0	0	0	0	0	0	0	0	0	0	0	0	0	0	0	0	0	0	0	0	0	0	0	0	0	0	0	0	0	0	0	0	0	0	0	0	0	0	0	0	0	0
ENSG00000227591	0	0.02600963	0	0	0.02166077	0	0.06236842	0	0.03839543	0.07034026	0.24460087	0.06558552	0.06573391	0.08413432	0	0.18729688	0.17524782	0.09367927	0.04273419	0.03447898	0.06537525	0.06632946	0.07867129	0	0	0	0.10023299	0	0.08365362	0.09370824	0.05899829	0	0.06370343	0	0	0	0.03593357	0.11738708	0	0.12474804	0.14508087	0.34766989	0.13337821	0.29836998	0.25073187	0.26667267	0.10373486	0.18151866
ENSG00000227592	0	0	0	0	0	0	0	0	0	0	0	0	0	0	0	0	0	0	0	0	0	0	0	0	0	0	0	0	0	0	0	0	0	0	0	0	0	0	0	0	0	0	0	0	0	0	0	0
ENSG00000227593	0	0	0	0	0	0.02544877	0	0	0	0.03072759	0	0.02865052	0.05743068	0.03675341	0	0.03272767	0	0.04092305	0	0.03012374	0	0	0.03436693	0.063083	0	0	0	0	0.02436228	0	0	0	0	0.10678953	0	0	0	0	0.03417246	0	0	0	0.03884348	0	0	0.04659754	0	0.031718
ENSG00000227597	0	0	0	0	0	0	0	0	0	0	0	0	0	0	0	0	0	0	0	0	0	0	0	0	0	0	0	0	0	0	0	0	0	0	0	0	0	0	0	0	0	0	0	0	0	0	0	0
ENSG00000227598	0.50228166	0.44858162	0.49122244	0.3246288	0.18678894	0.43956963	0.60505463	0.40999074	0.16554915	0.07582131	0.31639282	0.28278432	0.2125681	0.27207071	0.36402719	0.64605261	0.75561396	0.10097896	0.36851302	0.14866263	0.5637554	0.50048596	0.84801516	1.01178578	0.1598288	0.62004325	0.64826013	0.31561026	0.5410325	0.87542152	0.50876449	0	0.41200398	0.52701325	0.16303805	0.44679912	0.2324015	0.16871216	0	0.26893732	0.31277174	0.29980884	0.28754264	0.07147102	0.07721983	0.8048666	0.37272698	0
ENSG00000227599	0	0	0	0	0	0	0	0	0	0	0	0	0	0	0	0	0	0	0	0	0	0	0	0	0	0	0	0	0	0	0	0	0	0	0	0	0	0	0	0	0	0	0	0	0	0	0	0
ENSG00000227602	0	0	0	0	0	0.0732616	0	0	0	0	0	0	0	0	0.08493968	0	0	0	0.10748297	0.08671987	0	0	0	0	0	0	0.08403372	0	0	0	0	0	0	0	0	0	0	0	0	0	0	0	0	0	0.18017961	0	0	0.09130939
ENSG00000227603	3.03174108	4.34259145	3.01060353	4.401203	2.28958362	3.81363228	0.33711438	0.50255015	0.23059487	0.8871423	0.17628254	0.19694639	0.11843519	0.35370505	0.44620989	0.13498386	0.35083379	0.73140329	0.82128807	1.32527032	0.35336694	0.6373772	0.70872468	0.303547	0.71240625	0.23031033	0.76250569	0.42203167	0.36843104	0.67535023	0.56693004	0.85598362	0.22955359	1.2479384	0.635872	0.33192024	0.56110444	0.32900092	0.28188572	0.67428931	0.0871325	0.66817021	0.48062482	0.47785255	0.64536112	0.19218956	0.41533976	0.39245858
ENSG00000227604	0	0	0	0	0	0	0	0	0	0	0	0	0	0	0	0	0	0	0	0	0	0	0	0	0	0	0	0	0	0	0	0	0	0	0	0	0	0	0	0	0	0	0	0	0	0	0	0
ENSG00000227606	0	0	0	0	0	0	0	0	0	0	0	0	0.05455914	0.4189889	0	0	0.09697046	0.0777538	0	0	0	0	0	0	0	0	0	0	0	0	0	0	0	0.10145005	0	0.05733922	0	0	0.06492768	0	0	0	0.07380261	0.16509806	0.17837781	0.44267663	0.28699977	0.12052839
ENSG00000227607	0	0	0	0	0	0	0	0	0	0	0	0	0	0	0	0	0	0	0	0	0	0	0	0	0	0	0	0	0	0	0	0	0	0	0	0	0	0	0	0	0	0	0	0	0	0	0	0
ENSG00000227609	0	0	0	0	0	0	0	0	0	0	0	0	0	0	0	0	0	0	0	0	0	0	0	0	0	0	0	0	0	0	0	0	0	0	0	0	0	0	0	0	0	0	0	0	0	0	0	0
ENSG00000227610	0	0	0	0	0	0	0	0	0	0	0	0	0	0	0	0	0	0	0	0.06262047	0	0	0	0	0	0	0	0	0	0.1701922	0	0	0	0	0	0	0	0	0	0	0	0.12628709	0	0	0	0	0	0
ENSG00000227611	0	0	0	0	0	0	0	0	0	0	0	0	0	0	0	0	0	0	0	0	0	0	0	0	0	0	0	0	0	0	0	0	0	0	0	0	0	0	0	0	0	0	0	0	0	0	0	0
ENSG00000227613	0	0	0.03269721	0	0	0	0	0	0	0	0	0	0	0	0	0	0	0	0	0	0	0	0	0	0	0	0	0	0	0	0	0	0	0	0	0	0	0	0	0	0.06245701	0	0	0.02854392	0	0	0	0
ENSG00000227615	0.16155258	0	0.07899776	0.2088256	0	0.24236922	0.12973881	0.47472612	0.19967551	0.14632184	0.40705509	0.13643103	0.27347942	0.08750812	0.14050172	0.07792301	0.12151687	0	0	0.0717232	0.13599363	0	0.08182602	0.07509881	0.61688311	0.19942912	0.34750787	0.36544346	0	0.25990922	0.24545655	0.29648355	0.3975477	0.12713039	0	0.14370732	0.29899608	0.16279244	0	0.64875232	0.45269594	0.28928923	0.18496895	0.34481632	0.52157256	0.22189305	0.07192977	0.22655712
ENSG00000227616	0	0	0	0	0	0.07348428	0	0	0	0	0	0	0	0	0	0	0	0	0	0	0	0	0	0	0	0	0	0	0	0	0	0	0	0	0	0	0	0	0	0	0	0	0	0	0	0	0	0
ENSG00000227617	0.01253263	0.00559637	0.00817112	0	0.01398193	0.00626736	0.00335488	0.00818387	0.00413068	0	0.01578888	0	0.00353591	0.00905139	0.01453278	0.00402998	0.01256908	0.00503913	0.01379237	0.00370934	0.01054986	0.04638334	0.04655015	0.01941959	0.01196385	0.01031396	0.01078333	0.03149966	0.01799935	0.02016276	0.00634719	0.00511112	0.00685338	0.00657486	0.01627211	0.02229652	0.00773166	0.0042096	0.01262366	0	0	0.00748065	0	0.0035666	0	0.00573787	0.00372002	0.0078113
ENSG00000227619	0.0394811	0	0.03861181	0	0	0.00987192	0.08455008	0.06445342	0.03253186	0.07151786	0.04973917	0.02222784	0.07797346	0.14257142	0.06867316	0.16504151	0.1979797	0.14287141	0.01448321	0.02337081	0.04431315	0.02247997	0.05332558	0.02447074	0.01256311	0.01083057	0.03397035	0.04961615	0.03780183	0.04234536	0	0.04830418	0.02158997	0	0.02563073	0.01170666	0.08524868	0.07956821	0.02651191	0.04227884	0.19667966	0.11783014	0.30135815	0.3146009	0.315627	0.16268231	0.12890966	0.27068442
ENSG00000227620	0	0.02840525	0	0	0	0	0	0	0	0	0	0	0	0	0	0	0	0	0	0	0	0	0	0.03942688	0.08096591	0	0	0	0	0	0	0	0	0	0	0	0	0	0	0	0	0	0	0	0	0	0	0
ENSG00000227621	0.11951696	0.02668478	0	0	0	0.08965264	0	0.0390226	0.01969605	0.03608307	0.05019004	0.03364399	0.06744023	0.12947741	0	0.03843175	0	0.0480555	0	0	0.06707226	0.03402562	0	0	0	0.13114498	0.10283484	0.15019772	0.0858251	0.0320469	0.06052977	0.09748408	0	0	0.07758918	0.03543833	0.07373266	0	0	0.06399312	0.07442344	0.14267788	0.04561348	0.03401278	0.07349725	0.10943798	0	0.0372461
ENSG00000227623	0	0	0	0	0	0	0	0	0	0	0	0	0	0	0	0	0	0	0	0	0	0	0	0	0	0	0	0	0	0	0	0	0	0	0	0	0	0	0	0	0	0	0	0	0	0	0	0
ENSG00000227624	0	0	0	0	0	0	0	0.11438147	0	0	0	0	0	0	0	0	0	0	0	0	0	0	0	0	0	0	0	0	0	0.09393458	0	0	0	0	0	0.1038754	0	0.11767062	0.11762261	0.18757404	0	0	0	0	0.21543214	0.16039008	0.10398543	0
ENSG00000227625	0	0	0	0	0	0	0	0	0	0	0	0	0	0	0	0	0	0	0	0	0	0	0	0	0	0	0	0	0	0	0	0	0	0	0	0	0	0.15613986	0	0	0	0	0	0	0	0	0	0
ENSG00000227627	2.28476285	3.32122932	2.37708195	1.7594263	1.32872295	2.07850796	1.36636688	1.28562707	1.51410052	1.67310081	0.97987922	1.84737493	2.05728299	1.10592846	1.52199604	1.50063502	2.12077172	0.70365426	1.81894249	2.02869552	1.18671363	1.8268115	1.92050492	1.31066113	1.34576818	2.76042384	2.30047064	2.45585724	2.8624763	2.50265534	1.55104106	1.0705605	1.51523837	1.22413334	1.42012784	1.2540252	0.58480116	0.73477582	0.68551093	1.79595777	1.90705846	2.17621426	0.83487117	0.87155837	0.94166267	1.53568062	0.60603271	1.40889143
ENSG00000227628	0	0	0	0	0	0	0	0	0	0	0	0	0	0	0	0	0	0	0	0	0	0	0	0	0	0	0	0	0	0	0	0	0	0	0	0	0	0	0	0	0	0	0	0	0	0	0	0
ENSG00000227629	0	0	0	0	0	0	0	0	0	0	0	0	0	0	0	0	0	0	0	0	0	0	0	0	0	0	0	0	0	0	0	0	0	0	0	0	0	0	0	0	0	0	0	0	0	0	0	0
ENSG00000227630	0.12745745	0.05122381	0.09972088	0.0549179	0.18959594	0.06373933	0.00682386	0.04161519	0.08821941	0	0.18198357	0.08611017	0.00719208	0.01841062	0	0.01639403	0	0	0.03740509	0.0226345	0.04291705	0.02177173	0.00860759	0.03949964	0.01622307	0.0419574	0	0	0.00610181	0	0	0.03118822	0.01393985	0	0.03309763	0	0.00786313	0	0.00855888	0.01364894	0.07936799	0.03043143	0.00972879	0	0.02351408	0.01167088	0.01513313	0.01588827
ENSG00000227632	0.73012355	0.83836857	0.71404772	1.0785942	0.64000901	0.54768384	0.33505363	0.35758092	0.36096679	0.33064473	0.65701853	0.13212617	0.0441417	0.05649796	0.27213683	0.10061904	0.23536519	0.25163041	0.11478763	0.04630672	0.17560342	0.17816652	0.15848827	0.1939445	0.04978486	0.04291921	0.13461713	0.19661804	0.18725055	0.20975684	0.6338975	0.44664431	0.51333829	0.65663463	0.30470704	0.60308242	0.33782203	0.10510385	0.15759145	0.25131279	0.09742485	0	0	0.22262413	0.19242483	0.14326105	0.13932028	0.09751488
ENSG00000227633	0	0	0	0	0	0	0	0	0	0	0	0	0	0	0	0	0	0	0	0	0	0	0	0	0	0	0	0	0	0	0	0	0	0	0	0	0	0	0	0	0	0	0	0	0	0	0	0
ENSG00000227634	0	0	0	0	0	0	0	0	0	0.05873482	0	0	0	0	0	0.05639858	0	0	0	0	0	0	0	0	0	0.05336836	0	0	0.04656774	0.05216488	0	0	0	0	0	0	0	0	0	0	0	0	0	0.05536487	0	0	0	0
ENSG00000227635	0	0	0	0	0	0	0	0	0	0	0	0	0	0	0	0	0	0	0	0	0	0	0	0	0	0	0	0	0	0	0	0	0	0	0	0	0	0	0	0	0	0	0	0	0	0	0	0
ENSG00000227637	0	0	0	0	0	0	0	0	0	0	0	0	0	0	0	0	0	0	0	0	0	0	0	0	0	0	0	0	0	0	0	0	0	0	0	0	0	0	0	0	0	0	0	0	0	0	0	0
ENSG00000227638	0.03447031	0.06926628	0.10113403	0.0445569	0.05768482	0	0.02768224	0.06752789	0.08520912	0.06244108	0	0	0	0	0.02997871	0	0	0	0	0	0	0	0.03491827	0	0	0.05673598	0.02965896	0.05198287	0.02475312	0	0	0	0	0	0	0.06132537	0.09569474	0.03473486	0.03472068	0.1107389	0	0.2469014	0.03946664	0.02942924	0	0.04734509	0	0

ENSG00000227640	8.19715925	9.75958442	7.93037061	7.9468392	6.91899005	8.12742439	0.92916986	1.03726285	1.14403678	1.02129258	[illegible]
ENSG00000227644	0	0	0	0	0	0	0	0	0	0	[illegible]
ENSG00000227645	0	0	0	0	0	0	0	0	0	0	[illegible]
ENSG00000227646	0	0	0	0	0.0110162	0	0.01585962	0.03868785	0.01952709	0.05366031	[illegible]
ENSG00000227649	0	0	0	0	0	0	0	0	0	0	[illegible]
ENSG00000227653	0	0	0	0	0.04609855	0	0	0	0	0	[illegible]
ENSG00000227657	0	0	0	0	0	0	0	0	0	0	[illegible]
ENSG00000227658	0.08459249	0.05666139	0.16545988	0.1093457	0	0.06345493	0	0.16571804	0	0.30646936	[illegible]
ENSG00000227659	0	0	0	0	0	0	0	0	0	0	[illegible]
ENSG00000227660	0	0	0	0	0	0	0	0	0	0	[illegible]
ENSG00000227661	0	0	0	0	0	0	0	0	0	0	[illegible]
ENSG00000227663	0	0	0	0	0	0	0	0	0	0	[illegible]
ENSG00000227666	0.71394993	0.54653141	0.29924152	0.5273507	0.34136269	0.61205897	0.49144733	0.69931965	0.40339508	0.55426341	[illegible]
ENSG00000227667	0	0	0	0	0	0	0	0	0	0	[illegible]
ENSG00000227671	7.04536296	6.90635923	6.32152034	4.7992204	6.03439954	5.40793674	4.83171278	5.0242122	4.93909353	5.03063814	[illegible]
ENSG00000227673	0	0	0	0	0	0	0	0	0	0	[illegible]
ENSG00000227674	0.2402643	0.65522655	0.38602899	0.3549368	0.23932954	0.43769712	0.50993984	0.25215085	0.26302304	0.24870037	[illegible]
ENSG00000227675	0.03894833	0.06522052	0	0.0125863	0.02172621	0.02921611	0	0	0.00962786	0.0088191	[illegible]
ENSG00000227676	1.48182712	1.36475802	1.17747525	0	0.61994603	0.34736105	0	0	0	0	[illegible]
ENSG00000227678	0	0	0	0	0	0	0.01194411	0.0291364	0	0	[illegible]
[illegible]	[illegible]	[illegible]	[illegible]	[illegible]	[illegible]	[illegible]	[illegible]	[illegible]	[illegible]	[illegible]	[illegible]

ENSG00000227919	0	0	0	[illegible]
ENSG00000227920	0	0	0	[illegible]
ENSG00000227921	0.00848598	0.01136808	0.01659827	[illegible]
ENSG00000227922	0	0	0	[illegible]
ENSG00000227923	0	0	0	[illegible]
ENSG00000227924	0	0	0	[illegible]
ENSG00000227925	0	0	0	[illegible]
ENSG00000227927	0	0.01740967	0	[illegible]
ENSG00000227929	0	0	0	[illegible]
ENSG00000227930	0	0	0	[illegible]
ENSG00000227932	0	0	0	[illegible]
ENSG00000227933	0.05614937	0	0.05491308	[illegible]
ENSG00000227934	0	0.03767538	0.05500891	[illegible]
ENSG00000227935	0	0	0	[illegible]
ENSG00000227937	0	0	0	[illegible]
ENSG00000227938	0	0	0	[illegible]
ENSG00000227939	0.21432911	0.34095746	0.26201252	[illegible]
ENSG00000227940	0	0	0	[illegible]
ENSG00000227941	0	0	0	[illegible]
ENSG00000227942	0.02114812	0	0	[illegible]
ENSG00000227945	0	0	0.04191504	[illegible]
ENSG00000227946	5.8765733	5.72064256	5.23632241	[illegible]
ENSG00000227947	0	0	0	[illegible]
ENSG00000227948	0	0	0	[illegible]
ENSG00000227949	0	0	0	[illegible]
ENSG00000227950	0	0	0	[illegible]
ENSG00000227952	0	0	0	[illegible]
ENSG00000227953	0.05217471	0.12390458	0.07421953	[illegible]
ENSG00000227954	0	0	0	[illegible]
ENSG00000227958	0	0	0	[illegible]
ENSG00000227959	0.03565237	0.04776104	0	[illegible]
ENSG00000227960	0	0	0	[illegible]
ENSG00000227962	0	0.15310632	0.44709371	[illegible]
ENSG00000227963	0.16046979	0.07441284	0.08450431	[illegible]
ENSG00000227964	0.04156888	0.0278435	0.08130724	[illegible]
ENSG00000227965	0	0	0	[illegible]
ENSG00000227968	0	0	0	[illegible]
ENSG00000227969	0.06770954	0	0	[illegible]
ENSG00000227970	0	0	0	[illegible]
ENSG00000227971	0	0	0	[illegible]
ENSG00000227972	0	0	0	[illegible]
ENSG00000227973	0.08138823	0.05451513	0.15919246	[illegible]
ENSG00000227979	0	0	0.84053618	[illegible]
ENSG00000227981	0	0	0	[illegible]
ENSG00000227982	0	0	0	[illegible]
ENSG00000227983	0	0	0	[illegible]
ENSG00000227986	2.9972101	3.25905655	2.47440451	[illegible]
ENSG00000227987	0	0	0	[illegible]
ENSG00000227988	0	0	0	[illegible]
ENSG00000227989	0	0	0	[illegible]
ENSG00000227992	1.39321642	0.37327938	1.81672084	[illegible]
ENSG00000227994	0	0	0	[illegible]
ENSG00000227995	0	0	0	[illegible]
ENSG00000227997	0	0	0	[illegible]
ENSG00000227999	0	0	0	[illegible]
ENSG00000228000	0	0	0	[illegible]
ENSG00000228002	0.04191124	0.02807281	0.02049422	[illegible]
ENSG00000228004	0	0	0	[illegible]
ENSG00000228005	0	0.08443803	0.04109532	[illegible]
ENSG00000228007	0.10040417	0.13450461	0.19638696	[illegible]
ENSG00000228008	0	0	0	[illegible]
ENSG00000228010	0.90180537	1.49697745	0.82443101	[illegible]
ENSG00000228012	0	0	0	[illegible]
ENSG00000228013	0.01835407	0	0	[illegible]
ENSG00000228014	0.83371689	0.5878282	0.40768007	[illegible]
ENSG00000228015	0	0	0	[illegible]
ENSG00000228016	0	0.00737295	0.00538253	[illegible]
ENSG00000228019	0	0.11422217	0	[illegible]
ENSG00000228020	0	0	0	[illegible]
ENSG00000228021	0	0	0	[illegible]
ENSG00000228022	0.02710659	0.00907821	0.02650976	[illegible]
ENSG00000228024	0	0	0	[illegible]
ENSG00000228027	0	0	0	[illegible]
ENSG00000228028	0	0	0	[illegible]
ENSG00000228030	0	0	0	[illegible]
ENSG00000228031	11.4231989	9.17392004	10.373706	[illegible]
ENSG00000228032	0.0595377	0.09305168	0.1164536	[illegible]
ENSG00000228033	0	0	0	[illegible]
ENSG00000228034	0	0	0	[illegible]
ENSG00000228035	0	0	0	[illegible]
ENSG00000228036	0	0	0	[illegible]
ENSG00000228037	0.05148521	0	0	[illegible]
ENSG00000228038	0	0	0	[illegible]
ENSG00000228039	0	0	0	[illegible]
ENSG00000228040	0.2066009	0	0	[illegible]
ENSG00000228041	0.12589742	0	0.12312542	[illegible]
ENSG00000228042	0	0	0	[illegible]
ENSG00000228043	0.10773172	0.15634756	0.19315943	[illegible]
ENSG00000228044	0	0.00890226	0.02599596	[illegible]
ENSG00000228045	0	0	0	[illegible]
ENSG00000228046	0	0	0	[illegible]
ENSG00000228048	0	0	0	[illegible]
ENSG00000228049	0	0	0	[illegible]
ENSG00000228050	0	0	0	[illegible]
ENSG00000228051	0	0	0.01651132	[illegible]
ENSG00000228053	0.06742623	0	0	[illegible]
ENSG00000228054	0	0	0	[illegible]
ENSG00000228055	0	0	0	[illegible]
ENSG00000228056	0	0	0.03229519	[illegible]
ENSG00000228057	0	0	0	[illegible]
ENSG00000228058	0	0	0	[illegible]
ENSG00000228060	0	0	0	[illegible]
ENSG00000228061	0	0.02650459	0	[illegible]
ENSG00000228063	0.22258108	0.447265	0.30475241	[illegible]
ENSG00000228064	0	0	0	[illegible]
ENSG00000228065	0.28568988	0.43337324	0.40266409	[illegible]
ENSG00000228067	0	0	0	[illegible]
ENSG00000228069	0	0	0	[illegible]
ENSG00000228071	0.34780295	0.11648196	0.22676336	[illegible]
ENSG00000228072	0	0	0	[illegible]
ENSG00000228073	0	0	0	[illegible]
ENSG00000228074	0	0	0	[illegible]
ENSG00000228075	0	0	0	[illegible]
ENSG00000228076	0	0	0	[illegible]
ENSG00000228078	0	0	0	[illegible]
ENSG00000228079	0.22898572	0.2300674	0.11197196	[illegible]
ENSG00000228081	0	0	0	[illegible]
ENSG00000228082	0	0	0	[illegible]
ENSG00000228083	0	0	0	[illegible]
ENSG00000228084	0.19682284	0.59325776	0.5293453	[illegible]
ENSG00000228085	0	0	0	[illegible]
ENSG00000228086	0	0	0	[illegible]
ENSG00000228089	0.14990577	0.25102315	0.07330257	[illegible]
ENSG00000228092	0	0	0	[illegible]
ENSG00000228095	0	0.03426665	0	[illegible]
ENSG00000228097	0.09620818	0.22554617	0.04704494	[illegible]
ENSG00000228098	0	0	0	[illegible]
ENSG00000228100	0	0	0	[illegible]
ENSG00000228105	0	0	0	[illegible]
ENSG00000228106	1.29610214	1.22312654	1.23376289	[illegible]
ENSG00000228107	0.91930466	1.23152966	1.00483561	[illegible]
ENSG00000228108	0	0	0	[illegible]
ENSG00000228109	3.35697071	4.84666922	3.40720627	[illegible]
ENSG00000228110	0	0.01451781	0	[illegible]
ENSG00000228111	0	0	0	[illegible]
ENSG00000228112	0	0	0	[illegible]
ENSG00000228113	0.14336465	0.24692893	0.24703324	[illegible]
ENSG00000228115	0	0	0	[illegible]
ENSG00000228118	0.03522376	0.04718686	0.0344482	[illegible]
ENSG00000228120	0	0	0	[illegible]
ENSG00000228121	0.11805765	0.02635896	0.03848609	[illegible]
ENSG00000228122	0.31597784	0.42329393	0.15451033	[illegible]
ENSG00000228123	0	0	0	[illegible]
ENSG00000228124	0.54626678	0.50310995	0.40067932	[illegible]
ENSG00000228125	0	0	0.05529843	[illegible]
ENSG00000228126	0.28471502	0.11442398	0.1670677	[illegible]
ENSG00000228127	0	0	0	[illegible]
ENSG00000228131	0	0	0	[illegible]
ENSG00000228132	0	0.06444176	0	[illegible]
ENSG00000228133	0	0	0	[illegible]
ENSG00000228134	0	0	0	[illegible]
ENSG00000228135	0	0	0	[illegible]
ENSG00000228136	0	0	0	[illegible]
ENSG00000228137	0.16528072	0.71959969	0.32328314	[illegible]
ENSG00000228139	0	0	0	[illegible]
ENSG00000228140	0.05601925	0.05003011	0.05478582	[illegible]
ENSG00000228141	0.00622436	0.01250753	0	[illegible]
ENSG00000228142	0	0	0	[illegible]
ENSG00000228143	0	0	0	[illegible]
ENSG00000228144	0	0	0	[illegible]
ENSG00000228146	0	0.01802003	0.03288826	[illegible]
ENSG00000228149	0	0.01782658	0	[illegible]
ENSG00000228150	0.43949645	0.88314507	0.50145624	[illegible]
ENSG00000228151	0.03099013	0.01383846	0.04041039	[illegible]
ENSG00000228153	0	0	0	[illegible]
ENSG00000228154	0	0	0	[illegible]
ENSG00000228155	0	0.2439321	0.1780797	[illegible]
ENSG00000228156	0	0	0	[illegible]
ENSG00000228157	0.00594645	0	0	[illegible]
ENSG00000228158	0.25179484	0.40055842	0.30781354	[illegible]
ENSG00000228159	0	0	0	[illegible]
ENSG00000228160	0	0	0	[illegible]
ENSG00000228161	0	0	0	[illegible]
ENSG00000228162	0	0	0	[illegible]
ENSG00000228165	0	0	0	[illegible]
ENSG00000228166	0	0.04928765	0	[illegible]
ENSG00000228167	0	0	0	[illegible]
ENSG00000228168	0.13415084	0.02246409	0.03279928	[illegible]
ENSG00000228169	0.0655076	0.04387803	0.06406526	[illegible]
ENSG00000228170	0.13912118	0.18637114	0.3174687	[illegible]
ENSG00000228171	0	0	0	[illegible]

ENSG00000228172	0.16927384	0.22676461 0.19865613 0.1312837 0.09442455 0.15237182 0.19031539 0.23212711 0.11716254 0.45994548 0.38385894 0.14295163 0.17193008 0.1100286 0.14721688 0.06531782 0.10185973 [illegible]
ENSG00000228173	0	0 0 0 0 0 0 0 0 0 0 0 0 0 0 0 0 [illegible]
ENSG00000228174	0	0 0 0 0 0 0 0 0 0 0 0 0 0 0 0 0 [illegible]
ENSG00000228175	0.82640358	0.52190746 0.32328314 0.3662479 0.75074784 0.40736672 0.51196931 0.39317061 0.46693349 0.44909548 0.59492667 0.55831776 0.15988028 0.30695157 0.1232092 0.18221997 0.03552031 [illegible]
ENSG00000228176	0	0 0 0 0 0 0.05197368 0.06339214 0 0.05861688 0 0 0.05477826 0.21033579 0.22514132 0 0 [illegible]
ENSG00000228181	0	0 0 0 0 0 0 0 0 0 0 0 0 0 0 0 0 [illegible]
ENSG00000228182	0	0 0 0 0 0 0 0 0 0 0 0 0 0 0 0 0 [illegible]
ENSG00000228184	0	0 0 0 0 0 0 0 0 0 0 0 0 0 0 0 0 [illegible]
ENSG00000228187	0	0 0 0 0 0 0 0.05528772 0 0 0 0 0 0 0 0 0 [illegible]
ENSG00000228189	0	0 0 0 0 0 0 0 0 0 0 0 0.01434257 0 0.0442115 0.01634663 0 [illegible]
ENSG00000228190	0	0 0 0.0989565 0 0.05742596 0 0 0 0 0 0 0.06479708 0 0 0 0 [illegible]
ENSG00000228191	0	0 0 0 0.11906248 0 0 0 0 0 0 0 0 0 0 0 0 [illegible]
ENSG00000228192	0.3523146	0.14159154 0.12404065 0.1639468 0.22011202 0.19028156 1.13173995 1.11810767 0.91271002 1.11046565 0.72792022 1.11871059 0.22663396 0.29007393 0.26963798 0.33986972 0.21200363 [illegible]
ENSG00000228193	0	0 0 0 0 0 0 0 0 0 0 0 0 0 0 0 0 [illegible]
ENSG00000228194	0	0 0 0 0 0 0 0 0 0 0 0 0 0 0 0 0 [illegible]
ENSG00000228195	0	0 0 0 0 0 0 0 0 0.03313417 0 0 0 0 0 0 0 [illegible]
ENSG00000228196	0.03006506	0 0 0 0 0 0 0 0 0 0 0 0 0 0 0 0 [illegible]
ENSG00000228198	0.03037676	0 0 0 0.0169448 0 0 0 0 0 0 0 0 0 0 0 0 [illegible]
ENSG00000228201	0.0828528	0.38847284 0.48617131 0.5354846 0.09243411 0.12429989 1.26420298 1.78540954 1.18788961 0.60033329 1.3569375 0.62972215 0.56101949 0.62830383 1.22496554 0.71933557 0.49856277 [illegible]
ENSG00000228203	0.03983352	0.05929138 0.02597097 0.0457684 0.06912884 0.04648017 0.77840614 0.80202116 1.08313406 0.27659891 0.5854701 0.70268913 0.01873082 0.02876881 0.02309538 0.03415686 0.04660761 [illegible]
ENSG00000228204	0	[illegible]
ENSG00000228205	0.44029699	[illegible]
ENSG00000228206	0	[illegible]
ENSG00000228207	0	[illegible]
ENSG00000228208	0.14324329	[illegible]
ENSG00000228209	0	[illegible]
ENSG00000228210	0	[illegible]
ENSG00000228211	0	[illegible]
ENSG00000228212	0	[illegible]
ENSG00000228213	0	[illegible]
ENSG00000228214	3.00429407	[illegible]
ENSG00000228215	0.01571416	[illegible]
ENSG00000228216	0	[illegible]
ENSG00000228217	0	[illegible]
ENSG00000228218	0	[illegible]
ENSG00000228219	0	[illegible]
ENSG00000228221	0.01527476	[illegible]
ENSG00000228222	0.69761342	[illegible]
ENSG00000228223	0.05448815	[illegible]
ENSG00000228224	0.14676566	[illegible]
ENSG00000228225	0	[illegible]
ENSG00000228226	0	[illegible]
ENSG00000228229	0	[illegible]
ENSG00000228231	0	[illegible]
ENSG00000228232	0.48104089	[illegible]
ENSG00000228234	0	[illegible]
ENSG00000228235	0	[illegible]
ENSG00000228236	0	[illegible]
ENSG00000228237	0	[illegible]
ENSG00000228238	0	[illegible]
ENSG00000228239	0	[illegible]
ENSG00000228240	0	[illegible]
ENSG00000228241	0	[illegible]
ENSG00000228242	0.02117591	[illegible]
ENSG00000228243	0	[illegible]
ENSG00000228247	0.69761342	[illegible]
ENSG00000228248	0	[illegible]
ENSG00000228251	0.3655547	[illegible]
ENSG00000228252	0.01198132	[illegible]
ENSG00000228253	113.349036	[illegible]
ENSG00000228255	0	[illegible]
ENSG00000228257	0	[illegible]
ENSG00000228259	0	[illegible]
ENSG00000228261	0	[illegible]
ENSG00000228262	0.00605708	[illegible]
ENSG00000228264	0	[illegible]
ENSG00000228265	0.24819382	[illegible]
ENSG00000228271	0	[illegible]
ENSG00000228272	0	[illegible]
ENSG00000228273	0	[illegible]
ENSG00000228274	0.68905527	[illegible]
ENSG00000228275	0.46042485	[illegible]
ENSG00000228277	0	[illegible]
ENSG00000228278	0	[illegible]
ENSG00000228280	0	[illegible]
ENSG00000228283	0.01353622	[illegible]
ENSG00000228285	0	[illegible]
ENSG00000228286	0	[illegible]
ENSG00000228288	0.46685603	[illegible]
ENSG00000228289	0.09130238	[illegible]
ENSG00000228290	0	[illegible]
ENSG00000228291	0	[illegible]
ENSG00000228292	0	[illegible]
ENSG00000228293	0.18480355	[illegible]
ENSG00000228294	0	[illegible]
ENSG00000228295	0	[illegible]
ENSG00000228296	0	[illegible]
ENSG00000228300	2.1396806	[illegible]
ENSG00000228301	0	[illegible]
ENSG00000228302	0.02309548	[illegible]
ENSG00000228303	0.02025754	[illegible]
ENSG00000228304	0	[illegible]
ENSG00000228305	0.02458409	[illegible]
ENSG00000228307	0	[illegible]
ENSG00000228308	0.04028717	[illegible]
ENSG00000228309	0	[illegible]
ENSG00000228312	0	[illegible]
ENSG00000228313	0.02411503	[illegible]
ENSG00000228314	0	[illegible]
ENSG00000228315	0.00633446	[illegible]
ENSG00000228316	0.2445352	[illegible]
ENSG00000228317	0	[illegible]
ENSG00000228318	0	[illegible]
ENSG00000228319	0	[illegible]
ENSG00000228322	0	[illegible]
ENSG00000228323	0.22024879	[illegible]
ENSG00000228325	0	[illegible]
ENSG00000228326	0	[illegible]
ENSG00000228327	0.51391385	[illegible]
ENSG00000228328	0.07747534	[illegible]
ENSG00000228329	0	[illegible]
ENSG00000228330	0.0406428	[illegible]
ENSG00000228331	0	[illegible]
ENSG00000228334	0	[illegible]
ENSG00000228335	0.15929031	[illegible]
ENSG00000228336	0	[illegible]
ENSG00000228337	0	[illegible]
ENSG00000228338	0	[illegible]
ENSG00000228339	0	[illegible]
ENSG00000228340	0.00406993	[illegible]
ENSG00000228341	0	[illegible]
ENSG00000228343	0	[illegible]
ENSG00000228345	0	[illegible]
ENSG00000228347	0	[illegible]
ENSG00000228348	0	[illegible]
ENSG00000228349	0	[illegible]
ENSG00000228350	0.49331234	[illegible]
ENSG00000228351	0	[illegible]
ENSG00000228352	0	[illegible]
ENSG00000228353	0	[illegible]
ENSG00000228354	0	[illegible]
ENSG00000228355	0	[illegible]
ENSG00000228358	0.01506767	[illegible]
ENSG00000228360	0.11719905	[illegible]
ENSG00000228361	0	[illegible]
ENSG00000228363	0.02912107	[illegible]
ENSG00000228364	0	[illegible]
ENSG00000228365	0	[illegible]
ENSG00000228366	0	[illegible]
ENSG00000228367	0	[illegible]
ENSG00000228368	0	[illegible]
ENSG00000228369	0	[illegible]
ENSG00000228372	0	[illegible]
ENSG00000228373	0	[illegible]
ENSG00000228374	0	[illegible]
ENSG00000228375	0	[illegible]
ENSG00000228376	0	[illegible]
ENSG00000228379	0	[illegible]
ENSG00000228380	0	[illegible]
ENSG00000228382	0.04399964	[illegible]
ENSG00000228383	0	[illegible]
ENSG00000228384	0	[illegible]
ENSG00000228386	0	[illegible]
ENSG00000228389	0.70531305	[illegible]
ENSG00000228391	0	[illegible]
ENSG00000228392	0	[illegible]
ENSG00000228393	0.63195568	[illegible]
ENSG00000228395	0.4580454	[illegible]
ENSG00000228397	0	[illegible]
ENSG00000228398	0	[illegible]
ENSG00000228399	0	[illegible]
ENSG00000228400	0	[illegible]
ENSG00000228401	0	[illegible]
ENSG00000228403	0	[illegible]
ENSG00000228404	0.52076779	[illegible]
ENSG00000228407	0.09479335	[illegible]
ENSG00000228408	0	[illegible]
ENSG00000228409	0.6891787	[illegible]
ENSG00000228410	0	[illegible]
ENSG00000228411	0	[illegible]
ENSG00000228412	0.5310449	[illegible]
ENSG00000228413	0	[illegible]
ENSG00000228414	0.02853452	[illegible]
ENSG00000228415	0.12071064	[illegible]
ENSG00000228417	0	[illegible]
ENSG00000228418	0	[illegible]
ENSG00000228420	0	[illegible]
ENSG00000228421	0	[illegible]
ENSG00000228422	0.01848036	[illegible]
ENSG00000228423	0.45651189	[illegible]
ENSG00000228426	0	[illegible]

ENSG00000228427	0.13318074	0.05947105	0.04341612	0.1147678	0.04952737	0.13320292	0.07130273	0.08696773	0.10973902	0.04020827	0.05592802	0.26243242	0.11272551	0.14427992	0.03860894	0.04282546	0
ENSG00000228429	0	0.05486148	0.08010192	0.0529361	0.02284426	0.24575684	0.09866414	0.04011345	0.08098656	0.03709175	0.10318614	0.20750692	0.13865094	0.08873124	0.07123277	0.07901215	0.12321532
ENSG00000228430	0	0	0	0	0.00177547	0.0047751	0.00255608	0.00311765	0	0	0	0.00268793	0	0	0	0	0
ENSG00000228431	0	0	0	0	0.04657626	0	0	0	0	0.07562489	0	0	0	0	0	0	0
ENSG00000228432	0	0	0	0	0	0	0	0	0	0	0	0	0	0	0	0	0
ENSG00000228433	0	0	0	0	0	0	0	0	0.0152043	0	0	0	0	0	0	0	0
ENSG00000228434	3.57532483	8.91979675	5.74321343	3.7508028	3.42033464	5.86917321	3.48156809	3.97576763	2.9033204	4.34895784	4.41723488	4.33212391	1.41806995	1.75888516	1.32189081	1.06636765	1.16925794
ENSG00000228436	0.60363392	0.75088663	0.54817577	0.5294671	0.50507978	0.74388706	0.48476321	0.54903108	0.43698885	0.78103562	0.54319392	0.56438643	0.49267455	0.74736035	0.61872448	0.35354635	0.45404228
ENSG00000228437	0.04021178	0.02693449	0.0393264	0.0259892	0	0	0	0	0	0	0	0	0	0	0	0	0
ENSG00000228438	0	0	0	0	0	0	0	0	0	0	0	0	0	0	0	0	0
ENSG00000228439	0	0	0.14715269	0.0777978	0.03357317	0.06772081	1.61919123	1.53277583	1.32412268	1.58084964	1.51647975	1.21985392	1.1207294	1.40184583	1.33476637	1.45150709	2.12773649
ENSG00000228440	0	0	0	0	0	0	0	0	0	0	0	0	0	0	0	0	0
ENSG00000228444	0.03725981	0.04991443	0	0.0481627	0.08313727	0.11179805	0.08976726	0.03649629	0.07368372	0	0.04694075	0.03146589	0.06307416	0	0.06480947	0.03594368	0.05605229
ENSG00000228445	0	0	0	0	0	0	0	0	0	0	0	0	0	0	0	0	0
ENSG00000228446	1.03821291	1.11870539	1.147791	0.8752248	0.67985679	1.45599743	0.8700132	1.0611525	0.84803362	1.30828936	0.5686799	0.83864957	1.14620052	0.78242558	1.05996153	0.87090426	0.67906484
ENSG00000228450	0	0	0	0	0	0	0	0	0	0	0	0	0	0	0	0	0
ENSG00000228451	0.82039338	0.64867264	0.56706029	0.5262404	0.46107374	0.94391793	0.66379093	0.71899428	0.48183515	0.95534857	0.6916236	0.49487256	0.48032931	0.51454777	0.45599196	0.41653392	0.41757614
ENSG00000228452	0	0	0	0	0	0	0	0.0652258	0	0	0	0	0	0	0	0	0
ENSG00000228453	0	0	0	0	0	0.06345493	0	0	0	0	0	0	0	0	0	0	0
ENSG00000228459	0	0	0	0	0	0	0.05409173	0	0	0.03050283	0.08485631	0.08532285	0.42757951	0.54726868	1.08371313	0.61727728	0.55730149
ENSG00000228460	0	0	0	0	0	0	0	0	0	0	0	0	0	0	0	0	0
ENSG00000228462	0	0	0	0	0	0	0	0	0	0	0	0	0	0	0	0	0
ENSG00000228463	0.08078979	0.08658285	0.09481315	0.1044302	0.09914578	0.11312454	0.16003794	0.15299287	0.08787191	0.20000659	0.23748848	0.18193844	0.10029255	0.1517061	0.08431512	0.05715309	0.06481983
[truncated: 2,450,392 more chars]
